# A Proposed Complete‐Cycle Mechanism for Conversion of N_2_ to NH_3_ by Mo‐Nitrogenase

**DOI:** 10.1002/cbic.70429

**Published:** 2026-07-24

**Authors:** Ian Dance

**Affiliations:** ^1^ School of Chemistry UNSW Sydney Sydney NSW Australia

**Keywords:** density functional calculations, hydrogen atom transfer, nitrogenases, reaction mechanisms, transition states

## Abstract

How does the enzyme nitrogenase convert N_2_ to NH_3_ under ambient conditions? This perspective expounds the Dance chemical mechanism comprising 40 explicit intermediates and 31 reaction steps, from start to finish, to effect the enzyme stoichiometry N_2_ + 8H^+^ + 8e^−^ → 2NH_3_ + H_2_. Energy profiles and kinetic barriers are derived from density functional simulations with a 483+ atom quantum model. The key to the mechanism is discovery of the unique N_2_ capture step, in which N_2_ tumbles into a preformed gallery of H atoms, and is activated there by *concerted* formation of two H—N and three Fe—N bonds, forming bound HNNH. This sets up subsequent hydrogenations that break N–N and form NH_3_ in concert. Retention of bridging S2B adjacent to bound intermediates is a crucial stereochemical component. An inconsistency with kinetic data is still to be resolved. The well‐known H_2_/N_2_ exchange occurs because H_2_ is formed where its dissociation creates the space required for N_2_ capture. Possible quantum tunneling by H atoms is considered. I discuss architecture and function in the reaction space and surrounding protein. An enabling attribute of this mechanism is the favourable geometry and propitious stereochemistry involved in each of the chemical steps.

## Graphical abstract

How does the enzyme nitrogenase convert N_2_ to NH_3_ under ambient conditions? Here I describe a proposed complete cycle of the chemical mechanism, with computed energy profiles and kinetic barriers for a sequence of 31 reaction steps, encompassing 40 intermediates.

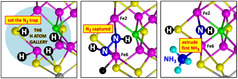

## 1 Introduction

Nitrogenase is the bacterial enzyme that converts inert atmospheric nitrogen to biologically usable ammonia, supporting all life on earth, and underpinning about half of the world's food supply [1, 2]. Conversion of inert N_2_ to NH_3_ is notorious as a chemically difficult transformation. The best industrial catalysts operate at hundreds of degrees and hundreds of atmospheres, and yet enzymatic catalysis occurs at ambient temperature and less than one atmosphere. How can this be? After five decades of research [3], this N_2_ → NH_3_ question is still a holy grail of biochemical and inorganic research [4, 5, 6, 7, 8, 9, 10, 11], and is attracting additional interest due to prospects for using ammonia as a convenient carrier for hydrogen as a renewable fuel [12, 13, 14]. Nitrogenase has valuable chemical secrets, and the research described here aims to reveal them.

The enzyme contains two proteins. Component 1 contains the catalytic site and machinery, while component 2 is a reductase. Component 1 occurs as isozymes, labelled according to the heterometal as Mo‐, V‐, or Fe‐nitrogenase. Here the focus is on the predominant Mo‐nitrogenase, for which the two component proteins are labelled, respectively, the MoFe protein and the Fe protein. The MoFe protein contains the iron–molybdenum‐cofactor (FeMo‐co), which is the catalytic site, and a P‐cluster, which is an electron transfer agent.

In the overall reaction (Equation (1)), ATP hydrolysis occurs in the Fe protein, with reduction of an Fe_4_S_4_ cluster on its surface, which transfers an electron to the P‐cluster when the Fe‐protein docks with the MoFe protein [15]. This Fe‐protein reductase cycle of docking and undocking occurs eight times in the mechanistic cycle. Conversion of N_2_ to 2NH_3_ is always coupled with formation of at least one H_2_.

(1) $$N_{2}+8e^{-}+8H^{+}+16\text{MgATP}\;\to \;2{\mathrm{NH}}_{3}+H_{2}+16\text{MgADP}+16P_{i}$$

This reaction implies a mechanistic cycle with at least 27 chemical steps: eight introductions of a proton, eight additions of an electron, N_2_ binding, breaking the N—N bond, formation of six N—H bonds, two dissociations of NH_3_, and formation of one H—H bond. In addition, each introduction of a proton, usually but not necessarily coupled to introduction of an electron, involves multiple auxiliary steps as the proton is relayed from the protein surface to FeMo‐co, and then suitably positioned on FeMo‐co to be able to form H—N bonds. Additional auxiliary steps are involved in translocating NH_3_ from the active site to the protein surface.

## 2 The Catalytic Reaction Space

**FIGURE 1:**
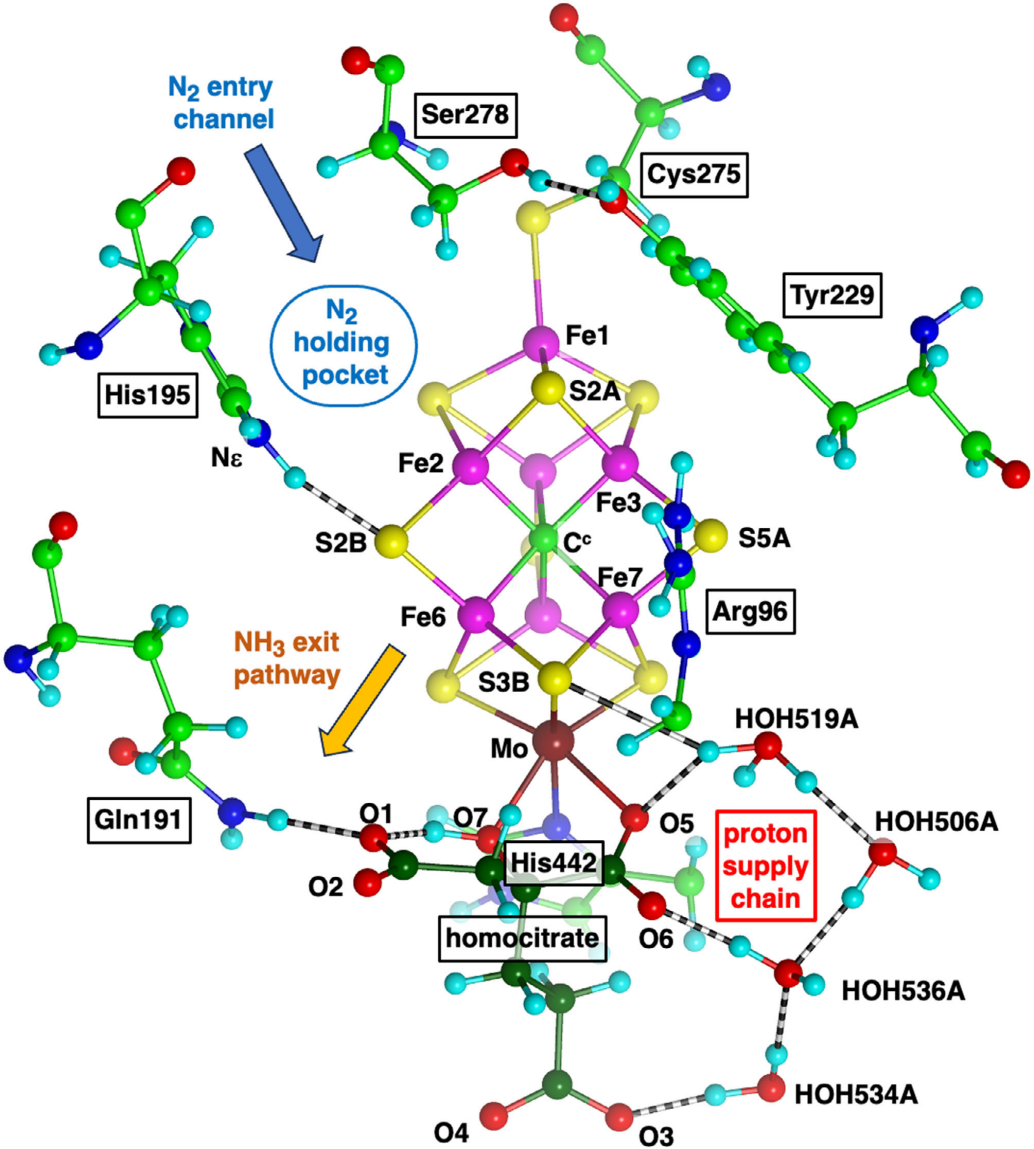
Structure of the active site of Mo‐nitrogenase, with FeMo‐co at the centre. Significant surrounding amino acids and water molecules are included, with labels for *Azotobacter vinelandii* protein, crystal PDB 3U7Q. Val70, omitted for clarity, covers the Fe2‐S2B‐Fe6 reaction zone, which is bounded on the right side by the side chain of Arg96. Homocitrate C atoms are dark green, and hydrogen bonds are striped. The N_2_ entry channel is between His195 and Ser278, with an N_2_ holding pocket approaching Fe2. The four penultimate water molecules of the extended proton supply chain [16] are shown, with hydrogen bonds to O3, O6, and O5 of homocitrate, and with the final proton directed towards S3B. The direction of NH_3_ exit from the reaction zone is indicated.

The catalytic site, FeMo‐co, is a CFe_7_MoS_9_ cluster, with homocitrate providing bidentate chelation of Mo. FeMo‐co is further coordinated by His442 at Mo (completing octahedral coordination) and Fe1 is bonded to the sidechain of Cys275. These components are shown in the centre of Figure 1. The reaction zone is the front Fe2‐Fe3‐Fe6‐Fe7 face of FeMo‐co, together with doubly‐bridging S2B (often dubbed a ‘belt sulfur’) and triply‐bridging S3B. Three key residues immediately surrounding the reaction zone are the sidechain of His195 hydrogen bonded via Nε with S2B, the sidechain of Arg96 hydrogen bonded with S5A, and the sidechain of Val70 (not shown in Figure 1) which covers the Fe2‐S2B‐Fe6 region.

The protein surrounding FeMo‐co is distinctly anhydrous—a desert [17]—but through this desert there are two functional rivers—chains of water molecules. One is the proton supply chain and the other is the pathway along which NH_3_ moves from catalytic site to the protein exterior. These are described in Sections 5.1 and 5.2.

## 3 Prologue

I have developed a mechanistic cycle for the catalytic conversion of N_2_ to 2NH_3_ by Mo‐nitrogenase, from start to finish, comprising a sequence of 40 explicit intermediates, with energy profiles and kinetic barriers for 31 reaction steps. The chemical evaluations and rationale that initiated development of this mechanism, and the density functional (DF) computations that tested and quantified the numerous hypotheses, have been described in detail in a collection of recent publications [18, 19, 20, 21, 22, 23, 24]. These papers have described the favourable stereochemistry incorporated in the intermediates and reaction steps, the alternative pathways considered, and how the requirement of mechanistic competence differentiates alternatives. However, these separate publications, each describing details of a part of the mechanism, do not show the complete picture and the continuity of the whole mechanistic cycle. The purpose of the present paper is to collect and compile the essential components of the mechanism, describing for the first time this proposed chemical mechanistic cycle from start to finish.

There is a remarkably rich milieu of research on the chemical mechanism of nitrogenase, extending over more than five decades [1, 3, 25, 26, 27, 28, 29, 30, 31, 32, 33, 34, 35, 36, 37, 38, 39, 40, 41, 42, 43, 44, 45, 46]. Before describing my proposed mechanism, I outline the context of experimental and computational knowledge. Other authors have presented different notions of intermediates and reactivity. The following section is a brief description of context. I am preparing a separate full up‐to‐date critical review of research on the chemical mechanism of nitrogenase, continuing from the 2020 review [47].

## 4 Context

Here is a brief summary of experimental information and relevant computational studies.

### 4.1 Current Experimental Information

Experimental access to intermediates in the nitrogenase mechanistic cycle has been thwarted by (1) their multiplicity, (2) their transiency, (3) the continuing and competitive reduction of unavoidable protons, and (4) the even‐electron count of many intermediates, obscuring them to spin resonance techniques [44]. Freeze‐quench techniques [48] have been deployed to trap intermediates. Advanced ENDOR spectroscopic techniques were used to assign a structure for the intermediate E4H4 (after addition of four electrons and four protons) in freeze‐quenched ^Val^70^Ile^ protein [49, 50]. This proposed structure has two belt SH bridges (Fe2–S2BH–Fe6 and Fe3–S5AH–Fe7) coupled with two H bridges (Fe2–H–Fe6) and (Fe3–H–Fe7); there are reservations about the data analysis that yielded this structure and its properties [51, 52, 53]. Spectro‐electrochemical experiments on Mo‐nitrogenase turning over under CO and N_2_ revealed SH stretching frequencies assigned to terminal SH and doubly bridging SH [54].

Cryoannealing at −50 °C allowed characterisation of the N_2_/H_2_ exchange reaction at the E4H4 stage [55]. Kinetic analyses have characterised the reactivity of intermediates and developed kinetic schemes [27, 56, 57], but do not reveal structure. Multiple experimental characteristics of the ‘HD’ reaction (D_2_ + 2H^+^ + 2e^−^ → 2HD) introduce experimental criteria for proposed mechanisms [27, 58, 59, 60, 61, 62, 63, 64, 65, 66]. There is a large collection of reactivity data on mutant proteins [64, 65, 67, 68, 69, 70, 71, 72, 73, 74, 75, 76, 77, 78], and mutation of Val70 is particularly significant: increasing the size of the sidechain from valine to isoleucine significantly decreased the N_2_ reduction activity [73]. An intermediate in the reduction of the nonphysiological substrate propargyl alcohol was trapped and characterised in the ^Val^70^Ala^ mutant [73, 79, 80]. Substrate acetylene was captured in the ^Arg^96^Gln^ protein, near but not bound to FeMo‐co [81].

Since 2014 the structures of crystals obtained from the enzyme under turnover conditions and with alternative substrates have revealed disruptions of the FeMo cofactor. The S2B site was substituted with CO bridging Fe2 and Fe6 [82]. With SeCN^‐^ as substrate, S2B was replaced by Se, and, while turning over under acetylene, Se migrated to the two other belt positions S3A and S5A [83]. Other experiments yielded crystals that indicate the possible presence of N_2_ in the belt bridging positions [84]. The V‐nitrogenase and Fe‐nitrogenase isozymes have been reported to undergo analogous substitutions at the S2B position [85, 86, 87, 88]. These structures have been reviewed [24].

### 4.2 Current Computational Information and Investigations of Mechanism

Computational studies avoid limitations inherent with experiments, but are accompanied with questions of methodology and accuracy. An early report of large variability in calculated results, associated with different electronic states of the cofactor and different density functionals [89], introduced some doubts which have now been dispelled. All authors have identified the same small number of stable electronic states of ligated FeMo‐co in intermediates [51, 90, 91, 92, 93, 94, 95, 96, 97, 98, 99, 100], and the rationale is understood [18]. Similarly the density functionals that yield validated accurate results have been identified and are used [90, 94, 96, 99, 101, 102, 103, 104]. An accuracy of 0.01 Å for the C^c^—Fe and Fe—S bond lengths and 0.02 Å for Fe–Fe distances (reference resting FeMo‐co) is achieved with my computational methodology [21, 22, 23].

I reviewed computational investigations of the nitrogenase mechanism in 2020 [47]. Since then many computational studies of possibilities for various intermediates have been published, together with various mechanistic sequences of intermediates. Bjornsson et al. have described the E2 state and H_2_ formation [93], the E3 state [97], and N_2_ binding to the E4 state [51, 96]. Ryde et al. have reported exhaustive explorations of the E2 state [95], H_2_ formation for the E2–E4 states [105], the E4 state [53], N_2_H_2_ binding [106], proton transfer pathways [107], and N_2_ binding to the E0–E4 states [98]. Zhang et al. studied the binding of N_2_ at E4 and formation of bound N_2_H_2_ [108].

**FIGURE 2:**
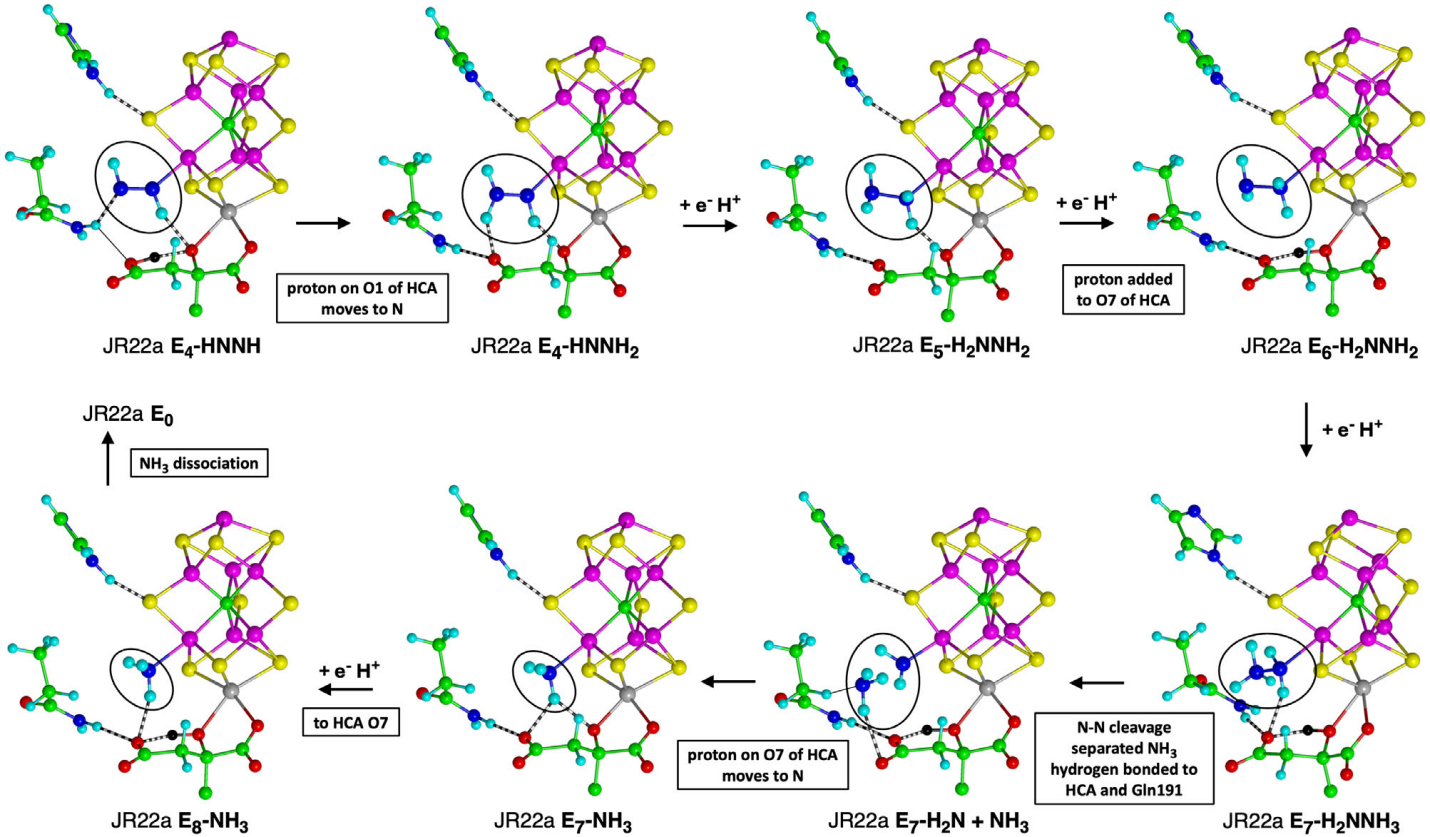
Mechanism JR22a calculated by Jiang and Ryde [94], redrawn with the published coordinates. The progressively hydrogenated intermediates starting with HNNH are encircled. S2B has no involvement.

**FIGURE 3:**
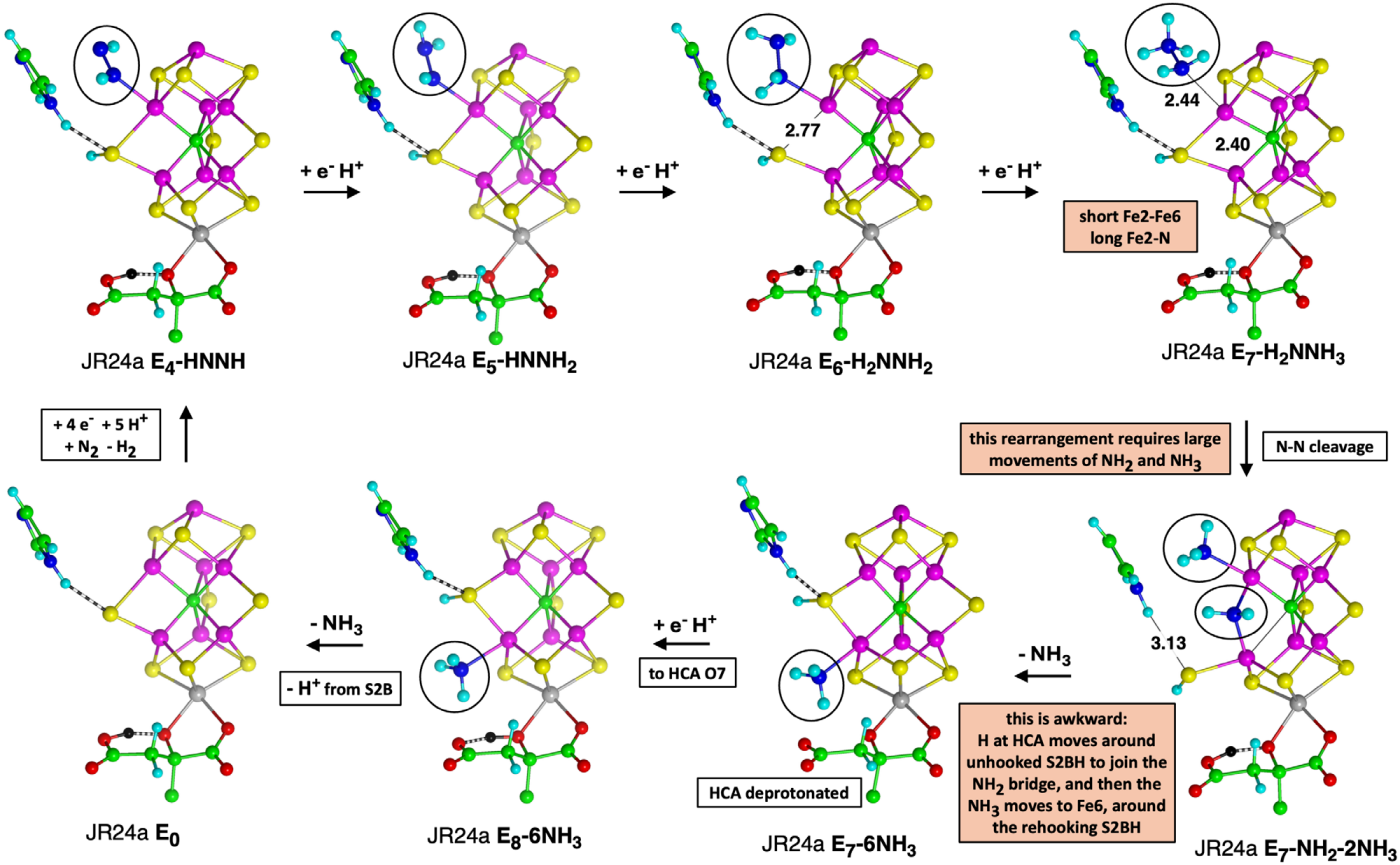
The sequence of intermediates JR24a calculated by Jiang and Ryde [94], redrawn with the published coordinates. The commentary in salmon boxes notes unusual or difficult components, such as the very short Fe2–Fe6 distance in **E_7_‐H_2_NNH_3_**.

**FIGURE 4:**
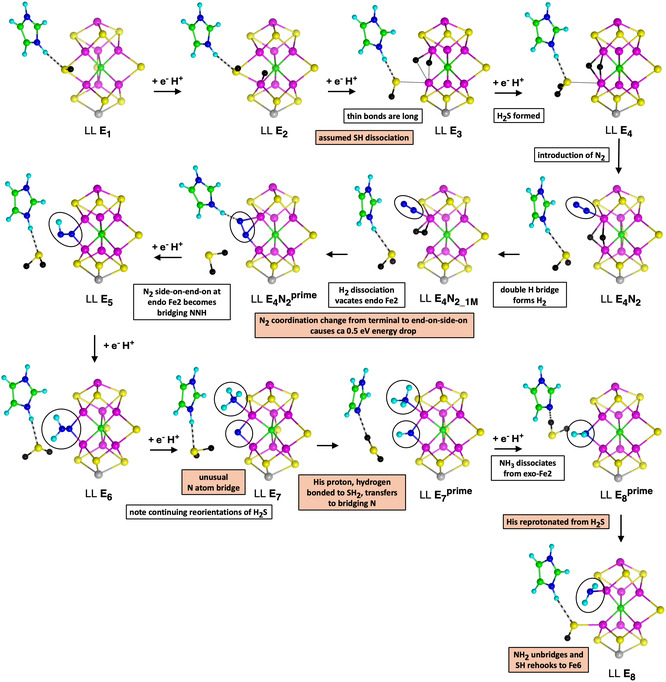
The Li and Head‐Gordon (LHG23) mechanism [100] redrawn with the published coordinates. Original labels are retained. The hydrogenated forms of N_2_ are encircled, and H donor atoms are black. The commentary in salmon boxes notes unusual or difficult components.

**FIGURE 5:**
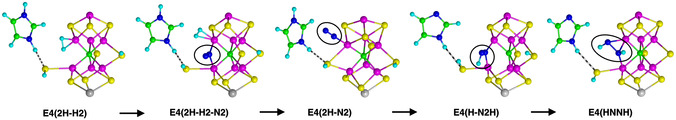
The R18 sequence of intermediates [92].

Ryde et al. described three mechanistic sequences of intermediates starting with the E4 state containing bound HNNH. One of these (CR20) has S2B completely absent from FeMo‐co [102]. Another mechanism (JR22a) [94] from E4 to E8 with S2B intact but not protonated is shown with commentary in Figure 2. A mechanism from E4 to E8 with S2B protonated and bridging except in one intermediate where it is unhooked (JR24a) [99] is shown in Figure 3. Li and Head‐Gordon [100] presented a mechanism (LHG23) with 13 intermediates from E0 to E8, displayed in Figure 4. S2B is dissociated as H_2_S through most of this sequence. Raugei et al. [92] proposed a sequence of intermediates (R18) at the E4 level, shown in Figure 5. Siegbahn proposed alternative mechanistic models [109, 110, 111, 112, 113]. Mechanisms proposed by other authors are compared with the Dance mechanism in Section 9.

## 5 Introduction to the Dance Mechanism

Here I describe a full mechanistic cycle for the catalytic conversion of N_2_ to 2NH_3_, with 40 intermediates and energy profiles and kinetic barriers for 31 reaction steps. This information derives from computer simulations, using DF calculations on a 483+ atom model including all relevant residues and involved water molecules. These calculations of mechanism have been described in full in recent publications [19, 20, 21, 22, 23], each dealing with one section of the mechanism. These papers described in detail the computations, stereochemical analyses and logic used in exploring and calculating intermediates, and reaction trajectories. These papers evaluated alternative intermediates and pathways. They covered the N_2_/H_2_ exchange, the initial activation and binding of N_2_, formation of the key HNNH intermediate, the breaking of the N—N bond, and the formation of first one and then the second NH_3_ molecule. However, because this detailed reporting was necessarily fragmented, the overall picture and the full mechanistic trail of reaction steps and their energy profiles were obscured. This account extracts the conclusions and relevant information from the detailed publications and consolidates them into a single complete presentation of the mechanism. Ancillary components of the mechanism are proton ingress and NH_3_ egress, and these were described previously [16, 114]. Molecular details of pathways and steps for electron transfer via the P‐cluster to FeMo‐co are more difficult to establish [33, 115] and are not included.

The mechanism can be regarded as a complex molecular dance, with N and H atoms changing partners with each other and with atoms of FeMo‐co. Accordingly, in Section 7 below, the mechanism is presented as a continuous choreographic score, with skeletal representations of the intermediates, linked by reaction energy barriers showing movement difficulties. The score is annotated with explanation and commentary on the rationale. This simplified description is intended to aid understanding. As preparation for this, I first describe the ancillary components: how the catalytic site is connected with the supply chain for protons; the entrance of substrate N_2_; and the egress of product NH_3_. Then follows description of attendant aspects of the mechanism: (a) accounting electrons and protons; (b) electronic states for the system; (c) the computational model and methodology; (d) entropy components; and (e) possible quantum tunneling by H atoms.

### 5.1 Proton Wire

A chain of water molecules extends from the protein surface on the right side of Figure 1 towards FeMo‐co, and culminates in a *fully conserved* chain of eight water molecules, the last four of which are shown in Figure 1. This is the proton supply chain—a proton wire—that supports sequential provision of the eight exogenous protons required in the mechanism cycle [116]. Full details of the fully characterised Grotthuss proton transfer mechanism that enables sequential supply of these eight protons from the protein surface are in ref. [16]. For most of this 20‐step mechanistic sequence for each proton the potential energy surface is remarkably flat, undulating by <5 kcal mol^−1^. Electron addition to FeMo‐co is calculated to increase the basicity of its S atoms, and so electronation of FeMo‐co could be the trigger for proton transfer from the proton wire to S of FeMo‐co [19]. Electron plus proton addition creates an H atom on S3B, from which it can migrate to other atoms of FeMo‐co [117], as will be described in Section 7.

### 5.2 NH_3_ Egress

The other functional water feature in the MoFe protein extends from the lower left side of FeMo‐co, and facilitates the transport of product NH_3_ away from Fe6 to the protein surface [17]. The molecular mechanism for translocation involves NH_3_ skipping through a sequence of hydrogen bonds with eleven water molecules and surrounding amino acids [114]. The moving entity in the early stages of egress is the NH_3_ molecule, possibly becoming NH_4_^+^ only near the protein surface. The side chain of Gln191 has significant involvement, which is consistent with experimental data on mutation of this residue [118]. This NH_3_ egress pathway is conserved in species *A. vinelandii*, *Klebsiella pneumoniae,* and *Clostridium pasteurianum*.

### 5.3 Role of Homocitrate

Homocitrate, (R)‐hydroxybutane‐1,2,4‐tricarboxylate, is an obligatory component of nitrogenase [119]. Referring to Figure 1, carboxylate atoms O1, O2, and O4 of homocitrate are part of the NH_3_ egress mechanism [17], while carboxylate atoms O5, O6, and O3 are essential components of the proton supply chain. In this way homocitrate separates the acidic component of the mechanism—proton supply from the right—and the basic component—NH_3_ transport to the left. The physiological reaction of nitrogenase consumes acid and generates base, and so homocitrate has the role of preventing acid–base conflict. This is elaborated in ref. [17].

### 5.4 Role of His195

**FIGURE 6:**
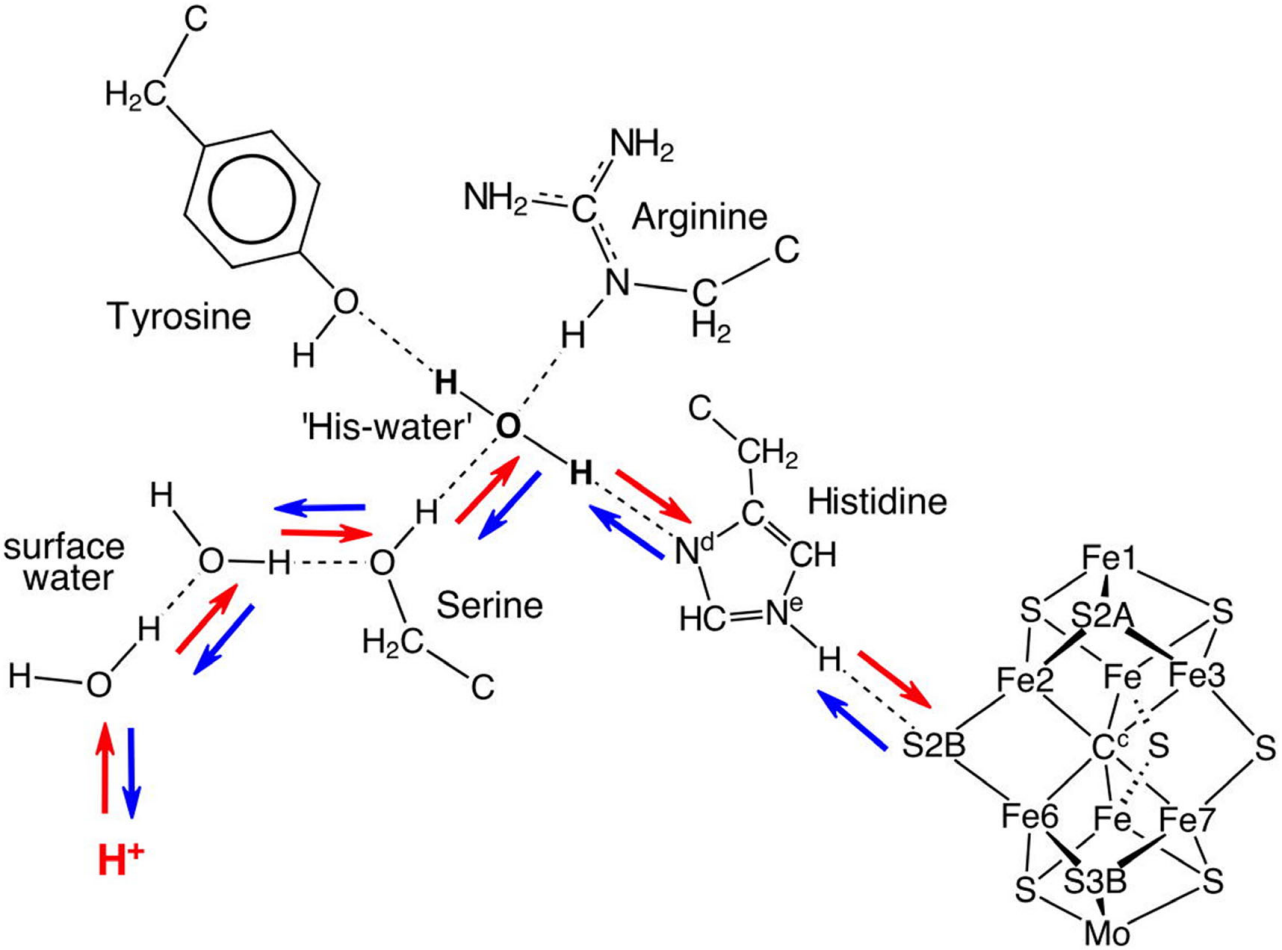
Conserved residues forming the environment of the single water molecule, ‘His‐water,’ hydrogen bonded to the back side (Nδ) of histidine. Possible proton slides link surface water to proton buffering at S2B.

The imsidechain of His195, protonatable at Nε, is within hydrogen bonding distance of S2B. Despite this, His195 cannot translocate protons *from the protein surface* to FeMo‐co [120]. However, it is able to function as a proton buffer with S2B, adjusting the protonation of S2B, and does so at two stages in the mechanism described in Section 7. Behind the imidazole group of His195 there is a conserved water molecule hydrogen bonded to Nδ, and further hydrogen bonded to strictly conserved Arg, Tyr, and Ser residues. As illustrated in Figure 6, I postulate that this structural feature enhances, and possibly controls, the proton‐buffering function of the histidine sidechain with S2B and the reaction zone of the cofactor [17].

### 5.5 Coordination Geometry of FeMo‐co

In the resting state of the enzyme the Fe atoms in FeMo‐co are four‐coordinate with pseudo‐tetrahedral stereochemistry. At all except Fe1 this coordination can be expanded, using an ‘exo’ position that is trans to the Fe—C^c^ bond (yielding trigonal bipyramidal Fe), or an ‘endo’ position that is approximately cis to the Fe—C^c^ bond (yielding square pyramidal Fe), or both exo and endo (yielding pseudo‐octahedral Fe coordination) [36]. Note that for endo coordination the S2B‐Fe2‐S2A and S2B‐Fe6‐S3B angles can expand towards 180° as doubly‐bridging S2B swings backward.

As outlined in Section 4.1, there is experimental indication that S2B may not be retained as a bridge between Fe2 and Fe6 during the mechanism [82, 83, 84, 85, 86, 88, 121]. I critically reviewed the experimental evidence and related computational studies in ref. [24]. There is a dilemma: does nitrogenase use a disruptive mechanism in which S2B is separated from FeMo‐co, moved to a storage location, and finally returned to FeMo‐co, or has the enzyme evolved a conservative mechanism with bridging S2B retained intact, and with S2B actively contributing to the catalysis? The Dance mechanism is strictly conservative, and uses standard chemistry throughout. Significantly, intact S2B is a crucial agent at two stages in this mechanism.

### 5.6 N_2_ Entry

Figure 1 shows the location of a protein channel through which the aprotic substrate N_2_ diffuses towards Fe2 of FeMo‐co. There is an N_2_ holding pocket, from which N_2_ can coordinate end‐on (η^1^) at the *exo* coordination position of Fe2, or diffuse past Fe2 and enter the reaction space between Fe2 and Fe6. Both behaviours occur during the Dance mechanism. The thermodynamics of N_2_ binding at the *exo*‐Fe2 position are unfavourable in the resting state (as observed), but can become favourable according to the coordination of Fe2 and Fe6 by other ligands during the mechanism [20]. N_2_ bound at *exo*‐Fe2 is unproductive and not reducible because there are no H atom sources in the vicinity. This N_2_ is described as ‘nonreducible’ N_2_ in the following (and dubbed ‘2N2x’ in schemes), and is differentiated from the ‘reducible’ N_2_ molecule that can diffuse into the reaction space and be hydrogenated there. It is important to understand that nonreducible N_2_ does not lead to any observable product, and so is not part of observables such as the N_2_‐dependent formation of H_2_, or of kinetic analyses of product formation [27, 56, 57].

## 6 Ancillary Components of the Mechanism

### 6.1 Accounting Electrons and Protons

Pioneering kinetic investigations by Thorneley and Lowe [27] led them to a mechanistic framework in which the intermediates are labelled by the number of added electrons, as E_*n*_, *n* = 0 to 8. It is frequently assumed that electrons and protons are added concurrently, and that the intermediates can be classified and labelled as E_*n*_H_*n*_. However, there is no experimental evidence for equal numbers of added electrons and protons at each stage of the mechanism. As will be explained in Section 7.2, the present mechanism requires that some intermediates contain one added proton more than the number of added electrons. Therefore all intermediates will be labelled with a status box, [Ex yH^+^].

### 6.2 Electronic States

As a cluster containing eight transition metals the cofactor has a complex electronic structure, with multiple electronic states. The Fe—S bonding and the Fe—C^c^ bonding are polar covalent in this soft metal sulfide cluster. The electronic states of FeMo‐co and its ligated forms are described as sets of signed spin populations on the seven Fe atoms of the cluster, together with the net spin S. The electronic state descriptors are abbreviated lists of the identities of the Fe atoms with negative spin population. For *resting state* FeMo‐co (*S* = 3/2) the electronic states computed to be most stable are 235 (i.e., negative spin densities at Fe2, Fe3, and Fe5), 247 and 346 [51, 90, 91, 92, 93, 94, 95, 96, 97, 98]. These are the three states with the maximum number of oppositely signed spins along the three axial Fe–Fe edges of the central Fe_6_ trigonal prism of FeMo‐co. In reference [18] and the Supporting Information, I explain that *in reaction intermediates*, with ligation of the Fe2 and/or Fe6 atoms, there are two most favourable electronic states that have the spin sign combinations ‐Fe3,+Fe4,‐Fe5,+Fe7, or +Fe3,‐Fe4,+Fe5,‐Fe7. These states are labelled ‘35’ and ‘47’ respectively. This stability of the 35 and 47 electronic states is consistent with the findings and procedures of other authors [51, 90, 91, 92, 93, 94, 95, 96, 97, 98].

### 6.3 Computational Model

The computed protein model is a 483+ atom extract from crystal PDB 3U7Q, including all relevant amino acids. This model includes nine of the ten active‐site residues that are conserved across all analysed extant nitrogenases [122]: the exception, Gly424, is outside the reaction zone. Details of the protein model and the rationale for inclusion of amino acids and truncation of uninvolved side chains, are provided in the Supporting Information. Some constraints on the protein structure are required during optimization calculations because the modelled protein is incomplete and the influences of the complete protein outside the computational model are absent. The strategy for protein structure constraints is described in the Supporting Information. There are no constraints on bond distances or bond angles, including during transition state searches. Trial calculations on the modes of diffusion and coordination of N_2_/H_2_ suggested that the front helical chain near Val70 should move slightly away from FeMo‐co [123]. This is consistent with experimental data on mutation of amino acid 70 with smaller and larger side chains [37, 124, 125]. Also included is a small libratory movement of the sidechain of Arg96, without changing the hydrogen bond from NH_2_ of Arg96 to S5A: details are in the Supporting Information.

### 6.4 Computational Methodology

DF calculations use the distinctive DMol3 methodology of Delley [126, 127, 128, 129, 130, 131], with accurate double numerical plus polarisation basis sets [129]. The real‐space cutoff in the calculation of atomic basis sets was 9 au (4.8 Å), which gives results within 0.1% of the limit for larger extensions of the basis sets. The gradient‐corrected functional PBE [132] was used because validation tests demonstrate that when used with the numerical basis sets of DMol3 it is more accurate than other commonly used functionals [101]. These validation tests used 19 experimental test systems, including geometries and reaction energies for coordination of N_2_, CO, H_2_, and C_2_H_2_ at metal sites, structures of Fe_*x*_H_*y*_ clusters, and hydrogen bonding by water, and tested 11 DFs. The most accurate functional, PBE, also reproduces the lengths for C^c^—Fe bonds and for Fe—S bonds in resting state FeMo‐co to 0.01 Å or better. Details are provided in the Supporting Information Section 4, Validation. Details of validation calculations of the enthalpy of metal‐N_2_ coordination are contained in Section 7.3 below. Torbjörnsson and Ryde [103] made a valuable comprehensive investigation of the performance of many DFs against the same set of experimental structural, enthalpy, and entropy data as used in my validation tests, but with Gaussian basis sets rather than numerical basis sets. There are general agreements and disagreements between the two methodologies. At the conclusion of this article I urge other theoreticians to apply their methodologies to the Dance mechanism.

As explained in the Supporting Information, the DMol3 methodology obviates the need for empirical treatment [133] of the long‐range dispersion energies that are involved when the computational model is comprised of separate molecular units. The calculations were all‐electron, spin‐unrestricted, with no imposed symmetry. The conductor‐like screening model [134, 135, 136] was used with a dielectric constant of 5. Constraints on interatomic distances used the Lagrange Multiplier Algorithm. Control of electronic states was via the input spin populations for Fe1, Fe3, Fe4, Fe5, and Fe7. Output spin populations are calculated by the Mulliken method [137].

The procedure for determination of transition states and reaction potential energy barriers is described in the Supporting Information Section 5. The reacting atoms were not constrained.

### 6.5 Entropy Contributions

The mechanism involves isolated molecules N_2_, H_2_, and NH_3_ *within the protein*, associating with or dissociating from the cofactor. The small‐molecule translational entropic contributions in these steps need to be considered. It is estimated that *T*Δ*S* for association of N_2_ is ca −4 kcal mol^−1^ and no larger than −6 kcal mol^−1^ [20], and that *T*Δ*S* for dissociation of NH_3_ is ca +2.4 kcal mol^−1^ [23].

### 6.6 H Atom Tunneling

Quantum mechanical tunneling through a reaction barrier is maximized for the smallest atom encountering the barrier, hydrogen. The majority of the reaction steps in the nitrogenase mechanism involve movements of hydrogen atoms, and so H atom tunneling could be significant [47]. I previously analysed some reaction trajectories for nitrogenase steps [21, 23, 138], referencing these trajectories to proton transfer in the enzyme aromatic amine dehydrogenase, where a detailed investigation concluded that the reaction is dominated by quantum tunneling over a distance of ca 0.6 Å, at ∼10 kcal mol^−1^ below the top of the classical potential energy barrier [139]. I am not aware of other reports of H tunneling involving S—H or Fe—H bonds, and so most steps in the nitrogenase reaction have no precedential guidance. My analyses adopt the basic criterion that the potential energy profile for H transfer be symmetrical about the classical barrier, for a narrow (∼0.6 Å) section of the H atom transfer coordinate, during which heavier atoms do not move. In many of the reaction steps described below there are movements of N or S atoms during H transfer, diminishing or negating tunneling. However in one reaction step (**Fe2‐brNH2‐Fe6H** → **Fe6NH3** below) in which H transfers from 6Hx to NH_2_ I estimate that H tunneling could occur ∼4 kcal mol^−1^ below the classical barrier [23].

## 7 The Score

### 7.1 Introduction and Definitions

Scheme 1 is a pictorial account (‘choreographic score’) of all steps in the Dance mechanism, from resting state to resting state, using skeletal representations of the intermediates and ‘mountain‐valley’ depictions of the energy barrier and energy change for each reaction step (all on the same vertical scale). The energy unit is kcal mol^−1^. Each lettered line of the score continues from the previous. The electron–proton status of each intermediate is marked, boxed in red. Brief commentary included within boxes on the score is descriptive and (in coloured boxes) explanatory. Labels 3b6, 3b5, 3b4, 3b3, and 3b2 describe configurations for an H atom on S3B [117], as shown in the skeletons. Labels 6Hn and 7Hn are for H atoms in *endo* positions, 6Hx is *exo*. In the labels for intermediates an atom labelled ‘br’ bridges Fe2 and Fe6; ‘core’ is used for bare FeMo‐co when HisNε is unprotonated; the resting state contains HisNεH. For simplicity, 2N2x (nonreducible N_2_ at *exo*‐Fe2) is omitted from many labels of intermediates.

SCHEME 1The sequence of intermediates and reaction steps in the Dance mechanism, with energy profiles in kcal mol^−1^ (all on the same vertical scale). Each lettered line continues from the previous intermediate. Electronic states are marked, with spin S in parentheses. The electron–proton status of each intermediate is red and boxed. Commentary included within boxes on the score is descriptive and (in coloured boxes) explanatory. Labels 6Hn and 7Hn are for H atoms in *endo* positions, 6Hx is *exo*; an atom labelled ‘br’ bridges Fe2 and Fe6; ‘core’ is used for bare FeMo‐co when HisNε is unprotonated. Labels 3b6, 3b5, 3b4, 3b3, 3b2 describe configurations for an H atom on S3B [117], as shown in the skeletons. For simplicity, 2N2x (nonreducible N_2_ at *exo*‐Fe2) is omitted from many labels of intermediates. Reaction energies and barriers for other electronic states at each step are very similar to those marked.

**Figure:**
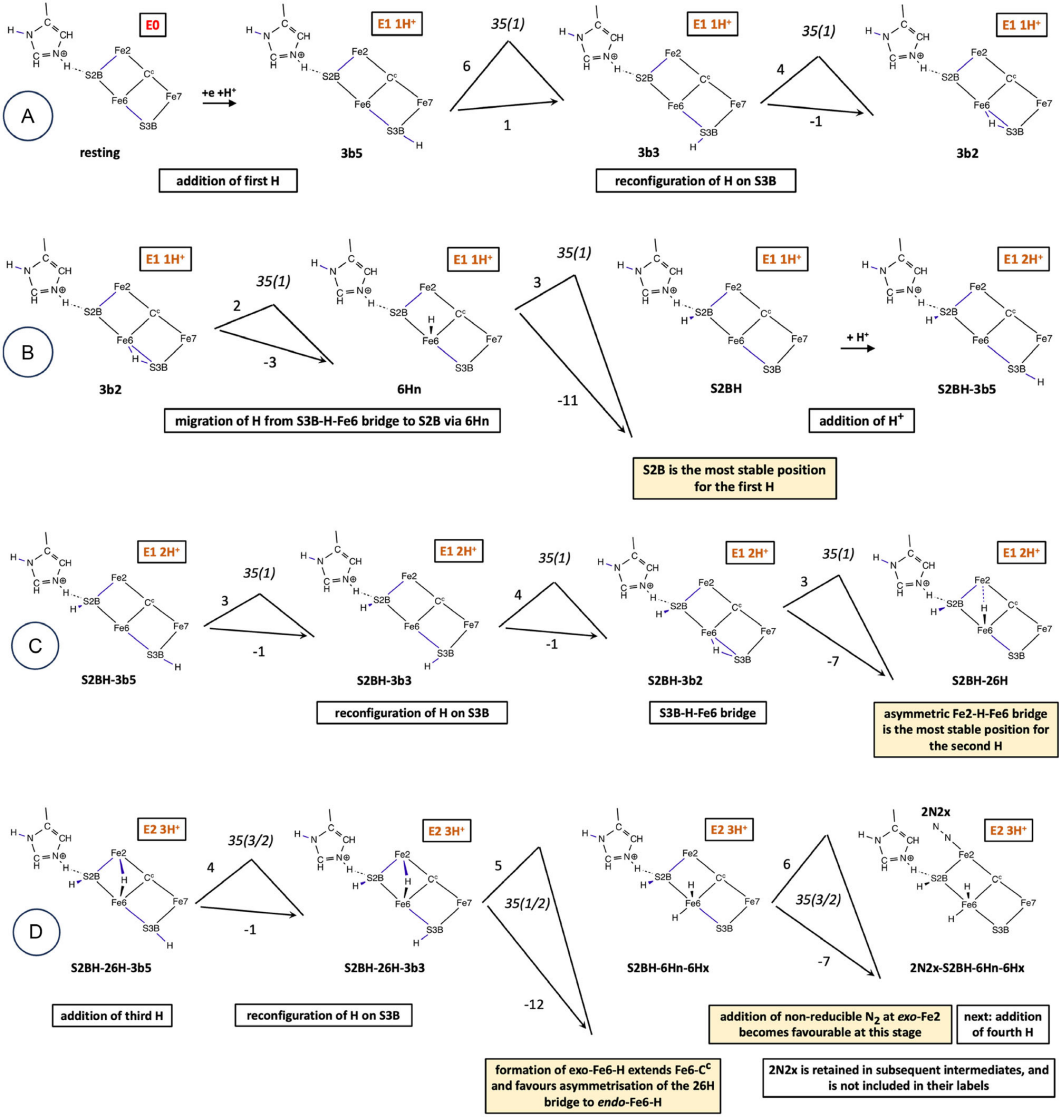

**Figure:**
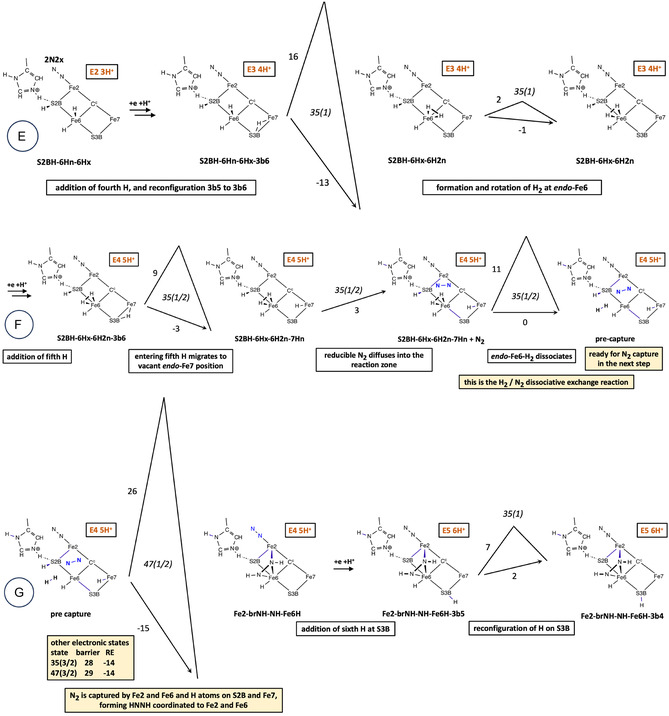

**Figure:**
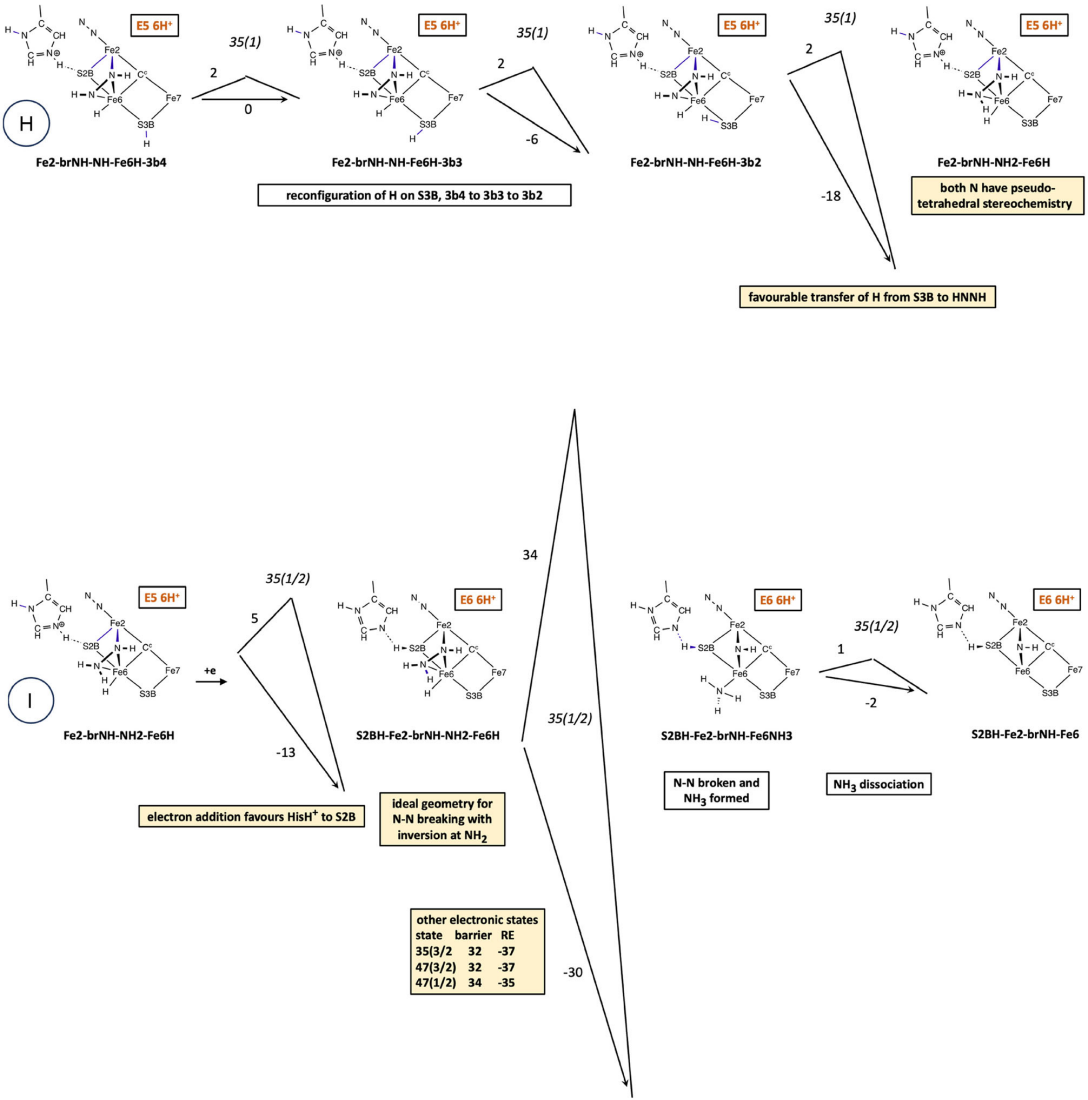

**Figure:**
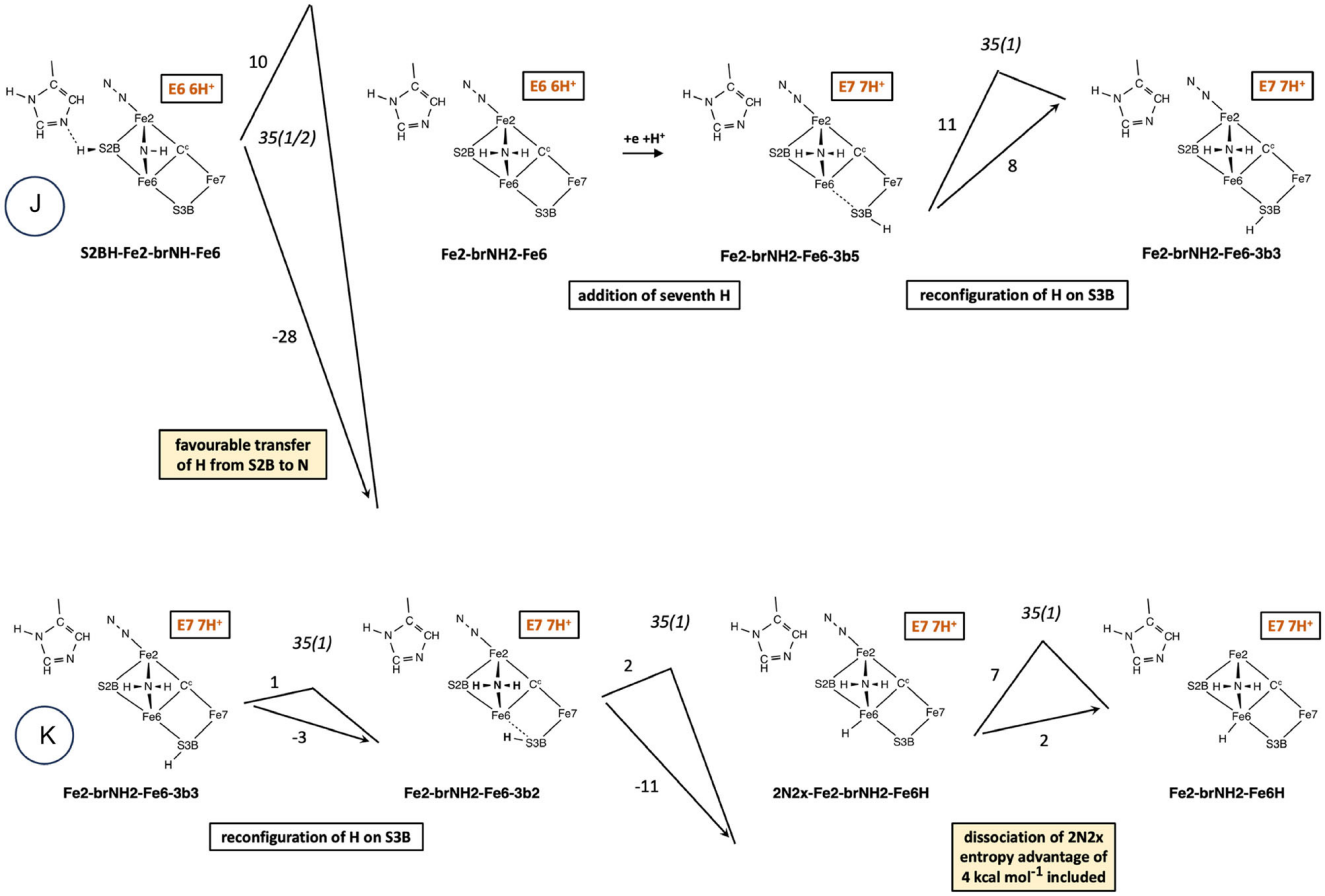

**Figure:**
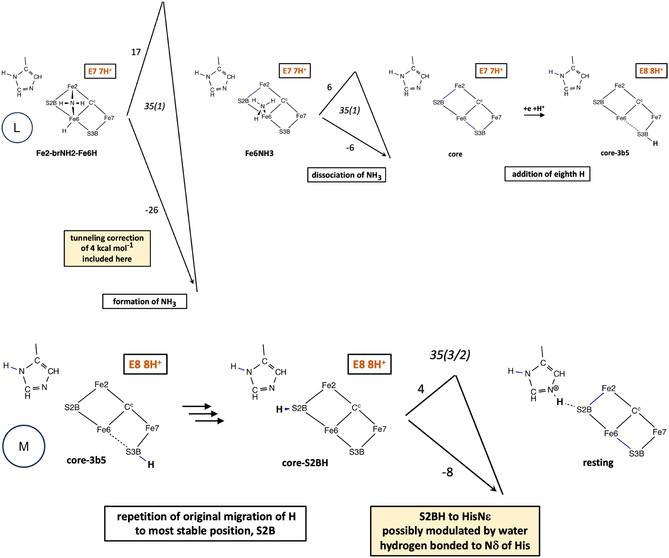

### 7.2 Details and Explanations

Scheme 1 is a simplified display of the complete sequence of computed reaction energies and reaction barriers. The energies calculated for reasonable alternative electronic states at each step are not significantly different from those marked, and are excluded to avoid complicating reader appreciation of the essence of the sequence. These additional results, together with details and elaboration of the intermediates and reaction steps, including explicit pictures of intermediates and transition states, can be found in the original reports, which are refs. [16, 19, 52, 117, 123] for lines A to F of Scheme 1, ref. [21] for line G, ref. [22] for lines H and I, ref. [23] for lines J to M. Results and discussion for alternative pathways and steps that were examined can also be found in these papers.

The reason for accumulation of five protons at the E4 stage, in line F of Scheme 1 (intermediates **S2BH‐6Hx‐6H2n‐7Hn** to **pre‐capture**), is the required sum of two H for dissociating H_2_ in the H_2_/N_2_ exchange step (line F), two H atoms at S2B and Fe7n for the double hydrogenation of N_2_ in the capture step (line G), and 6Hx at the *exo* position of Fe6. This 6Hx is required to be in place at this early stage so that the subsequent steps—H_2_ formation, H_2_/N_2_ exchange, and then N_2_ capture—are forced to use the *endo*‐Fe6 position because *exo*‐Fe6 is blocked. 6Hx is then used in the later key N–N breaking step, **S2BH‐Fe2‐brNH‐NH2‐Fe6H** → **S2BH‐Fe2‐brNH‐Fe6NH3** (line I).

In the N_2_ capture step (line G), reducible N_2_ diffused into the reaction zone can tumble freely (ca 4 kcal mol^−1^ [21]) and orient obliquely to Fe2 and Fe6, favouring N_2_ capture. This step (**pre‐capture** → **Fe2‐brNH‐NH‐Fe6H**) is a concerted double hydrogenation of N_2_, coupled to formation of one N—Fe2 bond and two N—Fe6 bonds. Detailed analysis of the trajectory shows sequential phases along the multivariable reaction coordinate [21]. The first phase is H atom movement from Fe7 towards N in cooperation with Fe2—N bond formation, and then S2BH approaches the other N atom and transfers H to it, forming *trans* HNNH, coupled with further Fe—N bond formation. There is one barrier for this complete transformation in electronic states 47(1/2), 35(3/2) and 47(3/2), while in electronic state 35(1/2), there is an Fe‐bound NNH intermediate in a very shallow (ca 1 kcal mol^−1^) energy well. The geometry idealisation that enables this remarkable activation and hydrogenation and binding of N_2_ is illustrated in Section 8. Ref. [21] also describes alternative capture intermediates in which NNH or HNNH is bonded to only one Fe: these are less stable than bridged analogs.

Three criteria are used in assessing intermediates and reaction steps at the N_2_‐capture stage and beyond. These are energy, stereochemistry, and mechanistic competence, i.e., the ability to undergo further feasible and productive reactions. These assessments are reported in ref. [22], which also describes branch pathways from **Fe2‐brNH‐NH‐Fe6** through to **S2BH‐Fe2‐brNH‐Fe6,** and the consequences of alternative stages for introduction of the fifth and sixth electrons, and details of the dissociation of the first NH_3_ into the egress passageway.

Ref. [23] describes and discusses alternative sequences and energy profiles for the steps shown in lines K and L, from **Fe2‐brNH2‐Fe6‐3b2** to **core**. These steps generate 6Hx (again, after its first use in line I) and involve formation of the second NH_3_ at *endo*‐Fe2 or *endo*‐Fe6, dissociation of NH_3_, and dissociation of 2N2x. The entropy components of the dissociations of two small molecules, NH_3_ and 2N2x, are included in this analysis, together with the contribution from possible H tunneling during formation of the second NH_3_.

The effect of electron addition to FeMo‐co favouring H transfer to S3B [19] (Section 5.1) is evident also in the HisH to S2B transfer (**Fe2‐brNH‐NH‐Fe6H** → S2BH‐**Fe2‐brNH‐NH‐Fe6H, line I**). Without electron addition the reaction energy is −5 kcal mol^−1^ with a barrier of 8 kcal mol^−1^, but with electron addition (as in line I) the reaction energy is −12 kcal mol^−1^ with barrier 4 kcal mol^−1^.

### 7.3 Kinetics

Experimental kinetic information against which a mechanism and computed reaction barriers should be assessed has been provided by the data and analyses of Harris [56, 57]. These update the first kinetic analyses of Thorneley and Lowe [27]. They yield much information about the Fe protein cycle and the processes that generate H_2_: the result that is relevant here is the overall rate of NH_3_ formation, ca 1 s^−1^. The computed barriers for two of the steps in the Dance mechanism are not consistent with this rate. The energy barrier corresponding to a rate of 1 s^−1^ is ca 18 kcal mole^−1^, but the capture step **pre‐capture** → **Fe2‐brNH‐NH‐Fe6** is calculated with a barrier of 26–29 kcal mol^−1^, and the N–N breaking step **S2BH‐Fe2‐brNH‐NH2‐Fe6H** → **S2BH‐Fe2‐brNH‐Fe6NH3** is calculated to have a barrier of 32–36 kcal mol^−1^. These are the two most difficult steps in the overall reaction.

Both of the high‐barrier steps, **pre‐capture** → **Fe2‐brNH‐NH‐Fe6** and **S2BH‐Fe2‐brNH‐NH2‐Fe6H** → **S2BH‐Fe2‐brNH‐Fe6NH3**, are founded on ideal geometry and stereochemistry. There are no awkward bond angles and no unusual coordination. As described in Section 8, there is a well‐developed architectural framework for these reaction steps. Therefore, the discrepant large computed energy barriers are probably attributable to deficiencies in the computational methodology. Here it is appropriate to revisit the results of the methodology validation calculations for relevant experimental systems involving metal‐N_2_ coordination [101].

Fe(CO)_4_N_2_ → Fe(CO)_4_ + N_2_; Δ*H*_298_ calc 18.8, 20.0, exp 17.6 ± 1.8 kcal mol^−1^.

Ni(CO)_3_N_2_ → Ni(CO)_3_ + N_2_; Δ*H*_298_ calc 10.2, exp ca 10 calc kcal mol^−1^.

Cr(CO)_6_ + N_2_ → Cr(CO)_5_N_2_ + CO; Δ*H*_298_ calc 24.7, exp 20.1 kcal mol^−1^.

(C_5_Me_5_)MoCl(PMe_3_)_2_ + N_2_ → (C_5_Me_5_)MoCl(PMe_3_)_2_N_2_; Δ*H*_298_ calc −25.9, exp −22.8 ± 2.1 kcal mol^−1^; Δ*S* −40.3, exp −67 ± 7 cal mol^−1 ^K^−1^.

(PCy_3_)_2_Mo(CO)_3_ + N_2_ → (PCy_3_)_2_Mo(CO)_3_(N_2_); Δ*H*_298_ calc −11.8, exp −9.0 ± 0.6 kcal mol^−1^; Δ*S*_298_ calc −41.4, exp −32.1 ± 3.2 cal mol^−1 ^K^−1^.

(PCy_3_)_2_W(CO)_3_ + N_2_ → (PCy_3_)_2_W(CO)_3_(N_2_); Δ*H*_298_ calc −15.1, exp −13.5 ± 1.0 kcal mol^−1^.

Note that in all cases the calculated values are close to, but exceed slightly, the experimental values, signifying that this methodology is slightly overbinding. This partly accounts for the discrepancy between the large energy barriers and the experimental kinetics of Mo‐nitrogenase.

In addition, there are probably additional dynamic, electrostatic and environmental effects not included in the present potential energy calculations. These two large‐barrier steps should be independently investigated with QM/MM methodology, possibly including molecular dynamics [140], and using other DFs that yield reliable results. It will be valuable to learn what the models and methodologies of Ryde et al. (QM/MM, functionals r^2^SCAN and TPSSh) [99], Bjornsson et al. (QM/MM, functionals r^2^SCAN, TPSSh, and B97‐D3) [97], Siegbahn (170 atom QM, functional B3LYP) [112], Zhang et al. (QM/MM, functional TPSSh [108], and Li and Head‐Gordon (ab initio molecular dynamics 300K) [100] yield for the barriers of the **pre‐capture** → **Fe2‐brNH‐NH‐Fe6** and **S2BH‐Fe2‐brNH‐NH2‐Fe6H** → **S2BH‐Fe2‐brNH‐Fe6NH3** reaction steps.

Also, it is likely that H atom tunneling is more influential than the crude estimates made above (Section 6.6). Nandi et al. reviewed examples of organometallic reactions involving H—M and H—C bonds which occur with H atom tunneling [141]: these systems are chemically similar to steps in the proposed chemical mechanism of nitrogenase. Matxain and Huertos recently reviewed hydrogen tunneling in catalytic reactions involving transition metals [142]. Computational methods for evaluating tunneling contributions are recently reviewed in ref. [141].

To enable these alternative calculations, atomic coordinates for all intermediates and transition states (including electronic state variants) are provided in the Supporting Information. Calculations of quantum tunneling in the nitrogenase mechanism are likely to be challenging computationally. However, in Supporting Table S2 I provide coordinates for small models of the transition states of the N_2_‐capture step and the N–N breaking step: these could be truncated even further with retention of chemical integrity, and be amenable to higher level computations of the tunneling characteristics [141]. It will be valuable to acquire an understanding of the extent and consequences of H atom tunneling in the nitrogenase mechanism.

### 7.4 Non‐obligatory H_2_ Evolution

Nitrogenase activity involves production of H_2_. This occurs in the H_2_/N_2_ dissociative exchange step, line F, in which bound H_2_ occupying the *endo*‐Fe6 position moves away so that N_2_ can be captured at the same location. This is the stoichiometry represented in Equation (1), and this H_2_ is called obligatory H_2_ [1]. Under N_2_‐deficient conditions nitrogenase releases H_2_ in excess of the equivalent N_2_ converted, and in the absence of N_2_ also produces H_2_. This is non‐obligatory H_2_, not dependent on N_2_. Protons are an unavoidable substrate of nitrogenase. Analysis of kinetic data, originally by Thorneley and Lowe [27] and recently extended [56, 57], uses models with H_2_ released at the E2, E3, and E4 stages. Computed pathways for non‐obligatory H_2_ formation and dissociation at the E2, E3, and E4 stages are shown in Supporting Scheme S1.

## 8 Architecture and Function

**FIGURE 7:**
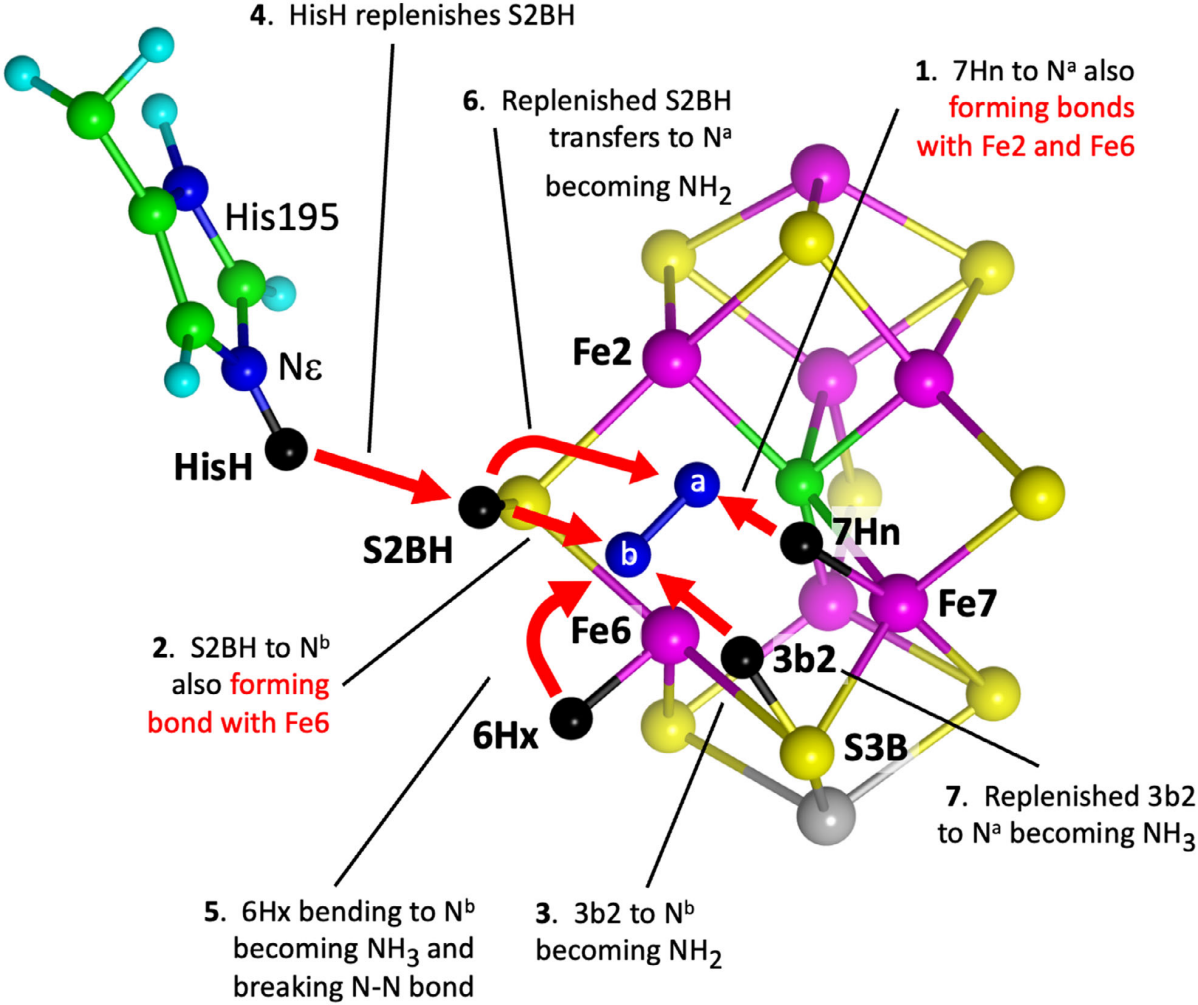
Key components of the mechanism in the catalytic reaction domain between Fe2, S2B, Fe6, S3B and Fe7. 2N2x is omitted. Reducible N_2_ is shown in its capture space, and H atoms that will transfer to atoms a and b of N_2_ are coloured black. The sequence of H atom transfers is numbered. Bonds formed with Fe2 and Fe6 are not shown, for clarity, but are described (red text) in the information captions. The H transfers shown occur from the beginning of the capture step, after the dissociation of H_2_ from the *endo*‐Fe6 position. The second transfer from 3b2 (replenished from the proton wire after transfer #3), to 6Hx then N^a^ (Scheme 1 lines K, L) is not drawn, for clarity.

**FIGURE 8:**
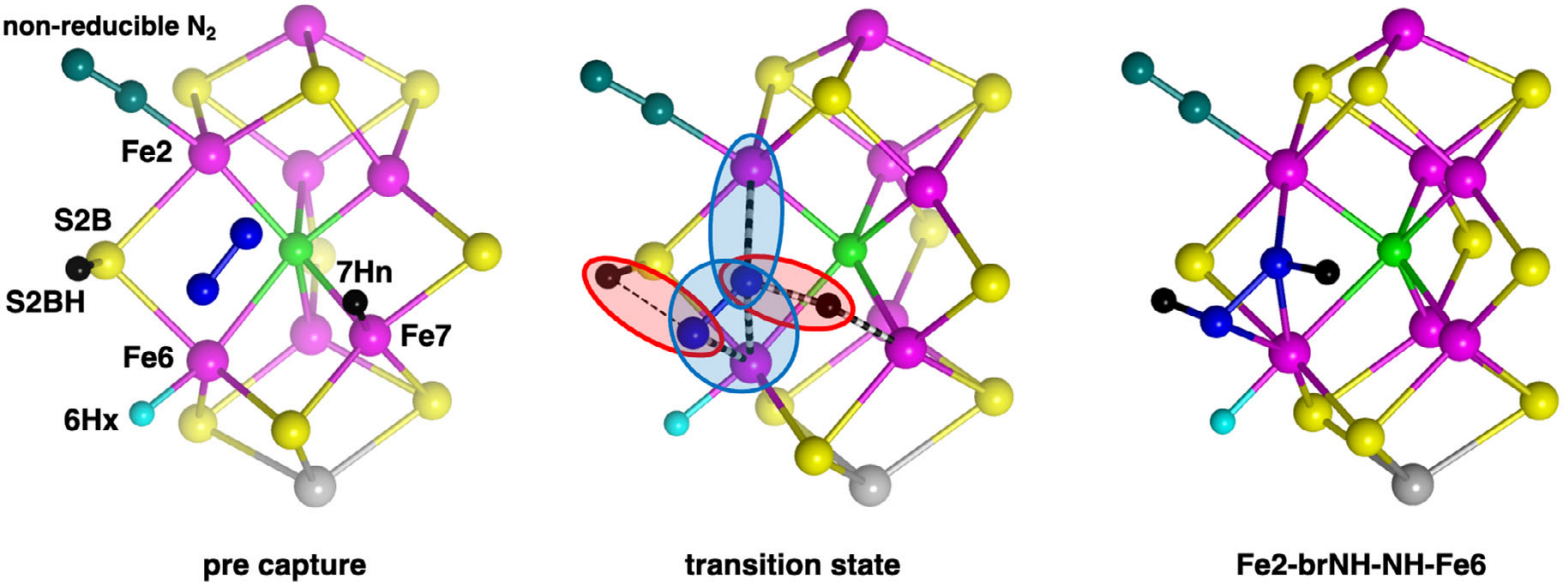
Detail of the N_2_ capture step: the two capturing H atoms are black. In **pre‐capture**, N_2_ is >3.2 Å from Fe2 and Fe6. At the **transition state** red enclosures emphasise the two H—N bonds forming and blue enclosures emphasise the three N—Fe bonds forming.

**FIGURE 9:**
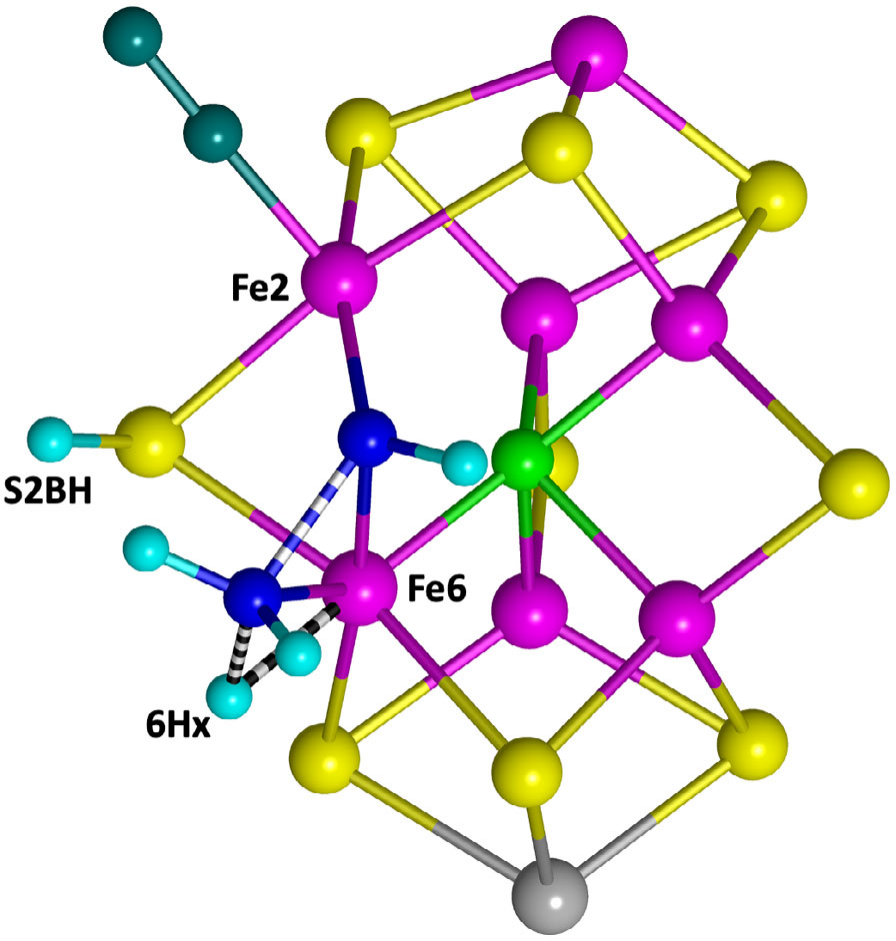
Transition state for the N–N breaking step, **S2BH‐Fe2‐brNH‐NH2‐Fe6H** → **S2BH‐Fe2‐brNH‐Fe6NH3**. The changing bonds are striped, and are coplanar with the two Fe6—N bonds.

This Dance chemical mechanism is founded on the architecture of FeMo‐co and immediate surrounds as performance space for the choreography of the catalysis. Figure 7 is an overview of key features, and demonstrates the significant correlation between the sequence of steps and the advantageous geometry that occurs at each step. The H transfer steps marked as red arrows on Figure 7 yield the six H—N bonds required to form two NH_3_. With reference to Figure 7, item 1, at the beginning of the capture step 7Hn moves towards N^a^ and the N^a^—Fe2 bond begins to form. This involves good stereochemistry (pseudo‐tetrahedral) at N^a^. Then, item 2, the continuing capture involves H transfer from S2BH to N^b^ together with formation of N^a^—Fe6 and N^b^—Fe6 bonds. Figure 8 shows detail of reactant, transition state, and product of N_2_ capture, with emphasis on the five bonds that are formed in concert. In the product **Fe2‐brNH‐NH‐Fe6H** with bound *trans* HNNH the stereochemistry at both N atoms and at Fe2 and Fe6 is standard. The next hydrogenation is from 3b2 (item 3 in Figure 7), forming **Fe2‐brNH‐NH2‐Fe6H**. Then (item 4) S2BH is replenished by transfer from HisH. Intermediate **Fe2‐brNH‐NH2‐Fe6H** is ideally suited for the next step, N–N breaking, because the reactant, the transition state (shown in Figure 9) and the product have almost coplanar positioning of 6Hx, N^b^, N^a^, Fe6, and C^c^. This means that the reaction is in effect a standard inversion at N^b^H_2_, with N^b^ separating from N^a^ and forming a bond with 6Hx (item 5 in Figure 7). The angular geometry is ideal: the relatively large energy barrier for this step comes from the breaking of the N^a^—N^b^ bond and of the Fe6—H bond.

Then, after dissociation of this first NH_3_ from *exo* Fe6, the N^a^‐H entity bridging Fe2 and Fe6 in **S2BH‐Fe2‐brNH‐Fe6** is ideally placed and oriented to accept an H from S2BH, which is item 6 in Figure 7. This H on S2B has been obtained from HisH, where it has been stored from the outset of the cycle. The reason for prior placement of H on His, ready to replenish S2BH (Figure 7, item 4), is that the previous intermediates contain N^a^ and N^b^ entities that block H migration from S3B to S2B. Remember that this use of S2BH to form bridging N^a^H_2_ (**S2BH‐Fe2‐brNH‐Fe6** → **Fe2‐brNH2‐Fe6**) is the second occurrence of substrate hydrogenation by S2BH: the first was in the N_2_ capture step. Now, N^a^H_2_ bridging Fe2 and Fe6 is well‐positioned to accept H from 3b2, which has been replenished from the supply chain (item 7, Figure 7), forming the second NH_3_.

It is significant that all hydrogenation steps forming H—N bonds are intramolecular. No external H sources are involved in these steps, consistent with the fact that the desert nature of the surrounding protein is devoid of external H donors [17]. The only external agent is His195, which has a crucial role as proton buffer, but not proton source. This mechanism is under tight internal control, using a relatively small internal part of FeMo‐co.

Two important characteristics of the relationship between architecture and function in the Dance mechanism are the tight control with intramolecular hydrogenation only and the almost ideal geometry and stereochemistry that prevail throughout. This mechanism does not bend any of the rules of chemistry, and is conservative [24].

There is a clear rationale for the H_2_/N_2_ exchange in this mechanism. The N_2_ capture reaction requires use of the endo position of Fe6. The early accumulation of H atoms on FeMo‐co places an H atom at the *endo*‐Fe6 position or the nearby Fe6‐H‐Fe2 bridge position (Scheme 1, lines C, D). This would block the capture step, and so this H needs to be cleared. This can be achieved by converting it to H_2_ in the *endo*‐Fe6 position (line E intermediate **S2BH‐6Hx‐6H2n**), which then upon dissociation leaves the *endo*‐Fe6 position vacant and primed for the crucial N_2_ capture step. Thus, this H_2_/N_2_ exchange is an essential component of the mechanism. This rationale is founded on architecture, not changes in electronic structure at Fe [143].

**FIGURE 10:**
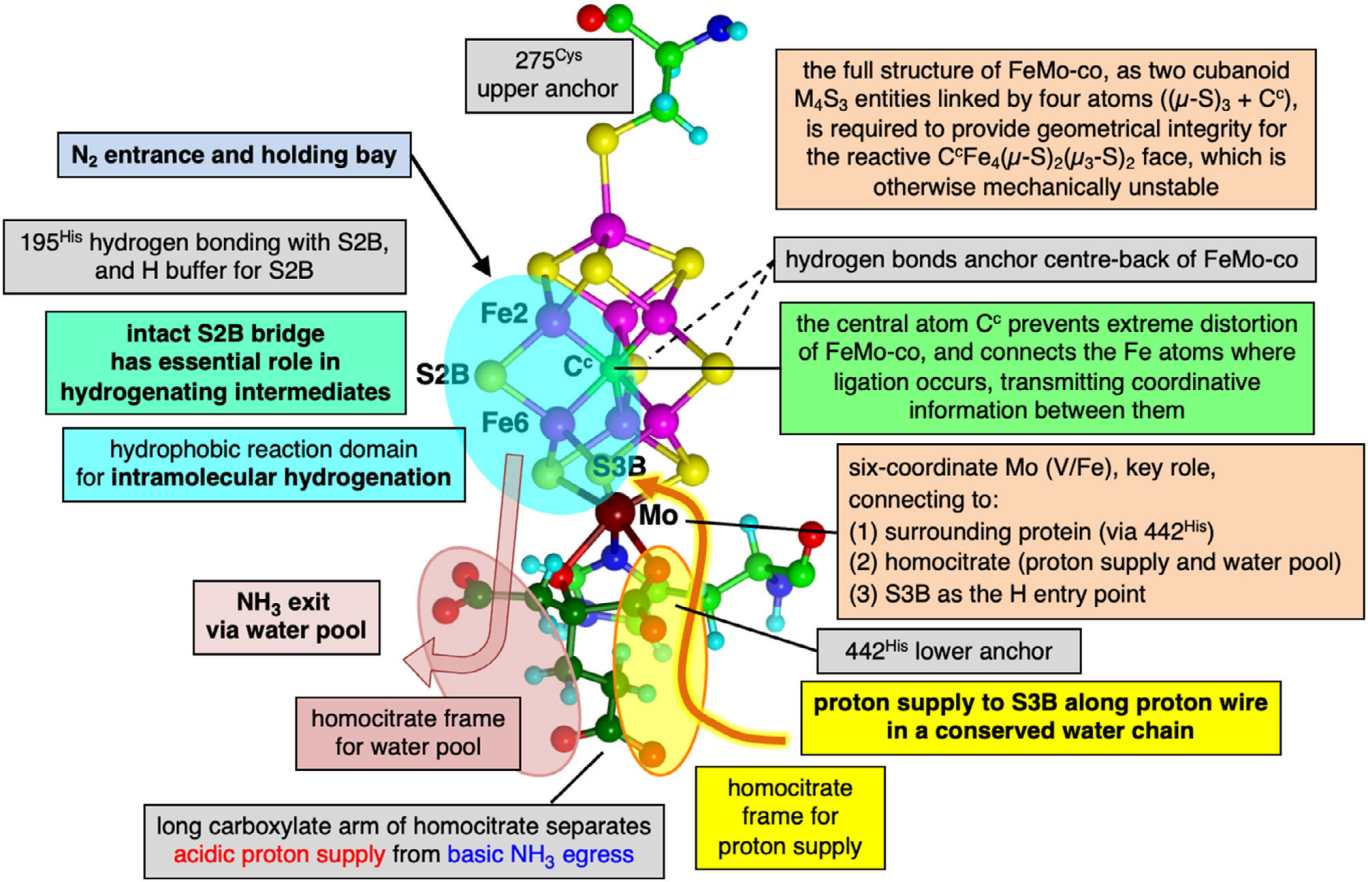
Architecture and function within and around the active site of nitrogenase.

Figure 10 shows the macrostructural components and their functions in and adjacent to the reaction domain. These include structural elements (Mo, C^c^) that form and maintain the FeMo‐co cluster geometry, and structural elements that anchor it to protein surrounds. The opposing directions of proton supply and NH_3_ egress are marked. The long carboxylate arm of homocitrate has an important role in separating the acidic section supplying protons from the basic section and water pool that translocates NH_3_ away from the reaction zone. The immediate environment of the reaction zone is hydrophobic, consistent with the intramolecular hydrogenation character of the mechanism.

The unreactivity of N_2_ derives from its remarkably strong bond (225 kcal mol^−1^), nonpolarity, and huge HOMO–LUMO gap (10.8 eV). The very negative reduction potential for N_2_ + H^+^ + e^−^ = N_2_H, *E* ^0^ = −3.20V versus NHE [9] emphasises the kinetic difficulty at the beginning of the activation of N_2_ on its route to NH_3_. The singular N_2_‐capture step in the Dance mechanism allows the enzyme to snub this difficult initial hydrogenation of N_2_, by concerted formation of five new bonds: two H—N and three Fe—N. In addition, the enzyme adopts a side‐on‐end‐on binding mode, known to be effective in activating and functionalising N_2_ [144].

Almost all of the steps in this mechanism involve movements of H atoms, and therefore the possible occurrence of H atom quantum tunneling needs to be considered. Hydrogenation steps that involve very little movement of atoms heavier than H might benefit kinetically from H atom tunneling. Some relatively crude assessments of computed reaction trajectories have been made, seeking to identify cases where the peak of the potential energy profile involves movement only of H atoms [21, 23, 138]. Expert evaluation of H tunneling is needed.

## 9 Comparisons with Other Mechanisms

As described briefly in Section 4.2, amongst other computational studies there are some mechanistic sequences proposed by other investigators. Here I compare the Dance mechanism with mechanisms CR20 [102], JR22a (Figure 2) [94], JR24a (Figure 3) [99], LHG23 (Figure 4) [100], and R18 (Figure 5) [92].

1. Completeness of cycle. Like the Dance mechanism, the LHG23 mechanism describes a complete cycle. The three Ryde mechanisms (CR20, JR22a, and JR24a) start at the E4 N_2_H_2_ stage and continue to the dissociation of 2NH_3_, and another paper [98] examined N_2_ binding to the E0–E4 states but not mechanistic progression to the N_2_H_2_ intermediate [98].
2. S2B status. The three Ryde mechanisms explore options with S2B absent [102], unhooked [99], or intact [94]. Another Ryde study of proton transfers concluded that it is unlikely that S2B reversibly dissociates during the reaction cycle [107]. The LHG23 mechanism has S2B dissociated as H_2_S through most of the cycle. Intact S2B is a crucial agent in the Dance mechanism.
3. Location of intermediates. Mechanisms CR20, LHG, and R18 contain intermediates bridging Fe2 and Fe6, while JR24a has just one intermediate using this bridging position, all other intermediates being bound at *exo*‐Fe2 or *exo*‐Fe6 positions. All intermediates in JR22a are bound at *exo*‐Fe6. Only the Dance mechanism has both S2B and intermediates simultaneously bridging Fe2 and Fe6.
4. Sources of H. The Dance mechanism includes transport of exogenous protons to S3B [16]. The Ryde group reported an exhaustive study [107] of proton transfers from FeMo‐co atoms S3B, S4B, and S5A to intermediates in the CR20 mechanism (S2B absent) and intermediates analogous to those in mechanism JR24a (Figure 3, S2B present). Computed activation barriers were prohibitively high if S2B was absent. LHG23 invokes the Dance proton supply chain.
5. H donors. The initial papers describing the Ryde mechanisms CR20, JR22a, and JR24a did not describe the H atom donor or H transfer step for each hydrogenation of an intermediate in the sequence, but ref. [107] includes most of this information as reaction and activation energies for H transfer from Fe6‐H or S2B‐H to N. In mechanism JR22a, homocitrate functions as H donor at one step. The Dance mechanism has four H transfer agents, S2B, Fe7, Fe6, and S3B, deployed around intermediates in the reaction space.
6. Reaction energies and reaction barriers. The LHG23 mechanism contains the energies of all 13 intermediates on one scale, by referencing each e^‐^ H^+^ addition as the half energy of H_2_. Therefore reaction energies are available for each step, although the authors acknowledge that this is not fully optimal. Some reaction barrier energies are reported. The reaction energy and activation barrier for the N–N cleavage step and the NH_3_ dissociation steps in mechanisms JR22a and JR24a are reported [94, 99]. The Dance mechanism contains reaction energies and activation barriers for all steps, but does not attempt to put the intermediates on a single energy scale.

## 10 Epilogue

In this perspective, I have shown that it is possible to construct a complete cycle for the nitrogenase catalysis, from resting state to resting state, using steps that embody the chemical essence of the catalytic site. The key aspect of this chemistry is that all reaction steps occur within a small reaction space (Fe2‐S2B‐Fe6‐S3B‐Fe7) where reactivity is under tight control. All key N‐containing intermediates do not stray outside to *exo*‐coordination positions of the Fe atoms where control is surrendered.

The tight compactness of the reaction space enables just three of the surrounding atoms (S2B, S3B, and Fe7) to deploy as the H atom donors in five of the six H—N bond formations. The exception, H donation from 6Hx, appropriately generates NH_3_ outside the internal reaction space at *exo*‐Fe6 (**S2BH‐Fe2‐brNH‐Fe6NH3**), where it is logically positioned for dissociation.

S3B is crucial. It is the enabling conduit for the eight incoming protons, from the proton wire to various atoms on FeMo‐co. S3BH is configurationally athletic, with multiple directions for its S—H bond, and by extending its bonds to Fe7 or Fe6 is able to donate H in multiple ways in this mechanism.

S2B is also crucial. It is connected to Fe2 and Fe6, which support many of the intermediates. As a bridge between Fe2 and Fe6 it is close to all of the intermediates that also bridge Fe2 and Fe6, thereby facilitating H donation from S2BH to N. S2B also connects with the sidechain of His195, a proton buffer, and allows S2BH to form H—N bonds at two stages in the mechanism.

The compactness of the reaction space enables the concerted double hydrogenation of N_2_, from opposite sides (Fe7‐H and S2BH) in the formidable N_2_ capture step, and also facilitates the concurrent formation of three N—Fe bonds. This concurrency avoids the difficulties of the often proposed mechanistic concept of binding N_2_ first and then adding H atoms.

It is hard to conceive of a better role for FeMo‐co as the host for this compact reaction space, particularly as it enables propitious stereochemistry throughout the cycle. But while the stereochemistry is strong there is a weakness, namely inconsistency of some calculated reaction barriers with the experimental kinetics. This needs to be resolved with alternative calculations of the difficult reaction steps. The present description of the Dance chemical mechanism of nitrogenase is intended to invite critical assessment through independent calculations, for which coordinates are provided. Calculations from different authors should lead to further improvements in the understanding of the chemical catalysis effected by this marvellous enzyme.

## Conflicts of Interest

The author declares no conflicts of interest.

## Supporting information

File Complete_Mechanism_Nitrogenase_SuppInfo.docx describes 1. Protein model, 2. Computational constraints, 3. Density functional procedures, 4. Validation, 5. Determination of transition states, 6. Energetically favorable electronic states, 7. Movement of Val70 and the contiguous chain, 8. Libratory movement of the Arg96 sidechain, 9. Non‐obligatory H_2_ evolution, 10. Truncated models for quantum tunneling calculations. File Mechanism_Mo‐nitrogenase_coordinates.docx contains atom coordinates and Fe spin populations for reactant, TS and product in each reactions step, and for alternative electronic states.

Supplementary Material

## Data Availability

File Mechanism_Mo‐nitrogenase_coordinates.docx contains atom coordinates and Fe spin populations for reactant, TS and product in each reaction step, and for alternative electronic states.
