# Supplementary material for: A Proposed Complete‐Cycle Mechanism for Conversion of N2 to NH3 by Mo‐Nitrogenase
Source: Chembiochem. 2026 Jul 24;27(14):e70429. doi: 10.1002/cbic.70429 (PMC13398036; doi:10.1002/cbic.70429)
Supplement: Supplementary file 1 — File Complete_Mechanism_Nitrogenase_SuppInfo.docx describes 1. Protein model, 2. Computational constraints, 3. Density functional procedures, 4. Validation, 5. Determination of transition states, 6. Energetically favorable electronic states, 7. Movement of Val70 and the contiguous chain, 8. Libratory movement of the Arg96 sidechain, 9. Non‐obligatory H2 evolution, 10. Truncated models for quantum tunneling calculations. File Mechanism_Mo‐nitrogenase_coordinates.docx contains atom coordinates and Fe spin populations for reactant, TS and product in each reactions step, and for alternative electronic states. [file CBIC-27-e70429-s002.pdf]

A Proposed Complete-cycle Mechanism for Conversion of N_2 to NH_3 by Mo-Nitrogenase

Ian Dance

School of Chemistry, UNSW Sydney, NSW 2052, Australia

E-mail: i.dance@unsw.edu.au

Supplementary Information

Atomic coordinates and Fe spin densities for reactants, transition states and products of the reaction steps

Intermediates are labelled as in Scheme 1 of the main article.

Translation of Fe numbering

Fe1 = Fe143

Fe2 = Fe140

Fe3 = Fe141

Fe4 = Fe142

Fe5 = Fe139

Fe6 = Fe145

Fe7 = Fe144

Table format

**Fe spin densities**	**filename**<br>**coordinates**

S2BH-26H-3b5 to S2BH-26H-3b3

35, S=3/2

reactant

Fe spin densities	bm522bh263b4a.car_4			
Fe(139) -2.346	C1	-7.954591863	-4.755005255	2.541171149
Fe(140) 1.046	C2	-8.574526959	-5.906479140	1.741525358
Fe(141) -2.500	C3	-8.389397666	-2.672098883	3.763440316
Fe(142) 2.309	C4	-7.541610577	-2.898433346	5.012294079
Fe(143) 2.891	C5	-6.977353653	-4.365094855	6.881815157
Fe(144) 2.364	C6	-5.488780540	-4.583946734	6.596641193
Fe(145) -1.181	C7	-3.773540132	-5.195855302	4.972478035
	C8	-3.106178018	-3.837791970	4.745607059
	C9	-3.356050873	-1.547886776	3.934334848
	C10	-3.049752987	-0.827559247	5.255721510
	C11	-3.758272223	-0.315213959	7.519985975
	C12	-2.663390022	-0.943506153	8.391165941
	C13	-1.820861912	-2.984086813	9.448034341
	C14	-0.444679654	-3.394747052	8.908103943
	C15	0.801669962	-4.294646591	6.987303525
	C16	1.587864506	-3.345039613	6.079632670
	C17	1.485175202	-1.373368674	4.572064708
	C18	1.962845291	-0.151568820	5.390148910
	C19	0.540859312	-0.995006331	3.410796597
	C20	1.139250833	0.137187456	2.569436860
	C21	0.230801036	-2.231040545	2.559333796
	C22	1.314663903	1.724302453	6.861912533
	C23	1.800568370	1.395530156	8.290438902
	C24	0.111074546	2.700795589	6.862706299

	C25	0.367010737	3.898453817	7.785269772
	C26	-0.199809171	3.181491665	5.439369112
	C27	1.365119762	0.335704341	10.436335267
	C28	2.798362084	-0.114869298	10.624012901
	C29	5.590323349	-11.810228074	3.803787590
	C30	6.154629797	-10.601531060	3.057115888
	C31	6.611728339	-8.159771632	3.302518148
	C32	7.951450667	-7.845645909	3.996191427
	C33	5.564282882	-7.071195136	3.597599051
	C34	5.997079689	-5.678344030	3.135996820
	C35	4.852598636	-4.671329109	3.236618275
	C36	5.076688457	-2.160635318	3.191185730
	C37	10.267641538	-7.121773371	3.667426376
	C38	10.603630471	-5.651139841	3.560474179
	C39	-5.726481322	-2.365727068	-0.727683012
	C40	-5.914690000	-0.889560086	-0.330763274
	C41	-4.206669993	-2.631577327	-0.837307867
	C42	-3.792130428	-4.113675389	-0.935128584
	C43	-3.716673692	-4.683608594	-2.351291895
	C44	-6.298935002	1.347407092	-1.294421327
	C45	-7.232790277	1.934892394	-0.226241858
	C46	-6.590373159	1.905192056	-2.694384062
	C47	-9.282531752	1.627626060	1.084852534
	C48	-8.702673937	1.685567675	2.502957531
	C49	-6.846835935	1.092907523	3.995612786
	C50	-5.591452808	1.963003188	4.019293749
	C51	-4.106120675	3.515056161	2.859114363
	C52	-4.406482651	4.861208684	3.558930041
	C53	-3.673727975	3.887640546	1.433763396
	C54	-3.136104372	2.795194553	0.573074401
	C55	-2.628887616	1.541391163	0.811630010
	C56	-2.474124826	1.924222729	-1.373637486
	C57	-6.089046818	6.648458090	3.770986731
	C58	-5.843760282	6.868452099	5.262669056
	C59	-7.592693271	6.787303374	3.435896024
	C60	-8.133511318	8.183063468	3.326444008
	C61	-7.882145093	9.313711301	4.080274023
	C62	-9.363051798	9.756577825	2.492256499
	C63	9.621123800	1.108197935	2.368085732
	C64	10.552537262	-0.062969130	2.115942840
	C65	8.178073649	0.544537443	2.373153131
	C66	7.132751467	1.605302602	2.627389477
	C67	6.731778724	1.937727407	3.935047994
	C68	6.521929679	2.285637340	1.560107560
	C69	5.745032633	2.900491352	4.169048421
	C70	5.545987610	3.257164951	1.778546394
	C71	5.148733692	3.555481548	3.085265265
	C72	5.495638492	6.380768603	-2.279839311
	C73	4.498457667	7.542283153	-2.427199233
	C74	4.784605714	5.149969622	-1.724875132
	C75	3.411297554	9.105847410	-4.002352617
	C76	1.910403674	8.806211383	-3.985891807
	C77	0.195298277	7.024501081	-3.671415255
	C78	-0.190474500	6.766595284	-2.192736560
	C79	0.074217605	5.749357855	-4.537549315
	C80	-1.229978601	4.945377093	-4.490308210
	C81	-2.433948199	5.534991894	-5.243027532
	C82	-3.724725169	7.598155777	-4.678612367
	C83	0.431508896	5.918148751	0.012114471
	C84	0.557920971	7.184139444	0.880536880
	C85	1.306688672	4.817649535	0.637042915
	C86	1.941218471	9.154242307	1.348955189
	C87	1.383600636	10.541926965	1.061414328
	C88	-0.099119207	11.891173769	-0.334006455
	C89	-1.235653535	12.576786628	0.465340503
	C90	-0.445611133	11.861891948	-1.826404097
	C91	-1.704296388	11.065187173	-2.146911816
	C92	-3.039700079	12.306361085	2.101994832
	C93	-2.770981410	13.450821215	3.069420844
	C94	-3.705346333	11.115604113	2.844640994
	C95	-4.142623883	10.021491261	1.895941363
	C96	-5.405438882	10.076340343	1.284408722
	C97	-3.288659867	8.959603611	1.552683559
	C98	-5.797584217	9.121602043	0.342260770
	C99	-3.650401005	8.012548062	0.588485676
	C100	-4.902529000	8.110442650	-0.011269520
	C101	2.712851412	-4.589065196	-9.095398366
	C102	1.373096917	-4.220472792	-8.481191026
	C103	0.187636736	-2.546511669	-7.078874173
	C104	-0.299117584	-1.200305729	-7.628136512

C105	0.222380364	1.198989447	-7.774477383
C106	1.432211120	2.069782030	-8.104458714
C107	3.811399435	2.535891839	-7.879560053
C108	4.236635531	2.630452906	-9.332691733
C109	-4.052753525	-3.683057999	-8.518209081
C110	-4.958055421	-2.510029651	-8.124684462
C111	-3.473848938	-4.341976965	-7.244471581
C112	-2.883765603	-5.730754004	-7.552216312
C113	-2.092617719	-6.245399389	-6.370843421
C114	-5.131031191	-0.140129009	-7.549431171
C115	-4.661760757	1.089317419	-8.301162957
C116	-5.281205946	0.051938538	-6.019876408
C117	-3.957738282	-0.041905433	-5.310671688
C118	-3.093957132	1.064484846	-5.246840372
C119	-3.514025214	-1.276972804	-4.809731426
C120	-1.798783451	0.917530656	-4.741460494
C121	-2.219716951	-1.423423634	-4.302366398
C122	-1.350887787	-0.328096500	-4.292044735
C123	6.766480094	-6.963430307	-5.231129406
C124	8.055892588	-7.094099751	-4.403529859
C125	5.532309849	-7.144725679	-4.332401760
C126	4.204429358	-6.871311349	-4.964566310
C127	3.659684384	-7.488875548	-6.071346872
C128	2.124019744	-6.206305596	-5.125904939
C129	9.412059807	-8.366052504	-2.848213724
C130	10.678775239	-8.219887062	-3.661779515
C131	0.022747543	-6.916005914	0.195291527
C132	1.534378175	-7.082974244	0.342987936
C133	2.291696645	-7.289712938	-1.005463334
C134	2.041706893	-8.734094787	-1.519626528
C135	2.930128512	-9.186230396	-2.681157813
C136	2.804671971	-10.686876851	-3.030919286
C137	3.802175649	-7.120193215	-0.769791752
C138	3.179132611	-1.076054861	-2.440104203
Fe139	3.210966109	-2.395737505	-3.944881176
Fe140	1.959382798	0.125138744	-1.562666998
Fe141	4.563520554	0.127253446	-1.650804108
Fe142	3.155651929	0.216199115	-3.895077958
Fe143	3.391850966	2.375225756	-2.365658033
Fe144	4.443683045	-2.457530506	-1.660798539
Fe145	1.872412067	-2.286760434	-1.755105702
H146	-6.891665040	-7.040367716	1.521634508
H147	-7.219220481	-6.105197815	0.225183225
H148	-9.830560166	-3.971044355	2.872220727
H149	-9.370693151	-5.500221053	1.097123422
H150	-9.078570193	-6.564879593	2.469675038
H151	-7.780489810	-2.065174483	3.077367488
H152	-8.471566521	-4.688188885	5.367411733
H153	-9.269930641	-2.080904449	4.048916736
H154	-7.388121961	-5.277397429	7.334489800
H155	-5.891663810	-5.187662687	4.664032827
H156	-7.039908241	-3.557364817	7.623427734
H157	-2.425863382	-1.558466442	3.353611156
H158	-4.803005362	-3.128849066	3.821863220
H159	-3.723136260	-5.783516589	4.046399089
H160	-3.478001860	0.732369611	7.349846248
H161	-4.834620079	-1.476101217	6.058303971
H162	-3.193130513	-5.714776872	5.744717905
H163	-4.109423970	-0.984398174	3.365697395
H164	-3.453462219	-2.829833002	8.072713577
H165	-4.707824509	-0.338271434	8.072132357
H166	-0.112357624	-3.077117366	3.171474249
H167	-0.573677572	-2.012191687	1.839449165
H168	1.115579165	-2.553371629	1.988026686
H169	-0.396592699	-0.631644773	3.864112794
H170	-2.327005835	-3.899331836	9.788649185
H171	-0.415721353	2.348881321	4.754795085
H172	0.107821316	0.255251749	8.722894022
H173	0.529812766	3.598853757	8.830985602
H174	2.071802229	-0.173257012	2.074835498
H175	1.356735113	1.032083975	3.171103569
H176	9.838344190	1.498937956	3.380502808
H177	-1.075118788	3.847916621	5.447514352
H178	-0.498587076	4.576575358	7.761004110
H179	0.652129199	3.751454253	5.033414288
H180	1.251456805	4.465572691	7.454440012
H181	-0.765751200	2.148860023	7.246747880
H182	2.177977679	2.211003992	6.385368218
H183	0.028018153	0.246839244	5.978481793
H184	0.439775921	0.444353085	1.777740234

H185	1.321943692	3.941881381	-0.036151410
H186	0.829582518	4.523197273	1.584901007
H187	2.971204338	4.932271704	1.701339216
H188	3.956071865	4.612169720	4.192079339
H189	5.064357945	3.760366645	0.941029914
H190	6.792640448	2.028494628	0.533428567
H191	7.181583598	1.423473380	4.787950915
H192	-0.133940497	-2.350268964	5.586740940
H193	0.545433922	-5.180919097	6.386362843
H194	1.486322495	-4.609275056	7.783797055
H195	-1.254800705	-3.755911900	7.032713747
H196	-1.637656298	-2.346374217	10.321230737
H197	5.426442618	3.123753798	5.190536873
H198	2.392232487	-1.827809611	4.158259361
H199	1.181111438	1.220787326	11.067022633
H200	0.724158094	-0.484348413	10.797079530
H201	3.239446195	-0.667780451	9.763094181
H202	11.047087556	-7.715478271	3.165876587
H203	-2.687034112	-6.036437229	-3.491558442
H204	0.558970453	-6.137639878	-1.498271890
H205	-3.703853705	-6.436013094	-7.749904058
H206	-2.243232848	-5.684017692	-8.445002763
H207	-4.261401382	-4.450759471	-6.483304114
H208	-4.237781420	0.877557496	-9.310468881
H209	-4.747496258	-4.404565703	-8.972755006
H210	-3.816350953	-2.072029402	-1.703035017
H211	-4.503344416	-4.743276305	-0.373281171
H212	-3.336000709	-1.215619695	-8.035751332
H213	-6.147806966	-0.322968811	-7.952299842
H214	-2.308499341	-2.757584764	-9.166597567
H215	-2.052416424	-5.846756398	-1.860253409
H216	-2.709785104	-3.676445957	-6.812889946
H217	-2.686277984	-4.138444082	-9.979480163
H218	-7.333241650	-3.079491754	-1.811837429
H219	-3.738154539	-2.205343599	0.061887837
H220	-0.457259873	-5.633283570	-7.275556718
H221	-2.818718827	-4.251487838	-0.442485998
H222	-3.280001872	3.057027577	3.420228737
H223	-2.525281324	0.979816199	1.731230426
H224	-7.541949796	1.522183779	4.730485977
H225	-7.085108509	0.497424896	1.960061077
H226	-6.368975717	4.675874311	2.987162182
H227	-7.221861627	9.500877788	4.919484403
H228	-6.009197620	5.956733963	5.886849763
H229	-8.177613969	6.194929921	4.162572527
H230	-5.499393916	7.388752576	3.206647730
H231	-10.062130571	10.327392588	1.886616397
H232	-2.901542037	4.668210957	1.533192284
H233	-4.529430872	4.351393332	0.916900056
H234	-8.725912327	11.265528944	3.852096783
H235	-7.755290416	6.315930330	2.453508086
H236	-5.839299884	2.539198491	2.031864450
H237	-6.574143870	0.091167339	4.359273685
H238	-9.686382837	2.628768092	0.879811425
H239	-2.259601372	1.780607231	-2.429069343
H240	-10.123982479	0.923114988	1.084593656
H241	10.671733920	2.596459078	1.429055750
H242	9.050518619	2.784600885	1.342512194
H243	1.595158442	5.737339231	-1.799000004
H244	-0.620447954	5.607577461	0.093670210
H245	2.123004203	7.829865531	-0.289022945
H246	-3.419586428	3.834685194	-1.311593849
H247	-5.127793593	4.408899296	-2.347434825
H248	-6.922062453	3.673704223	-3.474776917
H249	-4.661410816	5.699357682	-1.540789221
H250	-5.870477337	1.460308435	-3.400103311
H251	-7.610308400	1.624694448	-2.999851917
H252	0.419694818	11.505156426	-2.402399444
H253	-0.638746224	12.902839819	-2.129161498
H254	-0.700324656	10.027867658	-3.640069667
H255	-2.396988840	9.620603862	-3.373526806
H256	-3.733382156	9.148996802	-6.025904120
H257	-3.056299730	4.720138822	-5.643934521
H258	-6.789238992	9.167353920	-0.114652962
H259	-2.315678347	8.845767272	2.038182877
H260	-4.476667599	7.932003166	-2.787867341
H261	0.886196657	5.069867043	-4.233175389
H262	2.317609194	6.825073430	-3.715335219
H263	-1.515141699	4.721099312	-3.449823910
H264	-8.488067819	0.339472443	-0.438692025

H265	3.661279400	9.492369672	-4.998657020
H266	-6.492898935	-0.646063964	-2.223474897
H267	0.286525381	6.037096954	-5.580489884
H268	-2.945349845	7.239300170	0.282895834
H269	3.572649102	9.917621780	-3.278318576
H270	-3.203536299	7.629435465	-6.677045359
H271	-4.970101174	9.066749968	-3.992373132
H272	-5.854233601	-3.301824190	-2.557609196
H273	-6.110295815	-2.956473470	0.124713174
H274	-6.102195156	10.875931372	1.547290476
H275	-5.294569355	1.673509867	-0.984877388
H276	-0.481002343	7.810019033	-4.023085134
H277	-2.076574123	6.119052293	-6.100859211
H278	-1.011119473	3.975903399	-4.960267094
H279	-3.620464719	5.933538243	-3.519775084
H280	1.677429613	8.901651184	2.386838871
H281	0.091259573	9.746979244	-0.311075380
H282	0.752422867	12.577970583	-0.208284682
H283	3.035140374	9.231609888	1.284547931
H284	-1.566127403	10.870535743	1.515039751
H285	3.268430208	-3.677574166	-9.359414864
H286	3.285532280	-5.087327854	-8.285308915
H287	6.778985765	-5.933722815	-5.617698370
H288	3.420047520	-5.817629314	-10.557750713
H289	1.908494610	-6.195690127	-10.052873569
H290	5.518269922	4.337477225	-1.652268126
H291	6.219474359	7.091752559	-0.502637811
H292	4.789150168	7.543265683	-4.462535852
H293	4.374469832	5.368384683	-0.731114978
H294	7.149451675	7.502472015	-1.787189693
H295	4.654855932	2.108902269	-7.314549961
H296	5.923946202	6.135680749	-3.264402439
H297	5.353722744	-7.066218732	4.680506406
H298	6.760966521	-8.231380649	2.218204532
H299	2.795431123	0.818644832	-7.133131559
H300	5.304822024	-0.160554923	2.824154600
H301	-0.391906844	1.128900177	-8.680644344
H302	-1.883664229	-2.391432787	-3.924093381
H303	-0.374680882	1.758028482	-7.033445670
H304	4.549139570	-4.528300252	4.280280361
H305	1.222259138	-0.319800131	-6.598299532
H306	3.965710931	-5.056153472	2.704513417
H307	4.637574818	-7.368187808	3.080836803
H308	6.333725435	-5.718397480	2.089293520
H309	6.845633510	-5.313760785	3.736575190
H310	-5.968915286	-0.724260644	-5.656128730
H311	5.811556022	-9.561779688	4.731194926
H312	4.602290214	-12.002269596	3.357033275
H313	-0.324500914	-0.430570546	-3.938920152
H314	6.277683243	-11.862861310	5.743166977
H315	4.673023605	-12.166115013	5.610294216
H316	5.509140759	-3.406428982	1.666191663
H317	-1.111740157	1.766616207	-4.708760618
H318	-4.185018340	-2.139253517	-4.819555967
H319	6.644176248	-10.558191600	-1.964594261
H320	5.514516345	-8.184581028	-3.962386308
H321	5.937866642	-8.674164422	-0.371412740
H322	5.607470017	-11.107091806	-2.982383948
H323	3.871411617	1.801729112	-9.985271406
H324	5.932210038	-5.530382192	-0.699654647
H325	1.742202639	-7.903902740	1.040369087
H326	2.211193938	-2.521770499	-7.708071991
H327	2.211294370	-9.405136202	-0.663090385
H328	1.658057926	-7.368878955	-6.821690558
H329	1.162003753	-5.744138521	-4.946150600
H330	6.229841380	-12.669776025	3.539212860
H331	5.414752238	-1.164265912	1.396973914
H332	7.979853643	-6.394595528	0.133312242
H333	6.925901486	-4.341957535	-0.816063619
H334	8.772656109	-7.377522956	2.137189062
H335	4.433220862	-2.761457010	5.036337283
H336	2.670272028	-8.617154716	-3.585973604
H337	3.985672527	-8.977573372	-2.458697196
H338	0.980481856	-8.831147585	-1.797177214
H339	0.453998300	-2.459297621	-6.014022695
H340	-3.430525013	2.030084003	-5.631000243
H341	-5.755185694	1.033221028	-5.864515890
H342	1.885010410	-6.150498473	0.816514606
H343	7.971077498	-7.946990038	0.029143208
H344	6.736896398	-9.763122690	0.382593126

	H345	4.404870592	-1.086569242	4.784123201
	H346	4.086335264	-8.204129437	-6.763112546
	H347	5.639898751	-6.509680884	-3.445136919
	H348	-0.621841807	-3.271221153	-7.191139390
	H349	3.627926773	3.549394573	-7.491884525
	H350	8.124753951	-0.233095590	3.149970920
	H351	7.988928060	0.062189587	1.400616722
	H352	7.564969436	-7.688066252	-6.974361492
	H353	6.823216331	-8.842961786	-6.083289453
	H354	9.409883593	-9.382896180	-2.424490521
	H355	7.506044593	-8.956455251	-3.600871639
	H356	10.572495041	-8.479146471	-4.742504008
	H357	9.426664154	-7.658206110	-2.004517945
	H358	10.630217627	-0.358104025	1.041030647
	H359	-1.706509479	13.571099820	3.381067754
	H360	10.282110243	-7.377986305	4.742218350
	H361	9.722600449	-4.964527302	3.548288040
	H362	-3.736388107	12.682899504	1.334590045
	H363	-2.998453942	10.718155771	3.589916657
	H364	-4.570335571	11.513120838	3.394308615
	H365	3.798118373	-4.729693840	0.410965494
	H366	-6.197439910	7.217145590	-1.165148361
	H367	0.025910344	-1.272989427	0.029867288
	H368	2.170599048	-1.026640727	-0.453073103
	H369	-1.715582487	0.150398953	-0.603144206
	Mo370	3.138449048	-4.692885492	-2.489583960
	N371	-7.657394320	-6.694915277	0.937149301
	N372	-8.845349876	-3.890980470	3.112727290
	N373	-7.763846870	-4.039975602	5.704337324
	N374	-5.167238276	-5.081848165	5.376247372
	N375	-3.853921945	-2.903172077	4.111580548
	N376	-3.954601686	-0.991495304	6.247553909
	N377	-2.705183108	-2.294083111	8.521535657
	N378	-0.402712377	-3.762803430	7.597024885
	N379	0.877651005	-2.370209400	5.457410253
	N380	1.007393482	0.525043600	6.077485738
	N381	1.028927893	0.567494571	9.039626106
	N382	5.429258934	-11.585197456	5.245173342
	N383	6.126475112	-9.452373268	3.761420506
	N384	5.219631435	-3.380453049	2.645239533
	N385	4.837014463	-1.984643870	4.506725262
	N386	5.191768658	-1.079435202	2.403751756
	N387	8.945965644	-7.444956282	3.164173304
	N388	-6.423247386	-2.649220834	-1.987739671
	N389	-2.757665991	-5.603828156	-2.564417963
	N390	-6.336085596	-0.103839180	-1.356915067
	N391	-8.354009633	1.226776428	0.041116656
	N392	-7.542673417	1.005597132	2.724812554
	N393	-5.242371672	2.603048917	2.862058768
	N394	-3.022707073	3.001136999	-0.795677495
	N395	-2.236410935	1.030049044	-0.410013242
	N396	-5.683919893	5.295886685	3.412246623
	N397	-9.058576195	8.473347671	2.336590235
	N398	-8.671247628	10.302439066	3.531760967
	N399	9.796433815	2.085507324	1.291112257
	N400	6.608021709	6.733310125	-1.381329163
	N401	4.287063255	7.988940993	-3.699512966
	N402	1.563286331	7.514218600	-3.811964592
	N403	-3.281713493	6.375576798	-4.392434029
	N404	-3.555148503	8.159519523	-5.888088207
	N405	-4.314812054	8.341658022	-3.711101564
	N406	0.731504157	6.153588078	-1.399493781
	N407	1.485312563	8.093664866	0.467439971
	N408	0.340363906	10.605384758	0.178814675
	N409	-1.619109250	10.266328681	-3.244266398
	N410	-1.807285272	11.857279323	1.464087033
	N411	2.520538031	-5.410368810	-10.292494396
	N412	1.338547294	-3.041275024	-7.823494002
	N413	0.486607640	-0.149215962	-7.303732059
	N414	2.659396673	1.672310867	-7.683633125
	N415	-3.088766232	-3.299717898	-9.556754489
	N416	-4.353201099	-1.299803699	-7.947109383
	N417	6.741930275	-7.869551655	-6.392986611
	N418	3.226301693	-6.056203621	-4.390247303
	N419	2.355639519	-7.055052032	-6.152710274
	N420	8.219870525	-8.226296792	-3.666501667
	O421	-6.729433465	-4.624930741	2.706107225
	O422	-6.695193696	-2.056133459	5.361384021
	O423	-4.638420534	-4.326312963	7.464689609
	O424	-1.944875507	-3.608596742	5.126138287

	O425 -2.027941154 -0.132930340 5.386080300<br>O426 -1.822028283 -0.234521765 8.971317781<br>O427 0.531524254 -3.428135583 9.668403673<br>O428 2.807298057 -3.530303180 5.921111692<br>O429 3.162778937 0.184028103 5.406622844<br>O430 2.847233060 1.894580011 8.736726607<br>O431 3.410456351 0.039386034 11.671229813<br>O432 6.563388403 -10.730874461 1.884483834<br>O433 8.072265606 -7.953010293 5.228524520<br>O434 11.745657413 -5.222118171 3.508023077<br>O435 -5.678949280 -0.453969319 0.813739814<br>O436 -4.517136246 -4.336356732 -3.250731674<br>O437 -6.939848329 2.992403513 0.360287840<br>O438 -6.467187568 3.341242242 -2.680480234<br>O439 -9.306973683 2.324230842 3.374385250<br>O440 -4.935463588 2.072809139 5.061464280<br>O441 -3.520615877 5.523423826 4.105961656<br>O442 -5.541481871 7.939556691 5.760303439<br>O443 11.162691132 -0.664431671 2.987661829<br>O444 4.143733156 4.491913122 3.243382463<br>O445 3.923782997 8.051774830 -1.449072580<br>O446 1.103535065 9.744313990 -4.139383534<br>O447 -1.311516313 7.109995653 -1.780408146<br>O448 -0.125144999 7.313709672 1.904948254<br>O449 2.630706222 5.304194472 0.862992224<br>O450 1.858200972 11.538458466 1.620476432<br>O451 -1.553829769 13.748300426 0.219471328<br>O452 -2.740515271 11.181290263 -1.475674192<br>O453 -3.652387171 14.178907270 3.498203768<br>O454 -5.232118649 7.181292051 -1.020358348<br>O455 0.394256239 -5.000865564 -8.563333824<br>O456 -1.321640247 -1.104100564 -8.336679293<br>O457 1.255762802 3.139029972 -8.711980559<br>O458 4.956065813 3.516906148 -9.763830546<br>O459 -6.177643681 -2.667578908 -7.951989647<br>O460 -2.606764615 -6.760458280 -5.384125915<br>O461 -0.756226821 -6.065515885 -6.417687390<br>O462 -4.768086632 2.236346869 -7.889938582<br>O463 8.944704479 -6.221607132 -4.437373231<br>O464 11.762151969 -7.915266258 -3.182939565<br>O465 -0.361769567 -6.261486357 -0.905436917<br>O466 -0.778821696 -7.297149364 1.046972350<br>O467 3.887057966 -11.310927554 -3.312356521<br>O468 1.643924043 -11.185123929 -3.049229777<br>O469 4.390093707 -6.173869348 -1.455671259<br>O470 4.403350062 -7.867758136 0.025458476<br>O471 1.836064824 -6.295053095 -1.926339192<br>O472 8.523517224 -7.188377937 0.364385020<br>O473 6.690031680 -5.089505678 -0.235858032<br>O474 -4.334949678 4.970696707 -2.127271235<br>O475 6.591066020 -11.084593063 -2.792629010<br>O476 6.780082551 -9.199266865 -0.430455816<br>S477 3.371755453 4.615652591 -2.799890738<br>S478 1.474672576 1.583533995 -3.274651833<br>S479 1.317317925 -3.541884863 -3.484158333<br>S480 3.309264071 1.412149444 -0.311671304<br>S481 -0.047287214 -1.074212793 -1.309428901<br>S482 3.232849756 -1.064314986 -5.709522231<br>S483 3.136551390 -3.689205835 -0.229359514<br>S484 5.071317081 1.326116139 -3.462420288<br>S485 5.039473219 -3.633102705 -3.453360802<br>S486 6.056178804 -1.259393072 -0.776223221<br>end

TS

Fe(139) -2.412<br>Fe(140) 0.888<br>Fe(141) -2.482<br>Fe(142) 2.599<br>Fe(143) 1.782<br>Fe(144) 2.398<br>Fe(145) 0.141	bm522bh263b4d.car_1<br>C1 -7.944799897 -4.748502158 2.524232733<br>C2 -8.569728760 -5.900208707 1.728261656<br>C3 -8.380198359 -2.669690131 3.753363723<br>C4 -7.535194063 -2.894597644 5.004053262<br>C5 -6.973569545 -4.359124285 6.875566808<br>C6 -5.484879443 -4.576994729 6.591179488<br>C7 -3.769004701 -5.188262946 4.966768967<br>C8 -3.101285503 -3.829711418 4.744019530<br>C9 -3.352072418 -1.538305734 3.937167008<br>C10 -3.044523625 -0.818585071 5.258759680<br>C11 -3.749004077 -0.305531185 7.522895792<br>C12 -2.654026452 -0.935339581 8.393027498<br>C13 -1.816267467 -2.979389600 9.448299473<br>C14 -0.441220644 -3.399810300 8.913030920

	C15	0.812595653	-4.286778286	6.991518967
	C16	1.594781953	-3.341962262	6.075512671
	C17	1.489650221	-1.367731126	4.567885189
	C18	1.970922166	-0.150555747	5.391210193
	C19	0.536540495	-0.985097220	3.416356968
	C20	1.120410415	0.154024459	2.573606291
	C21	0.225047314	-2.218723126	2.563124412
	C22	1.325555844	1.728753188	6.860714850
	C23	1.812668281	1.397090537	8.287155114
	C24	0.122519053	2.705665865	6.867303772
	C25	0.381812929	3.900276244	7.793111003
	C26	-0.192579696	3.190309336	5.446470398
	C27	1.373257004	0.344915693	10.434066701
	C28	2.808201364	-0.097540143	10.626188963
	C29	5.592437185	-11.805205337	3.799265389
	C30	6.152554456	-10.594509190	3.053469816
	C31	6.610287554	-8.152637387	3.301574714
	C32	7.946773545	-7.834442706	3.999476926
	C33	5.556629460	-7.068126857	3.590211127
	C34	5.980374858	-5.673259553	3.123943186
	C35	4.820073206	-4.681484311	3.200041473
	C36	5.062746059	-2.170392393	3.171901393
	C37	10.266884574	-7.122659024	3.674430416
	C38	10.605273130	-5.652246737	3.569796098
	C39	-5.709019686	-2.363593648	-0.725432955
	C40	-5.904087363	-0.888863840	-0.326892342
	C41	-4.190057413	-2.626478686	-0.840217829
	C42	-3.774361040	-4.108361636	-0.941627749
	C43	-3.708423218	-4.679119525	-2.357918745
	C44	-6.307558521	1.346104788	-1.287312645
	C45	-7.246124398	1.927061159	-0.219763756
	C46	-6.600036841	1.903849984	-2.687132755
	C47	-9.293477428	1.605511805	1.091389352
	C48	-8.712106470	1.669707018	2.508496241
	C49	-6.851517439	1.090071110	4.000077794
	C50	-5.594727707	1.958183513	4.018066745
	C51	-4.110469427	3.506253091	2.850766330
	C52	-4.407255575	4.855700049	3.546427886
	C53	-3.677974565	3.869153183	1.423084126
	C54	-3.150231935	2.767882869	0.565965060
	C55	-2.669355914	1.504633288	0.807809082
	C56	-2.490930357	1.883426479	-1.377156626
	C57	-6.090557887	6.641774200	3.764714645
	C58	-5.851852455	6.861675912	5.257306743
	C59	-7.593641536	6.782372057	3.426790220
	C60	-8.136508042	8.177619069	3.317629661
	C61	-7.892289024	9.308287116	4.074044873
	C62	-9.368506885	9.748079216	2.480783797
	C63	9.626701599	1.107299242	2.385152676
	C64	10.556256957	-0.065707197	2.137404128
	C65	8.182121859	0.544857936	2.388238346
	C66	7.135993473	1.605623636	2.637990902
	C67	6.727800361	1.934825569	3.944113927
	C68	6.532343821	2.289974643	1.568799137
	C69	5.743428113	2.900607315	4.175119846
	C70	5.557348752	3.263129742	1.783907809
	C71	5.156000954	3.561223904	3.089776599
	C72	5.519579707	6.388063950	-2.278486734
	C73	4.514700948	7.540085551	-2.431760578
	C74	4.819542948	5.153564470	-1.714685599
	C75	3.431160427	9.101280532	-4.010047387
	C76	1.929571106	8.808930157	-3.982400354
	C77	0.210128296	7.034229986	-3.672071697
	C78	-0.165788858	6.780387037	-2.190352708
	C79	0.082192703	5.753652447	-4.531120324
	C80	-1.223120314	4.952311863	-4.473372983
	C81	-2.428273640	5.538197009	-5.227466792
	C82	-3.726150637	7.599037761	-4.662168284
	C83	0.461687137	5.931564075	0.010225921
	C84	0.584325702	7.200219936	0.873609180
	C85	1.343484701	4.837174461	0.637645062
	C86	1.958327369	9.176433848	1.336178997
	C87	1.386997626	10.559179815	1.051694397
	C88	-0.105497625	11.896816534	-0.343291073
	C89	-1.243977455	12.578947725	0.454577919
	C90	-0.452678867	11.862215738	-1.835575481
	C91	-1.707976215	11.059107291	-2.152517881
	C92	-3.042377056	12.308686208	2.097992814
	C93	-2.774617423	13.451247667	3.067070428
	C94	-3.709325932	11.116743946	2.838579127

	C95	-4.144259337	10.020975647	1.891184196
	C96	-5.408946102	10.069421753	1.282774314
	C97	-3.286275876	8.962425060	1.547590606
	C98	-5.799365413	9.110508445	0.344165930
	C99	-3.645936279	8.011684504	0.586255314
	C100	-4.900813863	8.102419044	-0.009237772
	C101	2.720658530	-4.589326850	-9.090739633
	C102	1.378076249	-4.224067168	-8.480559570
	C103	0.179131626	-2.552444198	-7.086743290
	C104	-0.304374533	-1.202245170	-7.631620904
	C105	0.224368337	1.195393201	-7.768642153
	C106	1.432443586	2.068250852	-8.101394959
	C107	3.811261518	2.541888970	-7.885270578
	C108	4.256766019	2.622259291	-9.332366076
	C109	-4.048413964	-3.682117768	-8.502142538
	C110	-4.955294699	-2.510472445	-8.106295692
	C111	-3.466481985	-4.344157988	-7.232360705
	C112	-2.879822371	-5.732951085	-7.548066454
	C113	-2.085013059	-6.253251478	-6.372288191
	C114	-5.129271631	-0.141002052	-7.530037947
	C115	-4.661275035	1.085918082	-8.287112243
	C116	-5.280422789	0.059268748	-6.002052584
	C117	-3.955675928	-0.035838782	-5.294598940
	C118	-3.088547215	1.068354277	-5.235123695
	C119	-3.511597711	-1.272012249	-4.797005157
	C120	-1.791146392	0.919189079	-4.735176210
	C121	-2.214731913	-1.420922765	-4.296264517
	C122	-1.343638432	-0.327198944	-4.286526233
	C123	6.773729339	-6.957230955	-5.237841179
	C124	8.063181233	-7.094669756	-4.409722327
	C125	5.536254262	-7.145850159	-4.342421865
	C126	4.207117406	-6.867224439	-4.971986300
	C127	3.668908563	-7.484147660	-6.081987955
	C128	2.120106282	-6.217834431	-5.136561597
	C129	9.414738029	-8.367150387	-2.850155713
	C130	10.682117268	-8.214016612	-3.661162660
	C131	0.014130174	-6.962688854	0.214099436
	C132	1.527016735	-7.149758089	0.346876759
	C133	2.285400175	-7.299670759	-1.007780892
	C134	2.047723530	-8.742993113	-1.535250905
	C135	2.934157875	-9.194502133	-2.696500309
	C136	2.802147057	-10.697821217	-3.036594637
	C137	3.793922318	-7.103426614	-0.785992369
	C138	3.194780044	-1.066120690	-2.441391749
	Fe139	3.187798922	-2.376365673	-3.944872883
	Fe140	1.915343905	0.070769002	-1.638747479
	Fe141	4.552183693	0.162671909	-1.638720021
	Fe142	3.141017841	0.258170834	-3.904160348
	Fe143	3.429278191	2.384291627	-2.418700416
	Fe144	4.482453404	-2.437062606	-1.677651857
	Fe145	1.897419500	-2.334465252	-1.694339112
	H146	-6.894398837	-7.043271320	1.507516895
	H147	-7.216385070	-6.104215162	0.212725425
	H148	-9.822172616	-3.976080799	2.875078478
	H149	-9.366837166	-5.492736471	1.085801563
	H150	-9.074665168	-6.555451241	2.458553896
	H151	-7.769100002	-2.066201685	3.066597180
	H152	-8.466684136	-4.683578763	5.359717917
	H153	-9.260749683	-2.076726544	4.035216423
	H154	-7.384513913	-5.270851127	7.328925636
	H155	-5.887515677	-5.184946879	4.659771488
	H156	-7.037268837	-3.550426546	7.615882703
	H157	-2.422371349	-1.548496407	3.355681169
	H158	-4.800552890	-3.116888221	3.828086642
	H159	-3.719470033	-5.772857731	4.038596797
	H160	-3.467403187	0.741513200	7.351633235
	H161	-4.829179848	-1.465668828	6.063000872
	H162	-3.188663415	-5.710478585	5.737094951
	H163	-4.105942290	-0.974159549	3.369820635
	H164	-3.443439643	-2.820035698	8.067741950
	H165	-4.697422790	-0.326606078	8.076962952
	H166	-0.129049345	-3.063113494	3.171266650
	H167	-0.568711542	-1.996419597	1.833442429
	H168	1.114891672	-2.544284028	2.001740454
	H169	-0.399001081	-0.627343798	3.877735791
	H170	-2.328733992	-3.890059200	9.791169396
	H171	-0.422393309	2.360222670	4.763269547
	H172	0.113665085	0.266362040	8.723102383
	H173	0.548249912	3.596837015	8.837177749
	H174	2.032258294	-0.157106388	2.042474142

	H175	1.369459621	1.037162479	3.180018747
	H176	9.842246626	1.502960888	3.395975922
	H177	-1.061013816	3.865717050	5.460421623
	H178	-0.484032025	4.578164531	7.774228159
	H179	0.662953472	3.750009846	5.034084337
	H180	1.265026590	4.468814144	7.461206657
	H181	-0.753456399	2.153350336	7.252624886
	H182	2.188302044	2.215945418	6.383736614
	H183	0.035005824	0.258437143	5.972043685
	H184	0.396619850	0.482344514	1.812798375
	H185	1.371521283	3.962323468	-0.036462710
	H186	0.862936627	4.539046368	1.582830442
	H187	3.003049678	4.956224499	1.707247338
	H188	3.966566513	4.623500426	4.195058326
	H189	5.081599804	3.768896820	0.944519686
	H190	6.809201841	2.036763201	0.542757864
	H191	7.169432368	1.415524466	4.798168102
	H192	-0.125520319	-2.341216490	5.591883907
	H193	0.561789818	-5.180219168	6.398885503
	H194	1.498373775	-4.591249089	7.791203253
	H195	-1.243387284	-3.743245870	7.030872596
	H196	-1.630668247	-2.340020357	10.319735989
	H197	5.418830554	3.121736812	5.195303778
	H198	2.394538103	-1.820804712	4.148165147
	H199	1.184025121	1.229969653	11.062921794
	H200	0.736402433	-0.477464894	10.796026276
	H201	3.252309564	-0.659545750	9.772584428
	H202	11.046649850	-7.716373933	3.173782302
	H203	-2.693594864	-6.043793829	-3.498485635
	H204	0.545740215	-6.178714651	-1.483828899
	H205	-3.701984134	-6.436178459	-7.745567841
	H206	-2.242900233	-5.683684425	-8.443137623
	H207	-4.252061191	-4.455681595	-6.469358436
	H208	-4.237532730	0.870406422	-9.295844877
	H209	-4.743886387	-4.403857940	-8.956586645
	H210	-3.801630788	-2.064851047	-1.705209575
	H211	-4.480133382	-4.738437778	-0.372959166
	H212	-3.333121537	-1.216828447	-8.010718584
	H213	-6.145986769	-0.326526247	-7.933079822
	H214	-2.310787223	-2.748523839	-9.157202865
	H215	-2.062763703	-5.864588751	-1.864531768
	H216	-2.700532741	-3.681592581	-6.799982868
	H217	-2.685463576	-4.132020710	-9.969516600
	H218	-7.319042034	-3.085749542	-1.799097916
	H219	-3.720625005	-2.203057348	0.059875251
	H220	-0.451780392	-5.638752405	-7.281136494
	H221	-2.796087202	-4.244284158	-0.457210231
	H222	-3.285561643	3.048172872	3.413324749
	H223	-2.586518090	0.940669245	1.727945028
	H224	-7.546091241	1.523946337	4.732732265
	H225	-7.086823132	0.492301295	1.965206478
	H226	-6.374199607	4.666034952	2.990458228
	H227	-7.236663513	9.497268859	4.916354113
	H228	-6.022220451	5.951235154	5.881934050
	H229	-8.179667807	6.189369805	4.152198616
	H230	-5.497511299	7.381323414	3.203160788
	H231	-10.067242005	10.317299068	1.873274245
	H232	-2.899489847	4.644236207	1.515970781
	H233	-4.530881176	4.337342196	0.905544612
	H234	-8.741673063	11.257902169	3.844350627
	H235	-7.754949623	6.311698016	2.443786929
	H236	-5.850364582	2.535705234	2.031811028
	H237	-6.579152490	0.090583748	4.370038347
	H238	-9.706526008	2.602853991	0.885593224
	H239	-2.271935405	1.736960342	-2.431459066
	H240	-10.128656092	0.893369563	1.092416805
	H241	10.680352244	2.589832414	1.440224117
	H242	9.058840644	2.778271584	1.347613513
	H243	1.614462645	5.733674760	-1.804656675
	H244	-0.588621339	5.616293562	0.094404174
	H245	2.147738454	7.848010997	-0.297494496
	H246	-3.410200474	3.807730296	-1.319234953
	H247	-5.132842527	4.399036302	-2.343416233
	H248	-6.936308625	3.672880403	-3.464722370
	H249	-4.659356594	5.684937198	-1.532873301
	H250	-5.878838669	1.460860681	-3.392838943
	H251	-7.619361555	1.621259795	-2.993187736
	H252	0.413597537	11.506909116	-2.411401448
	H253	-0.650269648	12.901641680	-2.140309141
	H254	-0.694710985	10.014287317	-3.632079297

	H255	-2.387527476	9.595297696	-3.361973774
	H256	-3.713895444	9.160721440	-5.994964938
	H257	-3.048096519	4.721782691	-5.628572153
	H258	-6.792519829	9.150003975	-0.110175084
	H259	-2.311491564	8.854803733	2.030901083
	H260	-4.502262112	7.918945673	-2.778370794
	H261	0.894736501	5.074197318	-4.226924266
	H262	2.331464685	6.825431210	-3.731668169
	H263	-1.504835698	4.738093444	-3.430045589
	H264	-8.491526299	0.324127248	-0.434253454
	H265	3.677067510	9.479298163	-5.010780980
	H266	-6.494817185	-0.648237708	-2.215943309
	H267	0.290182478	6.035812552	-5.576484946
	H268	-2.937677627	7.241300503	0.280536311
	H269	3.602653060	9.916168137	-3.291601585
	H270	-3.159720818	7.650768803	-6.647194975
	H271	-4.978022488	9.064023803	-3.981780618
	H272	-5.844662517	-3.297033353	-2.557127029
	H273	-6.087607846	-2.956089082	0.128055355
	H274	-6.108239170	10.866707842	1.545307617
	H275	-5.306598976	1.679302100	-0.975981452
	H276	-0.465752532	7.819728053	-4.023807780
	H277	-2.071971437	6.122332898	-6.085617618
	H278	-1.006947720	3.977950141	-4.934209795
	H279	-3.625372311	5.928716431	-3.510819554
	H280	1.702528679	8.921266066	2.375375925
	H281	0.098360306	9.752527983	-0.316442907
	H282	0.743690760	12.587386138	-0.220191883
	H283	3.051233856	9.263789211	1.267207145
	H284	-1.562119574	10.876396308	1.514162156
	H285	3.280856692	-3.677470254	-9.343330722
	H286	3.287131577	-5.097226895	-8.282269662
	H287	6.788219366	-5.924786675	-5.616589117
	H288	3.431691448	-5.810482034	-10.558508658
	H289	1.914109477	-6.183132331	-10.068457930
	H290	5.557029447	4.343671314	-1.635992176
	H291	6.229830021	7.096958302	-0.499175765
	H292	4.810860043	7.538632267	-4.467378580
	H293	4.412446973	5.376017398	-0.718927509
	H294	7.146867767	7.542709276	-1.780334364
	H295	4.651261299	2.130097821	-7.303978379
	H296	5.950088412	6.141173502	-3.261808558
	H297	5.342927374	-7.061569105	4.672370445
	H298	6.763848193	-8.226572283	2.217965433
	H299	2.803966058	0.815490291	-7.143886835
	H300	5.311211799	-0.169475857	2.825473491
	H301	-0.397273337	1.134368263	-8.670322122
	H302	-1.879235962	-2.391576243	-3.924982316
	H303	-0.364816771	1.750337039	-7.017955358
	H304	4.488501246	-4.541447985	4.235565079
	H305	1.235164663	-0.336690996	-6.615618263
	H306	3.953251204	-5.079813944	2.646679542
	H307	4.633409840	-7.372459646	3.071653180
	H308	6.328775119	-5.716734628	2.081545699
	H309	6.817724108	-5.295786744	3.732464872
	H310	-5.971081539	-0.712224026	-5.633506086
	H311	5.815877664	-9.558213977	4.730771524
	H312	4.606402467	-12.002734179	3.350672228
	H313	-0.317246228	-0.432492401	-3.934066058
	H314	6.276985983	-11.860862210	5.740470890
	H315	4.674033106	-12.167408916	5.602968820
	H316	5.438274545	-3.409314305	1.623708638
	H317	-1.102835637	1.767031545	-4.703429647
	H318	-4.183916692	-2.133232154	-4.805569857
	H319	6.649108545	-10.553326372	-1.960876872
	H320	5.518756933	-8.190517197	-3.985856082
	H321	5.950546968	-8.665557886	-0.385435296
	H322	5.608353484	-11.110912671	-2.970951842
	H323	3.902550231	1.785620565	-9.980988430
	H324	5.916097095	-5.524668901	-0.697861030
	H325	1.732283531	-7.999414758	1.010108626
	H326	2.206711205	-2.523146277	-7.699046169
	H327	2.220821235	-9.414700945	-0.680194897
	H328	1.670511766	-7.376809006	-6.840010502
	H329	1.153544536	-5.764267193	-4.959253694
	H330	6.235802720	-12.661792843	3.535143837
	H331	5.409871674	-1.156507571	1.388846751
	H332	7.976456736	-6.396991755	0.139069759
	H333	6.932564103	-4.355642201	-0.815031448
	H334	8.775789663	-7.378520806	2.141253352

H335	4.431856836	-2.777636937	5.019778785
H336	2.672071110	-8.627562555	-3.601311235
H337	3.989575613	-8.987501435	-2.472998015
H338	0.986339737	-8.845066831	-1.809248093
H339	0.436498803	-2.467532901	-6.019511811
H340	-3.425316031	2.034424699	-5.618460933
H341	-5.750763852	1.043199953	-5.851560504
H342	1.879224385	-6.248030813	0.880697132
H343	7.979969457	-7.946981226	0.032038480
H344	6.739020404	-9.756430644	0.377427737
H345	4.425786062	-1.102148002	4.781616412
H346	4.102649645	-8.193520064	-6.775641924
H347	5.643749835	-6.521977422	-3.447514733
H348	-0.630091606	-3.276342477	-7.207420909
H349	3.615631238	3.557983926	-7.510741904
H350	8.126858564	-0.230967146	3.166554565
H351	7.995855998	0.060441570	1.416135154
H352	7.573376110	-7.657570783	-6.990834527
H353	6.847671630	-8.828867525	-6.109173131
H354	9.413941240	-9.385798551	-2.431091316
H355	7.505386360	-8.951391360	-3.598800011
H356	10.580027445	-8.477731953	-4.741322589
H357	9.427121324	-7.663698661	-2.002644783
H358	10.625783522	-0.369991567	1.064545779
H359	-1.706358897	13.592418908	3.355597811
H360	10.280762070	-7.380547627	4.748579919
H361	9.724656586	-4.965380759	3.537043034
H362	-3.738993520	12.686528162	1.331035385
H363	-3.003817247	10.720206511	3.585742643
H364	-4.575045248	11.515464044	3.385571643
H365	2.752999867	-4.751182598	0.490974757
H366	-6.197711451	7.196688479	-1.151543431
H367	0.077089373	-1.226644995	0.106725158
H368	2.209646485	-1.154844682	-0.531182070
H369	-1.780492498	0.091948413	-0.596177363
Mo370	3.101389053	-4.669559811	-2.503389137
N371	-7.658046271	-6.693228193	0.923108010
N372	-8.834890526	-3.889110776	3.103739613
N373	-7.758716610	-4.035552346	5.696598675
N374	-5.162871364	-5.075251449	5.370894593
N375	-3.849690066	-2.893483703	4.113325430
N376	-3.948260709	-0.982489214	6.251551022
N377	-2.694810290	-2.286572245	8.518520581
N378	-0.393295183	-3.754783135	7.598215477
N379	0.884816970	-2.367093736	5.453516976
N380	1.015612995	0.531170946	6.074421760
N381	1.038078355	0.572114996	9.036773116
N382	5.429139513	-11.582851204	5.241180509
N383	6.128881226	-9.447132421	3.760633884
N384	5.172343206	-3.385488640	2.609611036
N385	4.843892755	-2.003019142	4.492718997
N386	5.181729887	-1.081433440	2.394121384
N387	8.946228420	-7.444629478	3.168603764
N388	-6.413164955	-2.649268336	-1.981610504
N389	-2.760242497	-5.610653828	-2.571512418
N390	-6.333712027	-0.105185213	-1.350800842
N391	-8.362376632	1.211172952	0.047402329
N392	-7.548254213	0.996047111	2.730399498
N393	-5.249478828	2.597709972	2.859349722
N394	-3.022956367	2.970960672	-0.802588229
N395	-2.277155390	0.984969609	-0.411298539
N396	-5.686007328	5.289240524	3.405689398
N397	-9.058925047	8.466194232	2.324894204
N398	-8.682994765	10.295014822	3.523809209
N399	9.804973550	2.078870584	1.303324912
N400	6.626834935	6.756229759	-1.380843787
N401	4.301493976	7.980197306	-3.706545420
N402	1.579009010	7.516853761	-3.819493945
N403	-3.281131136	6.376215227	-4.378555094
N404	-3.547628807	8.167943971	-5.866856158
N405	-4.330465371	8.333504365	-3.697651456
N406	0.758282542	6.163962812	-1.402634320
N407	1.507472490	8.112367190	0.456758910
N408	0.340699326	10.613978544	0.172090415
N409	-1.616487327	10.250793164	-3.242588409
N410	-1.810369678	11.861179367	1.457238828
N411	2.532632497	-5.399432741	-10.297051882
N412	1.336600660	-3.045468045	-7.822542485
N413	0.487811085	-0.156859805	-7.309555144
N414	2.663765279	1.670943421	-7.691640538

	N415 -3.089321203 -3.295502808 -9.543850745<br>N416 -4.350493636 -1.301388356 -7.922140541<br>N417 6.754130475 -7.853083501 -6.408271355<br>N418 3.219036944 -6.060990398 -4.396148058<br>N419 2.362089640 -7.060791319 -6.165409576<br>N420 8.223174129 -8.225752168 -3.670179779<br>O421 -6.718883769 -4.613711570 2.679738939<br>O422 -6.688438362 -2.052760472 5.353419788<br>O423 -4.634352026 -4.317668634 7.458388937<br>O424 -1.939223515 -3.602277313 5.123188145<br>O425 -2.022196140 -0.124654073 5.388839788<br>O426 -1.814497152 -0.227116701 8.976568489<br>O427 0.529900639 -3.448720730 9.678994588<br>O428 2.812681539 -3.532619899 5.910261695<br>O429 3.173354698 0.174562369 5.417420779<br>O430 2.861663106 1.891331225 8.733140948<br>O431 3.418461271 0.069305070 11.672727597<br>O432 6.555696007 -10.721646121 1.878555621<br>O433 8.061880106 -7.933703907 5.233160588<br>O434 11.748681355 -5.224225138 3.539483387<br>O435 -5.668887010 -0.453042690 0.817734568<br>O436 -4.505526416 -4.323447635 -3.256727296<br>O437 -6.959960367 2.985936957 0.367457735<br>O438 -6.479708828 3.339730511 -2.671641813<br>O439 -9.318985018 2.306201240 3.379560231<br>O440 -4.933096099 2.065734546 5.056971499<br>O441 -3.518451973 5.520127262 4.086031630<br>O442 -5.549945477 7.932749124 5.755436926<br>O443 11.172858734 -0.659588324 3.009857673<br>O444 4.156636950 4.503779598 3.246758227<br>O445 3.936508886 8.051744559 -1.456777360<br>O446 1.123621232 9.749958696 -4.122657258<br>O447 -1.281004103 7.131893609 -1.768894890<br>O448 -0.098029157 7.331567322 1.898483922<br>O449 2.661274629 5.335896472 0.873092868<br>O450 1.854739539 11.560156105 1.609040450<br>O451 -1.568533438 13.747593993 0.202981618<br>O452 -2.746533802 11.176433606 -1.485158757<br>O453 -3.661198948 14.159282658 3.519097573<br>O454 -5.231030449 7.168560420 -1.014047470<br>O455 0.401903095 -5.007811528 -8.567071082<br>O456 -1.332991472 -1.101071957 -8.331680838<br>O457 1.250318074 3.140512195 -8.701564336<br>O458 4.980662978 3.505879839 -9.762194457<br>O459 -6.175595087 -2.668644621 -7.939013491<br>O460 -2.595640483 -6.772115837 -5.385617945<br>O461 -0.748832536 -6.073128402 -6.423198088<br>O462 -4.768679811 2.234423025 -7.880385717<br>O463 8.957037014 -6.227532665 -4.448108137<br>O464 11.762807177 -7.901272952 -3.181030009<br>O465 -0.377488512 -6.318701399 -0.886504263<br>O466 -0.774098384 -7.322153237 1.087956420<br>O467 3.883815624 -11.334030978 -3.291693911<br>O468 1.636948924 -11.185013577 -3.069636792<br>O469 4.359590644 -6.144101759 -1.471986495<br>O470 4.416470327 -7.842543379 -0.001050141<br>O471 1.810627666 -6.290085441 -1.906826455<br>O472 8.527500628 -7.186008277 0.368798164<br>O473 6.682456046 -5.098612106 -0.235030334<br>O474 -4.337477937 4.957702531 -2.123358872<br>O475 6.593351271 -11.079358823 -2.789494798<br>O476 6.788720755 -9.193758351 -0.437097291<br>S477 3.406117530 4.575686503 -2.758263192<br>S478 1.463935040 1.682096628 -3.193268376<br>S479 1.294904995 -3.518463610 -3.497431351<br>S480 3.202190353 1.436404922 -0.387209182<br>S481 -0.045366676 -1.159515635 -1.245733479<br>S482 3.207354832 -1.049484845 -5.720535302<br>S483 2.989380845 -3.613290907 -0.261606855<br>S484 5.098529591 1.353101871 -3.456691897<br>S485 5.018401997 -3.649254155 -3.453250377<br>S486 6.085783157 -1.214081319 -0.810574618<br>end

product

Fe(139) -2.346<br>Fe(140) 1.139<br>Fe(141) -2.487	bm522bh263b4c.car_3<br>C1 -8.084327217 -4.453265502 2.554445674<br>C2 -8.736197484 -5.591557999 1.760365162<br>C3 -8.476433000 -2.355374620 3.771555980<br>C4 -7.636104307 -2.603439918 5.021037777

Fe(142) 2.320<br>Fe(143) 3.112<br>Fe(144) 1.494<br>Fe(145) -0.413	

C5	-7.125394710	-4.074499048	6.902647905
C6	-5.645283097	-4.349525981	6.621998689
C7	-3.948986782	-5.036767356	5.007953036
C8	-3.233937562	-3.705136317	4.765974511
C9	-3.409677925	-1.413491813	3.943106264
C10	-3.078949121	-0.697340967	5.261489031
C11	-3.775738593	-0.135938503	7.516925382
C12	-2.703023979	-0.790721614	8.395729233
C13	-1.927257918	-2.852902399	9.466031747
C14	-0.572280929	-3.332707987	8.932704791
C15	0.646161985	-4.264482522	7.009880296
C16	1.469399048	-3.350459986	6.099050523
C17	1.437934624	-1.387692069	4.573108229
C18	1.956611421	-0.178761356	5.387546901
C19	0.503062714	-0.985723376	3.414534084
C20	1.141469265	0.114702293	2.561648486
C21	0.149240736	-2.220955949	2.581145581
C22	1.363232798	1.728599264	6.845855251
C23	1.831676416	1.389750393	8.277871813
C24	0.192425309	2.744583555	6.836983655
C25	0.489697884	3.940836157	7.748741352
C26	-0.102078294	3.223123451	5.409302977
C27	1.356827196	0.358679558	10.428802205
C28	2.775201730	-0.135962028	10.617810023
C29	5.187681885	-11.955352180	3.889543603
C30	5.801602455	-10.771879097	3.142620302
C31	6.334871763	-8.346397315	3.377837400
C32	7.684701775	-8.073360494	4.068995894
C33	5.319507395	-7.226444439	3.665683178
C34	5.786651225	-5.852767751	3.181263600
C35	4.661840350	-4.819881960	3.252062839
C36	4.990652533	-2.322442674	3.186828432
C37	10.025556632	-7.440511118	3.734515030
C38	10.401707185	-5.978551480	3.629142115
C39	-5.771736996	-2.168502528	-0.727863667
C40	-5.909755014	-0.683521795	-0.343636007
C41	-4.263971613	-2.493095764	-0.827036181
C42	-3.901915671	-3.989879064	-0.905418404
C43	-3.841042218	-4.579321930	-2.314156056
C44	-6.231776251	1.557682483	-1.320016251
C45	-7.148505892	2.177862800	-0.256506676
C46	-6.509099424	2.114637751	-2.723360608
C47	-9.210481035	1.944439524	1.049991617
C48	-8.636929253	1.986918395	2.471040025
C49	-6.800025399	1.347678947	3.968764947
C50	-5.515864299	2.176010863	3.988549382
C51	-3.978830433	3.667765905	2.816950633
C52	-4.232121603	5.032028267	3.500144716
C53	-3.526717906	4.010697732	1.389889497
C54	-3.010023992	2.896055762	0.543938188
C55	-2.536913569	1.632476402	0.798750015
C56	-2.359415330	1.986641571	-1.389627020
C57	-5.854321052	6.874041798	3.706680001
C58	-5.598168819	7.098846007	5.195605478
C59	-7.351733424	7.064457668	3.369170902
C60	-7.841636561	8.477834548	3.251826229
C61	-7.546882839	9.603559448	3.996209158
C62	-9.021395231	10.087188710	2.414138757
C63	9.669620903	0.811117550	2.386472631
C64	10.557525739	-0.393819938	2.135147597
C65	8.206918465	0.298539658	2.378322690
C66	7.189911543	1.388581368	2.623746830
C67	6.795561740	1.740041063	3.928058287
C68	6.593434863	2.072455655	1.550160528
C69	5.830138226	2.726174462	4.153508534
C70	5.636542245	3.065037636	1.759329275
C71	5.247842965	3.384759229	3.063703118
C72	5.719035448	6.198287071	-2.319927264
C73	4.767737360	7.397875314	-2.470812580
C74	4.952359994	4.998734174	-1.769423298
C75	3.715054418	8.974004886	-4.059959832
C76	2.205832905	8.716908495	-4.044069334
C77	0.434617494	6.992137333	-3.724826705
C78	0.027900770	6.750458897	-2.248466437
C79	0.278255731	5.717309551	-4.588645823
C80	-1.049065693	4.951953848	-4.539545892
C81	-2.234420036	5.574108292	-5.295100454
C82	-3.458076129	7.679648725	-4.744946517
C83	0.619206859	5.907041429	-0.033008671
C84	0.790005344	7.168865128	0.832830253

	C85	1.448510178	4.774292742	0.598845051
	C86	2.239383456	9.093605251	1.294240101
	C87	1.735476661	10.499369182	0.993511115
	C88	0.308288069	11.894237501	-0.417458276
	C89	-0.800985227	12.623799738	0.379930742
	C90	-0.041329379	11.866033731	-1.909466149
	C91	-1.325203663	11.108747433	-2.224973900
	C92	-2.624707073	12.418984622	2.006866872
	C93	-2.320980413	13.557753884	2.970073649
	C94	-3.330986245	11.253913851	2.753706217
	C95	-3.799265313	10.168053503	1.810534015
	C96	-5.058338377	10.258035875	1.196929220
	C97	-2.979629365	9.077422516	1.476619671
	C98	-5.480123255	9.310712815	0.260633372
	C99	-3.371782094	8.134496906	0.520351817
	C100	-4.618650558	8.267190698	-0.082416762
	C101	2.573366144	-4.746686651	-9.050371624
	C102	1.246408260	-4.324029411	-8.443453362
	C103	0.116396250	-2.599451851	-7.056795501
	C104	-0.324820851	-1.242883806	-7.621209449
	C105	0.272381112	1.138321578	-7.781609910
	C106	1.510666471	1.962176638	-8.128305416
	C107	3.906254052	2.349025265	-7.910235338
	C108	4.325248505	2.431352083	-9.365915856
	C109	-4.137580623	-3.610260902	-8.489364409
	C110	-4.999576060	-2.401531952	-8.110948560
	C111	-3.590505222	-4.280652817	-7.208307561
	C112	-3.047193107	-5.689839940	-7.506909870
	C113	-2.277022832	-6.224879415	-6.320606953
	C114	-5.097575856	-0.027672070	-7.557987339
	C115	-4.590211017	1.186668716	-8.307407699
	C116	-5.264828548	0.167595648	-6.032509151
	C117	-3.952607824	0.038748887	-5.309530528
	C118	-3.053606967	1.117082229	-5.252370347
	C119	-3.552641871	-1.205027393	-4.791613773
	C120	-1.768591582	0.936592297	-4.733966898
	C121	-2.267622995	-1.384452002	-4.272402718
	C122	-1.364633078	-0.317624134	-4.265397449
	C123	6.541391313	-7.223768919	-5.162782758
	C124	7.825128984	-7.400123933	-4.335465166
	C125	5.307650495	-7.320551956	-4.250611645
	C126	3.987638027	-7.004752165	-4.878373062
	C127	3.418456186	-7.612947285	-5.977946769
	C128	1.921923864	-6.291611603	-5.025886724
	C129	9.129651218	-8.706766661	-2.768999565
	C130	10.405311812	-8.605342397	-3.575460671
	C131	-0.227699192	-6.945483690	0.291128717
	C132	1.269504995	-7.245326536	0.428923645
	C133	2.031587617	-7.408781094	-0.924331259
	C134	1.767344125	-8.839612222	-1.465365131
	C135	2.650392624	-9.305699782	-2.623945535
	C136	2.471471523	-10.800926915	-2.975424661
	C137	3.541387515	-7.240927571	-0.694366265
	C138	3.174411290	-1.184694927	-2.432021393
	Fe139	3.109940768	-2.527339476	-3.904946300
	Fe140	1.924437042	0.018497997	-1.610313867
	Fe141	4.543120644	0.042847953	-1.643955202
	Fe142	3.178768106	0.076088409	-3.914202530
	Fe143	3.390151817	2.294894037	-2.392827880
	Fe144	4.408906060	-2.494870908	-1.723035361
	Fe145	1.861360017	-2.387466968	-1.640358208
	H146	-7.090012010	-6.782619714	1.573107889
	H147	-7.382262948	-5.865336718	0.255050182
	H148	-9.941347364	-3.619554821	2.870330349
	H149	-9.507154421	-5.161232433	1.100974795
	H150	-9.274891642	-6.221216716	2.489474327
	H151	-7.855240735	-1.756624804	3.089612438
	H152	-8.631221467	-4.354998073	5.391286142
	H153	-9.347137805	-1.749844705	4.058287169
	H154	-7.571483558	-4.966098796	7.362815295
	H155	-6.065066731	-4.955832301	4.694440738
	H156	-7.157194176	-3.257888310	7.636228424
	H157	-2.481383851	-1.457282700	3.361425329
	H158	-4.908313296	-2.945321032	3.841133885
	H159	-3.916681826	-5.637478503	4.089149400
	H160	-3.463508310	0.900935152	7.338797325
	H161	-4.893216761	-1.265898472	6.062917108
	H162	-3.389443954	-5.566055718	5.788421000
	H163	-4.144621570	-0.826349072	3.374481964
	H164	-3.550290689	-2.651663206	8.086434668

H165	-4.725977516	-0.125142349	8.068278110
H166	-0.200223541	-3.054728671	3.206103424
H167	-0.660517326	-1.996615533	1.869314905
H168	1.020523615	-2.565275705	2.002958692
H169	-0.419094770	-0.584295051	3.867249294
H170	-2.472768178	-3.739521640	9.820508176
H171	-0.348148926	2.392053132	4.733096718
H172	0.095654567	0.316507807	8.716804164
H173	0.637230322	3.643976276	8.797666870
H174	2.071569452	-0.229516563	2.084968735
H175	1.379055342	1.011681863	3.152089100
H176	9.894596308	1.194241837	3.399708357
H177	-0.954296706	3.918461790	5.411689467
H178	-0.348207571	4.652105169	7.715039095
H179	0.768805884	3.757353452	4.996198104
H180	1.398523669	4.467684862	7.417275982
H181	-0.704382294	2.228372082	7.224686807
H182	2.243101232	2.188365273	6.373142140
H183	0.034346195	0.289519586	5.964923726
H184	0.460986831	0.431908393	1.757605187
H185	1.427097492	3.894639614	-0.069435898
H186	0.959417833	4.505722934	1.548087729
H187	3.114770832	4.828780210	1.664505576
H188	4.095024957	4.495073400	4.161496932
H189	5.164697314	3.569555450	0.916776137
H190	6.859480272	1.801443999	0.525724290
H191	7.232117292	1.222483951	4.785552079
H192	-0.214422375	-2.298842778	5.593147298
H193	0.355617618	-5.143389504	6.413580535
H194	1.316461604	-4.599481749	7.810134288
H195	-1.385448679	-3.637974427	7.049478226
H196	-1.717866607	-2.211296769	10.330793844
H197	5.516951486	2.965077150	5.173132960
H198	2.329354510	-1.872160107	4.160822292
H199	1.197712889	1.251614262	11.054941211
H200	0.689795381	-0.438847064	10.792031796
H201	3.192507949	-0.715847660	9.762623418
H202	10.786451217	-8.054795363	3.229941790
H203	-2.854080446	-5.982148589	-3.430757333
H204	0.419315814	-6.185156673	-1.411304511
H205	-3.888440387	-6.369794850	-7.704423041
H206	-2.402423148	-5.667534968	-8.397615880
H207	-4.386856802	-4.357185714	-6.452324589
H208	-4.160247031	0.962156292	-9.311827087
H209	-4.860162490	-4.307378366	-8.939524542
H210	-3.848371787	-1.960659092	-1.697676113
H211	-4.635052831	-4.590621493	-0.339544227
H212	-3.333727106	-1.160949230	-8.034260458
H213	-6.115079082	-0.181878507	-7.973121042
H214	-2.363165172	-2.751481117	-9.147841786
H215	-2.221049210	-5.790906146	-1.796145468
H216	-2.806779916	-3.639758898	-6.774898359
H217	-2.787199854	-4.129615555	-9.945604105
H218	-7.401581375	-2.834984932	-1.807068641
H219	-3.784691793	-2.073702183	0.069491587
H220	-0.622590212	-5.666977611	-7.223642991
H221	-2.935102419	-4.154576877	-0.408157556
H222	-3.170741100	3.188638349	3.386600303
H223	-2.454359144	1.078027591	1.724978560
H224	-7.481911657	1.803485528	4.700457255
H225	-7.051849991	0.753550686	1.935222045
H226	-6.204970968	4.903510188	2.946622613
H227	-6.875979309	9.775279115	4.830548469
H228	-5.810152454	6.204135306	5.830082831
H229	-7.959351669	6.499295501	4.098799131
H230	-5.239558204	7.587910383	3.135588087
H231	-9.704979010	10.677855446	1.810134288
H232	-2.732969843	4.769693231	1.486514857
H233	-4.364239618	4.491790430	0.859408861
H234	-8.322640662	11.582276225	3.758665508
H235	-7.528193834	6.592369411	2.389664520
H236	-5.743180083	2.745811181	1.996854917
H237	-6.563417493	0.339278014	4.339081958
H238	-9.574193466	2.959128899	0.837644585
H239	-2.145624311	1.824210242	-2.442311788
H240	-10.078596811	1.272217871	1.046784591
H241	10.773602198	2.265678773	1.452598398
H242	9.158510823	2.500014377	1.347014527
H243	1.792510695	5.692957873	-1.835912490
H244	-0.443793585	5.634528730	0.043644185

H245	2.378847977	7.754904297	-0.335892420
H246	-3.256292728	3.922444121	-1.351087977
H247	-4.966076557	4.559966708	-2.381726942
H248	-6.753579173	3.883183551	-3.534255104
H249	-4.444042921	5.838910356	-1.590235239
H250	-5.801815119	1.645609874	-3.426819059
H251	-7.536407763	1.859630987	-3.027293928
H252	0.812636934	11.480967111	-2.484421476
H253	-0.203067871	12.910818223	-2.217458449
H254	-0.355972605	10.026309083	-3.711436734
H255	-2.068768322	9.685135091	-3.448249628
H256	-3.436622751	9.214934967	-6.109068130
H257	-2.881255192	4.777289641	-5.693460758
H258	-6.469660775	9.388308157	-0.197480213
H259	-2.011917634	8.935160014	1.964950761
H260	-4.194140107	8.052512213	-2.855718738
H261	1.070176548	5.015968392	-4.281567137
H262	2.550978080	6.724535933	-3.742690262
H263	-1.340156093	4.740169351	-3.498154408
H264	-8.452633209	0.619511309	-0.460189757
H265	3.976642111	9.344495990	-5.059549731
H266	-6.486159941	-0.435095071	-2.236660862
H267	0.499631714	5.997213428	-5.631928422
H268	-2.692467243	7.335208868	0.222557956
H269	3.899259837	9.787126699	-3.342856036
H270	-2.964932400	7.672374394	-6.751273371
H271	-4.649288915	9.197334241	-4.067585541
H272	-5.931405208	-3.119065051	-2.547426992
H273	-6.179658687	-2.737289981	0.128358775
H274	-5.730021700	11.080774472	1.453683158
H275	-5.217675152	1.857692891	-1.015079453
H276	-0.213497824	7.797280442	-4.085363272
H277	-1.859452650	6.143544410	-6.155028252
H278	-0.858821084	3.974795271	-5.006295137
H279	-3.409394618	6.020480935	-3.574182462
H280	1.957516853	8.860320112	2.331832194
H281	0.432956441	9.743968379	-0.389938746
H282	1.183870331	12.551216545	-0.298222720
H283	3.335918749	9.129694402	1.240455352
H284	-1.211641270	10.927297682	1.414049152
H285	3.162381271	-3.858465538	-9.322744272
H286	3.125278620	-5.255845659	-8.232584940
H287	6.604845908	-6.204721254	-5.571884035
H288	3.236634762	-6.021657336	-10.494642979
H289	1.708739155	-6.330305057	-9.992031529
H290	5.642172074	4.146106855	-1.714854214
H291	6.469125952	6.881975735	-0.544888894
H292	5.029054243	7.355625034	-4.509550484
H293	4.561407912	5.224503082	-0.768664274
H294	7.421684147	7.240198669	-1.829501493
H295	4.735929721	1.884564895	-7.354285522
H296	6.138177423	5.934830773	-3.303581359
H297	5.114930677	-7.203824816	4.749321959
H298	6.481975140	-8.427631254	2.293910343
H299	2.832222244	0.672372421	-7.153093599
H300	5.316677324	-0.335099649	2.804598407
H301	-0.351829837	1.081718241	-8.682180532
H302	-1.961880248	-2.358326903	-3.884444250
H303	-0.292985371	1.731835709	-7.042529065
H304	4.351791099	-4.653350129	4.290111175
H305	1.214349839	-0.401167405	-6.587182393
H306	3.776109899	-5.190335765	2.710586519
H307	4.383212766	-7.506036925	3.156406254
H308	6.131274173	-5.921930195	2.138990369
H309	6.637824194	-5.495414444	3.783042266
H310	-5.975304629	-0.592055120	-5.677752691
H311	5.475301171	-9.711628450	4.807119725
H312	4.199151070	-12.120755441	3.433937822
H313	-0.346699624	-0.444323834	-3.897053420
H314	5.856453066	-12.011586540	5.835925411
H315	4.245643334	-12.272072178	5.691842794
H316	5.323319892	-3.596189850	1.657573558
H317	-1.054242918	1.762403044	-4.707643735
H318	-4.249184038	-2.046212012	-4.799502361
H319	6.289247856	-10.784724252	-1.878971926
H320	5.243234487	-8.345844861	-3.847376619
H321	5.646121930	-8.864799371	-0.292787502
H322	5.246019828	-11.308579834	-2.905730784
H323	3.948048408	1.601863243	-10.010923810
H324	5.709816418	-5.709582068	-0.604140532

H325	1.402677204	-8.137192688	1.052856766
H326	2.139944428	-2.647481951	-7.681446658
H327	1.917292252	-9.525137592	-0.616695847
H328	1.412932522	-7.446181436	-6.711498613
H329	0.971968848	-5.809493474	-4.837675269
H330	5.802230097	-12.837119659	3.639611870
H331	5.354575843	-1.353346421	1.387130686
H332	7.758365330	-6.663879565	0.196414944
H333	6.779593190	-4.601733274	-0.790307422
H334	8.523057495	-7.657514698	2.205784699
H335	4.344673890	-2.878672259	5.044741149
H336	2.415350749	-8.724239157	-3.527829321
H337	3.710151750	-9.133599247	-2.389606485
H338	0.705707661	-8.911242764	-1.748668363
H339	0.384786988	-2.509261590	-5.992674301
H340	-3.358745609	2.087443261	-5.651119973
H341	-5.716871741	1.160060624	-5.881101391
H342	1.694255964	-6.404599198	1.001459789
H343	7.703362323	-8.215269122	0.101054052
H344	6.409626347	-9.976421875	0.466228375
H345	4.384645949	-1.208202878	4.777395000
H346	3.819310176	-8.344869986	-6.667152689
H347	5.454466382	-6.667006046	-3.382731152
H348	-0.715922478	-3.299302724	-7.162728169
H349	3.764061325	3.366788472	-7.516848724
H350	8.120341812	-0.477940321	3.152831095
H351	8.012165743	-0.177241556	1.403602228
H352	7.295593001	-8.033523543	-6.888588470
H353	6.488086216	-9.122582552	-5.974760486
H354	9.095730710	-9.721032757	-2.340858689
H355	7.209580781	-9.240858556	-3.527343631
H356	10.297628877	-8.865147385	-4.655967068
H357	9.160731871	-7.995179251	-1.928779118
H358	10.611134030	-0.699088872	1.061623603
H359	-1.252035520	13.656021473	3.272495695
H360	10.035267637	-7.698167862	4.808717467
H361	9.537999449	-5.270009164	3.621503805
H362	-3.308601989	12.816922538	1.238319730
H363	-2.640631324	10.838554100	3.504665891
H364	-4.185303319	11.682983793	3.295961461
H365	1.477743133	-4.527753781	0.284473807
H366	-5.937607687	7.411469417	-1.237411421
H367	0.029804069	-1.219498201	0.122361741
H368	2.162430755	-1.259265895	-0.483051236
H369	-1.666059059	0.194160055	-0.594088888
Mo370	2.935295267	-4.788701003	-2.394275667
N371	-7.841322942	-6.424647425	0.977844661
N372	-8.955563005	-3.563006143	3.115687332
N373	-7.898178113	-3.731766549	5.720862803
N374	-5.338869226	-4.870219850	5.407522651
N375	-3.951842866	-2.749373360	4.128953134
N376	-3.993878797	-0.816851659	6.250710486
N377	-2.783051279	-2.139118061	8.530353184
N378	-0.535559871	-3.678910587	7.615618851
N379	0.795255828	-2.358665540	5.463094515
N380	1.021909017	0.537910828	6.063025035
N381	1.029492101	0.593327074	9.029783219
N382	5.020143774	-11.714644327	5.328218132
N383	5.808797984	-9.620452900	3.841880476
N384	5.061192869	-3.547142960	2.643420400
N385	4.776972427	-2.125135040	4.504366426
N386	5.145928759	-1.249017764	2.392272119
N387	8.694270119	-7.723131024	3.233223129
N388	-6.476738454	-2.438617306	-1.986958986
N389	-2.912596831	-5.534313328	-2.510253569
N390	-6.309062799	0.107650474	-1.374419632
N391	-8.290832293	1.505053551	0.014030495
N392	-7.495959833	1.277127951	2.697336277
N393	-5.144737478	2.795470287	2.826734678
N394	-2.883583126	3.083993205	-0.826882127
N395	-2.149595875	1.097754350	-0.414883002
N396	-5.496658211	5.505293372	3.360023847
N397	-8.763244261	8.792643595	2.266572016
N398	-8.304683586	10.615381024	3.445531159
N399	9.885098923	1.779860767	1.308636740
N400	6.843751378	6.500437392	-1.419805908
N401	4.561858764	7.838049915	-3.746589535
N402	1.819493095	7.437524975	-3.857661164
N403	-3.057884028	6.444479567	-4.450553506
N404	-3.256536320	8.228272098	-5.955448484

	N405	-4.022754045	8.448720569	-3.782930300
	N406	0.933185638	6.129003988	-1.443218239
	N407	1.750018013	8.043817335	0.418257855
	N408	0.701254730	10.595037721	0.102748261
	N409	-1.265407107	10.297919208	-3.315089527
	N410	-1.408171215	11.924435269	1.372058197
	N411	2.353685627	-5.573913765	-10.238631650
	N412	1.250664581	-3.138796981	-7.796253400
	N413	0.488656154	-0.213379229	-7.297117622
	N414	2.725374020	1.525782963	-7.709816598
	N415	-3.160199133	-3.273340401	-9.530915140
	N416	-4.353345688	-1.212025872	-7.944825555
	N417	6.462591107	-8.152830223	-6.304966483
	N418	3.033103615	-6.163095380	-4.298915549
	N419	2.125194967	-7.147291330	-6.051087712
	N420	7.947960526	-8.535505198	-3.595977063
	O421	-6.856723674	-4.357708732	2.726995011
	O422	-6.760908498	-1.788369718	5.364669925
	O423	-4.788393694	-4.111643529	7.489363984
	O424	-2.062862004	-3.514574474	5.139000220
	O425	-2.028456564	-0.046149647	5.394244064
	O426	-1.846389989	-0.101815521	8.977305062
	O427	0.392336807	-3.437487371	9.702249745
	O428	2.683476213	-3.576045051	5.949117401
	O429	3.169016372	0.108850575	5.409871959
	O430	2.894351582	1.852084609	8.725524791
	O431	3.396319536	0.010226385	11.660887682
	O432	6.216979089	-10.921263609	1.974453886
	O433	7.801205818	-8.171205376	5.302767162
	O434	11.553809359	-5.578555132	3.572957769
	O435	-5.657695361	-0.246470591	0.796834999
	O436	-4.627833320	-4.217569106	-3.219912921
	O437	-6.827951463	3.230519522	0.323937979
	O438	-6.345843796	3.546452134	-2.716341180
	O439	-9.227346106	2.642766384	3.339561975
	O440	-4.857550734	2.271319520	5.030519341
	O441	-3.318576559	5.671210057	4.027943554
	O442	-5.233476469	8.155862489	5.679915433
	O443	11.155271799	-1.010204333	3.004887261
	O444	4.265808520	4.346889187	3.213520696
	O445	4.226517357	7.941969742	-1.492197976
	O446	1.426132461	9.676463616	-4.210107416
	O447	-1.093457619	7.112743992	-1.852448847
	O448	0.110902935	7.325770740	1.856037047
	O449	2.791873728	5.204788527	0.821289932
	O450	2.239755043	11.480279966	1.552978124
	O451	-1.066004890	13.809348578	0.137429008
	O452	-2.358691928	11.266376629	-1.557581500
	O453	-3.184235735	14.302699575	3.406624044
	O454	-4.975585792	7.340370217	-1.084049592
	O455	0.240808588	-5.070075079	-8.520952570
	O456	-1.337056986	-1.123184063	-8.340753954
	O457	1.365583168	3.032202753	-8.742373653
	O458	5.052051848	3.306740969	-9.806708083
	O459	-6.224894195	-2.511373271	-7.939830877
	O460	-2.809967936	-6.719658868	-5.333412225
	O461	-0.934923061	-6.085215011	-6.363490122
	O462	-4.677529177	2.337658208	-7.902873502
	O463	8.744313120	-6.558988297	-4.366413478
	O464	11.494990071	-8.337081468	-3.089200578
	O465	-0.562139405	-6.238038266	-0.781681504
	O466	-1.040458654	-7.290330469	1.153047582
	O467	3.530237256	-11.464989657	-3.256304637
	O468	1.293171108	-11.255552656	-2.991605726
	O469	4.137095511	-6.301939809	-1.384149128
	O470	4.141616517	-7.987641984	0.100469389
	O471	1.581985337	-6.393097999	-1.832821688
	O472	8.278405886	-7.472098720	0.432571578
	O473	6.515323453	-5.315769441	-0.181370420
	O474	-4.150263413	5.092557382	-2.171744603
	O475	6.229041557	-11.310394636	-2.707411378
	O476	6.465174459	-9.421327463	-0.353353033
	S477	3.509205046	4.546731866	-2.837293401
	S478	1.480322681	1.445613217	-3.325250054
	S479	1.176539340	-3.588675259	-3.421197697
	S480	3.258506159	1.325159895	-0.337422589
	S481	-0.076566536	-1.185106835	-1.229400431
	S482	3.219472940	-1.237596508	-5.703669368
	S483	2.601402889	-3.850544485	-0.145037211
	S484	5.082923419	1.222457593	-3.448595486

	S485 4.889003129 -3.821128802 -3.344413525<br>S486 6.017828024 -1.395090101 -0.806229800<br>end

S2BH-26H-3b3 to S2BH-6Hn-6Hn

35, S=1/2

reactant

Fe(139) -2.305<br>Fe(140) -1.962<br>Fe(141) -2.232<br>Fe(142) 2.878<br>Fe(143) 2.905<br>Fe(144) 1.337<br>Fe(145) 0.171	bm522bh263b321tc.car_4
	C1 -8.326072821 -4.702337374 0.591819905<br>C2 -8.709441979 -5.893816948 -0.291581308<br>C3 -9.090126419 -2.598390658 1.600433793<br>C4 -8.585365078 -2.783417333 3.028676353<br>C5 -8.516433822 -4.199743862 5.018731180<br>C6 -7.002906550 -4.412883131 5.129565619<br>C7 -4.933373387 -5.073169653 4.017632205<br>C8 -4.216808623 -3.724442302 3.916062993<br>C9 -4.248131340 -1.460992442 2.998444609<br>C10 -4.289696377 -0.700766511 4.333947664<br>C11 -5.557687996 -0.114541627 6.317737279<br>C12 -4.727858942 -0.703229808 7.465686057<br>C13 -4.169471891 -2.701643484 8.765097480<br>C14 -2.703317973 -3.123053443 8.597577380<br>C15 -1.020831390 -4.076893765 7.078427916<br>C16 -0.026101695 -3.138575326 6.389913079<br>C17 0.270498073 -1.225452160 4.832284312<br>C18 0.527490925 0.030241589 5.697441150<br>C19 -0.349677829 -0.900424550 3.458503662<br>C20 0.445860512 0.203496493 2.755116116<br>C21 -0.427584897 -2.177189915 2.616699773<br>C22 -0.481941302 1.938348423 6.904051237<br>C23 -0.377249988 1.650319317 8.416571098<br>C24 -1.642833370 2.905854002 6.563214905<br>C25 -1.622432637 4.137687475 7.476911552<br>C26 -1.580522515 3.331412491 5.090013250<br>C27 -1.350033011 0.665624258 10.414551580<br>C28 -0.012004091 0.200644075 10.952823642<br>C29 4.406974162 -11.642590923 5.478475504<br>C30 5.151469644 -10.457221509 4.864804489<br>C31 5.543627587 -8.007503299 5.147648198<br>C32 6.664651953 -7.663521856 6.147620880<br>C33 4.454281309 -6.919419569 5.132382647<br>C34 4.989421564 -5.540221168 4.746767246<br>C35 3.856206087 -4.543012126 4.505236801<br>C36 4.090109110 -2.034781545 4.436710016<br>C37 8.993833573 -6.946950479 6.384136782<br>C38 9.341111939 -5.475228093 6.325800877<br>C39 -5.332797997 -2.407616565 -2.086270186<br>C40 -5.600273220 -0.917689658 -1.803439777<br>C41 -3.845587411 -2.689201218 -1.774135294<br>C42 -3.440637027 -4.176969247 -1.717733235<br>C43 -2.998830130 -4.788809264 -3.047086767<br>C44 -5.718680558 1.278426975 -2.913986020<br>C45 -6.888011408 1.902886081 -2.137646464<br>C46 -5.644743199 1.781327670 -4.362278917<br>C47 -9.208496860 1.637110422 -1.387701333<br>C48 -9.020548425 1.719544010 0.132116157<br>C49 -7.605044871 1.180612839 2.062739299<br>C50 -6.393680105 2.054867963 2.384269327<br>C51 -4.665129631 3.594797736 1.603877660<br>C52 -5.134371965 4.952906945 2.172944026<br>C53 -3.881847181 3.939765860 0.329798206<br>C54 -3.121671710 2.833354081 -0.324026485<br>C55 -2.666615157 1.609393083 0.100394161<br>C56 -1.962876597 1.902941620 -1.987721207<br>C57 -6.805727967 6.741518545 1.891329108<br>C58 -6.932313521 7.018715424 3.387884365<br>C59 -8.182759327 6.861118046 1.198924844<br>C60 -8.668900513 8.250724398 0.905977991<br>C61 -8.630959906 9.402130992 1.668184429<br>C62 -9.637486524 9.789779427 -0.266633702<br>C63 8.728848018 1.261229068 4.702824828<br>C64 9.685022709 0.084140818 4.750328505<br>C65 7.331855431 0.680216216 4.363455696<br>C66 6.247207144 1.730985957 4.301720817<br>C67 5.506456590 2.087238133 5.442940463

C68	5.937719008	2.374228407	3.088882744
C69	4.480078411	3.037131866	5.375921585
C70	4.927005810	3.332443808	3.010248955
C71	4.190469754	3.654850783	4.153261559
C72	5.889536685	6.363978133	-1.006045156
C73	4.967792511	7.516008442	-1.433663844
C74	5.053122328	5.148900428	-0.605443916
C75	4.302836648	9.003434911	-3.296074858
C76	2.853225625	8.684485649	-3.663209293
C77	1.114209891	6.899428965	-3.730691996
C78	0.353132689	6.671074143	-2.400059767
C79	1.234278270	5.606299111	-4.573113101
C80	-0.024551919	4.777627255	-4.855248612
C81	-1.003803953	5.337266831	-5.896632205
C82	-2.372946965	7.420262664	-5.736657312
C83	0.381541198	5.907348730	-0.080264786
C84	0.293595300	7.196792991	0.754889604
C85	1.053695159	4.825407598	0.782741461
C86	1.523952098	9.179405661	1.513420711
C87	1.080450287	10.560930166	1.052447019
C88	0.020965262	11.879897252	-0.712703150
C89	-1.275002826	12.586958274	-0.247828827
C90	0.074770279	11.800698853	-2.241720509
C91	-1.070050538	11.003503633	-2.858052010
C92	-3.445363033	12.348435526	0.866342000
C93	-3.431628422	13.528264125	1.829185052
C94	-4.281666180	11.175928996	1.452734512
C95	-4.457910710	10.050317082	0.458014665
C96	-5.523419523	10.074253649	-0.455701057
C97	-3.538983423	8.991623248	0.375920026
C98	-5.655958581	9.093622894	-1.441216064
C99	-3.639490143	8.018040636	-0.621331529
C100	-4.693332990	8.086843022	-1.528001250
C101	4.929470774	-4.862053695	-7.944012628
C102	3.479915928	-4.477517854	-7.697224077
C103	1.993004297	-2.763881738	-6.683465959
C104	1.671610865	-1.444243970	-7.393544034
C105	2.208410658	0.950986803	-7.491671095
C106	3.463445092	1.818261813	-7.527354473
C107	5.718871078	2.288732813	-6.745383417
C108	6.449539051	2.400868537	-8.069383892
C109	-1.721867153	-3.982886453	-9.143157264
C110	-2.691816695	-2.800611419	-9.041990881
C111	-1.503669721	-4.598933500	-7.742615052
C112	-0.869040861	-5.998225464	-7.840831048
C113	-0.406958415	-6.462193847	-6.478457401
C114	-2.997024689	-0.415305036	-8.613269552
C115	-2.334767886	0.793622202	-9.243204837
C116	-3.566211054	-0.177661066	-7.193500573
C117	-2.487777987	-0.253452305	-6.146511032
C118	-1.658224628	0.850165167	-5.884828706
C119	-2.213085818	-1.474963086	-5.508547896
C120	-0.545731018	0.716695753	-5.048108306
C121	-1.101751924	-1.608366981	-4.670607279
C122	-0.253074300	-0.518083470	-4.457970574
C123	7.854268529	-7.106285151	-3.103385085
C124	8.891745199	-7.198555881	-1.972121456
C125	6.434802000	-7.190485900	-2.519653002
C126	5.308927811	-6.952876643	-3.476321710
C127	5.066368051	-7.616033700	-4.661622820
C128	3.326252934	-6.337526138	-4.176524524
C129	9.802815627	-8.407095172	-0.078476592
C130	11.236211813	-8.275592634	-0.543016175
C131	-0.058964960	-6.897007181	0.464927812
C132	1.364984208	-7.107234243	0.981379970
C133	2.437368726	-7.315288029	-0.129951033
C134	2.353297702	-8.773157324	-0.655059775
C135	3.517921377	-9.239804249	-1.530828141
C136	3.488921369	-10.746759178	-1.877736238
C137	3.837997737	-7.092518750	0.463805254
C138	3.718115370	-1.121107861	-1.510542144
Fe139	4.049434593	-2.485148889	-2.922015113
Fe140	2.256981765	0.155531033	-1.188341981
Fe141	4.772813570	0.162337234	-0.447616819
Fe142	4.182209796	0.127935142	-3.027938246
Fe143	3.799376104	2.329532246	-1.522619900
Fe144	4.781238769	-2.371272095	-0.496658360
Fe145	2.301563225	-2.348187254	-1.040778593
H146	-7.034147659	-7.020625172	-0.006470182
H147	-6.997719347	-6.137094041	-1.379292987

	H148	-10.233783469	-3.952354326	0.410970269
	H149	-9.312204788	-5.528906527	-1.139367400
	H150	-9.385242861	-6.529202637	0.305058936
	H151	-8.338659307	-1.986962927	1.079819219
	H152	-9.576051021	-4.568464656	3.182638973
	H153	-10.025616570	-2.024136344	1.636942924
	H154	-9.025143647	-5.107017050	5.369201342
	H155	-6.901308827	-5.072294529	3.176976364
	H156	-8.768132528	-3.377609939	5.702738365
	H157	-3.199728881	-1.481218776	2.677767391
	H158	-5.621578158	-3.052830891	2.570762362
	H159	-4.648424370	-5.700346653	3.161946718
	H160	-5.238477580	0.927461410	6.188467346
	H161	-6.226908613	-1.326916612	4.667172743
	H162	-4.579699791	-5.558042205	4.935426159
	H163	-4.832374088	-0.919556331	2.241014731
	H164	-5.395262588	-2.609842219	7.013635139
	H165	-6.617345088	-0.121092572	6.607694908
	H166	-0.927660364	-2.993690672	3.155232976
	H167	-0.994444445	-2.006683871	1.688846671
	H168	0.581954078	-2.514476541	2.336647941
	H169	-1.370270866	-0.527019497	3.643164147
	H170	-4.749762118	-3.603519982	9.007820510
	H171	-1.636256098	2.470374987	4.409466240
	H172	-2.155254585	0.555375985	8.447127691
	H173	-1.760401256	3.874019924	8.535753618
	H174	1.479856459	-0.109664989	2.547440518
	H175	0.489779421	1.127010417	3.350077198
	H176	8.682261676	1.694953625	5.719896114
	H177	-2.415407090	4.008624197	4.856134793
	H178	-2.434320313	4.823525200	7.193676390
	H179	-0.640389419	3.870124184	4.888961281
	H180	-0.668116054	4.679986567	7.382537614
	H181	-2.593556163	2.366700784	6.730742111
	H182	0.473388121	2.418355889	6.648267536
	H183	-1.496351472	0.406504002	5.789175622
	H184	-0.016645060	0.467824737	1.792556609
	H185	1.228185285	3.925892872	0.165254493
	H186	0.346599293	4.572550521	1.589037765
	H187	2.415534777	4.938656579	2.212189502
	H188	2.731156356	4.708880160	4.872654322
	H189	4.678818867	3.803019783	2.059323418
	H190	6.473623821	2.095583228	2.178823173
	H191	5.715812935	1.601729709	6.400061820
	H192	-1.557138322	-2.189120268	5.415576709
	H193	-1.117042605	-4.971200649	6.442973984
	H194	-0.561695845	-4.379507914	8.027539795
	H195	-3.023111779	-3.554594452	6.592263240
	H196	-4.204169264	-2.030480161	9.632336411
	H197	3.896123646	3.279809602	6.267345584
	H198	1.252317146	-1.687226067	4.677692481
	H199	-1.662051399	1.570963354	10.959251509
	H200	-2.075964968	-0.134064729	10.629876691
	H201	0.610651737	-0.386615725	10.238627530
	H202	9.874836094	-7.543466477	6.104677819
	H203	-1.691155811	-6.149194675	-3.841445126
	H204	0.936436154	-6.167201468	-1.052346793
	H205	-1.619165692	-6.711088430	-8.211830197
	H206	-0.022308534	-5.983944637	-8.542063226
	H207	-2.464170774	-4.678468622	-7.210144422
	H208	-1.635924185	0.552813747	-10.078825086
	H209	-2.276729158	-4.719989640	-9.744144158
	H210	-3.223547213	-2.159525480	-2.514754059
	H211	-4.285196025	-4.785082564	-1.349726498
	H212	-1.141771977	-1.498706133	-8.578925856
	H213	-3.865813746	-0.612353916	-9.273734516
	H214	0.138976527	-3.079009233	-9.340894973
	H215	-1.498316949	-5.896470134	-2.108118861
	H216	-0.871323971	-3.919553741	-7.149549009
	H217	-0.026964257	-4.483634758	-10.186579993
	H218	-6.612553854	-3.141086389	-3.530698685
	H219	-3.632165669	-2.241424291	-0.792093313
	H220	1.401966150	-5.855768864	-6.954351261
	H221	-2.634416754	-4.304639917	-0.980776815
	H222	-4.009009531	3.154435234	2.367590213
	H223	-2.799481132	1.090145158	1.041275817
	H224	-8.465315869	1.626590389	2.581094330
	H225	-7.308427854	0.542261317	0.049638772
	H226	-6.889403926	4.742319922	1.129700331
	H227	-8.211268413	9.618840614	2.644478973

H228	-7.260139809	6.133555109	3.984821488
H229	-8.929061559	6.301628405	1.791519893
H230	-6.095320727	7.462746963	1.457458133
H231	-10.150217812	10.340262478	-1.050518107
H232	-3.171670769	4.738408714	0.602507622
H233	-4.578220316	4.369948199	-0.407779520
H234	-9.380291066	11.342109396	1.167042199
H235	-8.112838393	6.347116586	0.226738957
H236	-6.124637490	2.585042027	0.385487234
H237	-7.438900282	0.190618896	2.513887031
H238	-9.527533212	2.637873155	-1.709878729
H239	-1.489357723	1.721228745	-2.948125978
H240	-10.032623725	0.941297096	-1.594894087
H241	9.978821098	2.726717736	4.004555787
H242	8.434561589	2.885556479	3.495241468
H243	1.979258888	5.680214436	-1.514604274
H244	-0.658995040	5.601686750	-0.262787070
H245	2.112738995	7.807451743	0.016974552
H246	-2.927599529	3.786688916	-2.263910223
H247	-4.328384642	4.303336130	-3.720638978
H248	-5.755255435	3.517353728	-5.270348100
H249	-4.077737788	5.626440358	-2.866488910
H250	-4.767435975	1.318023748	-4.843160918
H251	-6.552014853	1.479100944	-4.907134965
H252	1.053238767	11.414632419	-2.558946629
H253	-0.015511155	12.829316157	-2.622079502
H254	0.269745447	9.931517646	-4.031845621
H255	-1.444891851	9.548597476	-4.209315297
H256	-2.072083263	8.909731051	-7.118250047
H257	-1.514392069	4.508165407	-6.409806702
H258	-6.496671376	9.119726358	-2.139267087
H259	-2.728075671	8.895487312	1.102810497
H260	-3.550961616	7.825126488	-4.094350486
H261	1.952479665	4.948366687	-4.057770398
H262	3.166253406	6.723540942	-3.187201383
H263	-0.561275532	4.550043023	-3.921039914
H264	-8.060226700	0.300392043	-2.609001522
H265	4.803381063	9.364261864	-4.204531678
H266	-5.677217000	-0.751191825	-3.787806072
H267	1.707857261	5.885344392	-5.529032840
H268	-2.883560163	7.238864128	-0.709694594
H269	4.266738771	9.836536312	-2.579532815
H270	-1.436457575	7.360100166	-7.573880642
H271	-3.741544467	8.907422474	-5.428157252
H272	-4.995149030	-3.417502399	-3.848795856
H273	-5.940061452	-2.962510879	-1.346993262
H274	-6.267103277	10.871554557	-0.387630465
H275	-4.822898506	1.619528157	-2.373216635
H276	0.550927418	7.671278700	-4.266756036
H277	-0.441286989	5.889082792	-6.661221843
H278	0.325354209	3.811108863	-5.246781156
H279	-2.575584313	5.795604949	-4.532236535
H280	0.996467421	8.960523777	2.453069914
H281	0.180479936	9.734271845	-0.588314859
H282	0.820304895	12.564659165	-0.390383536
H283	2.597066675	9.248526651	1.736342135
H284	-1.881361547	10.900668368	0.700890978
H285	5.534912598	-3.956839652	-8.100038555
H286	5.279123189	-5.323359691	-6.996473925
H287	8.002859313	-6.109843365	-3.546169225
H288	5.966015444	-6.163150431	-9.119368012
H289	4.367103390	-6.501139996	-9.011063429
H290	5.739657611	4.332899567	-0.343717004
H291	6.164026877	7.114231609	0.875644637
H292	5.738619114	7.424438965	-3.338354416
H293	4.419534754	5.395636730	0.256787648
H294	7.375536687	7.502746461	-0.160374278
H295	6.417417074	1.848960702	-6.016975337
H296	6.539575659	6.082850912	-1.849943042
H297	3.977848011	-6.884275368	6.126292864
H298	5.962580248	-8.115364090	4.139410344
H299	4.556639375	0.569332989	-6.256331209
H300	4.418683366	-0.045474925	4.074413648
H301	1.857035792	0.831733268	-8.524458116
H302	-0.887519021	-2.568718913	-4.196073807
H303	1.437894694	1.540642467	-6.965633761
H304	3.291507931	-4.370139070	5.428414658
H305	2.864983104	-0.507431243	-6.032716623
H306	3.144582769	-4.956428607	3.771039351
H307	3.689769381	-7.242989340	4.407989614

H308	5.586891499	-5.613663915	3.826713924
H309	5.652093280	-5.146056719	5.533098625
H310	-4.332695426	-0.944007765	-7.010644163
H311	4.396502942	-9.370338680	6.364048363
H312	3.578153846	-11.865521435	4.788841904
H313	0.638572283	-0.612055340	-3.838356445
H314	4.545684112	-11.621690540	7.532519405
H315	3.037047134	-11.938493725	6.981860076
H316	4.872760811	-3.331787558	3.101419715
H317	0.121438798	1.562270393	-4.864076660
H318	-2.865175971	-2.334609301	-5.675337067
H319	6.914428667	-10.569560163	0.134726690
H320	6.292340525	-8.191262396	-2.077323249
H321	5.818726279	-8.636991412	1.432291459
H322	6.185140471	-11.150609458	-1.105769485
H323	6.269313833	1.557942319	-8.778955163
H324	5.854848331	-5.494578580	1.051669905
H325	1.372345896	-7.946421629	1.684408225
H326	4.108182646	-2.762398266	-6.771550078
H327	2.304028995	-9.416820544	0.237076475
H328	3.317529806	-7.555745647	-5.897053250
H329	2.343845920	-5.892157225	-4.260344045
H330	5.094753875	-12.503722729	5.433241538
H331	4.866108363	-1.097675157	2.754525568
H332	7.654048831	-6.350869656	2.374271106
H333	6.901583930	-4.355046414	1.113081474
H334	7.929723625	-7.262823167	4.540146507
H335	3.009968949	-2.571183886	6.087981332
H336	3.509107246	-8.688814773	-2.483396076
H337	4.477249170	-9.024282678	-1.041655012
H338	1.402085069	-8.897467399	-1.195307923
H339	1.985134389	-2.628594118	-5.590874989
H340	-1.866349865	1.804612205	-6.374313964
H341	-4.057614858	0.807190985	-7.202571536
H342	1.615187245	-6.207560805	1.566851266
H343	7.681960154	-7.902332543	2.319389592
H344	6.386113475	-9.702829918	2.400722202
H345	3.044354463	-0.907306779	5.770236223
H346	5.666524317	-8.339833135	-5.200331562
H347	6.348892775	-6.482215312	-1.686543640
H348	1.231812971	-3.495228545	-6.961848545
H349	5.459176478	3.298673409	-6.395504523
H350	7.087550288	-0.070055168	5.129346467
H351	7.396814614	0.159964564	3.393674889
H352	9.026302876	-7.986775764	-4.538393668
H353	8.033116456	-9.048357977	-3.787636711
H354	9.692023018	-9.412118313	0.359659609
H355	8.164154870	-9.048170159	-1.281798795
H356	11.411431756	-8.556790738	-1.609386916
H357	9.601059283	-7.675389919	0.719755042
H358	10.070956643	-0.235666503	3.751236382
H359	-2.482646044	13.666752203	2.398936058
H360	8.744681692	-7.173757322	7.436301962
H361	8.482070399	-4.794964036	6.105111675
H362	-3.920365754	12.695163463	-0.067407118
H363	-3.793674169	10.807939602	2.369371818
H364	-5.259255021	11.587678675	1.740337416
H365	1.507664539	-4.275035315	0.879799109
H366	-5.636585968	7.162266525	-2.965459337
H367	0.086029659	-1.114922205	0.221157374
H368	2.278136799	-1.362499474	0.125289707
H369	-1.420371825	0.171892174	-0.968361952
Mo370	3.586547694	-4.716631763	-1.423684987
N371	-7.611524703	-6.704551384	-0.789888069
N372	-9.341379885	-3.839151172	0.884619985
N373	-8.976410566	-3.907974824	3.671601677
N374	-6.382119882	-4.945376864	4.047091300
N375	-4.776858929	-2.812521582	3.085337204
N376	-5.421728771	-0.835907157	5.062090147
N377	-4.792845643	-2.049932516	7.623788045
N378	-2.339672296	-3.536323366	7.351509162
N379	-0.548699279	-2.194237464	5.567776341
N380	-0.573305986	0.711104029	6.107306820
N381	-1.332614042	0.863040896	8.972213014
N382	3.864854558	-11.365269912	6.814208769
N383	4.958968011	-9.290235746	5.510398948
N384	4.362530460	-3.269395577	3.984303790
N385	3.530870632	-1.811446249	5.643593356
N386	4.402572676	-0.977137801	3.667345671
N387	7.840581779	-7.296229468	5.579830596

N388	-5.677603643	-2.732448691	-3.474829312
N389	-1.998169695	-5.686688751	-2.979278723
N390	-5.747548833	-0.174160038	-2.931381903
N391	-8.046560729	1.205178402	-2.143524201
N392	-7.952335745	1.058179939	0.659232865
N393	-5.763532076	2.675390266	1.339625676
N394	-2.663775402	2.985252826	-1.625401255
N395	-1.962523125	1.057946525	-0.953164130
N396	-6.331617721	5.377845422	1.694902228
N397	-9.302114542	8.506434530	-0.300417575
N398	-9.246053957	10.369856403	0.902418445
N399	9.172095298	2.196203699	3.665910303
N400	6.751911901	6.741602720	0.122896821
N401	5.076718467	7.909052861	-2.736880696
N402	2.467815880	7.400996769	-3.514249410
N403	-2.031406864	6.205312368	-5.312283366
N404	-1.887389181	7.941552429	-6.876701501
N405	-3.173071595	8.200211198	-4.968137188
N406	1.038891866	6.100651245	-1.370934836
N407	1.302093279	8.095482222	0.571462758
N408	0.298214495	10.604071785	-0.070353910
N409	-0.716571889	10.183137737	-3.882762111
N410	-2.092268983	11.888040678	0.579700319
N411	5.037975622	-5.734338435	-9.115153233
N412	3.289868038	-3.283779714	-7.095390601
N413	2.335968919	-0.371425520	-6.908479062
N414	4.552485875	1.425827360	-6.818979956
N415	-0.517244836	-3.632531686	-9.904181461
N416	-2.148434365	-1.585420857	-8.746976011
N417	8.077503236	-8.098820330	-4.169776187
N418	4.204929785	-6.142650738	-3.192498618
N419	3.818922725	-7.212351854	-5.081995910
N420	8.861391802	-8.305004510	-1.179238333
O421	-7.181897420	-4.539642547	1.051779400
O422	-7.856052347	-1.925885379	3.558291517
O423	-6.403066842	-4.124817234	6.178742333
O424	-3.185065636	-3.481845437	4.566957589
O425	-3.330928511	-0.004185985	4.707066203
O426	-4.075839917	0.036175396	8.223056552
O427	-1.941055368	-3.127180957	9.573065369
O428	1.192142482	-3.306751999	6.573836103
O429	1.685455871	0.387841481	5.987863762
O430	0.542825948	2.135664160	9.096854474
O431	0.339309323	0.389407315	12.108323613
O432	5.843917458	-10.620865962	3.839103500
O433	6.465595942	-7.722341823	7.373715539
O434	10.464280657	-5.036626241	6.518955477
O435	-5.654799484	-0.437016919	-0.654199660
O436	-3.554217537	-4.492497221	-4.130594408
O437	-6.744190016	2.979627215	-1.531014303
O438	-5.528910522	3.217741397	-4.371975936
O439	-9.836627400	2.362327294	0.806089190
O440	-6.023571908	2.186535554	3.556639586
O441	-4.413903771	5.628401551	2.912321228
O442	-6.747229079	8.105879976	3.906209869
O443	10.003148480	-0.505178094	5.773254992
O444	3.169045761	4.575344159	4.012104264
O445	4.176385967	8.063712094	-0.646149653
O446	2.119201774	9.602087860	-4.081626858
O447	-0.840453599	7.005720549	-2.309259342
O448	-0.629783809	7.357674619	1.563876979
O449	2.281672941	5.317589895	1.320309797
O450	1.407855495	11.572053516	1.683861003
O451	-1.504794316	13.755059230	-0.590067596
O452	-2.243113883	11.136901788	-2.477132676
O453	-4.395325685	14.260848289	1.990015597
O454	-4.748701457	7.126612728	-2.559459931
O455	2.546894290	-5.256688091	-8.006812367
O456	0.878540401	-1.389525438	-8.355838494
O457	3.443120930	2.879775727	-8.172067635
O458	7.207631821	3.316698621	-8.344366268
O459	-3.914445774	-2.954438030	-9.195584250
O460	-1.154531075	-6.947065535	-5.636074843
O461	0.895508044	-6.263678271	-6.186319874
O462	-2.562997312	1.952446497	-8.925788331
O463	9.751258824	-6.312813685	-1.799636195
O464	12.160339822	-7.957539111	0.192176535
O465	-0.144877849	-6.250692324	-0.694780083
O466	-1.048476484	-7.246723240	1.109372685
O467	4.608589493	-11.368410962	-1.866072035

O468	2.372027423	-11.252893989	-2.180259927
O469	4.557629588	-6.153154869	-0.091836669
O470	4.245446967	-7.797279573	1.406440247
O471	2.205823891	-6.349596476	-1.162896183
O472	8.132228480	-7.128630840	2.757170743
O473	6.502434117	-5.055840925	1.660947258
O474	-3.617545743	4.879796526	-3.327056033
O475	7.084916500	-11.115408234	-0.664959458
O476	6.640348757	-9.166759101	1.607173078
S477	3.941989148	4.575941133	-1.969581120
S478	2.303032566	1.448961384	-3.015524086
S479	2.099010925	-3.606626215	-2.921598299
S480	3.137369012	1.464999008	0.441242388
S481	0.319503773	-1.192475506	-1.111703184
S482	4.682176386	-1.271133916	-4.681027044
S483	2.743681662	-3.692674173	0.647198941
S484	5.812009582	1.362201312	-1.995643576
S485	5.684787023	-3.696853906	-1.920627681
S486	6.055428058	-1.183513285	0.759084001
end			

TS

Fe(139)	-2.436
Fe(140)	-1.369
Fe(141)	-2.379
Fe(142)	2.805
Fe(143)	2.863
Fe(144)	1.999
Fe(145)	-0.944

bm522bh263b321tc.car_1

C1	-8.333307022	-4.709779804	0.586419591
C2	-8.719204116	-5.896298330	-0.303989151
C3	-9.082011085	-2.598247514	1.592648147
C4	-8.578542247	-2.779970834	3.021473562
C5	-8.512096707	-4.188584791	5.017493311
C6	-7.000322995	-4.406948112	5.127968805
C7	-4.929493584	-5.066046907	4.016320305
C8	-4.214608560	-3.716913643	3.919112894
C9	-4.247982120	-1.452509656	3.000661844
C10	-4.287836481	-0.691544686	4.334280502
C11	-5.550104957	-0.105281335	6.319688702
C12	-4.719090557	-0.699317704	7.464417085
C13	-4.174450198	-2.700640826	8.764800200
C14	-2.709889339	-3.129490076	8.606722165
C15	-1.018442619	-4.068368256	7.088226739
C16	-0.023304151	-3.137365451	6.389578667
C17	0.271292370	-1.219225999	4.837094549
C18	0.529757613	0.033472476	5.706490695
C19	-0.348614454	-0.887441498	3.465448588
C20	0.450046671	0.214131905	2.761274529
C21	-0.435212727	-2.159302867	2.615795652
C22	-0.476645295	1.945192871	6.909443613
C23	-0.373192838	1.659306600	8.423246264
C24	-1.637633072	2.912876944	6.569348638
C25	-1.619150308	4.142909013	7.483920932
C26	-1.574940611	3.340470706	5.097075841
C27	-1.345840125	0.669142251	10.418498594
C28	-0.005506928	0.224203938	10.967594707
C29	4.413268244	-11.646495698	5.478396116
C30	5.153186344	-10.458442043	4.865170540
C31	5.538554628	-8.007830612	5.145430010
C32	6.656915840	-7.661482005	6.146735490
C33	4.449199126	-6.920086242	5.130671520
C34	4.981715826	-5.539929379	4.741252757
C35	3.847367337	-4.546215008	4.495085077
C36	4.090093559	-2.037906470	4.432580317
C37	8.985863458	-6.943959465	6.390593211
C38	9.332216720	-5.472536968	6.331686941
C39	-5.339636539	-2.413615451	-2.086765578
C40	-5.613733692	-0.926277164	-1.801531590
C41	-3.847566639	-2.682563464	-1.786311615
C42	-3.429271619	-4.165960119	-1.722608634
C43	-2.992715849	-4.783761308	-3.050995802
C44	-5.735764582	1.272871945	-2.906837380
C45	-6.906782591	1.890195404	-2.128336870
C46	-5.664341272	1.779103657	-4.353251054
C47	-9.225239318	1.616224004	-1.376289205
C48	-9.032047305	1.713580822	0.140968345
C49	-7.619255499	1.178546319	2.073345952
C50	-6.407064000	2.053229825	2.391388438
C51	-4.675351355	3.583984758	1.598258512
C52	-5.140496680	4.948181578	2.159029469
C53	-3.891172634	3.918420431	0.321964882
C54	-3.138350242	2.808324429	-0.332222837
C55	-2.693928173	1.578153714	0.084263874
C56	-1.988562223	1.877045854	-2.003113233
C57	-6.819225404	6.732434257	1.882480741

C58	-6.953491838	7.005153235	3.379185647
C59	-8.191382306	6.852382308	1.177967996
C60	-8.677747367	8.241948249	0.888724224
C61	-8.624757188	9.395936659	1.645934324
C62	-9.655842083	9.780456942	-0.276743995
C63	8.717909850	1.250326788	4.708718095
C64	9.679382886	0.077430126	4.743158550
C65	7.322257779	0.674099475	4.358515459
C66	6.242534313	1.729715303	4.304779829
C67	5.513514132	2.093118521	5.451447746
C68	5.926568743	2.371625906	3.093596108
C69	4.494364420	3.050581889	5.391212201
C70	4.922524260	3.337364082	3.020915997
C71	4.197622619	3.666957881	4.169370442
C72	5.904266566	6.353638130	-1.013620334
C73	4.986633965	7.509936381	-1.437758342
C74	5.070467516	5.132822743	-0.618122565
C75	4.325251339	9.004384741	-3.289854034
C76	2.871584794	8.688419458	-3.650418537
C77	1.134019177	6.904191307	-3.728252578
C78	0.375485779	6.682977687	-2.395242899
C79	1.244454047	5.606037928	-4.562766243
C80	-0.022790022	4.786357198	-4.833622444
C81	-0.999585999	5.344130529	-5.879806079
C82	-2.383391512	7.418168811	-5.726609695
C83	0.409107490	5.916830912	-0.077042138
C84	0.316917127	7.204750694	0.759756995
C85	1.085667738	4.835379310	0.782728307
C86	1.537872735	9.194292506	1.512186537
C87	1.086948227	10.572012563	1.046188733
C88	0.020460962	11.877743930	-0.720095234
C89	-1.275520319	12.585629869	-0.257894836
C90	0.071392479	11.799429199	-2.249440151
C91	-1.070008635	10.995611242	-2.861802036
C92	-3.443689637	12.349369539	0.862324843
C93	-3.427108440	13.524847529	1.830383067
C94	-4.282603954	11.177811617	1.447087896
C95	-4.459896425	10.053323337	0.451794278
C96	-5.522439782	10.080341740	-0.465832168
C97	-3.545986593	8.989432691	0.375118129
C98	-5.657357961	9.096478238	-1.447236761
C99	-3.649499505	8.010903479	-0.617814492
C100	-4.701751477	8.082065858	-1.526438022
C101	4.933052960	-4.862660103	-7.943899917
C102	3.482486077	-4.480565379	-7.701299642
C103	1.986216435	-2.766004004	-6.701788437
C104	1.662910561	-1.443584092	-7.406276266
C105	2.201687951	0.954386513	-7.496282329
C106	3.460202477	1.816875923	-7.539669567
C107	5.708058695	2.297219194	-6.737962411
C108	6.465850709	2.378541788	-8.048286825
C109	-1.734644751	-3.982549635	-9.140569552
C110	-2.706280409	-2.801483014	-9.033498816
C111	-1.508489173	-4.596363712	-7.740719694
C112	-0.867025246	-5.993358363	-7.838356621
C113	-0.405725806	-6.460910721	-6.477114698
C114	-3.013967375	-0.416182032	-8.603239370
C115	-2.359995471	0.793628831	-9.238843131
C116	-3.571001818	-0.180735166	-7.178306023
C117	-2.483696504	-0.246691733	-6.140640112
C118	-1.659261630	0.862424582	-5.888390785
C119	-2.196382358	-1.464635357	-5.500335653
C120	-0.539349709	0.735817759	-5.060484175
C121	-1.077922350	-1.590667293	-4.671076709
C122	-0.233977461	-0.495304619	-4.469652617
C123	7.861252172	-7.090908628	-3.104854538
C124	8.896364018	-7.190961987	-1.971917948
C125	6.440180227	-7.203033971	-2.529643640
C126	5.312467152	-6.965164841	-3.483952352
C127	5.067858445	-7.626540127	-4.668948955
C128	3.329414648	-6.346789485	-4.183495697
C129	9.802476815	-8.405835193	-0.077585710
C130	11.236308525	-8.272622647	-0.539546793
C131	-0.052053109	-6.910129019	0.454901061
C132	1.371699205	-7.123156913	0.970659514
C133	2.446247559	-7.331449127	-0.138668363
C134	2.358777477	-8.790009990	-0.666639730
C135	3.519136517	-9.255126119	-1.548112318
C136	3.489663857	-10.764355735	-1.885485202
C137	3.847102061	-7.113601657	0.458062491

	C138	3.711009751	-1.125609697	-1.510186778
	Fe139	4.066169844	-2.510112105	-2.913142057
	Fe140	2.292965336	0.115417459	-1.102382446
	Fe141	4.797887286	0.137530901	-0.445199541
	Fe142	4.137379168	0.103065139	-3.024707001
	Fe143	3.768395731	2.308281873	-1.483521373
	Fe144	4.708351877	-2.451511837	-0.393889682
	Fe145	2.302945495	-2.351114057	-1.068739275
	H146	-7.036569517	-7.018901328	-0.029547617
	H147	-7.010751356	-6.130237075	-1.397106168
	H148	-10.235178671	-3.945132093	0.402452094
	H149	-9.315215744	-5.520965505	-1.151624794
	H150	-9.400115194	-6.534907910	0.283970718
	H151	-8.325125521	-1.994336668	1.070979172
	H152	-9.570453924	-4.564151776	3.182929166
	H153	-10.014054305	-2.018297711	1.626225188
	H154	-9.027379687	-5.090545432	5.373965136
	H155	-6.898041612	-5.065207299	3.174946453
	H156	-8.758172180	-3.362201000	5.698241332
	H157	-3.200546501	-1.471735691	2.676560522
	H158	-5.619013255	-3.045059663	2.572946865
	H159	-4.643132523	-5.688210114	3.157251072
	H160	-5.226080474	0.935533626	6.191103488
	H161	-6.226538490	-1.311716074	4.666303630
	H162	-4.573449313	-5.555429052	4.931068760
	H163	-4.834819777	-0.912797963	2.243516710
	H164	-5.396541127	-2.601872496	7.012575761
	H165	-6.608537109	-0.109048645	6.612892275
	H166	-0.928368338	-2.981785075	3.151842421
	H167	-1.014984895	-1.981253646	1.696857602
	H168	0.571977661	-2.492561254	2.322968367
	H169	-1.367979364	-0.509919416	3.650865343
	H170	-4.760875921	-3.600544073	9.001362359
	H171	-1.627439992	2.481735807	4.412526192
	H172	-2.137078130	0.542200257	8.446835278
	H173	-1.744189589	3.876614668	8.543843958
	H174	1.482423618	-0.101969781	2.550353636
	H175	0.495024843	1.137887463	3.355963120
	H176	8.666560894	1.671457090	5.731054110
	H177	-2.412923160	4.014392894	4.863108603
	H178	-2.438197108	4.823629300	7.207857413
	H179	-0.637303153	3.884999448	4.897424117
	H180	-0.670676189	4.693744892	7.380743857
	H181	-2.587270343	2.372870418	6.736485562
	H182	0.479737490	2.424377992	6.654339302
	H183	-1.493141853	0.422006969	5.787010430
	H184	-0.011202359	0.475224585	1.797120921
	H185	1.265298349	3.939353990	0.162138881
	H186	0.378767171	4.574190039	1.585556945
	H187	2.433183762	4.964383502	2.222994306
	H188	2.757566734	4.739130032	4.901104746
	H189	4.667334448	3.805053043	2.070272365
	H190	6.451550917	2.086871795	2.178815981
	H191	5.728176448	1.608797107	6.407537531
	H192	-1.556689884	-2.176105265	5.429919235
	H193	-1.112752062	-4.970829094	6.464879170
	H194	-0.559504155	-4.358857930	8.040802649
	H195	-3.015294685	-3.532505163	6.594114833
	H196	-4.211635165	-2.029676808	9.632041200
	H197	3.918087164	3.299162160	6.285985412
	H198	1.252052685	-1.682433851	4.682912253
	H199	-1.674451935	1.571644701	10.959310817
	H200	-2.061441310	-0.140465394	10.632463534
	H201	0.628370360	-0.357660001	10.259432725
	H202	9.867027328	-7.542316425	6.113157115
	H203	-1.703032854	-6.163233798	-3.841980266
	H204	0.914147283	-6.180175347	-1.065886820
	H205	-1.611960237	-6.709205149	-8.213276102
	H206	-0.019262111	-5.974975016	-8.538355034
	H207	-2.466122260	-4.680040113	-7.203132283
	H208	-1.672953374	0.555697143	-10.084502568
	H209	-2.291861746	-4.720255496	-9.738740756
	H210	-3.236954394	-2.155364610	-2.537838959
	H211	-4.264672508	-4.780090908	-1.343746487
	H212	-1.155845275	-1.495536016	-8.581073945
	H213	-3.888294314	-0.616264428	-9.256595988
	H214	0.121796544	-3.071723064	-9.347394026
	H215	-1.509413664	-5.910104956	-2.109273549
	H216	-0.875250107	-3.915275032	-7.151139247
	H217	-0.041168227	-4.480057012	-10.186108864

H218	-6.617440233	-3.162362825	-3.527156982
H219	-3.626675400	-2.225531221	-0.810904880
H220	1.405921814	-5.858975221	-6.949758920
H221	-2.615796061	-4.280066878	-0.990797359
H222	-4.018199835	3.146188371	2.363160692
H223	-2.830776698	1.054445108	1.021616748
H224	-8.478934762	1.627831573	2.589898794
H225	-7.328328882	0.524207709	0.063611209
H226	-6.900611876	4.731507166	1.126747055
H227	-8.188806575	9.614622615	2.614165791
H228	-7.285924608	6.119686011	3.973173160
H229	-8.941948256	6.285996356	1.758127240
H230	-6.107371244	7.456821744	1.456600871
H231	-10.175000246	10.330872618	-1.057424273
H232	-3.176548280	4.714024855	0.591276542
H233	-4.583026071	4.351038330	-0.417558171
H234	-9.368757366	11.338450327	1.145436263
H235	-8.108679341	6.345106270	0.203553202
H236	-6.142289274	2.568775598	0.390030537
H237	-7.453812636	0.189704170	2.526831742
H238	-9.555164683	2.609919743	-1.709281130
H239	-1.514862721	1.697022677	-2.964596518
H240	-10.043245224	0.910669702	-1.574135271
H241	9.970319858	2.724738492	4.033105585
H242	8.427126421	2.891213490	3.521999214
H243	2.003279052	5.690631068	-1.516013593
H244	-0.630707807	5.609257065	-0.260277504
H245	2.131409165	7.824307869	0.017859638
H246	-2.938125781	3.769916904	-2.267574897
H247	-4.340759086	4.302426046	-3.719460885
H248	-5.770387448	3.517467839	-5.257377756
H249	-4.083823457	5.620146995	-2.861388238
H250	-4.788176678	1.315867071	-4.835937978
H251	-6.572203964	1.478622392	-4.898129890
H252	1.052371641	11.420486730	-2.569542155
H253	-0.026789112	12.828480977	-2.627972335
H254	0.275271880	9.915694321	-4.018490606
H255	-1.436179427	9.520720532	-4.190037541
H256	-2.064266081	8.915239724	-7.096689361
H257	-1.505428586	4.514911796	-6.397493490
H258	-6.499293514	9.120430204	-2.143360665
H259	-2.734586116	8.894596583	1.102343582
H260	-3.569438688	7.816607103	-4.089154459
H261	1.957620257	4.945808389	-4.043966206
H262	3.191916459	6.722119677	-3.201669983
H263	-0.559793376	4.577129876	-3.894565443
H264	-8.073239555	0.284399677	-2.604431821
H265	4.820718443	9.369144906	-4.199289636
H266	-5.690716814	-0.752849407	-3.785750798
H267	1.717961467	5.875402803	-5.520922046
H268	-2.892530085	7.231075007	-0.701969509
H269	4.294547690	9.834627373	-2.569410075
H270	-1.405563661	7.371440925	-7.544423284
H271	-3.757563847	8.899981861	-5.420938507
H272	-4.998088435	-3.418513530	-3.851734229
H273	-5.940151503	-2.972065189	-1.344331167
H274	-6.263746139	10.880900445	-0.399623548
H275	-4.841260813	1.616723015	-2.366686074
H276	0.573927060	7.675218520	-4.266635458
H277	-0.435550722	5.899059952	-6.640386929
H278	0.317175180	3.812193806	-5.213753997
H279	-2.575370486	5.796192405	-4.519219475
H280	1.011153431	8.973985206	2.452417201
H281	0.193480490	9.734417531	-0.592777914
H282	0.818454381	12.565477322	-0.399932329
H283	2.610512884	9.270389831	1.734283124
H284	-1.879292537	10.901920957	0.696023016
H285	5.541535103	-3.957663214	-8.088418913
H286	5.275613274	-5.330342453	-6.997059292
H287	7.999487778	-6.085934872	-3.530540527
H288	5.978074059	-6.147696976	-9.129532273
H289	4.382307924	-6.499303028	-9.020867396
H290	5.765448619	4.326641332	-0.349304008
H291	6.164931740	7.103206119	0.869052971
H292	5.765972770	7.431756018	-3.340691834
H293	4.435134620	5.376715982	0.243434544
H294	7.394221214	7.476622830	-0.148115962
H295	6.391503906	1.872239666	-5.986151155
H296	6.560583183	6.080993183	-1.855388950
H297	3.973017877	-6.885645577	6.125137868

H298	5.957063762	-8.114892475	4.136878927
H299	4.535352607	0.593059107	-6.229534765
H300	4.425251905	-0.050237803	4.074822528
H301	1.836358741	0.842677012	-8.524484686
H302	-0.854451145	-2.546229217	-4.191325801
H303	1.443492430	1.546841236	-6.955992390
H304	3.277916800	-4.372325639	5.414942526
H305	2.864869572	-0.515631035	-6.049502433
H306	3.141322376	-4.958122269	3.755162565
H307	3.683674048	-7.243422030	4.407006222
H308	5.579626211	-5.618386854	3.821528564
H309	5.643198438	-5.137911944	5.525361743
H310	-4.330248880	-0.952504084	-6.987911756
H311	4.392513699	-9.371247674	6.362299955
H312	3.581353623	-11.867469208	4.791668601
H313	0.662591564	-0.583889032	-3.856566724
H314	4.558596413	-11.624432872	7.531780793
H315	3.048909973	-11.949413349	6.987476205
H316	4.861742602	-3.332131480	3.091563110
H317	0.121733447	1.586908619	-4.879481392
H318	-2.845877218	-2.328455598	-5.658869484
H319	6.908340384	-10.569428922	0.136783123
H320	6.310559749	-8.213812307	-2.105009316
H321	5.819710565	-8.639490007	1.439421106
H322	6.179702421	-11.158461450	-1.102128368
H323	6.284743595	1.531001204	-8.751020832
H324	5.869818767	-5.515148106	1.039026029
H325	1.372254647	-7.967160161	1.669424096
H326	4.101199518	-2.752715225	-6.792957567
H327	2.313071306	-9.440445585	0.220427723
H328	3.316787690	-7.566653054	-5.900836526
H329	2.347880183	-5.898244407	-4.262347535
H330	5.101893981	-12.507585308	5.429913415
H331	4.868065131	-1.097650705	2.749714117
H332	7.657780419	-6.346699687	2.378782934
H333	6.900326172	-4.356697613	1.111402895
H334	7.926958422	-7.260489596	4.542945572
H335	3.007277734	-2.576307407	6.082118447
H336	3.502010696	-8.709946333	-2.503379553
H337	4.480338693	-9.032633834	-1.065266753
H338	1.404244608	-8.914611095	-1.200631407
H339	1.974599637	-2.637155499	-5.608195440
H340	-1.877711051	1.814710957	-6.377292857
H341	-4.069790683	0.800561640	-7.183729344
H342	1.621846475	-6.222658444	1.555251483
H343	7.682609920	-7.898882784	2.318378631
H344	6.391424856	-9.705118622	2.404994539
H345	3.053255609	-0.911626293	5.771023362
H346	5.666360559	-8.353575154	-5.204159250
H347	6.342545659	-6.510342699	-1.684867161
H348	1.227135368	-3.497148173	-6.987365404
H349	5.441295298	3.315034541	-6.414786570
H350	7.074785823	-0.082316335	5.117843795
H351	7.386787906	0.162765991	3.384336132
H352	9.049306216	-7.928189837	-4.550785307
H353	8.072534696	-9.018281843	-3.819613967
H354	9.689548121	-9.410678717	0.358568944
H355	8.162264133	-9.038098783	-1.282210321
H356	11.413257602	-8.554308891	-1.605548052
H357	9.600688886	-7.675333735	0.722131835
H358	10.037900808	-0.249563542	3.736796510
H359	-2.477614723	13.659396279	2.401361505
H360	8.733325215	-7.171396797	7.441622720
H361	8.477770904	-4.794577283	6.087948365
H362	-3.922587288	12.699538156	-0.067592811
H363	-3.795736776	10.810137884	2.364264890
H364	-5.258314649	11.592347697	1.738362405
H365	1.566734641	-3.765479475	0.703745798
H366	-5.653733185	7.150067799	-2.951238294
H367	0.021785110	-1.184769503	0.167034400
H368	2.238319531	-1.206247885	0.068489720
H369	-1.447804570	0.143876750	-0.992872979
Mo370	3.610625705	-4.754294369	-1.440522420
N371	-7.620528929	-6.702381925	-0.808061199
N372	-9.343897014	-3.840867670	0.881498516
N373	-8.973491205	-3.899930793	3.669894794
N374	-6.379517324	-4.940552785	4.045829456
N375	-4.776010990	-2.804662461	3.090034672
N376	-5.419804941	-0.824499447	5.062461586
N377	-4.786660773	-2.046246214	7.618888815

N378	-2.336649390	-3.525713671	7.357645918
N379	-0.546384353	-2.188940486	5.572279325
N380	-0.570151163	0.718935371	6.112181743
N381	-1.321755334	0.861729045	8.976157813
N382	3.873554983	-11.372228972	6.816027443
N383	4.954455904	-9.290796145	5.508351540
N384	4.355713113	-3.272568517	3.976705921
N385	3.535105888	-1.817619792	5.642944655
N386	4.401497730	-0.980160654	3.664364703
N387	7.834039525	-7.291992671	5.582079740
N388	-5.687490200	-2.741762831	-3.474766189
N389	-2.005210711	-5.695497058	-2.981038191
N390	-5.759750873	-0.179652007	-2.927294748
N391	-8.061059362	1.185495244	-2.131253842
N392	-7.966031349	1.050911201	0.670357211
N393	-5.777216789	2.666180743	1.342306473
N394	-2.679395880	2.963702381	-1.633711367
N395	-1.995806277	1.026682743	-0.973196394
N396	-6.339491584	5.371615024	1.683189150
N397	-9.326066434	8.495301354	-0.309552680
N398	-9.247242290	10.363215275	0.885508537
N399	9.164053053	2.199480329	3.685954919
N400	6.758644634	6.721892562	0.125428058
N401	5.096672874	7.905837800	-2.739714063
N402	2.489994928	7.401338421	-3.517409869
N403	-2.030649219	6.208603977	-5.297143479
N404	-1.917218894	7.935641434	-6.875950848
N405	-3.187818016	8.195056954	-4.959350649
N406	1.064566495	6.113008666	-1.368082393
N407	1.320361254	8.108224114	0.573393819
N408	0.301650576	10.605615981	-0.074859128
N409	-0.712007404	10.164733619	-3.875752308
N410	-2.091589556	11.888549568	0.571944335
N411	5.046708156	-5.726359400	-9.121083881
N412	3.285725596	-3.280900886	-7.113187792
N413	2.327737170	-0.372151043	-6.919497329
N414	4.542318492	1.433476706	-6.815636268
N415	-0.534008022	-3.629512973	-9.906960048
N416	-2.163346751	-1.584144910	-8.744318252
N417	8.101466282	-8.062332307	-4.187114023
N418	4.209510322	-6.154155189	-3.200559912
N419	3.820807971	-7.221270676	-5.088856243
N420	8.861388098	-8.297633956	-1.179244333
O421	-7.188229276	-4.552742598	1.046667634
O422	-7.845618852	-1.923207411	3.547540532
O423	-6.399442884	-4.118348885	6.176317643
O424	-3.182955497	-3.474416790	4.569261668
O425	-3.327972742	0.003914133	4.706733575
O426	-4.063536842	0.035893346	8.222804256
O427	-1.957069192	-3.148633656	9.589043625
O428	1.195436309	-3.312642645	6.564257520
O429	1.687342577	0.383601362	6.006070123
O430	0.537734951	2.157912896	9.106171370
O431	0.334638463	0.418975022	12.125353001
O432	5.846778279	-10.620862945	3.839923849
O433	6.456076138	-7.720343572	7.372510369
O434	10.450770972	-5.031725429	6.543761296
O435	-5.672507538	-0.449686339	-0.651098128
O436	-3.542972148	-4.481221428	-4.135131355
O437	-6.767723943	2.967053447	-1.520969306
O438	-5.547472273	3.216220846	-4.358545008
O439	-9.842781198	2.365826217	0.812169339
O440	-6.036287221	2.191143955	3.562791304
O441	-4.415894154	5.627050640	2.891280854
O442	-6.766233708	8.090129929	3.900793256
O443	10.030918426	-0.498440740	5.762632612
O444	3.179816976	4.593182779	4.034545973
O445	4.198130723	8.060063832	-0.648836655
O446	2.132680707	9.610651743	-4.049469140
O447	-0.817414990	7.019338223	-2.301328018
O448	-0.605874279	7.361104814	1.570096767
O449	2.310824297	5.330257401	1.324367125
O450	1.411129308	11.587548289	1.672335388
O451	-1.506342010	13.752676814	-0.602877744
O452	-2.243671221	11.130695705	-2.484152611
O453	-4.387931622	14.260800814	1.994732316
O454	-4.762972564	7.115937800	-2.552130915
O455	2.553215394	-5.267136600	-8.003842998
O456	0.866956118	-1.385685571	-8.366079277
O457	3.445755397	2.871283872	-8.196052036

	O458	7.244798473	3.278133752	-8.318282307
	O459	-3.929767665	-2.958232250	-9.176714740
	O460	-1.154956988	-6.947356515	-5.636992772
	O461	0.896703541	-6.266475544	-6.183413502
	O462	-2.584588929	1.951713290	-8.915699169
	O463	9.763416834	-6.312655843	-1.800866337
	O464	12.158922331	-7.951891695	0.196278542
	O465	-0.137720440	-6.259187462	-0.707252116
	O466	-1.045784069	-7.261045745	1.090615537
	O467	4.607539872	-11.388281449	-1.866119549
	O468	2.372687924	-11.270637101	-2.188134736
	O469	4.579585735	-6.185557977	-0.101359662
	O470	4.242474530	-7.814551120	1.407939261
	O471	2.221014673	-6.364373075	-1.172502371
	O472	8.134270500	-7.126719955	2.757076982
	O473	6.501317218	-5.061530605	1.654558233
	O474	-3.626012903	4.874013342	-3.325433872
	O475	7.078955278	-11.119162880	-0.660340895
	O476	6.642269132	-9.166752216	1.610685755
	S477	3.959984287	4.542373170	-1.974999789
	S478	2.262394823	1.406080911	-2.930343280
	S479	2.103681087	-3.641177907	-2.901204235
	S480	3.188209773	1.445593874	0.494671960
	S481	0.316341930	-1.150863894	-1.159266723
	S482	4.647385445	-1.288293920	-4.684306708
	S483	2.953682699	-3.730740166	0.694440134
	S484	5.772006692	1.337660417	-2.022071104
	S485	5.704003041	-3.708621236	-1.873190577
	S486	6.035195673	-1.212371229	0.807590714
	end			

product

	bm522bh263b321tb.car_5			
Fe(139) -2.250	C1	-7.780923924	-5.317716980	1.749802151
Fe(140) -2.450	C2	-8.333024807	-6.367244425	0.779050429
Fe(141) -2.423	C3	-8.334503213	-3.451920462	3.246790171
Fe(142) 2.710	C4	-7.502256332	-3.822140133	4.470205947
Fe(143) 2.927	C5	-6.900156485	-5.524728851	6.113534213
Fe(144) 2.311	C6	-5.402088738	-5.657998562	5.826478403
Fe(145) 0.019	C7	-3.650667765	-5.993091873	4.157769630
	C8	-3.020182103	-4.599224915	4.119844600
	C9	-3.338822124	-2.227852283	3.626036427
	C10	-3.070834162	-1.682463585	5.037887314
	C11	-3.820031861	-1.511605220	7.341820601
	C12	-2.714095913	-2.221649321	8.132281706
	C13	-1.814247708	-4.364388291	8.902147167
	C14	-0.418318417	-4.650236588	8.332179328
	C15	0.883790398	-5.220297220	6.325695526
	C16	1.640820329	-4.118935465	5.581358197
	C17	1.482014613	-1.970579176	4.356111629
	C18	1.904873779	-0.854713667	5.341130696
	C19	0.546844777	-1.470273353	3.236965216
	C20	1.130455391	-0.223517848	2.561437297
	C21	0.288427503	-2.592375689	2.226355514
	C22	1.165727080	0.766895163	7.055340407
	C23	1.649344417	0.252290921	8.428269192
	C24	-0.069769279	1.694781913	7.164031213
	C25	0.136691930	2.761966028	8.244344208
	C26	-0.370224542	2.353810362	5.811404367
	C27	1.240445973	-1.094710241	10.408177614
	C28	2.678906497	-1.559885498	10.504209198
	C29	5.957538673	-12.032342155	2.184304854
	C30	6.490928743	-10.713137027	1.629083521
	C31	6.869059408	-8.319813204	2.227130691
	C32	8.195294852	-8.068479661	2.968923322
	C33	5.787705759	-7.314867501	2.664022252
	C34	6.173556487	-5.857772245	2.398536040
	C35	4.991847378	-4.914392196	2.622598927
	C36	5.123413669	-2.417018256	2.927742760
	C37	10.488206585	-7.234626464	2.768417772
	C38	10.776339062	-5.755452320	2.897204768
	C39	-5.612771736	-2.408830694	-1.132912765
	C40	-5.844328461	-1.004878426	-0.546028451
	C41	-4.086227703	-2.618494714	-1.256653156
	C42	-3.624776027	-4.061383242	-1.546237247
	C43	-3.526300713	-4.438543025	-3.024753156
	C44	-6.285256042	1.329956691	-1.207872452
	C45	-7.238607022	1.738749159	-0.074627398
	C46	-6.599267390	2.059056733	-2.520916099
	C47	-9.294917829	1.203050889	1.152011545

C48	-8.735144623	1.061745890	2.571253778
C49	-6.881827920	0.305682643	3.988501927
C50	-5.657914557	1.206636933	4.162847430
C51	-4.223213105	2.972132335	3.270259448
C52	-4.584869824	4.188446990	4.151430308
C53	-3.788859181	3.569551699	1.923756279
C54	-3.170478239	2.641779557	0.932445444
C55	-2.568706089	1.409864258	1.016903804
C56	-2.451061931	2.075541916	-1.102291819
C57	-6.331354053	5.864905991	4.592164620
C58	-6.102956828	5.866724174	6.102486599
C59	-7.835801417	6.001653899	4.260825413
C60	-8.417717238	7.381953815	4.351352751
C61	-8.218911628	8.391172424	5.273736419
C62	-9.686547984	9.024463780	3.738459915
C63	9.583838644	1.109562095	2.642047217
C64	10.564348267	0.024493970	2.240084896
C65	8.165995307	0.494181306	2.532672906
C66	7.069169742	1.459708557	2.915272709
C67	6.629642181	1.574585717	4.247205344
C68	6.438944110	2.259367967	1.944258595
C69	5.583576887	2.435930819	4.598806984
C70	5.405031614	3.133920699	2.282089284
C71	4.968836912	3.210373499	3.607753299
C72	5.271127928	6.847971500	-1.271793973
C73	4.232739702	7.978439672	-1.258937383
C74	4.604662727	5.519212862	-0.904582894
C75	3.092959976	9.700433134	-2.615186525
C76	1.607575975	9.342112689	-2.674281130
C77	-0.039162485	7.472652910	-2.656477819
C78	-0.445176908	6.977686996	-1.245009989
C79	-0.095475369	6.341558918	-3.711854411
C80	-1.359335709	5.479164254	-3.821242255
C81	-2.583807143	6.124145825	-4.484916237
C82	-3.929848661	8.056216217	-3.656487547
C83	0.172402034	5.854209253	0.835051729
C84	0.259061333	6.972119120	1.889902784
C85	1.056415327	4.686265236	1.305031321
C86	1.565941765	8.896166518	2.667573277
C87	0.990371513	10.303016735	2.574477332
C88	-0.503034749	11.818958915	1.367614864
C89	-1.667074190	12.351695785	2.239259247
C90	-0.827817477	11.990051965	-0.120682051
C91	-2.065109830	11.223327400	-0.574631004
C92	-3.490825508	11.782940293	3.782509940
C93	-3.272938251	12.791679100	4.902453775
C94	-4.110991742	10.470274983	4.339108560
C95	-4.496273284	9.510548098	3.235194430
C96	-5.752700541	9.609341395	2.618020138
C97	-3.596044872	8.548684648	2.747247973
C98	-6.093277452	8.801823123	1.530279051
C99	-3.909114816	7.750931295	1.642481586
C100	-5.156166436	7.892256225	1.040917508
C101	2.958215531	-3.179349459	-9.582944265
C102	1.602004367	-2.954883950	-8.939886716
C103	0.345187708	-1.521157405	-7.343927067
C104	-0.177120689	-0.126314613	-7.708479882
C105	0.260874015	2.282628759	-7.505257857
C106	1.447878282	3.222431496	-7.693961934
C107	3.814331979	3.710452152	-7.421543984
C108	4.173704020	4.114821356	-8.837907166
C109	-3.792213889	-2.577927888	-9.014642566
C110	-4.743008055	-1.494141722	-8.493244005
C111	-3.215319155	-3.386507917	-7.830240174
C112	-2.593816517	-4.709115913	-8.313592889
C113	-1.822845288	-5.370899090	-7.194406819
C114	-4.998863576	0.765525602	-7.600348155
C115	-4.530801658	2.101217608	-8.142526020
C116	-5.218111756	0.728458218	-6.068227076
C117	-3.920140260	0.557233104	-5.328928011
C118	-3.083734517	1.657225438	-5.075166689
C119	-3.464392073	-0.731210169	-5.000394969
C120	-1.799103263	1.464419300	-4.559366169
C121	-2.180978124	-0.923701622	-4.482848572
C122	-1.336662923	0.172889948	-4.286309128
C123	7.060114434	-5.968256402	-6.083065602
C124	8.341492991	-6.157106062	-5.253190953
C125	5.831494484	-6.242701729	-5.201647437
C126	4.493867322	-5.957048242	-5.808201509
C127	3.990195450	-6.422277833	-7.005158280

C128	2.378368167	-5.397788964	-5.883864280
C129	9.714006098	-7.577078856	-3.847095280
C130	10.990213387	-7.272270261	-4.599384529
C131	0.287373955	-6.880990161	-0.724230970
C132	1.804803778	-7.005235794	-0.598915102
C133	2.565833814	-7.002267514	-1.957996289
C134	2.373221767	-8.367056152	-2.676032923
C135	3.300395638	-8.624213381	-3.867185015
C136	3.212367720	-10.053396291	-4.450217840
C137	4.067761824	-6.806221521	-1.698492742
C138	3.260835871	-0.597387214	-2.505268658
Fe139	3.171801651	-1.711256335	-4.119565550
Fe140	1.917323277	0.483501720	-1.650036334
Fe141	4.625879069	0.529852817	-1.621098768
Fe142	3.313085998	0.841791132	-3.859917888
Fe143	3.297486200	2.764847743	-1.969831178
Fe144	4.570042258	-2.014873687	-1.965323203
Fe145	1.996164969	-2.170938376	-1.804394589
H146	-6.598778426	-7.396817988	0.455916780
H147	-6.936936669	-6.306857849	-0.710425070
H148	-9.695529633	-4.669457267	2.141818422
H149	-9.121649191	-5.897182074	0.169586184
H150	-8.836900153	-7.137524820	1.388020022
H151	-7.740697559	-2.723158290	2.675440990
H152	-8.350717264	-5.685732961	4.531728607
H153	-9.250210328	-2.951404900	3.590148610
H154	-7.289746613	-6.501087829	6.430554827
H155	-5.762013606	-5.989231185	3.822569272
H156	-6.998984322	-4.828971201	6.958172895
H157	-2.401369151	-2.132871521	3.064564073
H158	-4.727314256	-3.822895040	3.269454387
H159	-3.574443659	-6.452311767	3.162720168
H160	-3.575417973	-0.441879671	7.327419416
H161	-4.846805524	-2.480355401	5.714698934
H162	-3.065323193	-6.595560582	4.861942208
H163	-4.102744290	-1.614274774	3.126864406
H164	-3.436802080	-4.068147180	7.540675652
H165	-4.773036780	-1.648095980	7.870173716
H166	-0.039622321	-3.522100747	2.712172185
H167	-0.506655449	-2.303138584	1.521583131
H168	1.190552662	-2.812057958	1.635161302
H169	-0.407537824	-1.187261205	3.711890851
H170	-2.291908907	-5.335953637	9.095733578
H171	-0.568157991	1.614292364	5.022837426
H172	-0.023280855	-0.971515450	8.697360911
H173	0.283914389	2.322819538	9.241752602
H174	2.100416261	-0.432563369	2.085575482
H175	1.275875523	0.606696846	3.268900113
H176	9.770676754	1.363159931	3.702942295
H177	-1.254508783	3.003672633	5.892671481
H178	-0.742177638	3.421878992	8.289223422
H179	0.479685441	2.979265545	5.491484786
H180	1.016809161	3.383592761	8.015864018
H181	-0.935973160	1.072204317	7.453210281
H182	2.016421408	1.347142435	6.669862460
H183	-0.048811437	-0.624549272	5.958250327
H184	0.455510625	0.139330803	1.772033581
H185	1.095859315	3.919543159	0.510342265
H186	0.572142704	4.249837111	2.192168908
H187	2.712060108	4.660806164	2.389155133
H188	3.706212363	4.036272918	4.827866959
H189	4.909865538	3.732249646	1.517074959
H190	6.737371703	2.172002520	0.896854439
H191	7.096622598	0.961878391	5.023111649
H192	-0.117473381	-3.145444256	5.175632296
H193	0.670575404	-6.022675725	5.602395685
H194	1.569207608	-5.618023963	7.083248669
H195	-1.192460174	-4.761510634	6.410770909
H196	-1.662357930	-3.857028832	9.862805614
H197	5.238404629	2.486138235	5.634230292
H198	2.412902699	-2.332456753	3.904917468
H199	1.068109528	-0.304276837	11.156625254
H200	0.605719824	-1.956773268	10.666981270
H201	3.105619048	-1.981168614	9.564377916
H202	11.285768727	-7.711835958	2.179348390
H203	-2.440541125	-5.591035080	-4.323878438
H204	0.773433469	-5.861942633	-2.290716704
H205	-3.395507968	-5.390664315	-8.631394270
H206	-1.933379100	-4.525945362	-9.173243599
H207	-4.010684041	-3.609467434	-7.102213829

H208	-4.049739362	2.048467358	-9.147231875
H209	-4.457428171	-3.252195708	-9.574781735
H210	-3.704000739	-1.939834854	-2.036030044
H211	-4.312220349	-4.785483856	-1.075276868
H212	-3.160093842	-0.186352008	-8.180274014
H213	-5.994477443	0.621068902	-8.068622494
H214	-2.069175361	-1.524918314	-9.507229734
H215	-1.826468633	-5.596539407	-2.673110617
H216	-2.469681028	-2.769628991	-7.304812662
H217	-2.398156673	-2.795548850	-10.504401471
H218	-7.191863600	-3.002259846	-2.325910776
H219	-3.635415619	-2.303301305	-0.304287003
H220	-0.185343874	-4.587652840	-7.945901629
H221	-2.646887293	-4.228055192	-1.071117971
H222	-3.384585882	2.470604456	3.772848852
H223	-2.418230140	0.746761308	1.859974382
H224	-7.600018751	0.597236967	4.767371292
H225	-7.077633682	0.009658341	1.885133565
H226	-6.537202953	4.005803897	3.546644200
H227	-7.573983242	8.472182487	6.141761693
H228	-6.237676964	4.865554935	6.578802428
H229	-8.406173458	5.291292883	4.885620341
H230	-5.765782581	6.700274447	4.150076887
H231	-10.401258120	9.655719651	3.215639020
H232	-3.071638018	4.375886555	2.150318026
H233	-4.663957269	4.045423182	1.452898555
H234	-9.117334018	10.332725131	5.318606293
H235	-7.974500627	5.676608721	3.217239986
H236	-5.897241876	2.053946969	2.273497975
H237	-6.579440159	-0.729896820	4.204238437
H238	-9.714810883	2.215975011	1.086236048
H239	-2.241771094	2.075489983	-2.168866147
H240	-10.122629994	0.491011007	1.033513270
H241	10.583248030	2.755927275	1.940503912
H242	8.957221353	2.891991111	1.853401226
H243	1.378980955	5.982199531	-0.951864088
H244	-0.875351438	5.520321429	0.842019036
H245	1.802023955	7.844267160	0.849545522
H246	-3.521886285	3.897152671	-0.804364361
H247	-5.233027526	4.545346705	-1.788338318
H248	-6.969399846	3.905630226	-3.066907349
H249	-4.819237364	5.731286366	-0.806349426
H250	-5.871471295	1.731534657	-3.281385495
H251	-7.612818259	1.794301335	-2.858616404
H252	0.054348312	11.731805612	-0.722942816
H253	-1.039874389	13.057944001	-0.282690774
H254	-1.017814916	10.431141313	-2.186673301
H255	-2.715283287	9.973938640	-2.024346409
H256	-4.056006203	9.744562208	-4.816048350
H257	-3.188419381	5.351295722	-4.983866780
H258	-7.079913396	8.886328814	1.067414033
H259	-2.626636348	8.395764119	3.228812470
H260	-4.670011008	8.126522755	-1.735835520
H261	0.744887854	5.659414126	-3.505661531
H262	2.092420600	7.359506538	-2.671742508
H263	-1.642651921	5.061812919	-2.841876159
H264	-8.452396111	0.160691585	-0.523740346
H265	3.345126721	10.240637287	-3.536506085
H266	-6.403419184	-0.524438933	-2.398917781
H267	0.114014884	6.799538143	-4.692445234
H268	-3.171265444	7.059897228	1.234718686
H269	3.212518842	10.397542226	-1.773068940
H270	-3.499486358	8.331023078	-5.655850458
H271	-5.216996892	9.383837615	-2.785234696
H272	-5.699136946	-3.100409632	-3.070747575
H273	-5.991550750	-3.116737230	-0.372076417
H274	-6.483638744	10.331547097	2.987579448
H275	-5.292498904	1.647022078	-0.854499055
H276	-0.736933925	8.280584558	-2.902489736
H277	-2.250283190	6.824346248	-5.261222080
H278	-1.082866532	4.617904562	-4.447379877
H279	-3.762380980	6.264062918	-2.714904561
H280	1.275797654	8.486275558	3.645847044
H281	-0.251062970	9.701011438	1.068176126
H282	0.330737606	12.499590926	1.601396203
H283	2.659891771	8.995338589	2.647930403
H284	-1.967467815	10.499151753	3.003440175
H285	3.474045407	-2.217502061	-9.718265407
H286	3.547609427	-3.757778733	-8.841160013
H287	7.061968849	-4.907946942	-6.377310183

H288	3.715863018	-4.183386188	-11.187153311
H289	2.210750673	-4.665244912	-10.756275562
H290	5.375403154	4.737553903	-0.931082986
H291	5.971803081	7.300170216	0.595994860
H292	4.534952154	8.270206114	-3.270693290
H293	4.176347881	5.580230723	0.104563463
H294	6.880702753	7.941567547	-0.608803344
H295	4.690909117	3.201781353	-6.990022369
H296	5.705462115	6.760565617	-2.280448793
H297	5.568048536	-7.467366646	3.733941292
H298	7.025179209	-8.236426600	1.144778363
H299	2.852586751	1.858859069	-6.970590535
H300	5.289516283	-0.381621770	2.848378620
H301	-0.334742455	2.312873913	-8.426070179
H302	-1.829508813	-1.931339580	-4.252374966
H303	-0.362583398	2.729816257	-6.712604356
H304	4.679079323	-4.927316707	3.672740506
H305	1.290706835	0.644992611	-6.525378755
H306	4.126366696	-5.247784218	2.025444421
H307	4.875776739	-7.564824018	2.098977800
H308	6.512532715	-5.745697195	1.357951583
H309	7.007657040	-5.545826032	3.047288878
H310	-5.900859577	-0.105530621	-5.851132715
H311	6.089256134	-9.935337254	3.427230685
H312	4.991517760	-12.203820420	1.685786873
H313	-0.317477340	0.036515742	-3.926217781
H314	6.601561021	-12.323043767	4.118300001
H315	5.018082321	-12.674851821	3.898398207
H316	5.590069770	-3.425175026	1.242786043
H317	-1.131774547	2.312927493	-4.389500986
H318	-4.113788063	-1.594391864	-5.160482214
H319	7.030935605	-9.936812730	-3.333439083
H320	5.840837884	-7.304432722	-4.900182154
H321	6.245082441	-8.324093808	-1.488603310
H322	6.022980138	-10.376574800	-4.430491093
H323	3.821751147	3.413269116	-9.631876575
H324	6.118553585	-5.179067507	-1.363742925
H325	2.043126762	-7.904213576	-0.018169864
H326	2.361654544	-1.333354063	-7.951166569
H327	2.544150201	-9.150127574	-1.921011002
H328	1.986876787	-6.289883426	-7.754090535
H329	1.391695233	-5.025324594	-5.642860667
H330	6.641109561	-12.821957764	1.830296591
H331	5.444337835	-1.168015821	1.298749787
H332	8.193131559	-6.087757580	-0.639133767
H333	7.083332856	-3.962945058	-1.307916373
H334	9.009606360	-7.311997153	1.204625829
H335	4.504792450	-3.287776634	4.672393601
H336	3.057054302	-7.924343399	-4.680959402
H337	4.346273162	-8.438087800	-3.585437501
H338	1.321969176	-8.452768132	-2.992263271
H339	0.611098184	-1.570809708	-6.276111031
H340	-3.433933173	2.662342779	-5.319117229
H341	-5.721816416	1.666990968	-5.790825768
H342	2.129709100	-6.132259360	-0.006757899
H343	8.245684590	-7.605091910	-0.969889851
H344	7.073309929	-9.487808851	-0.894093538
H345	4.400382497	-1.597786793	4.645269073
H346	4.463157008	-6.988381786	-7.798334215
H347	5.929559846	-5.671117747	-4.271365490
H348	-0.442146121	-2.249142408	-7.553140096
H349	3.625712442	4.615579273	-6.823597648
H350	8.132931815	-0.390745390	3.186326614
H351	8.014232155	0.151560285	1.496341716
H352	7.877636762	-6.545747664	-7.873131151
H353	7.110116739	-7.766883497	-7.098291215
H354	9.741637546	-8.641850816	-3.567224435
H355	7.857919734	-8.136087275	-4.734775415
H356	10.917024270	-7.366042661	-5.709862772
H357	9.687188555	-6.991575532	-2.914908545
H358	10.632542133	-0.133949658	1.136388111
H359	-2.214804238	12.919030971	5.231128187
H360	10.516782449	-7.662604619	3.785373020
H361	9.875011242	-5.096748571	2.946136149
H362	-4.198291767	12.238164776	3.069445031
H363	-3.387158219	9.999600808	5.022704723
H364	-4.993225601	10.750611578	4.931459830
H365	1.084038855	-3.135027789	-1.118457839
H366	-6.399459569	7.154306543	-0.268601647
H367	0.073883364	-1.188229431	-0.045678832

	H368	2.176042837	-1.579917834	-0.433743128
	H369	-1.580991863	0.269558080	-0.559394480
	Mo370	3.307960905	-4.205317634	-3.040228885
	N371	-7.365294861	-7.004269387	-0.096959826
	N372	-8.719053652	-4.580703362	2.413040839
	N373	-7.682867726	-5.062831610	4.980035222
	N374	-5.049754487	-5.973821846	4.554679102
	N375	-3.794193845	-3.608805473	3.615652493
	N376	-3.980771457	-2.007922964	5.984238879
	N377	-2.709991227	-3.577292547	8.069240916
	N378	-0.348079588	-4.822864575	6.983477316
	N379	0.897254610	-3.099040350	5.083279030
	N380	0.917086642	-0.320424612	6.103775629
	N381	0.885335457	-0.673049010	9.060887731
	N382	5.752556828	-12.014839491	3.638862687
	N383	6.420668026	-9.679054218	2.491458956
	N384	5.318059701	-3.541720578	2.220727354
	N385	4.873335878	-2.430430816	4.253567743
	N386	5.185618539	-1.230031670	2.297150966
	N387	9.179500291	-7.519627704	2.213416169
	N388	-6.287894644	-2.536798991	-2.430062727
	N389	-2.532826643	-5.287361695	-3.348642561
	N390	-6.275257748	-0.100166644	-1.464632988
	N391	-8.344007542	0.974956693	0.076948661
	N392	-7.560771902	0.385109287	2.708551073
	N393	-5.321431841	2.026478304	3.120116582
	N394	-3.077696725	3.026229030	-0.397980151
	N395	-2.141593136	1.088704010	-0.256485925
	N396	-5.873759061	4.597469204	4.040621293
	N397	-9.338413684	7.789501372	3.398991617
	N398	-9.031626890	9.427623165	4.863574635
	N399	9.731476180	2.236219196	1.715325722
	N400	6.370328649	7.099819564	-0.327075345
	N401	4.008897104	8.586888148	-2.460316192
	N402	1.311740750	8.025572233	-2.685101102
	N403	-3.448645158	6.822466863	-3.527797648
	N404	-3.772211512	8.770879070	-4.784266678
	N405	-4.530369224	8.656731882	-2.599272586
	N406	0.492681519	6.307124688	-0.518093407
	N407	1.148633626	7.971508098	1.626889119
	N408	-0.041882254	10.478058299	1.694323958
	N409	-1.944842794	10.591741244	-1.771927613
	N410	-2.238241622	11.474640936	3.102125244
	N411	2.803235683	-3.839472777	-10.881078949
	N412	1.510811503	-1.866601246	-8.146802970
	N413	0.556896893	0.893179452	-7.210503906
	N414	2.691072566	2.787964564	-7.368948415
	N415	-2.827591282	-2.033177354	-9.976740583
	N416	-4.176328631	-0.309641883	-8.126256127
	N417	7.045653495	-6.768541588	-7.319723436
	N418	3.468150490	-5.305848236	-5.122774887
	N419	2.661597910	-6.057090529	-7.030393415
	N420	8.534541635	-7.370568986	-4.668043375
	O421	-6.564930229	-5.156355043	1.958491122
	O422	-6.704795851	-3.004587643	4.963627089
	O423	-4.575002085	-5.502495243	6.740360811
	O424	-1.867101506	-4.391104506	4.536556468
	O425	-2.070206988	-0.985673938	5.274356438
	O426	-1.907726819	-1.575991868	8.824520410
	O427	0.548251389	-4.774246097	9.096692033
	O428	2.870729995	-4.220514772	5.432472712
	O429	3.089632260	-0.476309657	5.416609432
	O430	2.692743659	0.692020897	8.941411343
	O431	3.313737469	-1.550301269	11.549003424
	O432	6.919076633	-10.662188872	0.457433694
	O433	8.315751229	-8.354467759	4.173480843
	O434	11.904324255	-5.292263174	2.960721718
	O435	-5.637543046	-0.718670223	0.649808159
	O436	-4.335093233	-4.005417392	-3.877890387
	O437	-6.976683155	2.709562896	0.657739588
	O438	-6.513827559	3.483757702	-2.316873524
	O439	-9.366352468	1.546627083	3.519468917
	O440	-5.022076678	1.185966622	5.222505358
	O441	-3.729983736	4.796860807	4.801160830
	O442	-5.843161300	6.858863625	6.760891973
	O443	11.217682657	-0.647723265	3.024529549
	O444	3.911788281	4.060553617	3.875396040
	O445	3.635932238	8.321946854	-0.223517559
	O446	0.764856090	10.260043598	-2.713879632
	O447	-1.593937079	7.197075108	-0.824594437

	O448	-0.425196745	6.912015813	2.919029178
	O449	2.366679823	5.157594164	1.620798886
	O450	1.435638276	11.211942187	3.285890478
	O451	-2.005134251	13.541112273	2.164129415
	O452	-3.117774287	11.226655523	0.081291749
	O453	-4.189480747	13.403961714	5.426420623
	O454	-5.437418967	7.114657876	-0.102857388
	O455	0.661091404	-3.764490021	-9.120786840
	O456	-1.180731221	0.034122983	-8.433106820
	O457	1.241161312	4.374772938	-8.108844642
	O458	4.833487690	5.105946780	-9.103601078
	O459	-5.964247019	-1.699412641	-8.393080088
	O460	-2.350481771	-6.037935857	-6.310954720
	O461	-0.492934307	-5.146822276	-7.168063784
	O462	-4.698371326	3.173452960	-7.578069754
	O463	9.188270313	-5.250129293	-5.138887293
	O464	12.051455150	-7.009444702	-4.050801687
	O465	-0.131991087	-6.109679920	-1.733919259
	O466	-0.492200372	-7.401574043	0.071874544
	O467	4.313282632	-10.607611764	-4.798305206
	O468	2.061430388	-10.561864072	-4.572116539
	O469	4.627780116	-5.776167250	-2.277180860
	O470	4.696457528	-7.618399208	-0.994762388
	O471	2.069850341	-5.907121833	-2.732703390
	O472	8.770481350	-6.883422734	-0.525959598
	O473	6.860544513	-4.786579157	-0.836236255
	O474	-4.463015408	5.095493372	-1.477780115
	O475	7.004773965	-10.341501197	-4.229093050
	O476	7.101041773	-8.810591682	-1.617467290
	S477	3.229888302	5.062849585	-2.057012313
	S478	1.489132828	2.044594828	-3.186738309
	S479	1.394203571	-3.032888481	-3.825109221
	S480	3.237017039	1.518263123	-0.112255131
	S481	0.061075361	-0.897731302	-1.369945385
	S482	3.291445198	-0.262256546	-5.789361025
	S483	3.401634857	-3.499581325	-0.785248861
	S484	5.130245742	2.046203969	-3.158311583
	S485	5.072939368	-2.891775584	-3.951515095
	S486	6.129745061	-0.915369257	-0.889208865
	end			

S2BH-6Hn-6Hx to 2N2x-S2BH-6Hn-6Hx

35, S=3/2

reactant

		bm522bh6x6hn2n2xt3c_3_53067.67			
Fe(139)	-2.388	C1	-2.128655055	-8.588998414	-3.739763933
Fe(140)	-0.801	C2	-1.462289557	-9.234437871	-4.959763317
Fe(141)	-2.265	C3	-4.257416484	-8.111631521	-2.616565251
Fe(142)	2.695	C4	-3.930945171	-8.524644427	-1.184610740
Fe(143)	2.769	C5	-3.074469987	-10.248795225	0.319463008
Fe(144)	2.374	C6	-1.933495426	-9.453777399	0.959280121
Fe(145)	0.393	C7	0.043964469	-8.065344847	0.626396744
		C8	-0.486512582	-6.715929024	1.112186684
		C9	-2.097071553	-4.908799478	0.762111448
		C10	-2.829609274	-5.011963622	2.108353050
		C11	-4.279388202	-6.311363919	3.554454964
		C12	-3.394201167	-6.634533812	4.764668305
		C13	-1.686786499	-8.120161253	5.699700298
		C14	-0.373876142	-7.397689129	6.025863895
		C15	1.617369952	-6.274333592	5.121923042
		C16	1.690112577	-4.745819604	5.063872723
		C17	0.595201074	-2.669245111	4.206924198
		C18	-0.239544845	-2.090327888	5.374968492
		C19	0.029492833	-2.311961811	2.818046674
		C20	-0.281117518	-0.813434731	2.694534558
		C21	0.989928918	-2.781914166	1.721015312
		C22	-2.449475514	-2.014065066	6.484812925
		C23	-2.317885208	-2.779745840	7.817511004
		C24	-3.906904267	-2.007974849	5.958168412
		C25	-4.898075086	-1.614739634	7.060177097
		C26	-4.047530876	-1.069208888	4.752534714
		C27	-2.448300763	-4.891453244	9.011038901
		C28	-1.333830142	-4.521897665	9.966726141
		C29	10.995412604	-6.947389690	4.022830222
		C30	10.659018671	-5.457495379	4.007626665
		C31	9.076695640	-3.777750533	4.954920970

	C32	9.447733711	-3.267859047	6.361022233
	C33	7.553612812	-3.786320496	4.740585988
	C34	6.921615748	-2.401609556	4.883179956
	C35	5.463557160	-2.395391630	4.424020534
	C36	3.807470713	-0.597626470	5.048827072
	C37	10.453701158	-1.435818033	7.636714406
	C38	9.631876021	-0.229237298	8.032316855
	C39	-1.548504465	-4.082662102	-4.448643080
	C40	-2.882428228	-3.473236230	-3.976932132
	C41	-0.400547965	-3.401086624	-3.670584002
	C42	0.979756470	-4.081788414	-3.775808770
	C43	1.848762443	-3.635911936	-4.951545300
	C44	-4.519069699	-1.715738488	-4.549789103
	C45	-5.828563253	-2.424868005	-4.180178538
	C46	-4.687371258	-0.706686769	-5.694215302
	C47	-7.209992313	-4.432578713	-4.429394876
	C48	-7.317355758	-4.848790992	-2.957125220
	C49	-6.188274434	-5.076749374	-0.790194439
	C50	-6.067134850	-3.865949898	0.134372694
	C51	-5.923705689	-1.437431452	0.381943462
	C52	-7.293352136	-1.066453753	0.999314093
	C53	-5.478844128	-0.207614617	-0.421334827
	C54	-4.119731704	-0.264034997	-1.036297591
	C55	-3.009145881	-1.047883518	-0.831620634
	C56	-2.507105171	0.433037050	-2.417925561
	C57	-9.708196656	-0.924186247	0.527440435
	C58	-10.147754699	-1.500690416	1.873152323
	C59	-10.638848336	-1.426167783	-0.602391910
	C60	-11.961689018	-0.731291414	-0.745094575
	C61	-12.851268661	-0.258755333	0.200148781
	C62	-13.621789427	0.122193479	-1.842457358
	C63	4.417351202	4.313370067	7.839461626
	C64	5.919173578	4.147342725	7.988275495
	C65	3.932648124	3.209999348	6.865232231
	C66	2.436527457	3.223971944	6.662175372
	C67	1.579812707	2.480971879	7.493893767
	C68	1.855007169	3.982055962	5.629005607
	C69	0.193483610	2.484161085	7.302024285
	C70	0.473419334	4.009254398	5.436287066
	C71	-0.355718117	3.250760747	6.266571058
	C72	-0.594282294	8.272155489	2.797314201
	C73	-2.004984745	8.617384331	2.292789126
	C74	-0.280687928	6.796237294	2.548008853
	C75	-3.340603760	9.965116723	0.718418115
	C76	-4.079379513	8.985491274	-0.196424193
	C77	-3.943845582	6.769845938	-1.321112599
	C78	-4.406291913	5.616811512	-0.392494332
	C79	-2.854513076	6.306169401	-2.318615302
	C80	-3.116738172	5.069610266	-3.188429389
	C81	-4.051471124	5.256886557	-4.395865326
	C82	-6.525553102	5.602319381	-4.354465432
	C83	-3.995803296	4.239117064	1.591602881
	C84	-5.172390464	4.626838488	2.496788898
	C85	-2.836627504	3.779947631	2.505942395
	C86	-5.966674371	6.352596376	4.035053667
	C87	-7.250669796	7.085784805	3.689448736
	C88	-8.688866422	7.905841947	1.912867800
	C89	-10.122834239	7.319577503	1.991032086
	C90	-8.442679824	8.516488937	0.531119518
	C91	-8.506438293	7.507619763	-0.606365338
	C92	-11.507556922	5.299998355	2.160732837
	C93	-12.453259059	5.634485587	3.306387752
	C94	-11.254912716	3.766005826	2.110254096
	C95	-10.436206079	3.353423548	0.907581021
	C96	-11.064275538	3.049376800	-0.311608675
	C97	-9.032586099	3.306802831	0.956627209
	C98	-10.320342982	2.728546657	-1.449166395
	C99	-8.270284598	3.018174600	-0.178820380
	C100	-8.926844585	2.737105966	-1.373197546
	C101	7.685621335	3.467067080	-6.315582450
	C102	6.422289242	2.676124095	-6.604626933
	C103	4.051138233	2.354052249	-5.940336163
	C104	2.915582146	3.296226557	-6.356435637
	C105	1.543769207	5.208454789	-5.665929770
	C106	1.730968708	6.586391884	-5.037260465
	C107	2.742133379	8.006695310	-3.335087491
	C108	3.487268796	9.068441813	-4.121165218
	C109	2.687026177	0.230319284	-9.734065847
	C110	1.155275473	0.299600733	-9.737473594
	C111	3.169081252	-0.607874772	-8.528029058

C112	4.641540608	-1.029744639	-8.695547713
C113	5.179308007	-1.596419342	-7.401346182
C114	-0.858867845	1.432650418	-8.942273578
C115	-1.244238362	2.898322633	-9.008790504
C116	-1.550597401	0.636192564	-7.808119116
C117	-0.888762644	0.880571619	-6.479681638
C118	-1.219718040	2.004171640	-5.704495621
C119	0.180035398	0.065052029	-6.069157058
C120	-0.457482736	2.335398172	-4.579712773
C121	0.942856911	0.397820528	-4.946974267
C122	0.639930339	1.549162147	-4.214811761
C123	10.822319521	1.883746202	-1.350223002
C124	11.480160756	1.994216653	0.035418680
C125	10.002489326	0.590239343	-1.474689181
C126	9.165824012	0.448953789	-2.708072710
C127	9.600773153	0.413278728	-4.017206925
C128	7.445667958	-0.087235115	-3.951979729
C129	12.745549387	0.968754995	1.834059961
C130	13.662677328	2.161003531	1.987452953
C131	5.102637212	-4.610053827	-1.179546409
C132	6.107313908	-4.006876597	-0.202169649
C133	7.131992588	-3.028620807	-0.846973575
C134	8.188919297	-3.824951961	-1.658721223
C135	9.402728104	-3.015980826	-2.124825551
C136	10.525449544	-3.869438933	-2.756883589
C137	7.867722575	-2.257045041	0.261712312
C138	3.454740968	2.296699601	-0.185207948
Fe139	4.770825162	2.131346651	-1.679045388
Fe140	1.622321991	2.211941207	-0.175333230
Fe141	3.258471537	3.335632965	1.451404381
Fe142	3.205040378	4.080950945	-1.053062656
Fe143	1.190930733	4.674480545	0.637540430
Fe144	5.039251593	1.580973160	0.831607026
Fe145	3.460238504	0.064329985	-0.566721925
H146	0.485088541	-9.006238671	-4.400017575
H147	-0.041875906	-7.835734754	-5.408238485
H148	-3.968843401	-9.220539222	-4.416009737
H149	-2.084045600	-9.034445463	-5.847215229
H150	-1.500989537	-10.326333716	-4.804457273
H151	-4.106898441	-7.023252829	-2.667513473
H152	-3.333079242	-10.377960497	-1.814459683
H153	-5.320436668	-8.317963519	-2.800127063
H154	-2.768278045	-11.299645480	0.235433080
H155	-1.144206850	-8.996693025	-0.893111700
H156	-3.926520805	-10.195063412	1.010967345
H157	-1.354164443	-4.108258425	0.866642886
H158	-1.792378377	-6.630501390	-0.474729647
H159	0.781990511	-7.898540573	-0.169726091
H160	-4.827626138	-5.390780837	3.790075321
H161	-3.650420988	-6.820223400	1.555847550
H162	0.551536187	-8.537848319	1.475871591
H163	-2.806687715	-4.623651429	-0.027903943
H164	-2.401961071	-8.092745383	3.683117614
H165	-5.002968903	-7.126716646	3.416948012
H166	1.194097240	-3.859713345	1.784695832
H167	0.568331204	-2.587365506	0.721765650
H168	1.949031199	-2.244456516	1.779871430
H169	-0.921260628	-2.859556257	2.715594850
H170	-1.437339762	-9.171246429	5.490961568
H171	-3.408244640	-1.371418862	3.911091139
H172	-2.658994926	-4.627209629	6.913148311
H173	-4.879934119	-2.310290162	7.911392776
H174	0.614177972	-0.224380185	2.447677653
H175	-0.710750466	-0.388568268	3.613123145
H176	3.963237729	4.117541571	8.830209017
H177	-5.089149150	-1.066571626	4.399374983
H178	-5.918278606	-1.608996773	6.649934041
H179	-3.784120928	-0.036863710	5.035857167
H180	-4.681181806	-0.605210439	7.443569580
H181	-4.149136075	-3.036094867	5.631861076
H182	-2.148049527	-0.985050752	6.726562689
H183	-1.905244739	-3.120459089	4.728998781
H184	-1.023893722	-0.647585282	1.900126675
H185	-1.902383332	3.762158814	1.922260394
H186	-3.070916748	2.754397117	2.826225621
H187	-2.402063417	4.087439548	4.398095579
H188	-2.188274477	2.740345240	6.663083933
H189	0.037248888	4.591670682	4.624521911
H190	2.495293867	4.544947068	4.946516514
H191	1.998092356	1.876469034	8.303144805

	H192	0.030725087	-4.705490406	3.877573145
	H193	2.290819949	-6.659941652	4.340996905
	H194	2.022429664	-6.574914720	6.095428390
	H195	-0.060030619	-6.979963552	4.018333484
	H196	-2.295984241	-8.088295745	6.610990050
	H197	-0.453298265	1.879528759	7.942475717
	H198	1.598958544	-2.245530490	4.316902601
	H199	-3.415706781	-4.783360863	9.530543385
	H200	-2.311167901	-5.954781039	8.755333606
	H201	-0.385123031	-4.186998210	9.487780440
	H202	11.518044952	-1.156712820	7.604191808
	H203	3.805003540	-3.360953485	-5.492781050
	H204	5.372049157	-2.927373351	-2.085494600
	H205	4.708523295	-1.810214406	-9.466643813
	H206	5.250568178	-0.172423347	-9.016744145
	H207	2.549288127	-1.511287008	-8.420879070
	H208	-0.509583114	3.539063194	-9.549937047
	H209	2.922415423	-0.342730394	-10.643245046
	H210	-0.323492073	-2.352849541	-4.004236921
	H211	0.856449189	-5.175351041	-3.857785629
	H212	1.172097706	1.931207020	-8.450829293
	H213	-1.226324868	1.011677991	-9.900740657
	H214	3.247414486	2.102756000	-9.026550491
	H215	3.585047492	-3.818983638	-3.808038588
	H216	3.031295270	-0.021916971	-7.606042344
	H217	4.273636527	1.465880996	-10.147397046
	H218	-1.700330501	-4.761330530	-6.392389480
	H219	-0.689827545	-3.395643270	-2.608886163
	H220	5.999456213	0.153829829	-7.040015555
	H221	1.531239483	-3.916432205	-2.837950950
	H222	-5.248236797	-1.621604185	1.229295450
	H223	-2.838740482	-1.871998042	-0.150132989
	H224	-7.143676582	-5.564905211	-0.551734218
	H225	-5.296801615	-4.475853177	-2.634196811
	H226	-8.212182460	-1.940305436	-0.615021497
	H227	-12.826971616	-0.243738477	1.284360853
	H228	-9.737252771	-2.518760241	2.081020280
	H229	-10.776451308	-2.517341918	-0.497688053
	H230	-9.737858620	0.174780654	0.586788757
	H231	-14.293606360	0.461755746	-2.626080271
	H232	-5.525222759	0.660272115	0.257259759
	H233	-6.210946860	-0.025211474	-1.224109770
	H234	-14.724055776	0.721775093	-0.121765833
	H235	-10.108253718	-1.275663115	-1.556099247
	H236	-6.066475552	-2.533101131	-1.465989662
	H237	-5.389642293	-5.785599200	-0.523627332
	H238	-8.139741685	-3.899213082	-4.668415083
	H239	-1.948385740	0.993679405	-3.164516924
	H240	-7.180633262	-5.341949526	-5.043768838
	H241	4.272752150	6.347751491	8.033498703
	H242	3.150838906	5.704782911	7.033906656
	H243	-2.645349960	5.690155564	0.737690522
	H244	-4.340449384	3.377892422	1.002089781
	H245	-4.357801916	6.501285389	2.676516850
	H246	-4.450794737	1.327814437	-2.462077093
	H247	-5.721625030	1.486569039	-4.088119119
	H248	-5.972475847	0.697329082	-6.167484344
	H249	-6.552790881	2.106457325	-2.880021950
	H250	-3.713464232	-0.218634001	-5.865612749
	H251	-4.988316920	-1.228229514	-6.614996156
	H252	-7.491223546	9.065979450	0.535692181
	H253	-9.251791502	9.243678550	0.361318041
	H254	-6.642365520	8.129227812	-1.264515966
	H255	-7.418552838	6.817571523	-2.156843737
	H256	-7.281605673	7.384889535	-5.040105852
	H257	-3.698811931	4.645896572	-5.240843909
	H258	-10.824373793	2.475606060	-2.385505677
	H259	-8.502745118	3.484742417	1.895687183
	H260	-7.798348616	4.320588636	-3.354129870
	H261	-1.942833232	6.105984150	-1.732908736
	H262	-2.429584144	7.787514876	-0.216626467
	H263	-3.465242508	4.223079605	-2.575920274
	H264	-5.336867412	-3.997703572	-5.386710215
	H265	-3.170149319	10.893094525	0.156350774
	H266	-2.871973374	-2.568537870	-5.750917746
	H267	-2.619143951	7.164173412	-2.969152933
	H268	-7.181000952	3.051675787	-0.132931679
	H269	-4.031553400	10.194372046	1.543141347
	H270	-5.630913472	7.082463950	-5.484362000
	H271	-8.570389587	5.546750133	-4.304490762

	H272	-0.410331394	-3.730789514	-6.128265223
	H273	-1.577265015	-5.143050513	-4.133777553
	H274	-12.154965679	3.052924019	-0.372712059
	H275	-4.259039664	-1.162926339	-3.637715242
	H276	-4.839058871	7.118082333	-1.846343891
	H277	-4.015643523	6.303072720	-4.725305614
	H278	-2.139842787	4.770225514	-3.593028916
	H279	-5.571945303	3.904707523	-3.772912787
	H280	-6.236801742	5.476434810	4.643583550
	H281	-6.966507673	6.630021153	1.715439422
	H282	-8.725460803	8.743399502	2.627699488
	H283	-5.366038897	7.029318486	4.658262549
	H284	-9.376740528	5.459965297	2.314323327
	H285	7.424235384	4.485196414	-5.992017524
	H286	8.158191945	2.967015070	-5.443547722
	H287	10.143164642	2.747124653	-1.411833119
	H288	9.469738137	3.839227262	-7.224625510
	H289	8.645971973	2.583978241	-7.877036559
	H290	0.733686271	6.602023032	2.922659657
	H291	-1.225069315	8.063721285	4.727175753
	H292	-1.210898729	9.888651929	0.881275553
	H293	-1.000909288	6.171882691	3.095288451
	H294	-0.589277788	9.538815688	4.416729866
	H295	3.296411862	7.836742510	-2.398022097
	H296	0.145693947	8.892580347	2.267522589
	H297	7.097703521	-4.492174359	5.454934421
	H298	9.545943477	-3.142405776	4.192738779
	H299	3.178238418	5.947879548	-3.677688090
	H300	2.631366733	1.051726735	5.343006584
	H301	1.465451905	5.353097909	-6.750410486
	H302	1.783939402	-0.232050005	-4.646169936
	H303	0.560405866	4.840710265	-5.324877712
	H304	4.849866962	-3.047626654	5.055291838
	H305	2.987169349	4.097502895	-4.483515615
	H306	5.389196093	-2.789412599	3.396497769
	H307	7.375417710	-4.178690225	3.726584768
	H308	7.477385133	-1.672787428	4.274511717
	H309	6.966682291	-2.059427603	5.929353543
	H310	-1.503988482	-0.429761179	-8.074301904
	H311	9.223505266	-5.896719903	5.329552357
	H312	10.673878965	-7.343054301	3.047609674
	H313	1.246807520	1.844747596	-3.359293822
	H314	10.890049553	-7.709853550	5.934235099
	H315	10.170164755	-8.660894357	4.810784794
	H316	5.377835226	-0.373934037	3.802026171
	H317	-0.695780981	3.219479192	-3.984639081
	H318	0.429090358	-0.831928338	-6.640292466
	H319	12.385840212	-2.370454984	0.429868032
	H320	10.689118079	-0.273132460	-1.443711017
	H321	10.137262376	-2.364812930	1.602401202
	H322	12.477951385	-2.725241707	-1.084384020
	H323	4.086783368	8.690664116	-4.984091696
	H324	7.932186998	-0.191963760	1.912169420
	H325	6.619493353	-4.817088870	0.329580892
	H326	5.445531962	3.738547547	-5.152119796
	H327	8.532743664	-4.643977639	-1.008030883
	H328	8.496013871	-0.092466865	-5.781702107
	H329	6.464828833	-0.385779747	-4.296546772
	H330	12.095742944	-7.019708991	4.047588813
	H331	3.829848891	1.242269428	4.081668248
	H332	9.544977027	-0.154900423	3.665469311
	H333	7.670547092	1.143482815	2.666678481
	H334	10.169099895	-1.508581110	5.504521327
	H335	3.294446384	-2.351822385	5.970766427
	H336	9.082555680	-2.282029178	-2.879641558
	H337	9.832205560	-2.450066760	-1.287328564
	H338	7.692678241	-4.288442604	-2.525205601
	H339	3.844133059	1.908092361	-4.953942425
	H340	-2.051971598	2.640600658	-6.016605843
	H341	-2.608056426	0.942486627	-7.800057392
	H342	5.503152091	-3.455482673	0.538995724
	H343	10.724708259	-1.093302510	3.289472100
	H344	11.199074037	-3.092453308	2.456185343
	H345	2.175133737	-1.097535184	6.154508263
	H346	10.585523908	0.568021621	-4.443073652
	H347	9.353818390	0.490613000	-0.596300237
	H348	4.121385602	1.559347715	-6.685221566
	H349	1.750728312	8.398797008	-3.065170588
	H350	4.250127820	2.240800435	7.277888005
	H351	4.442864315	3.349633183	5.898891159

	H352	12.287289594	2.920545536	-2.342114037
	H353	12.530991826	1.302127723	-2.347535512
	H354	13.360328927	0.064796860	1.963703426
	H355	12.183598333	0.016556049	0.010069097
	H356	14.133294248	2.517879179	1.040762012
	H357	11.992924798	0.975181549	2.638977198
	H358	6.517084283	4.626455180	7.175624927
	H359	-11.973247160	6.081211407	4.208936476
	H360	10.324236364	-2.188280694	8.433795528
	H361	8.597400299	-0.203790318	7.610983381
	H362	-12.001328470	5.612291441	1.225325882
	H363	-10.754076052	3.456661895	3.041023239
	H364	-12.238544804	3.275799794	2.090867723
	H365	3.300232378	-1.399427628	-0.810372191
	H366	-8.721276138	2.080922650	-3.198871022
	H367	0.699375797	-0.502262018	-0.033807096
	H368	2.729082023	-0.411810845	0.642534776
	H369	-1.053950155	-0.890178055	-1.738313092
	Mo370	6.143104553	-0.108857182	-1.002172940
	N371	-0.090652412	-8.841754031	-5.229168419
	N372	-3.475793924	-8.792067617	-3.636674727
	N373	-3.453036282	-9.776476068	-1.002714645
	N374	-1.003346311	-8.937449644	0.116312046
	N375	-1.450221403	-6.143823368	0.350490304
	N376	-3.537032131	-6.145337099	2.314898040
	N377	-2.462141545	-7.605323907	4.581793871
	N378	0.302664324	-6.869107054	4.967589096
	N379	0.715058254	-4.121941598	4.358626224
	N380	-1.529238890	-2.509173657	5.457368481
	N381	-2.397821017	-4.133776060	7.770593899
	N382	10.312784747	-7.689076909	5.090483673
	N383	9.610105942	-5.119922727	4.783008357
	N384	4.909931267	-1.037195812	4.422499775
	N385	3.164835375	-1.334871887	5.978971472
	N386	3.330040388	0.620970983	4.742920568
	N387	10.022351083	-2.039472162	6.391381558
	N388	-1.404123852	-3.916716686	-5.899137109
	N389	3.175377268	-3.626775692	-4.728129493
	N390	-3.433461505	-2.622162227	-4.882981685
	N391	-6.052201296	-3.622114403	-4.767587516
	N392	-6.174099949	-4.802819827	-2.215276302
	N393	-5.975360154	-2.631736634	-0.450258860
	N394	-3.772861004	0.639724781	-2.030208942
	N395	-2.037168363	-0.598373416	-1.703462097
	N396	-8.360584858	-1.381323706	0.221390239
	N397	-12.455786414	-0.486930891	-2.017050125
	N398	-13.897837935	0.281410241	-0.517460523
	N399	4.137841570	5.646987390	7.300533425
	N400	-0.459817971	8.538943460	4.238240742
	N401	-2.073713911	9.497698265	1.250603011
	N402	-3.386150995	7.890083187	-0.571352660
	N403	-5.437829751	4.872380370	-4.115175134
	N404	-6.475796527	6.768093195	-5.021827314
	N405	-7.721241548	5.205876900	-3.860986498
	N406	-3.606413385	5.307047124	0.667579306
	N407	-5.179037565	5.929426378	2.894545997
	N408	-7.630382048	7.034006484	2.375169373
	N409	-7.487038850	7.594847942	-1.503015959
	N410	-10.236682226	6.002833381	2.298189229
	N411	8.536088298	3.531787242	-7.505612334
	N412	5.332388748	3.045968720	-5.898039118
	N413	2.571911309	4.211796228	-5.423768545
	N414	2.632606113	6.737390189	-4.034009125
	N415	3.287339568	1.559011585	-9.896377649
	N416	0.584887550	1.294319660	-8.998414710
	N417	11.806613374	2.021253747	-2.439515450
	N418	7.803568670	0.142765911	-2.689599711
	N419	8.503691035	0.076190958	-4.779213747
	N420	12.141674120	0.906093232	0.513425296
	N421	-2.325482982	1.590412050	0.637942680
	N422	-1.540205698	1.926447444	-0.074067016
	O423	-1.500304285	-7.959714442	-2.871503098
	O424	-4.104562923	-7.728686680	-0.243529568
	O425	-1.871967511	-9.318938939	2.192355476
	O426	-0.052138270	-6.178002831	2.144639269
	O427	-2.771397042	-4.093189998	2.942778619
	O428	-3.564176037	-6.055654169	5.851671914
	O429	0.041156588	-7.371375912	7.192176099
	O430	2.641622226	-4.164729893	5.614937935
	O431	0.260562191	-1.289154245	6.186422259

	O432 -2.165466107 -2.172266140 8.890716596<br>O433 -1.427548839 -4.660458470 11.177958200<br>O434 11.325338252 -4.677130587 3.297012747<br>O435 9.224794901 -3.959785892 7.369700928<br>O436 10.042454247 0.653729240 8.769070655<br>O437 -3.392308191 -3.720568022 -2.866165129<br>O438 1.356849318 -3.324225219 -6.060731761<br>O439 -6.606984218 -1.913956643 -3.355552041<br>O440 -5.687220168 0.269488348 -5.340388587<br>O441 -8.412034595 -5.217965828 -2.513249463<br>O442 -6.048584441 -4.023951388 1.360029316<br>O443 -7.382174703 -0.447119729 2.062968697<br>O444 -10.909488267 -0.945418276 2.645464462<br>O445 6.454883829 3.533658245 8.898972604<br>O446 -1.713290239 3.284704535 6.008527868<br>O447 -3.029474100 8.138770482 2.809761126<br>O448 -5.246219898 9.236811480 -0.554453229<br>O449 -5.466728899 5.017530125 -0.634165391<br>O450 -5.997965325 3.784068750 2.869952158<br>O451 -2.690409422 4.636480681 3.639746309<br>O452 -7.903842676 7.674326211 4.559222650<br>O453 -11.105375428 8.056279116 1.824171964<br>O454 -9.440988421 6.702017853 -0.725117716<br>O455 -13.652746057 5.411924855 3.259335292<br>O456 -8.146353371 2.493210763 -2.525039022<br>O457 6.432072569 1.722494615 -7.420466306<br>O458 2.380074882 3.225620910 -7.481851733<br>O459 1.015567424 7.521506215 -5.432443464<br>O460 3.467303866 10.250893488 -3.823191925<br>O461 0.471927187 -0.529343595 -10.361491540<br>O462 4.959104610 -2.742750020 -7.024266506<br>O463 5.889533575 -0.755540466 -6.623035847<br>O464 -2.281988290 3.365040333 -8.561966760<br>O465 11.459939153 3.061314260 0.680263308<br>O466 13.946621090 2.660272754 3.068020783<br>O467 4.740031078 -3.806906295 -2.186830089<br>O468 4.616086811 -5.728928356 -1.027555489<br>O469 11.728883350 -3.593298155 -2.413196388<br>O470 10.185224233 -4.757851573 -3.588107918<br>O471 7.773293474 -0.953937549 0.223014939<br>O472 8.533943864 -2.871435819 1.116472289<br>O473 6.404534188 -2.111461777 -1.668487158<br>O474 10.395711542 -0.508717277 4.026758853<br>O475 7.919416683 0.212428187 2.817355027<br>O476 -5.668240365 2.126813191 -3.326043901<br>O477 12.989887594 -2.285092118 -0.341935968<br>O478 11.054020687 -2.242156567 1.969283555<br>S479 -0.368619529 6.336333574 0.755692950<br>S480 0.977889960 3.867753375 -1.488841891<br>S481 4.399663586 0.142375278 -2.625496792<br>S482 1.123483593 2.822407785 1.921538025<br>S483 1.208981842 0.186719695 -1.089430751<br>S484 4.589522138 4.060091821 -2.783910107<br>S485 4.886497223 -0.639414042 0.924782810<br>S486 3.333986907 5.443192885 0.762489505<br>S487 6.707936282 2.157333258 -0.526538242<br>S488 4.924407650 2.663633443 2.743468169<br>end

TS

Fe(139) -2.291<br>Fe(140) -1.029<br>Fe(141) -2.486<br>Fe(142) 2.754<br>Fe(143) 2.806<br>Fe(144) 2.306<br>Fe(145) 0.760	bm522bh6x6hn2n2xt3g_orig<br>C1 -2.136921062 -8.611733602 -3.743239496<br>C2 -1.468665179 -9.248655152 -4.966482877<br>C3 -4.259413528 -8.116683801 -2.618323993<br>C4 -3.931340347 -8.526573803 -1.186195399<br>C5 -3.075329852 -10.247900911 0.319402977<br>C6 -1.934259395 -9.452028383 0.959830449<br>C7 0.043126025 -8.063069821 0.627819633<br>C8 -0.488025904 -6.713592967 1.112879131<br>C9 -2.099602608 -4.906743777 0.762105707<br>C10 -2.831752977 -5.010518863 2.107391366<br>C11 -4.281001351 -6.309472324 3.554440413<br>C12 -3.395105107 -6.634371988 4.765636237<br>C13 -1.689069086 -8.121763027 5.699639205<br>C14 -0.373879169 -7.402274976 6.026550412<br>C15 1.620667184 -6.283461727 5.124562619<br>C16 1.687679395 -4.756548667 5.067501632<br>C17 0.582759724 -2.689386672 4.220958726<br>C18 -0.247987093 -2.102343882 5.385385333<br>C19 0.023389726 -2.322247999 2.827446800

C20	-0.287092613	-0.822091408	2.702542867
C21	0.988669011	-2.783412692	1.731376077
C22	-2.456819234	-2.022857098	6.493792622
C23	-2.327803194	-2.787616335	7.828547826
C24	-3.912286682	-2.010566604	5.961828234
C25	-4.903284737	-1.614862351	7.062608853
C26	-4.047219198	-1.070704366	4.756827118
C27	-2.466185397	-4.900794504	9.029716556
C28	-1.345699318	-4.528231251	9.977306799
C29	10.999291571	-6.950867112	4.027124362
C30	10.661118089	-5.460494543	4.009854943
C31	9.076642062	-3.779772051	4.954221456
C32	9.447033013	-3.268005738	6.360659224
C33	7.554006820	-3.787889162	4.739405722
C34	6.920988058	-2.402941047	4.881440827
C35	5.462875097	-2.396457433	4.421564480
C36	3.807693240	-0.597106569	5.044534111
C37	10.453946900	-1.436104219	7.637168451
C38	9.632541984	-0.229829013	8.033051861
C39	-1.558055150	-4.099413714	-4.458019031
C40	-2.888555517	-3.484210905	-3.981560606
C41	-0.411469243	-3.414141591	-3.678144304
C42	0.972700659	-4.087575167	-3.778611679
C43	1.843431148	-3.638101513	-4.951998778
C44	-4.518957341	-1.715529081	-4.547875381
C45	-5.829047184	-2.425376523	-4.178949715
C46	-4.687671950	-0.706919390	-5.694128995
C47	-7.209102323	-4.433331418	-4.429732162
C48	-7.317129584	-4.849219474	-2.957922475
C49	-6.189511246	-5.075087067	-0.789860488
C50	-6.070234739	-3.863554809	0.134699444
C51	-5.936175611	-1.433949349	0.383506423
C52	-7.308108494	-1.065932045	1.000846484
C53	-5.487912359	-0.202436696	-0.415437172
C54	-4.123980313	-0.252733771	-1.020579139
C55	-3.015421494	-1.035488082	-0.814283635
C56	-2.508640046	0.450379376	-2.396793124
C57	-9.730197248	-0.936878878	0.530084074
C58	-10.163993416	-1.511540292	1.877237969
C59	-10.652142464	-1.436424503	-0.605276670
C60	-11.971613276	-0.738451036	-0.748611734
C61	-12.857719423	-0.262519571	0.197708104
C62	-13.628150387	0.123770305	-1.844557117
C63	4.416638002	4.312595964	7.840656360
C64	5.918613916	4.146971412	7.988398984
C65	3.930693932	3.208866019	6.866529159
C66	2.434736013	3.222931628	6.664810553
C67	1.577761545	2.480715676	7.496834092
C68	1.853797409	3.983764384	5.633581930
C69	0.191942130	2.484621279	7.304768489
C70	0.471621917	4.013148503	5.442248673
C71	-0.357344040	3.253551051	6.271056845
C72	-0.590599292	8.271246684	2.797562562
C73	-2.002468913	8.615704178	2.292448253
C74	-0.279142191	6.798838799	2.545894862
C75	-3.340316483	9.965448944	0.719450695
C76	-4.081266817	8.986421182	-0.193960652
C77	-3.948882066	6.771000847	-1.321187596
C78	-4.411700241	5.616912495	-0.394349308
C79	-2.860178483	6.306763884	-2.319548371
C80	-3.122965223	5.070625826	-3.188377560
C81	-4.057498449	5.258210111	-4.396852127
C82	-6.533141638	5.603899740	-4.357042830
C83	-4.001304645	4.227034167	1.578978615
C84	-5.172473128	4.618671446	2.491372381
C85	-2.842095130	3.751601250	2.481190375
C86	-5.966551665	6.350535410	4.027062739
C87	-7.253581610	7.084284012	3.685765658
C88	-8.699895774	7.906358972	1.914427138
C89	-10.134181822	7.321784577	1.990255160
C90	-8.449029549	8.517602236	0.532261530
C91	-8.510056499	7.507314139	-0.604293709
C92	-11.523225828	5.303937000	2.163151384
C93	-12.464772504	5.634547922	3.310725013
C94	-11.262904012	3.770051639	2.111677637
C95	-10.442918074	3.356516555	0.908658067
C96	-11.071842489	3.050868181	-0.308976488
C97	-9.038554895	3.310379660	0.957188774
C98	-10.329107109	2.729197812	-1.446248667
C99	-8.277260862	3.020470360	-0.178716711

C100	-8.936074899	2.738642108	-1.371910962
C101	7.677423404	3.462866554	-6.295872321
C102	6.417357154	2.673039098	-6.594203603
C103	4.044170336	2.351076679	-5.938783892
C104	2.909719731	3.293803144	-6.357728034
C105	1.541094563	5.207759360	-5.666209470
C106	1.731628048	6.582938507	-5.033077886
C107	2.744410873	7.990721747	-3.322641145
C108	3.487224369	9.055639360	-4.110363110
C109	2.686057717	0.230316819	-9.731473593
C110	1.154409814	0.299761083	-9.737413866
C111	3.168462708	-0.608091611	-8.526363584
C112	4.639837261	-1.030310380	-8.694349386
C113	5.178041642	-1.596359074	-7.400322708
C114	-0.861144220	1.431767221	-8.944216110
C115	-1.247432100	2.897431395	-9.009557312
C116	-1.554366380	0.633908586	-7.810465424
C117	-0.892127768	0.881278549	-6.482867466
C118	-1.223548561	2.005648215	-5.709778765
C119	0.178332706	0.067255363	-6.071865613
C120	-0.457486278	2.343752553	-4.589855229
C121	0.945621633	0.406358988	-4.955202287
C122	0.645103101	1.563748008	-4.229777944
C123	10.820917136	1.886544358	-1.352681074
C124	11.476696653	1.998162811	0.032493960
C125	10.005045165	0.590533070	-1.475121622
C126	9.167315881	0.447237022	-2.706680066
C127	9.600060452	0.413447107	-4.016078310
C128	7.445418874	-0.089816874	-3.947427845
C129	12.730618091	0.971023349	1.835723069
C130	13.653641063	2.160196189	1.989203538
C131	5.102048469	-4.606492603	-1.179866263
C132	6.107599908	-4.003952462	-0.201806141
C133	7.130179673	-3.023638927	-0.846819505
C134	8.186749099	-3.821043220	-1.660080307
C135	9.401900945	-3.016035304	-2.126824093
C136	10.525321489	-3.870633834	-2.758575597
C137	7.866658568	-2.254589496	0.261077621
C138	3.435044057	2.275884854	-0.176648907
Fe139	4.743440707	2.151837197	-1.650738729
Fe140	1.251260912	2.191553263	-0.083559217
Fe141	3.373638052	3.423859779	1.443057637
Fe142	3.125943490	4.005965344	-1.060520401
Fe143	1.253547041	4.759921668	0.664424767
Fe144	5.053550433	1.652316113	0.831070557
Fe145	3.458093281	0.089064310	-0.551282404
H146	0.477322309	-9.010218709	-4.401127243
H147	-0.053548585	-7.840036654	-5.407329404
H148	-3.982953861	-9.227295882	-4.420158094
H149	-2.088774234	-9.044290838	-5.854045546
H150	-1.502684377	-10.340894043	-4.817206903
H151	-4.102573949	-7.029014966	-2.673750671
H152	-3.333692451	-10.380300636	-1.812035654
H153	-5.324448047	-8.317102213	-2.797791151
H154	-2.767842024	-11.299047387	0.237789277
H155	-1.144978359	-8.994433466	-0.893528853
H156	-3.926074132	-10.193604071	1.012149975
H157	-1.357608573	-4.106652995	0.866067493
H158	-1.794902539	-6.631071421	-0.472139974
H159	0.782056069	-7.896195236	-0.168111800
H160	-4.827165382	-5.388426325	3.790682042
H161	-3.652418455	-6.819280969	1.556311540
H162	0.549507279	-8.536065383	1.478105914
H163	-2.808617756	-4.623366257	-0.027820708
H164	-2.402617229	-8.090365593	3.682378850
H165	-5.005104033	-7.124657893	3.418086937
H166	1.195390397	-3.860742275	1.786608815
H167	0.567478180	-2.582876478	0.733369862
H168	1.945162330	-2.244085673	1.797800692
H169	-0.927088820	-2.870538746	2.717439625
H170	-1.441662644	-9.172685881	5.490369687
H171	-3.406836547	-1.373180495	3.917522377
H172	-2.671195383	-4.637503771	6.927459605
H173	-4.885940188	-2.310865740	7.913857525
H174	0.607826064	-0.239019148	2.438930816
H175	-0.699624056	-0.389254364	3.626254926
H176	3.963157736	4.117373105	8.830750112
H177	-5.088005514	-1.067199930	4.400907972
H178	-5.923021426	-1.607069873	6.652147595
H179	-3.785131015	-0.038518177	5.040979327

H180	-4.684092141	-0.605621633	7.445901367
H181	-4.155842655	-3.037307518	5.634642373
H182	-2.151385109	-0.994967210	6.736126641
H183	-1.913102842	-3.129327884	4.738957660
H184	-1.040875339	-0.655840416	1.917143045
H185	-1.914516925	3.721792537	1.887171934
H186	-3.083254595	2.727188628	2.802974225
H187	-2.392415307	4.067357750	4.375702458
H188	-2.190279849	2.738708250	6.660449100
H189	0.035107916	4.596526699	4.631376228
H190	2.493793546	4.545676261	4.949364204
H191	1.995905257	1.875207771	8.304807767
H192	0.024825209	-4.723638177	3.883716639
H193	2.294393808	-6.668233343	4.343346734
H194	2.024744835	-6.585054781	6.097813130
H195	-0.057793073	-6.986235165	4.018633107
H196	-2.298546530	-8.088206703	6.611479003
H197	-0.455510030	1.879482005	7.944024823
H198	1.587637446	-2.265421663	4.330708638
H199	-3.431370245	-4.791156775	9.549635428
H200	-2.328191998	-5.963012176	8.772452650
H201	-0.399461432	-4.195802637	9.491400759
H202	11.518365550	-1.157156067	7.602572167
H203	3.799934132	-3.360734440	-5.491907480
H204	5.372478176	-2.920484467	-2.083031913
H205	4.706444206	-1.810768429	-9.465449510
H206	5.249111817	-0.173454633	-9.017021978
H207	2.547827676	-1.510925660	-8.419808769
H208	-0.512575665	3.537872348	-9.550594303
H209	2.921453982	-0.342077685	-10.641677045
H210	-0.338996163	-2.365729904	-4.012569419
H211	0.856633923	-5.182530409	-3.860686515
H212	1.168725424	1.930827493	-8.450944984
H213	-1.228509156	1.010938464	-9.903312686
H214	3.245855253	2.103961503	-9.025111470
H215	3.581254157	-3.818369205	-3.808124075
H216	3.030307694	-0.021922944	-7.604617322
H217	4.272260365	1.466156465	-10.146248485
H218	-1.705550536	-4.769897287	-6.404240025
H219	-0.701817028	-3.408160701	-2.616377775
H220	5.998726766	0.154013285	-7.039688939
H221	1.521261029	-3.919390124	-2.840051611
H222	-5.262380843	-1.618675214	1.231883407
H223	-2.846509947	-1.866789100	-0.141789823
H224	-7.144897769	-5.563143164	-0.551589893
H225	-5.297237610	-4.474809456	-2.633270493
H226	-8.225267818	-1.941722789	-0.612546277
H227	-12.831309251	-0.246117695	1.281850427
H228	-9.747248306	-2.526598181	2.087187377
H229	-10.789761094	-2.527330143	-0.506054757
H230	-9.761326549	0.163064160	0.589306794
H231	-14.299578368	0.466220356	-2.628483294
H232	-5.536756886	0.664073764	0.263911484
H233	-6.215623066	-0.018811587	-1.222847439
H234	-14.727980899	0.726428896	-0.122195127
H235	-10.116332431	-1.281031451	-1.554956508
H236	-6.070555085	-2.530497038	-1.464858619
H237	-5.390687060	-5.782877680	-0.522400212
H238	-8.139128943	-3.901070925	-4.669210294
H239	-1.952083131	1.008568856	-3.146983788
H240	-7.178496894	-5.342523491	-5.043895880
H241	4.272083668	6.347492494	8.033565120
H242	3.149633192	5.703596066	7.034474947
H243	-2.649522601	5.679671462	0.730624652
H244	-4.343565227	3.368744918	0.983205501
H245	-4.359136159	6.495390301	2.667885689
H246	-4.451054123	1.337497541	-2.447719875
H247	-5.730466662	1.482180340	-4.092423882
H248	-5.972312723	0.694224555	-6.169585697
H249	-6.566565492	2.104134363	-2.884819453
H250	-3.714367700	-0.217260931	-5.863106770
H251	-4.984536602	-1.232223718	-6.614812274
H252	-7.496739488	9.066098306	0.539465040
H253	-9.256976392	9.245142220	0.359297275
H254	-6.644544733	8.128736186	-1.259827436
H255	-7.418543224	6.815226693	-2.150796874
H256	-7.284766597	7.389471955	-5.037587309
H257	-3.704350360	4.646423556	-5.240726725
H258	-10.832669079	2.475057163	-2.381735255
H259	-8.507702334	3.488529690	1.896153990

H260	-7.804871792	4.321079677	-3.357365156
H261	-1.948131408	6.104380606	-1.733410508
H262	-2.432728528	7.787325508	-0.217293461
H263	-3.470635070	4.224444225	-2.574710346
H264	-5.336322908	-3.997013962	-5.385863231
H265	-3.168689625	10.891698995	0.155364072
H266	-2.874622825	-2.570537088	-5.751967717
H267	-2.623500032	7.165913559	-2.969577292
H268	-7.187591384	3.052998150	-0.132992188
H269	-4.030723450	10.196383146	1.543828546
H270	-5.634404995	7.086765473	-5.479347991
H271	-8.576981468	5.547187229	-4.306417612
H272	-0.417383346	-3.738823305	-6.133404621
H273	-1.587900256	-5.158361341	-4.142289352
H274	-12.161777707	3.054219546	-0.369296899
H275	-4.257118224	-1.163088602	-3.635699783
H276	-4.843608846	7.120906546	-1.846245207
H277	-4.020831262	6.303457860	-4.726443672
H278	-2.146544745	4.770918854	-3.592864015
H279	-5.579764382	3.906552130	-3.775445339
H280	-6.236115495	5.476025194	4.638387237
H281	-6.977446169	6.630008013	1.710759633
H282	-8.735008516	8.743332926	2.629328607
H283	-5.366694664	7.028550303	4.648983976
H284	-9.392366094	5.461909795	2.313806635
H285	7.414619933	4.478912205	-5.969392974
H286	8.150288199	2.959110229	-5.427420003
H287	10.139960804	2.747760295	-1.415953290
H288	9.461806980	3.842174661	-7.208924411
H289	8.636968779	2.589163159	-7.865293044
H290	0.732551928	6.602042733	2.922129952
H291	-1.225025061	8.061721358	4.726262289
H292	-1.210910444	9.888647573	0.880627419
H293	-1.000420117	6.170983052	3.087541103
H294	-0.589565433	9.536886392	4.417834400
H295	3.298862662	7.818101466	-2.387614907
H296	0.148797433	8.891492932	2.268234642
H297	7.097696355	-4.492930727	5.453550738
H298	9.545855081	-3.144327710	4.192038049
H299	3.176387429	5.934256919	-3.673373120
H300	2.637890402	1.056945530	5.339886161
H301	1.463117873	5.354576533	-6.750165246
H302	1.785537783	-0.224429103	-4.653140581
H303	0.557914151	4.840143983	-5.325103429
H304	4.848704507	-3.049254056	5.051834936
H305	2.982793995	4.092459444	-4.484328668
H306	5.389396354	-2.789953124	3.393825076
H307	7.375411875	-4.179714364	3.725457475
H308	7.476988905	-1.673336666	4.272915962
H309	6.965168936	-2.060836195	5.927473343
H310	-1.506152535	-0.431624151	-8.077040589
H311	9.223458052	-5.897604654	5.330632378
H312	10.678201595	-7.346461563	3.051901156
H313	1.256069897	1.865950482	-3.379388910
H314	10.889295055	-7.712970561	5.938229386
H315	10.169806384	-8.662735662	4.813577087
H316	5.379053336	-0.375843418	3.799657715
H317	-0.697181806	3.226595229	-3.994619366
H318	0.425017820	-0.832980211	-6.638821929
H319	12.380064756	-2.367600697	0.428362935
H320	10.694647412	-0.270551284	-1.444392696
H321	10.134475692	-2.364775182	1.603625182
H322	12.473516848	-2.723384428	-1.085824223
H323	4.085179389	8.680012475	-4.975138709
H324	7.934417142	-0.187604323	1.909724262
H325	6.618945356	-4.815932198	0.328140762
H326	5.437041965	3.733969828	-5.146471270
H327	8.530741276	-4.641439995	-1.010460093
H328	8.492552737	-0.090401672	-5.779073391
H329	6.465983740	-0.391181402	-4.291945648
H330	12.100226683	-7.022925633	4.052570228
H331	3.841181869	1.246654269	4.087833551
H332	9.541559848	-0.149762968	3.664924575
H333	7.640301359	1.139958341	2.664117843
H334	10.167179451	-1.508012788	5.504057532
H335	3.293873419	-2.350217911	5.968680526
H336	9.083252200	-2.281503709	-2.882471701
H337	9.832579317	-2.449415767	-1.289725081
H338	7.691734513	-4.287110545	-2.526392067
H339	3.834351226	1.906340770	-4.952934379

H340	-2.058876070	2.638315724	-6.019286438
H341	-2.611906089	0.940451784	-7.803187274
H342	5.504294721	-3.453489073	0.540281291
H343	10.719655121	-1.091252025	3.288987013
H344	11.197516227	-3.092020095	2.456658831
H345	2.173247489	-1.096425016	6.151105547
H346	10.583764914	0.570258604	-4.443739291
H347	9.358373010	0.489886010	-0.595738801
H348	4.116734507	1.556382464	-6.683905585
H349	1.753353526	8.385746868	-3.052966288
H350	4.248636051	2.240173411	7.279436165
H351	4.441045982	3.348289444	5.900855547
H352	12.285241554	2.924536015	-2.343809243
H353	12.531028389	1.306062907	-2.348360208
H354	13.344272837	0.065745019	1.968835430
H355	12.177768885	0.019692665	0.009441421
H356	14.126685640	2.513664562	1.041945338
H357	11.976720823	0.980082487	2.638975618
H358	6.515244808	4.624065157	7.174023264
H359	-11.982363038	6.081839330	4.211528337
H360	10.324709086	-2.190047691	8.434019296
H361	8.598311483	-0.203142336	7.611447349
H362	-12.018699006	5.613356038	1.227592998
H363	-10.759531688	3.463400363	3.043599960
H364	-12.244333749	3.273277474	2.093538716
H365	3.285256374	-1.392008405	-0.776743503
H366	-8.735558322	2.082893303	-3.197105023
H367	0.707202572	-0.544882549	-0.289776620
H368	2.724774311	-0.566446010	0.562083799
H369	-1.071264686	-0.902127307	-1.741524683
Mo370	6.159114240	-0.093417435	-0.989349290
N371	-0.098315863	-8.846888892	-5.230748091
N372	-3.485420738	-8.806410568	-3.639223164
N373	-3.453967032	-9.777784135	-1.001873487
N374	-1.004180306	-8.935800994	0.116511528
N375	-1.451125836	-6.141366291	0.350604797
N376	-3.538044188	-6.144436339	2.314878350
N377	-2.462348072	-7.604283497	4.581406938
N378	0.306015088	-6.879493215	4.968339081
N379	0.703886623	-4.138353231	4.369057407
N380	-1.536742581	-2.521203569	5.469658783
N381	-2.416653235	-4.141643500	7.785922299
N382	10.313907490	-7.691671501	5.093890524
N383	9.610730840	-5.121954425	4.783336856
N384	4.910043278	-1.037887954	4.420540191
N385	3.162194062	-1.333531027	5.972512752
N386	3.332653817	0.623458674	4.736896927
N387	10.022061330	-2.039562959	6.390988714
N388	-1.411544325	-3.927110162	-5.906745314
N389	3.170340334	-3.626860881	-4.727502769
N390	-3.435321440	-2.624951476	-4.883110066
N391	-6.052254390	-3.622285406	-4.767077805
N392	-6.174924753	-4.801880130	-2.215366308
N393	-5.984765703	-2.628768108	-0.448980430
N394	-3.773806346	0.656408802	-2.008621774
N395	-2.040671799	-0.581819316	-1.683609731
N396	-8.376669211	-1.387369466	0.226474569
N397	-12.464680110	-0.490425896	-2.020709802
N398	-13.902546724	0.283508527	-0.518456696
N399	4.136722994	5.646050118	7.300626304
N400	-0.458827044	8.537638356	4.239089975
N401	-2.073033025	9.497784215	1.251447958
N402	-3.389629499	7.890340404	-0.570799844
N403	-5.444907587	4.873949292	-4.117295207
N404	-6.483097288	6.767957455	-5.027401419
N405	-7.728002785	5.205514946	-3.864722791
N406	-3.610924221	5.301995420	0.663037872
N407	-5.179785644	5.923436839	2.886632392
N408	-7.639638556	7.032470018	2.373625563
N409	-7.489289221	7.594911702	-1.499762176
N410	-10.251330926	6.005980402	2.297404521
N411	8.527846318	3.535011295	-7.488501812
N412	5.325378716	3.042242852	-5.892166500
N413	2.568038920	4.209663982	-5.425483762
N414	2.631898524	6.725892296	-4.027136133
N415	3.286052409	1.559825026	-9.894983237
N416	0.582939517	1.293383090	-8.998787311
N417	11.805939291	2.024572399	-2.441353654
N418	7.806290973	0.139111780	-2.685137969
N419	8.501433561	0.075927921	-4.776122790

	N420	12.132614009	0.908579212	0.513528042
	N421	-1.630951758	1.773373420	0.262730448
	N422	-0.524175023	1.953357264	0.123568300
	O423	-1.506440357	-7.987328818	-2.872584565
	O424	-4.103582440	-7.728101028	-0.246998225
	O425	-1.871895420	-9.317058905	2.193057779
	O426	-0.053430752	-6.176148748	2.145648229
	O427	-2.774055217	-4.092401679	2.942375574
	O428	-3.567364302	-6.056571418	5.853021127
	O429	0.039977010	-7.375435245	7.193269024
	O430	2.637903315	-4.169119643	5.613941214
	O431	0.255011454	-1.295046009	6.189157881
	O432	-2.172205498	-2.178849772	8.900773534
	O433	-1.431565972	-4.661623633	11.189604135
	O434	11.326427495	-4.680032172	3.298317737
	O435	9.223382825	-3.960317253	7.368986230
	O436	10.042954250	0.651963130	8.771124283
	O437	-3.397017346	-3.730221600	-2.869796105
	O438	1.352790111	-3.325527036	-6.061435557
	O439	-6.608095837	-1.913959227	-3.355459853
	O440	-5.691040360	0.264549524	-5.342462880
	O441	-8.411678758	-5.219374421	-2.514794300
	O442	-6.049816988	-4.023152808	1.360309247
	O443	-7.396541163	-0.445671412	2.064070315
	O444	-10.922929507	-0.956081379	2.652388530
	O445	6.454700294	3.533740923	8.899177383
	O446	-1.714843306	3.287586593	6.010495520
	O447	-3.026395934	8.136544479	2.809984320
	O448	-5.248542660	9.238251430	-0.550295463
	O449	-5.475893052	5.023689427	-0.635298079
	O450	-5.998159498	3.779124666	2.870588760
	O451	-2.678717951	4.611416875	3.613490642
	O452	-7.901991854	7.672660468	4.559399934
	O453	-11.114234098	8.061492437	1.821842789
	O454	-9.442951486	6.699716007	-0.722818259
	O455	-13.664159118	5.410809238	3.267719858
	O456	-8.158444521	2.494443307	-2.524750788
	O457	6.429511234	1.722333175	-7.413635128
	O458	2.375237204	3.222997833	-7.483775588
	O459	1.020308727	7.522491943	-5.425464296
	O460	3.464326754	10.238067848	-3.812389397
	O461	0.472286406	-0.530078067	-10.361520202
	O462	4.957954552	-2.742464134	-7.022555813
	O463	5.887660701	-0.754913761	-6.622453764
	O464	-2.284593215	3.364421819	-8.561555960
	O465	11.456636908	3.065113500	0.677083525
	O466	13.938923092	2.658600786	3.069811695
	O467	4.740820212	-3.802051606	-2.186903114
	O468	4.614553288	-5.725322887	-1.029672351
	O469	11.728190759	-3.595650272	-2.413432606
	O470	10.184376345	-4.757537191	-3.590771274
	O471	7.775444345	-0.950343055	0.223564372
	O472	8.532007349	-2.867863225	1.115829632
	O473	6.397732447	-2.101598709	-1.658446556
	O474	10.390559063	-0.506817917	4.026383132
	O475	7.919547211	0.217400069	2.814132640
	O476	-5.682854332	2.127554362	-3.333297340
	O477	12.982582739	-2.279657129	-0.343815049
	O478	11.049750109	-2.241292119	1.968920131
	S479	-0.360501279	6.357806150	0.749594564
	S480	0.906448905	3.954171038	-1.440524672
	S481	4.422792471	0.146995944	-2.600969170
	S482	1.238875560	3.016769370	2.062319885
	S483	1.309893261	0.218932379	-1.250559258
	S484	4.535368907	4.038707668	-2.781909479
	S485	4.915694060	-0.567019154	0.963149664
	S486	3.402177990	5.498590290	0.648924037
	S487	6.709586405	2.170077059	-0.559559405
	S488	5.002240452	2.708543465	2.748978094
	end			

product

	bm522bh6x6hn2n2xt3g.car_2			
Fe(139) -2.268	C1	-2.136920861	-8.611733535	-3.743239227
Fe(140) -0.962	C2	-1.468664978	-9.248655085	-4.966482608
Fe(141) -2.601	C3	-4.259413327	-8.116683734	-2.618323724
Fe(142) 2.757	C4	-3.931340146	-8.526573736	-1.186195130
Fe(143) 2.713	C5	-3.075329651	-10.247900844	0.319403246
	C6	-1.934259194	-9.452028316	0.959830718
	C7	0.043126226	-8.063069754	0.627819902

Fe(144) 2.295<br>Fe(145) 0.892	C8 -0.488025703 -6.713592900 1.112879400<br>C9 -2.099602407 -4.906743710 0.762105976<br>C10 -2.831752776 -5.010518796 2.107391635<br>C11 -4.281001150 -6.309472257 3.554440682<br>C12 -3.395104906 -6.634371921 4.765636506<br>C13 -1.689068885 -8.121762960 5.699639474<br>C14 -0.373878968 -7.402274909 6.026550681<br>C15 1.620667385 -6.283461660 5.124562888<br>C16 1.687679596 -4.756548600 5.067501901<br>C17 0.582759925 -2.689386605 4.220958995<br>C18 -0.247986892 -2.102343815 5.385385602<br>C19 0.023389927 -2.322247932 2.827447069<br>C20 -0.287092412 -0.822091341 2.702543136<br>C21 0.988669212 -2.783412625 1.731376346<br>C22 -2.456819033 -2.022857031 6.493792891<br>C23 -2.327802993 -2.787616268 7.828548095<br>C24 -3.912286481 -2.010566537 5.961828503<br>C25 -4.903284536 -1.614862284 7.062609122<br>C26 -4.047218997 -1.070704299 4.756827387<br>C27 -2.466185196 -4.900794437 9.029716825<br>C28 -1.345699117 -4.528231184 9.977307068<br>C29 10.999291772 -6.950867045 4.027124631<br>C30 10.661118290 -5.460494476 4.009855212<br>C31 9.076642263 -3.779771984 4.954221725<br>C32 9.447033214 -3.268005671 6.360659493<br>C33 7.554007021 -3.787889095 4.739405991<br>C34 6.920988259 -2.402940980 4.881441096<br>C35 5.462875298 -2.396457366 4.421564749<br>C36 3.807693441 -0.597106502 5.044534380<br>C37 10.453947101 -1.436104152 7.637168720<br>C38 9.632542185 -0.229828946 8.033052130<br>C39 -1.558054949 -4.099413647 -4.458018762<br>C40 -2.888555316 -3.484210838 -3.981560337<br>C41 -0.411469042 -3.414141524 -3.678144035<br>C42 0.972700860 -4.087575100 -3.778611410<br>C43 1.843431349 -3.638101446 -4.951998509<br>C44 -4.518957140 -1.715529014 -4.547875112<br>C45 -5.829046983 -2.425376456 -4.178949446<br>C46 -4.687671749 -0.706919323 -5.694128726<br>C47 -7.209102122 -4.433331351 -4.429731893<br>C48 -7.317129383 -4.849219407 -2.957922206<br>C49 -6.189511045 -5.075087000 -0.789860219<br>C50 -6.070234538 -3.863554742 0.134699713<br>C51 -5.936175410 -1.433949282 0.383506692<br>C52 -7.308108293 -1.065931978 1.000846753<br>C53 -5.487912158 -0.202436629 -0.415436903<br>C54 -4.123980112 -0.252733704 -1.020578870<br>C55 -3.015421293 -1.035488015 -0.814283366<br>C56 -2.508639845 0.450379443 -2.396792855<br>C57 -9.730197047 -0.936878811 0.530084343<br>C58 -10.163993215 -1.511540225 1.877238238<br>C59 -10.652142263 -1.436424436 -0.605276401<br>C60 -11.971613075 -0.738450969 -0.748611465<br>C61 -12.857719222 -0.262519504 0.197708373<br>C62 -13.628150186 0.123770372 -1.844556848<br>C63 4.416638203 4.312596031 7.840656629<br>C64 5.918614117 4.146971479 7.988399253<br>C65 3.930694133 3.208866086 6.866529428<br>C66 2.434736214 3.222931695 6.664810822<br>C67 1.577761746 2.480715743 7.496834361<br>C68 1.853797610 3.983764451 5.633582199<br>C69 0.191942331 2.484621346 7.304768758<br>C70 0.471622118 4.013148570 5.442248942<br>C71 -0.357343839 3.253551118 6.271057114<br>C72 -0.590599091 8.271246751 2.797562831<br>C73 -2.002468712 8.615704245 2.292448522<br>C74 -0.279141990 6.798838866 2.545895131<br>C75 -3.340316282 9.965449011 0.719450964<br>C76 -4.081266616 8.986421249 -0.193960383<br>C77 -3.948881865 6.771000914 -1.321187327<br>C78 -4.411700040 5.616912562 -0.394349039<br>C79 -2.860178282 6.306763951 -2.319548102<br>C80 -3.122965022 5.070625893 -3.188377291<br>C81 -4.057498248 5.258210178 -4.396851858<br>C82 -6.533141437 5.603899807 -4.357042561<br>C83 -4.001304444 4.227034234 1.578978884<br>C84 -5.172472927 4.618671513 2.491372650<br>C85 -2.842094929 3.751601317 2.481190644<br>C86 -5.966551464 6.350535477 4.027063008<br>C87 -7.253581409 7.084284079 3.685765927

C88	-8.699895573	7.906359039	1.914427407
C89	-10.134181621	7.321784644	1.990255429
C90	-8.449029348	8.517602303	0.532261799
C91	-8.510056298	7.507314206	-0.604293440
C92	-11.523225627	5.303937067	2.163151653
C93	-12.464772303	5.634547989	3.310725282
C94	-11.262903811	3.770051706	2.111677906
C95	-10.442917873	3.356516622	0.908658336
C96	-11.071842288	3.050868248	-0.308976219
C97	-9.038554694	3.310379727	0.957189043
C98	-10.329106908	2.729197879	-1.446248398
C99	-8.277260661	3.020470427	-0.178716442
C100	-8.936074698	2.738642175	-1.371910693
C101	7.677423605	3.462866621	-6.295872052
C102	6.417357355	2.673039165	-6.594203334
C103	4.044170537	2.351076746	-5.938783623
C104	2.909719932	3.293803211	-6.357727765
C105	1.541094764	5.207759427	-5.666209201
C106	1.731628249	6.582938574	-5.033077617
C107	2.744411074	7.990721814	-3.322640876
C108	3.487224570	9.055639427	-4.110362841
C109	2.686057918	0.230316886	-9.731473324
C110	1.154410015	0.299761150	-9.737413597
C111	3.168462909	-0.608091544	-8.526363315
C112	4.639837462	-1.030310313	-8.694349117
C113	5.178041843	-1.596359007	-7.400322439
C114	-0.861144019	1.431767288	-8.944215841
C115	-1.247431899	2.897431462	-9.009557043
C116	-1.554366179	0.633908653	-7.810465155
C117	-0.892127567	0.881278616	-6.482867197
C118	-1.223548360	2.005648282	-5.709778496
C119	0.178332907	0.067255430	-6.071865344
C120	-0.457486077	2.343752620	-4.589854960
C121	0.945621834	0.406359055	-4.955202018
C122	0.645103302	1.563748075	-4.229777675
C123	10.820917337	1.886544425	-1.352680805
C124	11.476696854	1.998162878	0.032494229
C125	10.005045366	0.590533137	-1.475121353
C126	9.167316082	0.447237089	-2.706679797
C127	9.600060653	0.413447174	-4.016078041
C128	7.445419075	-0.089816807	-3.947427576
C129	12.730618292	0.971023416	1.835723338
C130	13.653641264	2.160196256	1.989203807
C131	5.102048670	-4.606492536	-1.179865994
C132	6.107600109	-4.003952395	-0.201805872
C133	7.130179874	-3.023638860	-0.846819236
C134	8.186749300	-3.821043153	-1.660080038
C135	9.401901146	-3.016035237	-2.126823824
C136	10.525321690	-3.870633767	-2.758575328
C137	7.866658769	-2.254589429	0.261077890
C138	3.435044258	2.275884921	-0.176648638
Fe139	4.743440908	2.151837264	-1.650738460
Fe140	1.251261113	2.191553330	-0.083558948
Fe141	3.373638253	3.423859846	1.443057906
Fe142	3.125943691	4.005965411	-1.060520132
Fe143	1.253547242	4.759921735	0.664425036
Fe144	5.053550634	1.652316180	0.831070826
Fe145	3.458093482	0.089064377	-0.551282135
H146	0.477322510	-9.010218642	-4.401126974
H147	-0.053548384	-7.840036587	-5.407329135
H148	-3.982953660	-9.227295815	-4.420157825
H149	-2.088774033	-9.044290771	-5.854045277
H150	-1.502684176	-10.340893976	-4.817206634
H151	-4.102573748	-7.029014899	-2.673750402
H152	-3.333692250	-10.380300569	-1.812035385
H153	-5.324447846	-8.317102146	-2.797790882
H154	-2.767841823	-11.299047320	0.237789546
H155	-1.144978158	-8.994433399	-0.893528584
H156	-3.926073931	-10.193604004	1.012150244
H157	-1.357608372	-4.106652928	0.866067762
H158	-1.794902338	-6.631071354	-0.472139705
H159	0.782056270	-7.896195169	-0.168111531
H160	-4.827165181	-5.388426258	3.790682311
H161	-3.652418254	-6.819280902	1.556311809
H162	0.549507480	-8.536065316	1.478106183
H163	-2.808617555	-4.623366190	-0.027820439
H164	-2.402617028	-8.090365526	3.682379119
H165	-5.005103832	-7.124657826	3.418087206
H166	1.195390598	-3.860742208	1.786609084
H167	0.567478381	-2.582876411	0.733370131

	H168	1.945162531	-2.244085606	1.797800961
	H169	-0.927088619	-2.870538679	2.717439894
	H170	-1.441662443	-9.172685814	5.490369956
	H171	-3.406836346	-1.373180428	3.917522646
	H172	-2.671195182	-4.637503704	6.927459874
	H173	-4.885939987	-2.310865673	7.913857794
	H174	0.607826265	-0.239019081	2.438931085
	H175	-0.699623855	-0.389254297	3.626255195
	H176	3.963157937	4.117373172	8.830750381
	H177	-5.088005313	-1.067199863	4.400908241
	H178	-5.923021225	-1.607069806	6.652147864
	H179	-3.785130814	-0.038518110	5.040979596
	H180	-4.684091940	-0.605621566	7.445901636
	H181	-4.155842454	-3.037307451	5.634642642
	H182	-2.151384908	-0.994967143	6.736126910
	H183	-1.913102641	-3.129327817	4.738957929
	H184	-1.040875138	-0.655840349	1.917143314
	H185	-1.914516724	3.721792604	1.887172203
	H186	-3.083254394	2.727188695	2.802974494
	H187	-2.392415106	4.067357817	4.375702727
	H188	-2.190279648	2.738708317	6.660449369
	H189	0.035108117	4.596526766	4.631376497
	H190	2.493793747	4.545676328	4.949364473
	H191	1.995905458	1.875207838	8.304808036
	H192	0.024825410	-4.723638110	3.883716908
	H193	2.294394009	-6.668233276	4.343347003
	H194	2.024745036	-6.585054714	6.097813399
	H195	-0.057792872	-6.986235098	4.018633376
	H196	-2.298546329	-8.088206636	6.611479272
	H197	-0.455509829	1.879482072	7.944025092
	H198	1.587637647	-2.265421596	4.330708907
	H199	-3.431370044	-4.791156708	9.549635697
	H200	-2.328191797	-5.963012109	8.772452919
	H201	-0.399461231	-4.195802570	9.491401028
	H202	11.518365751	-1.157156000	7.602572436
	H203	3.799934333	-3.360734373	-5.491907211
	H204	5.372478377	-2.920484400	-2.083031644
	H205	4.706444407	-1.810768362	-9.465449241
	H206	5.249112018	-0.173454566	-9.017021709
	H207	2.547827877	-1.510925593	-8.419808500
	H208	-0.512575464	3.537872415	-9.550594034
	H209	2.921454183	-0.342077618	-10.641676776
	H210	-0.338995962	-2.365729837	-4.012569150
	H211	0.856634124	-5.182530342	-3.860686246
	H212	1.168725625	1.930827560	-8.450944715
	H213	-1.228508955	1.010938531	-9.903312417
	H214	3.245855454	2.103961570	-9.025111201
	H215	3.581254358	-3.818369138	-3.808123806
	H216	3.030307895	-0.021922877	-7.604617053
	H217	4.272260566	1.466156532	-10.146248216
	H218	-1.705550335	-4.769897220	-6.404239756
	H219	-0.701816827	-3.408160634	-2.616377506
	H220	5.998726967	0.154013352	-7.039688670
	H221	1.521261230	-3.919390057	-2.840051342
	H222	-5.262380642	-1.618675147	1.231883676
	H223	-2.846509746	-1.866789033	-0.141789554
	H224	-7.144897568	-5.563143097	-0.551589624
	H225	-5.297237409	-4.474809389	-2.633270224
	H226	-8.225267617	-1.941722722	-0.612546008
	H227	-12.831309050	-0.246117628	1.281850696
	H228	-9.747248105	-2.526598114	2.087187646
	H229	-10.789760893	-2.527330076	-0.506054488
	H230	-9.761326348	0.163064227	0.589307063
	H231	-14.299578167	0.466220423	-2.628483025
	H232	-5.536756685	0.664073831	0.263911753
	H233	-6.215622865	-0.018811520	-1.222847170
	H234	-14.727980698	0.726428963	-0.122194858
	H235	-10.116332230	-1.281031384	-1.554956239
	H236	-6.070554884	-2.530496971	-1.464858350
	H237	-5.390686859	-5.782877613	-0.522399943
	H238	-8.139128742	-3.901070858	-4.669210025
	H239	-1.952082930	1.008568923	-3.146983519
	H240	-7.178496693	-5.342523424	-5.043895611
	H241	4.272083869	6.347492561	8.033565389
	H242	3.149633393	5.703596133	7.034475216
	H243	-2.649522400	5.679671529	0.730624921
	H244	-4.343565026	3.368744985	0.983205770
	H245	-4.359135958	6.495390368	2.667885958
	H246	-4.451053922	1.337497608	-2.447719606
	H247	-5.730466461	1.482180407	-4.092423613

H248	-5.972312522	0.694224622	-6.169585428
H249	-6.566565291	2.104134430	-2.884819184
H250	-3.714367499	-0.217260864	-5.863106501
H251	-4.984536401	-1.232223651	-6.614812005
H252	-7.496739287	9.066098373	0.539465309
H253	-9.256976191	9.245142287	0.359297544
H254	-6.644544532	8.128736253	-1.259827167
H255	-7.418543023	6.815226760	-2.150796605
H256	-7.284766396	7.389472022	-5.037587040
H257	-3.704350159	4.646423623	-5.240726456
H258	-10.832668878	2.475057230	-2.381734986
H259	-8.507702133	3.488529757	1.896154259
H260	-7.804871591	4.321079744	-3.357364887
H261	-1.948131207	6.104380673	-1.733410239
H262	-2.432728327	7.787325575	-0.217293192
H263	-3.470634869	4.224444292	-2.574710077
H264	-5.336322707	-3.997013895	-5.385862962
H265	-3.168689424	10.891699062	0.155364341
H266	-2.874622624	-2.570537021	-5.751967448
H267	-2.623499831	7.165913626	-2.969577023
H268	-7.187591183	3.052998217	-0.132991919
H269	-4.030723249	10.196383213	1.543828815
H270	-5.634404794	7.086765540	-5.479347722
H271	-8.576981267	5.547187296	-4.306417343
H272	-0.417383145	-3.738823238	-6.133404352
H273	-1.587900055	-5.158361274	-4.142289083
H274	-12.161777506	3.054219613	-0.369296630
H275	-4.257118023	-1.163088535	-3.635699514
H276	-4.843608645	7.120906613	-1.846244938
H277	-4.020831061	6.303457927	-4.726443403
H278	-2.146544544	4.770918921	-3.592863746
H279	-5.579764181	3.906552197	-3.775445070
H280	-6.236115294	5.476025261	4.638387506
H281	-6.977445968	6.630008080	1.710759902
H282	-8.735008315	8.743332993	2.629328876
H283	-5.366694463	7.028550370	4.648984245
H284	-9.392365893	5.461909862	2.313806904
H285	7.414620134	4.478912272	-5.969392705
H286	8.150288400	2.959110296	-5.427419734
H287	10.139961005	2.747760362	-1.415953021
H288	9.461807181	3.842174728	-7.208924142
H289	8.636968980	2.589163226	-7.865292775
H290	0.732552129	6.602042800	2.922130221
H291	-1.225024860	8.061721425	4.726262558
H292	-1.210910243	9.888647640	0.880627688
H293	-1.000419916	6.170983119	3.087541372
H294	-0.589565232	9.536886459	4.417834669
H295	3.298862863	7.818101533	-2.387614638
H296	0.148797634	8.891492999	2.268234911
H297	7.097696556	-4.492930660	5.453551007
H298	9.545855282	-3.144327643	4.192038318
H299	3.176387630	5.934256986	-3.673372851
H300	2.637890603	1.056945597	5.339886430
H301	1.463118074	5.354576600	-6.750164977
H302	1.785537984	-0.224429036	-4.653140312
H303	0.557914352	4.840144050	-5.325103160
H304	4.848704708	-3.049253989	5.051835205
H305	2.982794196	4.092459511	-4.484328399
H306	5.389396555	-2.789953057	3.393825345
H307	7.375412076	-4.179714297	3.725457744
H308	7.476989106	-1.673336599	4.272916231
H309	6.965169137	-2.060836128	5.927473612
H310	-1.506152334	-0.431624084	-8.077040320
H311	9.223458253	-5.897604587	5.330632647
H312	10.678201796	-7.346461496	3.051901425
H313	1.256070098	1.865950549	-3.379388641
H314	10.889295256	-7.712970494	5.938229655
H315	10.169806585	-8.662735595	4.813577356
H316	5.379053537	-0.375843351	3.799657984
H317	-0.697181605	3.226595296	-3.994619097
H318	0.425018021	-0.832980144	-6.638821660
H319	12.380064957	-2.367600630	0.428363204
H320	10.694647613	-0.270551217	-1.444392427
H321	10.134475893	-2.364775115	1.603625451
H322	12.473517049	-2.723384361	-1.085823954
H323	4.085179590	8.680012542	-4.975138440
H324	7.934417343	-0.187604256	1.909724531
H325	6.618945557	-4.815932131	0.328141031
H326	5.437042166	3.733969895	-5.146471001
H327	8.530741477	-4.641439928	-1.010459824

H328	8.492552938	-0.090401605	-5.779073122
H329	6.465983941	-0.391181335	-4.291945379
H330	12.100226884	-7.022925566	4.052570497
H331	3.841182070	1.246654336	4.087833820
H332	9.541560049	-0.149762901	3.664924844
H333	7.640301560	1.139958408	2.664118112
H334	10.167179652	-1.508012721	5.504057801
H335	3.293873620	-2.350217844	5.968680795
H336	9.083252401	-2.281503642	-2.882471432
H337	9.832579518	-2.449415700	-1.289724812
H338	7.691734714	-4.287110478	-2.526391798
H339	3.834351427	1.906340837	-4.952934110
H340	-2.058875869	2.638315791	-6.019286169
H341	-2.611905888	0.940451851	-7.803187005
H342	5.504294922	-3.453489006	0.540281560
H343	10.719655322	-1.091251958	3.288987282
H344	11.197516428	-3.092020028	2.456659100
H345	2.173247690	-1.096424949	6.151105816
H346	10.583765115	0.570258671	-4.443739022
H347	9.358373211	0.489886077	-0.595738532
H348	4.116734708	1.556382531	-6.683905316
H349	1.753353727	8.385746935	-3.052966019
H350	4.248636252	2.240173478	7.279436434
H351	4.441046183	3.348289511	5.900855816
H352	12.285241755	2.924536082	-2.343808974
H353	12.531028590	1.306062974	-2.348359939
H354	13.344273038	0.065745086	1.968835699
H355	12.177769086	0.019692732	0.009441690
H356	14.126685841	2.513664629	1.041945607
H357	11.976721024	0.980082554	2.638975887
H358	6.515245009	4.624065224	7.174023533
H359	-11.982362837	6.081839397	4.211528606
H360	10.324709287	-2.190047624	8.434019565
H361	8.598311684	-0.203142269	7.611447618
H362	-12.018698805	5.613356105	1.227593267
H363	-10.759531487	3.463400430	3.043600229
H364	-12.244333548	3.273277541	2.093538985
H365	3.285256575	-1.392008338	-0.776743234
H366	-8.735558121	2.082893370	-3.197104754
H367	0.707202773	-0.544882482	-0.289776351
H368	2.724774512	-0.566445943	0.562084068
H369	-1.071264485	-0.902127240	-1.741524414
Mo370	6.159114441	-0.093417368	-0.989349021
N371	-0.098315662	-8.846888825	-5.230747822
N372	-3.485420537	-8.806410501	-3.639222895
N373	-3.453966831	-9.777784068	-1.001873218
N374	-1.004180105	-8.935800927	0.116511797
N375	-1.451125635	-6.141366224	0.350605066
N376	-3.538043987	-6.144436272	2.314878619
N377	-2.462347871	-7.604283430	4.581407207
N378	0.306015289	-6.879493148	4.968339350
N379	0.703886824	-4.138353164	4.369057676
N380	-1.536742380	-2.521203502	5.469659052
N381	-2.416653034	-4.141643433	7.785922568
N382	10.313907691	-7.691671434	5.093890793
N383	9.610731041	-5.121954358	4.783337125
N384	4.910043479	-1.037887887	4.420540460
N385	3.162194263	-1.333530960	5.972513021
N386	3.332654018	0.623458741	4.736897196
N387	10.022061531	-2.039562892	6.390988983
N388	-1.411544124	-3.927110095	-5.906745045
N389	3.170340535	-3.626860814	-4.727502500
N390	-3.435321239	-2.624951409	-4.883109797
N391	-6.052254189	-3.622285339	-4.767077536
N392	-6.174924552	-4.801880063	-2.215366039
N393	-5.984765502	-2.628768041	-0.448980161
N394	-3.773806145	0.656408869	-2.008621505
N395	-2.040671598	-0.581819249	-1.683609462
N396	-8.376669010	-1.387369399	0.226474838
N397	-12.464679909	-0.490425829	-2.020709533
N398	-13.902546523	0.283508594	-0.518456427
N399	4.136723195	5.646050185	7.300626573
N400	-0.458826843	8.537638423	4.239090244
N401	-2.073032824	9.497784282	1.251448227
N402	-3.389629298	7.890340471	-0.570799575
N403	-5.444907386	4.873949359	-4.117294938
N404	-6.483097087	6.767957522	-5.027401150
N405	-7.728002584	5.205515013	-3.864722522
N406	-3.610924020	5.301995487	0.663038141
N407	-5.179785443	5.923436906	2.886632661

	N408	-7.639638355	7.032470085	2.373625832
	N409	-7.489289020	7.594911769	-1.499761907
	N410	-10.251330725	6.005980469	2.297404790
	N411	8.527846519	3.535011362	-7.488501543
	N412	5.325378917	3.042242919	-5.892166231
	N413	2.568039121	4.209664049	-5.425483493
	N414	2.631898725	6.725892363	-4.027135864
	N415	3.286052610	1.559825093	-9.894982968
	N416	0.582939718	1.293383157	-8.998787042
	N417	11.805939492	2.024572466	-2.441353385
	N418	7.806291174	0.139111847	-2.685137700
	N419	8.501433762	0.075927988	-4.776122521
	N420	12.132614210	0.908579279	0.513528311
	N421	-1.630951557	1.773373487	0.262730717
	N422	-0.524174822	1.953357331	0.123568569
	O423	-1.506440156	-7.987328751	-2.872584296
	O424	-4.103582239	-7.728100961	-0.246997956
	O425	-1.871895219	-9.317058838	2.193058048
	O426	-0.053430551	-6.176148681	2.145648498
	O427	-2.774055016	-4.092401612	2.942375843
	O428	-3.567364101	-6.056571351	5.853021396
	O429	0.039977211	-7.375435178	7.193269293
	O430	2.637903516	-4.169119576	5.613941483
	O431	0.255011655	-1.295045942	6.189158150
	O432	-2.172205297	-2.178849705	8.900773803
	O433	-1.431565771	-4.661623566	11.189604404
	O434	11.326427696	-4.680032105	3.298318006
	O435	9.223383026	-3.960317186	7.368986499
	O436	10.042954451	0.651963197	8.771124552
	O437	-3.397017145	-3.730221533	-2.869795836
	O438	1.352790312	-3.325526969	-6.061435288
	O439	-6.608095636	-1.913959160	-3.355459584
	O440	-5.691040159	0.264549591	-5.342462611
	O441	-8.411678557	-5.219374354	-2.514794031
	O442	-6.049816787	-4.023152741	1.360309516
	O443	-7.396540962	-0.445671345	2.064070584
	O444	-10.922929306	-0.956081312	2.652388799
	O445	6.454700495	3.533740990	8.899177652
	O446	-1.714843105	3.287586660	6.010495789
	O447	-3.026395733	8.136544546	2.809984589
	O448	-5.248542459	9.238251497	-0.550295194
	O449	-5.475892851	5.023689494	-0.635297810
	O450	-5.998159297	3.779124733	2.870589029
	O451	-2.678717750	4.611416942	3.613490911
	O452	-7.901991653	7.672660535	4.559400203
	O453	-11.114233897	8.061492504	1.821843058
	O454	-9.442951285	6.699716074	-0.722817990
	O455	-13.664158917	5.410809305	3.267720127
	O456	-8.158444320	2.494443374	-2.524750519
	O457	6.429511435	1.722333242	-7.413634859
	O458	2.375237405	3.222997900	-7.483775319
	O459	1.020308928	7.522492010	-5.425464027
	O460	3.464326955	10.238067915	-3.812389128
	O461	0.472286607	-0.530078000	-10.361519933
	O462	4.957954753	-2.742464067	-7.022555544
	O463	5.887660902	-0.754913694	-6.622453495
	O464	-2.284593014	3.364421886	-8.561555691
	O465	11.456637109	3.065113567	0.677083794
	O466	13.938923293	2.658600853	3.069811964
	O467	4.740820413	-3.802051539	-2.186902845
	O468	4.614553489	-5.725322820	-1.029672082
	O469	11.728190960	-3.595650205	-2.413432337
	O470	10.184376546	-4.757537124	-3.590771005
	O471	7.775444546	-0.950342988	0.223564641
	O472	8.532007550	-2.867863158	1.115829901
	O473	6.397732648	-2.101598642	-1.658446287
	O474	10.390559264	-0.506817850	4.026383401
	O475	7.919547412	0.217400136	2.814132909
	O476	-5.682854131	2.127554429	-3.333297071
	O477	12.982582940	-2.279657062	-0.343814780
	O478	11.049750310	-2.241292052	1.968920400
	S479	-0.360501078	6.357806217	0.749594833
	S480	0.906449106	3.954171105	-1.440524403
	S481	4.422792672	0.146996011	-2.600968901
	S482	1.238875761	3.016769437	2.062320154
	S483	1.309893462	0.218932446	-1.250558989
	S484	4.535369108	4.038707735	-2.781909210
	S485	4.915694261	-0.567019087	0.963149933
	S486	3.402178191	5.498590357	0.648924306
	S487	6.709586606	2.170077126	-0.559559136

	S488 5.002240653 2.708543532 2.748978363<br>end

S2BH-6Hn-6Hx-3b6 to S2BH-6Hx-6H2n

35, S=1

reactant

	bm522bh2n2x6x263b1b.car_4
Fe(139) -2.377<br>Fe(140) -1.047<br>Fe(141) -2.545<br>Fe(142) 2.686<br>Fe(143) 2.704<br>Fe(144) 2.196<br>Fe(145) 0.114	C1 -7.829259340 -5.264596169 1.911536358<br>C2 -8.360941860 -6.416602410 1.051794252<br>C3 -8.405325515 -3.247718223 3.180836149<br>C4 -7.618163553 -3.474593061 4.467564944<br>C5 -7.083325646 -4.971574291 6.321678790<br>C6 -5.569254223 -5.080963436 6.123561517<br>C7 -3.723569538 -5.537821388 4.594094457<br>C8 -3.129401042 -4.135375673 4.448385584<br>C9 -3.486938834 -1.835441524 3.698220666<br>C10 -3.297495925 -1.141044793 5.055171660<br>C11 -4.136669354 -0.760348368 7.298237424<br>C12 -3.051612355 -1.359627483 8.202927537<br>C13 -2.150164083 -3.390769933 9.230614808<br>C14 -0.726119508 -3.722400480 8.766321921<br>C15 0.683449734 -4.487960482 6.901233515<br>C16 1.468001976 -3.471566175 6.068456414<br>C17 1.314362493 -1.447225421 4.623163460<br>C18 1.686460557 -0.236613351 5.508865884<br>C19 0.376408504 -1.074400538 3.455343631<br>C20 0.879623154 0.152190965 2.686643789<br>C21 0.184044019 -2.272840329 2.520145476<br>C22 0.852845246 1.555763778 6.996335413<br>C23 1.278091907 1.198593560 8.436646898<br>C24 -0.404037957 2.462079763 6.965400020<br>C25 -0.263145557 3.635464299 7.941480655<br>C26 -0.671441423 2.974926153 5.544788965<br>C27 0.776325293 0.055156038 10.520759890<br>C28 2.226918101 -0.289173423 10.789170350<br>C29 6.015091032 -11.624234949 3.715207600<br>C30 6.550130601 -10.357984389 3.050119468<br>C31 6.848458724 -7.910470912 3.418838655<br>C32 8.122496477 -7.536891412 4.201040874<br>C33 5.712769326 -6.906760359 3.681248391<br>C34 6.083469038 -5.476429722 3.294164084<br>C35 4.869640418 -4.550570937 3.302256260<br>C36 5.021150285 -2.030145235 3.353990331<br>C37 10.412983913 -6.684832406 4.019099277<br>C38 10.703090782 -5.201491586 3.978786149<br>C39 -5.549012089 -2.658082934 -1.117206893<br>C40 -5.861990828 -1.214874549 -0.681379303<br>C41 -4.011507157 -2.816493535 -1.166379758<br>C42 -3.487405908 -4.262206031 -1.283773190<br>C43 -3.314702444 -4.783294010 -2.710265226<br>C44 -6.363234649 1.025348157 -1.583225958<br>C45 -7.394427248 1.508891830 -0.553871190<br>C46 -6.594891764 1.621164772 -2.977657373<br>C47 -9.471690478 1.010700761 0.644500611<br>C48 -8.964718926 1.071610854 2.089409855<br>C49 -7.144154003 0.574236430 3.657228243<br>C50 -5.948116791 1.519647026 3.762197303<br>C51 -4.490311470 3.168301406 2.702419997<br>C52 -4.891522834 4.485396515 3.408533547<br>C53 -3.986905654 3.572003785 1.309298074<br>C54 -3.433829907 2.481403200 0.454330670<br>C55 -3.014514458 1.197655595 0.696431292<br>C56 -2.724794396 1.602844694 -1.476471063<br>C57 -6.688111979 6.159124201 3.634194139<br>C58 -6.532573891 6.319143094 5.146082200<br>C59 -8.177920334 6.228001562 3.230247994<br>C60 -8.779738732 7.597583249 3.116663831<br>C61 -8.629869439 8.721626612 3.906998237<br>C62 -10.045892337 9.123903313 2.250278927<br>C63 9.393205021 1.514907331 2.963258626<br>C64 10.391918414 0.396737948 2.730875627<br>C65 7.978892364 0.888599229 2.870503867<br>C66 6.878623459 1.892670767 3.119141268<br>C67 6.377358463 2.128102753 4.412425646<br>C68 6.321316213 2.626057263 2.055687762<br>C69 5.352986792 3.053902798 4.637918218

	C70	5.312646052	3.565712777	2.267207376
	C71	4.819154993	3.769732601	3.559227433
	C72	5.299554829	6.737065132	-1.751195165
	C73	4.250550016	7.846813253	-1.934765440
	C74	4.626329725	5.447478784	-1.290788718
	C75	3.179461580	9.402834066	-3.523858957
	C76	1.691879396	9.046168762	-3.556979778
	C77	0.045589096	7.185184714	-3.401776090
	C78	-0.351413845	6.880353223	-1.935156317
	C79	0.006130617	5.919807858	-4.289098079
	C80	-1.261752462	5.058191725	-4.308985263
	C81	-2.453666840	5.595113833	-5.117739856
	C82	-3.919943924	7.548440044	-4.577438986
	C83	0.271927582	5.997492493	0.260382147
	C84	0.205497726	7.250982417	1.145464680
	C85	1.274291422	5.021546090	0.906964778
	C86	1.391832748	9.308336662	1.741574036
	C87	0.723682543	10.644150272	1.459486166
	C88	-0.778787791	11.886635256	0.002791700
	C89	-1.994780908	12.481344806	0.756678234
	C90	-1.045560795	11.865463313	-1.505324521
	C91	-2.219846424	10.982959971	-1.904342983
	C92	-3.853521375	12.060354114	2.305883481
	C93	-3.692737650	13.170097843	3.335508333
	C94	-4.485118010	10.806183651	2.974855086
	C95	-4.800949041	9.715827458	1.975667891
	C96	-6.036325922	9.700296995	1.307848315
	C97	-3.860502559	8.723844234	1.646762896
	C98	-6.317355733	8.746310827	0.327561990
	C99	-4.112919591	7.776615196	0.649377614
	C100	-5.342697923	7.804655038	-0.003465381
	C101	3.447402828	-4.122832050	-9.046052417
	C102	2.046024879	-3.855269338	-8.526207546
	C103	0.662419276	-2.307175833	-7.166678244
	C104	0.147921386	-0.955959757	-7.675348796
	C105	0.580606951	1.461685614	-7.705936634
	C106	1.751913273	2.410821987	-7.943527565
	C107	4.072621326	3.030136364	-7.546359037
	C108	4.645045531	3.136170979	-8.946175876
	C109	-3.397513344	-3.604390589	-8.811677794
	C110	-4.394627551	-2.501884307	-8.432636914
	C111	-2.843030751	-4.275914724	-7.534020743
	C112	-2.167168103	-5.620613220	-7.864475086
	C113	-1.393058120	-6.136303406	-6.672885051
	C114	-4.734023856	-0.168072099	-7.782513250
	C115	-4.285321988	1.103429204	-8.476933434
	C116	-4.988863443	-0.016719297	-6.262302351
	C117	-3.696463024	-0.030791547	-5.494254767
	C118	-2.923300918	1.135665095	-5.364519877
	C119	-3.170012246	-1.249074344	-5.035165386
	C120	-1.622233016	1.064695182	-4.856676611
	C121	-1.869548193	-1.320246471	-4.529102125
	C122	-1.081527479	-0.167233542	-4.473147480
	C123	7.423306196	-6.390355867	-5.072268444
	C124	8.673201531	-6.488800878	-4.182235868
	C125	6.156719364	-6.732805116	-4.271550534
	C126	4.851497765	-6.487744648	-4.961158082
	C127	4.405110153	-7.072323755	-6.128990254
	C128	2.752432084	-5.916863434	-5.217948847
	C129	9.986892680	-7.733793381	-2.568567530
	C130	11.297752445	-7.492412482	-3.284051938
	C131	0.422479383	-6.949384236	-0.014768407
	C132	1.933931127	-7.037835826	0.187536437
	C133	2.765749232	-7.158684283	-1.123296472
	C134	2.617245860	-8.592914898	-1.700291075
	C135	3.583888058	-8.955600843	-2.830380710
	C136	3.560488274	-10.451490495	-3.216864547
	C137	4.252456110	-6.920148244	-0.810964729
	C138	3.346896004	-0.806718938	-2.275166731
	Fe139	3.479121623	-2.098755106	-3.778045678
	Fe140	1.907552156	0.616774095	-1.505939087
	Fe141	4.768809393	0.390516643	-1.558443318
	Fe142	3.480298486	0.432914048	-3.792793135
	Fe143	3.551174910	2.611454561	-2.235585760
	Fe144	4.657010410	-2.115877606	-1.556810091
	Fe145	2.083266596	-2.382962864	-1.440171051
	H146	-6.623013488	-7.470215433	0.860752772
	H147	-6.934723071	-6.496184071	-0.410324584
	H148	-9.752008639	-4.576952073	2.193710087
	H149	-9.151764356	-6.026745580	0.390971361

	H150	-8.859730720	-7.126204418	1.733781948
	H151	-7.782302953	-2.601333250	2.544497015
	H152	-8.466066555	-5.322398706	4.709078477
	H153	-9.319833421	-2.694885377	3.433647330
	H154	-7.457665415	-5.928446734	6.709758982
	H155	-5.815150516	-5.670843076	4.160071723
	H156	-7.245893994	-4.202547705	7.089109669
	H157	-2.527478064	-1.766675167	3.170750211
	H158	-4.823715237	-3.496609974	3.474772631
	H159	-3.581804491	-6.088216639	3.654448462
	H160	-3.904517249	0.304648572	7.174565279
	H161	-5.068716474	-1.935613383	5.749839571
	H162	-3.157116000	-6.046232564	5.383504706
	H163	-4.247142830	-1.306798391	3.105639754
	H164	-3.705417813	-3.272369102	7.764308130
	H165	-5.108780316	-0.852112931	7.802744404
	H166	-0.196655604	-3.155330794	3.052740967
	H167	-0.536426749	-2.030382455	1.722250516
	H168	1.129102732	-2.546812945	2.026160784
	H169	-0.595697785	-0.810667025	3.903713730
	H170	-2.631964665	-4.335833588	9.522109713
	H171	-0.824301617	2.155304085	4.828714290
	H172	-0.373826325	-0.039663867	8.734380321
	H173	-0.138816019	3.304198734	8.982546193
	H174	1.731690499	-0.092018399	2.034086291
	H175	1.188455858	0.973536838	3.349937862
	H176	9.536108035	1.878347772	3.998967329
	H177	-1.573488411	3.603985439	5.533762859
	H178	-1.159836716	4.269820517	7.893096197
	H179	0.173090570	3.588925229	5.190818043
	H180	0.606993040	4.257998647	7.678548781
	H181	-1.265221525	1.847521728	7.284910372
	H182	1.708873135	2.111077393	6.587186283
	H183	-0.297012242	0.051901132	5.984834546
	H184	0.072180135	0.543381990	2.051555565
	H185	1.505053313	4.218892189	0.191023122
	H186	0.774418984	4.577042574	1.780200947
	H187	2.793995680	5.295211641	2.134110668
	H188	3.531680668	4.733410939	4.645152914
	H189	4.879827176	4.114844307	1.430988205
	H190	6.664292220	2.439542904	1.035017275
	H191	6.777807147	1.566211329	5.259968863
	H192	-0.278323120	-2.562781683	5.517975637
	H193	0.513560301	-5.369059831	6.263576830
	H194	1.332906687	-4.788776953	7.731730842
	H195	-1.397738211	-4.051019492	6.831688848
	H196	-2.048514627	-2.769395354	10.128866582
	H197	4.958510937	3.203031546	5.646727576
	H198	2.259392823	-1.822208830	4.216112531
	H199	0.484481571	0.908010593	11.156296559
	H200	0.175040565	-0.817551821	10.821791018
	H201	2.756651752	-0.795338160	9.949284422
	H202	11.236524285	-7.229989903	3.532628360
	H203	-2.153199707	-6.042723408	-3.833177892
	H204	0.940342085	-6.117946067	-1.674084645
	H205	-2.938641538	-6.360332240	-8.119379335
	H206	-1.496983891	-5.504618831	-8.728859057
	H207	-3.656428966	-4.452613530	-6.813187465
	H208	-3.784078820	0.936604370	-9.458743415
	H209	-4.023351506	-4.349338846	-9.326221049
	H210	-3.627153938	-2.212555453	-2.005509407
	H211	-4.171307709	-4.956738504	-0.765863105
	H212	-2.856770504	-1.129608796	-8.181084523
	H213	-5.713994913	-0.391739707	-8.251483125
	H214	-1.694267645	-2.536798121	-9.343052747
	H215	-1.622272712	-5.874306717	-2.164122008
	H216	-2.133149209	-3.589411118	-7.047110341
	H217	-1.930471034	-3.916548122	-10.213656619
	H218	-7.055456539	-3.430041475	-2.302073737
	H219	-3.612449915	-2.389080963	-0.234130768
	H220	0.240699955	-5.399015185	-7.486752704
	H221	-2.525672629	-4.339303514	-0.755472008
	H222	-3.674933609	2.742480801	3.303971529
	H223	-2.984237585	0.619910097	1.610768196
	H224	-7.903561168	0.936587517	4.363704262
	H225	-7.239517177	0.021460667	1.598054131
	H226	-6.826993493	4.203462790	2.778596395
	H227	-8.022506629	8.922577374	4.782008072
	H228	-6.668605651	5.366113657	5.714626243
	H229	-8.765226873	5.597518028	3.921124772

H230	-6.114310589	6.955525470	3.134858066
H231	-10.746666115	9.670137905	1.624187616
H232	-3.206350231	4.334891349	1.460197023
H233	-4.808817682	4.057856879	0.759904000
H234	-9.559578030	10.633346018	3.671237960
H235	-8.272696451	5.765792988	2.234770250
H236	-6.139800481	2.134234971	1.779978349
H237	-6.822402152	-0.415714241	4.013784208
H238	-9.939129500	1.984841261	0.444789298
H239	-2.455902060	1.469701438	-2.522910562
H240	-10.255874346	0.245002902	0.585089780
H241	10.429025073	3.074508847	2.131539946
H242	8.807970006	3.207077445	1.972401974
H243	1.464628301	5.916901846	-1.536884986
H244	-0.726309669	5.538679935	0.299604898
H245	1.784672806	8.040286227	0.098771858
H246	-3.576631376	3.558250759	-1.412941644
H247	-5.299125396	4.174747076	-2.472395707
H248	-6.989794638	3.395867127	-3.711909882
H249	-4.907791198	5.437899922	-1.585068597
H250	-5.793457295	1.260607001	-3.642497897
H251	-7.564922019	1.285356570	-3.374141678
H252	-0.127176717	11.586112360	-2.038786952
H253	-1.302328175	12.895296522	-1.797177974
H254	-1.040485179	10.038456859	-3.317253324
H255	-2.704303064	9.480375644	-3.155066286
H256	-3.880511795	9.166862677	-5.836957867
H257	-2.997846778	4.760209548	-5.585319896
H258	-7.288870927	8.738745886	-0.173327272
H259	-2.901964290	8.669807620	2.171084683
H260	-4.856335648	7.739356297	-2.748239938
H261	0.833664821	5.268086290	-3.964156015
H262	2.175914733	7.069367343	-3.429482037
H263	-1.584611673	4.809945924	-3.285757593
H264	-8.513693668	-0.169489497	-0.872384014
H265	3.451669793	9.814242970	-4.504643325
H266	-6.368558386	-0.938447024	-2.590397064
H267	0.247903679	6.230811556	-5.318728153
H268	-3.342633819	7.059347718	0.362701666
H269	3.288879880	10.206861977	-2.782215302
H270	-3.165664996	7.731539059	-6.488863404
H271	-5.293686963	8.923263271	-3.935757710
H272	-5.530404113	-3.537771360	-2.980210550
H273	-5.925464301	-3.303527016	-0.301900982
H274	-6.797175089	10.442573112	1.556553681
H275	-5.407912810	1.405569582	-1.195150507
H276	-0.658379967	7.943241434	-3.755917511
H277	-2.085573047	6.225854066	-5.937163334
H278	-0.969009376	4.106011335	-4.773948183
H279	-3.800838295	5.836551927	-3.488885709
H280	1.107254499	9.002414743	2.760100653
H281	-0.399654188	9.767013414	-0.006258128
H282	0.013377813	12.630956340	0.178323168
H283	2.476553263	9.485299455	1.732506307
H284	-2.267550028	10.733710185	1.749096941
H285	3.968851390	-3.169948571	-9.219329302
H286	3.980938603	-4.632220089	-8.216373112
H287	7.378734836	-5.336259651	-5.383369523
H288	4.326728535	-5.256097118	-10.493908767
H289	2.791911642	-5.706228515	-10.144340121
H290	5.403273351	4.680656271	-1.176747469
H291	5.843848347	7.418786074	0.097105490
H292	4.649079616	7.910992943	-3.953092773
H293	4.125103656	5.609938172	-0.326674013
H294	6.834397347	7.939992923	-1.096874327
H295	4.879747362	2.684825976	-6.881911916
H296	5.811080671	6.554511429	-2.708726031
H297	5.426779180	-6.955410336	4.745121186
H298	7.064555958	-7.932080013	2.342960873
H299	3.125972382	1.234837312	-6.897572875
H300	5.242116647	-0.011767142	3.075073481
H301	0.001766517	1.413309381	-8.636146475
H302	-1.460766524	-2.276466135	-4.194847364
H303	-0.067070734	1.949700225	-6.957431211
H304	4.455971300	-4.451818552	4.312265312
H305	1.632836025	-0.077027560	-6.593858640
H306	4.067891778	-4.967976460	2.669956158
H307	4.848024514	-7.243365384	3.087107782
H308	6.515696837	-5.468888503	2.282513161
H309	6.846353360	-5.069042163	3.975879522

H310	-5.634147968	-0.849323679	-5.948707606
H311	6.055132922	-9.415106103	4.742983021
H312	5.070536280	-11.862078332	3.203722662
H313	-0.051214180	-0.208430928	-4.124025734
H314	6.586906692	-11.703370318	5.693262780
H315	5.016792489	-12.101631780	5.451288531
H316	5.708681097	-3.232332195	1.879240504
H317	-1.005183023	1.961755177	-4.769985875
H318	-3.775446416	-2.155946041	-5.092523310
H319	7.313241468	-10.090167635	-1.915421692
H320	6.188241008	-7.801669637	-3.994195861
H321	6.435149631	-8.308972356	-0.312387117
H322	6.360563308	-10.685645792	-2.991409460
H323	4.387507487	2.292507811	-9.631274241
H324	6.242785842	-5.206037591	-0.517892607
H325	2.153408852	-7.869890911	0.868154162
H326	2.724685203	-2.150153570	-7.617572752
H327	2.778959402	-9.284925559	-0.858637931
H328	2.447394228	-7.006936002	-6.994887339
H329	1.759310745	-5.515287091	-5.067993338
H330	6.722395415	-12.434884052	3.469022061
H331	5.494742989	-0.961820080	1.628939483
H332	8.283907777	-5.944514602	0.406298383
H333	7.187008685	-3.974388347	-0.565906679
H334	9.013441744	-6.950880498	2.409159522
H335	4.228841673	-2.707078061	5.123199695
H336	3.329600919	-8.376028089	-3.730610114
H337	4.613181398	-8.688373045	-2.554334535
H338	1.577244002	-8.730651518	-2.035339769
H339	0.838205323	-2.268721853	-6.079876437
H340	-3.333586284	2.089301032	-5.704866726
H341	-5.541055050	0.924101093	-6.117950006
H342	2.214854116	-6.107501510	0.709771084
H343	8.388876368	-7.486832887	0.236214841
H344	7.255938216	-9.378168180	0.443044953
H345	4.191358809	-1.022922864	4.927088656
H346	4.911172568	-7.721220916	-6.835278344
H347	6.168136405	-6.177404041	-3.326403286
H348	-0.097047016	-3.061406904	-7.381648143
H349	3.769313940	4.033927592	-7.211573102
H350	7.924392033	0.074665966	3.608592737
H351	7.860167355	0.442745722	1.869975908
H352	8.367301064	-6.905871123	-6.818137851
H353	7.702832333	-8.186321580	-6.045899410
H354	10.012178277	-8.763983015	-2.179008848
H355	8.154594995	-8.384638769	-3.442168429
H356	11.291073339	-7.723345359	-4.376197822
H357	9.912267686	-7.055344866	-1.702392356
H358	10.564896348	0.151587206	1.654982110
H359	-2.653090179	13.327185992	3.706715754
H360	10.395062839	-6.982063640	5.082743263
H361	9.803871203	-4.540642186	3.924243550
H362	-4.535509683	12.429900703	1.522051496
H363	-3.797727232	10.431842672	3.748957827
H364	-5.403568792	11.135584876	3.480671621
H365	1.038095909	-3.286136437	-0.804704779
H366	-6.533848238	6.860000412	-1.223282805
H367	-0.052624956	-0.985135210	-0.297028740
H368	2.132885139	-1.541199059	-0.182245853
H369	4.597270468	-2.954514383	0.009755707
H370	-2.170951142	-0.235104423	-0.672200810
Mo371	3.520203607	-4.486387136	-2.473760254
N372	-7.377154819	-7.132223567	0.257678918
N373	-8.781411337	-4.463854780	2.476795874
N374	-7.817188195	-4.648594740	5.109261859
N375	-5.142358702	-5.522902042	4.913757727
N376	-3.900100786	-3.226146376	3.804747348
N377	-4.233349721	-1.400466641	5.996215406
N378	-3.013305483	-2.715044398	8.273196537
N379	-0.582370837	-4.035688818	7.448070753
N380	0.738366251	-2.516587287	5.441150346
N381	0.658977060	0.371566797	6.155503575
N382	0.513725922	0.302968088	9.110897592
N383	5.753582396	-11.461002683	5.152384928
N384	6.424998339	-9.247001329	3.801414461
N385	5.224982789	-3.227452778	2.778366101
N386	4.648255442	-1.905257004	4.641685691
N387	5.201389833	-0.919720895	2.618573163
N388	9.132282877	-7.042354347	3.442085586
N389	-6.165429761	-2.941505551	-2.418011950

N390	-2.296689540	-5.642233746	-2.900025578
N391	-6.289595348	-0.421504185	-1.698705068
N392	-8.467487141	0.709421467	-0.361388280
N393	-7.769092274	0.472186462	2.351748663
N394	-5.577958075	2.201126980	2.633770125
N395	-3.240021747	2.698697624	-0.903688905
N396	-2.590642980	0.682259154	-0.513309125
N397	-6.187309224	4.849518956	3.235247984
N398	-9.667844013	7.861354559	2.087089263
N399	-9.439819124	9.680925849	3.336511373
N400	9.581559875	2.537615451	1.931444719
N401	6.319656817	7.120813732	-0.760889771
N402	4.085010087	8.311941624	-3.207892774
N403	1.395407870	7.731278627	-3.503667967
N404	-3.410303400	6.348839541	-4.300194972
N405	-3.698571354	8.172441070	-5.748212922
N406	-4.649716244	8.194895481	-3.640469311
N407	0.588128724	6.299341272	-1.137713378
N408	1.073987899	8.244259031	0.807458663
N409	-0.271357673	10.630278137	0.521089053
N410	-2.003916370	10.202863558	-2.998288582
N411	-2.570298939	11.703822571	1.708403242
N412	3.393467009	-4.890818972	-10.292042621
N413	1.892233983	-2.703623968	-7.839860245
N414	0.896681490	0.104022816	-7.298399177
N415	2.972920972	2.085043261	-7.448180323
N416	-2.411632078	-3.123304681	-9.785714065
N417	-3.871099074	-1.273696378	-8.153865847
N418	7.539991884	-7.205658805	-6.294470799
N419	3.799993581	-5.752253101	-4.408921468
N420	3.087503164	-6.699186153	-6.267650853
N421	8.842724116	-7.627539906	-3.457512069
N422	-0.039303083	2.270426936	-0.057604652
N423	0.719420207	1.630220814	-0.606210368
O424	-6.618668174	-5.077995204	2.125603712
O425	-6.839718979	-2.601441042	4.893111231
O426	-4.791334755	-4.791145477	7.047870282
O427	-2.006220851	-3.851804053	4.898525882
O428	-2.334055072	-0.382366201	5.255050579
O429	-2.289518365	-0.625745747	8.855365416
O430	0.201719151	-3.745983905	9.585935266
O431	2.702439484	-3.590300071	5.972668189
O432	2.866556518	0.151687764	5.609369163
O433	2.271382408	1.733042710	8.957991301
O434	2.767157399	-0.104932110	11.869846058
O435	7.024958460	-10.411643653	1.896934296
O436	8.181377540	-7.687832453	5.433888665
O437	11.831055464	-4.735569059	4.022165410
O438	-5.711296790	-0.806081921	0.487611706
O439	-4.089362663	-4.451719915	-3.637269114
O440	-7.215727719	2.568768771	0.072959740
O441	-6.573283462	3.060258053	-2.898059817
O442	-9.654120927	1.640263878	2.946437690
O443	-5.352854146	1.646476901	4.837907217
O444	-4.059660391	5.184073445	3.995483766
O445	-6.327295215	7.378718337	5.711539566
O446	10.955867516	-0.220007645	3.622454940
O447	3.789378484	4.679364710	3.706990392
O448	3.598379085	8.307912261	-0.981622738
O449	0.845210836	9.956815868	-3.645655725
O450	-1.492262055	7.164471235	-1.533143193
O451	-0.551458128	7.297961433	2.123949501
O452	2.471078027	5.697575639	1.302408488
O453	1.068554454	11.666658536	2.063541802
O454	-2.371943401	13.636842358	0.515882401
O455	-3.300200794	11.008006319	-1.297091159
O456	-4.633389278	13.826063271	3.754271619
O457	-5.573915194	6.878452469	-1.042763910
O458	1.121719010	-4.685331517	-8.707055374
O459	-0.869766994	-0.862421016	-8.392136325
O460	1.546528130	3.479305389	-8.542810107
O461	5.363478113	4.054751998	-9.303730778
O462	-5.613683660	-2.729080923	-8.363543001
O463	-1.911950942	-6.721341426	-5.727977012
O464	-0.068840907	-5.877313772	-6.656597591
O465	-4.483686890	2.232984555	-8.051885996
O466	9.528933046	-5.582969987	-4.155914634
O467	12.322174009	-7.146225549	-2.711832941
O468	0.033199466	-6.329916333	-1.137780070
O469	-0.384148384	-7.357089230	0.817384738

	O470 4.690502845 -11.022819259 -3.411524425<br>O471 2.429659134 -11.002280717 -3.341568747<br>O472 4.835362948 -5.959228518 -1.476728951<br>O473 4.843264705 -7.637894170 0.019510833<br>O474 2.299269888 -6.157574573 -2.031419600<br>O475 8.871534009 -6.708126821 0.629977024<br>O476 6.922745440 -4.714914693 0.012235699<br>O477 -4.537669669 4.756605826 -2.200720411<br>O478 7.331745164 -10.595700162 -2.758100545<br>O479 7.310465755 -8.778818466 -0.344404114<br>S480 3.356638341 4.857258467 -2.502571977<br>S481 1.752991563 1.855415041 -3.383733195<br>S482 1.678212184 -3.399082223 -3.472705676<br>S483 3.530131430 1.670182401 -0.213465815<br>S484 0.265194787 -1.043679164 -1.621304843<br>S485 3.578285390 -0.848734801 -5.600818690<br>S486 3.312365720 -3.649472374 -0.209868032<br>S487 5.335520034 1.658633956 -3.283288175<br>S488 5.375805301 -3.266566593 -3.278963794<br>S489 6.224234172 -0.946301354 -0.573727145<br>end

TS

| Fe(139) -2.402<br>Fe(140) -1.031<br>Fe(141) -2.581<br>Fe(142) 2.665<br>Fe(143) 2.697<br>Fe(144) 2.216<br>Fe(145) 0.088 | bm522bh2n2x6x263b1d_1_53094.41<br>C1 -8.252906043 -4.775415966 1.281926567<br>C2 -8.808591279 -5.873534470 0.368096548<br>C3 -8.777590062 -2.731577220 2.534330104<br>C4 -8.103021456 -3.018661022 3.871609188<br>C5 -7.799739275 -4.563757805 5.739332523<br>C6 -6.285591142 -4.764860869 5.644324827<br>C7 -4.368526771 -5.318966045 4.243238941<br>C8 -3.681766084 -3.954239889 4.159752314<br>C9 -3.844019904 -1.628808218 3.418768673<br>C10 -3.704255084 -0.962851382 4.795865340<br>C11 -4.670226214 -0.551725178 6.979329252<br>C12 -3.694444301 -1.229943897 7.949983615<br>C13 -3.006417858 -3.328637136 9.006409347<br>C14 -1.578775955 -3.758816768 8.645499703<br>C15 -0.084504392 -4.586506091 6.875683192<br>C16 0.823378386 -3.624218288 6.103893811<br>C17 0.900221268 -1.571221946 4.685349551<br>C18 1.282184236 -0.395714801 5.616407061<br>C19 0.056675905 -1.131167423 3.472729869<br>C20 0.662095656 0.088137888 2.760359407<br>C21 -0.131690681 -2.300715417 2.500606722<br>C22 0.456695390 1.435869072 7.061183667<br>C23 0.758137359 1.035599987 8.519775533<br>C24 -0.734314193 2.421450782 6.959608892<br>C25 -0.587198131 3.572043948 7.962851091<br>C26 -0.869618501 2.967494848 5.532540055<br>C27 0.037904811 -0.090599856 10.549325584<br>C28 1.446325665 -0.512669145 10.911439239<br>C29 4.994096384 -12.016429584 3.976930415<br>C30 5.650607886 -10.783156600 3.359882149<br>C31 6.076289548 -8.358393326 3.770138565<br>C32 7.311740345 -8.069776516 4.645746066<br>C33 4.986137720 -7.291634227 3.964190429<br>C34 5.455755325 -5.882448695 3.602899812<br>C35 4.293045824 -4.893379552 3.554023814<br>C36 4.596830371 -2.390568839 3.652563263<br>C37 9.662274167 -7.380526417 4.648292056<br>C38 10.046064228 -5.917090420 4.641040349<br>C39 -5.604039325 -2.285479485 -1.536856791<br>C40 -5.862495556 -0.832438999 -1.095795204<br>C41 -4.081442975 -2.545519329 -1.472052027<br>C42 -3.647961556 -4.022689193 -1.576939114<br>C43 -3.408571717 -4.534848640 -2.997576865<br>C44 -6.158081033 1.446027518 -1.996907269<br>C45 -7.232085689 1.979580649 -1.038484587<br>C46 -6.254625022 2.067270379 -3.397600705<br>C47 -9.419816275 1.590494676 -0.000058664<br>C48 -9.009741474 1.616169571 1.476681353<br>C49 -7.336437103 0.995820549 3.161411201<br>C50 -6.089042413 1.856435072 3.366371478<br>C51 -4.456617199 3.419932261 2.435267072<br>C52 -4.822652620 4.755593338 3.124256007<br>C53 -3.840438356 3.807322505 1.082631173<br>C54 -3.290042505 2.695660139 0.254544705<br>C55 -2.960765227 1.389697053 0.516328193<br>C56 -2.509324906 1.785034749 -1.631728574 |

	C57	-6.529872681	6.528738095	3.233315893
	C58	-6.481555628	6.667189785	4.754057702
	C59	-7.979714028	6.694434202	2.718360459
	C60	-8.488483607	8.099751331	2.590401660
	C61	-8.320828567	9.201390107	3.407712681
	C62	-9.594707827	9.715060297	1.668276679
	C63	9.237443170	0.886161326	3.642998440
	C64	10.173095894	-0.295929214	3.469416466
	C65	7.795959801	0.350781390	3.444125555
	C66	6.742858067	1.418990316	3.623062267
	C67	6.172090888	1.679091215	4.882530002
	C68	6.310855916	2.195569730	2.532649566
	C69	5.192759178	2.663261968	5.049943189
	C70	5.347085801	3.191025438	2.688587776
	C71	4.780941339	3.417418317	3.945623270
	C72	5.831301739	6.398993787	-1.271982490
	C73	4.867980541	7.571791802	-1.522979049
	C74	5.048933594	5.148612613	-0.878556268
	C75	4.016174841	9.203962938	-3.163982592
	C76	2.513712373	8.946989830	-3.299512920
	C77	0.745082721	7.196076915	-3.291618335
	C78	0.229047839	6.910509212	-1.856686485
	C79	0.684323549	5.938773785	-4.189495369
	C80	-0.635421673	5.166816096	-4.304296237
	C81	-1.727478061	5.786237473	-5.192982985
	C82	-3.097350004	7.826413092	-4.717779291
	C83	0.636482789	5.973245410	0.366545381
	C84	0.591561787	7.223854945	1.253823177
	C85	1.525431374	4.930778247	1.075860051
	C86	1.864685103	9.193959747	1.949918362
	C87	1.290661199	10.568662566	1.643087259
	C88	-0.032731828	11.907601252	0.102467813
	C89	-1.259734814	12.569658221	0.779712013
	C90	-0.200834503	11.923611069	-1.419768509
	C91	-1.393883233	11.114478970	-1.909436102
	C92	-3.240224268	12.253096808	2.197734755
	C93	-3.078913210	13.340835409	3.250974827
	C94	-3.992319100	11.033951365	2.804171422
	C95	-4.308837827	9.977225196	1.767730639
	C96	-5.494673550	10.044896529	1.019546071
	C97	-3.408363943	8.936408716	1.484021316
	C98	-5.769021470	9.117917645	0.010163416
	C99	-3.651129024	8.016289666	0.460001674
	C100	-4.832955692	8.121347012	-0.268141863
	C101	3.811184100	-4.242361548	-8.842117457
	C102	2.392561499	-3.894097000	-8.424715183
	C103	1.013705298	-2.279791319	-7.134880914
	C104	0.623532497	-0.890343566	-7.654849840
	C105	1.204566175	1.495099947	-7.620447621
	C106	2.443345473	2.380063352	-7.750503160
	C107	4.767621952	2.849582756	-7.191360592
	C108	5.472802327	2.895246402	-8.533840499
	C109	-2.988405463	-3.293759976	-9.066866162
	C110	-3.935779263	-2.135352293	-8.733943920
	C111	-2.565682901	-4.018724991	-7.769383130
	C112	-1.953780047	-5.399454804	-8.075626112
	C113	-1.295903912	-5.973989678	-6.842579886
	C114	-4.171989460	0.208618153	-8.083995141
	C115	-3.598043740	1.459804941	-8.719595506
	C116	-4.531369486	0.354285676	-6.584089511
	C117	-3.299784233	0.252517577	-5.727033226
	C118	-2.466816229	1.366353421	-5.520845173
	C119	-2.877122069	-1.004672313	-5.260538848
	C120	-1.206585156	1.205191761	-4.937742271
	C121	-1.618077383	-1.165115782	-4.678972088
	C122	-0.765260245	-0.063778834	-4.551933505
	C123	7.360320357	-6.804532227	-4.634379189
	C124	8.534242331	-6.997050454	-3.658707926
	C125	6.017728624	-7.091837757	-3.942797296
	C126	4.783373134	-6.754209908	-4.718667147
	C127	4.385057196	-7.296545946	-5.922757168
	C128	2.747345689	-6.043897717	-5.119221656
	C129	9.646478501	-8.331916443	-1.964972263
	C130	11.012434014	-8.173224161	-2.595023530
	C131	-0.011202011	-6.967962643	-0.114632681
	C132	1.471125822	-7.150696769	0.197840107
	C133	2.383287904	-7.323781556	-1.053665493
	C134	2.185607404	-8.742616436	-1.652448972
	C135	3.205791131	-9.165950336	-2.713782473
	C136	3.110435124	-10.655326747	-3.121243647

	C137	3.858876174	-7.189488413	-0.638326467
	C138	3.450959469	-0.999169933	-2.044798379
	Fe139	3.551089960	-2.281887729	-3.555363878
	Fe140	2.085735420	0.454410123	-1.315255868
	Fe141	4.936140375	0.075215296	-1.238652762
	Fe142	3.762659143	0.233914830	-3.526899329
	Fe143	3.893945612	2.372271442	-1.934307598
	Fe144	4.674465632	-2.419473962	-1.274413171
	Fe145	2.061997853	-2.451624008	-1.258963026
	H146	-7.148735804	-7.058214695	0.269160949
	H147	-7.296235993	-6.037251574	-0.994747052
	H148	-10.137448229	-3.957889401	1.441339791
	H149	-9.523567268	-5.416485017	-0.334625468
	H150	-9.402876702	-6.552437473	1.003230852
	H151	-8.068879382	-2.124006587	1.952369148
	H152	-9.093137382	-4.802717109	4.034003736
	H153	-9.668904939	-2.119247306	2.726272140
	H154	-8.258470352	-5.500570676	6.082400916
	H155	-6.428553442	-5.308541114	3.655885979
	H156	-7.970090243	-3.795754652	6.505520011
	H157	-2.852463426	-1.611915228	2.949914575
	H158	-5.261912683	-3.198609982	3.080741542
	H159	-4.191572392	-5.868192399	3.308777263
	H160	-4.361040143	0.497062692	6.884261853
	H161	-5.565891532	-1.654177584	5.356310484
	H162	-3.896274976	-5.870816229	5.064636033
	H163	-4.533621935	-1.043579613	2.794511939
	H164	-4.433280137	-3.091356936	7.430730483
	H165	-5.677239666	-0.580332609	7.416818300
	H166	-0.590882028	-3.174629053	2.983856440
	H167	-0.781899780	-2.003227884	1.662282584
	H168	0.829813658	-2.608646346	2.061802787
	H169	-0.926968234	-0.833158135	3.869984341
	H170	-3.574271457	-4.238195867	9.250256942
	H171	-1.030245997	2.166635385	4.797323362
	H172	-0.993255239	-0.086613318	8.688436519
	H173	-0.567555857	3.223935001	9.005618820
	H174	1.430464923	-0.211310985	2.032531704
	H175	1.122061485	0.808817011	3.451620254
	H176	9.331414680	1.234692578	4.689113431
	H177	-1.724021322	3.658276174	5.470105967
	H178	-1.433258898	4.266931589	7.855830614
	H179	0.037802790	3.523340078	5.246175663
	H180	0.340419175	4.137193678	7.778810743
	H181	-1.654348964	1.863161048	7.211533139
	H182	1.374042352	1.937445097	6.720544997
	H183	-0.709649860	0.024346789	5.940069180
	H184	-0.123023866	0.634437934	2.217719236
	H185	1.750353306	4.114958823	0.372065042
	H186	0.932786536	4.523563580	1.909210387
	H187	2.970626789	5.091412288	2.406113226
	H188	3.483043703	4.459968850	4.948477193
	H189	5.004794326	3.772839889	1.832471771
	H190	6.711038762	1.999115180	1.535045437
	H191	6.486077245	1.091679111	5.749780766
	H192	-0.812777097	-2.583126706	5.465476686
	H193	-0.265028105	-5.457457628	6.227117467
	H194	0.486834288	-4.925122038	7.748131427
	H195	-2.117287279	-3.998949783	6.657432379
	H196	-2.928921075	-2.721943986	9.916982586
	H197	4.745172012	2.832272872	6.033242980
	H198	1.846854107	-1.997855689	4.336749986
	H199	-0.253873703	0.765239649	11.180354676
	H200	-0.626033209	-0.936209698	10.790592032
	H201	2.004596239	-1.048601175	10.109902713
	H202	10.488735060	-7.976564149	4.230608285
	H203	-2.256318662	-5.852871362	-4.060889170
	H204	0.685266426	-6.153996692	-1.717974685
	H205	-2.751183431	-6.085785000	-8.393906362
	H206	-1.222386217	-5.317075459	-8.892510994
	H207	-3.434096810	-4.153245345	-7.106424177
	H208	-3.017268323	1.277157389	-9.654830286
	H209	-3.624180472	-3.988172372	-9.637096946
	H210	-3.594210383	-1.955685290	-2.266665465
	H211	-4.412414813	-4.674042938	-1.118603127
	H212	-2.334592894	-0.863018435	-8.375681877
	H213	-5.128771760	0.048638821	-8.622885744
	H214	-1.189499054	-2.331590756	-9.463973535
	H215	-1.831977544	-5.742477031	-2.357592819
	H216	-1.848160421	-3.385667237	-7.224495613

H217	-1.447790754	-3.685507336	-10.365886647
H218	-7.066696794	-2.931530195	-2.844200947
H219	-3.730352815	-2.159085335	-0.503357726
H220	0.430017608	-5.343343902	-7.544397542
H221	-2.734414542	-4.173872200	-0.983566448
H222	-3.712034600	2.936243926	3.083235599
H223	-3.020436034	0.810345345	1.428177741
H224	-8.117012679	1.408379084	3.816096159
H225	-7.318133792	0.468284391	1.093225263
H226	-6.726965835	4.576464255	2.377948225
H227	-7.766201102	9.353715528	4.326414450
H228	-6.717351748	5.720101915	5.298683121
H229	-8.655616805	6.088025480	3.347986823
H230	-5.877322020	7.294065222	2.784499427
H231	-10.214449090	10.312711900	1.005853859
H232	-3.032895771	4.527674715	1.291705400
H233	-4.599720694	4.338654311	0.486361797
H234	-9.110565625	11.171936366	3.142882912
H235	-8.021392284	6.253290167	1.710055945
H236	-6.099387231	2.501810623	1.385991910
H237	-7.113167400	-0.017115358	3.529221820
H238	-9.827387028	2.586089850	-0.223689238
H239	-2.182287634	1.642919834	-2.660319297
H240	-10.234789828	0.863851192	-0.119642484
H241	10.424557505	2.380787604	2.901147767
H242	8.831146365	2.615715792	2.629245160
H243	1.941495109	5.817271980	-1.346905335
H244	-0.389501766	5.579760570	0.335543125
H245	2.290177312	7.911842421	0.326543595
H246	-3.250304519	3.786687152	-1.610498412
H247	-4.838438873	4.526297113	-2.773922986
H248	-6.507175022	3.872216756	-4.125532506
H249	-4.424745722	5.749747175	-1.843343697
H250	-5.432222772	1.662489083	-4.010902386
H251	-7.213478577	1.791950331	-3.862768354
H252	0.734852198	11.602117927	-1.897650183
H253	-0.381851726	12.971940521	-1.703240947
H254	-0.160720368	10.079894947	-3.210244387
H255	-1.862205288	9.619221386	-3.173605761
H256	-2.863275003	9.466092513	-5.929421255
H257	-2.283198586	4.989692865	-5.710560424
H258	-6.703970448	9.173444088	-0.553444714
H259	-2.495445188	8.813876768	2.071944999
H260	-4.175010091	8.041272616	-2.967269603
H261	1.442100929	5.231785478	-3.814366945
H262	2.859997170	6.941522023	-3.182207724
H263	-1.049127409	4.941487927	-3.308629327
H264	-8.425550157	0.378405095	-1.467060971
H265	4.375533829	9.597307967	-4.124240940
H266	-6.203442744	-0.504944966	-3.030691118
H267	1.016065464	6.238719281	-5.197161090
H268	-2.909582975	7.254075814	0.219548579
H269	4.128294087	9.998589322	-2.412794588
H270	-2.189450497	7.999574728	-6.563469227
H271	-4.409718677	9.291079144	-4.148098415
H272	-5.506257103	-3.141348484	-3.407693315
H273	-6.084610136	-2.919917354	-0.768887744
H274	-6.223829200	10.831697082	1.228396591
H275	-5.213004646	1.768069626	-1.537243026
H276	0.116283388	7.998272647	-3.689705438
H277	-1.256909621	6.402141536	-5.970065562
H278	-0.377746736	4.197654025	-4.753675377
H279	-3.179419485	6.087879232	-3.667583727
H280	1.501291246	8.894180275	2.943910432
H281	0.223049050	9.769311404	0.092173577
H282	0.789558895	12.601804487	0.340001549
H283	2.955835202	9.308389426	2.008019340
H284	-1.695901865	10.838671420	1.745758943
H285	4.403550440	-3.323243531	-8.960198729
H286	4.245756761	-4.797942730	-7.985017092
H287	7.394706255	-5.742444616	-4.919014758
H288	4.723814936	-5.413759093	-10.238890960
H289	3.139471478	-5.764441985	-10.014213889
H290	5.770628057	4.337722339	-0.713982499
H291	6.265648259	7.018794799	0.625291265
H292	5.420298657	7.630816066	-3.505692663
H293	4.488217707	5.332966991	0.048048716
H294	7.370649630	7.508546637	-0.478989796
H295	5.489159293	2.471735774	-6.450181944
H296	6.404118625	6.190399687	-2.189183575

	H297	4.639309374	-7.322798060	5.010320688
	H298	6.368529107	-8.387721818	2.713071264
	H299	3.669860262	1.110116738	-6.636362858
	H300	4.959415145	-0.388114692	3.423656292
	H301	0.702267585	1.490257129	-8.596042004
	H302	-1.291605737	-2.149624033	-4.337076896
	H303	0.526711249	2.012685415	-6.919904624
	H304	3.825801168	-4.780232911	4.539331268
	H305	2.081131997	-0.119719475	-6.459700108
	H306	3.509782473	-5.265135826	2.873230084
	H307	4.137125085	-7.582419610	3.324438636
	H308	5.938416813	-5.891207951	2.614313113
	H309	6.204078135	-5.522461087	4.326909942
	H310	-5.247277773	-0.442227962	-6.335358598
	H311	5.113763399	-9.819445623	5.028659539
	H312	4.073187587	-12.191914627	3.399912764
	H313	0.236445718	-0.178285504	-4.140997922
	H314	5.425052882	-12.155873447	5.985803844
	H315	3.850310312	-12.437767897	5.636546985
	H316	5.136189428	-3.589751249	2.120267689
	H317	-0.541753502	2.058898961	-4.791764136
	H318	-3.531610691	-1.872235552	-5.375218990
	H319	6.774050240	-10.525332358	-1.544544056
	H320	5.969741425	-8.167411750	-3.699379660
	H321	5.909905805	-8.702741454	0.011226835
	H322	5.868076611	-11.059284671	-2.691626528
	H323	5.204552031	2.072998799	-9.240880430
	H324	5.937889019	-5.598434865	-0.201050448
	H325	1.593858986	-7.991202109	0.891245217
	H326	3.107750242	-2.252713320	-7.434256132
	H327	2.247372905	-9.442144310	-0.803882061
	H328	2.507962792	-7.098412321	-6.930179076
	H329	1.771934275	-5.581869809	-5.041747398
	H330	5.667119355	-12.865384081	3.768643088
	H331	5.265560793	-1.334330987	1.993609865
	H332	7.859649697	-6.469594083	0.884866105
	H333	6.891665735	-4.376842841	-0.101788042
	H334	8.373803816	-7.543214803	2.930175100
	H335	3.690128328	-3.036895956	5.372129497
	H336	3.059035915	-8.567539051	-3.626110629
	H337	4.228212538	-8.975041999	-2.360192130
	H338	1.165137897	-8.820633753	-2.057279824
	H339	1.113684740	-2.261060733	-6.037937150
	H340	-2.794122837	2.348217111	-5.869157316
	H341	-5.036738730	1.325212858	-6.466728940
	H342	1.772476760	-6.237243133	0.737625936
	H343	7.868950608	-8.013212204	0.713426654
	H344	6.595722236	-9.831983778	0.812626812
	H345	3.805660433	-1.355137233	5.220581425
	H346	4.896168019	-7.978032798	-6.591918715
	H347	5.992698796	-6.564299642	-2.982431372
	H348	0.223791509	-2.979698957	-7.415078596
	H349	4.496435716	3.875322921	-6.898479551
	H350	7.637745586	-0.461170357	4.169799638
	H351	7.723992984	-0.083440368	2.433713211
	H352	8.408536926	-7.325773964	-6.319327088
	H353	7.623016207	-8.588769449	-5.636755958
	H354	9.576026124	-9.363288633	-1.586233073
	H355	7.836311723	-8.852544382	-2.964281024
	H356	11.056184030	-8.423725557	-3.682311787
	H357	9.554886849	-7.654098393	-1.101169829
	H358	10.409564808	-0.540510483	2.404933591
	H359	-2.053727784	13.441707381	3.678489072
	H360	9.532178927	-7.674599295	5.705355059
	H361	9.194113865	-5.203057987	4.526313215
	H362	-3.845753158	12.674003550	1.377668793
	H363	-3.379069853	10.604437249	3.612240465
	H364	-4.918825796	11.411194494	3.260933923
	H365	0.940359560	-3.248427575	-0.638960810
	H366	-5.996500518	7.256345855	-1.570178345
	H367	-0.123056835	-0.955365601	-0.342693881
	H368	2.066318137	-1.491756274	-0.047141851
	H369	3.504309578	-2.446961417	0.038630872
	H370	-2.114803398	-0.084391499	-0.805128665
	Mo371	3.416566034	-4.684155720	-2.294479682
	N372	-7.830973997	-6.649400650	-0.374203185
	N373	-9.183400394	-3.913173707	1.790809915
	N374	-8.422994189	-4.182011162	4.481965494
	N375	-5.803564632	-5.216620821	4.458568996
	N376	-4.346779024	-2.992296863	3.474204460

N377	-4.718134731	-1.174137121	5.666366570
N378	-3.746356590	-2.586305115	7.997614318
N379	-1.352531997	-4.050447794	7.333791411
N380	0.206274219	-2.611440011	5.447403396
N381	0.249437910	0.273657218	6.191791269
N382	-0.113152045	0.193193715	9.129385739
N383	4.648948546	-11.848807459	5.395333646
N384	5.547359996	-9.669970677	4.111550381
N385	4.731097419	-3.584880530	3.057713098
N386	4.205709639	-2.264133082	4.936760488
N387	4.861866749	-1.277972445	2.940589437
N388	8.410782725	-7.652167232	3.967858838
N389	-6.138186531	-2.506420983	-2.885470672
N390	-2.439157491	-5.457904308	-3.132669684
N391	-6.160016905	-0.000657503	-2.128236075
N392	-8.362802635	1.244668441	-0.936911557
N393	-7.871681807	0.945607582	1.812986529
N394	-5.597440100	2.526217979	2.278749328
N395	-2.998055745	2.905877133	-1.086276151
N396	-2.491538434	0.854333965	-0.668618912
N397	-6.077456307	5.197612267	2.854340496
N398	-9.284127155	8.434369152	1.505928271
N399	-9.028912670	10.218119033	2.801146515
N400	9.562065634	1.898836805	2.635678910
N401	6.790711665	6.712000503	-0.200162695
N402	4.825360365	8.055529659	-2.799018674
N403	2.130611583	7.653166355	-3.295805153
N404	-2.697533619	6.585975663	-4.437101883
N405	-2.725746230	8.464681733	-5.840837023
N406	-3.863193359	8.496100960	-3.828733884
N407	1.068977293	6.258399694	-1.004255126
N408	1.545563025	8.158485251	0.984810020
N409	0.363728141	10.619588672	0.637523202
N410	-1.135543596	10.302864967	-2.971231108
N411	-1.947219182	11.821644482	1.678634647
N412	3.803459256	-4.989540168	-10.102656050
N413	2.259826831	-2.748272777	-7.724512889
N414	1.406589419	0.115990461	-7.209802949
N415	3.607259786	1.976076091	-7.180322875
N416	-1.908944167	-2.866424772	-9.964913790
N417	-3.355640577	-0.943896080	-8.413913397
N418	7.528906409	-7.594776623	-5.868464322
N419	3.742220394	-5.957668885	-4.234050856
N420	3.108144410	-6.837728423	-6.152106767
N421	8.574027339	-8.144816831	-2.928159877
N422	0.151585660	2.252458279	-0.022803761
N423	0.903106293	1.544140120	-0.488296686
O424	-7.050362348	-4.679172563	1.582117315
O425	-7.299787019	-2.203379911	4.360907965
O426	-5.558200427	-4.544062480	6.626639052
O427	-2.579662087	-3.742296405	4.692602991
O428	-2.715600554	-0.264375710	5.072575607
O429	-2.940045177	-0.557944702	8.674145728
O430	-0.728421840	-3.876137782	9.536804634
O431	2.050699303	-3.829786846	6.086272112
O432	2.474276864	-0.092312105	5.812381601
O433	1.750857497	1.489792504	9.114038600
O434	1.916612527	-0.356296640	12.028897473
O435	6.191986190	-10.861245699	2.238223698
O436	7.266854215	-8.220278729	5.879600667
O437	11.194299962	-5.519891621	4.765998574
O438	-5.776446935	-0.452432568	0.089143424
O439	-4.090884117	-4.135648416	-3.969173090
O440	-7.034873315	3.016706547	-0.380524804
O441	-6.150621699	3.501041099	-3.298780643
O442	-9.720984129	2.218929887	2.292084175
O443	-5.564809377	1.923973474	4.482920423
O444	-3.993814504	5.403589048	3.768494682
O445	-6.252662293	7.707412035	5.345522764
O446	10.625759241	-0.956748300	4.393246871
O447	3.800414666	4.388797504	4.029904084
O448	4.172666887	8.064104195	-0.616584816
O449	1.733233392	9.910756509	-3.427249482
O450	-0.911644548	7.275629158	-1.527547679
O451	-0.223682051	7.321852682	2.180731280
O452	2.733760789	5.523047473	1.560060057
O453	1.652881432	11.564268351	2.280580932
O454	-1.549480331	13.747244222	0.528083374
O455	-2.518883348	11.220420207	-1.401385751
O456	-4.004634658	14.042044279	3.627059782

	O457 -5.050259735 7.217086702 -1.329815985<br>O458 1.437592798 -4.662653801 -8.695955056<br>O459 -0.333376576 -0.725873015 -8.440223598<br>O460 2.333662602 3.467793880 -8.339648610<br>O461 6.303032437 3.741673747 -8.822079315<br>O462 -5.168715863 -2.290229175 -8.733527476<br>O463 -1.916758860 -6.519452528 -5.937073884<br>O464 0.039289935 -5.807716648 -6.741451743<br>O465 -3.778657488 2.594249121 -8.299779133<br>O466 9.451447700 -6.157246561 -3.574184493<br>O467 12.019323892 -7.871621890 -1.969689095<br>O468 -0.274881633 -6.313957103 -1.253484485<br>O469 -0.905222080 -7.339164186 0.642706690<br>O470 4.211128989 -11.300861850 -3.228588288<br>O471 1.958887854 -11.120904894 -3.353051736<br>O472 4.547382068 -6.259060020 -1.247248098<br>O473 4.344225477 -7.950451777 0.218442827<br>O474 2.053019428 -6.283960725 -1.975991809<br>O475 8.370772819 -7.272716905 1.153036063<br>O476 6.607929097 -5.170400098 0.389802543<br>O477 -4.059056846 5.047910778 -2.438460525<br>O478 6.824546243 -11.020881365 -2.392090745<br>O479 6.752360531 -9.229595921 0.041571483<br>S480 3.841730331 4.628266472 -2.182216561<br>S481 2.109691141 1.746609342 -3.175521454<br>S482 1.672062602 -3.487664712 -3.291727104<br>S483 3.709419448 1.410949287 0.064550106<br>S484 0.322316838 -1.070326600 -1.624526496<br>S485 3.865286992 -1.022793473 -5.352319084<br>S486 3.209116972 -3.963581650 -0.011041326<br>S487 5.666516571 1.325973532 -2.915547786<br>S488 5.354600073 -3.564635454 -3.032376001<br>S489 6.240029394 -1.371105740 -0.176888546<br>end

product

	bm522bh2n2x6x263b1a.car_4
Fe(139) -2.458<br>Fe(140) -1.064<br>Fe(141) -2.606<br>Fe(142) 2.696<br>Fe(143) 2.737<br>Fe(144) 2.310<br>Fe(145) 0.076	C1 -7.947905497 -5.126118074 1.800643081<br>C2 -8.492810748 -6.249132152 0.911721492<br>C3 -8.499347023 -3.111281690 3.084258523<br>C4 -7.731473394 -3.364140112 4.378202716<br>C5 -7.243532328 -4.897801012 6.217938178<br>C6 -5.729541227 -5.030559847 6.031851349<br>C7 -3.878469066 -5.514373653 4.516206561<br>C8 -3.255926240 -4.123228830 4.386067626<br>C9 -3.562759893 -1.810562690 3.654227475<br>C10 -3.365369919 -1.136997800 5.020194110<br>C11 -4.215189794 -0.746346113 7.255300748<br>C12 -3.149193037 -1.377692078 8.160031683<br>C13 -2.303612328 -3.438369874 9.175848388<br>C14 -0.880618080 -3.792691492 8.724572962<br>C15 0.535435436 -4.566325485 6.866746450<br>C16 1.339271807 -3.552793756 6.048968027<br>C17 1.234627706 -1.513070131 4.631068369<br>C18 1.630355601 -0.318976445 5.528664772<br>C19 0.318047820 -1.104224470 3.458404023<br>C20 0.875732787 0.111258101 2.708458381<br>C21 0.090043523 -2.298406416 2.526530995<br>C22 0.820229326 1.473226318 7.031261407<br>C23 1.223003402 1.096335233 8.472266845<br>C24 -0.418981448 2.402147206 6.991213365<br>C25 -0.268095075 3.565913784 7.977798366<br>C26 -0.661808716 2.930333627 5.571360132<br>C27 0.684984138 -0.058812154 10.540621956<br>C28 2.127022437 -0.438773924 10.807103974<br>C29 5.753001722 -11.786078071 3.658716820<br>C30 6.323975896 -10.523180350 3.012585720<br>C31 6.665068151 -8.083426833 3.399563802<br>C32 7.940076162 -7.749779793 4.198260052<br>C33 5.553093358 -7.050137041 3.654370096<br>C34 5.958094398 -5.623342606 3.278777378<br>C35 4.762096185 -4.673222824 3.302323963<br>C36 4.934019971 -2.156116037 3.357035026<br>C37 10.249152107 -6.942907195 4.045987232<br>C38 10.567197948 -5.464858988 4.028022187<br>C39 -5.589650052 -2.533448641 -1.195268119<br>C40 -5.876918387 -1.087745222 -0.748953053<br>C41 -4.055309645 -2.723726274 -1.232675966<br>C42 -3.564414393 -4.181653303 -1.352930169<br>C43 -3.378644094 -4.692678006 -2.781668129

	C44	-6.323674402	1.170926849	-1.632354732
	C45	-7.352879889	1.667243373	-0.606810616
	C46	-6.529339400	1.784050926	-3.023367515
	C47	-9.451075754	1.203434362	0.569741326
	C48	-8.959251266	1.233646780	2.021563730
	C49	-7.158890248	0.685995200	3.597782789
	C50	-5.944509571	1.605779351	3.720458868
	C51	-4.446421413	3.237899487	2.692061467
	C52	-4.822095653	4.555670647	3.411587758
	C53	-3.922504125	3.644440299	1.307062009
	C54	-3.389109792	2.549022520	0.445635918
	C55	-3.016854505	1.248545238	0.677423160
	C56	-2.688920947	1.671474172	-1.487420852
	C57	-6.590882057	6.262656515	3.640257884
	C58	-6.441436309	6.412889641	5.153536245
	C59	-8.076500003	6.360267515	3.223291956
	C60	-8.651944195	7.742024659	3.120669970
	C61	-8.477899241	8.855250424	3.920887297
	C62	-9.877812536	9.305610960	2.263098174
	C63	9.404307998	1.300381234	3.065743842
	C64	10.381219127	0.163516075	2.830339868
	C65	7.978979917	0.700671156	2.948813657
	C66	6.892678747	1.721930041	3.192226140
	C67	6.373213852	1.946731701	4.480061723
	C68	6.369727557	2.483981556	2.130246525
	C69	5.363542513	2.889295992	4.702697684
	C70	5.376722530	3.439693738	2.340025597
	C71	4.865634672	3.633775032	3.626501729
	C72	5.454939070	6.643732357	-1.647258895
	C73	4.427878499	7.775706431	-1.823195671
	C74	4.749808043	5.362694453	-1.209579821
	C75	3.393854970	9.369670358	-3.400876150
	C76	1.900589558	9.041413969	-3.469662381
	C77	0.219663596	7.208212345	-3.331033234
	C78	-0.196051497	6.891812267	-1.871880921
	C79	0.165389872	5.955451952	-4.235437217
	C80	-1.117409993	5.116579705	-4.276271735
	C81	-2.293312333	5.684883426	-5.087196924
	C82	-3.720201058	7.663515972	-4.540339774
	C83	0.393884651	5.978259890	0.320017124
	C84	0.341413429	7.223735570	1.216669547
	C85	1.373484142	4.979307944	0.968227060
	C86	1.560357925	9.252131241	1.846310349
	C87	0.922896560	10.605850111	1.576595332
	C88	-0.534233746	11.898474485	0.117075470
	C89	-1.743782010	12.514096175	0.864644403
	C90	-0.783225943	11.895500377	-1.394515169
	C91	-1.976247155	11.050330404	-1.817703445
	C92	-3.629254114	12.114543951	2.387316918
	C93	-3.453860175	13.208370009	3.431424242
	C94	-4.293024909	10.866829596	3.036919640
	C95	-4.621026079	9.792047868	2.024046550
	C96	-5.848506837	9.810244205	1.342400160
	C97	-3.699159426	8.781943632	1.698129914
	C98	-6.139481542	8.869808732	0.351550040
	C99	-3.960375756	7.849257814	0.689525118
	C100	-5.181601178	7.909494829	0.023792095
	C101	3.446043601	-4.101101739	-9.044216722
	C102	2.044724916	-3.815187371	-8.535807651
	C103	0.676237448	-2.249282389	-7.179033274
	C104	0.189865602	-0.884793930	-7.680325899
	C105	0.676216743	1.523262849	-7.694021951
	C106	1.877109488	2.438297110	-7.916132850
	C107	4.199653826	3.005686666	-7.474998433
	C108	4.768750842	3.131434791	-8.875186143
	C109	-3.379490240	-3.450098028	-8.869412311
	C110	-4.355366672	-2.329783931	-8.488378915
	C111	-2.852891476	-4.145270428	-7.593087657
	C112	-2.206354869	-5.503307019	-7.928764040
	C113	-1.458717668	-6.046013643	-6.732745371
	C114	-4.651392680	0.004291684	-7.825469822
	C115	-4.178387054	1.273524807	-8.507248013
	C116	-4.917512957	0.146938601	-6.307223929
	C117	-3.635828170	0.100286781	-5.523453104
	C118	-2.838464086	1.248894864	-5.376369744
	C119	-3.146785447	-1.131161023	-5.056459130
	C120	-1.552077134	1.146214720	-4.839411592
	C121	-1.860926596	-1.232614084	-4.519115151
	C122	-1.049902233	-0.096535352	-4.442499077
	C123	7.363977361	-6.514894802	-5.111653929

C124	8.596279010	-6.635988738	-4.199528540
C125	6.086216324	-6.843418394	-4.325893626
C126	4.785392707	-6.558258921	-5.006645889
C127	4.318726526	-7.097483157	-6.187220331
C128	2.693462531	-5.941197567	-5.227201150
C129	9.854348593	-7.912636212	-2.564347305
C130	11.181785776	-7.684539618	-3.252941303
C131	0.317024285	-6.934657225	-0.083610482
C132	1.824960191	-7.063772919	0.131417720
C133	2.656214135	-7.221775571	-1.177681897
C134	2.470871069	-8.648004347	-1.759782818
C135	3.432820380	-9.028524314	-2.890436404
C136	3.368838919	-10.518599085	-3.292652811
C137	4.151580644	-7.032981792	-0.871071422
C138	3.354576544	-0.855208533	-2.239261817
Fe139	3.423188003	-2.157855975	-3.756155707
Fe140	1.940337883	0.630050003	-1.565376214
Fe141	4.822322619	0.279220825	-1.529281493
Fe142	3.543758110	0.358958491	-3.763247195
Fe143	3.667006004	2.543342815	-2.220831824
Fe144	4.659204284	-2.238518824	-1.544893212
Fe145	2.053801003	-2.464943819	-1.264983256
H146	-6.783223809	-7.350864312	0.743084523
H147	-7.035152779	-6.346856819	-0.518070307
H148	-9.858913988	-4.407294697	2.070900123
H149	-9.254536785	-5.822053044	0.239598173
H150	-9.029076636	-6.954764836	1.568680858
H151	-7.856928247	-2.472108200	2.460423480
H152	-8.614574149	-5.197809130	4.586319045
H153	-9.406389795	-2.543179097	3.330144340
H154	-7.640696397	-5.854342588	6.582234700
H155	-5.970144746	-5.605771585	4.063544622
H156	-7.400533844	-4.140193527	6.997091770
H157	-2.599566152	-1.754833567	3.131778334
H158	-4.928260189	-3.443429127	3.400844164
H159	-3.736686724	-6.061547138	3.574493025
H160	-3.957692655	0.314423811	7.141762526
H161	-5.171803207	-1.876486364	5.687835625
H162	-3.331994642	-6.039599502	5.308392942
H163	-4.310441665	-1.260641060	3.065109566
H164	-3.842313217	-3.270865319	7.698235750
H165	-5.191204856	-0.819340200	7.754979019
H166	-0.358844795	-3.151281228	3.053975805
H167	-0.588531403	-2.025781837	1.702176697
H168	1.033313714	-2.640230287	2.074692091
H169	-0.649239314	-0.807659654	3.896063407
H170	-2.806378556	-4.377768107	9.448607076
H171	-0.819346415	2.118530433	4.847349089
H172	-0.456156222	-0.112140970	8.745622380
H173	-0.154890366	3.223658896	9.016344489
H174	1.817024168	-0.120133508	2.186547363
H175	1.069517491	0.959578846	3.381949355
H176	9.543739766	1.651378777	4.105706519
H177	-1.554107993	3.573716060	5.553901499
H178	-1.155788252	4.213241377	7.929804314
H179	0.195299013	3.534946810	5.231875026
H180	0.612330496	4.178010666	7.725655877
H181	-1.295245775	1.801310292	7.296194032
H182	1.691335935	2.012908029	6.633911673
H183	-0.348315695	-0.010215359	6.012248877
H184	0.145655303	0.457973060	1.960946898
H185	1.599839043	4.179486756	0.247305559
H186	0.854095749	4.535873701	1.831045237
H187	2.889590567	5.207967147	2.208236653
H188	3.563577088	4.593909387	4.701449941
H189	4.971224460	4.011541860	1.505355536
H190	6.726090506	2.305878632	1.112777383
H191	6.750737273	1.364688095	5.325472396
H192	-0.389235059	-2.603020938	5.504456665
H193	0.360320172	-5.441338468	6.222022354
H194	1.174353176	-4.881380637	7.700568594
H195	-1.538447196	-4.097026288	6.781299534
H196	-2.199338097	-2.831124683	10.083274218
H197	4.956229548	3.032894200	5.707036861
H198	2.175154438	-1.909775833	4.231636949
H199	0.408291845	0.791853682	11.185428930
H200	0.066577212	-0.924014475	10.827851672
H201	2.642994969	-0.953947482	9.963837996
H202	11.065342049	-7.497619831	3.558036567
H203	-2.220906239	-5.956220979	-3.902840286

	H204	0.886178040	-6.110882899	-1.728961042
	H205	-2.993166936	-6.221569967	-8.200038944
	H206	-1.524013252	-5.395466899	-8.784946547
	H207	-3.676545549	-4.308313631	-6.880725431
	H208	-3.676378728	1.106287009	-9.488915503
	H209	-4.016152832	-4.177896447	-9.395990052
	H210	-3.653827508	-2.126097241	-2.068438751
	H211	-4.280135574	-4.860796321	-0.858567374
	H212	-2.789889778	-0.991293025	-8.219916294
	H213	-5.632407372	-0.199476231	-8.302519206
	H214	-1.647608272	-2.419106406	-9.379528262
	H215	-1.700563380	-5.804915323	-2.228847883
	H216	-2.130157109	-3.481513044	-7.093471635
	H217	-1.912012314	-3.784037011	-10.266625001
	H218	-7.101037956	-3.263489724	-2.398524250
	H219	-3.652046009	-2.308897417	-0.296224673
	H220	0.198902671	-5.329923618	-7.516630694
	H221	-2.618006320	-4.292027811	-0.804198343
	H222	-3.647679391	2.783160276	3.294686295
	H223	-3.021591076	0.656997290	1.583966983
	H224	-7.915362197	1.056041062	4.303479637
	H225	-7.249045412	0.158257588	1.532314208
	H226	-6.750047316	4.327519909	2.745815799
	H227	-7.867575185	9.034888971	4.798241253
	H228	-6.597877421	5.459949018	5.716584411
	H229	-8.681535134	5.730620431	3.899861399
	H230	-6.000014960	7.051082432	3.147950002
	H231	-10.560914705	9.874172240	1.637744446
	H232	-3.127170178	4.389794526	1.469232362
	H233	-4.729674349	4.152259886	0.755160927
	H234	-9.361926169	10.789983580	3.699643840
	H235	-8.167284638	5.911044603	2.221284937
	H236	-6.112951787	2.250851438	1.745204187
	H237	-6.859993547	-0.314592228	3.944636694
	H238	-9.891450135	2.191590518	0.375891234
	H239	-2.410690981	1.543724228	-2.531914596
	H240	-10.255369475	0.460440629	0.491333737
	H241	10.477656921	2.847607606	2.257131966
	H242	8.861239271	3.010565156	2.081104975
	H243	1.599568184	5.893524702	-1.469147043
	H244	-0.612129030	5.535809635	0.345974458
	H245	1.941523676	7.997377068	0.191033336
	H246	-3.483605430	3.648313530	-1.411111336
	H247	-5.188142380	4.303066307	-2.478867038
	H248	-6.876815025	3.572654421	-3.749307016
	H249	-4.778088410	5.549578277	-1.576203711
	H250	-5.728375387	1.413081193	-3.683683713
	H251	-7.501046080	1.468267895	-3.432587289
	H252	0.134892982	11.594938294	-1.917988558
	H253	-1.005250656	12.935044069	-1.682080107
	H254	-0.809920353	10.097700560	-3.242198704
	H255	-2.493486583	9.583566251	-3.099445370
	H256	-3.652490643	9.286966938	-5.794615795
	H257	-2.851208994	4.864948546	-5.564710494
	H258	-7.106300907	8.886631699	-0.158908362
	H259	-2.749918569	8.702012207	2.234578102
	H260	-4.666165253	7.859298282	-2.717749172
	H261	0.978402284	5.285868754	-3.911046197
	H262	2.346880494	7.057376865	-3.325741605
	H263	-1.452113562	4.861550840	-3.258560545
	H264	-8.504978712	0.016813088	-0.949368570
	H265	3.690975243	9.797441537	-4.367445394
	H266	-6.363659318	-0.782178194	-2.659452719
	H267	0.422420220	6.275793373	-5.258576367
	H268	-3.203276001	7.117564823	0.407086698
	H269	3.502641356	10.154654127	-2.639280712
	H270	-2.963444488	7.840061883	-6.452614656
	H271	-5.074616623	9.057417023	-3.899723650
	H272	-5.573884813	-3.402295072	-3.063021492
	H273	-5.985885659	-3.178021517	-0.388804836
	H274	-6.595383811	10.567489695	1.588632060
	H275	-5.364127987	1.525711515	-1.231111555
	H276	-0.467610778	7.983521469	-3.681166642
	H277	-1.906080392	6.314389965	-5.898413119
	H278	-0.838104299	4.165668569	-4.751448740
	H279	-3.645326350	5.940807549	-3.464655257
	H280	1.262123702	8.938561582	2.858012998
	H281	-0.203767641	9.771430522	0.089651044
	H282	0.274254003	12.621751651	0.308172412
	H283	2.647887097	9.408996433	1.847133543

H284	-2.068541713	10.759705854	1.828407952
H285	3.985562772	-3.156708101	-9.207024144
H286	3.961968552	-4.626523118	-8.213385883
H287	7.339271151	-5.459042126	-5.419663085
H288	4.318828287	-5.239077416	-10.493442496
H289	2.773533447	-5.666052002	-10.158703851
H290	5.510630541	4.581936305	-1.082472263
H291	5.999882174	7.302756371	0.210171541
H292	4.833532436	7.852530443	-3.838477639
H293	4.232347280	5.528815977	-0.254739425
H294	7.011475064	7.805724208	-0.973758985
H295	5.003015255	2.626501468	-6.824445925
H296	5.968952985	6.462033744	-2.603671122
H297	5.256770697	-7.097743062	4.715772418
H298	6.893614853	-8.103941802	2.326403603
H299	3.203591667	1.230575898	-6.844018483
H300	5.172185603	-0.143524265	3.078634948
H301	0.107511402	1.492537648	-8.631428853
H302	-1.482745601	-2.198726737	-4.177498644
H303	0.034921440	2.028166276	-6.950991120
H304	4.359465298	-4.573497075	4.316692767
H305	1.676672181	-0.044405908	-6.575022414
H306	3.948174643	-5.072673785	2.674508106
H307	4.685194309	-7.363347844	3.051749379
H308	6.388521040	-5.613858485	2.266364410
H309	6.731172522	-5.244356493	3.965287021
H310	-5.583921903	-0.674242275	-6.006535933
H311	5.818515711	-9.579655615	4.703030678
H312	4.808040829	-11.992730061	3.133772319
H313	-0.031501509	-0.160016852	-4.065199114
H314	6.301547058	-11.898591630	5.640221770
H315	4.727356031	-12.263041869	5.376832094
H316	5.460644537	-3.343377437	1.813357601
H317	-0.914478496	2.026735619	-4.735492940
H318	-3.770474523	-2.025447572	-5.127803453
H319	7.121312375	-10.218166361	-1.956285181
H320	6.092094380	-7.917874134	-4.071156869
H321	6.286977765	-8.435919526	-0.340819629
H322	6.163661875	-10.800942525	-3.037514902
H323	4.520406262	2.290878181	-9.567550558
H324	6.178894145	-5.352564449	-0.584730884
H325	2.017331635	-7.890564927	0.826575902
H326	2.739604405	-2.120166514	-7.624926138
H327	2.610295459	-9.349435084	-0.922374547
H328	2.354056185	-6.976343202	-7.030934865
H329	1.708617983	-5.521400005	-5.063962610
H330	6.444095297	-12.609242181	3.409430669
H331	5.425945178	-1.094106666	1.638780125
H332	8.184975466	-6.113816627	0.396043716
H333	7.067069812	-4.077923970	-0.562858855
H334	8.855322816	-7.154337388	2.421686309
H335	4.178585601	-2.826942862	5.139449645
H336	3.198152147	-8.433762349	-3.786397411
H337	4.468726045	-8.795899807	-2.607001463
H338	1.429223494	-8.752542720	-2.100104270
H339	0.851931914	-2.218759422	-6.091747933
H340	-3.218557009	2.212314006	-5.722996805
H341	-5.451596780	1.097570910	-6.158795609
H342	2.133068961	-6.128039174	0.629632238
H343	8.246853146	-7.659074920	0.240485618
H344	7.083205955	-9.529113477	0.409074513
H345	4.154977422	-1.143745133	4.949159705
H346	4.802148873	-7.749116190	-6.905960780
H347	6.109720637	-6.313391425	-3.366716489
H348	-0.096895545	-2.988365502	-7.397981362
H349	3.922495981	4.008193267	-7.116278256
H350	7.898159143	-0.118402068	3.679141570
H351	7.865142998	0.266445563	1.942199482
H352	8.336873526	-7.048938708	-6.833261366
H353	7.615771189	-8.313993452	-6.087442611
H354	9.857405261	-8.948137582	-2.189501046
H355	8.028654687	-8.523129460	-3.477370847
H356	11.185774205	-7.896527342	-4.349150521
H357	9.771450084	-7.245235614	-1.691301780
H358	10.543783839	-0.086199577	1.753681758
H359	-2.415429210	13.339502438	3.815524532
H360	10.216551610	-7.253412957	5.105252146
H361	9.679609807	-4.787746587	3.980654580
H362	-4.298022450	12.509548974	1.604847761
H363	-3.620830103	10.469533813	3.813547600

	H364	-5.209782680	11.209473183	3.537531381
	H365	1.031317037	-3.361011281	-0.599296305
	H366	-6.379149577	7.001581966	-1.218309580
	H367	-0.126129658	-0.886505558	-0.331706687
	H368	1.950143299	-1.459235786	0.079359090
	H369	2.617605776	-1.893927970	0.206508339
	H370	-2.221132342	-0.199284458	-0.700584503
	Mo371	3.505536712	-4.556591808	-2.471869739
	N372	-7.512283090	-6.980633990	0.127954968
	N373	-8.889880482	-4.313848985	2.365512383
	N374	-7.957700081	-4.542242898	5.003087292
	N375	-5.300359060	-5.473515706	4.822852528
	N376	-4.003807082	-3.193875081	3.744437947
	N377	-4.320773509	-1.373118796	5.947910630
	N378	-3.142060657	-2.733811954	8.217803502
	N379	-0.729275927	-4.101575242	7.406124593
	N380	0.627529750	-2.576463988	5.432314659
	N381	0.611776892	0.298422684	6.181222816
	N382	0.434333749	0.211221100	9.132302766
	N383	5.479060559	-11.632350983	5.094202597
	N384	6.202004187	-9.412764630	3.766912412
	N385	5.123262482	-3.350892071	2.777632492
	N386	4.602034949	-2.028469361	4.657518143
	N387	5.080296861	-1.043167168	2.612245900
	N388	8.965392780	-7.264750185	3.454050822
	N389	-6.199438633	-2.793937212	-2.503276364
	N390	-2.362652391	-5.554049139	-2.970156637
	N391	-6.279566857	-0.275897727	-1.762112719
	N392	-8.443609967	0.889034684	-0.428874330
	N393	-7.776019488	0.610729586	2.286640115
	N394	-5.556308319	2.297088114	2.603907747
	N395	-3.174015172	2.775751456	-0.907022860
	N396	-2.598010492	0.734245540	-0.535185128
	N397	-6.110821072	4.944860954	3.240406397
	N398	-9.529369213	8.035830843	2.089830429
	N399	-9.262833658	9.838324830	3.356318960
	N400	9.622869238	2.327464454	2.044144284
	N401	6.474604494	6.997586302	-0.645847125
	N402	4.276020196	8.258309333	-3.091274035
	N403	1.579175354	7.732749420	-3.410129607
	N404	-3.238778394	6.450965128	-4.269186422
	N405	-3.474990305	8.291881616	-5.703769498
	N406	-4.440453729	8.320811034	-3.602339980
	N407	0.727079782	6.286558424	-1.072786512
	N408	1.227848346	8.207247210	0.894987004
	N409	-0.063244302	10.625384709	0.628875907
	N410	-1.768441291	10.274657420	-2.915627220
	N411	-2.351080444	11.736154286	1.795462781
	N412	3.389899653	-4.859751497	-10.296473979
	N413	1.900250873	-2.660333311	-7.853379243
	N414	0.952139518	0.157681158	-7.285416710
	N415	3.079309865	2.084113735	-7.395759724
	N416	-2.375701764	-2.984007357	-9.832148848
	N417	-3.807623210	-1.114301780	-8.200758870
	N418	7.486606291	-7.327753725	-6.335083627
	N419	3.752164941	-5.822929034	-4.424147697
	N420	3.007297615	-6.695564907	-6.304740638
	N421	8.732197519	-7.780508352	-3.476565792
	N422	-0.016931975	2.341071606	-0.207896729
	N423	0.746685964	1.685183459	-0.724246240
	O424	-6.736024791	-4.965861544	2.030310453
	O425	-6.944832454	-2.507234571	4.821034444
	O426	-4.953388620	-4.757522156	6.962521482
	O427	-2.130531354	-3.864920374	4.846663737
	O428	-2.376811013	-0.418230523	5.241248920
	O429	-2.375843368	-0.666484324	8.824555022
	O430	0.036960862	-3.836716181	9.554612988
	O431	2.571624007	-3.688114500	5.955454331
	O432	2.816608289	0.048258790	5.629784868
	O433	2.225062413	1.600263411	9.007264075
	O434	2.671512212	-0.275359472	11.888863219
	O435	6.820747817	-10.576711059	1.869017241
	O436	7.988316942	-7.922526593	5.428959221
	O437	11.703331187	-5.020742196	4.082142142
	O438	-5.726003165	-0.692758508	0.424832156
	O439	-4.143990010	-4.348134509	-3.711856415
	O440	-7.154443556	2.716379020	0.032158979
	O441	-6.481384563	3.221424458	-2.931404775
	O442	-9.647279368	1.801961773	2.879720542
	O443	-5.349993243	1.703091632	4.799454388

O444	-3.978199626	5.229887543	4.009446460
O445	-6.215077134	7.465107157	5.724087420
O446	10.939725235	-0.461402023	3.719676210
O447	3.854514158	4.565613258	3.771841176
O448	3.780474384	8.237352534	-0.867204434
O449	1.075829689	9.968693595	-3.589032036
O450	-1.335481631	7.189987572	-1.476268500
O451	-0.421149743	7.272130281	2.190493240
O452	2.575193236	5.632798857	1.384072377
O453	1.285073555	11.612510687	2.196496373
O454	-2.087615043	13.682231178	0.637301620
O455	-3.064087861	11.102959247	-1.225530474
O456	-4.384519251	13.880720359	3.847313028
O457	-5.421119507	6.997559872	-1.026412997
O458	1.112293274	-4.634683801	-8.720471794
O459	-0.816103746	-0.767831104	-8.410072238
O460	1.710720056	3.509346557	-8.522941018
O461	5.479060467	4.059280160	-9.225612752
O462	-5.578869007	-2.533329874	-8.423233549
O463	-2.000730120	-6.631844503	-5.801726407
O464	-0.130931922	-5.808022958	-6.694266405
O465	-4.361116445	2.402923936	-8.074738245
O466	9.465108640	-5.744130251	-4.152994571
O467	12.204325526	-7.367815227	-2.660877775
O468	-0.043627135	-6.294459368	-1.202252063
O469	-0.508917333	-7.333251297	0.735452550
O470	4.483422591	-11.123306298	-3.471231993
O471	2.222004071	-11.029273909	-3.443284245
O472	4.776706304	-6.106031355	-1.552758823
O473	4.716296030	-7.764231035	-0.036082081
O474	2.219413700	-6.213341974	-2.090375478
O475	8.740192828	-6.893249229	0.646098396
O476	6.860677623	-4.878479665	-0.045303574
O477	-4.417134656	4.865007876	-2.193090871
O478	7.135060612	-10.720729072	-2.801264186
O479	7.156410647	-8.919763034	-0.368133032
S480	3.495242147	4.800305926	-2.451556691
S481	1.870681806	1.854692221	-3.444231153
S482	1.611930276	-3.440882337	-3.299325202
S483	3.561336732	1.593561098	-0.205888106
S484	0.318814346	-1.033807455	-1.610027343
S485	3.577533467	-0.916279357	-5.582413690
S486	3.569922952	-3.779457469	-0.274356515
S487	5.436093084	1.542833223	-3.246614939
S488	5.312526260	-3.307660681	-3.384198206
S489	6.233725821	-1.097398875	-0.526708067
end			

S2BH-6Hx-6H2n-3b6 to S2BH-6Hx-6H2n-7Hn

35, S=1/2

reactant

		bm522bh2n2x6x6h2n3b6c.car_2			
Fe(139)	-2.473	C1	-7.619855381	-5.416716633	2.418913509
Fe(140)	-1.164	C2	-8.238966643	-6.530911526	1.568811630
Fe(141)	-2.592	C3	-8.045856457	-3.412278445	3.761079907
Fe(142)	2.599	C4	-7.144275523	-3.670108660	4.963391591
Fe(143)	2.753	C5	-6.445550956	-5.205848187	6.728846671
Fe(144)	1.745	C6	-4.951526419	-5.268104444	6.403888601
Fe(145)	-0.083	C7	-3.210130972	-5.631243975	4.737770082
		C8	-2.650911844	-4.214827199	4.582637247
		C9	-3.134500054	-1.902607058	3.936368598
		C10	-2.845165911	-1.252389767	5.297310432
		C11	-3.499166003	-0.982068493	7.616020733
		C12	-2.335079006	-1.599634194	8.403588920
		C13	-1.344388163	-3.652717695	9.299792898
		C14	0.032848911	-3.986155025	8.710207420
		C15	1.275592332	-4.691590857	6.705233646
		C16	1.963938055	-3.628792511	5.845775262
		C17	1.677345557	-1.572183134	4.484619110
		C18	2.115398178	-0.386572628	5.375303824
		C19	0.638699297	-1.162316099	3.418637363
		C20	1.065948580	0.092126908	2.637647820
		C21	0.368330499	-2.343954202	2.481051010
		C22	1.413872630	1.364432498	6.970519408
		C23	1.964368382	0.985800224	8.360374479
		C24	0.162106870	2.272990767	7.059237984

	C25	0.384028055	3.421846005	8.050933034
	C26	-0.208458331	2.820506825	5.674815343
	C27	1.656513676	-0.209647391	10.452763807
	C28	3.126707797	-0.547590564	10.581239890
	C29	6.411878356	-11.660317806	2.877061149
	C30	6.869522298	-10.368744827	2.199509199
	C31	7.154660350	-7.922234264	2.600045945
	C32	8.486554700	-7.556097234	3.284773292
	C33	6.035146829	-6.941232143	2.987176819
	C34	6.342182941	-5.496080627	2.592955783
	C35	5.117354317	-4.594298111	2.735994443
	C36	5.238013694	-2.073418505	2.849712453
	C37	10.756663459	-6.700789422	2.945513806
	C38	11.034368065	-5.214740496	2.907366801
	C39	-5.626904095	-2.681570495	-0.718422453
	C40	-5.907692264	-1.255220657	-0.206753804
	C41	-4.095846431	-2.827436345	-0.881420175
	C42	-3.567360557	-4.259401532	-1.105023903
	C43	-3.505298005	-4.716357639	-2.562359832
	C44	-6.487757990	1.010128618	-1.000691498
	C45	-7.439236869	1.455053073	0.118958718
	C46	-6.833790400	1.638034592	-2.358765786
	C47	-9.405676520	0.896426472	1.469936681
	C48	-8.781085920	0.915409613	2.870019051
	C49	-6.822413076	0.412826080	4.260347467
	C50	-5.627120435	1.364471137	4.293549891
	C51	-4.281711934	3.068202289	3.170528457
	C52	-4.641806068	4.357325484	3.947404115
	C53	-3.900616425	3.516139915	1.751935330
	C54	-3.416281581	2.457144637	0.818011584
	C55	-2.962663363	1.172926911	0.982169418
	C56	-2.887331133	1.642352918	-1.195227152
	C57	-6.440274369	5.992943209	4.367865630
	C58	-6.158557410	6.099677366	5.866072392
	C59	-7.960831506	6.053179260	4.092095624
	C60	-8.574241402	7.422184608	4.067298440
	C61	-8.335361583	8.530807192	4.857048515
	C62	-9.914013037	8.968379954	3.364423565
	C63	9.593910171	1.532931142	2.220575744
	C64	10.585985624	0.427656662	1.910921987
	C65	8.180819329	0.896819974	2.203068782
	C66	7.096097545	1.879685235	2.574930431
	C67	6.727816985	2.089343803	3.917254550
	C68	6.428459136	2.622449626	1.585085610
	C69	5.718165646	2.996078743	4.261159212
	C70	5.429801553	3.540353619	1.913933701
	C71	5.069283102	3.718521325	3.253248738
	C72	5.091333347	6.833441255	-1.987542407
	C73	4.023008160	7.938619058	-2.057644623
	C74	4.466560943	5.521235979	-1.523172530
	C75	2.815561728	9.532965710	-3.499205007
	C76	1.332053884	9.164782503	-3.422815791
	C77	-0.283732778	7.289789561	-3.189059862
	C78	-0.551704980	6.948002200	-1.700260699
	C79	-0.396642289	6.038354484	-4.091110366
	C80	-1.665408511	5.180438405	-4.017851319
	C81	-2.916553006	5.731598883	-4.724373830
	C82	-4.344777573	7.652626124	-3.997634275
	C83	0.260828461	6.008230382	0.407829748
	C84	0.263246684	7.239703537	1.324892919
	C85	1.319026071	5.024173724	0.947689907
	C86	1.473246506	9.295081894	1.866531602
	C87	0.766598978	10.629065068	1.681709738
	C88	-0.881408898	11.885899173	0.407006241
	C89	-2.036017630	12.446454952	1.277021865
	C90	-1.276828890	11.906969604	-1.072495081
	C91	-2.466325674	11.015712938	-1.400373292
	C92	-3.741536560	11.971288300	2.981183179
	C93	-3.494416759	13.040883132	4.035124009
	C94	-4.307809525	10.686890039	3.653498442
	C95	-4.701785091	9.625153925	2.650780858
	C96	-5.993158308	9.608787065	2.099979482
	C97	-3.783669294	8.659431129	2.205427310
	C98	-6.351989951	8.676400139	1.122637122
	C99	-4.113771655	7.737201850	1.206321169
	C100	-5.400069232	7.761063050	0.673081772
	C101	2.714241200	-3.808305000	-9.394219007
	C102	1.354939056	-3.580673188	-8.758147542
	C103	0.072722465	-2.099010582	-7.233416553
	C104	-0.480996701	-0.732945138	-7.656479413

C105	-0.057477854	1.683271267	-7.653263395
C106	1.083369169	2.650114421	-7.956004507
C107	3.420458703	3.289752723	-7.728259303
C108	3.890145030	3.396771638	-9.166519894
C109	-4.095861904	-3.371441857	-8.592844496
C110	-5.064561560	-2.295094343	-8.090046164
C111	-3.430937904	-4.081937791	-7.391390893
C112	-2.775148473	-5.406784088	-7.823947063
C113	-1.899853254	-5.950485961	-6.718795823
C114	-5.370590807	0.016928908	-7.354207878
C115	-4.981587070	1.312591664	-8.038832628
C116	-5.518493020	0.119764578	-5.815593700
C117	-4.170919073	0.100012932	-5.151782023
C118	-3.405897621	1.273492057	-5.046030736
C119	-3.586361494	-1.128107750	-4.791680952
C120	-2.059179740	1.206726062	-4.675009607
C121	-2.240255545	-1.193487273	-4.426040990
C122	-1.465718022	-0.030377186	-4.410161609
C123	7.045012016	-6.146940627	-5.889503313
C124	8.361024736	-6.264070779	-5.103988377
C125	5.849331164	-6.568153645	-5.021934965
C126	4.487284169	-6.319564979	-5.590708571
C127	3.951809690	-6.867157919	-6.737305823
C128	2.368727831	-5.760240802	-5.656711542
C129	9.800184402	-7.526919664	-3.618071818
C130	11.040484690	-7.288835026	-4.452458980
C131	0.457005298	-6.998410182	-0.332352533
C132	1.979086882	-7.077586878	-0.249520270
C133	2.699808641	-7.171386657	-1.625826414
C134	2.530454242	-8.598050577	-2.215214199
C135	3.403230501	-8.911899786	-3.433579495
C136	3.367908944	-10.397971712	-3.854608548
C137	4.204793045	-6.921725297	-1.436658124
C138	3.131444866	-0.706850255	-2.534297735
Fe139	3.130695383	-1.979672074	-4.079499481
Fe140	1.772193750	0.730931893	-1.766634278
Fe141	4.630607159	0.440559738	-1.923688506
Fe142	3.202285522	0.518806048	-4.072188842
Fe143	3.400414704	2.694150496	-2.514129247
Fe144	4.497329166	-2.079476467	-1.923890215
Fe145	1.829221656	-2.502605872	-1.559940191
H146	-6.527350665	-7.568754127	1.174299194
H147	-6.955713101	-6.545139887	-0.020882811
H148	-9.497464541	-4.696552096	2.867639746
H149	-9.085844621	-6.114391114	1.000215312
H150	-8.675615189	-7.269036860	2.262798430
H151	-7.472622830	-2.768114351	3.078243898
H152	-7.976905864	-5.516523726	5.248925432
H153	-8.923859354	-2.848602606	4.104215175
H154	-6.766441368	-6.184814146	7.107889174
H155	-5.329296450	-5.803183917	4.442552362
H156	-6.559164304	-4.474315049	7.540588100
H157	-2.235053815	-1.775837818	3.322957359
H158	-4.439823637	-3.589633968	3.798768373
H159	-3.115056543	-6.161363514	3.780304421
H160	-3.293575841	0.090905766	7.518571905
H161	-4.535110332	-2.114705418	6.102888540
H162	-2.589004729	-6.145981883	5.481289571
H163	-3.966758413	-1.381235883	3.443208887
H164	-3.012095002	-3.504843062	7.966976596
H165	-4.425478988	-1.113061183	8.191244881
H166	-0.047427708	-3.211297477	3.010338993
H167	-0.340252759	-2.063646840	1.685581988
H168	1.303384904	-2.670178415	1.999940993
H169	-0.287766513	-0.918043564	3.962107660
H170	-1.806417701	-4.599513909	9.616426196
H171	-0.422872939	2.017038153	4.956333310
H172	0.353852204	-0.273981411	8.772519384
H173	0.569326272	3.062158692	9.073158242
H174	1.688875921	-0.162197201	1.767079082
H175	1.641875964	0.802413108	3.248373413
H176	9.795364165	1.877432203	3.252800919
H177	-1.102201185	3.458433200	5.744256683
H178	-0.504662712	4.069399563	8.076171941
H179	0.614934849	3.433584342	5.272664528
H180	1.244451007	4.039525444	7.747605084
H181	-0.677237985	1.655875141	7.428507501
H182	2.230775197	1.925454809	6.494864919
H183	0.180896236	-0.125867216	6.039002593
H184	0.179710119	0.635410386	2.275235729

H185	1.503489287	4.244061920	0.194014913
H186	0.887178686	4.551566616	1.842815937
H187	2.926447973	5.275140047	2.061107939
H188	3.887937179	4.649285523	4.481606743
H189	4.911665613	4.099503597	1.135188051
H190	6.673315135	2.461122205	0.532533638
H191	7.222332801	1.520724240	4.709293070
H192	0.155443647	-2.763040211	5.415492596
H193	1.063403338	-5.554383501	6.054893411
H194	2.002991507	-5.008100600	7.461984607
H195	-0.800708083	-4.246059285	6.831003576
H196	-1.160241659	-3.043900277	10.193218018
H197	5.428024174	3.128022272	5.307430466
H198	2.587749934	-1.925375944	3.989235311
H199	1.420570831	0.621905133	11.137811777
H200	1.091431820	-1.095056992	10.783870004
H201	3.589475814	-1.019629331	9.683830863
H202	11.547013269	-7.230941441	2.392349648
H203	-2.443116020	-5.925634114	-3.827460583
H204	0.847233912	-6.140404157	-2.016157881
H205	-3.561050781	-6.146043288	-8.034227703
H206	-2.183703651	-5.256789651	-8.738751734
H207	-4.177890637	-4.290959500	-6.610171421
H208	-4.527031751	1.177679036	-9.048222878
H209	-4.754644165	-4.106050143	-9.081296820
H210	-3.773574063	-2.181144556	-1.715377792
H211	-4.198345385	-4.986607785	-0.565274083
H212	-3.524155415	-0.910599009	-7.937587707
H213	-6.380371187	-0.200643554	-7.759503347
H214	-2.447080773	-2.273539296	-9.220832694
H215	-1.770762172	-5.825398655	-2.206571994
H216	-2.687341477	-3.405820755	-6.941449087
H217	-2.746094752	-3.621783696	-10.118866259
H218	-7.211001993	-3.419840448	-1.820227575
H219	-3.636077470	-2.442046039	0.041442602
H220	-0.344030938	-5.182619995	-7.647853848
H221	-2.565476809	-4.346613022	-0.659066681
H222	-3.416529307	2.635481139	3.691271003
H223	-2.845194418	0.569616430	1.871862809
H224	-7.521493396	0.752235144	5.037236697
H225	-7.080709816	-0.080773465	2.201034169
H226	-6.611056575	4.072440467	3.439605871
H227	-7.643578056	8.712975032	5.672539343
H228	-6.230062170	5.124619952	6.407257049
H229	-8.482877297	5.402227751	4.816141782
H230	-5.924816042	6.819525683	3.853548879
H231	-10.668018795	9.532420181	2.821284090
H232	-3.116178965	4.282857908	1.855913129
H233	-4.771768172	4.007721083	1.289446573
H234	-9.278002291	10.448395077	4.755518619
H235	-8.136651052	5.611097646	3.098305264
H236	-5.992000240	2.038988777	2.355565824
H237	-6.467187464	-0.583988119	4.562348681
H238	-9.893047314	1.872431234	1.338129451
H239	-2.716796894	1.544088283	-2.264976443
H240	-10.188655192	0.127297181	1.450653568
H241	10.560486560	3.123488813	1.367398973
H242	8.932312865	3.234066868	1.294746204
H243	1.293637881	5.979620757	-1.489184816
H244	-0.728713456	5.542124375	0.523029962
H245	1.737838913	8.073858899	0.163775881
H246	-3.743946432	3.586607386	-0.995727768
H247	-5.532550980	4.202911029	-1.900656634
H248	-7.340069811	3.424988337	-2.991737104
H249	-5.084714076	5.443221044	-1.007916908
H250	-6.085090429	1.299814928	-3.094441886
H251	-7.828952318	1.300019328	-2.684643213
H252	-0.402628608	11.660999673	-1.690820027
H253	-1.572970186	12.941583746	-1.304493264
H254	-1.365288092	10.090980399	-2.890673080
H255	-3.004083315	9.502100199	-2.614612219
H256	-4.399888655	9.326753908	-5.185648785
H257	-3.479140872	4.908047579	-5.189330727
H258	-7.363767145	8.670305363	0.709901343
H259	-2.782939500	8.603330405	2.641556995
H260	-5.139801469	7.765525227	-2.094876151
H261	0.454427233	5.381127287	-3.848231576
H262	1.835545745	7.188238631	-3.413488585
H263	-1.907656225	4.922464545	-2.974536520
H264	-8.562684746	-0.229222282	-0.153075120

H265	3.000237258	9.980258010	-4.485276578
H266	-6.556375173	-0.924746126	-2.060534313
H267	-0.242090135	6.366271280	-5.132466560
H268	-3.362895952	7.039771148	0.833998679
H269	2.979879712	10.310709711	-2.739857571
H270	-3.752401336	7.914638285	-5.956491814
H271	-5.659987412	9.000812362	-3.193928132
H272	-5.740216844	-3.481240247	-2.613520195
H273	-5.945919388	-3.359732447	0.094416609
H274	-6.738988359	10.331303483	2.438665078
H275	-5.509601346	1.393913182	-0.679178482
H276	-1.021130254	8.046728054	-3.470429032
H277	-2.608389436	6.399643697	-5.538560495
H278	-1.413627581	4.231822857	-4.512060858
H279	-4.149541225	5.895119026	-2.996264214
H280	1.280641621	8.956069147	2.895376796
H281	-0.483377299	9.771856176	0.308872835
H282	-0.084834097	12.635102516	0.537202582
H283	2.549897155	9.487740743	1.768593305
H284	-2.188462579	10.680835683	2.264804525
H285	3.210557546	-2.843000509	-9.571458526
H286	3.317859096	-4.341411298	-8.630472270
H287	6.953549664	-5.078775662	-6.135768497
H288	3.488110977	-4.888457857	-10.940660271
H289	1.979047110	-5.349581004	-10.503365381
H290	5.260677006	4.764166469	-1.484755650
H291	5.755785887	7.458776332	-0.161149557
H292	4.255908992	8.071202602	-4.098735908
H293	4.032399917	5.648842359	-0.521382871
H294	6.646645425	8.042951721	-1.401722723
H295	4.275092322	2.953719895	-7.120365590
H296	5.533468117	6.684502566	-2.985269447
H297	5.858432907	-7.011963520	4.073139766
H298	7.282543501	-7.914818165	1.510185715
H299	2.542871133	1.475311885	-7.034278391
H300	5.445046627	-0.051549601	2.612500237
H301	-0.706361472	1.653374758	-8.537498192
H302	-1.789537707	-2.154373339	-4.166293465
H303	-0.649972884	2.148165366	-6.846520351
H304	4.789090765	-4.532615963	3.779998126
H305	1.086575305	0.118954654	-6.673301830
H306	4.273750956	-5.010141415	2.159561042
H307	5.119698808	-7.281413632	2.477266623
H308	6.668849851	-5.463036525	1.542486866
H309	7.163416136	-5.088033772	3.203320949
H310	-6.132837403	-0.728243541	-5.480310253
H311	6.494253912	-9.471123595	3.947267204
H312	5.434878642	-11.905617049	2.432589594
H313	-0.404745120	-0.065952466	-4.171749667
H314	7.133814539	-11.780592643	4.801906844
H315	5.553720247	-12.189715575	4.669750192
H316	5.776656920	-3.216691960	1.274799882
H317	-1.447351451	2.108725466	-4.613555305
H318	-4.181392939	-2.043841947	-4.820914358
H319	7.191408474	-9.952313433	-2.825522652
H320	5.923842834	-7.649662108	-4.814289066
H321	6.444167523	-8.239010692	-1.100961681
H322	6.169049946	-10.547638698	-3.841717639
H323	3.558386509	2.565843058	-9.834383498
H324	6.213892244	-5.180569300	-1.267811879
H325	2.259860160	-7.916534140	0.398938392
H326	2.094665099	-1.916361466	-7.821360398
H327	2.777796072	-9.305328447	-1.407243158
H328	1.928556120	-6.801443973	-7.435559508
H329	1.386673626	-5.372119136	-5.420357963
H330	7.111707830	-12.449753190	2.555163955
H331	5.587459573	-0.956950501	1.123446424
H332	8.334968782	-5.865949060	-0.473961014
H333	7.068749863	-3.884819981	-1.285340559
H334	9.236249175	-6.931910844	1.440843000
H335	4.626347926	-2.807621162	4.668780143
H336	3.060676821	-8.310841555	-4.290027141
H337	4.449145297	-8.633171505	-3.242249571
H338	1.469757997	-8.757617340	-2.463816023
H339	0.330495866	-2.093038485	-6.162282393
H340	-3.859073065	2.231601762	-5.310894625
H341	-6.065870930	1.051433373	-5.607494222
H342	2.296618235	-6.149829695	0.255891653
H343	8.443731366	-7.398429690	-0.687521804
H344	7.346774613	-9.306842157	-0.440531255

H345	4.563612944	-1.116637180	4.523913620
H346	4.398135975	-7.503275875	-7.492627430
H347	5.920859597	-6.067973263	-4.049939820
H348	-0.699924903	-2.849173549	-7.413876338
H349	3.130351618	4.289852066	-7.372893005
H350	8.186202550	0.052802440	2.908506013
H351	7.991853290	0.491789041	1.195896316
H352	7.875839228	-6.543651288	-7.722359710
H353	7.287696255	-7.878809224	-6.980510578
H354	9.855348190	-8.559989700	-3.241110066
H355	7.899757960	-8.166909523	-4.344257243
H356	10.927205615	-7.527673408	-5.537059244
H357	9.805085998	-6.854831398	-2.745397684
H358	10.705214804	0.209005756	0.822238764
H359	-2.425862191	13.195064389	4.316519031
H360	10.812915744	-7.012986247	4.003293335
H361	10.130744429	-4.559035439	2.946326230
H362	-4.490115032	12.370322732	2.277094485
H363	-3.553367918	10.289873518	4.351057991
H364	-5.178197550	10.990990146	4.252299590
H365	0.800956133	-3.482964879	-1.049162393
H366	4.644772144	-2.835428853	-0.479810911
H367	-6.684592919	6.832387797	-0.461503971
H368	-0.174835233	-0.853823812	-0.480782898
H369	2.087323764	-1.598905390	-0.255908599
H370	1.532376613	-2.255494802	-0.040409573
H371	-2.252224206	-0.214933431	-0.500618514
Mo372	3.338921680	-4.431473174	-2.903650507
N373	-7.334434918	-7.211333999	0.656733090
N374	-8.505654542	-4.611213053	3.076295997
N375	-7.288037147	-4.854507879	5.598165834
N376	-4.606268821	-5.650272123	5.148148598
N377	-3.488955307	-3.309210086	4.026033661
N378	-3.692730112	-1.570768118	6.300984996
N379	-2.283138288	-2.955926396	8.431871176
N380	0.059283277	-4.279245523	7.380960360
N381	1.165665225	-2.677317313	5.301020237
N382	1.148540475	0.191601751	6.132272763
N383	1.265926698	0.072630493	9.080843407
N384	6.259783511	-11.534323216	4.331956148
N385	6.780106189	-9.275090825	2.982114629
N386	5.399286302	-3.250612786	2.222131353
N387	4.989433978	-1.989295337	4.169521466
N388	5.348082959	-0.941933529	2.131887378
N389	9.437893527	-7.055064952	2.457254585
N390	-6.336486268	-2.916471965	-1.980836704
N391	-2.505077138	-5.561274406	-2.871301475
N392	-6.410845518	-0.431434732	-1.162657794
N393	-8.485853531	0.637305619	0.374777223
N394	-7.551539804	0.342343738	3.008206985
N395	-5.361311137	2.087876296	3.161587280
N396	-3.355724463	2.714621503	-0.544724819
N397	-2.654343239	0.695870913	-0.276726135
N398	-5.950915837	4.710299198	3.877522702
N399	-9.561746633	7.708433554	3.138481714
N400	-9.196275110	9.502696094	4.391993147
N401	9.712019714	2.576958026	1.200252817
N402	6.175312619	7.200241616	-1.060415356
N403	3.749601924	8.439488312	-3.297878613
N404	1.048545109	7.845969407	-3.394755128
N405	-3.827483727	6.438022728	-3.817185698
N406	-4.194774585	8.333353199	-5.147193467
N407	-5.000364228	8.257364817	-2.980654138
N408	0.454643233	6.345268625	-1.004718858
N409	1.085893936	8.253185894	0.933419832
N410	-0.316616409	10.623688476	0.844412084
N411	-2.306747557	10.235386870	-2.503427131
N412	-2.517722450	11.642943953	2.257715034
N413	2.572252079	-4.529973629	-10.661800624
N414	1.248549570	-2.463480319	-8.010179609
N415	0.293902542	0.317549388	-7.307955203
N416	2.340003176	2.333993171	-7.554181218
N417	-3.195965251	-2.847286787	-9.626825853
N418	-4.530215244	-1.068754375	-7.824290929
N419	7.090836490	-6.889569379	-7.162782562
N420	3.477397088	-5.615241894	-4.928990754
N421	2.623973956	-6.502121902	-6.757540459
N422	8.583062079	-7.408294343	-4.402018192
N423	-0.088055363	2.352640946	-0.151878037
N424	0.625211992	1.742471230	-0.783457857

	O425 -6.392677987 -5.258600014 2.538757015<br>O426 -6.319285743 -2.810276726 5.324880065<br>O427 -4.109506810 -4.997278699 7.276088290<br>O428 -1.496494177 -3.921633247 4.945791567<br>O429 -1.884648545 -0.478601384 5.445211741<br>O430 -1.524129091 -0.878047726 9.010533982<br>O431 1.024395920 -4.032180591 9.449596779<br>O432 3.192187650 -3.701288367 5.658771458<br>O433 3.294850334 0.017035356 5.372318935<br>O434 2.995673020 1.518414947 8.804565641<br>O435 3.751956653 -0.394341008 11.621158768<br>O436 7.259055793 -10.388063920 1.013858984<br>O437 8.640596111 -7.722794023 4.507162616<br>O438 12.158956245 -4.741716279 2.854046871<br>O439 -5.666923824 -0.887745051 0.960516738<br>O440 -4.351898270 -4.348880580 -3.409119424<br>O441 -7.219429664 2.498154727 0.760135658<br>O442 -6.819206127 3.074313258 -2.247351960<br>O443 -9.410290109 1.429066779 3.803691667<br>O444 -4.940027231 1.458347144 5.316175551<br>O445 -3.778543456 5.044483966 4.500557648<br>O446 -5.917348076 7.141123315 6.450349376<br>O447 11.201992055 -0.201389019 2.758920645<br>O448 4.049189168 4.612444541 3.521074970<br>O449 3.449398034 8.370172552 -1.041374444<br>O450 0.474790767 10.069623885 -3.405172333<br>O451 -1.651211500 7.220543185 -1.191235530<br>O452 -0.396321624 7.254439179 2.372564468<br>O453 2.535321900 5.702915240 1.272652440<br>O454 1.157934169 11.641919943 2.274238865<br>O455 -2.446896994 13.601245408 1.094395738<br>O456 -3.511307463 11.033480318 -0.734230340<br>O457 -4.397305147 13.670118577 4.562565230<br>O458 -5.712712191 6.857446683 -0.364570866<br>O459 0.424262128 -4.410105996 -8.908456948<br>O460 -1.552243500 -0.620739902 -8.288209566<br>O461 0.817879324 3.725806540 -8.518376953<br>O462 4.602865889 4.300335302 -9.571230170<br>O463 -6.269033776 -2.540927096 -7.911660171<br>O464 -2.338550115 -6.560889983 -5.749103040<br>O465 -0.579700352 -5.690373670 -6.810453229<br>O466 -5.181483658 2.427380774 -7.578708566<br>O467 9.224689796 -5.366482788 -5.144654767<br>O468 12.115945253 -6.940260792 -3.985753928<br>O469 -0.024308842 -6.372135165 -1.412057086<br>O470 -0.277249533 -7.430662001 0.554960042<br>O471 4.485560617 -10.949456063 -4.149118019<br>O472 2.237113642 -10.959558103 -3.901033253<br>O473 4.729479457 -5.953893268 -2.146411238<br>O474 4.868460325 -7.636754262 -0.664438226<br>O475 2.155151048 -6.160427723 -2.475525216<br>O476 8.947500350 -6.624772324 -0.311732326<br>O477 6.908439229 -4.691274092 -0.759028287<br>O478 -4.757142669 4.783580453 -1.669509919<br>O479 7.152834422 -10.424730517 -3.686526047<br>O480 7.326448506 -8.684473675 -1.210829161<br>S481 3.129237022 4.950377255 -2.669118721<br>S482 1.540329381 1.995670375 -3.611668915<br>S483 1.380639932 -3.342933028 -3.636543547<br>S484 3.460818564 1.729439781 -0.502619900<br>S485 0.153949533 -0.942150329 -1.799290017<br>S486 3.124488036 -0.718324334 -5.911190897<br>S487 3.384988625 -3.749403904 -0.640084996<br>S488 5.112891312 1.709187906 -3.666300335<br>S489 5.067448886 -3.099356994 -3.806005483<br>S490 6.163782983 -0.926587514 -1.095076657<br>end

TS

	bm522bh2n2x6x6h2n3b6b_1_53144.80<br>C1 -7.417945207 -5.608628429 2.571699486<br>C2 -8.016177669 -6.747357308 1.738878523<br>C3 -7.883737279 -3.596533979 3.890158762<br>C4 -6.967533462 -3.812189151 5.088562302<br>C5 -6.204253593 -5.307696569 6.862340499<br>C6 -4.711593395 -5.324065389 6.526343087<br>C7 -2.971469096 -5.654393336 4.851577553<br>C8 -2.464456039 -4.222099732 4.664189392<br>C9 -3.036338251 -1.940430283 3.980698294<br>C10 -2.747893770 -1.257456361 5.326534881

	C11	-3.373231351	-0.978305832	7.653335929
	C12	-2.185827552	-1.549153883	8.442635046
	C13	-1.126342466	-3.564403141	9.351462922
	C14	0.255531976	-3.863170000	8.754247131
	C15	1.500581952	-4.557560485	6.747426348
	C16	2.152822556	-3.489021829	5.867389529
	C17	1.783223201	-1.460184839	4.481000299
	C18	2.195289285	-0.251089132	5.351438751
	C19	0.709283671	-1.108747711	3.428973305
	C20	1.039558875	0.177722242	2.645942236
	C21	0.490973900	-2.307142748	2.499857426
	C22	1.456536308	1.499816637	6.929712714
	C23	2.031722691	1.148671004	8.317095351
	C24	0.180204215	2.372294444	7.027823003
	C25	0.378808461	3.538252306	8.005259300
	C26	-0.232508095	2.893637023	5.645243932
	C27	1.776003465	-0.021333009	10.429786556
	C28	3.259921667	-0.295752690	10.554739881
	C29	6.788530177	-11.420073583	2.958193856
	C30	7.194314563	-10.125599954	2.251512522
	C31	7.422973541	-7.665297166	2.613759554
	C32	8.748972379	-7.245196260	3.278100524
	C33	6.272070124	-6.717365511	2.997048314
	C34	6.522104708	-5.266497799	2.577263960
	C35	5.258629087	-4.413176153	2.712391647
	C36	5.324920539	-1.887243458	2.784955428
	C37	10.990908311	-6.334029261	2.905819456
	C38	11.227419153	-4.841100376	2.843379856
	C39	-5.541565883	-2.858885574	-0.617254518
	C40	-5.858483617	-1.435334564	-0.119413713
	C41	-4.007954986	-2.962224345	-0.792522482
	C42	-3.433286934	-4.377855924	-1.005600933
	C43	-3.383338018	-4.856498193	-2.456441609
	C44	-6.523078254	0.798031702	-0.930483268
	C45	-7.477387457	1.225182322	0.194057571
	C46	-6.899714041	1.402605138	-2.291304263
	C47	-9.411039989	0.613147079	1.574274435
	C48	-8.772970418	0.664589704	2.967452766
	C49	-6.782261540	0.257608333	4.339909391
	C50	-5.617077049	1.246394298	4.344672987
	C51	-4.335156157	2.970926436	3.181441348
	C52	-4.727006109	4.259574397	3.944021003
	C53	-3.984590023	3.411620373	1.753328082
	C54	-3.469526565	2.358916369	0.829419032
	C55	-2.967839618	1.093978955	1.006646272
	C56	-2.941069452	1.531825877	-1.177980171
	C57	-6.570615203	5.843212577	4.360115931
	C58	-6.282591404	5.977165914	5.854129626
	C59	-8.094925618	5.850681905	4.097202914
	C60	-8.755548896	7.197464936	4.068747378
	C61	-8.546700850	8.323676351	4.842861224
	C62	-10.144598244	8.693419289	3.351469974
	C63	9.568689163	1.852316341	2.094296125
	C64	10.586287810	0.768937548	1.789299691
	C65	8.173954638	1.175043978	2.099112337
	C66	7.063698347	2.127432948	2.474059511
	C67	6.704200439	2.338209302	3.818693249
	C68	6.364945478	2.841924816	1.485566163
	C69	5.673730533	3.219673801	4.167525447
	C70	5.345662662	3.735600408	1.819114067
	C71	4.997052193	3.917700463	3.161169138
	C72	4.859027456	6.960437547	-2.130441575
	C73	3.753755613	8.028084749	-2.205228686
	C74	4.285426118	5.628388663	-1.651264837
	C75	2.486512102	9.565944546	-3.657405174
	C76	1.015528104	9.155286916	-3.553614278
	C77	-0.539464366	7.234317228	-3.279660149
	C78	-0.775988064	6.905178282	-1.782634610
	C79	-0.626252067	5.967003065	-4.160994253
	C80	-1.868390383	5.073477430	-4.052505918
	C81	-3.144527047	5.574142396	-4.751089727
	C82	-4.622535493	7.459605275	-4.030532346
	C83	0.091676660	6.026814798	0.327571487
	C84	0.057856604	7.273655721	1.222470473
	C85	1.186495475	5.085109486	0.871395737
	C86	1.216097671	9.372170345	1.718394044
	C87	0.459094699	10.675474455	1.521667257
	C88	-1.246769029	11.855465816	0.254218554
	C89	-2.404514462	12.391358502	1.136223579
	C90	-1.668012055	11.847656537	-1.217846396

	C91	-2.822384904	10.901337047	-1.511929313
	C92	-4.073274495	11.886226850	2.867015351
	C93	-3.839281271	12.971861703	3.908082082
	C94	-4.595032525	10.595307935	3.562911162
	C95	-4.963524386	9.508613835	2.578107697
	C96	-6.260755695	9.444161174	2.045300669
	C97	-4.021532237	8.566398336	2.132844467
	C98	-6.603456876	8.488446938	1.085059755
	C99	-4.336758575	7.619743970	1.152510621
	C100	-5.630342023	7.594544979	0.637831102
	C101	2.718499379	-3.844302076	-9.379458554
	C102	1.358278030	-3.648712239	-8.735010133
	C103	0.049875624	-2.190474819	-7.207709393
	C104	-0.551203993	-0.846848497	-7.637281137
	C105	-0.208767717	1.582813641	-7.663693890
	C106	0.894200109	2.584265829	-7.995081025
	C107	3.215820372	3.297304746	-7.808751917
	C108	3.689935264	3.380804879	-9.248050356
	C109	-4.086940806	-3.611049901	-8.493144691
	C110	-5.079984955	-2.554663402	-7.995550797
	C111	-3.391596980	-4.287488920	-7.289860882
	C112	-2.698295168	-5.596576525	-7.712030398
	C113	-1.786724136	-6.094211689	-6.614174801
	C114	-5.449116094	-0.245950472	-7.284447504
	C115	-5.116683260	1.057460820	-7.984634234
	C116	-5.579735702	-0.134573239	-5.745270033
	C117	-4.224036942	-0.106710936	-5.098198236
	C118	-3.487105730	1.086180155	-5.014071840
	C119	-3.605112232	-1.315481113	-4.729011712
	C120	-2.136228796	1.055807862	-4.655380584
	C121	-2.255368436	-1.344566976	-4.376039433
	C122	-1.508748112	-0.162660682	-4.381336380
	C123	7.171352573	-6.006046598	-5.893541247
	C124	8.500052117	-6.074564037	-5.126283738
	C125	6.002543316	-6.472574422	-5.012356645
	C126	4.628440905	-6.264922909	-5.565457442
	C127	4.083805218	-6.851180019	-6.688590595
	C128	2.501119055	-5.740164202	-5.612414541
	C129	9.987387317	-7.276416202	-3.635714285
	C130	11.213721651	-7.021124834	-4.485469552
	C131	0.668771048	-6.958289726	-0.259623707
	C132	2.193970599	-6.997324393	-0.191501740
	C133	2.902955609	-7.095742971	-1.575165099
	C134	2.768111731	-8.537590701	-2.136036843
	C135	3.632141601	-8.846942832	-3.362001376
	C136	3.630865348	-10.338165926	-3.765042578
	C137	4.402140629	-6.800390536	-1.402401441
	C138	3.130453866	-0.643466203	-2.565017578
	Fe139	3.192870158	-1.927823597	-4.081400949
	Fe140	1.741291768	0.731717280	-1.774934892
	Fe141	4.663226509	0.524457911	-2.053483963
	Fe142	3.129493232	0.577348180	-4.125545490
	Fe143	3.316093716	2.735304284	-2.601749738
	Fe144	4.428038291	-2.006095175	-1.887695293
	Fe145	1.849729640	-2.454983866	-1.543683014
	H146	-6.282537448	-7.745527327	1.334660525
	H147	-6.749518854	-6.743489938	0.135396151
	H148	-9.307831991	-4.927321531	3.022768053
	H149	-8.879243205	-6.358745567	1.174926605
	H150	-8.424581212	-7.489327980	2.445732004
	H151	-7.336209479	-2.946194224	3.191995943
	H152	-7.728310877	-5.686153300	5.389410548
	H153	-8.774066829	-3.052539893	4.232785281
	H154	-6.492962417	-6.291581137	7.253764485
	H155	-5.085978747	-5.902580698	4.576863032
	H156	-6.336951682	-4.570278370	7.665498000
	H157	-2.151689060	-1.796199980	3.349632945
	H158	-4.284718725	-3.674826740	3.892765186
	H159	-2.866373257	-6.198821405	3.903585128
	H160	-3.196150051	0.097597829	7.536676458
	H161	-4.390963565	-2.168279498	6.172636946
	H162	-2.326324592	-6.133225700	5.598635505
	H163	-3.893578748	-1.457508323	3.490426408
	H164	-2.805762303	-3.478044206	8.030871767
	H165	-4.289324810	-1.123247824	8.241681001
	H166	0.142840069	-3.196135424	3.042435396
	H167	-0.253988533	-2.077939554	1.722085085
	H168	1.431831780	-2.579105594	1.997847206
	H169	-0.221463106	-0.926899837	3.988346419
	H170	-1.558845898	-4.522461398	9.675264182

H171	-0.454506190	2.076665612	4.944357481
H172	0.461770330	-0.149887816	8.762189237
H173	0.582954201	3.194707647	9.029195492
H174	1.477885697	-0.039288313	1.660847418
H175	1.755049521	0.828742819	3.168063298
H176	9.769734958	2.218589226	3.118967770
H177	-1.133880219	3.518678744	5.729293620
H178	-0.528805451	4.158379397	8.031358607
H179	0.568209863	3.513001653	5.208661652
H180	1.218271152	4.177320259	7.687997553
H181	-0.634707445	1.734633206	7.415665190
H182	2.252494995	2.080479945	6.441622547
H183	0.256256541	-0.027877679	6.016003125
H184	0.129495360	0.774594955	2.483645014
H185	1.385341703	4.299765964	0.126705354
H186	0.777488260	4.613409957	1.777810413
H187	2.798048241	5.396573446	1.962877195
H188	3.794318685	4.819948982	4.391538991
H189	4.797738040	4.269620708	1.042744295
H190	6.599954375	2.677043919	0.431459851
H191	7.221735445	1.789241489	4.609543631
H192	0.314860689	-2.688424048	5.443392556
H193	1.303543183	-5.435676350	6.113180321
H194	2.241377317	-4.843088161	7.502778008
H195	-0.586429533	-4.163575931	6.885175753
H196	-0.955192950	-2.946377818	10.241190632
H197	5.394256477	3.351549210	5.216714285
H198	2.698611192	-1.785564781	3.975329323
H199	1.507959396	0.812489568	11.099884245
H200	1.249625763	-0.924694070	10.776628620
H201	3.731062365	-0.780210657	9.667790293
H202	11.789592668	-6.850532834	2.352150106
H203	-2.300151954	-6.048339111	-3.720692315
H204	1.030966508	-6.120221253	-1.962385802
H205	-3.461227919	-6.365241742	-7.899704372
H206	-2.126775978	-5.442758368	-8.639069688
H207	-4.125596145	-4.510334780	-6.500172773
H208	-4.668764989	0.933269679	-8.998259009
H209	-4.732446639	-4.367227634	-8.965733369
H210	-3.709453028	-2.314076832	-1.634186742
H211	-4.025458875	-5.116540586	-0.437866589
H212	-3.580684943	-1.122375281	-7.879974962
H213	-6.456573149	-0.503512493	-7.672023601
H214	-2.479007656	-2.473408845	-9.155338232
H215	-1.605935695	-5.898989358	-2.112799058
H216	-2.667659249	-3.582247803	-6.853594001
H217	-2.742793068	-3.845820090	-10.027066254
H218	-7.119385352	-3.648532831	-1.691226032
H219	-3.553866039	-2.553780052	0.123209833
H220	-0.273534087	-5.295750444	-7.585858752
H221	-2.419641547	-4.417493189	-0.581048957
H222	-3.451768648	2.572824995	3.699115299
H223	-2.821620174	0.505537359	1.901744167
H224	-7.486804958	0.590126346	5.115046459
H225	-7.042054081	-0.270214531	2.287722639
H226	-6.687490547	3.915088355	3.440586877
H227	-7.853332412	8.538519504	5.649552053
H228	-6.337725920	5.008457537	6.407566341
H229	-8.589441222	5.183158127	4.825785130
H230	-6.087267937	6.677846126	3.828103189
H231	-10.919172248	9.223378952	2.803549838
H232	-3.233088409	4.213259400	1.839036267
H233	-4.878525562	3.862136008	1.292645925
H234	-9.544275720	10.211784155	4.717764344
H235	-8.263655027	5.401081142	3.105482861
H236	-6.024444024	1.880892496	2.402477068
H237	-6.393416045	-0.721331447	4.658661747
H238	-9.934494043	1.569909802	1.441593727
H239	-2.785124513	1.423924224	-2.248312031
H240	-10.165099223	-0.184155609	1.569692292
H241	10.475519334	3.459674732	1.204986124
H242	8.843415293	3.517028828	1.150868790
H243	1.101947842	6.001257653	-1.582095250
H244	-0.879371139	5.527909874	0.459325764
H245	1.503422977	8.126057587	0.038333127
H246	-3.860408795	3.449313082	-0.995589629
H247	-5.682599611	4.001538424	-1.876176511
H248	-7.476614823	3.160927122	-2.941045127
H249	-5.253495913	5.265907263	-1.010032511
H250	-6.140900237	1.089120656	-3.027319819

H251	-7.880278390	1.019540775	-2.611942546
H252	-0.794977496	11.635124039	-1.849333868
H253	-2.013081605	12.866017579	-1.452173838
H254	-1.700284458	9.989362639	-2.992161323
H255	-3.303641577	9.329886037	-2.673317787
H256	-4.727350046	9.126776807	-5.223510593
H257	-3.686508786	4.726689764	-5.196362721
H258	-7.620857093	8.443319855	0.689404045
H259	-3.013578051	8.547121808	2.555095812
H260	-5.429295304	7.559567183	-2.129527172
H261	0.248122425	5.340681768	-3.919440052
H262	1.577791876	7.195358827	-3.536138220
H263	-2.088074638	4.832562137	-3.000141639
H264	-8.552018733	-0.492911609	-0.052755657
H265	2.645933676	10.000049923	-4.653983777
H266	-6.543537511	-1.152679338	-1.967506572
H267	-0.495483115	6.282832756	-5.209452555
H268	-3.571074985	6.937925423	0.782224099
H269	2.635591357	10.365203337	-2.917160896
H270	-4.026135193	7.736626806	-5.986688258
H271	-5.965113438	8.786349548	-3.233911553
H272	-5.654838291	-3.690094892	-2.498368006
H273	-5.831948023	-3.537093342	0.206786118
H274	-7.024032282	10.147520638	2.384779396
H275	-5.555409351	1.215012869	-0.618319008
H276	-1.304156717	7.964106486	-3.560838578
H277	-2.867564598	6.239188821	-5.578996132
H278	-1.597336159	4.121222416	-4.529521981
H279	-4.368956706	5.717282979	-3.016026313
H280	1.056288734	9.048014299	2.757905349
H281	-0.782190020	9.754652227	0.179810444
H282	-0.474983940	12.632768522	0.363483511
H283	2.282301186	9.604549691	1.594476581
H284	-2.497539086	10.630501028	2.139565395
H285	3.187663552	-2.866905245	-9.566560020
H286	3.340070640	-4.355962551	-8.616087352
H287	7.033446420	-4.942862351	-6.141056156
H288	3.507836232	-4.919743142	-10.921396117
H289	2.013221106	-5.411147459	-10.468870245
H290	5.109919074	4.904869175	-1.610707121
H291	5.507446071	7.618735283	-0.308626789
H292	3.966958015	8.142825954	-4.249196239
H293	3.854767486	5.750478149	-0.647376239
H294	6.376536808	8.224239652	-1.555044239
H295	4.078399665	3.010268213	-7.187270619
H296	5.304957141	6.821786267	-3.128070090
H297	6.107889927	-6.778651916	4.085835542
H298	7.537754245	-7.673160753	1.522400183
H299	2.405921832	1.461654799	-7.093241963
H300	5.496662661	0.133193249	2.529337263
H301	-0.870767693	1.522130101	-8.536843403
H302	-1.780557421	-2.291100122	-4.107023910
H303	-0.803335412	2.035400377	-6.851580725
H304	4.941696298	-4.344163041	3.759464439
H305	1.000023009	0.067604930	-6.684938445
H306	4.429352763	-4.885316733	2.160849921
H307	5.364388366	-7.099735182	2.502953473
H308	6.845049330	-5.236230059	1.525560540
H309	7.325686374	-4.815594739	3.180871541
H310	-6.161836672	-0.999525417	-5.395840227
H311	6.844933126	-9.214333960	3.997505606
H312	5.813417125	-11.701750900	2.532091840
H313	-0.443781690	-0.169059749	-4.155442240
H314	7.543867198	-11.505443040	4.871276318
H315	5.967221168	-11.938740049	4.770677042
H316	5.698171787	-3.035516844	1.169853282
H317	-1.546634062	1.973391360	-4.608356086
H318	-4.179882962	-2.244533312	-4.739389376
H319	7.454067397	-9.765091190	-2.780378457
H320	6.115262747	-7.552048227	-4.812358004
H321	6.685489418	-8.048544305	-1.078539147
H322	6.439637873	-10.411974776	-3.770789807
H323	3.374097690	2.531041196	-9.900000053
H324	6.359479750	-4.995208640	-1.287297725
H325	2.505399089	-7.815886409	0.468642156
H326	2.057800152	-1.953798293	-7.825661665
H327	3.052719842	-9.218053755	-1.317697059
H328	2.050280691	-6.831395705	-7.359005929
H329	1.516405212	-5.366799407	-5.365377216
H330	7.506236624	-12.192684399	2.634571879

H331	5.694935090	-0.780370059	1.055779529
H332	8.514313001	-5.614039407	-0.505134402
H333	7.141435045	-3.654278665	-1.299309062
H334	9.462095274	-6.625342967	1.417787644
H335	4.755074005	-2.607967557	4.621672984
H336	3.266081034	-8.266440027	-4.222923678
H337	4.672826132	-8.541327033	-3.184254270
H338	1.708397499	-8.729892973	-2.364463310
H339	0.321427055	-2.167480860	-6.140501603
H340	-3.966072056	2.030831157	-5.283189737
H341	-6.157319331	0.778464527	-5.535741590
H342	2.492059210	-6.054248141	0.297875685
H343	8.658884276	-7.147684587	-0.692952172
H344	7.615929194	-9.086909540	-0.412276519
H345	4.718846102	-0.922197175	4.478248657
H346	4.529383841	-7.494889641	-7.438603139
H347	6.067608183	-5.975504533	-4.037975471
H348	-0.702721657	-2.964482595	-7.371593955
H349	2.887949329	4.296581928	-7.484885860
H350	8.211204315	0.337788421	2.811677555
H351	7.986741623	0.754965701	1.097353114
H352	7.995954449	-6.377924538	-7.734237518
H353	7.459269962	-7.730518587	-6.985452411
H354	10.070641082	-8.300206029	-3.239783026
H355	8.096302888	-7.974642656	-4.330124755
H356	11.097503696	-7.284078435	-5.564474418
H357	9.983465035	-6.588188644	-2.775838298
H358	10.697178090	0.535443134	0.703097809
H359	-2.773691750	13.127767220	4.200480039
H360	11.064911393	-6.629111226	3.967412739
H361	10.306063541	-4.210931011	2.866833371
H362	-4.841931940	12.255408765	2.168635421
H363	-3.824988844	10.234464829	4.262463275
H364	-5.471616331	10.882217432	4.161293661
H365	0.900247908	-3.479720686	-0.996515433
H366	4.102069071	-2.192064568	-0.254085994
H367	-6.900023357	6.599158460	-0.457071225
H368	-0.146647261	-0.840799883	-0.431100672
H369	2.097196469	-1.547820387	-0.218235959
H370	1.598074379	-2.236585519	-0.002147367
H371	-2.234637842	-0.292121635	-0.467743919
Mo372	3.451357803	-4.349602339	-2.889982617
N373	-7.103921393	-7.412809994	0.823036831
N374	-8.317515650	-4.816276821	3.225939892
N375	-7.063651009	-4.996705988	5.732294038
N376	-4.363021419	-5.715920042	5.274413248
N377	-3.341483619	-3.356912386	4.102335861
N378	-3.564578816	-1.593817778	6.350301704
N379	-2.090154642	-2.902791726	8.484016697
N380	0.279447751	-4.166375805	7.427344945
N381	1.320422544	-2.572315283	5.315787676
N382	1.216055700	0.313653048	6.103226157
N383	1.369555127	0.221787047	9.053922633
N384	6.658716081	-11.278298261	4.412951360
N385	7.099871935	-9.024349789	3.022481987
N386	5.451608489	-3.069392018	2.159681171
N387	5.126962333	-1.797302947	4.114594666
N388	5.405202923	-0.760508026	2.051878892
N389	9.677639803	-6.729500655	2.433899600
N390	-6.258005598	-3.128371551	-1.869158504
N391	-2.360279739	-5.671459385	-2.769275753
N392	-6.402740907	-0.641265049	-1.078344014
N393	-8.494459346	0.377845264	0.470513259
N394	-7.520361430	0.141925474	3.096175238
N395	-5.383034232	1.956936236	3.198242695
N396	-3.435269053	2.598170337	-0.537030728
N397	-2.662183124	0.608508640	-0.249661302
N398	-6.047684126	4.568877378	3.885454749
N399	-9.755295081	7.441208491	3.141175710
N400	-9.438451488	9.263063428	4.368342911
N401	9.642967137	2.883864152	1.057419441
N402	5.931816929	7.369581569	-1.208189445
N403	3.455724357	8.505489784	-3.448815404
N404	0.771311817	7.828621448	-3.513302186
N405	-4.065938368	6.263812772	-3.842101059
N406	-4.491162681	8.140397652	-5.182744190
N407	-5.301852523	8.046526102	-3.019956878
N408	0.259001308	6.348696425	-1.092172248
N409	0.853226098	8.300037338	0.810743392
N410	-0.634697758	10.619237534	0.699458350

N411	-2.643026756	10.100830277	-2.597262840
N412	-2.850274892	11.584068556	2.131396075
N413	2.585842407	-4.579579170	-10.640101865
N414	1.226103162	-2.527457768	-7.996543945
N415	0.194626252	0.232622104	-7.311495933
N416	2.166835279	2.312190555	-7.610065583
N417	-3.212558369	-3.077413720	-9.544139703
N418	-4.580760975	-1.308126718	-7.756004892
N419	7.227772611	-6.748420960	-7.166927134
N420	3.618699320	-5.560429885	-4.905382520
N421	2.750136519	-6.507436073	-6.697041854
N422	8.760709445	-7.201353591	-4.409153859
N423	-0.137524242	2.365985219	-0.210496544
N424	0.592195995	1.737003860	-0.802921661
O425	-6.193288009	-5.420317073	2.679230526
O426	-6.173850148	-2.918436591	5.439561167
O427	-3.872829502	-5.010564176	7.387578114
O428	-1.316069364	-3.881143009	5.003583777
O429	-1.813636089	-0.448417879	5.448647639
O430	-1.397958295	-0.795508012	9.039961131
O431	1.252658302	-3.877594728	9.487559099
O432	3.381889340	-3.527063578	5.672107635
O433	3.365632521	0.176961420	5.340198935
O434	3.051759977	1.714259618	8.746680517
O435	3.892296339	-0.078047854	11.578203316
O436	7.547771208	-10.153553754	1.055164332
O437	8.920299792	-7.389071284	4.501205697
O438	12.339072005	-4.337819346	2.786835639
O439	-5.612507491	-1.045242218	1.039567290
O440	-4.256856762	-4.532278203	-3.293736797
O441	-7.282594138	2.281384898	0.822009817
O442	-6.945630938	2.838530067	-2.191036144
O443	-9.412049434	1.159917116	3.904568680
O444	-4.923855848	1.377533965	5.359142202
O445	-3.879078943	4.980685471	4.476899254
O446	-6.055217684	7.029592648	6.423845290
O447	11.226337722	0.167101376	2.639417952
O448	3.957323167	4.788601789	3.430927084
O449	3.172971017	8.452063881	-1.189823633
O450	0.132797551	10.034484096	-3.526303815
O451	-1.877616666	7.148414369	-1.263652122
O452	-0.603244552	7.290205782	2.269370760
O453	2.385368413	5.803779384	1.174456629
O454	0.822496531	11.710455914	2.093013437
O455	-2.847514470	13.533192889	0.950181866
O456	-3.859447530	10.891922561	-0.833545852
O457	-4.747254653	13.609204009	4.417235529
O458	-5.928093066	6.665601269	-0.382100149
O459	0.450352231	-4.505852823	-8.868769967
O460	-1.633868505	-0.777533969	-8.255237078
O461	0.586317244	3.643884078	-8.566032100
O462	4.388766658	4.288414642	-9.668187701
O463	-6.274821278	-2.834340266	-7.803891951
O464	-2.186636059	-6.701618870	-5.626270289
O465	-0.476501939	-5.795744318	-6.736254137
O466	-5.348049862	2.167174805	-7.526370156
O467	9.338388461	-5.154521912	-5.191902356
O468	12.282751264	-6.638582497	-4.032002354
O469	0.163667289	-6.361270850	-1.343050465
O470	-0.046917101	-7.394655946	0.641884201
O471	4.763387960	-10.868893901	-4.041138518
O472	2.513349456	-10.925669289	-3.816846359
O473	4.891427378	-5.818834914	-2.120433388
O474	5.094699108	-7.488647674	-0.631572982
O475	2.318908038	-6.122808020	-2.441538200
O476	9.145194262	-6.355674937	-0.333113499
O477	7.053860176	-4.487296198	-0.797374298
O478	-4.917646885	4.604637825	-1.665932596
O479	7.420655003	-10.255618966	-3.631307597
O480	7.576922639	-8.474788056	-1.189819216
S481	2.959107993	4.984814600	-2.771960119
S482	1.429373536	1.974871999	-3.625906570
S483	1.461634395	-3.310519123	-3.621540528
S484	3.461505971	1.764908051	-0.602470572
S485	0.146452250	-0.966834181	-1.756551205
S486	3.094809803	-0.706783243	-5.928108679
S487	3.473830364	-3.698697883	-0.648037269
S488	5.024016080	1.817154131	-3.819720491
S489	5.163398697	-2.952455250	-3.747192037
S490	6.218485551	-0.783554182	-1.261128591

	end

product

Fe(139) -2.408<br>Fe(140) -1.000<br>Fe(141) -2.576<br>Fe(142) 2.721<br>Fe(143) 2.747<br>Fe(144) 1.339<br>Fe(145) 0.104	bm522bh2n2x6x6h2n3b6ba.car_3

```
C1      -7.688839219   -5.447916436    2.139963084
C2      -8.279802909   -6.553591871    1.259298727
C3      -8.162979272   -3.454960678    3.485484167
C4      -7.296633587   -3.721238851    4.712224608
C5      -6.650873233   -5.269994596    6.486398691
C6      -5.147750242   -5.330488672    6.207422698
C7      -3.353849280   -5.676876362    4.594823881
C8      -2.791858520   -4.258885379    4.468974305
C9      -3.256393907   -1.943151830    3.826669255
C10     -3.010186898   -1.301013741    5.199479476
C11     -3.736167894   -1.053133806    7.501894854
C12     -2.594998599   -1.675430014    8.318996609
C13     -1.627818597   -3.734372639    9.226909681
C14     -0.232686788   -4.053177780    8.671816955
C15      1.069107131   -4.754077412    6.702205760
C16      1.782565207   -3.678920815    5.877933969
C17      1.544731916   -1.614925196    4.525779240
C18      1.953415650   -0.433226822    5.434401916
C19      0.546257247   -1.191718566    3.427835137
C20      1.011231391    0.055667046    2.662715450
C21      0.284975371   -2.365461024    2.478194391
C22      1.196645659    1.301296563    7.026012146
C23      1.701185012    0.911684230    8.430528103
C24     -0.058873482    2.208374847    7.077752596
C25      0.128167553    3.352252140    8.083172483
C26     -0.382872624    2.766267376    5.685682150
C27      1.336991285   -0.304652775   10.500386515
C28      2.801733322   -0.645612045   10.655154491
C29      6.334040687  -11.687930089    2.977846331
C30      6.808343562  -10.387254033    2.326664520
C31      7.075182325   -7.942183226    2.752181022
C32      8.382639608   -7.582803400    3.484838629
C33      5.944603983   -6.958984108    3.106599317
C34      6.268157944   -5.509371881    2.737798589
C35      5.036109134   -4.608749242    2.848829375
C36      5.141257122   -2.089423730    2.962974201
C37     10.662032378   -6.722524117    3.231933728
C38     10.939651154   -5.236244753    3.207623585
C39     -5.603762713   -2.681856124   -0.917757430
C40     -5.903791271   -1.261205829   -0.400770490
C41     -4.067895426   -2.822303785   -1.037571825
C42     -3.529741155   -4.251772290   -1.254308102
C43     -3.425516814   -4.700486214   -2.711899425
C44     -6.453180077    1.013040589   -1.194851647
C45     -7.436560575    1.453044386   -0.101498479
C46     -6.757438062    1.650908240   -2.558713269
C47     -9.447423825    0.887705266    1.180513751
C48     -8.867873796    0.892996369    2.599997134
C49     -6.954247551    0.373177606    4.045709113
C50     -5.761391124    1.324927327    4.124933371
C51     -4.380522775    3.039036893    3.063008766
C52     -4.764619182    4.322890559    3.837762214
C53     -3.950286927    3.501726515    1.662975565
C54     -3.445538570    2.450114330    0.731540989
C55     -3.010778311    1.159464815    0.896354821
C56     -2.857671174    1.652295503   -1.271849426
C57     -6.573342119    5.954143138    4.217001076
C58     -6.338423909    6.057506644    5.723497887
C59     -8.085050599    6.018818709    3.897904936
C60     -8.697485851    7.388424527    3.865207794
C61     -8.492972102    8.488295406    4.676395652
C62    -10.011600899    8.940326184    3.127165335
C63      9.519474093    1.515715691    2.527230291
C64     10.523233107    0.415709299    2.234883251
C65      8.108835286    0.878414419    2.459523969
C66      7.011499679    1.857139907    2.804237633
C67      6.602864545    2.057996601    4.136346550
C68      6.369403627    2.601912780    1.799809704
C69      5.580774635    2.959566000    4.455207803
C70      5.358536106    3.514936437    2.103903779
C71      4.958910117    3.685354303    3.432974898
C72      5.142937490    6.844760404   -1.777541778
C73      4.074936673    7.948454969   -1.869528862
C74      4.510049913    5.529398293   -1.330124152
C75      2.914185780    9.558861126   -3.331943576
C76      1.429322888    9.189980788   -3.306101903
```

	C77	-0.188778739	7.308795099	-3.135451673
	C78	-0.500664501	6.957210156	-1.658092096
	C79	-0.270856261	6.066197720	-4.052789970
	C80	-1.540011674	5.206626212	-4.028224161
	C81	-2.767564688	5.765180143	-4.768675978
	C82	-4.221136771	7.677943925	-4.073773277
	C83	0.241384977	5.998231590	0.465035222
	C84	0.208644419	7.222193207	1.392356242
	C85	1.281278459	5.009430103	1.030236042
	C86	1.403249844	9.268833595	2.001505180
	C87	0.708608701	10.607139480	1.800331231
	C88	-0.894183501	11.876757041	0.480035775
	C89	-2.079846233	12.429300852	1.314622379
	C90	-1.240964427	11.908487312	-1.010962657
	C91	-2.422790602	11.023503010	-1.380945194
	C92	-3.835292087	11.940875546	2.963732180
	C93	-3.623912184	13.006295648	4.030198566
	C94	-4.421365170	10.650456189	3.607388000
	C95	-4.784892229	9.598179368	2.583782378
	C96	-6.059132216	9.587655502	1.993118732
	C97	-3.853400985	8.635571136	2.159431940
	C98	-6.385795480	8.665948770	0.994353664
	C99	-4.151578913	7.722642790	1.142082270
	C100	-5.419948842	7.754117546	0.566522921
	C101	3.011470895	-3.735344948	-9.335396281
	C102	1.636030766	-3.515228983	-8.731906233
	C103	0.307650023	-2.043437760	-7.237827999
	C104	-0.234530375	-0.676406207	-7.670622976
	C105	0.187404828	1.739465881	-7.638094714
	C106	1.337372955	2.708939743	-7.894861615
	C107	3.668703722	3.342550819	-7.590977100
	C108	4.167474552	3.483661787	-9.016317225
	C109	-3.823287130	-3.305211815	-8.747056511
	C110	-4.807546293	-2.234451050	-8.262438000
	C111	-3.195002514	-4.025511939	-7.531182155
	C112	-2.524596603	-5.346897351	-7.952629403
	C113	-1.692505004	-5.899428090	-6.818734498
	C114	-5.138887928	0.071415383	-7.518724882
	C115	-4.730209222	1.370282115	-8.185868835
	C116	-5.335237307	0.168041276	-5.984930768
	C117	-4.009131359	0.145733459	-5.280276817
	C118	-3.249946318	1.320042298	-5.142312531
	C119	-3.434057728	-1.084130872	-4.911932434
	C120	-1.914544052	1.251734739	-4.733147289
	C121	-2.099434057	-1.150525949	-4.507247787
	C122	-1.327272842	0.013497717	-4.458923372
	C123	7.235772774	-6.101774007	-5.718130252
	C124	8.526819319	-6.219909518	-4.893386819
	C125	6.016973005	-6.522061521	-4.884373490
	C126	4.674521193	-6.270907854	-5.494666182
	C127	4.169786154	-6.804219923	-6.662525740
	C128	2.561770051	-5.706796141	-5.608155952
	C129	9.917177741	-7.494517677	-3.371658987
	C130	11.179822641	-7.244914002	-4.166827002
	C131	0.477351698	-6.995941842	-0.374193533
	C132	1.995243825	-7.065083568	-0.246600384
	C133	2.758298661	-7.148207165	-1.600768383
	C134	2.602064461	-8.567943058	-2.207714719
	C135	3.509138082	-8.874119686	-3.402361841
	C136	3.495214430	-10.356949049	-3.833290809
	C137	4.256400638	-6.911099456	-1.358053035
	C138	3.207184733	-0.692539660	-2.439409591
	Fe139	3.335821017	-1.940862060	-3.984995856
	Fe140	1.813979871	0.704152305	-1.678701585
	Fe141	4.752476208	0.419178418	-1.832104414
	Fe142	3.303538045	0.580848335	-3.966489481
	Fe143	3.464920964	2.688988056	-2.362286017
	Fe144	4.397456679	-2.084516054	-1.680236559
	Fe145	1.846997219	-2.464807097	-1.521170102
	H146	-6.554334044	-7.584499052	0.907866540
	H147	-6.947064165	-6.550106973	-0.289130368
	H148	-9.580620747	-4.732331109	2.529843321
	H149	-9.110367670	-6.132042133	0.670501218
	H150	-8.734850122	-7.299694291	1.932985874
	H151	-7.573145475	-2.798815652	2.828394376
	H152	-8.131654140	-5.572439786	4.954721585
	H153	-9.054457709	-2.900469539	3.808233825
	H154	-6.982244956	-6.251469032	6.848815546
	H155	-5.462325577	-5.849373283	4.230077234
	H156	-6.789105997	-4.543885891	7.299042074

H157	-2.337377974	-1.813894421	3.244014452
H158	-4.558314202	-3.627879926	3.639327410
H159	-3.226277857	-6.198582739	3.636684953
H160	-3.531913282	0.021536402	7.421921414
H161	-4.719151686	-2.175479413	5.946794610
H162	-2.754621496	-6.197070857	5.351970171
H163	-4.070834649	-1.417347448	3.309843256
H164	-3.259547512	-3.578329990	7.851280163
H165	-4.679968093	-1.196291600	8.045563140
H166	-0.169399839	-3.226454382	2.985669131
H167	-0.390975570	-2.065483946	1.661745314
H168	1.226271333	-2.712190385	2.025885065
H169	-0.394284368	-0.932348598	3.939659343
H170	-2.094175271	-4.686043150	9.522269958
H171	-0.570032975	1.968985697	4.953205625
H172	0.074046196	-0.344379881	8.787613339
H173	0.282854189	2.986584299	9.108438592
H174	1.746614506	-0.187690846	1.879635936
H175	1.472758431	0.811008228	3.316732325
H176	9.690292577	1.849728218	3.568526395
H177	-1.280772164	3.400787724	5.728955144
H178	-0.762895281	3.997379207	8.084951552
H179	0.451254912	3.386177800	5.317759407
H180	0.996578037	3.973016436	7.810222329
H181	-0.909443967	1.588868236	7.415594833
H182	2.028140038	1.867037424	6.582521133
H183	0.001567914	-0.194430983	6.054598183
H184	0.145021618	0.535647271	2.177368081
H185	1.480415126	4.231753937	0.278063595
H186	0.823891623	4.534846404	1.911730250
H187	2.857381014	5.253222147	2.190110035
H188	3.739728567	4.607072780	4.630604671
H189	4.860354510	4.076148083	1.313417917
H190	6.643301796	2.445241857	0.753677870
H191	7.077440613	1.487245278	4.938984305
H192	-0.013300360	-2.812904174	5.392253899
H193	0.875353728	-5.607187095	6.033869662
H194	1.773575079	-5.080915348	7.476370973
H195	-1.013990112	-4.322023563	6.771547222
H196	-1.470380361	-3.133778125	10.131076531
H197	5.259565256	3.083958048	5.492937899
H198	2.469732799	-1.969990613	4.059503528
H199	1.083276046	0.518146430	11.190336221
H200	0.766140439	-1.192306838	10.805560621
H201	3.288415122	-1.105990299	9.764322523
H202	11.472377800	-7.253341594	2.708503309
H203	-2.324699419	-5.900265390	-3.953469169
H204	0.914078303	-6.111945145	-2.032130386
H205	-3.301895550	-6.083143445	-8.200767901
H206	-1.898234426	-5.188133335	-8.842096253
H207	-3.965819800	-4.240152459	-6.775185631
H208	-4.256636596	1.238404322	-9.186893272
H209	-4.466780077	-4.035490712	-9.261814789
H210	-3.725032455	-2.172003266	-1.860030031
H211	-4.172960362	-4.983623793	-0.735744878
H212	-3.274673528	-0.850267963	-8.051453248
H213	-6.135417799	-0.145168138	-7.956494904
H214	-2.155845762	-2.202174720	-9.314289980
H215	-1.702203032	-5.814239680	-2.313348193
H216	-2.464833885	-3.352704933	-7.054230338
H217	-2.430283555	-3.540237888	-10.235685660
H218	-7.155483638	-3.406317850	-2.073556710
H219	-3.634125869	-2.441517378	-0.100043579
H220	-0.102643232	-5.126993437	-7.683057912
H221	-2.541212314	-4.339245788	-0.780016198
H222	-3.534426549	2.600602836	3.609903379
H223	-2.932430950	0.544764027	1.783122025
H224	-7.676734379	0.704571875	4.804789031
H225	-7.150303533	-0.102975974	1.976161063
H226	-6.719534081	4.035646001	3.279829009
H227	-7.831782675	8.663655191	5.517914104
H228	-6.428556333	5.082119516	6.262013912
H229	-8.628321797	5.363037309	4.601603596
H230	-6.040700999	6.781014842	3.721776398
H231	-10.743863699	9.508897988	2.559778627
H232	-3.164244233	4.261648286	1.802266229
H233	-4.803521674	4.005318532	1.180071985
H234	-9.430943321	10.406793574	4.556546139
H235	-8.233496017	5.585032554	2.896133156
H236	-6.064395516	2.020309621	2.182902291

H237	-6.608327441	-0.626641305	4.348444971
H238	-9.928967384	1.865410074	1.042011567
H239	-2.657551978	1.561936598	-2.337128039
H240	-10.230474796	0.120387306	1.128603049
H241	10.506713894	3.116727986	1.715859177
H242	8.880376616	3.223373606	1.597711367
H243	1.334930618	5.981051203	-1.396888101
H244	-0.750711076	5.529203858	0.541254887
H245	1.729281695	8.062646160	0.296764373
H246	-3.705948905	3.600385381	-1.079670859
H247	-5.468182214	4.215370819	-2.038482404
H248	-7.233451964	3.444374235	-3.196067144
H249	-5.051526588	5.452711270	-1.124151531
H250	-5.989419523	1.312097958	-3.273783019
H251	-7.744511225	1.320219214	-2.914598528
H252	-0.349360992	11.663822951	-1.604180611
H253	-1.527121602	12.945484594	-1.246665428
H254	-1.292945582	10.128304987	-2.865018976
H255	-2.940358674	9.542960392	-2.641815243
H256	-4.249844720	9.356155434	-5.256778159
H257	-3.317418687	4.945920335	-5.255040297
H258	-7.384543234	8.663843505	0.550217690
H259	-2.866120525	8.576617180	2.625643667
H260	-5.070237717	7.781706573	-2.194825174
H261	0.574020509	5.408529945	-3.789479824
H262	1.936423942	7.215715937	-3.294585966
H263	-1.815670624	4.939665564	-2.995742696
H264	-8.555716656	-0.223308176	-0.425770945
H265	3.128583965	10.016604212	-4.307083830
H266	-6.487616247	-0.912943828	-2.272038159
H267	-0.085138846	6.406726562	-5.084545908
H268	-3.389230703	7.027821018	0.787699181
H269	3.055681122	10.328643206	-2.560193967
H270	-3.570564547	7.951099262	-6.012303392
H271	-5.562966213	9.019425534	-3.303864743
H272	-5.660164924	-3.469070879	-2.820630323
H273	-5.942628065	-3.369530125	-0.121359220
H274	-6.815203340	10.306713662	2.316989016
H275	-5.483395324	1.392193220	-0.844064073
H276	-0.918634981	8.067411591	-3.432435267
H277	-2.434468153	6.438453755	-5.568404015
H278	-1.271285455	4.263393210	-4.523566415
H279	-4.046186716	5.918965238	-3.073183982
H280	1.171243236	8.923277727	3.019817284
H281	-0.497244360	9.763508833	0.380507406
H282	-0.103044750	12.625631759	0.643373022
H283	2.483802330	9.458268337	1.947690056
H284	-2.255650311	10.660392360	2.292443034
H285	3.506335734	-2.766866385	-9.499016218
H286	3.598410493	-4.266082154	-8.557616686
H287	7.150991811	-5.033342382	-5.966400030
H288	3.828559731	-4.806223165	-10.865657942
H289	2.311505928	-5.276503433	-10.468531171
H290	5.307166529	4.777474647	-1.266880725
H291	5.762276543	7.465175998	0.066701698
H292	4.375030159	8.105908295	-3.901348128
H293	4.047399574	5.657683459	-0.341617928
H294	6.683756006	8.052984297	-1.148558233
H295	4.510330670	2.989135370	-6.974461508
H296	5.609115234	6.700113450	-2.764772196
H297	5.728978162	-7.041327552	4.184915038
H298	7.239554898	-7.929259761	1.667396146
H299	2.770826335	1.516560986	-6.955970960
H300	5.352245706	-0.065930488	2.739449655
H301	-0.429075188	1.712551921	-8.545108794
H302	-1.654766142	-2.112301123	-4.241293852
H303	-0.435250556	2.202685907	-6.853433734
H304	4.685560210	-4.547507130	3.885455276
H305	1.295690650	0.172156359	-6.627044677
H306	4.207181694	-5.034630356	2.259184561
H307	5.048167444	-7.293112965	2.559997762
H308	6.630518770	-5.462839565	1.700256676
H309	7.068320360	-5.109417717	3.381091423
H310	-5.959257632	-0.681586007	-5.672049738
H311	6.373358107	-9.501103966	4.067129600
H312	5.371554452	-11.929856674	2.501379791
H313	-0.274640710	-0.022663169	-4.185028861
H314	6.994866308	-11.826528919	4.923619731
H315	5.420797168	-12.237234551	4.736872509
H316	5.603939694	-3.219850683	1.357148963

	H317	-1.307248439	2.154349520	-4.647133292
	H318	-4.027070390	-1.999590398	-4.967833397
	H319	7.289403949	-9.926261590	-2.681438503
	H320	6.085606186	-7.603277100	-4.672598325
	H321	6.483704144	-8.233542631	-0.968967522
	H322	6.301086167	-10.511019259	-3.734458208
	H323	3.857810879	2.663018460	-9.707584595
	H324	6.256707749	-5.155998118	-1.110586182
	H325	2.264533034	-7.902566420	0.408308526
	H326	2.343855292	-1.848876993	-7.774778988
	H327	2.825159175	-9.279152152	-1.395951978
	H328	2.165019745	-6.724723962	-7.412291154
	H329	1.572860135	-5.325538449	-5.389389887
	H330	7.045199510	-12.472358497	2.668516837
	H331	5.608383241	-0.968651119	1.268348241
	H332	8.359750533	-5.857428138	-0.271273297
	H333	7.071501045	-3.834497612	-1.072140593
	H334	9.194346253	-6.942354673	1.673474442
	H335	4.469109767	-2.824524344	4.760319911
	H336	3.191794333	-8.272228156	-4.267651571
	H337	4.547034262	-8.591653224	-3.178742666
	H338	1.547690043	-8.717846173	-2.487058188
	H339	0.533692053	-2.042739512	-6.159689492
	H340	-3.697668041	2.278857059	-5.413440956
	H341	-5.889601204	1.098820050	-5.790512220
	H342	2.290193737	-6.136571255	0.270526717
	H343	8.465541843	-7.392140965	-0.482915022
	H344	7.367340585	-9.304864103	-0.290051231
	H345	4.438420234	-1.136281348	4.631094354
	H346	4.635550006	-7.427604065	-7.417746442
	H347	6.059547696	-6.021017417	-3.911017779
	H348	-0.457529881	-2.794356446	-7.445163200
	H349	3.370068248	4.332722409	-7.215511353
	H350	8.092509978	0.028195630	3.157473189
	H351	7.952702800	0.481202564	1.443592030
	H352	8.116232627	-6.499022425	-7.527782474
	H353	7.506619732	-7.833868160	-6.802332722
	H354	9.961245881	-8.532708543	-3.006395549
	H355	8.040013107	-8.127028247	-4.161066014
	H356	11.099847844	-7.473977482	-5.256671556
	H357	9.894417542	-6.832048133	-2.491829600
	H358	10.664207796	0.199155669	1.148477087
	H359	-2.562378570	13.175006374	4.329591689
	H360	10.677819945	-7.036659781	4.291056005
	H361	10.034465303	-4.582300344	3.218650074
	H362	-4.560826131	12.343295052	2.237727079
	H363	-3.687329632	10.246807450	4.322705005
	H364	-5.309008487	10.948072149	4.183543870
	H365	0.838635270	-3.484682563	-1.066700987
	H366	4.245210931	-1.518697987	-0.308525306
	H367	-6.667708842	6.841047645	-0.621325316
	H368	-0.185055737	-0.859067387	-0.477287807
	H369	2.036354287	-1.586446196	-0.167122629
	H370	1.540298061	-2.277726425	0.019011910
	H371	-2.269392794	-0.221162036	-0.577538935
	Mo372	3.439083040	-4.391095734	-2.803259785
	N373	-7.346262052	-7.223983969	0.369005650
	N374	-8.596500364	-4.648382221	2.774500187
	N375	-7.457386097	-4.911097328	5.332421651
	N376	-4.762405767	-5.700928340	4.959650966
	N377	-3.614525957	-3.349505019	3.896405102
	N378	-3.886062189	-1.629487791	6.175476989
	N379	-2.545072875	-3.031932527	8.341051950
	N380	-0.169223545	-4.352002414	7.345195089
	N381	1.000299480	-2.723221341	5.316524944
	N382	0.963371001	0.132965841	6.170925612
	N383	0.979289360	-0.004672777	9.122405906
	N384	6.135941318	-11.577079682	4.428517209
	N385	6.685506951	-9.298151345	3.111801459
	N386	5.309283666	-3.263420654	2.334352424
	N387	4.864710521	-2.009644328	4.280121130
	N388	5.279029592	-0.954771234	2.250673406
	N389	9.362173579	-7.075759189	2.695034885
	N390	-6.273881816	-2.905998520	-2.203084130
	N391	-2.416459874	-5.543561985	-2.996831024
	N392	-6.375243064	-0.427697535	-1.364780876
	N393	-8.493314308	0.637255897	0.113349488
	N394	-7.645025297	0.314780299	2.771646408
	N395	-5.459250682	2.059854652	3.008913046
	N396	-3.336080887	2.722250574	-0.625220182

	N397	-2.666052365	0.692747782	-0.357212521
	N398	-6.072163314	4.672914120	3.737253829
	N399	-9.648415099	7.683415100	2.901529556
	N400	-9.337305900	9.463664776	4.189139794
	N401	9.664765554	2.569598946	1.520549789
	N402	6.203864870	7.208921552	-0.822599876
	N403	3.841469557	8.462865692	-3.113142952
	N404	1.147359237	7.870362934	-3.296018205
	N405	-3.703243652	6.465956583	-3.882545062
	N406	-4.049457075	8.361311654	-5.219223430
	N407	-4.904832350	8.279506689	-3.072825920
	N408	0.482796676	6.347782826	-0.936630904
	N409	1.052042923	8.234119818	1.046092229
	N410	-0.343444460	10.611630102	0.925316125
	N411	-2.244105316	10.273063549	-2.502417892
	N412	-2.586219004	11.621723047	2.279529767
	N413	2.904190408	-4.453311222	-10.608520805
	N414	1.505965582	-2.400386531	-7.983381483
	N415	0.526263228	0.373032423	-7.286637335
	N416	2.582524248	2.385071135	-7.463656894
	N417	-2.892880834	-2.770928136	-9.747634297
	N418	-4.283141249	-1.010308011	-7.968664770
	N419	7.316262941	-6.844442935	-6.989742060
	N420	3.652138276	-5.571005728	-4.852377151
	N421	2.842861448	-6.436820142	-6.711518165
	N422	8.725478814	-7.368588261	-4.192178564
	N423	-0.085940121	2.346245619	-0.142849324
	N424	0.655283573	1.720067598	-0.724381094
	O425	-6.466253100	-5.293100139	2.304820541
	O426	-6.484999603	-2.862670152	5.105393635
	O427	-4.334041867	-5.066210275	7.108450168
	O428	-1.647225828	-3.967549631	4.865018314
	O429	-2.056928305	-0.525248376	5.378925963
	O430	-1.800444027	-0.958446216	8.952301137
	O431	0.740439453	-4.082459164	9.436577553
	O432	3.016369713	-3.745739714	5.729257531
	O433	3.128774879	-0.019710986	5.458982600
	O434	2.721009233	1.436954498	8.908611226
	O435	3.407182716	-0.491511346	11.706528125
	O436	7.234740613	-10.396952085	1.153401003
	O437	8.492981792	-7.760226293	4.710673837
	O438	12.064955111	-4.761449663	3.195983173
	O439	-5.704102248	-0.908474480	0.778685141
	O440	-4.247824797	-4.327926090	-3.580371317
	O441	-7.232446818	2.489285075	0.555628629
	O442	-6.738824332	3.086362508	-2.437593062
	O443	-9.524919455	1.402070710	3.517333578
	O444	-5.108117506	1.407981932	5.170453792
	O445	-3.916513637	5.009944917	4.414695364
	O446	-6.114492277	7.098760673	6.314989690
	O447	11.126737866	-0.212082631	3.092810358
	O448	3.928763685	4.575354090	3.675016030
	O449	3.465977079	8.366649390	-0.868859349
	O450	0.570635542	10.093330834	-3.310521072
	O451	-1.613225539	7.230017485	-1.178100769
	O452	-0.497968948	7.232040594	2.408789653
	O453	2.491754842	5.680574332	1.389055035
	O454	1.082367123	11.613722832	2.414029111
	O455	-2.490876681	13.582244758	1.121322643
	O456	-3.479544955	11.025759433	-0.733763313
	O457	-4.545609486	13.618814665	4.544823385
	O458	-5.699698742	6.861114993	-0.490152998
	O459	0.711396238	-4.346187041	-8.906680146
	O460	-1.282015606	-0.561828094	-8.339306240
	O461	1.088603693	3.792951868	-8.448121703
	O462	4.878630811	4.402369216	-9.387153261
	O463	-6.016564470	-2.483564835	-8.122840848
	O464	-2.168681673	-6.512960558	-5.869126286
	O465	-0.370068437	-5.639284765	-6.858575928
	O466	-4.933300905	2.483263726	-7.722888078
	O467	9.390179457	-5.320944811	-4.900136593
	O468	12.239894690	-6.895631674	-3.666470969
	O469	0.027910785	-6.354983565	-1.460543757
	O470	-0.282727351	-7.443192380	0.482796737
	O471	4.624599475	-10.900077669	-4.097003620
	O472	2.369602204	-10.925064978	-3.920301390
	O473	4.807667390	-5.933082899	-2.032297157
	O474	4.890818026	-7.646339947	-0.579439292
	O475	2.244912831	-6.129172337	-2.462245888
	O476	8.958366197	-6.622677634	-0.084526177

	O477	6.950224960	-4.676266404	-0.592120513
	O478	-4.702080874	4.796414595	-1.778047289
	O479	7.279218501	-10.394693024	-3.545376209
	O480	7.370393166	-8.676551866	-1.055449768
	S481	3.203916779	4.950303529	-2.508136356
	S482	1.620779644	2.010745864	-3.501428094
	S483	1.533350688	-3.265057093	-3.633084301
	S484	3.508616487	1.650996944	-0.396016593
	S485	0.177152463	-0.947013488	-1.788327648
	S486	3.347438651	-0.668581933	-5.800820366
	S487	3.422519677	-3.739796345	-0.582156932
	S488	5.208375376	1.758484544	-3.528500949
	S489	5.226197901	-3.047972107	-3.599663004
	S490	6.264086654	-0.957336796	-1.016469102
	end			

S2BH-6Hx-6H2n-7Hn + N2 to pre-capture

35, S=1/2

reactant

	bm52etc6hx7hnn2tum6h2ndtf.car_3			
Fe(139) -2.396	C1	-7.842258365	-5.365623110	0.071858537
Fe(140) -0.893	C2	-8.109076982	-6.503882097	-0.919943997
Fe(141) -2.550	C3	-8.815277383	-3.440745602	1.246598721
Fe(142) 2.688	C4	-8.365726758	-3.710484191	2.679045972
Fe(143) 2.689	C5	-8.193030502	-5.301097663	4.524928935
Fe(144) 1.284	C6	-6.673300868	-5.395244939	4.694317856
Fe(145) 0.101	C7	-4.492073006	-5.687165115	3.639319740
	C8	-3.980236477	-4.245237551	3.741649711
	C9	-4.350608507	-1.933627240	2.981584542
	C10	-4.579774987	-1.308691036	4.365241023
	C11	-5.917718784	-1.175832788	6.369083769
	C12	-5.015042234	-1.741384519	7.474840174
	C13	-4.182412409	-3.772283909	8.576796135
	C14	-2.684284342	-4.096681371	8.471284140
	C15	-0.852100105	-4.896722072	7.032965293
	C16	-0.042930907	-3.732682062	6.449088562
	C17	-0.216489810	-1.520171696	5.358271775
	C18	-0.216119469	-0.318526640	6.327355245
	C19	-0.889959530	-1.297024996	3.979730582
	C20	-0.677972452	0.096019522	3.358530354
	C21	-0.478531605	-2.410580554	3.008845300
	C22	-1.478943355	1.461061367	7.454554137
	C23	-1.320872079	1.136447034	8.953345812
	C24	-2.765116305	2.265054295	7.142585783
	C25	-2.961325304	3.421198382	8.130024420
	C26	-2.734673498	2.790394688	5.701285327
	C27	-2.039447633	-0.173840901	10.869695263
	C28	-0.628450097	-0.421420964	11.364459301
	C29	5.266481311	-11.626483110	4.492507484
	C30	5.959592051	-10.350039942	4.032132510
	C31	6.159731023	-7.924848959	4.573071321
	C32	7.234141911	-7.522855958	5.600017504
	C33	4.939291789	-6.991958959	4.639884641
	C34	5.290339469	-5.541338470	4.329932464
	C35	4.048130021	-4.678641762	4.112107763
	C36	4.302832826	-2.184338051	4.251651919
	C37	9.481228494	-6.594074441	5.897689394
	C38	9.717280345	-5.100475222	5.918048216
	C39	-5.048694081	-2.652895101	-2.354828383
	C40	-5.475631649	-1.224153916	-1.969318731
	C41	-3.533095540	-2.798241817	-2.082624693
	C42	-2.982410769	-4.238517879	-2.125415679
	C43	-2.508589525	-4.719773231	-3.496419794
	C44	-5.827558016	1.023589000	-2.917121539
	C45	-7.053273548	1.467432852	-2.108593333
	C46	-5.794203564	1.645244806	-4.319863176
	C47	-9.338953074	0.937183788	-1.398444909
	C48	-9.164030841	0.959435742	0.122815771
	C49	-7.726866065	0.433380765	2.036742498
	C50	-6.593482736	1.384536725	2.425294551
	C51	-4.921155855	3.034186850	1.741545717
	C52	-5.462224786	4.336791373	2.374678870
	C53	-4.145006408	3.483838219	0.494645708
	C54	-3.409890253	2.428505335	-0.260863695
	C55	-3.048014873	1.135720873	0.021105580
	C56	-2.302064150	1.615739803	-2.024236520

C57	-7.256106395	6.016014941	2.223685314
C58	-7.390219874	6.163574369	3.738464124
C59	-8.644170929	6.079177636	1.541085213
C60	-9.251415664	7.438831101	1.356546318
C61	-9.286211236	8.539639420	2.189652234
C62	-10.370800769	8.964466586	0.305417088
C63	8.631455604	1.606442281	4.742440965
C64	9.665270085	0.494356473	4.714523160
C65	7.279799421	0.964977304	4.346946435
C66	6.122307779	1.933871472	4.401832761
C67	5.380905590	2.125368514	5.584484276
C68	5.764135413	2.689780232	3.272141878
C69	4.311128684	3.026751408	5.634897065
C70	4.711369639	3.604286656	3.313104356
C71	3.981013983	3.768503411	4.494708458
C72	5.506617296	6.866361133	-0.550943614
C73	4.492372232	7.968501800	-0.899158440
C74	4.798010544	5.525104370	-0.351722865
C75	3.734469855	9.566158558	-2.625171528
C76	2.300602180	9.186385223	-3.003010574
C77	0.695958216	7.294213944	-3.213448130
C78	0.019581272	6.941236847	-1.863189060
C79	0.860863899	6.052092156	-4.120220090
C80	-0.361676437	5.178930479	-4.424107211
C81	-1.367056361	5.722775421	-5.452173813
C82	-2.944203666	7.639642142	-5.186255699
C83	0.204643619	5.978103359	0.378452598
C84	-0.086420752	7.197686760	1.265204347
C85	1.085966067	5.004946487	1.188439881
C86	0.887622943	9.249357075	2.178951670
C87	0.301404667	10.596263640	1.782646783
C88	-0.873574128	11.872506521	0.070414337
C89	-2.230118666	12.424413146	0.570491492
C90	-0.813614325	11.892449251	-1.459399301
C91	-1.869602249	11.022565287	-2.129258810
C92	-4.359504190	11.933287274	1.679597262
C93	-4.432557488	13.030117218	2.733590810
C94	-5.095235946	10.658250372	2.179923206
C95	-5.186515859	9.592581439	1.109600878
C96	-6.255488983	9.594864971	0.198638099
C97	-4.186534109	8.619669587	0.951553344
C98	-6.318689525	8.671579614	-0.847677590
C99	-4.217202594	7.703855538	-0.106134481
C100	-5.284390372	7.747147459	-0.999520315
C101	5.359409844	-3.914036247	-8.348771986
C102	3.881206555	-3.670996517	-8.101445908
C103	2.248424006	-2.150527613	-7.006281771
C104	1.827147724	-0.786194928	-7.566028863
C105	2.230510946	1.635235194	-7.436093609
C106	3.414805498	2.599223545	-7.464093502
C107	5.603403323	3.271446484	-6.638780310
C108	6.413048895	3.428149395	-7.910443329
C109	-1.395700782	-3.420189601	-9.496665070
C110	-2.460272493	-2.335146370	-9.307800304
C111	-1.108705150	-4.109483390	-8.142012899
C112	-0.337974940	-5.426954369	-8.340438083
C113	0.155286122	-5.962324599	-7.014844916
C114	-2.957596224	-0.022899238	-8.695678430
C115	-2.396028269	1.268049777	-9.257422798
C116	-3.550701910	0.099291633	-7.270785872
C117	-2.452959328	0.103128149	-6.245097769
C118	-1.763534599	1.286492156	-5.931757318
C119	-1.986253552	-1.112694259	-5.716938371
C120	-0.591551006	1.240612242	-5.171383380
C121	-0.815051442	-1.158066134	-4.957992553
C122	-0.094262147	0.016659991	-4.717986243
C123	8.518119973	-6.203505870	-3.613538103
C124	9.550544648	-6.305219237	-2.478982057
C125	7.117637172	-6.575092219	-3.103186440
C126	5.978420422	-6.329794728	-4.039048270
C127	5.782592604	-6.895075072	-5.281494468
C128	3.968658584	-5.768120647	-4.698977010
C129	10.495468059	-7.550725599	-0.625574730
C130	11.923568008	-7.307440770	-1.062332375
C131	0.610155817	-6.924437395	-0.155225505
C132	2.040187581	-6.980130667	0.370970880
C133	3.126301718	-7.090492832	-0.740239078
C134	3.119533013	-8.521490091	-1.345538859
C135	4.306799243	-8.863749661	-2.249656767
C136	4.386762865	-10.354363149	-2.650031862

	C137	4.511782907	-6.846600418	-0.122539699
	C138	3.873188502	-0.684023023	-1.699729873
	Fe139	4.347430957	-1.993627504	-3.089684095
	Fe140	2.353662299	0.723498656	-1.296551653
	Fe141	5.261421133	0.467136467	-0.827362759
	Fe142	4.296617125	0.522036023	-3.211993562
	Fe143	4.149517972	2.706031772	-1.713611043
	Fe144	4.855061994	-2.014469501	-0.595593420
	Fe145	2.301596889	-2.345599539	-0.964828708
	H146	-6.353914903	-7.518806793	-0.690440412
	H147	-6.363855301	-6.522585680	-1.984450697
	H148	-9.812850882	-4.781286871	-0.081540812
	H149	-8.720749026	-6.113507335	-1.750204160
	H150	-8.745065880	-7.239637684	-0.397632373
	H151	-8.084139241	-2.741122753	0.816045634
	H152	-9.110355705	-5.615414044	2.599970915
	H153	-9.790271376	-2.935063985	1.277772113
	H154	-8.640829750	-6.267749475	4.789431931
	H155	-6.413159321	-5.850119286	2.699750652
	H156	-8.536406971	-4.554791803	5.253716514
	H157	-3.310488252	-1.723798062	2.701251782
	H158	-5.330755697	-3.698797231	2.292564335
	H159	-4.099790121	-6.151655103	2.724248475
	H160	-5.758686649	-0.089760983	6.348950549
	H161	-6.380524989	-2.294403983	4.581945504
	H162	-4.106513017	-6.244281642	4.501445498
	H163	-5.002560178	-1.451703306	2.239905855
	H164	-5.275432098	-3.648801893	6.741891211
	H165	-6.964685601	-1.373724582	6.638158020
	H166	-0.751409966	-3.402274593	3.392208554
	H167	-0.980834452	-2.278300661	2.037772073
	H168	0.607158790	-2.392329704	2.823658621
	H169	-1.963247805	-1.393363827	4.173101213
	H170	-4.718317542	-4.720262329	8.737069729
	H171	-2.628419111	1.979045160	4.969181298
	H172	-2.798072570	-0.328337227	8.879729458
	H173	-3.081813951	3.072230622	9.165968392
	H174	-0.002856901	0.053607451	2.493993572
	H175	-0.263614301	0.832012621	4.059852931
	H176	8.544562718	1.951513784	5.790423210
	H177	-3.666856111	3.324517650	5.467886819
	H178	-3.861468499	3.990971147	7.856494105
	H179	-1.897244988	3.494727281	5.566186975
	H180	-2.101601083	4.109344217	8.101821922
	H181	-3.619612121	1.572792012	7.248565628
	H182	-0.602808958	2.084188849	7.219574008
	H183	-2.253761405	-0.029047408	6.110489086
	H184	-1.639207286	0.503642138	3.011740929
	H185	1.479585336	4.235264440	0.508776781
	H186	0.434201853	4.515669878	1.927426623
	H187	2.281559191	5.297064438	2.727801079
	H188	2.444887730	4.628176101	5.313369303
	H189	4.427348279	4.161422545	2.420496807
	H190	6.291791614	2.532674626	2.328876295
	H191	5.630522568	1.548897364	6.479586360
	H192	-1.796208677	-2.829439511	5.927057667
	H193	-0.773043279	-5.743843841	6.335842168
	H194	-0.374860955	-5.191232409	7.976405014
	H195	-2.886233990	-4.628871887	6.474778803
	H196	-4.298628763	-3.149896595	9.471619527
	H197	3.728930371	3.138409755	6.552996695
	H198	0.849262481	-1.730640760	5.210885212
	H199	-2.503878096	0.610918575	11.489450970
	H200	-2.600112261	-1.108943125	11.027136599
	H201	0.085880021	-0.794418791	10.593939152
	H202	10.408847359	-7.101435493	5.591188369
	H203	-1.147147623	-5.959701520	-4.388011567
	H204	1.475335360	-6.050942743	-1.642227306
	H205	-1.007356523	-6.177529602	-8.784712316
	H206	0.507051075	-5.271538524	-9.026593506
	H207	-2.053193350	-4.318534011	-7.617328502
	H208	-1.665092236	1.126007288	-10.086942856
	H209	-1.888179208	-4.160278312	-10.144060279
	H210	-2.988634214	-2.169847135	-2.807725647
	H211	-3.756537883	-4.948046588	-1.785358450
	H212	-1.026279654	-0.960757569	-8.692604636
	H213	-3.806870296	-0.251623638	-9.374744502
	H214	0.369720370	-2.332781541	-9.634349618
	H215	-0.966005094	-5.835897169	-2.640912151
	H216	-0.540493996	-3.414831158	-7.504279333

	H217	0.332787312	-3.687441028	-10.572611444
	H218	-6.261126650	-3.423916561	-3.840946146
	H219	-3.346854131	-2.395514902	-1.075657915
	H220	1.927997720	-5.227918392	-7.437769508
	H221	-2.155319378	-4.333559804	-1.406525676
	H222	-4.250091985	2.589517311	2.489775050
	H223	-3.221466096	0.517806668	0.891675448
	H224	-8.626584992	0.776819257	2.565828636
	H225	-7.362802516	-0.069329241	-0.006609394
	H226	-7.206090781	4.075249431	1.324736292
	H227	-8.867801086	8.725272344	3.172687210
	H228	-7.624188986	5.206196335	4.263924055
	H229	-9.338860820	5.410998795	2.080578977
	H230	-6.608873133	6.824195941	1.848164645
	H231	-10.954371523	9.514239511	-0.429211537
	H232	-3.426656615	4.251896572	0.824616399
	H233	-4.845927366	3.977978120	-0.197325358
	H234	-10.212586251	10.436028002	1.835054022
	H235	-8.529485442	5.652912226	0.531440299
	H236	-6.318697187	2.014647374	0.456967188
	H237	-7.486312349	-0.561987485	2.439900868
	H238	-9.755350662	1.914706914	-1.678130471
	H239	-1.817667956	1.519544228	-2.994187684
	H240	-10.087814448	0.174353390	-1.647296573
	H241	9.799042281	3.189178659	4.171190335
	H242	8.247126821	3.309682291	3.676415490
	H243	1.728978231	5.969755710	-1.154017797
	H244	-0.765730379	5.493206245	0.198187237
	H245	1.667557689	8.051431633	0.623830986
	H246	-3.167237081	3.564337092	-2.084602390
	H247	-4.647140538	4.190611906	-3.477806608
	H248	-6.029516549	3.435837072	-5.080623059
	H249	-4.481247310	5.437754360	-2.503953770
	H250	-4.885067240	1.287226070	-4.830675101
	H251	-6.676316801	1.325274346	-4.895596255
	H252	0.201505755	11.634860176	-1.791783160
	H253	-1.010392276	12.929656954	-1.772732089
	H254	-0.427080242	10.185535470	-3.356377425
	H255	-2.086660964	9.631954714	-3.569879682
	H256	-2.731242672	9.259043876	-6.431960410
	H257	-1.775713617	4.890552828	-6.046539496
	H258	-7.166129339	8.676082073	-1.538444997
	H259	-3.360513701	8.548343744	1.664259969
	H260	-4.212115498	7.804625102	-3.568741058
	H261	1.615460570	5.402867280	-3.647219243
	H262	2.785530651	7.214541605	-2.798509044
	H263	-0.887847734	4.896232325	-3.498681429
	H264	-8.068305097	-0.203900867	-2.700600031
	H265	4.202539317	10.030495699	-3.503522036
	H266	-5.581709348	-0.920095089	-3.935981032
	H267	1.305313142	6.394683594	-5.068925562
	H268	-3.395019977	6.999880122	-0.248514176
	H269	3.650873576	10.333884859	-1.842534611
	H270	-1.923547532	7.821302291	-6.972712124
	H271	-4.445664186	8.977907293	-4.819933568
	H272	-4.625988492	-3.486215287	-4.192237140
	H273	-5.577590377	-3.318721040	-1.647291183
	H274	-7.062630188	10.322599995	0.310891536
	H275	-4.977513721	1.417136192	-2.342225752
	H276	0.059978276	8.050642654	-3.683116037
	H277	-0.846493166	6.386210600	-6.154441730
	H278	0.035326119	4.243106848	-4.842436470
	H279	-3.027822267	5.900419441	-4.130222450
	H280	0.357209068	8.911206136	3.081533210
	H281	-0.476804177	9.754984920	0.086896480
	H282	-0.148055071	12.620878337	0.426628868
	H283	1.939217563	9.422210984	2.447656185
	H284	-2.675689019	10.639106128	1.424688901
	H285	5.891713490	-2.953023664	-8.411521701
	H286	5.729343645	-4.425120122	-7.435502310
	H287	8.524190085	-5.145039900	-3.912969316
	H288	6.519593808	-5.008659401	-9.617785397
	H289	4.958422470	-5.502244953	-9.558889050
	H290	5.560140941	4.778193565	-0.096414194
	H291	5.578859401	7.405876424	1.417768533
	H292	5.293382659	8.115929724	-2.788576984
	H293	4.077404399	5.606383547	0.474371911
	H294	6.799930932	8.041663658	0.532674586
	H295	6.290048363	2.929350193	-5.848528606
	H296	6.233200556	6.764473809	-1.372699600

	H297	4.477172263	-7.078539785	5.637582399
	H298	6.582606756	-7.902050181	3.561036356
	H299	4.594059615	1.448615072	-6.184497926
	H300	4.785487921	-0.194428057	4.114142618
	H301	1.799965609	1.613735068	-8.443822310
	H302	-0.458835343	-2.113574542	-4.564756276
	H303	1.475146052	2.103047510	-6.781341672
	H304	3.468187526	-4.577861628	5.036726513
	H305	3.071009121	0.064456670	-6.198873123
	H306	3.387672593	-5.135131287	3.357980277
	H307	4.208978279	-7.360920040	3.901403737
	H308	5.899024164	-5.501083665	3.414241280
	H309	5.894186619	-5.097213348	5.137144685
	H310	-4.231115369	-0.750376908	-7.114120911
	H311	5.154943346	-9.496923836	5.650407539
	H312	4.441481416	-11.789206069	3.782482542
	H313	0.844263392	-0.002985342	-4.166936072
	H314	5.416586756	-11.836406777	6.536436902
	H315	3.923094005	-12.170060747	5.954605242
	H316	5.102780432	-3.377147998	2.840340558
	H317	-0.037600919	2.152028169	-4.941212935
	H318	-2.531055000	-2.039222335	-5.912007177
	H319	7.768884047	-9.936445702	-0.613175285
	H320	7.108793698	-7.648695777	-2.847118183
	H321	6.560657844	-8.190088365	0.791617595
	H322	7.086520963	-10.536808660	-1.876618131
	H323	6.282798690	2.609025527	-8.657692491
	H324	6.332563450	-5.078503261	0.647201648
	H325	2.128700580	-7.803243140	1.090554521
	H326	4.351465245	-1.969774874	-7.069405064
	H327	3.111668477	-9.218109749	-0.493278500
	H328	4.037613066	-6.831566038	-6.521367544
	H329	2.959208987	-5.381233230	-4.746521990
	H330	5.982399581	-12.452974099	4.342026410
	H331	5.539358502	-1.187564823	2.927932049
	H332	8.149753396	-5.814230827	1.963085926
	H333	7.140623992	-3.777299203	0.874686004
	H334	8.453692047	-6.877682690	4.033148449
	H335	2.826233865	-2.760500330	5.619784899
	H336	4.244605499	-8.278462715	-3.179262078
	H337	5.252249253	-8.588099086	-1.762425174
	H338	2.173000639	-8.668306345	-1.887056086
	H339	2.227382439	-2.135236105	-5.905444477
	H340	-2.122188256	2.238565298	-6.329751086
	H341	-4.141384429	1.027673643	-7.244352372
	H342	2.192111140	-6.036142485	0.922399410
	H343	8.316987678	-7.349488160	1.766661182
	H344	7.216628665	-9.267745879	1.677107795
	H345	3.427357837	-1.145657548	5.790626828
	H346	6.422063574	-7.534109962	-5.876949932
	H347	6.927064356	-6.041354150	-2.165473469
	H348	1.543278342	-2.900431965	-7.368740146
	H349	5.212408874	4.254493159	-6.334976909
	H350	7.102353654	0.125434036	5.034728680
	H351	7.378546392	0.553974727	3.329035367
	H352	9.826934577	-6.686561954	-5.115258714
	H353	9.011573957	-7.981686553	-4.534553153
	H354	10.440043788	-8.581306222	-0.241621601
	H355	8.895610266	-8.207128324	-1.873213155
	H356	12.127242156	-7.544577359	-2.134904036
	H357	10.239616542	-6.873103902	0.204552080
	H358	10.104014990	0.301523470	3.705375496
	H359	-3.497775152	13.182533342	3.323554317
	H360	9.252743127	-6.898939724	6.933896326
	H361	8.817782502	-4.474569840	5.694928318
	H362	-4.870807024	12.309907023	0.778397191
	H363	-4.573717070	10.270259746	3.068481388
	H364	-6.101999265	10.966571343	2.495810138
	H365	1.195040524	-3.327672929	-0.649541822
	H366	4.458167466	-1.392111221	0.707387808
	H367	-6.175980166	6.831005488	-2.473057033
	H368	0.046145292	-0.710118140	-0.558471056
	H369	2.237650413	-1.350177934	0.336065432
	H370	1.697212928	-2.025546637	0.469932019
	H371	-1.941175120	-0.256757286	-1.179032668
	Mo372	4.116120789	-4.362214429	-1.788225442
	N373	-6.941565876	-7.181048598	-1.456729015
	N374	-8.940639410	-4.629468988	0.418917818
	N375	-8.627079088	-4.937754849	3.185703863
	N376	-5.948507977	-5.753184157	3.604134471

	N377	-4.613805315	-3.361135588	2.931241330
	N378	-5.663593041	-1.735927055	5.053102852
	N379	-4.798568830	-3.082833638	7.449735364
	N380	-2.254855320	-4.605021175	7.279230802
	N381	-0.786904321	-2.709884089	5.971948737
	N382	-1.412141615	0.260719883	6.615966801
	N383	-2.082678045	0.131003006	9.449944783
	N384	4.717974436	-11.537046008	5.852781283
	N385	5.734030697	-9.289389226	4.830123218
	N386	4.452005345	-3.357296666	3.628996682
	N387	3.473771347	-2.033613522	5.299311843
	N388	5.028409842	-1.134421657	3.816406942
	N389	8.361429582	-6.987996757	5.067383593
	N390	-5.377541720	-2.916749109	-3.760382191
	N391	-1.479999529	-5.586352110	-3.492336953
	N392	-5.696797033	-0.416974698	-3.039758205
	N393	-8.135332809	0.657567056	-2.163588325
	N394	-8.049139877	0.356700970	0.624582367
	N395	-5.969611808	2.073524174	1.417692856
	N396	-2.933575653	2.693082408	-1.538666726
	N397	-2.373972732	0.662570387	-1.086697512
	N398	-6.682553854	4.714373435	1.917608368
	N399	-9.930534167	7.718059766	0.180894590
	N400	-9.998706375	9.499731579	1.501941293
	N401	9.019539093	2.647742707	3.789978291
	N402	6.253041588	7.187904730	0.676432971
	N403	4.579033465	8.479706720	-2.162869305
	N404	2.024246462	7.865561004	-3.022837288
	N405	-2.492599671	6.434766984	-4.838658919
	N406	-2.478546239	8.290338114	-6.266339417
	N407	-3.844068957	8.273919145	-4.399803152
	N408	0.791722552	6.335324290	-0.916296847
	N409	0.812486958	8.217082423	1.162382874
	N410	-0.465372576	10.605836025	0.648837677
	N411	-1.435329024	10.332133612	-3.219458116
	N412	-2.977122328	11.607558071	1.355620271
	N413	5.555035346	-4.669923057	-9.589870693
	N414	3.582166391	-2.524997838	-7.455821088
	N415	2.481240264	0.265421679	-7.023961149
	N416	4.534455505	2.292464677	-6.760094753
	N417	-0.234111179	-2.907211083	-10.234085904
	N418	-2.020443872	-1.112911182	-8.887539308
	N419	8.904598570	-6.996286950	-4.795540672
	N420	4.832804211	-5.611605026	-3.695226741
	N421	4.514635464	-6.528828463	-5.675852164
	N422	9.563934492	-7.444794422	-1.734823285
	N423	0.137009587	2.267201112	-0.133374013
	N424	2.329117005	1.237530492	3.428770995
	N425	1.015231304	1.714637959	-0.587026250
	N426	2.557705419	0.332827431	2.828838786
	O427	-6.725691689	-5.136966211	0.568544511
	O428	-7.788328202	-2.822155646	3.330913157
	O429	-6.155138058	-5.154229082	5.797386584
	O430	-3.067205371	-3.916587918	4.520847757
	O431	-3.794350014	-0.463551507	4.819846493
	O432	-4.566929344	-0.994436703	8.361586986
	O433	-1.947513642	-3.956594051	9.455618760
	O434	1.202329371	-3.779406096	6.420211139
	O435	0.856382542	0.077082571	6.809937109
	O436	-0.540055185	1.781012329	9.674428047
	O437	-0.300808319	-0.295155943	12.535568204
	O438	6.633817428	-10.350116756	2.982249068
	O439	7.045163268	-7.689643175	6.817506250
	O440	10.799040237	-4.594322832	6.173759077
	O441	-5.587651813	-0.841824262	-0.787435421
	O442	-3.070438563	-4.355542626	-4.555589275
	O443	-7.019973708	2.512299982	-1.435095552
	O444	-5.780989284	3.081890557	-4.208096672
	O445	-10.031450738	1.496980613	0.823128867
	O446	-6.269589914	1.504975810	3.610864678
	O447	-4.780819256	5.017428704	3.145672273
	O448	-7.302230993	7.220800138	4.337819709
	O449	9.991289234	-0.165888746	5.689542937
	O450	2.922216687	4.653822514	4.463574620
	O451	3.649042649	8.376468736	-0.082086681
	O452	1.482384277	10.085278133	-3.280654566
	O453	-1.178042738	7.212151542	-1.679578627
	O454	-1.042093185	7.198355974	2.052079695
	O455	2.160027318	5.687891157	1.838675311
	O456	0.504494046	11.603616288	2.470744468

O457	-2.572567263	13.583245926	0.296444683
O458	-3.045295778	10.998189533	-1.738697993
O459	-5.440244284	13.686823430	2.942932035
O460	-5.276484911	6.854575879	-2.092488631
O461	3.023584880	-4.516067818	-8.455907310
O462	0.968457180	-0.679828879	-8.465228066
O463	3.295940846	3.670370773	-8.081947531
O464	7.186882010	4.352554044	-8.098578170
O465	-3.664376299	-2.566212716	-9.505972488
O466	-0.566984094	-6.553893856	-6.217819220
O467	1.442613118	-5.715538873	-6.701244383
O468	-2.737145405	2.386010815	-8.898256064
O469	10.379060037	-5.397739190	-2.270714446
O470	12.817779989	-6.959259913	-0.303730878
O471	0.461726733	-6.280583158	-1.317706871
O472	-0.347103071	-7.388003024	0.463341281
O473	5.548326889	-10.893728964	-2.651449802
O474	3.310776153	-10.930062198	-2.976356441
O475	5.220179264	-5.873139129	-0.636405665
O476	4.927472502	-7.572732926	0.799705026
O477	2.853469568	-6.084146910	-1.718309520
O478	8.687040102	-6.579691237	2.282233141
O479	6.846912485	-4.586417435	1.336910378
O480	-3.973262763	4.772856485	-3.033209818
O481	7.981768136	-10.430130871	-1.436375723
O482	7.433394588	-8.643757349	0.938387429
S483	3.879671627	4.969519940	-1.863457578
S484	2.569711415	1.964825073	-3.162851458
S485	2.463330972	-3.263003445	-3.060770648
S486	3.768791076	1.772806806	0.245214174
S487	0.744763621	-0.900354684	-1.715138206
S488	4.799360561	-0.814467202	-4.906794659
S489	3.617304582	-3.588010505	0.341761064
S490	6.082544822	1.729677699	-2.461318788
S491	6.071904030	-3.085012771	-2.199735700
S492	6.535310431	-0.820143644	0.409579007
end			

TS

Fe(139)	-2.209
Fe(140)	-1.071
Fe(141)	-2.652
Fe(142)	2.649
Fe(143)	2.794
Fe(144)	1.492
Fe(145)	-0.065

bm52etc6hx7hnn2tum6h2ndti_1_53370.24

C1	-8.398999766	-0.241262283	4.430846688
C2	-8.959623729	-1.462826381	5.170160176
C3	-8.935953311	1.596193108	2.888819272
C4	-8.336999351	2.786145789	3.630565479
C5	-8.134111725	4.030393356	5.725174647
C6	-6.633413535	3.905949967	5.999704522
C7	-4.673522627	2.462258262	6.136411847
C8	-3.966578632	2.898204030	4.846565712
C9	-4.135476224	2.909138139	2.386362264
C10	-4.092825850	4.432814207	2.204725745
C11	-5.116103419	6.585845211	2.585832579
C12	-4.164956840	7.320271766	3.542106083
C13	-3.466057436	7.619296509	5.879369652
C14	-2.054390777	7.172770363	6.290176754
C15	-0.559550614	5.276876983	6.773898877
C16	0.316738326	4.955055684	5.558401648
C17	0.296739570	4.619513044	3.106758014
C18	0.582057710	5.882333347	2.269661731
C19	-0.529506348	3.510937648	2.408385652
C20	-0.225113009	3.297357278	0.912757213
C21	-0.423645711	2.201997808	3.199241243
C22	-0.285932516	7.682580753	0.837794676
C23	0.024592634	8.968879761	1.628257797
C24	-1.490506648	7.836211327	-0.122704930
C25	-1.407920844	9.136976764	-0.929716534
C26	-1.578339436	6.630178079	-1.066787799
C27	-0.599597420	10.505162113	3.401166064
C28	0.814817275	10.660174690	3.920300431
C29	4.247612755	-0.089869389	12.844144930
C30	5.031270315	-0.260100606	11.547898606
C31	5.604811975	0.924847956	9.432255721
C32	6.851296255	1.829355492	9.474826365
C33	4.548444046	1.466970605	8.458448107
C34	5.053768804	1.552897556	7.023360107
C35	3.922458160	1.814623927	6.031481860
C36	4.514241861	2.644127815	3.739631600
C37	9.222136499	2.017547018	8.889150692
C38	9.658337774	2.449244682	7.506782878
C39	-5.613712185	-2.172420676	1.347051155
C40	-5.797747816	-1.307829486	0.084238042

	C41	-4.109537849	-2.207620427	1.698819658
	C42	-3.763872695	-2.768794622	3.093137427
	C43	-3.542364958	-4.280036090	3.155890917
	C44	-5.978936325	-1.473009786	-2.371509908
	C45	-7.019748537	-0.377521813	-2.638447280
	C46	-6.054945695	-2.613915714	-3.393961873
	C47	-9.242760395	0.504175484	-2.105703163
	C48	-8.874942586	1.911349392	-1.624850567
	C49	-7.272682265	3.319367598	-0.414605695
	C50	-5.987813449	3.783410170	-1.103825509
	C51	-4.215092991	3.351040177	-2.732275451
	C52	-4.495872380	4.424553771	-3.807144201
	C53	-3.561860191	2.182215946	-3.485985948
	C54	-3.074146006	1.037174971	-2.665203587
	C55	-2.846036593	0.855129441	-1.325205323
	C56	-2.319640463	-1.044584958	-2.364439966
	C57	-6.056985921	5.068235387	-5.600102984
	C58	-5.978619936	6.559251990	-5.276894235
	C59	-7.500844339	4.671182174	-5.989809975
	C60	-7.944531268	5.002084815	-7.384483434
	C61	-7.733819213	6.123222726	-8.165867215
	C62	-8.979190668	4.645270628	-9.251988273
	C63	9.296165362	3.530721970	0.717086200
	C64	10.158570142	3.009005977	1.852190532
	C65	7.832284013	3.196465479	1.080843680
	C66	6.831676962	3.724439691	0.080732633
	C67	6.273601836	5.009090345	0.207732958
	C68	6.433398032	2.940114776	-1.016870740
	C69	5.344199253	5.495964626	-0.718652749
	C70	5.522718609	3.418324105	-1.956547629
	C71	4.973674514	4.694377128	-1.804566605
	C72	6.239289763	0.631261513	-6.197769123
	C73	5.346913999	0.791134667	-7.441095896
	C74	5.383814111	0.526825429	-4.935919800
	C75	4.583027600	-0.235013473	-9.553476943
	C76	3.072963185	-0.460125610	-9.431403987
	C77	1.217281623	-0.960187046	-7.847601292
	C78	0.678969392	0.313528967	-7.146974666
	C79	1.099857535	-2.211023242	-6.943887012
	C80	-0.258163835	-2.551332001	-6.316939353
	C81	-1.304384231	-3.198123961	-7.240665013
	C82	-2.569900614	-2.114155974	-9.101885566
	C83	1.027122383	2.110892980	-5.525342293
	C84	1.027289491	3.357890513	-6.421812878
	C85	1.865060541	2.432671908	-4.271221254
	C86	2.374364915	4.654753117	-7.998712835
	C87	1.912745200	4.780545850	-9.440248630
	C88	0.702625558	3.738988530	-11.280427562
	C89	-0.495077932	4.595580907	-11.757503715
	C90	0.556986687	2.296831923	-11.774825259
	C91	-0.677986760	1.583945957	-11.243030366
	C92	-2.509409030	5.840812199	-11.125389021
	C93	-2.294175576	7.173131074	-11.832341934
	C94	-3.337045334	6.057738072	-9.827427640
	C95	-3.706548689	4.752319574	-9.161216088
	C96	-4.873179995	4.067067109	-9.535810249
	C97	-2.867164260	4.152909113	-8.207484324
	C98	-5.186230947	2.817321394	-8.995067115
	C99	-3.147998384	2.889537638	-7.676170522
	C100	-4.306883930	2.235038810	-8.082638521
	C101	3.661546716	-9.901548297	1.527821951
	C102	2.273972772	-9.351936910	1.247717502
	C103	1.017227750	-7.594828860	0.021188369
	C104	0.711915895	-7.642635836	-1.481086767
	C105	1.441853546	-6.868030605	-3.695074238
	C106	2.723931445	-6.792583561	-4.520351596
	C107	5.070430893	-6.175442435	-4.722291119
	C108	5.728900204	-7.460800126	-5.182306335
	C109	-3.065779145	-9.714333922	0.134776659
	C110	-3.946837440	-9.035199082	-0.920319600
	C111	-2.695437829	-8.695837621	1.238766640
	C112	-2.125791248	-9.404112329	2.483404091
	C113	-1.537112210	-8.399346040	3.448376054
	C114	-4.050508880	-7.690534131	-2.958643569
	C115	-3.399321987	-7.927345904	-4.306780665
	C116	-4.426008721	-6.214674072	-2.673821600
	C117	-3.214837985	-5.427246100	-2.262110555
	C118	-2.338907514	-4.896469315	-3.222377335
	C119	-2.848479449	-5.374282333	-0.905597085
	C120	-1.089900296	-4.399403407	-2.835127388

	C121	-1.600142060	-4.880502520	-0.520019412
	C122	-0.704191693	-4.424877443	-1.492841568
	C123	7.086210505	-6.634748980	5.442400305
	C124	8.236173409	-5.768588512	5.981728419
	C125	5.725630144	-6.048473202	5.845800998
	C126	4.520125029	-6.682657730	5.229979621
	C127	4.096500305	-7.989737388	5.350895245
	C128	2.526933559	-6.809117253	4.330465538
	C129	9.242295513	-4.559508129	7.831494981
	C130	10.622690715	-5.127519311	7.582665489
	C131	-0.335498449	-2.321119471	6.559495737
	C132	1.132007297	-2.072061982	6.900129242
	C133	2.045146504	-3.326223063	6.745322458
	C134	1.766341097	-4.321957284	7.905309343
	C135	2.767677767	-5.470876245	8.036171323
	C136	2.608901473	-6.306612604	9.325809320
	C137	3.518638770	-2.897059545	6.831443470
	C138	3.497017560	-2.442605681	0.437544357
	Fe139	3.603369927	-4.197888904	1.280410194
	Fe140	2.254088235	-1.384292330	-0.928561673
	Fe141	5.132620041	-1.499137821	-0.252919049
	Fe142	3.889448930	-3.569693734	-1.135616310
	Fe143	4.217945984	-1.507791918	-2.770622770
	Fe144	4.437386313	-1.941557457	2.160969782
	Fe145	1.893399255	-2.188971955	2.059807278
	H146	-7.350641487	-1.857870217	6.362337779
	H147	-7.404420313	-2.779467554	5.015097410
	H148	-10.271136310	0.116805040	3.648914140
	H149	-9.616621049	-2.017074770	4.480557613
	H150	-9.616737805	-1.078237663	5.969346962
	H151	-8.183415337	1.279441409	2.151867391
	H152	-9.341163036	2.287527942	5.341797084
	H153	-9.820150539	1.943235769	2.337389218
	H154	-8.664620839	4.063886144	6.685681381
	H155	-6.707341735	1.851140696	5.827427414
	H156	-8.283868981	4.994535984	5.219647658
	H157	-3.123910403	2.535796521	2.183446780
	H158	-5.408572674	1.902970173	3.778909011
	H159	-4.467493429	1.400455270	6.325549940
	H160	-4.815356419	6.854647578	1.565168104
	H161	-5.956084761	4.655010983	3.059792925
	H162	-4.257635633	3.046963559	6.964366055
	H163	-4.812172051	2.460543674	1.645841950
	H164	-4.751200277	6.101416541	5.094298707
	H165	-6.134419755	6.957866897	2.761921422
	H166	-0.793954691	2.315244117	4.226208701
	H167	-1.022843496	1.411547069	2.716044087
	H168	0.617371941	1.849851961	3.240053644
	H169	-1.567886895	3.855259495	2.464031306
	H170	-4.105981263	7.586029834	6.774961888
	H171	-1.680224501	5.682486459	-0.522937024
	H172	-1.633217450	8.715340602	2.861990158
	H173	-1.411961879	10.028635774	-0.285913827
	H174	0.322830959	2.361742219	0.743665649
	H175	0.359574667	4.108398914	0.459464486
	H176	9.398389320	4.633402915	0.709459576
	H177	-2.453027879	6.724881402	-1.728023613
	H178	-2.269284075	9.204269262	-1.609251454
	H179	-0.679070363	6.576401274	-1.703539208
	H180	-0.490490924	9.162007173	-1.539187643
	H181	-2.405487557	7.869583010	0.494113951
	H182	0.622485782	7.501109831	0.243750263
	H183	-1.413880107	6.108236417	1.774495633
	H184	-1.166587033	3.239287324	0.347614313
	H185	2.051571946	1.496828079	-3.725412796
	H186	1.253540983	3.091575893	-3.634574317
	H187	3.282788586	3.767461041	-3.957592789
	H188	3.701678860	5.978612796	-2.514133658
	H189	5.202213650	2.789876938	-2.789033967
	H190	6.816194168	1.921638942	-1.119110359
	H191	6.563112310	5.642824474	1.051237394
	H192	-1.355312588	5.004613402	4.393202802
	H193	-0.691066312	4.348554841	7.346296159
	H194	-0.007900795	5.989451462	7.400374531
	H195	-2.600319042	5.172752678	6.187370061
	H196	-3.371538481	8.662410769	5.557096796
	H197	4.905065100	6.489212982	-0.590155067
	H198	1.296182964	4.235122010	3.343570694
	H199	-0.858116773	11.391040436	2.796910998
	H200	-1.266677575	10.485942436	4.277683377

	H201	1.357176374	9.703423543	4.101872162
	H202	10.019751031	1.424609617	9.360875550
	H203	-2.487416993	-5.713360920	4.163355345
	H204	0.443959136	-3.596206775	5.335082515
	H205	-2.937804324	-9.938149034	2.996973761
	H206	-1.363869524	-10.136311596	2.184187959
	H207	-3.579476300	-8.110232140	1.534173147
	H208	-2.814154717	-8.874148010	-4.370435009
	H209	-3.729518578	-10.466807130	0.586994029
	H210	-3.585875978	-2.793012720	0.923809276
	H211	-4.573229243	-2.531539043	3.804639570
	H212	-2.276249089	-8.289208662	-1.908753131
	H213	-5.007275983	-8.249548174	-3.023523634
	H214	-1.206629447	-9.805049536	-0.788329670
	H215	-2.068603897	-4.065589805	4.619747928
	H216	-1.965314435	-7.979862162	0.829659281
	H217	-1.540921724	-11.087878245	0.189599465
	H218	-7.097879077	-3.600603637	1.506423433
	H219	-3.742761753	-1.170476358	1.653744232
	H220	0.241933386	-8.873189351	2.763000742
	H221	-2.867646191	-2.259308561	3.475253603
	H222	-3.512246224	3.805616109	-2.021058572
	H223	-2.991075323	1.520160966	-0.484253047
	H224	-8.044291501	4.068476531	-0.637841292
	H225	-7.245343166	1.189945577	-0.557581324
	H226	-6.377323468	3.635337029	-4.046210708
	H227	-7.178210172	7.039070612	-7.998892852
	H228	-6.262399619	6.811544131	-4.226166123
	H229	-8.199068263	5.094604398	-5.244837325
	H230	-5.362610799	4.851995849	-6.426410864
	H231	-9.565880897	4.207835753	-10.054910445
	H232	-2.713200987	2.599143414	-4.051486837
	H233	-4.278403420	1.794710725	-4.228189264
	H234	-8.442107517	6.500608283	-10.149283098
	H235	-7.576405198	3.576463928	-5.893891880
	H236	-5.890550483	2.070780512	-2.288694736
	H237	-7.102073953	3.354449039	0.671997285
	H238	-9.552672662	0.613332097	-3.154771936
	H239	-1.981511513	-2.064060112	-2.539357854
	H240	-10.115030226	0.162692861	-1.531392857
	H241	10.578680536	3.276322077	-0.858927566
	H242	9.005510649	3.089181963	-1.259362732
	H243	2.331236113	0.422640904	-5.874567976
	H244	-0.015919298	1.957252425	-5.211617910
	H245	2.773949881	2.705794892	-7.283582428
	H246	-2.926926299	-0.376303710	-4.294188566
	H247	-4.475496200	-1.262278908	-5.446671622
	H248	-6.151126346	-2.751901697	-5.349110494
	H249	-4.018947002	0.001093812	-6.296281385
	H250	-5.282626549	-3.356333471	-3.137413718
	H251	-7.043166334	-3.098173587	-3.350641089
	H252	1.477159883	1.736074065	-11.560161883
	H253	0.446556393	2.348737837	-12.870856538
	H254	0.478229138	-0.046330957	-10.695379438
	H255	-1.253952973	-0.136376692	-10.368453550
	H256	-2.306521689	-2.821600270	-11.013292007
	H257	-1.897662635	-3.937394161	-6.679078255
	H258	-6.110542439	2.306732144	-9.279064725
	H259	-1.971189335	4.668252555	-7.850785146
	H260	-3.612120589	-0.352298992	-8.842276567
	H261	1.816676011	-2.083020191	-6.116092556
	H262	3.315380256	-0.943421149	-7.462673994
	H263	-0.695127519	-1.669264263	-5.824552097
	H264	-8.311135344	-1.268953885	-1.331748430
	H265	4.987544003	-1.001830834	-10.227017260
	H266	-6.121694627	-3.047570402	-0.833277170
	H267	1.459258776	-3.073220413	-7.529896112
	H268	-2.448423231	2.417266101	-6.985102353
	H269	4.710177538	0.741010986	-10.042508881
	H270	-1.744737359	-3.887008190	-9.763604377
	H271	-3.812008237	-1.114858087	-10.386404143
	H272	-5.545307514	-4.217442311	1.586070702
	H273	-6.129040201	-1.622587437	2.156943279
	H274	-5.555902565	4.517177916	-10.260989229
	H275	-5.017459952	-0.958862389	-2.501362556
	H276	0.632674388	-1.075563428	-8.765859236
	H277	-0.796129282	-3.746814741	-8.042702638
	H278	-0.049455367	-3.274546923	-5.516211629
	H279	-2.743224965	-1.633205756	-7.134444479
	H280	1.943074127	5.493621485	-7.432158760

	H281	0.826417867	3.058950136	-9.238967641
	H282	1.556670003	4.180496692	-11.816259340
	H283	3.466438791	4.775743279	-8.003162337
	H284	-1.058419143	4.936109798	-9.837527315
	H285	4.307336851	-9.756776949	0.650001956
	H286	4.076197348	-9.262215250	2.336474756
	H287	7.186835914	-6.588880021	4.348251867
	H288	4.495711217	-11.610863623	2.252721076
	H289	2.905711146	-11.451399759	2.610072525
	H290	6.064183706	0.414936043	-4.080453286
	H291	6.623069517	2.635440379	-6.098842902
	H292	5.894411860	-1.081841843	-8.094887519
	H293	4.789994239	1.442207808	-4.809271896
	H294	7.810052428	1.789305075	-6.843692017
	H295	5.798729727	-5.631755685	-4.101031246
	H296	6.843174065	-0.284301192	-6.294817130
	H297	4.208996228	2.454425118	8.810872277
	H298	5.888538459	-0.091666718	9.130977574
	H299	3.902942942	-6.116944330	-2.938602217
	H300	5.242421076	3.035274074	1.865192597
	H301	0.892868453	-7.757633202	-4.025771321
	H302	-1.317864925	-4.867825548	0.536263140
	H303	0.835264861	-5.994905266	-3.991687348
	H304	3.490481521	2.813242132	6.179145399
	H305	2.228502453	-6.311749603	-1.749903292
	H306	3.107349040	1.086764591	6.167555904
	H307	3.685749991	0.782792331	8.505290745
	H308	5.539112545	0.605298057	6.742881555
	H309	5.811505065	2.345749027	6.916518068
	H310	-5.182904799	-6.211884114	-1.877022552
	H311	4.535998967	1.638821836	11.162772515
	H312	3.323089830	-0.672796544	12.721059864
	H313	0.291804220	-4.081808534	-1.221975216
	H314	4.645347190	1.757966641	13.664243824
	H315	3.059094122	1.350467467	13.709790308
	H316	4.986606439	0.841639009	4.500489432
	H317	-0.393974234	-4.003590720	-3.575888952
	H318	-3.538199422	-5.741534056	-0.143741371
	H319	6.251379431	-4.809752432	9.859884580
	H320	5.616017149	-6.118577978	6.940935332
	H321	5.467898064	-2.766184306	8.551492123
	H322	5.336356275	-6.062722666	9.970745525
	H323	5.403878502	-8.380827950	-4.639393227
	H324	5.641804243	-1.954180084	5.544195690
	H325	1.205475048	-1.658930420	7.912717260
	H326	3.101386226	-7.942161748	0.017985970
	H327	1.773986647	-3.733477715	8.835935308
	H328	2.231122758	-8.853779054	4.753258634
	H329	1.578748335	-6.571045560	3.866402040
	H330	4.835839881	-0.585470405	13.635405529
	H331	5.676898177	1.493144857	2.482735471
	H332	7.505339666	-1.233940190	6.800821661
	H333	6.643461492	-1.500413566	4.451877502
	H334	7.929610330	0.347568012	8.475641737
	H335	3.159215559	3.993993261	4.588546227
	H336	2.651720273	-6.158150245	7.182633114
	H337	3.796949240	-5.090469889	8.004887036
	H338	0.748748218	-4.720889501	7.783917805
	H339	1.138149631	-6.552237290	0.354454198
	H340	-2.622850345	-4.915839235	-4.276993500
	H341	-4.886465926	-5.818186585	-3.591696636
	H342	1.471144382	-1.292678862	6.198246539
	H343	7.441318175	-1.899875781	8.206417377
	H344	6.097506378	-2.367449623	9.901797312
	H345	3.952837043	4.524727818	3.143047378
	H346	4.565317196	-8.849667499	5.812724485
	H347	5.717182666	-4.976892205	5.612034872
	H348	0.177863670	-8.049718735	0.550440488
	H349	4.848542679	-5.551594125	-5.602743654
	H350	7.638021584	3.625696362	2.073958012
	H351	7.739863471	2.101779896	1.159608450
	H352	8.118428799	-8.405629116	5.522009428
	H353	7.220611495	-8.128196096	6.861963688
	H354	9.101940725	-4.507923910	8.922533263
	H355	7.422072488	-5.664520787	7.913928497
	H356	10.659509225	-6.238587558	7.470775573
	H357	9.182023496	-3.532943086	7.438810479
	H358	10.441047002	1.932310111	1.756157456
	H359	-1.285756266	7.628351159	-11.693629158
	H360	9.083732405	2.938158918	9.484164512

H361	8.828520576	2.573088351	6.768768875
H362	-3.081488483	5.195499171	-11.812062009
H363	-2.760919371	6.690519185	-9.135342672
H364	-4.241875386	6.616280492	-10.109057527
H365	0.714259106	-1.942924424	2.985503275
H366	4.220553729	-0.470940128	1.952765302
H367	-5.498876879	0.729728735	-7.750923589
H368	-0.058931241	-0.766544149	0.683271783
H369	2.135478430	0.244737977	1.852010942
H370	1.430535419	0.143899000	2.075284785
H371	-2.089599044	-0.879540206	-0.301481220
Mo372	3.272588098	-3.701918800	3.929841488
N373	-7.988798042	-2.372044588	5.749371720
N374	-9.326953721	0.484799345	3.739185754
N375	-8.689453099	2.949174116	4.925883664
N376	-6.117466032	2.652713044	6.059380962
N377	-4.578282209	2.487878932	3.705618959
N378	-5.115152614	5.140529613	2.735221946
N379	-4.123418131	6.863219342	4.821327506
N380	-1.865833172	5.831100491	6.456887912
N381	-0.340017235	4.938712595	4.376833564
N382	-0.482246725	6.527291733	1.721759129
N383	-0.786550097	9.260192159	2.675887851
N384	3.897975544	1.308242011	13.129336715
N385	5.025019359	0.832773965	10.760242348
N386	4.434143433	1.685919634	4.667359690
N387	3.832131166	3.797627249	3.841738437
N388	5.331541476	2.439763997	2.684801708
N389	7.959781445	1.304005835	8.894728945
N390	-6.154157993	-3.518008022	1.123752154
N391	-2.647660254	-4.705289843	4.065576514
N392	-6.052476990	-2.035602023	-1.035277360
N393	-8.197081289	-0.496762473	-1.984209987
N394	-7.785028567	2.022546575	-0.814335313
N395	-5.413214209	2.934385531	-2.012544805
N396	-2.738057994	-0.161535173	-3.279670893
N397	-2.386653192	-0.437981896	-1.172151125
N398	-5.702408535	4.299351421	-4.417278345
N399	-8.721456894	4.087960477	-8.076209034
N400	-8.399132671	5.876036918	-9.348044190
N401	9.694668910	2.879245308	-0.532592641
N402	7.164269049	1.766497731	-6.049200994
N403	5.335039502	-0.260515907	-8.311303051
N404	2.624282879	-0.826774350	-8.212752563
N405	-2.236433216	-2.224007555	-7.816635793
N406	-2.154039212	-2.998149733	-10.026054410
N407	-3.285892230	-1.045439383	-9.519663281
N408	1.484843228	0.903987622	-6.218683605
N409	2.029369663	3.405920122	-7.343610572
N410	1.016036056	3.839801306	-9.867623891
N411	-0.477659455	0.290617490	-10.871170290
N412	-1.236601159	5.207613090	-10.800833073
N413	3.594510877	-11.325989400	1.862245277
N414	2.220937257	-8.346915138	0.350152759
N415	1.568296099	-6.931136201	-2.249737388
N416	3.877483730	-6.400594559	-3.920494861
N417	-1.952943953	-10.442301677	-0.487377077
N418	-3.298741901	-8.353922799	-1.911701319
N419	7.213396725	-8.048981657	5.840154466
N420	3.526652827	-5.959130023	4.571469557
N421	2.844071286	-8.043744074	4.779355593
N422	8.198797765	-5.416285119	7.295537390
N423	0.406405293	0.536647030	-2.169059377
N424	2.808648919	2.941421753	0.209893360
N425	1.145909442	-0.202427871	-1.735211303
N426	3.076103474	2.146478820	0.930153985
O427	-7.205927107	0.102857739	4.484553262
O428	-7.561884483	3.559766442	3.040684767
O429	-5.942469874	4.924362551	6.168993717
O430	-2.927745979	3.582647669	4.852594879
O431	-3.148616943	4.969258979	1.606989305
O432	-3.523950096	8.309069488	3.146735068
O433	-1.171767473	8.011173510	6.510182983
O434	1.530477984	4.708575366	5.699988342
O435	1.752699419	6.269340944	2.120293142
O436	0.968133656	9.711898544	1.308743756
O437	1.313175102	11.743282049	4.190069747
O438	5.584873883	-1.348063795	11.287946254
O439	6.804869332	2.955176996	10.000909725
O440	10.819504599	2.671709815	7.202168885

O441	-5.700116213	-0.064682892	0.086942533
O442	-4.179145382	-5.075164256	2.426486690
O443	-6.758371108	0.564685428	-3.407652531
O444	-5.830279643	-2.085561618	-4.716249606
O445	-9.578215707	2.867702684	-1.974187351
O446	-5.501586124	4.882766769	-0.822516657
O447	-3.642909264	5.248596133	-4.147746692
O448	-5.684182818	7.423267531	-6.084869359
O449	10.509889641	3.677425974	2.812942319
O450	4.050750847	5.098357086	-2.748940656
O451	4.678874865	1.819341434	-7.649875587
O452	2.349895466	-0.310241411	-10.435757264
O453	-0.444160541	0.756760901	-7.438694740
O454	0.202975394	4.266142329	-6.254904136
O455	3.101385877	3.063263521	-4.613403730
O456	2.329858204	5.695399301	-10.161235044
O457	-0.711040596	4.742293119	-12.969294840
O458	-1.789943131	2.130789406	-11.208111940
O459	-3.166870360	7.725916677	-12.482766439
O460	-4.562447561	0.943713392	-7.574392693
O461	1.270979569	-9.802770583	1.853234917
O462	-0.240532789	-8.307526821	-1.937824764
O463	2.664700735	-7.038079662	-5.736297979
O464	6.582949919	-7.500078273	-6.053327851
O465	-5.186237348	-9.084338607	-0.861852955
O466	-2.214982584	-7.710400240	4.203517883
O467	-0.197673606	-8.242370319	3.413515147
O468	-3.525503652	-7.194700138	-5.278150323
O469	9.194035204	-5.437338065	5.256584967
O470	11.643190112	-4.453804581	7.578997727
O471	-0.540304625	-3.202522072	5.572207339
O472	-1.262605153	-1.722182865	7.101450586
O473	3.685029359	-6.639751582	9.934964246
O474	1.438284966	-6.627202121	9.677381594
O475	4.281204474	-3.215905011	5.816057518
O476	3.945110755	-2.304928531	7.841160567
O477	1.792189801	-3.906951301	5.464786804
O478	7.975339424	-1.251588814	7.670400784
O479	6.306456431	-1.249616003	5.334148159
O480	-3.676628721	-0.783888796	-5.800240369
O481	6.286528466	-5.765448422	10.087090653
O482	6.287661159	-2.913365649	9.096804971
S483	4.226969139	-0.921152645	-4.983633047
S484	2.374304970	-2.843493319	-2.640764324
S485	1.635152117	-4.371613951	2.341957420
S486	3.989082353	0.135585619	-1.316617248
S487	0.443610152	-1.996302913	0.372130557
S488	3.968073562	-5.637682590	-0.357314436
S489	3.037984345	-1.398433170	3.783178348
S490	5.902396423	-2.795729763	-1.889812935
S491	5.289689853	-4.002946606	2.725321863
S492	6.368163544	-0.929759418	1.469567321
end			

product

Fe(139) -2.127
Fe(140) -1.051
Fe(141) -2.507
Fe(142) 1.778
Fe(143) 2.625
Fe(144) 1.250
Fe(145) 1.230

bm52etc6hx7hnn2tum6h2ndtc.car_4

C1	-7.728382723	-5.481050119	-0.793202433
C2	-7.940864389	-6.494711075	-1.922978940
C3	-8.777054137	-3.720169722	0.560074878
C4	-8.358633999	-4.146492526	1.962558294
C5	-8.201629919	-5.941798179	3.615069108
C6	-6.686351469	-6.033034034	3.822263781
C7	-4.464474060	-6.148719571	2.825967286
C8	-3.992396769	-4.717303578	3.115791078
C9	-4.399478676	-2.341955967	2.601385305
C10	-4.689990853	-1.893649497	4.042107228
C11	-6.088116945	-2.034978424	6.004675228
C12	-5.197616258	-2.704551110	7.062342186
C13	-4.344255144	-4.835085402	7.942238115
C14	-2.832023135	-5.102054007	7.846964595
C15	-0.943340442	-5.704939099	6.384323632
C16	-0.140026053	-4.470279454	5.963753692
C17	-0.329638767	-2.144917526	5.130812239
C18	-0.382648643	-1.068416463	6.235518803
C19	-1.001565811	-1.782428266	3.788134992
C20	-0.757837294	-0.339429544	3.310975705
C21	-0.613455880	-2.821142122	2.728763937
C22	-1.720860721	0.539523223	7.522168861
C23	-1.598795662	0.043217715	8.975840927
C24	-3.015390141	1.352397475	7.267784131

	C25	-3.260616918	2.383708121	8.375584693
	C26	-2.951733529	2.048317299	5.901091533
	C27	-2.331531705	-1.494812554	10.703453448
	C28	-0.928502137	-1.776088572	11.203376869
	C29	5.385790980	-11.984570568	3.244853507
	C30	6.071881591	-10.652215418	2.966337545
	C31	6.187287781	-8.301885591	3.789174446
	C32	7.229257951	-8.009534879	4.886016656
	C33	4.946731004	-7.404933417	3.930147652
	C34	5.272199666	-5.922426950	3.795570761
	C35	4.013811321	-5.069705986	3.640768782
	C36	4.228885320	-2.613236854	4.086520122
	C37	9.446055325	-7.080973721	5.356834094
	C38	9.645339344	-5.597254336	5.572268885
	C39	-4.928500761	-2.447685946	-2.808280024
	C40	-5.399561707	-1.085789197	-2.265813437
	C41	-3.418876178	-2.593233781	-2.505097311
	C42	-2.828685958	-4.005011490	-2.699295754
	C43	-2.307312690	-4.313214774	-4.103297793
	C44	-5.762230451	1.257284203	-2.953944595
	C45	-7.016814773	1.584911444	-2.132933356
	C46	-5.709126967	2.038993755	-4.274637106
	C47	-9.311259080	0.935355160	-1.563054112
	C48	-9.176505832	0.785183597	-0.043915994
	C49	-7.788590515	0.053408480	1.839408134
	C50	-6.686317451	0.971442508	2.370213399
	C51	-5.030887554	2.716612384	1.933068793
	C52	-5.615870951	3.926235265	2.699312419
	C53	-4.233263069	3.320695441	0.767240146
	C54	-3.450017743	2.376664512	-0.082776504
	C55	-3.039902078	1.076293297	0.062520699
	C56	-2.261147866	1.812862252	-1.890662342
	C57	-7.437489725	5.584580746	2.692918117
	C58	-7.604994471	5.559506555	4.211124840
	C59	-8.811446380	5.701070115	1.990796714
	C60	-9.436179407	7.064861424	1.944447704
	C61	-9.541874531	8.052264808	2.905272106
	C62	-10.543507458	8.685735359	1.032321389
	C63	8.457688175	1.187160787	5.145514887
	C64	9.518701567	0.107154048	5.022510507
	C65	7.132549090	0.573030982	4.646851095
	C66	5.955743330	1.510446783	4.782628724
	C67	5.171837016	1.543373390	5.950482009
	C68	5.616440987	2.391785542	3.739077228
	C69	4.083281851	2.415740830	6.073792166
	C70	4.547668351	3.279206618	3.855310958
	C71	3.775554220	3.285696004	5.020180083
	C72	5.381445579	6.966709999	0.435595408
	C73	4.356623541	8.087494234	0.182648565
	C74	4.685267587	5.609752152	0.461154962
	C75	3.615851529	9.866927233	-1.362909989
	C76	2.200096263	9.512182590	-1.824292652
	C77	0.637035754	7.632798881	-2.313142307
	C78	-0.071737953	7.108117616	-1.038989955
	C79	0.851348084	6.515690128	-3.358585834
	C80	-0.346978324	5.664345027	-3.794364616
	C81	-1.332447102	6.302900960	-4.786509881
	C82	-2.954588160	8.148253952	-4.342070615
	C83	0.074692755	5.899455311	1.082609861
	C84	-0.260421289	6.991649618	2.109088815
	C85	0.952265099	4.844881653	1.789142799
	C86	0.639395952	8.942960002	3.281644774
	C87	0.023082719	10.311312912	3.035449674
	C88	-1.117257148	11.759569675	1.444315941
	C89	-2.497531507	12.227053390	1.967352608
	C90	-1.019461007	11.961866879	-0.070583111
	C91	-2.035335522	11.159509331	-0.872898996
	C92	-4.651482690	11.571320366	2.932099078
	C93	-4.771869970	12.530821696	4.108518543
	C94	-5.378913854	10.237518573	3.259577602
	C95	-5.415957678	9.305281997	2.069517533
	C96	-6.451097201	9.401133550	1.126373338
	C97	-4.398914955	8.364272604	1.840466070
	C98	-6.460108413	8.608172402	-0.021928177
	C99	-4.376796454	7.580225546	0.683494283
	C100	-5.407958416	7.718176836	-0.240903171
	C101	5.673485544	-2.810096928	-8.593347853
	C102	4.184691884	-2.614841171	-8.360872754
	C103	2.494958255	-1.259858384	-7.146111897
	C104	2.069983813	0.153224470	-7.560144068

	C105	2.409868263	2.549135488	-7.138695146
	C106	3.567616434	3.533800057	-7.000606088
	C107	5.719695379	4.133627710	-6.033953630
	C108	6.553149863	4.469657874	-7.256005913
	C109	-1.065206200	-2.296433985	-9.879224051
	C110	-2.155228896	-1.258073726	-9.596551548
	C111	-0.798582167	-3.140946585	-8.610463237
	C112	0.019725429	-4.403140092	-8.938607413
	C113	0.475611062	-5.083421421	-7.666401251
	C114	-2.717049750	0.957215070	-8.729795421
	C115	-2.165564053	2.313893341	-9.119871722
	C116	-3.338861778	0.893419148	-7.311918022
	C117	-2.268086480	0.791220456	-6.261521271
	C118	-1.612052075	1.943438080	-5.794659181
	C119	-1.798827039	-0.469224338	-5.851795075
	C120	-0.461963022	1.831916321	-5.006802651
	C121	-0.650132016	-0.579383954	-5.064268629
	C122	0.040816733	0.570807551	-4.672451643
	C123	8.756638245	-5.591898216	-4.065714974
	C124	9.754698090	-5.803545132	-2.917100603
	C125	7.348573588	-6.039064478	-3.649391860
	C126	6.249546930	-5.737594293	-4.614791402
	C127	6.123105922	-6.178404769	-5.916643920
	C128	4.251627952	-5.172853340	-5.295668501
	C129	10.665631586	-7.238735585	-1.189391195
	C130	12.100217337	-6.907633639	-1.533129116
	C131	0.766901812	-6.817438965	-0.972820345
	C132	2.178242238	-6.923986314	-0.406213036
	C133	3.310823231	-6.892495478	-1.479595167
	C134	3.347541423	-8.250515634	-2.230869196
	C135	4.564511461	-8.459311550	-3.135108160
	C136	4.684859225	-9.883709255	-3.718177534
	C137	4.659719762	-6.689002393	-0.766881314
	C138	3.929068220	-0.415434233	-1.659791775
	Fe139	4.481534186	-1.551046769	-3.209733270
	Fe140	2.414131153	0.970966838	-1.113050104
	Fe141	5.232246338	0.645584834	-0.605815023
	Fe142	4.336091124	0.947573171	-2.982277451
	Fe143	4.140127990	3.003445389	-1.277474864
	Fe144	4.850163934	-1.878996800	-0.682813583
	Fe145	2.494616035	-2.152192696	-1.645634777
	H146	-6.170498209	-7.498375146	-1.765756773
	H147	-6.169014596	-6.354967726	-2.933032622
	H148	-9.697382637	-4.901048582	-0.959205141
	H149	-8.536109310	-6.014749094	-2.716395987
	H150	-8.577019206	-7.298678899	-1.515317392
	H151	-8.057617519	-2.953229433	0.238099194
	H152	-9.060210324	-6.041742391	1.645281017
	H153	-9.765248259	-3.244898767	0.624961056
	H154	-8.637701270	-6.941045296	3.746055779
	H155	-6.344707853	-6.222632615	1.795867651
	H156	-8.582602572	-5.298040430	4.419073274
	H157	-3.359494714	-2.066230844	2.385047068
	H158	-5.298340559	-4.047852597	1.681573889
	H159	-4.024530997	-6.495749521	1.881115863
	H160	-5.965095159	-0.950688070	6.120082938
	H161	-6.472485497	-2.931935531	4.079097826
	H162	-4.103191941	-6.791130354	3.637519402
	H163	-5.042851330	-1.794544589	1.899410583
	H164	-5.388390766	-4.517140975	6.103342524
	H165	-7.134731781	-2.297473112	6.213557131
	H166	-0.953903597	-3.826144902	3.008116799
	H167	-1.064841929	-2.573094149	1.754507775
	H168	0.475417419	-2.852029407	2.585096374
	H169	-2.076909611	-1.871366900	3.974591416
	H170	-4.856599648	-5.809023784	7.963667803
	H171	-2.813773018	1.336152484	5.076717057
	H172	-3.032257007	-1.439779610	8.685728682
	H173	-3.405354029	1.915785477	9.360587356
	H174	-0.008634782	-0.293484562	2.512219406
	H175	-0.429771183	0.329620383	4.116732893
	H176	8.341224290	1.410090323	6.224407383
	H177	-3.885777301	2.596136528	5.711403233
	H178	-4.163040329	2.967811575	8.141512421
	H179	-2.122408608	2.773607356	5.880356466
	H180	-2.414211364	3.085063732	8.453524818
	H181	-3.860592890	0.641521860	7.264528000
	H182	-0.854797346	1.205654830	7.389831841
	H183	-2.418262776	-0.792979806	5.985399329
	H184	-1.694506744	0.090673644	2.923927359

H185	1.386246256	4.178049242	1.029674997
H186	0.286124903	4.253852376	2.435018651
H187	2.081104339	4.974455005	3.400415399
H188	2.201556084	4.014521830	5.887423668
H189	4.276088448	3.932618735	3.025887907
H190	6.178912385	2.358886813	2.803371409
H191	5.403025273	0.866538539	6.778584245
H192	-1.894167898	-3.536718667	5.517858128
H193	-0.829660553	-6.461729411	5.594689902
H194	-0.485734903	-6.099521705	7.300554036
H195	-2.967504178	-5.422092391	5.800197459
H196	-4.505674336	-4.333403736	8.903273300
H197	3.465660069	2.403635885	6.976127024
H198	0.742025008	-2.312870587	4.975465985
H199	-2.819441018	-0.780681211	11.386251238
H200	-2.885269328	-2.445876633	10.746857706
H201	-0.200042614	-2.065272262	10.411124389
H202	10.392993998	-7.529047134	5.016428744
H203	-0.888525898	-5.406943919	-5.092247608
H204	1.675890539	-5.776946480	-2.323255816
H205	-0.611423795	-5.111697814	-9.494317109
H206	0.885733702	-4.142900315	-9.564034069
H207	-1.750626497	-3.442933478	-8.146717855
H208	-1.407063684	2.285397112	-9.937735229
H209	-1.525442227	-2.958962181	-10.626596152
H210	-2.868247501	-1.869676971	-3.129948219
H211	-3.590993300	-4.767741492	-2.466633049
H212	-0.764867091	0.057914996	-8.787881678
H213	-3.545416499	0.791416499	-9.447980323
H214	0.686651371	-1.175520436	-9.832409578
H215	-0.754509665	-5.481242990	-3.338075867
H216	-0.273032913	-2.515970754	-7.869947623
H217	0.696024456	-2.403632090	-10.929952629
H218	-6.073298660	-3.063582504	-4.411967801
H219	-3.270886431	-2.310148773	-1.451598201
H220	2.248516065	-4.288178267	-7.945299542
H221	-2.017091369	-4.161060997	-1.973335177
H222	-4.370931985	2.200794407	2.644699944
H223	-3.210181939	0.358740895	0.854883093
H224	-8.708637034	0.318705027	2.377663912
H225	-7.358542837	-0.200519778	-0.237434875
H226	-7.323019189	3.764175771	1.574419885
H227	-9.169278823	8.128887766	3.920301873
H228	-7.813450931	4.542867174	4.624481004
H229	-9.502311270	4.963638721	2.436827252
H230	-6.798587012	6.440176952	2.422823783
H231	-11.101661738	9.312287408	0.341709249
H232	-3.543127093	4.060833877	1.204854236
H233	-4.929868746	3.874254655	0.117287598
H234	-10.492697567	9.961391847	2.735935664
H235	-8.667942902	5.395981788	0.941489312
H236	-6.370820961	1.830937752	0.498791018
H237	-7.538022530	-0.978816313	2.126922295
H238	-9.743934718	1.930664580	-1.735149239
H239	-1.751647514	1.842818062	-2.851578269
H240	-10.034850813	0.190660314	-1.919406710
H241	9.603039915	2.850202962	4.803410148
H242	8.065555291	2.992816832	4.267827346
H243	1.647957945	6.123595040	-0.380436768
H244	-0.881578192	5.425840782	0.816413936
H245	1.480351067	7.958931508	1.610503269
H246	-3.198260390	3.723179611	-1.756524605
H247	-4.634157273	4.489492515	-3.108798518
H248	-5.955307951	3.905297892	-4.825505080
H249	-4.522714641	5.611029233	-1.985627851
H250	-4.783921432	1.757298046	-4.804290351
H251	-6.574239255	1.772705168	-4.901991695
H252	0.008816794	11.765487134	-0.403693552
H253	-1.233308655	13.025336569	-0.262860159
H254	-0.534949914	10.500174716	-2.139960693
H255	-2.176627080	9.947362824	-2.476267310
H256	-2.771701994	9.902920287	-5.390350671
H257	-1.708472183	5.536874208	-5.480862293
H258	-7.282516797	8.683607393	-0.738070643
H259	-3.597114534	8.220586357	2.570101354
H260	-4.276913249	8.087902560	-2.758756601
H261	1.607096546	5.828683146	-2.945215210
H262	2.717925881	7.540381074	-1.852419431
H263	-0.898392633	5.280484543	-2.921465113
H264	-7.980449627	-0.019507170	-2.951561898

	H265	4.098682125	10.441363845	-2.164121515
	H266	-5.447515004	-0.548749299	-4.185118747
	H267	1.312411865	6.979351854	-4.246000418
	H268	-3.542785445	6.902501733	0.495661426
	H269	3.494408121	10.535660995	-0.498434986
	H270	-1.903532890	8.563880506	-6.070000208
	H271	-4.484661066	9.418798498	-3.852855017
	H272	-4.429299178	-3.046360905	-4.715266612
	H273	-5.461973895	-3.206958399	-2.206309138
	H274	-7.272333571	10.103209096	1.290897245
	H275	-4.931566989	1.595489117	-2.318434656
	H276	-0.000516244	8.432034997	-2.704460926
	H277	-0.809107544	7.052130148	-5.394094140
	H278	0.076842242	4.783769744	-4.296015036
	H279	-3.032906025	6.295861280	-3.504721750
	H280	0.106490537	8.482856493	4.127041676
	H281	-0.666356885	9.665283348	1.222273371
	H282	-0.418436866	12.472530267	1.909326934
	H283	1.681629015	9.111117928	3.586157911
	H284	-2.941822428	10.340424528	2.563185170
	H285	6.189753037	-1.840265950	-8.533742006
	H286	6.030742881	-3.417424902	-7.734902798
	H287	8.749408407	-4.505956992	-4.243832343
	H288	6.883716126	-3.732215532	-9.945918295
	H289	5.334119855	-4.253654080	-9.987111747
	H290	5.445898660	4.842838153	0.651405445
	H291	5.385210563	7.296081573	2.451508546
	H292	5.212236680	8.478249058	-1.646850681
	H293	3.937896956	5.589320519	1.266457611
	H294	6.625881494	8.031693241	1.681186074
	H295	6.392494913	3.703034953	-5.275831653
	H296	6.132490470	6.968061763	-0.369544582
	H297	4.464029504	-7.607774247	4.900392809
	H298	6.633669645	-8.144879852	2.798636976
	H299	4.736864159	2.252045820	-5.841807883
	H300	4.673813107	-0.620273523	4.199861744
	H301	2.030039913	2.630512623	-8.163180288
	H302	-0.283416866	-1.563120401	-4.764618814
	H303	1.608231100	2.917709855	-6.476390432
	H304	3.407087548	-5.090983617	4.553741205
	H305	3.236477932	0.865424227	-6.050644575
	H306	3.386175047	-5.453799555	2.821479230
	H307	4.244802904	-7.705006046	3.135848836
	H308	5.904019743	-5.762511875	2.909042748
	H309	5.845179640	-5.563063188	4.665859319
	H310	-4.003311912	0.018269972	-7.279230190
	H311	5.176517857	-10.007812722	4.631799707
	H312	4.587411299	-12.074320143	2.493430213
	H313	0.957869312	0.496733919	-4.092292738
	H314	5.475480102	-12.422028590	5.256493873
	H315	4.007226217	-12.713594511	4.590140694
	H316	5.073448485	-3.613837114	2.557727224
	H317	0.065981450	2.723319635	-4.662762955
	H318	-2.322951177	-1.374503489	-6.166713098
	H319	7.992371515	-9.655697985	-1.540779923
	H320	7.349773193	-7.130861321	-3.484358313
	H321	6.693995556	-8.114066367	0.031608909
	H322	7.353347574	-10.115731785	-2.881621820
	H323	6.456867566	3.754721283	-8.108743626
	H324	6.381948348	-4.990436149	0.266114948
	H325	2.251061055	-7.828939753	0.209575973
	H326	4.600012720	-1.043922541	-7.119979315
	H327	3.331599524	-9.041894187	-1.464166806
	H328	4.425341238	-6.031498711	-7.215813984
	H329	3.234071007	-4.810370751	-5.350588590
	H330	6.119473879	-12.777828824	3.022502925
	H331	5.476281884	-1.448160359	2.928789225
	H332	8.201793097	-5.866227817	1.513044842
	H333	7.202853343	-3.734248404	0.664233349
	H334	8.473856890	-7.157158042	3.441483311
	H335	2.713302471	-3.360138388	5.326839123
	H336	4.514156045	-7.759729921	-3.984037858
	H337	5.489485910	-8.232358303	-2.586918077
	H338	2.419846997	-8.349832931	-2.816365824
	H339	2.431935482	-1.378268249	-6.053104204
	H340	-1.981526241	2.926420838	-6.094064070
	H341	-3.951076168	1.798749011	-7.186458703
	H342	2.295282051	-6.055035402	0.263676520
	H343	8.410273826	-7.362255790	1.135773868
	H344	7.360030042	-9.278055972	0.796875286

	H345	3.309705921	-1.784864890	5.721972000
	H346	6.809336336	-6.731155124	-6.546619377
	H347	7.101991439	-5.588875684	-2.679245806
	H348	1.819257455	-1.970803411	-7.626197252
	H349	5.305973440	5.068110605	-5.622875355
	H350	6.954706470	-0.343870533	5.226400003
	H351	7.259583162	0.284529010	3.590934570
	H352	10.119656851	-5.884091388	-5.567513925
	H353	9.307939096	-7.246778825	-5.165087355
	H354	10.630332002	-8.310687433	-0.938010933
	H355	9.120526632	-7.771755158	-2.558825965
	H356	12.359437994	-7.001870920	-2.614387423
	H357	10.358585232	-6.677241987	-0.293241230
	H358	9.969834175	0.014369226	4.005435563
	H359	-3.871306787	12.591327872	4.764944635
	H360	9.200511350	-7.522392758	6.339850161
	H361	8.745744812	-4.963592959	5.378045623
	H362	-5.145488760	12.046115892	2.067897643
	H363	-4.880887757	9.754837717	4.115519286
	H364	-6.401234612	10.492133455	3.574520530
	H365	1.383345043	-3.072264957	-1.202537937
	H366	4.274842196	-1.480707805	0.645166087
	H367	-6.235571948	6.974014178	-1.841734456
	H368	0.243291559	-0.867199114	-0.539300704
	H369	1.884517536	-1.306604991	1.396602524
	H370	1.392300831	-1.760970544	1.061969267
	H371	-1.817539639	-0.117032888	-1.247297879
	Mo372	4.284218972	-4.057779392	-2.250460387
	N373	-6.745881999	-7.081568023	-2.502134458
	N374	-8.848698218	-4.801371832	-0.408352974
	N375	-8.605360343	-5.428039365	2.317249065
	N376	-5.917022275	-6.234471935	2.723685478
	N377	-4.609163034	-3.762425191	2.374336226
	N378	-5.782492727	-2.422923176	4.638623028
	N379	-4.945921225	-4.028550713	6.886700111
	N380	-2.358932151	-5.470821244	6.621232966
	N381	-0.888372195	-3.407002049	5.596442443
	N382	-1.600171022	-0.550159805	6.549154960
	N383	-2.338535306	-1.040308739	9.323509933
	N384	4.792442734	-12.061405055	4.586748206
	N385	5.779601855	-9.693294252	3.864584713
	N386	4.394250240	-3.694156511	3.318649887
	N387	3.377864315	-2.605077323	5.127323201
	N388	4.962403578	-1.514567676	3.814957849
	N389	8.357663267	-7.393204164	4.452150607
	N390	-5.207781524	-2.546614854	-4.245745840
	N391	-1.256176108	-5.150477393	-4.169404354
	N392	-5.604535915	-0.159059227	-3.239780903
	N393	-8.081972139	0.773471743	-2.321491044
	N394	-8.069400581	0.138888790	0.418514702
	N395	-6.049663286	1.782879698	1.469860704
	N396	-2.950450272	2.802469245	-1.307988582
	N397	-2.315346544	0.757115392	-1.069199484
	N398	-6.831202856	4.335568774	2.256570012
	N399	-10.067260422	7.472694289	0.779844136
	N400	-10.242852481	9.076258616	2.302622258
	N401	8.851187731	2.339751532	4.333231311
	N402	6.085029615	7.162456111	1.713955725
	N403	4.471360667	8.750071895	-1.005912457
	N404	1.948670645	8.199893900	-2.015041291
	N405	-2.488541984	6.918226999	-4.128007999
	N406	-2.460402912	8.939973613	-5.310808712
	N407	-3.897396202	8.659004906	-3.518359001
	N408	0.689262582	6.422222489	-0.139857612
	N409	0.611912633	8.037415363	2.147839731
	N410	-0.701824557	10.440831580	1.882960223
	N411	-1.550216238	10.607384412	-2.018722440
	N412	-3.255124048	11.306392269	2.613695310
	N413	5.913659406	-3.414528503	-9.903433845
	N414	3.850213035	-1.563732297	-7.583642095
	N415	2.679256514	1.147272029	-6.874106152
	N416	4.671435470	3.162998287	-6.303179291
	N417	0.106610934	-1.679657861	-10.513778580
	N418	-1.751506086	-0.086705217	-9.023745863
	N419	9.192848733	-6.239782488	-5.316688784
	N420	5.070994812	-5.094613497	-4.245970661
	N421	4.858829494	-5.812021995	-6.322408076
	N422	9.769816108	-7.020767704	-2.311307467
	N423	0.093874885	2.250567099	0.184237927
	N424	2.105204851	1.071360921	3.656480688

N425	1.005102363	1.802616907	-0.313986810
N426	2.603768688	0.235754158	3.121978847
O427	-6.637364276	-5.309584947	-0.221621389
O428	-7.816920840	-3.327110646	2.726191308
O429	-6.211276116	-5.934790533	4.966221907
O430	-3.129917917	-4.457412932	3.973569283
O431	-3.938790664	-1.093475884	4.620193596
O432	-4.790137983	-2.055516095	8.040725165
O433	-2.122498865	-5.036112400	8.858104646
O434	1.106274817	-4.501741610	5.953303323
O435	0.662898561	-0.713952243	6.802563128
O436	-0.864660508	0.622394466	9.795313591
O437	-0.619657431	-1.759695692	12.385035689
O438	6.804122039	-10.520057676	1.964860124
O439	7.016121925	-8.325101036	6.069422603
O440	10.698667633	-5.108547384	5.949450927
O441	-5.556750834	-0.850896964	-1.051040292
O442	-2.848910058	-3.839793183	-5.129363713
O443	-7.019286519	2.537881665	-1.333376116
O444	-5.723611707	3.452626346	-3.994971237
O445	-10.069276294	1.232487731	0.687717960
O446	-6.397633724	0.953849616	3.570257381
O447	-4.967791521	4.521414503	3.564386212
O448	-7.567062592	6.545943764	4.925575554
O449	9.851143249	-0.632974733	5.936935104
O450	2.701470533	4.150990762	5.061689067
O451	3.479105976	8.382695825	1.012893374
O452	1.372883079	10.427152811	-2.002262852
O453	-1.284379945	7.312970345	-0.871012809
O454	-1.227346880	6.868171958	2.872606831
O455	1.993424680	5.456884091	2.553314026
O456	0.172755287	11.232078722	3.847541306
O457	-2.845517495	13.409244223	1.833779815
O458	-3.223352805	11.068991197	-0.531774324
O459	-5.786062056	13.166918885	4.346544809
O460	-5.349640273	6.961330460	-1.430110130
O461	3.348275733	-3.416137992	-8.844748399
O462	1.243023362	0.348458015	-8.473978357
O463	3.448558308	4.670854637	-7.485682514
O464	7.302973063	5.430605494	-7.304193697
O465	-3.348167044	-1.481804883	-9.859366929
O466	-0.266161622	-5.764690742	-6.966654103
O467	1.750488836	-4.864524767	-7.287095995
O468	-2.540796064	3.375428561	-8.643287369
O469	10.556482158	-4.911182913	-2.577404322
O470	12.948995781	-6.635936298	-0.695075488
O471	0.654125527	-6.039151991	-2.055677625
O472	-0.207568880	-7.357983484	-0.451099262
O473	5.854065520	-10.406882105	-3.736773863
O474	3.634352940	-10.422096838	-4.169193177
O475	5.342626504	-5.627586563	-1.104421917
O476	5.048424841	-7.527968873	0.070043886
O477	3.063202838	-5.805582103	-2.370082476
O478	8.754011348	-6.652379156	1.747339605
O479	6.882173497	-4.584128331	1.022384219
O480	-3.985799064	5.028154359	-2.578878337
O481	8.234735881	-10.050759171	-2.406879149
O482	7.574969784	-8.567269809	0.142173496
S483	3.836273310	5.242637989	-1.144794006
S484	2.675023846	2.360188394	-2.837925116
S485	2.768883792	-2.907767309	-3.684181683
S486	3.767274748	1.868589966	0.563418598
S487	0.845992321	-0.634658922	-1.734561244
S488	4.926854119	-0.061165412	-4.819786809
S489	3.416482295	-3.442034819	-0.136817928
S490	6.030951819	2.103484050	-2.123471281
S491	6.202516273	-2.661897695	-2.349098329
S492	6.474270049	-0.780124927	0.499181763
end			

pre-capture to Fe2-brNH-NH-Fe6

35, S=3/2

reactant

bm526hx7hncapexp3th.car_3			
C1	-7.735400237	-5.367301944	1.275103472

C2	-8.146365782	-6.509053955	0.337556994
C3	-8.524667698	-3.428715717	2.562024325
C4	-7.881315431	-3.687129139	3.920139615
C5	-7.446742378	-5.269543827	5.733297449
C6	-5.922205033	-5.390875950	5.666003763
C7	-3.948074113	-5.699103842	4.273964483
C8	-3.441073136	-4.255429161	4.297857319
C9	-3.873187180	-1.941844309	3.587975502
C10	-3.886851676	-1.305574701	4.987510631
C11	-4.904408621	-1.140081260	7.170154543
C12	-3.850706934	-1.690683724	8.142115985
C13	-2.843942988	-3.705592134	9.121854987
C14	-1.377908009	-4.028164041	8.795447244
C15	0.235399193	-4.819701361	7.113552853
C16	0.972146796	-3.663673177	6.427071651
C17	0.630196611	-1.437667680	5.377564951
C18	0.765204712	-0.222585302	6.310378106
C19	-0.261189591	-1.233174147	4.136256137
C20	-0.033195819	0.123972027	3.446369012
C21	-0.106271271	-2.430578517	3.189919865
C22	-0.353559856	1.535786810	7.604538228
C23	0.018019614	1.215724706	9.064774580
C24	-1.680013136	2.321390428	7.472577807
C25	-1.740936855	3.488489687	8.463707239
C26	-1.862106468	2.831404694	6.036310777
C27	-0.397717716	-0.087896379	11.067505006
C28	1.068664880	-0.351355261	11.346935247
C29	5.899201337	-11.595210907	3.761028587
C30	6.516214889	-10.319568876	3.196881617
C31	6.782269751	-7.890042095	3.679036813
C32	8.002042161	-7.491671891	4.530506892
C33	5.599529102	-6.938600809	3.919752744
C34	5.932094392	-5.496182526	3.550625516
C35	4.690773468	-4.609910618	3.507250336
C36	4.900586462	-2.107673351	3.532332527
C37	10.265961375	-6.558085104	4.485327100
C38	10.499346817	-5.064763679	4.472959025
C39	-5.331069556	-2.677640325	-1.556335546
C40	-5.692110007	-1.244139522	-1.120869091
C41	-3.792082108	-2.823331064	-1.502834218
C42	-3.246763181	-4.262203411	-1.620254649
C43	-2.979117122	-4.747554652	-3.046195911
C44	-6.185896466	0.995536423	-2.025501237
C45	-7.276978042	1.448758748	-1.045316014
C46	-6.364997165	1.603775599	-3.422710044
C47	-9.430848085	0.922718265	-0.001101621
C48	-9.034166585	0.945924700	1.477977938
C49	-7.327347821	0.439792502	3.161389874
C50	-6.155816902	1.400428424	3.371125014
C51	-4.604252295	3.042071399	2.434526463
C52	-5.047011976	4.349755832	3.133197862
C53	-4.023697967	3.478737845	1.080836424
C54	-3.406624229	2.417159102	0.234626264
C55	-2.986205996	1.133223740	0.470752023
C56	-2.551621439	1.603372971	-1.664754308
C57	-6.849404347	6.030412718	3.236643226
C58	-6.758919098	6.185535812	4.753487058
C59	-8.320802079	6.085040174	2.764691058
C60	-8.945534980	7.444663671	2.652031613
C61	-8.869711980	8.548038152	3.479305715
C62	-10.203635990	8.963597917	1.759146571
C63	9.233098498	1.620496600	3.433129183
C64	10.260767368	0.515048267	3.267469728
C65	7.838229894	0.978801185	3.253306168
C66	6.707299856	1.956954013	3.468269686
C67	6.139915901	2.157920306	4.738980576
C68	6.189246236	2.699256286	2.391659731
C69	5.088587957	3.062610507	4.931143376
C70	5.156176166	3.618943201	2.570685564
C71	4.597333349	3.790566762	3.839733603
C72	5.370370092	6.857368588	-1.390462730
C73	4.317133935	7.961348141	-1.600167484
C74	4.695228075	5.521150570	-1.101477417
C75	3.308067415	9.544967948	-3.206200098
C76	1.833635933	9.164340479	-3.366334263
C77	0.214637855	7.268015037	-3.325569579
C78	-0.253280483	6.924962159	-1.887766114
C79	0.244715403	6.023402871	-4.240207893
C80	-1.009009442	5.147144887	-4.347148495
C81	-2.152469810	5.681953022	-5.225846155

	C82	-3.672545213	7.603279720	-4.741981394
	C83	0.266161549	5.984946431	0.310425853
	C84	0.114962979	7.207622390	1.228074356
	C85	1.260242556	5.016807350	0.983191438
	C86	1.203380620	9.271026706	1.965313710
	C87	0.559227031	10.613068606	1.653904079
	C88	-0.855051404	11.872078007	0.123473672
	C89	-2.128069488	12.427772773	0.808695235
	C90	-1.020868452	11.880210592	-1.398121645
	C91	-2.162381995	11.004640052	-1.897511327
	C92	-4.071917344	11.945028725	2.221209192
	C93	-3.983152718	13.045646651	3.270724629
	C94	-4.729186613	10.677421261	2.834617852
	C95	-4.971578800	9.604612984	1.797217014
	C96	-6.157419640	9.596611282	1.046752513
	C97	-4.004495696	8.626017248	1.515246689
	C98	-6.364841071	8.660220046	0.032338582
	C99	-4.181888206	7.696959482	0.485420494
	C100	-5.362362032	7.731954654	-0.251165004
	C101	4.086698392	-3.980963723	-9.005016524
	C102	2.660928322	-3.730164620	-8.548254526
	C103	1.207998837	-2.197243579	-7.238599709
	C104	0.709053690	-0.838804807	-7.745002284
	C105	1.127027486	1.582987547	-7.698834037
	C106	2.297431004	2.542245867	-7.904685941
	C107	4.576309184	3.224046968	-7.398675741
	C108	5.182003165	3.371367526	-8.780981322
	C109	-2.767604212	-3.495478033	-9.151787893
	C110	-3.793490327	-2.406597376	-8.811944834
	C111	-2.276305572	-4.174786531	-7.851259519
	C112	-1.537441282	-5.492089619	-8.151365220
	C113	-0.856103478	-6.017768267	-6.907994422
	C114	-4.199512876	-0.087290618	-8.154271608
	C115	-3.720522901	1.197969199	-8.800668721
	C116	-4.568910386	0.043429457	-6.654659593
	C117	-3.330591295	0.056152791	-5.802962067
	C118	-2.604575785	1.243656832	-5.605769904
	C119	-2.791302652	-1.154868168	-5.338318731
	C120	-1.324601066	1.198548348	-5.043475280
	C121	-1.512604242	-1.199651570	-4.776524578
	C122	-0.762217086	-0.024788762	-4.667334836
	C123	7.909288295	-6.225982305	-4.760163694
	C124	9.095135583	-6.319137145	-3.788654368
	C125	6.599332284	-6.600795537	-4.053317065
	C126	5.344110334	-6.382016815	-4.832682722
	C127	4.979720955	-6.955865190	-6.033828415
	C128	3.255142166	-5.845887463	-5.204469817
	C129	10.305939612	-7.554492326	-2.092868414
	C130	11.655093288	-7.317235266	-2.734342640
	C131	0.598100684	-6.941467174	-0.185077287
	C132	2.088754618	-6.981037292	0.132255672
	C133	3.004868754	-7.098524491	-1.122878479
	C134	2.909366381	-8.533041081	-1.706924624
	C135	3.945804232	-8.872992260	-2.780645705
	C136	3.970801228	-10.364037397	-3.181820712
	C137	4.460551546	-6.839031946	-0.698435831
	C138	3.586932880	-0.699221999	-2.235082234
	Fe139	3.870290290	-1.994903751	-3.710192104
	Fe140	2.187336578	0.825803744	-1.627400148
	Fe141	5.053356403	0.466370954	-1.563742622
	Fe142	3.783229837	0.541486578	-3.828473814
	Fe143	3.899648343	2.741843410	-2.380760414
	Fe144	4.673153425	-2.058962525	-1.327307842
	Fe145	2.181234864	-2.383667126	-1.721270442
	H146	-6.385250118	-7.538245546	0.323380686
	H147	-6.568796593	-6.548199172	-0.960802347
	H148	-9.701938558	-4.772207876	1.395363819
	H149	-8.863944555	-6.115255031	-0.399884343
	H150	-8.707426422	-7.235656426	0.949618129
	H151	-7.863993818	-2.726401155	2.032889470
	H152	-8.636185297	-5.589562789	3.967582906
	H153	-9.485055258	-2.923113320	2.727958817
	H154	-7.864676220	-6.227731802	6.066527222
	H155	-5.988796103	-5.839355207	3.653180450
	H156	-7.666029879	-4.514304504	6.499243875
	H157	-2.879335571	-1.749836231	3.162648386
	H158	-4.957913815	-3.699399263	3.023173486
	H159	-3.702322852	-6.166584330	3.311307867
	H160	-4.749976525	-0.054672803	7.115042733
	H161	-5.633369406	-2.281548765	5.489560125

H162	-3.424970947	-6.243198118	5.068905313
H163	-4.616472174	-1.442540050	2.950467472
H164	-4.188841656	-3.604018216	7.459583062
H165	-5.898853216	-1.332923664	7.596851370
H166	-0.436134097	-3.365944449	3.659532518
H167	-0.696709177	-2.287802532	2.271332503
H168	0.938154815	-2.559559794	2.879998544
H169	-1.293865985	-1.224772792	4.501887707
H170	-3.346936340	-4.651020616	9.375382262
H171	-1.867506067	2.014180216	5.303284066
H172	-1.446774648	-0.257465474	9.214508889
H173	-1.687709736	3.154014231	9.509564036
H174	0.678608793	0.057686859	2.626670636
H175	0.344396490	0.896425274	4.129017214
H176	9.308833216	1.978180697	4.478518198
H177	-2.817483236	3.366987489	5.939805705
H178	-2.684255517	4.037813377	8.329211620
H179	-1.051828448	3.531521653	5.773733031
H180	-0.910593222	4.191350751	8.290097387
H181	-2.505105836	1.623904605	7.705970786
H182	0.475083998	2.167342942	7.249099935
H183	-1.287549201	0.027413005	6.392543692
H184	-0.978192982	0.501469786	3.027488704
H185	1.554182449	4.246836255	0.255675330
H186	0.722931401	4.526988268	1.808253400
H187	2.666530555	5.320207361	2.331049756
H188	3.211757775	4.680089374	4.869777194
H189	4.755576694	4.175509429	1.723345051
H190	6.584051313	2.535260797	1.386325946
H191	6.515931825	1.592039221	5.596168602
H192	-0.813404361	-2.716576189	6.263625701
H193	0.210746804	-5.662856762	6.408466505
H194	0.833179923	-5.124980148	7.981688609
H195	-1.863562852	-4.563567969	6.844682386
H196	-2.824319646	-3.071835525	10.016645859
H197	4.644988349	3.191488945	5.922324787
H198	1.658024272	-1.647556437	5.058558767
H199	-0.745854007	0.718804791	11.733465614
H200	-0.945993600	-1.006575445	11.328249737
H201	1.657254807	-0.732858207	10.480073265
H202	11.138053823	-7.063646226	4.043548384
H203	-1.758586388	-5.991133887	-4.123740692
H204	1.227865873	-6.065333619	-1.784662339
H205	-2.264477148	-6.246689217	-8.484420050
H206	-0.802413245	-5.341988094	-8.955227088
H207	-3.127321688	-4.384654895	-7.184504303
H208	-3.118351677	1.046646010	-9.727584545
H209	-3.354018991	-4.237917507	-9.713858265
H210	-3.354450074	-2.196714623	-2.298912959
H211	-3.956179829	-4.973967516	-1.163844500
H212	-2.286910109	-1.026973696	-8.428927593
H213	-5.140187813	-0.322087152	-8.694530318
H214	-1.044451574	-2.407422861	-9.554579306
H215	-1.331907451	-5.865824936	-2.421401388
H216	-1.621910159	-3.472558004	-7.310812705
H217	-1.214326967	-3.766702249	-10.468506153
H218	-6.741031965	-3.459751079	-2.849149565
H219	-3.460747859	-2.415292489	-0.536015946
H220	0.832825905	-5.286132523	-7.592852548
H221	-2.320413743	-4.345780211	-1.033508106
H222	-3.828776837	2.603196337	3.078284227
H223	-3.023465241	0.522251724	1.363187516
H224	-8.141364973	0.782952022	3.814330826
H225	-7.265104291	-0.074135274	1.089920754
H226	-6.925228847	4.080573557	2.354487604
H227	-8.319374553	8.737491786	4.393244809
H228	-6.909040340	5.229984841	5.312435475
H229	-8.929893298	5.423312425	3.406268512
H230	-6.264421783	6.833926765	2.762395553
H231	-10.882703612	9.511735869	1.111493923
H232	-3.263381426	4.248582644	1.288872208
H233	-4.819346179	3.967186495	0.495464688
H234	-9.834578288	10.441855579	3.247571917
H235	-8.353570283	5.646902235	1.754903898
H236	-6.174190270	2.011841537	1.379063749
H237	-7.020815907	-0.550176937	3.530631056
H238	-9.877486210	1.904412313	-0.211647459
H239	-2.209180763	1.503208460	-2.692225904
H240	-10.212583023	0.164696058	-0.138368362
H241	10.298067350	3.203557190	2.692511916

H242	8.691743540	3.310289907	2.415888258
H243	1.547875514	5.973469360	-1.428874557
H244	-0.720264714	5.498980716	0.284070095
H245	1.736091501	8.068923435	0.310198133
H246	-3.436438995	3.542983572	-1.608090988
H247	-5.110238484	4.156907054	-2.784947496
H248	-6.714480886	3.390622415	-4.155708253
H249	-4.796237453	5.409878118	-1.851201215
H250	-5.542366819	1.243914923	-4.062778033
H251	-7.322566736	1.275884402	-3.855752669
H252	-0.067779598	11.615114217	-1.875966311
H253	-1.262225192	12.914867054	-1.687874785
H254	-0.909490804	10.159843334	-3.314426787
H255	-2.580177702	9.595518878	-3.276373380
H256	-3.664721476	9.214540048	-6.013983022
H257	-2.646411681	4.848820975	-5.748636405
H258	-7.300731823	8.656667417	-0.532868198
H259	-3.085740271	8.567038936	2.104014703
H260	-4.704776685	7.762913247	-2.963974473
H261	1.064564861	5.378502963	-3.885459878
H262	2.343909711	7.195495969	-3.228378465
H263	-1.395801319	4.886481406	-3.349600815
H264	-8.365612756	-0.225647764	-1.470804121
H265	3.639749427	10.004569780	-4.146804450
H266	-6.092783148	-0.955996048	-3.051890609
H267	0.537784628	6.362378750	-5.247283145
H268	-3.387194448	6.990955379	0.239809624
H269	3.340189288	10.317905070	-2.424768074
H270	-2.914419026	7.787821203	-6.654173360
H271	-5.099282188	8.950704328	-4.161325303
H272	-5.173665815	-3.516887895	-3.430607368
H273	-5.757057477	-3.339760426	-0.780137608
H274	-6.940789507	10.327354679	1.260422522
H275	-5.258804990	1.392589925	-1.588397185
H276	-0.485683701	8.021945702	-3.697598106
H277	-1.737628324	6.341840216	-5.997875813
H278	-0.676665639	4.203322201	-4.801251919
H279	-3.603218337	5.869291077	-3.678700909
H280	0.816046709	8.933676456	2.938493063
H281	-0.455923982	9.756165989	0.099122903
H282	-0.089048866	12.623843941	0.368961607
H283	2.281975897	9.449551968	2.066752836
H284	-2.444849792	10.646352418	1.724403377
H285	4.605813802	-3.023036170	-9.153159517
H286	4.585935735	-4.488545898	-8.152782793
H287	7.868390106	-5.168260631	-5.060211599
H288	5.048275903	-5.082291290	-10.423201465
H289	3.512612261	-5.574297503	-10.134713279
H290	5.484404311	4.772882555	-0.956971021
H291	5.704794374	7.426277702	0.542290439
H292	4.825885950	8.091645378	-3.589401704
H293	4.094700617	5.596559089	-0.184071026
H294	6.810599438	8.027021793	-0.503253997
H295	5.374388408	2.887641236	-6.719552412
H296	5.980208646	6.760817645	-2.302025267
H297	5.290179145	-7.009988359	4.975880351
H298	7.048363968	-7.874523713	2.614073397
H299	3.645153951	1.401445604	-6.792173453
H300	5.168941987	-0.094077444	3.281583229
H301	0.559060564	1.550817728	-8.636063531
H302	-1.092888443	-2.152125980	-4.444276477
H303	0.473313022	2.057879567	-6.946614534
H304	4.259431893	-4.489898412	4.507945024
H305	2.141704362	0.023264845	-6.584405023
H306	3.914786204	-5.067813013	2.873203490
H307	4.762289565	-7.299700153	3.300933996
H308	6.402204569	-5.473631167	2.556291687
H309	6.655362545	-5.064245361	4.259812456
H310	-5.215256014	-0.806939607	-6.393671070
H311	5.926665372	-9.449549418	4.897527955
H312	4.985118447	-11.773353546	3.174162392
H313	0.253094321	-0.046118328	-4.279162408
H314	6.338755983	-11.766342995	5.765316425
H315	4.783818074	-12.132067720	5.405213288
H316	5.529749047	-3.329044360	2.053053747
H317	-0.742489358	2.111605221	-4.909278752
H318	-3.363668091	-2.078937821	-5.442063927
H319	7.619442124	-9.940531166	-1.667241691
H320	6.630795613	-7.669158841	-3.778153089
H321	6.622115072	-8.186479382	-0.101446271

H322	6.754433121	-10.544911052	-2.810697297
H323	4.955398583	2.538386286	-9.489027861
H324	6.357813684	-5.060748835	-0.222445794
H325	2.287126394	-7.797222934	0.838047186
H326	3.282126399	-2.026439789	-7.600774675
H327	3.027019851	-9.230663392	-0.863078672
H328	3.069533997	-6.910113968	-7.008478714
H329	2.246142491	-5.467559074	-5.109985600
H330	6.594560893	-12.418380825	3.523618138
H331	5.744326581	-1.049614890	1.950251434
H332	8.366128530	-5.801311071	0.788995421
H333	7.228846636	-3.779415009	-0.156369894
H334	8.970398654	-6.851707153	2.795057722
H335	3.716409560	-2.707824315	5.149518018
H336	3.736108740	-8.287578034	-3.688868359
H337	4.953278613	-8.592544885	-2.445318750
H338	1.894200665	-8.680130759	-2.106422988
H339	1.348041164	-2.170022939	-6.146080022
H340	-3.028190171	2.193043320	-5.941865103
H341	-5.152430413	0.969706807	-6.544895354
H342	2.309570765	-6.031031010	0.647383959
H343	8.505699374	-7.337427636	0.582137663
H344	7.412809880	-9.258348237	0.676050043
H345	4.156469084	-1.044031961	5.118863330
H346	5.532318177	-7.588871193	-6.715968809
H347	6.531070316	-6.049070239	-3.107998659
H348	0.457142840	-2.949330838	-7.487493878
H349	4.238863111	4.212660542	-7.051267712
H350	7.758883031	0.147760015	3.969342427
H351	7.779104326	0.555388954	2.237950476
H352	8.980071329	-6.716736416	-6.439593494
H353	8.260992084	-8.008314116	-5.734771414
H354	10.307613469	-8.579890689	-1.691872891
H355	8.546909085	-8.224838354	-3.096131141
H356	11.697892288	-7.545528103	-3.826313440
H357	10.172029535	-6.867450033	-1.242134280
H358	10.545230481	0.306081065	2.207861301
H359	-2.974289384	13.186910135	3.724859282
H360	10.195764432	-6.864838565	5.544102720
H361	9.577844595	-4.438560811	4.387957030
H362	-4.710962999	12.318007366	1.403548993
H363	-4.086827842	10.296459123	3.644190178
H364	-5.678988069	10.991775288	3.291414135
H365	1.099251783	-3.206732126	-1.003327245
H366	4.400958231	-1.404337884	-0.036221473
H367	-6.459276364	6.803472072	-1.567523441
H368	0.081843079	-0.929864257	-0.565704021
H369	-2.035454845	-0.251731978	-0.869630743
Mo370	3.848036232	-4.402584029	-2.411070106
N371	-7.072666464	-7.198843883	-0.353702482
N372	-8.766515430	-4.619227740	1.764310860
N373	-8.071293367	-4.909093452	4.470380019
N374	-5.389077222	-5.768796211	4.476896829
N375	-4.169372639	-3.366291957	3.574624813
N376	-4.852585319	-1.719649123	5.839398269
N377	-3.625713634	-3.030202761	8.093326602
N378	-1.119819198	-4.515720249	7.546754262
N379	0.188510275	-2.606133187	6.119665455
N380	-0.387471313	0.333107321	6.770841855
N381	-0.649471864	0.198161664	9.666470772
N382	5.545034597	-11.488713930	5.183222120
N383	6.381927273	-9.248021246	4.001057376
N384	5.030433628	-3.300851353	2.945281293
N385	4.299709541	-1.974489515	4.735162585
N386	5.394282907	-1.016823389	2.920184646
N387	9.035159021	-6.957794337	3.832222944
N388	-5.858014334	-2.948137891	-2.898423386
N389	-1.955360610	-5.607566263	-3.192953392
N390	-6.077636918	-0.447078022	-2.152040405
N391	-8.355125444	0.640011321	-0.935857228
N392	-7.854890787	0.350419519	1.813018653
N393	-5.687871532	2.079248119	2.277714729
N394	-3.124511903	2.675005375	-1.100784578
N395	-2.471406536	0.658366369	-0.720046864
N396	-6.320644575	4.727231589	2.854983878
N397	-9.781506148	7.717140648	1.580895781
N398	-9.672968663	9.502910295	2.893146678
N399	9.477437926	2.654216903	2.424735159
N400	6.273947602	7.185140769	-0.275680152
N401	4.214828427	8.462664844	-2.866660097

N402	1.556089146	7.844222310	-3.338692197
N403	-3.174498315	6.400177831	-4.458051846
N404	-3.355743083	8.258205291	-5.872694144
N405	-4.455374120	8.234291588	-3.837011030
N406	0.652953620	6.331772588	-1.059230343
N407	0.976952384	8.232820495	0.977571734
N408	-0.365072690	10.611696033	0.646083139
N409	-1.887542679	10.299651398	-3.029350679
N410	-2.751360175	11.615621350	1.698761282
N411	4.098438620	-4.743136427	-10.255151841
N412	2.463893054	-2.580688515	-7.870173679
N413	1.434702822	0.217233428	-7.312959254
N414	3.499376153	2.248089413	-7.347354993
N415	-1.728924896	-2.986131357	-10.055768736
N416	-3.299055186	-1.179868657	-8.475558480
N417	8.117271602	-7.024179693	-5.981903974
N418	4.251755974	-5.676430436	-4.335494326
N419	3.665068054	-6.604255796	-6.244082345
N420	9.223011766	-7.457932371	-3.055629305
N421	0.127533220	2.269977815	-0.120505900
N422	2.482580974	-0.438706814	1.324001040
N423	0.952866481	1.751069171	-0.694552558
N424	1.785180170	-1.300301122	1.212636895
O425	-6.557615169	-5.150003887	1.610570683
O426	-7.217804357	-2.794143975	4.475565670
O427	-5.230217571	-5.157294703	6.670681289
O428	-2.445625425	-3.921575688	4.957678323
O429	-3.041824984	-0.454862738	5.305576557
O430	-3.290282175	-0.932894180	8.953424670
O431	-0.505944476	-3.895641151	9.662924936
O432	2.188570492	-3.747118446	6.173876454
O433	1.889931923	0.202226451	6.620898682
O434	0.887536868	1.870494073	9.664264209
O435	1.569615115	-0.222976253	12.453444536
O436	7.057718898	-10.330517441	2.072808664
O437	7.999699565	-7.655615502	5.763744355
O438	11.605685884	-4.556931366	4.566842489
O439	-5.615948174	-0.847922107	0.058780946
O440	-3.688530350	-4.383072618	-4.012325021
O441	-7.140615707	2.495141001	-0.386382768
O442	-6.339288625	3.041837522	-3.327269858
O443	-9.791691202	1.483769483	2.295941589
O444	-5.664183926	1.531970250	4.495447343
O445	-4.261682869	5.033839157	3.794580490
O446	-6.588985698	7.245732167	5.330344229
O447	10.738214259	-0.122352936	4.195166153
O448	3.548513548	4.683233932	3.954839904
O449	3.603994057	8.384059840	-0.673018399
O450	0.987311986	10.066599658	-3.519106670
O451	-1.412235810	7.188601241	-1.530852671
O452	-0.700019918	7.204480123	2.160176633
O453	2.417355343	5.703725404	1.465496645
O454	0.858061963	11.627364395	2.295446287
O455	-2.506481759	13.584669576	0.576392946
O456	-3.270993513	10.986436768	-1.343634612
O457	-4.944478303	13.710490781	3.621046764
O458	-5.513004365	6.832496005	-1.326643950
O459	1.757091942	-4.572250117	-8.771741502
O460	-0.276568928	-0.737302033	-8.504051320
O461	2.104169545	3.600596756	-8.525533290
O462	5.896902338	4.306691260	-9.101243765
O463	-5.013304911	-2.640267816	-8.825143233
O464	-1.452537266	-6.605865809	-6.012316711
O465	0.463464454	-5.768156195	-6.789124557
O466	-3.995510658	2.320747709	-8.400407207
O467	9.939916116	-5.406796145	-3.701105562
O468	12.651834038	-6.974064574	-2.115202441
O469	0.280609362	-6.294947982	-1.314885008
O470	-0.259960331	-7.412955602	0.558633966
O471	5.122696136	-10.901956756	-3.348037277
O472	2.861122883	-10.945041701	-3.347408784
O473	5.070202639	-5.843904426	-1.289411061
O474	5.008339483	-7.567867013	0.150790038
O475	2.597851347	-6.110771693	-2.071757500
O476	8.947940283	-6.563289999	1.029945834
O477	6.986812738	-4.568055855	0.366042564
O478	-4.378383558	4.744022856	-2.452617492
O479	7.705760629	-10.440709226	-2.509102169
O480	7.508373909	-8.637373515	-0.090286264
S481	3.587400632	4.990505104	-2.488218973

	S482	2.095224443	1.999375229	-3.527392483
	S483	2.100601085	-3.368491478	-3.665823088
	S484	3.817326466	1.898987124	-0.351370466
	S485	0.557159289	-0.828811090	-1.840370352
	S486	4.065538243	-0.819248877	-5.566273398
	S487	3.415063625	-3.507283390	-0.270445486
	S488	5.703250828	1.680276911	-3.314764421
	S489	5.713118323	-3.100889342	-3.079124708
	S490	6.426660146	-0.851131973	-0.451397003
	end			

TS

	bm526hx7hncapexp3th_1_53246.76			
Fe(139) -2.213	C1	-7.723150441	-5.358647881	1.268747404
Fe(140) -0.318	C2	-8.149017918	-6.501951087	0.338605689
Fe(141) -2.536	C3	-8.506813144	-3.418470186	2.558342880
Fe(142) 2.535	C4	-7.856288014	-3.677739812	3.911634277
Fe(143) 2.540	C5	-7.423545683	-5.254033796	5.731930131
Fe(144) 1.698	C6	-5.899129954	-5.368730903	5.666186850
Fe(145) 1.350	C7	-3.922077968	-5.686588551	4.278098972
	C8	-3.419213415	-4.241069455	4.294581126
	C9	-3.860505061	-1.930901503	3.571649107
	C10	-3.877422529	-1.289446513	4.969177031
	C11	-4.889396012	-1.123259589	7.153062920
	C12	-3.843065201	-1.672818969	8.133718944
	C13	-2.836661374	-3.689658551	9.114228936
	C14	-1.371966491	-4.019260143	8.787654660
	C15	0.230181150	-4.836377710	7.106327506
	C16	0.964760963	-3.675140018	6.424624960
	C17	0.618795714	-1.455270976	5.364129712
	C18	0.757348957	-0.240019221	6.295314032
	C19	-0.267087988	-1.252418822	4.115249594
	C20	-0.060407601	0.110647023	3.426038415
	C21	-0.071706353	-2.418354659	3.138145446
	C22	-0.349436470	1.523597649	7.592626782
	C23	0.030409019	1.207183539	9.052420853
	C24	-1.674374984	2.311750175	7.464511545
	C25	-1.731985582	3.478192870	8.456533658
	C26	-1.859394258	2.820345411	6.027965386
	C27	-0.394307603	-0.087542573	11.063885171
	C28	1.072573317	-0.347072910	11.342777755
	C29	5.900440529	-11.583708638	3.767990468
	C30	6.516502450	-10.311499843	3.199220110
	C31	6.778004524	-7.881434492	3.677007923
	C32	7.996919499	-7.483294945	4.529249065
	C33	5.591966649	-6.935307972	3.915982358
	C34	5.919740649	-5.492932899	3.549131562
	C35	4.678903612	-4.606753642	3.514155309
	C36	4.910298599	-2.107576039	3.562048092
	C37	10.265200648	-6.559917178	4.488373488
	C38	10.505647952	-5.067645208	4.476941838
	C39	-5.316002883	-2.673610904	-1.552983552
	C40	-5.687192859	-1.243646201	-1.112330933
	C41	-3.779323605	-2.819922371	-1.505259835
	C42	-3.243701124	-4.262707453	-1.620614791
	C43	-2.978464525	-4.750290051	-3.045271665
	C44	-6.186742974	0.997005700	-2.018907874
	C45	-7.280770835	1.448299865	-1.040361420
	C46	-6.368784871	1.601528383	-3.415284815
	C47	-9.435463176	0.916768228	0.001888374
	C48	-9.039237000	0.942648937	1.481131303
	C49	-7.328481142	0.441255729	3.165653149
	C50	-6.152582165	1.398264846	3.373649032
	C51	-4.602341290	3.040290496	2.436301338
	C52	-5.047180501	4.344462431	3.135680222
	C53	-4.027793905	3.478260723	1.080894381
	C54	-3.407948944	2.417329629	0.235010520
	C55	-2.994214669	1.131011242	0.469628926
	C56	-2.557379866	1.600021987	-1.665096109
	C57	-6.847198488	6.025946785	3.231505396
	C58	-6.761745584	6.182509411	4.748519216
	C59	-8.317757642	6.080991562	2.755147321
	C60	-8.946537337	7.439593001	2.650621969
	C61	-8.868553446	8.543501483	3.476656077
	C62	-10.206611506	8.957128829	1.759073910
	C63	9.236042890	1.618184328	3.447459090
	C64	10.254493003	0.504970914	3.277987371
	C65	7.840001938	0.978653625	3.263760389
	C66	6.707111662	1.955741768	3.475961599
	C67	6.140710611	2.160801488	4.746172891

C68	6.183966677	2.690573276	2.396382135
C69	5.086920868	3.063216189	4.937497866
C70	5.148491849	3.608651328	2.574308587
C71	4.594399157	3.787129295	3.843677092
C72	5.374523436	6.868516600	-1.384909675
C73	4.319198311	7.966849393	-1.594210765
C74	4.696125868	5.527085067	-1.100485783
C75	3.309807133	9.544720608	-3.204983294
C76	1.835426966	9.163111871	-3.364791074
C77	0.213374887	7.272133786	-3.321617716
C78	-0.255955361	6.925242001	-1.885565863
C79	0.244560107	6.024888101	-4.234124392
C80	-1.008045360	5.147133698	-4.342803678
C81	-2.151554998	5.681566445	-5.223581781
C82	-3.670096729	7.604567585	-4.738875632
C83	0.262865048	5.986943886	0.314869084
C84	0.109835796	7.211739455	1.228470632
C85	1.251417653	5.016101385	0.991841812
C86	1.207400227	9.273492795	1.962697648
C87	0.561827880	10.616594432	1.652158878
C88	-0.856348886	11.876766800	0.121241717
C89	-2.128686148	12.429590424	0.808510424
C90	-1.027043114	11.880084104	-1.401789840
C91	-2.164861425	10.998113584	-1.896870328
C92	-4.074113883	11.945316925	2.219896175
C93	-3.979839312	13.044562902	3.269327201
C94	-4.727643204	10.675296048	2.833256430
C95	-4.974305883	9.600713081	1.797139871
C96	-6.163624993	9.589517466	1.051422105
C97	-4.004351066	8.627105975	1.509155969
C98	-6.371221354	8.653873862	0.036052258
C99	-4.181711316	7.700797817	0.476691799
C100	-5.365681358	7.730773919	-0.254464113
C101	4.086704214	-3.978870719	-9.003335571
C102	2.659753444	-3.727838026	-8.547371223
C103	1.209722769	-2.200074270	-7.229150747
C104	0.714082151	-0.838961376	-7.732209347
C105	1.128825895	1.580246102	-7.688406959
C106	2.294422696	2.546354057	-7.896469077
C107	4.574929513	3.238086530	-7.399221785
C108	5.194478145	3.368792295	-8.776275871
C109	-2.762813137	-3.492663296	-9.142040091
C110	-3.790828151	-2.406719678	-8.800435797
C111	-2.273990942	-4.177759799	-7.845865918
C112	-1.535933875	-5.493689995	-8.150053972
C113	-0.851284305	-6.016538507	-6.907161078
C114	-4.195467516	-0.087679227	-8.143911982
C115	-3.719370706	1.198210426	-8.790158957
C116	-4.569912270	0.044062900	-6.646270373
C117	-3.333221943	0.052086831	-5.794471218
C118	-2.605489395	1.238256382	-5.596356202
C119	-2.793734753	-1.161163959	-5.335282901
C120	-1.323586454	1.191244590	-5.037720981
C121	-1.514063274	-1.207284936	-4.777745222
C122	-0.761264102	-0.033158197	-4.669063076
C123	7.905163120	-6.221404953	-4.755239570
C124	9.091234943	-6.318192312	-3.783948181
C125	6.594496293	-6.597911273	-4.046914371
C126	5.333324648	-6.357631557	-4.811100275
C127	4.957010925	-6.923965300	-6.011896646
C128	3.246288058	-5.802069992	-5.168286356
C129	10.304716083	-7.559175616	-2.093945196
C130	11.653255318	-7.314169892	-2.736539659
C131	0.593304932	-6.953612643	-0.182261597
C132	2.084330944	-6.989929879	0.133497443
C133	3.001320822	-7.100353644	-1.120025179
C134	2.899980684	-8.529135442	-1.718507145
C135	3.939882728	-8.874290754	-2.789236263
C136	3.964116034	-10.367023502	-3.185956995
C137	4.460345623	-6.856999987	-0.699202929
C138	3.600406519	-0.689530642	-2.236109772
Fe139	3.791378446	-2.020961819	-3.648058950
Fe140	2.218526153	0.764833269	-1.518808280
Fe141	5.079270672	0.497839803	-1.556022294
Fe142	3.793782687	0.497384960	-3.793760908
Fe143	3.923717351	2.744614779	-2.383934591
Fe144	4.741902853	-1.996975694	-1.241410533
Fe145	2.196837682	-2.397385624	-1.548492228
H146	-6.393844305	-7.544179664	0.311607756
H147	-6.587108119	-6.561353445	-0.978707677

	H148	-9.689443649	-4.761486762	1.398108185
	H149	-8.869163310	-6.104617252	-0.394821150
	H150	-8.713092751	-7.221691755	0.956412602
	H151	-7.850702224	-2.714821574	2.025018951
	H152	-8.617517572	-5.578038041	3.969693086
	H153	-9.467293136	-2.914411346	2.731700045
	H154	-7.839159325	-6.213316870	6.067198381
	H155	-5.963712587	-5.839245861	3.660943024
	H156	-7.647988052	-4.497576680	6.494967362
	H157	-2.868731240	-1.732406766	3.144403952
	H158	-4.933348161	-3.697423823	3.013568079
	H159	-3.674621126	-6.160187989	3.318824245
	H160	-4.729680397	-0.039791984	7.091001796
	H161	-5.613558184	-2.277306171	5.479146925
	H162	-3.397226777	-6.222290222	5.076569984
	H163	-4.608106745	-1.440008583	2.931511290
	H164	-4.170519729	-3.583829006	7.444321453
	H165	-5.886830327	-1.308058294	7.577172994
	H166	-0.340688545	-3.381679673	3.593458815
	H167	-0.696574694	-2.282126533	2.242569201
	H168	0.968194777	-2.465300507	2.788248084
	H169	-1.301383999	-1.271976902	4.476758222
	H170	-3.345669179	-4.631479726	9.367556423
	H171	-1.865661636	2.001904625	5.295658860
	H172	-1.443988093	-0.252249263	9.210701863
	H173	-1.670484053	3.141891100	9.500367632
	H174	0.595620149	0.030308644	2.547346820
	H175	0.368674244	0.871753756	4.093236838
	H176	9.309795314	1.975184129	4.492459549
	H177	-2.814798021	3.355762389	5.930650004
	H178	-2.679011959	4.023242079	8.329329053
	H179	-1.048727343	3.519975162	5.763024794
	H180	-0.905264114	4.184129644	8.277864304
	H181	-2.500125111	1.615711141	7.700984782
	H182	0.478388048	2.150540093	7.227738571
	H183	-1.292754331	0.012127357	6.388687297
	H184	-1.029884933	0.509209344	3.086624522
	H185	1.538261885	4.241015004	0.266635668
	H186	0.711557899	4.533825665	1.819478882
	H187	2.661247854	5.315179239	2.336392861
	H188	3.208592988	4.680941690	4.870396440
	H189	4.746110498	4.162204405	1.726240236
	H190	6.578138842	2.522960301	1.391340520
	H191	6.517320107	1.598740426	5.605393702
	H192	-0.829120169	-2.752993610	6.211617849
	H193	0.200396860	-5.673562081	6.394496773
	H194	0.832241379	-5.149323159	7.968602303
	H195	-1.868212949	-4.583085015	6.849678992
	H196	-2.814416268	-3.055868631	10.007488366
	H197	4.644542364	3.195231170	5.928929130
	H198	1.646857378	-1.662852679	5.042676401
	H199	-0.748103771	0.714616504	11.731442515
	H200	-0.936356914	-1.010913926	11.319231074
	H201	1.656288633	-0.739670792	10.477435065
	H202	11.135588875	-7.070132582	4.048396925
	H203	-1.762079513	-5.996597276	-4.121357034
	H204	1.208472275	-6.058907648	-1.771703699
	H205	-2.263219485	-6.248337411	-8.484613400
	H206	-0.801844232	-5.341020712	-8.955098313
	H207	-3.127090862	-4.390338059	-7.183311310
	H208	-3.123451062	1.048689100	-9.722175191
	H209	-3.348706506	-4.234242267	-9.707342816
	H210	-3.342918065	-2.198966193	-2.305522842
	H211	-3.961344824	-4.967929146	-1.166583185
	H212	-2.284554335	-1.026111560	-8.424169507
	H213	-5.135386207	-0.320975520	-8.685937747
	H214	-1.037015872	-2.406889389	-9.545487979
	H215	-1.335046434	-5.871827063	-2.417473541
	H216	-1.620226610	-3.479245663	-7.299666902
	H217	-1.216896304	-3.761195190	-10.465971264
	H218	-6.732847198	-3.455301815	-2.838291297
	H219	-3.444637953	-2.411558752	-0.540038093
	H220	0.833188804	-5.280372919	-7.597653728
	H221	-2.321512774	-4.355011723	-1.030444260
	H222	-3.822380809	2.603562878	3.074773133
	H223	-3.038783808	0.519081902	1.361164671
	H224	-8.141294812	0.788572654	3.817992734
	H225	-7.267056065	-0.071242811	1.092828922
	H226	-6.922292384	4.080111562	2.343379008
	H227	-8.314169343	8.736585631	4.387658095

	H228	-6.903689168	5.226011894	5.307867038
	H229	-8.924996457	5.412704326	3.391842232
	H230	-6.260453193	6.829352935	2.758801309
	H231	-10.884940056	9.505134006	1.111114952
	H232	-3.272648566	4.252897011	1.287160288
	H233	-4.828568506	3.960989233	0.496364555
	H234	-9.832733041	10.438827786	3.242689642
	H235	-8.348294135	5.649041454	1.741516864
	H236	-6.171404949	2.007895929	1.381899203
	H237	-7.027314365	-0.549764315	3.534880787
	H238	-9.885680779	1.896835220	-0.211731863
	H239	-2.219582159	1.499561234	-2.693992300
	H240	-10.214345914	0.155692820	-0.134636573
	H241	10.300688143	3.198758972	2.697627550
	H242	8.693218418	3.306390584	2.427962516
	H243	1.548154776	5.982552076	-1.422208038
	H244	-0.724278131	5.502863057	0.284505042
	H245	1.739535244	8.062870460	0.314931630
	H246	-3.437170193	3.541992709	-1.610199102
	H247	-5.104259521	4.154402090	-2.781032415
	H248	-6.720159716	3.388162072	-4.146555191
	H249	-4.794882398	5.405838970	-1.847688095
	H250	-5.552221071	1.234158244	-4.059518708
	H251	-7.330261918	1.278576189	-3.842162173
	H252	-0.072959603	11.621114660	-1.882018979
	H253	-1.275609725	12.912634042	-1.692120840
	H254	-0.910217525	10.144417936	-3.307760415
	H255	-2.580700286	9.578548372	-3.265109048
	H256	-3.650411153	9.221131156	-6.003867695
	H257	-2.642514616	4.848560313	-5.747726019
	H258	-7.308176424	8.649028848	-0.526741099
	H259	-3.085171109	8.566616928	2.096585443
	H260	-4.709430001	7.758007093	-2.964319271
	H261	1.063602580	5.379249560	-3.876592563
	H262	2.343585712	7.193802259	-3.220937299
	H263	-1.395563375	4.884438393	-3.346217973
	H264	-8.363104902	-0.232135229	-1.463067323
	H265	3.643653478	10.000551544	-4.146654801
	H266	-6.084324043	-0.954842915	-3.043549665
	H267	0.539669716	6.361924445	-5.241176786
	H268	-3.385563491	6.997544087	0.226525936
	H269	3.342357779	10.319095434	-2.425415787
	H270	-2.901439659	7.794356827	-6.644318354
	H271	-5.098355788	8.951650595	-4.159559522
	H272	-5.169289539	-3.512435704	-3.429962818
	H273	-5.738180090	-3.338459949	-0.776616159
	H274	-6.949332810	10.317010981	1.268743577
	H275	-5.260796039	1.398981298	-1.581687799
	H276	-0.483733638	8.026017379	-3.696133696
	H277	-1.734924847	6.341439346	-5.995323409
	H278	-0.674684167	4.204279784	-4.797837887
	H279	-3.607677704	5.866097926	-3.681202680
	H280	0.825744835	8.941260050	2.939097660
	H281	-0.455777262	9.760089735	0.098481151
	H282	-0.089798618	12.628940078	0.360876336
	H283	2.286828385	9.451009461	2.060272559
	H284	-2.445606371	10.646765333	1.722996880
	H285	4.607408344	-3.020479868	-9.147749093
	H286	4.586075175	-4.490282494	-8.153675790
	H287	7.863764790	-5.162493466	-5.051894097
	H288	5.046662965	-5.074659213	-10.428831895
	H289	3.513742122	-5.569689521	-10.137495296
	H290	5.484441001	4.778726873	-0.950279559
	H291	5.709058753	7.430775100	0.549244739
	H292	4.831202485	8.093958464	-3.581720974
	H293	4.091203145	5.600812384	-0.186111175
	H294	6.819067144	8.025116091	-0.495511407
	H295	5.366163057	2.914885371	-6.704865378
	H296	5.985706927	6.770357223	-2.295478951
	H297	5.280555333	-7.007126642	4.971385385
	H298	7.044561798	-7.864797648	2.612368605
	H299	3.645600510	1.422186662	-6.772234777
	H300	5.203534124	-0.094468611	3.301932666
	H301	0.560904885	1.546906094	-8.624585311
	H302	-1.094227246	-2.159229919	-4.444220208
	H303	0.473548742	2.052740763	-6.936723914
	H304	4.245718048	-4.495374687	4.515068399
	H305	2.147872281	0.023478064	-6.571222681
	H306	3.904020425	-5.052259681	2.869597835
	H307	4.756879134	-7.299946087	3.295216496

	H308	6.386192564	-5.467121559	2.552573901
	H309	6.645507857	-5.063090453	4.256578787
	H310	-5.220972427	-0.804155253	-6.387998113
	H311	5.921359991	-9.437048655	4.895856419
	H312	4.983864302	-11.767821973	3.185394709
	H313	0.254264403	-0.056963662	-4.282170196
	H314	6.347959084	-11.741964552	5.771391018
	H315	4.794245704	-12.120690488	5.419994747
	H316	5.522931838	-3.322286715	2.071107472
	H317	-0.742039701	2.104775978	-4.901583143
	H318	-3.367697991	-2.084477142	-5.438312862
	H319	7.615364920	-9.942515820	-1.664031301
	H320	6.625355923	-7.671050866	-3.789471908
	H321	6.614744395	-8.188295348	-0.102876687
	H322	6.749669671	-10.546956482	-2.807802040
	H323	4.958296425	2.539050392	-9.484811310
	H324	6.363863538	-5.077338429	-0.239666049
	H325	2.282994732	-7.807614228	0.836228417
	H326	3.284514693	-2.026691772	-7.601531610
	H327	3.013277757	-9.228893913	-0.876002670
	H328	3.044822082	-6.863908679	-6.977059085
	H329	2.241089803	-5.416052184	-5.065951836
	H330	6.596605522	-12.407806765	3.533860039
	H331	5.809563762	-1.075748294	2.004953752
	H332	8.366839926	-5.805080511	0.783465079
	H333	7.231576431	-3.792250684	-0.178024213
	H334	8.971880404	-6.848451286	2.796225905
	H335	3.693020747	-2.696065622	5.155721005
	H336	3.733796236	-8.294012643	-3.702373244
	H337	4.947276220	-8.595250422	-2.453175033
	H338	1.884981873	-8.672060865	-2.120319946
	H339	1.348510868	-2.179454844	-6.135991647
	H340	-3.027879876	2.188157624	-5.933406589
	H341	-5.149931283	0.973318312	-6.539209478
	H342	2.304292319	-6.040673933	0.651286007
	H343	8.505832654	-7.342070944	0.582137859
	H344	7.406486533	-9.256569805	0.676879371
	H345	4.154815997	-1.036593372	5.133703892
	H346	5.502395614	-7.560840869	-6.696667574
	H347	6.537573831	-6.061699248	-3.092540371
	H348	0.458688993	-2.949814882	-7.482845218
	H349	4.228003167	4.230169322	-7.069521514
	H350	7.757251709	0.143673984	3.974789248
	H351	7.785036542	0.559066054	2.246275550
	H352	8.977708699	-6.702009614	-6.436111524
	H353	8.262051451	-7.998421109	-5.739291817
	H354	10.312521883	-8.589933201	-1.705826093
	H355	8.541289784	-8.223781293	-3.091855183
	H356	11.699858431	-7.547805192	-3.827351869
	H357	10.172510281	-6.881849763	-1.235874752
	H358	10.536647862	0.298214675	2.217094035
	H359	-2.969403569	13.181458112	3.723338818
	H360	10.191381096	-6.867375491	5.546756132
	H361	9.587538475	-4.436879856	4.380980372
	H362	-4.714186881	12.319655690	1.404199186
	H363	-4.080477943	10.293522305	3.638218754
	H364	-5.675096607	10.988925690	3.294716350
	H365	1.142805199	-3.239584402	-0.879623357
	H366	3.779091680	-1.170841006	0.051993272
	H367	-6.462306736	6.796763923	-1.567326113
	H368	0.127380383	-0.853502288	-0.534911793
	H369	-2.041687870	-0.254282081	-0.871655541
	Mo370	3.840839609	-4.400029164	-2.336337159
	N371	-7.086900481	-7.202828576	-0.358785579
	N372	-8.751482312	-4.608417347	1.761447414
	N373	-8.046955326	-4.897805124	4.465871556
	N374	-5.363253053	-5.759366093	4.482553897
	N375	-4.146018111	-3.357695096	3.562959013
	N376	-4.835042045	-1.710765335	5.826370389
	N377	-3.612456086	-3.011036130	8.083414711
	N378	-1.123040826	-4.535255231	7.548596997
	N379	0.175592900	-2.625784988	6.102715919
	N380	-0.390675878	0.316653553	6.764050116
	N381	-0.644919911	0.200579379	9.662018162
	N382	5.549832959	-11.472940828	5.191089811
	N383	6.380521660	-9.239135066	4.000424441
	N384	5.032243820	-3.297107441	2.968258446
	N385	4.292672374	-1.969298898	4.753538450
	N386	5.435389458	-1.023795188	2.961910636
	N387	9.033565696	-6.953634810	3.832647207

N388	-5.850119879	-2.943148576	-2.893621066
N389	-1.960649098	-5.617300983	-3.189258240
N390	-6.073457695	-0.445574950	-2.143223122
N391	-8.357766764	0.636837186	-0.932703617
N392	-7.856686009	0.353920764	1.816002996
N393	-5.684568269	2.075433985	2.279981218
N394	-3.123238038	2.675529964	-1.100429736
N395	-2.482534824	0.653859198	-0.721918829
N396	-6.320746990	4.721268305	2.854126628
N397	-9.786254667	7.710096164	1.582041092
N398	-9.673096941	9.498148984	2.890965417
N399	9.478331112	2.649750318	2.436937913
N400	6.276541202	7.186906755	-0.268732279
N401	4.215348142	8.462758733	-2.862109057
N402	1.557243600	7.843171906	-3.331827239
N403	-3.176441606	6.398178896	-4.458368646
N404	-3.341272462	8.265020894	-5.862052835
N405	-4.458050720	8.230747910	-3.836204898
N406	0.650928926	6.334923631	-1.054604656
N407	0.977801441	8.232910970	0.978253820
N408	-0.362355265	10.616145919	0.643633562
N409	-1.888894571	10.286415059	-3.025415238
N410	-2.753924304	11.615259227	1.694817631
N411	4.096949419	-4.736854742	-10.256954437
N412	2.464933791	-2.581944084	-7.863649255
N413	1.443136456	0.215840835	-7.303933055
N414	3.499766301	2.259231784	-7.341916481
N415	-1.725954384	-2.980347577	-10.046938613
N416	-3.296645489	-1.179845296	-8.466177693
N417	8.114897391	-7.013853025	-5.981009198
N418	4.248199443	-5.644849913	-4.303707618
N419	3.644595476	-6.558496962	-6.215321219
N420	9.219158803	-7.458279875	-3.052827261
N421	0.111150009	2.301734621	-0.157254132
N422	2.554621479	-0.747594073	0.363375920
N423	0.952813259	1.756906432	-0.676087399
N424	1.768144884	-1.521066061	0.737838900
O425	-6.543428220	-5.146560429	1.599652490
O426	-7.186699879	-2.786417919	4.461981133
O427	-5.209682778	-5.126122470	6.670422989
O428	-2.427501951	-3.904087771	4.958008915
O429	-3.039754525	-0.428664384	5.279428775
O430	-3.293329132	-0.913534348	8.950483365
O431	-0.492232137	-3.864376321	9.643487874
O432	2.185989081	-3.745547513	6.190129456
O433	1.884986240	0.184181972	6.599158328
O434	0.908284559	1.857116383	9.645048381
O435	1.579211306	-0.206610175	12.445049909
O436	7.060528264	-10.326355676	2.076534665
O437	7.992405878	-7.644106793	5.763254423
O438	11.612636213	-4.563637023	4.580770272
O439	-5.619111534	-0.852679659	0.069233094
O440	-3.685666370	-4.383937645	-4.012584813
O441	-7.149062359	2.494758689	-0.380793216
O442	-6.332582542	3.038814555	-3.324445526
O443	-9.799152440	1.476824949	2.299157234
O444	-5.660127110	1.528452142	4.497595386
O445	-4.265420410	5.029473547	3.799705958
O446	-6.603992655	7.245588066	5.323932464
O447	10.722173837	-0.143053942	4.203369865
O448	3.548096747	4.683469439	3.956763924
O449	3.605408637	8.386710677	-0.666472204
O450	0.987646529	10.063404999	-3.522095562
O451	-1.417783867	7.180256094	-1.533654804
O452	-0.708011585	7.213546136	2.157896839
O453	2.413295964	5.698141829	1.469814837
O454	0.863117219	11.629914545	2.293875003
O455	-2.504858236	13.588090774	0.580210865
O456	-3.274143255	10.978268730	-1.344466081
O457	-4.938798267	13.712235543	3.620325893
O458	-5.515401934	6.830027724	-1.329696197
O459	1.755271953	-4.567703402	-8.775395595
O460	-0.273288350	-0.736137296	-8.488918225
O461	2.093443990	3.601713346	-8.520368656
O462	5.928459596	4.291229335	-9.091202417
O463	-5.010066321	-2.644463345	-8.807847113
O464	-1.445345864	-6.606426574	-6.011061394
O465	0.467022535	-5.761436684	-6.790777657
O466	-3.994775082	2.320392112	-8.388566292
O467	9.938941766	-5.408510580	-3.697179073

	O468 12.645591443 -6.961433574 -2.115858520<br>O469 0.272482064 -6.302728500 -1.310545656<br>O470 -0.264193433 -7.427075403 0.560107089<br>O471 5.116470958 -10.907702196 -3.342215175<br>O472 2.855041547 -10.946792048 -3.356143633<br>O473 5.085345534 -5.880790827 -1.303027703<br>O474 4.997607881 -7.586768159 0.154232977<br>O475 2.599223046 -6.091649659 -2.047973140<br>O476 8.949574819 -6.566008252 1.026025198<br>O477 6.990373372 -4.579267647 0.346713408<br>O478 -4.373052353 4.741979708 -2.448621632<br>O479 7.701452884 -10.438565305 -2.508448367<br>O480 7.501485136 -8.637571458 -0.090801982<br>S481 3.586959106 4.983561556 -2.485344660<br>S482 2.105368815 1.943526338 -3.458182313<br>S483 2.021060312 -3.378227329 -3.483621483<br>S484 3.850692173 1.933373416 -0.348718451<br>S485 0.544576293 -0.829575791 -1.846927422<br>S486 4.013284852 -0.847324058 -5.521807652<br>S487 3.554974817 -3.547615520 -0.166459201<br>S488 5.675845868 1.681560735 -3.358103879<br>S489 5.667618039 -3.061949313 -3.045814015<br>S490 6.390017495 -0.803308390 -0.372549285<br>end

product

Fe(139) -2.427<br>Fe(140) 0.116<br>Fe(141) -2.519<br>Fe(142) 2.599<br>Fe(143) 2.478<br>Fe(144) 2.268<br>Fe(145) 0.151	bm526hx7hncapexp3tc.car_6<br>C1 -7.713882706 -5.459293634 0.766161893<br>C2 -8.003577379 -6.598797784 -0.215490193<br>C3 -8.684068743 -3.577015379 2.015770105<br>C4 -8.130539669 -3.842126079 3.412444814<br>C5 -7.806146706 -5.447845659 5.227653026<br>C6 -6.279008420 -5.546105238 5.267700805<br>C7 -4.215522404 -5.818457851 3.996479177<br>C8 -3.708782098 -4.376067631 4.087447583<br>C9 -4.078110155 -2.046870216 3.404855990<br>C10 -4.187256424 -1.447853445 4.814455689<br>C11 -5.366221229 -1.305617354 6.914040826<br>C12 -4.356204521 -1.859680854 7.927484898<br>C13 -3.386368170 -3.877062370 8.931933063<br>C14 -1.885025139 -4.108438675 8.700387670<br>C15 -0.125663039 -4.775698642 7.110496603<br>C16 0.627297656 -3.620561523 6.442613164<br>C17 0.336589861 -1.384622980 5.404015011<br>C18 0.393639133 -0.159266679 6.331578205<br>C19 -0.445055239 -1.177011997 4.087609356<br>C20 -0.013737718 0.105692448 3.367575806<br>C21 -0.296298101 -2.404057883 3.183065457<br>C22 -0.858540483 1.539585951 7.590489836<br>C23 -0.581411731 1.189434099 9.066953301<br>C24 -2.192672639 2.296383281 7.384285012<br>C25 -2.328380493 3.458267481 8.374016080<br>C26 -2.300710257 2.805254648 5.940453670<br>C27 -1.109397246 -0.180159032 11.000624303<br>C28 0.345946303 -0.420905646 11.350296797<br>C29 5.778620910 -11.564188835 4.016859735<br>C30 6.435610545 -10.280284833 3.523573605<br>C31 6.672959082 -7.863521085 4.057852228<br>C32 7.837566150 -7.465282637 4.983388539<br>C33 5.475357124 -6.913962968 4.234377614<br>C34 5.822955039 -5.469191190 3.891059988<br>C35 4.586925281 -4.579047592 3.806081751<br>C36 4.699141532 -2.074763430 3.797197328<br>C37 10.060334223 -6.439418253 5.089055298<br>C38 10.237255832 -4.938294074 5.075757886<br>C39 -5.173362541 -2.726787502 -1.914495785<br>C40 -5.588690776 -1.301567566 -1.503060507<br>C41 -3.640788558 -2.845213344 -1.764779207<br>C42 -3.068993047 -4.274544735 -1.846299553<br>C43 -2.707845581 -4.763556528 -3.248664682<br>C44 -6.043488654 0.933926301 -2.431727294<br>C45 -7.192210953 1.367671500 -1.508864852<br>C46 -6.165880254 1.545531593 -3.832743909<br>C47 -9.404668467 0.816348224 -0.606080668<br>C48 -9.100264635 0.835192523 0.895401534<br>C49 -7.508727920 0.310861620 2.682874640<br>C50 -6.355198263 1.273156149 2.970169151<br>C51 -4.781127243 2.948015227 2.134991299<br>C52 -5.267257362 4.262996216 2.796215672<br>C53 -4.111553456 3.366374265 0.816526343

	C54	-3.387319997	2.321825852	0.024632868
	C55	-2.854019327	1.083868062	0.293295155
	C56	-2.344843572	1.580825442	-1.810861149
	C57	-7.100947720	5.922357086	2.836892170
	C58	-7.078326235	6.081767203	4.355162763
	C59	-8.551030832	5.963679465	2.302453105
	C60	-9.176550804	7.320255296	2.152067501
	C61	-9.159960540	8.424625889	2.982250649
	C62	-10.381420028	8.834988155	1.180076693
	C63	8.998898892	1.735399457	3.996522805
	C64	10.046444390	0.646798266	3.858858630
	C65	7.622827515	1.074656335	3.729437597
	C66	6.468099928	2.037013367	3.872025883
	C67	5.811985420	2.213926456	5.104484511
	C68	6.022142639	2.794948836	2.775305334
	C69	4.739619663	3.104548400	5.237753368
	C70	4.967014273	3.698791709	2.897292018
	C71	4.321117211	3.848015065	4.128095058
	C72	5.352324542	6.944894909	-1.029631638
	C73	4.292762655	8.027322305	-1.298225638
	C74	4.688119044	5.595898488	-0.746498083
	C75	3.366600926	9.602620224	-2.959045381
	C76	1.909488846	9.207459017	-3.220761796
	C77	0.318868684	7.285273818	-3.281656483
	C78	-0.234656674	6.913087466	-1.882212783
	C79	0.429935010	6.052743911	-4.208794505
	C80	-0.793773095	5.153877450	-4.409039498
	C81	-1.898348419	5.680281100	-5.340545679
	C82	-3.472337094	7.572003798	-4.934392619
	C83	0.175575582	5.972383874	0.343242851
	C84	-0.065852901	7.177719334	1.263375502
	C85	1.148372706	5.017804445	1.063045900
	C86	0.931522123	9.251659530	2.094831936
	C87	0.294429932	10.591231023	1.756168405
	C88	-1.007859314	11.861575117	0.133670811
	C89	-2.326622132	12.398294624	0.740088602
	C90	-1.074070682	11.879776614	-1.395462758
	C91	-2.177061962	11.004745514	-1.978469184
	C92	-4.351457015	11.878509831	2.021646969
	C93	-4.353729633	12.977205136	3.075481562
	C94	-5.019377291	10.594047555	2.588723321
	C95	-5.184793790	9.524564991	1.533291746
	C96	-6.317335678	9.512519598	0.705585620
	C97	-4.193865109	8.555354171	1.311320504
	C98	-6.450586891	8.579302532	-0.324464316
	C99	-4.295619228	7.631643130	0.266522758
	C100	-5.425503843	7.658657557	-0.544525582
	C101	4.693550361	-3.877775893	-8.756542579
	C102	3.239605496	-3.649116393	-8.382321344
	C103	1.679342970	-2.129674852	-7.181451990
	C104	1.199263921	-0.781686415	-7.733960848
	C105	1.569868367	1.648958190	-7.662419204
	C106	2.743226460	2.619713594	-7.765093961
	C107	4.994209337	3.281954637	-7.129281006
	C108	5.617126204	3.505741451	-8.493520895
	C109	-2.131520875	-3.484317102	-9.323965455
	C110	-3.187809859	-2.408507540	-9.045147599
	C111	-1.716723275	-4.165054253	-8.000587251
	C112	-0.953101108	-5.478046320	-8.259364524
	C113	-0.355390352	-6.000420199	-6.971808069
	C114	-3.659198306	-0.096168722	-8.415837798
	C115	-3.152618000	1.198134230	-9.021544945
	C116	-4.149135271	0.023162184	-6.952971873
	C117	-2.969348389	0.078220566	-6.028160012
	C118	-2.293661807	1.287622462	-5.801416272
	C119	-2.415266844	-1.118892755	-5.542230175
	C120	-1.038670248	1.282438527	-5.182590336
	C121	-1.167581425	-1.119186788	-4.918160927
	C122	-0.457118486	0.078226305	-4.779764478
	C123	8.254289114	-6.121714976	-4.295659045
	C124	9.380000277	-6.200657742	-3.250210651
	C125	6.903682015	-6.477597173	-3.655198797
	C126	5.682299257	-6.259373408	-4.492200665
	C127	5.388992480	-6.823397370	-5.716919284
	C128	3.602885605	-5.752331956	-4.963712439
	C129	10.512550115	-7.420924027	-1.492206810
	C130	11.898731380	-7.154601816	-2.038647981
	C131	0.707032145	-6.896259449	-0.101661604
	C132	2.182955709	-6.911384789	0.280177566
	C133	3.153527227	-7.007072900	-0.931523060

	C134	3.108249690	-8.439611746	-1.536396111
	C135	4.222190943	-8.769154266	-2.534235967
	C136	4.286472773	-10.257652546	-2.945403512
	C137	4.590244038	-6.742587979	-0.457944026
	C138	3.743690098	-0.633627539	-2.016745296
	Fe139	4.009114179	-1.922530075	-3.479589231
	Fe140	2.191153082	0.654657941	-1.229398891
	Fe141	5.087019800	0.566189253	-1.159748737
	Fe142	3.936704712	0.607445122	-3.483141565
	Fe143	3.973089557	2.791065137	-1.941584921
	Fe144	4.981889627	-1.956127247	-1.140212767
	Fe145	2.261649080	-2.209022995	-1.168666918
	H146	-6.226294884	-7.589463208	-0.059023130
	H147	-6.308788645	-6.598585713	-1.356098808
	H148	-9.710273167	-4.949764718	0.742627489
	H149	-8.648411640	-6.212050337	-1.021269104
	H150	-8.614400789	-7.342600771	0.325152159
	H151	-8.019598089	-2.834529129	1.550508026
	H152	-8.855210182	-5.757408163	3.373641420
	H153	-9.676852782	-3.118829566	2.122471408
	H154	-8.233488085	-6.416667686	5.518402787
	H155	-6.208733898	-5.938686492	3.247922058
	H156	-8.094873060	-4.708712862	5.986607155
	H157	-3.056488139	-1.852870574	3.056652086
	H158	-5.129108150	-3.783115043	2.718596067
	H159	-3.911634212	-6.260310127	3.037829114
	H160	-5.224700302	-0.217152326	6.885616074
	H161	-5.982986060	-2.407851103	5.160766268
	H162	-3.736412699	-6.383005571	4.804914168
	H163	-4.771359855	-1.524539214	2.731539705
	H164	-4.653251262	-3.769195538	7.209917383
	H165	-6.380307325	-1.521364666	7.277454697
	H166	-0.646499292	-3.320786573	3.674713931
	H167	-0.891661767	-2.276877355	2.265501932
	H168	0.746782135	-2.538402546	2.861496493
	H169	-1.503499376	-1.060740351	4.361232808
	H170	-3.852517576	-4.858844401	9.103710059
	H171	-2.267881266	1.986987332	5.208138418
	H172	-2.041218679	-0.301240264	9.083630350
	H173	-2.318609696	3.121963903	9.420526207
	H174	0.989466784	0.004577201	2.925264163
	H175	-0.009576661	0.980210039	4.036143491
	H176	9.008988100	2.074006358	5.050827312
	H177	-3.250181060	3.338907043	5.791489528
	H178	-3.275869905	3.987948155	8.198808196
	H179	-1.479082343	3.506565147	5.719110570
	H180	-1.505586434	4.178514397	8.241729682
	H181	-3.017031964	1.583542946	7.568983050
	H182	-0.024773764	2.199610127	7.306096538
	H183	-1.669353118	0.000857202	6.328775216
	H184	-0.715650104	0.326728604	2.546925371
	H185	1.491816984	4.256292627	0.347458144
	H186	0.573656668	4.516137271	1.854484141
	H187	2.458301586	5.346264345	2.499687968
	H188	2.840442178	4.686061597	5.060802391
	H189	4.617417061	4.266533180	2.035196549
	H190	6.488353311	2.651187568	1.797797340
	H191	6.132789996	1.635573039	5.976135814
	H192	-1.141604948	-2.643752113	6.285333594
	H193	-0.079234908	-5.636651665	6.427096159
	H194	0.417178646	-5.042121783	8.026610707
	H195	-2.206468141	-4.571043598	6.694963106
	H196	-3.462510843	-3.289266490	9.854150597
	H197	4.229686954	3.215611652	6.198861260
	H198	1.384043148	-1.590370311	5.156077209
	H199	-1.521769523	0.582244829	11.680136963
	H200	-1.642724002	-1.126145686	11.181243223
	H201	0.979652451	-0.776919385	10.505167536
	H202	10.980099702	-6.918365368	4.718699322
	H203	-1.412355483	-6.007630116	-4.233404199
	H204	1.381182419	-6.004158523	-1.667437984
	H205	-1.652003739	-6.233498125	-8.646050623
	H206	-0.164611353	-5.318459017	-9.008813386
	H207	-2.606346064	-4.384880636	-7.389765121
	H208	-2.443847789	1.062499065	-9.872205945
	H209	-2.674024134	-4.229770429	-9.925364492
	H210	-3.161328866	-2.208963771	-2.526839010
	H211	-3.790305511	-4.996914274	-1.426367810
	H212	-1.721789197	-1.011932713	-8.575808802
	H213	-4.553707768	-0.338579746	-9.027426304

	H214	-0.404694753	-2.369053825	-9.630280146
	H215	-1.103921235	-5.869973522	-2.505723793
	H216	-1.100642307	-3.463959895	-7.415780712
	H217	-0.498241221	-3.740068001	-10.539561584
	H218	-6.486643369	-3.539781459	-3.288230002
	H219	-3.376208429	-2.430362571	-0.781044567
	H220	1.361670771	-5.228539200	-7.530352239
	H221	-2.177087491	-4.339793700	-1.205768421
	H222	-4.049292324	2.516178034	2.831170509
	H223	-2.883796602	0.484737414	1.195469295
	H224	-8.361781335	0.649025879	3.288078260
	H225	-7.310687786	-0.184312720	0.615281923
	H226	-7.115895291	3.968704010	1.957128428
	H227	-8.669977180	8.615968665	3.930421772
	H228	-7.244218488	5.126759642	4.911682027
	H229	-9.182366102	5.307027586	2.927256168
	H230	-6.504169379	6.732724069	2.389017086
	H231	-11.014703722	9.380989599	0.486077344
	H232	-3.394154531	4.165468810	1.067991853
	H233	-4.875604468	3.823756943	0.167060332
	H234	-10.120181560	10.312764191	2.690496240
	H235	-8.543251770	5.516195060	1.296582264
	H236	-6.273505943	1.909486990	0.987519460
	H237	-7.223386988	-0.681073228	3.063436992
	H238	-9.850192229	1.793546547	-0.839124324
	H239	-1.932784988	1.509494978	-2.813907544
	H240	-10.167128813	0.048712768	-0.792235851
	H241	10.082218837	3.338496434	3.326366800
	H242	8.486591249	3.439019526	2.984295080
	H243	1.562621446	6.020248641	-1.306170741
	H244	-0.793216340	5.460389797	0.247580827
	H245	1.592872781	8.081962902	0.463990074
	H246	-3.387330965	3.441302325	-1.823212161
	H247	-4.976361209	4.110272563	-3.091539238
	H248	-6.483824330	3.335049119	-4.574471125
	H249	-4.733482612	5.344944542	-2.119547446
	H250	-5.318162465	1.190357104	-4.441249842
	H251	-7.104951980	1.218644114	-4.305086114
	H252	-0.089994292	11.627950183	-1.815156296
	H253	-1.302436435	12.915445660	-1.692077705
	H254	-0.846252787	10.197490385	-3.347268543
	H255	-2.518969999	9.640248887	-3.423325252
	H256	-3.435493319	9.171006083	-6.223332331
	H257	-2.354615401	4.843257842	-5.891770186
	H258	-7.345139973	8.574100577	-0.952714820
	H259	-3.315744601	8.499154761	1.960794203
	H260	-4.557451138	7.731263443	-3.191972213
	H261	1.238238928	5.416789771	-3.813984490
	H262	2.437576616	7.250624056	-3.046738052
	H263	-1.231382160	4.851910250	-3.444347083
	H264	-8.240399745	-0.310496736	-2.015721468
	H265	3.756562388	10.073147038	-3.870906016
	H266	-5.858596022	-1.010293621	-3.458521625
	H267	0.778508055	6.414895279	-5.189299634
	H268	-3.482796879	6.930597592	0.071982992
	H269	3.340781859	10.366560840	-2.168939517
	H270	-2.662087258	7.739870679	-6.825443524
	H271	-4.951790838	8.883864443	-4.417528358
	H272	-4.888506667	-3.561153490	-3.775512364
	H273	-5.635665372	-3.394995034	-1.164037053
	H274	-7.117920414	10.238226003	0.869318969
	H275	-5.142553145	1.340387731	-1.950827829
	H276	-0.367660848	8.030332501	-3.691698296
	H277	-1.452644755	6.349022592	-6.088305166
	H278	-0.412950028	4.231696402	-4.868658793
	H279	-3.420718724	5.844101457	-3.859742526
	H280	0.480718863	8.899401155	3.034727619
	H281	-0.580355473	9.749594381	0.110330494
	H282	-0.264718973	12.615308142	0.437328602
	H283	1.997505160	9.441615454	2.278787989
	H284	-2.683408018	10.604740569	1.612824065
	H285	5.198254697	-2.911796487	-8.902910079
	H286	5.159765051	-4.351371448	-7.867200983
	H287	8.236247588	-5.068276429	-4.611945827
	H288	5.747677431	-5.004944380	-10.087341039
	H289	4.201614541	-5.503827701	-9.879214024
	H290	5.488711504	4.867006847	-0.564054279
	H291	5.588896251	7.530632027	0.910229092
	H292	4.928769992	8.176564586	-3.249191122
	H293	4.051182623	5.670959189	0.146206734

H294	6.730548121	8.142898066	-0.088652978
H295	5.783529172	2.897117719	-6.465118011
H296	6.004030134	6.844860424	-1.912120990
H297	5.105646369	-6.990490345	5.270206184
H298	7.000015287	-7.836130215	3.010626357
H299	4.031645732	1.447264509	-6.612913981
H300	4.896561153	-0.046421123	3.519954402
H301	1.086731977	1.610426515	-8.646396204
H302	-0.734733339	-2.047415056	-4.538834644
H303	0.842631190	2.114183830	-6.974735410
H304	4.127564209	-4.459088174	4.793976130
H305	2.494608316	0.113825766	-6.448268473
H306	3.830506116	-5.028682215	3.142904545
H307	4.676001609	-7.273024013	3.566089410
H308	6.333481267	-5.440694322	2.917197738
H309	6.517511286	-5.041506624	4.631798403
H310	-4.782159058	-0.849695879	-6.737344759
H311	5.740692736	-9.437928831	5.200151774
H312	4.903091341	-11.724201277	3.369581236
H313	0.524748471	0.067049161	-4.308827676
H314	6.086001919	-11.794222210	6.040419452
H315	4.554313093	-12.130520215	5.571588213
H316	5.489980531	-3.296918064	2.390864195
H317	-0.497680757	2.215330576	-5.017382312
H318	-2.954763777	-2.059453162	-5.676414662
H319	7.859437916	-9.830065512	-1.231181427
H320	6.918199218	-7.542909832	-3.365185761
H321	6.732500942	-8.087611171	0.268429373
H322	7.061976710	-10.431943436	-2.420617722
H323	5.442557785	2.685439181	-9.230191358
H324	6.442961068	-4.955832268	0.144618686
H325	2.361992586	-7.726809569	0.991560073
H326	3.772022712	-1.918256713	-7.436117912
H327	3.171618599	-9.138006255	-0.687146853
H328	3.537283628	-6.799265570	-6.793105876
H329	2.582963266	-5.394803730	-4.913705657
H330	6.483899466	-12.386022676	3.804366521
H331	5.622944670	-1.013122067	2.261035025
H332	8.375655596	-5.697126357	1.293012424
H333	7.308127588	-3.677849456	0.304774845
H334	8.883961998	-6.761764957	3.319735097
H335	3.413196587	-2.686339952	5.325383446
H336	4.074468121	-8.182887348	-3.453486465
H337	5.199912267	-8.483303740	-2.123460558
H338	2.123618318	-8.593566205	-2.004436302
H339	1.735090877	-2.095243242	-6.081357183
H340	-2.733856654	2.223271671	-6.155234707
H341	-4.767955529	0.931259082	-6.890163275
H342	2.361549961	-5.957465980	0.804606172
H343	8.554174244	-7.227599600	1.078518986
H344	7.470363003	-9.168126379	1.088249156
H345	3.797541383	-1.009885082	5.292092026
H346	5.987487235	-7.440027831	-6.375254358
H347	6.794257488	-5.916561280	-2.720073842
H348	0.961169712	-2.894425148	-7.484620789
H349	4.659509375	4.249059086	-6.723188438
H350	7.509138155	0.242006569	4.438599163
H351	7.633414994	0.648761536	2.713934389
H352	9.420353517	-6.637202146	-5.900684298
H353	8.659887854	-7.917284255	-5.224499717
H354	10.509974199	-8.444690138	-1.087581476
H355	8.839632975	-8.128948740	-2.608245264
H356	12.029930746	-7.387679945	-3.123181573
H357	10.312032618	-6.734515876	-0.654265846
H358	10.377874694	0.459496122	2.809088317
H359	-3.376163670	13.151026009	3.582498264
H360	9.922828324	-6.738828791	6.143909762
H361	9.296669810	-4.353164574	4.923281099
H362	-4.947587824	12.241866493	1.167488898
H363	-4.414856602	10.218278584	3.429216010
H364	-5.996792773	10.890105652	2.995155584
H365	1.262082887	-3.274328271	-0.894977680
H366	3.210186394	-0.822223657	0.692778186
H367	-6.417515665	6.732964976	-1.943438994
H368	0.525738983	-1.391947454	0.307070202
H369	-1.550151909	-0.146967437	-0.968035805
Mo370	4.017000448	-4.296747648	-2.125158425
N371	-6.852205632	-7.263023875	-0.799728595
N372	-8.811485599	-4.760253226	1.179866434
N373	-8.344448921	-5.075361085	3.929419283

	N374	-5.664338042	-5.896029368	4.110118481
	N375	-4.381979968	-3.468118760	3.335456951
	N376	-5.225590719	-1.860277829	5.578022345
	N377	-4.130177541	-3.198521441	7.879585831
	N378	-1.513320803	-4.500968117	7.447148467
	N379	-0.140134793	-2.552781985	6.126479365
	N380	-0.799521346	0.356706119	6.732416119
	N381	-1.289476090	0.162353522	9.600254062
	N382	5.335153767	-11.491207819	5.415974590
	N383	6.256367652	-9.224750065	4.339965912
	N384	4.961978822	-3.272002041	3.267597497
	N385	4.009397622	-1.942993479	4.949991048
	N386	5.153666947	-0.969206030	3.178631342
	N387	8.891660476	-6.883841428	4.356494807
	N388	-5.614549915	-3.007565678	-3.286220262
	N389	-1.679353756	-5.630588591	-3.318217053
	N390	-5.916298593	-0.506931229	-2.557596781
	N391	-8.270739139	0.551161852	-1.475130608
	N392	-7.948444181	0.231110257	1.302683519
	N393	-5.850674088	1.985029600	1.917058462
	N394	-3.056130626	2.595881925	-1.298176704
	N395	-2.224057239	0.648049420	-0.860289754
	N396	-6.538135116	4.622941782	2.479075771
	N397	-9.942231134	7.591335890	1.028098659
	N398	-9.928295345	9.375943205	2.346209100
	N399	9.276697695	2.788551779	3.017157656
	N400	6.198121313	7.294055893	0.120422589
	N401	4.257722538	8.524602060	-2.569647606
	N402	1.646757626	7.883564953	-3.202114057
	N403	-2.964902263	6.381611533	-4.620426394
	N404	-3.143033363	8.211851097	-6.069255828
	N405	-4.284349964	8.207056842	-4.055853713
	N406	0.637122818	6.352492244	-0.994058977
	N407	0.788925355	8.223769882	1.081866326
	N408	-0.544529497	10.597635209	0.675450643
	N409	-1.837168621	10.328312992	-3.109085897
	N410	-2.994914853	11.572490572	1.583998840
	N411	4.787032537	-4.670133219	-9.985296011
	N412	2.980822993	-2.497271136	-7.726954844
	N413	1.864277783	0.289643114	-7.248504558
	N414	3.914338265	2.308057734	-7.154033810
	N415	-1.047686641	-2.963366988	-10.166553063
	N416	-2.727771947	-1.177786334	-8.676730794
	N417	8.530237633	-6.936536099	-5.492174393
	N418	4.550550655	-5.580722866	-4.041224484
	N419	4.080803459	-6.489970970	-5.991599895
	N420	9.494399076	-7.346208826	-2.524719683
	N421	0.071688794	2.266907268	0.011093752
	N422	2.309394483	-0.735355394	0.203754714
	N423	0.889306468	1.663433172	-0.471243066
	N424	1.467298937	-1.657946806	0.658822721
	O425	-6.576749260	-5.203002992	1.201048849
	O426	-7.521558588	-2.951179762	4.029444616
	O427	-5.663519988	-5.327230761	6.324041134
	O428	-2.761135266	-4.059347402	4.823105855
	O429	-3.354801558	-0.623512750	5.222745862
	O430	-3.828986172	-1.110734326	8.768534849
	O431	-1.088194164	-3.984826811	9.639406429
	O432	1.848226334	-3.713915974	6.213190623
	O433	1.488234475	0.303211928	6.689414680
	O434	0.250641301	1.823925541	9.737957155
	O435	0.788725999	-0.309780395	12.483988769
	O436	7.051138603	-10.270678613	2.437456957
	O437	7.773057097	-7.659968421	6.209615061
	O438	11.312082474	-4.381664538	5.236272644
	O439	-5.605121776	-0.906728377	-0.321175880
	O440	-3.344936309	-4.405104164	-4.265185511
	O441	-7.104284653	2.405840447	-0.829562627
	O442	-6.148841086	2.983720602	-3.730171316
	O443	-9.906459076	1.373975772	1.665116735
	O444	-5.918752136	1.383986643	4.120646383
	O445	-4.510276114	4.962376032	3.474591543
	O446	-6.941734385	7.145832684	4.934162890
	O447	10.488700717	-0.002357381	4.795675719
	O448	3.260870068	4.731639621	4.182314377
	O449	3.522987395	8.436350565	-0.412441504
	O450	1.064421599	10.096966491	-3.437353257
	O451	-1.426425790	7.122427244	-1.609129824
	O452	-0.939751348	7.143130665	2.138639470
	O453	2.268026213	5.717070209	1.613752687

	O454	0.522428947	11.594829133	2.441593220
	O455	-2.700960765	13.553753303	0.494717056
	O456	-3.313255874	10.965203878	-1.484963126
	O457	-5.357241542	13.604168298	3.375182198
	O458	-5.491999745	6.764036357	-1.632671544
	O459	2.367003027	-4.510660939	-8.646168495
	O460	0.285632509	-0.705385603	-8.580264378
	O461	2.581736328	3.695481590	-8.364776347
	O462	6.294894230	4.482446280	-8.765780172
	O463	-4.402185789	-2.656978032	-9.128195743
	O464	-1.000365732	-6.608303670	-6.124353084
	O465	0.945867081	-5.716188679	-6.754119708
	O466	-3.510631580	2.312938720	-8.669711868
	O467	10.190676082	-5.266442013	-3.096064810
	O468	12.838263311	-6.783206065	-1.349591183
	O469	0.422589544	-6.262939310	-1.247984394
	O470	-0.171800642	-7.375495323	0.611497786
	O471	5.449502713	-10.789552182	-3.024376438
	O472	3.195848968	-10.836979166	-3.210822892
	O473	5.231364303	-5.772896939	-1.061540301
	O474	5.101598323	-7.444877117	0.433071727
	O475	2.768098041	-6.002911359	-1.872899953
	O476	8.956511399	-6.450838412	1.557419267
	O477	7.012102855	-4.469344198	0.793531614
	O478	-4.271330478	4.696239544	-2.705557542
	O479	7.996956534	-10.320887777	-2.072556674
	O480	7.615045281	-8.537106027	0.336919333
	S481	3.632655477	5.008210307	-2.150918681
	S482	2.223218265	2.029600250	-3.124139619
	S483	2.195671352	-3.251626378	-3.199673127
	S484	3.774874077	1.897954508	0.057595558
	S485	0.603204264	-0.856963773	-1.968129114
	S486	4.303135774	-0.646357666	-5.271980767
	S487	3.740921410	-3.427002094	0.028788419
	S488	5.755713091	1.799304134	-2.903519350
	S489	5.868496318	-3.016536795	-2.878833168
	S490	6.387553268	-0.751928513	0.031449778
	end			

47, S=1/2

reactant

Fe(139) 2.233<br>Fe(140) 0.089<br>Fe(141) 1.361<br>Fe(142) -2.277<br>Fe(143) 1.807<br>Fe(144) -1.192<br>Fe(145) -1.412	bm52capexp147ti.car_3			
	C1	-7.739425235	-5.368194268	1.281024482
	C2	-8.132637476	-6.512104125	0.338238181
	C3	-8.550761800	-3.438782619	2.566938530
	C4	-7.914968078	-3.698868285	3.928962467
	C5	-7.482605050	-5.290852149	5.735313756
	C6	-5.957920253	-5.412051029	5.667325890
	C7	-3.985052282	-5.717227511	4.271359733
	C8	-3.471170524	-4.276447830	4.307930206
	C9	-3.889006620	-1.955076480	3.614317506
	C10	-3.904226358	-1.326590476	5.016873795
	C11	-4.925140083	-1.164638967	7.195897787
	C12	-3.861078525	-1.717068458	8.155842020
	C13	-2.857448432	-3.732668235	9.132904211
	C14	-1.389475731	-4.047783782	8.810784332
	C15	0.242336234	-4.792913264	7.123308426
	C16	0.982541074	-3.645443679	6.426209142
	C17	0.648359566	-1.404903850	5.395745919
	C18	0.778350104	-0.190544956	6.329749604
	C19	-0.263250204	-1.194336234	4.171759141
	C20	-0.004853482	0.149398838	3.474611452
	C21	-0.151594429	-2.412104579	3.241736413
	C22	-0.360823916	1.557388438	7.621780245
	C23	0.004110443	1.230845921	9.081610055
	C24	-1.689834346	2.337839348	7.484415231
	C25	-1.756555733	3.504848617	8.475111021
	C26	-1.867885962	2.847986613	6.047496681
	C27	-0.407850738	-0.088439106	11.072441858
	C28	1.057537587	-0.358314657	11.352727712
	C29	5.894416955	-11.603444418	3.747236363
	C30	6.515182338	-10.326043229	3.192259956
	C31	6.789472061	-7.900002657	3.682221054
	C32	8.011530165	-7.505890150	4.531834241
	C33	5.611959219	-6.941215615	3.923053594
	C34	5.947759444	-5.499707535	3.549860759

C35	4.704115652	-4.616141161	3.496358293
C36	4.888844303	-2.113946838	3.496903478
C37	10.269675610	-6.555929445	4.482076761
C38	10.490238010	-5.060414925	4.469029752
C39	-5.336953833	-2.677315990	-1.563527194
C40	-5.695883347	-1.241924294	-1.130683689
C41	-3.798748308	-2.823179524	-1.503702591
C42	-3.245207178	-4.258392067	-1.621367779
C43	-2.977352366	-4.745792252	-3.046226085
C44	-6.182290751	0.998020548	-2.036635791
C45	-7.271476231	1.453824210	-1.054969919
C46	-6.359081735	1.607365321	-3.433047109
C47	-9.425757430	0.933106724	-0.008133270
C48	-9.028523943	0.951992560	1.471056796
C49	-7.324563153	0.438200260	3.154168393
C50	-6.155212767	1.401547341	3.365910083
C51	-4.606505737	3.047353975	2.432896235
C52	-5.047527927	4.357069235	3.129373899
C53	-4.019266313	3.481308722	1.081000293
C54	-3.400285282	2.420136958	0.234686380
C55	-2.973710932	1.138193680	0.470737559
C56	-2.541252333	1.609601275	-1.664907560
C57	-6.852381998	6.036258336	3.243486839
C58	-6.756193948	6.191609316	4.759820967
C59	-8.325166763	6.090724460	2.776464278
C60	-8.946406078	7.451736950	2.660126708
C61	-8.873226440	8.555107147	3.487983187
C62	-10.197202434	8.972626250	1.760845032
C63	9.236601349	1.631131656	3.427186942
C64	10.272050532	0.533236860	3.260688143
C65	7.844087750	0.982379849	3.245527833
C66	6.709341130	1.957135585	3.459384005
C67	6.137881047	2.153586190	4.729238414
C68	6.196099949	2.706465281	2.385059752
C69	5.086942757	3.058391899	4.923078587
C70	5.162893040	3.625894090	2.565902308
C71	4.600503378	3.792976437	3.833875288
C72	5.381972611	6.856949909	-1.393101399
C73	4.314485577	7.944362881	-1.606624191
C74	4.734654556	5.513064781	-1.076065329
C75	3.310708067	9.537117319	-3.206901091
C76	1.834558288	9.165419334	-3.368112212
C77	0.212592700	7.270385081	-3.331472795
C78	-0.252141610	6.928724093	-1.892138393
C79	0.244756914	6.024269195	-4.244786200
C80	-1.008863357	5.147497826	-4.350538732
C81	-2.153567786	5.684339671	-5.226807736
C82	-3.675899163	7.604737465	-4.745493953
C83	0.270433147	5.982487420	0.304999434
C84	0.117636228	7.201518217	1.226214315
C85	1.268496621	5.012878092	0.970300643
C86	1.197799537	9.267257335	1.968456129
C87	0.553910295	10.610367084	1.659383962
C88	-0.856105858	11.876325275	0.129193664
C89	-2.130468949	12.430895449	0.811031617
C90	-1.014819719	11.885827025	-1.393528985
C91	-2.159907351	11.017794066	-1.899258710
C92	-4.074603972	11.950681752	2.223465908
C93	-3.988797023	13.051716074	3.271909596
C94	-4.732275305	10.682955388	2.836800910
C95	-4.972558676	9.609495148	1.800095648
C96	-6.153485879	9.606709030	1.042262702
C97	-4.007079408	8.627750977	1.524212666
C98	-6.358313727	8.670409317	0.027571310
C99	-4.182675962	7.698282287	0.494304050
C100	-5.358874563	7.736963317	-0.248679772
C101	4.086611759	-3.980557345	-8.997332818
C102	2.659406365	-3.727326345	-8.543481488
C103	1.201349157	-2.191944630	-7.244334542
C104	0.703272659	-0.835435483	-7.756768549
C105	1.121760712	1.586790135	-7.711241252
C106	2.299297111	2.537519719	-7.916249066
C107	4.577838878	3.209131473	-7.404415452
C108	5.163341904	3.373954540	-8.792686905
C109	-2.763126474	-3.493956929	-9.154175460
C110	-3.789228633	-2.403394175	-8.822600843
C111	-2.272188381	-4.169009924	-7.852727435
C112	-1.538889446	-5.489645367	-8.150964833
C113	-0.860945679	-6.018839981	-6.907216688
C114	-4.193880365	-0.085277545	-8.162585588

C115	-3.714906642	1.199205822	-8.809504982
C116	-4.567375248	0.046160157	-6.664508999
C117	-3.330748040	0.061095338	-5.811018831
C118	-2.605163418	1.249255482	-5.615654792
C119	-2.793623314	-1.149178984	-5.342003421
C120	-1.327916888	1.205415020	-5.047318777
C121	-1.518202889	-1.191550119	-4.772375470
C122	-0.768592401	-0.016488591	-4.662858886
C123	7.918187188	-6.238805539	-4.774079557
C124	9.098852064	-6.322268325	-3.795954319
C125	6.605790181	-6.611690088	-4.071653743
C126	5.357315298	-6.417095518	-4.867078896
C127	5.006779030	-7.006988307	-6.065048479
C128	3.269172911	-5.899139006	-5.257322918
C129	10.306737696	-7.545914772	-2.092211917
C130	11.659072386	-7.322483611	-2.733166070
C131	0.608027991	-6.922915541	-0.201199897
C132	2.097132789	-6.967043122	0.122507958
C133	3.017038028	-7.093540726	-1.130986264
C134	2.927125864	-8.536686612	-1.695917090
C135	3.958707015	-8.878613756	-2.773086497
C136	3.984247778	-10.367996863	-3.180670080
C137	4.467584427	-6.817742209	-0.701774000
C138	3.581548030	-0.696597064	-2.228867725
Fe139	3.887752272	-1.968003230	-3.737601381
Fe140	2.268732139	0.717254770	-1.400209119
Fe141	5.007388543	0.461140511	-1.604196757
Fe142	3.743538709	0.553393414	-3.811899209
Fe143	3.871848727	2.734197747	-2.482086300
Fe144	4.665196463	-2.050500428	-1.277795155
Fe145	2.178142840	-2.406219741	-1.805842210
H146	-6.363300863	-7.529223379	0.338133743
H147	-6.539689257	-6.533072187	-0.942644926
H148	-9.711920075	-4.787504868	1.388023618
H149	-8.847318533	-6.123486785	-0.405201183
H150	-8.692680236	-7.245402722	0.943996308
H151	-7.888854973	-2.732117818	2.044812108
H152	-8.662940360	-5.604392996	3.963515115
H153	-9.514912154	-2.938682844	2.728331460
H154	-7.901449157	-6.250347210	6.065173993
H155	-6.025369350	-5.841094529	3.650840297
H156	-7.701373805	-4.539264581	6.504833462
H157	-2.892838619	-1.768483075	3.192342997
H158	-4.988839580	-3.701498941	3.042201107
H159	-3.744587897	-6.176710200	3.303261645
H160	-4.775034219	-0.078283701	7.145794146
H161	-5.661640213	-2.291573476	5.507121065
H162	-3.463559099	-6.271935569	5.060008313
H163	-4.627099353	-1.447048078	2.977522796
H164	-4.216020249	-3.631486062	7.481792981
H165	-5.916270707	-1.364141710	7.626856204
H166	-0.567941522	-3.307372099	3.720166528
H167	-0.699681283	-2.254885251	2.298930594
H168	0.893179500	-2.623355186	2.977944765
H169	-1.289801507	-1.154219827	4.556055512
H170	-3.354062363	-4.681934109	9.383485534
H171	-1.871408057	2.031474300	5.313381182
H172	-1.450324407	-0.255259134	9.214635529
H173	-1.714616631	3.170448575	9.521455375
H174	0.811798687	0.088484398	2.761658388
H175	0.240136769	0.954819964	4.180699632
H176	9.308161091	1.990367021	4.471928662
H177	-2.823280316	3.383146687	5.948276922
H178	-2.697215476	4.056521724	8.332107668
H179	-1.057265655	3.548801154	5.787491840
H180	-0.922528421	4.205144472	8.308982293
H181	-2.514029288	1.638111839	7.713977699
H182	0.466222032	2.194123712	7.272517785
H183	-1.277997615	0.043834843	6.404781636
H184	-0.901416349	0.474505812	2.926292105
H185	1.557578733	4.249657118	0.233991528
H186	0.735514617	4.517233381	1.794722804
H187	2.666378846	5.326104738	2.324455455
H188	3.215019156	4.682297478	4.863892286
H189	4.768755172	4.191045618	1.721164617
H190	6.597291002	2.550895245	1.380858319
H191	6.510886094	1.584415516	5.585421457
H192	-0.786205674	-2.664031146	6.327647880
H193	0.224850825	-5.644446621	6.427924471
H194	0.833726353	-5.085801049	7.999997404

H195	-1.853814604	-4.527997635	6.838861947
H196	-2.841896852	-3.103382200	10.030752685
H197	4.641776502	3.184650442	5.913989028
H198	1.671436592	-1.613602143	5.062131483
H199	-0.750912628	0.719072313	11.739685086
H200	-0.961303897	-1.004452683	11.331356504
H201	1.648867497	-0.727528893	10.482808110
H202	11.144645370	-7.053469350	4.036330848
H203	-1.752437510	-5.981681208	-4.124058158
H204	1.270631445	-6.069744289	-1.808498642
H205	-2.268911051	-6.241248100	-8.485183326
H206	-0.802415246	-5.342446883	-8.953931004
H207	-3.122934325	-4.374233659	-7.184385691
H208	-3.105239590	1.047041218	-9.731276338
H209	-3.351377324	-4.238461862	-9.712221510
H210	-3.358828833	-2.193416639	-2.295722155
H211	-3.946560862	-4.975107821	-1.160678066
H212	-2.281635470	-1.027454785	-8.429413638
H213	-5.133160175	-0.320751171	-8.704496121
H214	-1.044033401	-2.403290372	-9.566542688
H215	-1.312461859	-5.844118108	-2.425393201
H216	-1.614849917	-3.466480844	-7.316827507
H217	-1.208476829	-3.773224202	-10.465657061
H218	-6.743574323	-3.461639500	-2.858119593
H219	-3.474140783	-2.416554850	-0.534105865
H220	0.831861585	-5.292207186	-7.588619758
H221	-2.317082541	-4.334399235	-1.036715074
H222	-3.834181969	2.606764225	3.079157217
H223	-3.007328014	0.527344961	1.363606833
H224	-8.139552130	0.777458177	3.808232186
H225	-7.263001333	-0.074254544	1.081980557
H226	-6.931068329	4.083604767	2.365475406
H227	-8.328094916	8.743993836	4.405349245
H228	-6.908571441	5.236370392	5.319332556
H229	-8.932437094	5.435022622	3.425668233
H230	-6.268766854	6.839911146	2.767562474
H231	-10.871928780	9.521404023	1.109623726
H232	-3.257784344	4.248759611	1.293307594
H233	-4.811380093	3.971815226	0.492804194
H234	-9.835524784	10.450244515	3.251450432
H235	-8.363445949	5.647256123	1.768845976
H236	-6.174827235	2.016823530	1.374670124
H237	-7.015980791	-0.552419840	3.520194391
H238	-9.868327029	1.917362871	-0.216402794
H239	-2.198309843	1.510308072	-2.692496899
H240	-10.211070008	0.178621788	-0.145861761
H241	10.296737256	3.214719987	2.683188191
H242	8.689402123	3.319871788	2.408549599
H243	1.549888077	5.979966835	-1.431814960
H244	-0.714824821	5.494924998	0.277700749
H245	1.734256260	8.073303271	0.307810490
H246	-3.437296083	3.543014039	-1.610990990
H247	-5.115937447	4.160789773	-2.787753556
H248	-6.709485063	3.392549761	-4.167390197
H249	-4.799949458	5.410692119	-1.851696033
H250	-5.532095762	1.252606910	-4.069961180
H251	-7.313341220	1.275236865	-3.870000445
H252	-0.061240184	11.614788270	-1.867686719
H253	-1.247845741	12.922051860	-1.684655572
H254	-0.912467805	10.178920040	-3.324241534
H255	-2.587064677	9.626405431	-3.294100029
H256	-3.689772818	9.202432083	-6.033636087
H257	-2.648921608	4.851388254	-5.748512272
H258	-7.289253925	8.671669890	-0.545389438
H259	-3.092766478	8.565373237	2.119721403
H260	-4.697411490	7.770933741	-2.963278656
H261	1.066870595	5.380540204	-3.891395804
H262	2.343125068	7.199861550	-3.246299072
H263	-1.393260958	4.884642157	-3.352592009
H264	-8.366462918	-0.217071199	-1.480857192
H265	3.645431187	9.998637955	-4.145479046
H266	-6.095587816	-0.955180719	-3.062715147
H267	0.535900562	6.363343396	-5.252503419
H268	-3.391617207	6.985760174	0.256840943
H269	3.347355205	10.305679230	-2.421159078
H270	-2.943227367	7.772274698	-6.667344483
H271	-5.106422232	8.947809281	-4.166030933
H272	-5.175477905	-3.519048266	-3.436172874
H273	-5.768321823	-3.337032909	-0.787749742
H274	-6.933742209	10.342989577	1.248592272

	H275	-5.253349533	1.390226066	-1.598638454
	H276	-0.489000142	8.022958548	-3.703822028
	H277	-1.738561309	6.343584899	-5.999539005
	H278	-0.678079970	4.204557514	-4.807499242
	H279	-3.602312352	5.873849276	-3.677413560
	H280	0.807240836	8.928149207	2.939580087
	H281	-0.462704338	9.758000555	0.102359501
	H282	-0.087766299	12.624084449	0.379222939
	H283	2.275795845	9.446578151	2.076343903
	H284	-2.446049944	10.651432717	1.730693444
	H285	4.606048563	-3.023321009	-9.150507189
	H286	4.586224285	-4.485544383	-8.143618632
	H287	7.874191680	-5.183094940	-5.080659269
	H288	5.048761629	-5.091372565	-10.409083522
	H289	3.511019212	-5.577097784	-10.125154138
	H290	5.532950899	4.778337779	-0.919298903
	H291	5.728451929	7.441713419	0.532919981
	H292	4.833341245	8.089717982	-3.593050759
	H293	4.123942296	5.594985807	-0.165749485
	H294	6.793245760	8.077995654	-0.530705473
	H295	5.386106255	2.855331883	-6.745321776
	H296	5.985585225	6.753539273	-2.308350473
	H297	5.303932624	-7.009284476	4.979626377
	H298	7.054532706	-7.884878487	2.617124413
	H299	3.634690910	1.398623290	-6.781380424
	H300	5.153043338	-0.092840156	3.231759029
	H301	0.555794727	1.554474068	-8.649838247
	H302	-1.097829658	-2.141213347	-4.433255853
	H303	0.469290408	2.068219661	-6.961999984
	H304	4.271979313	-4.488014468	4.495723360
	H305	2.137130442	0.026624715	-6.596500618
	H306	3.930332534	-5.082617596	2.866081405
	H307	4.772539142	-7.300118666	3.306062495
	H308	6.423292880	-5.481225346	2.558345167
	H309	6.666883599	-5.064962360	4.261656265
	H310	-5.211938033	-0.805722314	-6.403802973
	H311	5.928706187	-9.460620487	4.897630000
	H312	4.982254416	-11.777825304	3.156552825
	H313	0.242844237	-0.035640232	-4.264809423
	H314	6.323325467	-11.799830176	5.751368184
	H315	4.765793239	-12.144214854	5.381598501
	H316	5.522451255	-3.345367902	2.021043445
	H317	-0.745220306	2.118416247	-4.913470064
	H318	-3.365887587	-2.073495320	-5.446434418
	H319	7.622743881	-9.933952712	-1.670173339
	H320	6.648003517	-7.672393275	-3.768488745
	H321	6.624138818	-8.180223820	-0.099789099
	H322	6.760444769	-10.541650588	-2.815181290
	H323	4.939246586	2.541897212	-9.502611167
	H324	6.336495972	-5.055955317	-0.197712965
	H325	2.290091506	-7.782437406	0.830530573
	H326	3.278384135	-2.022511319	-7.593199415
	H327	3.055652180	-9.223435441	-0.844606862
	H328	3.110443430	-6.978320008	-7.061874739
	H329	2.256608450	-5.529484599	-5.169030211
	H330	6.589792724	-12.426529885	3.508060109
	H331	5.758079863	-1.068615943	1.928678585
	H332	8.356777498	-5.801261271	0.798533621
	H333	7.241848698	-3.799097153	-0.154146257
	H334	8.971666439	-6.854610971	2.796298526
	H335	3.730305587	-2.712609539	5.130795299
	H336	3.742065979	-8.291307701	-3.678177123
	H337	4.966977890	-8.597099768	-2.440548532
	H338	1.910372901	-8.698888401	-2.086148784
	H339	1.335429454	-2.163089197	-6.151276672
	H340	-3.027271296	2.197594836	-5.956725798
	H341	-5.152962869	0.971457001	-6.556872660
	H342	2.320718542	-6.018298065	0.638691363
	H343	8.506665147	-7.335608020	0.584658564
	H344	7.415208965	-9.256529321	0.673568742
	H345	4.162886699	-1.044178544	5.085644159
	H346	5.569944601	-7.633079069	-6.745610767
	H347	6.518426507	-6.036106070	-3.142240150
	H348	0.453936132	-2.947011219	-7.494561681
	H349	4.251973125	4.193019035	-7.033483640
	H350	7.766929728	0.150148609	3.960805160
	H351	7.789704932	0.558714751	2.229880504
	H352	8.999131634	-6.745048467	-6.444136577
	H353	8.267418144	-8.028484328	-5.736249101
	H354	10.303718372	-8.565283937	-1.675540283

	H355	8.555962721	-8.228562353	-3.100699484
	H356	11.699268795	-7.551047037	-3.825707119
	H357	10.173507333	-6.846639814	-1.251462254
	H358	10.546360473	0.316893394	2.199624904
	H359	-2.980576679	13.197116297	3.726476778
	H360	10.205449115	-6.863679323	5.540968100
	H361	9.563079493	-4.441955998	4.393508400
	H362	-4.711523867	12.321567432	1.403214958
	H363	-4.092535404	10.302482862	3.648609672
	H364	-5.683006881	10.998314906	3.290267229
	H365	1.116653134	-3.236874229	-1.131741150
	H366	4.456074172	-1.623570513	0.146486487
	H367	-6.454190485	6.809249107	-1.565841258
	H368	-0.006681724	-1.112165784	-0.589160295
	H369	-2.013025795	-0.240630657	-0.870232586
	Mo370	3.838151871	-4.393972990	-2.447560687
	N371	-7.045748775	-7.189919863	-0.344029259
	N372	-8.779946157	-4.628708480	1.763691475
	N373	-8.105901042	-4.923337602	4.473907194
	N374	-5.425917910	-5.783179208	4.475669698
	N375	-4.196382043	-3.377231563	3.593717996
	N376	-4.877613691	-1.737377302	5.861809381
	N377	-3.644438827	-3.058286304	8.108341860
	N378	-1.114873273	-4.483962649	7.546919811
	N379	0.211709115	-2.571289096	6.146985353
	N380	-0.381515666	0.357432492	6.784523927
	N381	-0.659428357	0.204786830	9.672952736
	N382	5.535257694	-11.507320366	5.168948158
	N383	6.383798122	-9.257360732	4.001524917
	N384	5.035894996	-3.310229854	2.921211020
	N385	4.297836795	-1.975523576	4.702131673
	N386	5.359467191	-1.019696630	2.870447731
	N387	9.040118290	-6.964494421	3.832528941
	N388	-5.861195479	-2.948643633	-2.906970640
	N389	-1.946681698	-5.597425690	-3.192993723
	N390	-6.080989824	-0.445554064	-2.162983577
	N391	-8.351797874	0.647759902	-0.944532878
	N392	-7.851608524	0.351707760	1.805370657
	N393	-5.691344516	2.086579191	2.274728893
	N394	-3.119796444	2.678762345	-1.101509066
	N395	-2.458333730	0.664995480	-0.720590236
	N396	-6.323979691	4.732361521	2.860502612
	N397	-9.776442225	7.725463038	1.584793127
	N398	-9.672007370	9.511281156	2.897649417
	N399	9.475964557	2.664834655	2.417727921
	N400	6.292625951	7.216832906	-0.293066576
	N401	4.210090740	8.446192584	-2.873373473
	N402	1.553599290	7.845764569	-3.346065167
	N403	-3.173539124	6.404246305	-4.457261893
	N404	-3.361795417	8.254339068	-5.880328048
	N405	-4.454724705	8.239337845	-3.839800081
	N406	0.653749658	6.331975996	-1.065180335
	N407	0.975308591	8.231010088	0.977325814
	N408	-0.369647503	10.612608341	0.650699840
	N409	-1.889216268	10.322137048	-3.037862589
	N410	-2.752813830	11.620671835	1.703715126
	N411	4.099729396	-4.747859514	-10.245171376
	N412	2.461266897	-2.576419433	-7.867961046
	N413	1.421663741	0.221571570	-7.318573437
	N414	3.496946976	2.237694763	-7.353596777
	N415	-1.726013206	-2.989510073	-10.062708051
	N416	-3.294028226	-1.178622016	-8.481145005
	N417	8.132049539	-7.044851604	-5.989572270
	N418	4.259084370	-5.709785579	-4.384304312
	N419	3.690695915	-6.667444045	-6.287190353
	N420	9.228212918	-7.458315941	-3.059198211
	N421	0.139336543	2.269704084	-0.079493827
	N422	2.329666939	-0.246381208	1.328992716
	N423	0.998415211	1.703881128	-0.551022414
	N424	2.064240106	-1.325233785	1.374716508
	O425	-6.566383263	-5.143294800	1.628411202
	O426	-7.256365633	-2.806652352	4.491417798
	O427	-5.266238003	-5.188562940	6.674426140
	O428	-2.470823482	-3.953574926	4.966317735
	O429	-3.056138116	-0.483643603	5.347119530
	O430	-3.284364258	-0.961053139	8.957311554
	O431	-0.530317374	-3.952650741	9.695851479
	O432	2.190379182	-3.749324384	6.141124171
	O433	1.898460523	0.240111406	6.646529290
	O434	0.866451412	1.886363396	9.690382144

	O435	1.556313947	-0.245445750	12.461959687
	O436	7.057198188	-10.333403768	2.068497699
	O437	8.014271716	-7.674267843	5.764381306
	O438	11.592846711	-4.542380162	4.552469152
	O439	-5.615791413	-0.843625672	0.048016993
	O440	-3.695339811	-4.393650499	-4.010938842
	O441	-7.131378933	2.497994535	-0.393296960
	O442	-6.342142570	3.045158260	-3.334998768
	O443	-9.783275875	1.492640017	2.289961869
	O444	-5.662983385	1.529992606	4.490465888
	O445	-4.257078106	5.044140482	3.781768288
	O446	-6.579387964	7.251405800	5.335157947
	O447	10.768781501	-0.087955255	4.188948135
	O448	3.552769630	4.686587943	3.949374551
	O449	3.595464285	8.359960692	-0.681039916
	O450	0.990811315	10.070000745	-3.516452850
	O451	-1.408696641	7.196296652	-1.531615457
	O452	-0.693485272	7.190814068	2.161544401
	O453	2.427909211	5.696760989	1.450478902
	O454	0.854691948	11.623211200	2.302346888
	O455	-2.509364938	13.586972996	0.575836120
	O456	-3.267886333	10.997351570	-1.344242627
	O457	-4.953749659	13.710940555	3.622719273
	O458	-5.508079156	6.837860920	-1.324324071
	O459	1.755565821	-4.569272592	-8.768231540
	O460	-0.277508734	-0.737159026	-8.523816501
	O461	2.114275704	3.594762045	-8.542879367
	O462	5.862588857	4.319958129	-9.115813767
	O463	-5.009512894	-2.634568996	-8.846769795
	O464	-1.461944285	-6.605644408	-6.013469287
	O465	0.459852473	-5.777674774	-6.788241193
	O466	-4.000418930	2.322153578	-8.416742749
	O467	9.938447831	-5.405197249	-3.704926411
	O468	12.658375279	-6.985750784	-2.114592152
	O469	0.305054947	-6.289166101	-1.338608782
	O470	-0.255630710	-7.381877262	0.546067052
	O471	5.136476593	-10.903549168	-3.354673647
	O472	2.875606364	-10.951125058	-3.345084164
	O473	5.066553659	-5.810200318	-1.276528112
	O474	5.016592303	-7.549165421	0.146259286
	O475	2.609596659	-6.118242992	-2.095579850
	O476	8.946464893	-6.559382729	1.032039142
	O477	6.970795955	-4.563591115	0.388815461
	O478	-4.383102873	4.747231307	-2.455958908
	O479	7.711602748	-10.433058145	-2.512380522
	O480	7.510413668	-8.632690639	-0.090051019
	S481	3.645349236	4.914727987	-2.444808998
	S482	2.028085866	1.870195454	-3.371272352
	S483	2.137360814	-3.361265363	-3.771763325
	S484	3.970209040	1.965783008	-0.446511839
	S485	0.570985732	-0.862407316	-1.801528288
	S486	4.052105437	-0.734026423	-5.576916096
	S487	3.338049154	-3.513566210	-0.315386508
	S488	5.622754996	1.706568692	-3.331178184
	S489	5.702877152	-3.059038964	-3.017129378
	S490	6.470525950	-0.757105937	-0.649831927
	end			

TS

	bm52capexp147tf_1_53248.92			
Fe(139) 1.371	C1	-7.721382127	-5.357420614	1.267580175
Fe(140) -0.209	C2	-8.142712582	-6.501703371	0.336871883
Fe(141) 1.646	C3	-8.514990622	-3.421868371	2.559856463
Fe(142) -2.144	C4	-7.870573220	-3.681324539	3.917177011
Fe(143) 2.159	C5	-7.437719320	-5.262023591	5.732355814
Fe(144) -1.792	C6	-5.912904416	-5.380487655	5.666156858
Fe(145) -0.370	C7	-3.937446903	-5.692294050	4.275817484
	C8	-3.432280345	-4.247737493	4.296772787
	C9	-3.868193271	-1.936225041	3.581049098
	C10	-3.882693677	-1.297867907	4.979855395
	C11	-4.898718646	-1.131846233	7.163268501
	C12	-3.848374823	-1.682006506	8.138699130
	C13	-2.841342389	-3.697639718	9.118510130
	C14	-1.375925789	-4.022892250	8.792014758
	C15	0.232608275	-4.823929127	7.109518016
	C16	0.968062358	-3.665735298	6.423740701
	C17	0.624016580	-1.443215603	5.365853730
	C18	0.760495869	-0.228520859	6.299835005
	C19	-0.258348718	-1.239492032	4.116477013
	C20	-0.027433071	0.114674712	3.419161500

	C21	-0.079121120	-2.416750176	3.151631931
	C22	-0.351995608	1.531278292	7.598034322
	C23	0.022818489	1.212695738	9.057776592
	C24	-1.677953848	2.318155659	7.468858938
	C25	-1.737497390	3.484851266	8.460413599
	C26	-1.861461643	2.827921238	6.032650878
	C27	-0.395466756	-0.087710370	11.062708475
	C28	1.070738526	-0.349068055	11.343637173
	C29	5.896301779	-11.584191505	3.759954107
	C30	6.515184616	-10.311780016	3.195825561
	C31	6.780165602	-7.884199659	3.678566389
	C32	7.999392518	-7.486326644	4.530242411
	C33	5.595593590	-6.936050389	3.918873003
	C34	5.926384388	-5.494224553	3.551169622
	C35	4.686405755	-4.607203017	3.511576515
	C36	4.907946487	-2.108616836	3.550588886
	C37	10.264982699	-6.557464657	4.486498736
	C38	10.501509689	-5.064595616	4.474796769
	C39	-5.315144222	-2.671264964	-1.555666585
	C40	-5.685628324	-1.240776603	-1.117704764
	C41	-3.778760785	-2.819030521	-1.504503443
	C42	-3.242756083	-4.260943288	-1.620766078
	C43	-2.978082879	-4.748496036	-3.045880198
	C44	-6.186721699	0.997702719	-2.022342115
	C45	-7.279362721	1.449974802	-1.043398689
	C46	-6.366868929	1.604496341	-3.419460720
	C47	-9.433265016	0.920991341	0.000236960
	C48	-9.036846783	0.945179443	1.479723663
	C49	-7.328537099	0.441591150	3.163135125
	C50	-6.155385789	1.400507816	3.372247098
	C51	-4.603166065	3.041016727	2.435233279
	C52	-5.046878569	4.347310111	3.134079911
	C53	-4.025039821	3.477760725	1.080672518
	C54	-3.406845223	2.416076926	0.234506708
	C55	-2.986946769	1.131722564	0.470402742
	C56	-2.553351538	1.600882718	-1.664827298
	C57	-6.846725857	6.028473956	3.232872099
	C58	-6.759041665	6.184441491	4.750041667
	C59	-8.318367986	6.084002314	2.760017036
	C60	-8.944979016	7.443438019	2.650789990
	C61	-8.869130953	8.547081739	3.478108923
	C62	-10.204865810	8.961617165	1.759206413
	C63	9.238119236	1.622916582	3.444931148
	C64	10.259883542	0.512584363	3.275553123
	C65	7.842300738	0.982062544	3.261600274
	C66	6.708970750	1.958740754	3.472839635
	C67	6.140466841	2.161018686	4.743046654
	C68	6.187217592	2.696105959	2.394554372
	C69	5.087518898	3.063843791	4.934534396
	C70	5.151709781	3.613685654	2.572593324
	C71	4.595315166	3.789777787	3.842116249
	C72	5.378219532	6.871957236	-1.383488223
	C73	4.319255146	7.967150571	-1.595759394
	C74	4.712355889	5.529870519	-1.084438336
	C75	3.308406375	9.545136281	-3.205714419
	C76	1.833505530	9.167489108	-3.365435675
	C77	0.210757009	7.275053691	-3.323842774
	C78	-0.257024534	6.928389537	-1.886676667
	C79	0.243052965	6.027559766	-4.236702531
	C80	-1.009705102	5.149408435	-4.344804049
	C81	-2.152913652	5.683893173	-5.224393715
	C82	-3.672952790	7.605792031	-4.740578612
	C83	0.263170921	5.987122704	0.312372163
	C84	0.111413548	7.211048153	1.228236259
	C85	1.255492049	5.017728955	0.986695183
	C86	1.203163764	9.274430174	1.963598793
	C87	0.558774823	10.617421526	1.653621409
	C88	-0.857935179	11.879819257	0.123777928
	C89	-2.131085550	12.432713501	0.809633621
	C90	-1.024867857	11.883545028	-1.398402689
	C91	-2.164594770	11.003861097	-1.895913284
	C92	-4.075247626	11.947879845	2.221483990
	C93	-3.983484852	13.047549540	3.271267999
	C94	-4.729891687	10.677820300	2.833937993
	C95	-4.973286451	9.604585128	1.796903366
	C96	-6.160304224	9.594762459	1.048122666
	C97	-4.004594877	8.628177037	1.512214595
	C98	-6.367612123	8.658126329	0.033593178
	C99	-4.181949360	7.699780660	0.481857328
	C100	-5.363801349	7.731930421	-0.252591435

C101	4.086446151	-3.977920452	-9.002570794
C102	2.659914651	-3.727346423	-8.546353646
C103	1.207652181	-2.196684544	-7.232607524
C104	0.710209706	-0.837296415	-7.739363375
C105	1.127237876	1.583513354	-7.694856542
C106	2.295337662	2.545666752	-7.902171325
C107	4.574642029	3.235621648	-7.405502027
C108	5.186748656	3.373600622	-8.784625800
C109	-2.761382525	-3.491179476	-9.142545406
C110	-3.788882092	-2.404107776	-8.803728818
C111	-2.272777254	-4.174499842	-7.844864128
C112	-1.536350889	-5.491927258	-8.149167534
C113	-0.853591943	-6.017579097	-6.906848269
C114	-4.193974564	-0.085340908	-8.146273409
C115	-3.718081760	1.199608956	-8.794188252
C116	-4.570390599	0.045027360	-6.649243908
C117	-3.333621388	0.055917053	-5.797223435
C118	-2.607264962	1.242995694	-5.600763723
C119	-2.794490919	-1.156876358	-5.337369944
C120	-1.325429657	1.195925196	-5.043399009
C121	-1.514473980	-1.202580327	-4.779725161
C122	-0.762594708	-0.028523708	-4.673258441
C123	7.905956875	-6.222809210	-4.758240917
C124	9.092425550	-6.317779367	-3.786818708
C125	6.595926402	-6.596388103	-4.050687827
C126	5.336760195	-6.361886737	-4.820469561
C127	4.961502136	-6.934143436	-6.018951179
C128	3.248522136	-5.810170288	-5.181415867
C129	10.304627094	-7.555305595	-2.093389979
C130	11.653609198	-7.314966473	-2.734852164
C131	0.594896596	-6.947485698	-0.182810067
C132	2.086002467	-6.985746812	0.132358396
C133	3.002339023	-7.100847310	-1.122461055
C134	2.903370893	-8.533077490	-1.712345314
C135	3.941864244	-8.872955171	-2.784690895
C136	3.967259012	-10.364432741	-3.183796524
C137	4.462944187	-6.855317807	-0.708686086
C138	3.600936538	-0.685086291	-2.236987027
Fe139	3.802384090	-1.997418485	-3.623225970
Fe140	2.244330883	0.706814868	-1.427721632
Fe141	5.034757178	0.433973230	-1.534791954
Fe142	3.829915092	0.491476720	-3.775680668
Fe143	3.855172963	2.735993396	-2.485116066
Fe144	4.751679599	-2.054540117	-1.222841216
Fe145	2.171473124	-2.401362758	-1.463757471
H146	-6.387459015	-7.540531154	0.317205390
H147	-6.573263286	-6.554598582	-0.970777024
H148	-9.689400192	-4.767859912	1.394960973
H149	-8.863346044	-6.107151315	-0.397631733
H150	-8.704787841	-7.223233529	0.954582055
H151	-7.857142580	-2.716076905	2.031804506
H152	-8.628145793	-5.583151042	3.968027284
H153	-9.477311522	-2.920397893	2.728068975
H154	-7.854764566	-6.220662457	6.066813270
H155	-5.978466207	-5.838615485	3.656894517
H156	-7.658588060	-4.506155189	6.497191062
H157	-2.874801277	-1.742679340	3.155019554
H158	-4.947259916	-3.697968691	3.017727666
H159	-3.691126392	-6.162305744	3.314484182
H160	-4.742440609	-0.046870849	7.105600937
H161	-5.625042958	-2.278749132	5.485529676
H162	-3.414460551	-6.233706425	5.072545652
H163	-4.612992551	-1.440249786	2.942545958
H164	-4.181285084	-3.594404142	7.452902875
H165	-5.894237972	-1.321572618	7.588812198
H166	-0.369173946	-3.369322746	3.615304582
H167	-0.699354609	-2.277663523	2.252240263
H168	0.960053706	-2.486096702	2.804071211
H169	-1.293804702	-1.241475151	4.477084783
H170	-3.346317398	-4.641651459	9.372544424
H171	-1.867365407	2.010178791	5.300119577
H172	-1.446196211	-0.254632190	9.211280673
H173	-1.680752789	3.149793436	9.505845233
H174	0.662626904	0.022661471	2.568877659
H175	0.370858183	0.883872013	4.095767888
H176	9.313097995	1.980457678	4.489949955
H177	-2.817134159	3.363142591	5.936721112
H178	-2.682298578	4.032286051	8.329075068
H179	-1.051258926	3.527908246	5.769115772
H180	-0.908439076	4.188706675	8.284498198

H181	-2.502863138	1.620740246	7.702909965
H182	0.476479704	2.161174942	7.239435850
H183	-1.290625909	0.022621137	6.388160998
H184	-0.984396938	0.504421055	3.037433069
H185	1.544582610	4.245123363	0.259554598
H186	0.716840555	4.531344940	1.813183179
H187	2.663527148	5.319597307	2.332886514
H188	3.209760716	4.681035472	4.870273250
H189	4.750727417	4.168769370	1.724524385
H190	6.582388037	2.531701666	1.389373327
H191	6.515764818	1.595967729	5.600891741
H192	-0.821002802	-2.730104517	6.239056350
H193	0.206093868	-5.664944603	6.401747301
H194	0.832677325	-5.131720450	7.975186372
H195	-1.865900578	-4.569818291	6.846276837
H196	-2.820831611	-3.063689470	10.012811520
H197	4.644151873	3.194148991	5.925743575
H198	1.653219648	-1.651814307	5.049021622
H199	-0.745516329	0.717101702	11.729771672
H200	-0.942225573	-1.007585843	11.322428576
H201	1.658469767	-0.734428116	10.478455858
H202	11.136552437	-7.065054218	4.045711871
H203	-1.760206826	-5.993535154	-4.122690412
H204	1.215696196	-6.061631899	-1.777390123
H205	-2.264703166	-6.245316022	-8.482810712
H206	-0.802871542	-5.340422151	-8.954218005
H207	-3.125724621	-4.385849600	-7.181341673
H208	-3.120345799	1.048136236	-9.724047976
H209	-3.348469309	-4.232899110	-9.705676175
H210	-3.339604246	-2.195789102	-2.301744882
H211	-3.958151896	-4.966721875	-1.164629692
H212	-2.282180984	-1.024786352	-8.423071762
H213	-5.133447316	-0.319870332	-8.688918822
H214	-1.037411278	-2.404589037	-9.549398398
H215	-1.332206344	-5.866367556	-2.419724983
H216	-1.618728742	-3.475622852	-7.300247613
H217	-1.214626016	-3.762237058	-10.465717357
H218	-6.732061840	-3.453185863	-2.841056208
H219	-3.446038434	-2.411789236	-0.538046901
H220	0.832600521	-5.282640583	-7.594341828
H221	-2.318242383	-4.351091164	-1.032732531
H222	-3.826358806	2.602586451	3.077486411
H223	-3.027257472	0.520986758	1.362916186
H224	-8.142396102	0.786165680	3.815796904
H225	-7.266410852	-0.071840678	1.091146816
H226	-6.923916096	4.079840775	2.349866271
H227	-8.317529090	8.737626332	4.391154063
H228	-6.907249594	5.228596928	5.309456665
H229	-8.926781919	5.420715231	3.400661358
H230	-6.261846248	6.832720484	2.759489264
H231	-10.884911334	9.508954284	1.112138867
H232	-3.267030104	4.250124292	1.287610993
H233	-4.822982994	3.964031032	0.496548888
H234	-9.834641903	10.441120141	3.246014240
H235	-8.351037136	5.648083700	1.748883876
H236	-6.173052037	2.010754005	1.380186721
H237	-7.023720609	-0.548890694	3.532837236
H238	-9.881189663	1.902163872	-0.211001979
H239	-2.212403898	1.500285512	-2.692840259
H240	-10.213856994	0.161552414	-0.136720718
H241	10.300752577	3.205584646	2.696711833
H242	8.693363486	3.309551101	2.423261742
H243	1.547207892	5.982821495	-1.423517034
H244	-0.723084538	5.501628439	0.285021282
H245	1.736333706	8.068554704	0.312205352
H246	-3.437188069	3.541287574	-1.608489918
H247	-5.107060188	4.157203420	-2.783058040
H248	-6.716597880	3.391040628	-4.151488272
H249	-4.794221040	5.408693692	-1.849938195
H250	-5.547016822	1.240718107	-4.060464967
H251	-7.326299107	1.278894044	-3.849737751
H252	-0.071060984	11.619736159	-1.876032794
H253	-1.269354011	12.916547967	-1.691235326
H254	-0.910320128	10.157314438	-3.310914930
H255	-2.580181942	9.590429822	-3.270855460
H256	-3.660264390	9.219096286	-6.010107782
H257	-2.645705692	4.850247756	-5.747340374
H258	-7.304497835	8.653486492	-0.529794748
H259	-3.085624533	8.568898595	2.100769431
H260	-4.708827164	7.761484105	-2.964905768

	H261	1.063872447	5.384366220	-3.879997323
	H262	2.340368142	7.199637693	-3.225981658
	H263	-1.396991378	4.887151433	-3.347832632
	H264	-8.364815520	-0.227401935	-1.467265172
	H265	3.641826526	10.003086891	-4.146549995
	H266	-6.087432930	-0.954261971	-3.048560987
	H267	0.537326170	6.364081560	-5.244302073
	H268	-3.387501085	6.993836974	0.236039960
	H269	3.342186074	10.317992118	-2.424175387
	H270	-2.910461887	7.792295482	-6.650055869
	H271	-5.099920239	8.952790241	-4.160251899
	H272	-5.167937835	-3.513980765	-3.430380330
	H273	-5.740441314	-3.334894206	-0.779684762
	H274	-6.944976993	10.323930000	1.263069658
	H275	-5.260478640	1.396758388	-1.585017694
	H276	-0.488545310	8.028550589	-3.697594946
	H277	-1.737481110	6.343241619	-5.996646529
	H278	-0.676872772	4.206347963	-4.800436010
	H279	-3.606552994	5.869699676	-3.679945712
	H280	0.818637817	8.938987178	2.938381220
	H281	-0.458150144	9.763328560	0.098211841
	H282	-0.091146068	12.631438016	0.366562627
	H283	2.282131932	9.452411686	2.063893218
	H284	-2.447069054	10.650196974	1.723468964
	H285	4.606254469	-3.019899514	-9.149468405
	H286	4.586069719	-4.486990709	-8.151152892
	H287	7.865518848	-5.164743470	-5.056843254
	H288	5.046939614	-5.077248747	-10.424504862
	H289	3.512114054	-5.569875758	-10.135257143
	H290	5.500448119	4.782126057	-0.937620372
	H291	5.714806650	7.436360597	0.549478690
	H292	4.831427302	8.096332295	-3.584610139
	H293	4.111520028	5.610552926	-0.167522009
	H294	6.816539559	8.043564999	-0.497586834
	H295	5.369913979	2.905605707	-6.719025636
	H296	5.986747956	6.771987395	-2.295566731
	H297	5.285547751	-7.008816647	4.974536093
	H298	7.046375447	-7.868362013	2.613771623
	H299	3.643834993	1.419819148	-6.774693709
	H300	5.180271415	-0.092587847	3.291941303
	H301	0.558257855	1.551381258	-8.631633492
	H302	-1.095011668	-2.154047180	-4.444650677
	H303	0.473473686	2.058126806	-6.942521601
	H304	4.252883523	-4.491267731	4.512040284
	H305	2.142022310	0.024244963	-6.576743773
	H306	3.911024391	-5.056814304	2.870492197
	H307	4.759646353	-7.298139560	3.298989722
	H308	6.394242117	-5.470764530	2.555614150
	H309	6.651462006	-5.062842491	4.258940158
	H310	-5.218117923	-0.805080818	-6.390696870
	H311	5.923548427	-9.442517131	4.896062857
	H312	4.980881858	-11.763731179	3.176131621
	H313	0.253640937	-0.048834788	-4.288265261
	H314	6.341504962	-11.752790347	5.763874908
	H315	4.787063506	-12.123859229	5.408731452
	H316	5.525606221	-3.323959561	2.061897994
	H317	-0.742172337	2.108396110	-4.908240342
	H318	-3.368598441	-2.080207142	-5.439992494
	H319	7.615702704	-9.937764626	-1.665598769
	H320	6.624549668	-7.667465178	-3.784532833
	H321	6.615804266	-8.183125008	-0.101012373
	H322	6.750689472	-10.542300226	-2.809565570
	H323	4.957704887	2.540338432	-9.491497078
	H324	6.363914747	-5.072956898	-0.236997522
	H325	2.283830090	-7.802027147	0.837651424
	H326	3.281283240	-2.023666300	-7.601053879
	H327	3.018999048	-9.232804004	-0.869579210
	H328	3.051321420	-6.879765462	-6.986827877
	H329	2.240775787	-5.428890933	-5.078746610
	H330	6.591105747	-12.408697320	3.524156841
	H331	5.783248766	-1.068886575	1.987322173
	H332	8.365262134	-5.801136427	0.787117882
	H333	7.229420700	-3.788812517	-0.167301930
	H334	8.970401198	-6.848849716	2.795793750
	H335	3.702302354	-2.702255210	5.152602221
	H336	3.733986741	-8.288767107	-3.694183951
	H337	4.949331872	-8.593045111	-2.448620301
	H338	1.888023123	-8.675853702	-2.112711205
	H339	1.347236996	-2.170235762	-6.139958596
	H340	-3.031534056	2.192256304	-5.936348455

H341	-5.152571411	0.972481521	-6.541790187
H342	2.305427022	-6.036252968	0.649283719
H343	8.503640698	-7.337376194	0.581637874
H344	7.409161500	-9.253561111	0.675332593
H345	4.154449343	-1.041297324	5.126665756
H346	5.508454788	-7.570418825	-6.703757380
H347	6.535007550	-6.054013283	-3.100100553
H348	0.457023040	-2.948609406	-7.482470280
H349	4.232171472	4.225170378	-7.066172784
H350	7.759671505	0.148631619	3.974456466
H351	7.788122933	0.560693789	2.245143326
H352	8.977588612	-6.709632044	-6.438116491
H353	8.259898790	-8.003121109	-5.736703916
H354	10.308738566	-8.583032080	-1.697613935
H355	8.543772424	-8.223139374	-3.094310635
H356	11.697252520	-7.545132033	-3.826750449
H357	10.170199712	-6.872426064	-1.239495212
H358	10.541604653	0.305995167	2.214418284
H359	-2.973630539	13.185839948	3.724770981
H360	10.193002271	-6.864511763	5.545117474
H361	9.581618788	-4.436575573	4.385703504
H362	-4.714617222	12.320862168	1.404150547
H363	-4.085288889	10.296915787	3.641673371
H364	-5.678932277	10.990681216	3.292714130
H365	1.089526746	-3.309967526	-0.954239225
H366	3.862350856	-1.144576070	0.035471526
H367	-6.459845208	6.800662583	-1.568055126
H368	0.138080348	-0.938185807	-0.537491440
H369	-2.022937109	-0.248942876	-0.869243174
Mo370	3.843438245	-4.402026166	-2.350699547
N371	-7.076651228	-7.199601102	-0.357349742
N372	-8.753033305	-4.611216097	1.760174041
N373	-8.060667834	-4.903117595	4.468126027
N374	-5.378304395	-5.763144842	4.479363992
N375	-4.160850950	-3.361551039	3.570306351
N376	-4.845568169	-1.714379770	5.834089969
N377	-3.621176788	-3.020964066	8.089523959
N378	-1.121497271	-4.521431383	7.547065245
N379	0.181802590	-2.612757687	6.107258101
N380	-0.389790868	0.327924778	6.765061347
N381	-0.647325682	0.199064347	9.662246448
N382	5.545273215	-11.477862238	5.183710202
N383	6.381841895	-9.241555552	4.000883131
N384	5.037413555	-3.298410591	2.960077596
N385	4.298966757	-1.973899113	4.748251159
N386	5.412254593	-1.019063119	2.945396068
N387	9.033975778	-6.954507475	3.832587242
N388	-5.848580074	-2.942528712	-2.896240676
N389	-1.958012914	-5.612911138	-3.191122261
N390	-6.075855120	-0.444750311	-2.148499098
N391	-8.356390808	0.639386398	-0.933919622
N392	-7.856180017	0.352437928	1.814542856
N393	-5.686757239	2.078398434	2.278752328
N394	-3.125407835	2.673400221	-1.101216908
N395	-2.473541430	0.655508871	-0.720701093
N396	-6.319758261	4.725018499	2.852865072
N397	-9.782867026	7.715204041	1.580955385
N398	-9.673442618	9.501633419	2.892490369
N399	9.479891005	2.654526103	2.434191379
N400	6.282425374	7.200268590	-0.270848860
N401	4.212288128	8.460482435	-2.865188068
N402	1.553296421	7.847981418	-3.335472442
N403	-3.176291011	6.401621675	-4.457907859
N404	-3.351826152	8.262683036	-5.868686538
N405	-4.457320353	8.235134070	-3.836112447
N406	0.649397488	6.334792258	-1.057564861
N407	0.975385438	8.234876047	0.977061818
N408	-0.365629436	10.618449508	0.645693949
N409	-1.888197713	10.295809159	-3.025448383
N410	-2.754422887	11.619187383	1.698207557
N411	4.097495582	-4.738167723	-10.254267629
N412	2.463328101	-2.579737706	-7.865659439
N413	1.437092032	0.218419369	-7.309435490
N414	3.500145580	2.255493460	-7.347855356
N415	-1.724931966	-2.981382357	-10.048553912
N416	-3.294430098	-1.177600764	-8.467903166
N417	8.114496135	-7.018654089	-5.981719140
N418	4.250790969	-5.646761398	-4.316954656
N419	3.648946913	-6.572203724	-6.224161560
N420	9.221159161	-7.457273069	-3.055316989

	N421 0.109490614 2.280474082 -0.147645107<br>N422 2.622178119 -0.821997089 0.295518525<br>N423 0.956728324 1.709361265 -0.629702583<br>N424 1.898943930 -1.617741018 0.783579225<br>O425 -6.542765056 -5.141533674 1.599082171<br>O426 -7.205815448 -2.789105297 4.472152368<br>O427 -5.222395511 -5.144510560 6.671248257<br>O428 -2.438525591 -3.912022372 4.957898982<br>O429 -3.040668936 -0.443070986 5.294706396<br>O430 -3.292626212 -0.923593558 8.952581404<br>O431 -0.500582085 -3.880839009 9.654673846<br>O432 2.187174881 -3.743770912 6.181310197<br>O433 1.886591816 0.194515354 6.609310160<br>O434 0.894833584 1.866005865 9.655369536<br>O435 1.572726548 -0.214766732 12.449067397<br>O436 7.057861405 -10.324030854 2.072260937<br>O437 7.996222001 -7.648484455 5.763641187<br>O438 11.608222122 -4.558380891 4.572407514<br>O439 -5.615200652 -0.846829452 0.063010234<br>O440 -3.687323553 -4.383930420 -4.012333095<br>O441 -7.145146948 2.496443026 -0.384032914<br>O442 -6.335982211 3.042294450 -3.325744751<br>O443 -9.795102980 1.482226421 2.297624103<br>O444 -5.664036767 1.532040804 4.496686506<br>O445 -4.263121799 5.031895479 3.796883828<br>O446 -6.594557276 7.246016022 5.326182037<br>O447 10.732311666 -0.131768316 4.200898626<br>O448 3.547587733 4.683448583 3.955745211<br>O449 3.604477638 8.388897419 -0.669707331<br>O450 0.988169602 10.069883676 -3.520014835<br>O451 -1.417068412 7.187502854 -1.531499673<br>O452 -0.703982932 7.209387312 2.159748855<br>O453 2.414942427 5.702163164 1.466573629<br>O454 0.860106414 11.630692421 2.295581500<br>O455 -2.509246724 13.589906951 0.578549680<br>O456 -3.273523570 10.984470273 -1.342898668<br>O457 -4.943325626 13.713970715 3.622472130<br>O458 -5.513408899 6.831354776 -1.327835309<br>O459 1.756244444 -4.569258577 -8.772032170<br>O460 -0.275412640 -0.736501165 -8.498865569<br>O461 2.098017383 3.602305959 -8.525086239<br>O462 5.909816428 4.303561787 -9.102693188<br>O463 -5.008557352 -2.639300640 -8.816623120<br>O464 -1.448925021 -6.607604079 -6.011813086<br>O465 0.466127597 -5.765790608 -6.789559918<br>O466 -3.995409558 2.322483793 -8.395590280<br>O467 9.938084046 -5.406010428 -3.699655235<br>O468 12.648725896 -6.967658035 -2.115317166<br>O469 0.274785888 -6.298707100 -1.311026557<br>O470 -0.262384693 -7.419607932 0.561098332<br>O471 5.119392933 -10.903508088 -3.344981904<br>O472 2.857795825 -10.945205175 -3.350551433<br>O473 5.092845838 -5.891123665 -1.330382444<br>O474 5.001557006 -7.573572617 0.154326877<br>O475 2.595776742 -6.104581507 -2.063094474<br>O476 8.947637101 -6.562637911 1.027255649<br>O477 6.982941188 -4.574802241 0.356284194<br>O478 -4.374457669 4.743535785 -2.450645117<br>O479 7.702141231 -10.434041625 -2.509733818<br>O480 7.503312542 -8.632299875 -0.090977408<br>S481 3.594912789 4.955519839 -2.443027822<br>S482 2.097805268 1.839556558 -3.469177702<br>S483 2.037653021 -3.323318985 -3.456157134<br>S484 3.876098009 1.935934805 -0.438072916<br>S485 0.539224855 -0.867454610 -1.844858280<br>S486 3.981072298 -0.834194212 -5.504083541<br>S487 3.535368364 -3.616505826 -0.184781444<br>S488 5.649495696 1.689374371 -3.275545953<br>S489 5.664843399 -3.038446347 -3.038001482<br>S490 6.373475425 -0.806732932 -0.352217692<br>end

product

Fe(139) 2.398<br>Fe(140) 0.189<br>Fe(141) 0.094<br>Fe(142) -2.361	bm52capexp147tf.car_7<br>C1 -7.714458406 -5.350019965 1.285298579<br>C2 -8.061173483 -6.505849943 0.339177983<br>C3 -8.602200240 -3.454988138 2.574035204<br>C4 -7.973353208 -3.723235595 3.939337875<br>C5 -7.565128240 -5.328697062 5.739775776<br>C6 -6.039205085 -5.445201136 5.683487506

Fe(143) 2.763	C7	-4.057103012	-5.762231613	4.296469225
Fe(144) -2.139	C8	-3.512776366	-4.332475846	4.339089288
Fe(145) -0.202	C9	-3.888480927	-1.996887259	3.686094888
	C10	-3.893913141	-1.390045248	5.096468289
	C11	-4.944582649	-1.223522937	7.263211254
	C12	-3.880058976	-1.791581605	8.212938727
	C13	-2.883519857	-3.819666885	9.166348868
	C14	-1.408500900	-4.091039620	8.837248655
	C15	0.240608503	-4.716967071	7.117933119
	C16	0.969301178	-3.582064068	6.394812405
	C17	0.669301962	-1.332038881	5.391596916
	C18	0.789091722	-0.106187908	6.311265051
	C19	-0.170278994	-1.111301741	4.114339266
	C20	0.292001853	0.138914691	3.357741329
	C21	-0.125866888	-2.357678315	3.225718282
	C22	-0.378814083	1.606459364	7.630345990
	C23	-0.021998234	1.256190230	9.087657460
	C24	-1.715410498	2.373188220	7.496065869
	C25	-1.795977329	3.531671135	8.495773268
	C26	-1.894536611	2.889749836	6.061964332
	C27	-0.436784477	-0.106536455	11.049380417
	C28	1.025776810	-0.389194527	11.327439852
	C29	5.880141005	-11.621202356	3.698552354
	C30	6.515390171	-10.342364585	3.164887181
	C31	6.818034478	-7.929976867	3.685041617
	C32	8.044657509	-7.543261987	4.530656840
	C33	5.645814983	-6.966797465	3.932717786
	C34	5.987826518	-5.526526404	3.563488153
	C35	4.749673582	-4.636278635	3.512396186
	C36	4.902703111	-2.132372252	3.513355289
	C37	10.287756711	-6.554278073	4.469817661
	C38	10.472383704	-5.053600567	4.465701107
	C39	-5.314377643	-2.664581411	-1.582562508
	C40	-5.687239962	-1.234475936	-1.150345624
	C41	-3.777532357	-2.807755733	-1.517249658
	C42	-3.228660793	-4.244759595	-1.627746592
	C43	-2.966322215	-4.748009455	-3.047234129
	C44	-6.163798428	1.005723462	-2.058611926
	C45	-7.252798016	1.466754260	-1.078667510
	C46	-6.341164284	1.612273209	-3.456321818
	C47	-9.409603243	0.954255474	-0.033291509
	C48	-9.014164492	0.960005692	1.446309782
	C49	-7.317374336	0.418103777	3.128295074
	C50	-6.137562263	1.366668304	3.346489309
	C51	-4.599629063	3.027359628	2.418261629
	C52	-5.029570870	4.347995496	3.106956409
	C53	-4.003561409	3.433083783	1.061725368
	C54	-3.365320003	2.366034839	0.227430444
	C55	-2.858507899	1.111531187	0.466685671
	C56	-2.458653046	1.589745314	-1.664590579
	C57	-6.832333970	6.033506105	3.246515985
	C58	-6.716140472	6.197220052	4.760487467
	C59	-8.312901355	6.092693849	2.801978214
	C60	-8.926983153	7.456958412	2.680321866
	C61	-8.863045860	8.557655104	3.512634075
	C62	-10.159745690	8.985197920	1.767407108
	C63	9.252987250	1.661044925	3.418350906
	C64	10.297748782	0.574066168	3.246879074
	C65	7.866477676	0.997539377	3.229093412
	C66	6.722340361	1.961824268	3.436188378
	C67	6.139357870	2.147387049	4.702299076
	C68	6.214163651	2.715104203	2.362175670
	C69	5.086715934	3.050422363	4.894982396
	C70	5.179441813	3.633036972	2.542076623
	C71	4.611069133	3.795432644	3.808041557
	C72	5.402197229	6.870545015	-1.408579665
	C73	4.326792945	7.951536109	-1.612666196
	C74	4.764995049	5.528373361	-1.050789315
	C75	3.311092322	9.540580580	-3.209463110
	C76	1.834981896	9.169911628	-3.375036979
	C77	0.211379458	7.272859157	-3.333403231
	C78	-0.256833228	6.928581360	-1.895757823
	C79	0.246773875	6.027838435	-4.248970605
	C80	-1.003302154	5.146787566	-4.358932702
	C81	-2.149114612	5.683385389	-5.231961352
	C82	-3.677285966	7.595846663	-4.751162464
	C83	0.268794517	5.969514082	0.296285267
	C84	0.114247325	7.180750682	1.229088273
	C85	1.270894501	4.995498301	0.948196599
	C86	1.188814694	9.248179778	1.980150759

C87	0.553674262	10.596323534	1.674335492
C88	-0.846992685	11.878861266	0.144114020
C89	-2.124445715	12.430875260	0.820526009
C90	-0.993880619	11.890525008	-1.380151992
C91	-2.147141571	11.041349643	-1.900653672
C92	-4.071847820	11.946668936	2.224773816
C93	-3.990756404	13.052944842	3.268564206
C94	-4.730915386	10.680833657	2.839065396
C95	-4.968730911	9.607147835	1.802318152
C96	-6.140860875	9.612718659	1.031502828
C97	-4.008321838	8.617109888	1.539819280
C98	-6.341323184	8.677055411	0.015567702
C99	-4.180620813	7.686396444	0.510490208
C100	-5.347414135	7.733888959	-0.246710745
C101	4.083115904	-3.985577123	-8.999552979
C102	2.657385496	-3.730001254	-8.546405293
C103	1.199758221	-2.175109317	-7.261477845
C104	0.704665369	-0.828646645	-7.798964001
C105	1.106261712	1.595829168	-7.764236181
C106	2.289814327	2.544205133	-7.954599555
C107	4.578405290	3.181288076	-7.447188881
C108	5.114506211	3.393768474	-8.849836072
C109	-2.743724822	-3.498993057	-9.152052866
C110	-3.764327836	-2.400072232	-8.831062938
C111	-2.250900971	-4.164388349	-7.847116306
C112	-1.535090830	-5.495593680	-8.140846990
C113	-0.862341896	-6.027932999	-6.895795828
C114	-4.161288921	-0.079667842	-8.183703937
C115	-3.670864239	1.201387174	-8.827424370
C116	-4.580898663	0.061446739	-6.698570341
C117	-3.355445351	0.085842681	-5.833310030
C118	-2.630266977	1.275394756	-5.652164573
C119	-2.807329867	-1.125117551	-5.377212669
C120	-1.338449819	1.232629388	-5.116897636
C121	-1.521539717	-1.162925940	-4.837738125
C122	-0.767796270	0.011611860	-4.748192388
C123	7.907104815	-6.259797061	-4.789421378
C124	9.090809583	-6.337109660	-3.810985774
C125	6.595799136	-6.561953985	-4.048705806
C126	5.327041860	-6.338858157	-4.808181195
C127	4.943493167	-6.918406166	-6.000264050
C128	3.225965031	-5.813432143	-5.145922103
C129	10.310440213	-7.553428958	-2.110335777
C130	11.666108079	-7.335700433	-2.746347925
C131	0.616061032	-6.897279745	-0.136383666
C132	2.110970130	-6.933105684	0.170193648
C133	3.014465510	-7.047201532	-1.093276400
C134	2.919967651	-8.483435285	-1.679388723
C135	3.958311938	-8.832010461	-2.749328349
C136	3.981803403	-10.324590200	-3.147279253
C137	4.479890296	-6.790071056	-0.706122670
C138	3.605050079	-0.684543398	-2.237435689
Fe139	3.777006309	-1.979909805	-3.716602060
Fe140	2.008348465	0.560602145	-1.293113557
Fe141	4.950243940	0.476519499	-1.501143714
Fe142	3.748458396	0.535744802	-3.731059950
Fe143	3.752856610	2.689028363	-2.320875418
Fe144	4.830806032	-2.034097668	-1.404602879
Fe145	2.122927475	-2.254122282	-1.322857368
H146	-6.269667897	-7.483979031	0.374197220
H147	-6.445780251	-6.496839191	-0.914039880
H148	-9.703067992	-4.819897453	1.354433747
H149	-8.773626204	-6.141596875	-0.418712019
H150	-8.612659969	-7.249826491	0.940133319
H151	-7.958612403	-2.717089167	2.072953856
H152	-8.733837295	-5.625642456	3.957580287
H153	-9.585262323	-2.991242213	2.733123013
H154	-7.984111019	-6.292892046	6.055892184
H155	-6.092710287	-5.861549677	3.664441020
H156	-7.793770621	-4.586889723	6.515937816
H157	-2.886156715	-1.831925688	3.271970046
H158	-5.042367417	-3.708356039	3.109707835
H159	-3.823014672	-6.218742960	3.325287932
H160	-4.793448176	-0.136507373	7.229028491
H161	-5.683974723	-2.314923756	5.552976178
H162	-3.546713327	-6.332152994	5.081770898
H163	-4.610890646	-1.461070679	3.054695947
H164	-4.265406331	-3.700979376	7.535220834
H165	-5.936809836	-1.428052679	7.689619532
H166	-0.479607809	-3.249073801	3.759665564

H167	-0.776786829	-2.220641891	2.347274866
H168	0.886381538	-2.538244028	2.837034660
H169	-1.210671988	-0.945467566	4.431396532
H170	-3.355889654	-4.789872925	9.380827596
H171	-1.894081134	2.075663971	5.324458402
H172	-1.473587388	-0.239594411	9.189623715
H173	-1.757300711	3.189426889	9.539697861
H174	1.307380972	0.015303313	2.951972195
H175	0.278760287	1.035544968	3.995694102
H176	9.318676766	2.019200250	4.463666000
H177	-2.851130735	3.422556802	5.963491843
H178	-2.739521151	4.078055865	8.352194886
H179	-1.085423263	3.593350974	5.805092930
H180	-0.965914412	4.239047757	8.340009294
H181	-2.533935238	1.664437542	7.718650754
H182	0.443178571	2.259284069	7.300272063
H183	-1.271673225	0.073097888	6.416525036
H184	-0.379508802	0.332096780	2.507666539
H185	1.546303333	4.229801044	0.208506470
H186	0.748615972	4.502179124	1.780678634
H187	2.675699851	5.318942337	2.293654286
H188	3.220776123	4.680820208	4.834625271
H189	4.788192060	4.201324999	1.698356928
H190	6.620180248	2.564673714	1.359099984
H191	6.505322032	1.570162453	5.556320314
H192	-0.770386023	-2.543691667	6.399437344
H193	0.232085070	-5.583475250	6.440616531
H194	0.834616408	-4.985140128	8.001127311
H195	-1.854220907	-4.454322857	6.832712532
H196	-2.885179219	-3.222081006	10.085949683
H197	4.634538429	3.169361446	5.883621656
H198	1.699992120	-1.557171817	5.093243355
H199	-0.778929774	0.686294629	11.734567460
H200	-0.996150007	-1.024780047	11.287104162
H201	1.617924524	-0.743810975	10.452273957
H202	11.169149565	-7.029279846	4.012118937
H203	-1.732757224	-5.986484436	-4.113882337
H204	1.233716816	-6.014164027	-1.734733975
H205	-2.276037438	-6.237860999	-8.471713935
H206	-0.797908200	-5.360408599	-8.945626206
H207	-3.100525364	-4.356460424	-7.173723643
H208	-3.013182312	1.048082310	-9.714917526
H209	-3.338945434	-4.247546397	-9.697450636
H210	-3.326282429	-2.176585313	-2.300340482
H211	-3.930329722	-4.956308996	-1.158873051
H212	-2.251672114	-1.032407466	-8.430577452
H213	-5.087790678	-0.312366078	-8.750840190
H214	-1.030646244	-2.409672696	-9.597243275
H215	-1.297484810	-5.827034666	-2.414531320
H216	-1.584449023	-3.466070364	-7.317851547
H217	-1.193920200	-3.799796110	-10.465997566
H218	-6.724674494	-3.442752900	-2.877463656
H219	-3.454671061	-2.399108037	-0.548778813
H220	0.827174416	-5.289267971	-7.567501828
H221	-2.298315601	-4.316297261	-1.046478568
H222	-3.835028679	2.582466813	3.070466357
H223	-2.853871898	0.513886894	1.369465621
H224	-8.129550216	0.760368034	3.784489510
H225	-7.253817750	-0.074496232	1.051943622
H226	-6.925896813	4.077703491	2.374048859
H227	-8.332009736	8.742236582	4.439161218
H228	-6.861966808	5.245347532	5.327674306
H229	-8.913921379	5.448781057	3.468221618
H230	-6.253686677	6.834199366	2.759740631
H231	-10.821536262	9.538786944	1.107170620
H232	-3.246711251	4.208178811	1.266956253
H233	-4.792844636	3.915001910	0.462109026
H234	-9.816761669	10.456349416	3.268873464
H235	-8.371375708	5.639931076	1.799483854
H236	-6.177889731	2.011770271	1.366497245
H237	-7.018826961	-0.578240098	3.486482692
H238	-9.844406228	1.943268697	-0.235544205
H239	-2.100614862	1.511312637	-2.687884716
H240	-10.201141649	0.207273434	-0.175930085
H241	10.295163669	3.258660112	2.672765415
H242	8.686632133	3.343167771	2.398913203
H243	1.551519992	5.988961857	-1.431159356
H244	-0.714805997	5.478706013	0.268295161
H245	1.727295175	8.060406346	0.314697521
H246	-3.437083361	3.485605025	-1.619434892

	H247	-5.106679593	4.160593249	-2.794501644
	H248	-6.689372344	3.394596793	-4.199056179
	H249	-4.786907295	5.397271285	-1.844427308
	H250	-5.512065551	1.259184500	-4.091267780
	H251	-7.294250354	1.276481224	-3.893405321
	H252	-0.039925034	11.605840977	-1.845784594
	H253	-1.207883857	12.929981940	-1.674585594
	H254	-0.910761929	10.212265076	-3.343541792
	H255	-2.594116234	9.692935214	-3.334325233
	H256	-3.751768278	9.161556278	-6.070398708
	H257	-2.644880087	4.850545318	-5.753031330
	H258	-7.264526032	8.686259033	-0.569356269
	H259	-3.101425714	8.548799537	2.146354326
	H260	-4.665552778	7.781879409	-2.953666603
	H261	1.069855072	5.385848333	-3.894777232
	H262	2.341340259	7.206535567	-3.237405366
	H263	-1.386296855	4.874104720	-3.363466430
	H264	-8.347730853	-0.205715425	-1.496600255
	H265	3.647032697	10.007595874	-4.144742962
	H266	-6.063756887	-0.943702213	-3.085712072
	H267	0.539697841	6.368605878	-5.255781708
	H268	-3.394705243	6.964295183	0.285068594
	H269	3.346545051	10.303742486	-2.418686406
	H270	-3.039783993	7.720455909	-6.704429904
	H271	-5.110479977	8.935216505	-4.168103758
	H272	-5.155522014	-3.521921274	-3.449450371
	H273	-5.746313002	-3.327822746	-0.809188951
	H274	-6.916330756	10.356746423	1.227211955
	H275	-5.232980645	1.395029527	-1.622009886
	H276	-0.489303767	8.026351947	-3.707447704
	H277	-1.737549476	6.346529435	-6.003216865
	H278	-0.669105062	4.208034639	-4.821275045
	H279	-3.588219257	5.872014788	-3.671363490
	H280	0.790454132	8.906528502	2.946994278
	H281	-0.463205268	9.759527052	0.109087656
	H282	-0.079252660	12.624412619	0.402157471
	H283	2.266587479	9.422434027	2.095908050
	H284	-2.442223859	10.648755499	1.735955425
	H285	4.601284038	-3.029930089	-9.162942253
	H286	4.581167833	-4.478204444	-8.138852101
	H287	7.892987178	-5.214833790	-5.133134080
	H288	5.041847076	-5.114826894	-10.398205745
	H289	3.505352006	-5.596694892	-10.102399044
	H290	5.564503723	4.787322578	-0.932666980
	H291	5.793584615	7.477007488	0.502247842
	H292	4.832844793	8.095277748	-3.602513589
	H293	4.203626438	5.621737283	-0.111134522
	H294	6.836367751	8.096539297	-0.594052294
	H295	5.404881322	2.788611152	-6.835671869
	H296	5.983676832	6.753826774	-2.336098843
	H297	5.339364456	-7.036552793	4.989618489
	H298	7.080487370	-7.909961149	2.619403939
	H299	3.622670039	1.363504426	-6.857527115
	H300	5.110344881	-0.110008478	3.234543275
	H301	0.557123985	1.557648481	-8.712955823
	H302	-1.093277418	-2.105713538	-4.490632278
	H303	0.441262867	2.080270639	-7.028715426
	H304	4.312762215	-4.514285583	4.510388348
	H305	2.080472372	0.058465165	-6.595624701
	H306	3.977364843	-5.090962542	2.871104095
	H307	4.803451857	-7.319800004	3.316421408
	H308	6.463804627	-5.508291989	2.571931423
	H309	6.708950033	-5.096727305	4.276170031
	H310	-5.228953928	-0.791456192	-6.449070262
	H311	5.944564088	-9.496304378	4.885643051
	H312	4.974713484	-11.783353082	3.094377993
	H313	0.240277176	-0.023067685	-4.341137275
	H314	6.279083970	-11.866926001	5.704221370
	H315	4.716075693	-12.155910048	5.309704193
	H316	5.585424534	-3.357961542	2.055209043
	H317	-0.757689594	2.149047173	-4.998353773
	H318	-3.382141601	-2.049579332	-5.467791995
	H319	7.653873537	-9.929758688	-1.679981913
	H320	6.606096715	-7.617796713	-3.726550539
	H321	6.644344738	-8.161733393	-0.117642730
	H322	6.779877807	-10.519696281	-2.820661231
	H323	4.894058614	2.566991773	-9.567185807
	H324	6.376251501	-5.034651894	-0.228005284
	H325	2.314247060	-7.747995140	0.875633487
	H326	3.272930727	-1.992899145	-7.663228835

H327	3.038622630	-9.177128721	-0.832235339
H328	3.021912005	-6.890880847	-6.946042577
H329	2.216336999	-5.438899508	-5.034012224
H330	6.573218906	-12.445268928	3.456677611
H331	5.739011653	-1.075247494	1.930840378
H332	8.363402527	-5.799530360	0.802965378
H333	7.281999355	-3.779529093	-0.134640456
H334	8.983420007	-6.864642989	2.793898973
H335	3.691804512	-2.730183996	5.111028763
H336	3.754989363	-8.252129103	-3.662218621
H337	4.966577311	-8.555474571	-2.413184550
H338	1.904458536	-8.632895552	-2.076339010
H339	1.333361625	-2.128641370	-6.168326340
H340	-3.061622436	2.223343137	-5.983184916
H341	-5.172164705	0.985789851	-6.615980623
H342	2.334618605	-5.980951987	0.680875366
H343	8.518259734	-7.331463615	0.579494307
H344	7.412985643	-9.250516444	0.660642521
H345	4.120667046	-1.062096674	5.067770285
H346	5.489892794	-7.553629054	-6.686643051
H347	6.565069329	-5.969414606	-3.127355772
H348	0.454487567	-2.936941520	-7.499623019
H349	4.279013019	4.152679860	-7.025331488
H350	7.792076012	0.164178204	3.943367337
H351	7.823106013	0.573604820	2.213203831
H352	8.955450135	-6.856925764	-6.447072033
H353	8.195432266	-8.093387254	-5.690669374
H354	10.306198338	-8.566829397	-1.680240317
H355	8.576780455	-8.260105653	-3.131814192
H356	11.708470537	-7.564538853	-3.838916749
H357	10.173157580	-6.843854688	-1.278801779
H358	10.564863028	0.358647691	2.183928846
H359	-2.982387411	13.208438326	3.719015733
H360	10.242822439	-6.871317522	5.526647801
H361	9.529111440	-4.458863571	4.398406801
H362	-4.707310420	12.315387878	1.402158244
H363	-4.092882636	10.301949092	3.652953388
H364	-5.682640821	10.997260562	3.289661738
H365	1.131414247	-3.322739658	-1.006942279
H366	3.201077773	-0.891489902	0.527370329
H367	-6.433846396	6.807224788	-1.571559468
H368	0.507855955	-1.484264842	0.206826829
H369	-1.661340668	-0.155325484	-0.858861825
Mo370	3.828541784	-4.345581851	-2.363114236
N371	-6.947493996	-7.161888660	-0.320830536
N372	-8.782644405	-4.638205186	1.747472650
N373	-8.178217273	-4.949905933	4.476650315
N374	-5.499410315	-5.808664752	4.493608724
N375	-4.233134023	-3.409721626	3.651419061
N376	-4.894363516	-1.778222702	5.921252278
N377	-3.683651014	-3.135326446	8.159446251
N378	-1.115797899	-4.410532930	7.542655231
N379	0.214890106	-2.487789540	6.148311214
N380	-0.377738337	0.423262801	6.769934147
N381	-0.685622313	0.216826316	9.656241880
N382	5.507856116	-11.540639510	5.117491737
N383	6.406100711	-9.286963489	3.994314696
N384	5.100499450	-3.329007390	2.955921619
N385	4.284323250	-1.997734198	4.705751044
N386	5.357120741	-1.031894595	2.886583301
N387	9.061449354	-6.982882930	3.828213399
N388	-5.836892696	-2.938275761	-2.928412183
N389	-1.927195593	-5.591523376	-3.187878583
N390	-6.067698941	-0.437721255	-2.184567108
N391	-8.338518014	0.666574716	-0.971978736
N392	-7.842853392	0.348477078	1.777703872
N393	-5.695403570	2.080353469	2.266711982
N394	-3.102798830	2.629237407	-1.113047695
N395	-2.313791596	0.653799180	-0.720364265
N396	-6.311129919	4.725360960	2.861112066
N397	-9.739890225	7.737466982	1.593299882
N398	-9.649993392	9.518339258	2.913907431
N399	9.481325575	2.697911978	2.408835558
N400	6.337288739	7.241194866	-0.334272233
N401	4.211261476	8.449115901	-2.880173969
N402	1.552177503	7.850656226	-3.345672105
N403	-3.167135267	6.400880067	-4.457833309
N404	-3.371102279	8.236462679	-5.896457941
N405	-4.446160478	8.237941806	-3.842959317
N406	0.649661067	6.333798100	-1.069158534

	N407	0.968376641	8.215600023	0.984574653
	N408	-0.365768502	10.610434105	0.662292602
	N409	-1.884444105	10.366942314	-3.054010246
	N410	-2.748688428	11.618102254	1.709492387
	N411	4.093619710	-4.769193948	-10.235957379
	N412	2.458772543	-2.569693526	-7.885839910
	N413	1.406506978	0.237053395	-7.357161894
	N414	3.487220428	2.221305820	-7.400781588
	N415	-1.712554293	-3.009875506	-10.076450936
	N416	-3.264202175	-1.180310636	-8.479731588
	N417	8.083704223	-7.115450955	-5.975984965
	N418	4.235911322	-5.638527030	-4.292897160
	N419	3.624424805	-6.572747887	-6.191606259
	N420	9.235753152	-7.477799680	-3.083703990
	N421	0.045598775	2.239333998	0.058357640
	N422	2.262184694	-0.819939307	0.103761092
	N423	0.797496274	1.585347493	-0.471807550
	N424	1.448378473	-1.756714796	0.572441487
	O425	-6.554629978	-5.098457600	1.659077139
	O426	-7.312015495	-2.840040731	4.512341080
	O427	-5.355335497	-5.227547831	6.697401290
	O428	-2.488877790	-4.036979705	4.974216742
	O429	-3.020301237	-0.584243612	5.453888344
	O430	-3.282628402	-1.048771833	9.011698584
	O431	-0.560855831	-4.058409874	9.738725013
	O432	2.163540095	-3.714899605	6.064715604
	O433	1.903669899	0.345366201	6.618852249
	O434	0.836437051	1.900648301	9.713107023
	O435	1.522587721	-0.299789973	12.440368468
	O436	7.051998525	-10.336297837	2.038235161
	O437	8.060923654	-7.728711694	5.760297274
	O438	11.561925731	-4.509275935	4.551086021
	O439	-5.630783698	-0.839760343	0.030127305
	O440	-3.693079693	-4.421003677	-4.013890608
	O441	-7.111518934	2.511323106	-0.417885890
	O442	-6.329897827	3.050917482	-3.361744833
	O443	-9.766574416	1.500493864	2.268081756
	O444	-5.624270030	1.460367577	4.465651418
	O445	-4.228334967	5.037178607	3.744949569
	O446	-6.534227689	7.260185213	5.328186167
	O447	10.809523769	-0.039840613	4.171303733
	O448	3.567465743	4.694090281	3.923497294
	O449	3.616515656	8.366677979	-0.681087981
	O450	0.994652245	10.076001433	-3.531267679
	O451	-1.415581934	7.191157844	-1.539416055
	O452	-0.693888124	7.161872377	2.166676474
	O453	2.442121832	5.672799663	1.411376243
	O454	0.857896973	11.603062941	2.325026348
	O455	-2.502960802	13.587563473	0.587364189
	O456	-3.255177102	11.021642160	-1.345663098
	O457	-4.959293470	13.706983670	3.619428745
	O458	-5.489569627	6.834596917	-1.322736179
	O459	1.754362108	-4.577566528	-8.747617063
	O460	-0.253080893	-0.747214893	-8.594516331
	O461	2.115479783	3.613037835	-8.563638542
	O462	5.772428801	4.366719753	-9.176687412
	O463	-4.985981969	-2.621947588	-8.871227862
	O464	-1.462701907	-6.624958057	-6.009146941
	O465	0.456833170	-5.773288337	-6.766191861
	O466	-4.000255694	2.323412215	-8.470297057
	O467	9.910821215	-5.404814253	-3.702293336
	O468	12.663939808	-6.999860534	-2.125562238
	O469	0.287345916	-6.246816162	-1.260093477
	O470	-0.233648396	-7.374300280	0.613204976
	O471	5.133253484	-10.858176466	-3.330454437
	O472	2.872529153	-10.909595622	-3.297302157
	O473	5.090893391	-5.825951192	-1.348836737
	O474	5.040727721	-7.496503557	0.152143726
	O475	2.587368269	-6.050885485	-2.024767147
	O476	8.954877559	-6.556660616	1.031460935
	O477	6.990144575	-4.552993710	0.383466582
	O478	-4.372370061	4.740890182	-2.456384155
	O479	7.734816870	-10.420914580	-2.528130750
	O480	7.522364819	-8.627276871	-0.102083050
	S481	3.575811605	4.939586063	-2.338501148
	S482	1.971237831	1.798622272	-3.319345436
	S483	1.966444802	-3.284573057	-3.357774310
	S484	3.724210673	1.767587017	-0.287788787
	S485	0.336718310	-0.941226282	-1.920146313
	S486	3.962331914	-0.711985053	-5.524904678

	S487 3.640761321 -3.490207689 -0.194385371<br>S488 5.598369592 1.757079635 -3.150806865<br>S489 5.654436117 -3.068537392 -3.192843066<br>S490 6.301388023 -0.761374559 -0.338778314<br>end

47, S=3/2

reactant

Fe(139) 2.323<br>Fe(140) 0.829<br>Fe(141) 1.454<br>Fe(142) -2.433<br>Fe(143) 2.902<br>Fe(144) -0.986<br>Fe(145) -1.531	bm52capexp347tf.car_6<br>C1 -7.734132254 -5.365012870 1.276604324<br>C2 -8.135095861 -6.509447875 0.337697222<br>C3 -8.542266679 -3.434833417 2.564909518<br>C4 -7.904586471 -3.695017532 3.925772290<br>C5 -7.472554345 -5.284144170 5.734509489<br>C6 -5.947916775 -5.405031596 5.666779587<br>C7 -3.974526188 -5.711226394 4.272326340<br>C8 -3.463373429 -4.269210762 4.307144548<br>C9 -3.885620162 -1.950437432 3.606634116<br>C10 -3.901796370 -1.318888201 5.007921171<br>C11 -4.919983835 -1.157099533 7.188735644<br>C12 -3.858294284 -1.708688511 8.151596358<br>C13 -2.853486602 -3.724262863 9.129149260<br>C14 -1.385905188 -4.041750987 8.806375371<br>C15 0.241012621 -4.801885830 7.120788103<br>C16 0.982416258 -3.653212613 6.426467315<br>C17 0.644416790 -1.415925815 5.389442684<br>C18 0.775525275 -0.201278523 6.324885437<br>C19 -0.264658075 -1.208764723 4.160292876<br>C20 -0.036905604 0.146219692 3.472912911<br>C21 -0.136328042 -2.410321624 3.213774049<br>C22 -0.357769944 1.551163263 7.617731148<br>C23 0.008562942 1.227270525 9.077618505<br>C24 -1.686391070 2.332687168 7.481501753<br>C25 -1.751986824 3.500047822 8.471891701<br>C26 -1.865845570 2.842350687 6.044621656<br>C27 -0.404814848 -0.087708881 11.070366157<br>C28 1.060151879 -0.355847498 11.351400638<br>C29 5.897438758 -11.599477165 3.752826448<br>C30 6.515109272 -10.322149756 3.194306265<br>C31 6.786739860 -7.895604748 3.681069787<br>C32 8.007947931 -7.501893869 4.531792055<br>C33 5.607863757 -6.938593258 3.920726303<br>C34 5.942207087 -5.497114713 3.547990804<br>C35 4.698287110 -4.614084078 3.497846122<br>C36 4.896158159 -2.113339938 3.511592423<br>C37 10.268917503 -6.559342420 4.484984470<br>C38 10.493878240 -5.064815543 4.471899230<br>C39 -5.329820364 -2.675253566 -1.562613377<br>C40 -5.692860319 -1.241572292 -1.128322278<br>C41 -3.791583510 -2.821571234 -1.504547471<br>C42 -3.244071881 -4.259171157 -1.621370396<br>C43 -2.976988784 -4.746658587 -3.046282312<br>C44 -6.183862568 0.997988729 -2.033538777<br>C45 -7.273827425 1.452982777 -1.052564252<br>C46 -6.361075868 1.606883985 -3.430021444<br>C47 -9.427898140 0.930348014 -0.006368156<br>C48 -9.030699668 0.950428247 1.472806420<br>C49 -7.325362296 0.439000544 3.155712533<br>C50 -6.155049882 1.401097318 3.366966910<br>C51 -4.605098204 3.045594005 2.433939223<br>C52 -5.046854949 4.354197080 3.130952169<br>C53 -4.020209484 3.480098665 1.081377156<br>C54 -3.402965453 2.417824599 0.234845671<br>C55 -2.981172767 1.133960065 0.469933970<br>C56 -2.547690903 1.605324815 -1.665338245<br>C57 -6.849273214 6.033666756 3.239678770<br>C58 -6.755909664 6.189682711 4.756428035<br>C59 -8.322537089 6.088922064 2.772004571<br>C60 -8.945267436 7.449626443 2.657513575<br>C61 -8.871846814 8.553007491 3.485323793<br>C62 -10.198693907 8.969697737 1.760179309<br>C63 9.237686775 1.628910296 3.433849452

C64	10.269303793	0.527581377	3.265961846
C65	7.845389819	0.982658597	3.250750780
C66	6.709668151	1.956636238	3.461767442
C67	6.139784088	2.155645915	4.731869500
C68	6.191397600	2.699411147	2.385384554
C69	5.087851758	3.059368520	4.924798834
C70	5.156803308	3.617619937	2.564950327
C71	4.598311884	3.789521948	3.834032190
C72	5.383191279	6.858685926	-1.385085721
C73	4.324989542	7.959208293	-1.597045966
C74	4.698852697	5.531125550	-1.098262093
C75	3.314160314	9.544044228	-3.201954322
C76	1.839594791	9.161968384	-3.365236751
C77	0.216065770	7.270657545	-3.328513642
C78	-0.253169829	6.927332490	-1.890722879
C79	0.245003373	6.024673038	-4.242902343
C80	-1.008079362	5.147104815	-4.349479383
C81	-2.152316458	5.682633518	-5.226592316
C82	-3.673260666	7.602827886	-4.743899390
C83	0.268942385	5.982485670	0.306372589
C84	0.115119622	7.202671269	1.225957410
C85	1.265350621	5.013469963	0.975877320
C86	1.199353237	9.267336187	1.966153954
C87	0.555887218	10.610767544	1.657731085
C88	-0.855685449	11.875464793	0.127184267
C89	-2.130072045	12.430592283	0.810150004
C90	-1.016197506	11.883580059	-1.395343550
C91	-2.159569859	11.012427401	-1.899336108
C92	-4.073956176	11.948219387	2.222095369
C93	-3.987108932	13.049918640	3.270989674
C94	-4.731361174	10.680565451	2.835046187
C95	-4.972190027	9.607000122	1.798443365
C96	-6.154531881	9.602678048	1.042679860
C97	-4.006061335	8.626393081	1.520663033
C98	-6.360069336	8.666298447	0.028020718
C99	-4.182222166	7.697152038	0.490687385
C100	-5.359638504	7.734431240	-0.250435150
C101	4.086761212	-3.979197898	-8.998739705
C102	2.660105600	-3.726271740	-8.544102819
C103	1.204285542	-2.191311682	-7.242924338
C104	0.707097328	-0.834022967	-7.754558396
C105	1.122611543	1.587715882	-7.711900278
C106	2.296165926	2.542446861	-7.913440391
C107	4.575760538	3.213399525	-7.404814295
C108	5.169628498	3.373799853	-8.789356960
C109	-2.761123087	-3.492841836	-9.150223984
C110	-3.787153112	-2.402996715	-8.816533210
C111	-2.270604897	-4.170100407	-7.849189053
C112	-1.538112897	-5.490303357	-8.150473374
C113	-0.860099292	-6.019182701	-6.906957997
C114	-4.191700907	-0.084983649	-8.157008905
C115	-3.713913388	1.199562033	-8.804585987
C116	-4.569408299	0.045820740	-6.660166883
C117	-3.334177572	0.058411715	-5.805593412
C118	-2.607642526	1.245799583	-5.610187954
C119	-2.798045732	-1.153073672	-5.339158204
C120	-1.328521151	1.199621888	-5.046967464
C121	-1.520808387	-1.197900945	-4.774076507
C122	-0.769255634	-0.024008926	-4.667636959
C123	7.914975220	-6.235921298	-4.770929301
C124	9.096912089	-6.322801127	-3.794110912
C125	6.602924794	-6.605129414	-4.066380631
C126	5.351455459	-6.397397417	-4.853830250
C127	4.996199944	-6.986501931	-6.050137220
C128	3.261911801	-5.876922584	-5.238666527
C129	10.307927783	-7.550477505	-2.093392706
C130	11.658760312	-7.321966256	-2.734924833
C131	0.601453562	-6.925198813	-0.196784370
C132	2.092256707	-6.968840807	0.121029270
C133	3.011757127	-7.093483184	-1.132748949
C134	2.921335994	-8.534682529	-1.700373720
C135	3.954494311	-8.875327210	-2.776732727
C136	3.978834253	-10.365132834	-3.179894983
C137	4.467741997	-6.827072245	-0.710496413
C138	3.588716262	-0.695288962	-2.229729898
Fe139	3.876336923	-2.025436924	-3.720688680
Fe140	2.222662579	0.818331092	-1.518946186
Fe141	5.015746138	0.430307490	-1.635596709
Fe142	3.798116639	0.516151440	-3.838420559
Fe143	3.791597206	2.786236903	-2.488509547

Fe144	4.680500200	-2.055097214	-1.277713957
Fe145	2.163085956	-2.417726232	-1.752547046
H146	-6.370108375	-7.533002531	0.332357210
H147	-6.548548847	-6.539547393	-0.950245677
H148	-9.705256833	-4.783381044	1.389267487
H149	-8.851849077	-6.119634962	-0.403014371
H150	-8.695405760	-7.239201765	0.947302023
H151	-7.881923365	-2.727636826	2.041847003
H152	-8.655826823	-5.599457795	3.964906408
H153	-9.506458834	-2.935170931	2.727607164
H154	-7.891098246	-6.243308434	6.065691684
H155	-6.014987045	-5.841236180	3.651954463
H156	-7.691449498	-4.531291041	6.502798451
H157	-2.889721536	-1.761117121	3.185100487
H158	-4.977665544	-3.701489760	3.034493242
H159	-3.732824791	-6.171849720	3.305123593
H160	-4.768889694	-0.071044817	7.136447001
H161	-5.653834117	-2.290225737	5.503390768
H162	-3.452931733	-6.263996653	5.062238776
H163	-4.624725731	-1.445961449	2.968197441
H164	-4.207383828	-3.622517121	7.474281657
H165	-5.911867954	-1.354459356	7.619088368
H166	-0.522110707	-3.325640622	3.681474709
H167	-0.699470669	-2.247238147	2.281522634
H168	0.909218729	-2.589797995	2.927849769
H169	-1.292923914	-1.189789340	4.542104244
H170	-3.352018340	-4.672259716	9.380852146
H171	-1.870748782	2.025428246	5.311075858
H172	-1.449541786	-0.255165337	9.215969032
H173	-1.706970700	3.166176932	9.518264033
H174	0.757817255	0.110285263	2.728176054
H175	0.224455283	0.942382578	4.184321632
H176	9.311671102	1.987601663	4.478578296
H177	-2.821048703	3.378057612	5.946362357
H178	-2.693760729	4.050239947	8.330755294
H179	-1.055180928	3.542575121	5.783228559
H180	-0.919389346	4.201465116	8.303423709
H181	-2.510642858	1.633416672	7.712312273
H182	0.469708383	2.186661609	7.267026039
H183	-1.279100378	0.037068529	6.403079767
H184	-0.957201232	0.466111569	2.960423220
H185	1.557794621	4.248956254	0.242217907
H186	0.730054169	4.519308174	1.799920918
H187	2.665700649	5.321491900	2.329383862
H188	3.214132007	4.680580239	4.864476315
H189	4.759762351	4.177736904	1.718232975
H190	6.589183005	2.540220866	1.380413537
H191	6.514606125	1.588784688	5.588798281
H192	-0.789782738	-2.680707952	6.314305877
H193	0.220010076	-5.650388128	6.421877116
H194	0.833322036	-5.100194341	7.994946250
H195	-1.855537935	-4.538997676	6.840400642
H196	-2.836696628	-3.093945609	10.026206782
H197	4.645178428	3.188928772	5.916405760
H198	1.670165437	-1.623686683	5.061824106
H199	-0.750692759	0.717122935	11.739474441
H200	-0.957585835	-1.003529806	11.330958664
H201	1.649398263	-0.728841654	10.481865329
H202	11.143243622	-7.060119971	4.042069202
H203	-1.753528603	-5.984933742	-4.123916350
H204	1.241612940	-6.065216176	-1.805170829
H205	-2.268888078	-6.240824620	-8.485128197
H206	-0.802366869	-5.342199866	-8.953983117
H207	-3.121986586	-4.377215219	-7.182292988
H208	-3.109453652	1.047398843	-9.729863709
H209	-3.348899848	-4.237815876	-9.708160442
H210	-3.349752386	-2.193930269	-2.297161902
H211	-3.950740254	-4.971949038	-1.162695010
H212	-2.279314031	-1.026650431	-8.428005352
H213	-5.130657996	-0.319821620	-8.700603991
H214	-1.039895919	-2.403270159	-9.564228203
H215	-1.318593246	-5.851022320	-2.423405933
H216	-1.613527402	-3.471314121	-7.307931804
H217	-1.210037610	-3.770401836	-10.466589027
H218	-6.739894408	-3.458688420	-2.853214958
H219	-3.464825512	-2.414975519	-0.535702085
H220	0.831553624	-5.290990469	-7.589031153
H221	-2.317467562	-4.340271980	-1.034974613
H222	-3.831702692	2.605613479	3.079375548
H223	-3.016645194	0.523686127	1.363177043

H224	-8.139921298	0.779831264	3.809460270
H225	-7.263967746	-0.073101237	1.083316459
H226	-6.928999047	4.082481369	2.361327466
H227	-8.325181758	8.742316894	4.401625676
H228	-6.907725894	5.234236485	5.315752152
H229	-8.930493947	5.431509353	3.419011481
H230	-6.266302175	6.837883970	2.763949807
H231	-10.875071173	9.517915148	1.110140313
H232	-3.259298719	4.248537108	1.292228420
H233	-4.813396977	3.970349090	0.494272182
H234	-9.835270441	10.447667040	3.250136432
H235	-8.360260050	5.647428421	1.763480782
H236	-6.173381900	2.015352485	1.375568463
H237	-7.018005248	-0.551381003	3.523466891
H238	-9.871673876	1.913900871	-0.215313513
H239	-2.205334134	1.505556436	-2.693082581
H240	-10.212089415	0.174699341	-0.143892716
H241	10.297668266	3.214054909	2.687169062
H242	8.690116418	3.315169691	2.412311521
H243	1.554286960	5.989011630	-1.430900002
H244	-0.715914117	5.494138574	0.278802977
H245	1.734947298	8.069534251	0.308708446
H246	-3.435522374	3.543414277	-1.608842997
H247	-5.111367255	4.158521897	-2.786004794
H248	-6.710586643	3.392898280	-4.162947612
H249	-4.796453477	5.409375029	-1.851220661
H250	-5.536263025	1.249199120	-4.068115541
H251	-7.316820690	1.276998339	-3.865330568
H252	-0.062242722	11.614029697	-1.869586994
H253	-1.252059850	12.918859364	-1.687701336
H254	-0.909492632	10.171478018	-3.320942117
H255	-2.583622453	9.616353530	-3.289585748
H256	-3.681398308	9.204582112	-6.027434988
H257	-2.646946364	4.849719047	-5.749060270
H258	-7.292595025	8.666074689	-0.542439791
H259	-3.090645697	8.564373455	2.114442280
H260	-4.698004508	7.766313906	-2.963327929
H261	1.064917517	5.379876350	-3.888302561
H262	2.344307729	7.189594368	-3.219185352
H263	-1.391803215	4.883406231	-3.351642539
H264	-8.366340394	-0.219566338	-1.477714492
H265	3.646377695	10.004391032	-4.142106393
H266	-6.094306217	-0.954868216	-3.059969298
H267	0.537359736	6.362530829	-5.250644436
H268	-3.390256950	6.986302654	0.251169612
H269	3.342261111	10.317069733	-2.420623761
H270	-2.933921753	7.775441767	-6.662986224
H271	-5.102223542	8.947694174	-4.163847683
H272	-5.173343100	-3.518063469	-3.435743653
H273	-5.758370111	-3.336333666	-0.786573294
H274	-6.936005810	10.337041360	1.251387743
H275	-5.255620791	1.391629602	-1.595505528
H276	-0.482917275	8.025404482	-3.701586893
H277	-1.737135809	6.342301992	-5.998832133
H278	-0.675736284	4.204611370	-4.806679569
H279	-3.600672491	5.871060536	-3.677353830
H280	0.809983919	8.928943446	2.938000501
H281	-0.459002870	9.759954457	0.098563917
H282	-0.089037383	12.625793359	0.375195304
H283	2.277748454	9.445622383	2.071877320
H284	-2.445580022	10.650775954	1.729587584
H285	4.606680398	-3.021548275	-9.146735517
H286	4.585851689	-4.482931217	-8.143519709
H287	7.872602586	-5.180014797	-5.076947712
H288	5.048126918	-5.085614490	-10.412756487
H289	3.510284555	-5.573286565	-10.124122727
H290	5.466682107	4.758510109	-0.972449698
H291	5.727504982	7.442320203	0.543829885
H292	4.825793295	8.083993158	-3.587093679
H293	4.100586947	5.605621503	-0.180107165
H294	6.810887275	8.052453980	-0.514488032
H295	5.377187343	2.863039429	-6.736798192
H296	5.991842430	6.757859346	-2.297302574
H297	5.298598175	-7.007062793	4.976891794
H298	7.052740462	-7.881095696	2.616267017
H299	3.633650208	1.400495130	-6.786983973
H300	5.143820759	-0.098005707	3.230958763
H301	0.557065026	1.554662600	-8.651250991
H302	-1.101513111	-2.148568266	-4.436344555
H303	0.465361376	2.062473168	-6.962229931

H304	4.265826111	-4.489650086	4.497467867
H305	2.135485067	0.030995940	-6.592214486
H306	3.924847844	-5.077134890	2.864546647
H307	4.769503908	-7.298374335	3.302816120
H308	6.415745554	-5.477959615	2.555429778
H309	6.662824359	-5.062835032	4.258597509
H310	-5.215230408	-0.805714270	-6.401759732
H311	5.927315185	-9.454776118	4.898063561
H312	4.985580267	-11.777405613	3.162572277
H313	0.245403483	-0.043813415	-4.278279896
H314	6.329091831	-11.788676759	5.757480407
H315	4.772635628	-12.139015417	5.389669000
H316	5.524548205	-3.340675547	2.032226436
H317	-0.744013772	2.111620960	-4.914334356
H318	-3.371442831	-2.076591540	-5.443574114
H319	7.621784007	-9.938440780	-1.668589638
H320	6.635246842	-7.669604437	-3.777159087
H321	6.624244566	-8.179361146	-0.104758487
H322	6.758480361	-10.542051865	-2.814292982
H323	4.943639785	2.542115983	-9.498915908
H324	6.348989282	-5.057615138	-0.215980777
H325	2.287436627	-7.785554471	0.827066854
H326	3.278926083	-2.017328339	-7.601961058
H327	3.050186771	-9.221602845	-0.848908073
H328	3.096182403	-6.959834528	-7.039057022
H329	2.249153395	-5.506964303	-5.152078602
H330	6.594069624	-12.421313622	3.514627777
H331	5.758680909	-1.074605790	1.941219304
H332	8.363166932	-5.802190927	0.795428289
H333	7.232893061	-3.786590479	-0.137129341
H334	8.973531686	-6.855134431	2.797414549
H335	3.720909254	-2.709304602	5.136336750
H336	3.743326718	-8.289952589	-3.684698873
H337	4.962914332	-8.595202325	-2.443197749
H338	1.903983843	-8.691139350	-2.091152235
H339	1.338901958	-2.161597345	-6.149639759
H340	-3.029839962	2.194354144	-5.950409586
H341	-5.153566498	0.972071593	-6.553237238
H342	2.316247830	-6.020909685	0.638736291
H343	8.506577654	-7.336867313	0.582794386
H344	7.407974161	-9.256135795	0.675790487
H345	4.162381478	-1.041300632	5.094770150
H346	5.558463666	-7.618846792	-6.725341032
H347	6.528781458	-6.041641905	-3.128664946
H348	0.455777948	-2.945663683	-7.493250506
H349	4.246961837	4.197962915	-7.039038262
H350	7.765972327	0.149657560	3.964733300
H351	7.796880051	0.560790035	2.234571051
H352	8.997031086	-6.735475216	-6.440168339
H353	8.269579905	-8.023173935	-5.738227267
H354	10.307162062	-8.571821122	-1.681864683
H355	8.553900023	-8.229784704	-3.098717352
H356	11.699367400	-7.550391624	-3.827329248
H357	10.174344362	-6.855544761	-1.248956272
H358	10.544482350	0.316109125	2.204310828
H359	-2.978342818	13.194087223	3.724567171
H360	10.202836583	-6.867650261	5.543407671
H361	9.568131932	-4.444765306	4.391763229
H362	-4.711585076	12.321055776	1.403147783
H363	-4.090203920	10.301018781	3.646063706
H364	-5.681885547	10.995245646	3.289595401
H365	1.089117796	-3.227609340	-1.028340495
H366	4.387534249	-1.536404524	0.084026807
H367	-6.454854748	6.805725569	-1.567471775
H368	0.102261590	-0.953739253	-0.540547641
H369	-2.015541639	-0.243430412	-0.871194992
Mo370	3.846050045	-4.400884148	-2.432238646
N371	-7.054218978	-7.193255413	-0.347932514
N372	-8.772224574	-4.624006281	1.761575979
N373	-8.095701126	-4.918745939	4.472336017
N374	-5.415403380	-5.778984388	4.476228729
N375	-4.188244191	-3.373564404	3.588092552
N376	-4.871530606	-1.732211895	5.855825230
N377	-3.639170540	-3.049373031	8.103863834
N378	-1.115268895	-4.492026826	7.546607833
N379	0.208514187	-2.583222454	6.139111841
N380	-0.381875465	0.349373004	6.782077290
N381	-0.656672954	0.204050925	9.671608608
N382	5.539704892	-11.500122054	5.174690278
N383	6.381948599	-9.252583175	4.001537629

N384	5.031998225	-3.307060063	2.928599388
N385	4.285817810	-1.971768243	4.705386538
N386	5.399195869	-1.024271447	2.900951614
N387	9.039172569	-6.963910531	3.833712367
N388	-5.857003525	-2.946773208	-2.905007139
N389	-1.949359287	-5.601824964	-3.192728822
N390	-6.079502700	-0.445260600	-2.160178152
N391	-8.353314229	0.645822489	-0.942277184
N392	-7.852796299	0.351802777	1.807072137
N393	-5.689714132	2.084548800	2.275530082
N394	-3.121677650	2.676442549	-1.101012607
N395	-2.466992114	0.659580812	-0.721734110
N396	-6.322346292	4.729796971	2.858562762
N397	-9.777330318	7.722816590	1.583508071
N398	-9.672109558	9.508690742	2.896187394
N399	9.477882016	2.661732993	2.423738834
N400	6.291980162	7.201264414	-0.277545890
N401	4.227239631	8.467730041	-2.860469684
N402	1.560152911	7.842281066	-3.341318767
N403	-3.173073746	6.401324748	-4.457768963
N404	-3.357569929	8.254615768	-5.876998728
N405	-4.453431930	8.236148036	-3.838501262
N406	0.652380264	6.332443994	-1.063115957
N407	0.974790522	8.230593838	0.976082309
N408	-0.367842260	10.613262752	0.649017686
N409	-1.886831895	10.313956420	-3.035602393
N410	-2.752542917	11.619725840	1.702152828
N411	4.098842799	-4.744286760	-10.246303752
N412	2.462211261	-2.574306874	-7.870535306
N413	1.430520619	0.223174831	-7.322673779
N414	3.496362496	2.240361960	-7.356276553
N415	-1.724703870	-2.987277646	-10.059059687
N416	-3.291754869	-1.178342039	-8.474795720
N417	8.129761781	-7.039240791	-5.988306480
N418	4.254293218	-5.691194007	-4.367375799
N419	3.680576497	-6.644695125	-6.269483386
N420	9.226670294	-7.459791439	-3.058266531
N421	0.120119513	2.228528301	-0.052023178
N422	2.638203499	-0.443579139	1.460337651
N423	0.968244466	1.722062424	-0.604638901
N424	1.958572841	-1.297041250	1.238619805
O425	-6.559295229	-5.141535252	1.618692908
O426	-7.244194471	-2.802946637	4.486285212
O427	-5.256185221	-5.178528832	6.673154007
O428	-2.466782457	-3.942321462	4.969028798
O429	-3.055866296	-0.471847204	5.333171132
O430	-3.285811792	-0.951847033	8.955275348
O431	-0.523643867	-3.935768071	9.687103841
O432	2.192597779	-3.752953033	6.149832654
O433	1.896997827	0.226304729	6.641302185
O434	0.872775486	1.882252627	9.684361509
O435	1.559934462	-0.238608749	12.459773875
O436	7.056874300	-10.330260268	2.070339891
O437	8.008085945	-7.669285412	5.764478839
O438	11.597568317	-4.549737387	4.558741195
O439	-5.615970490	-0.844605996	0.051009621
O440	-3.691977018	-4.390345391	-4.011654680
O441	-7.135027192	2.497736135	-0.391552421
O442	-6.339261797	3.044551189	-3.332735263
O443	-9.786099446	1.490148251	2.291602978
O444	-5.663195182	1.530116226	4.491569478
O445	-4.258503025	5.041030948	3.786061686
O446	-6.582807192	7.249888392	5.332302151
O447	10.761762553	-0.100356999	4.192098599
O448	3.551359977	4.684262718	3.949776704
O449	3.607128616	8.374150763	-0.669849791
O450	0.994147486	10.065124928	-3.518931208
O451	-1.412905256	7.189773620	-1.535188555
O452	-0.697680623	7.195322171	2.159965502
O453	2.422917905	5.698766814	1.459189887
O454	0.856562243	11.623042587	2.301596771
O455	-2.508951576	13.587025287	0.576450369
O456	-3.268100064	10.992120377	-1.345282469
O457	-4.950099493	13.711903310	3.622199064
O458	-5.508749534	6.834666520	-1.325862927
O459	1.755972601	-4.568614536	-8.765328490
O460	-0.276432007	-0.734504556	-8.516999468
O461	2.107365342	3.603059576	-8.532193901
O462	5.875368402	4.315976672	-9.109630266
O463	-5.007323197	-2.634533173	-8.838028521

	O464	-1.460524243	-6.605772976	-6.012816078
	O465	0.460431943	-5.775425118	-6.787550346
	O466	-3.996095510	2.322620730	-8.409755536
	O467	9.938114722	-5.407131893	-3.704957120
	O468	12.657173608	-6.982679658	-2.116220852
	O469	0.288960062	-6.285443343	-1.330991400
	O470	-0.258254441	-7.388103988	0.551397286
	O471	5.130791982	-10.901885976	-3.350702777
	O472	2.869570410	-10.947445139	-3.343021842
	O473	5.078048233	-5.837586741	-1.307324274
	O474	5.016233481	-7.548078653	0.145885211
	O475	2.603999264	-6.117775629	-2.095282363
	O476	8.949562586	-6.562425964	1.030044811
	O477	6.972262779	-4.569761517	0.384267556
	O478	-4.378609879	4.744753800	-2.453670305
	O479	7.709897384	-10.435540120	-2.512174018
	O480	7.508717229	-8.635439195	-0.089704775
	S481	3.572761615	5.057574482	-2.484918746
	S482	2.066169851	1.842257751	-3.556125732
	S483	2.090686095	-3.393983615	-3.692967203
	S484	3.909830603	1.855219391	-0.432860388
	S485	0.591015352	-0.829106802	-1.807451302
	S486	4.067438019	-0.812757698	-5.567011952
	S487	3.374510284	-3.513143679	-0.295284463
	S488	5.610110379	1.769065281	-3.315198010
	S489	5.720579017	-3.090061712	-3.048150312
	S490	6.413409653	-0.779413139	-0.560934812
	end			

TS

	bm52capexp347tg_1_53249.10			
Fe(139) 2.318	C1	-7.721612170	-5.357528774	1.267310014
Fe(140) -0.328	C2	-8.143776791	-6.501647436	0.336778067
Fe(141) 2.595	C3	-8.513860167	-3.421330352	2.559347949
Fe(142) -2.214	C4	-7.868652602	-3.680697863	3.916300973
Fe(143) 2.184	C5	-7.435804518	-5.260737729	5.732036158
Fe(144) -1.858	C6	-5.910978401	-5.378881529	5.665935805
Fe(145) -0.101	C7	-3.935406106	-5.691247981	4.275886762
	C8	-3.430509898	-4.246574557	4.296123878
	C9	-3.867251474	-1.935395820	3.579414012
	C10	-3.881934789	-1.296582140	4.978059046
	C11	-4.897487833	-1.130485039	7.161674075
	C12	-3.847680652	-1.680522237	8.137733270
	C13	-2.840662688	-3.696284704	9.117662159
	C14	-1.375358103	-4.022097226	8.791080296
	C15	0.232176680	-4.825838668	7.108936665
	C16	0.967487577	-3.667196094	6.424002184
	C17	0.623186168	-1.445274949	5.367362985
	C18	0.760057649	-0.230040454	6.299783914
	C19	-0.259789394	-1.241348589	4.117031540
	C20	-0.032097030	0.114249054	3.420534406
	C21	-0.078445511	-2.417550315	3.150654129
	C22	-0.351717429	1.530174025	7.597391383
	C23	0.023577950	1.212045583	9.057416163
	C24	-1.677492529	2.317337850	7.468120311
	C25	-1.736784137	3.484076473	8.459605129
	C26	-1.861086659	2.826996316	6.031850730
	C27	-0.395963445	-0.087353290	11.064691618
	C28	1.070686894	-0.348556568	11.343994132
	C29	5.896933236	-11.584190282	3.760922184
	C30	6.515431059	-10.311645008	3.196085262
	C31	6.779837863	-7.883670941	3.678088404
	C32	7.999064028	-7.485769446	4.529938699
	C33	5.595001336	-6.935807706	3.918258438
	C34	5.925343902	-5.493819231	3.550722877
	C35	4.685235442	-4.606709721	3.511639487
	C36	4.907266252	-2.107271520	3.551207749
	C37	10.265479095	-6.558264204	4.487224367
	C38	10.502178956	-5.065125947	4.475024518
	C39	-5.314906902	-2.671359069	-1.555552625
	C40	-5.685669005	-1.240977756	-1.117338113
	C41	-3.778704394	-2.819131377	-1.504746915
	C42	-3.243033974	-4.261100867	-1.620956205
	C43	-2.978321813	-4.748544243	-3.046068153
	C44	-6.186878865	0.997613911	-2.021979407
	C45	-7.279639458	1.449825169	-1.043165962
	C46	-6.367287690	1.604239949	-3.419181967
	C47	-9.433571657	0.920514435	0.000258916
	C48	-9.037200870	0.944941032	1.479797222
	C49	-7.328641647	0.441772875	3.163192968

	C50	-6.155191389	1.400327992	3.372217152
	C51	-4.603206516	3.041048612	2.435063578
	C52	-5.047211813	4.347330797	3.134152835
	C53	-4.025464207	3.477962156	1.080408407
	C54	-3.407054077	2.416473780	0.234108434
	C55	-2.987677312	1.131917103	0.470077371
	C56	-2.553961206	1.601082547	-1.665314914
	C57	-6.847785337	6.028765214	3.232950693
	C58	-6.759701911	6.184481386	4.749944001
	C59	-8.318733265	6.083739964	2.759248211
	C60	-8.945414657	7.443079255	2.650331731
	C61	-8.869160231	8.546793093	3.477536167
	C62	-10.205220442	8.961327444	1.758878199
	C63	9.239262761	1.622978043	3.446448339
	C64	10.259520619	0.511884685	3.276144875
	C65	7.842522210	0.982124250	3.262086840
	C66	6.709183012	1.958956737	3.473335381
	C67	6.140575019	2.161076973	4.743484231
	C68	6.187327909	2.696175947	2.394972048
	C69	5.087517309	3.063941093	4.934827043
	C70	5.152098157	3.614063849	2.572854758
	C71	4.595683795	3.789918954	3.842319585
	C72	5.378032217	6.872830267	-1.382869730
	C73	4.319300720	7.967891178	-1.595631100
	C74	4.711007177	5.532013208	-1.082404533
	C75	3.308366112	9.545211711	-3.206036941
	C76	1.833578493	9.167242853	-3.365700598
	C77	0.210884270	7.275300149	-3.323832009
	C78	-0.256899309	6.928161178	-1.886629287
	C79	0.243165355	6.027572241	-4.236575790
	C80	-1.009629174	5.149380419	-4.344760798
	C81	-2.152816741	5.683819942	-5.224448654
	C82	-3.672663897	7.605768178	-4.740563793
	C83	0.262810629	5.987135553	0.312414194
	C84	0.111199815	7.211289537	1.227924167
	C85	1.254936546	5.017582473	0.986837788
	C86	1.203524524	9.274605961	1.963076359
	C87	0.559061749	10.617667744	1.653104027
	C88	-0.858240713	11.880686768	0.123480901
	C89	-2.131197227	12.433028872	0.809499680
	C90	-1.025342599	11.883493940	-1.398857810
	C91	-2.164762670	11.003251161	-1.896313877
	C92	-4.075806821	11.948797747	2.221379969
	C93	-3.983271787	13.047865183	3.271132674
	C94	-4.729838803	10.677947753	2.833630703
	C95	-4.973300419	9.604615034	1.796569801
	C96	-6.160639433	9.594397020	1.048261851
	C97	-4.004547182	8.628286452	1.511518975
	C98	-6.368085170	8.657758190	0.033720314
	C99	-4.181982512	7.699963387	0.481079435
	C100	-5.364094010	7.731900059	-0.252953439
	C101	4.086649253	-3.977198268	-9.000441316
	C102	2.660018473	-3.727464826	-8.546034094
	C103	1.207630691	-2.196830753	-7.232201734
	C104	0.710077293	-0.837555545	-7.738887961
	C105	1.127315252	1.583475669	-7.694504426
	C106	2.295026358	2.546218855	-7.901745690
	C107	4.574734992	3.235672877	-7.403747486
	C108	5.187833135	3.372923467	-8.783175824
	C109	-2.760252716	-3.490721513	-9.141112633
	C110	-3.788738448	-2.404252455	-8.802927158
	C111	-2.272497879	-4.174796577	-7.844451804
	C112	-1.536159250	-5.492104073	-8.149433900
	C113	-0.853396388	-6.017519219	-6.907124412
	C114	-4.192953995	-0.085371402	-8.145168074
	C115	-3.717971212	1.199619588	-8.793667032
	C116	-4.570223874	0.044956528	-6.648671046
	C117	-3.333481328	0.055575628	-5.796905749
	C118	-2.607174019	1.242667208	-5.600491406
	C119	-2.794455572	-1.157313396	-5.337389272
	C120	-1.325393110	1.195529599	-5.043213453
	C121	-1.514368322	-1.203165578	-4.779877750
	C122	-0.762583068	-0.029062322	-4.673362248
	C123	7.905687946	-6.222464411	-4.757906317
	C124	9.092298251	-6.317565341	-3.786669746
	C125	6.595931090	-6.597020037	-4.050430956
	C126	5.336546289	-6.362638635	-4.819979941
	C127	4.961346692	-6.933389146	-6.019223717
	C128	3.248095193	-5.810792088	-5.180791126
	C129	10.304354170	-7.555263597	-2.093563513

C130	11.653280329	-7.314518686	-2.735141790
C131	0.594659000	-6.948347625	-0.182294639
C132	2.085351398	-6.986259286	0.132767942
C133	3.001935271	-7.102037613	-1.121549057
C134	2.902880610	-8.533300202	-1.712910285
C135	3.941578217	-8.872690421	-2.785305652
C136	3.966799090	-10.364427325	-3.184134561
C137	4.461627227	-6.856419353	-0.707673575
C138	3.601179277	-0.684492102	-2.234792422
Fe139	3.805947144	-2.004996879	-3.658444236
Fe140	2.243726382	0.704796701	-1.454220245
Fe141	5.051385138	0.444428912	-1.508891004
Fe142	3.823936863	0.494216450	-3.767451281
Fe143	3.858349302	2.750666188	-2.519406647
Fe144	4.751685172	-2.057362378	-1.238076289
Fe145	2.183460948	-2.374571584	-1.457612418
H146	-6.388658970	-7.540764084	0.316353592
H147	-6.575214575	-6.555310045	-0.971916379
H148	-9.689349559	-4.766845639	1.395125192
H149	-8.864433638	-6.106699245	-0.397482146
H150	-8.705999498	-7.222996052	0.954611031
H151	-7.856177844	-2.715789312	2.030740654
H152	-8.626786387	-5.582242899	3.968141412
H153	-9.475915278	-2.919523685	2.728122331
H154	-7.852648441	-6.219403398	6.066591473
H155	-5.976455924	-5.838584501	3.657096721
H156	-7.656914503	-4.504776751	6.496713456
H157	-2.874007467	-1.741338737	3.153264628
H158	-4.945494430	-3.697805687	3.016812675
H159	-3.688865522	-6.161725140	3.314840746
H160	-4.740815974	-0.045603215	7.103495636
H161	-5.623470972	-2.278354673	5.484450164
H162	-3.412298186	-6.232086946	5.072935539
H163	-4.612400168	-1.440062851	2.940843164
H164	-4.179766545	-3.592793900	7.451464051
H165	-5.893198014	-1.319631304	7.587017553
H166	-0.365783672	-3.371945476	3.613083550
H167	-0.698997590	-2.278168307	2.251440080
H168	0.961413571	-2.483835868	2.803444890
H169	-1.295436221	-1.245753566	4.477396205
H170	-3.346111298	-4.640061642	9.371694047
H171	-1.867099771	2.009182696	5.299431021
H172	-1.446035718	-0.254266946	9.211777961
H173	-1.679312607	3.148937676	9.504981080
H174	0.652796203	0.024519741	2.565638322
H175	0.371278150	0.882250580	4.095676836
H176	9.313394728	1.980369680	4.491274131
H177	-2.816767812	3.362227470	5.935947553
H178	-2.681803771	4.031266424	8.328819139
H179	-1.050933352	3.526922472	5.768061785
H180	-0.907967380	4.188101309	8.283214156
H181	-2.502452565	1.620031931	7.702280390
H182	0.476804524	2.159671976	7.238190546
H183	-1.290889826	0.021480284	6.387970300
H184	-0.991145640	0.505635646	3.045533349
H185	1.546875289	4.244188538	0.261480252
H186	0.715812874	4.532045549	1.813700785
H187	2.664064185	5.318031275	2.331733868
H188	3.209729332	4.680913604	4.870348207
H189	4.749398537	4.166414459	1.723991759
H190	6.580697812	2.530416160	1.389400736
H191	6.515900405	1.596111992	5.601367843
H192	-0.822330255	-2.733388919	6.235751025
H193	0.205278403	-5.666267923	6.400478428
H194	0.832649407	-5.134179849	7.974146992
H195	-1.866370334	-4.571687654	6.846500843
H196	-2.819999624	-3.062213595	10.011877194
H197	4.644081540	3.194383593	5.925993950
H198	1.652251881	-1.653525406	5.049359018
H199	-0.746138147	0.717451193	11.730991017
H200	-0.942124843	-1.007799104	11.322911349
H201	1.657531123	-0.734659855	10.478474718
H202	11.136642046	-7.065805382	4.046008818
H203	-1.760446367	-5.993840668	-4.122779083
H204	1.211711980	-6.061331850	-1.776760850
H205	-2.264437149	-6.245533383	-8.483132529
H206	-0.802894987	-5.340405084	-8.954639755
H207	-3.125872436	-4.386404605	-7.181603077
H208	-3.121055470	1.048218693	-9.724073060
H209	-3.347684298	-4.232076168	-9.705006678

H210	-3.340113366	-2.196072185	-2.302442483
H211	-3.958671883	-4.966701475	-1.164961122
H212	-2.282179937	-1.024953584	-8.423584852
H213	-5.132899116	-0.319519964	-8.688001493
H214	-1.036782386	-2.404412729	-9.549307015
H215	-1.333071657	-5.867022381	-2.419823363
H216	-1.618726827	-3.476012795	-7.299574722
H217	-1.215092570	-3.761760188	-10.466041582
H218	-6.732200902	-3.453190454	-2.840913015
H219	-3.445828944	-2.411681795	-0.538442055
H220	0.832250497	-5.282522604	-7.594932004
H221	-2.318731375	-4.351522718	-1.032655483
H222	-3.826102411	2.602879683	3.077155329
H223	-3.028805743	0.520848372	1.362381501
H224	-8.142411587	0.786641590	3.815818640
H225	-7.266572979	-0.071683152	1.091241766
H226	-6.923813085	4.080087424	2.349012231
H227	-8.317146434	8.737425069	4.390332066
H228	-6.906979793	5.228439422	5.309201109
H229	-8.927123832	5.419890279	3.399268005
H230	-6.262389430	6.832648789	2.759462365
H231	-10.885282671	9.508632652	1.111829438
H232	-3.267628819	4.250532751	1.287052377
H233	-4.823650924	3.963888748	0.496389627
H234	-9.834399858	10.440938108	3.245410424
H235	-8.350580423	5.648349999	1.747858044
H236	-6.173109128	2.010693082	1.380275651
H237	-7.024103422	-0.548781519	3.532948461
H238	-9.881743174	1.901536666	-0.211132924
H239	-2.213560973	1.500466561	-2.693468915
H240	-10.213928295	0.160830705	-0.136686092
H241	10.301061670	3.205435391	2.696639002
H242	8.693310278	3.308761193	2.423670832
H243	1.547837246	5.982701051	-1.423029656
H244	-0.723668432	5.502107593	0.284913276
H245	1.736774236	8.067876432	0.312486640
H246	-3.437249190	3.541856768	-1.608713833
H247	-5.107101748	4.157248546	-2.783126714
H248	-6.717085140	3.390777133	-4.151130332
H249	-4.794359902	5.408785633	-1.850112674
H250	-5.547789084	1.240017671	-4.060404234
H251	-7.326960687	1.278813686	-3.849059446
H252	-0.071487772	11.619928726	-1.876607976
H253	-1.270342841	12.916264522	-1.691982377
H254	-0.910115242	10.155940857	-3.310127996
H255	-2.579868870	9.588922947	-3.269898500
H256	-3.659024196	9.219666008	-6.009462507
H257	-2.645434696	4.850164685	-5.747590441
H258	-7.305179751	8.652919524	-0.529355287
H259	-3.085368158	8.568952645	2.099763315
H260	-4.709208182	7.761084202	-2.965168358
H261	1.063892117	5.384429560	-3.879547894
H262	2.340384493	7.199064054	-3.224842831
H263	-1.396916543	4.887130652	-3.347779753
H264	-8.364733896	-0.227772530	-1.466965647
H265	3.641732894	10.003074457	-4.146932994
H266	-6.087300092	-0.954263662	-3.048177668
H267	0.537586023	6.363737378	-5.244273741
H268	-3.387314688	6.994439521	0.234727424
H269	3.341993357	10.318210341	-2.424617716
H270	-2.909174345	7.792944425	-6.649559489
H271	-5.099637312	8.952916142	-4.160322273
H272	-5.168258158	-3.513844356	-3.430419228
H273	-5.740223248	-3.335115847	-0.779684016
H274	-6.945536455	10.323196464	1.263622436
H275	-5.260749073	1.397038906	-1.584767341
H276	-0.488506025	8.028736509	-3.697727257
H277	-1.737251439	6.343156588	-5.996642416
H278	-0.676808731	4.206309864	-4.800525783
H279	-3.606773035	5.869503880	-3.680279001
H280	0.819424224	8.939347066	2.938136762
H281	-0.457790291	9.763603783	0.097892755
H282	-0.091035996	12.631964082	0.365761081
H283	2.282563288	9.452550318	2.063110632
H284	-2.447357193	10.650414303	1.723165193
H285	4.606477792	-3.019470334	-9.148488381
H286	4.586547338	-4.487107260	-8.150110226
H287	7.865250570	-5.164290476	-5.056032526
H288	5.046799176	-5.075970937	-10.424817140
H289	3.512203478	-5.569085169	-10.135346626

	H290	5.497766077	4.782211951	-0.938055939
	H291	5.714301839	7.435690085	0.549582051
	H292	4.831434216	8.096350380	-3.584617995
	H293	4.111102666	5.611415408	-0.165341535
	H294	6.817655469	8.042238924	-0.496727509
	H295	5.369632278	2.906701929	-6.716572983
	H296	5.986068920	6.771986868	-2.295329280
	H297	5.284843925	-7.008794593	4.973871065
	H298	7.046182220	-7.867921742	2.613321229
	H299	3.643969534	1.419563662	-6.776443588
	H300	5.182921791	-0.093039157	3.293470034
	H301	0.558324048	1.550981085	-8.631187699
	H302	-1.095157447	-2.154836694	-4.445120458
	H303	0.473664105	2.058092650	-6.942084837
	H304	4.251856240	-4.491481690	4.512293216
	H305	2.139745892	0.025878828	-6.575404713
	H306	3.909865529	-5.056666248	2.870655622
	H307	4.759151067	-7.298173235	3.298374369
	H308	6.393025509	-5.470088411	2.555063818
	H309	6.650653664	-5.062589469	4.258348627
	H310	-5.218292775	-0.804933461	-6.390344047
	H311	5.923320320	-9.441802075	4.895873862
	H312	4.981480044	-11.764009746	3.177248386
	H313	0.253964257	-0.049134433	-4.289163059
	H314	6.342349076	-11.751065242	5.764861992
	H315	4.788101996	-12.123335455	5.410093390
	H316	5.525427176	-3.322319350	2.063288713
	H317	-0.742331346	2.108086536	-4.907969610
	H318	-3.368678523	-2.080558365	-5.440039817
	H319	7.615517190	-9.938186853	-1.665435647
	H320	6.624736907	-7.668236942	-3.785088668
	H321	6.615682717	-8.183748412	-0.101307128
	H322	6.750404179	-10.542645290	-2.809447296
	H323	4.957838118	2.540067080	-9.490411788
	H324	6.364580545	-5.072432446	-0.236836271
	H325	2.283557718	-7.802342643	0.838099643
	H326	3.282164609	-2.024168938	-7.604660116
	H327	3.018004502	-9.233544789	-0.870564044
	H328	3.050858744	-6.878312469	-6.986871650
	H329	2.240308292	-5.429283939	-5.078107716
	H330	6.591847130	-12.408542152	3.525360921
	H331	5.783976273	-1.067148608	1.985226971
	H332	8.365879766	-5.801451314	0.786331917
	H333	7.229277906	-3.787268026	-0.167483537
	H334	8.970721968	-6.848618709	2.795808370
	H335	3.700930043	-2.701195033	5.152892176
	H336	3.733730200	-8.288983493	-3.695081435
	H337	4.949154224	-8.592893338	-2.449419150
	H338	1.887581273	-8.674730668	-2.113747467
	H339	1.346128472	-2.171258062	-6.139372447
	H340	-3.031444679	2.191928026	-5.935959676
	H341	-5.152121370	0.972601345	-6.541528797
	H342	2.304794711	-6.036762778	0.649773711
	H343	8.503913260	-7.337845172	0.581439844
	H344	7.409167820	-9.253808870	0.675110352
	H345	4.154095329	-1.040527861	5.127783575
	H346	5.508249713	-7.569466406	-6.704148050
	H347	6.535266529	-6.055292611	-3.099427517
	H348	0.457295802	-2.948840617	-7.482729115
	H349	4.231857254	4.225746672	-7.066453265
	H350	7.759365174	0.148270427	3.974400036
	H351	7.787653385	0.560998698	2.245454283
	H352	8.977096330	-6.708262305	-6.438054845
	H353	8.259993933	-8.002231598	-5.737038421
	H354	10.308994509	-8.583474576	-1.698976503
	H355	8.543464052	-8.223003271	-3.094359659
	H356	11.697169596	-7.545197498	-3.826947644
	H357	10.169943469	-6.873235341	-1.239044349
	H358	10.540771153	0.305343165	2.214819714
	H359	-2.973247513	13.185460654	3.724415628
	H360	10.192764165	-6.865073445	5.545558860
	H361	9.582527770	-4.436804028	4.384852522
	H362	-4.715033184	12.321283152	1.403805161
	H363	-4.084785944	10.296916986	3.640968788
	H364	-5.678767214	10.990348386	3.292893127
	H365	1.103722661	-3.291975183	-0.966201730
	H366	3.860194583	-1.221022607	0.048111199
	H367	-6.460339461	6.800524977	-1.568282591
	H368	0.146770338	-0.930298687	-0.538772616
	H369	-2.026658678	-0.250212272	-0.869050864

Mo370	3.839073950	-4.406058612	-2.347461153
N371	-7.078254641	-7.199977697	-0.357862300
N372	-8.752791414	-4.610792255	1.760105597
N373	-8.058757091	-4.902276403	4.467687098
N374	-5.376242110	-5.762275364	4.479425604
N375	-4.159006919	-3.360933090	3.568952633
N376	-4.844210982	-1.713565036	5.832738864
N377	-3.620049969	-3.019403849	8.088450628
N378	-1.121798740	-4.523482167	7.547058645
N379	0.180712173	-2.614806639	6.107271205
N380	-0.390057413	0.326571040	6.765013324
N381	-0.647380996	0.199482801	9.663193397
N382	5.545944506	-11.477144131	5.184503456
N383	6.381675972	-9.241099201	4.000628112
N384	5.035477612	-3.297713646	2.960439693
N385	4.298005489	-1.973017746	4.749011286
N386	5.414553609	-1.019385306	2.946314539
N387	9.034185633	-6.954551699	3.832640547
N388	-5.848695711	-2.942543974	-2.896031710
N389	-1.958514568	-5.613336773	-3.191253322
N390	-6.075613336	-0.444813703	-2.148098274
N391	-8.356537145	0.639082815	-0.933750482
N392	-7.856315265	0.352634701	1.814609874
N393	-5.686439144	2.078012044	2.278632416
N394	-3.125502597	2.673719204	-1.101596167
N395	-2.474036254	0.655684091	-0.720963943
N396	-6.320174384	4.725062221	2.852949549
N397	-9.783466744	7.714835109	1.580666806
N398	-9.673479104	9.501377932	2.891985620
N399	9.480213433	2.654096246	2.434977042
N400	6.282354062	7.199752740	-0.270417015
N401	4.212541014	8.461025989	-2.865250821
N402	1.553582622	7.847831627	-3.335799298
N403	-3.176443043	6.401335248	-4.458235833
N404	-3.350845123	8.263114233	-5.868230290
N405	-4.457449691	8.234817370	-3.836274090
N406	0.649234828	6.334601501	-1.057597029
N407	0.975419115	8.235006430	0.976688264
N408	-0.365505114	10.618919500	0.645262508
N409	-1.888270855	10.295172406	-3.025801070
N410	-2.754833170	11.619448077	1.697799292
N411	4.097464667	-4.737043298	-10.253304142
N412	2.463414039	-2.580189117	-7.864383264
N413	1.437471611	0.218293376	-7.309602419
N414	3.499648083	2.256669324	-7.346639993
N415	-1.724678661	-2.980964532	-10.047899748
N416	-3.294305200	-1.177698604	-8.467410341
N417	8.114141908	-7.017736544	-5.981708582
N418	4.250169980	-5.648567124	-4.315810230
N419	3.648920611	-6.571083577	-6.224410443
N420	9.220737226	-7.457042500	-3.055200833
N421	0.118280758	2.277788331	-0.151905503
N422	2.630243160	-0.814927226	0.356862407
N423	0.962600905	1.709872650	-0.642074309
N424	1.882214887	-1.586228887	0.836415488
O425	-6.542814861	-5.142220249	1.598452066
O426	-7.203342397	-2.788502866	4.470635113
O427	-5.220546305	-5.142147647	6.670874678
O428	-2.437027241	-3.910472593	4.957382907
O429	-3.040438021	-0.441105389	5.292253272
O430	-3.292776277	-0.922046824	8.952098883
O431	-0.499292371	-3.878137374	9.652682200
O432	2.186833269	-3.744324165	6.182687317
O433	1.886285828	0.193391886	6.608110500
O434	0.896430431	1.865077667	9.654043749
O435	1.574030265	-0.213345098	12.448672106
O436	7.058010812	-10.324238299	2.072400030
O437	7.995434501	-7.647734521	5.763364803
O438	11.608915034	-4.559147528	4.573250677
O439	-5.615588314	-0.847361907	0.063481645
O440	-3.687058108	-4.383479587	-4.012618126
O441	-7.145537000	2.496381630	-0.383958437
O442	-6.335848412	3.041951160	-3.325718970
O443	-9.795673240	1.481742926	2.297633070
O444	-5.663731819	1.531755926	4.496578571
O445	-4.263509474	5.031682672	3.797203711
O446	-6.595632689	7.246113259	5.326076106
O447	10.731164781	-0.133775854	4.201061049
O448	3.547598151	4.683563897	3.955872133
O449	3.604436438	8.389872310	-0.669796704

	O450 0.987939561 10.069496891 -3.520351278<br>O451 -1.417429661 7.186920712 -1.532171335<br>O452 -0.704472060 7.210341106 2.159303645<br>O453 2.413041158 5.703899894 1.467698830<br>O454 0.860454965 11.630980295 2.295035432<br>O455 -2.509273424 13.590284263 0.578500286<br>O456 -3.273737180 10.983697457 -1.343407196<br>O457 -4.942840501 13.714559721 3.622555762<br>O458 -5.513851408 6.831370813 -1.328301182<br>O459 1.756240854 -4.568411207 -8.772741747<br>O460 -0.275504729 -0.736409379 -8.497804735<br>O461 2.097240138 3.602549612 -8.524585072<br>O462 5.911840465 4.302171154 -9.100997222<br>O463 -5.008295156 -2.639784462 -8.815736351<br>O464 -1.448414364 -6.607371525 -6.011916011<br>O465 0.466406745 -5.765025444 -6.789755860<br>O466 -3.995308136 2.322393186 -8.394887517<br>O467 9.938116049 -5.406010590 -3.699854706<br>O468 12.648232150 -6.966757352 -2.115654441<br>O469 0.274313885 -6.298307206 -1.310736010<br>O470 -0.262785711 -7.420685602 0.560607738<br>O471 5.118791986 -10.903636149 -3.344829798<br>O472 2.857115378 -10.944649274 -3.351072902<br>O473 5.089819423 -5.890685644 -1.330348817<br>O474 5.001770847 -7.573731330 0.154015748<br>O475 2.598236534 -6.106452309 -2.062502210<br>O476 8.947967421 -6.563271118 1.027095725<br>O477 6.985023699 -4.575186960 0.354607211<br>O478 -4.374566306 4.743523938 -2.450720375<br>O479 7.701806536 -10.433619585 -2.510056051<br>O480 7.503065329 -8.632630685 -0.091318901<br>S481 3.590891810 4.967593669 -2.440282977<br>S482 2.092967845 1.856262860 -3.478295715<br>S483 2.017473812 -3.364916884 -3.447070375<br>S484 3.839382163 2.014716913 -0.425159243<br>S485 0.540773344 -0.874014624 -1.842964160<br>S486 3.993957626 -0.813354200 -5.518672734<br>S487 3.530728484 -3.629756307 -0.182417230<br>S488 5.652384041 1.684026968 -3.293870947<br>S489 5.673676060 -3.065962293 -3.046884809<br>S490 6.380703249 -0.865037346 -0.335834794<br>end

product

Fe(139) 2.416<br>Fe(140) 0.020<br>Fe(141) 2.627<br>Fe(142) -2.342<br>Fe(143) 2.373<br>Fe(144) -2.287<br>Fe(145) -0.161	bm52capexp347tg.car_6<br>C1 -7.718196511 -5.352898645 1.290072937<br>C2 -8.055309545 -6.509440248 0.341622422<br>C3 -8.618108083 -3.462385737 2.576097494<br>C4 -7.988527564 -3.730318018 3.941305396<br>C5 -7.584772214 -5.336284121 5.743091369<br>C6 -6.058787113 -5.454981761 5.688530758<br>C7 -4.076041724 -5.775869210 4.303546090<br>C8 -3.525144431 -4.348686083 4.347349185<br>C9 -3.889564906 -2.009653104 3.704947089<br>C10 -3.892450980 -1.407962363 5.117339591<br>C11 -4.947735751 -1.237231246 7.281716398<br>C12 -3.885133107 -1.812513504 8.229522525<br>C13 -2.891932090 -3.846464297 9.173569206<br>C14 -1.415589991 -4.104940764 8.843842827<br>C15 0.237185520 -4.699170018 7.117812770<br>C16 0.962792251 -3.564102574 6.390909710<br>C17 0.669537458 -1.313802553 5.392615873<br>C18 0.787021688 -0.086593742 6.310664676<br>C19 -0.158142990 -1.094191192 4.108168663<br>C20 0.332118036 0.146923945 3.351437850<br>C21 -0.123197806 -2.345782293 3.227201847<br>C22 -0.386802056 1.618567093 7.634458425<br>C23 -0.032748760 1.263275716 9.090789835<br>C24 -1.724875528 2.382738639 7.499829442<br>C25 -1.807972421 3.539244752 8.501590988<br>C26 -1.904092372 2.900579520 6.066013213<br>C27 -0.447471745 -0.110500916 11.044731593<br>C28 1.014957994 -0.397395460 11.322018738<br>C29 5.881751941 -11.626740449 3.689987025<br>C30 6.519423088 -10.347941135 3.159693324<br>C31 6.823601965 -7.937319293 3.684101215<br>C32 8.051440643 -7.551473685 4.527995772<br>C33 5.652203709 -6.974105137 3.935198272<br>C34 5.994838500 -5.533511418 3.568166483<br>C35 4.757595756 -4.641728777 3.520820935

	C36	4.898534991	-2.137225183	3.514435238
	C37	10.290092677	-6.551949614	4.464320033
	C38	10.467726825	-5.050257679	4.465200272
	C39	-5.320830990	-2.666286783	-1.588800276
	C40	-5.692644145	-1.235148768	-1.156479792
	C41	-3.784909960	-2.806670642	-1.520875733
	C42	-3.231182397	-4.241496488	-1.628126769
	C43	-2.967934607	-4.745724734	-3.047226302
	C44	-6.163179402	1.006279653	-2.066420365
	C45	-7.251014580	1.468881404	-1.086957043
	C46	-6.340383643	1.610911847	-3.464957991
	C47	-9.409244446	0.959859395	-0.042005422
	C48	-9.015426993	0.959999729	1.438458250
	C49	-7.317204393	0.407803055	3.116516499
	C50	-6.130475966	1.348847339	3.331114211
	C51	-4.601071741	3.020274195	2.404301491
	C52	-5.027953427	4.348116678	3.082019680
	C53	-3.992866398	3.419818736	1.050658081
	C54	-3.329391814	2.360871485	0.225105749
	C55	-2.781157468	1.124366178	0.470158061
	C56	-2.426515903	1.590243755	-1.672298118
	C57	-6.836885123	6.025236574	3.250658657
	C58	-6.713036520	6.186229560	4.764289084
	C59	-8.318891100	6.086921092	2.811890257
	C60	-8.928610250	7.453298108	2.687906825
	C61	-8.863318953	8.553937125	3.520326904
	C62	-10.150198898	8.987477457	1.769388441
	C63	9.251943088	1.658552181	3.409912883
	C64	10.298367969	0.573896804	3.234572200
	C65	7.865014573	0.993716806	3.222962078
	C66	6.722427388	1.958340539	3.435547477
	C67	6.136785432	2.136709230	4.701327322
	C68	6.221576086	2.723467637	2.366423583
	C69	5.087506271	3.042975967	4.897828258
	C70	5.191777077	3.645743219	2.550693486
	C71	4.619477159	3.799547124	3.815738633
	C72	5.386529335	6.867462314	-1.399558518
	C73	4.320044182	7.955784367	-1.608513956
	C74	4.732683069	5.539844061	-1.026408840
	C75	3.309217292	9.540915185	-3.209272354
	C76	1.832878242	9.168804253	-3.373841739
	C77	0.210227678	7.271568869	-3.330102763
	C78	-0.260546744	6.925734929	-1.892709401
	C79	0.245784458	6.028952443	-4.247742202
	C80	-1.003957941	5.147497026	-4.359557890
	C81	-2.151987338	5.684757743	-5.230526148
	C82	-3.683688702	7.592587005	-4.749430831
	C83	0.264362883	5.967970876	0.302375192
	C84	0.104217290	7.178543086	1.231127570
	C85	1.273877091	5.002932776	0.959595452
	C86	1.182048787	9.241857790	1.984311118
	C87	0.550837802	10.592882735	1.679083363
	C88	-0.845032249	11.876505877	0.146039599
	C89	-2.122569771	12.428855015	0.821394835
	C90	-0.989474333	11.889160036	-1.378228410
	C91	-2.145221092	11.045489241	-1.902995066
	C92	-4.070985581	11.945692609	2.224059947
	C93	-3.989739524	13.053181289	3.266522933
	C94	-4.731150630	10.681030105	2.839676081
	C95	-4.968748225	9.606493480	1.803816795
	C96	-6.139137933	9.613597449	1.030342050
	C97	-4.009376269	8.614955708	1.543767025
	C98	-6.338704785	8.677724361	0.014128816
	C99	-4.180803376	7.684069353	0.514593107
	C100	-5.345647626	7.732689514	-0.245331598
	C101	4.081554416	-3.990439210	-9.002013102
	C102	2.656964000	-3.730995739	-8.544195681
	C103	1.205612322	-2.177607116	-7.262872985
	C104	0.703810067	-0.831434303	-7.803377221
	C105	1.106503283	1.599254774	-7.774182458
	C106	2.291439457	2.541187220	-7.956045345
	C107	4.580747101	3.174266182	-7.450965968
	C108	5.105520210	3.398997610	-8.855624630
	C109	-2.743649357	-3.503529976	-9.160291562
	C110	-3.764326617	-2.403671950	-8.844151916
	C111	-2.251558060	-4.165000725	-7.853969183
	C112	-1.536733705	-5.498007819	-8.143584508
	C113	-0.869190605	-6.026022976	-6.894551369
	C114	-4.155972512	-0.081504733	-8.200946327
	C115	-3.656866308	1.200094110	-8.837594478

	C116	-4.578239354	0.059435323	-6.717539637
	C117	-3.355651620	0.088915251	-5.849479221
	C118	-2.635931791	1.281961247	-5.669094531
	C119	-2.813579518	-1.117974547	-5.377178618
	C120	-1.352678248	1.246008142	-5.114322097
	C121	-1.537429676	-1.148456010	-4.814055532
	C122	-0.787974055	0.028568537	-4.726700371
	C123	7.892327830	-6.271405536	-4.792876872
	C124	9.079766629	-6.343490540	-3.817145702
	C125	6.581447899	-6.566610094	-4.048513868
	C126	5.312143937	-6.343981334	-4.807821359
	C127	4.932188652	-6.918572094	-6.003427854
	C128	3.207317702	-5.830390139	-5.141441416
	C129	10.308070869	-7.554355757	-2.117037410
	C130	11.663332011	-7.336092324	-2.753353155
	C131	0.613962156	-6.899657256	-0.111503008
	C132	2.111248512	-6.933994381	0.185633312
	C133	3.008565049	-7.052209041	-1.080731988
	C134	2.912447332	-8.487401136	-1.667108650
	C135	3.951524296	-8.833735531	-2.737376440
	C136	3.979012861	-10.325874516	-3.137676122
	C137	4.476376901	-6.798566669	-0.700919197
	C138	3.599357723	-0.688650324	-2.240557868
	Fe139	3.750849165	-1.984855322	-3.702257375
	Fe140	2.130825397	0.607584794	-1.336987583
	Fe141	4.983395718	0.495229954	-1.464999086
	Fe142	3.770543098	0.535027618	-3.729127445
	Fe143	3.740571091	2.743114442	-2.404623116
	Fe144	4.874420084	-2.044459274	-1.421271281
	Fe145	2.152406763	-2.247033145	-1.294476489
	H146	-6.256438398	-7.471901563	0.384439190
	H147	-6.435079300	-6.487025746	-0.904556118
	H148	-9.709591695	-4.830174769	1.350341162
	H149	-8.767120961	-6.149514868	-0.418853984
	H150	-8.604126985	-7.257972198	0.939634658
	H151	-7.977466009	-2.721129516	2.076221595
	H152	-8.754423931	-5.630537566	3.961435812
	H153	-9.603688766	-3.003914212	2.735239041
	H154	-8.004339696	-6.300816246	6.057699488
	H155	-6.110814345	-5.857910473	3.667229171
	H156	-7.813568245	-4.595274165	6.520054152
	H157	-2.885255811	-1.851010982	3.293146554
	H158	-5.057216751	-3.712108111	3.126887057
	H159	-3.841870098	-6.233204407	3.332749347
	H160	-4.792701588	-0.150579879	7.252337099
	H161	-5.690941686	-2.318660064	5.566808223
	H162	-3.569495605	-6.348002093	5.089693388
	H163	-4.606804147	-1.466102747	3.074300469
	H164	-4.278689242	-3.718935957	7.546975545
	H165	-5.940594979	-1.440241559	7.707056967
	H166	-0.487196849	-3.226798875	3.769716514
	H167	-0.771587647	-2.208107655	2.347342621
	H168	0.888354098	-2.539096672	2.844090524
	H169	-1.198931125	-0.910845773	4.414614470
	H170	-3.358840184	-4.821851322	9.375692612
	H171	-1.902619615	2.087127675	5.327507249
	H172	-1.484357839	-0.233887591	9.185692760
	H173	-1.768305521	3.194286357	9.544550314
	H174	1.364813857	0.019049798	2.990921552
	H175	0.289768497	1.053504554	3.975294306
	H176	9.318739278	2.015840272	4.455063473
	H177	-2.861095372	3.433118201	5.968380932
	H178	-2.752375034	4.084316277	8.358885253
	H179	-1.095363205	3.604814302	5.809826221
	H180	-0.978719041	4.247749393	8.346841604
	H181	-2.542345788	1.672073962	7.720342863
	H182	0.434098076	2.274320034	7.308050446
	H183	-1.273946413	0.080659751	6.422473518
	H184	-0.299063301	0.327982113	2.467811704
	H185	1.575791704	4.246065268	0.220492082
	H186	0.746631645	4.497895939	1.781503757
	H187	2.675964788	5.313091548	2.309484648
	H188	3.226158234	4.679454512	4.843041704
	H189	4.805651092	4.221997052	1.710020038
	H190	6.628618091	2.578237141	1.363161785
	H191	6.497904358	1.551584366	5.552087976
	H192	-0.771865439	-2.516769212	6.412666983
	H193	0.232113930	-5.567255500	6.441886925
	H194	0.832648864	-4.964192586	8.001481039
	H195	-1.857300725	-4.440430945	6.833698074

	H196	-2.899658940	-3.259816346	10.100034217
	H197	4.632367029	3.156052339	5.885774020
	H198	1.701396219	-1.541752982	5.101459981
	H199	-0.787355915	0.678868609	11.733968015
	H200	-1.008467748	-1.028497298	11.278382111
	H201	1.607217107	-0.748705203	10.445069725
	H202	11.172504286	-7.021877085	4.003308306
	H203	-1.732128005	-5.985452984	-4.111265227
	H204	1.215092263	-6.012837195	-1.712181746
	H205	-2.277623336	-6.239914403	-8.475154417
	H206	-0.797005447	-5.364662406	-8.946184826
	H207	-3.101628650	-4.354843848	-7.180248989
	H208	-2.979976104	1.049837788	-9.710715017
	H209	-3.339286083	-4.254247841	-9.702953333
	H210	-3.336121168	-2.175686520	-2.305436728
	H211	-3.928640259	-4.955623584	-1.156677679
	H212	-2.249504257	-1.040140512	-8.440181423
	H213	-5.081953618	-0.309499057	-8.771026867
	H214	-1.030559822	-2.417365272	-9.610250144
	H215	-1.305904731	-5.829544147	-2.408341680
	H216	-1.584422983	-3.465621258	-7.326554307
	H217	-1.196329467	-3.808829039	-10.476132294
	H218	-6.732911317	-3.439642996	-2.884181672
	H219	-3.464086942	-2.394481904	-0.553187785
	H220	0.821754277	-5.283057358	-7.558266618
	H221	-2.299678744	-4.307580440	-1.047925379
	H222	-3.842478258	2.577192853	3.064457406
	H223	-2.737850831	0.540078605	1.380802040
	H224	-8.125431837	0.754247672	3.775393733
	H225	-7.260054046	-0.080982814	1.039793201
	H226	-6.937124058	4.064761366	2.387793700
	H227	-8.335052604	8.736495740	4.448962097
	H228	-6.854584383	5.232589999	5.329828001
	H229	-8.919751064	5.447644702	3.482699711
	H230	-6.259154876	6.825762829	2.762376589
	H231	-10.805731355	9.544255886	1.105746007
	H232	-3.251825764	4.208782605	1.260780001
	H233	-4.781418164	3.885230199	0.437487510
	H234	-9.806939906	10.457022350	3.272119660
	H235	-8.382773662	5.631337416	1.811067081
	H236	-6.185437751	2.010075457	1.356322749
	H237	-7.024753208	-0.590586852	3.473604221
	H238	-9.839545178	1.951302729	-0.242121746
	H239	-2.082732035	1.508926328	-2.700436269
	H240	-10.203743854	0.216258543	-0.187462817
	H241	10.291229500	3.257444628	2.661560740
	H242	8.681830770	3.341367720	2.393570546
	H243	1.547329524	5.984160182	-1.427846851
	H244	-0.715951525	5.470325646	0.272199486
	H245	1.716336769	8.057670839	0.317401110
	H246	-3.443121023	3.465811385	-1.627262328
	H247	-5.115270916	4.160431448	-2.798811410
	H248	-6.692205663	3.390661605	-4.210950796
	H249	-4.789649350	5.390831669	-1.843034623
	H250	-5.509088319	1.260334848	-4.098860686
	H251	-7.291469268	1.270215898	-3.902839367
	H252	-0.035908282	11.601474738	-1.842983478
	H253	-1.198989201	12.929604077	-1.672347582
	H254	-0.909703286	10.217967762	-3.347333629
	H255	-2.596702901	9.706680708	-3.344275642
	H256	-3.765445052	9.153183409	-6.074335945
	H257	-2.645468248	4.852437242	-5.755209497
	H258	-7.260698520	8.688776816	-0.573125429
	H259	-3.103981290	8.545780048	2.152170374
	H260	-4.664821334	7.782346108	-2.949026260
	H261	1.068064462	5.385266142	-3.895523947
	H262	2.339431096	7.205680916	-3.226851284
	H263	-1.385651957	4.870863570	-3.364456187
	H264	-8.347061109	-0.202869944	-1.501903036
	H265	3.645578465	10.006577949	-4.145240528
	H266	-6.065380555	-0.944674762	-3.093560657
	H267	0.538368105	6.371602103	-5.253753862
	H268	-3.395677579	6.960703789	0.291458466
	H269	3.344853823	10.304280981	-2.418411354
	H270	-3.059997339	7.708920026	-6.706943733
	H271	-5.115335742	8.932335887	-4.164818669
	H272	-5.163392844	-3.524876084	-3.453935211
	H273	-5.754313178	-3.327927653	-0.815589495
	H274	-6.913825323	10.359393496	1.223654455
	H275	-5.230833066	1.393339370	-1.630404525

H276	-0.490374799	8.025225871	-3.702345685
H277	-1.740561145	6.350963761	-5.999366986
H278	-0.668953890	4.210653667	-4.826033594
H279	-3.590510461	5.869693461	-3.667626865
H280	0.782276369	8.900152012	2.950887229
H281	-0.463192668	9.756246163	0.112527761
H282	-0.076408245	12.620733715	0.405675754
H283	2.260666961	9.412948790	2.100998149
H284	-2.443541489	10.645713448	1.733481508
H285	4.603214980	-3.036144343	-9.168276616
H286	4.583173936	-4.483317173	-8.140407294
H287	7.879310169	-5.228531762	-5.143103603
H288	5.038304484	-5.120770857	-10.402423330
H289	3.503548691	-5.605333691	-10.098914148
H290	5.518315857	4.784441256	-0.909949493
H291	5.791124285	7.486022247	0.502848883
H292	4.830093697	8.092490948	-3.598328282
H293	4.176814978	5.649376697	-0.084783043
H294	6.842025399	8.080617781	-0.601249025
H295	5.411899114	2.773673608	-6.848127523
H296	5.959840181	6.733485245	-2.330098929
H297	5.347315444	-7.045232306	4.992470977
H298	7.084246455	-7.915749814	2.617974135
H299	3.623936468	1.356806093	-6.865707472
H300	5.090222114	-0.115125745	3.222505098
H301	0.565582554	1.555338317	-8.727158629
H302	-1.111767604	-2.085084435	-4.446121192
H303	0.434565165	2.082400057	-7.044245904
H304	4.325280350	-4.519791065	4.520824199
H305	2.070449922	0.061348681	-6.592088919
H306	3.983101508	-5.095329889	2.881108388
H307	4.808702772	-7.325639475	3.319529964
H308	6.469760425	-5.513722615	2.576178760
H309	6.717082522	-5.105395358	4.280651829
H310	-5.223944641	-0.794628280	-6.466785087
H311	5.946738990	-9.505298887	4.881219963
H312	4.979332199	-11.789948796	3.081832361
H313	0.212366111	-0.003603445	-4.300552908
H314	6.270546464	-11.881707507	5.696766444
H315	4.707991765	-12.159111784	5.294873299
H316	5.588833857	-3.364418907	2.061189138
H317	-0.775054737	2.163845474	-4.989493331
H318	-3.385107224	-2.043896968	-5.473987245
H319	7.661515765	-9.932781779	-1.681028773
H320	6.588426570	-7.621345792	-3.723049752
H321	6.646825109	-8.161784792	-0.120014218
H322	6.784398776	-10.518017661	-2.820999200
H323	4.887755346	2.572869472	-9.574094720
H324	6.372561221	-5.032032205	-0.225114594
H325	2.318425851	-7.746744209	0.891879140
H326	3.274479444	-1.991363196	-7.671791823
H327	3.034479082	-9.178714452	-0.818737642
H328	3.011129428	-6.899359312	-6.949106710
H329	2.194371225	-5.464299319	-5.028920619
H330	6.575988199	-12.450494250	3.450836307
H331	5.753772837	-1.084724024	1.940135039
H332	8.366070646	-5.794491554	0.800465074
H333	7.253898435	-3.760747162	-0.116249240
H334	8.983954388	-6.864197268	2.791263690
H335	3.664281841	-2.726773329	5.101266359
H336	3.749296008	-8.252499144	-3.649940365
H337	4.959299967	-8.556644189	-2.400152980
H338	1.897049961	-8.640059934	-2.063584117
H339	1.338233557	-2.125113364	-6.170853881
H340	-3.066467534	2.225227822	-6.013632075
H341	-5.171429818	0.982221292	-6.635040918
H342	2.338973743	-5.980186376	0.692014811
H343	8.520637413	-7.327766085	0.577573483
H344	7.413889674	-9.250319783	0.659877769
H345	4.118660968	-1.064334008	5.067998913
H346	5.484207728	-7.546020291	-6.692491572
H347	6.554887474	-5.971359113	-3.128787299
H348	0.458311871	-2.937222910	-7.498045437
H349	4.287051563	4.142067600	-7.015577539
H350	7.792730560	0.158653110	3.935265240
H351	7.818167253	0.572195715	2.206177572
H352	8.936730622	-6.882276729	-6.448249332
H353	8.173458377	-8.111424255	-5.683695214
H354	10.305578972	-8.566630389	-1.684100301
H355	8.575433747	-8.268330762	-3.134726844

	H356	11.706870315	-7.565109838	-3.845777546
	H357	10.170531744	-6.843064980	-1.287025433
	H358	10.565773525	0.362493679	2.170655152
	H359	-2.981271833	13.209265906	3.716908121
	H360	10.249796395	-6.872067033	5.520218034
	H361	9.521466640	-4.459944586	4.400562311
	H362	-4.706000664	12.313627164	1.400810427
	H363	-4.094014631	10.302484847	3.654345746
	H364	-5.682864100	10.999012951	3.289292822
	H365	1.164039285	-3.303418384	-0.920308522
	H366	3.284555618	-0.880031295	0.487679841
	H367	-6.430117002	6.807847623	-1.572414127
	H368	0.564944287	-1.347658024	0.247132646
	H369	-1.578353415	-0.130500829	-0.865807931
	Mo370	3.823856674	-4.358048847	-2.346829718
	N371	-6.933908865	-7.156788181	-0.314141077
	N372	-8.791342793	-4.645075166	1.747085119
	N373	-8.196348734	-4.956162577	4.479613149
	N374	-5.518796647	-5.817331574	4.498189609
	N375	-4.243825300	-3.420098233	3.665894486
	N376	-4.899335581	-1.787337558	5.937970318
	N377	-3.694533353	-3.156838525	8.172232537
	N378	-1.118999922	-4.395747186	7.543607185
	N379	0.210535044	-2.467401930	6.149143130
	N380	-0.381073953	0.438663342	6.769993063
	N381	-0.696717916	0.220977560	9.653569354
	N382	5.504270792	-11.548283143	5.107673004
	N383	6.411173580	-9.294422262	3.991573057
	N384	5.109043157	-3.334806091	2.964718306
	N385	4.267798487	-2.000113632	4.700149128
	N386	5.353888559	-1.036889676	2.887582993
	N387	9.064184026	-6.984476921	3.824980294
	N388	-5.842939845	-2.939410503	-2.933980232
	N389	-1.927726813	-5.588605386	-3.186110950
	N390	-6.069618716	-0.438025506	-2.192091803
	N391	-8.337801101	0.670676878	-0.979754069
	N392	-7.846791973	0.342109926	1.767493228
	N393	-5.697680463	2.073676671	2.254255012
	N394	-3.092914728	2.616031904	-1.121861263
	N395	-2.241870168	0.669048833	-0.720652676
	N396	-6.316820590	4.715995537	2.863067133
	N397	-9.735604309	7.737693740	1.597227583
	N398	-9.643066027	9.518242483	2.918068635
	N399	9.476733158	2.696346451	2.400359337
	N400	6.330334898	7.235652941	-0.332351043
	N401	4.208533396	8.449809998	-2.877542921
	N402	1.550655468	7.849689152	-3.340945862
	N403	-3.171201660	6.398898805	-4.454963075
	N404	-3.380918952	8.230041861	-5.897842583
	N405	-4.449116142	8.236706027	-3.840071279
	N406	0.645319547	6.331762684	-1.064570840
	N407	0.957321156	8.213590123	0.986201954
	N408	-0.366198468	10.608002239	0.664752225
	N409	-1.883565913	10.375021182	-3.058955124
	N410	-2.747969043	11.615798922	1.709363148
	N411	4.089386266	-4.776882890	-10.237117345
	N412	2.460039862	-2.568208346	-7.889538427
	N413	1.402059787	0.238191263	-7.360773170
	N414	3.488113415	2.214932717	-7.406008149
	N415	-1.713497479	-3.018006004	-10.086899572
	N416	-3.262177604	-1.184986386	-8.493218959
	N417	8.064772244	-7.134799477	-5.974507648
	N418	4.216600760	-5.654120582	-4.287717356
	N419	3.610618863	-6.581237681	-6.192089928
	N420	9.231222391	-7.482869926	-3.088795861
	N421	0.129356305	2.262433043	0.037393173
	N422	2.347706424	-0.758224556	0.077650788
	N423	0.887529367	1.630007852	-0.504229523
	N424	1.504901304	-1.654688106	0.573802663
	O425	-6.561371340	-5.098633133	1.671377743
	O426	-7.324616857	-2.848283101	4.513143792
	O427	-5.375582867	-5.241641468	6.703791093
	O428	-2.496797923	-4.058850621	4.977917388
	O429	-3.010007452	-0.613847823	5.479862751
	O430	-3.283990427	-1.074973631	9.030554528
	O431	-0.571062837	-4.087115206	9.748537999
	O432	2.154901614	-3.700408710	6.053757616
	O433	1.899809412	0.371253169	6.614575552
	O434	0.825224006	1.903800978	9.720542704
	O435	1.511647883	-0.314945690	12.435051395

	O436 7.057288890 -10.340960496 2.033556786<br>O437 8.071947751 -7.741204773 5.756809883<br>O438 11.554528634 -4.501066015 4.551871882<br>O439 -5.637532369 -0.840126929 0.024246242<br>O440 -3.692567698 -4.417930789 -4.015012560<br>O441 -7.108017487 2.513807357 -0.426943393<br>O442 -6.335535781 3.049155065 -3.371667714<br>O443 -9.765134416 1.502154764 2.261736831<br>O444 -5.606106809 1.429020383 4.446540418<br>O445 -4.215431653 5.048422646 3.693917219<br>O446 -6.525687522 7.247287302 5.333507311<br>O447 10.811735282 -0.042249415 4.157155800<br>O448 3.578169525 4.700449317 3.934172021<br>O449 3.608285208 8.375626642 -0.679773484<br>O450 0.992478982 10.074526580 -3.532450167<br>O451 -1.420915135 7.185641703 -1.539338180<br>O452 -0.705238115 7.159673642 2.167148089<br>O453 2.424842258 5.697839475 1.445287742<br>O454 0.855501679 11.598813632 2.331020367<br>O455 -2.500139172 13.586000047 0.588906391<br>O456 -3.254199272 11.027653035 -1.349738341<br>O457 -4.958347920 13.707298241 3.616710048<br>O458 -5.486440380 6.832928742 -1.321344702<br>O459 1.755275991 -4.581006724 -8.740534306<br>O460 -0.252090905 -0.752736088 -8.602029337<br>O461 2.119639597 3.613489675 -8.558889215<br>O462 5.751552296 4.380867758 -9.182823476<br>O463 -4.986244680 -2.623226808 -8.888771089<br>O464 -1.472366441 -6.623616723 -6.010042018<br>O465 0.447738246 -5.764550548 -6.757096193<br>O466 -3.996343817 2.321133984 -8.486159915<br>O467 9.896337915 -5.407616076 -3.712337327<br>O468 12.660277548 -6.998868349 -2.131832377<br>O469 0.276921062 -6.248502707 -1.233812243<br>O470 -0.231454786 -7.375763172 0.642606518<br>O471 5.132116836 -10.853654861 -3.326361790<br>O472 2.871078410 -10.914592819 -3.284384423<br>O473 5.088374044 -5.841603171 -1.353521330<br>O474 5.040120058 -7.501111448 0.157866136<br>O475 2.579920725 -6.054887759 -2.008718214<br>O476 8.957485052 -6.552725891 1.029180579<br>O477 6.984586223 -4.551690179 0.388139743<br>O478 -4.378921905 4.735588984 -2.457772944<br>O479 7.739766316 -10.420505788 -2.531085487<br>O480 7.524858289 -8.626602786 -0.102461482<br>S481 3.541678154 4.962744070 -2.314961544<br>S482 1.988153895 1.826228151 -3.374687429<br>S483 1.947539784 -3.301143615 -3.305460146<br>S484 3.751890978 1.980358591 -0.310920343<br>S485 0.440210451 -0.883791164 -1.973319365<br>S486 3.930743344 -0.722382121 -5.521432807<br>S487 3.680184643 -3.492713183 -0.177789970<br>S488 5.564884826 1.772681081 -3.235954901<br>S489 5.635753885 -3.097712091 -3.225858844<br>S490 6.339261496 -0.830383982 -0.340563649<br>end

Fe2-brNH-NH-Fe6 35, S=3/2

Fe(139) -2.398<br>Fe(140) 0.126<br>Fe(141) -2.527<br>Fe(142) 2.542<br>Fe(143) 2.508<br>Fe(144) 2.282<br>Fe(145) 0.148	bm522brnhnhb335brte.car_3<br>C1 -7.836862957 -5.528239694 -0.072021410<br>C2 -8.051342284 -6.531562609 -1.210552898<br>C3 -8.860073676 -3.793016980 1.327919778<br>C4 -8.308613083 -4.199059722 2.691511410<br>C5 -8.022674577 -5.978082313 4.347572863<br>C6 -6.495820327 -6.040440445 4.442522844<br>C7 -4.359244365 -6.172563912 3.273704497<br>C8 -3.820266842 -4.752769081 3.464015805<br>C9 -4.231642006 -2.365521357 3.124821097<br>C10 -4.352746190 -2.001949678 4.610922018<br>C11 -5.672752131 -2.113976541 6.638062449<br>C12 -4.686289457 -2.782883405 7.603148879<br>C13 -3.621125539 -4.876146442 8.301295106<br>C14 -2.106633623 -4.961139633 8.060055767<br>C15 -0.312449945 -5.180666608 6.391111770

	C16	0.459181672	-4.047142039	5.711838885
	C17	0.268212691	-1.784220427	4.618877644
	C18	0.347798282	-0.759781172	5.806742447
	C19	-0.614637408	-1.314211085	3.455991551
	C20	-0.112960254	0.030356291	2.926709098
	C21	-0.696949869	-2.432492519	2.408434721
	C22	-0.962004495	0.451190897	7.574669012
	C23	-0.799756747	-0.079050606	9.032618099
	C24	-2.290832274	1.244900957	7.423604403
	C25	-2.456931082	2.271363803	8.554541680
	C26	-2.379740318	1.951920664	6.065622720
	C27	-1.709509818	-1.254967667	10.979308686
	C28	-0.332439017	-1.687564722	11.440397215
	C29	5.640722754	-12.040549217	2.951540173
	C30	6.289083951	-10.700724017	2.609385477
	C31	6.519332152	-8.352685582	3.418381744
	C32	7.642565195	-8.077838836	4.435529457
	C33	5.320773936	-7.411841027	3.647982798
	C34	5.682488291	-5.935823640	3.482869093
	C35	4.452851096	-5.026130420	3.490003013
	C36	4.511158008	-2.512352916	3.709349508
	C37	9.859816573	-7.078923120	4.755267496
	C38	10.021644962	-5.579937628	4.891627416
	C39	-5.183822236	-2.479931390	-2.441182893
	C40	-5.611181142	-1.109761057	-1.883320936
	C41	-3.659824232	-2.616431025	-2.215288610
	C42	-3.064424854	-4.021955539	-2.424976152
	C43	-2.637860134	-4.358487355	-3.852900123
	C44	-5.961072184	1.234004743	-2.567516285
	C45	-7.147205127	1.594402334	-1.662258606
	C46	-5.994227908	1.996891169	-3.898806629
	C47	-9.394151258	0.978713802	-0.901454394
	C48	-9.148327557	0.788546725	0.599469740
	C49	-7.600153802	0.055761963	2.353093536
	C50	-6.437312931	0.949207136	2.786462611
	C51	-4.912939698	2.790395530	2.243501181
	C52	-5.417257579	4.023037401	3.038740749
	C53	-4.191698380	3.349435017	1.007768077
	C54	-3.413845306	2.389583358	0.163693697
	C55	-2.874605489	1.141435025	0.359326266
	C56	-2.254018639	1.817914695	-1.661490916
	C57	-7.281954354	5.621915788	3.291567555
	C58	-7.314147380	5.604029609	4.817888325
	C59	-8.711472170	5.746710316	2.714331735
	C60	-9.299342264	7.127448217	2.684312350
	C61	-9.246597022	8.158879531	3.602278706
	C62	-10.440364463	8.758449440	1.833957909
	C63	8.896577643	1.251911485	4.514050513
	C64	9.949978451	0.186510728	4.276130943
	C65	7.535532240	0.640300040	4.091925125
	C66	6.373291152	1.581065037	4.302820799
	C67	5.639353368	1.587038217	5.502969757
	C68	5.991304476	2.487711453	3.297079951
	C69	4.558141353	2.456508312	5.694120122
	C70	4.930321167	3.374596626	3.480711293
	C71	4.204373507	3.350324423	4.674698290
	C72	5.426579687	6.971672567	-0.130337044
	C73	4.375181475	8.081531785	-0.306999480
	C74	4.751647928	5.609064953	0.017226693
	C75	3.489145931	9.837518055	-1.798497484
	C76	2.041101321	9.481962363	-2.142667534
	C77	0.454573041	7.583329051	-2.478805751
	C78	-0.140902483	7.059272099	-1.146891740
	C79	0.594946743	6.469371610	-3.541487925
	C80	-0.620774740	5.602205312	-3.887487055
	C81	-1.698263115	6.239150910	-4.778944626
	C82	-3.266927209	8.084794036	-4.198190405
	C83	0.210955434	5.893631171	0.981148471
	C84	-0.100585540	6.987750939	2.012437469
	C85	1.193851585	4.896841032	1.632129231
	C86	0.833485037	8.953814833	3.133200391
	C87	0.218344948	10.329979949	2.936182821
	C88	-1.007688099	11.801555937	1.425365878
	C89	-2.345941003	12.295435548	2.026597797
	C90	-0.991521542	11.985473370	-0.094561810
	C91	-2.083360042	11.213971593	-0.825996966
	C92	-4.424020495	11.666478880	3.176690872
	C93	-4.455025694	12.627839825	4.357579779
	C94	-5.121174770	10.331012042	3.565735177
	C95	-5.235697335	9.381874671	2.394182105

	C96	-6.332987997	9.457281675	1.522721646
	C97	-4.232179817	8.439359197	2.112811162
	C98	-6.412744790	8.644561984	0.390097306
	C99	-4.280330335	7.637757857	0.969043500
	C100	-5.374417551	7.754133357	0.115350988
	C101	5.008166941	-2.935093354	-8.798019344
	C102	3.531081799	-2.737887852	-8.511367410
	C103	1.886184021	-1.324930743	-7.294663544
	C104	1.455452214	0.077373448	-7.742227879
	C105	1.822094292	2.485255465	-7.387147115
	C106	3.029831544	3.412760767	-7.279287563
	C107	5.278671721	3.856364410	-6.470621743
	C108	5.852496759	4.396845425	-7.765898328
	C109	-1.743975658	-2.449262443	-9.673074253
	C110	-2.799099854	-1.389303377	-9.335725546
	C111	-1.395275750	-3.271490665	-8.413740518
	C112	-0.649072041	-4.565908554	-8.788009556
	C113	-0.113312254	-5.240237754	-7.546902893
	C114	-3.272260684	0.862359824	-8.515412164
	C115	-2.746237994	2.196803736	-9.002637715
	C116	-3.797175147	0.858320063	-7.060662995
	C117	-2.664421612	0.774439963	-6.079566929
	C118	-1.954113345	1.926858863	-5.707792940
	C119	-2.205002872	-0.480735519	-5.642929617
	C120	-0.770226161	1.817628278	-4.971173851
	C121	-1.028319657	-0.585695355	-4.899911996
	C122	-0.290272330	0.561394730	-4.592281870
	C123	8.402415951	-5.714798155	-4.677530210
	C124	9.480419952	-5.878397745	-3.594694854
	C125	7.027663145	-6.084072709	-4.096606280
	C126	5.833208241	-5.786802503	-4.946909608
	C127	5.576556973	-6.240450640	-6.224661487
	C128	3.773472986	-5.226091170	-5.434252547
	C129	10.525615164	-7.247930922	-1.893307934
	C130	11.934677967	-6.912486877	-2.330036715
	C131	0.703242986	-6.827842603	-0.831527930
	C132	2.161918723	-6.889547968	-0.386471718
	C133	3.192475342	-6.903801455	-1.553263011
	C134	3.184590842	-8.286676833	-2.261112975
	C135	4.314084089	-8.508185436	-3.270589476
	C136	4.402046252	-9.942558119	-3.839423115
	C137	4.607684344	-6.678274276	-0.995484155
	C138	3.728862740	-0.469054340	-1.788480669
	Fe139	4.005564822	-1.563845332	-3.427418690
	Fe140	2.217170157	0.743583095	-0.902646177
	Fe141	5.111222832	0.630512889	-0.831856713
	Fe142	3.964256243	0.924750136	-3.128248811
	Fe143	4.043623533	2.934775022	-1.430169030
	Fe144	5.000711812	-1.884301403	-1.119721621
	Fe145	2.309411398	-2.122335073	-1.197068233
	H146	-6.259555117	-7.498555756	-1.082684207
	H147	-6.288099800	-6.333800133	-2.223353927
	H148	-9.823431138	-4.990921285	-0.153265448
	H149	-8.661992007	-6.048575639	-1.990642071
	H150	-8.667373604	-7.355573727	-0.812226117
	H151	-8.208146088	-2.990156852	0.953655287
	H152	-9.035123730	-6.096534772	2.455244458
	H153	-9.863814987	-3.371497922	1.475104408
	H154	-8.425141421	-6.987722102	4.504116062
	H155	-6.309764803	-6.225663004	2.392153487
	H156	-8.362715837	-5.345517503	5.178652553
	H157	-3.212205997	-2.123363055	2.810509200
	H158	-5.359859961	-4.001829771	2.329343837
	H159	-3.998349172	-6.578504203	2.318904132
	H160	-5.578454281	-1.030112642	6.787663082
	H161	-6.217154482	-2.884679480	4.702459205
	H162	-3.950520974	-6.784234000	4.087252765
	H163	-4.928391986	-1.755631859	2.535846459
	H164	-4.913881763	-4.580535853	6.620569389
	H165	-6.693195719	-2.414800074	6.913303747
	H166	-1.274632220	-3.285668356	2.786390297
	H167	-1.178536989	-2.080029975	1.482027876
	H168	0.305695323	-2.790879379	2.145355659
	H169	-1.623996630	-1.147855337	3.867482024
	H170	-4.007209926	-5.906292609	8.295568571
	H171	-2.366290284	1.244766604	5.226153369
	H172	-2.517010181	-1.281311866	8.994570205
	H173	-2.474151491	1.806498015	9.550546757
	H174	0.957335998	0.009082891	2.675973810
	H175	-0.271369857	0.829860788	3.666964650

	H176	8.861733922	1.450890093	5.602280076
	H177	-3.320525728	2.518858515	6.002891866
	H178	-3.404733987	2.813920931	8.421499744
	H179	-1.548887809	2.665748484	5.940870180
	H180	-1.639961674	3.009747972	8.538046550
	H181	-3.125198939	0.523204724	7.489531372
	H182	-0.117482267	1.145917775	7.463063987
	H183	-1.678993643	-0.960686563	6.144444847
	H184	-0.660028959	0.314863353	2.019167468
	H185	1.609505681	4.248022799	0.846326103
	H186	0.611133340	4.274227698	2.325488722
	H187	2.423244648	5.083895693	3.158255000
	H188	2.685586449	4.083237111	5.637269654
	H189	4.632134257	4.057979358	2.685779261
	H190	6.514198562	2.481461202	2.337624498
	H191	5.907128442	0.892897646	6.304548768
	H192	-1.280419769	-3.137234839	5.209705070
	H193	-0.258552629	-6.056318186	5.723760246
	H194	0.238465653	-5.427167693	7.307274995
	H195	-2.387089713	-4.911448066	6.007592690
	H196	-3.752173252	-4.468080062	9.311073928
	H197	3.985759832	2.427566633	6.624894799
	H198	1.296412903	-1.951875037	4.280954377
	H199	-2.054645085	-0.409110597	11.594981759
	H200	-2.393207173	-2.103758768	11.135694749
	H201	0.287641169	-2.187010431	10.662012416
	H202	10.799064044	-7.518392115	4.385590732
	H203	-1.286796806	-5.481671486	-4.905464957
	H204	1.458033780	-5.833784475	-2.296528443
	H205	-1.349239255	-5.256307364	-9.279756944
	H206	0.170997453	-4.346666739	-9.487382885
	H207	-2.314096235	-3.535631246	-7.868328735
	H208	-2.065570701	2.129653308	-9.883875204
	H209	-2.275867190	-3.123642844	-10.361332669
	H210	-3.141488788	-1.887842674	-2.860084804
	H211	-3.791481671	-4.791919542	-2.112817095
	H212	-1.336639855	-0.048066693	-8.716604564
	H213	-4.151655778	0.681009659	-9.167848375
	H214	0.000177007	-1.321895058	-9.831566471
	H215	-1.046127716	-5.519315536	-3.163411567
	H216	-0.792756749	-2.653679300	-7.730219898
	H217	-0.068381312	-2.618291776	-10.849419537
	H218	-6.424151692	-3.095259497	-3.973708338
	H219	-3.463367476	-2.323050433	-1.173152670
	H220	1.630935964	-4.428126316	-7.934281433
	H221	-2.199461263	-4.145428742	-1.756460388
	H222	-4.207889105	2.275644712	2.911269893
	H223	-2.939530334	0.472879782	1.208579062
	H224	-8.465173479	0.340653019	2.969369030
	H225	-7.334381586	-0.173584520	0.250873657
	H226	-7.286659362	3.753853182	2.239421652
	H227	-8.756519011	8.258117198	4.564427771
	H228	-7.484413837	4.591396418	5.257247124
	H229	-9.377364890	5.042966192	3.244847289
	H230	-6.664570464	6.473918833	2.965291198
	H231	-11.047508975	9.382597809	1.184051408
	H232	-3.498561736	4.126536261	1.369948433
	H233	-4.933867398	3.855322462	0.369358840
	H234	-10.132944356	10.101584092	3.456649686
	H235	-8.675891934	5.406148716	1.667721175
	H236	-6.388366878	1.921281189	0.943601242
	H237	-7.347418429	-0.981421486	2.618626710
	H238	-9.800113580	1.993001659	-1.018161683
	H239	-1.784357439	1.837699182	-2.642048272
	H240	-10.170980408	0.270995648	-1.217985235
	H241	10.004743557	2.928501691	4.113482284
	H242	8.429910699	3.071299771	3.700753533
	H243	1.643856428	6.122337255	-0.607118950
	H244	-0.737024473	5.367424172	0.799654030
	H245	1.576927800	7.990208382	1.404154954
	H246	-3.326688971	3.663740262	-1.575621741
	H247	-4.882166121	4.474018823	-2.803070747
	H248	-6.263196117	3.853545096	-4.469282878
	H249	-4.666727129	5.591054755	-1.684471715
	H250	-5.109182425	1.705070205	-4.487581376
	H251	-6.899735078	1.722950697	-4.461178453
	H252	0.008163881	11.751195343	-0.486346915
	H253	-1.177258179	13.053005037	-0.291169186
	H254	-0.701659829	10.523810764	-2.213678288
	H255	-2.381982908	10.030009323	-2.434220741

	H256	-3.242680876	9.796236867	-5.320791837
	H257	-2.143176157	5.473805877	-5.433371049
	H258	-7.280834675	8.703082438	-0.272189242
	H259	-3.388031335	8.305644393	2.795002759
	H260	-4.381970129	8.076042821	-2.465394222
	H261	1.391017452	5.788546032	-3.199562263
	H262	2.566042688	7.519243195	-2.191033723
	H263	-1.083259907	5.184856123	-2.979029754
	H264	-8.184080490	-0.015325697	-2.371031980
	H265	3.902241558	10.414140175	-2.636363822
	H266	-5.754384810	-0.584755110	-3.800119951
	H267	0.972823173	6.942769813	-4.462701496
	H268	-3.460936452	6.954015597	0.742366640
	H269	3.443418666	10.502251629	-0.923345777
	H270	-2.506098441	8.428883333	-6.080107709
	H271	-4.744584725	9.353264171	-3.575079539
	H272	-4.799031269	-3.098648385	-4.365931055
	H273	-5.687089806	-3.232068637	-1.805547815
	H274	-7.143772892	10.160748752	1.728028563
	H275	-5.077617406	1.555427814	-1.996891577
	H276	-0.221820210	8.374742680	-2.818216278
	H277	-1.232903105	6.990305894	-5.430275781
	H278	-0.225598531	4.744829850	-4.449080638
	H279	-3.262945417	6.247419800	-3.326269323
	H280	0.338719917	8.486022328	3.997659318
	H281	-0.613530013	9.698444611	1.180570381
	H282	-0.267506721	12.499831725	1.846580131
	H283	1.889655368	9.110299285	3.393738471
	H284	-2.746702252	10.428280684	2.705169509
	H285	5.527681955	-1.965465483	-8.781814369
	H286	5.398093157	-3.524254437	-7.941495022
	H287	8.410909982	-4.644092911	-4.931844616
	H288	6.162998555	-3.894401567	-10.175825166
	H289	4.610840372	-4.413567775	-10.139120022
	H290	5.541724023	4.860175099	0.156702169
	H291	5.634120818	7.366353884	1.861209803
	H292	5.056546905	8.447375699	-2.214859904
	H293	4.091130106	5.613587719	0.895512856
	H294	6.794959670	8.066073324	0.944210389
	H295	6.075147396	3.287774494	-5.966505614
	H296	6.091419164	6.951361881	-1.008065370
	H297	4.907620774	-7.600878243	4.652921398
	H298	6.897242327	-8.213325570	2.397987269
	H299	4.222622227	2.011057450	-6.295967489
	H300	4.751277009	-0.472077975	3.635188247
	H301	1.446487901	2.544308909	-8.416103667
	H302	-0.674373564	-1.558673362	-4.552394471
	H303	1.041688824	2.916652947	-6.737828440
	H304	3.980181143	-5.013964158	4.479050110
	H305	2.571180266	0.826483277	-6.216892470
	H306	3.701992154	-5.389420381	2.768527608
	H307	4.550232601	-7.691181917	2.912178014
	H308	6.209007667	-5.795199932	2.527354723
	H309	6.367504167	-5.608621453	4.280812367
	H310	-4.472402943	-0.002777427	-6.951039733
	H311	5.564367003	-10.047829705	4.355511635
	H312	4.799720912	-12.154424012	2.250144659
	H313	0.630223989	0.466948854	-4.018202322
	H314	5.844056831	-12.519537625	4.945553552
	H315	4.322433158	-12.727797533	4.375959256
	H316	5.381248008	-3.600583704	2.243578578
	H317	-0.202678541	2.707257907	-4.692701943
	H318	-2.767195052	-1.382259886	-5.897807921
	H319	7.896014276	-9.673378020	-2.017169291
	H320	7.017279635	-7.165328960	-3.874888185
	H321	6.698824402	-8.120400801	-0.346790083
	H322	7.158228989	-10.131219184	-3.305102463
	H323	5.651078148	3.769795338	-8.667552454
	H324	6.444970115	-5.005345511	-0.161854785
	H325	2.301047375	-7.752654014	0.274868466
	H326	3.988743561	-1.085999879	-7.402753172
	H327	3.262560130	-9.042062401	-1.463349430
	H328	3.760643751	-6.111922947	-7.351446473
	H329	2.754639209	-4.865289035	-5.388684043
	H330	6.371995942	-12.824490380	2.692824799
	H331	5.536698674	-1.325271364	2.333033813
	H332	8.299310328	-5.888042572	0.994870132
	H333	7.303304712	-3.761932293	0.197718548
	H334	8.761100548	-7.196929513	2.912496230
	H335	3.119150048	-3.222146552	5.095269904

H336	4.181410768	-7.825358181	-4.123955562
H337	5.284812579	-8.265735077	-2.816587869
H338	2.208183439	-8.431215204	-2.747903510
H339	1.863256245	-1.404911310	-6.196191555
H340	-2.315595227	2.904106978	-6.035369662
H341	-4.385907323	1.778549840	-6.926943482
H342	2.329292660	-5.980359004	0.216393322
H343	8.491265043	-7.386622059	0.621276456
H344	7.388685488	-9.311575992	0.353907866
H345	3.523049452	-1.564749534	5.210479420
H346	6.192842238	-6.808514765	-6.911786611
H347	6.904167784	-5.581107549	-3.130833864
H348	1.192183815	-2.049275793	-7.725670035
H349	4.998280069	4.699831628	-5.820557722
H350	7.386710613	-0.280279961	4.675850469
H351	7.599139034	0.356682604	3.028674043
H352	9.616528829	-6.161736026	-6.267683643
H353	8.799491423	-7.461711520	-5.704556353
H354	10.519506765	-8.308468483	-1.596063466
H355	8.931625852	-7.864344875	-3.167176627
H356	12.125100106	-7.021911562	-3.424834989
H357	10.262399828	-6.654286830	-1.003729368
H358	10.276102649	0.099365734	3.211013438
H359	-3.495164260	12.722191061	4.918097214
H360	9.673850840	-7.480518242	5.766892571
H361	9.079417047	-4.992304370	4.760424212
H362	-4.985415317	12.139603726	2.353942160
H363	-4.555219062	9.859613287	4.384487734
H364	-6.117328313	10.582135165	3.956389500
H365	1.277809794	-3.196333719	-1.046782258
H366	3.322467242	-0.899706725	0.830325165
H367	-6.306279797	6.999308733	-1.416426006
H368	0.652102839	-1.462995759	0.373861284
H369	-1.470485162	0.033877655	-0.914195951
Mo370	4.067156066	-4.084277091	-2.417457943
N371	-6.848351183	-7.083047457	-1.809361204
N372	-8.956627810	-4.883030670	0.367844022
N373	-8.528397343	-5.475974668	3.082103593
N374	-5.813576814	-6.233157561	3.285685990
N375	-4.524734678	-3.766215409	2.860588051
N376	-5.460639968	-2.456542283	5.241969436
N377	-4.406018013	-4.089492075	7.362319264
N378	-1.693173309	-4.919319713	6.760127766
N379	-0.265752898	-3.055694157	5.152085537
N380	-0.812887745	-0.556510397	6.509625732
N381	-1.721527070	-0.940738028	9.543469058
N382	5.136888914	-12.113954913	4.329120025
N383	6.085616853	-9.736575823	3.528838107
N384	4.840504816	-3.663440323	3.109409483
N385	3.796638332	-2.478595081	4.851299365
N386	4.935034553	-1.354013926	3.166578637
N387	8.730439216	-7.447198221	3.925072511
N388	-5.544764231	-2.587728033	-3.859307433
N389	-1.590242762	-5.196954623	-3.968629729
N390	-5.864028399	-0.188233810	-2.851160867
N391	-8.235565391	0.797126529	-1.759874550
N392	-7.996841856	0.158135497	0.962026291
N393	-5.972660858	1.860970655	1.878171770
N394	-3.011866005	2.778436269	-1.109309808
N395	-2.169478416	0.810869812	-0.784917637
N396	-6.707314641	4.373950782	2.799563334
N397	-10.048039573	7.515390147	1.583617137
N398	-9.977030682	9.186204503	3.042363046
N399	9.221327170	2.421877365	3.693851893
N400	6.255932747	7.203785696	1.062394264
N401	4.368619895	8.716547096	-1.516422279
N402	1.778832776	8.166339011	-2.293584429
N403	-2.783050814	6.859536894	-4.011899393
N404	-2.881053357	8.851317351	-5.237640081
N405	-4.096290136	8.628844493	-3.277626576
N406	0.708068387	6.420758876	-0.291427832
N407	0.741529386	8.060952891	1.991512907
N408	-0.589695138	10.474007437	1.842200793
N409	-1.699357625	10.651728798	-2.003792908
N410	-3.057595363	11.395518264	2.750978427
N411	5.195015343	-3.573098724	-10.103314470
N412	3.221430843	-1.641890791	-7.785923573
N413	2.043341000	1.087718165	-7.066058953
N414	4.163092536	2.951290059	-6.695399499
N415	-0.626820042	-1.872174362	-10.429949881

N416	-2.341524768	-0.201270420	-8.844466973
N417	8.704790952	-6.462549118	-5.911414466
N418	4.690495615	-5.143919227	-4.469694496
N419	4.279779338	-5.872662791	-6.510438536
N420	9.567061810	-7.084755687	-2.970989128
N421	0.102601115	2.302836887	0.416507036
N422	2.407761414	-0.790355396	0.363409880
N423	0.921850436	1.697752041	-0.064971939
N424	1.588697174	-1.773397857	0.705343653
O425	-6.728034335	-5.326668098	0.453348289
O426	-7.698765353	-3.372484777	3.394884775
O427	-5.935775722	-5.935272306	5.546352888
O428	-2.802840664	-4.523594741	4.142900442
O429	-3.479496426	-1.343030814	5.196704473
O430	-4.224479398	-2.153120260	8.570582512
O431	-1.343209817	-5.117756846	9.022276358
O432	1.704838354	-4.122210143	5.691754519
O433	1.422726740	-0.204223605	6.079661531
O434	0.124102862	0.357052644	9.743023562
O435	0.054162118	-1.576314328	12.595165979
O436	6.916975906	-10.569069335	1.538229168
O437	7.519133552	-8.405247847	5.628683786
O438	11.083576693	-5.030672387	5.138826559
O439	-5.687068358	-0.863677473	-0.664186641
O440	-3.239027403	-3.905512449	-4.854045069
O441	-7.080800687	2.558872599	-0.879221804
O442	-5.989778895	3.415004006	-3.644000329
O443	-9.991924300	1.203816226	1.405015196
O444	-5.972401602	0.841607541	3.926370746
O445	-4.656107442	4.674322264	3.759474235
O446	-7.206084805	6.594910016	5.519905101
O447	10.403560674	-0.544488486	5.144520298
O448	3.137057730	4.220723450	4.784181436
O449	3.585343107	8.394070039	0.600434808
O450	1.203096428	10.394457587	-2.272384341
O451	-1.343144508	7.233884195	-0.893824949
O452	-1.017993630	6.844417058	2.829834040
O453	2.252465365	5.565987236	2.323754998
O454	0.436975827	11.243931143	3.740493254
O455	-2.692099929	13.477451966	1.889550749
O456	-3.246386439	11.159700285	-0.400711674
O457	-5.459799181	13.235058595	4.691442646
O458	-5.396315159	6.977951264	-1.061641947
O459	2.683884113	-3.575689916	-8.902442433
O460	0.650663962	0.250827090	-8.680513370
O461	2.932072875	4.578962628	-7.697117929
O462	6.527388062	5.411465213	-7.828504979
O463	-4.011559481	-1.615836462	-9.484534287
O464	-0.799750529	-5.930359402	-6.801449432
O465	1.179666058	-5.002918671	-7.243087981
O466	-3.066882009	3.281125705	-8.535436171
O467	10.266914391	-4.954673082	-3.307025655
O468	12.833553486	-6.612529979	-1.556136719
O469	0.479188265	-6.109333770	-1.940915694
O470	-0.213327519	-7.344465039	-0.196180798
O471	5.577542123	-10.427579727	-4.001545522
O472	3.321369571	-10.523550521	-4.137276985
O473	5.274352044	-5.666192902	-1.494380227
O474	5.086011739	-7.445132292	-0.140817133
O475	2.853568388	-5.832847125	-2.435430394
O476	8.873026630	-6.667313630	1.196022569
O477	6.987136614	-4.608219065	0.566061258
O478	-4.193089534	5.014524196	-2.332115189
O479	8.076524454	-10.049312220	-2.907809303
O480	7.569513844	-8.596260362	-0.308608561
S481	3.730980825	5.161133807	-1.462712622
S482	2.259838908	2.298839920	-2.641228288
S483	2.216699944	-2.936809182	-3.342505142
S484	3.823064442	1.870012508	0.487562899
S485	0.594646093	-0.677964869	-1.767034460
S486	4.304468074	-0.096601407	-5.049887573
S487	3.764403391	-3.514430737	-0.176917454
S488	5.820607021	2.003468556	-2.454015318
S489	5.894068979	-2.679415767	-2.991078816
S490	6.425045357	-0.821421604	0.166097895
end			

Fe2-brNH-NH-Fe6H-3b3 to Fe2-brNH-NH-Fe6H-3b2

35, S=1

reactant

Fe(139)	-2.288	bm522brnhnh63b32td.car_2			
Fe(140)	0.088	C1	-7.711444129	-5.271926332	2.230886154
Fe(141)	-2.578	C2	-8.169882775	-6.500494652	1.436003191
Fe(142)	2.578	C3	-8.427653486	-3.179944621	3.306693774
Fe(143)	2.457	C4	-7.740226296	-3.286501754	4.665107096
Fe(144)	2.506	C5	-7.252467444	-4.652388155	6.629760828
Fe(145)	0.108	C6	-5.728135293	-4.749737264	6.506318153
		C7	-3.821636055	-5.221216427	5.053781314
		C8	-3.292303262	-3.793526120	4.899373959
		C9	-3.740511204	-1.552199767	3.985844031
		C10	-3.585869561	-0.789744398	5.308793804
		C11	-4.418359878	-0.336157387	7.530468970
		C12	-3.268200981	-0.821712252	8.424768590
		C13	-2.268958057	-2.738133339	9.573612623
		C14	-0.821338488	-3.007209011	9.138537378
		C15	0.650796567	-3.863830889	7.362655492
		C16	1.314888548	-2.888724164	6.387301806
		C17	0.950831927	-1.003865675	4.811164345
		C18	1.379267719	0.291592190	5.532095680
		C19	-0.093234767	-0.772172168	3.710511107
		C20	0.365642901	0.374889885	2.803804804
		C21	-0.360517669	-2.092891321	2.978001209
		C22	0.600760694	2.218267355	6.885845805
		C23	1.033527364	1.968340553	8.343889602
		C24	-0.648969743	3.128475584	6.785493340
		C25	-0.506909270	4.380512995	7.659025917
		C26	-0.921618576	3.518831577	5.326821469
		C27	0.582665500	0.892901732	10.476904409
		C28	2.036838644	0.556066168	10.732506913
		C29	6.215030596	-11.147318086	4.723402631
		C30	6.728469948	-9.935853554	3.947085514
		C31	7.019785859	-7.466970395	4.110969344
		C32	8.268412709	-7.007418312	4.886466396
		C33	5.876417127	-6.447495849	4.254787366
		C34	6.263796815	-5.055648053	3.756751230
		C35	5.054190265	-4.132229855	3.650166473
		C36	5.081640554	-1.630332962	3.431323750
		C37	10.525348398	-6.076986189	4.689461810
		C38	10.757262500	-4.594564158	4.502988817
		C39	-5.449719168	-2.894598663	-1.048133898
		C40	-5.841077470	-1.444029660	-0.719084366
		C41	-3.906577430	-2.985258688	-1.045385569
		C42	-3.315183947	-4.409617660	-1.044942951
		C43	-3.111726348	-5.049194081	-2.417252717
		C44	-6.412425361	0.699638690	-1.760766687
		C45	-7.560166242	1.197970791	-0.882525561
		C46	-6.560537727	1.300566197	-3.174109309
		C47	-9.572722150	0.686500619	0.394896263
		C48	-9.096333103	0.935390654	1.831088785
		C49	-7.271846577	0.703678700	3.458883661
		C50	-6.071435137	1.646757644	3.500531205
		C51	-4.622129393	3.222268680	2.307411324
		C52	-5.040654762	4.597889497	2.890083214
		C53	-4.117357128	3.492722384	0.883643820
		C54	-3.436996627	2.376639901	0.151620374
		C55	-2.824319828	1.200523191	0.511971584
		C56	-2.553520707	1.442819471	-1.675327434
		C57	-6.882951671	6.243705691	3.009804919
		C58	-6.747402976	6.524719752	4.504585637
		C59	-8.367356098	6.253656746	2.580135112
		C60	-8.996889302	7.600656147	2.374312193
		C61	-8.864697385	8.782448936	3.077918243
		C62	-10.289460795	9.037674192	1.400263475
		C63	9.292507225	1.963942604	3.001954013
		C64	10.298216483	0.834798040	2.871219937
		C65	7.884456689	1.328041004	2.904978792
		C66	6.770063451	2.334188745	3.075509534
		C67	6.224094097	2.612396570	4.342631017
		C68	6.244289406	3.024359864	1.967924785
		C69	5.186950928	3.537602940	4.503108752
		C70	5.223677373	3.963234312	2.115218674
		C71	4.685338034	4.209366069	3.381054538
		C72	5.185223564	6.698452255	-2.209878257
		C73	4.119571418	7.777436634	-2.471510256

C74	4.534430999	5.434146591	-1.652790718
C75	3.023080234	9.189795280	-4.180863753
C76	1.545630364	8.790362953	-4.236661063
C77	-0.047703203	6.896489615	-3.910128091
C78	-0.463379665	6.687259407	-2.429699281
C79	-0.024751339	5.575249271	-4.713172338
C80	-1.268706762	4.682317343	-4.723161259
C81	-2.436862454	5.132723158	-5.614599584
C82	-3.976066509	7.064470144	-5.258101218
C83	0.169528213	6.007860889	-0.158251684
C84	0.029461781	7.325578163	0.616462904
C85	1.225589710	5.148138820	0.570580877
C86	1.140519994	9.457414859	1.077560590
C87	0.449120385	10.746089699	0.664377832
C88	-1.031640389	11.817964783	-0.943262309
C89	-2.281149634	12.434620424	-0.268324979
C90	-1.245722580	11.659213456	-2.450407036
C91	-2.406030417	10.744700513	-2.820959229
C92	-4.163909881	12.093703175	1.262981361
C93	-4.035903207	13.293980259	2.190289988
C94	-4.786039197	10.894288599	2.032325539
C95	-5.063076765	9.716617861	1.125046764
C96	-6.272033548	9.633297747	0.417714218
C97	-4.102096239	8.715135650	0.912037288
C98	-6.505096963	8.604547768	-0.497248615
C99	-4.305265580	7.693271966	-0.020104363
C100	-5.509174001	7.653819131	-0.718094067
C101	3.705148892	-4.690459505	-8.680731422
C102	2.288919704	-4.433064671	-8.197385396
C103	0.842161882	-2.807703132	-6.991923602
C104	0.313945020	-1.527532716	-7.650811851
C105	0.685388642	0.886236456	-7.910119004
C106	1.842525056	1.854882559	-8.133796777
C107	4.136329138	2.538112757	-7.697988834
C108	4.676719101	2.631062080	-9.112444196
C109	-3.138801711	-4.360312384	-8.572356161
C110	-4.151877948	-3.242897719	-8.297154723
C111	-2.590445249	-4.915655244	-7.239462235
C112	-1.892893608	-6.272984045	-7.447907255
C113	-1.119565526	-6.669581227	-6.211709275
C114	-4.536054031	-0.865625364	-7.880823285
C115	-4.080467343	0.340238102	-8.678279677
C116	-4.878383313	-0.572229137	-6.398746067
C117	-3.612708075	-0.464327352	-5.600974062
C118	-2.887106810	0.736714510	-5.567538366
C119	-3.032227009	-1.628803237	-5.066042900
C120	-1.568929749	0.747875346	-5.098205905
C121	-1.720622002	-1.612540688	-4.591980844
C122	-0.971840910	-0.432406393	-4.648809138
C123	7.692198122	-6.642207781	-4.525160063
C124	8.909061066	-6.627324693	-3.583949480
C125	6.411562073	-7.019811017	-3.765586242
C126	5.125333013	-6.844751985	-4.507400065
C127	4.693469195	-7.509733388	-5.635122517
C128	3.044179140	-6.244784843	-4.867399913
C129	10.213225385	-7.688231978	-1.835838659
C130	11.535445174	-7.508275372	-2.548574723
C131	0.661943984	-6.913165538	0.425856897
C132	2.158440344	-7.061570996	0.689996734
C133	3.058103313	-7.269380768	-0.567283764
C134	2.986957084	-8.758725052	-0.992885315
C135	3.972878141	-9.168000407	-2.085606813
C136	3.987087624	-10.684305807	-2.375389460
C137	4.517670398	-6.925490540	-0.220622713
C138	3.422924073	-0.909977930	-2.163070167
Fe139	3.563243899	-2.308022505	-3.545266257
Fe140	1.971429749	0.610647462	-1.339049815
Fe141	4.848158233	0.387362246	-1.582846413
Fe142	3.486854030	0.199755225	-3.769088293
Fe143	3.695310482	2.534974405	-2.474100910
Fe144	4.754219993	-2.133492870	-1.356312785
Fe145	2.015122316	-2.129138465	-1.121438357
H146	-6.393277245	-7.502082457	1.399971297
H147	-6.690326093	-6.656225772	0.035234252
H148	-9.659781206	-4.619240633	2.325475432
H149	-8.942965764	-6.187920773	0.715875814
H150	-8.677045428	-7.166957249	2.155058332
H151	-7.773281642	-2.561847563	2.674358175
H152	-8.516642692	-5.158148208	4.961057539
H153	-9.374961802	-2.641897991	3.445171923

H154	-7.631069531	-5.583926614	7.071004016
H155	-5.888958042	-5.427261487	4.563400636
H156	-7.459986894	-3.831463473	7.329162223
H157	-2.802436138	-1.430220633	3.434280152
H158	-4.904048433	-3.328949616	3.717647164
H159	-3.643371659	-5.777986774	4.123775275
H160	-4.258701945	0.734480430	7.350954770
H161	-5.328187174	-1.613476216	6.042675161
H162	-3.252795361	-5.698361560	5.860546514
H163	-4.542826257	-1.095270932	3.390806194
H164	-3.834442382	-2.783718593	8.109699778
H165	-5.365858487	-0.471914486	8.068954833
H166	-0.808839967	-2.832949111	3.652450394
H167	-1.058067524	-1.940011343	2.137832758
H168	0.566744925	-2.505881024	2.560504985
H169	-1.026902549	-0.450995784	4.197618422
H170	-2.697074232	-3.693935544	9.909646911
H171	-1.083018187	2.642360127	4.684772448
H172	-0.576101117	0.690402675	8.707769623
H173	-0.364128441	4.134842974	8.721143085
H174	-0.294567165	0.467431318	1.930776312
H175	1.384739787	0.218702900	2.428999980
H176	9.403732218	2.391025065	4.017272442
H177	-1.820479893	4.149719724	5.264447328
H178	-1.412425012	4.998318093	7.572611807
H179	-0.075039621	4.095432598	4.919296625
H180	0.351605932	4.989744335	7.333764228
H181	-1.511559894	2.545516935	7.158013799
H182	1.460087272	2.720583252	6.418721375
H183	-0.578650678	0.627204705	6.066006829
H184	0.345353321	1.339085644	3.332398827
H185	1.529351254	4.323761262	-0.090895631
H186	0.737231651	4.720205822	1.458027442
H187	2.673763818	5.608617246	1.822986449
H188	3.354915271	5.207622712	4.384137488
H189	4.812890574	4.476801513	1.246676436
H190	6.622251102	2.807661785	0.966229848
H191	6.604298423	2.087293038	5.222585024
H192	-0.504301313	-2.052795840	5.976907809
H193	0.576805856	-4.835111463	6.848628100
H194	1.334089201	-3.984602980	8.212405899
H195	-1.463690069	-3.613296625	7.259821780
H196	-2.212031467	-2.064252832	10.437902752
H197	4.757713513	3.717993458	5.492596362
H198	1.866341485	-1.402134642	4.353919096
H199	0.303374276	1.766088348	11.089751950
H200	-0.012343288	0.031396952	10.819225379
H201	2.559375220	0.039278606	9.894198073
H202	11.388967517	-6.634421781	4.294895994
H203	-1.890386642	-6.348421848	-3.422191107
H204	1.278337033	-6.280664853	-1.291750681
H205	-2.651048654	-7.044995565	-7.640067156
H206	-1.218934897	-6.226010314	-8.316508630
H207	-3.411315554	-5.044365857	-6.516779756
H208	-3.467196824	0.091803958	-9.576793761
H209	-3.751724890	-5.151341433	-9.030953340
H210	-3.518142009	-2.414321750	-1.904522371
H211	-3.963449404	-5.089929167	-0.465782235
H212	-2.636235642	-1.830047279	-8.153621870
H213	-5.489227475	-1.153868638	-8.371595046
H214	-1.448410346	-3.314274120	-9.185069977
H215	-1.362538719	-6.001222973	-1.781910641
H216	-1.900009408	-4.179501991	-6.799481620
H217	-1.647428483	-4.774840044	-9.921018610
H218	-6.904199236	-3.790568499	-2.206152416
H219	-3.555534858	-2.478110880	-0.134551566
H220	0.500190026	-5.936904418	-7.055713318
H221	-2.351489336	-4.394450737	-0.514522062
H222	-3.807905855	2.862713093	2.952075986
H223	-2.720633003	0.727800943	1.480574173
H224	-8.048777658	1.150251288	4.095865948
H225	-7.300276012	-0.049695932	1.460572186
H226	-6.979756608	4.213331999	2.340633956
H227	-8.262297363	9.060804795	3.935675468
H228	-6.885074875	5.623084330	5.150197384
H229	-8.953488252	5.650307097	3.296631886
H230	-6.317193890	7.010601103	2.457352300
H231	-10.999402783	9.525647178	0.736889039
H232	-3.408319610	4.334380852	0.956337509
H233	-4.962876485	3.849307282	0.272490692

	H234	-9.823733864	10.657010450	2.701812421
	H235	-8.431085444	5.729831148	1.612930129
	H236	-6.235566052	2.059702081	1.477112359
	H237	-6.975542190	-0.244085806	3.934808802
	H238	-10.066323587	1.611199100	0.066592268
	H239	-2.236021657	1.264551495	-2.700185219
	H240	-10.321879705	-0.114809070	0.406756429
	H241	10.350363789	3.475903914	2.112097846
	H242	8.736434783	3.589951225	1.890331053
	H243	1.371211863	5.820347640	-1.937937286
	H244	-0.801151836	5.496618458	-0.084505932
	H245	1.616302611	8.073710307	-0.446068423
	H246	-3.639870234	3.281771637	-1.796872826
	H247	-5.320500063	3.885897608	-2.936618226
	H248	-7.177613351	2.962085307	-2.338640756
	H249	-4.956388927	5.200421946	-2.112631813
	H250	-5.685306584	1.030882800	-3.780934914
	H251	-7.465105629	0.895747356	-3.657261773
	H252	-0.307465092	11.338066414	-2.924587425
	H253	-1.487124430	12.657135280	-2.848920459
	H254	-1.224729877	9.788793467	-4.228428596
	H255	-2.895506380	9.244186748	-4.075508686
	H256	-4.106879409	8.519834691	-6.691923417
	H257	-2.934916390	4.253003178	-6.049566678
	H258	-7.458871134	8.543133477	-1.028457680
	H259	-3.165648587	8.715401661	1.476735307
	H260	-4.914381229	7.394945590	-3.451278946
	H261	0.809453557	4.969109164	-4.323930876
	H262	2.081963928	6.860892256	-3.883678114
	H263	-1.630931252	4.494647904	-3.700036283
	H264	-8.437222146	-0.638292990	-0.868435907
	H265	3.312759122	9.551479195	-5.175537800
	H266	-6.300150117	-1.312609472	-2.661856854
	H267	0.244855053	5.831591808	-5.751053235
	H268	-3.517690588	6.964884166	-0.217889519
	H269	3.086706922	10.037613254	-3.483193807
	H270	-3.399655518	7.038838156	-7.234267615
	H271	-5.415531397	8.426216182	-4.749094645
	H272	-5.359675722	-3.916028900	-2.832175201
	H273	-5.821097993	-3.500586751	-0.200241811
	H274	-7.049567567	10.383446281	0.580486307
	H275	-5.511320486	1.131503440	-1.297602319
	H276	-0.771827169	7.602121559	-4.328311599
	H277	-2.053315848	5.730119648	-6.452056256
	H278	-0.936079355	3.709796732	-5.110011002
	H279	-3.831058448	5.449964045	-4.034859706
	H280	0.827116807	9.223907616	2.105829827
	H281	-0.594520927	9.716348920	-0.761621274
	H282	-0.265980304	12.597693424	-0.808451069
	H283	2.220337212	9.660686242	1.089171545
	H284	-2.561103267	10.746633286	0.821044440
	H285	4.183367446	-3.739742601	-8.960354069
	H286	4.258300511	-5.079247889	-7.800663413
	H287	7.603632157	-5.608743711	-4.890999552
	H288	4.644353829	-5.936923259	-9.992838281
	H289	3.125218446	-6.410228481	-9.604192211
	H290	5.331679599	4.704924131	-1.458686219
	H291	5.722199271	7.528858352	-0.421347700
	H292	4.530322747	7.708813838	-4.485722649
	H293	4.008558334	5.660608561	-0.714800380
	H294	6.712249712	7.956784805	-1.653476810
	H295	4.960504066	2.202913902	-7.049709811
	H296	5.696793905	6.448780263	-3.152396287
	H297	5.563595536	-6.408128115	5.311421691
	H298	7.262470098	-7.588480030	3.048292559
	H299	3.207581469	0.750006793	-7.000962837
	H300	5.219866229	0.359546121	2.963470711
	H301	0.203287639	0.720499125	-8.881854578
	H302	-1.269877174	-2.517343852	-4.177743457
	H303	-0.052100254	1.414139026	-7.281308357
	H304	4.617417433	-3.952227442	4.639471794
	H305	1.686736006	-0.491330201	-6.567585810
	H306	4.272791942	-4.602037790	3.029906490
	H307	5.027021060	-6.831246036	3.667235502
	H308	6.723733363	-5.136395681	2.760805441
	H309	7.009741571	-4.592897613	4.422165924
	H310	-5.508685563	-1.396214737	-6.034201667
	H311	6.240037899	-8.859641476	5.561335923
	H312	5.274185094	-11.445702173	4.235749588
	H313	0.056799192	-0.427949583	-4.295784174

H314	6.788674828	-11.057533898	6.699235950
H315	5.220135084	-11.480574264	6.494012122
H316	5.875378292	-2.942934790	2.110518543
H317	-0.989165536	1.672834615	-5.091703260
H318	-3.605507009	-2.559024891	-5.047388856
H319	7.640485219	-10.018894304	-1.022885927
H320	6.479598024	-8.075885479	-3.454088299
H321	6.681117834	-8.134887781	0.452406042
H322	6.750191438	-10.760000390	-2.067586756
H323	4.409970716	1.773400276	-9.776266521
H324	6.431030287	-5.075725665	-0.041343386
H325	2.301945731	-7.886574372	1.396163934
H326	2.898860116	-2.629104537	-7.456674463
H327	3.181555195	-9.360051868	-0.090544114
H328	2.761947169	-7.480027597	-6.553302455
H329	2.054776668	-5.813437383	-4.773418946
H330	6.934698014	-11.964392346	4.545237820
H331	5.687054624	-0.713500220	1.666449913
H332	8.462208281	-5.677548544	0.972119226
H333	7.357414321	-3.845102388	-0.224043272
H334	9.180439016	-6.529000716	3.073963291
H335	4.130146694	-2.167021336	5.180600421
H336	3.706524199	-8.652368210	-3.020692956
H337	4.991840938	-8.855680306	-1.817663047
H338	1.955765957	-8.981007682	-1.309086651
H339	1.003727872	-2.653995520	-5.913449502
H340	-3.341955834	1.647439912	-5.965339470
H341	-5.469066280	0.355643946	-6.377683141
H342	2.464041369	-6.147905500	1.223702802
H343	8.599913421	-7.226712091	0.944833193
H344	7.507086505	-9.138542928	1.279794503
H345	4.024010782	-0.519888873	4.798778525
H346	5.193356533	-8.231683337	-6.269570932
H347	6.360623842	-6.435487628	-2.839949325
H348	0.103088513	-3.598124634	-7.138605897
H349	3.834566937	3.542933028	-7.365014172
H350	7.815155439	0.552160122	3.681394746
H351	7.799203228	0.832618533	1.924268829
H352	8.739511897	-7.205031400	-6.197369505
H353	8.085302675	-8.470045792	-5.389299052
H354	10.238571634	-8.670973534	-1.338859270
H355	8.428065664	-8.471310194	-2.695041786
H356	11.539565581	-7.830762709	-3.617861488
H357	10.109860990	-6.924775399	-1.048853856
H358	10.529874108	0.548859998	1.816327285
H359	-3.015578998	13.463772293	2.607631328
H360	10.477520422	-6.260726818	5.777700264
H361	9.835292119	-3.984289162	4.337992999
H362	-4.840721113	12.384008946	0.441974018
H363	-4.106595039	10.599430238	2.847625635
H364	-5.718853027	11.250272219	2.493154465
H365	0.948267916	-3.147835285	-0.829488495
H366	-6.639820783	6.609896071	-1.912482714
H367	0.508993542	-1.326299049	0.494718488
H368	3.219358126	-0.729825170	0.570664927
H369	2.306308035	-4.142774823	0.595654647
H370	-1.552857591	-0.121363342	-0.732439753
Mo371	3.813532490	-4.737315546	-2.190450804
N372	2.273217110	-0.680929353	0.170536211
N373	1.481575280	-1.610655637	0.717228167
N374	-7.134680328	-7.245698292	0.743291607
N375	-8.714915295	-4.452457702	2.663006809
N376	-7.931441047	-4.429638456	5.363695217
N377	-5.248363403	-5.255493273	5.340543254
N378	-4.058513972	-2.963630063	4.150308707
N379	-4.508765347	-1.044277265	6.263786556
N380	-3.166823699	-2.167756430	8.580652395
N381	-0.653599052	-3.477471455	7.871100223
N382	0.494891660	-1.994626223	5.785143208
N383	0.380562347	0.966900316	6.160188907
N384	0.293048516	1.090208150	9.066956956
N385	5.954094679	-10.864712598	6.141223166
N386	6.598760072	-8.766970271	4.605222788
N387	5.433237599	-2.859080575	3.028895945
N388	4.539086906	-1.397825397	4.644600398
N389	5.259537913	-0.588556161	2.597916800
N390	9.282154235	-6.545137246	4.112478639
N391	-6.023236156	-3.289965880	-2.339348280
N392	-2.046185683	-5.864227432	-2.532454061
N393	-6.276281131	-0.740459940	-1.801337441

N394	-8.520472667	0.320918484	-0.538328981
N395	-7.854134169	0.470747556	2.150693787
N396	-5.702475504	2.244800829	2.326300966
N397	-3.253749875	2.492975442	-1.221278318
N398	-2.296614907	0.639417701	-0.638134253
N399	-6.349032864	4.917253658	2.715336617
N400	-9.891609969	7.773370503	1.330159179
N401	-9.690927995	9.683382927	2.440959669
N402	9.512342447	2.922704485	1.916348095
N403	6.199763599	7.166220676	-1.252809760
N404	3.948708181	8.145700719	-3.775142818
N405	1.281945727	7.484344277	-4.025181612
N406	-3.442127517	5.905836812	-4.881269464
N407	-3.711553942	7.613971157	-6.459006620
N408	-4.729409978	7.771188246	-4.384140244
N409	0.486655567	6.210017017	-1.574899555
N410	0.878567697	8.315927109	0.220684684
N411	-0.506384457	10.624847415	-0.307085679
N412	-2.186349596	9.955364108	-3.907540685
N413	-2.865955608	11.711799543	0.720377862
N414	3.695160992	-5.591464816	-9.834922948
N415	2.089718365	-3.234240827	-7.611545722
N416	1.008800366	-0.410313623	-7.341835581
N417	3.047908007	1.583680291	-7.571669757
N418	-2.149486965	-3.953432758	-9.577634854
N419	-3.647096159	-1.989809185	-8.109320660
N420	7.899263891	-7.510609058	-5.697885523
N421	4.076931851	-6.045357298	-4.046433400
N422	3.389557731	-7.117197530	-5.841049771
N423	9.088863381	-7.691536566	-2.756348450
N424	0.009734730	2.438045771	-0.175265093
N425	0.775695370	1.730692173	-0.605020940
O426	-6.521321624	-5.050665573	2.515781190
O427	-7.039837672	-2.348908074	5.087852524
O428	-4.993644577	-4.396994114	7.443147326
O429	-2.233622243	-3.423819617	5.434971811
O430	-2.659394538	0.019115714	5.480280507
O431	-2.521130488	-0.016379277	9.006569022
O432	0.106592615	-2.850843474	9.943519084
O433	2.536868776	-2.978342386	6.168363240
O434	2.562269466	0.679349107	5.524968967
O435	2.013018698	2.555565628	8.833198651
O436	2.589543096	0.760554908	11.802918308
O437	7.190156048	-10.082151881	2.796770982
O438	8.299705487	-7.053095720	6.128876220
O439	11.862757507	-4.076964941	4.545399791
O440	-5.759597131	-0.952253603	0.423564881
O441	-3.902967705	-4.855870918	-3.369017656
O442	-7.572263507	2.396948015	-0.519525274
O443	-6.630127758	2.736096612	-3.127165144
O444	-9.837851370	1.528611849	2.623877180
O445	-5.483393350	1.854387032	4.565861402
O446	-4.209858392	5.361543905	3.387225141
O447	-6.545316658	7.627459327	4.981545667
O448	10.808048990	0.252972325	3.817363419
O449	3.642925700	5.113747502	3.457716006
O450	3.458638901	8.290137206	-1.552314000
O451	0.685426308	9.659385967	-4.474958808
O452	-1.617162536	6.961445088	-2.065072048
O453	-0.757132651	7.432717069	1.565878715
O454	2.369616722	5.920103308	0.946511328
O455	0.741568401	11.824342113	1.196389419
O456	-2.671614867	13.565213456	-0.593527514
O457	-3.483202671	10.764556342	-2.208313103
O458	-4.979451246	14.007945836	2.490423104
O459	-5.689188345	6.648419944	-1.690913881
O460	1.398838364	-5.310117670	-8.310897547
O461	-0.655465297	-1.538425793	-8.435852958
O462	1.642250904	2.887228818	-8.794627107
O463	5.381086807	3.548352663	-9.499963279
O464	-5.369613754	-3.478261147	-8.228902315
O465	-1.633540978	-7.201117764	-5.233408667
O466	0.192966323	-6.358728280	-6.195507597
O467	-4.388296649	1.495241780	-8.422712386
O468	9.735091000	-5.693602571	-3.607997819
O469	12.552949019	-7.108232127	-2.000377093
O470	0.335543068	-6.386980514	-0.758393532
O471	-0.183229855	-7.204136249	1.270830050
O472	5.134703209	-11.244681417	-2.489517086
O473	2.872225039	-11.263157504	-2.506891527

	O474 5.098241958 -6.028701655 -0.971666724<br>O475 5.086972656 -7.506870471 0.722349298<br>O476 2.612886662 -6.387788022 -1.600290244<br>O477 9.058077338 -6.405403491 1.276907364<br>O478 7.100854712 -4.519519117 0.432491326<br>O479 -4.566705161 4.478572025 -2.664921882<br>O480 7.709531926 -10.576927339 -1.829177870<br>O481 7.567813357 -8.586991107 0.459040365<br>S482 3.310471826 4.711681649 -2.840527522<br>S483 1.838219782 1.687003682 -3.391528741<br>S484 1.857592897 -3.606393456 -2.966175038<br>S485 3.716795204 1.873474935 -0.372074324<br>S486 0.246639410 -0.862493459 -1.779221918<br>S487 3.601450381 -1.190649729 -5.459277257<br>S488 3.464018112 -3.670946038 0.045950159<br>S489 5.361808908 1.418761895 -3.495928621<br>S490 5.536415707 -3.360199884 -3.032718813<br>S491 6.323644624 -0.860281547 -0.501595410<br>end

TS

	bm522brnhnh63b32tc_1_53316.64<br>C1 -7.713827442 -5.276988791 2.224031470<br>C2 -8.180384727 -6.505273166 1.432937306<br>C3 -8.417403549 -3.180146731 3.298732127<br>C4 -7.730492242 -3.286507642 4.657414202<br>C5 -7.241089745 -4.651574543 6.621901905<br>C6 -5.716845125 -4.747508352 6.496323381<br>C7 -3.811998098 -5.218098781 5.041342507<br>C8 -3.284662044 -3.789366246 4.889948238<br>C9 -3.734155377 -1.549335998 3.970728076<br>C10 -3.579611471 -0.784128792 5.292097150<br>C11 -4.408392583 -0.326760361 7.513834287<br>C12 -3.260099250 -0.812343847 8.410560464<br>C13 -2.266797937 -2.727843908 9.566864765<br>C14 -0.819825133 -3.004748134 9.134408951<br>C15 0.650922513 -3.870080421 7.361234427<br>C16 1.319271278 -2.893067737 6.390242000<br>C17 0.959878899 -1.005327523 4.817247929<br>C18 1.389478847 0.289723235 5.537685883<br>C19 -0.080745857 -0.772323996 3.713690498<br>C20 0.385242912 0.370473004 2.805364161<br>C21 -0.350157197 -2.093675341 2.983397172<br>C22 0.609072729 2.219618386 6.886205114<br>C23 1.039779876 1.973034436 8.345477200<br>C24 -0.642136630 3.127306354 6.781907360<br>C25 -0.505540144 4.380294254 7.654762719<br>C26 -0.912455945 3.516022460 5.322268244<br>C27 0.584811789 0.900019362 10.479111297<br>C28 2.038492174 0.566548710 10.741007825<br>C29 6.217823527 -11.145629646 4.728749800<br>C30 6.727668710 -9.933651922 3.951573193<br>C31 7.013577326 -7.464952087 4.113837573<br>C32 8.261604713 -7.005456558 4.889837989<br>C33 5.870744263 -6.445152018 4.256009152<br>C34 6.260653658 -5.054322085 3.756804353<br>C35 5.051922574 -4.129971777 3.647083971<br>C36 5.088369672 -1.627740717 3.428663841<br>C37 10.521099572 -6.082049240 4.693256190<br>C38 10.757181533 -4.600261384 4.508265157<br>C39 -5.450039833 -2.892717823 -1.041229169<br>C40 -5.839761233 -1.441632165 -0.712676204<br>C41 -3.907440056 -2.985776570 -1.039382696<br>C42 -3.319381235 -4.411360936 -1.041134341<br>C43 -3.119590766 -5.049962736 -2.414416621<br>C44 -6.405993796 0.701823204 -1.758723444<br>C45 -7.548932902 1.203891860 -0.876146865<br>C46 -6.559724745 1.295665082 -3.174355564<br>C47 -9.567561599 0.697793807 0.394878709<br>C48 -9.091957753 0.943667232 1.831765965<br>C49 -7.271377077 0.703906139 3.462130772<br>C50 -6.073151521 1.649526487 3.505690107<br>C51 -4.620577876 3.221823271 2.313255144<br>C52 -5.043621513 4.596466455 2.895566454<br>C53 -4.114568578 3.491510305 0.889761853<br>C54 -3.435474956 2.373995863 0.157958279<br>C55 -2.821418582 1.198483330 0.518453967<br>C56 -2.554772347 1.438925860 -1.669832160<br>C57 -6.887072822 6.241298633 3.007018502<br>C58 -6.753444422 6.521512265 4.502187619

C59	-8.370610461	6.249834089	2.574642709
C60	-9.000508864	7.596558180	2.368767140
C61	-8.870344570	8.777531358	3.074091972
C62	-10.290898304	9.034394125	1.393141969
C63	9.289481020	1.961321000	3.008933696
C64	10.293997368	0.830935764	2.880020380
C65	7.881331549	1.325393130	2.913903728
C66	6.766632999	2.331540564	3.082330990
C67	6.224122812	2.615597912	4.349787538
C68	6.235956507	3.014925804	1.972791633
C69	5.186419086	3.540367570	4.508863143
C70	5.214295713	3.952970327	2.118527882
C71	4.680056394	4.205522375	3.384995083
C72	5.185461006	6.697522574	-2.203692554
C73	4.119138755	7.774732895	-2.468948845
C74	4.533163123	5.433294405	-1.648427387
C75	3.025846416	9.185851109	-4.180405549
C76	1.548248664	8.787133053	-4.232404915
C77	-0.046972457	6.894981943	-3.912186928
C78	-0.462418285	6.690177048	-2.431019607
C79	-0.026443701	5.570268452	-4.709799997
C80	-1.272289966	4.679871936	-4.715428045
C81	-2.440385527	5.129677645	-5.607810908
C82	-3.978797033	7.063089703	-5.250688212
C83	0.167050187	6.008956633	-0.159446371
C84	0.029167979	7.327789489	0.613508786
C85	1.219889977	5.147935563	0.572434396
C86	1.143189267	9.458321361	1.071788905
C87	0.450046410	10.746052316	0.658616191
C88	-1.033177006	11.817277576	-0.946865222
C89	-2.280809170	12.434899540	-0.269106207
C90	-1.252013789	11.658275641	-2.453394719
C91	-2.410436911	10.739843267	-2.819559934
C92	-4.164903470	12.093617491	1.261222251
C93	-4.034956098	13.292997208	2.189306130
C94	-4.786074063	10.893239333	2.030243909
C95	-5.063030864	9.714721139	1.123646800
C96	-6.274147435	9.628269764	0.420356708
C97	-4.101397524	8.714099700	0.908941373
C98	-6.508912310	8.597705223	-0.492210793
C99	-4.306077006	7.690703343	-0.021334982
C100	-5.512287843	7.648182951	-0.715134132
C101	3.704075010	-4.687720414	-8.680032241
C102	2.288266802	-4.429942294	-8.196045106
C103	0.843044378	-2.806761573	-6.986504897
C104	0.313979751	-1.526970604	-7.645299949
C105	0.682035133	0.887148087	-7.903171596
C106	1.837208520	1.858207149	-8.127221861
C107	4.131845765	2.543026394	-7.692134658
C108	4.684629673	2.620960745	-9.102450290
C109	-3.138010636	-4.359895722	-8.571264333
C110	-4.151940265	-3.243349443	-8.296214810
C111	-2.590155615	-4.915447872	-7.238218382
C112	-1.891091346	-6.271815049	-7.447763337
C113	-1.117184692	-6.667653273	-6.211835825
C114	-4.537808389	-0.867356797	-7.875892319
C115	-4.083032753	0.339654031	-8.671871010
C116	-4.883242328	-0.576369380	-6.393920041
C117	-3.618030624	-0.469076737	-5.595377291
C118	-2.892829328	0.732096542	-5.560802174
C119	-3.036048651	-1.634400392	-5.063960039
C120	-1.573295828	0.742486602	-5.095360989
C121	-1.722704798	-1.619195669	-4.594705642
C122	-0.974259911	-0.438847986	-4.651306494
C123	7.692841249	-6.631371655	-4.517007702
C124	8.912734257	-6.621376528	-3.580598847
C125	6.412531689	-7.008308772	-3.755818863
C126	5.128323890	-6.830793126	-4.500961499
C127	4.698088905	-7.500305259	-5.626479384
C128	3.046148222	-6.234381335	-4.865327625
C129	10.215537518	-7.686613738	-1.834786434
C130	11.537427290	-7.509766231	-2.548999958
C131	0.654999031	-6.898072656	0.423707976
C132	2.153890677	-7.030150902	0.686555115
C133	3.047807171	-7.255534954	-0.573137729
C134	2.972527162	-8.747912400	-0.990691901
C135	3.956722233	-9.166130828	-2.083481877
C136	3.971357352	-10.684071156	-2.370144660
C137	4.510099137	-6.925250212	-0.224728466
C138	3.422696720	-0.910819924	-2.160258553

Fe139	3.580536613	-2.312271539	-3.547833970
Fe140	1.961658774	0.600190471	-1.343755943
Fe141	4.849670500	0.396961237	-1.582650431
Fe142	3.475807375	0.202026061	-3.766413744
Fe143	3.685658433	2.538510944	-2.476968875
Fe144	4.754870680	-2.136714800	-1.356722168
Fe145	2.017857790	-2.145740931	-1.122691426
H146	-6.405887833	-7.509995581	1.386355344
H147	-6.709706996	-6.664197678	0.023191791
H148	-9.659761171	-4.616207903	2.326107038
H149	-8.957592657	-6.191353267	0.717854769
H150	-8.684051297	-7.170572236	2.155576903
H151	-7.759235347	-2.568105015	2.664319494
H152	-8.507178596	-5.157922036	4.954798532
H153	-9.361317494	-2.635678734	3.435228340
H154	-7.618181631	-5.583534838	7.063514864
H155	-5.880029332	-5.427721376	4.554503786
H156	-7.448298310	-3.830949358	7.321740055
H157	-2.796608436	-1.426179059	3.418324394
H158	-4.892520602	-3.329108197	3.702078408
H159	-3.634917805	-5.772441609	4.109693942
H160	-4.245672379	0.742825827	7.330766694
H161	-5.319239345	-1.609446735	6.030964296
H162	-3.241433425	-5.696476966	5.846109304
H163	-4.537948147	-1.095243055	3.375393618
H164	-3.828787653	-2.774077018	8.099297038
H165	-5.356409337	-0.457945652	8.052532236
H166	-0.804211179	-2.830820959	3.657209426
H167	-1.043434269	-1.939772885	2.140052733
H168	0.576575165	-2.511377913	2.569604974
H169	-1.015130255	-0.446683449	4.196327833
H170	-2.699245719	-3.680428172	9.906476633
H171	-1.070156545	2.638892087	4.680102487
H172	-0.568799397	0.693392872	8.707461898
H173	-0.365083957	4.135665387	8.717384165
H174	-0.273054366	0.464235300	1.931102129
H175	1.403608579	0.207818707	2.431734905
H176	9.401854636	2.391089008	4.022915332
H177	-1.812981775	4.144321877	5.257767314
H178	-1.412418980	4.995585937	7.565247252
H179	-0.066638936	4.094869762	4.916261899
H180	0.352407137	4.991284192	7.331354457
H181	-1.504260681	2.542525130	7.152676851
H182	1.468535401	2.721894358	6.419325342
H183	-0.568856451	0.628406756	6.065826684
H184	0.369537143	1.336282851	3.331178558
H185	1.522873109	4.321374592	-0.086624225
H186	0.728602966	4.723196090	1.459770865
H187	2.669198867	5.603960362	1.825062724
H188	3.350958076	5.206845652	4.386835586
H189	4.799115241	4.460337468	1.248403020
H190	6.610765725	2.793693702	0.970985181
H191	6.607601749	2.095706968	5.231327810
H192	-0.498217348	-2.053205594	5.980710389
H193	0.573048952	-4.839011032	6.843347566
H194	1.332858611	-3.997155304	8.211110776
H195	-1.462655810	-3.613489001	7.256836410
H196	-2.207900314	-2.050541134	10.428268945
H197	4.759972138	3.725017169	5.498800281
H198	1.875962053	-1.405730091	4.362355281
H199	0.301721101	1.773047209	11.090292454
H200	-0.009789737	0.038140965	10.821132066
H201	2.565680355	0.050389026	9.905489396
H202	11.383128436	-6.641836481	4.298701810
H203	-1.897494567	-6.346243151	-3.422893039
H204	1.257786614	-6.279531538	-1.305184945
H205	-2.648789582	-7.044110902	-7.640389232
H206	-1.217703301	-6.223088501	-8.316611678
H207	-3.411212802	-5.046393095	-6.516020236
H208	-3.469684243	0.092306518	-9.570594724
H209	-3.750157615	-5.151434500	-9.030227966
H210	-3.517893436	-2.414600081	-1.897842382
H211	-3.968279640	-5.090229658	-0.461064245
H212	-2.637113107	-1.829887090	-8.148653699
H213	-5.490164565	-1.156051523	-8.368301854
H214	-1.447843988	-3.313024984	-9.183767365
H215	-1.369085995	-6.000148227	-1.782751561
H216	-1.901061367	-4.178711988	-6.797035767
H217	-1.647257389	-4.772770909	-9.921747588
H218	-6.908011654	-3.784681677	-2.199125657

H219	-3.555011164	-2.480546938	-0.128001110
H220	0.501902232	-5.936488304	-7.058148420
H221	-2.354712384	-4.398598071	-0.512446669
H222	-3.806461390	2.864570915	2.959322005
H223	-2.720648209	0.725740547	1.487405902
H224	-8.049124261	1.148129900	4.099673024
H225	-7.299635694	-0.049202918	1.463341678
H226	-6.978662262	4.212765725	2.330574965
H227	-8.269599987	9.055050151	3.933261343
H228	-6.891240033	5.619432431	5.146999629
H229	-8.957371698	5.645353776	3.289653631
H230	-6.321225187	7.009283870	2.456198637
H231	-10.999429132	9.522774302	0.728578240
H232	-3.404614097	4.332408178	0.962711097
H233	-4.959607384	3.849211526	0.278598568
H234	-9.828961771	10.652280021	2.697799427
H235	-8.432344657	5.726905478	1.606781297
H236	-6.229327970	2.055794358	1.479805580
H237	-6.973314317	-0.244204463	3.936199971
H238	-10.059618586	1.623759650	0.067704533
H239	-2.240524978	1.258662833	-2.695272495
H240	-10.318122652	-0.102251703	0.405071084
H241	10.345681276	3.471572680	2.114420233
H242	8.731511096	3.582844795	1.891618539
H243	1.370076520	5.818186069	-1.938455115
H244	-0.804897722	5.500066846	-0.086007822
H245	1.618508952	8.071349529	-0.448856510
H246	-3.643252836	3.276483905	-1.791285460
H247	-5.324298726	3.879462434	-2.934431458
H248	-7.189846110	2.962296240	-2.357846842
H249	-4.961304747	5.192998054	-2.108385063
H250	-5.685226349	1.025412791	-3.781913769
H251	-7.463938846	0.885258304	-3.653364834
H252	-0.314398247	11.339778799	-2.930511634
H253	-1.498254287	12.655457795	-2.850580363
H254	-1.224086877	9.775820193	-4.216196462
H255	-2.892779585	9.226197186	-4.060491693
H256	-4.101809386	8.524983879	-6.678659358
H257	-2.937708431	4.249498944	-6.042697565
H258	-7.464152408	8.535197857	-1.020618012
H259	-3.163114288	8.715753880	1.470660330
H260	-4.924723330	7.386648574	-3.446150623
H261	0.806491591	4.963892179	-4.318123652
H262	2.082847925	6.854582547	-3.889464893
H263	-1.634126850	4.496786005	-3.691234951
H264	-8.436865779	-0.627094045	-0.872221820
H265	3.313748747	9.543021443	-5.177231944
H266	-6.296210906	-1.312312337	-2.655803381
H267	0.243641460	5.820835047	-5.748938459
H268	-3.517897403	6.963365936	-0.220701840
H269	3.091051334	10.036401589	-3.486236039
H270	-3.389012470	7.047079436	-7.223295302
H271	-5.416634886	8.426731494	-4.741068221
H272	-5.363566220	-3.915528522	-2.824863525
H273	-5.821875786	-3.498488961	-0.193278031
H274	-7.052466608	10.377291595	0.584570838
H275	-5.502450365	1.134099935	-1.300920419
H276	-0.770370956	7.599821799	-4.333006628
H277	-2.056254356	5.726279764	-6.445523008
H278	-0.941686545	3.705136236	-5.098547184
H279	-3.837876472	5.445210031	-4.031324803
H280	0.832012658	9.225921614	2.101026312
H281	-0.593941452	9.715784856	-0.767046893
H282	-0.266920387	12.596855386	-0.814864706
H283	2.222904214	9.662240236	1.081136772
H284	-2.563679988	10.745276005	0.816795360
H285	4.183872980	-3.736580214	-8.955235757
H286	4.256625114	-5.081042901	-7.801624609
H287	7.603657447	-5.597086515	-4.880659766
H288	4.640037956	-5.930516608	-9.998113148
H289	3.119751445	-6.402054968	-9.610335196
H290	5.328343202	4.701582628	-1.455424522
H291	5.718438300	7.529725237	-0.414471586
H292	4.535695519	7.706380718	-4.481943580
H293	4.006595787	5.658938535	-0.710587606
H294	6.709376958	7.957903259	-1.645666180
H295	4.949932674	2.214932407	-7.032481037
H296	5.699461599	6.447256848	-3.144706288
H297	5.556825350	-6.404883206	5.312271029
H298	7.257092475	-7.587207861	3.051409204

	H299	3.202767074	0.755965433	-6.993348166
	H300	5.226660179	0.361614367	2.960288723
	H301	0.199519282	0.720941149	-8.874615545
	H302	-1.270078881	-2.525269181	-4.185267844
	H303	-0.056300907	1.413165557	-7.273762112
	H304	4.613372363	-3.948627750	4.635385581
	H305	1.686259805	-0.489971903	-6.562381088
	H306	4.271571839	-4.599579359	3.025376862
	H307	5.021349478	-6.828188390	3.667982898
	H308	6.721905183	-5.137809004	2.761508126
	H309	7.007002997	-4.592484516	4.422514066
	H310	-5.513779988	-1.401142729	-6.031454299
	H311	6.233889203	-8.857725184	5.564335339
	H312	5.278791090	-11.448641573	4.240516091
	H313	0.056251831	-0.435772130	-4.303367154
	H314	6.788592033	-11.049281790	6.705403630
	H315	5.222391140	-11.480372492	6.498992820
	H316	5.871109497	-2.943390794	2.104523985
	H317	-0.993725698	1.667519979	-5.089487979
	H318	-3.609478773	-2.564445195	-5.044929719
	H319	7.631457192	-10.023592483	-1.018015388
	H320	6.476190117	-8.066093616	-3.449207636
	H321	6.678958596	-8.138484155	0.448860805
	H322	6.738597311	-10.762836654	-2.061655958
	H323	4.419958509	1.757766462	-9.760037675
	H324	6.442666866	-5.079556440	-0.048684816
	H325	2.310013389	-7.830413329	1.419201015
	H326	2.900000086	-2.628367292	-7.450823505
	H327	3.172193176	-9.338888676	-0.082855276
	H328	2.764269227	-7.474658794	-6.545510916
	H329	2.056621540	-5.803285851	-4.768930403
	H330	6.940760733	-11.960452260	4.553444929
	H331	5.686033505	-0.711305880	1.661213062
	H332	8.465966741	-5.677248238	0.970382947
	H333	7.361299862	-3.841319490	-0.224743925
	H334	9.175996452	-6.530625069	3.077505774
	H335	4.158589041	-2.164182379	5.187732583
	H336	3.694115873	-8.654654559	-3.022496506
	H337	4.976614030	-8.856999208	-1.815698240
	H338	1.940768900	-8.975772971	-1.302014647
	H339	1.005474039	-2.652551164	-5.908235634
	H340	-3.349156572	1.643545902	-5.955149279
	H341	-5.474412694	0.351214623	-6.372623278
	H342	2.452564521	-6.094563734	1.186024132
	H343	8.599919983	-7.227008331	0.946578486
	H344	7.502488364	-9.137478298	1.283932247
	H345	4.041091460	-0.517809941	4.805279129
	H346	5.201429731	-8.225358011	-6.254769461
	H347	6.365573570	-6.427623023	-2.827178475
	H348	0.104287432	-3.597535773	-7.132717824
	H349	3.827392100	3.551193727	-7.371977212
	H350	7.812286138	0.551137645	3.691938002
	H351	7.796709430	0.828084100	1.934119235
	H352	8.736080876	-7.187173755	-6.192984004
	H353	8.088534158	-8.456251112	-5.387324354
	H354	10.239383224	-8.669589145	-1.338435873
	H355	8.423878317	-8.461545023	-2.687532035
	H356	11.541719143	-7.833679677	-3.617860787
	H357	10.115849345	-6.923091893	-1.047582469
	H358	10.525735149	0.543623814	1.825463277
	H359	-3.014047019	13.461001776	2.605931797
	H360	10.472645574	-6.266526613	5.781281380
	H361	9.836807098	-3.987584588	4.343876280
	H362	-4.842293910	12.384169479	0.440782639
	H363	-4.106096511	10.598709053	2.845234642
	H364	-5.719032945	11.248279381	2.491470501
	H365	0.969874647	-3.183057241	-0.814522597
	H366	-6.644850972	6.601060601	-1.905117357
	H367	0.502213281	-1.341635021	0.479984440
	H368	3.207582002	-0.723880080	0.583758794
	H369	2.264759304	-4.251394091	0.274007907
	H370	-1.557078170	-0.127621376	-0.727727791
	Mo371	3.830729671	-4.726205968	-2.198438358
	N372	2.263526446	-0.683382576	0.174756188
	N373	1.474337288	-1.619567335	0.714019744
	N374	-7.150871932	-7.252782075	0.734019662
	N375	-8.712380439	-4.452458499	2.658221869
	N376	-7.921674458	-4.429210433	5.356600369
	N377	-5.238319279	-5.253823938	5.330246803
	N378	-4.049327016	-2.961295594	4.136999776

	N379	-4.500357127	-1.038419639	6.249268613
	N380	-3.161650289	-2.158024896	8.570764679
	N381	-0.652793584	-3.480415545	7.869014436
	N382	0.501225894	-1.995147900	5.790600915
	N383	0.390420512	0.966813370	6.163005063
	N384	0.299130032	1.094723464	9.068112349
	N385	5.953834840	-10.861777253	6.145811302
	N386	6.592799776	-8.764687165	4.608257809
	N387	5.431887678	-2.857913022	3.023845665
	N388	4.558849069	-1.393706334	4.647605979
	N389	5.259257736	-0.586350073	2.593105017
	N390	9.276852245	-6.546559381	4.115905876
	N391	-6.025141961	-3.287345827	-2.332013122
	N392	-2.053571925	-5.863554278	-2.532466306
	N393	-6.272906942	-0.738645259	-1.796078035
	N394	-8.515799235	0.331176077	-0.538454312
	N395	-7.852919240	0.472137464	2.153360661
	N396	-5.699023590	2.242376560	2.330476903
	N397	-3.255186630	2.488694102	-1.215440139
	N398	-2.294408239	0.637111902	-0.632236116
	N399	-6.351150960	4.915501239	2.712905005
	N400	-9.892453528	7.770270004	1.322501016
	N401	-9.695178342	9.678997986	2.436108415
	N402	9.508369625	2.916779943	1.920229435
	N403	6.197504519	7.166951975	-1.244996001
	N404	3.951683433	8.142677915	-3.772940715
	N405	1.283842375	7.479819250	-4.029759586
	N406	-3.446996490	5.902558507	-4.875955120
	N407	-3.708716671	7.617636480	-6.447892467
	N408	-4.735915884	7.765730884	-4.377090242
	N409	0.485408963	6.208712516	-1.576219020
	N410	0.880487153	8.315711426	0.216895313
	N411	-0.506419383	10.624082388	-0.311898782
	N412	-2.187127622	9.942405348	-3.899533819
	N413	-2.867176699	11.711062558	0.717810682
	N414	3.691703715	-5.583771305	-9.837988353
	N415	2.090468793	-3.232911452	-7.606466161
	N416	1.007914278	-0.409285654	-7.336020699
	N417	3.042582944	1.589729025	-7.563802290
	N418	-2.148978838	-3.952035355	-9.576319062
	N419	-3.647868887	-1.990407387	-8.105106535
	N420	7.897754312	-7.496690213	-5.692662707
	N421	4.079442791	-6.031605558	-4.045973426
	N422	3.393463496	-7.109088672	-5.835853535
	N423	9.089234733	-7.685988123	-2.752433881
	N424	0.009593744	2.440186914	-0.182145979
	N425	0.767493699	1.721994065	-0.610718956
	O426	-6.521780099	-5.060153132	2.504073616
	O427	-7.029658348	-2.348992012	5.079656697
	O428	-4.981328651	-4.393056846	7.431674346
	O429	-2.229703796	-3.417360527	5.430970142
	O430	-2.654541696	0.026902859	5.460643695
	O431	-2.512253951	-0.006611513	8.990956973
	O432	0.107972867	-2.849121854	9.939717083
	O433	2.541122250	-2.983850476	6.172060966
	O434	2.573026218	0.675770179	5.532631153
	O435	2.017261355	2.562626897	8.835838109
	O436	2.586231967	0.773774960	11.813471766
	O437	7.191182858	-10.079697206	2.801920949
	O438	8.291840318	-7.049236255	6.132322036
	O439	11.863974339	-4.085496148	4.551341186
	O440	-5.757953576	-0.949128318	0.429628215
	O441	-3.913295454	-4.857202586	-3.364286879
	O442	-7.551810001	2.400093827	-0.504800337
	O443	-6.632597023	2.731109194	-3.136828250
	O444	-9.831715953	1.540549349	2.623560733
	O445	-5.490663273	1.863970863	4.572705467
	O446	-4.217545600	5.359970149	3.400824291
	O447	-6.552483093	7.624136470	4.979855124
	O448	10.802435786	0.249272890	3.827029409
	O449	3.637357179	5.109518772	3.460239082
	O450	3.454006993	8.286194917	-1.551950958
	O451	0.687391045	9.657730076	-4.462317963
	O452	-1.614945888	6.969510490	-2.065989751
	O453	-0.757848455	7.437739441	1.562319528
	O454	2.364966188	5.917978681	0.949488115
	O455	0.742079972	11.824694991	1.190041697
	O456	-2.669388836	13.566818620	-0.592064419
	O457	-3.488954461	10.762601579	-2.209379405
	O458	-4.977323801	14.008001288	2.490771086

O459	-5.693619409	6.641261103	-1.686388511
O460	1.396844985	-5.305301769	-8.312784517
O461	-0.655240420	-1.538439179	-8.430504103
O462	1.634584151	2.889179296	-8.789242671
O463	5.395140282	3.532316865	-9.492502950
O464	-5.369679992	-3.479162456	-8.230191471
O465	-1.630852210	-7.197418425	-5.232451133
O466	0.195474780	-6.356470460	-6.196639317
O467	-4.391713975	1.494218058	-8.415360022
O468	9.743633134	-5.692122201	-3.609178544
O469	12.555381085	-7.110582137	-2.000986304
O470	0.322315823	-6.385275830	-0.766853781
O471	-0.189648368	-7.179630472	1.271261588
O472	5.119385008	-11.243439167	-2.484969692
O473	2.857802166	-11.265178082	-2.501924272
O474	5.100552783	-6.031853363	-0.976747602
O475	5.075964136	-7.517031696	0.713484309
O476	2.607980547	-6.380276127	-1.610819568
O477	9.058513099	-6.406258253	1.278612095
O478	7.106241569	-4.519474547	0.428565352
O479	-4.570841844	4.471998801	-2.661361128
O480	7.698222021	-10.582585657	-1.823760359
O481	7.565629921	-8.589142945	0.461107522
S482	3.308788714	4.717102610	-2.840054878
S483	1.825746065	1.689469054	-3.391296420
S484	1.870054829	-3.617961640	-2.966142062
S485	3.704788923	1.873581277	-0.374725494
S486	0.249004116	-0.892341792	-1.788853352
S487	3.604830780	-1.193411631	-5.455068952
S488	3.480975541	-3.705119958	0.044592888
S489	5.350797353	1.426005778	-3.503198372
S490	5.559580281	-3.351652638	-3.036437074
S491	6.327091942	-0.845919414	-0.507960460
end			

product

Fe(139)	-2.310
Fe(140)	0.099
Fe(141)	-2.597
Fe(142)	2.541
Fe(143)	2.464
Fe(144)	2.463
Fe(145)	0.060

bm52nhnhb3b2brta.car_3

C1	-7.713183016	-5.281663075	2.213100216
C2	-8.184402646	-6.511344738	1.426028211
C3	-8.411765676	-3.184511162	3.291246558
C4	-7.723576973	-3.290898525	4.649198377
C5	-7.232299046	-4.656392425	6.612965641
C6	-5.707865409	-4.749567246	6.486104735
C7	-3.804780450	-5.219696613	5.027509389
C8	-3.281491415	-3.790043562	4.873381773
C9	-3.737684778	-1.551745230	3.954978683
C10	-3.581794889	-0.786103637	5.276215772
C11	-4.404863304	-0.327507825	7.499538746
C12	-3.255356387	-0.812096519	8.395379939
C13	-2.262686330	-2.725963131	9.554691030
C14	-0.817246534	-3.007696481	9.121562992
C15	0.651948633	-3.873354277	7.347218629
C16	1.322680954	-2.892774722	6.381453250
C17	0.966479559	-1.001389698	4.814358548
C18	1.394053616	0.291294254	5.538370038
C19	-0.074823796	-0.763649349	3.710072457
C20	0.381220536	0.383018579	2.800736872
C21	-0.344666410	-2.084206494	2.977199509
C22	0.610698275	2.219308092	6.885938376
C23	1.044183268	1.970952435	8.344079871
C24	-0.642544008	3.124125858	6.785414469
C25	-0.507161810	4.377652864	7.657136162
C26	-0.916907553	3.510567528	5.325686019
C27	0.589837713	0.891372426	10.476005566
C28	2.042850993	0.563314700	10.743256303
C29	6.211367180	-11.150833793	4.725851963
C30	6.719958288	-9.938576187	3.946384932
C31	7.010209130	-7.469565796	4.109183333
C32	8.258070478	-7.010531116	4.886042608
C33	5.868789397	-6.448505128	4.251297293
C34	6.260918714	-5.059723241	3.749057599
C35	5.059822174	-4.124065260	3.663852157
C36	5.100770383	-1.621180410	3.441952246
C37	10.518887438	-6.089049818	4.691618534
C38	10.755738791	-4.606848255	4.508192055
C39	-5.446556604	-2.894489143	-1.037158196
C40	-5.833409904	-1.442008662	-0.708772710
C41	-3.903826945	-2.992039222	-1.036452841
C42	-3.320049769	-4.419522031	-1.039749904
C43	-3.121222034	-5.057943909	-2.413043503

	C44	-6.401673670	0.701041113	-1.758945006
	C45	-7.541868628	1.204126089	-0.872694062
	C46	-6.561756585	1.287881413	-3.176945949
	C47	-9.565225731	0.699278460	0.391991187
	C48	-9.091154308	0.943735329	1.829655967
	C49	-7.272610424	0.700115731	3.461732044
	C50	-6.074383157	1.645388644	3.504853941
	C51	-4.621339000	3.216831767	2.312560558
	C52	-5.046543752	4.588857493	2.897584135
	C53	-4.119744611	3.489896016	0.888680987
	C54	-3.445028983	2.368986269	0.160788441
	C55	-2.846886165	1.189067646	0.532557372
	C56	-2.553016018	1.420613597	-1.654639088
	C57	-6.888385113	6.233826651	2.999747421
	C58	-6.757909995	6.513714207	4.494813672
	C59	-8.371867469	6.242293814	2.567195457
	C60	-9.002068966	7.588783908	2.361669745
	C61	-8.875436248	8.769051969	3.068845160
	C62	-10.294107185	9.025383027	1.386141418
	C63	9.284433513	1.953045007	3.016128002
	C64	10.290212161	0.823389558	2.890195329
	C65	7.876797663	1.316785359	2.922149392
	C66	6.762122630	2.323959499	3.087914445
	C67	6.220092453	2.612684662	4.354713843
	C68	6.231063410	3.003603149	1.976099593
	C69	5.181712011	3.537226507	4.510631728
	C70	5.208515464	3.941137506	2.118769116
	C71	4.674173055	4.197674830	3.384483014
	C72	5.181734283	6.695408341	-2.209105165
	C73	4.115446750	7.773442987	-2.473751855
	C74	4.535925499	5.424818179	-1.663769379
	C75	3.020993017	9.184649114	-4.184731729
	C76	1.543761652	8.785670893	-4.234767062
	C77	-0.047403716	6.889309286	-3.919148537
	C78	-0.459725669	6.686907549	-2.437244762
	C79	-0.028093758	5.564308293	-4.714535934
	C80	-1.274973257	4.675338128	-4.711620434
	C81	-2.445687553	5.122101096	-5.602737622
	C82	-3.983391273	7.056505818	-5.244816680
	C83	0.165234955	6.004032435	-0.166747688
	C84	0.030620429	7.322342418	0.607817682
	C85	1.212391138	5.136290802	0.563878251
	C86	1.142094828	9.454715691	1.066060646
	C87	0.445783260	10.740524179	0.651314087
	C88	-1.037941655	11.811164714	-0.953024862
	C89	-2.284063703	12.428309545	-0.271944594
	C90	-1.261721397	11.655482789	-2.459334805
	C91	-2.418397911	10.733652880	-2.822083694
	C92	-4.167459346	12.086591266	1.257870399
	C93	-4.038450939	13.285558973	2.186141986
	C94	-4.790155942	10.886864291	2.026541330
	C95	-5.067330471	9.708125126	1.120417556
	C96	-6.279945049	9.619033031	0.419992553
	C97	-4.104158442	8.709423390	0.903953883
	C98	-6.514928395	8.586906684	-0.490809946
	C99	-4.309025901	7.684912830	-0.024917007
	C100	-5.517191418	7.638895864	-0.714971366
	C101	3.696473216	-4.693487377	-8.683549087
	C102	2.281303659	-4.431740526	-8.201532154
	C103	0.839480786	-2.808519704	-6.990917963
	C104	0.307616873	-1.526807624	-7.645562456
	C105	0.670006010	0.888638943	-7.899115766
	C106	1.824385139	1.859924689	-8.128471162
	C107	4.119995219	2.543726557	-7.695611296
	C108	4.686425717	2.608094271	-9.102329556
	C109	-3.138913461	-4.365644285	-8.570608895
	C110	-4.152902749	-3.249128315	-8.295050056
	C111	-2.590271137	-4.920757294	-7.237749614
	C112	-1.889943396	-6.276533324	-7.445637326
	C113	-1.114618374	-6.672474560	-6.210120770
	C114	-4.539725126	-0.873176947	-7.873599964
	C115	-4.086057815	0.335742162	-8.667469747
	C116	-4.882292104	-0.584555487	-6.390700585
	C117	-3.618275471	-0.482716876	-5.589165531
	C118	-2.889928488	0.716464887	-5.549651660
	C119	-3.044995528	-1.648964795	-5.050697089
	C120	-1.575632397	0.723743186	-5.069278899
	C121	-1.737846211	-1.636036751	-4.563728282
	C122	-0.985589689	-0.457832920	-4.613902413
	C123	7.697574188	-6.615622113	-4.509829511

	C124	8.921495252	-6.615816242	-3.577717247
	C125	6.417829568	-6.974349946	-3.738767361
	C126	5.127535348	-6.791515046	-4.472410825
	C127	4.689523272	-7.469025225	-5.591031148
	C128	3.041155215	-6.204682903	-4.821938617
	C129	10.218625059	-7.689686797	-1.832923420
	C130	11.539879617	-7.515288198	-2.549152883
	C131	0.672420008	-6.868159582	0.425755962
	C132	2.175548348	-6.944109700	0.680941190
	C133	3.049315247	-7.205393100	-0.581175216
	C134	2.943687696	-8.694295394	-1.001484981
	C135	3.929713947	-9.130964093	-2.087240906
	C136	3.939758748	-10.652478369	-2.357363268
	C137	4.517989861	-6.898064107	-0.247986031
	C138	3.425532199	-0.914445287	-2.164574341
	Fe139	3.563891322	-2.310668327	-3.550302231
	Fe140	1.955085797	0.586831668	-1.329138267
	Fe141	4.873623596	0.361864043	-1.599652140
	Fe142	3.453409186	0.204913616	-3.756395304
	Fe143	3.705100357	2.517434262	-2.459073579
	Fe144	4.761705881	-2.174721987	-1.342047732
	Fe145	1.990158045	-2.182422808	-1.155074167
	H146	-6.412535157	-7.521386027	1.372618091
	H147	-6.719805387	-6.675229159	0.010150823
	H148	-9.659419266	-4.622008824	2.328457447
	H149	-8.964558681	-6.198548466	0.713785915
	H150	-8.685416671	-7.174522782	2.151883529
	H151	-7.753145225	-2.574691611	2.655466289
	H152	-8.499315535	-5.162469321	4.946214151
	H153	-9.354078428	-2.637472520	3.428683200
	H154	-7.607752822	-5.589398987	7.053646621
	H155	-5.873878802	-5.439637085	4.548756602
	H156	-7.440388493	-3.836534520	7.313439778
	H157	-2.800647301	-1.426628585	3.401870312
	H158	-4.891775320	-3.334828830	3.686310313
	H159	-3.629250506	-5.774364208	4.095795338
	H160	-4.241946776	0.741695650	7.314083976
	H161	-5.317630570	-1.614227225	6.021161656
	H162	-3.230628849	-5.696402468	5.830693800
	H163	-4.543233686	-1.098877450	3.360959658
	H164	-3.825282240	-2.773742481	8.088022428
	H165	-5.352157437	-0.456853456	8.040075289
	H166	-0.831119593	-2.811980600	3.638834152
	H167	-1.008670614	-1.924162200	2.112107003
	H168	0.587134632	-2.525411761	2.600826571
	H169	-1.010116886	-0.443280880	4.194859457
	H170	-2.696745680	-3.676301342	9.897946603
	H171	-1.080960332	2.632241788	4.686221329
	H172	-0.559504951	0.683695093	8.700715718
	H173	-0.366393353	4.133437186	8.719438331
	H174	-0.285433771	0.474366275	1.932528816
	H175	1.397490485	0.227070595	2.417896495
	H176	9.395270552	2.383597562	4.030130479
	H177	-1.815302716	4.142572293	5.262761892
	H178	-1.414818305	4.991403545	7.567338104
	H179	-0.070134656	4.084763198	4.914350076
	H180	0.349975824	4.989245116	7.333604276
	H181	-1.502803278	2.537647736	7.157815277
	H182	1.468137957	2.724482971	6.418903001
	H183	-0.565391719	0.628801467	6.061946504
	H184	0.364710665	1.349868219	3.325430574
	H185	1.502974710	4.304485774	-0.094138310
	H186	0.721669552	4.720124358	1.455424944
	H187	2.664324719	5.592904673	1.812835789
	H188	3.346150432	5.203517091	4.383052162
	H189	4.792121460	4.443810294	1.246525285
	H190	6.605815731	2.779249278	0.974901705
	H191	6.603996068	2.096004051	5.237994861
	H192	-0.495366637	-2.059950518	5.961382614
	H193	0.572383574	-4.840895792	6.826248342
	H194	1.332844656	-4.004548390	8.197664147
	H195	-1.461551052	-3.612231328	7.242961395
	H196	-2.200650012	-2.046053055	10.413561710
	H197	4.755200500	3.724223759	5.499995896
	H198	1.884922324	-1.398092334	4.358349778
	H199	0.300791789	1.763037281	11.085344959
	H200	-0.003796924	0.027835071	10.814515260
	H201	2.577138532	0.050595651	9.910607280
	H202	11.380123972	-6.648422538	4.295890370
	H203	-1.903261595	-6.358971610	-3.420688665

H204	1.243566396	-6.221548806	-1.295219475
H205	-2.646832478	-7.049988377	-7.636903199
H206	-1.216880827	-6.228767437	-8.314785687
H207	-3.410898517	-5.050887933	-6.515134339
H208	-3.477063810	0.090898693	-9.569845562
H209	-3.751306804	-5.156433357	-9.030059974
H210	-3.515581169	-2.421363010	-1.896073271
H211	-3.971233677	-5.096809562	-0.460670816
H212	-2.638600552	-1.834229295	-8.149232704
H213	-5.492712188	-1.161189742	-8.364933949
H214	-1.450524054	-3.315819889	-9.181910483
H215	-1.372310196	-6.011355076	-1.781978735
H216	-1.901622139	-4.182699721	-6.798643489
H217	-1.644681845	-4.777399244	-9.917058296
H218	-6.904162821	-3.786022218	-2.195260513
H219	-3.548783567	-2.487755706	-0.125156355
H220	0.503039099	-5.940901732	-7.057717511
H221	-2.355979476	-4.409883514	-0.510470512
H222	-3.805487736	2.859925030	2.956568477
H223	-2.772362417	0.711834120	1.502115879
H224	-8.050335994	1.145557494	4.098236717
H225	-7.299960020	-0.050864403	1.462395617
H226	-6.978050325	4.206126345	2.318650836
H227	-8.276315208	9.046973481	3.928993982
H228	-6.899817272	5.611722178	5.138384648
H229	-8.958209341	5.638235740	3.282807400
H230	-6.321603194	7.000842480	2.448485047
H231	-11.003521448	9.512911592	0.721736455
H232	-3.404757947	4.326294285	0.960651848
H233	-4.964819208	3.851062103	0.279816781
H234	-9.837474091	10.642522188	2.693759386
H235	-8.435344161	5.719128010	1.599424644
H236	-6.228894290	2.050804495	1.478005470
H237	-6.975043785	-0.247838325	3.936454505
H238	-10.056954481	1.625522355	0.065107752
H239	-2.233116150	1.231975515	-2.676949592
H240	-10.315827707	-0.100716281	0.400658705
H241	10.341127103	3.463096289	2.123286043
H242	8.727677525	3.574570561	1.897924645
H243	1.369698714	5.808091543	-1.944789937
H244	-0.809504927	5.499795534	-0.095510995
H245	1.619020814	8.068347592	-0.455517538
H246	-3.630773898	3.264147989	-1.791559947
H247	-5.324963648	3.866670709	-2.926627647
H248	-7.204728464	2.960407151	-2.383015072
H249	-4.963558572	5.180694534	-2.102267875
H250	-5.689894212	1.014214130	-3.786673112
H251	-7.467905615	0.874793210	-3.649932856
H252	-0.324938780	11.341847833	-2.941116564
H253	-1.513812221	12.653090834	-2.851553493
H254	-1.228314549	9.762579660	-4.210224460
H255	-2.894522436	9.207694171	-4.049690843
H256	-4.099303328	8.523299686	-6.668466818
H257	-2.941536075	4.240201875	-6.035649879
H258	-7.471093706	8.522578470	-1.017279618
H259	-3.164697196	8.713389573	1.463678550
H260	-4.938462420	7.373961601	-3.443525677
H261	0.806104191	4.958611075	-4.324495030
H262	2.082694861	6.853185907	-3.907278618
H263	-1.633344978	4.499485879	-3.684934586
H264	-8.437918148	-0.623474811	-0.880277008
H265	3.307456121	9.541292322	-5.181973111
H266	-6.290678449	-1.315424396	-2.651321135
H267	0.238097557	5.813309465	-5.755060367
H268	-3.519653223	6.959399388	-0.225746723
H269	3.086575413	10.035456790	-3.491195945
H270	-3.384920128	7.047347098	-7.214436474
H271	-5.421799972	8.420804704	-4.736496362
H272	-5.360413265	-3.916159957	-2.821141629
H273	-5.819356863	-3.498505824	-0.188836006
H274	-7.059279332	10.366819137	0.585693905
H275	-5.497272862	1.136468030	-1.305943151
H276	-0.773332013	7.592678919	-4.339153319
H277	-2.063552980	5.717595628	-6.441985623
H278	-0.947031728	3.697608131	-5.089638349
H279	-3.847161238	5.435138409	-4.028409767
H280	0.831974525	9.223433485	2.095872708
H281	-0.597851902	9.708913850	-0.775046917
H282	-0.270611726	12.589854345	-0.821942140
H283	2.221159000	9.662114571	1.075036627

H284	-2.566075483	10.737860968	0.813540070
H285	4.180515843	-3.744218625	-8.957464271
H286	4.246522433	-5.090491620	-7.805403600
H287	7.618422781	-5.581659494	-4.876396805
H288	4.633058291	-5.933626174	-10.004973270
H289	3.113142163	-6.407758483	-9.617115985
H290	5.340545045	4.703078588	-1.475836174
H291	5.692525342	7.510773053	-0.406826139
H292	4.535551279	7.709951712	-4.486445962
H293	4.013095588	5.642966305	-0.722133582
H294	6.706446371	7.947113149	-1.615590653
H295	4.931684608	2.223113642	-7.025100796
H296	5.704324524	6.455721928	-3.147998005
H297	5.557178758	-6.404553236	5.308079840
H298	7.254117644	-7.592236303	3.046833308
H299	3.191386950	0.755847429	-7.000643899
H300	5.233121887	0.363917647	2.954371460
H301	0.182981790	0.721575011	-8.868227287
H302	-1.293271488	-2.541874687	-4.144478207
H303	-0.065571597	1.414256940	-7.265841980
H304	4.637693959	-3.945366508	4.659774171
H305	1.675485038	-0.489210003	-6.557518936
H306	4.266779753	-4.580985747	3.048580950
H307	5.017243656	-6.830795729	3.666026205
H308	6.706048688	-5.144291091	2.746101266
H309	7.019947134	-4.604319368	4.404554526
H310	-5.515952171	-1.407483958	-6.029830214
H311	6.229452966	-8.862016722	5.559837406
H312	5.273160793	-11.455807224	4.237152757
H313	0.039577147	-0.455442126	-4.250204550
H314	6.781828020	-11.049821412	6.701733718
H315	5.217741007	-11.486587847	6.495535802
H316	5.862407498	-2.937955044	2.114352860
H317	-0.991394898	1.645745059	-5.058333150
H318	-3.620923852	-2.577546142	-5.035742349
H319	7.625498276	-10.029113678	-1.017774049
H320	6.475383774	-8.030582221	-3.425104199
H321	6.682162502	-8.140140805	0.432577726
H322	6.721417108	-10.758126625	-2.057435321
H323	4.426952020	1.737831125	-9.753905087
H324	6.491724653	-5.088364657	-0.107157785
H325	2.363633010	-7.702745987	1.449201738
H326	2.896389032	-2.631441149	-7.455028994
H327	3.118204712	-9.293875680	-0.094621541
H328	2.747513159	-7.456894704	-6.491720035
H329	2.050858465	-5.777384003	-4.723859930
H330	6.935922131	-11.963965836	4.550972733
H331	5.686341737	-0.720874768	1.662908009
H332	8.483710668	-5.678667560	0.944769555
H333	7.381807599	-3.828539398	-0.283806758
H334	9.172631687	-6.532850467	3.074695898
H335	4.187205116	-2.152053726	5.207826791
H336	3.673061006	-8.629363715	-3.033521436
H337	4.950626750	-8.823841702	-1.821689862
H338	1.911656039	-8.897695503	-1.327876294
H339	1.003093464	-2.657755072	-5.912273472
H340	-3.340331502	1.628923315	-5.948583463
H341	-5.469524065	0.345362908	-6.367026673
H342	2.456180987	-5.967648163	1.109512481
H343	8.598411155	-7.230819684	0.944015176
H344	7.496683393	-9.141417370	1.276792833
H345	4.052746953	-0.508540005	4.817474226
H346	5.187321871	-8.198096964	-6.220161658
H347	6.382765840	-6.387016068	-2.814655648
H348	0.100112487	-3.598388320	-7.138810521
H349	3.813049134	3.555388316	-7.389331932
H350	7.807403349	0.544816873	3.702673107
H351	7.791338067	0.816096675	1.944020276
H352	8.724702773	-7.185269075	-6.192378402
H353	8.073003867	-8.446973573	-5.378014066
H354	10.240742445	-8.674883136	-1.341115202
H355	8.419799217	-8.450667545	-2.682950401
H356	11.542154246	-7.840783301	-3.617706092
H357	10.123803746	-6.929635129	-1.041840271
H358	10.523787874	0.534188981	1.836710594
H359	-3.018056608	13.453935465	2.603204737
H360	10.469919679	-6.274097248	5.778964883
H361	9.835675490	-3.992933564	4.345408725
H362	-4.843490470	12.376850647	0.436120219
H363	-4.111377601	10.591714872	2.842257850

H364	-5.723034856	11.242982859	2.486984321
H365	0.962979889	-3.227270589	-0.825539289
H366	-6.650915700	6.589331142	-1.901090126
H367	0.534459305	-1.431508807	0.527230855
H368	3.242131752	-0.780724250	0.524419224
H369	2.619984439	-3.164897472	0.603371438
H370	-1.588314903	-0.156060856	-0.691087690
Mo371	3.810824543	-4.669465042	-2.158251179
N372	2.281993180	-0.733034315	0.156166404
N373	1.521881686	-1.688997828	0.710889513
N374	-7.159483945	-7.261761451	0.723558887
N375	-8.709625721	-4.456477069	2.651998742
N376	-7.913420299	-4.434072253	5.347974619
N377	-5.230109865	-5.258417854	5.320858549
N378	-4.049471819	-2.964473132	4.121069916
N379	-4.499264167	-1.041194215	6.236210626
N380	-3.157376953	-2.157423462	8.558006247
N381	-0.651675894	-3.482868615	7.855777036
N382	0.505099373	-1.995544862	5.779874563
N383	0.393477146	0.967057230	6.161767089
N384	0.307750555	1.085807175	9.063250677
N385	5.946632022	-10.865474170	6.141975958
N386	6.588286951	-8.769206382	4.603619631
N387	5.448741098	-2.852429189	3.045218975
N388	4.578129619	-1.380495983	4.662596679
N389	5.263779945	-0.587357670	2.595050370
N390	9.273957091	-6.551571068	4.113082067
N391	-6.021600478	-3.288578321	-2.327394928
N392	-2.058559920	-5.875873258	-2.530383195
N393	-6.266884250	-0.739893544	-1.792628396
N394	-8.512798870	0.333141806	-0.540816414
N395	-7.854209436	0.468264090	2.153135695
N396	-5.698143901	2.235399116	2.328930666
N397	-3.248146416	2.478540587	-1.210274210
N398	-2.311479535	0.620119701	-0.610543057
N399	-6.353233053	4.907805059	2.708028709
N400	-9.892863514	7.762144248	1.314507540
N401	-9.701172599	9.669816761	2.430823996
N402	9.504274381	2.908240722	1.927501533
N403	6.182665757	7.154931494	-1.233816514
N404	3.946927914	8.141062291	-3.778047392
N405	1.281630770	7.476622764	-4.040883181
N406	-3.453843914	5.894280656	-4.871891276
N407	-3.705991336	7.615977194	-6.438241556
N408	-4.745192577	7.755532909	-4.372597390
N409	0.486871405	6.202427832	-1.583026600
N410	0.882737236	8.309937622	0.213193784
N411	-0.509762240	10.617269904	-0.320113016
N412	-2.192363963	9.928899480	-3.896190769
N413	-2.869261115	11.703897541	0.715377911
N414	3.684042955	-5.588205775	-9.843770651
N415	2.085783225	-3.233712939	-7.613297184
N416	0.997820587	-0.407973832	-7.333225636
N417	3.030587914	1.591940537	-7.567131890
N418	-2.149545064	-3.956924668	-9.575255115
N419	-3.649419224	-1.995562951	-8.106074105
N420	7.887916007	-7.486507958	-5.684243325
N421	4.079356075	-5.991701688	-4.011190082
N422	3.382391007	-7.083377814	-5.790926713
N423	9.091022514	-7.680871634	-2.748549717
N424	0.018794814	2.403502679	-0.117749313
N425	0.773307306	1.692693650	-0.561872855
O426	-6.520030484	-5.062789048	2.484476006
O427	-7.022646129	-2.353237414	5.070939093
O428	-4.971729387	-4.389654437	7.418868278
O429	-2.226354403	-3.414586929	5.411787410
O430	-2.657321509	0.026230758	5.441566382
O431	-2.506349742	-0.005488379	8.973093412
O432	0.111573228	-2.855579361	9.926271510
O433	2.545528304	-2.979427513	6.169591185
O434	2.577845752	0.676467539	5.539594940
O435	2.018368640	2.563822729	8.836870653
O436	2.583370329	0.772390938	11.819034104
O437	7.180701296	-10.085227687	2.795663110
O438	8.287121438	-7.054337398	6.128578354
O439	11.862923062	-4.092918670	4.551202051
O440	-5.751859065	-0.948819133	0.433326830
O441	-3.913836689	-4.862343544	-3.363303509
O442	-7.539061623	2.397508580	-0.493237426
O443	-6.633096890	2.723109548	-3.148381920

	O444	-9.830340537	1.542816125	2.620567344
	O445	-5.492841856	1.862075276	4.572013715
	O446	-4.223674256	5.351512012	3.409884817
	O447	-6.558398951	7.615981042	4.973250369
	O448	10.798652831	0.244838432	3.839120419
	O449	3.630293883	5.100393092	3.456393139
	O450	3.449400139	8.283710990	-1.556753021
	O451	0.681017678	9.656626551	-4.455897130
	O452	-1.610428625	6.970703290	-2.069745480
	O453	-0.756243318	7.431309839	1.557105777
	O454	2.367386933	5.895705054	0.931059674
	O455	0.735456334	11.819941586	1.182441890
	O456	-2.673504006	13.560210895	-0.593895929
	O457	-3.497852245	10.758658546	-2.213575840
	O458	-4.981317234	13.999796898	2.487658877
	O459	-5.699277978	6.630312453	-1.684129787
	O460	1.387726683	-5.304840785	-8.317975311
	O461	-0.661815607	-1.538773730	-8.431101636
	O462	1.618971174	2.890202910	-8.790525276
	O463	5.400492651	3.515514424	-9.495219134
	O464	-5.370473773	-3.485383882	-8.226860802
	O465	-1.627257834	-7.203472771	-5.230854463
	O466	0.198909615	-6.362964782	-6.196448836
	O467	-4.390672147	1.490015786	-8.404931345
	O468	9.760620604	-5.694166683	-3.609455635
	O469	12.559510493	-7.117674116	-2.002883068
	O470	0.318888403	-6.361232592	-0.762763465
	O471	-0.163499235	-7.194429846	1.266145220
	O472	5.085593425	-11.215395282	-2.475669015
	O473	2.823613488	-11.232369463	-2.473866103
	O474	5.108786488	-6.000929407	-0.997683209
	O475	5.091011050	-7.507008768	0.673183546
	O476	2.607563836	-6.325654022	-1.612357912
	O477	9.058696528	-6.411371753	1.276262708
	O478	7.153063471	-4.521270209	0.363118101
	O479	-4.573162044	4.461566500	-2.656397263
	O480	7.683411688	-10.593726831	-1.820520854
	O481	7.567492361	-8.594208573	0.454108884
	S482	3.321966608	4.691703833	-2.853386071
	S483	1.827165732	1.702112675	-3.346698329
	S484	1.841334394	-3.608409556	-2.970893064
	S485	3.728528850	1.832055645	-0.371513409
	S486	0.243521912	-0.912480993	-1.752775820
	S487	3.555541976	-1.187634266	-5.455303021
	S488	3.839660538	-3.665602781	0.158590928
	S489	5.350057562	1.408009522	-3.521850513
	S490	5.532659267	-3.334387099	-3.085749766
	S491	6.351260156	-0.871673922	-0.541483284
	end			

Fe2-brNH-NH-Fe6H-3b2 to Fe2-brNH-NH2-Fe6H

35, S=1

reactant

		bm523b2toNth.car_2			
Fe(139)	-2.298	C1	-7.718063292	-5.288837599	2.205875066
Fe(140)	0.095	C2	-8.203819860	-6.516072987	1.424183980
Fe(141)	-2.592	C3	-8.388939633	-3.182976633	3.281777476
Fe(142)	2.531	C4	-7.703199771	-3.287688117	4.641094103
Fe(143)	2.484	C5	-7.210662010	-4.650262677	6.605957969
Fe(144)	2.463	C6	-5.686746582	-4.738631793	6.473975256
Fe(145)	0.049	C7	-3.788997943	-5.211706131	5.010099160
		C8	-3.270165950	-3.780150304	4.861000628
		C9	-3.717698629	-1.543947995	3.931397576
		C10	-3.556775734	-0.775746525	5.250479812
		C11	-4.378090018	-0.304751440	7.471902601
		C12	-3.238214825	-0.792834101	8.377386713
		C13	-2.259829184	-2.705261526	9.550598404
		C14	-0.817016302	-3.008625578	9.121616129
		C15	0.647899354	-3.878811657	7.346404816
		C16	1.324777328	-2.897061049	6.386374413
		C17	0.981787409	-1.007692807	4.817683261
		C18	1.413450305	0.287323481	5.533888101
		C19	-0.041807664	-0.777457750	3.697757596

	C20	0.454481317	0.337253229	2.771476483
	C21	-0.320569996	-2.109055253	2.990100289
	C22	0.627646265	2.218404194	6.876915347
	C23	1.057534762	1.971868867	8.336770915
	C24	-0.629353548	3.117000409	6.768464610
	C25	-0.506931820	4.370682181	7.641644981
	C26	-0.896351759	3.501875810	5.307030551
	C27	0.591299249	0.903343059	10.471298400
	C28	2.043018915	0.579978602	10.754272658
	C29	6.218319848	-11.142589961	4.737920437
	C30	6.723603683	-9.930261179	3.958256790
	C31	6.995701970	-7.459930029	4.115423810
	C32	8.242394006	-6.994733354	4.891663040
	C33	5.848548224	-6.445906205	4.255424275
	C34	6.236878088	-5.060283212	3.744385295
	C35	5.036568591	-4.126497831	3.645305327
	C36	5.114090838	-1.624781422	3.438092611
	C37	10.509872036	-6.089877335	4.696507078
	C38	10.755335159	-4.608597011	4.518531442
	C39	-5.450645520	-2.891349622	-1.025465869
	C40	-5.834644018	-1.438862365	-0.694325564
	C41	-3.908431815	-2.991294778	-1.025236997
	C42	-3.326823064	-4.419789860	-1.028757436
	C43	-3.130762121	-5.058317867	-2.402755649
	C44	-6.390897427	0.708943460	-1.749124924
	C45	-7.524516406	1.216889746	-0.856468876
	C46	-6.558382713	1.278751888	-3.173586377
	C47	-9.558010005	0.718993659	0.396176866
	C48	-9.085866275	0.958177986	1.835250491
	C49	-7.272799692	0.700229930	3.471046844
	C50	-6.077260915	1.649411498	3.516202176
	C51	-4.617701722	3.214537392	2.324745052
	C52	-5.049708830	4.584750153	2.907207980
	C53	-4.111455089	3.488863294	0.902368115
	C54	-3.433791531	2.370208571	0.172896029
	C55	-2.828789329	1.192510775	0.540023397
	C56	-2.562395340	1.418368163	-1.650725183
	C57	-6.892099599	6.229324888	2.996063642
	C58	-6.763619894	6.507679695	4.492285652
	C59	-8.374932686	6.237201037	2.560970514
	C60	-9.005866439	7.583606488	2.355732347
	C61	-8.880801018	8.763109673	3.064487216
	C62	-10.295971020	9.021098334	1.379197614
	C63	9.271457266	1.945765456	3.023699951
	C64	10.276200956	0.814816628	2.902421803
	C65	7.863561934	1.310189679	2.932633273
	C66	6.750043079	2.318471550	3.096747725
	C67	6.214882567	2.615654840	4.364591675
	C68	6.213190276	2.990779377	1.983329884
	C69	5.178383062	3.542114963	4.520221570
	C70	5.191856007	3.929605767	2.125359355
	C71	4.665053177	4.195317302	3.392555386
	C72	5.180834963	6.689476935	-2.189478649
	C73	4.113413036	7.763305992	-2.463163953
	C74	4.533024618	5.424364903	-1.629209756
	C75	3.029193746	9.175802459	-4.177351467
	C76	1.550846303	8.779798117	-4.221455299
	C77	-0.045757338	6.888038920	-3.916268848
	C78	-0.460268356	6.694377050	-2.433601105
	C79	-0.030964397	5.557020069	-4.702629436
	C80	-1.281350174	4.672869860	-4.695739367
	C81	-2.450561193	5.121639609	-5.587837845
	C82	-3.988163942	7.057051864	-5.231699665
	C83	0.161412291	6.011549560	-0.161801272
	C84	0.029075523	7.332387817	0.608405901
	C85	1.206381126	5.144878305	0.573925194
	C86	1.148057986	9.461243340	1.060920123
	C87	0.449667934	10.744592166	0.643791170
	C88	-1.038142941	11.807523699	-0.959160993
	C89	-2.280248886	12.426973162	-0.271818964
	C90	-1.272586857	11.653142487	-2.463786340
	C91	-2.427658462	10.727147115	-2.818962542
	C92	-4.164540173	12.084752577	1.256998490
	C93	-4.032318163	13.283826196	2.185288274
	C94	-4.787499010	10.885740930	2.026473709
	C95	-5.066881593	9.706080992	1.121962912
	C96	-6.283038735	9.613558177	0.428168446
	C97	-4.103477031	8.708400948	0.901607028
	C98	-6.521489620	8.579832159	-0.480185562
	C99	-4.311541197	7.682464562	-0.025126509

	C100	-5.523251046	7.633182434	-0.708894960
	C101	3.695898162	-4.691444856	-8.691128292
	C102	2.282920158	-4.428260391	-8.201874845
	C103	0.845001579	-2.806987756	-6.982727885
	C104	0.309737429	-1.527886619	-7.638249928
	C105	0.666314180	0.888848218	-7.889611211
	C106	1.815598804	1.866402023	-8.118370248
	C107	4.111784546	2.557708092	-7.687520872
	C108	4.703609258	2.595086949	-9.084396724
	C109	-3.145990862	-4.364340369	-8.574648158
	C110	-4.162056002	-3.249693382	-8.298659655
	C111	-2.597230514	-4.917481333	-7.240780406
	C112	-1.890832291	-6.270585475	-7.447353481
	C113	-1.115394776	-6.662088237	-6.210494080
	C114	-4.550777527	-0.875923559	-7.867437385
	C115	-4.098319986	0.334536912	-8.659467142
	C116	-4.896823836	-0.590473296	-6.384577953
	C117	-3.631874809	-0.485436875	-5.585550750
	C118	-2.906326099	0.715328780	-5.547835873
	C119	-3.050503329	-1.652635996	-5.057471976
	C120	-1.586357299	0.723749442	-5.083653099
	C121	-1.736573006	-1.639640210	-4.590371140
	C122	-0.987281807	-0.459654056	-4.645759645
	C123	7.701748501	-6.611984463	-4.500847967
	C124	8.927577242	-6.613681962	-3.572362833
	C125	6.422881752	-6.990110716	-3.735943052
	C126	5.133222164	-6.798728361	-4.469880800
	C127	4.698224803	-7.476625508	-5.589024638
	C128	3.050286044	-6.204442762	-4.833474340
	C129	10.225852012	-7.682935319	-1.824431559
	C130	11.545524493	-7.512443899	-2.543749598
	C131	0.660960978	-6.849321692	0.406925409
	C132	2.162225433	-6.939556245	0.664784165
	C133	3.040446637	-7.201296845	-0.595920838
	C134	2.943430507	-8.693463184	-1.006521867
	C135	3.926113574	-9.134784123	-2.093249475
	C136	3.933641289	-10.657826852	-2.354890734
	C137	4.509208972	-6.887483957	-0.260874536
	C138	3.421375845	-0.919724436	-2.168064389
	Fe139	3.585393371	-2.301681156	-3.565880822
	Fe140	1.932109303	0.568008015	-1.334429793
	Fe141	4.856007949	0.360522300	-1.568538693
	Fe142	3.456785616	0.217250966	-3.745997793
	Fe143	3.677027682	2.513136287	-2.434121163
	Fe144	4.748506495	-2.177032273	-1.341306815
	Fe145	1.961587440	-2.175161176	-1.217582721
	H146	-6.438297922	-7.535853823	1.350167410
	H147	-6.753575584	-6.682781037	-0.006011292
	H148	-9.657947139	-4.612677526	2.334182242
	H149	-8.990618143	-6.198586822	0.721324772
	H150	-8.699313361	-7.178061205	2.155533748
	H151	-7.720362989	-2.588164915	2.641960635
	H152	-8.480993015	-5.157626434	4.941784115
	H153	-9.323120792	-2.620718635	3.412379949
	H154	-7.582078604	-5.584974841	7.046429682
	H155	-5.859120200	-5.440317598	4.541613746
	H156	-7.419448957	-3.831677875	7.307685367
	H157	-2.782555293	-1.419875036	3.374509067
	H158	-4.872005039	-3.326520825	3.661193820
	H159	-3.616662750	-5.761483674	4.074912733
	H160	-4.204505459	0.761763206	7.280888365
	H161	-5.292640244	-1.598559336	6.001210000
	H162	-3.209849405	-5.690766570	5.808416602
	H163	-4.524837317	-1.090579767	3.339994273
	H164	-3.813797430	-2.753927407	8.074219686
	H165	-5.329031753	-0.420992249	8.008964270
	H166	-0.808024900	-2.821505970	3.666555182
	H167	-0.985552491	-1.961868714	2.123726144
	H168	0.607891048	-2.559811317	2.617076946
	H169	-0.977299175	-0.429057059	4.161720091
	H170	-2.705541486	-3.645246070	9.908020963
	H171	-1.040012235	2.622668283	4.663989529
	H172	-0.550073352	0.691174375	8.693525044
	H173	-0.373657585	4.126651724	8.705313876
	H174	-0.203125165	0.435308297	1.897201585
	H175	1.467366028	0.142434966	2.397637362
	H176	9.383462837	2.381319228	4.035215810
	H177	-1.803902404	4.119019722	5.237331310
	H178	-1.416715702	4.980326788	7.544479476
	H179	-0.054761415	4.089963735	4.905444200

H180	0.350207492	4.986266282	7.325034664
H181	-1.488208457	2.525103664	7.135655403
H182	1.485193946	2.724941171	6.410987319
H183	-0.545848509	0.628008507	6.052416183
H184	0.466434412	1.311853081	3.281467572
H185	1.503393548	4.313548017	-0.082124905
H186	0.710762665	4.727618823	1.462267902
H187	2.657298112	5.595603720	1.826855559
H188	3.341783807	5.207551228	4.391323565
H189	4.770642315	4.427005475	1.252422701
H190	6.582872963	2.760251492	0.981868928
H191	6.603546991	2.105340724	5.249412773
H192	-0.489359294	-2.049633028	5.968163552
H193	0.562723121	-4.841775256	6.819003972
H194	1.327570193	-4.018729814	8.195815289
H195	-1.463154210	-3.599668711	7.239755726
H196	-2.188221612	-2.013332768	10.399135237
H197	4.757459990	3.735950183	5.510723277
H198	1.901448221	-1.414740609	4.375113376
H199	0.296756342	1.777304111	11.075509771
H200	-0.002013760	0.039494337	10.810793408
H201	2.583577126	0.052969005	9.934578248
H202	11.366665275	-6.653922226	4.296853719
H203	-1.909462621	-6.350839096	-3.416252864
H204	1.242455194	-6.221221697	-1.318451929
H205	-2.644429550	-7.047252562	-7.639006641
H206	-1.217470068	-6.220025399	-8.316067992
H207	-3.418585163	-5.051359997	-6.519433501
H208	-3.488541328	0.091075268	-9.561724753
H209	-3.755780283	-5.157022106	-9.034966107
H210	-3.517442101	-2.421416249	-1.883916485
H211	-3.978971339	-5.095612308	-0.448871568
H212	-2.648787379	-1.835210633	-8.140257244
H213	-5.502852053	-1.164716090	-8.360871259
H214	-1.456927226	-3.313863516	-9.181486230
H215	-1.373529188	-6.002287478	-1.779088056
H216	-1.912153743	-4.176786042	-6.799843960
H217	-1.652425627	-4.771475508	-9.923834745
H218	-6.912531051	-3.772874174	-2.186966056
H219	-3.552216891	-2.488498332	-0.113990041
H220	0.502650301	-5.934874736	-7.061035799
H221	-2.361849340	-4.412235014	-0.500571833
H222	-3.802537710	2.859934386	2.970825298
H223	-2.723872124	0.730191226	1.513690901
H224	-8.050898656	1.140264970	4.110896854
H225	-7.301980370	-0.050126872	1.471173436
H226	-6.974619072	4.206155222	2.301875153
H227	-8.283199493	9.039953013	3.926026329
H228	-6.904462608	5.604484805	5.134863199
H229	-8.962217857	5.631522065	3.274506837
H230	-6.325601297	6.998849849	2.447988893
H231	-11.003760115	9.509309516	0.713784214
H232	-3.402085884	4.330028407	0.976225783
H233	-4.956453260	3.845511635	0.290883670
H234	-9.842319662	10.636659646	2.689358882
H235	-8.435599521	5.715650741	1.592270175
H236	-6.220595939	2.044662441	1.486038002
H237	-6.971737387	-0.248701293	3.941725283
H238	-10.047277819	1.647603638	0.071911706
H239	-2.249480508	1.229492941	-2.675219634
H240	-10.310943661	-0.078952296	0.402003500
H241	10.326787119	3.451982175	2.123109910
H242	8.713028704	3.560330875	1.896826366
H243	1.367037682	5.811085849	-1.938226829
H244	-0.813960400	5.508833757	-0.090165413
H245	1.623914690	8.067368459	-0.453327961
H246	-3.639913825	3.262611716	-1.779815468
H247	-5.331217324	3.860429447	-2.921238624
H248	-7.247525813	2.970158854	-2.455380535
H249	-4.971282588	5.172549380	-2.092542635
H250	-5.687857387	0.998696757	-3.782228178
H251	-7.464052144	0.856400598	-3.639385226
H252	-0.337970187	11.344264315	-2.952406457
H253	-1.533054725	12.650434634	-2.850996443
H254	-1.227060568	9.738434165	-4.182839443
H255	-2.890758440	9.176810608	-4.020386401
H256	-4.083260651	8.534885851	-6.648954109
H257	-2.947355348	4.241162729	-6.022547718
H258	-7.480330753	8.514651295	-1.001758119
H259	-3.160577764	8.714026840	1.455576827

H260	-4.959170567	7.363876261	-3.436679825
H261	0.800559730	4.950833895	-4.307473355
H262	2.084494054	6.841618877	-3.908720784
H263	-1.639984507	4.501167033	-3.668483571
H264	-8.442503183	-0.601257654	-0.888051739
H265	3.314036522	9.525748390	-5.177714834
H266	-6.282563761	-1.308577256	-2.637005026
H267	0.237387988	5.796702941	-5.744814507
H268	-3.521785976	6.958106103	-0.229120890
H269	3.098643303	10.030412756	-3.488604422
H270	-3.338436449	7.068680682	-7.188333655
H271	-5.428303142	8.422476034	-4.725915360
H272	-5.367772106	-3.913198169	-2.810020946
H273	-5.824718400	-3.497572182	-0.178811781
H274	-7.062683980	10.360411191	0.596142575
H275	-5.483173365	1.146709753	-1.305991703
H276	-0.768467722	7.590577898	-4.342080173
H277	-2.066077493	5.716654006	-6.426343136
H278	-0.956969823	3.692989545	-5.070658033
H279	-3.855561692	5.433471530	-4.018062295
H280	0.840689641	9.233340623	2.092359517
H281	-0.592458106	9.707014444	-0.780084726
H282	-0.271653683	12.588059595	-0.834376892
H283	2.227128579	9.668646127	1.066070815
H284	-2.566191945	10.733746620	0.808237017
H285	4.181558119	-3.741712924	-8.960682993
H286	4.247662821	-5.095461123	-7.817110285
H287	7.614288279	-5.574942381	-4.856365799
H288	4.620248630	-5.930353184	-10.021267695
H289	3.099062804	-6.396017989	-9.631591787
H290	5.332561813	4.698533424	-1.431499145
H291	5.701309920	7.526937149	-0.399576949
H292	4.545089245	7.700658392	-4.473879719
H293	4.004837215	5.653618643	-0.693310732
H294	6.696209318	7.959071438	-1.626434938
H295	4.913084673	2.256756884	-6.994738740
H296	5.699919110	6.437832799	-3.127309632
H297	5.538462740	-6.397942678	5.312520953
H298	7.240483903	-7.582175911	3.053166573
H299	3.190469230	0.766243405	-6.994392763
H300	5.261848283	0.360580049	2.961961112
H301	0.177212880	0.724272800	-8.857878518
H302	-1.284646366	-2.547297519	-4.183520883
H303	-0.069615914	1.410147351	-7.253401049
H304	4.600402061	-3.944880995	4.634704071
H305	1.684423255	-0.488892231	-6.559621489
H306	4.249794034	-4.584495966	3.022706851
H307	4.997731698	-6.835386496	3.673635424
H308	6.685018278	-5.151257230	2.743522758
H309	6.993142165	-4.598466007	4.398496152
H310	-5.526706515	-1.416833508	-6.024668296
H311	6.213679198	-8.852135750	5.564714547
H312	5.284131646	-11.453920001	4.245708465
H313	0.043765363	-0.457398463	-4.300380386
H314	6.778026270	-11.019049249	6.716256911
H315	5.221588491	-11.482724903	6.506178448
H316	5.834658150	-2.945666226	2.089114020
H317	-1.006153100	1.648416162	-5.074299048
H318	-3.624492971	-2.582359879	-5.040036296
H319	7.615307248	-10.034308036	-1.007357234
H320	6.482195507	-8.053304107	-3.445865012
H321	6.680433863	-8.141437052	0.432258392
H322	6.711153491	-10.765142463	-2.046061854
H323	4.455640965	1.714276804	-9.725043935
H324	6.472777536	-5.076901483	-0.092613998
H325	2.343873000	-7.704080382	1.429508092
H326	2.901566684	-2.629355713	-7.454714318
H327	3.126280680	-9.283283559	-0.094602196
H328	2.761670646	-7.457474157	-6.502328022
H329	2.060877012	-5.774512829	-4.739180327
H330	6.948501503	-11.952634205	4.571059376
H331	5.667838872	-0.721766200	1.652224517
H332	8.476079990	-5.679298095	0.953196531
H333	7.373634011	-3.824319659	-0.261846827
H334	9.162500087	-6.529731671	3.079261014
H335	4.249999858	-2.160072193	5.224084790
H336	3.666893101	-8.638515463	-3.041638942
H337	4.947813203	-8.826895316	-1.831817501
H338	1.910539519	-8.904490894	-1.324034864
H339	1.011625979	-2.652662808	-5.905171531

H340	-3.361907838	1.627994879	-5.940136217
H341	-5.488395873	0.336711808	-6.359827324
H342	2.449752694	-5.968415762	1.101045686
H343	8.595083975	-7.231265412	0.947833609
H344	7.493592985	-9.137620512	1.286217870
H345	4.091452490	-0.519160725	4.835563515
H346	5.197064795	-8.210245626	-6.212167340
H347	6.383430209	-6.421851897	-2.799613817
H348	0.106921572	-3.598993892	-7.126027233
H349	3.792712988	3.573510456	-7.409367261
H350	7.794907725	0.540214975	3.715058890
H351	7.778417518	0.807512481	1.955648448
H352	8.736251903	-7.152246090	-6.187555901
H353	8.096196705	-8.429514330	-5.390006826
H354	10.246731201	-8.666378669	-1.329009800
H355	8.420345927	-8.440716032	-2.666003204
H356	11.547276630	-7.842116315	-3.610716279
H357	10.133770157	-6.919823698	-1.035633837
H358	10.510029012	0.521441781	1.850090766
H359	-3.011137233	13.450255728	2.601826861
H360	10.461370915	-6.278947239	5.783571712
H361	9.838258325	-3.988999389	4.362520226
H362	-4.841585731	12.376703773	0.436767093
H363	-4.108187785	10.590841045	2.841881460
H364	-5.719814450	11.242834109	2.487358883
H365	0.920369462	-3.232557086	-0.935942261
H366	-6.659899042	6.577468579	-1.887808838
H367	0.483721899	-1.480792880	0.474405824
H368	3.187269270	-0.820398581	0.522692778
H369	2.611661530	-3.199766156	0.599800073
H370	-1.584332422	-0.153561855	-0.699756330
Mo371	3.794530777	-4.670454156	-2.187067233
N372	2.232047522	-0.770342265	0.143751558
N373	1.469200628	-1.747381732	0.659056389
N374	-7.189884164	-7.269855471	0.709087651
N375	-8.704382358	-4.455480865	2.651501394
N376	-7.894470927	-4.428958283	5.342109167
N377	-5.212626095	-5.253510666	5.309925841
N378	-4.031470473	-2.956388938	4.099316069
N379	-4.473763836	-1.024926430	6.212525189
N380	-3.147443301	-2.137705382	8.546699131
N381	-0.653451547	-3.483072214	7.855598622
N382	0.512000952	-1.991299943	5.789543352
N383	0.413007182	0.966094793	6.153394825
N384	0.318478837	1.089582488	9.056430314
N385	5.942319521	-10.852633212	6.151373273
N386	6.581242052	-8.760701519	4.611810635
N387	5.432166420	-2.858030082	3.024993068
N388	4.624039109	-1.385272198	4.673616473
N389	5.263692684	-0.587887613	2.593866989
N390	9.261159133	-6.542872893	4.118096890
N391	-6.026263010	-3.280926472	-2.317713498
N392	-2.064352887	-5.870398108	-2.524430406
N393	-6.259168085	-0.732593823	-1.778460902
N394	-8.505712618	0.351551032	-0.536280918
N395	-7.854652451	0.470458944	2.162140665
N396	-5.692342408	2.230565300	2.338480254
N397	-3.253516530	2.476428622	-1.200876866
N398	-2.308810807	0.620505397	-0.608038820
N399	-6.354136339	4.904378584	2.703707453
N400	-9.894350552	7.758021986	1.306710695
N401	-9.705424018	9.664303112	2.425834730
N402	9.490590569	2.895221481	1.929886578
N403	6.186371945	7.166073252	-1.227450468
N404	3.953021068	8.131227776	-3.768107314
N405	1.286048702	7.469001040	-4.040612308
N406	-3.459096538	5.894218685	-4.858155200
N407	-3.705927341	7.620508279	-6.420994640
N408	-4.758630946	7.750045534	-4.362338877
N409	0.483649311	6.206865886	-1.578291198
N410	0.886197688	8.314604531	0.211868635
N411	-0.506013326	10.616356700	-0.326946984
N412	-2.194149365	9.905768150	-3.878990471
N413	-2.867626865	11.700454094	0.712406096
N414	3.674446620	-5.579123838	-9.855796398
N415	2.090925836	-3.233087329	-7.606289228
N416	1.000513962	-0.408283876	-7.328897925
N417	3.023878381	1.603662314	-7.557883641
N418	-2.156487893	-3.952449859	-9.577656949
N419	-3.659491268	-1.996905260	-8.100994931

N420	7.901270706	-7.467521626	-5.685004630
N421	4.085074153	-5.993562992	-4.017851861
N422	3.393850846	-7.086120869	-5.798094556
N423	9.094844585	-7.674392357	-2.737013433
N424	0.002021100	2.413286492	-0.142286600
N425	0.751959379	1.693934063	-0.578763424
O426	-6.520545567	-5.077684202	2.466869950
O427	-7.000544421	-2.350323976	5.060946517
O428	-4.948090782	-4.369763598	7.401096426
O429	-2.223122878	-3.400547935	5.411230389
O430	-2.630688118	0.035885502	5.410907757
O431	-2.489760386	0.013049420	8.957233272
O432	0.110730989	-2.867674561	9.929801827
O433	2.546518307	-2.988291377	6.171806723
O434	2.597635012	0.671101086	5.532335992
O435	2.032238763	2.564039330	8.829097017
O436	2.575271842	0.808153058	11.830623882
O437	7.190928256	-10.076278884	2.810092026
O438	8.269384575	-7.031226523	6.134518611
O439	11.865440865	-4.100672159	4.558281274
O440	-5.754724852	-0.950176678	0.450070284
O441	-3.928381064	-4.867033528	-3.349836261
O442	-7.509238876	2.403966222	-0.461453946
O443	-6.629624368	2.714404733	-3.174249005
O444	-9.821744071	1.563369882	2.624494015
O445	-5.504344094	1.875934080	4.586108279
O446	-4.234659504	5.347250515	3.432090873
O447	-6.565647598	7.609814363	4.972168234
O448	10.783926018	0.239448533	3.853691482
O449	3.622873998	5.099888466	3.464224286
O450	3.441420921	8.275177377	-1.551275328
O451	0.688105021	9.654202062	-4.428305022
O452	-1.609930623	6.985127568	-2.067844012
O453	-0.760018702	7.448835495	1.554988664
O454	2.356815141	5.907583989	0.949332852
O455	0.737215757	11.826081698	1.171673412
O456	-2.666316548	13.561545598	-0.588536685
O457	-3.510977546	10.761541806	-2.217916556
O458	-4.973778240	13.999749328	2.487402332
O459	-5.707165467	6.622544372	-1.676451735
O460	1.387280386	-5.299131860	-8.319794474
O461	-0.662596676	-1.541620236	-8.419625460
O462	1.603762421	2.896853759	-8.777923518
O463	5.424787413	3.494914596	-9.481257544
O464	-5.379678954	-3.486694797	-8.235978941
O465	-1.628598414	-7.188139741	-5.229011814
O466	0.198319699	-6.353564653	-6.198176456
O467	-4.404720994	1.488158102	-8.396121012
O468	9.773103233	-5.698416135	-3.614837378
O469	12.565537474	-7.112651453	-1.999719949
O470	0.314153113	-6.348616357	-0.785413745
O471	-0.177168648	-7.154912711	1.253096961
O472	5.078427624	-11.223877785	-2.466306070
O473	2.816491165	-11.235695190	-2.472113826
O474	5.096414300	-5.982797533	-1.004643164
O475	5.085849551	-7.500944375	0.655169597
O476	2.597956371	-6.328881055	-1.636368721
O477	9.054584267	-6.411557093	1.280824877
O478	7.140938226	-4.520107141	0.380465007
O479	-4.579405751	4.454267642	-2.647860125
O480	7.672956882	-10.601203235	-1.808302695
O481	7.565258224	-8.595135022	0.460321324
S482	3.311224918	4.694302238	-2.813901327
S483	1.805135490	1.694435138	-3.357573755
S484	1.842651319	-3.593716842	-3.046700192
S485	3.693875406	1.810467664	-0.342046427
S486	0.214523995	-0.896607118	-1.797412197
S487	3.606297665	-1.155581037	-5.457586914
S488	3.825414078	-3.701514545	0.136230004
S489	5.346494969	1.415058911	-3.473236573
S490	5.542996407	-3.341017944	-3.076987428
S491	6.329236280	-0.876880940	-0.507156655
end			

TS

Fe(139) -2.286<br>Fe(140) 0.118<br>Fe(141) -2.599	bm523b2toNti_1_53321.40			
	C1	-7.717992377	-5.294921259	2.197975314
	C2	-8.215909943	-6.519534570	1.419855661
	C3	-8.369081967	-3.182549930	3.273636608
	C4	-7.680734937	-3.287036144	4.631263279

Fe(142) 2.506<br>Fe(143) 2.497<br>Fe(144) 2.521<br>Fe(145) 0.018	C5 -7.188364615 -4.646907418 6.597313809<br>C6 -5.664834051 -4.732855911 6.461594434<br>C7 -3.769887644 -5.201540008 4.995767954<br>C8 -3.265494194 -3.765739816 4.846674715<br>C9 -3.708779309 -1.534904606 3.912536873<br>C10 -3.547815014 -0.765915893 5.231066217<br>C11 -4.359193053 -0.290253509 7.453163761<br>C12 -3.222430498 -0.777513648 8.362932046<br>C13 -2.253917333 -2.691919892 9.544252725<br>C14 -0.815348819 -3.022499179 9.121795816<br>C15 0.647511406 -3.883420017 7.340330857<br>C16 1.328167034 -2.895572622 6.388755404<br>C17 0.989339441 -1.008703040 4.818253956<br>C18 1.426624835 0.286257045 5.530451326<br>C19 -0.029333158 -0.773085375 3.694444084<br>C20 0.478717295 0.337776137 2.769931786<br>C21 -0.314505146 -2.098620447 2.979635001<br>C22 0.639793383 2.214437465 6.876314340<br>C23 1.070748019 1.968064283 8.334811130<br>C24 -0.620676059 3.106947236 6.764681094<br>C25 -0.507152846 4.361586617 7.636989800<br>C26 -0.881538139 3.483329644 5.300058685<br>C27 0.594357419 0.907838256 10.469300196<br>C28 2.043515251 0.589888109 10.768389299<br>C29 6.227124547 -11.133733018 4.745043666<br>C30 6.731223168 -9.922924337 3.963801687<br>C31 6.982748267 -7.450623454 4.117922254<br>C32 8.226586568 -6.977842518 4.894088421<br>C33 5.828849828 -6.446214548 4.258536020<br>C34 6.212386492 -5.061698921 3.743182697<br>C35 5.010669296 -4.130989043 3.642951101<br>C36 5.124002911 -1.630375962 3.447823081<br>C37 10.502178889 -6.095798523 4.697647150<br>C38 10.757212159 -4.615779302 4.527523702<br>C39 -5.450006874 -2.890155687 -1.020037786<br>C40 -5.833011527 -1.437740297 -0.687025018<br>C41 -3.908225125 -2.992552975 -1.018729776<br>C42 -3.329078728 -4.422152880 -1.021115193<br>C43 -3.134561637 -5.061894484 -2.394838644<br>C44 -6.382870608 0.712771393 -1.742524747<br>C45 -7.511333221 1.223684921 -0.844521228<br>C46 -6.554513447 1.272375032 -3.170420728<br>C47 -9.553475480 0.731484193 0.397234033<br>C48 -9.082468981 0.966754915 1.837255232<br>C49 -7.271995310 0.698591912 3.474266619<br>C50 -6.079259048 1.651248234 3.519832033<br>C51 -4.618176945 3.215121227 2.330960825<br>C52 -5.054864571 4.582488167 2.914214886<br>C53 -4.111257245 3.493451729 0.909684104<br>C54 -3.435890288 2.374320603 0.179436263<br>C55 -2.836264809 1.194202459 0.547217141<br>C56 -2.570802444 1.417071089 -1.644240130<br>C57 -6.895810559 6.225905296 2.991772907<br>C58 -6.770035853 6.499709063 4.489346634<br>C59 -8.378939917 6.233993639 2.556858356<br>C60 -9.009913854 7.580297913 2.350574167<br>C61 -8.886289564 8.760050512 3.059165000<br>C62 -10.299358759 9.017092143 1.371919674<br>C63 9.263515353 1.939142137 3.034175069<br>C64 10.270196525 0.809300471 2.917499047<br>C65 7.856165910 1.303376562 2.942880129<br>C66 6.743167170 2.312901705 3.103694854<br>C67 6.211667134 2.617619613 4.371379273<br>C68 6.203182706 2.978940711 1.987981955<br>C69 5.175955769 3.545282994 4.524749021<br>C70 5.182195164 3.918521211 2.127620112<br>C71 4.659262232 4.191895241 3.394891050<br>C72 5.181002118 6.689617531 -2.183752259<br>C73 4.113699128 7.762780459 -2.460495253<br>C74 4.530040821 5.423349751 -1.629812138<br>C75 3.031624451 9.174835221 -4.175798858<br>C76 1.553362309 8.779025453 -4.218764321<br>C77 -0.044319670 6.888669990 -3.919432063<br>C78 -0.458011105 6.701805031 -2.435709414<br>C79 -0.031558637 5.553780063 -4.699118771<br>C80 -1.284085696 4.672636242 -4.686868638<br>C81 -2.453621353 5.121440127 -5.578940298<br>C82 -3.995227960 7.054872636 -5.223092972<br>C83 0.158574562 6.015145425 -0.164464219<br>C84 0.029483566 7.337172977 0.604200156

C85	1.199106666	5.145282603	0.573429212
C86	1.150803961	9.465419622	1.052810386
C87	0.449033042	10.746670144	0.634895733
C88	-1.041058277	11.807131925	-0.965525338
C89	-2.280290240	12.427614609	-0.273704288
C90	-1.283129411	11.655036067	-2.469409853
C91	-2.435117547	10.723347748	-2.819002499
C92	-4.164014781	12.085135805	1.255569679
C93	-4.030907073	13.283096526	2.185183283
C94	-4.787993615	10.886402303	2.024562361
C95	-5.068722214	9.706317211	1.120946253
C96	-6.287852624	9.610355448	0.432788935
C97	-4.104478978	8.710172191	0.897533027
C98	-6.528676507	8.574529633	-0.472700127
C99	-4.314899878	7.682402409	-0.026579118
C100	-5.529843791	7.629138595	-0.704276848
C101	3.693333592	-4.692029384	-8.692869969
C102	2.280655894	-4.427632514	-8.204109849
C103	0.845507526	-2.808639084	-6.979446794
C104	0.309841343	-1.527420285	-7.630100129
C105	0.664140120	0.889639628	-7.873855166
C106	1.811262415	1.867971214	-8.108671571
C107	4.106136054	2.566155406	-7.685082852
C108	4.716853306	2.584911756	-9.074497767
C109	-3.147224960	-4.364075066	-8.575620875
C110	-4.165419372	-3.251265787	-8.300088652
C111	-2.598609679	-4.916186148	-7.241216418
C112	-1.889070573	-6.267871966	-7.446525976
C113	-1.112735199	-6.656804377	-6.209266236
C114	-4.555735541	-0.878388133	-7.862751838
C115	-4.103176137	0.334027724	-8.651667073
C116	-4.901756113	-0.596388468	-6.379177274
C117	-3.636803728	-0.492486915	-5.579682067
C118	-2.910037859	0.707559646	-5.541762865
C119	-3.055951483	-1.660211868	-5.052106563
C120	-1.589455420	0.714500991	-5.079145363
C121	-1.741211549	-1.648752501	-4.587144805
C122	-0.990506793	-0.469692921	-4.642903672
C123	7.707661369	-6.608843616	-4.497699670
C124	8.935234232	-6.612564893	-3.571896328
C125	6.430201856	-6.999456493	-3.736930544
C126	5.140761620	-6.805427581	-4.470272479
C127	4.700576850	-7.483634999	-5.587338531
C128	3.063008825	-6.197271601	-4.834855885
C129	10.231274820	-7.676854023	-1.820173226
C130	11.550146263	-7.509924878	-2.541812652
C131	0.657531538	-6.844242465	0.396269290
C132	2.157470123	-6.944608919	0.655500065
C133	3.036233969	-7.206319129	-0.604929048
C134	2.939773858	-8.698728686	-1.013666060
C135	3.919563905	-9.142174126	-2.102237288
C136	3.925290805	-10.666209066	-2.357072272
C137	4.502072253	-6.891151713	-0.263393501
C138	3.421267591	-0.919538095	-2.170148944
Fe139	3.598600251	-2.294602645	-3.570502360
Fe140	1.926438673	0.570825128	-1.337368911
Fe141	4.852539038	0.356474340	-1.568105665
Fe142	3.466994866	0.213152791	-3.745444493
Fe143	3.683524643	2.514984897	-2.440193632
Fe144	4.763657099	-2.188144192	-1.334931130
Fe145	1.974978222	-2.190540562	-1.237157938
H146	-6.456797634	-7.549160054	1.330374976
H147	-6.775649923	-6.688444625	-0.019759008
H148	-9.653278966	-4.607215638	2.339388920
H149	-9.006926571	-6.198000441	0.723672810
H150	-8.708406816	-7.180658457	2.153938943
H151	-7.698000638	-2.595023442	2.629926198
H152	-8.465363990	-5.153160471	4.937564120
H153	-9.298668907	-2.613077021	3.405488136
H154	-7.559410365	-5.580790858	7.039771993
H155	-5.839338866	-5.452798431	4.536339622
H156	-7.394871461	-3.826900414	7.298067655
H157	-2.769463629	-1.414716755	3.361886501
H158	-4.854516418	-3.321252721	3.623710385
H159	-3.594785674	-5.750723190	4.060891030
H160	-4.180497055	0.774068931	7.254897792
H161	-5.276507734	-1.594640125	5.991856119
H162	-3.182806640	-5.673039320	5.792919351
H163	-4.509940583	-1.079342259	3.314614566
H164	-3.793189837	-2.737702590	8.052742895

	H165	-5.310110663	-0.397350456	7.992265222
	H166	-0.808045682	-2.814072110	3.648265460
	H167	-0.977075667	-1.941229403	2.112747777
	H168	0.612038776	-2.558742559	2.614071053
	H169	-0.965766434	-0.420475778	4.153435287
	H170	-2.713955152	-3.620086291	9.914334767
	H171	-1.012248449	2.598051854	4.662470076
	H172	-0.542961487	0.695466654	8.691257299
	H173	-0.381595535	4.117822447	8.701739484
	H174	-0.174953829	0.443008346	1.893535299
	H175	1.492514638	0.137300773	2.400288557
	H176	9.373950825	2.377627455	4.044710461
	H177	-1.793749992	4.091659401	5.218469331
	H178	-1.417433082	4.969335730	7.533008555
	H179	-0.040747464	4.074276168	4.900972042
	H180	0.351362463	4.978553723	7.326979467
	H181	-1.478195804	2.511774055	7.129834424
	H182	1.495041769	2.723063291	6.408670500
	H183	-0.530846759	0.619745076	6.059874829
	H184	0.496778610	1.311149638	3.281809046
	H185	1.494749845	4.312694347	-0.081655129
	H186	0.700367577	4.729925119	1.460943782
	H187	2.650967726	5.590791203	1.827525977
	H188	3.338404925	5.209793868	4.391296299
	H189	4.758006981	4.410337466	1.252867402
	H190	6.569562164	2.742549659	0.986762524
	H191	6.602908035	2.112559248	5.258087296
	H192	-0.483969560	-2.041006801	5.974503377
	H193	0.557780244	-4.840415356	6.803000464
	H194	1.325460006	-4.035289848	8.188938693
	H195	-1.461481761	-3.587088398	7.231990136
	H196	-2.171348698	-1.989855944	10.383458604
	H197	4.758065633	3.744586495	5.515394768
	H198	1.908802170	-1.421408675	4.379506181
	H199	0.292975167	1.784001038	11.066925337
	H200	0.000237386	0.045073813	10.810274025
	H201	2.590204433	0.044748192	9.964987805
	H202	11.354766467	-6.663180308	4.293591616
	H203	-1.912354397	-6.351244944	-3.410384754
	H204	1.250587326	-6.226804752	-1.332371973
	H205	-2.640789724	-7.046532298	-7.637582073
	H206	-1.215872827	-6.216609467	-8.315295536
	H207	-3.420514292	-5.051610320	-6.520772853
	H208	-3.497750766	0.092476419	-9.557377855
	H209	-3.754862178	-5.157788838	-9.036795792
	H210	-3.515988607	-2.423740763	-1.877532796
	H211	-3.982259264	-5.096156966	-0.440249836
	H212	-2.653421105	-1.836352250	-8.136173924
	H213	-5.507638804	-1.166019229	-8.356963110
	H214	-1.459180973	-3.310515382	-9.180135261
	H215	-1.373847189	-6.002442731	-1.774155251
	H216	-1.915652357	-4.173862081	-6.799663105
	H217	-1.651971309	-4.767521250	-9.923986939
	H218	-6.913864245	-3.764183727	-2.184972460
	H219	-3.552070404	-2.489797639	-0.107359616
	H220	0.504330923	-5.932944025	-7.063573875
	H221	-2.363850496	-4.415651416	-0.493357800
	H222	-3.802910998	2.861387059	2.977275162
	H223	-2.736176857	0.731392602	1.521144927
	H224	-8.049752013	1.134260244	4.117365517
	H225	-7.303681468	-0.050285345	1.474424063
	H226	-6.974255712	4.205952994	2.288029878
	H227	-8.289862098	9.037278839	3.921403275
	H228	-6.910525959	5.593604145	5.128068921
	H229	-8.966223449	5.629263919	3.271234372
	H230	-6.329643785	6.997789287	2.446678806
	H231	-11.006442729	9.504791844	0.705362887
	H232	-3.400917223	4.333648426	0.986416274
	H233	-4.955766755	3.852364718	0.298864305
	H234	-9.848132125	10.633213888	2.682105151
	H235	-8.440824715	5.711666208	1.588643296
	H236	-6.219003720	2.045531015	1.488214662
	H237	-6.966473011	-0.250941371	3.940742241
	H238	-10.040972454	1.661742467	0.074720765
	H239	-2.260496267	1.225895710	-2.669033344
	H240	-10.308134427	-0.064905144	0.400746459
	H241	10.318596493	3.443536840	2.130122961
	H242	8.704776657	3.549467836	1.901651058
	H243	1.364726206	5.808375598	-1.940607733
	H244	-0.818811893	5.516175453	-0.093632617

H245	1.626962172	8.067475146	-0.457383324
H246	-3.643942009	3.263873741	-1.774271627
H247	-5.333783068	3.855325164	-2.912984287
H248	-7.279363136	2.974731277	-2.511037959
H249	-4.976163600	5.167716646	-2.084656308
H250	-5.684692861	0.987883076	-3.777875306
H251	-7.460166419	0.844915655	-3.631400399
H252	-0.349626763	11.352383111	-2.963810151
H253	-1.552124061	12.652085468	-2.851204500
H254	-1.223538091	9.718411697	-4.161165908
H255	-2.884599120	9.149528896	-3.994144686
H256	-4.055934701	8.548298150	-6.630646051
H257	-2.949205230	4.241293334	-6.015730742
H258	-7.489637618	8.507349968	-0.990055296
H259	-3.159137911	8.717780313	1.447281098
H260	-4.972480753	7.354823515	-3.430376022
H261	0.798794361	4.948011914	-4.300905117
H262	2.086014163	6.838418974	-3.915323107
H263	-1.641332519	4.506429621	-3.658216609
H264	-8.446357452	-0.586460504	-0.896246651
H265	3.316293181	9.523435870	-5.176700328
H266	-6.275936281	-1.305276644	-2.629842298
H267	0.237351076	5.786968652	-5.742697417
H268	-3.524664798	6.959238682	-0.232758018
H269	3.101263627	10.030314933	-3.488149796
H270	-3.299292878	7.082185462	-7.165769285
H271	-5.436031526	8.419427553	-4.716372409
H272	-5.368339712	-3.912551677	-2.804406837
H273	-5.825742721	-3.497273534	-0.174787395
H274	-7.068381340	10.355693255	0.603569380
H275	-5.472404527	1.150488954	-1.305602900
H276	-0.766441343	7.589585569	-4.348640855
H277	-2.068936643	5.717057676	-6.416948992
H278	-0.962616115	3.690253844	-5.057743146
H279	-3.860658918	5.430176546	-4.010834193
H280	0.846511622	9.239530478	2.085683107
H281	-0.590405148	9.707019266	-0.789978194
H282	-0.273674356	12.587243570	-0.844093606
H283	2.229530641	9.674789509	1.054888555
H284	-2.564133482	10.734555638	0.808011557
H285	4.182189900	-3.742320745	-8.956356069
H286	4.242278379	-5.102694442	-7.820157151
H287	7.615894733	-5.569650551	-4.846193062
H288	4.614041773	-5.929835366	-10.027048993
H289	3.088749194	-6.387647231	-9.643698194
H290	5.328351972	4.697110376	-1.428678969
H291	5.691782981	7.524205620	-0.389845676
H292	4.548744530	7.700378695	-4.470522558
H293	3.996747282	5.650651940	-0.696357677
H294	6.694375090	7.957115012	-1.610369835
H295	4.900541474	2.286424517	-6.975446272
H296	5.704999205	6.439925132	-3.119332484
H297	5.519921462	-6.397721582	5.316017542
H298	7.227801737	-7.570507242	3.055435584
H299	3.193074637	0.770385983	-6.990295549
H300	5.272456274	0.357495338	2.984170756
H301	0.168686763	0.726126004	-8.838941010
H302	-1.289829837	-2.556963832	-4.181047369
H303	-0.067756289	1.410426734	-7.232653425
H304	4.575972844	-3.945412296	4.632325746
H305	1.685563374	-0.490828268	-6.549042009
H306	4.222181656	-4.592032654	3.025060696
H307	4.979209819	-6.841176954	3.678622455
H308	6.659246413	-5.154749302	2.741800415
H309	6.968707475	-4.596807563	4.395169911
H310	-5.531395681	-1.423804177	-6.021098884
H311	6.203638201	-8.846794060	5.564639579
H312	5.297416763	-11.452095909	4.249153301
H313	0.041385157	-0.468694128	-4.300086373
H314	6.775043525	-10.988310727	6.726228040
H315	5.224323235	-11.470808397	6.511487720
H316	5.769621983	-2.952977513	2.064657085
H317	-1.009000673	1.639061862	-5.069985576
H318	-3.631179500	-2.589150514	-5.033197176
H319	7.603924382	-10.038274700	-1.003340936
H320	6.494467975	-8.065128588	-3.457510873
H321	6.675868646	-8.143817151	0.434609804
H322	6.699636601	-10.771877149	-2.040886795
H323	4.477061018	1.696187323	-9.707441470
H324	6.460284434	-5.079923674	-0.110056411

H325	2.334142023	-7.714590670	1.415898851
H326	2.901454111	-2.630766987	-7.453862087
H327	3.125055589	-9.288084287	-0.101900354
H328	2.762124603	-7.454246671	-6.497686297
H329	2.076671117	-5.760611101	-4.738931996
H330	6.962283907	-11.941244426	4.587846562
H331	5.670889558	-0.717174730	1.665253647
H332	8.474921604	-5.679633098	0.950445417
H333	7.356266322	-3.821918349	-0.266599043
H334	9.152642836	-6.528108938	3.080320829
H335	4.275475257	-2.165130696	5.239857937
H336	3.656865854	-8.648938521	-3.051183627
H337	4.942146441	-8.833358845	-1.845310221
H338	1.906033621	-8.909514771	-1.328915720
H339	1.014304210	-2.658592657	-5.901660529
H340	-3.365360300	1.620995733	-5.932648534
H341	-5.493840553	0.330423632	-6.352946963
H342	2.448016898	-5.976885560	1.097753569
H343	8.592929370	-7.230552567	0.950029922
H344	7.494783749	-9.134759529	1.290423435
H345	4.151215694	-0.519973771	4.870383679
H346	5.193818136	-8.221722110	-6.209145538
H347	6.388054630	-6.441219122	-2.794417995
H348	0.106582569	-3.599580647	-7.124234762
H349	3.773368102	3.583404766	-7.430834042
H350	7.786073208	0.535074678	3.726850682
H351	7.771792181	0.798609818	1.966903448
H352	8.743495178	-7.135679000	-6.188186783
H353	8.107915267	-8.419203166	-5.396367257
H354	10.251723535	-8.659115057	-1.322306445
H355	8.419318359	-8.429803839	-2.652299516
H356	11.551281191	-7.845987616	-3.606819851
H357	10.140620141	-6.911307625	-1.033689845
H358	10.501331028	0.509236016	1.866546852
H359	-3.010014556	13.447516407	2.603186770
H360	10.454683014	-6.289749502	5.783991136
H361	9.844120156	-3.987823249	4.383013631
H362	-4.840781061	12.378434631	0.435657104
H363	-4.109019237	10.591055157	2.840121370
H364	-5.719976824	11.244385312	2.485477121
H365	0.930387491	-3.255521161	-1.024368802
H366	-6.668934613	6.569735164	-1.877992747
H367	0.520357324	-1.617773237	0.525277358
H368	3.212584476	-0.841459960	0.499551802
H369	2.415363836	-2.934991453	0.636827788
H370	-1.599416248	-0.157033562	-0.689578972
Mo371	3.800232433	-4.669662134	-2.183536925
N372	2.248300460	-0.788588230	0.142460050
N373	1.527646765	-1.817015492	0.633555195
N374	-7.210970887	-7.276559615	0.695135873
N375	-8.696455038	-4.455012119	2.649138816
N376	-7.873698881	-4.426824882	5.334262285
N377	-5.191746415	-5.256668270	5.301240781
N378	-4.026423094	-2.946209168	4.080715374
N379	-4.459483154	-1.016213135	6.197575197
N380	-3.131523996	-2.122250269	8.532745634
N381	-0.652308765	-3.485319215	7.851432078
N382	0.517181336	-1.986600843	5.793380285
N383	0.427284883	0.961402442	6.154977238
N384	0.330036632	1.085857857	9.052708370
N385	5.940703739	-10.836782034	6.155119581
N386	6.578754737	-8.753760478	4.614668167
N387	5.401337838	-2.865544118	3.014385541
N388	4.667967370	-1.392946215	4.698419741
N389	5.273274746	-0.588708344	2.610407728
N390	9.250351306	-6.539724035	4.119491788
N391	-6.024756136	-3.276745370	-2.313636797
N392	-2.067288888	-5.872326958	-2.517741258
N393	-6.254026651	-0.729407684	-1.770930239
N394	-8.501187452	0.363258806	-0.534984883
N395	-7.855484501	0.470359369	2.165976381
N396	-5.690755911	2.228789079	2.341556824
N397	-3.256379299	2.478688283	-1.194219046
N398	-2.319679060	0.619192029	-0.600682645
N399	-6.357012642	4.902487301	2.697847057
N400	-9.897086984	7.754148041	1.300331001
N401	-9.710416698	9.660798298	2.419150757
N402	9.483143530	2.885427250	1.937742654
N403	6.181269668	7.164184743	-1.215476013
N404	3.955402128	8.130588195	-3.765613570

N405	1.288328874	7.467151181	-4.046419594
N406	-3.463099580	5.892845396	-4.849243203
N407	-3.722701356	7.614652436	-6.414511367
N408	-4.767902040	7.745172053	-4.353342031
N409	0.482786168	6.207671497	-1.580891568
N410	0.889061231	8.316675617	0.206925305
N411	-0.507081099	10.615862261	-0.335185816
N412	-2.192875029	9.885187566	-3.863981313
N413	-2.867620477	11.700360862	0.709901155
N414	3.670019418	-5.573382298	-9.862473043
N415	2.090159812	-3.233501994	-7.606315233
N416	1.000290358	-0.408552619	-7.317068456
N417	3.022638637	1.607540219	-7.553403770
N418	-2.157618144	-3.949612606	-9.577494768
N419	-3.664072686	-1.998510433	-8.098989817
N420	7.909837739	-7.456480661	-5.687008653
N421	4.098886159	-5.992608150	-4.019325306
N422	3.398989944	-7.084552786	-5.797003764
N423	9.099341259	-7.669609323	-2.731423847
N424	-0.007564261	2.421388168	-0.164197257
N425	0.744054598	1.695669649	-0.589691324
O426	-6.517561789	-5.089315350	2.449266769
O427	-6.975471020	-2.350816944	5.048835646
O428	-4.924749366	-4.349902140	7.381943998
O429	-2.226380488	-3.376589909	5.405051860
O430	-2.623162817	0.048595684	5.387343910
O431	-2.478943009	0.030420166	8.946857924
O432	0.109144987	-2.906747988	9.937518522
O433	2.549970677	-2.985610511	6.175407147
O434	2.609818835	0.671729663	5.522944022
O435	2.046964699	2.557443231	8.827273068
O436	2.566526441	0.839165302	11.845041921
O437	7.206109561	-10.069228000	2.818808198
O438	8.248975863	-7.001619052	6.137487279
O439	11.871480397	-4.116705302	4.562991836
O440	-5.755038561	-0.951197728	0.458385339
O441	-3.934331056	-4.872632560	-3.340565300
O442	-7.485451424	2.405166975	-0.435668249
O443	-6.624360139	2.707386932	-3.189431418
O444	-9.816033726	1.576023017	2.625726149
O445	-5.510054437	1.882816930	4.590843272
O446	-4.246130571	5.344545759	3.449960763
O447	-6.576217498	7.600529685	4.973701769
O448	10.782828851	0.242565042	3.871314461
O449	3.617384164	5.096912788	3.464182968
O450	3.438916518	8.273818753	-1.550073076
O451	0.689747483	9.654681399	-4.417132948
O452	-1.604810360	7.002403216	-2.068470365
O453	-0.759925667	7.456588493	1.550237618
O454	2.350911872	5.904984299	0.950638284
O455	0.734518370	11.829163514	1.161636847
O456	-2.665825684	13.562899624	-0.588447414
O457	-3.523105963	10.766600097	-2.227097248
O458	-4.971643558	14.000037223	2.487149486
O459	-5.715757345	6.615942633	-1.668801280
O460	1.383638547	-5.296902590	-8.324435680
O461	-0.662547127	-1.539310537	-8.411480855
O462	1.593938043	2.898003072	-8.767032526
O463	5.442096346	3.480339642	-9.473818135
O464	-5.382924330	-3.489446489	-8.239962766
O465	-1.625655849	-7.179630655	-5.225939330
O466	0.201368366	-6.350126837	-6.199412353
O467	-4.404736819	1.487421888	-8.382077976
O468	9.787099292	-5.703740368	-3.622647828
O469	12.570487859	-7.106389097	-2.001000636
O470	0.316258666	-6.346338862	-0.797974675
O471	-0.183836230	-7.138237506	1.243930600
O472	5.069058499	-11.235402868	-2.461569934
O473	2.807246246	-11.242536097	-2.474408555
O474	5.086904670	-5.978549784	-0.998520476
O475	5.077601750	-7.508218512	0.651044577
O476	2.597569483	-6.337166504	-1.649476095
O477	9.051658724	-6.410951634	1.284004070
O478	7.137823984	-4.533663308	0.363269215
O479	-4.582809526	4.450399934	-2.640271332
O480	7.661290901	-10.605834717	-1.803733750
O481	7.561915425	-8.595323988	0.462235229
S482	3.314567184	4.696143401	-2.823125179
S483	1.811523018	1.682803532	-3.356016623
S484	1.855237013	-3.598179268	-3.073574497

	S485	3.688123179	1.815556603	-0.347839176
	S486	0.208288800	-0.905301571	-1.783364670
	S487	3.620594544	-1.152066672	-5.463920804
	S488	3.667996827	-3.624502861	0.094950861
	S489	5.352998061	1.416226930	-3.474103776
	S490	5.543247938	-3.340860523	-3.084274794
	S491	6.326226606	-0.871466872	-0.497901467
	end			

product

Fe(139) -2.318	bm523b2toNti.car_4			
Fe(140) 0.136	C1	-7.715479512	-5.289102137	2.204108550
Fe(141) -2.599	C2	-8.204322185	-6.515742787	1.423211413
Fe(142) 2.516	C3	-8.383836493	-3.182649460	3.280500732
Fe(143) 2.535	C4	-7.697561341	-3.287779418	4.639217862
Fe(144) 2.463	C5	-7.205817414	-4.649667777	6.604450026
Fe(145) -0.021	C6	-5.682237085	-4.737236918	6.471157398
	C7	-3.786016688	-5.210298530	5.007080869
	C8	-3.270896147	-3.778568585	4.854731174
	C9	-3.719428493	-1.543327442	3.926855563
	C10	-3.553500920	-0.775365413	5.246089114
	C11	-4.372980352	-0.301430610	7.468097586
	C12	-3.233739851	-0.789907880	8.373995809
	C13	-2.258361014	-2.702892129	9.549361423
	C14	-0.816543299	-3.011322153	9.121664965
	C15	0.648053834	-3.877760984	7.344258631
	C16	1.326720160	-2.896341595	6.384944530
	C17	0.984415658	-1.006770182	4.814911428
	C18	1.415849343	0.289507392	5.530586952
	C19	-0.035159525	-0.776220043	3.691992770
	C20	0.463629918	0.332936374	2.760898545
	C21	-0.318620573	-2.108171048	2.989871024
	C22	0.630050050	2.218194667	6.875893525
	C23	1.060676691	1.970183192	8.334744065
	C24	-0.627758906	3.115003716	6.767384072
	C25	-0.506921550	4.368550300	7.640684066
	C26	-0.893256180	3.497741408	5.305240945
	C27	0.592849913	0.902920004	10.468335983
	C28	2.043792411	0.581033976	10.756023668
	C29	6.219155364	-11.139254929	4.738870320
	C30	6.725440266	-9.927912719	3.959059991
	C31	6.992955798	-7.457371773	4.115221280
	C32	8.238918715	-6.990195383	4.891337545
	C33	5.844077227	-6.445521035	4.254693639
	C34	6.231090636	-5.060455524	3.740410429
	C35	5.028194309	-4.130244294	3.636503571
	C36	5.114813562	-1.628826577	3.431849897
	C37	10.508080514	-6.090837578	4.695594199
	C38	10.755157463	-4.609848796	4.520133155
	C39	-5.448547251	-2.890689593	-1.025228102
	C40	-5.832981100	-1.438569452	-0.693138333
	C41	-3.906388834	-2.991451461	-1.024568354
	C42	-3.326141693	-4.420795595	-1.026801372
	C43	-3.129765571	-5.060376998	-2.400440236
	C44	-6.389245620	0.709330310	-1.747550994
	C45	-7.521749930	1.217937196	-0.853875084
	C46	-6.556983995	1.277311639	-3.172609395
	C47	-9.557142550	0.721286425	0.396328638
	C48	-9.085284244	0.959664270	1.835522816
	C49	-7.272460596	0.699918479	3.471188895
	C50	-6.077294791	1.649409657	3.516392992
	C51	-4.617425418	3.214459134	2.325682505
	C52	-5.050312542	4.583731586	2.908868214
	C53	-4.111530165	3.490217385	0.903120109
	C54	-3.434748786	2.371718100	0.172660660
	C55	-2.831434426	1.193487316	0.540845613
	C56	-2.562249597	1.418034327	-1.648995237
	C57	-6.891270243	6.227994360	2.994289193
	C58	-6.764035615	6.505531953	4.490936682
	C59	-8.374662565	6.236510593	2.560039633
	C60	-9.005843014	7.582915312	2.354704949
	C61	-8.881544864	8.762504563	3.063452415
	C62	-10.296419208	9.019920010	1.377816682
	C63	9.269669811	1.944153017	3.025762458
	C64	10.275146189	0.813637612	2.905338689
	C65	7.861936606	1.308258299	2.934416373
	C66	6.748522753	2.316810897	3.098213984
	C67	6.213894331	2.615310929	4.366020681
	C68	6.211529259	2.988434524	1.984415323
	C69	5.177935853	3.542533662	4.521230611

	C70	5.190357681	3.927489985	2.125954523
	C71	4.664236845	4.194656321	3.393078224
	C72	5.181575051	6.690497068	-2.188136210
	C73	4.114363830	7.764575072	-2.462472790
	C74	4.530672562	5.426340556	-1.631390125
	C75	3.029683800	9.176104630	-4.177063804
	C76	1.551426965	8.779604281	-4.221231703
	C77	-0.045709503	6.888664635	-3.917031496
	C78	-0.459671817	6.696237755	-2.434044945
	C79	-0.031362314	5.556880891	-4.702031708
	C80	-1.281996789	4.673172820	-4.694125671
	C81	-2.451169761	5.121755868	-5.586384199
	C82	-3.989840879	7.056567695	-5.230157916
	C83	0.161161813	6.012351392	-0.162838996
	C84	0.029414440	7.333273959	0.607285534
	C85	1.205479867	5.145497430	0.573434785
	C86	1.148747016	9.461974064	1.059131507
	C87	0.449391240	10.744686037	0.641921801
	C88	-1.038526081	11.807447747	-0.960476697
	C89	-2.280068104	12.427130163	-0.272279027
	C90	-1.274509438	11.653697801	-2.465140994
	C91	-2.429060879	10.726571075	-2.819322638
	C92	-4.164015160	12.084183564	1.256628376
	C93	-4.031925639	13.283177317	2.185137032
	C94	-4.787495715	10.885577103	2.026149296
	C95	-5.067261584	9.705959016	1.121745598
	C96	-6.283888379	9.612903209	0.428806791
	C97	-4.103528670	8.708845926	0.900515120
	C98	-6.522924730	8.578416683	-0.478666792
	C99	-4.312253459	7.682315535	-0.025411854
	C100	-5.524943295	7.631393258	-0.707283057
	C101	3.694882203	-4.692510344	-8.693279461
	C102	2.282384404	-4.428203815	-8.203140877
	C103	0.845966823	-2.807601964	-6.981814122
	C104	0.310286394	-1.527735270	-7.635685943
	C105	0.666379076	0.888590899	-7.885721317
	C106	1.814978152	1.866576741	-8.116890931
	C107	4.110457341	2.560621380	-7.689680470
	C108	4.706456581	2.594026085	-9.084842768
	C109	-3.146435839	-4.364318665	-8.575217477
	C110	-4.162857558	-3.250122619	-8.299239185
	C111	-2.597650126	-4.917273427	-7.241313520
	C112	-1.890582665	-6.270001626	-7.447552615
	C113	-1.115054698	-6.660398731	-6.210301997
	C114	-4.551799441	-0.876537690	-7.866852541
	C115	-4.099330323	0.334363572	-8.658150851
	C116	-4.896711377	-0.591894427	-6.383540242
	C117	-3.631536041	-0.487497753	-5.584130167
	C118	-2.904760549	0.712617789	-5.546205237
	C119	-3.050782056	-1.654868000	-5.055751548
	C120	-1.585490014	0.720498221	-5.079552400
	C121	-1.737514804	-1.642249628	-4.586552333
	C122	-0.987580777	-0.462640257	-4.638728829
	C123	7.705006655	-6.615460349	-4.503135023
	C124	8.929596150	-6.615434063	-3.573193693
	C125	6.427279528	-7.005900412	-3.746274356
	C126	5.142765205	-6.828781643	-4.491033834
	C127	4.703171559	-7.500831106	-5.611373943
	C128	3.063375696	-6.222961514	-4.846747431
	C129	10.227328309	-7.682097979	-1.823773083
	C130	11.546820870	-7.511579395	-2.543380131
	C131	0.666891594	-6.841425810	0.396784337
	C132	2.166421344	-6.939659650	0.664259870
	C133	3.044016543	-7.208072737	-0.595897504
	C134	2.942951508	-8.701499395	-1.003093157
	C135	3.924349719	-9.140432872	-2.092323091
	C136	3.932806851	-10.661704781	-2.355854986
	C137	4.511933288	-6.902100690	-0.253982456
	C138	3.423009340	-0.918922021	-2.171185019
	Fe139	3.589755927	-2.291000130	-3.589802462
	Fe140	1.936055865	0.571899082	-1.305784618
	Fe141	4.861210651	0.355135656	-1.565926214
	Fe142	3.462271471	0.216355678	-3.740743763
	Fe143	3.685085221	2.520228389	-2.442360553
	Fe144	4.820229871	-2.188921364	-1.398034582
	Fe145	2.027893664	-2.194438341	-1.194138070
	H146	-6.441274367	-7.539131017	1.345932691
	H147	-6.757162808	-6.684625761	-0.009544939
	H148	-9.654262344	-4.611700716	2.335206334
	H149	-8.992296992	-6.197514605	0.721923243

	H150	-8.699721204	-7.176790679	2.155355029
	H151	-7.716409010	-2.587964203	2.639772864
	H152	-8.478498914	-5.156066337	4.941503848
	H153	-9.317415835	-2.619775447	3.411556557
	H154	-7.577311089	-5.584139610	7.045291532
	H155	-5.855712492	-5.444679920	4.540483049
	H156	-7.413979537	-3.830490145	7.305594538
	H157	-2.784653983	-1.419552903	3.370064074
	H158	-4.871746418	-3.326803430	3.651419459
	H159	-3.612154739	-5.761091832	4.072667051
	H160	-4.198813724	0.764534419	7.275265462
	H161	-5.288519110	-1.595882511	5.998334138
	H162	-3.203401642	-5.685380011	5.805408421
	H163	-4.524119076	-1.086987071	3.334280270
	H164	-3.810326055	-2.750761818	8.070944204
	H165	-5.324241223	-0.416459848	8.004904591
	H166	-0.821806012	-2.816573548	3.657495326
	H167	-0.967205762	-1.965862825	2.110091243
	H168	0.609719218	-2.595366548	2.668894545
	H169	-0.973887504	-0.427821108	4.149398659
	H170	-2.707295174	-3.640427154	9.909111649
	H171	-1.034785391	2.616953801	4.663741389
	H172	-0.550042246	0.693482862	8.692316858
	H173	-0.375411293	4.124264583	8.704516708
	H174	-0.196520521	0.436862354	1.888861571
	H175	1.477872702	0.143593565	2.383536435
	H176	9.381105974	2.380080627	4.037301102
	H177	-1.801656917	4.113031429	5.232508333
	H178	-1.416742372	4.977807048	7.542235759
	H179	-0.051609146	4.085966630	4.904120499
	H180	0.350582274	4.984277717	7.325584666
	H181	-1.486061389	2.522273174	7.134459925
	H182	1.486848857	2.725246073	6.409514222
	H183	-0.543310069	0.625204614	6.057034162
	H184	0.481092055	1.307535652	3.270046865
	H185	1.503692923	4.314706848	-0.082660551
	H186	0.708693154	4.728012443	1.461107056
	H187	2.656543138	5.594292402	1.827002741
	H188	3.342098430	5.208870803	4.391595440
	H189	4.768525716	4.423365388	1.252403700
	H190	6.579758998	2.756364652	0.982854867
	H191	6.603409117	2.106323117	5.251238144
	H192	-0.486114624	-2.042319093	5.973955001
	H193	0.561874719	-4.840192551	6.815784061
	H194	1.326867154	-4.020700721	8.193893635
	H195	-1.463109716	-3.598863329	7.238801093
	H196	-2.184987414	-2.009130227	10.396297974
	H197	4.757731196	3.737720151	5.511786708
	H198	1.907762059	-1.413944117	4.378955535
	H199	0.296697707	1.777711615	11.070396400
	H200	-0.000945753	0.039859528	10.808694303
	H201	2.587037037	0.050281988	9.940546548
	H202	11.364332191	-6.655550804	4.295364529
	H203	-1.908076999	-6.350843184	-3.414553248
	H204	1.299589866	-6.223277624	-1.328631317
	H205	-2.643622739	-7.047323314	-7.638963455
	H206	-1.217103061	-6.219418739	-8.316175372
	H207	-3.419051912	-5.051539994	-6.520068966
	H208	-3.490350438	0.091304840	-9.561084075
	H209	-3.755695690	-5.157238751	-9.035692154
	H210	-3.514843146	-2.422078926	-1.883408764
	H211	-3.979358625	-5.095651173	-0.446899546
	H212	-2.649687110	-1.835718962	-8.139495492
	H213	-5.503893092	-1.164976666	-8.360080874
	H214	-1.457557475	-3.313134777	-9.181178918
	H215	-1.368734861	-6.002271745	-1.778238934
	H216	-1.912926733	-4.176271615	-6.800344667
	H217	-1.652237086	-4.770669201	-9.923731571
	H218	-6.911432215	-3.770345993	-2.186298384
	H219	-3.551148197	-2.488074771	-0.113018701
	H220	0.503439246	-5.934314736	-7.060938169
	H221	-2.361367054	-4.414268031	-0.498171826
	H222	-3.802223550	2.859791814	2.971691006
	H223	-2.730517438	0.730608540	1.514709898
	H224	-8.050228053	1.138904679	4.112043242
	H225	-7.302438957	-0.050120500	1.471292390
	H226	-6.974123658	4.205788673	2.298344660
	H227	-8.284342167	9.039622860	3.925148795
	H228	-6.905571628	5.601810287	5.132776888
	H229	-8.961786596	5.631289698	3.274142766

	H230	-6.324871927	6.997972522	2.446759984
	H231	-11.004151618	9.507978685	0.712168812
	H232	-3.402295369	4.331470286	0.978239001
	H233	-4.956892780	3.847712518	0.292715267
	H234	-9.843440900	10.635822345	2.687782008
	H235	-8.436536100	5.714663050	1.591549250
	H236	-6.220591557	2.045282482	1.486341931
	H237	-6.970816171	-0.248985932	3.941431623
	H238	-10.046131466	1.650200864	0.072414596
	H239	-2.250204209	1.228887899	-2.673593400
	H240	-10.310359750	-0.076353749	0.401702353
	H241	10.325125446	3.449826447	2.124235497
	H242	8.711222158	3.558035959	1.897658306
	H243	1.367129734	5.810678104	-1.939559063
	H244	-0.814603747	5.510328048	-0.091385329
	H245	1.624552043	8.067591251	-0.454436369
	H246	-3.640617903	3.264116975	-1.778982996
	H247	-5.331760857	3.859664437	-2.919581972
	H248	-7.253229971	2.971714062	-2.465459370
	H249	-4.972264877	5.171184601	-2.091459637
	H250	-5.686639842	0.996305681	-3.781360303
	H251	-7.462785616	0.854602675	-3.637522264
	H252	-0.339899722	11.345833656	-2.954577220
	H253	-1.537019273	12.650946280	-2.851142018
	H254	-1.225716927	9.734420723	-4.178453836
	H255	-2.889271454	9.171250848	-4.015239377
	H256	-4.076804393	8.538301556	-6.645332576
	H257	-2.947672052	4.241279389	-6.021449612
	H258	-7.482227947	8.513121797	-0.999394522
	H259	-3.160211314	8.714804053	1.453801496
	H260	-4.961474584	7.362165164	-3.435508433
	H261	0.799824367	4.951090016	-4.305762932
	H262	2.084434594	6.840493409	-3.908018568
	H263	-1.640338062	4.502412692	-3.666588898
	H264	-8.443488123	-0.598499708	-0.890093772
	H265	3.314307861	9.525811072	-5.177585348
	H266	-6.281074795	-1.307890568	-2.635698875
	H267	0.237295175	5.795307404	-5.744415545
	H268	-3.522353850	6.958376113	-0.229997215
	H269	3.098584363	10.031024209	-3.488610188
	H270	-3.330923905	7.071114394	-7.184262716
	H271	-5.430164934	8.421815791	-4.723890728
	H272	-5.366830064	-3.913319840	-2.809660749
	H273	-5.822983494	-3.496818443	-0.178928778
	H274	-7.063743564	10.359439969	0.597575755
	H275	-5.480963283	1.146874195	-1.305985084
	H276	-0.768206630	7.591059911	-4.343377483
	H277	-2.066619304	5.716907036	-6.424830832
	H278	-0.958095480	3.692847106	-5.068211580
	H279	-3.856285811	5.432976463	-4.016650795
	H280	0.841674517	9.234634154	2.090897590
	H281	-0.591093765	9.706945275	-0.782757106
	H282	-0.271919594	12.588151609	-0.836265936
	H283	2.227815493	9.670071757	1.063586411
	H284	-2.565050359	10.733844524	0.808421005
	H285	4.181528095	-3.742933108	-8.961496575
	H286	4.245840198	-5.098106687	-7.819559384
	H287	7.611677156	-5.576773216	-4.853358351
	H288	4.618705094	-5.931627793	-10.022995826
	H289	3.096714928	-6.395670950	-9.634802642
	H290	5.327731966	4.697622711	-1.434406013
	H291	5.700288813	7.527277076	-0.397217928
	H292	4.544772840	7.700072863	-4.473265257
	H293	4.002391074	5.654330008	-0.695276735
	H294	6.696610228	7.959833763	-1.622712280
	H295	4.910564694	2.264180974	-6.993532875
	H296	5.701811714	6.439260922	-3.125424397
	H297	5.534446696	-6.396799044	5.312066854
	H298	7.237408644	-7.579577910	3.052983691
	H299	3.191882637	0.769954082	-6.990426963
	H300	5.267361157	0.357560343	2.966782906
	H301	0.176006224	0.723963294	-8.853299513
	H302	-1.286258528	-2.550571861	-4.180678272
	H303	-0.068695782	1.410156116	-7.248735690
	H304	4.593183260	-3.944941131	4.625992446
	H305	1.687385639	-0.489844192	-6.557356276
	H306	4.241209145	-4.593902340	3.018579199
	H307	4.994007055	-6.837265894	3.673271298
	H308	6.680947546	-5.153215623	2.740514835
	H309	6.985200123	-4.595847439	4.395245562

H310	-5.527055224	-1.418192391	-6.024011754
H311	6.212056703	-8.850210183	5.564075851
H312	5.285987681	-11.451673303	4.245449224
H313	0.042036327	-0.460772099	-4.289135383
H314	6.777376196	-11.012062629	6.717272441
H315	5.221870324	-11.479659285	6.506668223
H316	5.812619032	-2.949666387	2.068686733
H317	-1.005239942	1.645204202	-5.070793746
H318	-3.625095407	-2.584443566	-5.039214930
H319	7.612063676	-10.032623429	-1.007225087
H320	6.495370479	-8.066141844	-3.448480311
H321	6.675776118	-8.142954110	0.436911762
H322	6.707648002	-10.766328811	-2.045358714
H323	4.460584864	1.710940620	-9.723237299
H324	6.453144801	-5.070188083	-0.092551493
H325	2.342931442	-7.703333725	1.431400199
H326	2.902651475	-2.630816458	-7.451592139
H327	3.125635058	-9.293892932	-0.092800479
H328	2.761729041	-7.467026978	-6.517454810
H329	2.076459740	-5.787638554	-4.741298809
H330	6.950031163	-11.949012927	4.573965208
H331	5.676165870	-0.716863632	1.652067052
H332	8.473241090	-5.681048914	0.951107170
H333	7.359121087	-3.820530287	-0.252819617
H334	9.159521158	-6.529071695	3.077977469
H335	4.262167894	-2.164351622	5.225760456
H336	3.662374810	-8.640910257	-3.038170748
H337	4.946140412	-8.830997700	-1.832898242
H338	1.909168301	-8.909317871	-1.321251244
H339	1.014118754	-2.654353988	-5.904331942
H340	-3.359357800	1.625511358	-5.939396309
H341	-5.488421935	0.335244846	-6.358684334
H342	2.455692916	-5.966862717	1.096846720
H343	8.593851792	-7.231831571	0.948442109
H344	7.494487872	-9.137744447	1.286492339
H345	4.115830870	-0.520270549	4.843086501
H346	5.196945700	-8.231695593	-6.239252715
H347	6.374655971	-6.432229217	-2.813463753
H348	0.107480176	-3.599185579	-7.125401467
H349	3.788610327	3.576767545	-7.416202086
H350	7.793277230	0.538615894	3.717290948
H351	7.776506665	0.805539978	1.957396726
H352	8.744523424	-7.150181803	-6.189020983
H353	8.100258608	-8.428639429	-5.393676465
H354	10.248075011	-8.665383492	-1.328029413
H355	8.418496235	-8.436779302	-2.661402419
H356	11.548426220	-7.842832233	-3.609869290
H357	10.134596008	-6.918347584	-1.035957379
H358	10.508488060	0.518849948	1.853342907
H359	-3.010875112	13.449546672	2.602035110
H360	10.459686904	-6.280999482	5.782292079
H361	9.838949898	-3.987873286	4.366252366
H362	-4.841030036	12.376513859	0.436522441
H363	-4.108284778	10.590601140	2.841666965
H364	-5.719763866	11.243200178	2.486831460
H365	1.019291112	-3.299975460	-1.113475247
H366	-6.661738174	6.577028384	-1.886909901
H367	0.505372639	-1.609407435	0.666802180
H368	3.215614857	-0.904179691	0.571367771
H369	1.953950124	-2.525822651	1.237378717
H370	-1.580780784	-0.152138323	-0.698399403
Mo371	3.867774068	-4.683905571	-2.219042941
N372	2.273643365	-0.806677646	0.183987630
N373	1.494448971	-1.867933638	0.586645399
N374	-7.193200246	-7.271164594	0.706140455
N375	-8.700106822	-4.454418915	2.650014365
N376	-7.889576350	-4.428673801	5.340590684
N377	-5.208970575	-5.254211699	5.307724495
N378	-4.033533804	-2.955693708	4.093480851
N379	-4.469638235	-1.021605284	6.208666888
N380	-3.143261355	-2.134910302	8.543123052
N381	-0.652634309	-3.479838749	7.852833326
N382	0.513849128	-1.990118346	5.785972671
N383	0.415392191	0.966086171	6.151264809
N384	0.321921368	1.086613730	9.053027412
N385	5.941329314	-10.847924866	6.152019263
N386	6.580468166	-8.758276947	4.611722758
N387	5.415048475	-2.862482779	3.007518349
N388	4.637205308	-1.391253773	4.673613094
N389	5.257469754	-0.587973875	2.589973789

N390	9.258350276	-6.541052005	4.117301489
N391	-6.024359594	-3.279930891	-2.317302795
N392	-2.062892391	-5.871739946	-2.521820174
N393	-6.257626609	-0.732238986	-1.776821426
N394	-8.504681219	0.353541706	-0.535812819
N395	-7.854764810	0.470310018	2.162629069
N396	-5.692001988	2.230252857	2.338926441
N397	-3.255390357	2.476079665	-1.201043773
N398	-2.311115852	0.619521487	-0.606069337
N399	-6.354082267	4.903336909	2.702108336
N400	-9.894322342	7.756956961	1.305565255
N401	-9.706327146	9.663391608	2.424549583
N402	9.488824953	2.893037935	1.931589896
N403	6.186277027	7.166600654	-1.224645832
N404	3.954410341	8.132689301	-3.767312868
N405	1.286553806	7.468733927	-4.041729256
N406	-3.459959109	5.894023201	-4.856555113
N407	-3.710643005	7.618852147	-6.420169392
N408	-4.760417088	7.749332125	-4.360528549
N409	0.483788361	6.207213833	-1.579220024
N410	0.886424633	8.315340973	0.210204240
N411	-0.506838414	10.615896469	-0.328276124
N412	-2.193334359	9.901207819	-3.875664083
N413	-2.867302517	11.700188642	0.711768485
N414	3.673028350	-5.579311578	-9.858727172
N415	2.091382212	-3.233190091	-7.606918050
N416	1.000338288	-0.408457868	-7.324978338
N417	3.024450455	1.604669552	-7.559134811
N418	-2.156816097	-3.951834840	-9.577860402
N419	-3.660519176	-1.997392693	-8.101197102
N420	7.908320047	-7.466308688	-5.689442158
N421	4.101958415	-6.025003442	-4.035912243
N422	3.399464177	-7.103273205	-5.815604980
N423	9.097013183	-7.674951194	-2.736640654
N424	-0.002643659	2.421090569	-0.144239418
N425	0.755310651	1.694931516	-0.560497305
O426	-6.517271995	-5.077847445	2.462878954
O427	-6.993007624	-2.351291533	5.057959603
O428	-4.942211103	-4.364184707	7.395598567
O429	-2.221323699	-3.397107196	5.399692562
O430	-2.622724403	0.032318269	5.404323760
O431	-2.485877536	0.016364269	8.953921398
O432	0.110675485	-2.876606066	9.930945793
O433	2.547460061	-2.987223617	6.168419956
O434	2.600066163	0.670955474	5.526699863
O435	2.035420307	2.561875146	8.826986103
O436	2.572218816	0.814370849	11.833145750
O437	7.195798380	-10.074031765	2.812433717
O438	8.264543722	-7.023560011	6.134569303
O439	11.866667433	-4.104902632	4.559411407
O440	-5.753989206	-0.950603379	0.451839095
O441	-3.928565984	-4.869704086	-3.346770890
O442	-7.504103355	2.403833638	-0.455743866
O443	-6.628252842	2.712968894	-3.176623180
O444	-9.820447228	1.565543174	2.624933209
O445	-5.503944404	1.875273797	4.586486552
O446	-4.236690840	5.346210313	3.436305668
O447	-6.567605100	7.607409804	4.971907087
O448	10.783997291	0.240374334	3.857298221
O449	3.621516888	5.098846240	3.464286393
O450	3.441443887	8.276034711	-1.550847351
O451	0.688483733	9.654424818	-4.426525978
O452	-1.608529187	6.989555961	-2.067502236
O453	-0.759624960	7.450209237	1.553992470
O454	2.354718576	5.909060771	0.951011483
O455	0.736469608	11.826455018	1.169432189
O456	-2.666396918	13.561796479	-0.588443699
O457	-3.513229271	10.762355213	-2.219789737
O458	-4.973364244	13.999234155	2.487011451
O459	-5.709232952	6.619910495	-1.674024883
O460	1.385765620	-5.298272332	-8.321420101
O461	-0.662403695	-1.541363917	-8.417188904
O462	1.600891656	2.896679958	-8.776623965
O463	5.428657224	3.492730460	-9.482689624
O464	-5.380535271	-3.487477100	-8.236899841
O465	-1.628886158	-7.185395010	-5.228375769
O466	0.198449074	-6.353013013	-6.198208415
O467	-4.404875616	1.487907257	-8.393376862
O468	9.775940490	-5.701142357	-3.616933285
O469	12.566923539	-7.110868244	-2.000060843

O470	0.337006429	-6.337814127	-0.794452158
O471	-0.177519353	-7.144221652	1.241440035
O472	5.077813413	-11.228482984	-2.465152049
O473	2.815961198	-11.240349476	-2.474960983
O474	5.110293846	-6.008688214	-0.997204752
O475	5.077761766	-7.516270328	0.671275437
O476	2.609034277	-6.343618596	-1.644842551
O477	9.052335621	-6.411774426	1.282415653
O478	7.133078923	-4.526371213	0.381185550
O479	-4.579321548	4.453172827	-2.646366360
O480	7.669594382	-10.602402105	-1.806314122
O481	7.562709695	-8.595422947	0.460603903
S482	3.308984098	4.703902544	-2.821361937
S483	1.802784841	1.674633318	-3.325993690
S484	1.881093282	-3.625786092	-3.060015495
S485	3.707179477	1.822919381	-0.345195726
S486	0.213966380	-0.901228788	-1.768448618
S487	3.610785125	-1.132621843	-5.474164124
S488	3.638016241	-3.581073961	-0.094830614
S489	5.346707603	1.420723180	-3.483759133
S490	5.558694346	-3.324595690	-3.181405463
S491	6.344658237	-0.866342742	-0.496778831
end			

Fe2-brNH-NH2-Fe6H to S2BH-Fe2-brNH-NH2-Fe6H

35, S=1/2

reactant

Fe(139)	-2.356
Fe(140)	-0.004
Fe(141)	-2.565
Fe(142)	2.693
Fe(143)	1.673
Fe(144)	2.397
Fe(145)	-0.016

bm612n2xhisto2b135tg.car_4

C1	-6.996980381	5.866981913	-3.111736279
C2	-8.454258232	5.459334775	-3.358316806
C3	-5.041051272	7.029414461	-4.041013133
C4	-4.589156059	7.880900019	-2.859936951
C5	-5.201628389	9.379321274	-1.031726589
C6	-4.756944036	8.531672113	0.164833726
C7	-4.760704963	6.350088136	1.256397099
C8	-3.325396652	5.909786317	0.946941088
C9	-1.717099716	5.389355470	-0.845715318
C10	-0.676375459	6.444082262	-0.443237284
C11	-0.055237854	8.779479346	-0.360749287
C12	0.110343016	9.072425605	1.136069907
C13	-1.015282806	9.674453759	3.222534680
C14	-0.814617059	8.566374858	4.262203975
C15	-1.293904645	6.221250067	4.830919304
C16	-0.330541725	5.065447656	4.567980586
C17	1.007033366	3.796276577	2.891214812
C18	2.487141254	4.235007469	2.977574501
C19	0.634997463	3.269017168	1.497815669
C20	1.564134538	2.119615138	1.100553097
C21	-0.853082472	2.917549200	1.482864753
C22	4.256259572	5.635602180	1.943589164
C23	4.514674487	6.747077440	2.972158369
C24	4.583247982	6.097010390	0.498655242
C25	5.956167429	6.777473394	0.416254491
C26	4.512176445	4.924051194	-0.486143172
C27	3.805575886	8.858852270	3.933372867
C28	4.137595854	8.459146412	5.356680767
C29	-6.951398728	1.681351585	11.678638145
C30	-5.859598419	0.706855864	11.225414682
C31	-3.501122484	0.604499292	10.398228164
C32	-2.481923544	0.668717132	11.553787957
C33	-2.950806839	1.241624172	9.106855849
C34	-1.680213678	0.568557375	8.573611907
C35	-1.295220360	1.109701207	7.191215879
C36	0.918329909	0.717802723	6.033687340
C37	-1.022351709	-0.631158967	13.029418931
C38	0.394229717	-0.950617120	12.601622811
C39	-4.777421834	1.864354784	-3.599280611
C40	-3.503266604	2.275755008	-4.361237859
C41	-4.340851346	1.135490529	-2.305089938
C42	-5.434915786	0.924490886	-1.237992159
C43	-6.275687924	-0.344641931	-1.379612921
C44	-1.938954638	1.447692469	-6.086821023
C45	-1.588900363	2.766381239	-6.787794460
C46	-1.911524784	0.246534281	-7.043173064
C47	-2.436865548	4.812054061	-7.835878887

C48	-1.792094038	5.869362080	-6.932796220
C49	-1.072662744	6.494125013	-4.678176705
C50	0.226437921	5.936227591	-4.097181965
C51	1.869517231	4.126756296	-4.014157762
C52	3.112481896	4.756969845	-4.701227667
C53	1.957169896	2.609367928	-4.252958539
C54	0.964606755	1.713489001	-3.579451993
C55	0.151327250	1.802851416	-2.473395546
C56	-0.033239852	-0.261053282	-3.257575630
C57	4.009583769	5.641457576	-6.827480055
C58	4.631877036	6.884420445	-6.195377253
C59	3.415418803	5.958185099	-8.218413470
C60	4.384246376	6.027237783	-9.360979085
C61	5.661026751	6.548511533	-9.440743548
C62	5.045373744	5.712529801	-11.398528039
C63	5.529733729	-1.852260879	8.088356539
C64	4.823509964	-2.382465099	9.320999192
C65	4.442072666	-1.240103418	7.166464141
C66	5.021878182	-0.615780303	5.919572261
C67	5.434320965	0.729145962	5.900002867
C68	5.169394396	-1.367085762	4.739560207
C69	5.959684693	1.310724176	4.741074287
C70	5.705385476	-0.804932829	3.580554918
C71	6.089918474	0.537463356	3.580494957
C72	7.306458673	-4.717448233	0.633984604
C73	7.901147176	-4.513024701	-0.770448756
C74	6.059517500	-3.857763531	0.791578396
C75	8.396875614	-5.556262195	-2.954324283
C76	7.519717728	-4.875689173	-4.008583280
C77	5.338980641	-3.754419654	-4.420469886
C78	5.375693597	-2.248125014	-4.054702121
C79	3.936804216	-4.367768114	-4.203576000
C80	2.725169978	-3.694042147	-4.857646500
C81	2.523881064	-3.934914755	-6.363758830
C82	3.838356852	-2.962731472	-8.254295859
C83	5.446490827	-0.524266707	-2.323611923
C84	6.784451990	0.154566531	-2.644397446
C85	5.178064316	-0.328330372	-0.815639308
C86	9.206997569	-0.084067209	-2.420975637
C87	10.060791327	-0.178366387	-3.675097118
C88	10.167978838	-0.894943738	-5.998237255
C89	10.470129098	0.267768922	-6.976579045
C90	9.607894027	-2.099358471	-6.761453306
C91	8.278228474	-1.824593448	-7.449178395
C92	9.894999233	2.543517109	-7.699624836
C93	11.242941811	3.248493135	-7.674789696
C94	8.759853509	3.559261530	-7.379891843
C95	7.381275486	2.948496536	-7.509854182
C96	6.715713342	2.951800892	-8.746439916
C97	6.747305411	2.319578777	-6.424827648
C98	5.472871020	2.334094617	-8.904743232
C99	5.516352265	1.672647501	-6.569615131
C100	4.891982035	1.683930862	-7.814661388
C101	-5.078697360	-9.163675680	1.321408879
C102	-5.205807816	-8.077441254	0.267109982
C103	-3.948451057	-6.385847984	-1.048035016
C104	-3.112598982	-6.767308359	-2.276452241
C105	-0.836624369	-7.267755289	-3.057816821
C106	0.366664761	-8.097083284	-2.618194873
C107	1.904687851	-8.813361148	-0.875196713
C108	1.788412392	-10.320939828	-0.975944398
C109	-7.073061706	-5.828947876	-4.314808206
C110	-6.340582095	-5.149512278	-5.476640414
C111	-7.083231395	-4.901970835	-3.078610577
C112	-8.126067450	-5.367180958	-2.045114674
C113	-7.947118522	-4.631729316	-0.735593926
C114	-4.213487851	-4.685676028	-6.581051946
C115	-3.117204529	-5.629256988	-7.034355360
C116	-3.744510369	-3.230980283	-6.328232231
C117	-3.021075533	-3.152371435	-5.016122722
C118	-1.662657934	-3.493393144	-4.926051355
C119	-3.753010523	-2.944112189	-3.830834509
C120	-1.074773846	-3.709527958	-3.674863374
C121	-3.162857238	-3.157396601	-2.586827716
C122	-1.832360758	-3.585363532	-2.507870621
C123	-4.555139349	-7.370659107	7.124945188
C124	-3.917617569	-7.142120511	8.503029621
C125	-5.193973283	-6.086070392	6.577697828
C126	-5.622978316	-6.123224963	5.145642390
C127	-6.660366497	-6.837389734	4.582598354

	C128	-5.797351133	-5.480451634	3.059644568
	C129	-3.998306447	-6.136073809	10.704956995
	C130	-3.577416495	-7.412226414	11.398535471
	C131	-5.969759928	0.281749780	3.478222998
	C132	-5.490161863	-0.164946511	4.875002010
	C133	-5.757520993	-1.652341050	5.225136421
	C134	-7.243645892	-1.875144209	5.594089684
	C135	-7.575378806	-3.286765874	6.084501129
	C136	-9.022223313	-3.450247839	6.597816599
	C137	-4.888258745	-2.076197487	6.420026523
	C138	-0.224622819	-3.475549895	2.288251915
	Fe139	-1.730014386	-4.757100256	2.372381289
	Fe140	0.735716843	-2.010681514	0.764931733
	Fe141	1.579195661	-3.637473110	3.120696582
	Fe142	0.477634720	-4.941517337	1.185226399
	Fe143	2.987544263	-4.082222924	0.956752580
	Fe144	-0.702530883	-3.417725257	4.240364322
	Fe145	-1.619209490	-1.848166312	2.071231518
	H146	-8.815757319	4.579894450	-1.552633317
	H147	-8.428670695	3.515955941	-2.726968336
	H148	-6.980578382	6.869603096	-4.908426979
	H149	-8.577843753	5.207004586	-4.423711941
	H150	-9.071792287	6.357455887	-3.185443713
	H151	-4.424458896	6.118074678	-4.018801952
	H152	-6.491757517	8.564440823	-2.550155960
	H153	-4.801044537	7.571845449	-4.964769219
	H154	-6.082261934	9.967466215	-0.742991766
	H155	-5.871845489	6.916654658	-0.484929759
	H156	-4.378821368	10.076907470	-1.239006211
	H157	-1.364092148	4.426688733	-0.465047101
	H158	-3.803783665	5.796858915	-1.040443428
	H159	-5.415590066	5.468451124	1.287514803
	H160	0.929717191	8.486816136	-0.745123976
	H161	-1.884199370	7.952136461	-1.159128649
	H162	-4.755473697	6.812674329	2.250283170
	H163	-1.774131520	5.319990607	-1.939313205
	H164	-1.947539227	9.183720310	1.358031846
	H165	-0.377210001	9.699225173	-0.867815012
	H166	-1.474574483	3.817953094	1.514296647
	H167	-1.125612609	2.341972421	0.584916509
	H168	-1.128531241	2.338458714	2.370265819
	H169	0.780810012	4.086162220	0.774469197
	H170	-1.968466344	10.171846276	3.452365442
	H171	3.535284704	4.419894074	-0.468168944
	H172	2.751637713	7.742664929	2.463546228
	H173	6.015349044	7.679247319	1.042081680
	H174	1.340475809	1.778810170	0.082821654
	H175	1.474451773	1.248573505	1.764760771
	H176	6.199289945	-1.033186257	8.413357064
	H177	4.681933061	5.277034769	-1.513231770
	H178	6.158702987	7.070764521	-0.624503565
	H179	5.287385293	4.176190875	-0.254616217
	H180	6.754138412	6.089693459	0.737339293
	H181	3.819029835	6.842600603	0.209433600
	H182	4.935950748	4.818239645	2.227251730
	H183	2.207321849	5.458345505	1.340547195
	H184	2.618445294	2.435606565	1.103596113
	H185	4.391397373	-1.025749605	-0.492788931
	H186	4.805224365	0.699173362	-0.690671929
	H187	6.331397858	0.059791877	0.732230653
	H188	6.770688351	2.005780542	2.507667852
	H189	5.785612540	-1.392982393	2.666898343
	H190	4.826431706	-2.403398991	4.712059244
	H191	5.328274321	1.341521805	6.799110438
	H192	-0.200876224	5.546372826	2.582776655
	H193	-2.303642884	5.781566812	4.846117767
	H194	-1.071895191	6.599350223	5.835840558
	H195	-1.766330486	7.211439699	3.021123986
	H196	-0.204309615	10.398389706	3.375758312
	H197	6.248329135	2.365114034	4.736151123
	H198	0.888546399	2.999307562	3.636117529
	H199	4.612344170	9.506255469	3.552778446
	H200	2.879161176	9.452643353	3.961121117
	H201	3.704265010	7.487689531	5.689982852
	H202	-1.359118460	-1.383065283	13.759764032
	H203	-7.248308329	-1.765288628	-0.293026377
	H204	-5.540768863	-1.795402213	3.264415260
	H205	-9.134785772	-5.152081688	-2.425790989
	H206	-8.043451275	-6.451625982	-1.882574628
	H207	-7.314058178	-3.867612017	-3.377585111

H208	-3.294026064	-6.701220331	-6.780723310
H209	-8.110235933	-5.905520203	-4.675734785
H210	-3.892144764	0.167169971	-2.580941044
H211	-6.135136530	1.777900216	-1.242475977
H212	-4.497391194	-5.786398301	-4.758514689
H213	-4.904867623	-4.650386444	-7.448468445
H214	-5.670782663	-7.207506770	-3.646470553
H215	-6.345465383	-0.631696471	0.709048766
H216	-6.075830518	-4.884097498	-2.635772851
H217	-7.233287850	-7.700958422	-3.489366392
H218	-6.327488369	1.553285453	-4.929341324
H219	-3.543994740	1.738703535	-1.845544017
H220	-6.932716289	-6.181918224	-0.077993169
H221	-4.965804947	0.928261626	-0.243510374
H222	1.976832463	4.370299673	-2.947829508
H223	-0.047804144	2.634194856	-1.805194322
H224	-0.808906355	7.409886245	-5.224814709
H225	-2.208650137	4.766551954	-5.217396575
H226	1.976692062	5.140411906	-6.368023414
H227	6.306048938	7.015581274	-8.704050832
H228	3.899004019	7.526385101	-5.648854652
H229	2.816260168	6.883372517	-8.151748834
H230	4.786522406	4.866305269	-6.912642652
H231	5.105762411	5.431015156	-12.446651314
H232	2.966998670	2.298301503	-3.936855100
H233	1.896364985	2.430017884	-5.337566849
H234	6.966121381	6.595287264	-11.134930360
H235	2.707200035	5.149827584	-8.460146571
H236	0.086037819	4.183321643	-5.212700382
H237	-1.713345883	6.802720469	-3.838474069
H238	-1.791334146	4.740981757	-8.722642348
H239	-0.334718751	-1.302471035	-3.357282611
H240	-3.413430735	5.190173540	-8.164982790
H241	7.069368387	-3.197123065	7.929527425
H242	6.506368981	-2.673518677	6.486282850
H243	5.223002169	-2.650369092	-2.003549110
H244	4.674867762	0.025600835	-2.881149171
H245	7.746270262	-1.577199122	-2.115570740
H246	1.279636088	0.056784192	-4.913403428
H247	0.966064735	-0.122605375	-7.028052549
H248	-0.725535063	-0.446849429	-8.440729476
H249	2.483087564	0.346856356	-6.951673163
H250	-2.110118996	-0.663687825	-6.453347186
H251	-2.700432749	0.360963702	-7.802295773
H252	9.549836852	-2.968065441	-6.090774273
H253	10.335245498	-2.336167793	-7.553128742
H254	7.460277929	-3.510023742	-6.567998079
H255	6.405070000	-2.539646498	-7.593541349
H256	4.939436232	-4.251535438	-9.410209170
H257	1.452395502	-4.047206704	-6.586913637
H258	4.966376182	2.356556340	-9.873591304
H259	7.201986305	2.329192186	-5.430863913
H260	4.205733436	-0.938793525	-8.402175883
H261	3.750157612	-4.380139612	-3.117570774
H262	6.004803110	-4.722941353	-2.642784348
H263	2.716053250	-2.612592723	-4.648893755
H264	-3.571950385	3.166992468	-7.050006533
H265	8.586773219	-6.584630195	-3.289894564
H266	-3.955415868	0.738971763	-5.544398825
H267	3.981651569	-5.422993834	-4.520038636
H268	5.075097955	1.139484595	-5.727176856
H269	9.356879363	-5.019915708	-2.957266610
H270	3.713435539	-5.010887571	-8.452062946
H271	4.726340297	-1.890761995	-9.752700230
H272	-6.089384019	0.299584487	-3.846668764
H273	-5.270093988	2.810013380	-3.304884502
H274	7.172016901	3.445904683	-9.607213355
H275	-1.141494569	1.322048362	-5.340583446
H276	5.645385286	-3.823389054	-5.468106581
H277	3.006046432	-4.878455138	-6.651268025
H278	1.843649607	-4.105302310	-4.349118681
H279	2.671435822	-1.896440576	-6.973482194
H280	9.125408028	0.980463014	-2.154287126
H281	8.434153758	-0.842418217	-4.719732102
H282	11.167923532	-1.182955986	-5.637479064
H283	9.757313772	-0.593080628	-1.618477883
H284	9.131457298	1.454606647	-6.019357582
H285	-4.099266361	-9.657288454	1.235016935
H286	-5.086016004	-8.637056151	2.297858062
H287	-3.721049340	-7.667735325	6.471516411

	H288	-6.215790669	-10.701697006	2.027705228
	H289	-7.037406891	-9.651414936	1.076430111
	H290	5.669245773	-4.006548139	1.808132457
	H291	8.612619177	-3.408401399	1.498996262
	H292	7.433707111	-6.453371710	-1.270849711
	H293	6.319143553	-2.797394039	0.658557157
	H294	9.098927531	-4.965139074	1.611468610
	H295	2.077099557	-8.559094287	0.183146819
	H296	7.033894907	-5.775977789	0.771173514
	H297	-2.765939949	2.312626671	9.295563567
	H298	-3.774656885	-0.439303542	10.199176002
	H299	0.182804526	-7.587918610	-0.595506934
	H300	2.611859096	0.102178042	5.081449205
	H301	-1.362165713	-7.838430671	-3.833488800
	H302	-3.724967180	-2.986400348	-1.666703584
	H303	-0.432844009	-6.363350163	-3.545583939
	H304	-0.972582692	2.155492490	7.257388131
	H305	-1.468711787	-6.581761410	-1.094948890
	H306	-2.173062144	1.081191973	6.524465384
	H307	-3.751786151	1.168612372	8.354110117
	H308	-1.841596697	-0.516918126	8.489993010
	H309	-0.837232989	0.720835601	9.266963291
	H310	-4.635459436	-2.586221082	-6.331879169
	H311	-4.695099926	2.311992175	10.953486285
	H312	-7.706301231	1.679301883	10.877400593
	H313	-1.375092297	-3.759810846	-1.535642173
	H314	-6.188117563	3.188748072	12.856052509
	H315	-7.221787918	3.720705647	11.701840432
	H316	-0.515556885	-0.658413547	6.335947860
	H317	-0.024159991	-3.993976169	-3.591856617
	H318	-4.798578783	-2.631140731	-3.889641989
	H319	-6.870531184	-4.052403783	9.858218015
	H320	-6.079068468	-5.837328761	7.188155044
	H321	-5.132296398	-2.428929393	8.952648252
	H322	-8.095579083	-4.497429696	8.998955086
	H323	0.741538561	-10.708913800	-1.017588460
	H324	-2.538748216	-2.939873157	7.295477711
	H325	-5.939412882	0.482382507	5.638433801
	H326	-3.188027735	-7.729000713	0.394841959
	H327	-7.483457398	-1.148993475	6.386422596
	H328	-7.458101781	-6.698790967	2.598931607
	H329	-5.667512818	-4.958355525	2.119858638
	H330	-7.427437090	1.225320186	12.563246597
	H331	1.480485607	-1.186399077	5.383507258
	H332	-2.152371257	-2.970027885	9.634938723
	H333	-1.050942063	-3.390117996	7.335949031
	H334	-2.210353652	-1.394924495	11.403291938
	H335	0.699692002	2.754280339	6.252175507
	H336	-7.431723985	-3.999694854	5.257125672
	H337	-6.886993425	-3.582973740	6.888198690
	H338	-7.864104058	-1.621181269	4.720744812
	H339	-3.503114014	-5.518786517	-0.535451886
	H340	-1.085584953	-3.638421463	-5.842568190
	H341	-3.105610943	-2.942156324	-7.177229878
	H342	-4.401316517	0.011295824	4.887604052
	H343	-3.535335774	-2.960118725	10.348346132
	H344	-5.550592944	-2.042430957	10.389246761
	H345	2.176886817	2.252208292	5.622041140
	H346	-7.335733084	-7.569743963	5.009028213
	H347	-4.490826284	-5.253283127	6.705159145
	H348	-4.955279225	-6.133409962	-1.384702310
	H349	2.784733151	-8.481432543	-1.446960423
	H350	3.897652649	-0.483317209	7.751502885
	H351	3.728974580	-2.034532386	6.892228501
	H352	-5.043972630	-9.331943565	7.477095380
	H353	-6.257876185	-8.290212307	7.828160623
	H354	-4.761208531	-5.656421881	11.339004852
	H355	-5.499232716	-5.950892712	9.200220540
	H356	-4.172412792	-8.314409016	11.118448795
	H357	-3.141139926	-5.446961054	10.642087303
	H358	4.349487787	-3.383602471	9.178964591
	H359	11.828496919	3.113821497	-6.734132313
	H360	-0.993771253	0.349254642	13.536785850
	H361	0.641946304	-0.683149671	11.545205849
	H362	9.745553720	2.143915056	-8.715773211
	H363	8.910894967	3.953500301	-6.362702184
	H364	8.871852936	4.401216783	-8.078455627
	H365	-3.011367163	-1.298073731	1.960883226
	H366	3.256275564	1.270982391	-8.790926694
	H367	-1.235364953	0.398627715	1.179551708

H368	0.406256924	-0.780084127	3.155549798
H369	-1.353557299	0.530055949	2.934018267
H370	-0.947126430	0.172588039	-1.428407769
Mo371	-3.460939273	-3.629198785	4.188031363
N372	0.035486062	-0.791788219	2.196889301
N373	-1.114015995	-0.023259408	2.109834422
N374	-8.962311838	4.368984706	-2.542931329
N375	-6.455956513	6.695248330	-4.054977578
N376	-5.530735684	8.610528772	-2.220528097
N377	-5.279674362	7.282071360	0.264016981
N378	-3.059874539	5.672329316	-0.358997622
N379	-1.024452872	7.734726293	-0.649979800
N380	-1.043099910	9.256130973	1.831455710
N381	-1.263607886	7.332878850	3.902607045
N382	0.118802379	4.897176178	3.300188596
N383	2.886739935	5.127449106	2.026292434
N384	3.567257819	7.715038746	3.075354676
N385	-6.470327185	3.053482284	11.882810788
N386	-4.716783101	1.296421381	10.815441405
N387	-0.240171567	0.302379015	6.563389739
N388	1.372318941	1.982485446	6.190545309
N389	1.656477291	-0.156949943	5.319485409
N390	-1.948388415	-0.523894967	11.918143359
N391	-5.614843738	1.004286221	-4.443636347
N392	-6.659151434	-0.929678559	-0.231703011
N393	-3.230502055	1.466784381	-5.420255329
N394	-2.629954920	3.502784024	-7.238934530
N395	-1.791575572	5.627385369	-5.592277416
N396	0.612256764	4.685496392	-4.491916822
N397	0.818327782	0.414676147	-4.048020634
N398	-0.458324401	0.573127790	-2.304240962
N399	2.917399741	5.167322721	-5.982092828
N400	4.011140948	5.509222132	-10.591107450
N401	6.064899254	6.336480432	-10.742193288
N402	6.212036904	-2.964263812	7.422593528
N403	8.276236068	-4.359143301	1.684395691
N404	7.861401030	-5.593793702	-1.605587793
N405	6.278004835	-4.529514919	-3.614803143
N406	3.042766628	-2.840258192	-7.193061494
N407	4.132614836	-4.156822334	-8.801247470
N408	4.422046488	-1.862990348	-8.783457029
N409	5.412989693	-1.930360234	-2.731288988
N410	7.878790713	-0.653134245	-2.535366706
N411	9.403220594	-0.538702009	-4.819785297
N412	7.345674431	-2.806796138	-7.310195848
N413	9.857607231	1.453037436	-6.731290698
N414	-6.149083162	-10.153527605	1.167357620
N415	-4.049921571	-7.496152132	-0.109485372
N416	-1.793640542	-6.899045190	-2.030195573
N417	0.697345422	-8.127427025	-1.303016204
N418	-6.589791510	-7.200528722	-4.106163538
N419	-4.984743520	-5.282661768	-5.507235970
N420	-5.519043277	-8.487244863	7.145540260
N421	-5.086654234	-5.285583479	4.169615741
N422	-6.751373260	-6.417638149	3.272967260
N423	-4.601302595	-6.393892746	9.409622505
N424	2.133097970	-0.348289862	-1.166907945
N425	1.587343512	-0.964689172	-0.389114777
O426	-6.353277723	5.514045441	-2.109187609
O427	-3.393326812	7.878876920	-2.515530311
O428	-3.963455164	8.995967233	0.999864220
O429	-2.465064541	5.787661352	1.837085297
O430	0.422042755	6.120516113	0.036764626
O431	1.232808210	9.172894538	1.656903212
O432	-0.315454487	8.832100884	5.363705753
O433	-0.052935456	4.307106796	5.513928435
O434	3.242943736	3.781340135	3.849016558
O435	5.551220710	6.773799064	3.654670644
O436	4.776995731	9.171801284	6.115815432
O437	-6.083083855	-0.520831911	11.236900550
O438	-2.203788492	1.752933338	12.098113955
O439	1.227542416	-1.445261657	13.344489983
O440	-2.780586895	3.236006274	-4.028728504
O441	-6.613475677	-0.791057112	-2.503024941
O442	-0.403562650	3.128261215	-6.900113770
O443	-0.625091286	0.163820526	-7.688638628
O444	-1.315805161	6.888844472	-7.451046559
O445	0.880655448	6.615278851	-3.297169566
O446	4.209874345	4.792790895	-4.136480170
O447	5.807261169	7.193674009	-6.292733211

	O448	4.746551817	-1.783971670	10.384545086
	O449	6.579838620	1.056784384	2.395500213
	O450	8.394633491	-3.429202978	-1.128103784
	O451	7.973566620	-4.690904922	-5.156523092
	O452	5.375788114	-1.392272216	-4.956356943
	O453	6.846668413	1.359825039	-2.919037696
	O454	6.372362846	-0.527179799	-0.049713357
	O455	11.272402220	0.070790056	-3.638697587
	O456	11.278854206	0.110071071	-7.902436287
	O457	8.085861829	-0.802617693	-8.123510606
	O458	11.652076550	3.937393315	-8.596217542
	O459	3.677887912	0.982952157	-7.958020846
	O460	-6.333872145	-7.740868630	-0.172483902
	O461	-3.633700709	-6.962751354	-3.396236579
	O462	1.039714063	-8.686743358	-3.482051896
	O463	2.749394167	-11.073791145	-0.979055156
	O464	-6.958434857	-4.496010572	-6.334955377
	O465	-8.377159152	-3.502330119	-0.530113888
	O466	-7.228404722	-5.265549590	0.214715928
	O467	-2.131918814	-5.284501457	-7.670619698
	O468	-2.828014868	-7.668010990	8.806331989
	O469	-2.713720394	-7.461881647	12.264212095
	O470	-5.905126840	-0.623254831	2.543777446
	O471	-6.321947376	1.466009849	3.305628759
	O472	-9.182757002	-4.065775515	7.710953439
	O473	-9.952293620	-2.992558341	5.875831968
	O474	-4.041006713	-3.049818679	6.235134486
	O475	-5.044405496	-1.496620898	7.514872692
	O476	-5.384663467	-2.461882127	4.087241513
	O477	-2.557094852	-2.923682639	10.537077094
	O478	-1.708027135	-2.864755387	7.830755132
	O479	1.840267289	-0.309443204	-6.590627367
	O480	-7.556768582	-4.754784115	9.807533720
	O481	-5.243155936	-2.822336935	9.863193392
	S482	4.738855907	-4.290298305	-0.425241492
	S483	1.367412120	-3.834705663	-0.563193958
	S484	-3.403342533	-3.398082733	1.811670310
	S485	2.794895579	-2.138333363	2.024294434
	S486	-1.321828704	-1.561094288	-0.201901265
	S487	-1.121611869	-6.488330980	1.106839026
	S488	-2.077423830	-1.665224089	4.384595058
	S489	2.292585870	-5.639636300	2.373428232
	S490	-1.859148282	-5.317995263	4.557822029
	S491	1.223261548	-3.349871928	5.306908762
	end			

TS

Fe(139) -2.358	bm612n2xhisto2b135th.car_1			
Fe(140) -0.011	C1	-6.994900083	5.857611724	-3.110773184
Fe(141) -2.555	C2	-8.452396393	5.459187225	-3.369650081
Fe(142) 2.699	C3	-5.017520829	6.993934614	-4.026280542
Fe(143) 1.660	C4	-4.568747212	7.849084693	-2.846927281
Fe(144) 2.407	C5	-5.181295620	9.356877082	-1.027267174
Fe(145) -0.010	C6	-4.749743203	8.509819315	0.174671259
	C7	-4.781159240	6.331330111	1.275311008
	C8	-3.349268025	5.874105130	0.969999690
	C9	-1.743580348	5.347865415	-0.821821217
	C10	-0.702903492	6.401504869	-0.415583834
	C11	-0.055838954	8.724966659	-0.337574802
	C12	0.104214377	9.037498697	1.155569178
	C13	-1.026279586	9.669298779	3.231256703
	C14	-0.835018466	8.559323712	4.269836561
	C15	-1.268790022	6.199917190	4.805023616
	C16	-0.287453047	5.064882617	4.525225548
	C17	1.038199594	3.787033924	2.852402016
	C18	2.518830065	4.214936097	2.934921024
	C19	0.674056002	3.266992315	1.450488410
	C20	1.613145827	2.139680237	1.014225392
	C21	-0.804150243	2.886682911	1.443145219
	C22	4.278263821	5.631874063	1.913342498
	C23	4.526140883	6.731200241	2.955923106
	C24	4.606220528	6.112656542	0.475482319
	C25	5.980182362	6.793040585	0.410526034
	C26	4.535737346	4.952454326	-0.523675831
	C27	3.824995681	8.850458092	3.899261706
	C28	4.160481702	8.458925038	5.323487550
	C29	-6.972200032	1.688248308	11.659473608
	C30	-5.878079776	0.707603069	11.221940665
	C31	-3.510950958	0.595588422	10.416535692
	C32	-2.490311680	0.660522729	11.570319681

C33	-2.961548530	1.228846049	9.121847459
C34	-1.688759926	0.561886759	8.583046583
C35	-1.335224059	1.085426211	7.184641064
C36	0.904492970	0.761569998	6.044377574
C37	-1.018277327	-0.638959803	13.034461070
C38	0.393171613	-0.966042612	12.597174219
C39	-4.770730586	1.856865646	-3.598425257
C40	-3.500284710	2.285305726	-4.357578255
C41	-4.336240201	1.128128674	-2.303796912
C42	-5.436226479	0.923104699	-1.241051341
C43	-6.279544617	-0.344898032	-1.380233748
C44	-1.916829862	1.462846033	-6.078875843
C45	-1.580772515	2.780742279	-6.787070949
C46	-1.890599960	0.260748099	-7.035111231
C47	-2.445317560	4.826770612	-7.817283796
C48	-1.788779007	5.874896399	-6.910987712
C49	-1.065815182	6.489493142	-4.654054476
C50	0.240040035	5.942658626	-4.078100818
C51	1.887279484	4.135340263	-3.994066824
C52	3.121430919	4.777062718	-4.681889461
C53	1.958833219	2.620736765	-4.238322435
C54	0.938833008	1.750266907	-3.556112963
C55	0.119897479	1.859368950	-2.454839904
C56	-0.118407905	-0.180348021	-3.199950986
C57	4.015477185	5.632572050	-6.821859732
C58	4.637974766	6.876750446	-6.194541565
C59	3.413600821	5.950052947	-8.209441844
C60	4.377535237	6.018901451	-9.356083851
C61	5.655470264	6.536705141	-9.438663913
C62	5.031502598	5.705807782	-11.395952143
C63	5.535295514	-1.850232338	8.086332830
C64	4.832543474	-2.374002509	9.323939182
C65	4.448625775	-1.239425023	7.163678819
C66	5.029794442	-0.613771838	5.917994843
C67	5.445642202	0.730296204	5.901719116
C68	5.177467702	-1.363118251	4.736728682
C69	5.972425887	1.313538150	4.744437740
C70	5.714994831	-0.799250960	3.579432626
C71	6.100843682	0.542917283	3.581969314
C72	7.310909017	-4.707719082	0.639633685
C73	7.899496428	-4.507144785	-0.768593996
C74	6.061336539	-3.852418498	0.799338492
C75	8.395592705	-5.556087814	-2.949555513
C76	7.520912876	-4.871000000	-4.002948985
C77	5.336102978	-3.760183862	-4.419310478
C78	5.380284303	-2.252910142	-4.057877692
C79	3.932907141	-4.368434318	-4.194849208
C80	2.721754446	-3.697660994	-4.852875013
C81	2.523694647	-3.945585214	-6.358442163
C82	3.840789906	-2.971540670	-8.249163538
C83	5.449098773	-0.523487345	-2.331618155
C84	6.790850228	0.149516346	-2.648830856
C85	5.174066127	-0.320047628	-0.826830048
C86	9.212345840	-0.097130963	-2.422111077
C87	10.058011146	-0.182636204	-3.682048852
C88	10.161165488	-0.900661470	-6.003160537
C89	10.466863797	0.267220157	-6.974732213
C90	9.599113033	-2.098636805	-6.775652572
C91	8.267001824	-1.816325332	-7.455367700
C92	9.888115939	2.540455388	-7.700481249
C93	11.235622962	3.245332666	-7.666555161
C94	8.753347240	3.557615871	-7.383073856
C95	7.374650319	2.947236805	-7.512299918
C96	6.706540940	2.954863237	-8.747536480
C97	6.743198368	2.314464541	-6.427917832
C98	5.463948543	2.336938805	-8.905384095
C99	5.512938269	1.666439607	-6.572794323
C100	4.886089428	1.682073587	-7.816627328
C101	-5.082582252	-9.165068717	1.316249342
C102	-5.206966417	-8.077274609	0.263570214
C103	-3.946215270	-6.383031057	-1.043287100
C104	-3.107374228	-6.759354846	-2.271105815
C105	-0.832416963	-7.254505503	-3.055095672
C106	0.375805534	-8.081045521	-2.624913605
C107	1.911042923	-8.811662586	-0.882773329
C108	1.781825149	-10.321119478	-0.944784475
C109	-7.069159635	-5.824738530	-4.316785440
C110	-6.337829782	-5.143606056	-5.479021177
C111	-7.083254485	-4.896132388	-3.080947086
C112	-8.124851661	-5.363324480	-2.046858978

	C113	-7.947190369	-4.629725279	-0.735734250
	C114	-4.210284425	-4.667925780	-6.580478742
	C115	-3.110802370	-5.611103286	-7.027518812
	C116	-3.745149640	-3.209902395	-6.335594878
	C117	-3.028868422	-3.129885456	-5.019786611
	C118	-1.673196570	-3.478763446	-4.920597886
	C119	-3.770056120	-2.929340790	-3.839427255
	C120	-1.099753426	-3.717670837	-3.667369586
	C121	-3.195850676	-3.170326802	-2.592892791
	C122	-1.870070202	-3.610785054	-2.507537418
	C123	-4.539522771	-7.365266545	7.127237540
	C124	-3.906193817	-7.135223226	8.506429956
	C125	-5.190568324	-6.084793339	6.583746814
	C126	-5.618466670	-6.121217638	5.151152659
	C127	-6.651721648	-6.842555806	4.590294950
	C128	-5.804033310	-5.477948693	3.065853875
	C129	-4.000750983	-6.135700468	10.710548239
	C130	-3.575587473	-7.411759448	11.401878480
	C131	-5.980373669	0.284235473	3.477712549
	C132	-5.507753062	-0.163741893	4.875538271
	C133	-5.771072111	-1.655349935	5.216754216
	C134	-7.258691434	-1.882914669	5.578205301
	C135	-7.591978641	-3.294152218	6.069515700
	C136	-9.039396539	-3.453926727	6.583795529
	C137	-4.902080968	-2.076913043	6.411541318
	C138	-0.223200965	-3.483178741	2.293051764
	Fe139	-1.733661418	-4.751086914	2.367603168
	Fe140	0.737992272	-1.975958032	0.744578219
	Fe141	1.576206773	-3.615490415	3.127285033
	Fe142	0.489726855	-4.943776600	1.198524726
	Fe143	2.994512738	-4.074642519	0.974407201
	Fe144	-0.705505588	-3.412004282	4.237361048
	Fe145	-1.625675111	-1.822060011	2.039118825
	H146	-8.827890295	4.573521667	-1.569668273
	H147	-8.444792232	3.512286021	-2.747649545
	H148	-6.950187982	6.850019376	-4.912393172
	H149	-8.569925567	5.211170413	-4.436569321
	H150	-9.065347746	6.360475856	-3.197119669
	H151	-4.411192183	6.075913299	-3.991243136
	H152	-6.467705301	8.549629519	-2.553201434
	H153	-4.760950840	7.525810390	-4.951743086
	H154	-6.059721750	9.951598984	-0.745177012
	H155	-5.879403964	6.905363938	-0.472370440
	H156	-4.353022519	10.048765412	-1.231770596
	H157	-1.392369270	4.384313021	-0.442914398
	H158	-3.832784756	5.742464304	-1.015990364
	H159	-5.447192361	5.458007082	1.306441521
	H160	0.926255647	8.405429500	-0.707798818
	H161	-1.896460561	7.918728559	-1.135581033
	H162	-4.774033955	6.796475408	2.268052029
	H163	-1.799634391	5.279132600	-1.915450648
	H164	-1.953552167	9.171488719	1.366205644
	H165	-0.354318831	9.642645409	-0.861660707
	H166	-1.450103154	3.768539540	1.511157273
	H167	-1.073823385	2.331920053	0.530860018
	H168	-1.044161927	2.279113343	2.319809873
	H169	0.798300809	4.096116915	0.738828728
	H170	-1.976440690	10.174501114	3.455734147
	H171	3.551220691	4.463641424	-0.526449946
	H172	2.777324775	7.743036092	2.421868246
	H173	6.033690702	7.687698671	1.047515996
	H174	1.357684483	1.811704739	-0.001056516
	H175	1.560677736	1.258289089	1.669372312
	H176	6.206446482	-1.030656512	8.407668899
	H177	4.726899776	5.312393209	-1.544185868
	H178	6.193095800	7.098438299	-0.624388510
	H179	5.293906200	4.189173114	-0.286649357
	H180	6.774644332	6.101320139	0.731799257
	H181	3.843956095	6.862934994	0.193388553
	H182	4.959926675	4.812279348	2.185074506
	H183	2.220641758	5.477072558	1.332717220
	H184	2.661179609	2.474499142	0.985747242
	H185	4.383123433	-1.012860553	-0.504909062
	H186	4.804974861	0.709956678	-0.707533294
	H187	6.321636593	0.070595284	0.725608889
	H188	6.777998771	2.013487787	2.510948067
	H189	5.796613974	-1.385378789	2.664697659
	H190	4.834851566	-2.399504305	4.707887613
	H191	5.341469935	1.340443487	6.802516259
	H192	-0.200842109	5.511561409	2.523147183

H193	-2.270796830	5.743444495	4.825851077
H194	-1.041341317	6.568832173	5.811853998
H195	-1.756167806	7.202788763	3.007079129
H196	-0.210403039	10.387104282	3.387321142
H197	6.262360439	2.367494537	4.741594808
H198	0.914179554	2.987109148	3.592945629
H199	4.631812069	9.495679468	3.513473437
H200	2.899259260	9.445568627	3.926569827
H201	3.719381653	7.494988522	5.667319026
H202	-1.352948333	-1.385491244	13.771366657
H203	-7.260074086	-1.758100179	-0.290235617
H204	-5.541485384	-1.778756558	3.252537970
H205	-9.134146106	-5.148897943	-2.426499457
H206	-8.041271352	-6.447830529	-1.885201271
H207	-7.316733936	-3.862557132	-3.380440991
H208	-3.289638372	-6.682991266	-6.774658680
H209	-8.105264545	-5.906921041	-4.678488334
H210	-3.888977992	0.158988018	-2.577350289
H211	-6.135722377	1.776980961	-1.254462141
H212	-4.491254121	-5.760392309	-4.752974087
H213	-4.901159610	-4.640309225	-7.448437085
H214	-5.658914225	-7.193768516	-3.646403492
H215	-6.353714135	-0.624818648	0.708134562
H216	-6.076008923	-4.874816361	-2.637805135
H217	-7.218128245	-7.694195509	-3.483203916
H218	-6.305691149	1.531863462	-4.942910852
H219	-3.535443717	1.724004866	-1.842678360
H220	-6.933870679	-6.180591276	-0.079846224
H221	-4.974548227	0.931446899	-0.243317417
H222	1.996555166	4.367120890	-2.925702363
H223	-0.066993593	2.725398701	-1.839584452
H224	-0.811363862	7.411929358	-5.194152173
H225	-2.197178380	4.759456473	-5.200314569
H226	1.992131266	5.094257227	-6.366924857
H227	6.303553197	7.000162177	-8.702352442
H228	3.906524329	7.518439924	-5.646957775
H229	2.815837444	6.875797009	-8.139144662
H230	4.793882108	4.858554557	-6.912742108
H231	5.087476668	5.426044927	-12.444687496
H232	2.964998574	2.295753093	-3.923440122
H233	1.901943653	2.444064346	-5.324444453
H234	6.955268494	6.583330660	-11.136644140
H235	2.703353331	5.142942787	-8.449115700
H236	0.096944948	4.183838305	-5.179314691
H237	-1.708953769	6.784037066	-3.810828057
H238	-1.812296026	4.765865671	-8.713470632
H239	-0.423626618	-1.215211180	-3.297199887
H240	-3.426328586	5.208004002	-8.129503112
H241	7.073390594	-3.196709699	7.935180623
H242	6.514553488	-2.677375152	6.489300763
H243	5.220146101	-2.648002983	-2.006409981
H244	4.682811794	0.026194081	-2.897161268
H245	7.746555925	-1.583961437	-2.111403340
H246	1.234042599	0.056255294	-4.848700405
H247	0.969448599	-0.128255953	-7.012122797
H248	-0.713084107	-0.428445976	-8.441457187
H249	2.481276261	0.345239689	-6.939360641
H250	-2.078185327	-0.649928114	-6.442543857
H251	-2.687141066	0.370526797	-7.787218863
H252	9.543036891	-2.972566793	-6.111675906
H253	10.323536173	-2.328167051	-7.572219094
H254	7.442055427	-3.483799279	-6.550279693
H255	6.381739770	-2.506629334	-7.563466367
H256	4.967971220	-4.250305679	-9.388042624
H257	1.452483906	-4.058831746	-6.582402942
H258	4.954393210	2.362653309	-9.872438585
H259	7.199337265	2.322377970	-5.434493259
H260	4.177841728	-0.942139088	-8.408573078
H261	3.748042941	-4.372892227	-3.108490481
H262	5.999546772	-4.734963788	-2.644071830
H263	2.710319176	-2.615086078	-4.648914258
H264	-3.568047084	3.173802405	-7.030165413
H265	8.578109774	-6.586020202	-3.284579143
H266	-3.916874647	0.729021619	-5.524238546
H267	3.975449468	-5.426079460	-4.503652664
H268	5.073152083	1.129766700	-5.731788475
H269	9.359185898	-5.026233028	-2.954602906
H270	3.762577303	-5.022857555	-8.417751685
H271	4.724283190	-1.896221297	-9.748762332
H272	-6.076183154	0.287384220	-3.847874791

H273	-5.272943185	2.797620479	-3.303456372
H274	7.160101569	3.453263005	-9.607226998
H275	-1.114228341	1.340932107	-5.337335918
H276	5.637989604	-3.834990849	-5.468163013
H277	3.005590708	-4.890593015	-6.641874727
H278	1.839283045	-4.106397456	-4.343469424
H279	2.666259285	-1.909242170	-6.972604050
H280	9.137655681	0.965198212	-2.144113960
H281	8.431458765	-0.862194967	-4.718526420
H282	11.160694520	-1.193949554	-5.645030042
H283	9.765693034	-0.616240569	-1.627977261
H284	9.110619566	1.444582959	-6.030920267
H285	-4.102364144	-9.657800427	1.232646218
H286	-5.094294498	-8.640033338	2.293698567
H287	-3.702368384	-7.652514436	6.473399282
H288	-6.221967905	-10.706022298	2.013665564
H289	-7.039855277	-9.654466780	1.060507306
H290	5.678398616	-3.998382825	1.819376979
H291	8.618771143	-3.388883726	1.487572595
H292	7.441088295	-6.452090237	-1.260134439
H293	6.316737173	-2.791746730	0.660036179
H294	9.108291497	-4.943271069	1.611823493
H295	2.107309722	-8.533294955	0.165402219
H296	7.045069992	-5.766987350	0.783993315
H297	-2.779254341	2.300688273	9.307795729
H298	-3.786752215	-0.448357291	10.221410876
H299	0.180529707	-7.602082554	-0.597361609
H300	2.624840599	0.169213958	5.129260199
H301	-1.357192637	-7.820471196	-3.834207950
H302	-3.771751919	-3.012001967	-1.679425905
H303	-0.435477999	-6.343774570	-3.536853850
H304	-1.064180499	2.147919217	7.218591513
H305	-1.464237574	-6.585904088	-1.087027780
H306	-2.215355871	0.997823502	6.526055555
H307	-3.764830458	1.152597101	8.371880658
H308	-1.836331303	-0.526894550	8.517591897
H309	-0.838487783	0.736187118	9.261604105
H310	-4.636936161	-2.566036941	-6.347183825
H311	-4.701343537	2.307472210	10.967832205
H312	-7.710037771	1.696227260	10.842667370
H313	-1.422542966	-3.817464646	-1.537748167
H314	-6.209487273	3.179655079	12.856127303
H315	-7.229277283	3.728850314	11.697617781
H316	-0.487850959	-0.656614244	6.342325071
H317	-0.050229061	-4.003786310	-3.579722730
H318	-4.810768715	-2.602849522	-3.904716933
H319	-6.883194880	-4.046772123	9.849355588
H320	-6.079727223	-5.848688910	7.193545733
H321	-5.142318310	-2.427994432	8.952167357
H322	-8.110305276	-4.490033380	8.990840218
H323	0.732381492	-10.701861724	-0.992084734
H324	-2.553329275	-2.931411120	7.295546429
H325	-5.964918256	0.478202100	5.638815548
H326	-3.190091419	-7.734529619	0.395614617
H327	-7.505410748	-1.155848620	6.367608445
H328	-7.456366114	-6.709116783	2.609530536
H329	-5.682715942	-4.953793664	2.126019695
H330	-7.469109709	1.230989353	12.531660477
H331	1.483586902	-1.117053093	5.361549155
H332	-2.161009436	-2.970869899	9.636084870
H333	-1.073331901	-3.405467489	7.345647430
H334	-2.214798765	-1.401722224	11.414283144
H335	0.670200069	2.774097173	6.367320067
H336	-7.450596019	-4.008271399	5.242772017
H337	-6.904303383	-3.590821840	6.873825324
H338	-7.874951532	-1.631360289	4.700997281
H339	-3.504884188	-5.516356540	-0.527199547
H340	-1.087180610	-3.612348826	-5.832785151
H341	-3.102785584	-2.925785178	-7.183829421
H342	-4.419839409	0.017361047	4.895928606
H343	-3.543001280	-2.961845859	10.350583047
H344	-5.560606932	-2.044496397	10.389617264
H345	2.120451982	2.330919295	5.646858741
H346	-7.320491359	-7.579656994	5.019023433
H347	-4.496209869	-5.245555610	6.716459158
H348	-4.952311035	-6.130888475	-1.382239577
H349	2.781865801	-8.503442796	-1.481166747
H350	3.903406875	-0.483092753	7.748691109
H351	3.736497950	-2.034326211	6.888644763
H352	-5.007637708	-9.331937109	7.474544727

H353	-6.232660397	-8.304502990	7.828038163
H354	-4.768317440	-5.662459398	11.343469716
H355	-5.496227105	-5.953500789	9.199984751
H356	-4.163426135	-8.316782159	11.115954668
H357	-3.147324499	-5.441418022	10.652952347
H358	4.353548654	-3.373603102	9.187646802
H359	11.807305573	3.123286512	-6.715877848
H360	-0.982262523	0.343298264	13.537654729
H361	0.635231652	-0.697470960	11.539777189
H362	9.742399412	2.142770605	-8.717914616
H363	8.903918616	3.954127575	-6.366703281
H364	8.865770382	4.397799511	-8.083641234
H365	-3.017261596	-1.269218610	1.949311390
H366	3.245861791	1.274685847	-8.787070131
H367	-1.268616250	0.470952180	1.210057680
H368	0.400963818	-0.750086599	3.126315134
H369	-1.395575634	0.509451256	2.952327482
H370	-0.966864016	-0.002346874	-1.213128307
Mo371	-3.471639555	-3.618900062	4.176435610
N372	0.023654591	-0.755998793	2.170828167
N373	-1.135475923	-0.004141644	2.106847784
N374	-8.972555796	4.368619685	-2.561535240
N375	-6.435524656	6.674035234	-4.053303469
N376	-5.509145648	8.586908240	-2.215626294
N377	-5.287407114	7.266948340	0.278671198
N378	-3.086057251	5.631262412	-0.335380501
N379	-1.041406714	7.693927548	-0.621578818
N380	-1.050205682	9.246764006	1.841481477
N381	-1.263224408	7.323808187	3.893642162
N382	0.144558770	4.888404823	3.251804990
N383	2.911413186	5.120068162	1.993577650
N384	3.584495454	7.705807270	3.044832076
N385	-6.483958958	3.054969064	11.879273320
N386	-4.725218737	1.291685649	10.831707017
N387	-0.249417394	0.315332675	6.561193731
N388	1.350156247	2.020481115	6.241938022
N389	1.655258507	-0.088161714	5.308908758
N390	-1.951768934	-0.531515052	11.929547567
N391	-5.596742824	0.988467664	-4.445222072
N392	-6.669883440	-0.923288722	-0.230772347
N393	-3.206893248	1.472211105	-5.407599458
N394	-2.629406732	3.511216475	-7.231367179
N395	-1.779590143	5.622416876	-5.572705470
N396	0.630967342	4.694780614	-4.470964291
N397	0.758773304	0.448009914	-4.009858765
N398	-0.534022859	0.646956427	-2.239893312
N399	2.926571911	5.157679060	-5.971486685
N400	3.999185343	5.503768500	-10.585755150
N401	6.054662407	6.326027872	-10.741732325
N402	6.217127068	-2.965552244	7.425455332
N403	8.284627514	-4.338409933	1.682973959
N404	7.860705873	-5.590627419	-1.600172741
N405	6.274953068	-4.535462881	-3.613590230
N406	3.042458280	-2.852889971	-7.189875511
N407	4.149579142	-4.164443208	-8.792903864
N408	4.409662292	-1.866007895	-8.782811194
N409	5.411292275	-1.931119212	-2.734965014
N410	7.881900787	-0.661205209	-2.533119791
N411	9.397400864	-0.550250834	-4.822704798
N412	7.323709998	-2.785194373	-7.295867941
N413	9.846307983	1.449057254	-6.733104933
N414	-6.151208172	-10.155179871	1.155456018
N415	-4.049821513	-7.495177593	-0.107850982
N416	-1.789295340	-6.899573517	-2.022615516
N417	0.704224401	-8.125124913	-1.309167050
N418	-6.578927053	-7.193339212	-4.104229107
N419	-4.980471353	-5.263347608	-5.504643994
N420	-5.491860771	-8.491697261	7.144773570
N421	-5.091428823	-5.278656465	4.174006607
N422	-6.749788212	-6.422730711	3.281521999
N423	-4.597787364	-6.393549691	9.412667318
N424	2.149642193	-0.366017116	-1.228906847
N425	1.607161755	-0.951343217	-0.429742439
O426	-6.365237596	5.506297813	-2.098534752
O427	-3.375368807	7.841266142	-2.494506819
O428	-3.954117529	8.969184229	1.010480715
O429	-2.488122827	5.749291955	1.859730590
O430	0.393847775	6.077225505	0.068470814
O431	1.225288085	9.130820445	1.681384520
O432	-0.354578801	8.823523196	5.380228318

	O433	0.024221651	4.320601596	5.470346862
	O434	3.282201618	3.735346570	3.785543687
	O435	5.544877645	6.744492729	3.665551311
	O436	4.806773075	9.174205772	6.074701789
	O437	-6.107729092	-0.518998610	11.227177866
	O438	-2.213681480	1.744087082	12.116468617
	O439	1.228329992	-1.467125913	13.333618437
	O440	-2.806516670	3.268969201	-4.031378451
	O441	-6.615134972	-0.796849756	-2.501804383
	O442	-0.399013881	3.149944725	-6.910874053
	O443	-0.609926343	0.184155213	-7.691296915
	O444	-1.314629290	6.897591390	-7.425584589
	O445	0.893040741	6.629491591	-3.282935775
	O446	4.212700452	4.868565343	-4.112168950
	O447	5.811837701	7.190274860	-6.301521636
	O448	4.765081956	-1.771984527	10.386171997
	O449	6.590732912	1.063789996	2.398026221
	O450	8.387813046	-3.422890056	-1.131587785
	O451	7.978759048	-4.673783535	-5.146819404
	O452	5.392704267	-1.399267333	-4.960874614
	O453	6.857269779	1.353722122	-2.926978825
	O454	6.362474943	-0.521694596	-0.052240056
	O455	11.267437852	0.077805213	-3.653392774
	O456	11.282813099	0.114351166	-7.895027410
	O457	8.080124361	-0.798817703	-8.137797577
	O458	11.656998879	3.922779543	-8.591120485
	O459	3.673366176	0.980656681	-7.959372340
	O460	-6.333203503	-7.739800269	-0.179072918
	O461	-3.624879215	-6.943228788	-3.393828665
	O462	1.053738783	-8.654712241	-3.495095382
	O463	2.737179185	-11.080468982	-0.919413552
	O464	-6.959351307	-4.501002108	-6.342525469
	O465	-8.378441966	-3.501338746	-0.528219847
	O466	-7.228745686	-5.264700330	0.214288530
	O467	-2.121503102	-5.267367374	-7.658053554
	O468	-2.814224941	-7.654724038	8.811515015
	O469	-2.715096263	-7.459414286	12.270801558
	O470	-5.892494423	-0.617956563	2.540787709
	O471	-6.350356836	1.462148925	3.304703817
	O472	-9.199737421	-4.062700912	7.700620384
	O473	-9.968890184	-2.999902149	5.858852233
	O474	-4.041610461	-3.039022627	6.221426910
	O475	-5.064092183	-1.503594130	7.507974498
	O476	-5.389123100	-2.456608077	4.076267302
	O477	-2.564572543	-2.927088692	10.539071688
	O478	-1.721659804	-2.860010882	7.830125445
	O479	1.845993220	-0.319623098	-6.585734701
	O480	-7.571304089	-4.747356626	9.799291681
	O481	-5.248622343	-2.822006226	9.862734248
	S482	4.737628465	-4.294376428	-0.410902493
	S483	1.374787075	-3.824289259	-0.546305068
	S484	-3.401241255	-3.384031558	1.793839451
	S485	2.773080438	-2.115441321	2.010355506
	S486	-1.289233494	-1.483460263	-0.306966445
	S487	-1.112622676	-6.483201568	1.115871440
	S488	-2.079314514	-1.656203895	4.347091112
	S489	2.302126541	-5.619864422	2.403057050
	S490	-1.869093782	-5.307234206	4.555677950
	S491	1.213562531	-3.305683797	5.306125639
	end			

product

	bm612n2xhisto2b135th.car_5			
Fe(139) -2.385	C1	-7.002072531	5.875307292	-3.108699772
Fe(140) 0.021	C2	-8.457658077	5.462180029	-3.357512268
Fe(141) -2.568	C3	-5.046708997	7.037063936	-4.042897078
Fe(142) 2.661	C4	-4.594542847	7.890823674	-2.863578032
Fe(143) 1.651	C5	-5.207125822	9.388927846	-1.035406592
Fe(144) 2.467	C6	-4.758924252	8.540500748	0.159402679
Fe(145) -0.020	C7	-4.756844649	6.356775044	1.246417458
	C8	-3.319983187	5.918679873	0.940466066
	C9	-1.703977101	5.399539904	-0.848420558
	C10	-0.669083097	6.456303704	-0.438672835
	C11	-0.051132558	8.791500518	-0.363156152
	C12	0.114015475	9.079925408	1.134244805
	C13	-1.013308750	9.675504143	3.221481862
	C14	-0.814362847	8.569133540	4.262971830
	C15	-1.300277464	6.227542178	4.839094746
	C16	-0.344495631	5.065936936	4.574796353
	C17	0.997487542	3.804289507	2.894617701

C18	2.478105426	4.240336541	2.981329231
C19	0.620492264	3.276803043	1.502902657
C20	1.540874226	2.122475619	1.099002391
C21	-0.869187371	2.930478888	1.485330821
C22	4.248593889	5.641304209	1.949722156
C23	4.509964263	6.754839502	2.975585187
C24	4.575700279	6.095314885	0.502432049
C25	5.948443608	6.775279221	0.416777643
C26	4.505125238	4.916332345	-0.475284212
C27	3.803203498	8.865449806	3.940404101
C28	4.134176989	8.464863820	5.363691050
C29	-6.945908713	1.675744580	11.676071284
C30	-5.854020487	0.703236181	11.221782507
C31	-3.497825800	0.607389610	10.392966934
C32	-2.478619300	0.670276598	11.548361983
C33	-2.947794453	1.248206881	9.104133322
C34	-1.678817869	0.573290273	8.572084571
C35	-1.283146625	1.122277429	7.196505130
C36	0.922500370	0.717429110	6.029211980
C37	-1.019041467	-0.632189746	13.020869136
C38	0.398651986	-0.949330987	12.595938331
C39	-4.787613226	1.866519924	-3.603337998
C40	-3.527645725	2.304612250	-4.374049686
C41	-4.341837575	1.134237798	-2.317383599
C42	-5.434955434	0.924141877	-1.249265947
C43	-6.274663049	-0.346089382	-1.386138622
C44	-1.924476546	1.457093378	-6.071551008
C45	-1.582463708	2.764738498	-6.793893588
C46	-1.904493938	0.250394034	-7.022664410
C47	-2.437842073	4.803723579	-7.844123682
C48	-1.790267356	5.859340236	-6.940466740
C49	-1.063244356	6.478411112	-4.687334240
C50	0.244054154	5.933144785	-4.109475257
C51	1.915641976	4.146998374	-4.022841293
C52	3.136382443	4.801521560	-4.699802715
C53	2.022372973	2.631773239	-4.278575361
C54	0.993801373	1.779909009	-3.599909018
C55	0.102613061	1.974842593	-2.563115030
C56	-0.187949440	-0.059509185	-3.201815738
C57	4.017666287	5.651592161	-6.832923270
C58	4.645031296	6.895084621	-6.207621133
C59	3.420364430	5.966789854	-8.224152078
C60	4.387745644	6.035017672	-9.368233864
C61	5.665561991	6.554152617	-9.445997921
C62	5.052582710	5.717665663	-11.404191344
C63	5.526575300	-1.853444026	8.086949366
C64	4.819367980	-2.385630907	9.318240371
C65	4.438974999	-1.240542781	7.165458938
C66	5.018008193	-0.616035582	5.918235967
C67	5.428036881	0.729584433	5.898018867
C68	5.169582080	-1.368207084	4.739091681
C69	5.954364728	1.311332355	4.739700879
C70	5.706539155	-0.805848799	3.580669134
C71	6.088390469	0.537541063	3.579685340
C72	7.311747550	-4.724221174	0.640349234
C73	7.902385067	-4.517652737	-0.767005299
C74	6.067211317	-3.862342062	0.801449223
C75	8.399223604	-5.557253345	-2.953284530
C76	7.526585798	-4.877789853	-4.012271677
C77	5.345979710	-3.752877713	-4.428246520
C78	5.386426459	-2.246721688	-4.061276789
C79	3.944338252	-4.367277129	-4.209555700
C80	2.730584355	-3.700878598	-4.866716310
C81	2.532378783	-3.950083432	-6.371843021
C82	3.846968379	-2.977280557	-8.264098117
C83	5.447747936	-0.523912154	-2.325349819
C84	6.785666705	0.157115819	-2.643881996
C85	5.179589505	-0.332482914	-0.816686631
C86	9.209705346	-0.080484304	-2.425521390
C87	10.066404765	-0.174471935	-3.678109622
C88	10.176815309	-0.889628158	-6.003099165
C89	10.477685324	0.270869856	-6.984513598
C90	9.615383205	-2.096674994	-6.761429843
C91	8.286021318	-1.823192968	-7.451085701
C92	9.903341510	2.548097926	-7.707104192
C93	11.250503505	3.253755701	-7.680124160
C94	8.767051012	3.562243861	-7.384448638
C95	7.388692224	2.950959925	-7.513944075
C96	6.724105589	2.952222641	-8.751033501
C97	6.753220520	2.324082276	-6.428414799

	C98	5.482131629	2.333096066	-8.910037738
	C99	5.522344343	1.677377538	-6.573673132
	C100	4.899759598	1.683622925	-7.820152339
	C101	-5.073967337	-9.160322022	1.327582846
	C102	-5.206675046	-8.078837648	0.270242809
	C103	-3.950833782	-6.396233529	-1.057380768
	C104	-3.116653958	-6.784694473	-2.284437007
	C105	-0.838862689	-7.284733197	-3.065140540
	C106	0.367062236	-8.107682189	-2.622768376
	C107	1.904687986	-8.814607700	-0.874571245
	C108	1.792785605	-10.322510287	-0.981381178
	C109	-7.076637710	-5.829373212	-4.315082022
	C110	-6.344606447	-5.149842748	-5.477385792
	C111	-7.085139970	-4.902707658	-3.078882146
	C112	-8.128248949	-5.366766384	-2.045129617
	C113	-7.948298643	-4.630238155	-0.736636578
	C114	-4.219148329	-4.688557869	-6.586038180
	C115	-3.123968381	-5.633585057	-7.039844793
	C116	-3.755801800	-3.228848119	-6.348531957
	C117	-3.023323259	-3.154645154	-5.042989953
	C118	-1.667498158	-3.508132795	-4.967821672
	C119	-3.747792592	-2.962088988	-3.852295527
	C120	-1.082402558	-3.773691267	-3.726755288
	C121	-3.160419782	-3.230113706	-2.616511759
	C122	-1.840789054	-3.690635553	-2.559544701
	C123	-4.560055490	-7.372066403	7.120183564
	C124	-3.918363691	-7.143438304	8.495962604
	C125	-5.192512412	-6.084071028	6.574876459
	C126	-5.621997998	-6.119508275	5.143786669
	C127	-6.656730189	-6.834985904	4.578728833
	C128	-5.800010463	-5.470653088	3.058967288
	C129	-3.991235933	-6.131651592	10.694991206
	C130	-3.575145933	-7.407272412	11.392624945
	C131	-5.961443548	0.279924358	3.480913688
	C132	-5.487595423	-0.158474908	4.881522417
	C133	-5.754296476	-1.645816872	5.234262575
	C134	-7.240299568	-1.871882102	5.601452913
	C135	-7.568694226	-3.284745150	6.089851598
	C136	-9.015759237	-3.448776766	6.603093169
	C137	-4.887964891	-2.066055326	6.430284087
	C138	-0.220772026	-3.468618099	2.277636834
	Fe139	-1.737431411	-4.745451861	2.377419474
	Fe140	0.739808553	-2.081422061	0.811885999
	Fe141	1.575267528	-3.658609825	3.147081811
	Fe142	0.486364025	-4.960797843	1.213974557
	Fe143	3.016311760	-4.139050859	1.004221289
	Fe144	-0.698812766	-3.424587607	4.253396295
	Fe145	-1.649832991	-1.840674928	2.114726678
	H146	-8.810058854	4.584352086	-1.550933186
	H147	-8.424161666	3.520103233	-2.724794166
	H148	-6.986396004	6.868547540	-4.908776922
	H149	-8.581427102	5.207820567	-4.422417584
	H150	-9.078648602	6.358123310	-3.185639103
	H151	-4.427521281	6.127157497	-4.023933630
	H152	-6.498580192	8.571335400	-2.550535571
	H153	-4.811340037	7.580354802	-4.967339197
	H154	-6.088594405	9.974792076	-0.744179759
	H155	-5.863165732	6.920944795	-0.498305477
	H156	-4.385890074	10.088154039	-1.243098857
	H157	-1.349220902	4.435497547	-0.474284313
	H158	-3.790759821	5.808485654	-1.047072799
	H159	-5.410655935	5.474248634	1.276720892
	H160	0.934323051	8.502993297	-0.749649237
	H161	-1.871382314	7.959638162	-1.175033690
	H162	-4.753458326	6.818509588	2.240776573
	H163	-1.757665601	5.333958237	-1.942358477
	H164	-1.944422038	9.181926067	1.357121665
	H165	-0.375932501	9.712355181	-0.866534428
	H166	-1.484212299	3.835352161	1.522821872
	H167	-1.138748659	2.369134811	0.577156094
	H168	-1.152616995	2.348912443	2.369990934
	H169	0.768843603	4.092353024	0.778664468
	H170	-1.965987001	10.174231451	3.450845200
	H171	3.529928539	4.409385069	-0.451652461
	H172	2.746932158	7.752180901	2.470402545
	H173	6.007987504	7.679622333	1.038833097
	H174	1.297348142	1.784617367	0.084255012
	H175	1.457993586	1.256461870	1.771921802
	H176	6.195814309	-1.034677953	8.413253035
	H177	4.670432144	5.262728549	-1.505458940

H178	6.150329083	7.063937840	-0.625398722
H179	5.282960205	4.171989523	-0.240957827
H180	6.746333245	6.088510087	0.740306116
H181	3.811229650	6.838946602	0.208844382
H182	4.926153067	4.823269956	2.236547199
H183	2.198881096	5.466609862	1.347693696
H184	2.596852860	2.433784018	1.093215701
H185	4.389175758	-1.028179548	-0.497713382
H186	4.811614323	0.695965532	-0.686577202
H187	6.340450249	0.055420528	0.725953879
H188	6.768217940	2.005340897	2.505719232
H189	5.793615372	-1.394442611	2.667982883
H190	4.831552285	-2.406283124	4.713692832
H191	5.319012422	1.342618146	6.796314772
H192	-0.195637526	5.565593365	2.597166438
H193	-2.312070008	5.792715522	4.859061577
H194	-1.073592966	6.607789400	5.842235977
H195	-1.770257553	7.212185060	3.025842769
H196	-0.201518482	10.398596725	3.374198023
H197	6.240921424	2.366266949	4.734884655
H198	0.880070622	3.007402726	3.639742899
H199	4.611643834	9.511681561	3.560944011
H200	2.877890968	9.461072383	3.968644365
H201	3.699781038	7.493706803	5.696749663
H202	-1.356671872	-1.385151560	13.749850564
H203	-7.244647129	-1.765612930	-0.293406243
H204	-5.539730024	-1.779202953	3.267488036
H205	-9.136759997	-5.151197660	-2.426046080
H206	-8.046394582	-6.451207477	-1.881958043
H207	-7.315034805	-3.868162803	-3.377726419
H208	-3.302030965	-6.705415003	-6.785340609
H209	-8.114131479	-5.904055363	-4.675220671
H210	-3.894071992	0.168741583	-2.601200605
H211	-6.137939152	1.775439381	-1.257080446
H212	-4.502266201	-5.795832380	-4.767228383
H213	-4.911821761	-4.660573083	-7.452973031
H214	-5.678380887	-7.213870742	-3.647491872
H215	-6.340106684	-0.630114143	0.702531160
H216	-6.077576201	-4.885314750	-2.636346872
H217	-7.242700201	-7.703628871	-3.494595963
H218	-6.330188162	1.554052075	-4.940937232
H219	-3.533894132	1.725638048	-1.864471429
H220	-6.934196991	-6.180297999	-0.079002368
H221	-4.969608337	0.933822567	-0.252821825
H222	2.008451523	4.365311418	-2.950356252
H223	-0.047786644	2.886143584	-1.994681689
H224	-0.807167506	7.398056421	-5.231712777
H225	-2.202608211	4.751171861	-5.224537994
H226	1.989268709	5.147173837	-6.368092221
H227	6.308996190	7.020223198	-8.707154193
H228	3.912405936	7.537619716	-5.661316121
H229	2.820975871	6.892009128	-8.157863083
H230	4.792387299	4.873925058	-6.920071558
H231	5.115082115	5.434700641	-12.451583381
H232	3.042155437	2.336322604	-3.972676931
H233	1.976472440	2.464587700	-5.368763567
H234	6.974594444	6.596715411	-11.136711735
H235	2.710895368	5.158866207	-8.464106598
H236	0.106016702	4.161860256	-5.189592613
H237	-1.704531317	6.781412388	-3.845710731
H238	-1.792788225	4.734383987	-8.731199141
H239	-0.513537204	-1.093304926	-3.288668324
H240	-3.414356594	5.182950996	-8.172300746
H241	7.063252791	-3.201623623	7.928008316
H242	6.507043764	-2.669874576	6.485201705
H243	5.222552202	-2.648192203	-2.011832886
H244	4.675709487	0.027436531	-2.881295808
H245	7.750579980	-1.575914065	-2.126042190
H246	1.271898739	0.035467917	-4.797776371
H247	0.963491207	-0.129595899	-7.004109612
H248	-0.730019165	-0.456807303	-8.422110990
H249	2.473845172	0.354292494	-6.952466345
H250	-2.099934897	-0.656729538	-6.427216719
H251	-2.699445443	0.364003681	-7.776730709
H252	9.556474598	-2.962656145	-6.087189624
H253	10.341448295	-2.339143489	-7.552711409
H254	7.469627171	-3.513132670	-6.578349609
H255	6.415536172	-2.544091847	-7.606285537
H256	4.944458035	-4.261859494	-9.429253688
H257	1.460671525	-4.059432346	-6.595944340

	H258	4.975870784	2.354902389	-9.879108973
	H259	7.206767573	2.335911039	-5.433787884
	H260	4.209324876	-0.952218979	-8.400441099
	H261	3.758212056	-4.376583074	-3.123263500
	H262	6.009743876	-4.727879704	-2.652986345
	H263	2.714151759	-2.618438260	-4.663876513
	H264	-3.568626991	3.167852228	-7.035305587
	H265	8.591547399	-6.585468039	-3.287711012
	H266	-3.921411809	0.726429652	-5.517871572
	H267	3.992969987	-5.423700014	-4.521539355
	H268	5.075835170	1.150495140	-5.730225762
	H269	9.358425432	-5.019535684	-2.952420386
	H270	3.719615410	-5.023982265	-8.471788498
	H271	4.725183396	-1.893323322	-9.759525224
	H272	-6.102801374	0.304255654	-3.852386468
	H273	-5.287907349	2.806055847	-3.301320093
	H274	7.180006435	3.446879025	-9.611688790
	H275	-1.127516635	1.331634753	-5.324106416
	H276	5.652134362	-3.821515902	-5.476031048
	H277	3.012517889	-4.896022409	-6.655555203
	H278	1.850259058	-4.114160854	-4.357784900
	H279	2.679842302	-1.912230144	-6.983817792
	H280	9.126322835	0.983958436	-2.158308677
	H281	8.441425687	-0.833966366	-4.727726501
	H282	11.176522178	-1.176327806	-5.640728760
	H283	9.759703662	-0.588644288	-1.622082440
	H284	9.138835116	1.458291711	-6.028956521
	H285	-4.093662985	-9.652131894	1.240135938
	H286	-5.080533571	-8.630720140	2.302464085
	H287	-3.729071461	-7.673617945	6.464909612
	H288	-6.207974439	-10.699405863	2.038471115
	H289	-7.032097825	-9.652661925	1.085328686
	H290	5.687431505	-4.002645666	1.823394611
	H291	8.622559025	-3.417652723	1.503439942
	H292	7.436897723	-6.457914226	-1.271025902
	H293	6.324924222	-2.802699041	0.656975186
	H294	9.105737193	-4.975293275	1.614863392
	H295	2.071887401	-8.563580214	0.185236548
	H296	7.037989879	-5.782354344	0.777951315
	H297	-2.761071810	2.318183699	9.296150891
	H298	-3.770151169	-0.436363195	10.191864567
	H299	0.177520420	-7.596976165	-0.602006414
	H300	2.613132865	0.084216402	5.081863908
	H301	-1.362699008	-7.857874569	-3.839853112
	H302	-3.726517125	-3.083426469	-1.694903193
	H303	-0.439615067	-6.379910081	-3.555626991
	H304	-0.949524490	2.163632582	7.273325340
	H305	-1.471367437	-6.592538049	-1.108600347
	H306	-2.157090303	1.107272392	6.524287975
	H307	-3.748282026	1.179081960	8.350506974
	H308	-1.845739595	-0.510499730	8.481356529
	H309	-0.838155364	0.715863218	9.270153893
	H310	-4.649321441	-2.587841232	-6.347726655
	H311	-4.695491667	2.312008169	10.949543660
	H312	-7.702590651	1.673426239	10.876594670
	H313	-1.385424278	-3.947336507	-1.606141686
	H314	-6.182754473	3.183851824	12.852957909
	H315	-7.216628843	3.715414906	11.698745792
	H316	-0.505085448	-0.656788156	6.364886752
	H317	-0.034170266	-4.064831327	-3.655479886
	H318	-4.786140709	-2.625241617	-3.898374129
	H319	-6.861932207	-4.057264563	9.857003626
	H320	-6.076254994	-5.830731461	7.185089020
	H321	-5.121250702	-2.429309218	8.951004740
	H322	-8.086928306	-4.503138192	8.998276876
	H323	0.747331827	-10.712486022	-1.034204551
	H324	-2.523597709	-2.935645311	7.293629344
	H325	-5.939040186	0.491692231	5.640971425
	H326	-3.188627205	-7.745178305	0.381451806
	H327	-7.482327811	-1.147003659	6.394173291
	H328	-7.455311970	-6.692930511	2.595733862
	H329	-5.677170160	-4.944394047	2.120655209
	H330	-7.419659555	1.219904654	12.561972801
	H331	1.484076056	-1.193612451	5.409744804
	H332	-2.145830825	-2.963071501	9.624816004
	H333	-1.045255431	-3.416397277	7.359902630
	H334	-2.206363794	-1.393091181	11.394074033
	H335	0.703328587	2.761336759	6.206613957
	H336	-7.424732784	-3.996321474	5.261388123
	H337	-6.879762776	-3.581214643	6.893138636

H338	-7.861668537	-1.618590934	4.728653413
H339	-3.505554140	-5.526015401	-0.550443419
H340	-1.091873872	-3.625356391	-5.888699035
H341	-3.122925317	-2.943595787	-7.203098202
H342	-4.399145154	0.019511103	4.896263697
H343	-3.526098262	-2.962682168	10.343952565
H344	-5.539861856	-2.047678476	10.386676995
H345	2.186069146	2.243837943	5.596553675
H346	-7.329644049	-7.569909067	5.004128373
H347	-4.485722962	-5.254831447	6.704049865
H348	-4.958193689	-6.144692627	-1.393180735
H349	2.785797591	-8.477092208	-1.441295698
H350	3.894496603	-0.484073690	7.750777063
H351	3.726604695	-2.035934277	6.892114201
H352	-5.060111407	-9.330214247	7.471416457
H353	-6.267131780	-8.281321219	7.825469051
H354	-4.750882164	-5.646277226	11.328351663
H355	-5.499478999	-5.952774184	9.197073985
H356	-4.173811567	-8.307900121	11.115020819
H357	-3.131051247	-5.446684492	10.628143893
H358	4.348028253	-3.387948198	9.174955996
H359	11.836602844	3.115309087	-6.740316569
H360	-0.991968881	0.347262194	13.530219324
H361	0.649131396	-0.678726563	11.540985420
H362	9.753867110	2.149985979	-8.723826783
H363	8.919394405	3.954168505	-6.366645400
H364	8.877902399	4.405798606	-8.081133379
H365	-3.044250544	-1.289421069	2.017833839
H366	3.266555765	1.266125569	-8.795301225
H367	-1.178214804	0.484965911	1.232674848
H368	0.417402269	-0.810247487	3.161910667
H369	-1.332264023	0.509137453	2.948295932
H370	-1.303324867	-0.525001913	-0.643938861
Mo371	-3.471433447	-3.630833029	4.215447950
N372	0.044064120	-0.817484000	2.203748848
N373	-1.093734929	-0.027545771	2.111812065
N374	-8.961182313	4.370983453	-2.540084727
N375	-6.461346240	6.699959914	-4.054425531
N376	-5.536618710	8.620850607	-2.224287234
N377	-5.276895151	7.288535888	0.254704862
N378	-3.049054177	5.680929494	-0.363772976
N379	-1.018128800	7.745616279	-0.653959378
N380	-1.040281982	9.256245938	1.830816055
N381	-1.266884674	7.335745140	3.907016599
N382	0.112803152	4.905545973	3.309714546
N383	2.877943583	5.135913895	2.034202360
N384	3.562943129	7.722830071	3.081652213
N385	-6.465189788	3.048223240	11.879811792
N386	-4.713844813	1.296300384	10.810706191
N387	-0.231307547	0.308279229	6.572329346
N388	1.374293175	1.984629768	6.160124997
N389	1.655928564	-0.168405644	5.320169473
N390	-1.943716914	-0.522736432	11.909209336
N391	-5.622537782	1.003820076	-4.448924207
N392	-6.652707219	-0.931994901	-0.235399044
N393	-3.220393311	1.477688580	-5.409180362
N394	-2.629005616	3.494114676	-7.249223324
N395	-1.781201094	5.611190046	-5.602118379
N396	0.652224930	4.694410565	-4.506910096
N397	0.788703240	0.471930431	-3.997378821
N398	-0.629479200	0.830647441	-2.321029207
N399	2.929526824	5.192129709	-5.983904533
N400	4.016222350	5.516706993	-10.598955301
N401	6.071790298	6.340550787	-10.746435585
N402	6.208385838	-2.964340546	7.418993144
N403	8.284271061	-4.367750384	1.688542666
N404	7.858248790	-5.595310462	-1.606013548
N405	6.284536538	-4.528294546	-3.621982621
N406	3.055672198	-2.858748274	-7.200730641
N407	4.138232507	-4.168921333	-8.819284654
N408	4.431089251	-1.873817440	-8.786775575
N409	5.413598531	-1.929607234	-2.735976139
N410	7.881690669	-0.650322913	-2.542203811
N411	9.411211390	-0.531556028	-4.825058557
N412	7.355936647	-2.809036661	-7.319283403
N413	9.866228186	1.456553743	-6.739707804
N414	-6.142563339	-10.152385428	1.177426727
N415	-4.051126818	-7.501129814	-0.113831587
N416	-1.796870303	-6.918612430	-2.037424592
N417	0.696698119	-8.132505237	-1.306450009

N418	-6.596659745	-7.202282833	-4.107906145
N419	-4.988524687	-5.282856266	-5.509530091
N420	-5.530545206	-8.482315565	7.141642323
N421	-5.089350120	-5.277335558	4.170180239
N422	-6.749464126	-6.411532481	3.270690248
N423	-4.597931010	-6.391181228	9.401881675
N424	2.081187958	-0.366842126	-1.143684690
N425	1.552368808	-0.993293176	-0.369093406
O426	-6.362173640	5.531615524	-2.100384217
O427	-3.398427029	7.892241919	-2.520871458
O428	-3.968616082	9.006616216	0.996532193
O429	-2.464392729	5.797486247	1.835768996
O430	0.425040229	6.140211489	0.056611029
O431	1.235944353	9.184645557	1.655493877
O432	-0.313560531	8.835855674	5.363582406
O433	-0.082396936	4.297717268	5.518543522
O434	3.232965016	3.782410895	3.852104251
O435	5.548428883	6.781556300	3.655412606
O436	4.774027555	9.176683349	6.123433198
O437	-6.074234736	-0.525177769	11.233299034
O438	-2.201078574	1.753367054	12.094899317
O439	1.230401491	-1.444993983	13.339753431
O440	-2.863730371	3.314705746	-4.070270992
O441	-6.617220132	-0.796752817	-2.505614070
O442	-0.400279649	3.127938226	-6.925158290
O443	-0.623226559	0.160390630	-7.676449346
O444	-1.319477452	6.882410007	-7.459082990
O445	0.888229461	6.627399413	-3.311649001
O446	4.239074912	4.873499387	-4.146699659
O447	5.820030228	7.206289924	-6.309046943
O448	4.738430467	-1.787648515	10.381618370
O449	6.579649614	1.055565777	2.395580029
O450	8.394085677	-3.432683108	-1.122361302
O451	7.983216747	-4.695892704	-5.158973314
O452	5.401944414	-1.389596959	-4.959559906
O453	6.845118455	1.364104354	-2.909099070
O454	6.370814505	-0.541512189	-0.048854343
O455	11.278485689	0.071887508	-3.637262619
O456	11.285738596	0.111016129	-7.910719222
O457	8.093091198	-0.799779514	-8.122609306
O458	11.658779823	3.947777197	-8.598306214
O459	3.689229532	0.980983251	-7.962124013
O460	-6.335301974	-7.741397458	-0.164895683
O461	-3.636880087	-6.984984934	-3.401966961
O462	1.043934424	-8.696620540	-3.483062777
O463	2.755350850	-11.073087466	-0.979304048
O464	-6.962690824	-4.494799351	-6.333812214
O465	-8.378586604	-3.501577533	-0.530203983
O466	-7.226060205	-5.263196585	0.213007314
O467	-2.140143660	-5.291442305	-7.679118264
O468	-2.827788533	-7.668991475	8.796037212
O469	-2.710965497	-7.457884106	12.257522200
O470	-5.890641665	-0.635112691	2.553198888
O471	-6.313663816	1.459984176	3.293916910
O472	-9.175909839	-4.065167473	7.715532020
O473	-9.944811859	-2.989608601	5.881105876
O474	-4.044920134	-3.048522217	6.248523771
O475	-5.037667223	-1.482775503	7.521846549
O476	-5.376560847	-2.455265455	4.099194968
O477	-2.547148635	-2.923534808	10.528634115
O478	-1.690886572	-2.850083764	7.822822072
O479	1.852392781	-0.326086780	-6.607687280
O480	-7.543716954	-4.763652022	9.803177741
O481	-5.231048619	-2.826285315	9.858971635
S482	4.741094448	-4.310310697	-0.402620954
S483	1.411178619	-3.886915455	-0.525698908
S484	-3.430242939	-3.391147714	1.847106868
S485	2.783654050	-2.171636154	2.027089213
S486	-1.349439353	-1.824431239	-0.154623206
S487	-1.137387226	-6.466547660	1.112274900
S488	-2.075448742	-1.663972623	4.407951338
S489	2.282850582	-5.669485838	2.426825859
S490	-1.864416134	-5.318885395	4.566254195
S491	1.219628660	-3.373571953	5.325900588
end			

S2BH-Fe2-brNH-NH2-Fe6H to S2BH-Fe2-brNH-Fe6NH3

35, S=1/2

reactant

	bm612n2xnewbrk2bh135tj_4_53421.05		
C1	-6.989498965	5.850879822	-3.111344080
C2	-8.448121595	5.458353747	-3.373314886
C3	-5.008625371	6.985077583	-4.023722912
C4	-4.561639833	7.841341649	-2.844639934
C5	-5.174702005	9.353283059	-1.029248915
C6	-4.745933639	8.506274534	0.173786830
C7	-4.787670560	6.328926249	1.277385221
C8	-3.356668240	5.866021223	0.973487086
C9	-1.750664313	5.334749497	-0.816017498
C10	-0.710465846	6.387994786	-0.407317901
C11	-0.053508785	8.707052054	-0.330028852
C12	0.104724643	9.026694009	1.161631197
C13	-1.028128909	9.669167457	3.233201731
C14	-0.842588802	8.558672655	4.272019478
C15	-1.258521152	6.194672896	4.796571696
C16	-0.275164245	5.063724650	4.507908196
C17	1.044566599	3.781798054	2.836706431
C18	2.525721746	4.205357635	2.913917226
C19	0.682951386	3.261010766	1.432660422
C20	1.624981737	2.142154769	0.981858010
C21	-0.789184883	2.859957650	1.424012421
C22	4.281038141	5.631503442	1.900712679
C23	4.525776990	6.724836118	2.948800842
C24	4.612214199	6.119046010	0.465884411
C25	5.986816438	6.799189045	0.407660408
C26	4.542664899	4.961304797	-0.535867772
C27	3.829706370	8.845896218	3.887373795
C28	4.168259140	8.461119142	5.312536511
C29	-6.976138235	1.690873081	11.650287671
C30	-5.881673411	0.708677254	11.219557343
C31	-3.513038289	0.594966981	10.420764310
C32	-2.492096900	0.660835654	11.573782405
C33	-2.963519067	1.226059586	9.125163748
C34	-1.691546841	0.558234766	8.585377011
C35	-1.346539118	1.078240771	7.183727386
C36	0.900533981	0.777374874	6.048046479
C37	-1.017165172	-0.638885845	13.034227796
C38	0.392569310	-0.968502312	12.594395671
C39	-4.763457107	1.852606109	-3.597254950
C40	-3.498002882	2.290498959	-4.360097307
C41	-4.332044103	1.125088472	-2.302387846
C42	-5.437078344	0.924111314	-1.243672106
C43	-6.281113813	-0.343659047	-1.381559133
C44	-1.904950060	1.468354710	-6.077547949
C45	-1.575092052	2.784079631	-6.791751536
C46	-1.881390364	0.266477297	-7.034580315
C47	-2.447661821	4.829678555	-7.814630803
C48	-1.787605147	5.875280041	-6.907417470
C49	-1.060937757	6.483426137	-4.649994497
C50	0.248653733	5.938096337	-4.078748104
C51	1.913239105	4.144346351	-4.002752362
C52	3.131778812	4.801526192	-4.679370615
C53	2.014467258	2.635556972	-4.276982966
C54	0.987565828	1.788822143	-3.600244392
C55	0.098712046	1.994482097	-2.565010907
C56	-0.196132065	-0.044728103	-3.186765149
C57	4.015462937	5.628338257	-6.820248816
C58	4.638580670	6.874586222	-6.196868625
C59	3.413764419	5.948406792	-8.207812827
C60	4.376058991	6.017455970	-9.355173428
C61	5.654129481	6.534719663	-9.438425468
C62	5.028093427	5.705718741	-11.395872483
C63	5.539723154	-1.847782800	8.085476353
C64	4.836411818	-2.369755206	9.323685683
C65	4.453012718	-1.237826365	7.162247054
C66	5.033311500	-0.611732236	5.916313741
C67	5.449792797	0.732252538	5.900658719
C68	5.180955413	-1.360414205	4.734574783
C69	5.976696377	1.316079896	4.743786645
C70	5.718465669	-0.795774918	3.577614798
C71	6.104627684	0.546419630	3.580573596
C72	7.316476968	-4.704502284	0.639926736
C73	7.901591917	-4.504350772	-0.770384721

C74	6.066043302	-3.851525242	0.799697142
C75	8.395758169	-5.554636060	-2.951402274
C76	7.521773738	-4.868537456	-4.004821620
C77	5.335687602	-3.759939013	-4.421843869
C78	5.382696035	-2.252097416	-4.061924281
C79	3.931896096	-4.366899675	-4.194427983
C80	2.720692556	-3.696777473	-4.852563187
C81	2.524225737	-3.948386053	-6.357672307
C82	3.843730517	-2.973371023	-8.247822374
C83	5.450526932	-0.520001839	-2.337084617
C84	6.793858565	0.151581964	-2.651820288
C85	5.171797727	-0.315310774	-0.833089805
C86	9.214789830	-0.098914571	-2.424412967
C87	10.058227425	-0.183702122	-3.685807781
C88	10.160206834	-0.901681158	-6.006512773
C89	10.465966849	0.268415696	-6.975022814
C90	9.597514054	-2.097882556	-6.781650865
C91	8.264720549	-1.813359983	-7.459407387
C92	9.886003643	2.540485553	-7.701585825
C93	11.233088577	3.245039688	-7.665399998
C94	8.751021764	3.558225772	-7.385889861
C95	7.372601153	2.947703558	-7.515098300
C96	6.703134938	2.956481844	-8.749484195
C97	6.742532499	2.313799480	-6.430569496
C98	5.460858848	2.337580804	-8.906691554
C99	5.512887343	1.664937356	-6.574903011
C100	4.884611602	1.680415473	-7.818280783
C101	-5.084411979	-9.164853320	1.311386694
C102	-5.208656484	-8.077041201	0.259019586
C103	-3.945699269	-6.380376216	-1.043179515
C104	-3.104641289	-6.754361633	-2.270499495
C105	-0.829691940	-7.248115148	-3.057213062
C106	0.379406067	-8.075834022	-2.631869886
C107	1.915449280	-8.812561516	-0.889940726
C108	1.780535865	-10.322325491	-0.938283336
C109	-7.065607270	-5.822551599	-4.317259042
C110	-6.334687725	-5.141142793	-5.479746421
C111	-7.081618063	-4.892749401	-3.082344609
C112	-8.123033744	-5.360477191	-2.048102432
C113	-7.946310790	-4.627049702	-0.737004853
C114	-4.206735737	-4.662764413	-6.579959916
C115	-3.107373235	-5.605972041	-7.027413448
C116	-3.745224985	-3.202471884	-6.342211337
C117	-3.030250884	-3.119593657	-5.027568442
C118	-1.674670229	-3.470023238	-4.931009184
C119	-3.771704570	-2.915656028	-3.849555736
C120	-1.106747123	-3.726953087	-3.680773222
C121	-3.202752055	-3.176870088	-2.603997711
C122	-1.886157059	-3.642887272	-2.527657375
C123	-4.535037172	-7.363432669	7.127054365
C124	-3.902633401	-7.132316782	8.506542817
C125	-5.191020222	-6.085270461	6.584832104
C126	-5.620442100	-6.123482712	5.153250922
C127	-6.652265386	-6.846710073	4.592275622
C128	-5.807364233	-5.479406234	3.068131627
C129	-4.002388852	-6.135504129	10.711543790
C130	-3.575382198	-7.411722981	11.401837487
C131	-5.980843454	0.284430868	3.476247177
C132	-5.512770915	-0.161748094	4.875784806
C133	-5.776845657	-1.652856460	5.215581493
C134	-7.265933603	-1.882069730	5.572315718
C135	-7.598981914	-3.293358428	6.064066469
C136	-9.046574102	-3.454996621	6.578951239
C137	-4.914400966	-2.071913560	6.414378510
C138	-0.218855126	-3.489378941	2.294721655
Fe139	-1.728180848	-4.766533933	2.378257551
Fe140	0.750423689	-2.023802074	0.775113188
Fe141	1.586004157	-3.614083569	3.130683405
Fe142	0.503821045	-4.955192830	1.212371969
Fe143	3.022822667	-4.091963854	0.995240730
Fe144	-0.691336102	-3.437064032	4.252507597
Fe145	-1.647329428	-1.852130515	2.102467145
H146	-8.832052228	4.571525837	-1.575694000
H147	-8.450203699	3.510531109	-2.754247164
H148	-6.941465576	6.845741435	-4.911022331
H149	-8.564231164	5.212490897	-4.440836366
H150	-9.057474661	6.362175082	-3.200581291
H151	-4.402992088	6.066662082	-3.987557151
H152	-6.459945674	8.549242886	-2.558223831
H153	-4.750832663	7.516135141	-4.949344949

H154	-6.052563137	9.949519511	-0.748560751
H155	-5.881497610	6.906628144	-0.471814585
H156	-4.345105460	10.043778890	-1.233440112
H157	-1.401271436	4.370346468	-0.438618905
H158	-3.842303392	5.720189360	-1.011129106
H159	-5.457690248	5.458657928	1.308480800
H160	0.927540526	8.378473392	-0.695086235
H161	-1.898041061	7.909561294	-1.129073734
H162	-4.779453375	6.794664404	2.269861652
H163	-1.805421615	5.266090531	-1.909756492
H164	-1.952970016	9.166345743	1.368191779
H165	-0.343486228	9.624164315	-0.859500278
H166	-1.452808802	3.726402347	1.518619262
H167	-1.048759282	2.323659936	0.496817250
H168	-1.004082990	2.230490300	2.290933880
H169	0.796091743	4.092481705	0.723020928
H170	-1.976230281	10.179434460	3.454748247
H171	3.555462202	4.478511188	-0.544540177
H172	2.783950503	7.744708014	2.406306389
H173	6.038424128	7.692690772	1.046680302
H174	1.351343896	1.817795056	-0.030250708
H175	1.586187108	1.259501299	1.636826655
H176	6.211106589	-1.028268295	8.405849979
H177	4.742162072	5.319866648	-1.555205834
H178	6.203783898	7.107074896	-0.625639291
H179	5.294446152	4.193342426	-0.293157744
H180	6.780397429	6.106961312	0.730213172
H181	3.851597039	6.871175983	0.184264761
H182	4.961725037	4.809824908	2.168298650
H183	2.218595784	5.487716412	1.329716084
H184	2.670477428	2.483651161	0.939817719
H185	4.378848729	-1.006777424	-0.512330528
H186	4.805607820	0.715038398	-0.711571898
H187	6.321855256	0.076919255	0.719671321
H188	6.782147732	2.016997313	2.510290940
H189	5.801517812	-1.381000213	2.662422128
H190	4.839745440	-2.397313598	4.705929055
H191	5.346837953	1.341676660	6.802112078
H192	-0.198433057	5.502370969	2.501107265
H193	-2.258657781	5.734264110	4.824133962
H194	-1.024436205	6.560721157	5.802883702
H195	-1.754691811	7.200744592	3.003288651
H196	-0.209295006	10.383395073	3.390803868
H197	6.267182762	2.369865615	4.741657733
H198	0.920743123	2.983197753	3.578032712
H199	4.638059451	9.486995176	3.496971598
H200	2.905639936	9.443838915	3.914766680
H201	3.724048490	7.501412103	5.664328588
H202	-1.351561197	-1.383242077	13.773681499
H203	-7.262317096	-1.756653729	-0.290790467
H204	-5.534177452	-1.775356560	3.252068666
H205	-9.132377628	-5.146545238	-2.427659602
H206	-8.038962397	-6.444965361	-1.886527926
H207	-7.316374044	-3.859843062	-3.383010257
H208	-3.289416003	-6.678508242	-6.779524341
H209	-8.101554046	-5.906201923	-4.679573978
H210	-3.883770474	0.156433785	-2.574586508
H211	-6.136489065	1.778418688	-1.261846144
H212	-4.488025342	-5.756326370	-4.753198527
H213	-4.897796474	-4.638062038	-7.448120480
H214	-5.654533729	-7.190220099	-3.645617828
H215	-6.359094349	-0.620952286	0.707279976
H216	-6.074344255	-4.869549241	-2.639051986
H217	-7.213396769	-7.691402129	-3.481880080
H218	-6.296469887	1.531207963	-4.944330096
H219	-3.526161180	1.714528670	-1.843497475
H220	-6.935452432	-6.178758738	-0.082037925
H221	-4.981414991	0.936115220	-0.242645865
H222	2.007774614	4.352658805	-2.928305757
H223	-0.059618402	2.905846652	-1.997981805
H224	-0.808824462	7.408577098	-5.186630038
H225	-2.193236985	4.755343539	-5.199083840
H226	1.992965306	5.092290467	-6.362133725
H227	6.302640449	6.997363891	-8.701910407
H228	3.904194382	7.514569480	-5.650069739
H229	2.818282708	6.875646927	-8.134932219
H230	4.792019857	4.852439072	-6.909996029
H231	5.082800685	5.426607777	-12.444774814
H232	3.026816860	2.325458746	-3.966672183
H233	1.959126221	2.475303463	-5.366810912

H234	6.952733718	6.581655424	-11.137289132
H235	2.700603255	5.143946447	-8.447924634
H236	0.102855752	4.169697212	-5.162848361
H237	-1.702442605	6.774364631	-3.804276998
H238	-1.818132653	4.772581072	-8.713575667
H239	-0.528534997	-1.077539281	-3.261398646
H240	-3.429887664	5.211904150	-8.121663170
H241	7.077521949	-3.194744648	7.936086607
H242	6.518913874	-2.677225390	6.489603893
H243	5.221778767	-2.643401154	-2.010390344
H244	4.685771665	0.029968959	-2.903731979
H245	7.746783830	-1.583540215	-2.113737938
H246	1.253619244	0.026078252	-4.783853098
H247	0.965985132	-0.122923419	-6.990625961
H248	-0.707342222	-0.420622440	-8.445672681
H249	2.476115054	0.350936598	-6.926638160
H250	-2.067059656	-0.644286885	-6.441526356
H251	-2.679930636	0.375969023	-7.785229241
H252	9.541836169	-2.973277169	-6.119616742
H253	10.321549629	-2.325016544	-7.579316746
H254	7.437215908	-3.475264662	-6.547553660
H255	6.375573740	-2.495311478	-7.556101655
H256	4.976503939	-4.248781397	-9.384557070
H257	1.452858127	-4.059005742	-6.582759248
H258	4.949573982	2.364462841	-9.872926226
H259	7.199310730	2.322250156	-5.437429265
H260	4.167575484	-0.941595299	-8.410302862
H261	3.748305553	-4.370468166	-3.107794463
H262	5.998516497	-4.739836620	-2.648578246
H263	2.708036072	-2.613912977	-4.651340355
H264	-3.565591010	3.176061240	-7.021671746
H265	8.576081741	-6.585136924	-3.285958263
H266	-3.899639967	0.727738901	-5.522573430
H267	3.973409980	-5.424956105	-4.502172370
H268	5.072384379	1.128895084	-5.733978468
H269	9.360235092	-5.026512369	-2.957136910
H270	3.775526948	-5.024339119	-8.411512337
H271	4.724427131	-1.895292954	-9.746651813
H272	-6.072328978	0.286373034	-3.849920176
H273	-5.267511729	2.792681762	-3.302491599
H274	7.154796133	3.456884474	-9.608917487
H275	-1.103169344	1.344185949	-5.334971471
H276	5.635336050	-3.836596436	-5.470765686
H277	3.004209198	-4.895070409	-6.638897106
H278	1.837867848	-4.104511585	-4.342981509
H279	2.665417789	-1.912913799	-6.973398046
H280	9.142773274	0.962903263	-2.143926099
H281	8.430378878	-0.864321538	-4.720542409
H282	11.159291400	-1.196362926	-5.648734156
H283	9.768495213	-0.620836844	-1.632348605
H284	9.103355238	1.442254297	-6.035645665
H285	-4.104361939	-9.658071783	1.227696420
H286	-5.096575340	-8.640008443	2.288878707
H287	-3.696563343	-7.646700936	6.472970313
H288	-6.223658361	-10.706407224	2.008018065
H289	-7.041976692	-9.656267327	1.053985446
H290	5.687263078	-3.994891661	1.821723521
H291	8.623202686	-3.381840401	1.482195717
H292	7.445013613	-6.450276928	-1.259575304
H293	6.316205043	-2.790412520	0.655063181
H294	9.114076689	-4.935600286	1.611868996
H295	2.120246430	-8.526343274	0.154244779
H296	7.052360578	-5.763905368	0.785855197
H297	-2.779813777	2.297827282	9.309773415
H298	-3.789424023	-0.449203645	10.227554966
H299	0.181436219	-7.610428758	-0.601660134
H300	2.632839328	0.187189013	5.153358274
H301	-1.356713281	-7.811713275	-3.836594691
H302	-3.781194714	-3.019332285	-1.691429654
H303	-0.433290535	-6.336888509	-3.538387039
H304	-1.093291479	2.145490804	7.210173958
H305	-1.459084016	-6.585834677	-1.087291293
H306	-2.225647440	0.970776054	6.526921238
H307	-3.767650389	1.150187303	8.376045559
H308	-1.837814077	-0.531043005	8.524330318
H309	-0.839210872	0.736358157	9.260181406
H310	-4.637273356	-2.559720319	-6.355537004
H311	-4.703207960	2.307768366	10.970431252
H312	-7.707997874	1.702512817	10.828212635
H313	-1.445309380	-3.900180571	-1.568896376

	H314	-6.213340290	3.176924398	12.853586992
	H315	-7.228573210	3.732159152	11.693869010
	H316	-0.478469859	-0.654710599	6.347759832
	H317	-0.056732352	-4.007906909	-3.587494370
	H318	-4.808687667	-2.577878971	-3.914428893
	H319	-6.886440993	-4.042856922	9.846566674
	H320	-6.079694088	-5.850724009	7.195780581
	H321	-5.146231617	-2.423285001	8.951621895
	H322	-8.114295763	-4.485912345	8.988803667
	H323	0.730777279	-10.700365762	-0.990985559
	H324	-2.559787423	-2.929518990	7.298509485
	H325	-5.972376065	0.480412005	5.637123534
	H326	-3.192066402	-7.739111451	0.392778940
	H327	-7.514212461	-1.155111230	6.361465191
	H328	-7.458202006	-6.713720419	2.612428190
	H329	-5.689408349	-4.955374099	2.127872211
	H330	-7.480982277	1.234136597	12.518401906
	H331	1.488049231	-1.094155163	5.363025766
	H332	-2.162436776	-2.970738575	9.636344408
	H333	-1.080431353	-3.406185357	7.344102009
	H334	-2.216825801	-1.401011492	11.416695373
	H335	0.657733292	2.780666163	6.401252352
	H336	-7.457229350	-4.007551024	5.237528492
	H337	-6.911573899	-3.589279600	6.868788377
	H338	-7.882055088	-1.630393314	4.695089391
	H339	-3.509432527	-5.511822562	-0.525755962
	H340	-1.086371545	-3.593211594	-5.842668063
	H341	-3.101039905	-2.921132943	-7.189419733
	H342	-4.424991084	0.019309713	4.898933303
	H343	-3.544769534	-2.960236215	10.349565656
	H344	-5.562510565	-2.044547687	10.390270919
	H345	2.102132405	2.358857534	5.656833425
	H346	-7.319559752	-7.585818874	5.020511198
	H347	-4.498653406	-5.244345411	6.716122011
	H348	-4.951722590	-6.130088671	-1.384293036
	H349	2.783589149	-8.512729956	-1.496542083
	H350	3.907252512	-0.481934906	7.747352983
	H351	3.741625979	-2.033623547	6.887710052
	H352	-4.997667600	-9.331517724	7.473515909
	H353	-6.225960552	-8.307021787	7.825221540
	H354	-4.772834259	-5.666184128	11.344183225
	H355	-5.494672768	-5.952582940	9.198343973
	H356	-4.159205255	-8.318304121	11.113158540
	H357	-3.151168502	-5.438447481	10.657810742
	H358	4.355064854	-3.368357514	9.187946884
	H359	11.801535379	3.126092727	-6.712180584
	H360	-0.978533223	0.344353033	13.535927519
	H361	0.633437629	-0.698494958	11.537122706
	H362	9.740941390	2.142173000	-8.718718417
	H363	8.900714733	3.956272348	-6.369943555
	H364	8.864235682	4.397407414	-8.087571610
	H365	-2.988965877	-1.168957622	2.015212066
	H366	3.241057635	1.274505652	-8.782705925
	H367	-1.311296040	0.518578769	1.241077412
	H368	0.408153104	-0.682854932	3.118757101
	H369	-1.442160854	0.483463861	2.966808988
	H370	-1.331965896	-0.495076062	-0.546696465
	Mo371	-3.467050144	-3.623040065	4.204902337
	N372	0.012049365	-0.721554910	2.170566787
	N373	-1.173850969	-0.014099013	2.108562163
	N374	-8.975061242	4.368686064	-2.568199524
	N375	-6.427182803	6.666716293	-4.052352907
	N376	-5.502619170	8.582293419	-2.216844308
	N377	-5.290094898	7.266513994	0.280383238
	N378	-3.093681223	5.620511972	-0.330691243
	N379	-1.045173782	7.681705930	-0.612685830
	N380	-1.050165250	9.245160995	1.844058120
	N381	-1.263061476	7.322173789	3.890516899
	N382	0.150173208	4.884206021	3.232858063
	N383	2.914328000	5.119117943	1.979325585
	N384	3.584578275	7.699759098	3.037447526
	N385	-6.485256841	3.055974319	11.875468986
	N386	-4.726796753	1.291912566	10.834489045
	N387	-0.250527078	0.320856104	6.562124057
	N388	1.341165254	2.034480571	6.256294228
	N389	1.657745918	-0.067393305	5.309228136
	N390	-1.952834194	-0.531245048	11.931637568
	N391	-5.590063172	0.984962001	-4.446217882
	N392	-6.674418089	-0.920235108	-0.231198408
	N393	-3.196667163	1.476405004	-5.407098723

N394	-2.628721285	3.512384857	-7.232656054
N395	-1.777564527	5.620475727	-5.570411942
N396	0.651573443	4.699292415	-4.480590423
N397	0.781030321	0.477453231	-3.986625472
N398	-0.632287562	0.852955549	-2.312356121
N399	2.928707570	5.166839632	-5.971031665
N400	3.996388422	5.503594157	-10.585004858
N401	6.052161703	6.324963902	-10.742088382
N402	6.221207832	-2.964251063	7.426029145
N403	8.290085752	-4.331045376	1.681038805
N404	7.861184303	-5.587927965	-1.601469983
N405	6.274540591	-4.535857653	-3.616518244
N406	3.046208908	-2.858101367	-7.188559611
N407	4.156334593	-4.165425744	-8.791776715
N408	4.406568211	-1.865017707	-8.781945108
N409	5.411556296	-1.928244176	-2.738659076
N410	7.883379146	-0.660605896	-2.534184764
N411	9.396663703	-0.553478134	-4.824943941
N412	7.317930858	-2.778082752	-7.294049894
N413	9.843036670	1.448977074	-6.733699096
N414	-6.152613288	-10.155377483	1.150047359
N415	-4.050481067	-7.492678642	-0.108310738
N416	-1.785741028	-6.895148309	-2.022213129
N417	0.709443118	-8.124138343	-1.316130902
N418	-6.574447106	-7.190534441	-4.103160337
N419	-4.976721129	-5.257323473	-5.503583879
N420	-5.483644939	-8.492553195	7.143154021
N421	-5.094745531	-5.278785295	4.176757043
N422	-6.751056754	-6.426274637	3.283742530
N423	-4.596412359	-6.392285513	9.412303537
N424	2.104868763	-0.385639799	-1.235902819
N425	1.570229304	-0.967493247	-0.431222296
O426	-6.362574855	5.496219780	-2.098642481
O427	-3.369875478	7.832518795	-2.488069984
O428	-3.947393088	8.962932772	1.008378065
O429	-2.495693073	5.741786687	1.863934938
O430	0.385390107	6.064071105	0.079401759
O431	1.225147845	9.118909737	1.689404914
O432	-0.369431572	8.822589887	5.385803846
O433	0.045300073	4.321320295	5.451660105
O434	3.294746298	3.711088778	3.751297508
O435	5.540071063	6.733470112	3.665085551
O436	4.819130994	9.178791805	6.057708368
O437	-6.111882246	-0.517840038	11.224572037
O438	-2.215547313	1.744352319	12.119839369
O439	1.227855927	-1.472567381	13.328616165
O440	-2.825195468	3.290326535	-4.042791923
O441	-6.614838166	-0.799454923	-2.501742447
O442	-0.396137287	3.156676689	-6.926802000
O443	-0.602496373	0.189778546	-7.694113229
O444	-1.313417742	6.898838228	-7.421745057
O445	0.896438791	6.628810164	-3.280730648
O446	4.226936535	4.904138699	-4.116859450
O447	5.810965445	7.193602279	-6.306510520
O448	4.770754352	-1.767255350	10.385694371
O449	6.595038901	1.067215416	2.397451801
O450	8.387800729	-3.419791497	-1.134554192
O451	7.980910377	-4.667873549	-5.147243972
O452	5.400048252	-1.399420118	-4.964857873
O453	6.862100376	1.355243555	-2.930915274
O454	6.357190406	-0.521597991	-0.053888680
O455	11.267449423	0.078263259	-3.659334226
O456	11.283959064	0.117783520	-7.893900133
O457	8.079840950	-0.797301841	-8.144303506
O458	11.657254899	3.920174473	-8.590463110
O459	3.673683625	0.978078048	-7.958634898
O460	-6.333287215	-7.739951652	-0.186364015
O461	-3.620823419	-6.935492032	-3.393497550
O462	1.056731583	-8.644334551	-3.505231007
O463	2.733274373	-11.083780134	-0.898349098
O464	-6.956838930	-4.500892177	-6.344135348
O465	-8.377440698	-3.499105602	-0.528469399
O466	-7.227395206	-5.262980061	0.213351712
O467	-2.115807403	-5.262298413	-7.654232097
O468	-2.809991519	-7.650102036	8.811888340
O469	-2.716934303	-7.457892765	12.272785108
O470	-5.885275059	-0.618772180	2.540312721
O471	-6.354893525	1.459915393	3.299840032
O472	-9.205323431	-4.061599998	7.697017632
O473	-9.976551291	-3.004361916	5.852782973

	O474 -4.055419088 -3.038942882 6.230197596<br>O475 -5.076054183 -1.496889934 7.508690821<br>O476 -5.383945921 -2.453903813 4.078784540<br>O477 -2.566422624 -2.926589226 10.538525129<br>O478 -1.725923641 -2.858142372 7.828543624<br>O479 1.849102352 -0.325205676 -6.584133148<br>O480 -7.573945225 -4.743615963 9.795784299<br>O481 -5.248739925 -2.819832859 9.860702570<br>S482 4.744726850 -4.306466346 -0.405791716<br>S483 1.412490178 -3.852112585 -0.527807722<br>S484 -3.402047427 -3.373953650 1.842076888<br>S485 2.786329127 -2.117738864 2.006902914<br>S486 -1.335015530 -1.810489758 -0.148798109<br>S487 -1.094394155 -6.492794629 1.127778341<br>S488 -2.053855691 -1.664268750 4.410081642<br>S489 2.314519288 -5.622929146 2.423194353<br>S490 -1.874462199 -5.323937675 4.569094876<br>S491 1.231598043 -3.325492594 5.308569299<br>end

TS

Fe(139) -2.332<br>Fe(140) 0.025<br>Fe(141) -2.573<br>Fe(142) 2.742<br>Fe(143) 1.653<br>Fe(144) 2.362<br>Fe(145) -0.002	bm612n2xnewbrk2bh135ti_1_53386.72<br>C1 -6.984892244 5.841432201 -3.110027733<br>C2 -8.444913820 5.456664100 -3.375699468<br>C3 -5.000983626 6.973279284 -4.018389974<br>C4 -4.553354866 7.827521136 -2.838175261<br>C5 -5.166007466 9.348078292 -1.029139117<br>C6 -4.740624526 8.499578054 0.173524477<br>C7 -4.799948433 6.326554937 1.282001577<br>C8 -3.369833565 5.858158042 0.979328469<br>C9 -1.763096135 5.317651231 -0.805958477<br>C10 -0.721157951 6.370213359 -0.399809917<br>C11 -0.050405041 8.681920980 -0.315477057<br>C12 0.104942520 9.010122273 1.174549949<br>C13 -1.031603230 9.668383666 3.239806041<br>C14 -0.857030509 8.557793328 4.280250080<br>C15 -1.245535349 6.186702483 4.789137172<br>C16 -0.259032554 5.063623453 4.482369533<br>C17 1.049126943 3.774837530 2.820294869<br>C18 2.531665871 4.197058967 2.885898734<br>C19 0.690745420 3.254187066 1.409774273<br>C20 1.635102192 2.142938525 0.948002015<br>C21 -0.778800261 2.843959774 1.390513662<br>C22 4.282771857 5.634925100 1.887022723<br>C23 4.525882973 6.719669441 2.941109679<br>C24 4.620830922 6.129972963 0.456362979<br>C25 5.996992485 6.808769062 0.405627635<br>C26 4.550333733 4.971999054 -0.544148479<br>C27 3.837578255 8.838374841 3.873995183<br>C28 4.180435116 8.467039534 5.300641919<br>C29 -6.982191819 1.693141870 11.644523390<br>C30 -5.887945260 0.708148316 11.222835900<br>C31 -3.518502126 0.593580532 10.430121317<br>C32 -2.496547333 0.660283042 11.581489928<br>C33 -2.969270199 1.221939198 9.133086404<br>C34 -1.698468127 0.547943098 8.595091695<br>C35 -1.360539494 1.057755038 7.186213592<br>C36 0.893199935 0.789029229 6.048396040<br>C37 -1.018680844 -0.639277269 13.038025834<br>C38 0.388655989 -0.972372872 12.594845861<br>C39 -4.755009806 1.846364421 -3.593934427<br>C40 -3.485021428 2.278859719 -4.352512042<br>C41 -4.329981314 1.122916529 -2.295067477<br>C42 -5.439188683 0.924852506 -1.240290314<br>C43 -6.282942161 -0.342856560 -1.380896574<br>C44 -1.905524767 1.468315805 -6.081817920<br>C45 -1.576110637 2.787322988 -6.791230369<br>C46 -1.878968235 0.266130676 -7.038125788<br>C47 -2.450417036 4.835890351 -7.809998912<br>C48 -1.785276779 5.879053031 -6.903735767<br>C49 -1.062192785 6.486001366 -4.643413538<br>C50 0.246598614 5.938830234 -4.072876227<br>C51 1.907085126 4.141865039 -3.999935118<br>C52 3.126893629 4.795823709 -4.676648102<br>C53 2.006335842 2.633708664 -4.275472323<br>C54 0.982817624 1.779808968 -3.602710728<br>C55 0.109580602 1.960508590 -2.549916192<br>C56 -0.164776121 -0.076863899 -3.188415186<br>C57 4.009506392 5.623560113 -6.814495702<br>C58 4.629429332 6.874553176 -6.196041394

C59	3.410233536	5.948128674	-8.203802231
C60	4.372685867	6.016253486	-9.351919829
C61	5.651237277	6.532254432	-9.436513400
C62	5.022803787	5.704398510	-11.393668992
C63	5.544171530	-1.847086582	8.087073935
C64	4.839491742	-2.366207998	9.325945422
C65	4.458893158	-1.237872371	7.163232103
C66	5.038576238	-0.611117603	5.917279291
C67	5.455252175	0.732891267	5.902336933
C68	5.184470667	-1.358710013	4.734558948
C69	5.981350933	1.317484382	4.745534432
C70	5.721225661	-0.793330998	3.577674029
C71	6.108711279	0.548642395	3.581754327
C72	7.319345690	-4.700371482	0.639213983
C73	7.903154055	-4.501253004	-0.771663730
C74	6.065187435	-3.852143160	0.798899632
C75	8.394899073	-5.554446059	-2.951009374
C76	7.520400155	-4.866437775	-4.002605665
C77	5.333005736	-3.761212396	-4.420145601
C78	5.381110697	-2.253468812	-4.061547754
C79	3.928672350	-4.365973032	-4.192773202
C80	2.718391580	-3.693641409	-4.850501676
C81	2.521612745	-3.944595902	-6.355530145
C82	3.841669262	-2.968928415	-8.242738143
C83	5.451614606	-0.519432870	-2.339284359
C84	6.795743632	0.150367272	-2.654320003
C85	5.171885867	-0.310725681	-0.835712432
C86	9.216898709	-0.102988420	-2.426040910
C87	10.057097402	-0.185769649	-3.689145828
C88	10.155597887	-0.904360361	-6.007363538
C89	10.464307734	0.267328320	-6.973801832
C90	9.594658725	-2.098484418	-6.786311580
C91	8.261241030	-1.811555065	-7.461204163
C92	9.881500115	2.538271682	-7.698754464
C93	11.228892863	3.243953199	-7.661335562
C94	8.748446295	3.558246003	-7.387015966
C95	7.370073316	2.948617062	-7.517164505
C96	6.699003837	2.958130093	-8.750645687
C97	6.742563980	2.312419918	-6.432817099
C98	5.456726817	2.338340446	-8.906452476
C99	5.513832412	1.661783093	-6.575949261
C100	4.882770509	1.679182205	-7.817742640
C101	-5.087412143	-9.166641356	1.307174819
C102	-5.209564475	-8.076241004	0.255753156
C103	-3.947510757	-6.374240455	-1.042801644
C104	-3.103040534	-6.744273305	-2.269296997
C105	-0.828699228	-7.238559382	-3.055967280
C106	0.380911709	-8.067750178	-2.633004649
C107	1.918678124	-8.813268882	-0.895759006
C108	1.778041658	-10.322316858	-0.927529839
C109	-7.062053992	-5.822312302	-4.315261283
C110	-6.329601997	-5.141119064	-5.476743873
C111	-7.079882485	-4.890191245	-3.081217356
C112	-8.120494039	-5.358706889	-2.046769548
C113	-7.944498924	-4.625738936	-0.735330720
C114	-4.202857820	-4.659133030	-6.576009877
C115	-3.104088156	-5.601635352	-7.025688446
C116	-3.740592673	-3.199867829	-6.335219772
C117	-3.031703786	-3.114563467	-5.017254020
C118	-1.676164007	-3.462886087	-4.913037043
C119	-3.778150086	-2.902786105	-3.843744943
C120	-1.111130073	-3.705141109	-3.659088957
C121	-3.212275084	-3.147881902	-2.594069527
C122	-1.894501891	-3.608742723	-2.509997080
C123	-4.528627317	-7.361785497	7.127564987
C124	-3.898336977	-7.131423708	8.508489785
C125	-5.188648392	-6.085504415	6.585524128
C126	-5.620431350	-6.125291500	5.154326218
C127	-6.650599915	-6.851369512	4.594345102
C128	-5.812729437	-5.480524239	3.069268040
C129	-4.007152138	-6.137456095	10.716467330
C130	-3.576180397	-7.413255629	11.404530484
C131	-5.990134976	0.288449283	3.474302440
C132	-5.524202932	-0.160298372	4.874869786
C133	-5.791529734	-1.650258418	5.217589436
C134	-7.280697532	-1.879751062	5.571904495
C135	-7.609169538	-3.293054885	6.061133623
C136	-9.055941885	-3.458912912	6.575466501
C137	-4.928031587	-2.068610582	6.416854295
C138	-0.216454501	-3.497612351	2.300896144

Fe139	-1.744833669	-4.787264864	2.373576545
Fe140	0.776122540	-1.954312162	0.717427344
Fe141	1.576100355	-3.625892812	3.136988696
Fe142	0.501810075	-4.976102967	1.212176982
Fe143	3.015846711	-4.114903817	0.997713058
Fe144	-0.700141248	-3.471452928	4.260888530
Fe145	-1.507885117	-2.009836029	2.076009489
H146	-8.840405397	4.568380778	-1.581466376
H147	-8.458557316	3.508016304	-2.760839791
H148	-6.933798887	6.841940822	-4.906379521
H149	-8.559027833	5.214098324	-4.444201826
H150	-9.050109901	6.363417024	-3.203324355
H151	-4.398238676	6.053083942	-3.982807610
H152	-6.448100928	8.548417470	-2.562948832
H153	-4.741067787	7.504750041	-4.943146383
H154	-6.044962436	9.943384139	-0.749979357
H155	-5.888701904	6.908598626	-0.469258109
H156	-4.336337980	10.039096422	-1.231456553
H157	-1.412988846	4.355813839	-0.423770385
H158	-3.858558709	5.683604643	-1.003887671
H159	-5.475588923	5.460705934	1.314757210
H160	0.928416830	8.338413033	-0.672317003
H161	-1.905539106	7.899894455	-1.108745871
H162	-4.789918882	6.794929318	2.273264888
H163	-1.815044700	5.242483228	-1.899370755
H164	-1.952266422	9.154104694	1.375531290
H165	-0.326732120	9.598837151	-0.852130202
H166	-1.446445794	3.705640679	1.509295823
H167	-1.037903103	2.333864709	0.447364892
H168	-0.990385436	2.190608398	2.240995364
H169	0.799615813	4.091232705	0.706534009
H170	-1.975110124	10.189652691	3.455091330
H171	3.558372543	4.499418366	-0.556194058
H172	2.794897788	7.753411861	2.384956490
H173	6.046451943	7.701791967	1.045824380
H174	1.348351631	1.816801310	-0.060193084
H175	1.604928607	1.260229240	1.602661371
H176	6.216334461	-1.027763845	8.407504062
H177	4.760647244	5.322889442	-1.563660446
H178	6.219255377	7.118732912	-0.626034759
H179	5.291451221	4.197251919	-0.290032508
H180	6.789354735	6.116335256	0.730560924
H181	3.863705376	6.885192746	0.173349313
H182	4.959192856	4.809254068	2.151542098
H183	2.215715089	5.512202343	1.325085478
H184	2.675897715	2.495268822	0.888085340
H185	4.378088929	-1.000694301	-0.515498458
H186	4.805139164	0.720144510	-0.720635041
H187	6.315926358	0.082633135	0.718692819
H188	6.788811504	2.019164962	2.513692732
H189	5.803506576	-1.377824049	2.661863598
H190	4.843105546	-2.395569930	4.705745442
H191	5.352838579	1.341745554	6.804252334
H192	-0.186337149	5.501402677	2.470291306
H193	-2.241088653	5.717084620	4.828905206
H194	-0.998290166	6.546140570	5.794475946
H195	-1.766240294	7.200113222	3.007770743
H196	-0.206294843	10.374802152	3.398864777
H197	6.270766230	2.371574826	4.743469977
H198	0.920470854	2.980085988	3.563829511
H199	4.649526022	9.473715861	3.479849556
H200	2.919527185	9.445593915	3.900346177
H201	3.726543790	7.518729302	5.669962934
H202	-1.352417407	-1.380326716	13.781138883
H203	-7.266456433	-1.757175080	-0.294179039
H204	-5.548924747	-1.786913516	3.258286531
H205	-9.129980508	-5.145309421	-2.426250046
H206	-8.035429224	-6.443028141	-1.885232887
H207	-7.316986249	-3.858293108	-3.383489367
H208	-3.288994485	-6.675102602	-6.784214309
H209	-8.097243893	-5.907179539	-4.678127375
H210	-3.879511634	0.153625809	-2.561623315
H211	-6.137379482	1.780157640	-1.261242837
H212	-4.481338406	-5.751074113	-4.747321135
H213	-4.895568534	-4.633059275	-7.442394055
H214	-5.649111138	-7.186901845	-3.640937180
H215	-6.370882074	-0.618354295	0.707870663
H216	-6.072949568	-4.863368089	-2.637355203
H217	-7.207235828	-7.690375732	-3.476760240
H218	-6.285088859	1.527979656	-4.945823802

H219	-3.526932697	1.716374134	-1.836091080
H220	-6.935016364	-6.177749150	-0.079589796
H221	-4.987641589	0.937780460	-0.237300373
H222	2.001869815	4.350949036	-2.925807851
H223	-0.050796233	2.858656652	-1.963727224
H224	-0.811518212	7.413951064	-5.175927902
H225	-2.189829663	4.757650656	-5.197475185
H226	1.987940429	5.088722715	-6.360489555
H227	6.300674935	6.994468714	-8.700662006
H228	3.894282361	7.514169616	-5.649650251
H229	2.818817959	6.878086703	-8.130457601
H230	4.788941207	4.850872896	-6.905272750
H231	5.076833736	5.425158909	-12.442673942
H232	3.017317312	2.322438426	-3.961965304
H233	1.954591400	2.474591063	-5.365508254
H234	6.949284848	6.576546078	-11.136065783
H235	2.693515451	5.147813810	-8.446627916
H236	0.104669877	4.179027104	-5.171263646
H237	-1.704455964	6.771669025	-3.796497386
H238	-1.827754687	4.781376388	-8.713781201
H239	-0.473601321	-1.117045121	-3.262870295
H240	-3.434582159	5.219963483	-8.108523630
H241	7.083660150	-3.191803189	7.940830635
H242	6.522546464	-2.680917206	6.493121081
H243	5.221400294	-2.643403513	-2.010836144
H244	4.687805895	0.028517512	-2.909206777
H245	7.747195340	-1.585505950	-2.115033576
H246	1.257635909	0.035131139	-4.810193063
H247	0.968922039	-0.122940783	-6.994122101
H248	-0.706530452	-0.408831066	-8.456658297
H249	2.479443616	0.347618554	-6.923649689
H250	-2.062962347	-0.644202803	-6.443854968
H251	-2.677310795	0.371809799	-7.789489583
H252	9.540067240	-2.975673084	-6.126702535
H253	10.318881887	-2.321935324	-7.584856894
H254	7.433408455	-3.467160123	-6.540079430
H255	6.367992883	-2.484695490	-7.541476104
H256	4.980268652	-4.242827796	-9.375967909
H257	1.450550504	-4.057781139	-6.580706554
H258	4.944278246	2.366750316	-9.872021941
H259	7.201111416	2.320751684	-5.440503858
H260	4.160125381	-0.935365217	-8.417605545
H261	3.745268762	-4.368755851	-3.106196627
H262	5.994866569	-4.743454970	-2.647717698
H263	2.707457493	-2.610837360	-4.648820107
H264	-3.567075936	3.176155812	-7.027842114
H265	8.572362699	-6.585504671	-3.285423384
H266	-3.907725585	0.734914491	-5.533451232
H267	3.968499839	-5.423993243	-4.500725036
H268	5.076893399	1.123161658	-5.734905819
H269	9.360495931	-5.028453339	-2.958518271
H270	3.783465655	-5.021412014	-8.399584650
H271	4.729658006	-1.898285440	-9.743669977
H272	-6.065088287	0.281303899	-3.852376705
H273	-5.258765063	2.787958817	-3.303805198
H274	7.149052716	3.459882282	-9.610247031
H275	-1.105215265	1.344605425	-5.337507381
H276	5.632969626	-3.838859918	-5.469204340
H277	3.003620802	-4.889861605	-6.637602100
H278	1.835075779	-4.100507723	-4.341044948
H279	2.658449120	-1.908726950	-6.971988661
H280	9.148704810	0.957991740	-2.141523289
H281	8.427367272	-0.868360906	-4.720833381
H282	11.154999428	-1.199785624	-5.650556039
H283	9.771330568	-0.629717011	-1.637648115
H284	9.095739746	1.436597962	-6.038059926
H285	-4.105036936	-9.656104265	1.227470635
H286	-5.101415358	-8.639464912	2.283818144
H287	-3.689198021	-7.642218624	6.473397750
H288	-6.222931333	-10.704673651	2.009109530
H289	-7.042372984	-9.659817941	1.050490611
H290	5.690066419	-3.994179183	1.822465047
H291	8.626970289	-3.375287567	1.478991972
H292	7.444830226	-6.447574609	-1.257381831
H293	6.310972374	-2.790442834	0.650958574
H294	9.117237964	-4.928919623	1.610454656
H295	2.131298267	-8.520123767	0.145141965
H296	7.059466546	-5.760543341	0.786180072
H297	-2.783686963	2.293680309	9.316514599
H298	-3.795989874	-0.450614600	10.238807348

H299	0.184253041	-7.611469482	-0.600969438
H300	2.649418142	0.193722915	5.203027085
H301	-1.357572066	-7.802643760	-3.833610141
H302	-3.793677912	-2.980865625	-1.684785009
H303	-0.429816492	-6.329545856	-3.539287354
H304	-1.120814209	2.128202198	7.207197973
H305	-1.458138937	-6.578878593	-1.083673769
H306	-2.243127107	0.937241760	6.536978090
H307	-3.775044978	1.147183310	8.385509883
H308	-1.848361722	-0.541513857	8.546536399
H309	-0.846480066	0.731809065	9.268833185
H310	-4.631604720	-2.555746133	-6.352541918
H311	-4.709239003	2.307136142	10.978889875
H312	-7.707356359	1.708576207	10.816679089
H313	-1.452595434	-3.847599873	-1.547686719
H314	-6.217295477	3.173178368	12.853994402
H315	-7.227958586	3.735276783	11.693677950
H316	-0.458990432	-0.668493733	6.364956833
H317	-0.060827648	-3.982950369	-3.557656880
H318	-4.816967386	-2.571884326	-3.914776980
H319	-6.888440782	-4.041466098	9.843525748
H320	-6.077587649	-5.853955719	7.197264500
H321	-5.152560866	-2.419277440	8.953357546
H322	-8.118974391	-4.482403192	8.988206419
H323	0.727213124	-10.696734234	-0.984459883
H324	-2.573090127	-2.930984427	7.302750367
H325	-5.979739299	0.485116471	5.636156805
H326	-3.194076697	-7.726028358	0.397707580
H327	-7.533620001	-1.153786128	6.360637245
H328	-7.459042721	-6.719502940	2.614838495
H329	-5.689413935	-4.956659243	2.129473464
H330	-7.496325139	1.238747587	12.508202993
H331	1.491692349	-1.080139383	5.377484751
H332	-2.164938874	-2.971716818	9.640882948
H333	-1.089955394	-3.397445844	7.333849681
H334	-2.221948674	-1.401434970	11.423372375
H335	0.631780364	2.774471100	6.457106303
H336	-7.464461498	-4.004620302	5.232865922
H337	-6.920297469	-3.588189858	6.864995566
H338	-7.895234186	-1.630052172	4.692948612
H339	-3.515684239	-5.505355287	-0.522050054
H340	-1.084516511	-3.596292714	-5.820926471
H341	-3.091991189	-2.918614314	-7.179110279
H342	-4.435708109	0.017922020	4.897905264
H343	-3.548277268	-2.958494673	10.351610832
H344	-5.567377858	-2.045379707	10.393798147
H345	2.079624663	2.392175881	5.690486923
H346	-7.314604328	-7.592767065	5.023162880
H347	-4.498521617	-5.242471003	6.716293935
H348	-4.953011125	-6.125267404	-1.386445383
H349	2.784767519	-8.519729592	-1.508316834
H350	3.912757756	-0.482130717	7.748256625
H351	3.748300498	-2.034550164	6.889220260
H352	-4.986928407	-9.330948227	7.475200435
H353	-6.218345755	-8.308958682	7.823114189
H354	-4.780928417	-5.670975085	11.346921767
H355	-5.489958775	-5.949930063	9.194485991
H356	-4.152487662	-8.322598433	11.110215604
H357	-3.157898237	-5.437580344	10.666020342
H358	4.354142173	-3.362825617	9.190332130
H359	11.795323819	3.129000518	-6.706544092
H360	-0.976737031	0.345301134	13.537071736
H361	0.628325349	-0.699494925	11.537914530
H362	9.739523233	2.141980798	-8.717293595
H363	8.897530146	3.958031497	-6.371690950
H364	8.864591865	4.395748437	-8.090295048
H365	-2.507732563	-0.569441738	2.040392327
H366	3.237287824	1.274533861	-8.779347179
H367	-1.428022536	0.519412795	1.236828635
H368	0.360894978	-0.672781884	3.093017902
H369	-1.521889398	0.488801610	2.940709079
H370	-1.344344048	-0.440805277	-0.445235899
Mo371	-3.477278816	-3.632890884	4.192278444
N372	0.062519169	-0.778845057	2.112337853
N373	-1.480229259	-0.070673490	2.078180974
N374	-8.980174933	4.368140089	-2.574914150
N375	-6.420162435	6.658993905	-4.048157321
N376	-5.492479159	8.576007067	-2.216494414
N377	-5.297609424	7.265907356	0.284235006
N378	-3.107229723	5.604053226	-0.323627879

N379	-1.051409232	7.665504138	-0.597468551
N380	-1.050325322	9.240837233	1.851708903
N381	-1.270317074	7.320647440	3.892707381
N382	0.156248184	4.882582941	3.204305574
N383	2.913569175	5.126722047	1.962871186
N384	3.585001041	7.694312279	3.028563718
N385	-6.487333500	3.056097651	11.874840112
N386	-4.731071480	1.290964722	10.843835343
N387	-0.257597471	0.317006317	6.556892307
N388	1.321841244	2.043821744	6.279403356
N389	1.668464647	-0.054502557	5.329156821
N390	-1.957513947	-0.531986520	11.938696753
N391	-5.580811894	0.980706999	-4.445929295
N392	-6.680626284	-0.919353674	-0.232225574
N393	-3.197299074	1.476183629	-5.412304768
N394	-2.629879475	3.517268766	-7.229723815
N395	-1.777893909	5.624892546	-5.566460148
N396	0.647886453	4.700842245	-4.478380995
N397	0.787825420	0.470503339	-4.000894780
N398	-0.600003448	0.806097831	-2.298316188
N399	2.923096186	5.161839212	-5.968047044
N400	3.991417268	5.503525461	-10.581955528
N401	6.048101616	6.322241618	-10.740529510
N402	6.226270116	-2.964973650	7.430889951
N403	8.293385998	-4.324057956	1.679057178
N404	7.862641103	-5.586436353	-1.600429108
N405	6.271848911	-4.537510354	-3.615004498
N406	3.040845093	-2.853432478	-7.186403949
N407	4.162056528	-4.161850217	-8.780257770
N408	4.398554136	-1.861055089	-8.783722226
N409	5.412190050	-1.928551036	-2.738654770
N410	7.884508347	-0.662459299	-2.535037910
N411	9.393037038	-0.556462655	-4.826528511
N412	7.311274898	-2.771599680	-7.287659011
N413	9.838213833	1.446407583	-6.732908593
N414	-6.152993536	-10.158671511	1.147648042
N415	-4.051988603	-7.488935680	-0.109668533
N416	-1.783876376	-6.881612782	-2.021456334
N417	0.713179138	-8.121116100	-1.318342492
N418	-6.569050106	-7.189269805	-4.098599535
N419	-4.971463946	-5.255130399	-5.499188174
N420	-5.474216024	-8.493551373	7.142752011
N421	-5.099926175	-5.279243335	4.176873474
N422	-6.752778094	-6.430396634	3.285987202
N423	-4.595305084	-6.393679100	9.413996519
N424	2.155650823	-0.373861199	-1.295042960
N425	1.616121887	-0.944472129	-0.484176120
O426	-6.359120913	5.480742433	-2.098667699
O427	-3.363562936	7.811068045	-2.475848750
O428	-3.934052804	8.948235300	1.004660920
O429	-2.505748277	5.741892383	1.868130509
O430	0.377314059	6.042510719	0.079116636
O431	1.224907880	9.100129629	1.704212424
O432	-0.391187273	8.820717146	5.397442396
O433	0.075117189	4.319370689	5.419339585
O434	3.311368197	3.685512949	3.702337163
O435	5.533647691	6.722830258	3.666878520
O436	4.842678513	9.188735103	6.032059634
O437	-6.119293810	-0.518100295	11.228535953
O438	-2.218714289	1.743968747	12.126580319
O439	1.223506441	-1.481780094	13.325924465
O440	-2.794617117	3.262189798	-4.022389685
O441	-6.611141231	-0.799275359	-2.502509999
O442	-0.397664124	3.163097308	-6.922536574
O443	-0.599290481	0.191162324	-7.697153857
O444	-1.307968016	6.901220740	-7.417641081
O445	0.892931073	6.625826320	-3.270395624
O446	4.223430918	4.895690739	-4.116534657
O447	5.799511810	7.199666975	-6.312504473
O448	4.776858572	-1.763199681	10.387853291
O449	6.599839361	1.069889394	2.399425141
O450	8.388689664	-3.417117551	-1.138140436
O451	7.980548773	-4.660712514	-5.143684078
O452	5.397034185	-1.401505788	-4.965254951
O453	6.864826135	1.353730575	-2.934610182
O454	6.355903271	-0.514884717	-0.055195777
O455	11.266132571	0.077256824	-3.665964930
O456	11.285602332	0.119070385	-7.890091906
O457	8.077657521	-0.796839139	-8.148493662
O458	11.654822590	3.916886089	-8.587223414

O459	3.670557186	0.976635840	-7.956065566
O460	-6.334391193	-7.739642833	-0.190102315
O461	-3.617244710	-6.925502466	-3.393670245
O462	1.057124003	-8.632395470	-3.509729640
O463	2.727889553	-11.086015685	-0.867906573
O464	-6.951490498	-4.502142892	-6.342425186
O465	-8.374672789	-3.496993543	-0.527707171
O466	-7.228951771	-5.262514348	0.216165983
O467	-2.109955800	-5.256796511	-7.648032345
O468	-2.806716234	-7.650228676	8.815703454
O469	-2.720269266	-7.456730258	12.278219909
O470	-5.891724569	-0.611583006	2.536853531
O471	-6.360587990	1.466265610	3.301275244
O472	-9.213226469	-4.060396743	7.696546268
O473	-9.987113030	-3.014936739	5.846511860
O474	-4.063264977	-3.027826120	6.230187908
O475	-5.095470265	-1.493878771	7.511926484
O476	-5.399926164	-2.455622733	4.082051993
O477	-2.570165041	-2.925077450	10.542276557
O478	-1.737140868	-2.862235354	7.830517288
O479	1.847148246	-0.320738456	-6.574435904
O480	-7.579631073	-4.739031320	9.796155211
O481	-5.251902501	-2.818423631	9.862315836
S482	4.743002476	-4.317036004	-0.403499527
S483	1.409658112	-3.843145748	-0.522177657
S484	-3.422084096	-3.417851719	1.816043910
S485	2.761543983	-2.130820430	1.986161084
S486	-1.314264676	-1.790042483	-0.193176007
S487	-1.096338831	-6.516634846	1.128935790
S488	-2.035796605	-1.705163679	4.377376097
S489	2.308871054	-5.642970818	2.430337427
S490	-1.892196883	-5.346942659	4.556850825
S491	1.217249492	-3.320571568	5.317628466
end			
end			

product

Fe(139)	-2.052
Fe(140)	-0.229
Fe(141)	-2.532
Fe(142)	2.682
Fe(143)	1.643
Fe(144)	2.481
Fe(145)	0.160

bm612n2xnewbrk2bh135ti.car_5

C1	-6.978119917	5.834638379	-3.104617014
C2	-8.439338956	5.448952344	-3.365617201
C3	-5.007533653	6.985780765	-4.023001915
C4	-4.562742079	7.845950943	-2.845838654
C5	-5.178987396	9.354800455	-1.029308454
C6	-4.748368038	8.509478772	0.174364271
C7	-4.782000300	6.330440477	1.276028408
C8	-3.349903472	5.870689546	0.971914694
C9	-1.742704790	5.339809031	-0.815882749
C10	-0.703831427	6.395874355	-0.410424546
C11	-0.054982323	8.718148429	-0.336127415
C12	0.105086437	9.034135392	1.156206351
C13	-1.026561146	9.668966381	3.230671303
C14	-0.836981565	8.558208121	4.268925026
C15	-1.261343834	6.195400743	4.797452732
C16	-0.280375439	5.059956062	4.514304545
C17	1.040790588	3.774489133	2.842541635
C18	2.519870339	4.203688156	2.924018294
C19	0.677922533	3.247303974	1.441912169
C20	1.623608589	2.132229580	0.994361637
C21	-0.784372536	2.810871441	1.438365081
C22	4.277556824	5.627009107	1.906931217
C23	4.523824502	6.723421618	2.952014186
C24	4.607515366	6.113306932	0.471021082
C25	5.981462353	6.794584983	0.409364641
C26	4.538297488	4.955733923	-0.531102292
C27	3.823176766	8.843027194	3.893696110
C28	4.161575987	8.455251036	5.318050170
C29	-6.970044531	1.684650198	11.642023748
C30	-5.875114671	0.704667403	11.212584347
C31	-3.507330553	0.595228992	10.416048946
C32	-2.487796722	0.660922113	11.570247752
C33	-2.960179830	1.229101034	9.121533210
C34	-1.688456037	0.562802747	8.582154410
C35	-1.336404951	1.084223811	7.182985534
C36	0.907607965	0.767324308	6.051784253
C37	-1.016246880	-0.638683156	13.034296355
C38	0.394443291	-0.966653853	12.595415333
C39	-4.753758826	1.840905396	-3.585161821
C40	-3.495383010	2.287109440	-4.354946661
C41	-4.323102522	1.113175663	-2.292936702
C42	-5.434035555	0.918463936	-1.239443734

C43	-6.280824738	-0.347760005	-1.377406540
C44	-1.903492472	1.467635088	-6.076101127
C45	-1.574907083	2.782514420	-6.791962527
C46	-1.881131924	0.265383170	-7.032684517
C47	-2.446750490	4.826900675	-7.816741291
C48	-1.788385742	5.873701537	-6.909622597
C49	-1.060269909	6.482716260	-4.653137952
C50	0.249756837	5.938447024	-4.081596052
C51	1.914093660	4.144605416	-4.001005856
C52	3.131815989	4.802001719	-4.677404273
C53	2.015849477	2.635894654	-4.275682690
C54	0.986282374	1.791831332	-3.600292211
C55	0.091612535	2.003471417	-2.571035331
C56	-0.205814067	-0.035998996	-3.188099739
C57	4.016546142	5.623262580	-6.818204425
C58	4.641256244	6.869190952	-6.195602870
C59	3.416041021	5.944886225	-8.206224464
C60	4.378298140	6.015623129	-9.353714226
C61	5.655478041	6.535081867	-9.437738707
C62	5.029629190	5.705997989	-11.395221139
C63	5.550111574	-1.843648980	8.095116480
C64	4.840778634	-2.368001335	9.328360770
C65	4.461201591	-1.236572573	7.169535692
C66	5.038117750	-0.612443559	5.920700721
C67	5.450997696	0.732487849	5.901890941
C68	5.184475323	-1.361787689	4.739155848
C69	5.975046306	1.316111912	4.743630667
C70	5.719755997	-0.797452113	3.580754209
C71	6.103787758	0.545249830	3.581316357
C72	7.336305610	-4.708276167	0.638538831
C73	7.916421480	-4.507061366	-0.773479405
C74	6.083269771	-3.858002511	0.794548752
C75	8.399651752	-5.556145162	-2.956543522
C76	7.526257632	-4.870273305	-4.010462326
C77	5.341727928	-3.758621849	-4.429243090
C78	5.386032763	-2.251009515	-4.066818090
C79	3.937709280	-4.365989354	-4.199912124
C80	2.723828927	-3.697714619	-4.855207929
C81	2.525253612	-3.949286475	-6.359910296
C82	3.844175050	-2.974536294	-8.250252478
C83	5.451445351	-0.521302464	-2.337455557
C84	6.794140060	0.151596161	-2.652878300
C85	5.175516113	-0.318766005	-0.831949290
C86	9.215549158	-0.097430946	-2.427265338
C87	10.062141906	-0.182641762	-3.687152013
C88	10.169109589	-0.898650862	-6.011251420
C89	10.471333839	0.270719782	-6.980510997
C90	9.602646973	-2.096160106	-6.782159867
C91	8.268959328	-1.812417360	-7.459202214
C92	9.889043186	2.543035395	-7.705722505
C93	11.235816726	3.247677426	-7.668999177
C94	8.752246750	3.558220038	-7.386938121
C95	7.373873262	2.946510451	-7.515231373
C96	6.703964700	2.954327263	-8.749445579
C97	6.743237011	2.313996247	-6.430069752
C98	5.461430918	2.335626327	-8.906227883
C99	5.513034626	1.665936138	-6.573862420
C100	4.884715268	1.679750355	-7.817219740
C101	-5.081333184	-9.161421547	1.314579735
C102	-5.208904478	-8.076331266	0.260712654
C103	-3.946805391	-6.383428079	-1.045963291
C104	-3.104872225	-6.759288479	-2.271620493
C105	-0.829904944	-7.253565962	-3.055958533
C106	0.378747794	-8.081951440	-2.632041413
C107	1.917781454	-8.824715653	-0.895674367
C108	1.782397191	-10.333986688	-0.948016948
C109	-7.056428013	-5.819539198	-4.306592299
C110	-6.328041014	-5.139680426	-5.470906813
C111	-7.076573925	-4.890336938	-3.073452267
C112	-8.122465009	-5.359596913	-2.044933708
C113	-7.947944888	-4.627250724	-0.733610900
C114	-4.200937336	-4.663181528	-6.570472173
C115	-3.104704035	-5.607029137	-7.022613826
C116	-3.743917276	-3.200927986	-6.340322937
C117	-3.026893239	-3.115805602	-5.027467350
C118	-1.671938878	-3.469418416	-4.934528638
C119	-3.766721068	-2.915742884	-3.848048054
C120	-1.103186078	-3.733685269	-3.686460402
C121	-3.196919202	-3.184383045	-2.604012600
C122	-1.881476560	-3.653770313	-2.532030342

	C123	-4.538586096	-7.363390753	7.126613811
	C124	-3.905148775	-7.132797764	8.505950106
	C125	-5.192072426	-6.083103867	6.584529492
	C126	-5.619534130	-6.116119344	5.151784728
	C127	-6.649058612	-6.841556914	4.588903747
	C128	-5.803091124	-5.470727545	3.068250622
	C129	-4.001766084	-6.135764911	10.710860427
	C130	-3.575524698	-7.412038072	11.401641462
	C131	-5.963642883	0.293532919	3.476649420
	C132	-5.509695007	-0.156113480	4.880032177
	C133	-5.777722487	-1.646141956	5.219913676
	C134	-7.264932475	-1.879337581	5.575386471
	C135	-7.594831764	-3.291937096	6.066148888
	C136	-9.042678797	-3.453297590	6.580814088
	C137	-4.911954988	-2.064573468	6.417123863
	C138	-0.198925963	-3.500674339	2.299279060
	Fe139	-1.767846616	-4.647808139	2.318741062
	Fe140	0.820479907	-2.023103475	0.814825171
	Fe141	1.633533023	-3.655048091	3.123097904
	Fe142	0.507669286	-5.008994817	1.249305371
	Fe143	3.059625384	-4.178155703	0.998679541
	Fe144	-0.643422706	-3.504755129	4.277853737
	Fe145	-1.357385125	-1.877764199	2.112437405
	H146	-8.830589239	4.565850751	-1.568635114
	H147	-8.450951451	3.502346008	-2.745894544
	H148	-6.943663146	6.836942211	-4.900044712
	H149	-8.557262198	5.205010497	-4.433500979
	H150	-9.043593837	6.356486644	-3.192957245
	H151	-4.394126436	6.072508266	-3.989736102
	H152	-6.464326808	8.545639795	-2.555708380
	H153	-4.758388975	7.518930158	-4.949798123
	H154	-6.057098808	9.950627044	-0.748531523
	H155	-5.877004034	6.905502509	-0.472716018
	H156	-4.350081912	10.045793145	-1.234717663
	H157	-1.392893417	4.376345900	-0.434503961
	H158	-3.833929577	5.731202092	-1.013351917
	H159	-5.449808863	5.458510515	1.306537411
	H160	0.926710206	8.395256415	-0.704626273
	H161	-1.895390221	7.913850288	-1.136169991
	H162	-4.774729656	6.795810512	2.268669728
	H163	-1.797587917	5.269914611	-1.909723957
	H164	-1.952671307	9.168194686	1.365774474
	H165	-0.350587578	9.635599215	-0.862235714
	H166	-1.461450220	3.665515470	1.561154968
	H167	-1.035615624	2.297026433	0.497922544
	H168	-0.970461396	2.127347237	2.274886561
	H169	0.780799332	4.080520609	0.730860047
	H170	-1.975963223	10.176043078	3.454332754
	H171	3.553289061	4.468462857	-0.536631990
	H172	2.776886143	7.736368598	2.415052432
	H173	6.034132135	7.688716684	1.047337094
	H174	1.353876794	1.816446597	-0.021189994
	H175	1.552605545	1.245915724	1.641578030
	H176	6.220692362	-1.023983235	8.415575800
	H177	4.732614455	5.316746541	-1.550681711
	H178	6.195786601	7.101395152	-0.624892775
	H179	5.294825979	4.191051490	-0.292685866
	H180	6.776275207	6.103111308	0.730682765
	H181	3.845654892	6.864394617	0.190075259
	H182	4.960237930	4.807238050	2.175773216
	H183	2.218390690	5.471410779	1.327517559
	H184	2.672439476	2.465824333	0.967833294
	H185	4.384649546	-1.012122568	-0.511279765
	H186	4.807092325	0.710871011	-0.711336930
	H187	6.324590928	0.074969749	0.719026436
	H188	6.778191228	2.015156145	2.507965771
	H189	5.802595330	-1.383280834	2.665897383
	H190	4.843521207	-2.398790115	4.710903501
	H191	5.346192898	1.343182770	6.802186694
	H192	-0.203446031	5.493909255	2.508949566
	H193	-2.263268405	5.738561153	4.820758917
	H194	-1.030964886	6.562535026	5.804336282
	H195	-1.750110933	7.200610799	3.001608360
	H196	-0.209668481	10.385556463	3.387667624
	H197	6.263026646	2.370549803	4.740108111
	H198	0.916603722	2.973448351	3.582066755
	H199	4.629755214	9.486502682	3.504982055
	H200	2.897558991	9.438232344	3.921247835
	H201	3.721388708	7.492294644	5.665870814
	H202	-1.350964366	-1.384457215	13.771983051

	H203	-7.263369656	-1.759616104	-0.286348395
	H204	-5.513048504	-1.770595304	3.256046605
	H205	-9.130146664	-5.144967297	-2.428636016
	H206	-8.039250568	-6.444428181	-1.885066733
	H207	-7.312156944	-3.858335925	-3.376402033
	H208	-3.288460678	-6.679709346	-6.776478852
	H209	-8.092450339	-5.902083567	-4.669805428
	H210	-3.876333971	0.143849407	-2.564141677
	H211	-6.130541346	1.774958715	-1.264471573
	H212	-4.483123861	-5.759324053	-4.745954567
	H213	-4.893198004	-4.641916191	-7.438328153
	H214	-5.650327852	-7.195056474	-3.639020418
	H215	-6.355014058	-0.623964449	0.709604107
	H216	-6.071261675	-4.865343331	-2.626378575
	H217	-7.212050252	-7.692597697	-3.481350488
	H218	-6.286201196	1.530605663	-4.935352700
	H219	-3.516974093	1.702402538	-1.834336883
	H220	-6.935820676	-6.178290985	-0.079685063
	H221	-4.985655141	0.935188361	-0.235284018
	H222	2.008093253	4.352538101	-2.926513012
	H223	-0.066091752	2.917499491	-2.007553967
	H224	-0.808182975	7.407100347	-5.191007858
	H225	-2.193823172	4.754493787	-5.200681325
	H226	1.992961324	5.090752786	-6.361006936
	H227	6.303789663	6.998551878	-8.701584595
	H228	3.907295812	7.510970157	-5.650424046
	H229	2.820485942	6.872068947	-8.132610322
	H230	4.792021364	4.846225564	-6.907633337
	H231	5.084145337	5.427584804	-12.444367314
	H232	3.027964112	2.325404341	-3.964935091
	H233	1.961307342	2.476158855	-5.365728852
	H234	6.953209397	6.584501441	-11.137320714
	H235	2.702879426	5.140877150	-8.447896006
	H236	0.102932397	4.166619431	-5.159983693
	H237	-1.701384848	6.774793983	-3.807553823
	H238	-1.814776663	4.768506261	-8.713953023
	H239	-0.545685447	-1.066360408	-3.265286430
	H240	-3.428142181	5.208666345	-8.126986943
	H241	7.084621846	-3.195500895	7.937818848
	H242	6.521037770	-2.673447200	6.494053379
	H243	5.226935023	-2.645871259	-2.015686773
	H244	4.685800424	0.029868476	-2.902093817
	H245	7.748705381	-1.582839467	-2.116334214
	H246	1.253262141	0.025298197	-4.777367126
	H247	0.967049698	-0.125892965	-6.986054009
	H248	-0.706284346	-0.425533867	-8.441173048
	H249	2.476274456	0.349632642	-6.923892053
	H250	-2.069505147	-0.644654580	-6.439386194
	H251	-2.678551105	0.376117085	-7.784359788
	H252	9.546220548	-2.969374943	-6.117168757
	H253	10.324321641	-2.328226095	-7.580470631
	H254	7.443117776	-3.478687683	-6.552239492
	H255	6.381888334	-2.499520583	-7.562723180
	H256	4.973703317	-4.250867461	-9.389497065
	H257	1.453579081	-4.059280666	-6.583763049
	H258	4.950446364	2.362270489	-9.872626752
	H259	7.199661597	2.323072518	-5.436755853
	H260	4.171441788	-0.943573201	-8.408412395
	H261	3.756140093	-4.369657213	-3.112881333
	H262	6.002706442	-4.738947318	-2.654976822
	H263	2.709084451	-2.614895857	-4.653721698
	H264	-3.565632090	3.175807254	-7.019784189
	H265	8.579741319	-6.586858399	-3.290349021
	H266	-3.893421426	0.721743380	-5.515015334
	H267	3.978861679	-5.424251175	-4.506750228
	H268	5.071557482	1.131938151	-5.732223098
	H269	9.363696131	-5.027550398	-2.962353073
	H270	3.770690909	-5.025092863	-8.417857793
	H271	4.723426137	-1.894587999	-9.748369476
	H272	-6.072065682	0.282516102	-3.843545169
	H273	-5.258303869	2.779960812	-3.287737885
	H274	7.155639260	3.454201019	-9.609213327
	H275	-1.100183820	1.343810577	-5.335173123
	H276	5.641025292	-3.834483268	-5.478275644
	H277	3.004518406	-4.896247525	-6.641409611
	H278	1.842705327	-4.108052791	-4.344610249
	H279	2.667405613	-1.913557084	-6.974345532
	H280	9.141733215	0.964680103	-2.148189223
	H281	8.437469078	-0.861645022	-4.726956209
	H282	11.167961573	-1.193294545	-5.653334382

	H283	9.768253725	-0.617511041	-1.633289372
	H284	9.110424590	1.444152844	-6.037716842
	H285	-4.101685778	-9.655004796	1.229655911
	H286	-5.093671013	-8.636971231	2.291948750
	H287	-3.700807180	-7.649073338	6.472675849
	H288	-6.221133280	-10.705450549	2.010024592
	H289	-7.039074700	-9.653936595	1.056647866
	H290	5.698565985	-4.003324460	1.814283512
	H291	8.636221091	-3.383061706	1.487082072
	H292	7.450722429	-6.450661336	-1.262833388
	H293	6.329401835	-2.795277219	0.653337198
	H294	9.130374611	-4.936736592	1.615513224
	H295	2.123995182	-8.541788428	0.149159357
	H296	7.071021338	-5.767402326	0.783937379
	H297	-2.778382717	2.300950157	9.307492509
	H298	-3.782665501	-0.449013563	10.221653241
	H299	0.189918574	-7.611570211	-0.604651327
	H300	2.638398911	0.177792437	5.152103599
	H301	-1.356899586	-7.816650152	-3.835865486
	H302	-3.776434309	-3.036295530	-1.690316230
	H303	-0.434706029	-6.341643828	-3.536657700
	H304	-1.074961227	2.149264158	7.213360364
	H305	-1.461992167	-6.581779339	-1.089238538
	H306	-2.214485099	0.986503869	6.522876546
	H307	-3.764756445	1.152953750	8.372801482
	H308	-1.835136890	-0.526255615	8.518543259
	H309	-0.837597256	0.738268294	9.259616295
	H310	-4.638161585	-2.561302645	-6.354010707
	H311	-4.699792874	2.306273447	10.964951368
	H312	-7.703656030	1.695587889	10.821695466
	H313	-1.442786610	-3.920730714	-1.575304708
	H314	-6.209864792	3.171252497	12.846794139
	H315	-7.226468444	3.725749916	11.688363658
	H316	-0.493766529	-0.646706613	6.326044969
	H317	-0.054559713	-4.021120157	-3.598577575
	H318	-4.804217725	-2.579356344	-3.910984419
	H319	-6.880498728	-4.044091097	9.843282241
	H320	-6.080788991	-5.849191851	7.195665622
	H321	-5.139546879	-2.424631321	8.947760214
	H322	-8.108653541	-4.488189308	8.985966776
	H323	0.731826628	-10.711034390	-0.994327064
	H324	-2.549356449	-2.928309805	7.294471002
	H325	-5.970938005	0.486765108	5.638968350
	H326	-3.192920084	-7.740403801	0.390512031
	H327	-7.514594954	-1.153460181	6.364947491
	H328	-7.451901690	-6.709526427	2.608667030
	H329	-5.678522626	-4.949699978	2.126936483
	H330	-7.472872439	1.227815954	12.511269471
	H331	1.488357338	-1.103182503	5.360745677
	H332	-2.159256552	-2.970353502	9.635337231
	H333	-1.071901401	-3.412446827	7.350963329
	H334	-2.213371191	-1.401087135	11.414585109
	H335	0.663213691	2.777198728	6.370085693
	H336	-7.453005851	-4.005939731	5.239525499
	H337	-6.907105426	-3.588212479	6.870550258
	H338	-7.880680222	-1.628835712	4.697760622
	H339	-3.510046856	-5.515362579	-0.528051166
	H340	-1.086370941	-3.591785170	-5.848008843
	H341	-3.102995366	-2.920900630	-7.190435046
	H342	-4.420743252	0.020180872	4.911399756
	H343	-3.541627273	-2.961855129	10.347951264
	H344	-5.555705630	-2.048035283	10.385822491
	H345	2.112826460	2.342139754	5.650052514
	H346	-7.316707178	-7.581266735	5.015768081
	H347	-4.498441140	-5.243611863	6.718301045
	H348	-4.952772847	-6.133340177	-1.386996571
	H349	2.784617602	-8.521314848	-1.502121477
	H350	3.912150966	-0.480575688	7.751267043
	H351	3.751618324	-2.035054841	6.897387691
	H352	-5.002883696	-9.331127131	7.473519999
	H353	-6.230173485	-8.306123853	7.826134507
	H354	-4.770795194	-5.664907353	11.344155941
	H355	-5.496912056	-5.953703677	9.200294439
	H356	-4.161501189	-8.317800839	11.114378720
	H357	-3.149695330	-5.439892170	10.655484627
	H358	4.359580926	-3.365921453	9.186209159
	H359	11.804732771	3.126137114	-6.716385584
	H360	-0.979465950	0.344160700	13.536492596
	H361	0.635319578	-0.697232921	11.537976256
	H362	9.743305567	2.145069564	-8.722816531

H363	8.902705495	3.954647416	-6.370451630
H364	8.863203351	4.398891735	-8.087135549
H365	-3.334147929	0.089336532	1.979075398
H366	3.240990161	1.272455346	-8.780907574
H367	-1.858952019	0.477559985	1.280379988
H368	0.759700473	-0.722214810	2.989281099
H369	-1.992855755	0.421068729	2.913382537
H370	-1.229302126	-0.517809914	-0.495274599
Mo371	-3.451294469	-3.573001669	4.197741762
N372	0.215452557	-0.876003182	2.126148779
N373	-2.318954380	-0.012367522	2.046795918
N374	-8.972841633	4.362508705	-2.561109354
N375	-6.423071691	6.656050658	-4.045543735
N376	-5.506508740	8.582051908	-2.216018107
N377	-5.286941035	7.267105036	0.279516349
N378	-3.086236388	5.627086732	-0.332574083
N379	-1.042827674	7.689022852	-0.618202183
N380	-1.049627252	9.246749353	1.841048076
N381	-1.260122087	7.322273442	3.889779650
N382	0.146749111	4.876967501	3.241188121
N383	2.911221746	5.113540191	1.986021485
N384	3.580925506	7.696684735	3.042156203
N385	-6.481719827	3.050755130	11.868539266
N386	-4.721297218	1.290127166	10.830033569
N387	-0.244522793	0.317527155	6.567992250
N388	1.349366188	2.027929633	6.251906767
N389	1.670528447	-0.082136912	5.331866028
N390	-1.950141815	-0.531329495	11.929900644
N391	-5.583932062	0.978708038	-4.437156736
N392	-6.672660128	-0.924825685	-0.227003677
N393	-3.193747870	1.473824551	-5.402662385
N394	-2.628727966	3.510239010	-7.233555376
N395	-1.777208057	5.618955129	-5.572659825
N396	0.652244947	4.698436564	-4.480102641
N397	0.778846485	0.479972288	-3.983772762
N398	-0.644494612	0.865218598	-2.318885589
N399	2.928736762	5.164590524	-5.969757986
N400	3.998733907	5.501994003	-10.583788453
N401	6.053152862	6.326495822	-10.741726448
N402	6.227508180	-2.960216265	7.431852471
N403	8.305255658	-4.333710944	1.681825597
N404	7.867380135	-5.588912861	-1.605963419
N405	6.279634776	-4.534691116	-3.622740001
N406	3.047349307	-2.859176298	-7.190561471
N407	4.154162517	-4.166411743	-8.796000519
N408	4.409466484	-1.866375594	-8.782285781
N409	5.412344378	-1.928924022	-2.742403782
N410	7.884462762	-0.660255579	-2.537733447
N411	9.404138488	-0.550896362	-4.828864485
N412	7.324053791	-2.779601875	-7.296981702
N413	9.847753382	1.450630069	-6.738368219
N414	-6.149609540	-10.153029476	1.152960399
N415	-4.051149551	-7.495478261	-0.111538422
N416	-1.786397267	-6.902370432	-2.021242910
N417	0.710138376	-8.134173587	-1.316229506
N418	-6.569656265	-7.189439998	-4.097029896
N419	-4.970327011	-5.255576833	-5.493905865
N420	-5.488479583	-8.491729838	7.143416210
N421	-5.094850368	-5.266856147	4.178282293
N422	-6.745872078	-6.420704461	3.280491128
N423	-4.597743360	-6.392719368	9.412504533
N424	2.125458924	-0.359951773	-1.224094122
N425	1.651530493	-0.962728408	-0.400215063
O426	-6.348328974	5.477645005	-2.095152879
O427	-3.370207420	7.844746565	-2.492383821
O428	-3.953832062	8.970175878	1.010383315
O429	-2.490101419	5.746533225	1.862462327
O430	0.392788740	6.075793642	0.075674527
O431	1.225812934	9.128388249	1.682765393
O432	-0.360688178	8.823124390	5.381343607
O433	0.034597766	4.320739746	5.463157335
O434	3.286994090	3.721822510	3.770997083
O435	5.541899955	6.737206761	3.663094451
O436	4.809054003	9.172986442	6.066198502
O437	-6.103925914	-0.522281400	11.217430496
O438	-2.211852043	1.744410115	12.116651162
O439	1.229868565	-1.469550673	13.330331682
O440	-2.832942263	3.295758553	-4.045157609
O441	-6.616729304	-0.801628834	-2.497091235
O442	-0.396029312	3.154569390	-6.928400129

	O443 -0.601442990 0.185529355 -7.690155501<br>O444 -1.315543467 6.897669794 -7.424442498<br>O445 0.898610406 6.631448079 -3.286662491<br>O446 4.227190842 4.906284109 -4.115519835<br>O447 5.813966417 7.186955108 -6.305474399<br>O448 4.767594740 -1.768362753 10.391355969<br>O449 6.592465369 1.064857779 2.396928839<br>O450 8.401118400 -3.422213154 -1.138374798<br>O451 7.985391741 -4.671817864 -5.153135060<br>O452 5.402118499 -1.396683986 -4.967715601<br>O453 6.860618982 1.355757375 -2.929079688<br>O454 6.360929945 -0.524033452 -0.054170478<br>O455 11.271254880 0.078999390 -3.656328976<br>O456 11.287671555 0.119954596 -7.900863132<br>O457 8.082224403 -0.795561382 -8.142272109<br>O458 11.659925751 3.924164951 -8.593032670<br>O459 3.673528717 0.977086038 -7.956351849<br>O460 -6.334292564 -7.739324438 -0.182569646<br>O461 -3.618909882 -6.941714007 -3.395148353<br>O462 1.054894235 -8.650718545 -3.505626589<br>O463 2.735679762 -11.095121525 -0.916370016<br>O464 -6.950820812 -4.501626772 -6.336435931<br>O465 -8.379259393 -3.499147426 -0.525363171<br>O466 -7.228369051 -5.262757293 0.216029050<br>O467 -2.115581115 -5.264193557 -7.653689732<br>O468 -2.812013091 -7.650478262 8.810065017<br>O469 -2.715862600 -7.458944681 12.271317055<br>O470 -5.811733670 -0.594406039 2.530421094<br>O471 -6.370117121 1.457155132 3.303177018<br>O472 -9.201956606 -4.061643800 7.697616101<br>O473 -9.971755105 -3.000132316 5.855157528<br>O474 -4.045431404 -3.023894311 6.226297759<br>O475 -5.074971652 -1.493403504 7.512399528<br>O476 -5.383981461 -2.445744954 4.081835724<br>O477 -2.563344709 -2.927657834 10.537560207<br>O478 -1.718329404 -2.860540859 7.828703983<br>O479 1.850763984 -0.327372503 -6.580554413<br>O480 -7.565320955 -4.747362247 9.790500422<br>O481 -5.242136736 -2.823192595 9.855676632<br>S482 4.772717943 -4.324197273 -0.419460397<br>S483 1.427846554 -3.934793976 -0.485384877<br>S484 -3.409542644 -3.242561250 1.844827502<br>S485 2.870561234 -2.203097325 2.000661163<br>S486 -1.214191746 -1.841663128 -0.156788645<br>S487 -1.167421965 -6.431881722 1.117176384<br>S488 -1.938468683 -1.705643420 4.434082171<br>S489 2.318474524 -5.700893168 2.430640164<br>S490 -1.906805620 -5.330644768 4.481648939<br>S491 1.281010124 -3.376816592 5.301534204<br>end

35, S=3/2

reactant

Fe(139) -2.481<br>Fe(140) 0.032<br>Fe(141) -2.526<br>Fe(142) 2.701<br>Fe(143) 1.631<br>Fe(144) 2.353<br>Fe(145) 0.148	bm612n2xnewbrk2bh335td.car_3<br>C1 -6.989354537 5.851746582 -3.109725959<br>C2 -8.447573485 5.456870915 -3.369894251<br>C3 -5.012347210 6.990618097 -4.025526128<br>C4 -4.565980474 7.848056809 -2.847041526<br>C5 -5.180073992 9.356593621 -1.029292219<br>C6 -4.749258674 8.510022533 0.173386286<br>C7 -4.783023433 6.330872030 1.273952854<br>C8 -3.351900865 5.869475606 0.969418600<br>C9 -1.744754501 5.343582256 -0.821487373<br>C10 -0.705277355 6.397196231 -0.411032065<br>C11 -0.055013750 8.720099447 -0.337176360<br>C12 0.104463806 9.035174463 1.155354272<br>C13 -1.026848645 9.669376441 3.229816084<br>C14 -0.835936796 8.558588616 4.267681814<br>C15 -1.263379043 6.197017012 4.798581509<br>C16 -0.283704132 5.060687105 4.517413256<br>C17 1.040156957 3.780021423 2.843772627<br>C18 2.519873987 4.207595647 2.926523722<br>C19 0.676685034 3.260581030 1.441514802<br>C20 1.618278810 2.138572329 0.996258656<br>C21 -0.798324699 2.867710315 1.438699957<br>C22 4.277803898 5.627814815 1.907562494<br>C23 4.523208170 6.724949253 2.952673970<br>C24 4.606696149 6.112973503 0.471238203

	C25	5.980434644	6.794301138	0.409071523
	C26	4.537379139	4.955107693	-0.530572226
	C27	3.822100412	8.845156759	3.895364494
	C28	4.159687681	8.455445653	5.319628578
	C29	-6.972506807	1.688779109	11.651202713
	C30	-5.878128663	0.708118264	11.217111177
	C31	-3.510623463	0.596309261	10.415757362
	C32	-2.489965431	0.661479312	11.569222079
	C33	-2.961782685	1.229368798	9.121146262
	C34	-1.689264147	0.562772641	8.582571969
	C35	-1.337646147	1.085517544	7.184010485
	C36	0.902944852	0.765763077	6.046182339
	C37	-1.017046695	-0.638598047	13.031518603
	C38	0.394073000	-0.966071300	12.593803418
	C39	-4.766004593	1.852509361	-3.596317121
	C40	-3.503362676	2.294115435	-4.361375149
	C41	-4.332021644	1.123248244	-2.303237752
	C42	-5.436015025	0.922556758	-1.243495065
	C43	-6.280578068	-0.344782134	-1.380458438
	C44	-1.905550715	1.468190239	-6.075251928
	C45	-1.575701956	2.782674388	-6.791349273
	C46	-1.883349629	0.266050189	-7.032155618
	C47	-2.446729390	4.826711878	-7.817548242
	C48	-1.788952525	5.873747267	-6.910307354
	C49	-1.060658299	6.482770995	-4.654094578
	C50	0.249037648	5.938290495	-4.081879437
	C51	1.914221561	4.144888443	-4.002569343
	C52	3.132232302	4.803041757	-4.679030191
	C53	2.015403575	2.635815003	-4.276219272
	C54	0.985288991	1.793578302	-3.599106850
	C55	0.091388522	2.009838449	-2.570458151
	C56	-0.211716253	-0.030511867	-3.183099639
	C57	4.017507814	5.626904112	-6.820963995
	C58	4.642058163	6.871537139	-6.196533021
	C59	3.415753021	5.946622008	-8.208020089
	C60	4.377951056	6.017005213	-9.355164817
	C61	5.655315247	6.535987959	-9.438580839
	C62	5.029941530	5.706578380	-11.396065934
	C63	5.537078455	-1.848030868	8.085031933
	C64	4.833932818	-2.371541028	9.322512013
	C65	4.449766355	-1.237714605	7.162013832
	C66	5.030133599	-0.612006480	5.915899070
	C67	5.446316994	0.732037890	5.899709878
	C68	5.178335998	-1.361197614	4.734588480
	C69	5.973350530	1.315480997	4.742688147
	C70	5.716151735	-0.796979673	3.577512513
	C71	6.101658442	0.545315866	3.579836479
	C72	7.316574661	-4.706454832	0.640308728
	C73	7.902217114	-4.505900807	-0.769818109
	C74	6.068728850	-3.850601677	0.800351072
	C75	8.395867140	-5.554754756	-2.951661101
	C76	7.521963783	-4.869706860	-4.005751972
	C77	5.336704878	-3.759173227	-4.422944534
	C78	5.382821447	-2.251443839	-4.062081194
	C79	3.933231429	-4.367065743	-4.195739870
	C80	2.721328953	-3.698081240	-4.853722583
	C81	2.524664332	-3.949694531	-6.358854783
	C82	3.843338855	-2.974741046	-8.249988796
	C83	5.448897311	-0.520491698	-2.335233158
	C84	6.791732984	0.152006765	-2.650575428
	C85	5.171406505	-0.316964790	-0.830216989
	C86	9.212994054	-0.096361140	-2.423448674
	C87	10.057920403	-0.182546912	-3.683985291
	C88	10.161500334	-0.900512580	-6.005632943
	C89	10.465611001	0.268830979	-6.974842091
	C90	9.598187056	-2.097825677	-6.778681311
	C91	8.266013090	-1.814445155	-7.458307988
	C92	9.886420010	2.541214719	-7.701417348
	C93	11.233352137	3.244999556	-7.666758685
	C94	8.750939998	3.558348378	-7.384436483
	C95	7.372468985	2.947701468	-7.513757627
	C96	6.704001610	2.955554258	-8.748700926
	C97	6.741344336	2.314834539	-6.429173246
	C98	5.461816305	2.336797568	-8.906485266
	C99	5.511494305	1.666461171	-6.573969257
	C100	4.884466466	1.680894800	-7.818032547
	C101	-5.085511357	-9.166572830	1.312164895
	C102	-5.209824784	-8.079141122	0.260126764
	C103	-3.945653057	-6.384717960	-1.042846248
	C104	-3.106457469	-6.760262236	-2.271024172

C105	-0.831150684	-7.254485449	-3.058127752
C106	0.377992825	-8.081654304	-2.632462010
C107	1.914524502	-8.815546675	-0.890435674
C108	1.782032562	-10.325959647	-0.946785489
C109	-7.068050115	-5.822573519	-4.318005832
C110	-6.337420321	-5.141007783	-5.480598635
C111	-7.083255560	-4.893960805	-3.082710182
C112	-8.125090174	-5.361305390	-2.048831369
C113	-7.947642599	-4.627561853	-0.738040063
C114	-4.209365199	-4.664399335	-6.581198338
C115	-3.109955424	-5.607924560	-7.027943580
C116	-3.748805556	-3.203107336	-6.346723097
C117	-3.030048360	-3.121129160	-5.034339224
C118	-1.674436438	-3.472811180	-4.942768950
C119	-3.768738051	-2.922958759	-3.853624428
C120	-1.105003605	-3.739774040	-3.695186343
C121	-3.198031921	-3.194464150	-2.610726509
C122	-1.882364662	-3.663980288	-2.540043781
C123	-4.538258789	-7.362588022	7.126257389
C124	-3.905821693	-7.132388305	8.505795669
C125	-5.189596981	-6.082380854	6.582747330
C126	-5.616561609	-6.118605056	5.149962901
C127	-6.649474774	-6.841469382	4.590214934
C128	-5.804981618	-5.474435375	3.065680122
C129	-4.002526912	-6.135435445	10.710052369
C130	-3.576553932	-7.411802891	11.401168629
C131	-5.974496534	0.281623387	3.478144143
C132	-5.505044240	-0.163791263	4.877030917
C133	-5.769031204	-1.655026524	5.216146096
C134	-7.258304760	-1.883800933	5.572421055
C135	-7.593364807	-3.294015362	6.065575699
C136	-9.041325955	-3.452439130	6.580531616
C137	-4.907558725	-2.073505001	6.415703716
C138	-0.219295407	-3.491147523	2.292517000
Fe139	-1.728735877	-4.760933932	2.368202705
Fe140	0.753901093	-2.032400150	0.796122069
Fe141	1.585326166	-3.607167628	3.125414953
Fe142	0.511471902	-4.960654541	1.213886494
Fe143	3.028887514	-4.083330327	0.993543806
Fe144	-0.695006812	-3.435287060	4.246126577
Fe145	-1.668455393	-1.804122770	2.112639011
H146	-8.829081448	4.571462074	-1.571153598
H147	-8.447503578	3.509571974	-2.749049821
H148	-6.946055149	6.845348653	-4.909613660
H149	-8.564530939	5.210106302	-4.437128699
H150	-9.057243189	6.360339145	-3.197243851
H151	-4.403439475	6.074285898	-3.990995156
H152	-6.466154641	8.549225937	-2.555813837
H153	-4.758118525	7.522766427	-4.951461093
H154	-6.058071677	9.952243310	-0.747776269
H155	-5.878895075	6.906465000	-0.474366757
H156	-4.351170393	10.047686082	-1.234230948
H157	-1.394804059	4.378523811	-0.445858089
H158	-3.834263656	5.739719565	-1.016780583
H159	-5.451266709	5.459238058	1.305731666
H160	0.927102930	8.399253672	-0.706369684
H161	-1.893866569	7.914874694	-1.139281426
H162	-4.774267230	6.796412270	2.266442845
H163	-1.799409744	5.277625544	-1.915492054
H164	-1.953359988	9.171993081	1.364299324
H165	-0.352167792	9.637421776	-0.862767872
H166	-1.455445017	3.740934522	1.511182558
H167	-1.059380709	2.312264069	0.524024294
H168	-1.020044796	2.260375470	2.320355206
H169	0.792524119	4.090141912	0.729696639
H170	-1.976704927	10.175266213	3.454097375
H171	3.552579575	4.467269264	-0.536031246
H172	2.776775833	7.738116143	2.415359196
H173	6.033048118	7.688418394	1.047009681
H174	1.352870058	1.814731848	-0.018101408
H175	1.571131720	1.256430730	1.651249314
H176	6.208038396	-1.028285901	8.405550143
H177	4.730678055	5.316380787	-1.550233732
H178	6.194355739	7.100938935	-0.625267895
H179	5.294505314	4.190844619	-0.292914811
H180	6.775160763	6.102751008	0.730313478
H181	3.844465629	6.863785208	0.190530360
H182	4.960378413	4.808072495	2.176829324
H183	2.218910992	5.474270421	1.329069667
H184	2.665515142	2.476781230	0.964677350

H185	4.377910438	-1.008260970	-0.510268764
H186	4.805339842	0.713620584	-0.711479797
H187	6.322564777	0.074321063	0.720611678
H188	6.778784608	2.015616997	2.508629226
H189	5.799747184	-1.382647530	2.662677797
H190	4.837319878	-2.398191252	4.706314878
H191	5.342738927	1.341916106	6.800765630
H192	-0.199464473	5.502727661	2.513729974
H193	-2.265817889	5.741427182	4.821088110
H194	-1.033980500	6.565464416	5.805190419
H195	-1.751996258	7.201519971	3.001958436
H196	-0.210748466	10.386899615	3.386600998
H197	6.263787316	2.369274490	4.740278950
H198	0.917107600	2.978418416	3.583008446
H199	4.629060512	9.488023577	3.506368639
H200	2.895555951	9.439018457	3.922824119
H201	3.720391021	7.491328744	5.666106137
H202	-1.351890810	-1.384696819	13.768939450
H203	-7.262474364	-1.756139449	-0.289164297
H204	-5.523203910	-1.774058440	3.251234871
H205	-9.134180101	-5.146958856	-2.428785517
H206	-8.041608094	-6.445874789	-1.887344693
H207	-7.316830768	-3.860607754	-3.382696310
H208	-3.290491640	-6.679978229	-6.776825534
H209	-8.104070653	-5.905475802	-4.680252342
H210	-3.885057082	0.154444918	-2.577271644
H211	-6.135280448	1.776989871	-1.262138235
H212	-4.491249214	-5.758740390	-4.755311809
H213	-4.900256232	-4.641309841	-7.449547231
H214	-5.658488724	-7.192070508	-3.647636816
H215	-6.353935848	-0.623626948	0.708330018
H216	-6.075948393	-4.872422939	-2.639616917
H217	-7.217591600	-7.692453955	-3.485106942
H218	-6.298811131	1.532100334	-4.942824660
H219	-3.524680932	1.711072835	-1.844913403
H220	-6.935992212	-6.178907562	-0.082957962
H221	-4.979881228	0.934943675	-0.242715236
H222	2.008612734	4.353243313	-2.928068480
H223	-0.065944733	2.927031583	-2.012165634
H224	-0.807954458	7.406729700	-5.192468562
H225	-2.195051032	4.754824785	-5.201061348
H226	1.994031464	5.091489765	-6.362463688
H227	6.303483211	6.999305119	-8.702196409
H228	3.908166656	7.512831765	-5.650725288
H229	2.819297939	6.873092649	-8.134353448
H230	4.792448004	4.849167576	-6.909668269
H231	5.084800887	5.427830238	-12.445067926
H232	3.027324811	2.324918379	-3.965359745
H233	1.960184943	2.474991597	-5.365961401
H234	6.953527431	6.584921020	-11.137719619
H235	2.703369778	5.141508754	-8.448359035
H236	0.102720151	4.166979360	-5.161103453
H237	-1.702088989	6.775851312	-3.809052327
H238	-1.814115549	4.768024765	-8.714280966
H239	-0.552813507	-1.060604781	-3.256623727
H240	-3.427959529	5.208251404	-8.128594190
H241	7.073859404	-3.195765993	7.933712320
H242	6.516185152	-2.675280828	6.487909145
H243	5.221577347	-2.644208363	-2.010577796
H244	4.683352434	0.029570795	-2.901119897
H245	7.746218838	-1.582460090	-2.113220074
H246	1.248032097	0.023714618	-4.771579875
H247	0.965152236	-0.124050105	-6.988614786
H248	-0.708465044	-0.426462030	-8.439829090
H249	2.475081973	0.351369601	-6.927390901
H250	-2.070748159	-0.644396762	-6.439160355
H251	-2.681303298	0.376945206	-7.783226660
H252	9.541775469	-2.972288270	-6.115372499
H253	10.322318945	-2.326850646	-7.575641913
H254	7.439145631	-3.479968795	-6.551966383
H255	6.379181642	-2.501448813	-7.564127657
H256	4.972546167	-4.251026118	-9.389127595
H257	1.453167905	-4.059921389	-6.583623695
H258	4.951336846	2.362776881	-9.873153937
H259	7.197464942	2.323639218	-5.435743113
H260	4.171700397	-0.943773018	-8.407136945
H261	3.749502323	-4.370873983	-3.109096527
H262	5.999996345	-4.737203811	-2.648854461
H263	2.707534262	-2.615242165	-4.652398866
H264	-3.565994938	3.175964691	-7.020682783

	H265	8.577740532	-6.584960405	-3.286152310
	H266	-3.896722400	0.724172567	-5.516168987
	H267	3.975644699	-5.425120094	-4.503273586
	H268	5.069732670	1.131607839	-5.732950266
	H269	9.359627241	-5.025350844	-2.956777390
	H270	3.769196981	-5.025144824	-8.417839632
	H271	4.721930799	-1.894422882	-9.747856028
	H272	-6.076228425	0.287443551	-3.848075402
	H273	-5.270253371	2.791418107	-3.298596979
	H274	7.156571208	3.454982949	-9.608199882
	H275	-1.102743859	1.343899597	-5.333854763
	H276	5.636800567	-3.835107007	-5.471854542
	H277	3.004324988	-4.896544430	-6.640058058
	H278	1.839051817	-4.106774757	-4.343971793
	H279	2.667307945	-1.913950232	-6.973474668
	H280	9.139159551	0.965952341	-2.145182842
	H281	8.430740737	-0.861948280	-4.719994880
	H282	11.160437197	-1.194634304	-5.647244933
	H283	9.766340372	-0.615992692	-1.629612350
	H284	9.106022493	1.444063938	-6.032630243
	H285	-4.106996629	-9.662475541	1.227089780
	H286	-5.097079173	-8.643272718	2.290141893
	H287	-3.700170714	-7.648771804	6.473073593
	H288	-6.227160202	-10.708627768	2.007587897
	H289	-7.044951017	-9.655911567	1.056160939
	H290	5.687536798	-3.994443255	1.821202902
	H291	8.623358806	-3.385771518	1.485564779
	H292	7.444341207	-6.451187306	-1.260719844
	H293	6.321198209	-2.789946213	0.656868523
	H294	9.113867464	-4.939970347	1.612191063
	H295	2.115360685	-8.535640620	0.156151003
	H296	7.050157023	-5.765393715	0.785481289
	H297	-2.779514039	2.301181398	9.306796809
	H298	-3.786418307	-0.447777646	10.221250306
	H299	0.183940659	-7.609768359	-0.603366842
	H300	2.623554941	0.174149567	5.129783236
	H301	-1.357576521	-7.817837799	-3.838029285
	H302	-3.774985702	-3.044024359	-1.696039489
	H303	-0.435543662	-6.342595614	-3.538769297
	H304	-1.072305489	2.149542222	7.214781018
	H305	-1.460566444	-6.594257919	-1.088468199
	H306	-2.216521156	0.990594743	6.524570546
	H307	-3.765258885	1.153109108	8.371392421
	H308	-1.835889246	-0.526240885	8.517879864
	H309	-0.838538720	0.737991052	9.260217988
	H310	-4.641653825	-2.561486231	-6.357828185
	H311	-4.701164027	2.308237643	10.966022025
	H312	-7.707989414	1.698241849	10.832365181
	H313	-1.442273125	-3.934692019	-1.584349240
	H314	-6.210755567	3.177511434	12.851612153
	H315	-7.228606807	3.729452771	11.692846620
	H316	-0.493152995	-0.651491823	6.335612760
	H317	-0.055539028	-4.023779520	-3.606689139
	H318	-4.805269864	-2.582994453	-3.914453436
	H319	-6.885107929	-4.043851989	9.847828601
	H320	-6.078920015	-5.846144589	7.192196545
	H321	-5.142819361	-2.425401675	8.950627322
	H322	-8.112121073	-4.487491346	8.989247230
	H323	0.732101425	-10.705060710	-0.994052845
	H324	-2.552067573	-2.931005500	7.298012823
	H325	-5.965487414	0.477446877	5.638477146
	H326	-3.193139096	-7.745076091	0.392570954
	H327	-7.505467969	-1.155972376	6.361125070
	H328	-7.456675960	-6.709661013	2.611076597
	H329	-5.688844603	-4.950371416	2.125146618
	H330	-7.472229383	1.231418458	12.521857907
	H331	1.484227568	-1.110162532	5.361899951
	H332	-2.160863471	-2.971469008	9.634625319
	H333	-1.072707989	-3.406646948	7.342685790
	H334	-2.215024426	-1.400569271	11.412392470
	H335	0.669732148	2.775705145	6.376865457
	H336	-7.453064320	-4.009451245	5.239910587
	H337	-6.906680608	-3.590542808	6.870707459
	H338	-7.873626273	-1.631617846	4.694888813
	H339	-3.507001000	-5.517001485	-0.526042309
	H340	-1.088027389	-3.590062132	-5.856455994
	H341	-3.107484300	-2.922042371	-7.196187268
	H342	-4.417298449	0.018120108	4.899629821
	H343	-3.543436827	-2.960850095	10.348413879
	H344	-5.560332450	-2.044357097	10.387937603

	H345	2.116839129	2.336181856	5.648428540
	H346	-7.316728826	-7.579850027	5.020104242
	H347	-4.495629298	-5.243296018	6.716670631
	H348	-4.951585888	-6.132198420	-1.382534005
	H349	2.784043116	-8.512290482	-1.493334605
	H350	3.904071892	-0.481586111	7.746829443
	H351	3.738058891	-2.033269751	6.887443610
	H352	-5.004994926	-9.329649039	7.471926981
	H353	-6.230943976	-8.303199828	7.825410098
	H354	-4.771399306	-5.664883176	11.343827551
	H355	-5.497775024	-5.953004282	9.199863639
	H356	-4.162899722	-8.317589547	11.114651495
	H357	-3.150345703	-5.439734290	10.654955541
	H358	4.354449081	-3.371028296	9.186256591
	H359	11.803883216	3.123992286	-6.715089525
	H360	-0.980376769	0.343957303	13.534485152
	H361	0.636032303	-0.697080734	11.536455465
	H362	9.739536812	2.141942967	-8.717848636
	H363	8.900672116	3.955892044	-6.368270475
	H364	8.863664962	4.397900308	-8.085652465
	H365	-3.021169278	-1.125127515	2.040293350
	H366	3.242236316	1.273438516	-8.784138349
	H367	-1.285542075	0.513748804	1.240705958
	H368	0.423698130	-0.685037112	3.139945932
	H369	-1.432483864	0.483107651	2.955489540
	H370	-1.346507805	-0.534374886	-0.557789859
	Mo371	-3.449088090	-3.606249380	4.213313435
	N372	0.053920017	-0.695942056	2.182979444
	N373	-1.107494185	0.014370454	2.110079042
	N374	-8.972822388	4.367510429	-2.563324187
	N375	-6.429767986	6.667748232	-4.051966861
	N376	-5.508033675	8.585697193	-2.216966171
	N377	-5.287856227	7.267552539	0.277619459
	N378	-3.087747335	5.629039574	-0.335631409
	N379	-1.041862962	7.690066513	-0.620270645
	N380	-1.050262618	9.247271507	1.840120270
	N381	-1.259185277	7.322064834	3.888642985
	N382	0.145006490	4.880401278	3.243756668
	N383	2.911658521	5.113848188	1.986267630
	N384	3.579839161	7.697935294	3.043711960
	N385	-6.483685535	3.054956572	11.874003520
	N386	-4.724552350	1.292367965	10.830164929
	N387	-0.248387102	0.317267359	6.564374142
	N388	1.349782811	2.022941573	6.246252537
	N389	1.652980043	-0.083146916	5.305635744
	N390	-1.950975303	-0.530693998	11.927281171
	N391	-5.593047403	0.985087111	-4.444705610
	N392	-6.671020838	-0.922282850	-0.229679747
	N393	-3.196757465	1.475994949	-5.403590673
	N394	-2.628971512	3.510234369	-7.234189094
	N395	-1.777492902	5.618864927	-5.573374460
	N396	0.652118142	4.698690384	-4.481082589
	N397	0.773651520	0.481524137	-3.979877110
	N398	-0.648020475	0.873837679	-2.315657534
	N399	2.929836507	5.166442922	-5.971347844
	N400	3.998699634	5.502976934	-10.585012710
	N401	6.053354590	6.327031673	-10.742361884
	N402	6.217974654	-2.963764898	7.423749234
	N403	8.290064152	-4.335359113	1.682267966
	N404	7.860604627	-5.588614371	-1.601994577
	N405	6.275519906	-4.534596861	-3.617125977
	N406	3.047238789	-2.859518213	-7.189582888
	N407	4.152468591	-4.166524235	-8.796402982
	N408	4.409374436	-1.866557649	-8.781321325
	N409	5.410632437	-1.928395038	-2.738402564
	N410	7.882082972	-0.659430561	-2.533733765
	N411	9.397450102	-0.552008238	-4.823847340
	N412	7.321066320	-2.781786453	-7.297673702
	N413	9.843829244	1.449809397	-6.732832857
	N414	-6.155836565	-10.155983405	1.150546292
	N415	-4.051055617	-7.496567197	-0.107990848
	N416	-1.787609105	-6.903110684	-2.023044838
	N417	0.708387583	-8.128224476	-1.316712854
	N418	-6.578102070	-7.191262197	-4.105594085
	N419	-4.979462484	-5.257821683	-5.504531757
	N420	-5.489654403	-8.489548183	7.142434796
	N421	-5.090553431	-5.273563236	4.172984538
	N422	-6.749319728	-6.421223504	3.281837548
	N423	-4.598938433	-6.392509839	9.412153441
	N424	2.090374086	-0.375926937	-1.226514115

	N425	1.568410343	-0.972840382	-0.424772955
	O426	-6.360441459	5.498433962	-2.097824141
	O427	-3.373387389	7.842964522	-2.492940922
	O428	-3.953963738	8.969545109	1.009460172
	O429	-2.493104169	5.739898854	1.861135906
	O430	0.389313186	6.075324448	0.079650975
	O431	1.225124138	9.128425738	1.682414075
	O432	-0.358866703	8.822830977	5.379608611
	O433	0.029245982	4.317669473	5.463576030
	O434	3.284122951	3.725489956	3.775355581
	O435	5.541605086	6.738341858	3.662900129
	O436	4.806124598	9.172786759	6.068833750
	O437	-6.107384872	-0.518638099	11.221936289
	O438	-2.213651497	1.744823068	12.115748131
	O439	1.229187683	-1.467624059	13.329957580
	O440	-2.839389958	3.301790437	-4.050079122
	O441	-6.617486721	-0.799392149	-2.500199430
	O442	-0.396526216	3.154317503	-6.927045369
	O443	-0.604341291	0.187313362	-7.690920792
	O444	-1.316378393	6.897906227	-7.425035158
	O445	0.897186233	6.630810246	-3.285733118
	O446	4.226701876	4.908057678	-4.115522087
	O447	5.815187590	7.188181509	-6.304888322
	O448	4.766440456	-1.769342304	10.384536554
	O449	6.591748333	1.065767250	2.396257874
	O450	8.388654394	-3.421159552	-1.133062884
	O451	7.980294644	-4.671907528	-5.148950704
	O452	5.400294911	-1.398138050	-4.964272507
	O453	6.858681645	1.355973638	-2.928375985
	O454	6.357181282	-0.523943252	-0.053029561
	O455	11.267262212	0.078647752	-3.655903642
	O456	11.282191970	0.117280135	-7.894833598
	O457	8.080519618	-0.797511688	-8.141688844
	O458	11.655621407	3.921218841	-8.591863730
	O459	3.673953673	0.978716177	-7.959009225
	O460	-6.334144316	-7.740755974	-0.184575402
	O461	-3.623341955	-6.940585001	-3.393597392
	O462	1.054816257	-8.651958679	-3.504926875
	O463	2.735844192	-11.086708086	-0.917308389
	O464	-6.959139435	-4.500047971	-6.344703317
	O465	-8.378673374	-3.499665774	-0.529515770
	O466	-7.227684212	-5.263006818	0.211925067
	O467	-2.120005228	-5.264986900	-7.657597175
	O468	-2.813032519	-7.650191205	8.810716171
	O469	-2.716793009	-7.458852651	12.270771144
	O470	-5.876775014	-0.621544849	2.542233842
	O471	-6.351977948	1.455809139	3.301816732
	O472	-9.201371622	-4.060875347	7.697369939
	O473	-9.970353263	-2.998101737	5.855478927
	O474	-4.048721686	-3.042201081	6.233511413
	O475	-5.068244218	-1.497726261	7.509145052
	O476	-5.373821917	-2.455102926	4.079126989
	O477	-2.564984843	-2.926742597	10.536744659
	O478	-1.718671892	-2.860641102	7.828511339
	O479	1.849744151	-0.326614888	-6.585957561
	O480	-7.571718792	-4.745464294	9.796180265
	O481	-5.247260069	-2.820936237	9.859715282
	S482	4.746810193	-4.300053188	-0.405572997
	S483	1.418881433	-3.858918351	-0.525239518
	S484	-3.390102769	-3.345513089	1.851073670
	S485	2.802465896	-2.114775673	2.014201756
	S486	-1.322216193	-1.835202210	-0.126529237
	S487	-1.091195838	-6.494621174	1.127846789
	S488	-2.035934408	-1.644111913	4.420540798
	S489	2.324830894	-5.612755530	2.423100929
	S490	-1.872965689	-5.318731372	4.567832248
	S491	1.229669536	-3.328201066	5.301428903
	end			

TS

Fe(139) -2.319	bm612n2xnewbrk2bh335te_1_53388.82			
Fe(140) 0.017	C1	-6.985996511	5.844119298	-3.110179313
Fe(141) -2.566	C2	-8.445726854	5.456955940	-3.374495484
Fe(142) 2.736	C3	-5.003876548	6.978010369	-4.020461043
Fe(143) 1.640	C4	-4.556818047	7.833432417	-2.840865545
Fe(144) 2.291	C5	-5.169734864	9.350257707	-1.029286512
	C6	-4.742998488	8.502481133	0.173756899
	C7	-4.794680692	6.327495968	1.280453439
	C8	-3.364024246	5.861110002	0.977344881
	C9	-1.758858215	5.324649828	-0.810369603

Fe(145) 0.066	C10	-0.717246834	6.377323623	-0.402609706
	C11	-0.052046706	8.692315930	-0.321485853
	C12	0.104480762	9.017143391	1.169187451
	C13	-1.030447731	9.668898251	3.237189605
	C14	-0.851137448	8.558263051	4.276784375
	C15	-1.251012998	6.190083662	4.792162250
	C16	-0.265644080	5.063258268	4.493482872
	C17	1.047611462	3.775412884	2.826920828
	C18	2.528987909	4.198175480	2.897424118
	C19	0.687943320	3.253718618	1.420090339
	C20	1.632429929	2.140676393	0.962077450
	C21	-0.782386162	2.842232921	1.406317057
	C22	4.281776494	5.632419693	1.892139272
	C23	4.525340799	6.720185999	2.943826635
	C24	4.616968781	6.125353316	0.459998488
	C25	5.992475350	6.804838122	0.406549608
	C26	4.547216041	4.968007492	-0.541678518
	C27	3.833204440	8.839502321	3.879482332
	C28	4.174787482	8.463199853	5.305288876
	C29	-6.980397859	1.692719207	11.646755016
	C30	-5.885585207	0.708653250	11.221164257
	C31	-3.516251576	0.594156878	10.426224117
	C32	-2.494978963	0.660595038	11.578496244
	C33	-2.966615979	1.223205039	9.129815813
	C34	-1.695639973	0.552407385	8.590312855
	C35	-1.355635455	1.067160976	7.184595231
	C36	0.896861756	0.784724969	6.050748077
	C37	-1.018194743	-0.638883228	13.036972682
	C38	0.390175333	-0.970709903	12.594600406
	C39	-4.757589322	1.848309259	-3.594353859
	C40	-3.489916271	2.283543097	-4.355293090
	C41	-4.330304311	1.123224539	-2.297038867
	C42	-5.438627073	0.924493423	-1.241513753
	C43	-6.282768412	-0.343209869	-1.380977460
	C44	-1.905052196	1.468784490	-6.080227894
	C45	-1.575633884	2.786267367	-6.791808699
	C46	-1.879637649	0.266764310	-7.036857847
	C47	-2.449662739	4.833593647	-7.811640836
	C48	-1.786668600	5.877687259	-6.904856526
	C49	-1.062149412	6.485227489	-4.645804763
	C50	0.246917910	5.938577194	-4.074998465
	C51	1.908912530	4.142919146	-4.000341209
	C52	3.127905065	4.798008124	-4.676609982
	C53	2.009259306	2.634467975	-4.275849666
	C54	0.984941584	1.783230184	-3.601372182
	C55	0.104802201	1.974260149	-2.555571687
	C56	-0.178603097	-0.063080108	-3.187163428
	C57	4.011074316	5.623757639	-6.815344367
	C58	4.632296773	6.873186897	-6.195497728
	C59	3.411525934	5.947434725	-8.204254170
	C60	4.373920207	6.016307500	-9.352286676
	C61	5.652100053	6.533230754	-9.436843128
	C62	5.024313575	5.705117820	-11.394105160
	C63	5.541950408	-1.847305767	8.086207370
	C64	4.838018189	-2.367511753	9.324844272
	C65	4.455878230	-1.237700301	7.162570866
	C66	5.036027904	-0.611115857	5.916795866
	C67	5.452840664	0.732808936	5.901617305
	C68	5.182698312	-1.359123784	4.734483322
	C69	5.979329792	1.317100131	4.744823920
	C70	5.719773110	-0.794050000	3.577538414
	C71	6.106746495	0.548038674	3.581193510
	C72	7.317347326	-4.701676479	0.639770218
	C73	7.902045150	-4.502291383	-0.771103334
	C74	6.065448066	-3.851299023	0.799438743
	C75	8.394959891	-5.554031023	-2.951307523
	C76	7.520515191	-4.866830433	-4.003523103
	C77	5.332947526	-3.760544981	-4.419957986
	C78	5.381395471	-2.252850054	-4.061413600
	C79	3.929197258	-4.366349571	-4.192801903
	C80	2.718660704	-3.694794179	-4.850769886
	C81	2.522098934	-3.946018585	-6.355817347
	C82	3.842062405	-2.970556248	-8.243415377
	C83	5.451305734	-0.519429061	-2.338505236
	C84	6.794918035	0.150878698	-2.653450648
	C85	5.172390860	-0.311698369	-0.836196095
	C86	9.215777620	-0.101103739	-2.424853375
	C87	10.056880114	-0.184759447	-3.687386260
	C88	10.155873331	-0.903594379	-6.006095573
	C89	10.463582456	0.267513488	-6.973198432

	C90	9.594929629	-2.098332594	-6.784205649
	C91	8.262011690	-1.812233936	-7.460529473
	C92	9.881803192	2.538716080	-7.698345905
	C93	11.229182863	3.243849906	-7.662177038
	C94	8.748387571	3.558270593	-7.385946524
	C95	7.370042912	2.948482274	-7.516005681
	C96	6.699707424	2.957481783	-8.749901907
	C97	6.741657362	2.313045223	-6.431615516
	C98	5.457463570	2.337858098	-8.906253496
	C99	5.512677446	1.663011690	-6.575231245
	C100	4.882599825	1.679518757	-7.817604072
	C101	-5.087794736	-9.167174640	1.307769872
	C102	-5.210161045	-8.077418376	0.256637946
	C103	-3.947055574	-6.376997118	-1.042401260
	C104	-3.103751710	-6.748105636	-2.269335869
	C105	-0.829081391	-7.241721039	-3.056305571
	C106	0.380158409	-8.070715793	-2.632842868
	C107	1.917290089	-8.813499233	-0.894333628
	C108	1.778685591	-10.323317461	-0.932079492
	C109	-7.064043449	-5.822225037	-4.316370429
	C110	-6.332281984	-5.140900915	-5.478371292
	C111	-7.081123495	-4.891130085	-3.082097607
	C112	-8.121864514	-5.359129617	-2.047435931
	C113	-7.945397319	-4.626014677	-0.736177292
	C114	-4.204529025	-4.660329911	-6.577322217
	C115	-3.105570470	-5.603110711	-7.026286181
	C116	-3.742705825	-3.200670483	-6.338074183
	C117	-3.031432879	-3.116238356	-5.021658179
	C118	-1.675885791	-3.465174216	-4.920745265
	C119	-3.776036639	-2.907231305	-3.846421152
	C120	-1.110113100	-3.714724759	-3.668471081
	C121	-3.209526271	-3.160631785	-2.598658114
	C122	-1.893105254	-3.626378173	-2.518756134
	C123	-4.531409016	-7.361567648	7.127168536
	C124	-3.900506280	-7.131041631	8.508049238
	C125	-5.189452132	-6.084355397	6.584359476
	C126	-5.620416054	-6.122776658	5.152618255
	C127	-6.651212850	-6.848998211	4.593588525
	C128	-5.813601799	-5.479304915	3.067819528
	C129	-4.006595199	-6.137003829	10.715916312
	C130	-3.576440786	-7.412982807	11.404292406
	C131	-5.984646406	0.287621570	3.474474703
	C132	-5.519507782	-0.161745613	4.874715093
	C133	-5.785186681	-1.652648114	5.215659443
	C134	-7.274644864	-1.881080024	5.571362327
	C135	-7.605648543	-3.293103617	6.062267326
	C136	-9.052850316	-3.457144759	6.576866473
	C137	-4.921586291	-2.072323070	6.414754812
	C138	-0.218490985	-3.496363694	2.299427351
	Fe139	-1.746878395	-4.781051131	2.365904510
	Fe140	0.776087536	-1.966653490	0.725073884
	Fe141	1.577203740	-3.613784979	3.133359588
	Fe142	0.509369448	-4.970408888	1.211152630
	Fe143	3.023169931	-4.106166892	0.998525192
	Fe144	-0.694670033	-3.479631383	4.266640715
	Fe145	-1.506744235	-2.044390069	2.085488700
	H146	-8.837411464	4.569654562	-1.579047745
	H147	-8.455811368	3.508872827	-2.758090635
	H148	-6.936786318	6.843277157	-4.907411034
	H149	-8.561193629	5.213476969	-4.442648550
	H150	-9.052171209	6.362834519	-3.201934235
	H151	-4.399647191	6.058829909	-3.984493384
	H152	-6.453175524	8.549145778	-2.561349835
	H153	-4.744987571	7.509147468	-4.945774507
	H154	-6.047971696	9.946393204	-0.749628630
	H155	-5.886055073	6.907846879	-0.469600178
	H156	-4.339841063	10.040724540	-1.232543232
	H157	-1.409965827	4.361197373	-0.431049723
	H158	-3.852759504	5.698845475	-1.006237109
	H159	-5.467691215	5.459593217	1.311888941
	H160	0.927765456	8.354829976	-0.681629718
	H161	-1.902826645	7.904155359	-1.117055199
	H162	-4.785942739	6.794297788	2.272463728
	H163	-1.812157072	5.253099180	-1.903828096
	H164	-1.952935081	9.160134302	1.372480125
	H165	-0.333778965	9.609325275	-0.855341789
	H166	-1.449482649	3.704123364	1.517400472
	H167	-1.039036208	2.324983293	0.467054657
	H168	-0.991216439	2.196967824	2.263682801
	H169	0.796126853	4.090094770	0.714859635

	H170	-1.976099000	10.185075904	3.455309397
	H171	3.557273973	4.491202280	-0.552990417
	H172	2.789319172	7.748406869	2.393209617
	H173	6.042827588	7.697986005	1.046376917
	H174	1.349448234	1.817244520	-0.047775295
	H175	1.596406820	1.257947776	1.615849396
	H176	6.213580028	-1.027848641	8.406310189
	H177	4.753514789	5.322636009	-1.560845098
	H178	6.212668127	7.114093485	-0.625724528
	H179	5.292741463	4.195972380	-0.292322750
	H180	6.785388389	6.112468542	0.730453344
	H181	3.858422329	6.879439210	0.177693407
	H182	4.960546054	4.808780405	2.157413059
	H183	2.215949092	5.501289980	1.328504388
	H184	2.674412983	2.489505400	0.909316351
	H185	4.379187305	-1.002240601	-0.514356667
	H186	4.806071719	0.718742643	-0.713946858
	H187	6.317330901	0.081526590	0.718654307
	H188	6.785691533	2.018573967	2.512146694
	H189	5.802610304	-1.378769488	2.661950145
	H190	4.841769889	-2.396160152	4.706037378
	H191	5.350463804	1.341827572	6.803450658
	H192	-0.191860686	5.499708200	2.483979301
	H193	-2.248923940	5.724939534	4.827043588
	H194	-1.009860706	6.552895598	5.797980852
	H195	-1.760239907	7.200297505	3.005173335
	H196	-0.208042066	10.378825907	3.395708260
	H197	6.269501682	2.370978390	4.742810273
	H198	0.921826319	2.978896840	3.570917015
	H199	4.642797728	9.476851482	3.486449324
	H200	2.913012816	9.442805859	3.906251833
	H201	3.725699188	7.509988882	5.667876385
	H202	-1.352207203	-1.381305309	13.778213657
	H203	-7.265442026	-1.756948555	-0.292601639
	H204	-5.541330295	-1.785091280	3.255247825
	H205	-9.131368406	-5.145571113	-2.426707356
	H206	-8.037132963	-6.443474579	-1.885669836
	H207	-7.317032114	-3.858740029	-3.383589073
	H208	-3.289267961	-6.676247502	-6.782353425
	H209	-8.099665473	-5.906826449	-4.679241995
	H210	-3.880796044	0.154446885	-2.566543005
	H211	-6.137120416	1.779558836	-1.261939013
	H212	-4.484764199	-5.753043954	-4.749898750
	H213	-4.896301163	-4.634649422	-7.444865118
	H214	-5.651763759	-7.187526456	-3.643098729
	H215	-6.367705586	-0.618561919	0.708063932
	H216	-6.073917343	-4.866209337	-2.638768823
	H217	-7.209903407	-7.690289862	-3.478520671
	H218	-6.289279665	1.529791561	-4.943984054
	H219	-3.526117295	1.715361804	-1.838445484
	H220	-6.935458804	-6.177946084	-0.080672886
	H221	-4.986159743	0.937864302	-0.238889042
	H222	2.003299196	4.351410771	-2.926115851
	H223	-0.054474786	2.878731792	-1.977656952
	H224	-0.810762849	7.411843031	-5.180233835
	H225	-2.191429710	4.756727051	-5.197741395
	H226	1.988708728	5.090093109	-6.360261257
	H227	6.301307573	6.995729218	-8.700936775
	H228	3.897349580	7.513567842	-5.649823177
	H229	2.818809650	6.876580173	-8.131208468
	H230	4.789320321	4.849657015	-6.905822448
	H231	5.078469313	5.426083733	-12.443151070
	H232	3.021496450	2.324536702	-3.964806387
	H233	1.955695215	2.475456930	-5.366009977
	H234	6.950061728	6.578918345	-11.136428394
	H235	2.695864434	5.146079414	-8.446981116
	H236	0.102710706	4.174597380	-5.166449051
	H237	-1.703925402	6.773418618	-3.799410303
	H238	-1.824048156	4.778056635	-8.713371153
	H239	-0.497995481	-1.100358191	-3.262888009
	H240	-3.433010169	5.216854439	-8.113764945
	H241	7.080784357	-3.192567163	7.938925927
	H242	6.521065251	-2.679252855	6.491550368
	H243	5.221591160	-2.643167618	-2.010809314
	H244	4.686841811	0.028965142	-2.906953733
	H245	7.746901565	-1.584694817	-2.114347956
	H246	1.257106577	0.030893336	-4.797530499
	H247	0.967353484	-0.122873477	-6.990687853
	H248	-0.706364180	-0.413192673	-8.452157283
	H249	2.477415772	0.349058855	-6.923015510

H250	-2.064535005	-0.643824378	-6.443282018
H251	-2.677947233	0.374256718	-7.788027689
H252	9.540073231	-2.975231237	-6.124196147
H253	10.319509913	-2.322272627	-7.582249356
H254	7.434155392	-3.470050835	-6.542984049
H255	6.370279774	-2.488533567	-7.547308499
H256	4.978429037	-4.244570033	-9.378626809
H257	1.450951894	-4.057948672	-6.581262773
H258	4.945618967	2.365854438	-9.872175911
H259	7.199787807	2.321334247	-5.439099640
H260	4.162313033	-0.937720860	-8.413926610
H261	3.745441029	-4.369760091	-3.106229759
H262	5.996316283	-4.742172290	-2.647869054
H263	2.707349352	-2.611916785	-4.649696855
H264	-3.566545867	3.176323529	-7.024559262
H265	8.573291454	-6.584891227	-3.285774479
H266	-3.903846890	0.731965648	-5.529291439
H267	3.969919524	-5.424237318	-4.501126538
H268	5.074349350	1.125519612	-5.734213234
H269	9.360197016	-5.027416594	-2.958268939
H270	3.780181030	-5.022605066	-8.404008803
H271	4.726487221	-1.897064106	-9.744056004
H272	-6.068110568	0.283528796	-3.850988471
H273	-5.261264225	2.789167375	-3.302476261
H274	7.150501122	3.458709078	-9.609415802
H275	-1.104222389	1.344951235	-5.336531869
H276	5.632669230	-3.837731542	-5.469272857
H277	3.003213484	-4.891864694	-6.637701578
H278	1.835377945	-4.101691236	-4.341279969
H279	2.660851598	-1.910037167	-6.971512400
H280	9.145782588	0.960236324	-2.142099735
H281	8.427282386	-0.866660585	-4.719580421
H282	11.155240549	-1.198576949	-5.649099503
H283	9.770050405	-0.625818334	-1.635026422
H284	9.097342849	1.438378717	-6.035701389
H285	-4.106679198	-9.658479386	1.226535939
H286	-5.100801513	-8.641324088	2.284686401
H287	-3.692107486	-7.643459153	6.473473700
H288	-6.225001293	-10.706177749	2.008190113
H289	-7.044189126	-9.658901095	1.051575455
H290	5.688606728	-3.994046594	1.822244075
H291	8.624688255	-3.377383605	1.479602837
H292	7.444853514	-6.448610189	-1.258582378
H293	6.312601170	-2.789857249	0.652743026
H294	9.115294543	-4.931052726	1.611010169
H295	2.127543471	-8.523097325	0.147225923
H296	7.055686005	-5.761455017	0.786413167
H297	-2.781395462	2.294980870	9.313305635
H298	-3.793271806	-0.450059361	10.234271552
H299	0.182936687	-7.611794698	-0.601299637
H300	2.641482627	0.194853377	5.181055495
H301	-1.357295998	-7.805394384	-3.835055979
H302	-3.789273949	-2.996419961	-1.687790825
H303	-0.431652193	-6.331633219	-3.538786788
H304	-1.112515299	2.136968463	7.207097068
H305	-1.458078542	-6.582174645	-1.084524669
H306	-2.236645236	0.950007604	6.532012681
H307	-3.771591022	1.147886952	8.381415709
H308	-1.842901387	-0.537133511	8.535172677
H309	-0.842502736	0.733795558	9.263311470
H310	-4.634075498	-2.557066065	-6.353720685
H311	-4.706643874	2.307555785	10.975290206
H312	-7.707750991	1.706473353	10.820702607
H313	-1.452212408	-3.876614427	-1.559072558
H314	-6.216156727	3.174910483	12.853683728
H315	-7.228649568	3.734312394	11.693645502
H316	-0.469654376	-0.660170967	6.353197934
H317	-0.059565645	-3.992188267	-3.569164213
H318	-4.813818306	-2.572572509	-3.915052390
H319	-6.888448470	-4.041846495	9.845207980
H320	-6.078303749	-5.851907039	7.195927396
H321	-5.150368527	-2.421031345	8.953043671
H322	-8.117922997	-4.483539308	8.988901698
H323	0.728036979	-10.698679555	-0.987343713
H324	-2.567158697	-2.929159283	7.299763079
H325	-5.977533184	0.481541329	5.636175718
H326	-3.194016976	-7.731942792	0.396097770
H327	-7.524526301	-1.154423047	6.360503025
H328	-7.459721172	-6.718940051	2.614006115
H329	-5.691712278	-4.956858253	2.127284898

H330	-7.491089849	1.236998725	12.511431654
H331	1.488487496	-1.082520941	5.365460303
H332	-2.164382261	-2.971470214	9.639771036
H333	-1.085917736	-3.399494220	7.336599455
H334	-2.219975336	-1.401127772	11.420718742
H335	0.642650217	2.779402442	6.433067642
H336	-7.462254962	-4.005946754	5.234906611
H337	-6.917489156	-3.588395397	6.866556661
H338	-7.890006087	-1.629561512	4.693623269
H339	-3.513768112	-5.508328165	-0.522553914
H340	-1.085453336	-3.594285176	-5.830004175
H341	-3.095606443	-2.919463928	-7.183072494
H342	-4.431320969	0.018672285	4.899467825
H343	-3.547403256	-2.958983535	10.351212125
H344	-5.565992069	-2.044383530	10.392668420
H345	2.086267092	2.379760566	5.673566602
H346	-7.315586901	-7.589952218	5.022801785
H347	-4.498433727	-5.242341100	6.715835912
H348	-4.952641291	-6.127404673	-1.385395315
H349	2.784289643	-8.518221450	-1.504952277
H350	3.910208032	-0.481821966	7.747775552
H351	3.744506470	-2.033553274	6.888084542
H352	-4.991061953	-9.330554153	7.474369568
H353	-6.221428170	-8.307932410	7.823899267
H354	-4.779108056	-5.669171876	11.347077507
H355	-5.492229366	-5.950684440	9.196439197
H356	-4.155400198	-8.321324353	11.111802769
H357	-3.156536462	-5.438273251	10.664069934
H358	4.354231062	-3.364998674	9.189609285
H359	11.797052561	3.127663819	-6.708399830
H360	-0.977469285	0.345001099	13.536701650
H361	0.630090630	-0.699036954	11.537549098
H362	9.738745907	2.141511908	-8.716285878
H363	8.897635642	3.957957221	-6.370590879
H364	8.864040455	4.395922600	-8.089106909
H365	-2.504764078	-0.542182710	2.043458003
H366	3.237579530	1.274248528	-8.779707853
H367	-1.416871442	0.520034428	1.247040756
H368	0.356307122	-0.678294972	3.096499134
H369	-1.510866906	0.467964408	2.953820231
H370	-1.337945736	-0.486024497	-0.472343326
Mo371	-3.465020646	-3.626152671	4.195616167
N372	0.065115444	-0.794092372	2.110980069
N373	-1.484564491	-0.056788212	2.082426041
N374	-8.978530342	4.368298771	-2.572102231
N375	-6.422518400	6.660988655	-4.049526079
N376	-5.496962822	8.578508292	-2.216631140
N377	-5.294402021	7.266195010	0.282966885
N378	-3.102067570	5.611285367	-0.326382005
N379	-1.049209134	7.672331744	-0.603750135
N380	-1.050623902	9.242931967	1.848618029
N381	-1.267013931	7.321448301	3.891526929
N382	0.153196282	4.880831417	3.217051963
N383	2.913887219	5.121894211	1.969699546
N384	3.583446609	7.694187156	3.032301960
N385	-6.487078455	3.056240444	11.875013990
N386	-4.729469470	1.291545984	10.839564092
N387	-0.254212106	0.319911723	6.560642895
N388	1.330860770	2.042124724	6.272137889
N389	1.664066750	-0.056956359	5.322684172
N390	-1.955772705	-0.531483943	11.935937212
N391	-5.584291747	0.982507035	-4.445146995
N392	-6.678134615	-0.920076268	-0.231594743
N393	-3.196978728	1.476609108	-5.410341248
N394	-2.629630454	3.515527541	-7.230810161
N395	-1.778404265	5.623312662	-5.567704382
N396	0.648816526	4.700359160	-4.479028810
N397	0.784979797	0.473611853	-3.994882213
N398	-0.614060881	0.825538081	-2.303315570
N399	2.924167543	5.163109975	-5.968254984
N400	3.993065844	5.503432954	-10.582431677
N401	6.049179837	6.323602752	-10.740875113
N402	6.223900138	-2.964649913	7.428654340
N403	8.291396816	-4.326263320	1.679822553
N404	7.861748154	-5.586749101	-1.600924155
N405	6.272448190	-4.536656913	-3.615339480
N406	3.042668779	-2.855154897	-7.186159221
N407	4.159342841	-4.162970006	-8.784107067
N408	4.401517489	-1.862512473	-8.781857731
N409	5.412011153	-1.928324770	-2.738481600

N410	7.883931596	-0.661664176	-2.534453039
N411	9.393257228	-0.555495224	-4.825072001
N412	7.313139165	-2.773976294	-7.290239315
N413	9.838815844	1.447147313	-6.731710034
N414	-6.155055756	-10.158566552	1.147709735
N415	-4.052085456	-7.490907384	-0.108668197
N416	-1.784749962	-6.886605359	-2.021581958
N417	0.711874432	-8.122677293	-1.317820730
N418	-6.571680289	-7.189527470	-4.100670097
N419	-4.974203369	-5.255904428	-5.501226214
N420	-5.478000165	-8.492595282	7.142802039
N421	-5.099807394	-5.276519507	4.174484731
N422	-6.753775348	-6.428923266	3.285088167
N423	-4.596594666	-6.393356066	9.414267464
N424	2.145148886	-0.368508448	-1.280219611
N425	1.608585016	-0.946243253	-0.474624223
O426	-6.359704952	5.485637579	-2.098482539
O427	-3.366294718	7.820490226	-2.480797420
O428	-3.939857967	8.954808201	1.006252719
O429	-2.501375142	5.740536944	1.866437992
O430	0.379751167	6.051031073	0.079795845
O431	1.224641407	9.107928476	1.698081128
O432	-0.382755854	8.821683005	5.392829481
O433	0.062619421	4.321541360	5.434747786
O434	3.304605665	3.694055044	3.722711147
O435	5.535729177	6.726461019	3.665947782
O436	4.831561016	9.184453441	6.041984755
O437	-6.116679798	-0.517702671	11.226709605
O438	-2.217828967	1.744276808	12.124063259
O439	1.225236228	-1.477960698	13.326958455
O440	-2.807443688	3.274330439	-4.031053168
O441	-6.613229229	-0.799078776	-2.501960491
O442	-0.397081336	3.160722524	-6.925235518
O443	-0.600101460	0.190886465	-7.695739639
O444	-1.310540300	6.900413640	-7.418903685
O445	0.893917168	6.627112197	-3.274437195
O446	4.224386208	4.898964495	-4.116362755
O447	5.803124268	7.196298251	-6.309738354
O448	4.774581613	-1.764447299	10.386698682
O449	6.597690489	1.069050905	2.398578404
O450	8.387662496	-3.417861685	-1.136398206
O451	7.980024572	-4.663123501	-5.145181768
O452	5.398238756	-1.400752603	-4.964969582
O453	6.863775627	1.354418739	-2.933084362
O454	6.357219507	-0.516705759	-0.054858457
O455	11.265991691	0.077905263	-3.663537626
O456	11.283535214	0.118511005	-7.890581294
O457	8.078188710	-0.796983616	-8.146950109
O458	11.654101345	3.917290772	-8.588142969
O459	3.670832094	0.976999109	-7.956193054
O460	-6.334552081	-7.739867216	-0.189350461
O461	-3.618861857	-6.928850243	-3.393213240
O462	1.056506664	-8.636901283	-3.508502222
O463	2.729782059	-11.086020533	-0.879807919
O464	-6.954157045	-4.501161170	-6.343442758
O465	-8.375909041	-3.497495412	-0.528078708
O466	-7.228705183	-5.262526312	0.214835115
O467	-2.112438668	-5.258650445	-7.650355009
O468	-2.808454873	-7.649498052	8.814359938
O469	-2.719004682	-7.457545462	12.276408437
O470	-5.881447387	-0.611254154	2.535341429
O471	-6.359564872	1.463682985	3.301446086
O472	-9.210662584	-4.060996648	7.696505091
O473	-9.983671321	-3.010368942	5.849183266
O474	-4.059826178	-3.035407520	6.230124823
O475	-5.086421114	-1.495943991	7.508920084
O476	-5.392666932	-2.456248781	4.078587826
O477	-2.569374875	-2.925322362	10.541718120
O478	-1.733240121	-2.862381683	7.830837852
O479	1.847573938	-0.322578541	-6.576436772
O480	-7.578877302	-4.740077451	9.797046327
O481	-5.251900393	-2.818676136	9.862338784
S482	4.744445847	-4.313354825	-0.404271781
S483	1.420657971	-3.844932762	-0.523102035
S484	-3.422415920	-3.421964069	1.833286699
S485	2.768629954	-2.120593558	1.988321959
S486	-1.310099216	-1.828186662	-0.187098407
S487	-1.088405007	-6.510501456	1.125531467
S488	-2.019519774	-1.693178552	4.398697237
S489	2.316072103	-5.630214595	2.432702121

	S490 -1.886259849 -5.344205526 4.560154035<br>S491 1.228689555 -3.326843735 5.316867496<br>end

product

Fe(139) -2.505<br>Fe(140) 0.068<br>Fe(141) -2.531<br>Fe(142) 2.651<br>Fe(143) 1.634<br>Fe(144) 1.984<br>Fe(145) 0.177	bm612n2xnewbrk2bh335te_5_53458.12

C1	-6.978198047	5.838927076	-3.103496246
C2	-8.438821095	5.446223144	-3.359556747
C3	-5.013793797	6.996510381	-4.026879752
C4	-4.568575976	7.856917795	-2.850061312
C5	-5.187758594	9.360961615	-1.030000063
C6	-4.753349823	8.516709543	0.173000545
C7	-4.774820617	6.335467882	1.269673124
C8	-3.342738438	5.878850104	0.964836428
C9	-1.728559454	5.351742226	-0.815702319
C10	-0.691373195	6.411475602	-0.415324981
C11	-0.055823520	8.740004956	-0.347335335
C12	0.106642983	9.049078888	1.146241741
C13	-1.022389622	9.669460072	3.225926365
C14	-0.826655781	8.558957027	4.263450096
C15	-1.271107707	6.201570288	4.803539399
C16	-0.295959087	5.058794472	4.528952198
C17	1.031949276	3.777319211	2.857312274
C18	2.509590537	4.213173809	2.941825691
C19	0.667110999	3.242057233	1.460487188
C20	1.616437445	2.126963716	1.019121861
C21	-0.788183306	2.779322175	1.461409873
C22	4.271024524	5.627177813	1.919065056
C23	4.520832286	6.729364990	2.958166266
C24	4.598723887	6.105937848	0.480348525
C25	5.971731700	6.787811416	0.411624397
C26	4.528960811	4.944841809	-0.517832187
C27	3.815628873	8.846492436	3.906370085
C28	4.151590341	8.452364149	5.329434378
C29	-6.963313078	1.678930550	11.641802339
C30	-5.867612726	0.701358280	11.207180337
C31	-3.500693615	0.598098394	10.406355332
C32	-2.481688551	0.662588786	11.561243663
C33	-2.953673634	1.235579787	9.113496292
C34	-1.680927872	0.570229767	8.575431992
C35	-1.317175175	1.098941643	7.181633605
C36	0.922324884	0.752377520	6.050699598
C37	-1.014712038	-0.638698611	13.027848477
C38	0.398585361	-0.962615951	12.593887810
C39	-4.758863153	1.843322305	-3.585227265
C40	-3.501990729	2.292728970	-4.355771880
C41	-4.324807323	1.111802229	-2.295816984
C42	-5.432228871	0.915331765	-1.238897419
C43	-6.279163383	-0.350863698	-1.374383441
C44	-1.903988998	1.466697316	-6.071948241
C45	-1.575240296	2.779734159	-6.790771744
C46	-1.884517443	0.263830939	-7.028044619
C47	-2.444288825	4.821526306	-7.821726091
C48	-1.788648743	5.870999517	-6.915696111
C49	-1.058327650	6.483051203	-4.661112433
C50	0.252736115	5.941024536	-4.089410652
C51	1.917963369	4.147701222	-4.004493118
C52	3.136524201	4.805019011	-4.680355265
C53	2.017846408	2.637715760	-4.274155691
C54	0.983727402	1.798681990	-3.598325151
C55	0.084371562	2.018729345	-2.575087892
C56	-0.218452015	-0.022570159	-3.184205157
C57	4.022700063	5.627109285	-6.822487153
C58	4.649991269	6.870544923	-6.197335239
C59	3.420075437	5.946886302	-8.209945427
C60	4.381818629	6.018324435	-9.357818403
C61	5.658599702	6.538772266	-9.440818176
C62	5.035614997	5.708152222	-11.398507759
C63	5.548103475	-1.844934511	8.094865862
C64	4.839580777	-2.371240559	9.327901891
C65	4.459989656	-1.235710426	7.170887098
C66	5.037536963	-0.611929607	5.922369666
C67	5.448619689	0.733426640	5.901662030
C68	5.184134654	-1.363026305	4.741971435
C69	5.971046505	1.315831692	4.741940782
C70	5.717210538	-0.799867142	3.581961321
C71	6.100087410	0.543283060	3.580662186
C72	7.339959250	-4.715135743	0.638280873
C73	7.924274064	-4.511317900	-0.771793519
C74	6.081916156	-3.870108242	0.792823478

C75	8.406226714	-5.556228382	-2.959316680
C76	7.532335986	-4.871899542	-4.013824653
C77	5.348713464	-3.756892254	-4.433406610
C78	5.389827214	-2.250385347	-4.068892361
C79	3.945743876	-4.366101869	-4.206869989
C80	2.730311938	-3.699593243	-4.861706206
C81	2.529292912	-3.950551632	-6.366419708
C82	3.845742314	-2.976446769	-8.258188975
C83	5.450751192	-0.524328446	-2.336438238
C84	6.792192772	0.152039843	-2.652393404
C85	5.173593251	-0.324201176	-0.830702118
C86	9.215283587	-0.092419584	-2.430680853
C87	10.066191515	-0.180153229	-3.687975780
C88	10.175981546	-0.894088099	-6.013484547
C89	10.476743000	0.272359020	-6.986834609
C90	9.609791927	-2.094250873	-6.779939568
C91	8.276605305	-1.812822847	-7.459138667
C92	9.894326851	2.546642497	-7.710262009
C93	11.240814325	3.252486295	-7.675287889
C94	8.756945346	3.559987090	-7.387979691
C95	7.378884394	2.947461359	-7.516011057
C96	6.710240291	2.953548341	-8.750966021
C97	6.747046202	2.316728402	-6.430481236
C98	5.467606168	2.335265870	-8.907926756
C99	5.516172276	1.669885828	-6.574436353
C100	4.889180355	1.681559296	-7.818542403
C101	-5.079100839	-9.159721440	1.318658330
C102	-5.207215198	-8.075464838	0.263896533
C103	-3.945208858	-6.388107447	-1.051904485
C104	-3.106840373	-6.768477094	-2.279261977
C105	-0.830463331	-7.266112277	-3.065231247
C106	0.377157558	-8.094238412	-2.637027045
C107	1.916290371	-8.827604360	-0.897249786
C108	1.786295467	-10.337280432	-0.962856782
C109	-7.059114962	-5.819864455	-4.305959799
C110	-6.329180329	-5.139442543	-5.469136212
C111	-7.076687752	-4.891961698	-3.071367579
C112	-8.123586634	-5.358867283	-2.042543017
C113	-7.949797632	-4.626550478	-0.730757592
C114	-4.204212984	-4.666349186	-6.573526588
C115	-3.106518299	-5.610005049	-7.023374021
C116	-3.745031734	-3.204952432	-6.339545076
C117	-3.023206523	-3.123851362	-5.028611461
C118	-1.667228825	-3.476012631	-4.941545664
C119	-3.758167542	-2.926852356	-3.845536448
C120	-1.091114737	-3.734552866	-3.695605312
C121	-3.180530626	-3.189319155	-2.603551534
C122	-1.860839029	-3.647600219	-2.535544228
C123	-4.541094535	-7.361140181	7.123793272
C124	-3.906851003	-7.133605453	8.502525760
C125	-5.183301134	-6.075127470	6.581797650
C126	-5.605213635	-6.105595300	5.147406742
C127	-6.637767187	-6.826641611	4.584905650
C128	-5.788627242	-5.459525779	3.063543817
C129	-3.997026968	-6.132635485	10.704665163
C130	-3.574513798	-7.409085484	11.397527393
C131	-5.947003228	0.310634843	3.481227965
C132	-5.501811736	-0.145448571	4.885523499
C133	-5.765666565	-1.639464497	5.217224078
C134	-7.252385428	-1.876002722	5.575165000
C135	-7.582010542	-3.288245385	6.068035409
C136	-9.030377211	-3.448267379	6.583133114
C137	-4.896569547	-2.061115270	6.412240959
C138	-0.195255675	-3.498311564	2.292360300
Fe139	-1.744500135	-4.740731786	2.352802953
Fe140	0.752442292	-2.107801771	0.864803250
Fe141	1.604457330	-3.720344829	3.159627447
Fe142	0.505178858	-4.984501912	1.183084951
Fe143	3.038726744	-4.218037581	1.001734974
Fe144	-0.680000161	-3.395848642	4.230422368
Fe145	-1.378861349	-1.793496608	2.168692522
H146	-8.821398274	4.565559663	-1.558918274
H147	-8.442203890	3.500757526	-2.735669830
H148	-6.950785683	6.840500274	-4.900124116
H149	-8.560070893	5.198617892	-4.426259092
H150	-9.046553843	6.351305140	-3.187406332
H151	-4.398164236	6.084768895	-3.996268744
H152	-6.473286541	8.543161883	-2.551385748
H153	-4.768202917	7.532124704	-4.953265498
H154	-6.067160643	9.954386881	-0.748169118

H155	-5.870527024	6.906366521	-0.479376751
H156	-4.360902528	10.054057181	-1.236233587
H157	-1.379893874	4.391242071	-0.424669934
H158	-3.816709552	5.753959683	-1.024372605
H159	-5.439470503	5.461228889	1.301941487
H160	0.927282142	8.429723116	-0.722792123
H161	-1.888471288	7.921689380	-1.149083936
H162	-4.766792950	6.800810351	2.262357174
H163	-1.779852661	5.274578562	-1.909315013
H164	-1.951514429	9.173231076	1.361459771
H165	-0.363325380	9.657523431	-0.866889199
H166	-1.487541934	3.612895240	1.608234059
H167	-1.030868578	2.277010524	0.512047115
H168	-0.938885439	2.070539140	2.285006256
H169	0.760398315	4.074130647	0.745725924
H170	-1.974067695	10.170645795	3.453470465
H171	3.547787825	4.449493461	-0.515433448
H172	2.765266666	7.732535510	2.432398595
H173	6.026354081	7.683631092	1.046822561
H174	1.363528125	1.820280153	-0.003923989
H175	1.525236684	1.237205361	1.659252183
H176	6.218213042	-1.025462020	8.417459737
H177	4.711928474	5.306508162	-1.539336315
H178	6.181292264	7.091390376	-0.624566881
H179	5.294086006	4.186913524	-0.285366096
H180	6.767869982	6.097299621	0.731703951
H181	3.835070332	6.854730311	0.198273863
H182	4.953627034	4.809239502	2.193644568
H183	2.219280291	5.452797993	1.321243827
H184	2.668784994	2.449318441	1.017204114
H185	4.381508703	-1.018092712	-0.512589094
H186	4.805621016	0.705324361	-0.708229843
H187	6.325993948	0.068611985	0.718742811
H188	6.773000407	2.012533305	2.505131731
H189	5.800927389	-1.387581415	2.668262558
H190	4.845188370	-2.400788438	4.716839854
H191	5.343395108	1.345761526	6.800861068
H192	-0.207825594	5.498790194	2.527543159
H193	-2.276289819	5.751607596	4.821939826
H194	-1.045383351	6.572175631	5.810497735
H195	-1.748569542	7.202510927	3.001961892
H196	-0.208920699	10.390192302	3.381498214
H197	6.257328047	2.370795374	4.736884225
H198	0.911832623	2.980003740	3.602219415
H199	4.620600013	9.493137715	3.521938416
H200	2.888443808	9.438493682	3.934505023
H201	3.714874864	7.485561005	5.670650512
H202	-1.350364867	-1.386930390	13.762421851
H203	-7.259039339	-1.762429204	-0.279146571
H204	-5.478008734	-1.757408475	3.256692445
H205	-9.130922633	-5.143163479	-2.426640642
H206	-8.041825950	-6.443762539	-1.882068056
H207	-7.308593477	-3.858611793	-3.372769992
H208	-3.286982888	-6.681903668	-6.771418504
H209	-8.095117865	-5.900379194	-4.669484513
H210	-3.881599519	0.142472810	-2.573632289
H211	-6.129811450	1.771030551	-1.261389523
H212	-4.483178611	-5.763512757	-4.748645685
H213	-4.897624611	-4.643230326	-7.439996014
H214	-5.654641534	-7.196856817	-3.638736452
H215	-6.344234531	-0.625589629	0.711807884
H216	-6.071020470	-4.871702960	-2.624780911
H217	-7.216905465	-7.693508448	-3.481806588
H218	-6.289562121	1.528490122	-4.935899517
H219	-3.515399749	1.697389238	-1.838256299
H220	-6.935450734	-6.176661176	-0.076310522
H221	-4.980152098	0.932360670	-0.236566596
H222	2.012270743	4.358596161	-2.930663457
H223	-0.077011913	2.938293385	-2.021488631
H224	-0.806896492	7.406271872	-5.201236653
H225	-2.194156058	4.754571675	-5.204661155
H226	1.999902642	5.091031695	-6.365563774
H227	6.305690246	7.002787807	-8.703946377
H228	3.918478670	7.514284945	-5.651525901
H229	2.821471085	6.872189185	-8.137483777
H230	4.797848822	4.849886858	-6.913835257
H231	5.092026985	5.428877602	-12.447391121
H232	3.027803570	2.324953218	-3.959025727
H233	1.965635418	2.473937916	-5.363726004
H234	6.957761457	6.589529146	-11.139251349

H235	2.709298918	5.140180966	-8.450055601
H236	0.105559736	4.165315624	-5.161483457
H237	-1.699115159	6.776928310	-3.815856036
H238	-1.808467115	4.759971854	-8.716082464
H239	-0.568825537	-1.049104289	-3.264522571
H240	-3.424308462	5.202423199	-8.137315733
H241	7.085720547	-3.192259141	7.939078043
H242	6.523986856	-2.670601224	6.494791275
H243	5.227421741	-2.650150327	-2.019704670
H244	4.683795567	0.025506021	-2.900845272
H245	7.751556999	-1.581641436	-2.123139672
H246	1.247050913	0.025347312	-4.767228945
H247	0.967484116	-0.127275690	-6.988909634
H248	-0.710787367	-0.435808766	-8.432576975
H249	2.476315263	0.350241281	-6.930950542
H250	-2.074675737	-0.645597317	-6.434414353
H251	-2.682167678	0.376445110	-7.779213649
H252	9.552158744	-2.964867300	-6.111749958
H253	10.332293729	-2.330277186	-7.576333145
H254	7.454076700	-3.486323944	-6.561594079
H255	6.393468598	-2.509645050	-7.575265671
H256	4.968439025	-4.255560669	-9.402210249
H257	1.456960570	-4.059363326	-6.588134314
H258	4.957570469	2.360795474	-9.874826481
H259	7.202800614	2.326562423	-5.436798893
H260	4.181710942	-0.946967192	-8.408216121
H261	3.763780408	-4.369264283	-3.119859530
H262	6.009386092	-4.734171624	-2.657955994
H263	2.713364775	-2.616932308	-4.659139057
H264	-3.565686005	3.174660877	-7.019814705
H265	8.587470515	-6.586594529	-3.293584446
H266	-3.889270380	0.717144600	-5.503645232
H267	3.988210087	-5.424237402	-4.514076690
H268	5.072760828	1.138767395	-5.732079717
H269	9.370422655	-5.027658387	-2.966366044
H270	3.761036941	-5.025663789	-8.433116870
H271	4.724031500	-1.892865984	-9.754730041
H272	-6.076014403	0.283665388	-3.840441969
H273	-5.264141123	2.780828799	-3.285410438
H274	7.163052882	3.451945795	-9.611106440
H275	-1.098632614	1.342649186	-5.333306351
H276	5.650728346	-3.831359235	-5.482278916
H277	3.007386578	-4.897943990	-6.648788929
H278	1.850909951	-4.112623276	-4.350096952
H279	2.672149336	-1.914386154	-6.980817643
H280	9.137761298	0.970359055	-2.155401985
H281	8.442818337	-0.852937265	-4.731571917
H282	11.175068094	-1.186296371	-5.654272819
H283	9.766519514	-0.608578558	-1.633271328
H284	9.121341664	1.449830072	-6.040215850
H285	-4.098638136	-9.651751383	1.234557199
H286	-5.091678704	-8.633593675	2.295243187
H287	-3.705690062	-7.653072718	6.469756113
H288	-6.216499093	-10.702273877	2.018910535
H289	-7.036399818	-9.652866977	1.065372938
H290	5.702996937	-4.007641169	1.815441382
H291	8.639885480	-3.388868987	1.487288430
H292	7.450738799	-6.450126404	-1.269563738
H293	6.321463409	-2.807480191	0.640973402
H294	9.133335308	-4.942263245	1.615165812
H295	2.110758824	-8.553257534	0.151943320
H296	7.079017138	-5.775209469	0.784644394
H297	-2.773191755	2.307215221	9.301915610
H298	-3.774526772	-0.446139000	10.209562529
H299	0.185422848	-7.620673802	-0.609457499
H300	2.648728813	0.142709998	5.149109566
H301	-1.356390879	-7.830614141	-3.845012831
H302	-3.757830613	-3.043925529	-1.688347873
H303	-0.431705568	-6.356578038	-3.548156297
H304	-1.031934715	2.157163561	7.223759280
H305	-1.463114049	-6.601829178	-1.096638696
H306	-2.196298562	1.027426623	6.519253666
H307	-3.757383604	1.159816346	8.363833168
H308	-1.830297262	-0.518166154	8.506200292
H309	-0.833309920	0.739216633	9.258698008
H310	-4.638964176	-2.564764195	-6.348067262
H311	-4.697166610	2.305898342	10.958693853
H312	-7.701447972	1.686400447	10.825496834
H313	-1.411591518	-3.903184637	-1.579462932
H314	-6.203241998	3.169978057	12.840609594

H315	-7.224561997	3.719224180	11.684027923
H316	-0.490593770	-0.647227927	6.333162874
H317	-0.042794052	-4.023984250	-3.613091081
H318	-4.797574319	-2.594775574	-3.902140004
H319	-6.871453605	-4.048816357	9.841123388
H320	-6.073481796	-5.837139285	7.189376492
H321	-5.130633712	-2.427886971	8.944575291
H322	-8.099204755	-4.492734162	8.984084889
H323	0.735438560	-10.715830066	-1.000828115
H324	-2.535209171	-2.928136474	7.293391689
H325	-5.966713130	0.492988477	5.646550586
H326	-3.189201272	-7.747467341	0.380917247
H327	-7.502377973	-1.150638654	6.365150793
H328	-7.439338330	-6.691561876	2.603145312
H329	-5.663188182	-4.935588788	2.124261549
H330	-7.458984421	1.221455719	12.514511657
H331	1.486824353	-1.133409654	5.368065475
H332	-2.149705639	-2.969557315	9.630344912
H333	-1.059223501	-3.414551534	7.344079847
H334	-2.209132497	-1.400093781	11.405590257
H335	0.680249056	2.774783454	6.310468827
H336	-7.441436539	-4.003871533	5.242572690
H337	-6.894533054	-3.583928409	6.872943027
H338	-7.870435404	-1.626811248	4.698614460
H339	-3.504523222	-5.518804856	-0.538629535
H340	-1.085327502	-3.600188702	-5.857273794
H341	-3.106467087	-2.921843941	-7.190412374
H342	-4.413574383	0.033443631	4.923286507
H343	-3.532837537	-2.964145728	10.342231877
H344	-5.546477740	-2.051431289	10.382569507
H345	2.144293100	2.311652647	5.636835904
H346	-7.306344644	-7.565147792	5.012004109
H347	-4.485976451	-5.239318805	6.720370398
H348	-4.951837864	-6.136629703	-1.390587075
H349	2.786977985	-8.515620605	-1.493852757
H350	3.913266156	-0.479079822	7.753894465
H351	3.747940579	-2.031731156	6.898275045
H352	-5.018357621	-9.325758431	7.467081713
H353	-6.238352546	-8.294243387	7.824632346
H354	-4.762699144	-5.656467258	11.338006242
H355	-5.498185743	-5.954570590	9.199359728
H356	-4.165947489	-8.312491470	11.113406783
H357	-3.142086082	-5.440778091	10.644674334
H358	4.361000444	-3.370511225	9.186593738
H359	11.813503209	3.127603618	-6.725608236
H360	-0.980982203	0.342831454	13.532399789
H361	0.642626522	-0.694155964	11.536843324
H362	9.748076115	2.150946928	-8.728254184
H363	8.908990232	3.953707455	-6.370734054
H364	8.865573452	4.402729699	-8.086024678
H365	-3.432368830	-0.131055402	1.989350710
H366	3.247813103	1.272453271	-8.784858811
H367	-2.087438994	0.552264176	1.268811957
H368	0.714663625	-0.645728661	3.013868792
H369	-2.227656865	0.555263471	2.897383667
H370	-1.173329829	-0.497500173	-0.470818581
Mo371	-3.439443073	-3.578662422	4.183618719
N372	0.185102289	-0.876776933	2.161350158
N373	-2.417844006	-0.001897098	2.060662406
N374	-8.965684418	4.359833505	-2.550609530
N375	-6.428446369	6.662140045	-4.046170256
N376	-5.514006661	8.587911262	-2.216782187
N377	-5.283728603	7.270467678	0.274343673
N378	-3.073731635	5.642132103	-0.339778423
N379	-1.035353180	7.702620358	-0.629449991
N380	-1.047760039	9.249424957	1.835658522
N381	-1.257612601	7.323869432	3.889641727
N382	0.138247558	4.877524531	3.257962919
N383	2.903897686	5.115500281	1.998690089
N384	3.576860089	7.701517430	3.050220580
N385	-6.477704452	3.046506881	11.863563452
N386	-4.715987267	1.289771046	10.822390448
N387	-0.237477236	0.317914799	6.563840938
N388	1.365415625	2.017192063	6.229590305
N389	1.683639061	-0.111754799	5.349224661
N390	-1.945739545	-0.530325483	11.921132069
N391	-5.587671537	0.978761939	-4.435113921
N392	-6.665930450	-0.929258341	-0.222237041
N393	-3.193266132	1.473380545	-5.396399550
N394	-2.628291541	3.506640142	-7.235222687

N395	-1.775232056	5.617254469	-5.578597128
N396	0.656043366	4.700262354	-4.485060129
N397	0.773274725	0.485040821	-3.976425335
N398	-0.657456752	0.884166082	-2.320779573
N399	2.935247817	5.166408094	-5.973304945
N400	4.003978305	5.503653269	-10.588043508
N401	6.057925211	6.329870273	-10.744216106
N402	6.228809298	-2.959410216	7.431461156
N403	8.308141591	-4.339171108	1.682680369
N404	7.874708174	-5.590225322	-1.608340858
N405	6.287019346	-4.531598763	-3.625768608
N406	3.051004015	-2.860642204	-7.197408959
N407	4.148742887	-4.167912680	-8.809204344
N408	4.416054820	-1.868825091	-8.786552069
N409	5.415409297	-1.931462416	-2.743519918
N410	7.885060014	-0.657907174	-2.542716674
N411	9.410081838	-0.543603269	-4.832188765
N412	7.335563893	-2.784900712	-7.304187624
N413	9.855247188	1.453379769	-6.744409947
N414	-6.146918891	-10.152569207	1.159690138
N415	-4.049423929	-7.498434766	-0.115075668
N416	-1.787774835	-6.911280348	-2.031399367
N417	0.708431358	-8.140565586	-1.321308799
N418	-6.573918225	-7.190337926	-4.096857937
N419	-4.971798047	-5.259076413	-5.495383144
N420	-5.499429423	-8.482683475	7.139615125
N421	-5.076345122	-5.259658217	4.172436374
N422	-6.734680204	-6.404400496	3.277031223
N423	-4.596900524	-6.390956787	9.408686276
N424	2.073384403	-0.409068667	-1.138161896
N425	1.580537184	-1.031939571	-0.341686341
O426	-6.344419641	5.486054457	-2.095056740
O427	-3.374532930	7.861563900	-2.501420538
O428	-3.963541412	8.981499892	1.011249019
O429	-2.487054139	5.748223661	1.858077147
O430	0.405449360	6.095105851	0.072888706
O431	1.227942115	9.147696841	1.670335477
O432	-0.343421879	8.823897629	5.372667490
O433	0.006977314	4.317388051	5.480524990
O434	3.271418187	3.748175339	3.803029727
O435	5.545031906	6.749078060	3.660114593
O436	4.794691266	9.168117021	6.083000274
O437	-6.094398090	-0.525990699	11.211167408
O438	-2.205006628	1.745622496	12.108244181
O439	1.232845642	-1.461316580	13.332923332
O440	-2.848534287	3.309267132	-4.051898633
O441	-6.619691924	-0.803696066	-2.493111728
O442	-0.395938601	3.150289846	-6.927970052
O443	-0.605520912	0.180622366	-7.686003092
O444	-1.318191385	6.895392252	-7.431939597
O445	0.902134445	6.636511142	-3.297123741
O446	4.230111775	4.912476259	-4.115436634
O447	5.824144961	7.183865641	-6.304791581
O448	4.763987168	-1.771160132	10.390512743
O449	6.588022965	1.061938925	2.395353149
O450	8.412590476	-3.426068444	-1.131165765
O451	7.990455753	-4.679322016	-5.158012430
O452	5.405475706	-1.393942216	-4.967640281
O453	6.855454755	1.357148352	-2.924729197
O454	6.359364081	-0.531162760	-0.053999216
O455	11.276359778	0.076020727	-3.653146210
O456	11.290443858	0.118696631	-7.909084050
O457	8.087613611	-0.793834017	-8.138405053
O458	11.662039344	3.932728725	-8.597850365
O459	3.677959987	0.979748227	-7.958142533
O460	-6.333707192	-7.735202476	-0.174091448
O461	-3.623788798	-6.952618842	-3.401180944
O462	1.052824657	-8.666654256	-3.508716032
O463	2.741406217	-11.096908332	-0.946692607
O464	-6.951075176	-4.497182594	-6.332242276
O465	-8.382632652	-3.499063537	-0.522095477
O466	-7.230032802	-5.261475806	0.218916746
O467	-2.118537413	-5.267236740	-7.656142421
O468	-2.814221476	-7.652804837	8.805733572
O469	-2.713088901	-7.458107346	12.265257917
O470	-5.751913636	-0.560713133	2.525406796
O471	-6.382542104	1.464113164	3.315015578
O472	-9.190482585	-4.059583733	7.698158037
O473	-9.958968339	-2.991366286	5.859279619
O474	-4.029753090	-3.018724712	6.218309438

	O475	-5.061032887	-1.492028516	7.508897100
	O476	-5.373275207	-2.435806208	4.074442285
	O477	-2.554789524	-2.927587178	10.532200970
	O478	-1.702822375	-2.861862603	7.825449674
	O479	1.853118485	-0.329018760	-6.589207536
	O480	-7.555529674	-4.753012425	9.788258763
	O481	-5.234553704	-2.826996323	9.851917952
	S482	4.763809008	-4.345767604	-0.408889236
	S483	1.452789821	-3.950626787	-0.549506206
	S484	-3.395855205	-3.349360742	1.834982500
	S485	2.829462303	-2.266645005	2.033988466
	S486	-1.290549009	-1.809631867	-0.098507325
	S487	-1.138668180	-6.479293990	1.138841796
	S488	-2.007575210	-1.640406453	4.411249932
	S489	2.280179943	-5.737223547	2.427261644
	S490	-1.850106338	-5.279623784	4.532999209
	S491	1.231047111	-3.350245234	5.320297319
	end			

47, S=3/2

reactant

	bm612n2xnewbrk2bh347tb_3_53419.84			
Fe(139) 2.473	C1	-6.986872953	5.846150117	-3.109881942
Fe(140) 0.124	C2	-8.446101249	5.456205371	-3.372371255
Fe(141) 2.667	C3	-5.007120911	6.982800948	-4.022600819
Fe(142) -2.550	C4	-4.560651782	7.839514322	-2.843681712
Fe(143) 3.145	C5	-5.174113296	9.352403208	-1.029301340
Fe(144) -2.321	C6	-4.745664773	8.505374826	0.173858607
Fe(145) -0.143	C7	-4.789237711	6.328323378	1.278095078
	C8	-3.358341698	5.864489396	0.974221028
	C9	-1.752840901	5.332724952	-0.815481521
	C10	-0.712351828	6.385526111	-0.406430436
	C11	-0.053569368	8.704002682	-0.328454389
	C12	0.104305711	9.024779135	1.162993624
	C13	-1.028903296	9.669019270	3.233907563
	C14	-0.844345001	8.558354147	4.272748629
	C15	-1.257072721	6.193426994	4.795488579
	C16	-0.273596942	5.062872303	4.505228365
	C17	1.045161945	3.779587040	2.834779034
	C18	2.525925355	4.203032271	2.910914771
	C19	0.683301518	3.258770818	1.430327386
	C20	1.626331267	2.140604438	0.977978262
	C21	-0.789751296	2.859207978	1.421130229
	C22	4.280721148	5.630786385	1.898942505
	C23	4.525089372	6.723155828	2.947836524
	C24	4.612675234	6.119816836	0.464742179
	C25	5.987422850	6.799954569	0.407500312
	C26	4.543244065	4.962429419	-0.537320230
	C27	3.829830444	8.844552020	3.886073560
	C28	4.168976862	8.460849557	5.311154344
	C29	-6.976638113	1.690409402	11.647390458
	C30	-5.881962326	0.707910973	11.218600099
	C31	-3.513208237	0.594441685	10.421631038
	C32	-2.492556022	0.660566345	11.574706308
	C33	-2.964027702	1.225195374	9.125989332
	C34	-1.692400972	0.556991135	8.586311543
	C35	-1.348193549	1.076083753	7.184161534
	C36	0.899335191	0.779179261	6.049789457
	C37	-1.017541474	-0.638967317	13.035133964
	C38	0.391891760	-0.969035771	12.594641113
	C39	-4.760227549	1.849329303	-3.594458961
	C40	-3.495287838	2.287596998	-4.357676865
	C41	-4.330196305	1.122249058	-2.299040303
	C42	-5.437221191	0.923109421	-1.242049693
	C43	-6.281790732	-0.344083246	-1.380608738
	C44	-1.905108205	1.468272386	-6.078158430
	C45	-1.575506246	2.784392336	-6.791836163
	C46	-1.881187391	0.266401822	-7.035116204
	C47	-2.448461848	4.830310285	-7.813996729
	C48	-1.787877228	5.875600527	-6.906821867
	C49	-1.061642930	6.483664645	-4.649106954
	C50	0.247726531	5.937987523	-4.077793659
	C51	1.911631295	4.143755186	-4.001831744
	C52	3.130279136	4.800477256	-4.678442420
	C53	2.012991022	2.635116046	-4.276761456
	C54	0.987358426	1.787221417	-3.600135040
	C55	0.100912347	1.990021032	-2.562098039
	C56	-0.191944523	-0.048865464	-3.185398824

C57	4.014215763	5.626763612	-6.819179329
C58	4.636796010	6.873370833	-6.196282972
C59	3.413033807	5.947497750	-8.206751054
C60	4.375376623	6.016647956	-9.354146257
C61	5.653352257	6.534113663	-9.437829956
C62	5.026790983	5.705377843	-11.395234293
C63	5.542363618	-1.846990771	8.088091864
C64	4.837597101	-2.368840031	9.325228042
C65	4.455569599	-1.237419254	7.163974660
C66	5.035977964	-0.611700264	5.917881099
C67	5.451296144	0.732595694	5.901670090
C68	5.183668982	-1.360623502	4.736387791
C69	5.977894559	1.316189499	4.744461514
C70	5.720626803	-0.796231464	3.579088011
C71	6.106211576	0.545933214	3.581721698
C72	7.323767819	-4.699794145	0.649822154
C73	7.904324731	-4.502418187	-0.766043226
C74	6.080557649	-3.842097486	0.812021504
C75	8.398334894	-5.553540539	-2.948470273
C76	7.523041682	-4.868292478	-4.002070705
C77	5.337379420	-3.761049176	-4.422021577
C78	5.383043383	-2.252685707	-4.063081638
C79	3.932475378	-4.366864355	-4.194362770
C80	2.720639985	-3.696381085	-4.852105145
C81	2.523982237	-3.947713884	-6.357379469
C82	3.843246365	-2.972821783	-8.247416435
C83	5.450485818	-0.519363306	-2.337394454
C84	6.794265827	0.151081266	-2.652935082
C85	5.173377280	-0.313123720	-0.832681849
C86	9.214870970	-0.099827048	-2.425234021
C87	10.058239032	-0.184026445	-3.686697962
C88	10.160963399	-0.901583393	-6.007873069
C89	10.466165794	0.268699421	-6.975799550
C90	9.597512772	-2.097710633	-6.782713411
C91	8.264308791	-1.813010441	-7.459590269
C92	9.885670980	2.540681695	-7.702188609
C93	11.232438074	3.245198535	-7.665261335
C94	8.750290365	3.558099695	-7.386349980
C95	7.371847645	2.947438246	-7.515458560
C96	6.701895565	2.956288993	-8.749600197
C97	6.742119017	2.313372604	-6.430811248
C98	5.459578940	2.337309155	-8.906491344
C99	5.512506870	1.664408645	-6.574850357
C100	4.883754630	1.680055457	-7.817942959
C101	-5.084433628	-9.163888197	1.311161002
C102	-5.209537676	-8.076821957	0.258643459
C103	-3.946239985	-6.379775612	-1.043122690
C104	-3.104753744	-6.753413589	-2.270190663
C105	-0.829801733	-7.247307233	-3.057359236
C106	0.379579334	-8.075082421	-2.633096823
C107	1.916215881	-8.814495621	-0.892278529
C108	1.779740558	-10.324614424	-0.937990019
C109	-7.063294757	-5.821716412	-4.314521094
C110	-6.332951977	-5.140744394	-5.477516266
C111	-7.080722699	-4.891746036	-3.080324907
C112	-8.122937207	-5.359982940	-2.047279976
C113	-7.946832404	-4.626705209	-0.736213516
C114	-4.205149207	-4.662127779	-6.577162663
C115	-3.106497704	-5.605249063	-7.025946561
C116	-3.745479962	-3.201182938	-6.341919406
C117	-3.032085805	-3.116471560	-5.027133391
C118	-1.676714625	-3.467635506	-4.929913984
C119	-3.775180907	-2.911020515	-3.850577449
C120	-1.111114146	-3.724546515	-3.679072008
C121	-3.207840369	-3.171942388	-2.604319201
C122	-1.892685787	-3.640073184	-2.527552629
C123	-4.534488234	-7.362427474	7.126260153
C124	-3.902761541	-7.131698735	8.506366353
C125	-5.189772374	-6.084054165	6.582922228
C126	-5.618852929	-6.121473286	5.150603442
C127	-6.650779809	-6.846296841	4.591623489
C128	-5.810275141	-5.478261269	3.065939348
C129	-4.003641253	-6.135940992	10.711964947
C130	-3.576026659	-7.412123200	11.402217647
C131	-5.982247973	0.284401206	3.476502110
C132	-5.513854517	-0.162806736	4.875212410
C133	-5.779513186	-1.653816801	5.214260377
C134	-7.268371539	-1.882495110	5.571755598
C135	-7.600888847	-3.293731001	6.063710756
C136	-9.048388529	-3.455459998	6.578555630

C137	-4.917137672	-2.073756934	6.412549432
C138	-0.214541417	-3.492532241	2.296793372
Fe139	-1.733777314	-4.744607757	2.355468683
Fe140	0.785822749	-2.043022947	0.804792014
Fe141	1.555938609	-3.606256976	3.155537893
Fe142	0.518296032	-5.001107274	1.261154101
Fe143	2.904354813	-4.023079839	0.817147465
Fe144	-0.715917038	-3.441299241	4.263543059
Fe145	-1.659249286	-1.812675380	2.102879565
H146	-8.833818344	4.569826614	-1.575555170
H147	-8.452470934	3.508455362	-2.753973738
H148	-6.940625105	6.843435509	-4.907972864
H149	-8.562308402	5.211462187	-4.440152533
H150	-9.053417316	6.361352978	-3.199595124
H151	-4.400487920	6.065110733	-3.986843714
H152	-6.458880664	8.548707070	-2.558974653
H153	-4.750488888	7.514245366	-4.948334483
H154	-6.052020916	9.948728597	-0.748946934
H155	-5.882850426	6.906825700	-0.471008645
H156	-4.344436988	10.042834811	-1.233467617
H157	-1.403671041	4.368270409	-0.438077863
H158	-3.845077060	5.716190758	-1.010315421
H159	-5.459924800	5.458589914	1.309205575
H160	0.927371211	8.374001943	-0.692548567
H161	-1.899180776	7.908374924	-1.126733267
H162	-4.780774501	6.794286267	2.270461381
H163	-1.807580321	5.264266763	-1.909232696
H164	-1.953371040	9.165407171	1.368935333
H165	-0.342018498	9.621117898	-0.858771332
H166	-1.452054383	3.726293497	1.515065735
H167	-1.049178796	2.322902216	0.494210353
H168	-1.006914080	2.231123328	2.288260167
H169	0.796420929	4.091029247	0.721245450
H170	-1.976618892	10.180198329	3.454963737
H171	3.555532725	4.480739201	-0.546674922
H172	2.784351269	7.744551427	2.403712073
H173	6.038756164	7.693358679	1.046716241
H174	1.350380777	1.816908192	-0.033867772
H175	1.591572033	1.257469967	1.632408516
H176	6.213586155	-1.027272142	8.408165235
H177	4.744199242	5.320554046	-1.556490071
H178	6.204990052	7.108172170	-0.625601732
H179	5.293835199	4.193656777	-0.293449777
H180	6.780909704	6.107716949	0.730281633
H181	3.852392001	6.872326753	0.183166423
H182	4.961478768	4.809024561	2.165903194
H183	2.217515284	5.489754154	1.329925027
H184	2.670847215	2.484602259	0.933016477
H185	4.382037328	-1.004680166	-0.508992482
H186	4.805538182	0.716957832	-0.713979310
H187	6.318912743	0.075439402	0.721886495
H188	6.781755798	2.016769733	2.510364242
H189	5.799040274	-1.381183283	2.663385619
H190	4.840985640	-2.396958530	4.706822479
H191	5.347201784	1.342477118	6.802689025
H192	-0.197096839	5.500729940	2.497987951
H193	-2.257008475	5.732527441	4.824318171
H194	-1.021880689	6.559149796	5.801695088
H195	-1.755573735	7.200448031	3.003327879
H196	-0.209501434	10.382547651	3.391753311
H197	6.267012454	2.370364575	4.741615373
H198	0.921170744	2.981035187	3.576894536
H199	4.638307371	9.484244410	3.494679907
H200	2.906147694	9.442766896	3.913190173
H201	3.724217377	7.502127964	5.665020910
H202	-1.351801109	-1.382994816	13.774861178
H203	-7.264195944	-1.756491002	-0.290884572
H204	-5.535329550	-1.774705498	3.250370339
H205	-9.131971257	-5.146081482	-2.427659790
H206	-8.038838200	-6.444502049	-1.885970820
H207	-7.316024816	-3.859248134	-3.381848147
H208	-3.289491047	-6.678047252	-6.779790520
H209	-8.099059644	-5.905659000	-4.677257285
H210	-3.882012921	0.153180748	-2.569989749
H211	-6.135915422	1.778034064	-1.262481139
H212	-4.486550839	-5.755818526	-4.750815773
H213	-4.896524173	-4.638486203	-7.445166178
H214	-5.653123964	-7.190509887	-3.643505001
H215	-6.360715515	-0.621257149	0.708056844
H216	-6.073857962	-4.867599172	-2.636334601

H217	-7.212311067	-7.691298324	-3.480648879
H218	-6.292918391	1.530653763	-4.942082732
H219	-3.524997407	1.712436999	-1.839822472
H220	-6.936310768	-6.178329934	-0.081622018
H221	-4.983315667	0.935576786	-0.240227418
H222	2.006219849	4.352041066	-2.927410465
H223	-0.058237491	2.900654443	-1.993868926
H224	-0.809540534	7.408988397	-5.185453111
H225	-2.193328796	4.755434288	-5.198725207
H226	1.991269176	5.091609047	-6.361296206
H227	6.302055141	6.996846783	-8.701560680
H228	3.902200148	7.513505638	-5.649987600
H229	2.818331944	6.875204196	-8.133551102
H230	4.790718802	4.850679903	-6.908421751
H231	5.081245502	5.426438622	-12.444225778
H232	3.025525112	2.325364624	-3.966746217
H233	1.957600282	2.475403987	-5.366661712
H234	6.951573756	6.581131770	-11.137064640
H235	2.699235182	5.143818945	-8.447525739
H236	0.102233471	4.170431125	-5.163371353
H237	-1.703201312	6.774182332	-3.803303803
H238	-1.819610821	4.773482992	-8.713428715
H239	-0.524331454	-1.081853298	-3.261727090
H240	-3.430885741	5.212710943	-8.120172032
H241	7.080031330	-3.194016102	7.937090081
H242	6.518848850	-2.677431737	6.491041521
H243	5.238359466	-2.645611788	-2.011204657
H244	4.686217238	0.029926882	-2.905598551
H245	7.747401722	-1.584024610	-2.113256595
H246	1.253738768	0.026789702	-4.786574041
H247	0.966049034	-0.123314890	-6.990090713
H248	-0.707020897	-0.419217248	-8.447026258
H249	2.476232946	0.350278267	-6.925236901
H250	-2.067032944	-0.644283697	-6.442007842
H251	-2.679422258	0.375597871	-7.786098084
H252	9.541929600	-2.973136275	-6.120645291
H253	10.320998304	-2.324994299	-7.580766538
H254	7.437055980	-3.474486814	-6.546561979
H255	6.374729640	-2.494306694	-7.554202836
H256	4.976478908	-4.248143563	-9.383729929
H257	1.452551413	-4.058731076	-6.582033060
H258	4.948094077	2.364371863	-9.872609131
H259	7.199166268	2.321873063	-5.437793264
H260	4.166174794	-0.940856788	-8.410982919
H261	3.749802086	-4.371330881	-3.107635815
H262	6.000736707	-4.732818628	-2.643502970
H263	2.707746736	-2.613432738	-4.651134371
H264	-3.566110127	3.176010505	-7.021926015
H265	8.577018531	-6.584182794	-3.283848100
H266	-3.900298915	0.727873216	-5.523351796
H267	3.972650314	-5.425007167	-4.501787417
H268	5.072380793	1.128283038	-5.733819004
H269	9.363264929	-5.026480698	-2.957944119
H270	3.775900985	-5.024075289	-8.410292101
H271	4.724602403	-1.895550791	-9.746358050
H272	-6.071042891	0.284788165	-3.848678867
H273	-5.264114784	2.789301227	-3.299726615
H274	7.153228743	3.456843678	-9.609117667
H275	-1.103234348	1.344353327	-5.335623967
H276	5.636351119	-3.838989914	-5.471139731
H277	3.003986751	-4.894323597	-6.638576330
H278	1.837365076	-4.104945395	-4.343773411
H279	2.664104705	-1.912288605	-6.973270595
H280	9.142718742	0.961822032	-2.144085303
H281	8.430863168	-0.865175634	-4.721624206
H282	11.159770979	-1.196664163	-5.649840838
H283	9.768454719	-0.622178189	-1.633342871
H284	9.102759137	1.441523609	-6.036288141
H285	-4.104566262	-9.657359395	1.227631875
H286	-5.097335477	-8.639413322	2.288602168
H287	-3.695639475	-7.646187541	6.472903017
H288	-6.223665049	-10.706114001	2.008099794
H289	-7.042247384	-9.656565045	1.053697525
H290	5.686225479	-3.982927409	1.826372215
H291	8.636780225	-3.381908191	1.488225601
H292	7.443655805	-6.446840675	-1.257967425
H293	6.329929073	-2.782316372	0.660384473
H294	9.125878873	-4.937434157	1.611519175
H295	2.122556918	-8.528174684	0.151404441
H296	7.055662854	-5.758410262	0.795673278

	H297	-2.780171909	2.296987128	9.310313293
	H298	-3.789613033	-0.449780191	10.228777915
	H299	0.182692622	-7.612685675	-0.602451700
	H300	2.632129504	0.189589666	5.152483034
	H301	-1.357234559	-7.810994732	-3.836532352
	H302	-3.785922737	-3.013984021	-1.691479730
	H303	-0.434013510	-6.336150303	-3.539137203
	H304	-1.097467361	2.144012074	7.209563211
	H305	-1.459414987	-6.586217374	-1.087368876
	H306	-2.226978375	0.965684837	6.527312686
	H307	-3.768373806	1.149436931	8.377062218
	H308	-1.838654115	-0.532367930	8.525825960
	H309	-0.839841934	0.735569020	9.260738828
	H310	-4.637925804	-2.559144987	-6.356503069
	H311	-4.703657132	2.307259122	10.970771570
	H312	-7.707302164	1.702617604	10.824302557
	H313	-1.452657463	-3.898881618	-1.569299446
	H314	-6.214104878	3.175158459	12.852399818
	H315	-7.228691002	3.731593846	11.692873072
	H316	-0.479716726	-0.654266081	6.344310650
	H317	-0.061329649	-4.006261044	-3.583170055
	H318	-4.812192073	-2.573846104	-3.916937048
	H319	-6.886284225	-4.042364887	9.845387586
	H320	-6.078908863	-5.849949463	7.193440936
	H321	-5.146201851	-2.423431658	8.950758866
	H322	-8.114719970	-4.485425034	8.988104642
	H323	0.729464138	-10.701610424	-0.990350370
	H324	-2.559904074	-2.930880285	7.297360652
	H325	-5.973205190	0.478883303	5.636982923
	H326	-3.193788184	-7.740181863	0.392236110
	H327	-7.515729443	-1.155259712	6.360978053
	H328	-7.459780746	-6.716229093	2.612823912
	H329	-5.694472781	-4.954389511	2.125297122
	H330	-7.482998425	1.233829688	12.514492240
	H331	1.486397119	-1.089600391	5.362987981
	H332	-2.162637881	-2.971888321	9.637018225
	H333	-1.078179443	-3.399088442	7.335993920
	H334	-2.217229574	-1.401142418	11.417451784
	H335	0.654915411	2.780530669	6.408331036
	H336	-7.458884266	-4.007756788	5.237107546
	H337	-6.913380694	-3.589437283	6.868472938
	H338	-7.884378702	-1.630526330	4.694658654
	H339	-3.510296089	-5.510852270	-0.526093875
	H340	-1.087786698	-3.592182022	-5.841031829
	H341	-3.100586695	-2.920678073	-7.188818196
	H342	-4.425879561	0.017794907	4.898124216
	H343	-3.545261460	-2.960080923	10.349708686
	H344	-5.562801992	-2.044933482	10.389349599
	H345	2.098946943	2.362512259	5.660888250
	H346	-7.316405279	-7.585822737	5.021865484
	H347	-4.497618549	-5.243345473	6.715931118
	H348	-4.952128833	-6.129375807	-1.384498150
	H349	2.784265582	-8.516467569	-1.499910279
	H350	3.909056187	-0.481505955	7.748345312
	H351	3.744355888	-2.033511720	6.889545350
	H352	-4.996598247	-9.330507336	7.473281052
	H353	-6.225248540	-8.306576864	7.824559748
	H354	-4.774445581	-5.667169542	11.344708071
	H355	-5.494710242	-5.951815724	9.197869996
	H356	-4.158858744	-8.319197147	11.112986885
	H357	-3.152697501	-5.438563580	10.658919271
	H358	4.355227925	-3.366835477	9.188235084
	H359	11.800365722	3.126486969	-6.711757724
	H360	-0.978637117	0.344391543	13.536359124
	H361	0.632344638	-0.698918780	11.537276099
	H362	9.740588693	2.142366642	-8.719252906
	H363	8.899903658	3.956259890	-6.370412187
	H364	8.863455156	4.397197585	-8.088087902
	H365	-3.032543072	-1.143031658	2.024108633
	H366	3.239540731	1.274133549	-8.781554925
	H367	-1.282545404	0.510254584	1.237524243
	H368	0.446542824	-0.693713959	3.130807063
	H369	-1.408018092	0.470898624	2.948880488
	H370	-1.315548005	-0.503332589	-0.534932008
	Mo371	-3.462634265	-3.609780176	4.209280266
	N372	0.074616089	-0.710922528	2.171484445
	N373	-1.103731796	-0.006288961	2.099699585
	N374	-8.976098727	4.367412579	-2.568240122
	N375	-6.425126138	6.662809729	-4.050458838
	N376	-5.501844941	8.580986888	-2.216720962

N377	-5.291203492	7.266299669	0.281136662
N378	-3.095855430	5.618804661	-0.330103895
N379	-1.046170481	7.679619184	-0.611090986
N380	-1.050621446	9.244900946	1.844802417
N381	-1.263507298	7.321713807	3.890346546
N382	0.150195155	4.882091946	3.229969601
N383	2.913977788	5.118296590	1.977163381
N384	3.583535431	7.697826861	3.036809242
N385	-6.485544148	3.055143398	11.874030898
N386	-4.726836316	1.291307504	10.835073317
N387	-0.250493007	0.320276877	6.563939212
N388	1.339424359	2.036046564	6.260964265
N389	1.657276012	-0.063973745	5.310445165
N390	-1.953541424	-0.531504276	11.932788260
N391	-5.587451398	0.983034271	-4.444215016
N392	-6.675813131	-0.920510864	-0.230571022
N393	-3.196580382	1.475860653	-5.407419407
N394	-2.629262114	3.512861511	-7.232226696
N395	-1.778182564	5.620845354	-5.569789607
N396	0.650237175	4.699094702	-4.479802969
N397	0.782564296	0.476079537	-3.987364403
N398	-0.627211137	0.846914567	-2.308682594
N399	2.927125150	5.165447298	-5.970225218
N400	3.995358618	5.503103601	-10.584038749
N401	6.051026020	6.324571966	-10.741660571
N402	6.222987488	-2.963764540	7.428298264
N403	8.303710340	-4.331288990	1.686022268
N404	7.869114584	-5.587469755	-1.596043518
N405	6.275266313	-4.536445220	-3.614656404
N406	3.045538308	-2.857462167	-7.188204073
N407	4.156934014	-4.165063707	-8.790094224
N408	4.405139225	-1.864464197	-8.782230252
N409	5.410214353	-1.927638485	-2.739974213
N410	7.883465704	-0.661715330	-2.535447877
N411	9.396983204	-0.553728670	-4.826108116
N412	7.317229385	-2.777338561	-7.292951487
N413	9.842468854	1.448834494	-6.734416599
N414	-6.152566938	-10.155256424	1.149901004
N415	-4.051380288	-7.492044095	-0.108567665
N416	-1.785713696	-6.894425563	-2.021975279
N417	0.710994291	-8.124343040	-1.317595373
N418	-6.572874806	-7.189988914	-4.101051888
N419	-4.975007092	-5.256444639	-5.500949101
N420	-5.482887987	-8.491870875	7.142457944
N421	-5.096055605	-5.275758611	4.172690085
N422	-6.752390661	-6.426863570	3.283036513
N423	-4.597158547	-6.392610598	9.412465570
N424	2.118919278	-0.397351298	-1.232501113
N425	1.596949465	-0.984898106	-0.426079861
O426	-6.359552193	5.489607658	-2.098181138
O427	-3.369161332	7.830725162	-2.486398288
O428	-3.946207862	8.961395640	1.007906684
O429	-2.497119253	5.740517864	1.864380332
O430	0.383548628	6.061011870	0.079669549
O431	1.224686110	9.116651517	1.690993362
O432	-0.372338816	8.822309173	5.387063923
O433	0.048185476	4.321086188	5.448956844
O434	3.296035774	3.707336130	3.746396518
O435	5.538809258	6.731615597	3.665035754
O436	4.820620303	9.179480917	6.054753131
O437	-6.112230411	-0.518665793	11.223795358
O438	-2.216095422	1.744149219	12.120690333
O439	1.227202031	-1.473529677	13.328576897
O440	-2.820828811	3.286032613	-4.039670507
O441	-6.615062138	-0.799849604	-2.501036989
O442	-0.396617773	3.157346949	-6.926661538
O443	-0.602052072	0.189611117	-7.694233451
O444	-1.313278928	6.898993153	-7.421065757
O445	0.895527163	6.628244984	-3.279445166
O446	4.225784605	4.902541530	-4.116498965
O447	5.808847835	7.193171435	-6.306992726
O448	4.771141311	-1.766793372	10.387460307
O449	6.595491495	1.066791141	2.397796203
O450	8.387418517	-3.417051061	-1.132121718
O451	7.981649218	-4.668923155	-5.145022172
O452	5.397776025	-1.400424915	-4.966366697
O453	6.862554546	1.354660379	-2.931971234
O454	6.360341917	-0.516172963	-0.056635319
O455	11.267321155	0.078393595	-3.660028830
O456	11.284323868	0.118408657	-7.894576598

	O457 8.079061761 -0.797261471 -8.144788978<br>O458 11.656888426 3.920071328 -8.590352716<br>O459 3.672742446 0.977512231 -7.957844798<br>O460 -6.333824937 -7.739980667 -0.187026614<br>O461 -3.620157316 -6.934555070 -3.393244321<br>O462 1.056296203 -8.642824011 -3.507163455<br>O463 2.732001092 -11.086463609 -0.895545990<br>O464 -6.955236954 -4.501362038 -6.342420451<br>O465 -8.377843850 -3.498819504 -0.527682027<br>O466 -7.227824352 -5.262743706 0.214131471<br>O467 -2.114931358 -5.261584994 -7.652823269<br>O468 -2.810357260 -7.649877694 8.811955151<br>O469 -2.717761417 -7.457941858 12.273390522<br>O470 -5.887652250 -0.619006220 2.540485336<br>O471 -6.355220109 1.460275082 3.300946706<br>O472 -9.206916543 -4.061451217 7.696955375<br>O473 -9.978449953 -3.005452393 5.852111065<br>O474 -4.059005890 -3.042815917 6.228059210<br>O475 -5.076324824 -1.498956916 7.506731439<br>O476 -5.386987066 -2.455277979 4.077122783<br>O477 -2.567021667 -2.926264243 10.538957995<br>O478 -1.727487486 -2.863383212 7.829249096<br>O479 1.848600015 -0.324922237 -6.581956309<br>O480 -7.573612052 -4.743340259 9.794523683<br>O481 -5.248716920 -2.820006789 9.859655712<br>S482 4.797998172 -4.318845103 -0.421499435<br>S483 1.184619673 -3.929741880 -0.586980236<br>S484 -3.396463101 -3.336895420 1.845391549<br>S485 2.814549997 -2.070014862 2.045546853<br>S486 -1.314351750 -1.813043466 -0.130106320<br>S487 -1.102569288 -6.503081965 1.142350850<br>S488 -2.049481940 -1.647123577 4.412445178<br>S489 2.354666261 -5.594170301 2.363406636<br>S490 -1.890696892 -5.325436056 4.554457327<br>S491 1.195190121 -3.314417009 5.329776931<br>end

TS

Fe(139) 2.309<br>Fe(140) 0.170<br>Fe(141) 2.739<br>Fe(142) -2.576<br>Fe(143) 3.085<br>Fe(144) -2.289<br>Fe(145) 0.009	bm612n2xnewbrk2bh347tf_1_53387.83<br>C1 -6.983682771 5.841201521 -3.109427015<br>C2 -8.444438714 5.456065326 -3.373994183<br>C3 -5.003252739 6.977896766 -4.020484621<br>C4 -4.556375518 7.833526686 -2.840984086<br>C5 -5.169350132 9.350135806 -1.029253169<br>C6 -4.742508721 8.502494319 0.173789324<br>C7 -4.793705925 6.327356237 1.280341778<br>C8 -3.363196874 5.861051520 0.977158072<br>C9 -1.757914620 5.325004272 -0.810689256<br>C10 -0.716435210 6.377816282 -0.402965980<br>C11 -0.051483055 8.692823437 -0.321923538<br>C12 0.105108216 9.017405987 1.168818245<br>C13 -1.029678040 9.668700514 3.237013618<br>C14 -0.850045092 8.558136857 4.276611946<br>C15 -1.250905683 6.190237176 4.792453696<br>C16 -0.265713546 5.063339306 4.494608020<br>C17 1.047825571 3.774821430 2.827203300<br>C18 2.529794927 4.198778089 2.898500378<br>C19 0.688497313 3.254109166 1.421058384<br>C20 1.632628886 2.140671313 0.963425897<br>C21 -0.780622596 2.843068242 1.407243607<br>C22 4.282427280 5.632315816 1.892682695<br>C23 4.526046276 6.720666006 2.944297390<br>C24 4.617327801 6.124774808 0.460314750<br>C25 5.992848993 6.804281006 0.406614364<br>C26 4.547656530 4.967413235 -0.541336044<br>C27 3.833751756 8.839470896 3.879714525<br>C28 4.175281452 8.463234797 5.305733040<br>C29 -6.978789765 1.691787167 11.645722351<br>C30 -5.884508902 0.708212320 11.220779670<br>C31 -3.515187181 0.593817063 10.425998082<br>C32 -2.494009991 0.660310751 11.578459008<br>C33 -2.965728777 1.223079808 9.129558727<br>C34 -1.694687398 0.552458445 8.589933505<br>C35 -1.354504889 1.067548175 7.184494182<br>C36 0.897980268 0.784836855 6.051724885<br>C37 -1.017563009 -0.638924151 13.037442878<br>C38 0.390786991 -0.970719232 12.595063717<br>C39 -4.755464597 1.846171844 -3.592374623<br>C40 -3.489097422 2.282907682 -4.354898533<br>C41 -4.328854554 1.122025211 -2.296408999

C42	-5.437719717	0.923879807	-1.241184253
C43	-6.282153407	-0.343762314	-1.380687554
C44	-1.904451820	1.468483921	-6.080155620
C45	-1.575017415	2.785912885	-6.791821838
C46	-1.879081686	0.266430585	-7.036729957
C47	-2.448946222	4.833152431	-7.811775484
C48	-1.786108126	5.877322633	-6.904986208
C49	-1.061474342	6.484860253	-4.645988001
C50	0.247584334	5.938317877	-4.075137225
C51	1.909710940	4.142714106	-4.000450265
C52	3.128838304	4.797966945	-4.676889845
C53	2.010214645	2.634331671	-4.275969777
C54	0.985869608	1.783175467	-3.601186622
C55	0.105430330	1.974937354	-2.555843251
C56	-0.178616148	-0.062735202	-3.187137810
C57	4.011663641	5.623255627	-6.815098073
C58	4.633257159	6.873055022	-6.195594765
C59	3.412248776	5.947233024	-8.204509526
C60	4.374639278	6.016113343	-9.352494701
C61	5.652819144	6.533015928	-9.436919559
C62	5.025109398	5.704852247	-11.394184050
C63	5.544680693	-1.846875696	8.088266436
C64	4.839440427	-2.367471928	9.325709000
C65	4.457929863	-1.237851651	7.163705113
C66	5.037968705	-0.611571213	5.917597261
C67	5.453727211	0.732639238	5.901956981
C68	5.184968476	-1.359742032	4.735424522
C69	5.980116062	1.316778988	4.744940188
C70	5.721946792	-0.794914590	3.578344840
C71	6.108147953	0.547180781	3.581758532
C72	7.323368724	-4.699679442	0.646632626
C73	7.904035664	-4.502203952	-0.768834468
C74	6.080204183	-3.844694424	0.809881530
C75	8.397028131	-5.554139506	-2.950246247
C76	7.521965419	-4.867354742	-4.002934299
C77	5.335056650	-3.761419067	-4.420632553
C78	5.382364679	-2.253361071	-4.062224327
C79	3.930445133	-4.366567580	-4.192888287
C80	2.719476076	-3.695028832	-4.850767141
C81	2.523010933	-3.946230892	-6.355886297
C82	3.842884699	-2.970950373	-8.243589751
C83	5.451551602	-0.518841435	-2.338791281
C84	6.795730302	0.150524760	-2.653840623
C85	5.173421209	-0.311446962	-0.834327532
C86	9.216406902	-0.101433372	-2.425220460
C87	10.057888791	-0.184992005	-3.687637626
C88	10.158058770	-0.903346982	-6.007050916
C89	10.465090983	0.267717629	-6.974051109
C90	9.596183425	-2.098300428	-6.784407518
C91	8.263005300	-1.812385459	-7.460446735
C92	9.883132092	2.538857730	-7.699280418
C93	11.230554011	3.244043108	-7.662742692
C94	8.749429014	3.558019318	-7.386293952
C95	7.371010642	2.948006235	-7.516077831
C96	6.700516019	2.957051155	-8.749898574
C97	6.742489114	2.312795036	-6.431612824
C98	5.458210410	2.337506590	-8.906260836
C99	5.513379079	1.662896358	-6.575207676
C100	4.883296617	1.679237178	-7.817549216
C101	-5.086250316	-9.166191536	1.308670558
C102	-5.209107727	-8.076701900	0.257007567
C103	-3.946135847	-6.377032549	-1.042733587
C104	-3.103148685	-6.748634675	-2.269820399
C105	-0.828727250	-7.242525632	-3.056727104
C106	0.380765538	-8.071233687	-2.633119340
C107	1.918387943	-8.815040322	-0.895007033
C108	1.779334659	-10.323764910	-0.932633831
C109	-7.061426366	-5.821682128	-4.314292745
C110	-6.330729478	-5.140791340	-5.477098108
C111	-7.079682916	-4.890841536	-3.080639909
C112	-8.121184107	-5.359306796	-2.047046648
C113	-7.944989940	-4.626267241	-0.735843691
C114	-4.202725890	-4.660495989	-6.575307206
C115	-3.104521762	-5.603383361	-7.025455956
C116	-3.742085540	-3.200463037	-6.338008172
C117	-3.030968087	-3.115423267	-5.021975145
C118	-1.675549957	-3.465033763	-4.921595978
C119	-3.775939186	-2.906896958	-3.847030026
C120	-1.110552434	-3.715441410	-3.669408975
C121	-3.209738199	-3.161141542	-2.599348812

C122	-1.894041221	-3.627632209	-2.520115874
C123	-4.530945974	-7.361899675	7.127072873
C124	-3.899993874	-7.131361051	8.507888073
C125	-5.188851663	-6.084678824	6.584173176
C126	-5.619885397	-6.123272134	5.152444670
C127	-6.650896804	-6.849311599	4.593521389
C128	-5.813264691	-5.479974292	3.067530200
C129	-4.005735332	-6.137228340	10.715841178
C130	-3.575715400	-7.413124164	11.404104775
C131	-5.983598658	0.287884277	3.473884209
C132	-5.518777912	-0.161961317	4.874522764
C133	-5.784692259	-1.652847542	5.215162357
C134	-7.273885346	-1.881198120	5.571549778
C135	-7.604782302	-3.293266350	6.062422790
C136	-9.051910933	-3.457254181	6.576971008
C137	-4.920893662	-2.073301181	6.413732590
C138	-0.214273527	-3.497709162	2.300512464
Fe139	-1.740584836	-4.773597199	2.358189975
Fe140	0.795422994	-1.983885845	0.743966016
Fe141	1.558815844	-3.607630321	3.161693038
Fe142	0.525678951	-5.002084212	1.246245623
Fe143	2.918846962	-4.060564092	0.867512113
Fe144	-0.706510219	-3.480060791	4.284231788
Fe145	-1.498940006	-2.056281326	2.083905716
H146	-8.836650420	4.569245935	-1.578692735
H147	-8.455149756	3.508236958	-2.757602923
H148	-6.936449035	6.842451836	-4.906604376
H149	-8.560244567	5.212898714	-4.442231927
H150	-9.050537213	6.362287226	-3.201464459
H151	-4.398581549	6.059007711	-3.984616508
H152	-6.452885556	8.548833237	-2.561170684
H153	-4.744886641	7.509130558	-4.945851443
H154	-6.047569507	9.946295158	-0.749555893
H155	-5.885195814	6.907545415	-0.469663544
H156	-4.339484286	10.040599166	-1.232594124
H157	-1.408217321	4.361747189	-0.431230498
H158	-3.851746737	5.699816457	-1.006537397
H159	-5.466588460	5.459309931	1.311755400
H160	0.928357705	8.355771872	-0.682302717
H161	-1.902018036	7.904237539	-1.117641435
H162	-4.784989811	6.794050751	2.272399282
H163	-1.811341553	5.253451823	-1.904196496
H164	-1.952302780	9.160248849	1.372285586
H165	-0.333642777	9.609825761	-0.855565478
H166	-1.449423420	3.704960107	1.518201807
H167	-1.037877103	2.323940578	0.468455942
H168	-0.987263513	2.198037585	2.265990036
H169	0.796644222	4.089809578	0.715430651
H170	-1.975467800	10.184534233	3.455319521
H171	3.557814193	4.490279177	-0.552489164
H172	2.789917514	7.748207222	2.394248828
H173	6.043258392	7.697436434	1.046404689
H174	1.349796579	1.817664320	-0.046638322
H175	1.596861255	1.256799194	1.616652601
H176	6.215974956	-1.027054343	8.407898756
H177	4.753599139	5.322283186	-1.560509827
H178	6.212869772	7.113466191	-0.625730065
H179	5.293444449	4.195574166	-0.292343385
H180	6.785748341	6.111900782	0.730448520
H181	3.858717183	6.878762730	0.178037738
H182	4.961236431	4.808682162	2.158088727
H183	2.216800219	5.500363517	1.328543370
H184	2.675252261	2.489267471	0.910930832
H185	4.381166690	-1.001960191	-0.512219122
H186	4.806369891	0.718561342	-0.712604022
H187	6.316671965	0.078989497	0.719142803
H188	6.785795865	2.018077272	2.512119964
H189	5.801360826	-1.379295079	2.662348187
H190	4.842433547	-2.396131323	4.705970444
H191	5.350773308	1.341866421	6.803554472
H192	-0.191465585	5.499915558	2.484927442
H193	-2.248859430	5.725279061	4.826981295
H194	-1.010082942	6.553124967	5.798255891
H195	-1.759234053	7.200190956	3.005065692
H196	-0.207464033	10.378836841	3.395453963
H197	6.269755088	2.370814294	4.742583349
H198	0.922629765	2.979324895	3.571246126
H199	4.643830585	9.478021064	3.486856812
H200	2.913351825	9.443259426	3.906711595
H201	3.726351943	7.509594283	5.667294567

	H202	-1.351614161	-1.381682351	13.778664942
	H203	-7.264823619	-1.757350173	-0.292369239
	H204	-5.540282494	-1.786272061	3.255669826
	H205	-9.130520437	-5.145755431	-2.426848199
	H206	-8.036532919	-6.443710133	-1.885582636
	H207	-7.316056048	-3.858817000	-3.382924618
	H208	-3.288731395	-6.676581343	-6.782221202
	H209	-8.096934649	-5.906329000	-4.677547966
	H210	-3.879677990	0.153165746	-2.565850224
	H211	-6.136009846	1.779109406	-1.261934771
	H212	-4.483843130	-5.753775650	-4.748773105
	H213	-4.894371575	-4.635372454	-7.443167033
	H214	-5.650243062	-7.188682264	-3.642304273
	H215	-6.366838722	-0.618895555	0.708298051
	H216	-6.072732885	-4.865609303	-2.636977556
	H217	-7.209120600	-7.690482985	-3.478585936
	H218	-6.287900270	1.529792855	-4.943066762
	H219	-3.524883041	1.714779688	-1.838194605
	H220	-6.934926093	-6.178167091	-0.080604156
	H221	-4.986065015	0.937362325	-0.238222140
	H222	2.004230135	4.351262174	-2.926175716
	H223	-0.053927627	2.879733017	-1.978664973
	H224	-0.810037481	7.411408821	-5.180538749
	H225	-2.190933094	4.756426831	-5.197808747
	H226	1.989594603	5.090070471	-6.360330210
	H227	6.301975833	6.995539008	-8.700990919
	H228	3.898316213	7.513386151	-5.649777105
	H229	2.819370615	6.876291873	-8.131430394
	H230	4.790076247	4.849616100	-6.906068135
	H231	5.079297486	5.425831688	-12.443225391
	H232	3.022463445	2.324432465	-3.965012980
	H233	1.956442611	2.475197240	-5.366028383
	H234	6.950788837	6.578790400	-11.136463143
	H235	2.696733213	5.145681340	-8.446988790
	H236	0.103367556	4.174165454	-5.166373711
	H237	-1.703248122	6.773257760	-3.799633078
	H238	-1.823139499	4.777503513	-8.713355340
	H239	-0.499689364	-1.099353774	-3.263232692
	H240	-3.432244510	5.216336151	-8.114153985
	H241	7.082620846	-3.193318909	7.938871438
	H242	6.521244279	-2.679131472	6.492096001
	H243	5.231885723	-2.644475387	-2.011056635
	H244	4.687626752	0.029825328	-2.907764568
	H245	7.747915684	-1.584959292	-2.113810933
	H246	1.257431501	0.030580098	-4.797421028
	H247	0.967953950	-0.123321714	-6.990578506
	H248	-0.705748652	-0.413751431	-8.451828428
	H249	2.478054165	0.348700488	-6.923085194
	H250	-2.064140205	-0.644129232	-6.443169902
	H251	-2.677342731	0.374060724	-7.787936306
	H252	9.541145885	-2.974881451	-6.123948253
	H253	10.320291923	-2.323062803	-7.582634345
	H254	7.435227379	-3.470677714	-6.543190775
	H255	6.371470960	-2.489189441	-7.547779923
	H256	4.978814196	-4.245076943	-9.379058870
	H257	1.451852400	-4.058200474	-6.581125483
	H258	4.946397911	2.365493979	-9.872174590
	H259	7.200486573	2.321089549	-5.439054595
	H260	4.163195369	-0.938137536	-8.413745928
	H261	3.747706901	-4.370917809	-3.106194022
	H262	5.999010440	-4.738693884	-2.645247402
	H263	2.707945022	-2.612026344	-4.650044556
	H264	-3.565879291	3.176059182	-7.024380362
	H265	8.574808169	-6.584934285	-3.285543632
	H266	-3.902994001	0.731356685	-5.528817308
	H267	3.970571061	-5.424501079	-4.500868699
	H268	5.074899525	1.125564111	-5.734144715
	H269	9.362210645	-5.027573363	-2.959098639
	H270	3.780448057	-5.022996799	-8.404527920
	H271	4.727107501	-1.897240676	-9.744152561
	H272	-6.067681541	0.282921930	-3.850626155
	H273	-5.259799211	2.787746482	-3.301509402
	H274	7.151262667	3.458270554	-9.609415274
	H275	-1.103560469	1.344717001	-5.336528472
	H276	5.633725405	-3.838707979	-5.469899858
	H277	3.004017131	-4.892161399	-6.637586662
	H278	1.835837643	-4.102618650	-4.342473508
	H279	2.661521427	-1.910404344	-6.971817640
	H280	9.146192423	0.959893430	-2.142398575
	H281	8.428990257	-0.866929664	-4.720445273

H282	11.157144000	-1.198619485	-5.649624044
H283	9.770464674	-0.625956877	-1.635117682
H284	9.098959416	1.438619741	-6.036354971
H285	-4.104955609	-9.657489528	1.227051986
H286	-5.099220953	-8.640289812	2.285893408
H287	-3.691674581	-7.643843502	6.473414150
H288	-6.223389924	-10.705788543	2.008169538
H289	-7.042432339	-9.658482533	1.051795151
H290	5.691904546	-3.987297539	1.826011421
H291	8.631913267	-3.378699868	1.483340989
H292	7.444681009	-6.447675593	-1.258461784
H293	6.326501934	-2.784318690	0.658251456
H294	9.124610351	-4.932901118	1.612005297
H295	2.127852101	-8.524321117	0.147576666
H296	7.056239687	-5.758556047	0.791502494
H297	-2.780687667	2.294842153	9.313187650
H298	-3.792222568	-0.450362070	10.233944094
H299	0.182607879	-7.613308360	-0.601029521
H300	2.642110056	0.194759394	5.176411788
H301	-1.356618635	-7.806311681	-3.835511963
H302	-3.788782674	-2.997720438	-1.687858467
H303	-0.431423681	-6.332352532	-3.539181573
H304	-1.110997097	2.137243685	7.207311392
H305	-1.458260308	-6.583735879	-1.083929001
H306	-2.235483847	0.950866170	6.531873187
H307	-3.770744186	1.147651697	8.381263368
H308	-1.841956941	-0.537054484	8.534485673
H309	-0.841687315	0.733681580	9.263076656
H310	-4.633807199	-2.557429126	-6.353950309
H311	-4.705692618	2.307154974	10.974997938
H312	-7.706899934	1.705874660	10.820483944
H313	-1.452861801	-3.878462938	-1.560964111
H314	-6.215364275	3.174394838	12.853451985
H315	-7.227984224	3.733704654	11.693525800
H316	-0.469268765	-0.660068519	6.352846385
H317	-0.060243426	-3.993888066	-3.569871225
H318	-4.813683959	-2.572490517	-3.915715848
H319	-6.887233380	-4.041906429	9.844881387
H320	-6.077683500	-5.852055270	7.195727945
H321	-5.149156688	-2.421288327	8.952393531
H322	-8.116744192	-4.483801700	8.988556265
H323	0.728733023	-10.699198764	-0.987460867
H324	-2.565768584	-2.929602931	7.299110730
H325	-5.976636345	0.481284324	5.636167586
H326	-3.193269849	-7.733135503	0.395042670
H327	-7.523523879	-1.154582901	6.360772454
H328	-7.459652959	-6.719317114	2.613956389
H329	-5.691418505	-4.957325496	2.127121315
H330	-7.489653974	1.236440840	12.511166399
H331	1.487821266	-1.081879714	5.362649113
H332	-2.163672533	-2.971741647	9.639962607
H333	-1.084015471	-3.398349420	7.335616682
H334	-2.218922672	-1.401330497	11.420891075
H335	0.643885351	2.779530321	6.431017605
H336	-7.461358673	-4.006173001	5.235096352
H337	-6.916571117	-3.588596181	6.866696510
H338	-7.889353601	-1.629746283	4.693814444
H339	-3.512688201	-5.508370318	-0.523046828
H340	-1.085171488	-3.594199610	-5.830929772
H341	-3.095307025	-2.919853480	-7.183426052
H342	-4.430644081	0.018086915	4.900085249
H343	-3.546533494	-2.959257147	10.351208024
H344	-5.564855795	-2.044884051	10.392151025
H345	2.087680660	2.378717747	5.673123488
H346	-7.315262557	-7.590159105	5.022936062
H347	-4.497751516	-5.242719624	6.715680117
H348	-4.951863739	-6.127689076	-1.385428262
H349	2.785342918	-8.518783778	-1.504710234
H350	3.911296369	-0.482010911	7.748116166
H351	3.746604738	-2.033868615	6.889173952
H352	-4.990741245	-9.330809718	7.474393936
H353	-6.220989609	-8.308102331	7.823987779
H354	-4.778030398	-5.669251078	11.346863459
H355	-5.491700973	-5.950885353	9.196491545
H356	-4.154962751	-8.321350034	11.111876492
H357	-3.155539948	-5.438485349	10.663599766
H358	4.355320787	-3.364654727	9.189237572
H359	11.798057841	3.127477827	-6.708756811
H360	-0.976981402	0.345190332	13.537343621
H361	0.630616141	-0.699175611	11.537895370

H362	9.739972133	2.141596974	-8.717247367
H363	8.898715370	3.957376900	-6.370805328
H364	8.864523906	4.396018443	-8.089135565
H365	-2.505249176	-0.551471685	2.039523055
H366	3.238291515	1.273884108	-8.779661911
H367	-1.491658205	0.552043758	1.242924455
H368	0.323245222	-0.650403277	3.089537521
H369	-1.583904971	0.495651733	2.954323912
H370	-1.328105409	-0.491593179	-0.469259941
Mo371	-3.466942916	-3.630078481	4.193020434
N372	0.023048184	-0.762338230	2.110889494
N373	-1.524943777	-0.052402393	2.077884964
N374	-8.977780761	4.367745736	-2.571713865
N375	-6.421570535	6.659821059	-4.048895809
N376	-5.496617179	8.578324954	-2.216550287
N377	-5.293563749	7.265922491	0.282937224
N378	-3.101134014	5.611196479	-0.326629841
N379	-1.048452053	7.672503232	-0.604202192
N380	-1.049968217	9.242770006	1.848405902
N381	-1.266298746	7.321237869	3.891563965
N382	0.153755808	4.880894951	3.217881480
N383	2.914477449	5.121630466	1.970249742
N384	3.584485893	7.694923441	3.032565652
N385	-6.486124665	3.055834332	11.874674828
N386	-4.728459296	1.291145324	10.839300143
N387	-0.253389195	0.319851273	6.560819245
N388	1.331996560	2.042050259	6.271853130
N389	1.665312959	-0.056901092	5.323959089
N390	-1.954984288	-0.531725722	11.936353764
N391	-5.583146516	0.981732156	-4.444419048
N392	-6.677409891	-0.920524591	-0.231392094
N393	-3.196182996	1.476145591	-5.410031546
N394	-2.628955002	3.515118493	-7.230836928
N395	-1.777718056	5.622908515	-5.567779319
N396	0.649497408	4.699934954	-4.478970500
N397	0.785667278	0.473271202	-3.994186515
N398	-0.614004206	0.826321587	-2.303362820
N399	2.925008852	5.163037996	-5.968433068
N400	3.993838150	5.503140430	-10.582560196
N401	6.049935614	6.323374430	-10.740916682
N402	6.225461328	-2.964278068	7.429729679
N403	8.301206988	-4.328376822	1.683420157
N404	7.867303389	-5.587572394	-1.598225525
N405	6.273649467	-4.537782443	-3.615192875
N406	3.043521445	-2.855309252	-7.186112808
N407	4.160000349	-4.163361557	-8.784191557
N408	4.402138592	-1.862891760	-8.781963128
N409	5.410316578	-1.927836638	-2.739372298
N410	7.884584237	-0.662250280	-2.534918248
N411	9.394898281	-0.555423498	-4.825789176
N412	7.314300547	-2.774374660	-7.290276702
N413	9.840170962	1.447282851	-6.732636984
N414	-6.153020116	-10.157403418	1.148332610
N415	-4.051185224	-7.490479961	-0.108766409
N416	-1.784297382	-6.887179430	-2.022158326
N417	0.712975593	-8.122447001	-1.318226823
N418	-6.570174751	-7.189440801	-4.099728262
N419	-4.972741437	-5.255825464	-5.499849002
N420	-5.477659163	-8.492846490	7.142796086
N421	-5.099230478	-5.277281883	4.174080841
N422	-6.753602141	-6.429410505	3.285005883
N423	-4.595962778	-6.393553227	9.414115877
N424	2.144494054	-0.371670598	-1.273655454
N425	1.619639080	-0.955803752	-0.464803090
O426	-6.357745599	5.483973892	-2.097460660
O427	-3.365746580	7.820896247	-2.481057087
O428	-3.939630855	8.954944250	1.006407899
O429	-2.500512730	5.740091968	1.866348843
O430	0.380541054	6.051303769	0.079592817
O431	1.225278417	9.108304761	1.697584401
O432	-0.381448835	8.821518642	5.392490531
O433	0.062205144	4.321553163	5.435771789
O434	3.304743513	3.695060054	3.724352423
O435	5.536663462	6.726638452	3.665928650
O436	4.831751190	9.184019225	6.043131997
O437	-6.115530309	-0.518128122	11.226461555
O438	-2.217085971	1.744099887	12.123890694
O439	1.225993482	-1.478020651	13.327183436
O440	-2.807481658	3.274488965	-4.031191936
O441	-6.612692566	-0.799296268	-2.501801554

	O442 -0.396385152 3.160167523 -6.925249522<br>O443 -0.599370464 0.190369778 -7.695421474<br>O444 -1.310073655 6.900066579 -7.418995722<br>O445 0.894705791 6.626896256 -3.274735851<br>O446 4.225107569 4.898787700 -4.116392158<br>O447 5.804197158 7.195902292 -6.309545210<br>O448 4.774643129 -1.764917810 10.387788534<br>O449 6.598332592 1.068448962 2.398442611<br>O450 8.387741321 -3.417407426 -1.135892581<br>O451 7.981271079 -4.664169156 -5.144889899<br>O452 5.398748138 -1.401228948 -4.965704614<br>O453 6.864421635 1.354144188 -2.933157025<br>O454 6.358809401 -0.515695141 -0.056696669<br>O455 11.266948498 0.077775496 -3.663172294<br>O456 11.284774884 0.118609551 -7.891644251<br>O457 8.078779652 -0.797179118 -8.146809057<br>O458 11.655618917 3.917700883 -8.588481544<br>O459 3.671240291 0.976511404 -7.955987793<br>O460 -6.333663072 -7.739899386 -0.189074817<br>O461 -3.618734875 -6.929398880 -3.393580550<br>O462 1.057200088 -8.637460747 -3.508718049<br>O463 2.730433095 -11.086480386 -0.880849274<br>O464 -6.953030510 -4.501860976 -6.342465480<br>O465 -8.375394587 -3.497716358 -0.527869752<br>O466 -7.228073270 -5.262729379 0.215005546<br>O467 -2.111867540 -5.259061378 -7.650368492<br>O468 -2.807965483 -7.649853737 8.814206123<br>O469 -2.718272168 -7.457783349 12.276199536<br>O470 -5.879004034 -0.609641803 2.534409908<br>O471 -6.359079035 1.464128644 3.301828647<br>O472 -9.209851591 -4.061208814 7.696571697<br>O473 -9.982728366 -3.010321080 5.849372072<br>O474 -4.060592850 -3.037527071 6.228175219<br>O475 -5.084704945 -1.496885560 7.508070361<br>O476 -5.393636089 -2.456780876 4.078188259<br>O477 -2.568531181 -2.925542685 10.541762180<br>O478 -1.732604680 -2.863589999 7.830925102<br>O479 1.848205868 -0.322968723 -6.575843947<br>O480 -7.576904440 -4.740788941 9.796032361<br>O481 -5.250627014 -2.819056586 9.861377161<br>S482 4.791379061 -4.324756692 -0.411812875<br>S483 1.245365868 -3.905156701 -0.573336336<br>S484 -3.423183783 -3.420978542 1.829585772<br>S485 2.779689387 -2.056276317 2.034117553<br>S486 -1.297039016 -1.834314675 -0.184159745<br>S487 -1.088861939 -6.512760738 1.118770013<br>S488 -2.027661448 -1.692990786 4.398728261<br>S489 2.330440864 -5.622952671 2.407911083<br>S490 -1.888475420 -5.348250676 4.555095323<br>S491 1.207601733 -3.323053357 5.343042002<br>end

product

Fe(139) 2.582<br>Fe(140) 0.005<br>Fe(141) 2.513<br>Fe(142) -2.547<br>Fe(143) 3.131<br>Fe(144) -1.542<br>Fe(145) -0.347	bm612n2xnewbrk2bh347tf_5_53456.90<br>C1 -6.975539592 5.834382682 -3.103226323<br>C2 -8.436813075 5.446032827 -3.360741239<br>C3 -5.009800124 6.991291487 -4.025004855<br>C4 -4.565355623 7.852036807 -2.848063171<br>C5 -5.183557241 9.358076617 -1.029621014<br>C6 -4.750615691 8.513522207 0.173721438<br>C7 -4.776566621 6.333106660 1.272536889<br>C8 -3.344296949 5.875551505 0.967970372<br>C9 -1.734206420 5.347882827 -0.817771264<br>C10 -0.696401077 6.405666494 -0.413998010<br>C11 -0.055052148 8.731349487 -0.342964974<br>C12 0.106483548 9.042956909 1.150108874<br>C13 -1.023634655 9.669073216 3.227680614<br>C14 -0.830001505 8.558247264 4.265305220<br>C15 -1.265688221 6.198322449 4.800194891<br>C16 -0.288706057 5.057783494 4.522728347<br>C17 1.036801299 3.774311317 2.849936738<br>C18 2.514914415 4.209361762 2.935880539<br>C19 0.672510899 3.243320621 1.451313685<br>C20 1.619100501 2.127360512 1.007732953<br>C21 -0.788174539 2.798977372 1.450109517<br>C22 4.275097589 5.626701001 1.914457655<br>C23 4.522911603 6.726654712 2.956018233<br>C24 4.603217640 6.108546149 0.476654166<br>C25 5.976473423 6.790347480 0.410524699<br>C26 4.533680537 4.949112214 -0.523551364

C27	3.818704591	8.843838671	3.900832471
C28	4.155882869	8.452807391	5.324985750
C29	-6.964712153	1.681154320	11.642107465
C30	-5.870015283	0.702985614	11.209753566
C31	-3.503021334	0.596872881	10.411558496
C32	-2.483890010	0.662351679	11.566031062
C33	-2.956205398	1.232547526	9.117892780
C34	-1.684113580	0.566276334	8.579597890
C35	-1.325106684	1.091355633	7.183538466
C36	0.915948262	0.757733282	6.050820080
C37	-1.015340239	-0.638148072	13.031625696
C38	0.396688552	-0.963779894	12.595973152
C39	-4.754156333	1.840444842	-3.583057065
C40	-3.497902158	2.289563326	-4.354680598
C41	-4.321471678	1.110213147	-2.292940635
C42	-5.431404960	0.915576082	-1.238180512
C43	-6.279391295	-0.349952511	-1.375081437
C44	-1.902805320	1.466795357	-6.073635663
C45	-1.574491690	2.780644341	-6.791253759
C46	-1.882274606	0.264201259	-7.029964703
C47	-2.444776970	4.823444910	-7.819644418
C48	-1.788166761	5.871880690	-6.913132861
C49	-1.058637759	6.482560982	-4.657771514
C50	0.251690050	5.939288787	-4.085733674
C51	1.916375746	4.145684051	-4.001966927
C52	3.134380825	4.803362045	-4.678241578
C53	2.017309791	2.636331193	-4.274314200
C54	0.985555618	1.795169659	-3.597878764
C55	0.088707774	2.012644359	-2.571917556
C56	-0.213622419	-0.027803679	-3.183570809
C57	4.020312007	5.623116814	-6.819776740
C58	4.646799145	6.867873246	-6.196194948
C59	3.419158567	5.944335413	-8.207643599
C60	4.381177163	6.015813987	-9.355109523
C61	5.657843939	6.536521826	-9.438746399
C62	5.033222840	5.706715766	-11.396282759
C63	5.550604319	-1.843903929	8.096803779
C64	4.841183619	-2.369488621	9.329505123
C65	4.463179735	-1.235350698	7.172160930
C66	5.041301788	-0.612067785	5.923735364
C67	5.451668512	0.733474769	5.903105459
C68	5.189052173	-1.363249518	4.743772221
C69	5.974796310	1.315822016	4.743620161
C70	5.723003448	-0.800313364	3.584199942
C71	6.104847543	0.542801912	3.582780661
C72	7.344914487	-4.701735675	0.650196416
C73	7.924499355	-4.502939196	-0.765283206
C74	6.099159624	-3.844647067	0.805440061
C75	8.407455995	-5.553127744	-2.950330892
C76	7.531678281	-4.870896634	-4.005877524
C77	5.348745538	-3.758435594	-4.429165043
C78	5.388618263	-2.249862201	-4.069369728
C79	3.944117137	-4.366676556	-4.201477774
C80	2.728618266	-3.699681241	-4.856267233
C81	2.528647258	-3.950627839	-6.361103447
C82	3.845348409	-2.975720653	-8.253531282
C83	5.453047501	-0.519710354	-2.338504723
C84	6.795242701	0.154260821	-2.653994315
C85	5.177331520	-0.317647259	-0.832500200
C86	9.216491362	-0.094136663	-2.428624308
C87	10.065485285	-0.180890017	-3.687164565
C88	10.173676018	-0.896616922	-6.012170382
C89	10.474396803	0.271552986	-6.983554827
C90	9.606803282	-2.095818548	-6.780248726
C91	8.273182385	-1.814013254	-7.457980160
C92	9.891771353	2.544544210	-7.707995864
C93	11.238696812	3.249570866	-7.672417027
C94	8.754538533	3.558516342	-7.387164610
C95	7.376267389	2.946332872	-7.515092901
C96	6.706929777	2.953133542	-8.749629209
C97	6.744811145	2.315186721	-6.429573395
C98	5.464414638	2.334580923	-8.906569005
C99	5.514309952	1.667616460	-6.573294917
C100	4.886958640	1.679989625	-7.817214673
C101	-5.079825996	-9.160703236	1.317738240
C102	-5.208548278	-8.076163515	0.263122422
C103	-3.944777508	-6.386165175	-1.047231097
C104	-3.104193197	-6.764164116	-2.273063366
C105	-0.829693398	-7.260851597	-3.058381446
C106	0.379690850	-8.087967393	-2.633235616

C107	1.918375950	-8.828100242	-0.896076161
C108	1.784920841	-10.336780565	-0.957442763
C109	-7.056137565	-5.819650817	-4.304600533
C110	-6.326152005	-5.139273548	-5.467352997
C111	-7.075256071	-4.890846569	-3.070338898
C112	-8.122592350	-5.358990942	-2.042931102
C113	-7.949111409	-4.626731710	-0.731505760
C114	-4.201365289	-4.665057865	-6.570176407
C115	-3.104081679	-5.608408244	-7.020608976
C116	-3.746696304	-3.201112332	-6.344148287
C117	-3.028593079	-3.113847141	-5.031961217
C118	-1.673833217	-3.469202246	-4.940105582
C119	-3.768089334	-2.915918224	-3.852263994
C120	-1.104526110	-3.733418242	-3.692497279
C121	-3.197184435	-3.184272502	-2.608456151
C122	-1.880556383	-3.648546107	-2.536323872
C123	-4.540522256	-7.362686568	7.125073671
C124	-3.906866840	-7.133059977	8.504113869
C125	-5.188392087	-6.080007390	6.581961198
C126	-5.614327172	-6.113513764	5.149008088
C127	-6.646088932	-6.837552818	4.588446091
C128	-5.802043830	-5.469051457	3.064978777
C129	-3.999840179	-6.135343470	10.708124046
C130	-3.575436661	-7.411625135	11.399706700
C131	-5.952887375	0.298753812	3.478895474
C132	-5.504256176	-0.154094062	4.882373223
C133	-5.772414174	-1.645143301	5.218541925
C134	-7.258702473	-1.878477417	5.576714094
C135	-7.588498269	-3.290843558	6.068045924
C136	-9.036494958	-3.450905966	6.582890976
C137	-4.902675808	-2.065650947	6.412927710
C138	-0.194507820	-3.501568294	2.297257574
Fe139	-1.755459333	-4.778108894	2.334150596
Fe140	0.817414759	-2.111916294	0.883439212
Fe141	1.563798138	-3.691315977	3.144163040
Fe142	0.522665873	-5.039142647	1.219921429
Fe143	2.915586740	-4.115154530	0.771473253
Fe144	-0.715244215	-3.385820117	4.167645676
Fe145	-1.353995782	-1.789917294	2.195372642
H146	-8.825329976	4.564230421	-1.561944086
H147	-8.446213039	3.499658698	-2.738829590
H148	-6.947116731	6.837269022	-4.898551763
H149	-8.556179375	5.200224924	-4.428041696
H150	-9.042213191	6.352964684	-3.188362621
H151	-4.393489619	6.079824432	-3.993967681
H152	-6.468959149	8.544292777	-2.553424397
H153	-4.764506275	7.526423969	-4.951685221
H154	-6.062372641	9.952585081	-0.748234045
H155	-5.872772293	6.905686488	-0.475902570
H156	-4.355847061	10.050265725	-1.235670030
H157	-1.384356356	4.385232187	-0.433839344
H158	-3.824020633	5.745858021	-1.018650306
H159	-5.442351320	5.459648319	1.303789376
H160	0.927376110	8.415485492	-0.715559596
H161	-1.890737972	7.919425425	-1.144781424
H162	-4.769130326	6.798330554	2.265253247
H163	-1.788002396	5.275978451	-1.911539082
H164	-1.951460205	9.171579585	1.362792548
H165	-0.357365745	9.649011143	-0.865129827
H166	-1.470304874	3.649215986	1.576255510
H167	-1.036454610	2.283948969	0.509169644
H168	-0.964352954	2.112396230	2.286863540
H169	0.771906334	4.075276969	0.737920488
H170	-1.974442996	10.172498732	3.453875762
H171	3.550900832	4.457107500	-0.524704088
H172	2.771135572	7.733093730	2.425931897
H173	6.030332944	7.685439871	1.046902638
H174	1.358095078	1.815027347	-0.011474241
H175	1.537525978	1.239967312	1.652077364
H176	6.221294948	-1.024701198	8.419042108
H177	4.721483154	5.310928069	-1.544152992
H178	6.187868885	7.095282110	-0.624895610
H179	5.295233019	4.188267636	-0.288903218
H180	6.772182887	6.099531068	0.730985626
H181	3.840129252	6.858191513	0.195290548
H182	4.958358082	4.808137494	2.186058549
H183	2.220549466	5.458353870	1.323406838
H184	2.669804795	2.455134992	0.994027132
H185	4.387386814	-1.011591032	-0.510454646
H186	4.808007617	0.711612970	-0.711716656

H187	6.324155283	0.070864380	0.721481218
H188	6.774401231	2.012642722	2.505903729
H189	5.801906724	-1.387405630	2.669848493
H190	4.847928564	-2.400177775	4.716625919
H191	5.344705913	1.345865411	6.802042796
H192	-0.203854832	5.496355346	2.520112865
H193	-2.269597190	5.745854277	4.820460925
H194	-1.037895192	6.567019848	5.807049651
H195	-1.747873439	7.201239340	3.001199266
H196	-0.208798173	10.388142049	3.383953975
H197	6.260040477	2.371084137	4.738123798
H198	0.915034101	2.975005919	3.591236515
H199	4.624623888	9.490775538	3.514747021
H200	2.891953246	9.437755845	3.929331185
H201	3.718296472	7.486926634	5.668312396
H202	-1.350511423	-1.384647668	13.768616077
H203	-7.261187512	-1.761720463	-0.282011055
H204	-5.500582779	-1.764484241	3.253678247
H205	-9.129486389	-5.143635420	-2.428172084
H206	-8.040203190	-6.443870556	-1.882937252
H207	-7.308702210	-3.858079931	-3.372415574
H208	-3.285976785	-6.680588504	-6.771141403
H209	-8.091955577	-5.900776917	-4.667554574
H210	-3.876814443	0.140453583	-2.566412180
H211	-6.127632223	1.772364876	-1.262858742
H212	-4.480373476	-5.760530405	-4.744028705
H213	-4.894844439	-4.647467908	-7.436801835
H214	-5.651188717	-7.196659713	-3.636755647
H215	-6.348128034	-0.627103236	0.711051740
H216	-6.070364272	-4.867327865	-2.622088849
H217	-7.213383616	-7.693842775	-3.481262413
H218	-6.285692036	1.528995255	-4.933885724
H219	-3.513530753	1.696807931	-1.834303097
H220	-6.935820138	-6.177130631	-0.077631360
H221	-4.982478902	0.932639144	-0.234341643
H222	2.010206728	4.354674315	-2.927564504
H223	-0.070234154	2.930441606	-2.015045379
H224	-0.806395267	7.406024620	-5.197117348
H225	-2.193400615	4.754102154	-5.203031864
H226	1.997155523	5.089624822	-6.362850878
H227	6.305507462	7.000454565	-8.702286849
H228	3.913639794	7.510406005	-5.650778057
H229	2.822053233	6.870515307	-8.134062281
H230	4.794945298	4.845445955	-6.910101969
H231	5.088242992	5.428413907	-12.445372078
H232	3.028701103	2.325109339	-3.962130931
H233	1.963489985	2.474734821	-5.364102759
H234	6.955575345	6.587838691	-11.138048393
H235	2.707149204	5.139114270	-8.448684942
H236	0.104565943	4.164952374	-5.160052170
H237	-1.699555400	6.776267340	-3.812567081
H238	-1.810359937	4.763152995	-8.715055857
H239	-0.561012038	-1.055379292	-3.262854879
H240	-3.425322986	5.204665576	-8.133251860
H241	7.087473454	-3.192675115	7.938907297
H242	6.522087740	-2.671913984	6.495564227
H243	5.252522610	-2.647337450	-2.017710806
H244	4.686124135	0.029868832	-2.903043444
H245	7.750841189	-1.580139249	-2.118211129
H246	1.249719553	0.024496550	-4.769234803
H247	0.967782451	-0.126663487	-6.985492367
H248	-0.707445436	-0.431729905	-8.435737863
H249	2.477060457	0.349857643	-6.925135642
H250	-2.072364825	-0.645316045	-6.436349043
H251	-2.679464272	0.376276007	-7.781767123
H252	9.550279233	-2.967444112	-6.113141307
H253	10.328327245	-2.330481722	-7.577785854
H254	7.449779169	-3.484658275	-6.555852385
H255	6.388726691	-2.507248526	-7.568604198
H256	4.970626753	-4.253728839	-9.395524699
H257	1.456593526	-4.060450243	-6.583357563
H258	4.953687271	2.360285463	-9.873174426
H259	7.200820563	2.324810102	-5.436093785
H260	4.177536955	-0.945599798	-8.406015292
H261	3.761994837	-4.371460001	-3.114656678
H262	6.011410638	-4.722087829	-2.645524347
H263	2.711809831	-2.617033611	-4.654007194
H264	-3.565099250	3.174898744	-7.019474010
H265	8.587595508	-6.584043313	-3.283757251
H266	-3.889402635	0.718132432	-5.507648838

H267	3.985804793	-5.424874467	-4.508387308
H268	5.071668734	1.135183900	-5.731212157
H269	9.371184860	-5.024189307	-2.960547647
H270	3.764447200	-5.025499635	-8.425375082
H271	4.723686620	-1.893494442	-9.750351504
H272	-6.073006022	0.282196278	-3.840090120
H273	-5.259087444	2.778635061	-3.283105747
H274	7.159102391	3.451929326	-9.609720754
H275	-1.098133978	1.342928059	-5.334188278
H276	5.649498936	-3.836334635	-5.477666069
H277	3.007383537	-4.897720518	-6.643060217
H278	1.848461668	-4.112503459	-4.345798336
H279	2.671024933	-1.914766609	-6.975036693
H280	9.141001291	0.968332264	-2.151327706
H281	8.442003251	-0.858282406	-4.728860975
H282	11.173015518	-1.189991531	-5.654389208
H283	9.768050058	-0.612907696	-1.633002928
H284	9.117195297	1.446750501	-6.037750212
H285	-4.099090133	-9.652537285	1.232318764
H286	-5.091173013	-8.634168367	2.294279382
H287	-3.703425161	-7.652064750	6.472028508
H288	-6.218212005	-10.704473503	2.014588345
H289	-7.036750912	-9.653148150	1.061907194
H290	5.688967907	-3.990745910	1.812430081
H291	8.657668416	-3.387850982	1.497528076
H292	7.451087335	-6.443112681	-1.259044346
H293	6.349836271	-2.783716893	0.662969022
H294	9.142147199	-4.944937259	1.618144874
H295	2.117485506	-8.551109919	0.152146340
H296	7.074116479	-5.759782565	0.794479089
H297	-2.775140990	2.304340929	9.304996516
H298	-3.777405401	-0.447422704	10.216117404
H299	0.186379760	-7.621809340	-0.603606006
H300	2.638998790	0.161534677	5.132530359
H301	-1.356487840	-7.826292178	-3.836655941
H302	-3.778280724	-3.044872115	-1.694713932
H303	-0.434555583	-6.349865238	-3.540639706
H304	-1.048934558	2.152058759	7.220974391
H305	-1.460371530	-6.598856066	-1.088630235
H306	-2.204295090	1.010944930	6.522338923
H307	-3.760418249	1.156548389	8.368762168
H308	-1.832712689	-0.522410289	8.513310327
H309	-0.835224043	0.738271956	9.260458423
H310	-4.642216665	-2.563325109	-6.357753205
H311	-4.697379667	2.306178915	10.961836728
H312	-7.702084147	1.690292427	10.825157406
H313	-1.439116787	-3.912393767	-1.579366635
H314	-6.205167128	3.170980656	12.843868375
H315	-7.224511654	3.722097581	11.686531924
H316	-0.488999874	-0.649878916	6.336837823
H317	-0.057800659	-4.028779550	-3.607208613
H318	-4.806382948	-2.582149410	-3.914070349
H319	-6.876016117	-4.046051816	9.843534236
H320	-6.077368111	-5.843026652	7.191644231
H321	-5.133463329	-2.428123409	8.945593948
H322	-8.103424205	-4.490573054	8.985642650
H323	0.734487375	-10.714414852	-0.998011089
H324	-2.544651144	-2.937697399	7.296683330
H325	-5.965831559	0.487262220	5.642264782
H326	-3.191869276	-7.749017539	0.384477312
H327	-7.507608893	-1.152795409	6.366604524
H328	-7.451261579	-6.705939490	2.608718081
H329	-5.678093108	-4.946627543	2.124449416
H330	-7.463054201	1.224395001	12.514159250
H331	1.481788605	-1.118048512	5.356575568
H332	-2.154099514	-2.971448227	9.633634198
H333	-1.061068481	-3.401611626	7.340870461
H334	-2.211120515	-1.400138337	11.411657804
H335	0.674068843	2.774527907	6.339250850
H336	-7.447557694	-4.005315215	5.241690421
H337	-6.900722903	-3.587196845	6.872370442
H338	-7.875588612	-1.628249212	4.699891372
H339	-3.507111861	-5.517931979	-0.530509182
H340	-1.089376264	-3.592290766	-5.854258797
H341	-3.107507868	-2.921334516	-7.195672766
H342	-4.415490067	0.022254343	4.916807135
H343	-3.537047677	-2.962899373	10.346177189
H344	-5.550686257	-2.048926373	10.382624018
H345	2.131938892	2.322736027	5.644379667
H346	-7.313238152	-7.576914084	5.016842121

	H347	-4.492223340	-5.242610281	6.716105483
	H348	-4.950707449	-6.134742009	-1.387487662
	H349	2.787819486	-8.519871372	-1.496304522
	H350	3.915021862	-0.478948088	7.754105541
	H351	3.753331462	-2.033607400	6.900484731
	H352	-5.010603225	-9.328755606	7.471179691
	H353	-6.234675102	-8.300702416	7.825768573
	H354	-4.767316004	-5.663006110	11.341999795
	H355	-5.498992592	-5.954596742	9.201042495
	H356	-4.163819475	-8.316500341	11.114736497
	H357	-3.146674345	-5.440803681	10.651200195
	H358	4.361613434	-3.368058271	9.186251035
	H359	11.809511493	3.125439850	-6.721078562
	H360	-0.980161699	0.344016569	13.535699283
	H361	0.639289523	-0.694717918	11.538885375
	H362	9.745405673	2.147089899	-8.725273643
	H363	8.905616286	3.953522633	-6.370240692
	H364	8.864011611	4.400299975	-8.086240526
	H365	-3.399769523	0.039412789	1.986860945
	H366	3.244363378	1.271533474	-8.782086813
	H367	-1.981225248	0.497641803	1.275229724
	H368	0.826408624	-0.668880827	2.968519853
	H369	-2.127858086	0.513014769	2.907515504
	H370	-1.166170204	-0.516402558	-0.462715202
	Mo371	-3.455669471	-3.573595670	4.167606567
	N372	0.209219198	-0.842520503	2.158311635
	N373	-2.380363183	-0.010708884	2.066065044
	N374	-8.967849549	4.359972641	-2.554162300
	N375	-6.424571251	6.657687716	-4.044897142
	N376	-5.510392204	8.584908061	-2.216253129
	N377	-5.283953982	7.268680077	0.276670600
	N378	-3.078366180	5.635470498	-0.336641859
	N379	-1.038333094	7.697734785	-0.625119391
	N380	-1.048061194	9.248551902	1.837555873
	N381	-1.257044104	7.322622205	3.888957668
	N382	0.143017537	4.876043642	3.250933145
	N383	2.908596245	5.113360341	1.993603017
	N384	3.579141663	7.698758237	3.047611357
	N385	-6.478220005	3.048613578	11.866145936
	N386	-4.717501969	1.290076918	10.826298989
	N387	-0.240637185	0.316485614	6.566324995
	N388	1.358479156	2.020093591	6.239427485
	N389	1.677526235	-0.098788899	5.341353212
	N390	-1.947654724	-0.530264060	11.926487709
	N391	-5.584110491	0.977383803	-4.434413026
	N392	-6.670044318	-0.927315350	-0.223902406
	N393	-3.192165523	1.472923754	-5.398484837
	N394	-2.627939371	3.507826885	-7.234438952
	N395	-1.775795546	5.617756898	-5.576058287
	N396	0.654371485	4.698551732	-4.481974248
	N397	0.775055353	0.482463088	-3.977862979
	N398	-0.651624868	0.877138055	-2.317711905
	N399	2.932541104	5.164651771	-5.971060358
	N400	4.002361005	5.501597269	-10.585132822
	N401	6.055958144	6.328286880	-10.742585385
	N402	6.229453399	-2.959513711	7.433410988
	N403	8.321088360	-4.336852057	1.690560600
	N404	7.881833463	-5.586173928	-1.596856448
	N405	6.285963144	-4.531791626	-3.618438134
	N406	3.049711222	-2.860468116	-7.192609770
	N407	4.151866181	-4.167531406	-8.801204820
	N408	4.413293970	-1.867749701	-8.783028897
	N409	5.416654168	-1.926630268	-2.745501115
	N410	7.885859785	-0.657922316	-2.540821413
	N411	9.408733499	-0.547695817	-4.830069885
	N412	7.330824284	-2.784256099	-7.299342331
	N413	9.851947853	1.451937712	-6.741059424
	N414	-6.147276624	-10.152053211	1.157677618
	N415	-4.050815202	-7.497302539	-0.112208610
	N416	-1.785727248	-6.905986175	-2.024272881
	N417	0.711207912	-8.137895645	-1.318005250
	N418	-6.570210081	-7.189870592	-4.095399759
	N419	-4.968485002	-5.255712216	-5.490806477
	N420	-5.494270820	-8.487840367	7.142044865
	N421	-5.088596940	-5.266879984	4.172921419
	N422	-6.745358756	-6.416582384	3.280393354
	N423	-4.598287542	-6.392120474	9.410825537
	N424	2.090761353	-0.411861645	-1.174456196
	N425	1.623575737	-1.032776497	-0.349295192
	O426	-6.342547380	5.478255940	-2.095301570

O427	-3.372031530	7.854668837	-2.497113714
O428	-3.959027045	8.976814575	1.011081877
O429	-2.486687790	5.747818885	1.860013020
O430	0.400033147	6.087606603	0.073850902
O431	1.227501145	9.139382285	1.675381928
O432	-0.349610387	8.823159853	5.375824796
O433	0.018324621	4.316421220	5.472789761
O434	3.278101531	3.738922720	3.792824058
O435	5.544845283	6.744303953	3.661189709
O436	4.800428013	9.169350307	6.076696845
O437	-6.097795203	-0.524150424	11.213819171
O438	-2.207477836	1.745842967	12.112144305
O439	1.231469946	-1.464060912	13.333345488
O440	-2.840960105	3.303103975	-4.048480183
O441	-6.618229913	-0.802893423	-2.494286055
O442	-0.395419698	3.152018980	-6.928260075
O443	-0.602638224	0.182167830	-7.687035309
O444	-1.316783547	6.896131509	-7.428780379
O445	0.900900211	6.633817632	-3.292438255
O446	4.228477697	4.910051692	-4.114305441
O447	5.820357892	7.183163790	-6.304270009
O448	4.765723507	-1.770356264	10.392672739
O449	6.591453357	1.061630207	2.396737977
O450	8.408568548	-3.417668863	-1.130376596
O451	7.988861107	-4.680242315	-5.151047807
O452	5.396033904	-1.396600701	-4.971550187
O453	6.861460627	1.358570455	-2.929219904
O454	6.364634257	-0.520593507	-0.057437810
O455	11.275039134	0.078355550	-3.654384161
O456	11.289159620	0.119484512	-7.905111556
O457	8.084690273	-0.796368494	-8.139388792
O458	11.661024076	3.928144737	-8.595743477
O459	3.675995773	0.977588837	-7.956584593
O460	-6.334521593	-7.737745260	-0.177443674
O461	-3.619914397	-6.947719174	-3.395759201
O462	1.056349539	-8.656922523	-3.506664232
O463	2.738650488	-11.097905136	-0.936104253
O464	-6.947936812	-4.499821885	-6.332607062
O465	-8.381450393	-3.499092686	-0.522830275
O466	-7.229145228	-5.261859282	0.217969247
O467	-2.116244148	-5.265959302	-7.653844519
O468	-2.813800225	-7.651149612	8.807732268
O469	-2.715194827	-7.459235666	12.268724532
O470	-5.778414786	-0.581165587	2.527908444
O471	-6.373496128	1.457256621	3.307788662
O472	-9.196419228	-4.060886359	7.698629413
O473	-9.964959866	-2.995185681	5.858107656
O474	-4.033888155	-3.020768987	6.217124192
O475	-5.066029949	-1.495123802	7.509030575
O476	-5.383704989	-2.442370788	4.076995701
O477	-2.558764299	-2.928090968	10.535553230
O478	-1.711930501	-2.861907414	7.827281346
O479	1.852644285	-0.328718690	-6.583322016
O480	-7.559467296	-4.750562475	9.789505952
O481	-5.237516455	-2.825250919	9.853894320
S482	4.829862762	-4.321158907	-0.444429291
S483	1.189838394	-4.032809518	-0.620261089
S484	-3.412822217	-3.331441561	1.835081451
S485	2.866444530	-2.196089321	2.067963436
S486	-1.258019947	-1.825563597	-0.072528541
S487	-1.124171586	-6.528662612	1.132151881
S488	-1.998407397	-1.650250191	4.416737230
S489	2.352419172	-5.667149421	2.332308308
S490	-1.857136430	-5.269643569	4.523978296
S491	1.163331741	-3.344631985	5.286583987
end			

47, S=1/2

reactant

Fe(139)	2.488	bm612n2xnewbrk2bh147tf_4_53416.63			
Fe(140)	-0.036	C1	-6.988494896	5.849391133	-3.110391823
Fe(141)	2.526	C2	-8.447209594	5.457296545	-3.372388685
Fe(142)	-2.365	C3	-5.008625062	6.985202897	-4.023569004
Fe(143)	1.700	C4	-4.561894099	7.841843812	-2.844698751
		C5	-5.175392631	9.353662197	-1.029383531
		C6	-4.746383602	8.506755315	0.173640321

Fe(144) -2.334<br>Fe(145) -0.152	C7	-4.787674689	6.329299790	1.277043850
	C8	-3.356675273	5.866520395	0.973165667
	C9	-1.750903717	5.335161729	-0.816264507
	C10	-0.710281543	6.388359678	-0.407375248
	C11	-0.053800062	8.708018937	-0.330531257
	C12	0.104490566	9.027447575	1.161195599
	C13	-1.028329867	9.669374500	3.232914702
	C14	-0.842460244	8.558794863	4.271616617
	C15	-1.258792761	6.194773231	4.796492154
	C16	-0.275762259	5.063409993	4.507922553
	C17	1.043920235	3.781775211	2.836929653
	C18	2.524587553	4.205078113	2.914162543
	C19	0.681322596	3.259575569	1.432892037
	C20	1.624338364	2.140866345	0.982276711
	C21	-0.791350148	2.857408050	1.424483927
	C22	4.280208823	5.631220626	1.901029168
	C23	4.525181772	6.724736861	2.948903781
	C24	4.611484676	6.118851976	0.466231110
	C25	5.986038507	6.799099014	0.407756485
	C26	4.541954042	4.961056134	-0.535441024
	C27	3.828969674	8.846322665	3.888130803
	C28	4.167384669	8.460853371	5.312883508
	C29	-6.976004155	1.690842496	11.649394317
	C30	-5.881329696	0.708541092	11.218898788
	C31	-3.512794788	0.595270596	10.420388610
	C32	-2.492025759	0.661174295	11.573520948
	C33	-2.963561751	1.226477732	9.124900531
	C34	-1.691602911	0.558543384	8.585463285
	C35	-1.345764493	1.078895165	7.184089775
	C36	0.899696431	0.777006545	6.047937553
	C37	-1.017479734	-0.638381014	13.034695059
	C38	0.392426004	-0.967710768	12.594767227
	C39	-4.762475080	1.851371836	-3.595914040
	C40	-3.497596039	2.289939554	-4.359108867
	C41	-4.331198248	1.123613108	-2.300928785
	C42	-5.437018198	0.923592809	-1.242862846
	C43	-6.281422833	-0.343830600	-1.380852392
	C44	-1.905133951	1.468432645	-6.077373938
	C45	-1.575391840	2.784091507	-6.791710040
	C46	-1.881635703	0.266461591	-7.034285014
	C47	-2.447889358	4.829567408	-7.814844497
	C48	-1.787924645	5.875271301	-6.907679325
	C49	-1.061219562	6.483601762	-4.650340879
	C50	0.248381004	5.938326186	-4.079049522
	C51	1.912742419	4.144386311	-4.002557627
	C52	3.131263403	4.801557351	-4.678979336
	C53	2.013914520	2.635521415	-4.276876654
	C54	0.986901521	1.789076747	-3.600204918
	C55	0.098371909	1.995158456	-2.564655756
	C56	-0.196881828	-0.044120917	-3.185005412
	C57	4.015373059	5.628005085	-6.820144704
	C58	4.638396501	6.873991493	-6.196625739
	C59	3.413699844	5.948112602	-8.207498386
	C60	4.375989320	6.017351165	-9.354899134
	C61	5.653933970	6.534899723	-9.438365237
	C62	5.027888890	5.705855419	-11.395796436
	C63	5.541064445	-1.846770878	8.086885125
	C64	4.836867470	-2.369038116	9.324406528
	C65	4.453607398	-1.237405603	7.163403343
	C66	5.032553271	-0.611497012	5.916619141
	C67	5.449192379	0.732458133	5.900708794
	C68	5.178951645	-1.360121802	4.734501836
	C69	5.975824292	1.316310709	4.743682729
	C70	5.716185334	-0.795381890	3.577333780
	C71	6.103231075	0.546684824	3.580435125
	C72	7.319880699	-4.702199919	0.643970878
	C73	7.899905925	-4.502629817	-0.769173269
	C74	6.085852064	-3.833307211	0.822720866
	C75	8.393490676	-5.553991278	-2.950093760
	C76	7.522080656	-4.868216610	-4.005748684
	C77	5.335480728	-3.758354266	-4.423022642
	C78	5.382665225	-2.249616154	-4.064739737
	C79	3.932136813	-4.365959277	-4.194421470
	C80	2.720192205	-3.696454952	-4.852233924
	C81	2.524180752	-3.948121867	-6.357510027
	C82	3.843544366	-2.973259727	-8.247783415
	C83	5.450964922	-0.516827945	-2.338563342
	C84	6.794484933	0.153719075	-2.653133583
	C85	5.173205383	-0.313017506	-0.832898036
	C86	9.215044212	-0.098187946	-2.424783755

	C87	10.058636229	-0.183289421	-3.686060812
	C88	10.160923245	-0.901119773	-6.007138697
	C89	10.466196974	0.268876170	-6.975595419
	C90	9.597769118	-2.097441682	-6.781794243
	C91	8.264931147	-1.812903901	-7.459514778
	C92	9.886140047	2.541063247	-7.702085661
	C93	11.232945573	3.245526761	-7.665695163
	C94	8.750778553	3.558449915	-7.386029069
	C95	7.372348218	2.947826911	-7.515216565
	C96	6.702796691	2.956413127	-8.749575098
	C97	6.742265057	2.314065647	-6.430594073
	C98	5.460515873	2.337460404	-8.906665293
	C99	5.512585731	1.665277209	-6.574834174
	C100	4.884248268	1.680469914	-7.818172240
	C101	-5.083827377	-9.163897322	1.311754093
	C102	-5.208043404	-8.076457638	0.259530055
	C103	-3.945162401	-6.380072877	-1.043083473
	C104	-3.104807566	-6.754757453	-2.270746696
	C105	-0.830143048	-7.248574226	-3.057705479
	C106	0.379119057	-8.075923335	-2.632442266
	C107	1.915008550	-8.812872391	-0.891252939
	C108	1.780189081	-10.323326401	-0.939803958
	C109	-7.064448565	-5.821882707	-4.315612473
	C110	-6.333811216	-5.140794073	-5.478410266
	C111	-7.081170862	-4.892203292	-3.081096268
	C112	-8.123196373	-5.360146010	-2.047696007
	C113	-7.946797580	-4.626856361	-0.736616856
	C114	-4.206118912	-4.662538350	-6.578380882
	C115	-3.107195302	-5.605703467	-7.026541682
	C116	-3.745975018	-3.201707751	-6.342633611
	C117	-3.031160223	-3.117694423	-5.028273287
	C118	-1.675743910	-3.468938368	-4.932013821
	C119	-3.773036881	-2.914002036	-3.850537766
	C120	-1.108711548	-3.726788143	-3.681775387
	C121	-3.204458296	-3.176171633	-2.605007944
	C122	-1.888560132	-3.643244845	-2.529082689
	C123	-4.535302846	-7.362241100	7.126039713
	C124	-3.903340158	-7.131517728	8.505985361
	C125	-5.189852264	-6.083605655	6.582656710
	C126	-5.618695887	-6.121000773	5.150384021
	C127	-6.650760980	-6.845297993	4.591194170
	C128	-5.809515324	-5.477464810	3.065673079
	C129	-4.003217144	-6.135387580	10.711321589
	C130	-3.575944258	-7.411588041	11.401796016
	C131	-5.980292996	0.284327320	3.476673790
	C132	-5.511611224	-0.162669039	4.875762380
	C133	-5.776632624	-1.653681018	5.215283306
	C134	-7.265737762	-1.882328759	5.572022301
	C135	-7.598923657	-3.293400741	6.064148628
	C136	-9.046479228	-3.454464680	6.579041255
	C137	-4.914826239	-2.073777097	6.414570834
	C138	-0.216595292	-3.489960063	2.295668939
	Fe139	-1.729316781	-4.760941719	2.356000431
	Fe140	0.768625734	-2.033573620	0.795210885
	Fe141	1.557024346	-3.583902374	3.154138776
	Fe142	0.527809619	-4.964115746	1.261030812
	Fe143	2.945956457	-4.169423631	0.861948384
	Fe144	-0.716800026	-3.443284308	4.262972641
	Fe145	-1.653324293	-1.843060536	2.106672105
	H146	-8.832329374	4.570946327	-1.574891602
	H147	-8.450742551	3.509677572	-2.753337800
	H148	-6.941719497	6.844847481	-4.909532053
	H149	-8.563598832	5.211748876	-4.439952911
	H150	-9.055850821	6.361511322	-3.199755682
	H151	-4.402222846	6.067365920	-3.987785820
	H152	-6.460498827	8.548984435	-2.558188283
	H153	-4.751782726	7.516571448	-4.949306497
	H154	-6.053351299	9.949768639	-0.748715298
	H155	-5.881721966	6.906871219	-0.471939759
	H156	-4.345912705	10.044279210	-1.233666090
	H157	-1.401090562	4.371036418	-0.438695381
	H158	-3.841975595	5.721274678	-1.011858518
	H159	-5.457649681	5.458997101	1.308250722
	H160	0.927373026	8.379984645	-0.695821676
	H161	-1.897905093	7.909922871	-1.129531147
	H162	-4.779352605	6.795070741	2.269463783
	H163	-1.805007344	5.266792344	-1.910065521
	H164	-1.953248367	9.166736127	1.367887530
	H165	-0.344290983	9.625185546	-0.859776698
	H166	-1.454301759	3.724018049	1.519220952

H167	-1.049852763	2.321459050	0.497085227
H168	-1.005827215	2.228292883	2.291751697
H169	0.793990333	4.091346853	0.723060534
H170	-1.976508646	10.179439286	3.454613760
H171	3.554899887	4.477974809	-0.543864060
H172	2.782908635	7.744520186	2.406659676
H173	6.037702839	7.692681516	1.046650459
H174	1.351162220	1.816856921	-0.030127310
H175	1.584118523	1.258732471	1.637545543
H176	6.212120631	-1.027000682	8.407067949
H177	4.741019170	5.319670520	-1.554829898
H178	6.202771322	7.106859052	-0.625641509
H179	5.294082491	4.193334167	-0.292966772
H180	6.779740300	6.106957040	0.730233393
H181	3.850786940	6.870918748	0.184618503
H182	4.961133138	4.809904724	2.168879065
H183	2.218070315	5.487046741	1.329446326
H184	2.669678627	2.482660985	0.941560800
H185	4.380049508	-1.004109441	-0.513063043
H186	4.807322403	0.717594793	-0.714017868
H187	6.321011429	0.076664369	0.719284903
H188	6.781788016	2.016763072	2.510045750
H189	5.798631368	-1.380800158	2.662345320
H190	4.838403751	-2.397276610	4.705834561
H191	5.346744213	1.341875783	6.802222969
H192	-0.198753317	5.502233393	2.501602191
H193	-2.259152033	5.734713242	4.823830649
H194	-1.025032775	6.561053450	5.802897007
H195	-1.755001522	7.200930309	3.003190489
H196	-0.209598885	10.383736062	3.390484450
H197	6.266587434	2.370012630	4.741577746
H198	0.919945016	2.982586251	3.578786860
H199	4.636798764	9.486812851	3.497568338
H200	2.904795403	9.443550629	3.915170330
H201	3.723298888	7.501039197	5.664656454
H202	-1.351948405	-1.382858526	13.773801941
H203	-7.263459800	-1.756110674	-0.290706817
H204	-5.532270023	-1.776306374	3.251862616
H205	-9.132324720	-5.146144263	-2.427793536
H206	-8.039206158	-6.444672652	-1.886324975
H207	-7.316036100	-3.859458344	-3.382151601
H208	-3.289332687	-6.678247439	-6.778653828
H209	-8.100331275	-5.905495334	-4.678286262
H210	-3.883247782	0.154804330	-2.573134844
H211	-6.135968086	1.778348073	-1.261956928
H212	-4.487261391	-5.755726577	-4.751550980
H213	-4.897392914	-4.638954587	-7.446495271
H214	-5.654063877	-7.190411016	-3.644703385
H215	-6.359050981	-0.621244244	0.707972182
H216	-6.074167647	-4.868744485	-2.637413435
H217	-7.213211794	-7.691153262	-3.481255283
H218	-6.295321930	1.531111860	-4.943032442
H219	-3.525129200	1.713027415	-1.842242720
H220	-6.935434549	-6.178291438	-0.081832686
H221	-4.982073107	0.935747244	-0.241547599
H222	2.007275332	4.352790456	-2.928173675
H223	-0.060167319	2.907149688	-1.998464589
H224	-0.809144738	7.408708052	-5.187064416
H225	-2.193525791	4.755412364	-5.199281633
H226	1.992503411	5.092142109	-6.362051834
H227	6.302437161	6.997673515	-8.701943358
H228	3.904083619	7.514326924	-5.650268231
H229	2.818348945	6.875403499	-8.134607420
H230	4.791690739	4.851704772	-6.909638801
H231	5.082570324	5.426833006	-12.444762429
H232	3.026265966	2.325530660	-3.966465447
H233	1.958748185	2.475432215	-5.366748292
H234	6.952433911	6.582139084	-11.137363011
H235	2.700410120	5.143862585	-8.447969872
H236	0.102503862	4.169721154	-5.162815748
H237	-1.702705763	6.774572100	-3.804633097
H238	-1.818202944	4.772351664	-8.713685634
H239	-0.530137590	-1.076859870	-3.259836270
H240	-3.430043830	5.211796789	-8.122110708
H241	7.078320429	-3.194239013	7.936421432
H242	6.518937711	-2.676597409	6.490160967
H243	5.234645024	-2.640173359	-2.010629048
H244	4.685751554	0.032752450	-2.905267116
H245	7.746454986	-1.582044864	-2.114232278
H246	1.252560802	0.026305287	-4.783010905

H247	0.965847239	-0.123212706	-6.989933747
H248	-0.707454519	-0.420943854	-8.445203804
H249	2.475851757	0.350983657	-6.926084899
H250	-2.067576777	-0.644220033	-6.441213603
H251	-2.679927456	0.376018542	-7.785161712
H252	9.542046486	-2.972710528	-6.119560509
H253	10.321583130	-2.324973782	-7.579494319
H254	7.437659663	-3.475522615	-6.548732107
H255	6.375891498	-2.495363763	-7.556985994
H256	4.975678731	-4.248711503	-9.385279151
H257	1.452820439	-4.058876822	-6.582472752
H258	4.949309563	2.364363277	-9.872932978
H259	7.199091133	2.322677811	-5.437456724
H260	4.167554306	-0.941518743	-8.410138141
H261	3.749088435	-4.371053432	-3.107703938
H262	6.002009050	-4.734723549	-2.649549227
H263	2.707255865	-2.613543015	-4.651334363
H264	-3.565922876	3.176120939	-7.021638721
H265	8.573926804	-6.584792214	-3.283595078
H266	-3.899347402	0.727226769	-5.521648797
H267	3.973824754	-5.423990616	-4.502189000
H268	5.071975382	1.129475967	-5.733866627
H269	9.358237573	-5.026336358	-2.953924960
H270	3.774333785	-5.024317514	-8.412437961
H271	4.723917258	-1.895230707	-9.746746336
H272	-6.072269909	0.285916333	-3.849111428
H273	-5.266428129	2.791089883	-3.300715961
H274	7.154480321	3.456774546	-9.609046676
H275	-1.103169679	1.344337496	-5.334971916
H276	5.634063832	-3.836250651	-5.472355460
H277	3.004196388	-4.894808742	-6.638593644
H278	1.837329001	-4.104772094	-4.343154691
H279	2.665087286	-1.912601193	-6.973354119
H280	9.142948291	0.963637899	-2.144344133
H281	8.430808527	-0.863453054	-4.721140535
H282	11.159888943	-1.195871397	-5.649225149
H283	9.768260754	-0.620264339	-1.632472958
H284	9.103697012	1.442592035	-6.035767690
H285	-4.104184784	-9.657668804	1.228302336
H286	-5.096575086	-8.639695069	2.289278640
H287	-3.696671876	-7.646460954	6.472592880
H288	-6.223697386	-10.705894583	2.008320658
H289	-7.041824600	-9.655508948	1.054119778
H290	5.695493039	-3.985844385	1.836520451
H291	8.632395975	-3.388416394	1.488424588
H292	7.449672315	-6.452049754	-1.255783469
H293	6.352214863	-2.775375457	0.686230829
H294	9.125617175	-4.943423860	1.603392989
H295	2.119676371	-8.528211960	0.153054710
H296	7.047616357	-5.760016158	0.788058363
H297	-2.779985706	2.298286146	9.309477303
H298	-3.789125575	-0.448895974	10.227099617
H299	0.182302123	-7.609366151	-0.602149653
H300	2.633290783	0.185110455	5.157641806
H301	-1.356824538	-7.812249537	-3.837383488
H302	-3.782480051	-3.019393027	-1.691967268
H303	-0.434166446	-6.336925140	-3.538488521
H304	-1.091664342	2.145949337	7.211039679
H305	-1.459264563	-6.587662367	-1.087117535
H306	-2.224627861	0.972003037	6.526799689
H307	-3.767648596	1.150520622	8.375725354
H308	-1.838015625	-0.530728033	8.523945352
H309	-0.839551552	0.736381714	9.260748513
H310	-4.638521221	-2.559694695	-6.356472690
H311	-4.703252176	2.307981675	10.969859412
H312	-7.707813945	1.702167885	10.827295579
H313	-1.446273548	-3.901215752	-1.571375817
H314	-6.213288668	3.176725633	12.852727544
H315	-7.228670521	3.731845616	11.693264347
H316	-0.484014629	-0.652516099	6.339933794
H317	-0.059005198	-4.008595754	-3.586115057
H318	-4.810102261	-2.576577546	-3.915647668
H319	-6.885887241	-4.042536253	9.845938031
H320	-6.078893775	-5.848862735	7.193091726
H321	-5.145513435	-2.423508336	8.950905444
H322	-8.114010488	-4.485629373	8.988350744
H323	0.730077266	-10.701118182	-0.991354378
H324	-2.558817567	-2.929823257	7.298058919
H325	-5.970855239	0.479154106	5.637552089
H326	-3.191563353	-7.739173876	0.392798686

H327	-7.513275593	-1.155042841	6.361104552
H328	-7.459054722	-6.714828170	2.612254409
H329	-5.693248617	-4.953480850	2.125148274
H330	-7.480658883	1.234030181	12.517269125
H331	1.486959408	-1.092955247	5.365247349
H332	-2.162369277	-2.971635435	9.636588773
H333	-1.078827904	-3.402056314	7.337191737
H334	-2.216766839	-1.400701235	11.416538371
H335	0.658277171	2.780862770	6.400535406
H336	-7.457269043	-4.007745212	5.237757317
H337	-6.911574399	-3.589372292	6.868923234
H338	-7.881470236	-1.630423894	4.694655484
H339	-3.507992021	-5.511387857	-0.526627306
H340	-1.087594876	-3.592183247	-5.843813549
H341	-3.102055555	-2.920844508	-7.190217193
H342	-4.423588049	0.017982146	4.898597619
H343	-3.544916194	-2.959768741	10.349529140
H344	-5.562408996	-2.044207024	10.389271610
H345	2.102805056	2.357414711	5.656573592
H346	-7.316797659	-7.584555940	5.021158650
H347	-4.497258741	-5.243216438	6.715353920
H348	-4.951276624	-6.129542365	-1.383733546
H349	2.783756095	-8.513818731	-1.497498805
H350	3.907717549	-0.481459372	7.748321861
H351	3.742384211	-2.033755364	6.889748192
H352	-4.998404540	-9.330103619	7.473230766
H353	-6.226462089	-8.305488231	7.824749203
H354	-4.773516940	-5.666027669	11.344283202
H355	-5.495078265	-5.951591651	9.197980014
H356	-4.159688720	-8.318304770	11.113194883
H357	-3.151919588	-5.438514877	10.657661020
H358	4.355372405	-3.367496394	9.187846041
H359	11.801495874	3.126369904	-6.712655847
H360	-0.979051046	0.344736635	13.536144872
H361	0.632981852	-0.698077189	11.537234547
H362	9.740798774	2.142665037	-8.719086053
H363	8.900512022	3.956374707	-6.370024446
H364	8.863873714	4.397709021	-8.087578620
H365	-2.969966159	-1.146623877	2.023141351
H366	3.240619922	1.274346792	-8.782349847
H367	-1.297929766	0.504617601	1.247163984
H368	0.405254311	-0.686925919	3.133515554
H369	-1.433728401	0.473816063	2.958818568
H370	-1.334027909	-0.504646779	-0.533008276
Mo371	-3.462054258	-3.614802915	4.208417047
N372	0.030689849	-0.721605119	2.177793176
N373	-1.140132819	-0.009783427	2.110440616
N374	-8.975212235	4.368078995	-2.567412494
N375	-6.426744807	6.665398757	-4.051506123
N376	-5.503163190	8.582467954	-2.216902276
N377	-5.290207905	7.266848545	0.280109508
N378	-3.093678576	5.621606225	-0.331118976
N379	-1.045016853	7.682158510	-0.613138967
N380	-1.050422659	9.245495023	1.843744422
N381	-1.263036446	7.322307712	3.890260008
N382	0.149457326	4.883621746	3.233077861
N383	2.913520546	5.118759937	1.979560326
N384	3.583702532	7.699545512	3.037906279
N385	-6.485251570	3.055793498	11.874651399
N386	-4.726521349	1.292042218	10.834136460
N387	-0.250241893	0.320322968	6.562696597
N388	1.341188546	2.033877033	6.255771231
N389	1.657051097	-0.067647809	5.308181918
N390	-1.953000268	-0.530905404	11.931735995
N391	-5.589369793	0.984367797	-4.444960872
N392	-6.674222006	-0.920694488	-0.230600208
N393	-3.196733472	1.476341495	-5.406694602
N394	-2.629035363	3.512343756	-7.232745815
N395	-1.777845988	5.620534516	-5.570683381
N396	0.651244399	4.699477337	-4.480703254
N397	0.780120953	0.477733324	-3.985972835
N398	-0.632743518	0.854018714	-2.311050306
N399	2.928288490	5.166564699	-5.970810285
N400	3.996318198	5.503536980	-10.584781119
N401	6.051923015	6.325263355	-10.742085327
N402	6.221886255	-2.963261689	7.426902292
N403	8.301951983	-4.339833448	1.681291828
N404	7.854735278	-5.585807818	-1.601322356
N405	6.274938620	-4.533934490	-3.618356410
N406	3.046174381	-2.857948148	-7.188384684

N407	4.156376472	-4.165363377	-8.791334911
N408	4.406451960	-1.864943087	-8.781899404
N409	5.412120889	-1.924492431	-2.742175228
N410	7.883499814	-0.659405143	-2.535198794
N411	9.397275548	-0.553020751	-4.825370109
N412	7.318258504	-2.777882737	-7.294652142
N413	9.843146054	1.449332334	-6.734166133
N414	-6.152460438	-10.154902694	1.150140551
N415	-4.049889739	-7.491707011	-0.107395357
N416	-1.786205336	-6.896159369	-2.022674521
N417	0.709660581	-8.123947774	-1.316859959
N418	-6.573966954	-7.190205948	-4.102169217
N419	-4.975949259	-5.256924388	-5.502056573
N420	-5.484320778	-8.491205857	7.142520476
N421	-5.095334022	-5.275604774	4.172640302
N422	-6.751913428	-6.425607952	3.282677368
N423	-4.597337741	-6.392176083	9.412176963
N424	2.111722197	-0.395932991	-1.236044468
N425	1.586224814	-0.983353236	-0.429712670
O426	-6.361086841	5.494164633	-2.098332936
O427	-3.370109480	7.833764983	-2.488279766
O428	-3.947963802	8.963601625	1.008250301
O429	-2.495889883	5.741954034	1.863623148
O430	0.385632583	6.064827102	0.079480776
O431	1.224931730	9.119726135	1.688966910
O432	-0.369032485	8.822720087	5.385306980
O433	0.044295776	4.321154090	5.451974174
O434	3.293430785	3.711868396	3.752450116
O435	5.539776878	6.733922162	3.664870355
O436	4.817958652	9.178693220	6.058122938
O437	-6.111464915	-0.518025396	11.224086030
O438	-2.215437086	1.744762102	12.119460334
O439	1.227668768	-1.471840036	13.329006692
O440	-2.825670948	3.290687218	-4.042811594
O441	-6.615817248	-0.799107542	-2.501103643
O442	-0.396461486	3.156812368	-6.926783904
O443	-0.602621522	0.189386972	-7.693597317
O444	-1.313807936	6.898848195	-7.422083248
O445	0.896233862	6.629183668	-3.281208739
O446	4.226564277	4.904335570	-4.116679517
O447	5.810724160	7.193041701	-6.306633616
O448	4.770393105	-1.766808543	10.386468871
O449	6.594155367	1.067036113	2.397326802
O450	8.386097143	-3.418745871	-1.134806604
O451	7.981881022	-4.669373740	-5.147971887
O452	5.397701559	-1.398345610	-4.969107287
O453	6.863379213	1.357486250	-2.931357114
O454	6.358753966	-0.519703119	-0.055520562
O455	11.267869379	0.078457666	-3.659234669
O456	11.284032103	0.118134613	-7.894588366
O457	8.079862366	-0.796709568	-8.144079531
O458	11.657026139	3.920736899	-8.590711013
O459	3.673391755	0.978105025	-7.958290667
O460	-6.332477507	-7.739495949	-0.186220330
O461	-3.621547297	-6.935741319	-3.393630890
O462	1.056282658	-8.644930862	-3.505685880
O463	2.733153551	-11.084587199	-0.900517702
O464	-6.956074650	-4.500997244	-6.343123311
O465	-8.377994191	-3.498969769	-0.528034653
O466	-7.227831315	-5.262772775	0.213654918
O467	-2.116177860	-5.262333288	-7.654438952
O468	-2.810916908	-7.649753703	8.811491888
O469	-2.717265880	-7.457689693	12.272578382
O470	-5.883837594	-0.617906089	2.540080402
O471	-6.354870302	1.459981488	3.301691986
O472	-9.205359300	-4.060957771	7.697149485
O473	-9.976469120	-3.003578678	5.853015449
O474	-4.057859379	-3.043317087	6.231964972
O475	-5.075295457	-1.497389420	7.508224144
O476	-5.384064654	-2.454958579	4.078050672
O477	-2.566603604	-2.925845077	10.538686348
O478	-1.725691452	-2.862521522	7.829370266
O479	1.849018453	-0.325181514	-6.583515625
O480	-7.573391025	-4.743371088	9.795196895
O481	-5.248598837	-2.819724412	9.860179784
S482	4.758337584	-4.224170454	-0.395725060
S483	1.263949817	-3.912791429	-0.553886731
S484	-3.402547635	-3.364234024	1.843931345
S485	2.831646402	-2.216082621	1.946746939
S486	-1.322657214	-1.818233864	-0.139310982

	S487 -1.075673285 -6.491912494 1.119599239<br>S488 -2.050605803 -1.652891259 4.419614201<br>S489 2.355746113 -5.581411613 2.431906362<br>S490 -1.884663770 -5.329253161 4.558178829<br>S491 1.207478802 -3.323046400 5.319628159<br>end

TS

Fe(139) 2.426<br>Fe(140) -0.030<br>Fe(141) 2.475<br>Fe(142) -2.458<br>Fe(143) 1.787<br>Fe(144) -2.280<br>Fe(145) -0.082	bm612n2xnewbrk2bh147tf_1_53382.93<br>C1 -6.985096531 5.842496717 -3.109751173<br>C2 -8.445375509 5.456739463 -3.374555881<br>C3 -5.003330283 6.977169114 -4.020047356<br>C4 -4.556254334 7.832459544 -2.840389951<br>C5 -5.169138876 9.349951396 -1.029270271<br>C6 -4.742630832 8.502109408 0.173706466<br>C7 -4.795671605 6.327450595 1.280800679<br>C8 -3.365231222 5.860681658 0.977635067<br>C9 -1.759143927 5.323941241 -0.809465725<br>C10 -0.718348251 6.376313114 -0.402269915<br>C11 -0.051846123 8.690515699 -0.320422649<br>C12 0.104453153 9.015990959 1.170135338<br>C13 -1.030733403 9.668907328 3.237658362<br>C14 -0.852236070 8.558336820 4.277433479<br>C15 -1.250166184 6.189642050 4.791656879<br>C16 -0.264700374 5.063561303 4.491930051<br>C17 1.047647480 3.774993183 2.825561984<br>C18 2.529720788 4.198257351 2.895474467<br>C19 0.688501583 3.254086569 1.418655305<br>C20 1.632567966 2.141652990 0.959345329<br>C21 -0.780699625 2.842348150 1.403314036<br>C22 4.281951268 5.633051556 1.891251058<br>C23 4.525328636 6.720453387 2.943490960<br>C24 4.617533789 6.126279935 0.459322245<br>C25 5.993211002 6.805640341 0.406403856<br>C26 4.547746277 4.968842997 -0.542135927<br>C27 3.833877430 8.839232299 3.878402570<br>C28 4.175800389 8.464195826 5.304478180<br>C29 -6.980298391 1.692548471 11.645383219<br>C30 -5.885914808 0.708543786 11.221205206<br>C31 -3.516572562 0.594101351 10.426873197<br>C32 -2.495223456 0.660587230 11.579030387<br>C33 -2.967078146 1.223058313 9.130300824<br>C34 -1.696080623 0.551661528 8.591085465<br>C35 -1.356820577 1.065899798 7.185005648<br>C36 0.895307623 0.786583897 6.051426409<br>C37 -1.018340350 -0.638921554 13.036964666<br>C38 0.389813719 -0.970909941 12.594681472<br>C39 -4.756490845 1.847240391 -3.593481434<br>C40 -3.488985868 2.282624471 -4.354628281<br>C41 -4.330062019 1.122951975 -2.296402915<br>C42 -5.438750257 0.924581511 -1.241108864<br>C43 -6.282964243 -0.343089902 -1.380799545<br>C44 -1.905212939 1.468814506 -6.080500054<br>C45 -1.575782547 2.786536157 -6.791717474<br>C46 -1.879604913 0.266748855 -7.037052572<br>C47 -2.449881781 4.834079181 -7.811312726<br>C48 -1.786512987 5.878003113 -6.904629363<br>C49 -1.062261090 6.485437230 -4.645362148<br>C50 0.246759594 5.938714357 -4.074637829<br>C51 1.908541207 4.142869494 -4.000387794<br>C52 3.127777281 4.797833826 -4.676775074<br>C53 2.008804168 2.634486635 -4.275897600<br>C54 0.984719640 1.782746759 -3.601695506<br>C55 0.105672439 1.972108382 -2.554805318<br>C56 -0.176310045 -0.065348524 -3.187409256<br>C57 4.010648202 5.623825302 -6.815131441<br>C58 4.631753475 6.873711083 -6.195673611<br>C59 3.411176675 5.947770200 -8.204349433<br>C60 4.373597689 6.016433155 -9.352309257<br>C61 5.651858748 6.533150439 -9.436805373<br>C62 5.023976154 5.705065206 -11.394053371<br>C63 5.543080984 -1.846811478 8.087021616<br>C64 4.838525194 -2.366983927 9.325426607<br>C65 4.456816158 -1.237583199 7.163157604<br>C66 5.036301997 -0.610993643 5.916968236<br>C67 5.453091081 0.732948859 5.901760573<br>C68 5.182583733 -1.359044651 4.734442503<br>C69 5.979528714 1.317317497 4.744898033<br>C70 5.719677655 -0.793844882 3.577453539<br>C71 6.106886225 0.548270277 3.581290646

	C72	7.319721862	-4.701248198	0.641044919
	C73	7.901922078	-4.501818873	-0.770733617
	C74	6.076896707	-3.845413057	0.813218087
	C75	8.394068781	-5.554128986	-2.951254216
	C76	7.520898295	-4.866755709	-4.004137966
	C77	5.333234176	-3.760347640	-4.421014604
	C78	5.381351239	-2.252509786	-4.062273471
	C79	3.929255467	-4.365977206	-4.192897232
	C80	2.718458021	-3.694384205	-4.850717971
	C81	2.522111042	-3.945621614	-6.355827182
	C82	3.842142029	-2.970205537	-8.243706805
	C83	5.451245685	-0.518780396	-2.338628168
	C84	6.795081174	0.151056372	-2.653788802
	C85	5.172333001	-0.311104183	-0.835400503
	C86	9.216035017	-0.101350726	-2.425285322
	C87	10.057107809	-0.184830087	-3.687908656
	C88	10.156617521	-0.903367948	-6.006902939
	C89	10.464130466	0.267879941	-6.973784691
	C90	9.595123269	-2.098054023	-6.784817724
	C91	8.261933917	-1.811798067	-7.460670424
	C92	9.882079347	2.539003434	-7.698974898
	C93	11.229464061	3.244254718	-7.662293784
	C94	8.748545470	3.558405421	-7.386373959
	C95	7.370169573	2.948508706	-7.516302243
	C96	6.699590532	2.957677090	-8.750067713
	C97	6.741873266	2.313025790	-6.431881592
	C98	5.457322913	2.338070653	-8.906326923
	C99	5.512883755	1.662942838	-6.575413131
	C100	4.882627744	1.679549853	-7.817680004
	C101	-5.087500936	-9.166585263	1.307954068
	C102	-5.209668569	-8.076564674	0.256582591
	C103	-3.947023713	-6.376135714	-1.042776238
	C104	-3.103486482	-6.747211552	-2.269692773
	C105	-0.829327218	-7.240991091	-3.056290386
	C106	0.380074839	-8.069867299	-2.632833438
	C107	1.917546414	-8.814148814	-0.895488189
	C108	1.778433937	-10.323189537	-0.931555380
	C109	-7.063007291	-5.821853239	-4.315473565
	C110	-6.331423751	-5.140721142	-5.477596990
	C111	-7.080624221	-4.890637561	-3.081403582
	C112	-8.121617493	-5.358919165	-2.047181288
	C113	-7.945375655	-4.625902726	-0.735861988
	C114	-4.203912187	-4.659977472	-6.576503502
	C115	-3.105219092	-5.602681347	-7.025890949
	C116	-3.742400526	-3.200260851	-6.337429509
	C117	-3.031679145	-3.115334379	-5.020814181
	C118	-1.676112631	-3.464476512	-4.919410225
	C119	-3.776694970	-2.905910847	-3.846066856
	C120	-1.110854436	-3.712739680	-3.666845961
	C121	-3.210291835	-3.158003461	-2.597863724
	C122	-1.893994013	-3.623126156	-2.517241075
	C123	-4.530969748	-7.361478552	7.127108948
	C124	-3.900186906	-7.130999174	8.507964939
	C125	-5.189432766	-6.084439745	6.584394459
	C126	-5.620714192	-6.123198964	5.152739586
	C127	-6.651487413	-6.849490906	4.593691958
	C128	-5.814071719	-5.479783948	3.067886946
	C129	-4.006557041	-6.136866270	10.715741217
	C130	-3.576359810	-7.412746113	11.404124466
	C131	-5.985798603	0.288034785	3.473936579
	C132	-5.520422147	-0.161251213	4.874616971
	C133	-5.786436657	-1.652133313	5.215741106
	C134	-7.275866115	-1.880575013	5.571628809
	C135	-7.606457296	-3.292864774	6.062154419
	C136	-9.053457164	-3.457325732	6.576635355
	C137	-4.922676699	-2.071837753	6.414609476
	C138	-0.216523887	-3.497244665	2.299801145
	Fe139	-1.743295091	-4.784483618	2.356462911
	Fe140	0.794308297	-1.960936228	0.739319964
	Fe141	1.559310614	-3.583754387	3.142935092
	Fe142	0.526469279	-4.972119549	1.244092799
	Fe143	2.967283252	-4.151811374	0.930647457
	Fe144	-0.703191044	-3.484442882	4.272329896
	Fe145	-1.510659402	-2.004451702	2.086723653
	H146	-8.838084145	4.569423871	-1.579429956
	H147	-8.456498944	3.508629295	-2.758479399
	H148	-6.936318789	6.842852437	-4.906889582
	H149	-8.560699330	5.213579421	-4.442820541
	H150	-9.051375039	6.362929736	-3.202051678
	H151	-4.399233106	6.057893869	-3.984167842

H152	-6.452347349	8.549082170	-2.561659159
H153	-4.744416055	7.508388872	-4.945277789
H154	-6.047485873	9.945980407	-0.749709131
H155	-5.886615551	6.908074615	-0.469475548
H156	-4.339265391	10.040492636	-1.232369612
H157	-1.410337615	4.360406189	-0.429661798
H158	-3.854121146	5.696126890	-1.005754078
H159	-5.469095264	5.459842396	1.312372195
H160	0.927776915	8.351963241	-0.679964903
H161	-1.903434642	7.903506032	-1.115577292
H162	-4.786789678	6.794462681	2.272695914
H163	-1.812984587	5.251192252	-1.903086820
H164	-1.952919997	9.159219379	1.373024113
H165	-0.332618809	9.607513777	-0.854770831
H166	-1.449250780	3.703731037	1.516681313
H167	-1.038362198	2.326621247	0.463157114
H168	-0.989985946	2.195169803	2.260044443
H169	0.796603287	4.090372550	0.713408041
H170	-1.976017342	10.185942948	3.455297874
H171	3.557398153	4.492792475	-0.553668379
H172	2.790319577	7.749534655	2.391897537
H173	6.043403256	7.698760896	1.046280010
H174	1.349116839	1.817316861	-0.050002739
H175	1.598028766	1.258609515	1.613597859
H176	6.214718076	-1.027201049	8.407092363
H177	4.754740544	5.322836545	-1.561366278
H178	6.213769209	7.115019379	-0.625765251
H179	5.292453287	4.196330020	-0.291950338
H180	6.786014678	6.113260957	0.730476070
H181	3.859296306	6.880579943	0.176881849
H182	4.960250730	4.808998773	2.156351601
H183	2.215761842	5.503289155	1.328061327
H184	2.674809723	2.490696968	0.905519409
H185	4.378651128	-1.000845436	-0.514093670
H186	4.806063053	0.719702786	-0.717638711
H187	6.317376656	0.081246338	0.718989083
H188	6.786177408	2.018703968	2.512309379
H189	5.802304819	-1.378588376	2.662002965
H190	4.841935500	-2.396157526	4.705830714
H191	5.350913986	1.341916389	6.803632417
H192	-0.191159491	5.500159919	2.481606326
H193	-2.247651701	5.723682832	4.827442051
H194	-1.007946349	6.551844022	5.797392207
H195	-1.761322806	7.200321147	3.005606336
H196	-0.207829463	10.378218008	3.396290297
H197	6.269686695	2.371190846	4.742938854
H198	0.921669532	2.979494720	3.569684780
H199	4.644226211	9.476837097	3.485111235
H200	2.914063673	9.443708804	3.905203396
H201	3.725816247	7.511778371	5.668024097
H202	-1.352347564	-1.381090931	13.778787527
H203	-7.265667811	-1.756903689	-0.292880329
H204	-5.542696056	-1.785886306	3.255998185
H205	-9.131086883	-5.145388622	-2.426640730
H206	-8.036837166	-6.443280117	-1.885550480
H207	-7.316955774	-3.858495049	-3.383367768
H208	-3.289290579	-6.675890906	-6.782570374
H209	-8.098408962	-5.906496956	-4.678441740
H210	-3.880403390	0.154022883	-2.565175092
H211	-6.137125975	1.779728683	-1.261843714
H212	-4.483962924	-5.752503828	-4.748805467
H213	-4.895753935	-4.634408828	-7.443853759
H214	-5.650845466	-7.187489962	-3.642387694
H215	-6.368143252	-0.618516388	0.708178090
H216	-6.073558466	-4.865252194	-2.637889604
H217	-7.209296424	-7.690022237	-3.478024860
H218	-6.288295735	1.529665676	-4.943884520
H219	-3.526170609	1.715425858	-1.837687987
H220	-6.935270807	-6.177822337	-0.080428093
H221	-4.986458685	0.937797369	-0.238438017
H222	2.002961316	4.351422549	-2.926115655
H223	-0.053849883	2.875467772	-1.975344229
H224	-0.810993623	7.412297427	-5.179459191
H225	-2.191228806	4.756929416	-5.197629613
H226	1.988575871	5.090048862	-6.360285797
H227	6.301099900	6.995596700	-8.700898726
H228	3.896734885	7.513832815	-5.649757740
H229	2.818637923	6.877001770	-8.131198517
H230	4.789192290	4.850215611	-6.905875279
H231	5.078098669	5.426001444	-12.443089301

H232	3.020891502	2.324467778	-3.964482412
H233	1.955493280	2.475467858	-5.366016743
H234	6.949845399	6.578600403	-11.136379253
H235	2.695373122	5.146505086	-8.446923208
H236	0.102863525	4.175373341	-5.167182218
H237	-1.704098341	6.773236481	-3.798864587
H238	-1.824784156	4.778749269	-8.713408454
H239	-0.494116509	-1.103103116	-3.262691137
H240	-3.433373197	5.217489535	-8.112802074
H241	7.081648074	-3.192534598	7.939318541
H242	6.521241787	-2.679370941	6.492053704
H243	5.225824559	-2.642472615	-2.011044179
H244	4.686925291	0.029465889	-2.907525638
H245	7.747063611	-1.584536836	-2.114317675
H246	1.257153006	0.031670780	-4.799736039
H247	0.967394586	-0.122802637	-6.991222222
H248	-0.706501004	-0.412366163	-8.452942551
H249	2.477571152	0.349074974	-6.923156327
H250	-2.064296289	-0.643804419	-6.443381561
H251	-2.677901769	0.373957465	-7.788264229
H252	9.540300853	-2.974868792	-6.124683751
H253	10.319359112	-2.322329503	-7.583070513
H254	7.434074405	-3.469482695	-6.542800123
H255	6.369870861	-2.487707843	-7.546514865
H256	4.978735328	-4.244341028	-9.378465865
H257	1.450965140	-4.057681900	-6.581093601
H258	4.945327379	2.366169877	-9.872144399
H259	7.200030919	2.321362618	-5.439385440
H260	4.161699727	-0.937234227	-8.414762361
H261	3.746101727	-4.370098954	-3.106205669
H262	5.997882481	-4.741430210	-2.649261948
H263	2.707155679	-2.611455200	-4.649749049
H264	-3.566688285	3.176371422	-7.025023528
H265	8.572688776	-6.585026475	-3.285447519
H266	-3.904542342	0.732482682	-5.529890696
H267	3.969603820	-5.423934265	-4.500906663
H268	5.074623222	1.125426465	-5.734370072
H269	9.359539385	-5.027703166	-2.957249693
H270	3.780628628	-5.022300015	-8.403464167
H271	4.727025990	-1.897001274	-9.744199986
H272	-6.067541414	0.283012914	-3.851039661
H273	-5.260497145	2.788625158	-3.302373133
H274	7.150184736	3.459035573	-9.609582395
H275	-1.104488763	1.345000877	-5.336677807
H276	5.632586638	-3.838100016	-5.470103485
H277	3.003216073	-4.891479865	-6.637611564
H278	1.835099003	-4.101463932	-4.341574727
H279	2.660271004	-1.909821157	-6.972032046
H280	9.146263057	0.959886851	-2.142099090
H281	8.427777835	-0.866758151	-4.720412678
H282	11.155817788	-1.198622710	-5.649706800
H283	9.770194732	-0.626414331	-1.635597648
H284	9.097368895	1.438447297	-6.036644641
H285	-4.105986176	-9.657462217	1.226888700
H286	-5.100693672	-8.640391733	2.285009203
H287	-3.691653403	-7.643049788	6.473349121
H288	-6.224369640	-10.705420473	2.008333060
H289	-7.043464936	-9.658683713	1.051340760
H290	5.697482371	-3.990511222	1.832219600
H291	8.626360252	-3.378237107	1.480800387
H292	7.447572647	-6.449821850	-1.257261070
H293	6.326546688	-2.785921969	0.660731383
H294	9.120154767	-4.931955218	1.610109578
H295	2.128094019	-8.523057574	0.146508718
H296	7.056285623	-5.761111038	0.787138928
H297	-2.781774369	2.294799952	9.313800771
H298	-3.793735667	-0.450079650	10.235013929
H299	0.182434058	-7.611659863	-0.600682927
H300	2.644151710	0.195027391	5.186944121
H301	-1.357300422	-7.804832948	-3.835070748
H302	-3.790138271	-2.993233418	-1.687091749
H303	-0.431560764	-6.331031287	-3.538782585
H304	-1.114330226	2.135762901	7.207065360
H305	-1.458753449	-6.582375208	-1.082819273
H306	-2.237707922	0.947581209	6.532755567
H307	-3.772177926	1.147702823	8.382115440
H308	-1.843862695	-0.537834503	8.536932269
H309	-0.843176762	0.733520817	9.264178771
H310	-4.633825009	-2.556755642	-6.353582329
H311	-4.707104541	2.307531400	10.975835791

H312	-7.707551498	1.706870299	10.819391868
H313	-1.451600187	-3.871157184	-1.557629393
H314	-6.216483650	3.174395760	12.853515814
H315	-7.228561903	3.734411383	11.693467397
H316	-0.469118222	-0.661159885	6.354461681
H317	-0.060433253	-3.990281008	-3.565774334
H318	-4.814692849	-2.572130218	-3.915233914
H319	-6.888178083	-4.041485959	9.844517751
H320	-6.078206306	-5.852157431	7.196176142
H321	-5.150573626	-2.420331168	8.952512579
H322	-8.117958721	-4.483110659	8.988444230
H323	0.727783693	-10.698476821	-0.986847244
H324	-2.568243761	-2.928766277	7.299675213
H325	-5.978033170	0.482374666	5.636161348
H326	-3.193655067	-7.730003550	0.396101202
H327	-7.526420710	-1.154090715	6.360729999
H328	-7.460102366	-6.719355577	2.613964036
H329	-5.691494604	-4.957042321	2.127606346
H330	-7.491931797	1.237297306	12.510252570
H331	1.489187084	-1.079875834	5.366452562
H332	-2.164623610	-2.971368100	9.639661904
H333	-1.087403588	-3.399150966	7.334743569
H334	-2.220383279	-1.401117560	11.421103622
H335	0.640703014	2.779305017	6.437199177
H336	-7.462710210	-4.005412673	5.234566429
H337	-6.918082565	-3.588176511	6.866295383
H338	-7.891110032	-1.629425138	4.693644750
H339	-3.513597893	-5.507818669	-0.522496211
H340	-1.085525383	-3.594454070	-5.828548317
H341	-3.095098569	-2.919265341	-7.182374121
H342	-4.432272055	0.018516347	4.899139170
H343	-3.547545244	-2.958693129	10.351005332
H344	-5.566052910	-2.044694973	10.392555792
H345	2.084934049	2.382301859	5.676155297
H346	-7.315731071	-7.590544721	5.022953648
H347	-4.498551968	-5.242227781	6.715663155
H348	-4.952663092	-6.126856520	-1.385806611
H349	2.784384177	-8.518897883	-1.506011603
H350	3.910751642	-0.481775228	7.748145170
H351	3.745770426	-2.033863972	6.888902137
H352	-4.990345337	-9.330534406	7.474636670
H353	-6.220915824	-8.308009380	7.823793235
H354	-4.779218811	-5.669330718	11.346797456
H355	-5.491833527	-5.950277819	9.195940730
H356	-4.154988028	-8.321241295	11.111488581
H357	-3.156624764	-5.437861162	10.664096533
H358	4.354329184	-3.364179882	9.189661324
H359	11.796778789	3.128138986	-6.708153687
H360	-0.977402532	0.345254481	13.536777584
H361	0.629732363	-0.698963146	11.537641756
H362	9.739145929	2.141903180	-8.717030613
H363	8.897763274	3.957948108	-6.370948334
H364	8.863975072	4.396210638	-8.089385293
H365	-2.516938062	-0.626141763	2.041026966
H366	3.237457018	1.274435926	-8.779607212
H367	-1.410204322	0.518220196	1.244247070
H368	0.348167243	-0.662851888	3.098095745
H369	-1.504584794	0.470945425	2.947939753
H370	-1.339178033	-0.479308141	-0.467402544
Mo371	-3.467872059	-3.629469266	4.192297358
N372	0.044976412	-0.771234508	2.122336617
N373	-1.439553188	-0.086436812	2.080224502
N374	-8.978919101	4.368263583	-2.572558440
N375	-6.421924346	6.660317986	-4.049014779
N376	-5.496231426	8.578136886	-2.216600325
N377	-5.294961310	7.266200231	0.283235598
N378	-3.103135320	5.609947459	-0.325799733
N379	-1.049688223	7.671155715	-0.602651415
N380	-1.050650384	9.242660176	1.849163214
N381	-1.267623092	7.321300399	3.891731669
N382	0.153791329	4.881178774	3.214847665
N383	2.913665003	5.122725091	1.968548717
N384	3.583770575	7.694631746	3.031652535
N385	-6.487036337	3.056222751	11.874654555
N386	-4.729642986	1.291493479	10.840184236
N387	-0.254657333	0.319764774	6.559976278
N388	1.328725533	2.042952224	6.272575835
N389	1.665101644	-0.055375701	5.322868465
N390	-1.956102585	-0.531524468	11.936423425
N391	-5.583452486	0.982070543	-4.445066463

N392	-6.678833035	-0.919706312	-0.231683160
N393	-3.197082746	1.476656825	-5.410712135
N394	-2.629733421	3.515919268	-7.230611119
N395	-1.778406513	5.623667111	-5.567420858
N396	0.648623021	4.700575318	-4.479003472
N397	0.785471969	0.473154894	-3.995854066
N398	-0.611740713	0.822486580	-2.302489294
N399	2.924001689	5.163144700	-5.968323435
N400	3.992689120	5.503554451	-10.582394083
N401	6.048925950	6.323422817	-10.740821425
N402	6.224635254	-2.964265494	7.429457131
N403	8.295905499	-4.328369268	1.679918688
N404	7.858058644	-5.585820510	-1.601733261
N405	6.272598955	-4.536336099	-3.616497767
N406	3.042624190	-2.854718305	-7.186288607
N407	4.160004013	-4.162751998	-8.783512463
N408	4.401060048	-1.862122740	-8.782381251
N409	5.411081486	-1.927588991	-2.739329658
N410	7.884041942	-0.661699479	-2.534801712
N411	9.393736622	-0.555510081	-4.825736040
N412	7.312794252	-2.773191321	-7.289714791
N413	9.839002010	1.447359660	-6.732468079
N414	-6.154165419	-10.157892122	1.147873303
N415	-4.051842203	-7.490031098	-0.108945632
N416	-1.784865193	-6.885320304	-2.021871709
N417	0.712079810	-8.121901128	-1.318048581
N418	-6.570844110	-7.189235795	-4.099911282
N419	-4.973439790	-5.255701068	-5.500465116
N420	-5.477360104	-8.492712525	7.142844180
N421	-5.100323298	-5.276996128	4.174467848
N422	-6.754171275	-6.429470186	3.285138475
N423	-4.596390161	-6.393238610	9.414030044
N424	2.148352316	-0.371119726	-1.284564491
N425	1.616303949	-0.946657619	-0.472676816
O426	-6.359128936	5.484214643	-2.098144465
O427	-3.365818287	7.818994269	-2.479881596
O428	-3.938881529	8.953777883	1.005962279
O429	-2.502053420	5.740851432	1.866718872
O430	0.379042718	6.049398902	0.079705303
O431	1.224601511	9.106619704	1.699155243
O432	-0.384357047	8.821604035	5.393641176
O433	0.064833327	4.321334098	5.432101128
O434	3.305674766	3.692352047	3.719082256
O435	5.535284169	6.725845384	3.666088227
O436	4.833515285	9.185355902	6.040391002
O437	-6.116931934	-0.517759662	11.226985648
O438	-2.218019713	1.744277611	12.124542232
O439	1.224897598	-1.478547700	13.326726510
O440	-2.805190559	3.272305122	-4.029556791
O441	-6.613100459	-0.798874333	-2.502064928
O442	-0.397223026	3.161251548	-6.924826891
O443	-0.599965007	0.191069226	-7.696032875
O444	-1.310153228	6.900629724	-7.418627267
O445	0.893680372	6.626955418	-3.273706468
O446	4.224206794	4.898458807	-4.116445229
O447	5.802498532	7.197105115	-6.310133834
O448	4.774797473	-1.764077768	10.387311482
O449	6.597989818	1.069213505	2.398683113
O450	8.387934680	-3.418047945	-1.137034683
O451	7.980612253	-4.662789927	-5.145401043
O452	5.398108711	-1.400406306	-4.965595594
O453	6.863993928	1.354549973	-2.933392589
O454	6.356732844	-0.516285395	-0.054918713
O455	11.266164158	0.077965892	-3.663850741
O456	11.284206958	0.118929235	-7.891043088
O457	8.078008656	-0.796717747	-8.147245751
O458	11.654693008	3.917692615	-8.588113167
O459	3.670759007	0.976911257	-7.956188533
O460	-6.334391230	-7.739637394	-0.189326505
O461	-3.619187198	-6.928061201	-3.393667284
O462	1.056572998	-8.635937386	-3.508754673
O463	2.729324707	-11.086108193	-0.877696093
O464	-6.953568436	-4.501458862	-6.342910094
O465	-8.375849036	-3.497375227	-0.527911382
O466	-7.228936163	-5.262513246	0.215210028
O467	-2.112085671	-5.258285384	-7.650055379
O468	-2.808245863	-7.649539874	8.814616020
O469	-2.719280001	-7.457150326	12.276633767
O470	-5.884213693	-0.610189841	2.535549040
O471	-6.359967442	1.465020080	3.301900335

	O472 -9.211249464 -4.060762670 7.696644160<br>O473 -9.984487852 -3.011186799 5.848813681<br>O474 -4.060445051 -3.034332861 6.229234886<br>O475 -5.087903799 -1.495802709 7.509161836<br>O476 -5.395016620 -2.456226278 4.079120325<br>O477 -2.569424410 -2.925084817 10.541438920<br>O478 -1.733993880 -2.862512505 7.830379839<br>O479 1.847458243 -0.322281398 -6.576126423<br>O480 -7.578340587 -4.739902289 9.796191005<br>O481 -5.251430867 -2.818524333 9.861623070<br>S482 4.741003860 -4.274437665 -0.377903988<br>S483 1.327544646 -3.869783358 -0.528516792<br>S484 -3.422429773 -3.421647268 1.830736436<br>S485 2.796572989 -2.160777512 1.956270908<br>S486 -1.304008420 -1.821654611 -0.184604250<br>S487 -1.077306738 -6.505808987 1.110570203<br>S488 -2.022122763 -1.695636498 4.399551945<br>S489 2.330724536 -5.609259855 2.446139330<br>S490 -1.888593566 -5.348925202 4.556410202<br>S491 1.225942902 -3.333438397 5.313849724<br>end

product

Fe(139) 2.312<br>Fe(140) -0.100<br>Fe(141) 2.556<br>Fe(142) -2.418<br>Fe(143) 2.534<br>Fe(144) -2.261<br>Fe(145) -0.825	bm612n2xnewbrk2bh147te_5_53451.82<br>C1 -6.985096531 5.842496717 -3.109751173<br>C2 -8.445375509 5.456739463 -3.374555881<br>C3 -5.003330283 6.977169114 -4.020047356<br>C4 -4.556254334 7.832459544 -2.840389951<br>C5 -5.169138876 9.349951396 -1.029270271<br>C6 -4.742630832 8.502109408 0.173706466<br>C7 -4.795671605 6.327450595 1.280800679<br>C8 -3.365231222 5.860681658 0.977635067<br>C9 -1.759143927 5.323941241 -0.809465725<br>C10 -0.718348251 6.376313114 -0.402269915<br>C11 -0.051846123 8.690515699 -0.320422649<br>C12 0.104453153 9.015990959 1.170135338<br>C13 -1.030733403 9.668907328 3.237658362<br>C14 -0.852236070 8.558336820 4.277433479<br>C15 -1.250166184 6.189642050 4.791656879<br>C16 -0.264700374 5.063561303 4.491930051<br>C17 1.047647480 3.774993183 2.825561984<br>C18 2.529720788 4.198257351 2.895474467<br>C19 0.688501583 3.254086569 1.418655305<br>C20 1.632567966 2.141652990 0.959345329<br>C21 -0.780699625 2.842348150 1.403314036<br>C22 4.281951268 5.633051556 1.891251058<br>C23 4.525328636 6.720453387 2.943490960<br>C24 4.617533789 6.126279935 0.459322245<br>C25 5.993211002 6.805640341 0.406403856<br>C26 4.547746277 4.968842997 -0.542135927<br>C27 3.833877430 8.839232299 3.878402570<br>C28 4.175800389 8.464195826 5.304478180<br>C29 -6.980298391 1.692548471 11.645383219<br>C30 -5.885914808 0.708543786 11.221205206<br>C31 -3.516572562 0.594101351 10.426873197<br>C32 -2.495223456 0.660587230 11.579030387<br>C33 -2.967078146 1.223058313 9.130300824<br>C34 -1.696080623 0.551661528 8.591085465<br>C35 -1.356820577 1.065899798 7.185005648<br>C36 0.895307623 0.786583897 6.051426409<br>C37 -1.018340350 -0.638921554 13.036964666<br>C38 0.389813719 -0.970909941 12.594681472<br>C39 -4.756490845 1.847240391 -3.593481434<br>C40 -3.488985868 2.282624471 -4.354628281<br>C41 -4.330062019 1.122951975 -2.296402915<br>C42 -5.438750257 0.924581511 -1.241108864<br>C43 -6.282964243 -0.343089902 -1.380799545<br>C44 -1.905212939 1.468814506 -6.080500054<br>C45 -1.575782547 2.786536157 -6.791717474<br>C46 -1.879604913 0.266748855 -7.037052572<br>C47 -2.449881781 4.834079181 -7.811312726<br>C48 -1.786512987 5.878003113 -6.904629363<br>C49 -1.062261090 6.485437230 -4.645362148<br>C50 0.246759594 5.938714357 -4.074637829<br>C51 1.908541207 4.142869494 -4.000387794<br>C52 3.127777281 4.797833826 -4.676775074<br>C53 2.008804168 2.634486635 -4.275897600<br>C54 0.984719640 1.782746759 -3.601695506<br>C55 0.105672439 1.972108382 -2.554805318<br>C56 -0.176310045 -0.065348524 -3.187409256

	C57	4.010648202	5.623825302	-6.815131441
	C58	4.631753475	6.873711083	-6.195673611
	C59	3.411176675	5.947770200	-8.204349433
	C60	4.373597689	6.016433155	-9.352309257
	C61	5.651858748	6.533150439	-9.436805373
	C62	5.023976154	5.705065206	-11.394053371
	C63	5.543080984	-1.846811478	8.087021616
	C64	4.838525194	-2.366983927	9.325426607
	C65	4.456816158	-1.237583199	7.163157604
	C66	5.036301997	-0.610993643	5.916968236
	C67	5.453091081	0.732948859	5.901760573
	C68	5.182583733	-1.359044651	4.734442503
	C69	5.979528714	1.317317497	4.744898033
	C70	5.719677655	-0.793844882	3.577453539
	C71	6.106886225	0.548270277	3.581290646
	C72	7.319721862	-4.701248198	0.641044919
	C73	7.901922078	-4.501818873	-0.770733617
	C74	6.076896707	-3.845413057	0.813218087
	C75	8.394068781	-5.554128986	-2.951254216
	C76	7.520898295	-4.866755709	-4.004137966
	C77	5.333234176	-3.760347640	-4.421014604
	C78	5.381351239	-2.252509786	-4.062273471
	C79	3.929255467	-4.365977206	-4.192897232
	C80	2.718458021	-3.694384205	-4.850717971
	C81	2.522111042	-3.945621614	-6.355827182
	C82	3.842142029	-2.970205537	-8.243706805
	C83	5.451245685	-0.518780396	-2.338628168
	C84	6.795081174	0.151056372	-2.653788802
	C85	5.172333001	-0.311104183	-0.835400503
	C86	9.216035017	-0.101350726	-2.425285322
	C87	10.057107809	-0.184830087	-3.687908656
	C88	10.156617521	-0.903367948	-6.006902939
	C89	10.464130466	0.267879941	-6.973784691
	C90	9.595123269	-2.098054023	-6.784817724
	C91	8.261933917	-1.811798067	-7.460670424
	C92	9.882079347	2.539003434	-7.698974898
	C93	11.229464061	3.244254718	-7.662293784
	C94	8.748545470	3.558405421	-7.386373959
	C95	7.370169573	2.948508706	-7.516302243
	C96	6.699590532	2.957677090	-8.750067713
	C97	6.741873266	2.313025790	-6.431881592
	C98	5.457322913	2.338070653	-8.906326923
	C99	5.512883755	1.662942838	-6.575413131
	C100	4.882627744	1.679549853	-7.817680004
	C101	-5.087500936	-9.166585263	1.307954068
	C102	-5.209668569	-8.076564674	0.256582591
	C103	-3.947023713	-6.376135714	-1.042776238
	C104	-3.103486482	-6.747211552	-2.269692773
	C105	-0.829327218	-7.240991091	-3.056290386
	C106	0.380074839	-8.069867299	-2.632833438
	C107	1.917546414	-8.814148814	-0.895488189
	C108	1.778433937	-10.323189537	-0.931555380
	C109	-7.063007291	-5.821853239	-4.315473565
	C110	-6.331423751	-5.140721142	-5.477596990
	C111	-7.080624221	-4.890637561	-3.081403582
	C112	-8.121617493	-5.358919165	-2.047181288
	C113	-7.945375655	-4.625902726	-0.735861988
	C114	-4.203912187	-4.659977472	-6.576503502
	C115	-3.105219092	-5.602681347	-7.025890949
	C116	-3.742400526	-3.200260851	-6.337429509
	C117	-3.031679145	-3.115334379	-5.020814181
	C118	-1.676112631	-3.464476512	-4.919410225
	C119	-3.776694970	-2.905910847	-3.846066856
	C120	-1.110854436	-3.712739680	-3.666845961
	C121	-3.210291835	-3.158003461	-2.597863724
	C122	-1.893994013	-3.623126156	-2.517241075
	C123	-4.530969748	-7.361478552	7.127108948
	C124	-3.900186906	-7.130999174	8.507964939
	C125	-5.189432766	-6.084439745	6.584394459
	C126	-5.620714192	-6.123198964	5.152739586
	C127	-6.651487413	-6.849490906	4.593691958
	C128	-5.814071719	-5.479783948	3.067886946
	C129	-4.006557041	-6.136866270	10.715741217
	C130	-3.576359810	-7.412746113	11.404124466
	C131	-5.985798603	0.288034785	3.473936579
	C132	-5.520422147	-0.161251213	4.874616971
	C133	-5.786436657	-1.652133313	5.215741106
	C134	-7.275866115	-1.880575013	5.571628809
	C135	-7.606457296	-3.292864774	6.062154419
	C136	-9.053457164	-3.457325732	6.576635355

C137	-4.922676699	-2.071837753	6.414609476
C138	-0.216523887	-3.497244665	2.299801145
Fe139	-1.743295091	-4.784483618	2.356462911
Fe140	0.794308297	-1.960936228	0.739319964
Fe141	1.559310614	-3.583754387	3.142935092
Fe142	0.526469279	-4.972119549	1.244092799
Fe143	2.967283252	-4.151811374	0.930647457
Fe144	-0.703191044	-3.484442882	4.272329896
Fe145	-1.510659402	-2.004451702	2.086723653
H146	-8.838084145	4.569423871	-1.579429956
H147	-8.456498944	3.508629295	-2.758479399
H148	-6.936318789	6.842852437	-4.906889582
H149	-8.560699330	5.213579421	-4.442820541
H150	-9.051375039	6.362929736	-3.202051678
H151	-4.399233106	6.057893869	-3.984167842
H152	-6.452347349	8.549082170	-2.561659159
H153	-4.744416055	7.508388872	-4.945277789
H154	-6.047485873	9.945980407	-0.749709131
H155	-5.886615551	6.908074615	-0.469475548
H156	-4.339265391	10.040492636	-1.232369612
H157	-1.410337615	4.360406189	-0.429661798
H158	-3.854121146	5.696126890	-1.005754078
H159	-5.469095264	5.459842396	1.312372195
H160	0.927776915	8.351963241	-0.679964903
H161	-1.903434642	7.903506032	-1.115577292
H162	-4.786789678	6.794462681	2.272695914
H163	-1.812984587	5.251192252	-1.903086820
H164	-1.952919997	9.159219379	1.373024113
H165	-0.332618809	9.607513777	-0.854770831
H166	-1.449250780	3.703731037	1.516681313
H167	-1.038362198	2.326621247	0.463157114
H168	-0.989985946	2.195169803	2.260044443
H169	0.796603287	4.090372550	0.713408041
H170	-1.976017342	10.185942948	3.455297874
H171	3.557398153	4.492792475	-0.553668379
H172	2.790319577	7.749534655	2.391897537
H173	6.043403256	7.698760896	1.046280010
H174	1.349116839	1.817316861	-0.050002739
H175	1.598028766	1.258609515	1.613597859
H176	6.214718076	-1.027201049	8.407092363
H177	4.754740544	5.322836545	-1.561366278
H178	6.213769209	7.115019379	-0.625765251
H179	5.292453287	4.196330020	-0.291950338
H180	6.786014678	6.113260957	0.730476070
H181	3.859296306	6.880579943	0.176881849
H182	4.960250730	4.808998773	2.156351601
H183	2.215761842	5.503289155	1.328061327
H184	2.674809723	2.490696968	0.905519409
H185	4.378651128	-1.000845436	-0.514093670
H186	4.806063053	0.719702786	-0.717638711
H187	6.317376656	0.081246338	0.718989083
H188	6.786177408	2.018703968	2.512309379
H189	5.802304819	-1.378588376	2.662002965
H190	4.841935500	-2.396157526	4.705830714
H191	5.350913986	1.341916389	6.803632417
H192	-0.191159491	5.500159919	2.481606326
H193	-2.247651701	5.723682832	4.827442051
H194	-1.007946349	6.551844022	5.797392207
H195	-1.761322806	7.200321147	3.005606336
H196	-0.207829463	10.378218008	3.396290297
H197	6.269686695	2.371190846	4.742938854
H198	0.921669532	2.979494720	3.569684780
H199	4.644226211	9.476837097	3.485111235
H200	2.914063673	9.443708804	3.905203396
H201	3.725816247	7.511778371	5.668024097
H202	-1.352347564	-1.381090931	13.778787527
H203	-7.265667811	-1.756903689	-0.292880329
H204	-5.542696056	-1.785886306	3.255998185
H205	-9.131086883	-5.145388622	-2.426640730
H206	-8.036837166	-6.443280117	-1.885550480
H207	-7.316955774	-3.858495049	-3.383367768
H208	-3.289290579	-6.675890906	-6.782570374
H209	-8.098408962	-5.906496956	-4.678441740
H210	-3.880403390	0.154022883	-2.565175092
H211	-6.137125975	1.779728683	-1.261843714
H212	-4.483962924	-5.752503828	-4.748805467
H213	-4.895753935	-4.634408828	-7.443853759
H214	-5.650845466	-7.187489962	-3.642387694
H215	-6.368143252	-0.618516388	0.708178090
H216	-6.073558466	-4.865252194	-2.637889604

H217	-7.209296424	-7.690022237	-3.478024860
H218	-6.288295735	1.529665676	-4.943884520
H219	-3.526170609	1.715425858	-1.837687987
H220	-6.935270807	-6.177822337	-0.080428093
H221	-4.986458685	0.937797369	-0.238438017
H222	2.002961316	4.351422549	-2.926115655
H223	-0.053849883	2.875467772	-1.975344229
H224	-0.810993623	7.412297427	-5.179459191
H225	-2.191228806	4.756929416	-5.197629613
H226	1.988575871	5.090048862	-6.360285797
H227	6.301099900	6.995596700	-8.700898726
H228	3.896734885	7.513832815	-5.649757740
H229	2.818637923	6.877001770	-8.131198517
H230	4.789192290	4.850215611	-6.905875279
H231	5.078098669	5.426001444	-12.443089301
H232	3.020891502	2.324467778	-3.964482412
H233	1.955493280	2.475467858	-5.366016743
H234	6.949845399	6.578600403	-11.136379253
H235	2.695373122	5.146505086	-8.446923208
H236	0.102863525	4.175373341	-5.167182218
H237	-1.704098341	6.773236481	-3.798864587
H238	-1.824784156	4.778749269	-8.713408454
H239	-0.494116509	-1.103103116	-3.262691137
H240	-3.433373197	5.217489535	-8.112802074
H241	7.081648074	-3.192534598	7.939318541
H242	6.521241787	-2.679370941	6.492053704
H243	5.225824559	-2.642472615	-2.011044179
H244	4.686925291	0.029465889	-2.907525638
H245	7.747063611	-1.584536836	-2.114317675
H246	1.257153006	0.031670780	-4.799736039
H247	0.967394586	-0.122802637	-6.991222222
H248	-0.706501004	-0.412366163	-8.452942551
H249	2.477571152	0.349074974	-6.923156327
H250	-2.064296289	-0.643804419	-6.443381561
H251	-2.677901769	0.373957465	-7.788264229
H252	9.540300853	-2.974868792	-6.124683751
H253	10.319359112	-2.322329503	-7.583070513
H254	7.434074405	-3.469482695	-6.542800123
H255	6.369870861	-2.487707843	-7.546514865
H256	4.978735328	-4.244341028	-9.378465865
H257	1.450965140	-4.057681900	-6.581093601
H258	4.945327379	2.366169877	-9.872144399
H259	7.200030919	2.321362618	-5.439385440
H260	4.161699727	-0.937234227	-8.414762361
H261	3.746101727	-4.370098954	-3.106205669
H262	5.997882481	-4.741430210	-2.649261948
H263	2.707155679	-2.611455200	-4.649749049
H264	-3.566688285	3.176371422	-7.025023528
H265	8.572688776	-6.585026475	-3.285447519
H266	-3.904542342	0.732482682	-5.529890696
H267	3.969603820	-5.423934265	-4.500906663
H268	5.074623222	1.125426465	-5.734370072
H269	9.359539385	-5.027703166	-2.957249693
H270	3.780628628	-5.022300015	-8.403464167
H271	4.727025990	-1.897001274	-9.744199986
H272	-6.067541414	0.283012914	-3.851039661
H273	-5.260497145	2.788625158	-3.302373133
H274	7.150184736	3.459035573	-9.609582395
H275	-1.104488763	1.345000877	-5.336677807
H276	5.632586638	-3.838100016	-5.470103485
H277	3.003216073	-4.891479865	-6.637611564
H278	1.835099003	-4.101463932	-4.341574727
H279	2.660271004	-1.909821157	-6.972032046
H280	9.146263057	0.959886851	-2.142099090
H281	8.427777835	-0.866758151	-4.720412678
H282	11.155817788	-1.198622710	-5.649706800
H283	9.770194732	-0.626414331	-1.635597648
H284	9.097368895	1.438447297	-6.036644641
H285	-4.105986176	-9.657462217	1.226888700
H286	-5.100693672	-8.640391733	2.285009203
H287	-3.691653403	-7.643049788	6.473349121
H288	-6.224369640	-10.705420473	2.008333060
H289	-7.043464936	-9.658683713	1.051340760
H290	5.697482371	-3.990511222	1.832219600
H291	8.626360252	-3.378237107	1.480800387
H292	7.447572647	-6.449821850	-1.257261070
H293	6.326546688	-2.785921969	0.660731383
H294	9.120154767	-4.931955218	1.610109578
H295	2.128094019	-8.523057574	0.146508718
H296	7.056285623	-5.761111038	0.787138928

H297	-2.781774369	2.294799952	9.313800771
H298	-3.793735667	-0.450079650	10.235013929
H299	0.182434058	-7.611659863	-0.600682927
H300	2.644151710	0.195027391	5.186944121
H301	-1.357300422	-7.804832948	-3.835070748
H302	-3.790138271	-2.993233418	-1.687091749
H303	-0.431560764	-6.331031287	-3.538782585
H304	-1.114330226	2.135762901	7.207065360
H305	-1.458753449	-6.582375208	-1.082819273
H306	-2.237707922	0.947581209	6.532755567
H307	-3.772177926	1.147702823	8.382115440
H308	-1.843862695	-0.537834503	8.536932269
H309	-0.843176762	0.733520817	9.264178771
H310	-4.633825009	-2.556755642	-6.353582329
H311	-4.707104541	2.307531400	10.975835791
H312	-7.707551498	1.706870299	10.819391868
H313	-1.451600187	-3.871157184	-1.557629393
H314	-6.216483650	3.174395760	12.853515814
H315	-7.228561903	3.734411383	11.693467397
H316	-0.469118222	-0.661159885	6.354461681
H317	-0.060433253	-3.990281008	-3.565774334
H318	-4.814692849	-2.572130218	-3.915233914
H319	-6.888178083	-4.041485959	9.844517751
H320	-6.078206306	-5.852157431	7.196176142
H321	-5.150573626	-2.420331168	8.952512579
H322	-8.117958721	-4.483110659	8.988444230
H323	0.727783693	-10.698476821	-0.986847244
H324	-2.568243761	-2.928766277	7.299675213
H325	-5.978033170	0.482374666	5.636161348
H326	-3.193655067	-7.730003550	0.396101202
H327	-7.526420710	-1.154090715	6.360729999
H328	-7.460102366	-6.719355577	2.613964036
H329	-5.691494604	-4.957042321	2.127606346
H330	-7.491931797	1.237297306	12.510252570
H331	1.489187084	-1.079875834	5.366452562
H332	-2.164623610	-2.971368100	9.639661904
H333	-1.087403588	-3.399150966	7.334743569
H334	-2.220383279	-1.401117560	11.421103622
H335	0.640703014	2.779305017	6.437199177
H336	-7.462710210	-4.005412673	5.234566429
H337	-6.918082565	-3.588176511	6.866295383
H338	-7.891110032	-1.629425138	4.693644750
H339	-3.513597893	-5.507818669	-0.522496211
H340	-1.085525383	-3.594454070	-5.828548317
H341	-3.095098569	-2.919265341	-7.182374121
H342	-4.432272055	0.018516347	4.899139170
H343	-3.547545244	-2.958693129	10.351005332
H344	-5.566052910	-2.044694973	10.392555792
H345	2.084934049	2.382301859	5.676155297
H346	-7.315731071	-7.590544721	5.022953648
H347	-4.498551968	-5.242227781	6.715663155
H348	-4.952663092	-6.126856520	-1.385806611
H349	2.784384177	-8.518897883	-1.506011603
H350	3.910751642	-0.481775228	7.748145170
H351	3.745770426	-2.033863972	6.888902137
H352	-4.990345337	-9.330534406	7.474636670
H353	-6.220915824	-8.308009380	7.823793235
H354	-4.779218811	-5.669330718	11.346797456
H355	-5.491833527	-5.950277819	9.195940730
H356	-4.154988028	-8.321241295	11.111488581
H357	-3.156624764	-5.437861162	10.664096533
H358	4.354329184	-3.364179882	9.189661324
H359	11.796778789	3.128138986	-6.708153687
H360	-0.977402532	0.345254481	13.536777584
H361	0.629732363	-0.698963146	11.537641756
H362	9.739145929	2.141903180	-8.717030613
H363	8.897763274	3.957948108	-6.370948334
H364	8.863975072	4.396210638	-8.089385293
H365	-2.516938062	-0.626141763	2.041026966
H366	3.237457018	1.274435926	-8.779607212
H367	-1.410204322	0.518220196	1.244247070
H368	0.348167243	-0.662851888	3.098095745
H369	-1.504584794	0.470945425	2.947939753
H370	-1.339178033	-0.479308141	-0.467402544
Mo371	-3.467872059	-3.629469266	4.192297358
N372	0.044976412	-0.771234508	2.122336617
N373	-1.439553188	-0.086436812	2.080224502
N374	-8.978919101	4.368263583	-2.572558440
N375	-6.421924346	6.660317986	-4.049014779
N376	-5.496231426	8.578136886	-2.216600325

N377	-5.294961310	7.266200231	0.283235598
N378	-3.103135320	5.609947459	-0.325799733
N379	-1.049688223	7.671155715	-0.602651415
N380	-1.050650384	9.242660176	1.849163214
N381	-1.267623092	7.321300399	3.891731669
N382	0.153791329	4.881178774	3.214847665
N383	2.913665003	5.122725091	1.968548717
N384	3.583770575	7.694631746	3.031652535
N385	-6.487036337	3.056222751	11.874654555
N386	-4.729642986	1.291493479	10.840184236
N387	-0.254657333	0.319764774	6.559976278
N388	1.328725533	2.042952224	6.272575835
N389	1.665101644	-0.055375701	5.322868465
N390	-1.956102585	-0.531524468	11.936423425
N391	-5.583452486	0.982070543	-4.445066463
N392	-6.678833035	-0.919706312	-0.231683160
N393	-3.197082746	1.476656825	-5.410712135
N394	-2.629733421	3.515919268	-7.230611119
N395	-1.778406513	5.623667111	-5.567420858
N396	0.648623021	4.700575318	-4.479003472
N397	0.785471969	0.473154894	-3.995854066
N398	-0.611740713	0.822486580	-2.302489294
N399	2.924001689	5.163144700	-5.968323435
N400	3.992689120	5.503554451	-10.582394083
N401	6.048925950	6.323422817	-10.740821425
N402	6.224635254	-2.964265494	7.429457131
N403	8.295905499	-4.328369268	1.679918688
N404	7.858058644	-5.585820510	-1.601733261
N405	6.272598955	-4.536336099	-3.616497767
N406	3.042624190	-2.854718305	-7.186288607
N407	4.160004013	-4.162751998	-8.783512463
N408	4.401060048	-1.862122740	-8.782381251
N409	5.411081486	-1.927588991	-2.739329658
N410	7.884041942	-0.661699479	-2.534801712
N411	9.393736622	-0.555510081	-4.825736040
N412	7.312794252	-2.773191321	-7.289714791
N413	9.839002010	1.447359660	-6.732468079
N414	-6.154165419	-10.157892122	1.147873303
N415	-4.051842203	-7.490031098	-0.108945632
N416	-1.784865193	-6.885320304	-2.021871709
N417	0.712079810	-8.121901128	-1.318048581
N418	-6.570844110	-7.189235795	-4.099911282
N419	-4.973439790	-5.255701068	-5.500465116
N420	-5.477360104	-8.492712525	7.142844180
N421	-5.100323298	-5.276996128	4.174467848
N422	-6.754171275	-6.429470186	3.285138475
N423	-4.596390161	-6.393238610	9.414030044
N424	2.148352316	-0.371119726	-1.284564491
N425	1.616303949	-0.946657619	-0.472676816
O426	-6.359128936	5.484214643	-2.098144465
O427	-3.365818287	7.818994269	-2.479881596
O428	-3.938881529	8.953777883	1.005962279
O429	-2.502053420	5.740851432	1.866718872
O430	0.379042718	6.049398902	0.079705303
O431	1.224601511	9.106619704	1.699155243
O432	-0.384357047	8.821604035	5.393641176
O433	0.064833327	4.321334098	5.432101128
O434	3.305674766	3.692352047	3.719082256
O435	5.535284169	6.725845384	3.666088227
O436	4.833515285	9.185355902	6.040391002
O437	-6.116931934	-0.517759662	11.226985648
O438	-2.218019713	1.744277611	12.124542232
O439	1.224897598	-1.478547700	13.326726510
O440	-2.805190559	3.272305122	-4.029556791
O441	-6.613100459	-0.798874333	-2.502064928
O442	-0.397223026	3.161251548	-6.924826891
O443	-0.599965007	0.191069226	-7.696032875
O444	-1.310153228	6.900629724	-7.418627267
O445	0.893680372	6.626955418	-3.273706468
O446	4.224206794	4.898458807	-4.116445229
O447	5.802498532	7.197105115	-6.310133834
O448	4.774797473	-1.764077768	10.387311482
O449	6.597989818	1.069213505	2.398683113
O450	8.387934680	-3.418047945	-1.137034683
O451	7.980612253	-4.662789927	-5.145401043
O452	5.398108711	-1.400406306	-4.965595594
O453	6.863993928	1.354549973	-2.933392589
O454	6.356732844	-0.516285395	-0.054918713
O455	11.266164158	0.077965892	-3.663850741
O456	11.284206958	0.118929235	-7.891043088

	O457 8.078008656 -0.796717747 -8.147245751<br>O458 11.654693008 3.917692615 -8.588113167<br>O459 3.670759007 0.976911257 -7.956188533<br>O460 -6.334391230 -7.739637394 -0.189326505<br>O461 -3.619187198 -6.928061201 -3.393667284<br>O462 1.056572998 -8.635937386 -3.508754673<br>O463 2.729324707 -11.086108193 -0.877696093<br>O464 -6.953568436 -4.501458862 -6.342910094<br>O465 -8.375849036 -3.497375227 -0.527911382<br>O466 -7.228936163 -5.262513246 0.215210028<br>O467 -2.112085671 -5.258285384 -7.650055379<br>O468 -2.808245863 -7.649539874 8.814616020<br>O469 -2.719280001 -7.457150326 12.276633767<br>O470 -5.884213693 -0.610189841 2.535549040<br>O471 -6.359967442 1.465020080 3.301900335<br>O472 -9.211249464 -4.060762670 7.696644160<br>O473 -9.984487852 -3.011186799 5.848813681<br>O474 -4.060445051 -3.034332861 6.229234886<br>O475 -5.087903799 -1.495802709 7.509161836<br>O476 -5.395016620 -2.456226278 4.079120325<br>O477 -2.569424410 -2.925084817 10.541438920<br>O478 -1.733993880 -2.862512505 7.830379839<br>O479 1.847458243 -0.322281398 -6.576126423<br>O480 -7.578340587 -4.739902289 9.796191005<br>O481 -5.251430867 -2.818524333 9.861623070<br>S482 4.741003860 -4.274437665 -0.377903988<br>S483 1.327544646 -3.869783358 -0.528516792<br>S484 -3.422429773 -3.421647268 1.830736436<br>S485 2.796572989 -2.160777512 1.956270908<br>S486 -1.304008420 -1.821654611 -0.184604250<br>S487 -1.077306738 -6.505808987 1.110570203<br>S488 -2.022122763 -1.695636498 4.399551945<br>S489 2.330724536 -5.609259855 2.446139330<br>S490 -1.888593566 -5.348925202 4.556410202<br>S491 1.225942902 -3.333438397 5.313849724<br>end

S2BH-Fe2-brNH-Fe6NH3 to S2BH-Fe2-brNH-Fe6

35, S=1/2

reactant

Fe(139) -2.099<br>Fe(140) -0.263<br>Fe(141) -2.562<br>Fe(142) 2.714<br>Fe(143) 1.615<br>Fe(144) 2.482<br>Fe(145) 0.343	bm612n2xnewbrk2bh135ti_5_53445.66<br>C1 -6.979042379 5.834667504 -3.105727756<br>C2 -8.440635744 5.451223507 -3.369193752<br>C3 -5.005818383 6.982255782 -4.021989994<br>C4 -4.560479414 7.841004638 -2.844104849<br>C5 -5.175186384 9.352769639 -1.029269425<br>C6 -4.746090185 8.506472340 0.174289037<br>C7 -4.786537784 6.328778635 1.278134733<br>C8 -3.354547597 5.866723790 0.974617886<br>C9 -1.749329280 5.333972926 -0.814534277<br>C10 -0.709504701 6.388350751 -0.407545879<br>C11 -0.054045612 8.707726467 -0.330464447<br>C12 0.104732918 9.027226956 1.161188659<br>C13 -1.028116642 9.668922260 3.233163631<br>C14 -0.842383459 8.558080302 4.271836374<br>C15 -1.257200892 6.193065737 4.795319923<br>C16 -0.274215525 5.060620376 4.506562281<br>C17 1.043980619 3.771918117 2.835220227<br>C18 2.523692522 4.199909680 2.914159324<br>C19 0.683281228 3.251616882 1.432241533<br>C20 1.626774032 2.135748098 0.981643670<br>C21 -0.782747779 2.829659202 1.425773021<br>C22 4.279574190 5.628105483 1.900936999<br>C23 4.524470595 6.721021910 2.948479271<br>C24 4.611307495 6.117628632 0.466506219<br>C25 5.985880799 6.798327243 0.408221829<br>C26 4.542120721 4.960625303 -0.535971070<br>C27 3.826325692 8.839445036 3.887979820

	C28	4.166363491	8.456716859	5.312901723
	C29	-6.972715323	1.687361251	11.642002773
	C30	-5.878558687	0.705981120	11.215096114
	C31	-3.510559518	0.594410527	10.420018346
	C32	-2.490690903	0.660508235	11.573567900
	C33	-2.962550929	1.226211761	9.124803604
	C34	-1.691327646	0.558789004	8.584808219
	C35	-1.345069145	1.078036096	7.183281163
	C36	0.902961700	0.774500614	6.053233761
	C37	-1.016905704	-0.639097751	13.035076558
	C38	0.392848288	-0.968511341	12.594803967
	C39	-4.753253516	1.841154858	-3.586723562
	C40	-3.492688324	2.285014000	-4.354834554
	C41	-4.324816081	1.115894624	-2.293439107
	C42	-5.435653165	0.920783052	-1.240151938
	C43	-6.281761293	-0.346179172	-1.379191599
	C44	-1.903976928	1.468127542	-6.077857403
	C45	-1.574945774	2.783885322	-6.792141096
	C46	-1.880208207	0.266021103	-7.034417476
	C47	-2.447865135	4.829447211	-7.814622882
	C48	-1.787813369	5.875114765	-6.907423323
	C49	-1.061048915	6.483416847	-4.650015511
	C50	0.248575865	5.938114980	-4.078692518
	C51	1.911927699	4.143557953	-4.000209379
	C52	3.129839000	4.800030275	-4.676505766
	C53	2.013609109	2.635008351	-4.275732234
	C54	0.986615603	1.788022575	-3.600456153
	C55	0.096741845	1.992636200	-2.565388490
	C56	-0.196248954	-0.045639183	-3.188125015
	C57	4.013848195	5.621926686	-6.815896645
	C58	4.637257270	6.869699029	-6.195020825
	C59	3.414427013	5.945105459	-8.204744600
	C60	4.376710883	6.015420102	-9.352593404
	C61	5.654202595	6.534062805	-9.437123446
	C62	5.027316202	5.705531934	-11.394505022
	C63	5.548140003	-1.844691481	8.092763119
	C64	4.839968562	-2.367785655	9.327238396
	C65	4.459259040	-1.237272981	7.167015162
	C66	5.036869399	-0.612356190	5.918886810
	C67	5.451427044	0.732151552	5.901489502
	C68	5.183120041	-1.360951586	4.736846691
	C69	5.976601717	1.316122013	4.743979189
	C70	5.719280502	-0.796178587	3.579098963
	C71	6.104644991	0.546213085	3.580963834
	C72	7.331691332	-4.706391143	0.638235900
	C73	7.911011624	-4.505274349	-0.773113865
	C74	6.078322924	-3.855075942	0.796612133
	C75	8.397189279	-5.555426975	-2.954327034
	C76	7.523802017	-4.869182455	-4.007645708
	C77	5.338979448	-3.759709286	-4.426634006
	C78	5.384331847	-2.251836798	-4.064744135
	C79	3.934404199	-4.366213340	-4.197109093
	C80	2.721595741	-3.696668390	-4.853120715
	C81	2.523915731	-3.948100920	-6.358033878
	C82	3.843385053	-2.973142983	-8.247678189
	C83	5.451123874	-0.520173497	-2.337609083
	C84	6.794390066	0.151437498	-2.652815240
	C85	5.174033100	-0.315964344	-0.832894603
	C86	9.215449488	-0.099100866	-2.425853978
	C87	10.059782665	-0.183775187	-3.687023215
	C88	10.164837413	-0.900723948	-6.009673333
	C89	10.468157181	0.269507857	-6.977239104
	C90	9.599432531	-2.097185385	-6.782965555
	C91	8.265824908	-1.812567521	-7.459594528
	C92	9.886295529	2.541170458	-7.702729244
	C93	11.233100481	3.245569335	-7.665944778
	C94	8.750503694	3.557968058	-7.386317314
	C95	7.372063244	2.946968859	-7.515242969
	C96	6.701903497	2.955266868	-8.749293759
	C97	6.742211981	2.313290077	-6.430417584
	C98	5.459468157	2.336180158	-8.906005491
	C99	5.512481663	1.664459133	-6.574226309
	C100	4.883389747	1.679311268	-7.817165199
	C101	-5.084091088	-9.164396162	1.311380734
	C102	-5.209390148	-8.077374736	0.258835152
	C103	-3.946624738	-6.381069986	-1.043642827
	C104	-3.104217449	-6.754944324	-2.269881671
	C105	-0.829350024	-7.248654645	-3.055748697
	C106	0.379380446	-8.077525530	-2.632989957
	C107	1.918707505	-8.822629969	-0.897393400

	C108	1.781376460	-10.331528783	-0.942448643
	C109	-7.058190531	-5.820081646	-4.309520446
	C110	-6.329957511	-5.140055151	-5.474079395
	C111	-7.078204874	-4.890562871	-3.076703127
	C112	-8.122345611	-5.359754607	-2.046261871
	C113	-7.947024617	-4.627131645	-0.735091050
	C114	-4.201640152	-4.662085150	-6.571947766
	C115	-3.104986163	-5.605659893	-7.023868729
	C116	-3.743535237	-3.200730192	-6.339744602
	C117	-3.028765125	-3.115830179	-5.025778626
	C118	-1.673586350	-3.468000353	-4.930143652
	C119	-3.770486803	-2.912428036	-3.848165610
	C120	-1.106419398	-3.727637382	-3.680556196
	C121	-3.202230907	-3.176414725	-2.602605055
	C122	-1.886937782	-3.645703759	-2.527930458
	C123	-4.535684298	-7.363231925	7.127432016
	C124	-3.903221763	-7.132215293	8.507316020
	C125	-5.191990629	-6.085005651	6.584780885
	C126	-5.621889873	-6.121830010	5.152855413
	C127	-6.652417589	-6.846852997	4.591352963
	C128	-5.807996852	-5.477155402	3.068588223
	C129	-4.003835231	-6.136531884	10.713300062
	C130	-3.575945319	-7.412805157	11.403031797
	C131	-5.971656656	0.289275541	3.474792320
	C132	-5.513424958	-0.159666545	4.877381059
	C133	-5.780450618	-1.649961424	5.218075797
	C134	-7.268358504	-1.880824127	5.573735759
	C135	-7.599123443	-3.293033239	6.064520853
	C136	-9.046821497	-3.455040676	6.579232097
	C137	-4.915459439	-2.068577531	6.415425497
	C138	-0.203881024	-3.502454118	2.300097110
	Fe139	-1.757685143	-4.671547943	2.334374866
	Fe140	0.828593088	-2.002451648	0.782938318
	Fe141	1.621558637	-3.636739426	3.128328001
	Fe142	0.511388037	-4.995175302	1.235727498
	Fe143	3.050144174	-4.153839744	0.994281056
	Fe144	-0.658326657	-3.497783470	4.287314027
	Fe145	-1.339920388	-1.908219395	2.110976617
	H146	-8.833282139	4.567713422	-1.573387799
	H147	-8.453042266	3.504808285	-2.750830731
	H148	-6.941313226	6.838031764	-4.901182414
	H149	-8.558537437	5.208595734	-4.437383805
	H150	-9.044396955	6.359042189	-3.196280582
	H151	-4.395644403	6.067032947	-3.987329406
	H152	-6.460152620	8.547381976	-2.557957595
	H153	-4.753516535	7.514608059	-4.948451774
	H154	-6.053053583	9.949140287	-0.748819999
	H155	-5.880432229	6.906196665	-0.470984351
	H156	-4.345635115	10.043267722	-1.233804502
	H157	-1.399756371	4.370074189	-0.435084246
	H158	-3.841494844	5.719117671	-1.010068616
	H159	-5.456281748	5.458300587	1.308520100
	H160	0.926991458	8.379088285	-0.695640800
	H161	-1.898462685	7.910037001	-1.129022404
	H162	-4.779108063	6.794424415	2.270620051
	H163	-1.804506750	5.264825486	-1.908331726
	H164	-1.952873922	9.165299701	1.368327010
	H165	-0.344022960	9.625097347	-0.859615243
	H166	-1.451833897	3.691207082	1.539169209
	H167	-1.038121479	2.309086754	0.490064315
	H168	-0.979595240	2.163954780	2.272946323
	H169	0.793120892	4.086843034	0.724944476
	H170	-1.976230173	10.179181796	3.454790538
	H171	3.555200803	4.477441710	-0.544196588
	H172	2.781517481	7.739583929	2.405784580
	H173	6.037699199	7.691983724	1.047043033
	H174	1.350872617	1.816395672	-0.031076420
	H175	1.569613932	1.251685438	1.633217611
	H176	6.218644182	-1.024821233	8.412217578
	H177	4.741395222	5.319688591	-1.555178373
	H178	6.202652979	7.106267890	-0.625192627
	H179	5.294387981	4.193019701	-0.293432616
	H180	6.779920836	6.106416214	0.730480114
	H181	3.850736863	6.869956230	0.185188595
	H182	4.961262049	4.807111553	2.167934236
	H183	2.217514216	5.482740571	1.329341646
	H184	2.673388453	2.474745459	0.944419730
	H185	4.382196357	-1.008501560	-0.512352122
	H186	4.806435451	0.714131343	-0.712006570
	H187	6.322463558	0.077691066	0.718889418

H188	6.781116289	2.016410888	2.509582935
H189	5.802674777	-1.381385539	2.663903334
H190	4.842285226	-2.398024406	4.708362723
H191	5.347713315	1.342048204	6.802472835
H192	-0.199088890	5.494258510	2.499882082
H193	-2.257774724	5.733379287	4.822902752
H194	-1.023431062	6.558995948	5.801856667
H195	-1.753143273	7.200112471	3.002533479
H196	-0.209286411	10.383152663	3.390886785
H197	6.266159409	2.370116852	4.741368984
H198	0.918266493	2.971715759	3.575659319
H199	4.634072071	9.480714269	3.497688134
H200	2.902899098	9.437920997	3.915343076
H201	3.723600935	7.497359819	5.667085602
H202	-1.351343640	-1.383458613	13.774243668
H203	-7.263925906	-1.758642323	-0.289230349
H204	-5.527025093	-1.778354806	3.255167401
H205	-9.130801368	-5.145691255	-2.428198555
H206	-8.038513218	-6.444425458	-1.885801223
H207	-7.314433370	-3.858739659	-3.379703301
H208	-3.289121596	-6.678597805	-6.779197444
H209	-8.094250567	-5.903735058	-4.673072416
H210	-3.877306040	0.147056510	-2.564279903
H211	-6.132608689	1.776860968	-1.263625158
H212	-4.484986777	-5.757599490	-4.747627209
H213	-4.893122892	-4.639211893	-7.440603282
H214	-5.650633523	-7.192935663	-3.641102074
H215	-6.360206889	-0.621280912	0.708506544
H216	-6.072201167	-4.865447245	-2.631181575
H217	-7.211401726	-7.691416921	-3.480834586
H218	-6.286185866	1.531605722	-4.936858839
H219	-3.520389473	1.707820055	-1.835351474
H220	-6.935658977	-6.178398399	-0.080743394
H221	-4.986246238	0.936085648	-0.236212362
H222	2.006016738	4.351642597	-2.925812576
H223	-0.060900888	2.903287890	-1.996354625
H224	-0.809081696	7.408533331	-5.186696934
H225	-2.193233637	4.755175447	-5.199178950
H226	1.990456471	5.090398332	-6.360027051
H227	6.302937117	6.997289754	-8.701222380
H228	3.902941827	7.511506007	-5.650201727
H229	2.820192124	6.873221685	-8.131248526
H230	4.790493383	4.846160758	-6.905688165
H231	5.081698212	5.426901427	-12.443631770
H232	3.026280906	2.325250448	-3.965915450
H233	1.958686876	2.475953496	-5.365894580
H234	6.951821868	6.582069020	-11.136901303
H235	2.700045779	5.142513246	-8.447296756
H236	0.102431900	4.169018236	-5.161687447
H237	-1.702436992	6.774165245	-3.804199253
H238	-1.818182502	4.772292691	-8.713487520
H239	-0.529203042	-1.078542711	-3.264610710
H240	-3.430018263	5.211682836	-8.121839682
H241	7.082875095	-3.195868645	7.937450397
H242	6.520277470	-2.675706983	6.492809403
H243	5.224368266	-2.644170533	-2.013057294
H244	4.686356063	0.029956798	-2.904456362
H245	7.747593484	-1.583673445	-2.114785391
H246	1.255272067	0.026543976	-4.784127613
H247	0.967026890	-0.125254392	-6.986579449
H248	-0.705711834	-0.420603075	-8.445273910
H249	2.476414001	0.349060265	-6.922559308
H250	-2.067367579	-0.644273971	-6.441136320
H251	-2.677872780	0.375650526	-7.786004131
H252	9.543424142	-2.971803948	-6.119821229
H253	10.321971042	-2.326370919	-7.581281971
H254	7.438648601	-3.475416696	-6.548311946
H255	6.376795629	-2.495249182	-7.556697502
H256	4.975831009	-4.248606762	-9.384776607
H257	1.452450532	-4.058889373	-6.582557311
H258	4.948307381	2.363475366	-9.872302860
H259	7.199175638	2.322088508	-5.437364953
H260	4.167445696	-0.941546584	-8.410062974
H261	3.752057177	-4.369686140	-3.110143515
H262	5.999545335	-4.740427623	-2.652524754
H263	2.708184469	-2.613844554	-4.651752365
H264	-3.565736795	3.176022390	-7.020993397
H265	8.576842147	-6.586260823	-3.288072351
H266	-3.897507708	0.725575057	-5.520984317
H267	3.974720751	-5.424569537	-4.503886191

	H268	5.072140220	1.129075456	-5.732896076
	H269	9.361491924	-5.027374530	-2.959584823
	H270	3.774692429	-5.024294210	-8.411889695
	H271	4.724236964	-1.895365274	-9.746481534
	H272	-6.071098557	0.282493145	-3.846640365
	H273	-5.257755061	2.781414546	-3.292855934
	H274	7.153228647	3.455784667	-9.608881653
	H275	-1.101993319	1.344413871	-5.335509180
	H276	5.638020196	-3.836587571	-5.475597359
	H277	3.003874177	-4.894735224	-6.639396442
	H278	1.839517294	-4.105571469	-4.342966151
	H279	2.664964075	-1.912339828	-6.972773743
	H280	9.143133484	0.962685471	-2.145209295
	H281	8.433491289	-0.864232045	-4.723907051
	H282	11.163222070	-1.196033308	-5.651177775
	H283	9.768836647	-0.620852243	-1.633412108
	H284	9.104739775	1.441678352	-6.036362631
	H285	-4.104434261	-9.658088301	1.227673408
	H286	-5.096880698	-8.640086760	2.288790175
	H287	-3.697149483	-7.646855263	6.473504080
	H288	-6.223786736	-10.706591200	2.008287508
	H289	-7.042018312	-9.656362491	1.054167667
	H290	5.695624632	-4.000341035	1.817088142
	H291	8.630730892	-3.380745293	1.484304686
	H292	7.448521214	-6.450204708	-1.260599782
	H293	6.323926703	-2.792503967	0.654523507
	H294	9.125002153	-4.934439804	1.615208071
	H295	2.125821445	-8.536714430	0.146354162
	H296	7.065374961	-5.765283952	0.784127606
	H297	-2.779385592	2.298013618	9.309712336
	H298	-3.786963352	-0.449799972	10.226940344
	H299	0.188377395	-7.613558917	-0.603846048
	H300	2.637894056	0.188509491	5.159298240
	H301	-1.356970370	-7.811647983	-3.835454792
	H302	-3.782092260	-3.022616285	-1.690004900
	H303	-0.433656440	-6.337267160	-3.537126272
	H304	-1.092066503	2.145370795	7.209909432
	H305	-1.460434105	-6.582431732	-1.086668048
	H306	-2.224002386	0.971605422	6.525785172
	H307	-3.767325201	1.150375208	8.376267196
	H308	-1.837525722	-0.530533048	8.523895540
	H309	-0.838960305	0.736650524	9.259694062
	H310	-4.636804776	-2.559802492	-6.354394286
	H311	-4.702174175	2.306549408	10.968875551
	H312	-7.705005485	1.699939309	10.820548608
	H313	-1.448423944	-3.909306956	-1.570424828
	H314	-6.213069966	3.172097561	12.849674830
	H315	-7.227463439	3.728533443	11.690716392
	H316	-0.483716085	-0.651938358	6.339007891
	H317	-0.056878481	-4.010512407	-3.588001293
	H318	-4.807896870	-2.576133182	-3.913556493
	H319	-6.884361968	-4.042567200	9.844613397
	H320	-6.080104487	-5.850856989	7.196660187
	H321	-5.144038074	-2.422825298	8.949221087
	H322	-8.112682375	-4.486175584	8.987230855
	H323	0.730344186	-10.706755383	-0.991361470
	H324	-2.557053169	-2.929089552	7.296254394
	H325	-5.973639337	0.483166411	5.637323303
	H326	-3.193126198	-7.738437477	0.392710805
	H327	-7.517192858	-1.154314297	6.362993062
	H328	-7.457428761	-6.714647238	2.611358715
	H329	-5.683320948	-4.954310646	2.128247846
	H330	-7.478240611	1.231367941	12.510108027
	H331	1.487757491	-1.092198955	5.358560913
	H332	-2.161698453	-2.970848542	9.636795814
	H333	-1.077928316	-3.407861695	7.347432876
	H334	-2.216117796	-1.401257721	11.416952289
	H335	0.655570213	2.779561621	6.394687011
	H336	-7.456933306	-4.006851970	5.237784841
	H337	-6.911398064	-3.588951589	6.868987751
	H338	-7.883578679	-1.629660173	4.695901655
	H339	-3.511344232	-5.512675515	-0.524970454
	H340	-1.086246862	-3.592173848	-5.842251054
	H341	-3.100393190	-2.920859644	-7.188198762
	H342	-4.424950112	0.019417671	4.906120780
	H343	-3.544049175	-2.960710118	10.349128164
	H344	-5.559760345	-2.046996757	10.388229346
	H345	2.100340849	2.357008421	5.657563333
	H346	-7.319202571	-7.586798275	5.019134968
	H347	-4.499383854	-5.244270758	6.716289333

H348	-4.952470769	-6.131305611	-1.385429832
H349	2.785522637	-8.520938770	-1.504718471
H350	3.910680944	-0.481409865	7.749416037
H351	3.749037958	-2.034838247	6.893813829
H352	-4.997855628	-9.331639019	7.473939227
H353	-6.226459387	-8.307431096	7.825364782
H354	-4.774380199	-5.667106073	11.345823381
H355	-5.495263663	-5.952898096	9.199249615
H356	-4.159173089	-8.319597188	11.113706059
H357	-3.152662411	-5.439407115	10.659710155
H358	4.357542090	-3.365414255	9.187063424
H359	11.801404367	3.126156713	-6.712729370
H360	-0.978461115	0.344139532	13.536346201
H361	0.633413017	-0.698336438	11.537540218
H362	9.741072380	2.142778620	-8.719701306
H363	8.900324846	3.955870215	-6.370294059
H364	8.863360714	4.397367501	-8.087751900
H365	-3.167040154	0.028359318	1.992023549
H366	3.238973094	1.272595067	-8.779956483
H367	-1.705570286	0.466697855	1.284200857
H368	0.706992127	-0.708038739	3.033169147
H369	-1.831990548	0.412184492	2.916364357
H370	-1.281847443	-0.514236139	-0.490210548
Mo371	-3.460050847	-3.593982491	4.197599109
N372	0.235931645	-0.915843582	2.140463216
N373	-2.160109018	-0.022438241	2.052004761
N374	-8.975547624	4.364582449	-2.565922956
N375	-6.421985252	6.655638545	-4.046237648
N376	-5.502910643	8.580673883	-2.216381691
N377	-5.289453122	7.266388889	0.281183092
N378	-3.092515269	5.620792869	-0.329785207
N379	-1.045659969	7.682439232	-0.612723430
N380	-1.050075930	9.245495955	1.843888914
N381	-1.262603624	7.321897666	3.890416856
N382	0.149452410	4.876478006	3.232534896
N383	2.912828171	5.115393272	1.980052390
N384	3.581378879	7.693878805	3.038013890
N385	-6.483214570	3.052891493	11.870727299
N386	-4.723998834	1.290455190	10.833324449
N387	-0.248402167	0.319170522	6.566157047
N388	1.342545495	2.034399395	6.261707532
N389	1.667153567	-0.069715351	5.327436640
N390	-1.952290836	-0.531637748	11.932113077
N391	-5.583358529	0.979355652	-4.439699719
N392	-6.674529895	-0.922521930	-0.229450548
N393	-3.195106431	1.474739826	-5.405969323
N394	-2.628946580	3.512134322	-7.232625990
N395	-1.777757498	5.620318695	-5.570439366
N396	0.650761817	4.698749027	-4.479344091
N397	0.781873589	0.477354392	-3.987658228
N398	-0.633922670	0.851013831	-2.313724453
N399	2.926226205	5.163097750	-5.968647405
N400	3.996444818	5.502302770	-10.582791485
N401	6.051451608	6.325222518	-10.741228065
N402	6.225914323	-2.961795134	7.430575994
N403	8.300167874	-4.331090063	1.681153843
N404	7.863773407	-5.588015366	-1.604329962
N405	6.276513216	-4.535868882	-3.620059966
N406	3.045662582	-2.857842611	-7.188703376
N407	4.156271048	-4.165299683	-8.791194354
N408	4.406590915	-1.864946626	-8.781718792
N409	5.411769952	-1.928504475	-2.740701283
N410	7.884002371	-0.660899000	-2.535576509
N411	9.399971640	-0.553324082	-4.827283041
N412	7.319164967	-2.777545539	-7.294108503
N413	9.843875054	1.449130282	-6.735124264
N414	-6.152642309	-10.155691965	1.150106680
N415	-4.051207757	-7.494089346	-0.109913221
N416	-1.785381105	-6.896932963	-2.020381083
N417	0.711333843	-8.131204262	-1.317425793
N418	-6.570173880	-7.189395964	-4.098750151
N419	-4.972135330	-5.255581507	-5.496733371
N420	-5.483908210	-8.492722070	7.143514172
N421	-5.097955218	-5.274618272	4.177668496
N422	-6.750879194	-6.426123656	3.282781974
N423	-4.597359283	-6.393202071	9.413622425
N424	2.135619786	-0.352640760	-1.258430371
N425	1.652680751	-0.948840734	-0.435705670
O426	-6.351734213	5.478183777	-2.094992520
O427	-3.368789831	7.835475940	-2.488137081

O428	-3.948385802	8.964278723	1.008897436
O429	-2.493412926	5.744358870	1.864305926
O430	0.387105004	6.066273488	0.077385822
O431	1.225281526	9.119929883	1.688702439
O432	-0.369213512	8.822679340	5.385776112
O433	0.045767853	4.321418297	5.453610260
O434	3.294453797	3.709867830	3.753081521
O435	5.539458393	6.732521435	3.664158762
O436	4.816862596	9.176985109	6.056140950
O437	-6.108456764	-0.520773710	11.221092707
O438	-2.214675117	1.744055322	12.119890556
O439	1.228147630	-1.472952264	13.328909948
O440	-2.824377964	3.287984874	-4.040690514
O441	-6.615111183	-0.800351019	-2.499138027
O442	-0.396253525	3.156662913	-6.927599738
O443	-0.600387111	0.187717608	-7.692099539
O444	-1.313707037	6.898627694	-7.421867293
O445	0.896830528	6.629369314	-3.281783328
O446	4.225900627	4.902987662	-4.115797021
O447	5.809154512	7.189684779	-6.307104425
O448	4.769827625	-1.767226802	10.389934770
O449	6.594598675	1.066386054	2.397443906
O450	8.394454020	-3.420091253	-1.138534071
O451	7.983100949	-4.668355498	-5.149769268
O452	5.400585555	-1.398324882	-4.966443864
O453	6.862107191	1.355135433	-2.930948075
O454	6.358760377	-0.521951287	-0.053783860
O455	11.268850494	0.078542018	-3.658861684
O456	11.285621546	0.119651810	-7.896714561
O457	8.080203658	-0.796582367	-8.144243200
O458	11.657537113	3.920683810	-8.590850788
O459	3.671852790	0.976675770	-7.955743416
O460	-6.333998632	-7.740044799	-0.185950221
O461	-3.618454257	-6.936139986	-3.393499893
O462	1.055601415	-8.644734914	-3.507542270
O463	2.733400086	-11.093462477	-0.900919236
O464	-6.952840494	-4.502232206	-6.339637246
O465	-8.377789325	-3.498735729	-0.527020461
O466	-7.228566748	-5.263094320	0.215106823
O467	-2.114711661	-5.262225491	-7.652820159
O468	-2.810270686	-7.649765230	8.812200347
O469	-2.717028941	-7.458937352	12.273496322
O470	-5.840073610	-0.604856939	2.532336480
O471	-6.364003134	1.457661689	3.300155667
O472	-9.205496237	-4.061694628	7.697060031
O473	-9.976443088	-3.004127518	5.852850566
O474	-4.050754401	-3.030169523	6.225824435
O475	-5.077686869	-1.495733306	7.510019004
O476	-5.388585981	-2.452154811	4.080970566
O477	-2.565812142	-2.927368413	10.538947624
O478	-1.724890696	-2.859514775	7.828754907
O479	1.849494739	-0.326062375	-6.577779189
O480	-7.570035112	-4.744880736	9.792262957
O481	-5.245293585	-2.821273376	9.857361971
S482	4.763701944	-4.314833443	-0.414900202
S483	1.429824846	-3.921144555	-0.507980602
S484	-3.410414088	-3.271363772	1.841774188
S485	2.857977142	-2.180623684	1.990816634
S486	-1.224296338	-1.840714950	-0.162020936
S487	-1.146079220	-6.450200531	1.128357249
S488	-1.968488888	-1.709980246	4.415769807
S489	2.322848183	-5.671794283	2.433936651
S490	-1.902112591	-5.339701065	4.500300939
S491	1.267060861	-3.353086419	5.310576913
end			

TS

	bm612bh135nh3distd_1_53445.11			
Fe(139) -2.364	C1	-6.973883209	5.834401218	-3.107166686
Fe(140) -0.030	C2	-8.434341805	5.447640048	-3.368925445
Fe(141) -2.524	C3	-4.999275373	6.979742642	-4.022960064
Fe(142) 2.469	C4	-4.553815389	7.838753678	-2.845243904
Fe(143) 1.402	C5	-5.168935857	9.349268084	-1.029561771
Fe(144) 2.252	C6	-4.739403929	8.503363855	0.174126754
Fe(145) 0.582	C7	-4.777203688	6.325099611	1.277084526
	C8	-3.344836357	5.864701725	0.973560852
	C9	-1.738519889	5.331308596	-0.814608568
	C10	-0.699340623	6.387018250	-0.408952979
	C11	-0.046876226	8.707365771	-0.332881686
	C12	0.112610010	9.025678205	1.158990202

	C13	-1.019795312	9.664708937	3.231912341
	C14	-0.832838870	8.553962189	4.270549562
	C15	-1.251832252	6.190193958	4.796342788
	C16	-0.269119858	5.057381091	4.509497010
	C17	1.050203111	3.772237416	2.839038039
	C18	2.529827805	4.198835233	2.917697920
	C19	0.685833548	3.242822986	1.438378241
	C20	1.632184059	2.130248034	0.987775841
	C21	-0.776015935	2.804895605	1.433247437
	C22	4.286493867	5.624556053	1.903134724
	C23	4.532316686	6.719097810	2.949843505
	C24	4.617678241	6.112067595	0.467976822
	C25	5.992005522	6.792806602	0.408289521
	C26	4.548352151	4.954633709	-0.534235173
	C27	3.834075237	8.839244081	3.889572985
	C28	4.172957970	8.453660241	5.314420088
	C29	-6.965729287	1.683064617	11.645164528
	C30	-5.870699605	0.702083266	11.215465241
	C31	-3.502238296	0.590616814	10.418614062
	C32	-2.482169079	0.656706342	11.572482299
	C33	-2.954181000	1.223129708	9.123497230
	C34	-1.682617100	0.556178260	8.583569012
	C35	-1.333975578	1.076358122	7.182844325
	C36	0.912672988	0.769207301	6.051964779
	C37	-1.009122767	-0.642503903	13.035824058
	C38	0.400842126	-0.971757105	12.595660570
	C39	-4.747943414	1.839526861	-3.589000548
	C40	-3.487218123	2.282706336	-4.356289331
	C41	-4.318009184	1.112361656	-2.295698265
	C42	-5.428544649	0.916135067	-1.241915154
	C43	-6.274073729	-0.350806386	-1.380160737
	C44	-1.895990043	1.463673984	-6.077239897
	C45	-1.567240762	2.779025479	-6.792360966
	C46	-1.872904304	0.261631049	-7.033956612
	C47	-2.439761987	4.824191877	-7.815640031
	C48	-1.780351433	5.870314905	-6.908453134
	C49	-1.053039268	6.478930311	-4.651410438
	C50	0.256837979	5.934066179	-4.080206984
	C51	1.920746815	4.139872882	-4.001192395
	C52	3.138648648	4.796813300	-4.677679175
	C53	2.022326661	2.631208144	-4.275983582
	C54	0.994147318	1.785293857	-3.600471104
	C55	0.101460423	1.992108097	-2.568326335
	C56	-0.192667175	-0.046038975	-3.189078779
	C57	4.022842152	5.619541021	-6.818011401
	C58	4.646788835	6.866219415	-6.195943923
	C59	3.422544481	5.941413075	-8.206249199
	C60	4.384833739	6.011610230	-9.353825326
	C61	5.662334067	6.530290529	-9.437846226
	C62	5.036043388	5.701460514	-11.395299836
	C63	5.555344200	-1.848856545	8.092228054
	C64	4.847669810	-2.372179526	9.327147357
	C65	4.467290478	-1.241181423	7.167294132
	C66	5.044997656	-0.616580105	5.919192042
	C67	5.459099044	0.728018583	5.901375540
	C68	5.191243360	-1.365440420	4.737364489
	C69	5.983823740	1.311857242	4.743598734
	C70	5.727096149	-0.800797623	3.579397924
	C71	6.112126467	0.541670688	3.580819222
	C72	7.336914766	-4.711000456	0.639129899
	C73	7.918725359	-4.510159838	-0.772885466
	C74	6.084784753	-3.860752410	0.797188005
	C75	8.405748821	-5.559793419	-2.955027733
	C76	7.532095144	-4.873692683	-4.008594895
	C77	5.346172313	-3.763626430	-4.425784899
	C78	5.391932610	-2.255954601	-4.065149403
	C79	3.942431592	-4.370733885	-4.197551408
	C80	2.729612775	-3.701379441	-4.853743277
	C81	2.531968369	-3.953049119	-6.358561588
	C82	3.851374520	-2.978084926	-8.247611818
	C83	5.458884481	-0.524124750	-2.337394829
	C84	6.802144313	0.147393338	-2.653087522
	C85	5.182392744	-0.320940552	-0.832034061
	C86	9.223236850	-0.102618089	-2.426438753
	C87	10.068202920	-0.187535402	-3.687052988
	C88	10.172396147	-0.904559455	-6.009365985
	C89	10.476437197	0.265358313	-6.978228669
	C90	9.607860415	-2.101228090	-6.782609289
	C91	8.274570823	-1.816882162	-7.459772374
	C92	9.894493224	2.537288626	-7.703559233

C93	11.241625219	3.242031438	-7.667260549
C94	8.758784888	3.553823860	-7.386713946
C95	7.380435444	2.942618440	-7.515505771
C96	6.710483251	2.950765125	-8.749661493
C97	6.750346944	2.309314309	-6.430597711
C98	5.468042368	2.331820885	-8.906439103
C99	5.520472344	1.660758150	-6.574451073
C100	4.891740385	1.675268261	-7.817577623
C101	-5.075003665	-9.166914979	1.313033191
C102	-5.201319717	-8.080562180	0.259737222
C103	-3.938693099	-6.385733800	-1.045148923
C104	-3.097137305	-6.760927321	-2.271218855
C105	-0.822058707	-7.254794205	-3.056723208
C106	0.386814622	-8.082972536	-2.632587671
C107	1.924837636	-8.824251485	-0.893763075
C108	1.788740478	-10.333731245	-0.943424858
C109	-7.050946383	-5.824549452	-4.309734701
C110	-6.321829379	-5.144275269	-5.473390182
C111	-7.070142131	-4.894926514	-3.076136508
C112	-8.114658225	-5.363882493	-2.046002382
C113	-7.939323598	-4.631170006	-0.734821064
C114	-4.194139387	-4.666829357	-6.572516419
C115	-3.097283205	-5.610423108	-7.023816085
C116	-3.735935800	-3.205197594	-6.340200536
C117	-3.020322003	-3.120567916	-5.026436166
C118	-1.665214420	-3.473281352	-4.931598259
C119	-3.761242374	-2.918267022	-3.848116945
C120	-1.097382101	-3.733359083	-3.682138771
C121	-3.192244617	-3.182535544	-2.602792977
C122	-1.876093550	-3.649543894	-2.527919682
C123	-4.529151681	-7.367377507	7.126787317
C124	-3.896266877	-7.136644387	8.506492547
C125	-5.184073760	-6.088257277	6.584291615
C126	-5.611221019	-6.122336400	5.151533038
C127	-6.641814743	-6.847390162	4.589639150
C128	-5.795436579	-5.477029495	3.068314119
C129	-3.995492536	-6.140535307	10.712479677
C130	-3.568135632	-7.416795822	11.402355590
C131	-5.976182810	0.288588451	3.483801271
C132	-5.509716783	-0.161669382	4.881383453
C133	-5.773353523	-1.652042304	5.220793826
C134	-7.260641914	-1.885203338	5.574278023
C135	-7.590279742	-3.297313424	6.064887171
C136	-9.037605864	-3.458843869	6.579481477
C137	-4.906387556	-2.069194051	6.416691236
C138	-0.198112921	-3.501737389	2.299593524
Fe139	-1.770351032	-4.695307173	2.318163000
Fe140	0.830977289	-1.994448352	0.793249505
Fe141	1.622311255	-3.642442322	3.128898726
Fe142	0.519970752	-4.981995414	1.234145282
Fe143	3.049582901	-4.166162938	0.991837985
Fe144	-0.665063550	-3.469066949	4.252654745
Fe145	-1.361481346	-1.878605352	2.141388273
H146	-8.824215804	4.563258976	-1.572095261
H147	-8.443978948	3.500580968	-2.749712292
H148	-6.934578514	6.835115336	-4.903331295
H149	-8.551461863	5.203463432	-4.436800819
H150	-9.039608780	6.354459884	-3.196024389
H151	-4.388546560	6.064721010	-3.988304993
H152	-6.454125324	8.542573276	-2.557342754
H153	-4.747152151	7.512186524	-4.949358691
H154	-6.046869251	9.945468659	-0.748991001
H155	-5.871091115	6.901692989	-0.472212421
H156	-4.339588631	10.039921039	-1.234386100
H157	-1.389118121	4.367489167	-0.433658271
H158	-3.830172462	5.719435373	-1.011215972
H159	-5.446225445	5.454088580	1.307438549
H160	0.934414237	8.380734157	-0.699238141
H161	-1.889896831	7.907471689	-1.132060372
H162	-4.769817956	6.790695057	2.269595746
H163	-1.793853246	5.261992849	-1.908510730
H164	-1.945019168	9.161627245	1.367126940
H165	-0.338793427	9.624914286	-0.860753748
H166	-1.446656547	3.665705514	1.553677712
H167	-1.026526837	2.286218883	0.495917264
H168	-0.966265170	2.109265924	2.259211442
H169	0.791192669	4.076303894	0.728409258
H170	-1.968384883	10.173769185	3.454330971
H171	3.562179370	4.469867731	-0.541309821
H172	2.787833593	7.735120496	2.410191243

	H173	6.044136261	7.686597575	1.046860107
	H174	1.357485745	1.811961244	-0.025679870
	H175	1.569495773	1.245228715	1.636935528
	H176	6.226458227	-1.029296411	8.412664537
	H177	4.745843158	5.314388386	-1.553619686
	H178	6.207899514	7.100291256	-0.625430808
	H179	5.302225622	4.188074317	-0.293315351
	H180	6.786262121	6.100991192	0.730266085
	H181	3.856553462	6.863739447	0.186618638
	H182	4.968337650	4.803744178	2.170948853
	H183	2.225464398	5.473801413	1.327657287
	H184	2.679465433	2.467468254	0.954263521
	H185	4.390234758	-1.013454559	-0.512844984
	H186	4.814269990	0.708553548	-0.711774220
	H187	6.331171174	0.072568266	0.718512322
	H188	6.787395184	2.011773659	2.508545624
	H189	5.810318373	-1.386187540	2.664342926
	H190	4.850658374	-2.402600677	4.709181787
	H191	5.354806982	1.338271980	6.802032324
	H192	-0.195104571	5.490521402	2.502900554
	H193	-2.252684502	5.731218324	4.822016571
	H194	-1.018764216	6.556281663	5.802865303
	H195	-1.743891874	7.195819051	3.001772235
	H196	-0.201649605	10.379746946	3.389461397
	H197	6.272545893	2.366067470	4.740620326
	H198	0.925166927	2.971971816	3.578340045
	H199	4.641651552	9.481609418	3.500011617
	H200	2.909313808	9.436069679	3.917202026
	H201	3.730592997	7.492731097	5.664858367
	H202	-1.343591658	-1.387792185	13.774275653
	H203	-7.256695219	-1.764078568	-0.289430506
	H204	-5.514803788	-1.789641010	3.260422269
	H205	-9.122896554	-5.149635054	-2.428418336
	H206	-8.031147170	-6.448625038	-1.885658570
	H207	-7.305933149	-3.862861246	-3.378740109
	H208	-3.281170788	-6.683247403	-6.778435221
	H209	-8.086773651	-5.907553758	-4.672576789
	H210	-3.870085155	0.143248441	-2.566157259
	H211	-6.125436017	1.772478412	-1.264745976
	H212	-4.476714109	-5.762447004	-4.747484073
	H213	-4.885798272	-4.644312167	-7.440498205
	H214	-5.643097951	-7.197794358	-3.640725602
	H215	-6.354680704	-0.625859551	0.707165985
	H216	-6.064298924	-4.869811379	-2.629994832
	H217	-7.204063948	-7.696169273	-3.481297924
	H218	-6.280523830	1.526783826	-4.938294652
	H219	-3.512088081	1.701832716	-1.837052737
	H220	-6.928050385	-6.182282342	-0.080592831
	H221	-4.979282317	0.931906620	-0.238683665
	H222	2.014554628	4.347739666	-2.926696251
	H223	-0.055447354	2.903102198	-1.999955983
	H224	-0.801037302	7.403787627	-5.188515739
	H225	-2.185637082	4.750673390	-5.199932954
	H226	1.999576850	5.086624158	-6.360993231
	H227	6.310826541	6.993494213	-8.701684504
	H228	3.912569315	7.507446743	-5.650419474
	H229	2.827563093	6.868996050	-8.132794414
	H230	4.799042524	4.843284043	-6.907648768
	H231	5.090466537	5.422865336	-12.444394138
	H232	3.034780755	2.321249693	-3.965737184
	H233	1.967511970	2.471813845	-5.366082441
	H234	6.960154616	6.578760305	-11.137379141
	H235	2.708911997	5.137841558	-8.447869167
	H236	0.110230337	4.163668932	-5.160919267
	H237	-1.694313294	6.770033282	-3.805605883
	H238	-1.809209851	4.766645704	-8.713888412
	H239	-0.527524910	-1.078008294	-3.265856076
	H240	-3.421623014	5.206310387	-8.124003717
	H241	7.091015417	-3.199677612	7.937334954
	H242	6.528369553	-2.679091994	6.492914951
	H243	5.233343290	-2.648043273	-2.014661754
	H244	4.693539764	0.025643818	-2.903601959
	H245	7.755840399	-1.587478388	-2.115505406
	H246	1.262521714	0.021817479	-4.782191734
	H247	0.974412006	-0.129277377	-6.986554399
	H248	-0.698464157	-0.426767038	-8.444132218
	H249	2.483880122	0.345505147	-6.923487975
	H250	-2.060380157	-0.648611603	-6.440702473
	H251	-2.670556092	0.371644824	-7.785522708
	H252	9.551933095	-2.975575259	-6.119153358

	H253	10.330548944	-2.330828736	-7.580755632
	H254	7.447913974	-3.480856657	-6.550194796
	H255	6.386226699	-2.501113142	-7.559303267
	H256	4.982411970	-4.253310722	-9.386453733
	H257	1.460479300	-4.063441053	-6.583062957
	H258	4.956876133	2.358863990	-9.872726656
	H259	7.207080755	2.318316433	-5.437437266
	H260	4.176492668	-0.946452130	-8.409417936
	H261	3.760675355	-4.374976743	-3.110625593
	H262	6.008935597	-4.745224920	-2.653080822
	H263	2.716027181	-2.618440500	-4.652804355
	H264	-3.558042445	3.171708691	-7.020688983
	H265	8.585544801	-6.590476588	-3.289002382
	H266	-3.888146933	0.719975727	-5.519244155
	H267	3.983695371	-5.428836472	-4.504844313
	H268	5.079514794	1.126122974	-5.732972081
	H269	9.370052227	-5.031578081	-2.960837765
	H270	3.780825591	-5.029124701	-8.414341812
	H271	4.731246870	-1.899912949	-9.746638369
	H272	-6.063866213	0.279022291	-3.846284824
	H273	-5.252134794	2.779081867	-3.292770690
	H274	7.161939230	3.451051437	-9.609310863
	H275	-1.093491746	1.339763852	-5.335342963
	H276	5.645436056	-3.839918046	-5.474862952
	H277	3.011755304	-4.899692870	-6.640167434
	H278	1.847599544	-4.110781663	-4.344101341
	H279	2.673146683	-1.917194590	-6.973209404
	H280	9.150207142	0.959263271	-2.146458947
	H281	8.441889141	-0.867335203	-4.724573167
	H282	11.171545810	-1.199194955	-5.651869701
	H283	9.776322656	-0.623784845	-1.633469002
	H284	9.114338137	1.438478186	-6.036962167
	H285	-4.094967201	-9.660081838	1.228483868
	H286	-5.087178495	-8.641985324	2.290410778
	H287	-3.690849574	-7.651977358	6.473003963
	H288	-6.214211409	-10.709907165	2.008722088
	H289	-7.032343627	-9.659226028	1.054873249
	H290	5.702926005	-4.004377508	1.817680132
	H291	8.639474487	-3.386057257	1.484622345
	H292	7.456696811	-6.454792318	-1.261757624
	H293	6.330629561	-2.798103620	0.652806701
	H294	9.132267736	-4.939675359	1.614277584
	H295	2.131947543	-8.539580985	0.150398595
	H296	7.072876358	-5.770307600	0.784551555
	H297	-2.771301587	2.294924345	9.308608113
	H298	-3.777919097	-0.453676796	10.224944068
	H299	0.191663782	-7.618174071	-0.603768839
	H300	2.646622296	0.180949435	5.156166937
	H301	-1.349310013	-7.817888445	-3.836317449
	H302	-3.771961375	-3.031100471	-1.689956090
	H303	-0.426348873	-6.343209558	-3.537782934
	H304	-1.077862021	2.142774943	7.210840595
	H305	-1.452765782	-6.587729453	-1.089045955
	H306	-2.212018884	0.973157187	6.523682072
	H307	-3.758719596	1.147390997	8.374701921
	H308	-1.828830893	-0.533082557	8.521872834
	H309	-0.830762697	0.733066926	9.259390880
	H310	-4.629473177	-2.564568725	-6.354330626
	H311	-4.693754871	2.302498328	10.967437056
	H312	-7.697641839	1.694734983	10.823208267
	H313	-1.438341523	-3.912545879	-1.569363633
	H314	-6.203887940	3.169048318	12.849361111
	H315	-7.219602784	3.724508039	11.690204537
	H316	-0.479357114	-0.652328307	6.331876405
	H317	-0.048170477	-4.018484219	-3.592817411
	H318	-4.798668893	-2.581800770	-3.912771634
	H319	-6.875900180	-4.047731205	9.844685333
	H320	-6.072756386	-5.854580685	7.195677774
	H321	-5.135565608	-2.428330413	8.950119953
	H322	-8.103906236	-4.491417945	8.987156377
	H323	0.738647083	-10.710987877	-0.993068257
	H324	-2.547366607	-2.933550617	7.297178509
	H325	-5.965423818	0.480467582	5.644591325
	H326	-3.185765478	-7.745275357	0.390654039
	H327	-7.509136569	-1.158468004	6.363319039
	H328	-7.445493181	-6.714973168	2.609919050
	H329	-5.671003053	-4.954551871	2.127646731
	H330	-7.470316406	1.226251707	12.513411185
	H331	1.494732290	-1.098825293	5.358307866
	H332	-2.152945250	-2.974925784	9.637746992

H333	-1.067909892	-3.413560458	7.350935477
H334	-2.207015757	-1.405099865	11.416957426
H335	0.665605722	2.775433058	6.383223878
H336	-7.447756480	-4.011028857	5.238183658
H337	-6.902155536	-3.592757535	6.869224184
H338	-7.875808360	-1.633865825	4.696323787
H339	-3.503335488	-5.516675450	-0.527562100
H340	-1.078522154	-3.596504215	-5.844368618
H341	-3.093549656	-2.925045113	-7.189153590
H342	-4.420961442	0.016256492	4.904033677
H343	-3.535975501	-2.965495911	10.349541841
H344	-5.551336710	-2.050704755	10.388199365
H345	2.112361841	2.348083854	5.653001511
H346	-7.309599728	-7.587396039	5.016380058
H347	-4.490958263	-5.248282969	6.716997821
H348	-4.944798677	-6.136175218	-1.386225744
H349	2.791687405	-8.523268779	-1.501542600
H350	3.918828876	-0.485303632	7.749717013
H351	3.757324451	-2.039061435	6.894688207
H352	-4.992084586	-9.335641190	7.473388303
H353	-6.220186616	-8.311095328	7.825320411
H354	-4.765456374	-5.670393665	11.345095623
H355	-5.488220331	-5.957711900	9.199470017
H356	-4.152309840	-8.323172472	11.113709550
H357	-3.143902440	-5.443934065	10.658063981
H358	4.365924437	-3.370022020	9.186588516
H359	11.810362797	3.121945870	-6.714374210
H360	-0.971426742	0.340732434	13.537466161
H361	0.641052410	-0.702028968	11.538154084
H362	9.749537768	2.139389754	-8.720873161
H363	8.908884222	3.951245801	-6.370587539
H364	8.871083119	4.393632972	-8.087784770
H365	-3.936451471	0.141974322	2.159826343
H366	3.247569635	1.268507135	-8.780710974
H367	-2.861310319	0.963328502	1.220184370
H368	0.694445193	-0.684284099	3.005158551
H369	-2.748570449	1.054791449	2.839231904
H370	-1.252535507	-0.481025627	-0.455889478
Mo371	-3.435292551	-3.577855914	4.192524849
N372	0.160989558	-0.875608792	2.139105049
N373	-2.954839014	0.411681673	2.072971742
N374	-8.966623617	4.360263238	-2.564635815
N375	-6.415941443	6.654073115	-4.047654721
N376	-5.496640789	8.577125315	-2.216581439
N377	-5.280991394	7.262513821	0.280403247
N378	-3.081923551	5.618829455	-0.330586146
N379	-1.037341208	7.680890803	-0.614906840
N380	-1.042145549	9.241991611	1.842472707
N381	-1.254833281	7.318221560	3.890411626
N382	0.156795305	4.875921318	3.236082750
N383	2.920040805	5.112081852	1.982264894
N384	3.590410528	7.693242121	3.039020976
N385	-6.475954235	3.048513087	11.871218397
N386	-4.716208006	1.286430765	10.832422361
N387	-0.238427156	0.314882480	6.567613483
N388	1.352805238	2.028665576	6.257065985
N389	1.677289884	-0.078171437	5.330174293
N390	-1.943880719	-0.535372086	11.932132682
N391	-5.577231362	0.975990312	-4.440512261
N392	-6.667606440	-0.928399238	-0.230122733
N393	-3.186877974	1.470379205	-5.405327430
N394	-2.621200088	3.507090071	-7.233303708
N395	-1.769886511	5.615683218	-5.571562915
N396	0.659417716	4.694668845	-4.480304175
N397	0.788874758	0.473704727	-3.986568631
N398	-0.631782942	0.851936589	-2.317328544
N399	2.935380500	5.160223257	-5.969864282
N400	4.005000265	5.498109281	-10.583892037
N401	6.059911010	6.321391387	-10.741825794
N402	6.233945131	-2.965697454	7.430470297
N403	8.307539176	-4.336058968	1.681043070
N404	7.872612578	-5.592637597	-1.604620794
N405	6.284922548	-4.539514013	-3.620385956
N406	3.054077682	-2.862709993	-7.188462393
N407	4.163179169	-4.169951566	-8.792379488
N408	4.414992651	-1.869937597	-8.781361978
N409	5.419368791	-1.932376061	-2.740941906
N410	7.891913283	-0.664936454	-2.536743759
N411	9.408389094	-0.556712255	-4.827426258
N412	7.328472643	-2.782628830	-7.295521034

N413	9.852785326	1.445279962	-6.736303534
N414	-6.142850072	-10.158083576	1.151328989
N415	-4.043337670	-7.497901072	-0.110548835
N416	-1.778279993	-6.903085525	-2.021864092
N417	0.718496177	-8.133149946	-1.316727177
N418	-6.562587230	-7.193809148	-4.098596145
N419	-4.963959516	-5.259826466	-5.496170131
N420	-5.477960754	-8.496505237	7.143144829
N421	-5.084991548	-5.274328709	4.177239103
N422	-6.738882468	-6.426380571	3.281235963
N423	-4.589668791	-6.397161755	9.413009376
N424	2.144756224	-0.356877507	-1.249957537
N425	1.648831364	-0.947394325	-0.427045077
O426	-6.345668375	5.477324188	-2.096374501
O427	-3.361745449	7.834929550	-2.490521514
O428	-3.943075631	8.962585308	1.009340255
O429	-2.484176338	5.743315918	1.863895755
O430	0.397724871	6.066954416	0.075907357
O431	1.233232807	9.119274040	1.686130465
O432	-0.358305996	8.818903963	5.383856787
O433	0.049043983	4.318104377	5.457399356
O434	3.299928759	3.710833910	3.758723976
O435	5.548359857	6.730501948	3.664024632
O436	4.822439418	9.171573584	6.060766161
O437	-6.100274130	-0.524695693	11.219893535
O438	-2.206034190	1.740295288	12.118447867
O439	1.236487727	-1.475987747	13.329398481
O440	-2.820057222	3.287329587	-4.043208289
O441	-6.608068097	-0.805696347	-2.500440160
O442	-0.388457895	3.151616548	-6.928594332
O443	-0.593253269	0.182715118	-7.691931538
O444	-1.306736904	6.894034526	-7.423242698
O445	0.905367682	6.626208381	-3.284166750
O446	4.234409273	4.900342917	-4.116355462
O447	5.819173826	7.185133913	-6.306544191
O448	4.776868851	-1.771934296	10.389947136
O449	6.601803789	1.061463426	2.397080281
O450	8.403345339	-3.425278100	-1.137557624
O451	7.990970174	-4.673918173	-5.150864020
O452	5.408983940	-1.402621521	-4.966677618
O453	6.869295519	1.351122249	-2.930610653
O454	6.367175432	-0.527412383	-0.053945124
O455	11.277301317	0.074333017	-3.658340211
O456	11.293679805	0.114904073	-7.897795394
O457	8.088666075	-0.800561044	-8.143790558
O458	11.665931076	3.917611909	-8.591882830
O459	3.680488778	0.972529469	-7.956573469
O460	-6.326033627	-7.744020834	-0.184995329
O461	-3.611076968	-6.942638450	-3.394754439
O462	1.063450965	-8.651316796	-3.506061033
O463	2.741723142	-11.094927443	-0.906786456
O464	-6.944465383	-4.506086241	-6.338878368
O465	-8.370484558	-3.503095382	-0.526537498
O466	-7.220175893	-5.266823720	0.214890234
O467	-2.107248428	-5.267193980	-7.653329090
O468	-2.803160446	-7.654206357	8.811039168
O469	-2.708839346	-7.463215830	12.272442788
O470	-5.841829204	-0.601839223	2.537335075
O471	-6.366127857	1.457790238	3.309556745
O472	-9.196635584	-4.066047644	7.696959310
O473	-9.966867972	-3.006664958	5.853404630
O474	-4.036048515	-3.024929064	6.223693181
O475	-5.070803771	-1.497977002	7.511941173
O476	-5.376082013	-2.454160546	4.085693794
O477	-2.557759097	-2.931815613	10.539457565
O478	-1.715053678	-2.863432206	7.829906181
O479	1.857636569	-0.330562814	-6.579598339
O480	-7.562086295	-4.749828416	9.792961645
O481	-5.237793610	-2.826090133	9.858510222
S482	4.772369246	-4.331398742	-0.412957320
S483	1.428283885	-3.909746239	-0.489920630
S484	-3.387648148	-3.245188899	1.847362063
S485	2.859630383	-2.194820202	1.989242726
S486	-1.241834949	-1.807999080	-0.125550436
S487	-1.127841047	-6.460616271	1.117832471
S488	-1.947437664	-1.667031607	4.391453383
S489	2.322832370	-5.676224737	2.427280715
S490	-1.885567363	-5.327508732	4.493821655
S491	1.266222887	-3.366690228	5.294641872
end			

product

Fe(139)	-2.289
Fe(140)	-0.017
Fe(141)	-2.511
Fe(142)	2.656
Fe(143)	1.614
Fe(144)	2.172
Fe(145)	0.138

bm612bh135nh3distb.car_2			
C1	-6.971172047	5.836645929	-3.104747185
C2	-8.430523819	5.435011486	-3.355129364
C3	-5.012589850	7.000753855	-4.034561282
C4	-4.566402619	7.862733826	-2.859355521
C5	-5.188469664	9.361429540	-1.036067266
C6	-4.750279952	8.516470004	0.164964234
C7	-4.764936836	6.332055733	1.254228016
C8	-3.332193583	5.874730367	0.955790352
C9	-1.696851298	5.342813504	-0.796747057
C10	-0.662801606	6.413201055	-0.415485596
C11	-0.047974214	8.750511111	-0.362883727
C12	0.117950692	9.057522694	1.131085142
C13	-1.009055457	9.661319183	3.216583304
C14	-0.810496304	8.550520273	4.253897173
C15	-1.273692695	6.198082518	4.804736886
C16	-0.307146315	5.045984307	4.536449830
C17	1.030956411	3.770870795	2.869767331
C18	2.504522588	4.219315210	2.954777763
C19	0.674438250	3.203447166	1.481486359
C20	1.666897488	2.123588337	1.046988288
C21	-0.748514636	2.651577849	1.473000436
C22	4.266130251	5.621802514	1.924639891
C23	4.521019575	6.727232570	2.959748271
C24	4.596182766	6.091373159	0.483717576
C25	5.969240796	6.772286377	0.409719520
C26	4.526973341	4.923026578	-0.506572608
C27	3.813845589	8.840429416	3.917600201
C28	4.150237779	8.443759030	5.340439834
C29	-6.946970504	1.665739732	11.649647913
C30	-5.850628826	0.691725306	11.204316616
C31	-3.485534883	0.595002325	10.393715690
C32	-2.465870740	0.658683858	11.549078801
C33	-2.939058093	1.237262314	9.103029430
C34	-1.667133210	0.571108812	8.565955952
C35	-1.288564132	1.107633543	7.179813793
C36	0.937743507	0.725091307	6.043815326
C37	-1.004638079	-0.641959958	13.022893464
C38	0.410462073	-0.963734583	12.593199391
C39	-4.756623447	1.836325095	-3.588943950
C40	-3.501468248	2.290573987	-4.361029340
C41	-4.317031752	1.100324426	-2.303304506
C42	-5.420993835	0.901595599	-1.242513303
C43	-6.266812246	-0.365961402	-1.377880405
C44	-1.894906699	1.457369432	-6.069808398
C45	-1.565095053	2.766940961	-6.793541681
C46	-1.877392682	0.251774617	-7.022854533
C47	-2.431839135	4.805763505	-7.831240727
C48	-1.778008344	5.858295741	-6.927314503
C49	-1.045960124	6.474930339	-4.674562954
C50	0.267684098	5.936643770	-4.104639837
C51	1.934332909	4.144982490	-4.010796001
C52	3.151872433	4.803164594	-4.684758632
C53	2.037363564	2.634741169	-4.279120207
C54	0.993269047	1.804406210	-3.608920532
C55	0.079180066	2.043185998	-2.602867628
C56	-0.232308538	-0.000068003	-3.190710081
C57	4.039412932	5.614860577	-6.827705722
C58	4.668489811	6.858316197	-6.203894791
C59	3.437101815	5.937344293	-8.215363306
C60	4.398710928	6.011063671	-9.363132944
C61	5.673961698	6.535183821	-9.447169127
C62	5.052092140	5.702212854	-11.404148528
C63	5.554219042	-1.854606217	8.089714737
C64	4.847840946	-2.384294787	9.322444761
C65	4.467382363	-1.243604190	7.167674879
C66	5.045337209	-0.621202516	5.918684091
C67	5.453477933	0.724953432	5.895352534
C68	5.194145966	-1.374351006	4.739978880
C69	5.974903063	1.305825383	4.734403156
C70	5.725854203	-0.812666735	3.578835772
C71	6.105655302	0.531304167	3.574598784
C72	7.342905967	-4.730277808	0.633068341
C73	7.929326408	-4.524126785	-0.776954596
C74	6.087588362	-3.883944265	0.795101790
C75	8.417899042	-5.565364625	-2.965451872
C76	7.543854822	-4.882586951	-4.021530057
C77	5.358465500	-3.765395410	-4.433655787

C78	5.400859528	-2.259488456	-4.069951124
C79	3.957298327	-4.378980108	-4.210801103
C80	2.742015667	-3.713305632	-4.866355170
C81	2.541889109	-3.962555366	-6.370952878
C82	3.855992104	-2.986630856	-8.260844583
C83	5.459441850	-0.534307960	-2.337650745
C84	6.799137783	0.145272173	-2.652399345
C85	5.182554437	-0.338145098	-0.832242653
C86	9.223029845	-0.094009733	-2.433869443
C87	10.076049516	-0.185602460	-3.689151096
C88	10.184768932	-0.900563290	-6.012964609
C89	10.485429173	0.264230008	-6.989031384
C90	9.623521173	-2.103111584	-6.778456711
C91	8.293042499	-1.824650266	-7.463517838
C92	9.904250422	2.538789991	-7.710573569
C93	11.251040529	3.245233298	-7.682307260
C94	8.768419281	3.553527292	-7.389455132
C95	7.390886731	2.941172734	-7.519010234
C96	6.724501802	2.943768271	-8.755105013
C97	6.758006974	2.312593235	-6.433073945
C98	5.482488633	2.324174712	-8.912359779
C99	5.527538298	1.665148542	-6.576990609
C100	4.902729398	1.672474908	-7.822308405
C101	-5.070319250	-9.170428439	1.319998817
C102	-5.202724509	-8.089124012	0.262411486
C103	-3.942392736	-6.408870781	-1.065793514
C104	-3.100938617	-6.789982674	-2.290333797
C105	-0.823068327	-7.285512624	-3.069091173
C106	0.383952766	-8.111546584	-2.634757215
C107	1.920019675	-8.839311882	-0.892932950
C108	1.797880621	-10.348436922	-0.980650229
C109	-7.055791364	-5.828272057	-4.308484901
C110	-6.324521825	-5.148411710	-5.471649720
C111	-7.068183827	-4.901649588	-3.071792377
C112	-8.115368553	-5.367247711	-2.042373530
C113	-7.941951474	-4.633992416	-0.730939930
C114	-4.197961813	-4.681730742	-6.577695843
C115	-3.100027814	-5.625457302	-7.027643676
C116	-3.733701297	-3.223848456	-6.334268809
C117	-3.008394968	-3.148447307	-5.024401095
C118	-1.651038736	-3.495889408	-4.941051108
C119	-3.739331216	-2.948972179	-3.839356113
C120	-1.065720874	-3.738963122	-3.696062777
C121	-3.152900896	-3.196283927	-2.598404299
C122	-1.828437303	-3.640494640	-2.531760649
C123	-4.536572507	-7.362115318	7.116228618
C124	-3.901151847	-7.139915196	8.496507662
C125	-5.168471227	-6.070044394	6.573932266
C126	-5.590022839	-6.092319211	5.138826551
C127	-6.614729325	-6.818152277	4.568280383
C128	-5.771037845	-5.437070219	3.056585904
C129	-3.985342226	-6.141685831	10.702305500
C130	-3.564716712	-7.417836151	11.396135293
C131	-6.116806101	0.309897019	3.555945509
C132	-5.549944729	-0.136489754	4.920263263
C133	-5.776185185	-1.633520386	5.244399345
C134	-7.254492382	-1.894430093	5.606408842
C135	-7.569176572	-3.312428536	6.086149498
C136	-9.017971802	-3.473793161	6.597892607
C137	-4.883997595	-2.048738010	6.420449128
C138	-0.190706094	-3.504346241	2.287724243
Fe139	-1.781957420	-4.680956573	2.294136010
Fe140	0.750820047	-1.973343360	0.816572470
Fe141	1.589223728	-3.658224910	3.132475962
Fe142	0.516883901	-4.981605667	1.195334299
Fe143	3.036891790	-4.142080088	1.001728038
Fe144	-0.701121966	-3.409101974	4.221171107
Fe145	-1.390491148	-1.978793834	2.148945842
H146	-8.801824590	4.556460369	-1.550417284
H147	-8.420335687	3.492133411	-2.726444809
H148	-6.948659705	6.836925505	-4.903270976
H149	-8.552041084	5.183289180	-4.420766027
H150	-9.045479925	6.335323300	-3.185779196
H151	-4.393709400	6.091526334	-4.007702611
H152	-6.474890987	8.532829799	-2.550997572
H153	-4.772819640	7.537284508	-4.962005989
H154	-6.069405269	9.951727345	-0.752863874
H155	-5.854585947	6.901178640	-0.498144076
H156	-4.364149156	10.057149095	-1.243460788
H157	-1.356818218	4.394446830	-0.367177404

	H158	-3.778633108	5.770855229	-1.042329527
	H159	-5.429647042	5.458000809	1.287106610
	H160	0.937491001	8.456321720	-0.745083992
	H161	-1.872496023	7.910642223	-1.158435908
	H162	-4.757618804	6.798711974	2.246322735
	H163	-1.732552173	5.230343963	-1.888499496
	H164	-1.940696551	9.166171223	1.353126617
	H165	-0.371757814	9.665528096	-0.877481131
	H166	-1.483977088	3.427765537	1.734162024
	H167	-0.990353938	2.252445975	0.475032457
	H168	-0.846646329	1.804241756	2.167309897
	H169	0.729799221	4.032039681	0.758928101
	H170	-1.960802190	10.161537507	3.446667365
	H171	3.550055240	4.419363550	-0.492148489
	H172	2.759029378	7.723246640	2.450484942
	H173	6.025719977	7.670825805	1.040751051
	H174	1.425544118	1.811688816	0.023736535
	H175	1.580279396	1.231211854	1.682764333
	H176	6.223315321	-1.034829589	8.414497965
	H177	4.698393056	5.278857828	-1.532320223
	H178	6.175714720	7.070591997	-0.628663052
	H179	5.300620959	4.173123396	-0.275624805
	H180	6.766275405	6.083229166	0.730970336
	H181	3.832343660	6.838203963	0.197057907
	H182	4.945988962	4.802524101	2.204288958
	H183	2.215236314	5.434120484	1.315735114
	H184	2.714507212	2.460806971	1.057077343
	H185	4.384446799	-1.027099282	-0.518019373
	H186	4.821539483	0.693230053	-0.706036812
	H187	6.337917910	0.047488807	0.715271399
	H188	6.774082611	1.999982495	2.495825494
	H189	5.809406922	-1.401552619	2.666078406
	H190	4.858753261	-2.413401290	4.717471507
	H191	5.346865357	1.339085439	6.793252199
	H192	-0.220052223	5.482389284	2.537565155
	H193	-2.282663259	5.757417267	4.822551476
	H194	-1.047301472	6.570380212	5.810464453
	H195	-1.743928947	7.196195504	3.000061114
	H196	-0.196240770	10.382999891	3.371192243
	H197	6.259208385	2.361282063	4.727186962
	H198	0.919442852	2.980217994	3.621813333
	H199	4.617672708	9.489553020	3.531907551
	H200	2.887714799	9.435167505	3.946412759
	H201	3.716032097	7.472534388	5.673682500
	H202	-1.341512374	-1.392889463	13.754564439
	H203	-7.251530950	-1.778686604	-0.287132893
	H204	-5.557062299	-1.734457468	3.276007907
	H205	-9.122331656	-5.150410675	-2.426912739
	H206	-8.034602602	-6.452225683	-1.881025217
	H207	-7.297471666	-3.867069957	-3.371090946
	H208	-3.278226066	-6.697575015	-6.773693303
	H209	-8.092642589	-5.906225890	-4.669437060
	H210	-3.875983907	0.131231270	-2.584643989
	H211	-6.119157146	1.756966683	-1.262078070
	H212	-4.480122572	-5.784674731	-4.757190728
	H213	-4.890256162	-4.653371114	-7.444729327
	H214	-5.652536419	-7.210401030	-3.646000410
	H215	-6.375468735	-0.625465301	0.712112686
	H216	-6.062034135	-4.884298938	-2.625682317
	H217	-7.215715090	-7.702931485	-3.484728587
	H218	-6.287386265	1.516172650	-4.944540880
	H219	-3.502432132	1.679367960	-1.847129611
	H220	-6.927467427	-6.182864648	-0.075966043
	H221	-4.965448649	0.914343005	-0.241915217
	H222	2.024555496	4.355287980	-2.936345496
	H223	-0.078401027	2.969306055	-2.058565692
	H224	-0.797653107	7.398256359	-5.215857844
	H225	-2.183463506	4.745835312	-5.213587427
	H226	2.016646314	5.081265653	-6.371739893
	H227	6.320251807	7.000707485	-8.710574752
	H228	3.936138416	7.502807412	-5.659910248
	H229	2.838335783	6.862490244	-8.140979985
	H230	4.813371014	4.836638831	-6.920492769
	H231	5.107816161	5.423643990	-12.453152580
	H232	3.045217269	2.322632235	-3.956147169
	H233	1.992522656	2.469656823	-5.369069921
	H234	6.972586544	6.587959669	-11.145571639
	H235	2.726035224	5.131385227	-8.457541014
	H236	0.119547376	4.154017560	-5.165334476
	H237	-1.685320892	6.767743587	-3.827807126

	H238	-1.793574004	4.740998196	-8.723666506
	H239	-0.585603482	-1.026087981	-3.259499406
	H240	-3.410817854	5.185874031	-8.150882877
	H241	7.092447438	-3.199320872	7.936825239
	H242	6.537967572	-2.673577163	6.491967403
	H243	5.238244351	-2.659589961	-2.021566699
	H244	4.691000048	0.015356377	-2.900071943
	H245	7.763271430	-1.587928883	-2.130578898
	H246	1.258010063	0.017105596	-4.753880807
	H247	0.978136872	-0.136040964	-6.986907623
	H248	-0.705559008	-0.455794758	-8.424944417
	H249	2.485936587	0.344151842	-6.935479707
	H250	-2.070624496	-0.655557680	-6.427045839
	H251	-2.674382541	0.364561111	-7.774708071
	H252	9.565338447	-2.972772625	-6.109069277
	H253	10.350053361	-2.338468864	-7.571338687
	H254	7.472918734	-3.504786237	-6.577421665
	H255	6.414707413	-2.531090375	-7.596460277
	H256	4.967852825	-4.264964061	-9.416808552
	H257	1.469834321	-4.070712408	-6.593046280
	H258	4.974075439	2.346780376	-9.880189233
	H259	7.213289249	2.324383906	-5.439271964
	H260	4.201418893	-0.959127089	-8.406568600
	H261	3.773964261	-4.388243743	-3.124097035
	H262	6.027401973	-4.745934818	-2.661765811
	H263	2.723960118	-2.630756970	-4.664392248
	H264	-3.555099015	3.164077035	-7.021702280
	H265	8.604279397	-6.594776608	-3.300450316
	H266	-3.875735677	0.704971390	-5.498682851
	H267	4.005233902	-5.435612443	-4.521900240
	H268	5.082341514	1.137178972	-5.733661005
	H269	9.379740888	-5.032279418	-2.968642748
	H270	3.754424881	-5.035270907	-8.453388986
	H271	4.732178758	-1.903985539	-9.758055060
	H272	-6.070266579	0.274246727	-3.844799719
	H273	-5.264613548	2.771524568	-3.285114886
	H274	7.178883788	3.439610779	-9.615980241
	H275	-1.090052392	1.333502128	-5.330739383
	H276	5.660407592	-3.835391036	-5.482207519
	H277	3.020635512	-4.908796716	-6.656126323
	H278	1.862357406	-4.126928495	-4.356614026
	H279	2.684103251	-1.924105496	-6.981273413
	H280	9.142389218	0.969929481	-2.164336354
	H281	8.449921551	-0.850155308	-4.734708443
	H282	11.185283575	-1.189112174	-5.654004709
	H283	9.774830186	-0.605006428	-1.633650317
	H284	9.136792522	1.444018898	-6.037272734
	H285	-4.089100109	-9.660667923	1.233436166
	H286	-5.077757934	-8.640397893	2.295008413
	H287	-3.702213675	-7.661629693	6.464227247
	H288	-6.203623434	-10.711937148	2.026488900
	H289	-7.026870040	-9.664419133	1.072182822
	H290	5.713339799	-4.027117632	1.818341611
	H291	8.642172893	-3.403387210	1.480133984
	H292	7.464802417	-6.465597803	-1.277973369
	H293	6.333827002	-2.821997607	0.652234733
	H294	9.135036595	-4.957615006	1.613026177
	H295	2.108808071	-8.573410903	0.159574225
	H296	7.082854318	-5.790801910	0.775731611
	H297	-2.757804390	2.308341563	9.293579523
	H298	-3.757262809	-0.448829165	10.192788214
	H299	0.179906958	-7.636830678	-0.610282921
	H300	2.650375403	0.109231734	5.116699853
	H301	-1.348853744	-7.854843428	-3.844775648
	H302	-3.723609435	-3.043112259	-1.680633124
	H303	-0.424970449	-6.377323052	-3.553940873
	H304	-0.975292054	2.156682367	7.239005504
	H305	-1.455535960	-6.590110623	-1.115375629
	H306	-2.165596903	1.068136183	6.512040065
	H307	-3.741130928	1.163669018	8.351492622
	H308	-1.820617023	-0.515958304	8.489596234
	H309	-0.821777287	0.730683604	9.254273731
	H310	-4.625404793	-2.580735097	-6.337158809
	H311	-4.684075970	2.298691707	10.948347364
	H312	-7.692933843	1.666128761	10.839959801
	H313	-1.373266163	-3.879312869	-1.573205159
	H314	-6.184604476	3.167259747	12.837714737
	H315	-7.211494464	3.706178980	11.679121936
	H316	-0.488775269	-0.658711408	6.347700478
	H317	-0.014443890	-4.019735373	-3.619714052

	H318	-4.781060565	-2.623025429	-3.893794257
	H319	-6.857487044	-4.066139780	9.843728891
	H320	-6.056721953	-5.828257692	7.182526249
	H321	-5.108392756	-2.447142189	8.951289961
	H322	-8.086246752	-4.511885242	8.988818009
	H323	0.749629495	-10.733373346	-1.020461215
	H324	-2.501338694	-2.920798184	7.285821291
	H325	-5.970435278	0.484714845	5.719551386
	H326	-3.185410374	-7.769442030	0.368051427
	H327	-7.511718094	-1.176065071	6.399793989
	H328	-7.410479207	-6.680793720	2.585463102
	H329	-5.641434236	-4.914205813	2.116699730
	H330	-7.435381053	1.206746353	12.526040093
	H331	1.494754849	-1.168782521	5.378911995
	H332	-2.134410466	-2.968056521	9.628120108
	H333	-1.043330979	-3.450999664	7.390306780
	H334	-2.193435769	-1.405059800	11.398798342
	H335	0.702172899	2.761835015	6.229194829
	H336	-7.426442707	-4.020094613	5.254194628
	H337	-6.879945373	-3.611390361	6.888414010
	H338	-7.876564400	-1.648698039	4.731798984
	H339	-3.505108212	-5.538057297	-0.553204997
	H340	-1.074091831	-3.625577745	-5.859224026
	H341	-3.096854240	-2.936693626	-7.184802841
	H342	-4.462350237	0.056453022	4.882911039
	H343	-3.514125421	-2.977579017	10.345009364
	H344	-5.527003810	-2.066884624	10.384572718
	H345	2.186748010	2.261215218	5.626342084
	H346	-7.282039096	-7.561277697	4.988578474
	H347	-4.464998343	-5.239378649	6.712938433
	H348	-4.948588617	-6.160078870	-1.407335140
	H349	2.793910671	-8.519316229	-1.480032199
	H350	3.923135390	-0.485745822	7.751224328
	H351	3.753835005	-2.038862812	6.897277066
	H352	-5.030412351	-9.324637388	7.453137874
	H353	-6.240122919	-8.284099261	7.817497558
	H354	-4.747851242	-5.661319379	11.336112304
	H355	-5.491088510	-5.963163964	9.201510222
	H356	-4.158803595	-8.320005956	11.114260312
	H357	-3.128078442	-5.452839678	10.638971646
	H358	4.373428612	-3.385612882	9.181580438
	H359	11.830832385	3.116923641	-6.737192057
	H360	-0.976480190	0.338192737	13.531198844
	H361	0.656357712	-0.695180842	11.536658579
	H362	9.755737104	2.141548825	-8.727740413
	H363	8.919531857	3.947293441	-6.372095890
	H364	8.878767463	4.395849169	-8.087893267
	H365	-4.062395367	0.380909591	2.277094575
	H366	3.263658297	1.260104956	-8.789904191
	H367	-3.154254844	1.526698109	1.504602577
	H368	0.518443863	-0.581433937	2.985220747
	H369	-3.063104716	1.386352379	3.131041219
	H370	-1.301515734	-0.357348964	-0.141212676
	Mo371	-3.445181743	-3.528621339	4.172399634
	N372	0.009524715	-0.843252145	2.126128151
	N373	-3.150982860	0.846319854	2.266178360
	N374	-8.949103694	4.347853376	-2.541271384
	N375	-6.425876302	6.660086556	-4.049569928
	N376	-5.513962610	8.588384171	-2.222792491
	N377	-5.272956321	7.266598856	0.259083632
	N378	-3.046959606	5.650267603	-0.347467058
	N379	-1.016143752	7.700865006	-0.640646036
	N380	-1.036215518	9.243792817	1.825555757
	N381	-1.249623897	7.316634193	3.885811793
	N382	0.132110383	4.864802820	3.268293658
	N383	2.897619210	5.113932141	2.004313780
	N384	3.573475144	7.695076722	3.063720833
	N385	-6.463666845	3.035731152	11.862827874
	N386	-4.702262860	1.282757018	10.813034361
	N387	-0.224352755	0.306093099	6.563351243
	N388	1.381992343	1.993182332	6.195914445
	N389	1.698827139	-0.152177538	5.362830864
	N390	-1.931188445	-0.534022337	11.912402807
	N391	-5.584587591	0.970512557	-4.439847654
	N392	-6.658937926	-0.945321673	-0.227389357
	N393	-3.185230990	1.466350824	-5.395888382
	N394	-2.617584123	3.492953891	-7.241175543
	N395	-1.763149334	5.607053723	-5.589922399
	N396	0.672610965	4.695202571	-4.496063999
	N397	0.778009502	0.488336075	-3.974821513

	N398	-0.679125377	0.921054947	-2.347809348
	N399	2.951391002	5.159540334	-5.978930995
	N400	4.021542448	5.495289157	-10.593069900
	N401	6.073216099	6.326741967	-10.750618680
	N402	6.237671923	-2.966694719	7.425588315
	N403	8.310515425	-4.353495344	1.677715732
	N404	7.880303596	-5.601453285	-1.616168462
	N405	6.299999876	-4.540146266	-3.629866364
	N406	3.062895177	-2.870441561	-7.199035673
	N407	4.154978024	-4.177362344	-8.814743043
	N408	4.431509953	-1.880920385	-8.787483837
	N409	5.425354415	-1.940969365	-2.744952322
	N410	7.894199110	-0.662421961	-2.546520715
	N411	9.419470299	-0.547512401	-4.833473940
	N412	7.356182387	-2.802376478	-7.319218073
	N413	9.867837123	1.446670499	-6.744190582
	N414	-6.137053444	-10.163013035	1.166908172
	N415	-4.045508986	-7.516258702	-0.125099287
	N416	-1.781614368	-6.925667892	-2.040072517
	N417	0.712365101	-8.151860898	-1.318118906
	N418	-6.573358314	-7.200275398	-4.100955138
	N419	-4.967758055	-5.276338240	-5.501693121
	N420	-5.504275667	-8.475721408	7.130113670
	N421	-5.066524186	-5.235149670	4.169997094
	N422	-6.709582437	-6.391127373	3.262088403
	N423	-4.587804782	-6.398055609	9.406151502
	N424	2.105901376	-0.348129276	-1.185943514
	N425	1.582594679	-0.944231718	-0.386777636
	O426	-6.331949987	5.485191975	-2.098711874
	O427	-3.370908908	7.874522059	-2.516270345
	O428	-3.965700276	8.983755876	1.006828184
	O429	-2.488517600	5.731835277	1.857416691
	O430	0.437090843	6.107715616	0.072172733
	O431	1.239743255	9.166406267	1.651660531
	O432	-0.319825468	8.815657720	5.359855392
	O433	-0.013313364	4.304258455	5.492306939
	O434	3.268200953	3.767324530	3.822208602
	O435	5.554785634	6.751170093	3.647236781
	O436	4.791147471	9.156734925	6.098566658
	O437	-6.073773404	-0.536324112	11.205423448
	O438	-2.189010516	1.742312619	12.094521117
	O439	1.243655198	-1.461754362	13.333881825
	O440	-2.856644769	3.314090068	-4.062823292
	O441	-6.599485978	-0.824527055	-2.496682796
	O442	-0.385485191	3.135185838	-6.933619556
	O443	-0.598476699	0.162278769	-7.680017548
	O444	-1.309479618	6.882438453	-7.446265520
	O445	0.917875687	6.636848419	-3.317042259
	O446	4.245011084	4.914248705	-4.119320688
	O447	5.842432853	7.172294949	-6.312603122
	O448	4.769235814	-1.784499943	10.384877455
	O449	6.590628152	1.048831045	2.388108918
	O450	8.419997532	-3.439586937	-1.134284425
	O451	8.000220123	-4.692878933	-5.166680232
	O452	5.420017594	-1.403379983	-4.968752612
	O453	6.860108330	1.351548345	-2.919574068
	O454	6.365661157	-0.556896885	-0.054038280
	O455	11.287079202	0.066428681	-3.652853580
	O456	11.296851674	0.109163434	-7.913041814
	O457	8.103380489	-0.803251683	-8.138891258
	O458	11.665110397	3.929632749	-8.605132459
	O459	3.692685691	0.968535967	-7.962174515
	O460	-6.330579202	-7.747959986	-0.170301692
	O461	-3.614797020	-6.983528435	-3.411671322
	O462	1.064556052	-8.687735898	-3.499858308
	O463	2.757666307	-11.102292789	-0.979067877
	O464	-6.944638683	-4.499333128	-6.330886123
	O465	-8.375974320	-3.507217383	-0.522502030
	O466	-7.220469254	-5.267764684	0.219147274
	O467	-2.114206326	-5.281038830	-7.662780393
	O468	-2.809096191	-7.662390792	8.796355849
	O469	-2.701255904	-7.467420512	12.261733612
	O470	-5.950677977	-0.554755128	2.587087202
	O471	-6.588397203	1.451555452	3.418301823
	O472	-9.182436167	-4.083539079	7.713072595
	O473	-9.942853913	-3.014063086	5.871544308
	O474	-4.005053547	-2.993306461	6.201461516
	O475	-5.032939514	-1.494585814	7.524913252
	O476	-5.382847675	-2.411273258	4.090664350
	O477	-2.535722783	-2.935795207	10.531858658

	O478 -1.674582554 -2.848093392 7.824903676<br>O479 1.866382537 -0.337703587 -6.592774792<br>O480 -7.540055050 -4.771644619 9.792218657<br>O481 -5.215897212 -2.845313114 9.857746491<br>S482 4.762650222 -4.344711215 -0.405229658<br>S483 1.410553836 -3.854210737 -0.497342262<br>S484 -3.442732989 -3.258404951 1.805626391<br>S485 2.793118752 -2.171620671 2.013594868<br>S486 -1.316158898 -1.717285132 -0.086210423<br>S487 -1.147517331 -6.438505114 1.082798259<br>S488 -1.967694232 -1.597321271 4.318584571<br>S489 2.298984925 -5.689843872 2.411282364<br>S490 -1.887134256 -5.262857263 4.483402855<br>S491 1.202935302 -3.398305157 5.300483212<br>end

**Fe2-brNH-Fe6NH3-S2BH to Fe2-brNH-Fe6-S2BH<br>35, S=1/2<br>reactant<br><br>files wrong ?**	bm612n2xnewbrk2bh135ti.car_4<br>C1 -6.979307250 5.834683181 -3.106222614<br>C2 -8.440930425 5.451738302 -3.370058291<br>C3 -5.005485483 6.981490254 -4.021779305<br>C4 -4.560001672 7.839841673 -2.843714787<br>C5 -5.174252113 9.352284235 -1.029255625<br>C6 -4.745563502 8.505747912 0.174229681<br>C7 -4.787693432 6.328370067 1.278613536<br>C8 -3.356101837 5.865803124 0.975056978<br>C9 -1.751014989 5.332514136 -0.814113102<br>C10 -0.710945045 6.386605547 -0.406861181<br>C11 -0.053753018 8.705120671 -0.329037560<br>C12 0.104659489 9.025531269 1.162454925<br>C13 -1.028483917 9.668894928 3.233786216<br>C14 -0.843714435 8.558129015 4.272668788<br>C15 -1.256262878 6.192629518 4.794804453<br>C16 -0.272691791 5.060795252 4.504826950<br>C17 1.044571836 3.771115895 2.833672134<br>C18 2.525012968 4.199195400 2.911640025<br>C19 0.684623511 3.252915507 1.429606108<br>C20 1.627380215 2.136782611 0.978489789<br>C21 -0.781935083 2.834180435 1.422361755<br>C22 4.280069029 5.628489691 1.899461028<br>C23 4.524678544 6.720560523 2.947644821<br>C24 4.612229052 6.118753431 0.465361374<br>C25 5.986997673 6.799278908 0.407938219<br>C26 4.543086345 4.961837587 -0.537117640<br>C27 3.827108811 8.838335165 3.886514531<br>C28 4.167641116 8.457177447 5.311637623<br>C29 -6.973360454 1.688061660 11.642012719<br>C30 -5.879452799 0.706243829 11.215726319<br>C31 -3.511371273 0.594206310 10.421024150<br>C32 -2.491408474 0.660489361 11.574392957<br>C33 -2.963129251 1.225518759 9.125609811<br>C34 -1.691991755 0.557728545 8.585459769<br>C35 -1.347228044 1.076556424 7.183334581<br>C36 0.902236344 0.776732710 6.054453005<br>C37 -1.017061937 -0.639195973 13.035136583<br>C38 0.392407460 -0.969085104 12.594729634<br>C39 -4.753079228 1.841273243 -3.587333038<br>C40 -3.492368827 2.284242600 -4.354772793<br>C41 -4.325429083 1.116814960 -2.293622354<br>C42 -5.436032811 0.921196713 -1.240109217<br>C43 -6.281562538 -0.345073316 -1.379560229<br>C44 -1.904212422 1.468248193 -6.078302057<br>C45 -1.575066237 2.784209960 -6.792189024<br>C46 -1.880014620 0.266180084 -7.034825365<br>C47 -2.448135411 4.830066980 -7.814107389<br>C48 -1.787677936 5.875423797 -6.906891511<br>C49 -1.061237437 6.483575603 -4.649264306<br>C50 0.248242622 5.938071481 -4.078072245<br>C51 1.911368602 4.143281695 -4.000051934<br>C52 3.129409692 4.799572492 -4.676351375<br>C53 2.013149642 2.634937217 -4.275860147<br>C54 0.986621091 1.787229707 -3.600334115<br>C55 0.098242659 1.990060409 -2.563894772<br>C56 -0.193735910 -0.048397245 -3.187998855<br>C57 4.013161519 5.621564770 -6.815236313<br>C58 4.636267748 6.869899170 -6.194865572<br>C59 3.414028785 5.945231931 -8.204390928

C60	4.376313537	6.015414844	-9.352384395
C61	5.653891143	6.533839815	-9.437023417
C62	5.026763711	5.705430831	-11.394368388
C63	5.547449840	-1.845024861	8.091959717
C64	4.839779761	-2.367762105	9.326972675
C65	4.458795789	-1.237463159	7.166406368
C66	5.036574977	-0.612319295	5.918459938
C67	5.451545141	0.732070649	5.901415432
C68	5.182750347	-1.360749841	4.736270170
C69	5.977002186	1.316120954	4.744035101
C70	5.719096196	-0.795828367	3.578666475
C71	6.104811776	0.546413166	3.580923342
C72	7.330296175	-4.705923441	0.638122316
C73	7.909651228	-4.504872083	-0.772900363
C74	6.076971754	-3.854492374	0.797085711
C75	8.396553941	-5.555317105	-2.953755690
C76	7.523171305	-4.868882566	-4.006867595
C77	5.338255271	-3.760041508	-4.425912459
C78	5.383907035	-2.252139132	-4.064147086
C79	3.933550361	-4.366301965	-4.196387069
C80	2.721034188	-3.696441022	-4.852631504
C81	2.523577648	-3.947828843	-6.357559056
C82	3.843214641	-2.972809443	-8.247037783
C83	5.451025935	-0.519727439	-2.337729101
C84	6.794556853	0.151349016	-2.652841388
C85	5.173572636	-0.315156973	-0.832266114
C86	9.215466164	-0.099512807	-2.425533336
C87	10.059202809	-0.184047126	-3.687029890
C88	10.163647029	-0.901270193	-6.009184731
C89	10.467379323	0.269192485	-6.976439587
C90	9.598649551	-2.097435631	-6.783164255
C91	8.265075282	-1.812632006	-7.459698591
C92	9.885513484	2.540640404	-7.701893374
C93	11.232435764	3.245070164	-7.665194300
C94	8.750100466	3.557926370	-7.386164993
C95	7.371638047	2.947103346	-7.515268089
C96	6.701415591	2.955507153	-8.749266822
C97	6.742000108	2.313130945	-6.430544190
C98	5.459003539	2.336315767	-8.905977171
C99	5.512387806	1.664099050	-6.574349251
C100	4.883103225	1.679202922	-7.817158645
C101	-5.084975434	-9.165346060	1.310593085
C102	-5.209348061	-8.077557032	0.258388906
C103	-3.946599780	-6.380437526	-1.043138116
C104	-3.104088382	-6.753904781	-2.269437864
C105	-0.829304421	-7.247451660	-3.055832911
C106	0.379606628	-8.076458529	-2.633047846
C107	1.918906475	-8.822119217	-0.897953704
C108	1.781069138	-10.330717148	-0.941159090
C109	-7.058844658	-5.820287469	-4.310455695
C110	-6.330380679	-5.140161887	-5.474800453
C111	-7.078591983	-4.890625110	-3.077494747
C112	-8.122296556	-5.359781408	-2.046562063
C113	-7.946794152	-4.627040707	-0.735357096
C114	-4.201906880	-4.661844223	-6.572533208
C115	-3.105058630	-5.605319077	-7.024150198
C116	-3.743411136	-3.200718482	-6.339534727
C117	-3.029227412	-3.115898000	-5.025330469
C118	-1.673984900	-3.467677829	-4.929052888
C119	-3.771459906	-2.911671403	-3.848182857
C120	-1.107195136	-3.726103888	-3.679087657
C121	-3.203584076	-3.174418809	-2.602250601
C122	-1.888272765	-3.643649200	-2.526899771
C123	-4.534948112	-7.363113651	7.127596248
C124	-3.902716512	-7.132096875	8.507585756
C125	-5.191901085	-6.085406546	6.584852243
C126	-5.622410727	-6.123174231	5.153063706
C127	-6.653216258	-6.848188546	4.591967144
C128	-5.809155067	-5.478520136	3.068747021
C129	-4.004361575	-6.136710478	10.713945473
C130	-3.576061207	-7.412971541	11.403346312
C131	-5.973664856	0.287757246	3.474606357
C132	-5.514369770	-0.160398140	4.876900042
C133	-5.781034887	-1.650940607	5.217453277
C134	-7.269154662	-1.881088313	5.573278056
C135	-7.600222953	-3.293221644	6.064080874
C136	-9.047747440	-3.455452803	6.578794259
C137	-4.916176693	-2.069730074	6.414913611
C138	-0.205394794	-3.503016592	2.300177592
Fe139	-1.753321565	-4.679781978	2.339060237

	Fe140	0.832966159	-2.004929408	0.765760167
	Fe141	1.616244941	-3.633257757	3.131095065
	Fe142	0.510985483	-4.991726215	1.232587117
	Fe143	3.047169007	-4.147313139	0.992853973
	Fe144	-0.663532914	-3.495504690	4.290301301
	Fe145	-1.339285448	-1.905437643	2.111378721
	H146	-8.833896049	4.568263708	-1.574595136
	H147	-8.453484334	3.505530630	-2.752120482
	H148	-6.940659835	6.838418110	-4.901490989
	H149	-8.559041970	5.209524658	-4.438343277
	H150	-9.044716156	6.359599869	-3.197189145
	H151	-4.396310296	6.065680256	-3.986821730
	H152	-6.459125992	8.547804399	-2.558535936
	H153	-4.752208724	7.513600719	-4.948132442
	H154	-6.052074396	9.948762637	-0.748897486
	H155	-5.881327446	6.906419625	-0.470594157
	H156	-4.344552098	10.042661774	-1.233573110
	H157	-1.401506637	4.368493911	-0.435016436
	H158	-3.843483533	5.715984753	-1.009120994
	H159	-5.457937137	5.458249482	1.309019457
	H160	0.927081738	8.375059561	-0.693384320
	H161	-1.899247734	7.909098834	-1.127216066
	H162	-4.780210193	6.794111050	2.271086154
	H163	-1.806083740	5.263379036	-1.907888916
	H164	-1.952920206	9.164546635	1.368984658
	H165	-0.342375344	9.622448818	-0.858936162
	H166	-1.449815785	3.697294539	1.534220241
	H167	-1.038698587	2.312091474	0.487694854
	H168	-0.981408865	2.173272943	2.272637540
	H169	0.796223740	4.088494055	0.723560710
	H170	-1.976265694	10.179961050	3.454906452
	H171	3.555649816	4.479691936	-0.545996845
	H172	2.782684570	7.740544348	2.403525783
	H173	6.038597026	7.692833694	1.046948552
	H174	1.350198909	1.816697235	-0.033726294
	H175	1.574266451	1.253214098	1.631294904
	H176	6.218175994	-1.025019743	8.411402293
	H177	4.743543096	5.320359070	-1.556240096
	H178	6.204377281	7.107494363	-0.625272600
	H179	5.294260164	4.193510931	-0.293524488
	H180	6.780816115	6.107264655	0.730429690
	H181	3.852018062	6.871392588	0.183922603
	H182	4.961446476	4.807181394	2.166044207
	H183	2.217314565	5.485674830	1.329652858
	H184	2.673524619	2.476874860	0.938758317
	H185	4.381626189	-1.007606802	-0.512881984
	H186	4.806377274	0.714841575	-0.712228210
	H187	6.321871790	0.078317584	0.718700694
	H188	6.781853843	2.016734821	2.509988577
	H189	5.802775073	-1.380926704	2.663392872
	H190	4.842045432	-2.397873442	4.707829469
	H191	5.348115913	1.341789866	6.802539093
	H192	-0.197937033	5.494384278	2.497818410
	H193	-2.256448735	5.732143867	4.823549469
	H194	-1.021561725	6.558131902	5.801258039
	H195	-1.753955244	7.200013356	3.002818159
	H196	-0.209166549	10.382529208	3.391690767
	H197	6.266936939	2.370009347	4.741678675
	H198	0.918740526	2.971825890	3.574072057
	H199	4.635223198	9.479390497	3.495880336
	H200	2.904378648	9.437973434	3.913864132
	H201	3.724100708	7.498752991	5.667429427
	H202	-1.351435543	-1.383172801	13.774841181
	H203	-7.263821412	-1.758197782	-0.289859466
	H204	-5.530447134	-1.780075637	3.255177166
	H205	-9.130980338	-5.145860493	-2.428017548
	H206	-8.038332243	-6.444406091	-1.885933653
	H207	-7.314992822	-3.858842501	-3.380536617
	H208	-3.289280199	-6.678305801	-6.779862619
	H209	-8.094673042	-5.904148709	-4.673897837
	H210	-3.877685766	0.147702008	-2.564711215
	H211	-6.133308438	1.777395460	-1.263350446
	H212	-4.485458022	-5.757205700	-4.748000691
	H213	-4.893075187	-4.638464604	-7.441177410
	H214	-5.650663215	-7.192409430	-3.641594700
	H215	-6.361485114	-0.620527238	0.708145712
	H216	-6.072398818	-4.865537230	-2.632397673
	H217	-7.211250865	-7.691056592	-3.480684466
	H218	-6.286153365	1.531787947	-4.937196143
	H219	-3.521355166	1.709432183	-1.835670856

H220	-6.935586857	-6.178433759	-0.080964520
H221	-4.986335795	0.936325358	-0.236625909
H222	2.005545948	4.351507014	-2.925696668
H223	-0.059623166	2.899483767	-1.993592024
H224	-0.809315269	7.408920209	-5.185624419
H225	-2.193069280	4.755337095	-5.198827534
H226	1.989903113	5.090340028	-6.359872313
H227	6.302700072	6.996980698	-8.701171002
H228	3.901919563	7.511780653	-5.650152714
H229	2.820080969	6.873554061	-8.131023933
H230	4.790186165	4.846244137	-6.905320956
H231	5.081155930	5.426718797	-12.443497150
H232	3.025809705	2.325203504	-3.966103790
H233	1.958058814	2.475853640	-5.365927783
H234	6.951516597	6.581453797	-11.136817475
H235	2.699354720	5.142990250	-8.447168573
H236	0.102321494	4.169666265	-5.162209215
H237	-1.702699066	6.774034448	-3.803365188
H238	-1.819046342	4.773213800	-8.713381267
H239	-0.524854270	-1.081604359	-3.264445975
H240	-3.430495858	5.212429234	-8.120542248
H241	7.082522636	-3.195960643	7.937392562
H242	6.520150992	-2.676285685	6.492528414
H243	5.223723454	-2.643847248	-2.012363116
H244	4.686479824	0.029909829	-2.905228111
H245	7.747364617	-1.583925707	-2.114480127
H246	1.256168479	0.027063383	-4.786052002
H247	0.966998220	-0.125112020	-6.986849984
H248	-0.705617051	-0.419367007	-8.446281376
H249	2.476505671	0.348909377	-6.922275545
H250	-2.066828229	-0.644189156	-6.441561271
H251	-2.677729914	0.375528767	-7.786381440
H252	9.542750868	-2.972408923	-6.120499999
H253	10.321451287	-2.325869011	-7.581485233
H254	7.437570393	-3.474614108	-6.547366750
H255	6.375543796	-2.494202009	-7.555194885
H256	4.976337755	-4.248007531	-9.383620164
H257	1.452182876	-4.058794575	-6.582270140
H258	4.947825998	2.363795543	-9.872248243
H259	7.199106327	2.321867499	-5.437561259
H260	4.166470681	-0.941043948	-8.410507277
H261	3.751017136	-4.369758984	-3.109464282
H262	5.998771381	-4.740781618	-2.651842945
H263	2.707983507	-2.613593919	-4.651263249
H264	-3.565769101	3.176050443	-7.021331292
H265	8.576125592	-6.586054093	-3.287485057
H266	-3.898649398	0.726576129	-5.522427747
H267	3.973705468	-5.424673307	-4.503173957
H268	5.072333266	1.128379930	-5.733082930
H269	9.361051630	-5.027306347	-2.958894787
H270	3.775670607	-5.024095791	-8.410441399
H271	4.724462371	-1.895603775	-9.746013219
H272	-6.070990997	0.282421327	-3.847431392
H273	-5.257644848	2.781808871	-3.294290648
H274	7.152668309	3.456185641	-9.608813409
H275	-1.102494361	1.344572558	-5.335574314
H276	5.637276170	-3.837169490	-5.474892469
H277	3.003729519	-4.894341741	-6.638919666
H278	1.838715710	-4.104937651	-4.342581699
H279	2.664373344	-1.912153263	-6.972410437
H280	9.143475670	0.962187544	-2.144479464
H281	8.432490425	-0.864820422	-4.723129282
H282	11.162041823	-1.196697974	-5.650579190
H283	9.768996318	-0.621668492	-1.633460606
H284	9.103332357	1.441074488	-6.036054331
H285	-4.105049527	-9.658715918	1.227249482
H286	-5.097663050	-8.640776669	2.288010015
H287	-3.696243170	-7.646276676	6.473675092
H288	-6.224354690	-10.706807966	2.007981427
H289	-7.042666770	-9.656966909	1.053617490
H290	5.694863404	-3.999551678	1.817794396
H291	8.629232340	-3.380183320	1.483483636
H292	7.447946120	-6.450034615	-1.260111860
H293	6.322061229	-2.791958662	0.654545212
H294	9.123519920	-4.933909105	1.615105500
H295	2.126075325	-8.535476889	0.145644812
H296	7.063875065	-5.764797172	0.784227035
H297	-2.779599899	2.297297470	9.310260800
H298	-3.788095620	-0.449964996	10.228275472
H299	0.187832734	-7.613997367	-0.603432217

H300	2.638105700	0.191053187	5.161106491
H301	-1.356878115	-7.810416982	-3.835345968
H302	-3.783466198	-3.018955155	-1.689947936
H303	-0.433291417	-6.336214999	-3.537405469
H304	-1.096239887	2.144412295	7.209121623
H305	-1.460085404	-6.582682242	-1.085987668
H306	-2.226397812	0.968040777	6.526570372
H307	-3.767932417	1.149756003	8.377125302
H308	-1.838115573	-0.531633703	8.525260081
H309	-0.839285382	0.736218196	9.259745921
H310	-4.636434378	-2.559414752	-6.354398824
H311	-4.702868063	2.306588097	10.969922719
H312	-7.705417203	1.701006171	10.820369513
H313	-1.449648969	-3.906312105	-1.569307776
H314	-6.213885007	3.172395698	12.850444322
H315	-7.227739064	3.729207256	11.691373073
H316	-0.481125552	-0.653316838	6.342262564
H317	-0.057396936	-4.007689477	-3.585239696
H318	-4.808845858	-2.575388153	-3.914128078
H319	-6.885300570	-4.042190049	9.844935692
H320	-6.079892704	-5.851173096	7.196904626
H321	-5.145180705	-2.422353786	8.949480282
H322	-8.113714475	-4.485637087	8.987585019
H323	0.729963926	-10.705560293	-0.990567938
H324	-2.558965322	-2.929311816	7.296683473
H325	-5.974300863	0.482481091	5.637113792
H326	-3.193096241	-7.737845014	0.393180298
H327	-7.517847454	-1.154482529	6.362470078
H328	-7.458656583	-6.715804453	2.611882667
H329	-5.684355008	-4.955193997	2.128648240
H330	-7.479552831	1.232310063	12.509824038
H331	1.487531599	-1.089944668	5.357990495
H332	-2.162256794	-2.971012571	9.637098476
H333	-1.079379288	-3.406805389	7.346345211
H334	-2.216828948	-1.401320561	11.417569660
H335	0.653624003	2.780304743	6.400649620
H336	-7.457887826	-4.007045504	5.237349771
H337	-6.912456761	-3.589046656	6.868577823
H338	-7.884406694	-1.629826479	4.695382081
H339	-3.511677227	-5.511931052	-0.524297742
H340	-1.086155965	-3.592348691	-5.840774401
H341	-3.099693334	-2.920857537	-7.187548705
H342	-4.426028704	0.019443721	4.904818789
H343	-3.544628439	-2.960422113	10.349409839
H344	-5.560768797	-2.046832792	10.388849849
H345	2.097397414	2.360816477	5.659337783
H346	-7.319744596	-7.588208961	5.020000156
H347	-4.499518255	-5.244395013	6.715940478
H348	-4.952428787	-6.130820922	-1.385131695
H349	2.785889591	-8.520693996	-1.505239326
H350	3.910328261	-0.481614577	7.748983151
H351	3.748349711	-2.034699172	6.892872864
H352	-4.996627428	-9.331687443	7.473977560
H353	-6.225547419	-8.307657926	7.825151353
H354	-4.775267850	-5.667585556	11.346219745
H355	-5.494824485	-5.952700974	9.198942741
H356	-4.158552445	-8.320025038	11.113527865
H357	-3.153377139	-5.439311256	10.660739784
H358	4.357036855	-3.365284197	9.187325977
H359	11.800613702	3.126202745	-6.711845169
H360	-0.978226774	0.344157214	13.536373740
H361	0.632957155	-0.698547428	11.537440357
H362	9.740536246	2.142239856	-8.718926372
H363	8.899761643	3.956189644	-6.370271882
H364	8.863452851	4.397005397	-8.087916857
H365	-3.121421802	0.006496662	1.996079143
H366	3.238523667	1.272626254	-8.779735219
H367	-1.667760675	0.468196578	1.284986470
H368	0.690501079	-0.700997424	3.047707647
H369	-1.792530510	0.412770977	2.915743528
H370	-1.295735207	-0.511973880	-0.488623986
Mo371	-3.461362170	-3.600447743	4.196041860
N372	0.226217758	-0.924077411	2.152238306
N373	-2.102946943	-0.042214297	2.055135406
N374	-8.976229585	4.365130185	-2.567118411
N375	-6.421716018	6.655473469	-4.046367385
N376	-5.502001870	8.580385584	-2.216480246
N377	-5.290060209	7.266221589	0.281583496
N378	-3.094023225	5.619195321	-0.329130976
N379	-1.046332138	7.680680206	-0.611332889

N380	-1.050171510	9.245197578	1.844584771
N381	-1.263331990	7.321744156	3.890596207
N382	0.150339331	4.876611117	3.230001526
N383	2.913085328	5.116036212	1.978618683
N384	3.581536354	7.693315394	3.036912961
N385	-6.483540860	3.053475316	11.871299636
N386	-4.724659126	1.290526235	10.834199793
N387	-0.249887546	0.319441698	6.565574909
N388	1.340726324	2.035691165	6.264083872
N389	1.666431109	-0.066859750	5.326216595
N390	-1.952807869	-0.531694330	11.932700901
N391	-5.583212863	0.979624411	-4.440388638
N392	-6.675359069	-0.922481440	-0.229620639
N393	-3.195218234	1.474964177	-5.406902349
N394	-2.628982549	3.512615402	-7.232376255
N395	-1.777844831	5.620710085	-5.569898556
N396	0.650472222	4.698856585	-4.479213739
N397	0.782098990	0.476385947	-3.988452849
N398	-0.631021813	0.847412921	-2.312426827
N399	2.925607766	5.162722688	-5.968359903
N400	3.995871557	5.502429076	-10.582602604
N401	6.051048830	6.324901994	-10.741131777
N402	6.225588649	-2.962158938	7.430299214
N403	8.298811473	-4.330444330	1.680940784
N404	7.862893943	-5.587788383	-1.603952853
N405	6.275776961	-4.536143762	-3.619383003
N406	3.045178254	-2.857378146	-7.188169937
N407	4.156794286	-4.165007077	-8.789953367
N408	4.405867645	-1.864610316	-8.781561405
N409	5.411673105	-1.928328472	-2.740205911
N410	7.883895848	-0.661051524	-2.535089813
N411	9.398906292	-0.553877880	-4.826895207
N412	7.317985419	-2.777034826	-7.293411767
N413	9.842903094	1.448754529	-6.734330063
N414	-6.153354674	-10.156379313	1.149473141
N415	-4.051304683	-7.493746828	-0.109556213
N416	-1.785181249	-6.895452620	-2.020311099
N417	0.711536478	-8.130373557	-1.317845941
N418	-6.570309162	-7.189376753	-4.099146327
N419	-4.972588897	-5.255588223	-5.497438318
N420	-5.482805218	-8.492886223	7.143493137
N421	-5.098708769	-5.276465295	4.177506069
N422	-6.752108171	-6.427425487	3.283301789
N423	-4.597242679	-6.393316369	9.413911614
N424	2.138844428	-0.349647021	-1.268014809
N425	1.652311677	-0.946932503	-0.445037921
O426	-6.352220564	5.478412036	-2.094679395
O427	-3.368421053	7.833007780	-2.487086517
O428	-3.946997079	8.962778892	1.008522372
O429	-2.494324886	5.743782987	1.864802359
O430	0.385817708	6.063813521	0.077867194
O431	1.225147354	9.117847357	1.690169656
O432	-0.371374688	8.822546340	5.386833458
O433	0.048508623	4.321629146	5.451094273
O434	3.296214253	3.707024554	3.748471526
O435	5.538801564	6.731386265	3.664400735
O436	4.818868514	9.178069399	6.053533555
O437	-6.109623142	-0.520381356	11.222087727
O438	-2.215377202	1.743993318	12.120667119
O439	1.227657742	-1.473726275	13.328539294
O440	-2.822005157	3.286332064	-4.039438408
O441	-6.614893253	-0.800327655	-2.500188363
O442	-0.396209374	3.157204073	-6.927360162
O443	-0.600190056	0.188250351	-7.692593473
O444	-1.313218919	6.898874158	-7.421248909
O445	0.896437015	6.628879335	-3.280524102
O446	4.225615302	4.902187989	-4.115875042
O447	5.807984773	7.190456397	-6.307554871
O448	4.770390347	-1.766940919	10.389620144
O449	6.595165818	1.066784912	2.397518899
O450	8.392751108	-3.419521446	-1.138476463
O451	7.982527032	-4.667501327	-5.148934188
O452	5.400192261	-1.398725639	-4.966117340
O453	6.862453269	1.355047385	-2.931440248
O454	6.358184309	-0.521406404	-0.053786776
O455	11.268261652	0.078413039	-3.659554818
O456	11.285103149	0.119544332	-7.895698580
O457	8.079722863	-0.796813763	-8.144750178
O458	11.656906959	3.919852820	-8.590323358
O459	3.671437795	0.976607952	-7.955607620

	O460 -6.334020162 -7.740178410 -0.186751128<br>O461 -3.618438022 -6.934813351 -3.393309046<br>O462 1.055821153 -8.643363614 -3.507987279<br>O463 2.732916113 -11.093026345 -0.896970173<br>O464 -6.953375368 -4.502348264 -6.340420219<br>O465 -8.377427261 -3.498667433 -0.527409364<br>O466 -7.228673303 -5.263134406 0.214852983<br>O467 -2.114454593 -5.261693554 -7.652605733<br>O468 -2.809833316 -7.649624800 8.812736759<br>O469 -2.717296008 -7.458928922 12.274028601<br>O470 -5.847213046 -0.607066222 2.532903136<br>O471 -6.362680712 1.458135079 3.299335849<br>O472 -9.206355688 -4.061726350 7.696936621<br>O473 -9.977646761 -3.005171383 5.852294640<br>O474 -4.052401872 -3.031511788 6.226128238<br>O475 -5.078602777 -1.495962372 7.509663139<br>O476 -5.389939911 -2.453206293 4.080793073<br>O477 -2.566352217 -2.927268776 10.539261488<br>O478 -1.726392292 -2.859230760 7.828740395<br>O479 1.849129325 -0.325585915 -6.577062874<br>O480 -7.571260811 -4.744357098 9.792807011<br>O481 -5.245991053 -2.820795289 9.857893431<br>S482 4.760530306 -4.312296166 -0.412822716<br>S483 1.431441916 -3.917331193 -0.516642678<br>S484 -3.411877026 -3.279406090 1.840798093<br>S485 2.855909735 -2.174347515 1.987928322<br>S486 -1.227508021 -1.839859710 -0.163891426<br>S487 -1.140216507 -6.453918184 1.132122003<br>S488 -1.976474018 -1.710261539 4.407917015<br>S489 2.324479735 -5.663778786 2.434941030<br>S490 -1.900257313 -5.342957841 4.504945752<br>S491 1.264446980 -3.346990285 5.313633162<br>end

S2BH-Fe2-brNH-Fe6NH3 to S2BH-Fe2-brNH-Fe6, 35, S=1/2, TS

**Fe2-brNH-Fe6NH3-S2BH to Fe2-brNH-Fe6-S2BH<br>35, S=1/2<br>TS**	bm612bh135nh3distd_1_53445.11<br>C1 -6.973883209 5.834401218 -3.107166686<br>C2 -8.434341805 5.447640048 -3.368925445<br>C3 -4.999275373 6.979742642 -4.022960064<br>C4 -4.553815389 7.838753678 -2.845243904<br>C5 -5.168935857 9.349268084 -1.029561771<br>C6 -4.739403929 8.503363855 0.174126754<br>C7 -4.777203688 6.325099611 1.277084526<br>C8 -3.344836357 5.864701725 0.973560852<br>C9 -1.738519889 5.331308596 -0.814608568<br>C10 -0.699340623 6.387018250 -0.408952979<br>C11 -0.046876226 8.707365771 -0.332881686<br>C12 0.112610010 9.025678205 1.158990202<br>C13 -1.019795312 9.664708937 3.231912341<br>C14 -0.832838870 8.553962189 4.270549562<br>C15 -1.251832252 6.190193958 4.796342788<br>C16 -0.269119858 5.057381091 4.509497010<br>C17 1.050203111 3.772237416 2.839038039<br>C18 2.529827805 4.198835233 2.917697920<br>C19 0.685833548 3.242822986 1.438378241<br>C20 1.632184059 2.130248034 0.987775841<br>C21 -0.776015935 2.804895605 1.433247437<br>C22 4.286493867 5.624556053 1.903134724<br>C23 4.532316686 6.719097810 2.949843505<br>C24 4.617678241 6.112067595 0.467976822<br>C25 5.992005522 6.792806602 0.408289521<br>C26 4.548352151 4.954633709 -0.534235173<br>C27 3.834075237 8.839244081 3.889572985<br>C28 4.172957970 8.453660241 5.314420088<br>C29 -6.965729287 1.683064617 11.645164528<br>C30 -5.870699605 0.702083266 11.215465241<br>C31 -3.502238296 0.590616814 10.418614062<br>C32 -2.482169079 0.656706342 11.572482299<br>C33 -2.954181000 1.223129708 9.123497230<br>C34 -1.682617100 0.556178260 8.583569012<br>C35 -1.333975578 1.076358122 7.182844325<br>C36 0.912672988 0.769207301 6.051964779<br>C37 -1.009122767 -0.642503903 13.035824058<br>C38 0.400842126 -0.971757105 12.595660570<br>C39 -4.747943414 1.839526861 -3.589000548<br>C40 -3.487218123 2.282706336 -4.356289331<br>C41 -4.318009184 1.112361656 -2.295698265<br>C42 -5.428544649 0.916135067 -1.241915154<br>C43 -6.274073729 -0.350806386 -1.380160737

C44	-1.895990043	1.463673984	-6.077239897
C45	-1.567240762	2.779025479	-6.792360966
C46	-1.872904304	0.261631049	-7.033956612
C47	-2.439761987	4.824191877	-7.815640031
C48	-1.780351433	5.870314905	-6.908453134
C49	-1.053039268	6.478930311	-4.651410438
C50	0.256837979	5.934066179	-4.080206984
C51	1.920746815	4.139872882	-4.001192395
C52	3.138648648	4.796813300	-4.677679175
C53	2.022326661	2.631208144	-4.275983582
C54	0.994147318	1.785293857	-3.600471104
C55	0.101460423	1.992108097	-2.568326335
C56	-0.192667175	-0.046038975	-3.189078779
C57	4.022842152	5.619541021	-6.818011401
C58	4.646788835	6.866219415	-6.195943923
C59	3.422544481	5.941413075	-8.206249199
C60	4.384833739	6.011610230	-9.353825326
C61	5.662334067	6.530290529	-9.437846226
C62	5.036043388	5.701460514	-11.395299836
C63	5.555344200	-1.848856545	8.092228054
C64	4.847669810	-2.372179526	9.327147357
C65	4.467290478	-1.241181423	7.167294132
C66	5.044997656	-0.616580105	5.919192042
C67	5.459099044	0.728018583	5.901375540
C68	5.191243360	-1.365440420	4.737364489
C69	5.983823740	1.311857242	4.743598734
C70	5.727096149	-0.800797623	3.579397924
C71	6.112126467	0.541670688	3.580819222
C72	7.336914766	-4.711000456	0.639129899
C73	7.918725359	-4.510159838	-0.772885466
C74	6.084784753	-3.860752410	0.797188005
C75	8.405748821	-5.559793419	-2.955027733
C76	7.532095144	-4.873692683	-4.008594895
C77	5.346172313	-3.763626430	-4.425784899
C78	5.391932610	-2.255954601	-4.065149403
C79	3.942431592	-4.370733885	-4.197551408
C80	2.729612775	-3.701379441	-4.853743277
C81	2.531968369	-3.953049119	-6.358561588
C82	3.851374520	-2.978084926	-8.247611818
C83	5.458884481	-0.524124750	-2.337394829
C84	6.802144313	0.147393338	-2.653087522
C85	5.182392744	-0.320940552	-0.832034061
C86	9.223236850	-0.102618089	-2.426438753
C87	10.068202920	-0.187535402	-3.687052988
C88	10.172396147	-0.904559455	-6.009365985
C89	10.476437197	0.265358313	-6.978228669
C90	9.607860415	-2.101228090	-6.782609289
C91	8.274570823	-1.816882162	-7.459772374
C92	9.894493224	2.537288626	-7.703559233
C93	11.241625219	3.242031438	-7.667260549
C94	8.758784888	3.553823860	-7.386713946
C95	7.380435444	2.942618440	-7.515505771
C96	6.710483251	2.950765125	-8.749661493
C97	6.750346944	2.309314309	-6.430597711
C98	5.468042368	2.331820885	-8.906439103
C99	5.520472344	1.660758150	-6.574451073
C100	4.891740385	1.675268261	-7.817577623
C101	-5.075003665	-9.166914979	1.313033191
C102	-5.201319717	-8.080562180	0.259737222
C103	-3.938693099	-6.385733800	-1.045148923
C104	-3.097137305	-6.760927321	-2.271218855
C105	-0.822058707	-7.254794205	-3.056723208
C106	0.386814622	-8.082972536	-2.632587671
C107	1.924837636	-8.824251485	-0.893763075
C108	1.788740478	-10.333731245	-0.943424858
C109	-7.050946383	-5.824549452	-4.309734701
C110	-6.321829379	-5.144275269	-5.473390182
C111	-7.070142131	-4.894926514	-3.076136508
C112	-8.114658225	-5.363882493	-2.046002382
C113	-7.939323598	-4.631170006	-0.734821064
C114	-4.194139387	-4.666829357	-6.572516419
C115	-3.097283205	-5.610423108	-7.023816085
C116	-3.735935800	-3.205197594	-6.340200536
C117	-3.020322003	-3.120567916	-5.026436166
C118	-1.665214420	-3.473281352	-4.931598259
C119	-3.761242374	-2.918267022	-3.848116945
C120	-1.097382101	-3.733359083	-3.682138771
C121	-3.192244617	-3.182535544	-2.602792977
C122	-1.876093550	-3.649543894	-2.527919682
C123	-4.529151681	-7.367377507	7.126787317

C124	-3.896266877	-7.136644387	8.506492547
C125	-5.184073760	-6.088257277	6.584291615
C126	-5.611221019	-6.122336400	5.151533038
C127	-6.641814743	-6.847390162	4.589639150
C128	-5.795436579	-5.477029495	3.068314119
C129	-3.995492536	-6.140535307	10.712479677
C130	-3.568135632	-7.416795822	11.402355590
C131	-5.976182810	0.288588451	3.483801271
C132	-5.509716783	-0.161669382	4.881383453
C133	-5.773353523	-1.652042304	5.220793826
C134	-7.260641914	-1.885203338	5.574278023
C135	-7.590279742	-3.297313424	6.064887171
C136	-9.037605864	-3.458843869	6.579481477
C137	-4.906387556	-2.069194051	6.416691236
C138	-0.198112921	-3.501737389	2.299593524
Fe139	-1.770351032	-4.695307173	2.318163000
Fe140	0.830977289	-1.994448352	0.793249505
Fe141	1.622311255	-3.642442322	3.128898726
Fe142	0.519970752	-4.981995414	1.234145282
Fe143	3.049582901	-4.166162938	0.991837985
Fe144	-0.665063550	-3.469066949	4.252654745
Fe145	-1.361481346	-1.878605352	2.141388273
H146	-8.824215804	4.563258976	-1.572095261
H147	-8.443978948	3.500580968	-2.749712292
H148	-6.934578514	6.835115336	-4.903331295
H149	-8.551461863	5.203463432	-4.436800819
H150	-9.039608780	6.354459884	-3.196024389
H151	-4.388546560	6.064721010	-3.988304993
H152	-6.454125324	8.542573276	-2.557342754
H153	-4.747152151	7.512186524	-4.949358691
H154	-6.046869251	9.945468659	-0.748991001
H155	-5.871091115	6.901692989	-0.472212421
H156	-4.339588631	10.039921039	-1.234386100
H157	-1.389118121	4.367489167	-0.433658271
H158	-3.830172462	5.719435373	-1.011215972
H159	-5.446225445	5.454088580	1.307438549
H160	0.934414237	8.380734157	-0.699238141
H161	-1.889896831	7.907471689	-1.132060372
H162	-4.769817956	6.790695057	2.269595746
H163	-1.793853246	5.261992849	-1.908510730
H164	-1.945019168	9.161627245	1.367126940
H165	-0.338793427	9.624914286	-0.860753748
H166	-1.446656547	3.665705514	1.553677712
H167	-1.026526837	2.286218883	0.495917264
H168	-0.966265170	2.109265924	2.259211442
H169	0.791192669	4.076303894	0.728409258
H170	-1.968384883	10.173769185	3.454330971
H171	3.562179370	4.469867731	-0.541309821
H172	2.787833593	7.735120496	2.410191243
H173	6.044136261	7.686597575	1.046860107
H174	1.357485745	1.811961244	-0.025679870
H175	1.569495773	1.245228715	1.636935528
H176	6.226458227	-1.029296411	8.412664537
H177	4.745843158	5.314388386	-1.553619686
H178	6.207899514	7.100291256	-0.625430808
H179	5.302225622	4.188074317	-0.293315351
H180	6.786262121	6.100991192	0.730266085
H181	3.856553462	6.863739447	0.186618638
H182	4.968337650	4.803744178	2.170948853
H183	2.225464398	5.473801413	1.327657287
H184	2.679465433	2.467468254	0.954263521
H185	4.390234758	-1.013454559	-0.512844984
H186	4.814269990	0.708553548	-0.711774220
H187	6.331171174	0.072568266	0.718512322
H188	6.787395184	2.011773659	2.508545624
H189	5.810318373	-1.386187540	2.664342926
H190	4.850658374	-2.402600677	4.709181787
H191	5.354806982	1.338271980	6.802032324
H192	-0.195104571	5.490521402	2.502900554
H193	-2.252684502	5.731218324	4.822016571
H194	-1.018764216	6.556281663	5.802865303
H195	-1.743891874	7.195819051	3.001772235
H196	-0.201649605	10.379746946	3.389461397
H197	6.272545893	2.366067470	4.740620326
H198	0.925166927	2.971971816	3.578340045
H199	4.641651552	9.481609418	3.500011617
H200	2.909313808	9.436069679	3.917202026
H201	3.730592997	7.492731097	5.664858367
H202	-1.343591658	-1.387792185	13.774275653
H203	-7.256695219	-1.764078568	-0.289430506

H204	-5.514803788	-1.789641010	3.260422269
H205	-9.122896554	-5.149635054	-2.428418336
H206	-8.031147170	-6.448625038	-1.885658570
H207	-7.305933149	-3.862861246	-3.378740109
H208	-3.281170788	-6.683247403	-6.778435221
H209	-8.086773651	-5.907553758	-4.672576789
H210	-3.870085155	0.143248441	-2.566157259
H211	-6.125436017	1.772478412	-1.264745976
H212	-4.476714109	-5.762447004	-4.747484073
H213	-4.885798272	-4.644312167	-7.440498205
H214	-5.643097951	-7.197794358	-3.640725602
H215	-6.354680704	-0.625859551	0.707165985
H216	-6.064298924	-4.869811379	-2.629994832
H217	-7.204063948	-7.696169273	-3.481297924
H218	-6.280523830	1.526783826	-4.938294652
H219	-3.512088081	1.701832716	-1.837052737
H220	-6.928050385	-6.182282342	-0.080592831
H221	-4.979282317	0.931906620	-0.238683665
H222	2.014554628	4.347739666	-2.926696251
H223	-0.055447354	2.903102198	-1.999955983
H224	-0.801037302	7.403787627	-5.188515739
H225	-2.185637082	4.750673390	-5.199932954
H226	1.999576850	5.086624158	-6.360993231
H227	6.310826541	6.993494213	-8.701684504
H228	3.912569315	7.507446743	-5.650419474
H229	2.827563093	6.868996050	-8.132794414
H230	4.799042524	4.843284043	-6.907648768
H231	5.090466537	5.422865336	-12.444394138
H232	3.034780755	2.321249693	-3.965737184
H233	1.967511970	2.471813845	-5.366082441
H234	6.960154616	6.578760305	-11.137379141
H235	2.708911997	5.137841558	-8.447869167
H236	0.110230337	4.163668932	-5.160919267
H237	-1.694313294	6.770033282	-3.805605883
H238	-1.809209851	4.766645704	-8.713888412
H239	-0.527524910	-1.078008294	-3.265856076
H240	-3.421623014	5.206310387	-8.124003717
H241	7.091015417	-3.199677612	7.937334954
H242	6.528369553	-2.679091994	6.492914951
H243	5.233343290	-2.648043273	-2.014661754
H244	4.693539764	0.025643818	-2.903601959
H245	7.755840399	-1.587478388	-2.115505406
H246	1.262521714	0.021817479	-4.782191734
H247	0.974412006	-0.129277377	-6.986554399
H248	-0.698464157	-0.426767038	-8.444132218
H249	2.483880122	0.345505147	-6.923487975
H250	-2.060380157	-0.648611603	-6.440702473
H251	-2.670556092	0.371644824	-7.785522708
H252	9.551933095	-2.975575259	-6.119153358
H253	10.330548944	-2.330828736	-7.580755632
H254	7.447913974	-3.480856657	-6.550194796
H255	6.386226699	-2.501113142	-7.559303267
H256	4.982411970	-4.253310722	-9.386453733
H257	1.460479300	-4.063441053	-6.583062957
H258	4.956876133	2.358863990	-9.872726656
H259	7.207080755	2.318316433	-5.437437266
H260	4.176492668	-0.946452130	-8.409417936
H261	3.760675355	-4.374976743	-3.110625593
H262	6.008935597	-4.745224920	-2.653080822
H263	2.716027181	-2.618440500	-4.652804355
H264	-3.558042445	3.171708691	-7.020688983
H265	8.585544801	-6.590476588	-3.289002382
H266	-3.888146933	0.719975727	-5.519244155
H267	3.983695371	-5.428836472	-4.504844313
H268	5.079514794	1.126122974	-5.732972081
H269	9.370052227	-5.031578081	-2.960837765
H270	3.780825591	-5.029124701	-8.414341812
H271	4.731246870	-1.899912949	-9.746638369
H272	-6.063866213	0.279022291	-3.846284824
H273	-5.252134794	2.779081867	-3.292770690
H274	7.161939230	3.451051437	-9.609310863
H275	-1.093491746	1.339763852	-5.335342963
H276	5.645436056	-3.839918046	-5.474862952
H277	3.011755304	-4.899692870	-6.640167434
H278	1.847599544	-4.110781663	-4.344101341
H279	2.673146683	-1.917194590	-6.973209404
H280	9.150207142	0.959263271	-2.146458947
H281	8.441889141	-0.867335203	-4.724573167
H282	11.171545810	-1.199194955	-5.651869701
H283	9.776322656	-0.623784845	-1.633469002

H284	9.114338137	1.438478186	-6.036962167
H285	-4.094967201	-9.660081838	1.228483868
H286	-5.087178495	-8.641985324	2.290410778
H287	-3.690849574	-7.651977358	6.473003963
H288	-6.214211409	-10.709907165	2.008722088
H289	-7.032343627	-9.659226028	1.054873249
H290	5.702926005	-4.004377508	1.817680132
H291	8.639474487	-3.386057257	1.484622345
H292	7.456696811	-6.454792318	-1.261757624
H293	6.330629561	-2.798103620	0.652806701
H294	9.132267736	-4.939675359	1.614277584
H295	2.131947543	-8.539580985	0.150398595
H296	7.072876358	-5.770307600	0.784551555
H297	-2.771301587	2.294924345	9.308608113
H298	-3.777919097	-0.453676796	10.224944068
H299	0.191663782	-7.618174071	-0.603768839
H300	2.646622296	0.180949435	5.156166937
H301	-1.349310013	-7.817888445	-3.836317449
H302	-3.771961375	-3.031100471	-1.689956090
H303	-0.426348873	-6.343209558	-3.537782934
H304	-1.077862021	2.142774943	7.210840595
H305	-1.452765782	-6.587729453	-1.089045955
H306	-2.212018884	0.973157187	6.523682072
H307	-3.758719596	1.147390997	8.374701921
H308	-1.828830893	-0.533082557	8.521872834
H309	-0.830762697	0.733066926	9.259390880
H310	-4.629473177	-2.564568725	-6.354330626
H311	-4.693754871	2.302498328	10.967437056
H312	-7.697641839	1.694734983	10.823208267
H313	-1.438341523	-3.912545879	-1.569363633
H314	-6.203887940	3.169048318	12.849361111
H315	-7.219602784	3.724508039	11.690204537
H316	-0.479357114	-0.652328307	6.331876405
H317	-0.048170477	-4.018484219	-3.592817411
H318	-4.798668893	-2.581800770	-3.912771634
H319	-6.875900180	-4.047731205	9.844685333
H320	-6.072756386	-5.854580685	7.195677774
H321	-5.135565608	-2.428330413	8.950119953
H322	-8.103906236	-4.491417945	8.987156377
H323	0.738647083	-10.710987877	-0.993068257
H324	-2.547366607	-2.933550617	7.297178509
H325	-5.965423818	0.480467582	5.644591325
H326	-3.185765478	-7.745275357	0.390654039
H327	-7.509136569	-1.158468004	6.363319039
H328	-7.445493181	-6.714973168	2.609919050
H329	-5.671003053	-4.954551871	2.127646731
H330	-7.470316406	1.226251707	12.513411185
H331	1.494732290	-1.098825293	5.358307866
H332	-2.152945250	-2.974925784	9.637746992
H333	-1.067909892	-3.413560458	7.350935477
H334	-2.207015757	-1.405099865	11.416957426
H335	0.665605722	2.775433058	6.383223878
H336	-7.447756480	-4.011028857	5.238183658
H337	-6.902155536	-3.592757535	6.869224184
H338	-7.875808360	-1.633865825	4.696323787
H339	-3.503335488	-5.516675450	-0.527562100
H340	-1.078522154	-3.596504215	-5.844368618
H341	-3.093549656	-2.925045113	-7.189153590
H342	-4.420961442	0.016256492	4.904033677
H343	-3.535975501	-2.965495911	10.349541841
H344	-5.551336710	-2.050704755	10.388199365
H345	2.112361841	2.348083854	5.653001511
H346	-7.309599728	-7.587396039	5.016380058
H347	-4.490958263	-5.248282969	6.716997821
H348	-4.944798677	-6.136175218	-1.386225744
H349	2.791687405	-8.523268779	-1.501542600
H350	3.918828876	-0.485303632	7.749717013
H351	3.757324451	-2.039061435	6.894688207
H352	-4.992084586	-9.335641190	7.473388303
H353	-6.220186616	-8.311095328	7.825320411
H354	-4.765456374	-5.670393665	11.345095623
H355	-5.488220331	-5.957711900	9.199470017
H356	-4.152309840	-8.323172472	11.113709550
H357	-3.143902440	-5.443934065	10.658063981
H358	4.365924437	-3.370022020	9.186588516
H359	11.810362797	3.121945870	-6.714374210
H360	-0.971426742	0.340732434	13.537466161
H361	0.641052410	-0.702028968	11.538154084
H362	9.749537768	2.139389754	-8.720873161
H363	8.908884222	3.951245801	-6.370587539

H364	8.871083119	4.393632972	-8.087784770
H365	-3.936451471	0.141974322	2.159826343
H366	3.247569635	1.268507135	-8.780710974
H367	-2.861310319	0.963328502	1.220184370
H368	0.694445193	-0.684284099	3.005158551
H369	-2.748570449	1.054791449	2.839231904
H370	-1.252535507	-0.481025627	-0.455889478
Mo371	-3.435292551	-3.577855914	4.192524849
N372	0.160989558	-0.875608792	2.139105049
N373	-2.954839014	0.411681673	2.072971742
N374	-8.966623617	4.360263238	-2.564635815
N375	-6.415941443	6.654073115	-4.047654721
N376	-5.496640789	8.577125315	-2.216581439
N377	-5.280991394	7.262513821	0.280403247
N378	-3.081923551	5.618829455	-0.330586146
N379	-1.037341208	7.680890803	-0.614906840
N380	-1.042145549	9.241991611	1.842472707
N381	-1.254833281	7.318221560	3.890411626
N382	0.156795305	4.875921318	3.236082750
N383	2.920040805	5.112081852	1.982264894
N384	3.590410528	7.693242121	3.039020976
N385	-6.475954235	3.048513087	11.871218397
N386	-4.716208006	1.286430765	10.832422361
N387	-0.238427156	0.314882480	6.567613483
N388	1.352805238	2.028665576	6.257065985
N389	1.677289884	-0.078171437	5.330174293
N390	-1.943880719	-0.535372086	11.932132682
N391	-5.577231362	0.975990312	-4.440512261
N392	-6.667606440	-0.928399238	-0.230122733
N393	-3.186877974	1.470379205	-5.405327430
N394	-2.621200088	3.507090071	-7.233303708
N395	-1.769886511	5.615683218	-5.571562915
N396	0.659417716	4.694668845	-4.480304175
N397	0.788874758	0.473704727	-3.986568631
N398	-0.631782942	0.851936589	-2.317328544
N399	2.935380500	5.160223257	-5.969864282
N400	4.005000265	5.498109281	-10.583892037
N401	6.059911010	6.321391387	-10.741825794
N402	6.233945131	-2.965697454	7.430470297
N403	8.307539176	-4.336058968	1.681043070
N404	7.872612578	-5.592637597	-1.604620794
N405	6.284922548	-4.539514013	-3.620385956
N406	3.054077682	-2.862709993	-7.188462393
N407	4.163179169	-4.169951566	-8.792379488
N408	4.414992651	-1.869937597	-8.781361978
N409	5.419368791	-1.932376061	-2.740941906
N410	7.891913283	-0.664936454	-2.536743759
N411	9.408389094	-0.556712255	-4.827426258
N412	7.328472643	-2.782628830	-7.295521034
N413	9.852785326	1.445279962	-6.736303534
N414	-6.142850072	-10.158083576	1.151328989
N415	-4.043337670	-7.497901072	-0.110548835
N416	-1.778279993	-6.903085525	-2.021864092
N417	0.718496177	-8.133149946	-1.316727177
N418	-6.562587230	-7.193809148	-4.098596145
N419	-4.963959516	-5.259826466	-5.496170131
N420	-5.477960754	-8.496505237	7.143144829
N421	-5.084991548	-5.274328709	4.177239103
N422	-6.738882468	-6.426380571	3.281235963
N423	-4.589668791	-6.397161755	9.413009376
N424	2.144756224	-0.356877507	-1.249957537
N425	1.648831364	-0.947394325	-0.427045077
O426	-6.345668375	5.477324188	-2.096374501
O427	-3.361745449	7.834929550	-2.490521514
O428	-3.943075631	8.962585308	1.009340255
O429	-2.484176338	5.743315918	1.863895755
O430	0.397724871	6.066954416	0.075907357
O431	1.233232807	9.119274040	1.686130465
O432	-0.358305996	8.818903963	5.383856787
O433	0.049043983	4.318104377	5.457399356
O434	3.299928759	3.710833910	3.758723976
O435	5.548359857	6.730501948	3.664024632
O436	4.822439418	9.171573584	6.060766161
O437	-6.100274130	-0.524695693	11.219893535
O438	-2.206034190	1.740295288	12.118447867
O439	1.236487727	-1.475987747	13.329398481
O440	-2.820057222	3.287329587	-4.043208289
O441	-6.608068097	-0.805696347	-2.500440160
O442	-0.388457895	3.151616548	-6.928594332
O443	-0.593253269	0.182715118	-7.691931538

	O444 -1.306736904 6.894034526 -7.423242698<br>O445 0.905367682 6.626208381 -3.284166750<br>O446 4.234409273 4.900342917 -4.116355462<br>O447 5.819173826 7.185133913 -6.306544191<br>O448 4.776868851 -1.771934296 10.389947136<br>O449 6.601803789 1.061463426 2.397080281<br>O450 8.403345339 -3.425278100 -1.137557624<br>O451 7.990970174 -4.673918173 -5.150864020<br>O452 5.408983940 -1.402621521 -4.966677618<br>O453 6.869295519 1.351122249 -2.930610653<br>O454 6.367175432 -0.527412383 -0.053945124<br>O455 11.277301317 0.074333017 -3.658340211<br>O456 11.293679805 0.114904073 -7.897795394<br>O457 8.088666075 -0.800561044 -8.143790558<br>O458 11.665931076 3.917611909 -8.591882830<br>O459 3.680488778 0.972529469 -7.956573469<br>O460 -6.326033627 -7.744020834 -0.184995329<br>O461 -3.611076968 -6.942638450 -3.394754439<br>O462 1.063450965 -8.651316796 -3.506061033<br>O463 2.741723142 -11.094927443 -0.906786456<br>O464 -6.944465383 -4.506086241 -6.338878368<br>O465 -8.370484558 -3.503095382 -0.526537498<br>O466 -7.220175893 -5.266823720 0.214890234<br>O467 -2.107248428 -5.267193980 -7.653329090<br>O468 -2.803160446 -7.654206357 8.811039168<br>O469 -2.708839346 -7.463215830 12.272442788<br>O470 -5.841829204 -0.601839223 2.537335075<br>O471 -6.366127857 1.457790238 3.309556745<br>O472 -9.196635584 -4.066047644 7.696959310<br>O473 -9.966867972 -3.006664958 5.853404630<br>O474 -4.036048515 -3.024929064 6.223693181<br>O475 -5.070803771 -1.497977002 7.511941173<br>O476 -5.376082013 -2.454160546 4.085693794<br>O477 -2.557759097 -2.931815613 10.539457565<br>O478 -1.715053678 -2.863432206 7.829906181<br>O479 1.857636569 -0.330562814 -6.579598339<br>O480 -7.562086295 -4.749828416 9.792961645<br>O481 -5.237793610 -2.826090133 9.858510222<br>S482 4.772369246 -4.331398742 -0.412957320<br>S483 1.428283885 -3.909746239 -0.489920630<br>S484 -3.387648148 -3.245188899 1.847362063<br>S485 2.859630383 -2.194820202 1.989242726<br>S486 -1.241834949 -1.807999080 -0.125550436<br>S487 -1.127841047 -6.460616271 1.117832471<br>S488 -1.947437664 -1.667031607 4.391453383<br>S489 2.322832370 -5.676224737 2.427280715<br>S490 -1.885567363 -5.327508732 4.493821655<br>S491 1.266222887 -3.366690228 5.294641872<br>end

S2BH-Fe2-brNH-Fe6NH3 to S2BH-Fe2-brNH-Fe6, 35, S=1/2, product

**Fe2-brNH-Fe6NH3-S2BH to Fe2-brNH-Fe6-S2BH<br>35, S=1/2<br>product**	bm612bh135nh3distb.car_2<br>C1 -6.971172047 5.836645929 -3.104747185<br>C2 -8.430523819 5.435011486 -3.355129364<br>C3 -5.012589850 7.000753855 -4.034561282<br>C4 -4.566402619 7.862733826 -2.859355521<br>C5 -5.188469664 9.361429540 -1.036067266<br>C6 -4.750279952 8.516470004 0.164964234<br>C7 -4.764936836 6.332055733 1.254228016<br>C8 -3.332193583 5.874730367 0.955790352<br>C9 -1.696851298 5.342813504 -0.796747057<br>C10 -0.662801606 6.413201055 -0.415485596<br>C11 -0.047974214 8.750511111 -0.362883727<br>C12 0.117950692 9.057522694 1.131085142<br>C13 -1.009055457 9.661319183 3.216583304<br>C14 -0.810496304 8.550520273 4.253897173<br>C15 -1.273692695 6.198082518 4.804736886<br>C16 -0.307146315 5.045984307 4.536449830<br>C17 1.030956411 3.770870795 2.869767331<br>C18 2.504522588 4.219315210 2.954777763<br>C19 0.674438250 3.203447166 1.481486359<br>C20 1.666897488 2.123588337 1.046988288<br>C21 -0.748514636 2.651577849 1.473000436<br>C22 4.266130251 5.621802514 1.924639891<br>C23 4.521019575 6.727232570 2.959748271<br>C24 4.596182766 6.091373159 0.483717576<br>C25 5.969240796 6.772286377 0.409719520<br>C26 4.526973341 4.923026578 -0.506572608<br>C27 3.813845589 8.840429416 3.917600201

C28	4.150237779	8.443759030	5.340439834
C29	-6.946970504	1.665739732	11.649647913
C30	-5.850628826	0.691725306	11.204316616
C31	-3.485534883	0.595002325	10.393715690
C32	-2.465870740	0.658683858	11.549078801
C33	-2.939058093	1.237262314	9.103029430
C34	-1.667133210	0.571108812	8.565955952
C35	-1.288564132	1.107633543	7.179813793
C36	0.937743507	0.725091307	6.043815326
C37	-1.004638079	-0.641959958	13.022893464
C38	0.410462073	-0.963734583	12.593199391
C39	-4.756623447	1.836325095	-3.588943950
C40	-3.501468248	2.290573987	-4.361029340
C41	-4.317031752	1.100324426	-2.303304506
C42	-5.420993835	0.901595599	-1.242513303
C43	-6.266812246	-0.365961402	-1.377880405
C44	-1.894906699	1.457369432	-6.069808398
C45	-1.565095053	2.766940961	-6.793541681
C46	-1.877392682	0.251774617	-7.022854533
C47	-2.431839135	4.805763505	-7.831240727
C48	-1.778008344	5.858295741	-6.927314503
C49	-1.045960124	6.474930339	-4.674562954
C50	0.267684098	5.936643770	-4.104639837
C51	1.934332909	4.144982490	-4.010796001
C52	3.151872433	4.803164594	-4.684758632
C53	2.037363564	2.634741169	-4.279120207
C54	0.993269047	1.804406210	-3.608920532
C55	0.079180066	2.043185998	-2.602867628
C56	-0.232308538	-0.000068003	-3.190710081
C57	4.039412932	5.614860577	-6.827705722
C58	4.668489811	6.858316197	-6.203894791
C59	3.437101815	5.937344293	-8.215363306
C60	4.398710928	6.011063671	-9.363132944
C61	5.673961698	6.535183821	-9.447169127
C62	5.052092140	5.702212854	-11.404148528
C63	5.554219042	-1.854606217	8.089714737
C64	4.847840946	-2.384294787	9.322444761
C65	4.467382363	-1.243604190	7.167674879
C66	5.045337209	-0.621202516	5.918684091
C67	5.453477933	0.724953432	5.895352534
C68	5.194145966	-1.374351006	4.739978880
C69	5.974903063	1.305825383	4.734403156
C70	5.725854203	-0.812666735	3.578835772
C71	6.105655302	0.531304167	3.574598784
C72	7.342905967	-4.730277808	0.633068341
C73	7.929326408	-4.524126785	-0.776954596
C74	6.087588362	-3.883944265	0.795101790
C75	8.417899042	-5.565364625	-2.965451872
C76	7.543854822	-4.882586951	-4.021530057
C77	5.358465500	-3.765395410	-4.433655787
C78	5.400859528	-2.259488456	-4.069951124
C79	3.957298327	-4.378980108	-4.210801103
C80	2.742015667	-3.713305632	-4.866355170
C81	2.541889109	-3.962555366	-6.370952878
C82	3.855992104	-2.986630856	-8.260844583
C83	5.459441850	-0.534307960	-2.337650745
C84	6.799137783	0.145272173	-2.652399345
C85	5.182554437	-0.338145098	-0.832242653
C86	9.223029845	-0.094009733	-2.433869443
C87	10.076049516	-0.185602460	-3.689151096
C88	10.184768932	-0.900563290	-6.012964609
C89	10.485429173	0.264230008	-6.989031384
C90	9.623521173	-2.103111584	-6.778456711
C91	8.293042499	-1.824650266	-7.463517838
C92	9.904250422	2.538789991	-7.710573569
C93	11.251040529	3.245233298	-7.682307260
C94	8.768419281	3.553527292	-7.389455132
C95	7.390886731	2.941172734	-7.519010234
C96	6.724501802	2.943768271	-8.755105013
C97	6.758006974	2.312593235	-6.433073945
C98	5.482488633	2.324174712	-8.912359779
C99	5.527538298	1.665148542	-6.576990609
C100	4.902729398	1.672474908	-7.822308405
C101	-5.070319250	-9.170428439	1.319998817
C102	-5.202724509	-8.089124012	0.262411486
C103	-3.942392736	-6.408870781	-1.065793514
C104	-3.100938617	-6.789982674	-2.290333797
C105	-0.823068327	-7.285512624	-3.069091173
C106	0.383952766	-8.111546584	-2.634757215
C107	1.920019675	-8.839311882	-0.892932950

C108	1.797880621	-10.348436922	-0.980650229
C109	-7.055791364	-5.828272057	-4.308484901
C110	-6.324521825	-5.148411710	-5.471649720
C111	-7.068183827	-4.901649588	-3.071792377
C112	-8.115368553	-5.367247711	-2.042373530
C113	-7.941951474	-4.633992416	-0.730939930
C114	-4.197961813	-4.681730742	-6.577695843
C115	-3.100027814	-5.625457302	-7.027643676
C116	-3.733701297	-3.223848456	-6.334268809
C117	-3.008394968	-3.148447307	-5.024401095
C118	-1.651038736	-3.495889408	-4.941051108
C119	-3.739331216	-2.948972179	-3.839356113
C120	-1.065720874	-3.738963122	-3.696062777
C121	-3.152900896	-3.196283927	-2.598404299
C122	-1.828437303	-3.640494640	-2.531760649
C123	-4.536572507	-7.362115318	7.116228618
C124	-3.901151847	-7.139915196	8.496507662
C125	-5.168471227	-6.070044394	6.573932266
C126	-5.590022839	-6.092319211	5.138826551
C127	-6.614729325	-6.818152277	4.568280383
C128	-5.771037845	-5.437070219	3.056585904
C129	-3.985342226	-6.141685831	10.702305500
C130	-3.564716712	-7.417836151	11.396135293
C131	-6.116806101	0.309897019	3.555945509
C132	-5.549944729	-0.136489754	4.920263263
C133	-5.776185185	-1.633520386	5.244399345
C134	-7.254492382	-1.894430093	5.606408842
C135	-7.569176572	-3.312428536	6.086149498
C136	-9.017971802	-3.473793161	6.597892607
C137	-4.883997595	-2.048738010	6.420449128
C138	-0.190706094	-3.504346241	2.287724243
Fe139	-1.781957420	-4.680956573	2.294136010
Fe140	0.750820047	-1.973343360	0.816572470
Fe141	1.589223728	-3.658224910	3.132475962
Fe142	0.516883901	-4.981605667	1.195334299
Fe143	3.036891790	-4.142080088	1.001728038
Fe144	-0.701121966	-3.409101974	4.221171107
Fe145	-1.390491148	-1.978793834	2.148945842
H146	-8.801824590	4.556460369	-1.550417284
H147	-8.420335687	3.492133411	-2.726444809
H148	-6.948659705	6.836925505	-4.903270976
H149	-8.552041084	5.183289180	-4.420766027
H150	-9.045479925	6.335323300	-3.185779196
H151	-4.393709400	6.091526334	-4.007702611
H152	-6.474890987	8.532829799	-2.550997572
H153	-4.772819640	7.537284508	-4.962005989
H154	-6.069405269	9.951727345	-0.752863874
H155	-5.854585947	6.901178640	-0.498144076
H156	-4.364149156	10.057149095	-1.243460788
H157	-1.356818218	4.394446830	-0.367177404
H158	-3.778633108	5.770855229	-1.042329527
H159	-5.429647042	5.458000809	1.287106610
H160	0.937491001	8.456321720	-0.745083992
H161	-1.872496023	7.910642223	-1.158435908
H162	-4.757618804	6.798711974	2.246322735
H163	-1.732552173	5.230343963	-1.888499496
H164	-1.940696551	9.166171223	1.353126617
H165	-0.371757814	9.665528096	-0.877481131
H166	-1.483977088	3.427765537	1.734162024
H167	-0.990353938	2.252445975	0.475032457
H168	-0.846646329	1.804241756	2.167309897
H169	0.729799221	4.032039681	0.758928101
H170	-1.960802190	10.161537507	3.446667365
H171	3.550055240	4.419363550	-0.492148489
H172	2.759029378	7.723246640	2.450484942
H173	6.025719977	7.670825805	1.040751051
H174	1.425544118	1.811688816	0.023736535
H175	1.580279396	1.231211854	1.682764333
H176	6.223315321	-1.034829589	8.414497965
H177	4.698393056	5.278857828	-1.532320223
H178	6.175714720	7.070591997	-0.628663052
H179	5.300620959	4.173123396	-0.275624805
H180	6.766275405	6.083229166	0.730970336
H181	3.832343660	6.838203963	0.197057907
H182	4.945988962	4.802524101	2.204288958
H183	2.215236314	5.434120484	1.315735114
H184	2.714507212	2.460806971	1.057077343
H185	4.384446799	-1.027099282	-0.518019373
H186	4.821539483	0.693230053	-0.706036812
H187	6.337917910	0.047488807	0.715271399

H188	6.774082611	1.999982495	2.495825494
H189	5.809406922	-1.401552619	2.666078406
H190	4.858753261	-2.413401290	4.717471507
H191	5.346865357	1.339085439	6.793252199
H192	-0.220052223	5.482389284	2.537565155
H193	-2.282663259	5.757417267	4.822551476
H194	-1.047301472	6.570380212	5.810464453
H195	-1.743928947	7.196195504	3.000061114
H196	-0.196240770	10.382999891	3.371192243
H197	6.259208385	2.361282063	4.727186962
H198	0.919442852	2.980217994	3.621813333
H199	4.617672708	9.489553020	3.531907551
H200	2.887714799	9.435167505	3.946412759
H201	3.716032097	7.472534388	5.673682500
H202	-1.341512374	-1.392889463	13.754564439
H203	-7.251530950	-1.778686604	-0.287132893
H204	-5.557062299	-1.734457468	3.276007907
H205	-9.122331656	-5.150410675	-2.426912739
H206	-8.034602602	-6.452225683	-1.881025217
H207	-7.297471666	-3.867069957	-3.371090946
H208	-3.278226066	-6.697575015	-6.773693303
H209	-8.092642589	-5.906225890	-4.669437060
H210	-3.875983907	0.131231270	-2.584643989
H211	-6.119157146	1.756966683	-1.262078070
H212	-4.480122572	-5.784674731	-4.757190728
H213	-4.890256162	-4.653371114	-7.444729327
H214	-5.652536419	-7.210401030	-3.646000410
H215	-6.375468735	-0.625465301	0.712112686
H216	-6.062034135	-4.884298938	-2.625682317
H217	-7.215715090	-7.702931485	-3.484728587
H218	-6.287386265	1.516172650	-4.944540880
H219	-3.502432132	1.679367960	-1.847129611
H220	-6.927467427	-6.182864648	-0.075966043
H221	-4.965448649	0.914343005	-0.241915217
H222	2.024555496	4.355287980	-2.936345496
H223	-0.078401027	2.969306055	-2.058565692
H224	-0.797653107	7.398256359	-5.215857844
H225	-2.183463506	4.745835312	-5.213587427
H226	2.016646314	5.081265653	-6.371739893
H227	6.320251807	7.000707485	-8.710574752
H228	3.936138416	7.502807412	-5.659910248
H229	2.838335783	6.862490244	-8.140979985
H230	4.813371014	4.836638831	-6.920492769
H231	5.107816161	5.423643990	-12.453152580
H232	3.045217269	2.322632235	-3.956147169
H233	1.992522656	2.469656823	-5.369069921
H234	6.972586544	6.587959669	-11.145571639
H235	2.726035224	5.131385227	-8.457541014
H236	0.119547376	4.154017560	-5.165334476
H237	-1.685320892	6.767743587	-3.827807126
H238	-1.793574004	4.740998196	-8.723666506
H239	-0.585603482	-1.026087981	-3.259499406
H240	-3.410817854	5.185874031	-8.150882877
H241	7.092447438	-3.199320872	7.936825239
H242	6.537967572	-2.673577163	6.491967403
H243	5.238244351	-2.659589961	-2.021566699
H244	4.691000048	0.015356377	-2.900071943
H245	7.763271430	-1.587928883	-2.130578898
H246	1.258010063	0.017105596	-4.753880807
H247	0.978136872	-0.136040964	-6.986907623
H248	-0.705559008	-0.455794758	-8.424944417
H249	2.485936587	0.344151842	-6.935479707
H250	-2.070624496	-0.655557680	-6.427045839
H251	-2.674382541	0.364561111	-7.774708071
H252	9.565338447	-2.972772625	-6.109069277
H253	10.350053361	-2.338468864	-7.571338687
H254	7.472918734	-3.504786237	-6.577421665
H255	6.414707413	-2.531090375	-7.596460277
H256	4.967852825	-4.264964061	-9.416808552
H257	1.469834321	-4.070712408	-6.593046280
H258	4.974075439	2.346780376	-9.880189233
H259	7.213289249	2.324383906	-5.439271964
H260	4.201418893	-0.959127089	-8.406568600
H261	3.773964261	-4.388243743	-3.124097035
H262	6.027401973	-4.745934818	-2.661765811
H263	2.723960118	-2.630756970	-4.664392248
H264	-3.555099015	3.164077035	-7.021702280
H265	8.604279397	-6.594776608	-3.300450316
H266	-3.875735677	0.704971390	-5.498682851
H267	4.005233902	-5.435612443	-4.521900240

	H268	5.082341514	1.137178972	-5.733661005
	H269	9.379740888	-5.032279418	-2.968642748
	H270	3.754424881	-5.035270907	-8.453388986
	H271	4.732178758	-1.903985539	-9.758055060
	H272	-6.070266579	0.274246727	-3.844799719
	H273	-5.264613548	2.771524568	-3.285114886
	H274	7.178883788	3.439610779	-9.615980241
	H275	-1.090052392	1.333502128	-5.330739383
	H276	5.660407592	-3.835391036	-5.482207519
	H277	3.020635512	-4.908796716	-6.656126323
	H278	1.862357406	-4.126928495	-4.356614026
	H279	2.684103251	-1.924105496	-6.981273413
	H280	9.142389218	0.969929481	-2.164336354
	H281	8.449921551	-0.850155308	-4.734708443
	H282	11.185283575	-1.189112174	-5.654004709
	H283	9.774830186	-0.605006428	-1.633650317
	H284	9.136792522	1.444018898	-6.037272734
	H285	-4.089100109	-9.660667923	1.233436166
	H286	-5.077757934	-8.640397893	2.295008413
	H287	-3.702213675	-7.661629693	6.464227247
	H288	-6.203623434	-10.711937148	2.026488900
	H289	-7.026870040	-9.664419133	1.072182822
	H290	5.713339799	-4.027117632	1.818341611
	H291	8.642172893	-3.403387210	1.480133984
	H292	7.464802417	-6.465597803	-1.277973369
	H293	6.333827002	-2.821997607	0.652234733
	H294	9.135036595	-4.957615006	1.613026177
	H295	2.108808071	-8.573410903	0.159574225
	H296	7.082854318	-5.790801910	0.775731611
	H297	-2.757804390	2.308341563	9.293579523
	H298	-3.757262809	-0.448829165	10.192788214
	H299	0.179906958	-7.636830678	-0.610282921
	H300	2.650375403	0.109231734	5.116699853
	H301	-1.348853744	-7.854843428	-3.844775648
	H302	-3.723609435	-3.043112259	-1.680633124
	H303	-0.424970449	-6.377323052	-3.553940873
	H304	-0.975292054	2.156682367	7.239005504
	H305	-1.455535960	-6.590110623	-1.115375629
	H306	-2.165596903	1.068136183	6.512040065
	H307	-3.741130928	1.163669018	8.351492622
	H308	-1.820617023	-0.515958304	8.489596234
	H309	-0.821777287	0.730683604	9.254273731
	H310	-4.625404793	-2.580735097	-6.337158809
	H311	-4.684075970	2.298691707	10.948347364
	H312	-7.692933843	1.666128761	10.839959801
	H313	-1.373266163	-3.879312869	-1.573205159
	H314	-6.184604476	3.167259747	12.837714737
	H315	-7.211494464	3.706178980	11.679121936
	H316	-0.488775269	-0.658711408	6.347700478
	H317	-0.014443890	-4.019735373	-3.619714052
	H318	-4.781060565	-2.623025429	-3.893794257
	H319	-6.857487044	-4.066139780	9.843728891
	H320	-6.056721953	-5.828257692	7.182526249
	H321	-5.108392756	-2.447142189	8.951289961
	H322	-8.086246752	-4.511885242	8.988818009
	H323	0.749629495	-10.733373346	-1.020461215
	H324	-2.501338694	-2.920798184	7.285821291
	H325	-5.970435278	0.484714845	5.719551386
	H326	-3.185410374	-7.769442030	0.368051427
	H327	-7.511718094	-1.176065071	6.399793989
	H328	-7.410479207	-6.680793720	2.585463102
	H329	-5.641434236	-4.914205813	2.116699730
	H330	-7.435381053	1.206746353	12.526040093
	H331	1.494754849	-1.168782521	5.378911995
	H332	-2.134410466	-2.968056521	9.628120108
	H333	-1.043330979	-3.450999664	7.390306780
	H334	-2.193435769	-1.405059800	11.398798342
	H335	0.702172899	2.761835015	6.229194829
	H336	-7.426442707	-4.020094613	5.254194628
	H337	-6.879945373	-3.611390361	6.888414010
	H338	-7.876564400	-1.648698039	4.731798984
	H339	-3.505108212	-5.538057297	-0.553204997
	H340	-1.074091831	-3.625577745	-5.859224026
	H341	-3.096854240	-2.936693626	-7.184802841
	H342	-4.462350237	0.056453022	4.882911039
	H343	-3.514125421	-2.977579017	10.345009364
	H344	-5.527003810	-2.066884624	10.384572718
	H345	2.186748010	2.261215218	5.626342084
	H346	-7.282039096	-7.561277697	4.988578474
	H347	-4.464998343	-5.239378649	6.712938433

H348	-4.948588617	-6.160078870	-1.407335140
H349	2.793910671	-8.519316229	-1.480032199
H350	3.923135390	-0.485745822	7.751224328
H351	3.753835005	-2.038862812	6.897277066
H352	-5.030412351	-9.324637388	7.453137874
H353	-6.240122919	-8.284099261	7.817497558
H354	-4.747851242	-5.661319379	11.336112304
H355	-5.491088510	-5.963163964	9.201510222
H356	-4.158803595	-8.320005956	11.114260312
H357	-3.128078442	-5.452839678	10.638971646
H358	4.373428612	-3.385612882	9.181580438
H359	11.830832385	3.116923641	-6.737192057
H360	-0.976480190	0.338192737	13.531198844
H361	0.656357712	-0.695180842	11.536658579
H362	9.755737104	2.141548825	-8.727740413
H363	8.919531857	3.947293441	-6.372095890
H364	8.878767463	4.395849169	-8.087893267
H365	-4.062395367	0.380909591	2.277094575
H366	3.263658297	1.260104956	-8.789904191
H367	-3.154254844	1.526698109	1.504602577
H368	0.518443863	-0.581433937	2.985220747
H369	-3.063104716	1.386352379	3.131041219
H370	-1.301515734	-0.357348964	-0.141212676
Mo371	-3.445181743	-3.528621339	4.172399634
N372	0.009524715	-0.843252145	2.126128151
N373	-3.150982860	0.846319854	2.266178360
N374	-8.949103694	4.347853376	-2.541271384
N375	-6.425876302	6.660086556	-4.049569928
N376	-5.513962610	8.588384171	-2.222792491
N377	-5.272956321	7.266598856	0.259083632
N378	-3.046959606	5.650267603	-0.347467058
N379	-1.016143752	7.700865006	-0.640646036
N380	-1.036215518	9.243792817	1.825555757
N381	-1.249623897	7.316634193	3.885811793
N382	0.132110383	4.864802820	3.268293658
N383	2.897619210	5.113932141	2.004313780
N384	3.573475144	7.695076722	3.063720833
N385	-6.463666845	3.035731152	11.862827874
N386	-4.702262860	1.282757018	10.813034361
N387	-0.224352755	0.306093099	6.563351243
N388	1.381992343	1.993182332	6.195914445
N389	1.698827139	-0.152177538	5.362830864
N390	-1.931188445	-0.534022337	11.912402807
N391	-5.584587591	0.970512557	-4.439847654
N392	-6.658937926	-0.945321673	-0.227389357
N393	-3.185230990	1.466350824	-5.395888382
N394	-2.617584123	3.492953891	-7.241175543
N395	-1.763149334	5.607053723	-5.589922399
N396	0.672610965	4.695202571	-4.496063999
N397	0.778009502	0.488336075	-3.974821513
N398	-0.679125377	0.921054947	-2.347809348
N399	2.951391002	5.159540334	-5.978930995
N400	4.021542448	5.495289157	-10.593069900
N401	6.073216099	6.326741967	-10.750618680
N402	6.237671923	-2.966694719	7.425588315
N403	8.310515425	-4.353495344	1.677715732
N404	7.880303596	-5.601453285	-1.616168462
N405	6.299999876	-4.540146266	-3.629866364
N406	3.062895177	-2.870441561	-7.199035673
N407	4.154978024	-4.177362344	-8.814743043
N408	4.431509953	-1.880920385	-8.787483837
N409	5.425354415	-1.940969365	-2.744952322
N410	7.894199110	-0.662421961	-2.546520715
N411	9.419470299	-0.547512401	-4.833473940
N412	7.356182387	-2.802376478	-7.319218073
N413	9.867837123	1.446670499	-6.744190582
N414	-6.137053444	-10.163013035	1.166908172
N415	-4.045508986	-7.516258702	-0.125099287
N416	-1.781614368	-6.925667892	-2.040072517
N417	0.712365101	-8.151860898	-1.318118906
N418	-6.573358314	-7.200275398	-4.100955138
N419	-4.967758055	-5.276338240	-5.501693121
N420	-5.504275667	-8.475721408	7.130113670
N421	-5.066524186	-5.235149670	4.169997094
N422	-6.709582437	-6.391127373	3.262088403
N423	-4.587804782	-6.398055609	9.406151502
N424	2.105901376	-0.348129276	-1.185943514
N425	1.582594679	-0.944231718	-0.386777636
O426	-6.331949987	5.485191975	-2.098711874
O427	-3.370908908	7.874522059	-2.516270345

	O428 -3.965700276 8.983755876 1.006828184<br>O429 -2.488517600 5.731835277 1.857416691<br>O430 0.437090843 6.107715616 0.072172733<br>O431 1.239743255 9.166406267 1.651660531<br>O432 -0.319825468 8.815657720 5.359855392<br>O433 -0.013313364 4.304258455 5.492306939<br>O434 3.268200953 3.767324530 3.822208602<br>O435 5.554785634 6.751170093 3.647236781<br>O436 4.791147471 9.156734925 6.098566658<br>O437 -6.073773404 -0.536324112 11.205423448<br>O438 -2.189010516 1.742312619 12.094521117<br>O439 1.243655198 -1.461754362 13.333881825<br>O440 -2.856644769 3.314090068 -4.062823292<br>O441 -6.599485978 -0.824527055 -2.496682796<br>O442 -0.385485191 3.135185838 -6.933619556<br>O443 -0.598476699 0.162278769 -7.680017548<br>O444 -1.309479618 6.882438453 -7.446265520<br>O445 0.917875687 6.636848419 -3.317042259<br>O446 4.245011084 4.914248705 -4.119320688<br>O447 5.842432853 7.172294949 -6.312603122<br>O448 4.769235814 -1.784499943 10.384877455<br>O449 6.590628152 1.048831045 2.388108918<br>O450 8.419997532 -3.439586937 -1.134284425<br>O451 8.000220123 -4.692878933 -5.166680232<br>O452 5.420017594 -1.403379983 -4.968752612<br>O453 6.860108330 1.351548345 -2.919574068<br>O454 6.365661157 -0.556896885 -0.054038280<br>O455 11.287079202 0.066428681 -3.652853580<br>O456 11.296851674 0.109163434 -7.913041814<br>O457 8.103380489 -0.803251683 -8.138891258<br>O458 11.665110397 3.929632749 -8.605132459<br>O459 3.692685691 0.968535967 -7.962174515<br>O460 -6.330579202 -7.747959986 -0.170301692<br>O461 -3.614797020 -6.983528435 -3.411671322<br>O462 1.064556052 -8.687735898 -3.499858308<br>O463 2.757666307 -11.102292789 -0.979067877<br>O464 -6.944638683 -4.499333128 -6.330886123<br>O465 -8.375974320 -3.507217383 -0.522502030<br>O466 -7.220469254 -5.267764684 0.219147274<br>O467 -2.114206326 -5.281038830 -7.662780393<br>O468 -2.809096191 -7.662390792 8.796355849<br>O469 -2.701255904 -7.467420512 12.261733612<br>O470 -5.950677977 -0.554755128 2.587087202<br>O471 -6.588397203 1.451555452 3.418301823<br>O472 -9.182436167 -4.083539079 7.713072595<br>O473 -9.942853913 -3.014063086 5.871544308<br>O474 -4.005053547 -2.993306461 6.201461516<br>O475 -5.032939514 -1.494585814 7.524913252<br>O476 -5.382847675 -2.411273258 4.090664350<br>O477 -2.535722783 -2.935795207 10.531858658<br>O478 -1.674582554 -2.848093392 7.824903676<br>O479 1.866382537 -0.337703587 -6.592774792<br>O480 -7.540055050 -4.771644619 9.792218657<br>O481 -5.215897212 -2.845313114 9.857746491<br>S482 4.762650222 -4.344711215 -0.405229658<br>S483 1.410553836 -3.854210737 -0.497342262<br>S484 -3.442732989 -3.258404951 1.805626391<br>S485 2.793118752 -2.171620671 2.013594868<br>S486 -1.316158898 -1.717285132 -0.086210423<br>S487 -1.147517331 -6.438505114 1.082798259<br>S488 -1.967694232 -1.597321271 4.318584571<br>S489 2.298984925 -5.689843872 2.411282364<br>S490 -1.887134256 -5.262857263 4.483402855<br>S491 1.202935302 -3.398305157 5.300483212<br>end

S2BH-Fe2-brNH-Fe6 to Fe2-brNH2-Fe6

35, S=1/2

reactant

Fe(139) -2.411<br>Fe(140) -0.235<br>Fe(141) -2.675<br>Fe(142) 2.396<br>Fe(143) 3.042	bm602n2x2b226nhtf.car_2<br>C1 -3.831191839 7.196884028 5.086084330<br>C2 -4.882123683 7.021120982 6.188156869<br>C3 -3.212276665 8.483624308 3.081283848<br>C4 -1.755357395 8.834141683 3.367475311<br>C5 -0.098734761 9.634724894 4.970860047<br>C6 0.762264162 8.372392360 5.094200879<br>C7 0.815284578 5.958129449 5.463310524

Fe(144) 2.229	C8	1.155834571	5.483352552	4.049644351
Fe(145) -1.695	C9	0.469251294	5.367725082	1.706809128
	C10	1.648879659	6.143780908	1.100662139
	C11	2.766435030	8.271494323	0.793405656
	C12	4.146628969	8.015352373	1.413666734
	C13	5.461667152	7.905500153	3.470299006
	C14	5.994849714	6.487187660	3.699034590
	C15	5.402581047	4.156290323	4.202202379
	C16	5.252055030	3.061112409	3.148853377
	C17	4.191708768	2.355379124	1.008295234
	C18	5.112408801	2.639764630	-0.196798413
	C19	2.695605039	2.301537433	0.632986609
	C20	2.466823771	1.397344588	-0.581545310
	C21	1.845871105	1.850738128	1.824964617
	C22	5.633485245	4.154200057	-2.073155514
	C23	6.970870362	4.791536629	-1.668603090
	C24	4.815438626	5.059886207	-3.029082997
	C25	5.675694842	5.582172864	-4.187256193
	C26	3.593818250	4.307388836	-3.573524445
	C27	8.143898216	6.418629480	-0.306472586
	C28	9.294952859	5.482261763	0.000844821
	C29	6.334308450	-1.963433742	11.972192401
	C30	6.197750959	-2.816817731	10.708388572
	C31	6.698786624	-2.829809910	8.259424415
	C32	8.140261035	-3.287443005	7.961120114
	C33	6.197873885	-1.816532903	7.213901090
	C34	6.162513451	-2.378028253	5.789329508
	C35	5.502357373	-1.405183939	4.805114185
	C36	5.547973964	-1.601697327	2.285543872
	C37	9.563090662	-5.162387165	7.287061136
	C38	9.813385257	-5.433144110	5.819412191
	C39	-4.562706506	3.433695264	2.521730262
	C40	-4.384901802	4.027805433	1.110322150
	C41	-3.592013400	2.247550736	2.699424852
	C42	-3.383804552	1.782245879	4.155088988
	C43	-4.374727341	0.741860042	4.675491085
	C44	-5.228200670	3.711355652	-1.207215902
	C45	-5.166990857	5.152293159	-1.723803537
	C46	-6.399655861	2.928681885	-1.820531423
	C47	-5.691362948	7.500317711	-1.292250035
	C48	-4.274927770	8.089324656	-1.317327779
	C49	-1.911486604	7.795625497	-0.735177255
	C50	-0.995737375	6.977816607	-1.649607804
	C51	-0.715794315	5.108183242	-3.204321740
	C52	-0.415164071	5.840652429	-4.520811704
	C53	-1.411527597	3.790064332	-3.579117247
	C54	-1.708685295	2.899067885	-2.420166599
	C55	-1.302743133	2.888026965	-1.101145500
	C56	-2.653247853	1.227434764	-1.302762344
	C57	-1.389994505	7.239289357	-6.280855843
	C58	-0.117619430	8.077314126	-6.404313707
	C59	-2.645419278	8.134545982	-6.416549075
	C60	-3.043866041	8.521867788	-7.810128407
	C61	-2.281385080	8.896838526	-8.898416622
	C62	-4.433875276	8.907082898	-9.424738082
	C63	8.482192888	-5.167641731	-0.927137235
	C64	8.915154318	-6.032292054	0.241492735
	C65	7.434246294	-4.163274262	-0.387348192
	C66	6.955614610	-3.190363591	-1.439042091
	C67	7.589354512	-1.949481121	-1.631651631
	C68	5.851683953	-3.499331085	-2.252272336
	C69	7.132169502	-1.041425336	-2.591846103
	C70	5.393610310	-2.609360679	-3.224769943
	C71	6.031102016	-1.376259265	-3.387389085
	C72	2.469463537	-5.298982550	-6.466445080
	C73	1.711375159	-4.647322263	-7.637568091
	C74	2.307259275	-4.455046662	-5.208208827
	C75	-0.132007763	-4.894649622	-9.266180772
	C76	-1.167170607	-3.805185208	-8.969514708
	C77	-2.169401668	-2.403152549	-7.170446666
	C78	-1.326990465	-1.137172516	-6.871757692
	C79	-2.908463953	-2.927542535	-5.916849396
	C80	-3.809716321	-1.973375998	-5.124712971
	C81	-5.184786548	-1.654724390	-5.737408231
	C82	-5.673239693	-0.197575630	-7.706124532
	C83	0.679184825	-0.152455951	-5.876846293
	C84	1.354602695	0.469830534	-7.108224667
	C85	1.781691779	-0.480246436	-4.849541288
	C86	2.667446006	-0.054263014	-9.109865315
	C87	2.065185214	0.206038202	-10.481229561

C88	0.034193554	0.337037790	-11.815659745
C89	-0.160814138	1.739830991	-12.439097072
C90	-1.276964171	-0.454860567	-11.841009739
C91	-2.398279773	0.187280191	-11.033890974
C92	-0.250028681	4.161920909	-12.061123397
C93	0.688920317	4.672145344	-13.143935320
C94	-0.200275512	5.112291949	-10.829743234
C95	-1.204369432	4.725239145	-9.767354035
C96	-2.517229196	5.220189755	-9.812624315
C97	-0.875282874	3.821041103	-8.742117261
C98	-3.479280032	4.804490420	-8.888397576
C99	-1.830274322	3.371911412	-7.826891140
C100	-3.129062060	3.865946291	-7.915620162
C101	-4.770426769	-8.524051194	4.119531365
C102	-5.266433227	-7.119435761	3.818629537
C103	-5.066708152	-5.209327431	2.242484589
C104	-5.749399893	-5.187528080	0.869055585
C105	-5.409376322	-5.580092116	-1.532157421
C106	-4.775524096	-6.632063432	-2.439366793
C107	-2.908523048	-8.102936478	-2.969678797
C108	-3.603448873	-9.442860113	-3.102566469
C109	-9.014556410	-3.211012346	3.381430322
C110	-9.343014332	-2.243181109	2.238099746
C111	-7.712320669	-2.774520540	4.093331992
C112	-7.570032473	-3.476640513	5.456542202
C113	-6.187355689	-3.269961771	6.029152611
C114	-8.978054214	-1.613646878	-0.092769134
C115	-9.137620804	-2.440535771	-1.352500201
C116	-8.024049252	-0.402004950	-0.234411538
C117	-6.595860863	-0.863682376	-0.196343874
C118	-5.966031359	-1.346994074	-1.354876459
C119	-5.943165546	-0.997923889	1.041361962
C120	-4.741882152	-2.013614357	-1.260787207
C121	-4.720819649	-1.664270162	1.131102882
C122	-4.135596599	-2.201128301	-0.018744764
C123	0.761022414	-8.888370863	6.683143501
C124	2.246433550	-9.240590695	6.857457258
C125	0.493525205	-7.419611679	7.043362739
C126	-0.862097452	-6.893022967	6.700279015
C127	-2.073534027	-7.304468795	7.216796778
C128	-2.388574618	-5.514316984	5.954357358
C129	4.275309139	-9.140861052	8.179629292
C130	4.590506099	-10.608136212	7.994747238
C131	-0.028601055	-0.323451384	6.962030383
C132	1.124321088	-1.312564128	7.133878448
C133	0.687205799	-2.798544710	7.297446070
C134	0.140313385	-3.027809236	8.734773957
C135	-0.085815048	-4.487234889	9.135816184
C136	-0.467238865	-4.684162245	10.620936069
C137	1.904135852	-3.716426339	7.106056303
C138	0.446373012	-4.078311907	0.977557075
Fe139	-0.662743193	-5.213756507	2.233803710
Fe140	0.225858501	-2.348330474	-0.290347553
Fe141	1.984816325	-4.720872976	-0.154553863
Fe142	-0.572504707	-5.124876897	-0.283520083
Fe143	0.734646465	-4.313621930	-2.448329832
Fe144	1.805756882	-4.593577421	2.387643831
Fe145	0.271551633	-2.553613304	2.267040717
H146	-3.921909819	5.601490052	7.295842682
H147	-5.030744778	4.988046260	6.268058852
H148	-4.912535532	8.777270552	4.334266709
H149	-5.881019172	7.173224901	5.748086014
H150	-4.733928687	7.852939233	6.898347673
H151	-3.197017840	7.585506116	2.444148705
H152	-2.232401837	9.520981205	5.236650324
H153	-3.654928346	9.300340920	2.494619506
H154	-0.108981922	10.153810545	5.937870614
H155	-0.896806740	7.230861947	5.547790214
H156	0.400328927	10.281529218	4.237152049
H157	0.697977314	4.302393318	1.596195478
H158	-0.680599901	6.107138160	3.359183963
H159	0.178624933	5.209935212	5.955455346
H160	2.849612070	8.063413359	-0.280978465
H161	0.934860424	7.917352027	1.878315627
H162	1.761559357	6.028794446	6.013122744
H163	-0.446217134	5.579899834	1.138351976
H164	3.343677796	8.188162923	3.317537019
H165	2.506193553	9.330219012	0.933043355
H166	1.979086665	2.510322717	2.694148939
H167	0.781386527	1.856086355	1.544174054

	H168	2.090330338	0.816840838	2.111921199
	H169	2.395105557	3.325487172	0.356641361
	H170	5.357382642	8.381470625	4.456096921
	H171	2.948098733	3.926464112	-2.768900289
	H172	6.054288236	6.021816541	-0.257669065
	H173	6.524887356	6.190594252	-3.844630724
	H174	1.408042169	1.431929209	-0.864575681
	H175	2.708835370	0.347901976	-0.356541116
	H176	9.364243089	-4.590694894	-1.263583798
	H177	2.981669498	4.968152758	-4.204083656
	H178	5.060140597	6.203374041	-4.854638424
	H179	3.913864051	3.452221557	-4.192129190
	H180	6.076701331	4.744571626	-4.779579079
	H181	4.461209045	5.928426848	-2.443363445
	H182	5.909638331	3.230439156	-2.602274127
	H183	4.059303623	4.367495808	-0.621393573
	H184	3.055341996	1.716240853	-1.455051242
	H185	1.356770538	-1.125365535	-4.065759953
	H186	2.085215841	0.470572252	-4.387019831
	H187	3.717432653	-0.820016782	-5.025675075
	H188	6.021156684	0.315321607	-4.336743217
	H189	4.523601337	-2.852478041	-3.835317949
	H190	5.320207513	-4.441201143	-2.097378248
	H191	8.446920689	-1.675300548	-1.011317688
	H192	3.976323425	4.215368709	2.046559577
	H193	4.785488520	3.851675334	5.062202005
	H194	6.453137813	4.147849786	4.518519338
	H195	4.061943236	5.750199544	3.818605930
	H196	6.238267699	8.439579436	2.908345007
	H197	7.618575659	-0.070422643	-2.708889957
	H198	4.502010393	1.373460078	1.384116186
	H199	8.462865583	7.126872510	-1.089099736
	H200	7.933535349	6.996137217	0.607591015
	H201	8.995437883	4.487571108	0.406162619
	H202	9.701632508	-6.097119262	7.851712310
	H203	-4.513288860	-0.852939759	5.950843304
	H204	-0.875158584	-1.879986606	6.173502038
	H205	-8.299309581	-3.050394465	6.158767612
	H206	-7.780977294	-4.550531565	5.356207172
	H207	-7.709252816	-1.683818714	4.252944663
	H208	-9.421614766	-3.504679152	-1.174737303
	H209	-9.840288274	-3.056453534	4.092571415
	H210	-3.936289549	1.406424647	2.078117925
	H211	-3.436538521	2.649388397	4.837587900
	H212	-8.052285540	-3.243974284	0.953580524
	H213	-9.993217148	-1.201073188	0.081503404
	H214	-8.271542063	-4.861730686	2.363473722
	H215	-2.885402552	-0.202355474	5.798704661
	H216	-6.853880741	-3.000897915	3.441639016
	H217	-9.086306395	-5.229359386	3.747555090
	H218	-6.506994812	3.771204226	3.141851279
	H219	-2.620765623	2.549286931	2.284771179
	H220	-5.699233934	-5.037682645	5.324115275
	H221	-2.364672652	1.383771578	4.262618642
	H222	0.255562331	4.917520644	-2.727357609
	H223	-0.629431639	3.583735585	-0.609221932
	H224	-1.883092181	8.831767287	-1.099777206
	H225	-3.527172081	6.500747328	-0.197307831
	H226	-2.240599099	6.771022557	-4.371273672
	H227	-1.210710671	8.981425465	-9.043042066
	H228	0.204743507	8.549812042	-5.445655827
	H229	-2.519003498	9.025009595	-5.775496384
	H230	-1.383606769	6.473128995	-7.072281935
	H231	-5.330055106	9.031012946	-10.026041599
	H232	-0.767321258	3.275210215	-4.311770011
	H233	-2.349312424	4.030418287	-4.107506026
	H234	-2.947905644	9.423991603	-10.860237366
	H235	-3.495143569	7.573201149	-5.998575431
	H236	-2.530952551	5.690916754	-2.202639204
	H237	-1.463416026	7.800423073	0.270535971
	H238	-6.113755604	7.690721644	-2.288307627
	H239	-3.262306731	0.346333322	-1.114955975
	H240	-6.293766436	8.059651714	-0.565202998
	H241	8.647322103	-6.521162132	-2.456389062
	H242	7.424894135	-5.454694891	-2.655461605
	H243	0.084050303	-2.223115369	-5.772744792
	H244	0.036329491	0.627188232	-5.442855683
	H245	1.649754089	-1.418146698	-7.860360037
	H246	-3.108712994	1.599802710	-3.382147155
	H247	-5.080416255	2.270489073	-4.296794972

H248	-7.165815684	2.639607336	-3.597800864
H249	-4.110597893	2.552232768	-5.517584624
H250	-6.350159816	1.892104577	-1.446614325
H251	-7.352880445	3.379626369	-1.499636426
H252	-1.089185036	-1.492362249	-11.530639784
H253	-1.611167520	-0.473890949	-12.890040574
H254	-2.764218967	-1.612534491	-10.085729645
H255	-3.761584590	-0.239939273	-9.611233234
H256	-6.551031731	-1.056139272	-9.353959467
H257	-5.940292972	-1.580764199	-4.940405321
H258	-4.491441757	5.215112315	-8.923748809
H259	0.147025831	3.450984139	-8.630767426
H260	-4.867816855	1.691715177	-7.880309586
H261	-2.143814681	-3.304537056	-5.218751689
H262	-0.778731682	-4.006402114	-6.978270161
H263	-3.284154568	-1.037131273	-4.879695509
H264	-6.181390341	5.800102615	-0.074720366
H265	-0.660788287	-5.751372098	-9.703318566
H266	-6.036651974	3.003087399	0.705364138
H267	-3.498726260	-3.804832527	-6.229174382
H268	-1.566628408	2.626620251	-7.077133857
H269	0.532062162	-4.478518032	-10.036772918
H270	-6.679375325	-1.988618582	-7.895571046
H271	-6.034537873	1.266205514	-9.087879622
H272	-5.982322583	2.194979703	3.345653941
H273	-4.244820930	4.233800306	3.217427038
H274	-2.798687961	5.945490875	-10.579216603
H275	-4.285065644	3.249665269	-1.535836476
H276	-2.873185918	-2.117649118	-7.958341335
H277	-5.496015871	-2.477577805	-6.394928394
H278	-3.997479836	-2.471031887	-4.164143704
H279	-4.857576210	0.442392685	-5.955738303
H280	3.204342376	0.855622872	-8.803938083
H281	0.186670813	0.095175603	-9.676500572
H282	0.717325605	-0.155129256	-12.525445872
H283	3.402714252	-0.861676719	-9.232965033
H284	0.248865163	2.623247447	-10.660236062
H285	-4.561779763	-9.054265008	3.178158644
H286	-3.797160636	-8.388792561	4.636294561
H287	0.551949150	-9.041040430	5.613980678
H288	-5.321012226	-10.080485795	5.317892999
H289	-6.074786531	-8.672742725	5.674821221
H290	2.866923610	-4.939128671	-4.397699618
H291	4.265225392	-4.531438393	-7.066315142
H292	0.420062935	-6.247466600	-7.708681176
H293	2.707737957	-3.445351266	-5.376620724
H294	4.020162142	-6.066448625	-7.575891840
H295	-1.904748413	-8.288743000	-2.555821852
H296	2.060614805	-6.303805422	-6.277919479
H297	6.838356139	-0.919605182	7.255539992
H298	6.027479617	-3.697206446	8.275695799
H299	-3.116371174	-6.925181572	-1.206922418
H300	5.411959853	-2.028193256	0.288301951
H301	-6.490782175	-5.766135869	-1.516804680
H302	-4.212223916	-1.756661590	2.092753732
H303	-5.260220959	-4.605769103	-2.029624033
H304	6.128982627	-0.518849596	4.652816379
H305	-3.929882364	-5.581952473	0.052430093
H306	4.535457129	-1.062018106	5.209870577
H307	5.180781309	-1.520985353	7.517975073
H308	5.600319890	-3.324268689	5.780056420
H309	7.180092783	-2.604879904	5.433641129
H310	-8.234632241	0.291981906	0.591967962
H311	7.137582815	-1.334440904	9.749576540
H312	5.324272180	-1.583623016	12.189264641
H313	-3.189134270	-2.736742389	0.032132234
H314	8.197796019	-1.092756191	12.070678800
H315	6.974890801	-0.062054021	12.436416529
H316	4.668874182	-2.890883803	3.556484757
H317	-4.239987366	-2.393359980	-2.151210921
H318	-6.400981616	-0.580568800	1.941557307
H319	2.953506374	-6.668104709	10.412595182
H320	0.637709610	-7.291994288	8.129509826
H321	3.650666100	-4.986621328	8.617947767
H322	1.499212750	-6.650047928	10.964306846
H323	-4.313726922	-9.685953121	-2.275858210
H324	3.464951151	-5.088382955	5.485767714
H325	1.747085582	-0.989218415	7.976846367
H326	-4.074016032	-7.074022257	2.154684614
H327	0.880429405	-2.582613603	9.418357143

H328	-4.002687802	-6.402561111	6.978278350
H329	-2.902947908	-4.691008190	5.477515873
H330	6.597595662	-2.655347005	12.791399235
H331	4.677683787	-3.233199263	1.317430979
H332	5.484919562	-5.986768294	6.354666048
H333	4.114906033	-5.775397361	4.253637242
H334	7.417047310	-5.173978821	7.436168984
H335	6.179340134	0.316640925	2.742915652
H336	-0.902192657	-4.909113570	8.530465721
H337	0.810990801	-5.087311755	8.929694353
H338	-0.793886963	-2.458245837	8.854753358
H339	-4.124137359	-4.640762347	2.213020369
H340	-6.458837682	-1.225609881	-2.322571252
H341	-8.273977159	0.098237032	-1.182273183
H342	1.734022368	-1.220011842	6.219460208
H343	5.366613961	-6.124503456	7.897161947
H344	4.713045035	-5.077052686	9.739567634
H345	6.356492584	-0.151417940	1.126700050
H346	-2.314730559	-8.111210416	7.898490411
H347	1.247772149	-6.787637028	6.560379263
H348	-5.741062107	-4.748640327	2.966604463
H349	-2.781681436	-7.660831023	-3.969522517
H350	7.894394606	-3.614944284	0.447783287
H351	6.581677029	-4.736871617	0.011989494
H352	0.069630630	-10.764597980	7.134370549
H353	0.128424202	-9.772476312	8.434622876
H354	4.546792667	-8.872893381	9.213619114
H355	2.394397797	-8.307927629	8.735341390
H356	3.746090813	-11.303937588	8.216688323
H357	4.904089553	-8.538486966	7.505350864
H358	8.196827092	-6.849262976	0.496342034
H359	1.679428917	4.160622722	-13.189824487
H360	10.332346426	-4.443361489	7.619727697
H361	9.214758035	-4.811672515	5.109231074
H362	-1.269804223	4.166242962	-12.480153256
H363	0.819231243	5.105133981	-10.412951510
H364	-0.398321205	6.130862998	-11.194179795
H365	-4.864075686	3.965892877	-7.034459576
H366	2.344973602	-1.969330553	1.079490638
H367	-0.670613377	-0.069503470	1.100539659
Mo368	0.284428547	-4.297271564	4.608542039
N369	1.368314907	-1.631557022	1.097873105
N370	-4.856098258	5.763196790	6.911624729
N371	-4.035527626	8.267250367	4.262154429
N372	-1.472749250	9.374827981	4.575561079
N373	0.122612547	7.238515597	5.476824232
N374	0.210297455	5.689698399	3.101544146
N375	1.704729313	7.466982635	1.378701106
N376	4.191971766	8.013557180	2.772991431
N377	5.055475120	5.507532440	3.810593364
N378	4.461433733	3.318013590	2.081771157
N379	4.843392109	3.775683086	-0.902268829
N380	6.933889976	5.705692986	-0.665833782
N381	7.246178936	-0.823615229	11.812186707
N382	6.665052467	-2.229499029	9.588022940
N383	5.252846225	-2.053510811	3.513616438
N384	6.273551589	-0.477437525	2.090453947
N385	5.118690041	-2.299999108	1.222011512
N386	8.265276406	-4.575609285	7.554172362
N387	-5.959974915	3.018603248	2.716910892
N388	-3.879402424	-0.155377810	5.550140474
N389	-5.345367559	3.613290736	0.239054419
N390	-5.781427643	6.090264440	-0.963763327
N391	-3.302987968	7.386361447	-0.672537676
N392	-1.538067983	5.914256090	-2.306266581
N393	-2.578749898	1.830306536	-2.531218348
N394	-1.891392234	1.850264595	-0.413402360
N395	-1.425834454	6.630815972	-4.963568040
N396	-4.387905632	8.532588536	-8.152636553
N397	-3.182733298	9.138638117	-9.913743248
N398	7.900379457	-6.032544330	-1.956717384
N399	3.902366217	-5.443596456	-6.771655373
N400	0.656867262	-5.356541840	-8.138107218
N401	-1.342916418	-3.498100335	-7.669113730
N402	-5.189704292	-0.392760013	-6.483014302
N403	-6.395000104	-1.130806971	-8.353955581
N404	-5.398243420	0.957469577	-8.357599833
N405	-0.150797156	-1.312520702	-6.207047596
N406	1.716372607	-0.420100096	-8.077187958
N407	0.702760234	0.317182983	-10.527854161

	N408	-3.140783940	-0.678564136	-10.290218268
	N409	0.110758211	2.809580962	-11.650369686
	N410	-5.768189972	-9.263768134	4.896509225
	N411	-4.795494361	-6.571750766	2.679589927
	N412	-4.940545495	-5.523602423	-0.158355755
	N413	-3.598773107	-7.196494403	-2.068328649
	N414	-9.086722759	-4.608926596	2.934956924
	N415	-8.721730043	-2.473095051	1.047146772
	N416	-0.114906125	-9.804371467	7.439096626
	N417	-1.082405394	-5.773750040	5.896504579
	N418	-3.015999947	-6.423191383	6.735147628
	N419	2.859648717	-8.861520718	8.011297230
	N420	-0.076772731	-0.160891112	-2.252563200
	N421	0.059669961	-0.949801694	-1.464874147
	O422	-2.832653606	6.466475073	4.966554937
	O423	-0.883360811	8.612240918	2.508740548
	O424	1.983308471	8.420756938	4.871635862
	O425	2.245234855	4.948700291	3.786627818
	O426	2.498966606	5.579114815	0.391706730
	O427	5.156636058	7.861275977	0.707954859
	O428	7.213295152	6.296082431	3.813340552
	O429	5.838003366	1.980339235	3.356349704
	O430	6.036137423	1.865019521	-0.493218156
	O431	8.033692706	4.496994436	-2.240703979
	O432	10.469307943	5.803687018	-0.111566284
	O433	5.661233191	-3.941444627	10.772968485
	O434	9.096065140	-2.504131490	8.105238226
	O435	10.617936304	-6.259867578	5.419661335
	O436	-3.461514596	4.806954342	0.807872981
	O437	-5.580472090	0.733345179	4.335064824
	O438	-4.566127002	5.430808201	-2.775834775
	O439	-6.309231786	2.954324288	-3.258349166
	O440	-4.082149753	9.164188001	-1.904315419
	O441	0.194761667	7.299355264	-1.763713797
	O442	0.621889413	5.656492534	-5.168846482
	O443	0.478339525	8.284093381	-7.448004763
	O444	9.949720824	-5.871802760	0.873727021
	O445	5.518286862	-0.519355704	-4.341591252
	O446	2.044967180	-3.548422487	-8.113386739
	O447	-1.785057792	-3.277193627	-9.915424886
	O448	-1.733039812	-0.023232132	-7.239521727
	O449	1.592451010	1.682832156	-7.166251762
	O450	2.900436865	-1.117996067	-5.475564391
	O451	2.786922747	0.321888246	-11.479815459
	O452	-0.496589173	1.851924737	-13.627276961
	O453	-2.636858691	1.401423864	-11.096066884
	O454	0.404413903	5.597431023	-13.888795343
	O455	-4.091709927	3.367836069	-7.017960840
	O456	-6.027801993	-6.521668560	4.617985072
	O457	-6.959099584	-4.907551781	0.735137651
	O458	-5.328428433	-6.912521877	-3.516518909
	O459	-3.393183411	-10.223169555	-4.016663260
	O460	-10.123458567	-1.289597500	2.402547480
	O461	-5.848264002	-2.255024773	6.626626016
	O462	-5.293522498	-4.258327340	5.813376603
	O463	-9.021668295	-2.000178439	-2.487506629
	O464	2.860807808	-9.903923718	5.998970063
	O465	5.699611984	-11.031627467	7.697008300
	O466	-1.096409171	-0.807272292	6.323480685
	O467	0.042489615	0.848077925	7.332850510
	O468	0.096081726	-5.653458475	11.243278350
	O469	-1.332133053	-3.905530875	11.112049960
	O470	1.805484279	-4.620373016	6.165905556
	O471	2.903091371	-3.601524114	7.841465781
	O472	-0.284422658	-3.083131766	6.285694175
	O473	6.012089960	-6.251568596	7.149092586
	O474	4.340400864	-5.279321017	5.062532502
	O475	-4.401467148	1.854922699	-4.889100676
	O476	2.314672762	-7.233224892	10.901889853
	O477	4.175088212	-5.652302003	9.139134075
	S478	0.527047052	-4.300292289	-4.724497010
	S479	-1.308720797	-3.556670164	-1.687398073
	S480	-1.584203211	-3.548994955	3.421211749
	S481	2.186051322	-2.930314105	-1.482239415
	S482	-1.303405988	-1.258905341	1.226454656
	S483	-1.941838411	-6.411142778	0.902325726
	S484	1.907248117	-2.775348584	3.740987013
	S485	1.006768287	-6.302392588	-1.388231861
	S486	0.869654767	-6.300612396	3.460802424
	S487	3.604419751	-5.201149808	1.273367452

	end

TS

	bm602n2x2b226nhte_1_53148.76
Fe(139) -2.306<br>Fe(140) -0.203<br>Fe(141) -2.676<br>Fe(142) 2.575<br>Fe(143) 2.584<br>Fe(144) 2.197<br>Fe(145) -1.701	C1 -3.841462336 7.369867265 4.830261183<br>C2 -4.894292392 7.251768867 5.939477332<br>C3 -3.204970770 8.573074377 2.781517378<br>C4 -1.749059866 8.941180659 3.051215305<br>C5 -0.090007583 9.785895171 4.630488080<br>C6 0.771473468 8.527595851 4.793400480<br>C7 0.823359050 6.128306577 5.248152106<br>C8 1.151982913 5.611102580 3.846848923<br>C9 0.450234391 5.434869638 1.510809037<br>C10 1.636150856 6.182711348 0.881721230<br>C11 2.775168843 8.286760447 0.507275873<br>C12 4.154873388 8.051526596 1.135518105<br>C13 5.474010480 8.023487782 3.193161723<br>C14 5.994518655 6.609789985 3.473360675<br>C15 5.384187854 4.300297473 4.035988795<br>C16 5.247958534 3.177847986 3.009528972<br>C17 4.174042739 2.400860575 0.901410444<br>C18 5.097787574 2.638141478 -0.310376364<br>C19 2.678884357 2.344877263 0.522403767<br>C20 2.452318730 1.406046979 -0.666599655<br>C21 1.826183887 1.943829581 1.730139742<br>C22 5.628422717 4.082623868 -2.234688812<br>C23 6.967458436 4.725650284 -1.845816882<br>C24 4.821503550 4.958730770 -3.226853941<br>C25 5.695061285 5.432052506 -4.396031038<br>C26 3.598995342 4.196110814 -3.750624880<br>C27 8.138543417 6.408029953 -0.558363001<br>C28 9.294610813 5.489039608 -0.223619050<br>C29 6.336115359 -1.535916690 12.037394449<br>C30 6.195500768 -2.437405487 10.808692345<br>C31 6.702325928 -2.548191713 8.362693207<br>C32 8.143777312 -3.011778859 8.076211455<br>C33 6.187871215 -1.577014712 7.284287228<br>C34 6.157954321 -2.186132754 5.878503924<br>C35 5.459876052 -1.262726360 4.873946547<br>C36 5.546374308 -1.525590718 2.361936280<br>C37 9.563343740 -4.912817817 7.469997286<br>C38 9.808553792 -5.227068981 6.010325423<br>C39 -4.556642085 3.525392987 2.399402963<br>C40 -4.377732980 4.064816829 0.966725879<br>C41 -3.584566146 2.348546579 2.625982861<br>C42 -3.382963885 1.938063963 4.100016177<br>C43 -4.368607936 0.909469688 4.651180718<br>C44 -5.224866433 3.668345611 -1.339804403<br>C45 -5.164244418 5.090883877 -1.904577238<br>C46 -6.399409618 2.867671002 -1.923197946<br>C47 -5.687685609 7.453581770 -1.550903670<br>C48 -4.270658099 8.038747032 -1.586643139<br>C49 -1.909166106 7.756677772 -1.000745322<br>C50 -0.993314378 6.897982381 -1.875227828<br>C51 -0.721275091 4.986472637 -3.378071203<br>C52 -0.420353731 5.678709481 -4.716062957<br>C53 -1.421183712 3.660180851 -3.714951600<br>C54 -1.726715915 2.809361565 -2.528029423<br>C55 -1.319419144 2.840190712 -1.209628242<br>C56 -2.666264908 1.173728358 -1.356484035<br>C57 -1.392689762 7.024695761 -6.514950577<br>C58 -0.125471941 7.865485246 -6.665293494<br>C59 -2.652222681 7.908415513 -6.682701602<br>C60 -3.050944128 8.239529855 -8.090928141<br>C61 -2.288168475 8.582782646 -9.189440704<br>C62 -4.440114429 8.565839479 -9.718982536<br>C63 8.484726798 -5.189797521 -0.746938253<br>C64 8.923222156 -6.012632482 0.449661209<br>C65 7.437064458 -4.168024521 -0.239183501<br>C66 6.958535593 -3.233493617 -1.324739166<br>C67 7.601973033 -2.005783878 -1.566274107<br>C68 5.855025781 -3.566032741 -2.130218920<br>C69 7.154147828 -1.131086840 -2.561690602<br>C70 5.408291865 -2.711622481 -3.139105678<br>C71 6.051338628 -1.487725925 -3.345326743<br>C72 2.471650847 -5.515333178 -6.273954396<br>C73 1.709538344 -4.906738091 -7.463344507<br>C74 2.314855599 -4.629526872 -5.037209824<br>C75 -0.130787407 -5.216652474 -9.080118828<br>C76 -1.160876705 -4.112763955 -8.824185977

	C77	-2.174144493	-2.656732418	-7.076474655
	C78	-1.326489092	-1.381224816	-6.828312028
	C79	-2.905858113	-3.134065345	-5.800622945
	C80	-3.808414193	-2.152010222	-5.046022695
	C81	-5.182354658	-1.857272629	-5.673186821
	C82	-5.668078920	-0.463420048	-7.691389844
	C83	0.687319222	-0.366287292	-5.874138490
	C84	1.361677957	0.206556114	-7.128965957
	C85	1.794300802	-0.659070912	-4.839513241
	C86	2.669250067	-0.388596046	-9.110850711
	C87	2.063658373	-0.166826152	-10.487356553
	C88	0.027459555	-0.079181179	-11.817404892
	C89	-0.167223103	1.301839805	-12.490465463
	C90	-1.287186669	-0.865584084	-11.808138751
	C91	-2.400770648	-0.192461528	-11.016929408
	C92	-0.256245657	3.735395940	-12.197514703
	C93	0.677309740	4.212054119	-13.298448913
	C94	-0.204120893	4.724398277	-10.997480881
	C95	-1.207315435	4.376756854	-9.919915106
	C96	-2.517197768	4.877084377	-9.980554291
	C97	-0.879841822	3.508349324	-8.863838694
	C98	-3.479461976	4.501020734	-9.039886050
	C99	-1.836842205	3.095986085	-7.931696982
	C100	-3.132316282	3.595528283	-8.035299841
	C101	-4.772141211	-8.371407413	4.412282669
	C102	-5.269907691	-6.980404337	4.061567760
	C103	-5.069910112	-5.124196839	2.421949648
	C104	-5.752367444	-5.154167822	1.048824114
	C105	-5.416921844	-5.626381462	-1.337656744
	C106	-4.776751450	-6.699058327	-2.213363960
	C107	-2.907208028	-8.187862858	-2.690766025
	C108	-3.577255374	-9.546724080	-2.749495114
	C109	-9.011896320	-3.081546592	3.491315437
	C110	-9.337103634	-2.153477342	2.315988429
	C111	-7.709732713	-2.623606298	4.186772089
	C112	-7.567597836	-3.279267844	5.572439180
	C113	-6.182833618	-3.057001775	6.136163312
	C114	-8.972641798	-1.606264624	-0.036154684
	C115	-9.123347186	-2.477318068	-1.268483941
	C116	-8.022546363	-0.395229491	-0.215569398
	C117	-6.594760675	-0.857496099	-0.168160590
	C118	-5.973283643	-1.386181824	-1.310985356
	C119	-5.936690405	-0.950409063	1.070409006
	C120	-4.759188139	-2.068782375	-1.196676932
	C121	-4.724439383	-1.632103219	1.180384973
	C122	-4.154099192	-2.227372965	0.051147201
	C123	0.758948472	-8.668199508	6.998958840
	C124	2.245658538	-9.007239402	7.181115409
	C125	0.480360091	-7.191774240	7.325227066
	C126	-0.874377172	-6.677271154	6.956533888
	C127	-2.088020091	-7.062278734	7.491345313
	C128	-2.395603371	-5.333980226	6.143103678
	C129	4.274677347	-8.852860365	8.496250403
	C130	4.597199728	-10.324286347	8.365506271
	C131	-0.070286678	-0.117475784	6.958553784
	C132	1.099989351	-1.074383264	7.197788727
	C133	0.686781266	-2.557352809	7.432079528
	C134	0.149646663	-2.731462311	8.879778171
	C135	-0.089805425	-4.175507205	9.327749062
	C136	-0.475062107	-4.322058141	10.818095401
	C137	1.913240642	-3.464856288	7.254682233
	C138	0.449532046	-4.038778062	1.123810170
	Fe139	-0.684242936	-5.052044057	2.420244504
	Fe140	0.198243695	-2.393645503	-0.335600145
	Fe141	1.992009265	-4.707472543	0.005006330
	Fe142	-0.573706152	-5.125649668	-0.128783457
	Fe143	0.775521147	-4.369732332	-2.280922198
	Fe144	1.807821783	-4.477569641	2.538819639
	Fe145	0.178120080	-2.510736667	2.311453599
	H146	-3.957699474	5.870223880	7.111494528
	H147	-5.066884699	5.223332089	6.104444382
	H148	-4.898419689	8.940600573	4.023254874
	H149	-5.891141690	7.401821008	5.494397366
	H150	-4.734873946	8.107326724	6.618259002
	H151	-3.186311102	7.642345828	2.194971740
	H152	-2.221549198	9.664996991	4.906169820
	H153	-3.651012814	9.354879971	2.152122918
	H154	-0.098095037	10.333928116	5.581715302
	H155	-0.884382870	7.409466407	5.313065586
	H156	0.405817245	10.411330263	3.876389407

H157	0.662162887	4.363615183	1.425572073
H158	-0.688947927	6.215040386	3.152993343
H159	0.190127357	5.396413027	5.768129852
H160	2.858082495	8.033925960	-0.557636911
H161	0.941520268	7.986386427	1.604931751
H162	1.772847586	6.211607298	5.790360741
H163	-0.465016049	5.646084369	0.941917030
H164	3.355988667	8.304426902	3.031785975
H165	2.523255004	9.351855853	0.600871221
H166	1.889442209	2.689370366	2.536078647
H167	0.772487140	1.839641654	1.427771851
H168	2.138412368	0.966971871	2.128000676
H169	2.380781283	3.360347600	0.213644591
H170	5.375667384	8.535194455	4.162117495
H171	2.949376662	3.843953378	-2.937386431
H172	6.048869428	6.030536394	-0.503594648
H173	6.545294115	6.045448713	-4.064762749
H174	1.389087045	1.411544634	-0.936221341
H175	2.723220684	0.368672278	-0.421277099
H176	9.365604613	-4.624702288	-1.105654640
H177	2.991599801	4.838215860	-4.403806595
H178	5.090217425	6.034984529	-5.089078533
H179	3.917643218	3.319413036	-4.338607535
H180	6.095393780	4.572686586	-4.956972196
H181	4.472753864	5.851721059	-2.676316746
H182	5.902282409	3.138811505	-2.729538765
H183	4.057580890	4.359024660	-0.788794958
H184	3.025119611	1.716511485	-1.553411412
H185	1.378815583	-1.288781433	-4.038371493
H186	2.093976874	0.305585123	-4.403866443
H187	3.731524673	-0.980974998	-5.015478005
H188	6.043484188	0.172243957	-4.348825323
H189	4.539263119	-2.973600078	-3.742785250
H190	5.313382274	-4.497112746	-1.948299746
H191	8.461618405	-1.715870634	-0.955439321
H192	3.944406507	4.282555207	1.891326440
H193	4.755129685	4.015395565	4.894339598
H194	6.430740172	4.300171251	4.363446924
H195	4.053642943	5.888433977	3.590966644
H196	6.253488600	8.533969303	2.612545873
H197	7.650204122	-0.169538775	-2.714170551
H198	4.478207900	1.429789510	1.310730339
H199	8.452511033	7.085439154	-1.369981223
H200	7.934227045	7.021018670	0.333412372
H201	9.006055232	4.513478503	0.231270362
H202	9.705892700	-5.826344473	8.067650448
H203	-4.503799281	-0.646648835	5.969284644
H204	-0.872363169	-1.754938005	6.265993889
H205	-8.296322825	-2.828239403	6.260763596
H206	-7.776031874	-4.357091840	5.506053267
H207	-7.705945328	-1.528853878	4.308934292
H208	-9.398569921	-3.538067579	-1.057725065
H209	-9.838758241	-2.903307773	4.195810337
H210	-3.925954310	1.486106562	2.032768203
H211	-3.452155066	2.826356877	4.753417134
H212	-8.051068682	-3.200567667	1.065193126
H213	-9.989402849	-1.191201351	0.121252621
H214	-8.272271667	-4.766545244	2.526796308
H215	-2.873916023	-0.012567408	5.792243263
H216	-6.850379417	-2.870751291	3.543515556
H217	-9.084781116	-5.087357797	3.921840267
H218	-6.501982794	3.882645698	2.999467790
H219	-2.610495419	2.630002403	2.203783887
H220	-5.700820574	-4.846374934	5.482470249
H221	-2.360392666	1.555166023	4.230241685
H222	0.247819932	4.804959275	-2.893382274
H223	-0.641877773	3.551400439	-0.745868966
H224	-1.880161174	8.772250018	-1.418182112
H225	-3.524493833	6.480684354	-0.423100325
H226	-2.237149129	6.628455466	-4.585617662
H227	-1.217895357	8.672987633	-9.333559178
H228	0.192886880	8.365920162	-5.718781628
H229	-2.525799519	8.828042291	-6.083052685
H230	-1.380973855	6.226156159	-7.273664720
H231	-5.336895876	8.657351720	-10.325689730
H232	-0.769260257	3.124333137	-4.425244911
H233	-2.355628979	3.886639883	-4.256605375
H234	-2.952744937	9.043174477	-11.168450982
H235	-3.499569182	7.364954233	-6.236789967
H236	-2.535100879	5.608671027	-2.403728717

	H237	-1.461611844	7.811069088	0.002964628
	H238	-6.105889116	7.613665210	-2.554573396
	H239	-3.260071166	0.288266999	-1.142710266
	H240	-6.287581378	8.041325740	-0.843918683
	H241	8.641300783	-6.601087286	-2.225025464
	H242	7.420413486	-5.540176150	-2.460627707
	H243	0.082136121	-2.428165898	-5.688485952
	H244	0.050226137	0.434935900	-5.472927114
	H245	1.649882059	-1.706458112	-7.810798377
	H246	-3.126162393	1.478969742	-3.446748156
	H247	-5.083514546	2.114288002	-4.374357547
	H248	-7.174642725	2.537065868	-3.691854948
	H249	-4.114562014	2.357759533	-5.603855875
	H250	-6.353870446	1.846686081	-1.509092632
	H251	-7.350850191	3.333359666	-1.619186347
	H252	-1.102522885	-1.890156359	-11.458270010
	H253	-1.628305051	-0.926434936	-12.853028386
	H254	-2.755053145	-1.954417227	-9.994333816
	H255	-3.742671531	-0.560410346	-9.563926439
	H256	-6.530602007	-1.376198010	-9.317217589
	H257	-5.940361019	-1.756818633	-4.881651764
	H258	-4.490241021	4.913518051	-9.088583686
	H259	0.141391442	3.136447704	-8.744213646
	H260	-4.879803777	1.429336281	-7.914894278
	H261	-2.136778647	-3.478389082	-5.090262471
	H262	-0.799589139	-4.265251514	-6.824480876
	H263	-3.284924900	-1.206134030	-4.837752770
	H264	-6.185416084	5.793706005	-0.281002785
	H265	-0.664698333	-6.088851346	-9.479771344
	H266	-6.025159357	3.020552316	0.595934820
	H267	-3.492210243	-4.026565786	-6.074698802
	H268	-1.576585868	2.376368456	-7.155495307
	H269	0.536857309	-4.839845890	-9.867956767
	H270	-6.647021647	-2.273031803	-7.836038738
	H271	-6.025243541	0.952060982	-9.123347130
	H272	-5.977179456	2.316889620	3.271620663
	H273	-4.242049443	4.355530178	3.060445210
	H274	-2.795757292	5.576465830	-10.771912192
	H275	-4.283221606	3.197874430	-1.658697011
	H276	-2.883857356	-2.405472897	-7.870823219
	H277	-5.489203902	-2.703734364	-6.302542869
	H278	-3.995962919	-2.611219253	-4.065925940
	H279	-4.861878427	0.231863250	-5.958451955
	H280	3.210663268	0.527766022	-8.833758014
	H281	0.187894457	-0.256033556	-9.672937937
	H282	0.707668144	-0.599833128	-12.509426885
	H283	3.401641480	-1.201636899	-9.208842399
	H284	0.262012661	2.248887391	-10.748162797
	H285	-4.554455694	-8.933896569	3.492518143
	H286	-3.803100577	-8.218209548	4.931180055
	H287	0.551130496	-8.846993619	5.933686390
	H288	-5.324760226	-9.891735445	5.652423979
	H289	-6.084357951	-8.474934369	5.964191183
	H290	2.884756847	-5.090793967	-4.220269001
	H291	4.263131090	-4.765108200	-6.908035919
	H292	0.422189811	-6.512908592	-7.475396708
	H293	2.712586806	-3.625001449	-5.239869491
	H294	4.029034237	-6.325954237	-7.343248385
	H295	-1.893369311	-8.336186838	-2.286654599
	H296	2.066295958	-6.514706655	-6.051689498
	H297	6.813734291	-0.669302704	7.296609192
	H298	6.033718116	-3.416565218	8.416053079
	H299	-3.123320315	-6.957903993	-0.963949878
	H300	5.464503571	-2.016865293	0.377938256
	H301	-6.496670751	-5.820153070	-1.311153848
	H302	-4.216082129	-1.698349568	2.144770669
	H303	-5.279838587	-4.667979916	-1.867951561
	H304	6.051666414	-0.355572245	4.705167321
	H305	-3.931674695	-5.566412266	0.242560241
	H306	4.482842429	-0.944051050	5.273118646
	H307	5.167501042	-1.284130242	7.580534571
	H308	5.622542870	-3.147607715	5.901746068
	H309	7.179350789	-2.393468401	5.520774980
	H310	-8.235651395	0.319088434	0.592730003
	H311	7.153222243	-1.001549558	9.796503260
	H312	5.327119075	-1.146453719	12.242767926
	H313	-3.220598159	-2.782005521	0.131090141
	H314	8.202380827	-0.669354998	12.110801320
	H315	6.983798562	0.378316030	12.429096526
	H316	4.631543033	-2.771761913	3.648162492

H317	-4.277714259	-2.491673769	-2.078812355
H318	-6.385917712	-0.496598811	1.957040726
H319	2.942918004	-6.298225502	10.645694221
H320	0.613250818	-7.036775961	8.409641884
H321	3.657184332	-4.678199542	8.799920624
H322	1.490611779	-6.264898820	11.207405947
H323	-4.286930490	-9.757283553	-1.913738947
H324	3.476935314	-4.872187170	5.673867501
H325	1.701994243	-0.700759649	8.035459987
H326	-4.071784083	-6.990364722	2.402949840
H327	0.896392457	-2.269157715	9.545808597
H328	-4.015254537	-6.172719454	7.205433442
H329	-2.904668092	-4.532887762	5.625007607
H330	6.600303567	-2.193977098	12.882991859
H331	4.643607831	-3.150466741	1.418459556
H332	5.487305534	-5.763905107	6.563930404
H333	4.103685162	-5.618914843	4.463948006
H334	7.418279711	-4.915304573	7.626207754
H335	6.230326015	0.377328199	2.778158773
H336	-0.909208198	-4.608693162	8.733976205
H337	0.803378764	-4.787539274	9.142985937
H338	-0.777969018	-2.147992611	8.984165064
H339	-4.127881972	-4.556064952	2.374500291
H340	-6.469504775	-1.295585607	-2.280728221
H341	-8.277825963	0.079469131	-1.175286626
H342	1.724960679	-1.015449332	6.291010849
H343	5.364169476	-5.846956825	8.112332147
H344	4.716652378	-4.749468629	9.923177416
H345	6.394142903	-0.123636536	1.179028647
H346	-2.339751825	-7.834859042	8.208589638
H347	1.234533501	-6.565914018	6.831891937
H348	-5.746858216	-4.640727038	3.129397607
H349	-2.804402326	-7.792017175	-3.712333989
H350	7.899706341	-3.591654118	0.575560478
H351	6.584993339	-4.725627772	0.181699870
H352	0.078935963	-10.540527068	7.491736059
H353	0.128943167	-9.518335907	8.768521215
H354	4.544592513	-8.552577853	9.521387836
H355	2.390189264	-8.010652303	9.025492176
H356	3.753697035	-11.016452790	8.602727065
H357	4.904195380	-8.271900742	7.803754262
H358	8.203539919	-6.815182594	0.743155978
H359	1.675161311	3.714213241	-13.320206842
H360	10.333159200	-4.183032020	7.778108499
H361	9.210262702	-4.619088911	5.288542315
H362	-1.278387982	3.722175851	-12.610251583
H363	0.815751928	4.729973910	-10.582234523
H364	-0.403299996	5.730072646	-11.395108470
H365	-4.863297706	3.739815837	-7.153035233
H366	2.252001338	-1.668707040	1.079678495
H367	0.077533935	-0.521477359	1.121569815
Mo368	0.281611588	-4.109675124	4.760138124
N369	1.239865970	-1.454807659	1.084231568
N370	-4.887639672	6.024696686	6.714090826
N371	-4.029891266	8.414275746	3.969296318
N372	-1.464094757	9.512683968	4.244348722
N373	0.133725187	7.410804013	5.227470764
N374	0.201736683	5.794889680	2.898853528
N375	1.708973886	7.512508044	1.123902126
N376	4.204562166	8.113794941	2.493151687
N377	5.045931185	5.641283900	3.604300421
N378	4.444708280	3.395381111	1.943692338
N379	4.833705589	3.749996879	-1.054229832
N380	6.928782417	5.681767053	-0.883707084
N381	7.253808295	-0.406814871	11.834199695
N382	6.675962709	-1.900148219	9.668990084
N383	5.226915837	-1.942853913	3.595414651
N384	6.316219190	-0.433293602	2.148757732
N385	5.108828096	-2.231642571	1.305904697
N386	8.267418943	-4.314746288	7.720494519
N387	-5.952455643	3.113717572	2.609262750
N388	-3.871079401	0.043190352	5.554854660
N389	-5.335108841	3.615706896	0.109430409
N390	-5.780781507	6.054149997	-1.177676879
N391	-3.300535652	7.352564310	-0.922854824
N392	-1.539885146	5.820809582	-2.504775002
N393	-2.591619441	1.733897970	-2.605993747
N394	-1.907018891	1.826880078	-0.488007582
N395	-1.426738035	6.463359983	-5.177131791
N396	-4.394099327	8.233018563	-8.435310799

N397	-3.188707579	8.784079119	-10.214407159
N398	7.897608703	-6.091293367	-1.741567969
N399	3.905466117	-5.664795609	-6.571004524
N400	0.653284033	-5.634482706	-7.932134284
N401	-1.350034609	-3.770865048	-7.534285982
N402	-5.186473425	-0.621634897	-6.461297717
N403	-6.376746528	-1.422106656	-8.315303071
N404	-5.397888587	0.674583098	-8.373304305
N405	-0.151417209	-1.534418261	-6.155022234
N406	1.718204120	-0.717523579	-8.066002084
N407	0.700619757	-0.056043498	-10.531961515
N408	-3.135379508	-1.030054491	-10.233775289
N409	0.112768565	2.399080015	-11.742907454
N410	-5.773412628	-9.089229678	5.205800362
N411	-4.796282885	-6.470884102	2.905779713
N412	-4.943295121	-5.518353696	0.031074546
N413	-3.599929427	-7.250783866	-1.822333176
N414	-9.085358655	-4.492331075	3.090976948
N415	-8.715564661	-2.423383872	1.133853430
N416	-0.110571463	-9.574283502	7.773586403
N417	-1.089655034	-5.595173191	6.100819145
N418	-3.027850737	-6.203967493	6.964969575
N419	2.859041421	-8.582743155	8.318320760
N420	-0.090693284	-0.210857278	-2.312484018
N421	0.038011236	-1.022343676	-1.545100824
O422	-2.856871307	6.617525315	4.734852526
O423	-0.879043645	8.702224017	2.195353345
O424	1.990616654	8.563703247	4.558663298
O425	2.238444744	5.066289250	3.592287072
O426	2.478856322	5.592049800	0.185512416
O427	5.162552345	7.861861619	0.434989367
O428	7.209794691	6.414334664	3.613433793
O429	5.848931152	2.110346922	3.238534590
O430	6.015716270	1.848007362	-0.582340719
O431	8.034214763	4.398368671	-2.393067118
O432	10.466449996	5.811136048	-0.362224523
O433	5.644112096	-3.551566636	10.916866351
O434	9.100012081	-2.223760405	8.188417606
O435	10.609060237	-6.065460599	5.626825586
O436	-3.456154041	4.836733814	0.641166203
O437	-5.575611360	0.887587726	4.314455546
O438	-4.560817164	5.332696847	-2.964424233
O439	-6.309894379	2.836656084	-3.360120231
O440	-4.076607551	9.101825922	-2.195066367
O441	0.201161836	7.204835432	-1.988088470
O442	0.609934372	5.466500607	-5.366066127
O443	0.468804244	8.043224088	-7.716068218
O444	9.962493000	-5.831518837	1.068986438
O445	5.545821557	-0.665764239	-4.333528296
O446	2.037962070	-3.824308738	-7.978771162
O447	-1.766240847	-3.603955640	-9.787965333
O448	-1.723650561	-0.280743810	-7.242261020
O449	1.603964203	1.416179121	-7.226594104
O450	2.916727057	-1.306182662	-5.449327156
O451	2.781824657	-0.078649546	-11.491099298
O452	-0.511157718	1.371814923	-13.679017169
O453	-2.643248472	1.018363906	-11.120916200
O454	0.382632911	5.099712079	-14.084272542
O455	-4.097684827	3.133752389	-7.121429640
O456	-6.036531203	-6.357936219	4.836291915
O457	-6.963858278	-4.886235980	0.907260385
O458	-5.326550689	-7.004784722	-3.285167346
O459	-3.343028757	-10.373176016	-3.615508050
O460	-10.116281122	-1.194102565	2.449793250
O461	-5.838139150	-2.027152088	6.704772374
O462	-5.291704829	-4.053238863	5.945683083
O463	-9.016969375	-2.069520357	-2.416578678
O464	2.862796995	-9.696252367	6.344454311
O465	5.713105868	-10.750912502	8.099765966
O466	-1.117362190	-0.652374701	6.338149199
O467	-0.016360882	1.073099863	7.272215920
O468	0.087765261	-5.268759458	11.475164597
O469	-1.341632008	-3.527147503	11.279681551
O470	1.836112874	-4.368514792	6.312024598
O471	2.903534813	-3.331440938	7.999750944
O472	-0.293430388	-2.900600291	6.443125254
O473	6.011110608	-6.000161045	7.369602406
O474	4.345777953	-5.090947625	5.247535080
O475	-4.399401082	1.682227557	-4.949116821
O476	2.306401589	-6.849208158	11.153262709

	O477 4.179390467 -5.336276295 9.333362811<br>S478 0.543387031 -4.451012506 -4.526270508<br>S479 -1.274772842 -3.642740707 -1.663509051<br>S480 -1.603024654 -3.366166947 3.564916583<br>S481 2.226079979 -2.967876654 -1.375639494<br>S482 -1.277413895 -1.176872262 1.127351990<br>S483 -1.947493510 -6.330570281 1.131557051<br>S484 1.851371905 -2.593216053 3.780415696<br>S485 1.025906476 -6.332025897 -1.178878607<br>S486 0.845198035 -6.133886117 3.657208642<br>S487 3.601109179 -5.142780063 1.446132976<br>end

product

Fe(139) -2.364<br>Fe(140) -0.318<br>Fe(141) -2.632<br>Fe(142) 2.596<br>Fe(143) 2.737<br>Fe(144) 2.209<br>Fe(145) -1.510	bm602n2x2b226nhte.car_4<br>C1 -3.824160647 7.371483515 4.829717368<br>C2 -4.872063147 7.219877004 5.938678345<br>C3 -3.218324381 8.593586296 2.783728519<br>C4 -1.760396335 8.952082358 3.053589524<br>C5 -0.103429990 9.813337990 4.627820588<br>C6 0.758159412 8.555598101 4.791984174<br>C7 0.814683301 6.158734281 5.255203233<br>C8 1.153669223 5.623805287 3.862881194<br>C9 0.471735899 5.414916343 1.525010865<br>C10 1.648873442 6.170408894 0.889423833<br>C11 2.760106468 8.289357574 0.505010922<br>C12 4.138914281 8.056917343 1.134740907<br>C13 5.458659190 8.023101055 3.190645055<br>C14 5.994531017 6.612775712 3.462598811<br>C15 5.408152996 4.304920797 4.066210605<br>C16 5.254005072 3.168866755 3.059206022<br>C17 4.209152614 2.394914365 0.936354634<br>C18 5.123469040 2.632398143 -0.280968581<br>C19 2.711767228 2.322054607 0.569448911<br>C20 2.484043580 1.374602279 -0.613997917<br>C21 1.880378954 1.928023841 1.794724924<br>C22 5.633186892 4.080788196 -2.211522024<br>C23 6.970906370 4.734303051 -1.830204060<br>C24 4.807125299 4.949674982 -3.193441648<br>C25 5.660407250 5.434382831 -4.371737542<br>C26 3.585485545 4.174534982 -3.703632458<br>C27 8.142547924 6.393651557 -0.508207792<br>C28 9.294031769 5.467708688 -0.176507065<br>C29 6.333164433 -1.548146497 12.026395321<br>C30 6.189934898 -2.441985249 10.791994369<br>C31 6.696421085 -2.539177075 8.344581327<br>C32 8.138042132 -3.011185643 8.068448880<br>C33 6.203183616 -1.558582945 7.264151369<br>C34 6.163218281 -2.172381484 5.860569108<br>C35 5.517095838 -1.227402296 4.841185288<br>C36 5.537570744 -1.501423765 2.328888316<br>C37 9.555362507 -4.910439870 7.456125675<br>C38 9.820819528 -5.215779536 5.998306710<br>C39 -4.568573700 3.517182549 2.401904760<br>C40 -4.389566668 4.066786595 0.971840101<br>C41 -3.597966670 2.338423057 2.615422280<br>C42 -3.384922153 1.916172889 4.083409482<br>C43 -4.376205661 0.896938902 4.642147022<br>C44 -5.230712030 3.666043573 -1.336312792<br>C45 -5.168585303 5.086289320 -1.905990547<br>C46 -6.402980168 2.862560560 -1.919367233<br>C47 -5.694080836 7.449467408 -1.554033509<br>C48 -4.278567504 8.038019627 -1.601109852<br>C49 -1.914819815 7.771020497 -1.015772229<br>C50 -0.996146867 6.920074745 -1.894912614<br>C51 -0.710979376 4.997593409 -3.382689559<br>C52 -0.414709206 5.677222577 -4.728303378<br>C53 -1.398234532 3.660828371 -3.706056552<br>C54 -1.708844095 2.819868758 -2.512765491<br>C55 -1.302494858 2.857794970 -1.193889228<br>C56 -2.667688717 1.205431158 -1.326033908<br>C57 -1.386795599 7.005441761 -6.539915275<br>C58 -0.112359756 7.835121586 -6.692131670<br>C59 -2.641147700 7.898175739 -6.708653572<br>C60 -3.040195388 8.236240701 -8.115202285<br>C61 -2.279279232 8.581768558 -9.214313822<br>C62 -4.432551766 8.577703217 -9.738112211<br>C63 8.471063385 -5.200381664 -0.744810327<br>C64 8.903056108 -6.028917200 0.449873740<br>C65 7.418807999 -4.183259066 -0.239202506

	C66	6.941017925	-3.245550260	-1.322444335
	C67	7.573809691	-2.010000997	-1.550727044
	C68	5.842120724	-3.580936316	-2.132813103
	C69	7.119323102	-1.130516236	-2.539153016
	C70	5.388096677	-2.721369059	-3.133538075
	C71	6.021191547	-1.490246004	-3.328106468
	C72	2.457769469	-5.515363391	-6.280556302
	C73	1.699407972	-4.899921927	-7.469218388
	C74	2.299752263	-4.639760961	-5.037783784
	C75	-0.138905588	-5.212545602	-9.093077983
	C76	-1.170026950	-4.110706882	-8.835120664
	C77	-2.170855483	-2.646432550	-7.084219107
	C78	-1.326552038	-1.370501167	-6.828979907
	C79	-2.911004157	-3.131814639	-5.817245259
	C80	-3.810764395	-2.150852219	-5.057480569
	C81	-5.186975452	-1.855041373	-5.678552001
	C82	-5.678511184	-0.467311361	-7.697716064
	C83	0.675048717	-0.350064506	-5.858153814
	C84	1.357637660	0.215156769	-7.111250953
	C85	1.770159746	-0.637067487	-4.812405387
	C86	2.664922846	-0.371967336	-9.094714263
	C87	2.065633038	-0.147714663	-10.473993522
	C88	0.037831270	-0.074642379	-11.819203969
	C89	-0.161892824	1.305360271	-12.491018973
	C90	-1.270714504	-0.870676451	-11.814752279
	C91	-2.394588381	-0.201620716	-11.033725048
	C92	-0.245786678	3.737479017	-12.197505441
	C93	0.688564574	4.213233422	-13.299818839
	C94	-0.197213285	4.729512718	-11.000441810
	C95	-1.203410966	4.381619336	-9.926916520
	C96	-2.517269208	4.869142291	-9.996571290
	C97	-0.873459579	3.520263195	-8.866238732
	C98	-3.481591353	4.486555126	-9.059957674
	C99	-1.831279728	3.102345385	-7.937627940
	C100	-3.131418192	3.589388896	-8.048834426
	C101	-4.778894216	-8.376082900	4.426109795
	C102	-5.269643119	-6.982706029	4.071515543
	C103	-5.062564050	-5.131367415	2.426167936
	C104	-5.745934161	-5.164343684	1.053966891
	C105	-5.401026012	-5.633117753	-1.332129528
	C106	-4.771281694	-6.718282471	-2.199376888
	C107	-2.915444055	-8.214671027	-2.682583517
	C108	-3.623684938	-9.548667069	-2.790424758
	C109	-9.025589892	-3.092968571	3.492869015
	C110	-9.349462662	-2.167641075	2.314625097
	C111	-7.721084357	-2.635258050	4.186145706
	C112	-7.578081857	-3.285349577	5.574668305
	C113	-6.195091187	-3.057248185	6.142603912
	C114	-8.990619115	-1.625144857	-0.038802762
	C115	-9.146621028	-2.494399711	-1.271081744
	C116	-8.050768547	-0.407809845	-0.225564437
	C117	-6.620474007	-0.861419385	-0.185897427
	C118	-5.998217254	-1.381258740	-1.332395273
	C119	-5.963095310	-0.960777942	1.052571841
	C120	-4.782697650	-2.062242594	-1.222479365
	C121	-4.749347891	-1.638996593	1.156954817
	C122	-4.177064847	-2.225533502	0.024172353
	C123	0.761066714	-8.651613072	6.987398668
	C124	2.246197402	-8.996666013	7.175249019
	C125	0.497068885	-7.166988708	7.277698935
	C126	-0.862349859	-6.655255125	6.924026294
	C127	-2.070797362	-7.054176212	7.459103359
	C128	-2.392230009	-5.291226955	6.160317995
	C129	4.274642597	-8.846585924	8.493228531
	C130	4.588104107	-10.319066095	8.358879580
	C131	-0.008772834	-0.066303369	6.959998663
	C132	1.137528695	-1.056152703	7.163473038
	C133	0.689130678	-2.531266948	7.383788129
	C134	0.137435012	-2.707914420	8.825960398
	C135	-0.082790111	-4.154400458	9.276500330
	C136	-0.467387597	-4.301804450	10.766289799
	C137	1.902494372	-3.460066630	7.224337474
	C138	0.434399248	-4.047811703	1.118657534
	Fe139	-0.674841497	-5.101253606	2.429626934
	Fe140	0.131278214	-2.376596735	-0.245670588
	Fe141	1.982600737	-4.679853321	0.003716814
	Fe142	-0.571143304	-5.178604882	-0.111095871
	Fe143	0.750678368	-4.359749100	-2.278749833
	Fe144	1.803805171	-4.476187024	2.544768957
	Fe145	0.122064899	-2.548672556	2.386232992

H146	-3.901506102	5.838387346	7.082452781
H147	-5.008847140	5.186667416	6.076382750
H148	-4.918079564	8.919777183	4.029721112
H149	-5.873820936	7.354985596	5.499122795
H150	-4.727939230	8.068871602	6.629189662
H151	-3.205580983	7.674020898	2.179488461
H152	-2.235704050	9.707193449	4.895719049
H153	-3.664746034	9.389272133	2.172207902
H154	-0.114168131	10.358589264	5.581330031
H155	-0.901224836	7.429215447	5.285872051
H156	0.394817099	10.441530738	3.877379884
H157	0.703384119	4.346264356	1.461143658
H158	-0.678498640	6.226807546	3.143606095
H159	0.183729774	5.428845497	5.780705868
H160	2.846730817	8.046733846	-0.561517026
H161	0.927606033	7.969649728	1.597938525
H162	1.760523315	6.254290802	5.801790941
H163	-0.443943800	5.598113819	0.946760090
H164	3.338890959	8.300233603	3.033034118
H165	2.498466570	9.351837568	0.607425943
H166	1.963112554	2.673658002	2.598263256
H167	0.817464613	1.830273527	1.522910340
H168	2.214598071	0.964410867	2.211074192
H169	2.398908140	3.331523209	0.256745889
H170	5.356893006	8.530408994	4.160911176
H171	2.951377922	3.810140610	-2.882970201
H172	6.053896152	6.002321698	-0.454474792
H173	6.510434779	6.053468447	-4.050352071
H174	1.421066604	1.373963293	-0.888645590
H175	2.771826302	0.339030401	-0.377053948
H176	9.353989081	-4.633058206	-1.095076607
H177	2.961627638	4.813928266	-4.344276952
H178	5.040861875	6.035700090	-5.053201310
H179	3.904620811	3.305103008	-4.301418642
H180	6.058671932	4.580004173	-4.941832107
H181	4.455369639	5.837874325	-2.636726967
H182	5.911937415	3.139689172	-2.708732843
H183	4.066560450	4.342692165	-0.760396182
H184	3.052838731	1.682140074	-1.503937255
H185	1.342562971	-1.240121608	-3.996256360
H186	2.084264062	0.332919454	-4.399539077
H187	3.702562954	-0.995623458	-4.978890471
H188	6.010110131	0.172789717	-4.328446507
H189	4.522711088	-2.987032170	-3.740965327
H190	5.308975839	-4.518177202	-1.959546270
H191	8.428569512	-1.715565920	-0.935158452
H192	4.017227182	4.299485667	1.893358211
H193	4.797373844	4.028295136	4.939795570
H194	6.460730424	4.310785238	4.375858527
H195	4.060910215	5.880795244	3.633545741
H196	6.230502200	8.542468327	2.608228876
H197	7.606886441	-0.162901145	-2.682373636
H198	4.521955371	1.428515821	1.349112886
H199	8.460888981	7.082794472	-1.308375481
H200	7.933090272	6.996811309	0.389150786
H201	8.997440530	4.482004608	0.251339178
H202	9.685184014	-5.830393130	8.047121147
H203	-4.517323263	-0.636098200	5.990332537
H204	-0.865289193	-1.643178691	6.226803673
H205	-8.310107139	-2.834550751	6.259677702
H206	-7.782590285	-4.364158545	5.511513190
H207	-7.712803937	-1.540247531	4.303241783
H208	-9.423012921	-3.554568056	-1.061274851
H209	-9.852603975	-2.912283860	4.197210898
H210	-3.944100619	1.480500750	2.018434900
H211	-3.429385620	2.801191760	4.743234860
H212	-8.063197874	-3.216176813	1.064652183
H213	-10.008836906	-1.215281045	0.121997694
H214	-8.280861304	-4.779499972	2.535869535
H215	-2.891952024	0.013020425	5.818132023
H216	-6.864063506	-2.889598715	3.543015344
H217	-9.095756060	-5.098257538	3.929056354
H218	-6.511664786	3.870730564	3.015182001
H219	-2.626504595	2.624246511	2.189625562
H220	-5.701173649	-4.844939971	5.489628125
H221	-2.366998710	1.514569187	4.196483987
H222	0.260176492	4.830483541	-2.896762819
H223	-0.617536214	3.566446753	-0.736035542
H224	-1.892342217	8.789102737	-1.428089316
H225	-3.525152790	6.491099859	-0.428629863

H226	-2.233882091	6.622753841	-4.609370135
H227	-1.209025353	8.667981317	-9.362120164
H228	0.213731693	8.329700926	-5.745532365
H229	-2.510816045	8.814954515	-6.106209669
H230	-1.381380314	6.206233599	-7.298214453
H231	-5.330324506	8.677259530	-10.342111605
H232	-0.735846607	3.123851901	-4.406049543
H233	-2.331346216	3.870685258	-4.256233796
H234	-2.947563531	9.055668570	-11.189148226
H235	-3.492727558	7.358098190	-6.265801390
H236	-2.526644732	5.610015535	-2.404373254
H237	-1.466067080	7.821557872	-0.012166853
H238	-6.122427115	7.608119529	-2.553918112
H239	-3.270627651	0.327894265	-1.104318859
H240	-6.289213997	8.035678195	-0.841240314
H241	8.643239896	-6.608887075	-2.221837721
H242	7.425655209	-5.546988200	-2.471130067
H243	0.072145495	-2.413301210	-5.670175202
H244	0.041672489	0.462283347	-5.472453538
H245	1.640948446	-1.696167859	-7.801966397
H246	-3.110837962	1.484909134	-3.422714231
H247	-5.077558909	2.113826580	-4.376469378
H248	-7.164288982	2.495340659	-3.684939769
H249	-4.113084821	2.358161239	-5.611047637
H250	-6.360457439	1.844283753	-1.498134409
H251	-7.354599355	3.332070666	-1.622249144
H252	-1.080201160	-1.892359541	-11.457814745
H253	-1.605328447	-0.943050197	-12.861282477
H254	-2.782223507	-1.974528255	-10.041577273
H255	-3.776836768	-0.583230690	-9.622088081
H256	-6.568074558	-1.377552828	-9.311699012
H257	-5.941295961	-1.750619210	-4.883641973
H258	-4.496524102	4.886778755	-9.119206163
H259	0.150514013	3.157713313	-8.742381642
H260	-4.870114014	1.413945538	-7.941105988
H261	-2.147144903	-3.487586761	-5.107294529
H262	-0.782978914	-4.243325109	-6.837706451
H263	-3.285840743	-1.205288089	-4.851346037
H264	-6.181014747	5.790092300	-0.279271930
H265	-0.673646242	-6.085014768	-9.490386364
H266	-6.037978180	3.025079221	0.598338213
H267	-3.501709081	-4.017791945	-6.103151044
H268	-1.568566993	2.386619229	-7.158237642
H269	0.525181045	-4.837442226	-9.884456185
H270	-6.693210540	-2.260756435	-7.822586816
H271	-6.041850124	0.948406258	-9.127617918
H272	-5.988716962	2.299960934	3.258821866
H273	-4.251960878	4.341997861	3.069968031
H274	-2.798309526	5.563166881	-10.792423311
H275	-4.288226512	3.194290728	-1.650307768
H276	-2.874327989	-2.390438561	-7.882799775
H277	-5.498712754	-2.700739606	-6.305762741
H278	-3.994509995	-2.610989037	-4.077918464
H279	-4.861062215	0.233207885	-5.972224533
H280	3.204073074	0.544550574	-8.812097706
H281	0.184693167	-0.245369408	-9.672507210
H282	0.727368164	-0.589768723	-12.506385716
H283	3.398672828	-1.183718609	-9.194619893
H284	0.262132060	2.250633295	-10.747205854
H285	-4.566760553	-8.942484974	3.506960600
H286	-3.805498676	-8.226623115	4.938206020
H287	0.551610723	-8.853339165	5.926785513
H288	-5.331286185	-9.887971335	5.675524968
H289	-6.083530800	-8.466286583	5.983493253
H290	2.862377817	-5.112523187	-4.222468494
H291	4.250196611	-4.762108238	-6.905602144
H292	0.416040010	-6.507077521	-7.488756833
H293	2.705789370	-3.636631987	-5.229013153
H294	4.013373005	-6.318027747	-7.356599028
H295	-1.915730406	-8.400850130	-2.257872473
H296	2.051103007	-6.516327992	-6.066343715
H297	6.849869459	-0.665184055	7.276245653
H298	6.019817049	-3.402021498	8.386076920
H299	-3.114355097	-6.982374830	-0.952907845
H300	5.420718144	-2.010468557	0.351740611
H301	-6.483497581	-5.810711538	-1.310393241
H302	-4.235837428	-1.712056670	2.117957999
H303	-5.246217934	-4.678289086	-1.863585437
H304	6.156662162	-0.356508058	4.659517600
H305	-3.923152822	-5.578692335	0.252225217

	H306	4.554604853	-0.854049611	5.229268620
	H307	5.189152077	-1.243414563	7.557948692
	H308	5.587280219	-3.109849964	5.884378818
	H309	7.179019714	-2.424391418	5.517037448
	H310	-8.263658009	0.305765931	0.583961776
	H311	7.138877131	-0.997709752	9.785981998
	H312	5.322984594	-1.167895942	12.241592445
	H313	-3.232952507	-2.763329262	0.110007262
	H314	8.193474827	-0.669320559	12.096470899
	H315	6.969021041	0.369119116	12.421895445
	H316	4.686899902	-2.763187577	3.645694800
	H317	-4.300127547	-2.482404768	-2.105617284
	H318	-6.413334341	-0.513050838	1.942069132
	H319	2.959087324	-6.299745058	10.637851157
	H320	0.649411136	-6.981240035	8.354962409
	H321	3.646966961	-4.686209183	8.782237635
	H322	1.505224308	-6.256691420	11.185282675
	H323	-4.339888101	-9.769426236	-1.962174835
	H324	3.459244202	-4.887872996	5.655475414
	H325	1.764378495	-0.710907619	7.994197566
	H326	-4.071158348	-7.000666964	2.412173162
	H327	0.870308193	-2.234999694	9.498855611
	H328	-4.000999767	-6.156734397	7.213242759
	H329	-2.908551454	-4.478056325	5.668250242
	H330	6.607791499	-2.211630112	12.864502571
	H331	4.653485266	-3.158387688	1.417310550
	H332	5.480668628	-5.761440157	6.553903042
	H333	4.108086271	-5.613282435	4.445140888
	H334	7.409112857	-4.909592245	7.598995176
	H335	6.186135510	0.422086224	2.726000596
	H336	-0.892037640	-4.605602567	8.682158620
	H337	0.820546380	-4.753333871	9.096553015
	H338	-0.800657802	-2.139814750	8.915685714
	H339	-4.118041192	-4.567905202	2.373233746
	H340	-6.493708861	-1.283548089	-2.301731238
	H341	-8.317717857	0.064652497	-1.183254804
	H342	1.745355071	-1.003511466	6.244721265
	H343	5.363684666	-5.842946055	8.102507905
	H344	4.710448570	-4.730540232	9.904758764
	H345	6.334868913	-0.087657339	1.118647616
	H346	-2.309915117	-7.845101142	8.160073557
	H347	1.247364503	-6.559205571	6.757908182
	H348	-5.735005078	-4.640471593	3.132630247
	H349	-2.776549436	-7.797270433	-3.691478455
	H350	7.876102260	-3.607412700	0.579214317
	H351	6.565897125	-4.743678309	0.176061412
	H352	0.064644489	-10.504743771	7.525243059
	H353	0.126897463	-9.453100114	8.778354074
	H354	4.544833022	-8.547164417	9.518789737
	H355	2.391969067	-7.992030781	9.015299826
	H356	3.740870353	-11.006464676	8.596254869
	H357	4.906620068	-8.267763391	7.801539742
	H358	8.168113318	-6.812847587	0.756265911
	H359	1.674903682	3.694296002	-13.342366698
	H360	10.325763252	-4.188286988	7.780911338
	H361	9.220673850	-4.616644572	5.270052108
	H362	-1.266799089	3.726378804	-12.614064437
	H363	0.821569899	4.735326054	-10.581543173
	H364	-0.393723908	5.733885907	-11.402215580
	H365	-4.865469595	3.722308032	-7.171375989
	H366	2.258311228	-1.753513155	1.125380782
	H367	1.127474787	-0.522972738	1.228364309
	Mo368	0.281235361	-4.118903767	4.754037117
	N369	1.260221050	-1.526191355	1.139324808
	N370	-4.838118831	5.981705094	6.696714153
	N371	-4.036577181	8.415484210	3.974190183
	N372	-1.476872509	9.540831749	4.238816348
	N373	0.118854941	7.436715295	5.218030600
	N374	0.210830383	5.794791610	2.904901554
	N375	1.698727641	7.503314277	1.115027773
	N376	4.185972972	8.104192398	2.493507793
	N377	5.054701280	5.638477186	3.623539291
	N378	4.479571002	3.393418553	1.973642770
	N379	4.848373779	3.740359307	-1.025339981
	N380	6.933966495	5.672238052	-0.849834499
	N381	7.242339700	-0.412535880	11.824699087
	N382	6.663035840	-1.896237401	9.653364293
	N383	5.261260720	-1.920966270	3.572455104
	N384	6.267547878	-0.388059742	2.092323791
	N385	5.083612281	-2.221033657	1.287690433

N386	8.259174311	-4.309657042	7.696295647
N387	-5.965086888	3.107439022	2.610148553
N388	-3.886284766	0.052822760	5.571142816
N389	-5.346552681	3.619673952	0.112637225
N390	-5.782728604	6.050820225	-1.178760725
N391	-3.303827404	7.359187178	-0.935646595
N392	-1.535724057	5.835298170	-2.517251969
N393	-2.586130915	1.753919321	-2.580845663
N394	-1.902029850	1.857691177	-0.462546864
N395	-1.425342138	6.448330387	-5.200726734
N396	-4.384341739	8.238160739	-8.456229390
N397	-3.181765153	8.792384570	-10.235905914
N398	7.893573207	-6.099688567	-1.747409004
N399	3.892051487	-5.664784373	-6.577266497
N400	0.648452304	-5.629708172	-7.946977246
N401	-1.342119710	-3.755867389	-7.546248964
N402	-5.191656089	-0.621194440	-6.470597413
N403	-6.398583391	-1.423945587	-8.312376742
N404	-5.401752863	0.663448918	-8.391158805
N405	-0.162600830	-1.519078828	-6.136280935
N406	1.712397494	-0.706080678	-8.052003321
N407	0.702782450	-0.047791972	-10.528778967
N408	-3.154042200	-1.046960695	-10.282192576
N409	0.120108070	2.401857771	-11.742897447
N410	-5.779124098	-9.085978429	5.227039522
N411	-4.795695542	-6.478641426	2.913526113
N412	-4.933257322	-5.527545275	0.038484293
N413	-3.599585078	-7.277842416	-1.806281054
N414	-9.096989711	-4.505257819	3.096316721
N415	-8.729145950	-2.440217288	1.132202832
N416	-0.115139249	-9.530304369	7.785053854
N417	-1.087040889	-5.553665653	6.097937478
N418	-3.015766347	-6.182868960	6.964209773
N419	2.859963209	-8.569250411	8.311476084
N420	-0.195922614	-0.218962656	-2.223746532
N421	-0.070275723	-1.015889371	-1.440946874
O422	-2.821130915	6.644101808	4.731040077
O423	-0.888316922	8.695172125	2.204757505
O424	1.979375060	8.596156915	4.567606292
O425	2.240426048	5.072303034	3.623983108
O426	2.502691299	5.582483822	0.203315989
O427	5.148718972	7.876781362	0.434961440
O428	7.213742500	6.422476532	3.567603544
O429	5.814608436	2.087027576	3.322203997
O430	6.043683321	1.845231380	-0.553396735
O431	8.032947392	4.426283993	-2.396586606
O432	10.467514767	5.790556713	-0.292184816
O433	5.643289893	-3.558873289	10.896251053
O434	9.096723054	-2.228743068	8.198860493
O435	10.638280940	-6.041150572	5.622341745
O436	-3.468598230	4.840729595	0.649416877
O437	-5.577583298	0.861732554	4.289160845
O438	-4.567713552	5.324594897	-2.967943218
O439	-6.308000347	2.821478892	-3.355979366
O440	-4.089730707	9.097849452	-2.216701287
O441	0.195011652	7.237472312	-2.016325191
O442	0.620404377	5.466007867	-5.371781156
O443	0.479475255	8.011092399	-7.744530241
O444	9.954231743	-5.870807046	1.054967518
O445	5.511242597	-0.664488456	-4.311249977
O446	2.028899836	-3.814280830	-7.977325792
O447	-1.789101148	-3.614394932	-9.797031624
O448	-1.715355090	-0.272727487	-7.257858429
O449	1.607154072	1.423870713	-7.205580848
O450	2.882553581	-1.316524185	-5.405991008
O451	2.790121961	-0.051745500	-11.472887663
O452	-0.506480263	1.376436531	-13.679539447
O453	-2.623998189	1.012822557	-11.120803703
O454	0.405572969	5.122913894	-14.064341412
O455	-4.096054716	3.121485674	-7.137632877
O456	-6.032502859	-6.354563731	4.845769193
O457	-6.958325331	-4.900563553	0.911110196
O458	-5.323230631	-7.028680728	-3.269351018
O459	-3.414856518	-10.348576992	-3.687895920
O460	-10.127057449	-1.206756280	2.446866610
O461	-5.856702665	-2.026483985	6.713647554
O462	-5.298928265	-4.048661668	5.954064179
O463	-9.045257859	-2.085002147	-2.418993299
O464	2.861761439	-9.692488492	6.343042245
O465	5.700639528	-10.752263601	8.089298132

	O466	-1.078467497	-0.564385849	6.336037304
	O467	0.068838322	1.115297624	7.297093391
	O468	0.103729653	-5.241519892	11.425815543
	O469	-1.339746643	-3.512139164	11.226044174
	O470	1.807255763	-4.387316472	6.306143583
	O471	2.898538661	-3.329948931	7.961056993
	O472	-0.287158817	-2.847571641	6.387055032
	O473	6.008178723	-5.996622531	7.357505084
	O474	4.334848008	-5.095117084	5.239646629
	O475	-4.400195287	1.681070781	-4.958274369
	O476	2.318346163	-6.843117331	11.147287212
	O477	4.175804016	-5.330126522	9.325341468
	S478	0.525544329	-4.454045974	-4.539031532
	S479	-1.316797949	-3.668643899	-1.602727026
	S480	-1.640830218	-3.438924139	3.584017323
	S481	2.156917428	-2.920593292	-1.373447326
	S482	-1.358593016	-1.405120593	1.232605725
	S483	-1.923794189	-6.414719158	1.147435952
	S484	1.836492507	-2.592445876	3.807573843
	S485	1.056548417	-6.319988426	-1.183681752
	S486	0.873968172	-6.148880965	3.670025053
	S487	3.610014939	-5.101673900	1.447117628
	end			

Fe2-brNH2-Fe6-3b5 to Fe2-brNH2-Fe6-3b3

35, S=1

reactant

Fe(139) -2.192	bm5expro2b2n2x3b5.car_3			
Fe(140) -0.056	C1	-7.916052984	-5.025655274	1.542412966
Fe(141) -2.528	C2	-8.374212012	-6.176213622	0.638691139
Fe(142) 2.514	C3	-8.602361485	-2.978880857	2.719126065
Fe(143) 2.653	C4	-8.013859977	-3.221709390	4.105911707
Fe(144) 2.068	C5	-7.692335725	-4.766523665	5.970951386
Fe(145) -0.671	C6	-6.169365410	-4.931797148	5.907542466
	C7	-4.216604256	-5.380731073	4.509912403
	C8	-3.612242139	-3.974367752	4.465032063
	C9	-3.891927264	-1.664818011	3.686476266
	C10	-3.792167162	-0.994646686	5.065283795
	C11	-4.743093900	-0.660998459	7.264758620
	C12	-3.714174342	-1.281188508	8.222561895
	C13	-2.950137150	-3.329934720	9.332281937
	C14	-1.526505389	-3.743354959	8.936749451
	C15	-0.076342231	-4.605007660	7.149448057
	C16	0.715955436	-3.508228990	6.432676590
	C17	0.626294141	-1.420717940	5.161395636
	C18	0.995058475	-0.183653958	5.991963139
	C19	-0.117797222	-1.085077775	3.849241133
	C20	0.525996260	0.111937076	3.138760558
	C21	-0.149824112	-2.310591118	2.928174682
	C22	0.222726892	1.767784841	7.268487774
	C23	0.586644927	1.437573630	8.727652486
	C24	-0.945564266	2.773265242	7.129260856
	C25	-0.722822876	4.005334229	8.015562484
	C26	-1.114191024	3.195838276	5.662789128
	C27	0.134931005	0.140757410	10.728518611
	C28	1.574521228	-0.310435490	10.876926533
	C29	5.444453338	-11.683300948	4.095225814
	C30	6.074022754	-10.448626708	3.456041889
	C31	6.437274153	-8.010248607	3.804086833
	C32	7.682512043	-7.672549859	4.645099385
	C33	5.338940890	-6.951137097	3.995892528
	C34	5.802150643	-5.551289510	3.594407785
	C35	4.661006762	-4.539983675	3.602157088
	C36	4.778039898	-2.029283195	3.535770379
	C37	9.991754149	-6.861284513	4.581450246
	C38	10.307188762	-5.383826521	4.535925873
	C39	-5.447455311	-2.561620686	-1.428805143
	C40	-5.843090754	-1.141656434	-0.975725337
	C41	-3.913072678	-2.699383535	-1.415899061
	C42	-3.383878598	-4.147882671	-1.479955242
	C43	-3.188334946	-4.733125494	-2.878229893
	C44	-6.129750378	1.143874351	-1.921223890
	C45	-7.235949525	1.688148229	-1.012083965
	C46	-6.231813727	1.702525751	-3.348070390
	C47	-9.427170679	1.287294329	0.004491935

C48	-9.043856624	1.344056077	1.488546475
C49	-7.385101604	0.827718944	3.212655983
C50	-6.175200625	1.738960477	3.433355690
C51	-4.508524169	3.267542323	2.488584268
C52	-4.885682263	4.614942038	3.124464982
C53	-3.871021857	3.589032054	1.127252906
C54	-3.366197445	2.397733232	0.386908957
C55	-3.103560656	1.092332867	0.749009224
C56	-2.615149985	1.215655302	-1.339642118
C57	-6.543116006	6.424230459	3.082905255
C58	-6.403528453	6.643445873	4.587511633
C59	-8.028064106	6.532531328	2.657449360
C60	-8.613084191	7.908612722	2.538738166
C61	-8.471019103	9.028671569	3.335834529
C62	-9.862904732	9.444461919	1.661985831
C63	9.189180879	1.240967756	3.432212718
C64	10.146622128	0.071594258	3.299165269
C65	7.758070439	0.679532000	3.239594337
C66	6.688728799	1.729426689	3.427045159
C67	6.123865923	1.979525528	4.691041056
C68	6.223404675	2.487297116	2.336772580
C69	5.121663297	2.939429853	4.863715249
C70	5.235905858	3.458559544	2.496696652
C71	4.678374087	3.677091378	3.759034580
C72	5.564285949	6.577546981	-1.549072261
C73	4.567491636	7.729332011	-1.754077802
C74	4.814383465	5.303681816	-1.148736553
C75	3.583687435	9.307597708	-3.381202440
C76	2.098745227	8.970420525	-3.535449914
C77	0.414551392	7.135290733	-3.450787612
C78	-0.085640469	6.854600219	-2.010247118
C79	0.413096031	5.876190843	-4.345097282
C80	-0.864455996	5.037302596	-4.452794993
C81	-1.996682412	5.607346480	-5.320949411
C82	-3.458103117	7.583058665	-4.874920159
C83	0.351866840	5.905829194	0.202749110
C84	0.278906829	7.154474360	1.090821006
C85	1.284639220	4.892180633	0.894297575
C86	1.495578467	9.167876991	1.769278280
C87	0.935904519	10.537666772	1.422251804
C88	-0.366973642	11.865436969	-0.143950077
C89	-1.583919795	12.494279694	0.573882062
C90	-0.566606574	11.879747291	-1.664375171
C91	-1.784936084	11.095412782	-2.137009349
C92	-3.584938657	12.120476704	1.919519422
C93	-3.428798899	13.262926235	2.911293988
C94	-4.312054380	10.927448759	2.598562255
C95	-4.617970610	9.827776997	1.610084271
C96	-5.796348676	9.862400330	0.849276808
C97	-3.704784717	8.787585679	1.370221402
C98	-6.046168012	8.908570947	-0.138789775
C99	-3.925360156	7.839466159	0.367847815
C100	-5.095319658	7.917305216	-0.383696543
C101	3.771495220	-4.315228052	-9.009407318
C102	2.373693454	-3.996050377	-8.506310760
C103	1.029933193	-2.389167506	-7.167516519
C104	0.562259607	-1.044406653	-7.739156397
C105	1.019793686	1.372624538	-7.768170319
C106	2.220598841	2.287538678	-7.987929266
C107	4.532139439	2.877040251	-7.527761568
C108	5.058437018	3.054401655	-8.936720644
C109	-2.988865752	-3.651870773	-9.073860698
C110	-3.962446667	-2.502588181	-8.787016669
C111	-2.534670955	-4.303178647	-7.747485970
C112	-1.888016938	-5.679090305	-7.995690640
C113	-1.186101018	-6.175088403	-6.751581535
C114	-4.254298690	-0.152767869	-8.187847211
C115	-3.682165214	1.087377999	-8.844816274
C116	-4.686487220	0.038688341	-6.713369731
C117	-3.469841866	0.058753076	-5.836493322
C118	-2.740894311	1.245357507	-5.653366862
C119	-2.939247894	-1.151661263	-5.361296589
C120	-1.458912536	1.199567659	-5.099653063
C121	-1.659633128	-1.193184207	-4.807612335
C122	-0.900035952	-0.022604225	-4.721948355
C123	7.566531784	-6.687081184	-4.670432162
C124	8.751765982	-6.818273545	-3.699679829
C125	6.238327601	-6.963862471	-3.948309985
C126	4.985804512	-6.674986349	-4.712980708
C127	4.565062010	-7.281887875	-5.878534000

	C128	2.944513430	-5.989975755	-5.103844365
	C129	9.921440884	-8.079225940	-1.990691677
	C130	11.274073295	-7.908205311	-2.644863957
	C131	0.360161214	-6.849190075	-0.000767719
	C132	1.850438203	-6.985174248	0.305107877
	C133	2.744969378	-7.186999454	-0.953710497
	C134	2.588112273	-8.640259413	-1.480409344
	C135	3.616404626	-9.081306193	-2.525185968
	C136	3.550176257	-10.582527970	-2.888200024
	C137	4.220242985	-6.973171550	-0.576698418
	C138	3.525230458	-0.926207360	-2.278471810
	Fe139	3.708595600	-2.257004712	-3.736717760
	Fe140	1.870759457	0.421191753	-1.324452378
	Fe141	4.875768420	0.275119583	-1.436520010
	Fe142	3.590678770	0.353827365	-3.736646551
	Fe143	3.758497445	2.494944441	-2.170948152
	Fe144	4.717819135	-2.274607387	-1.378013963
	Fe145	2.133033449	-2.035765344	-1.757640299
	H146	-6.632601616	-7.235677760	0.636438346
	H147	-6.825230158	-6.302672961	-0.688195728
	H148	-9.832736295	-4.277566860	1.555075170
	H149	-9.088727122	-5.783779751	-0.102134665
	H150	-8.949834655	-6.869916835	1.275714702
	H151	-7.876958881	-2.352647292	2.178531879
	H152	-8.884260833	-5.074110117	4.205305660
	H153	-9.528929479	-2.400935040	2.833268075
	H154	-8.137913761	-5.710388630	6.312412019
	H155	-6.260051479	-5.418738114	3.903532113
	H156	-7.893783350	-3.997232674	6.727941949
	H157	-2.908987075	-1.570025580	3.209188060
	H158	-5.126561847	-3.353962575	3.223748474
	H159	-4.018694715	-5.894442029	3.559608277
	H160	-4.493324418	0.403199489	7.168917821
	H161	-5.605131571	-1.793342721	5.640696316
	H162	-3.716210518	-5.932496665	5.314205394
	H163	-4.621091988	-1.128394617	3.063895940
	H164	-4.405882179	-3.176989217	7.767382839
	H165	-5.741192065	-0.753064836	7.714904897
	H166	-0.620136804	-3.179080062	3.410610838
	H167	-0.717866329	-2.086159352	2.011623409
	H168	0.871144172	-2.599946515	2.627485948
	H169	-1.146421661	-0.799066253	4.114117493
	H170	-3.493580294	-4.239140564	9.627265095
	H171	-1.327676093	2.342785809	5.003952736
	H172	-1.009930698	0.108199430	8.925064879
	H173	-0.654570326	3.748296366	9.082192960
	H174	-0.024200852	0.336335659	2.214835234
	H175	1.574275264	-0.090084922	2.865567328
	H176	9.269701157	1.616277524	4.469917984
	H177	-1.942918523	3.912002966	5.559279218
	H178	-1.556543546	4.711190510	7.885305860
	H179	-0.194912796	3.685313018	5.300136402
	H180	0.207515475	4.522464480	7.731056496
	H181	-1.872171797	2.269809274	7.460655731
	H182	1.133748982	2.229757421	6.859886411
	H183	-0.991805515	0.287878072	6.272341458
	H184	0.510677325	1.020529242	3.758094076
	H185	1.499047496	4.070315363	0.195095374
	H186	0.731754649	4.484512763	1.753065322
	H187	2.738995701	5.156355928	2.200715958
	H188	3.340304840	4.656774978	4.766509707
	H189	4.863770939	4.020637751	1.639781029
	H190	6.612950067	2.289584544	1.335291886
	H191	6.459676531	1.401278364	5.556439514
	H192	-1.028218509	-2.543545027	5.927242989
	H193	-0.209539545	-5.435723111	6.440023808
	H194	0.545227333	-4.961042892	7.979898542
	H195	-2.147403723	-4.161726713	7.001845465
	H196	-2.850247722	-2.683322574	10.212706449
	H197	4.676368371	3.102205170	5.849281637
	H198	1.602863245	-1.834652858	4.877827995
	H199	-0.062851604	0.953987659	11.445365597
	H200	-0.501289542	-0.717253422	10.997606873
	H201	2.037978899	-0.719891347	9.949020743
	H202	10.834500483	-7.430540004	4.159636268
	H203	-2.008879746	-6.034686743	-3.927791512
	H204	1.056793124	-6.079132151	-1.636351126
	H205	-2.670694186	-6.404415661	-8.259950499
	H206	-1.175199517	-5.617940307	-8.831451466
	H207	-3.392187463	-4.430678225	-7.068494136

H208	-2.992816632	0.883885983	-9.698146333
H209	-3.607906786	-4.387502267	-9.609647004
H210	-3.498357052	-2.107395684	-2.246356289
H211	-4.070567501	-4.826704992	-0.943218348
H212	-2.392428747	-1.202389917	-8.384203296
H213	-5.183282698	-0.345441521	-8.765860451
H214	-1.231821170	-2.637841031	-9.516103689
H215	-1.509436149	-5.805319926	-2.255257821
H216	-1.831032477	-3.625225514	-7.240298222
H217	-1.421248854	-4.049359071	-10.338012795
H218	-6.916523813	-3.260403735	-2.706017648
H219	-3.535505444	-2.229163537	-0.497782658
H220	0.492949833	-5.467526752	-7.490274886
H221	-2.427707305	-4.203619321	-0.938100383
H222	-3.776419882	2.796774461	3.159597937
H223	-3.239910523	0.636549507	1.726116260
H224	-8.198321526	1.220752578	3.838571354
H225	-7.309640348	0.240135106	1.156159704
H226	-6.717586882	4.479921344	2.206733127
H227	-7.870306484	9.224841018	4.215882404
H228	-6.606476319	5.729349855	5.196789755
H229	-8.633941474	5.907125049	3.337647222
H230	-5.933241127	7.177595532	2.559023899
H231	-10.552872998	9.997083274	1.029260031
H232	-3.051178408	4.301501036	1.320511179
H233	-4.612333594	4.120434187	0.506870620
H234	-9.387847557	10.946577518	3.095585626
H235	-8.119805351	6.071637687	1.660686184
H236	-6.079711127	2.254599361	1.423435558
H237	-7.131405007	-0.166314447	3.610389202
H238	-9.832470638	2.279172649	-0.240363515
H239	-2.277049144	0.992097145	-2.348850134
H240	-10.239725715	0.558122491	-0.116618875
H241	10.338099837	2.761175247	2.680407322
H242	8.746134229	2.921614044	2.351434020
H243	1.638281414	5.770656072	-1.525580463
H244	-0.660308143	5.476776095	0.184254194
H245	1.947248464	7.888588180	0.150197791
H246	-3.177115549	3.291066979	-1.547835349
H247	-4.937525680	4.216499786	-2.757234770
H248	-6.454367137	3.451037403	-4.201934292
H249	-4.610675061	5.479926591	-1.860964914
H250	-5.382215942	1.311115164	-3.933014551
H251	-7.172828253	1.362716421	-3.810911181
H252	0.355854918	11.543036017	-2.158154977
H253	-0.733047949	12.930477819	-1.950324601
H254	-0.612673422	10.063693674	-3.494819391
H255	-2.327053279	9.649597861	-3.436433991
H256	-3.389157074	9.153710485	-6.197227257
H257	-2.541541111	4.785717703	-5.811134172
H258	-6.974215752	8.940193362	-0.715617356
H259	-2.799506168	8.692269938	1.975628249
H260	-4.435710626	7.824185643	-3.082229611
H261	1.208610095	5.208799633	-3.978010504
H262	2.535681872	6.991630827	-3.305417554
H263	-1.254773399	4.767134165	-3.459501654
H264	-8.370278805	0.031239112	-1.381284070
H265	3.927943993	9.750246508	-4.325902205
H266	-6.038455648	-0.802470874	-2.923413057
H267	0.723565137	6.193176731	-5.354802900
H268	-3.178994758	7.072346511	0.160017124
H269	3.643521908	10.084079272	-2.604659928
H270	-2.684419866	7.682744256	-6.784563281
H271	-4.860174371	8.958614955	-4.315198238
H272	-5.355492583	-3.465522667	-3.275562898
H273	-5.856485499	-3.235581215	-0.651391917
H274	-6.536310145	10.646006713	1.029960360
H275	-5.192780487	1.498885024	-1.468202076
H276	-0.250755605	7.905670853	-3.852854696
H277	-1.566236694	6.226520147	-6.119350181
H278	-0.557456135	4.092360373	-4.920950749
H279	-3.397274707	5.893841269	-3.739997727
H280	1.091993824	8.881882208	2.752040386
H281	-0.078283348	9.723727445	-0.160833706
H282	0.450860683	12.569111434	0.077277664
H283	2.584697349	9.277494392	1.863990812
H284	-2.098462281	10.694772826	1.359291090
H285	4.305986526	-3.382724140	-9.244028770
H286	4.296947559	-4.777843315	-8.147432844
H287	7.582118761	-5.637638700	-5.000036631

	H288	4.631857926	-5.579300349	-10.356253816
	H289	3.072903538	-5.943392298	-10.008099234
	H290	5.558281302	4.509097943	-0.998839676
	H291	6.046838912	7.185814696	0.339713258
	H292	5.022946480	7.776627673	-3.760084024
	H293	4.264291241	5.468444854	-0.211404888
	H294	7.115009383	7.692435354	-0.793084907
	H295	5.356657734	2.473665223	-6.918091800
	H296	6.111068867	6.392233718	-2.487143842
	H297	5.014357257	-6.959051909	5.050146189
	H298	6.707440786	-8.057104082	2.741974077
	H299	3.543583276	1.092348029	-6.895289961
	H300	4.954070841	-0.028070559	3.145305209
	H301	0.465664078	1.319410756	-8.712865038
	H302	-1.245600284	-2.134578177	-4.440674535
	H303	0.367240775	1.887868283	-7.042417285
	H304	4.267606960	-4.422978538	4.618483214
	H305	1.968689128	-0.159131296	-6.564450857
	H306	3.825635179	-4.890751599	2.972442157
	H307	4.480342957	-7.258847626	3.377958744
	H308	6.240253197	-5.581402603	2.585023735
	H309	6.590002880	-5.195386376	4.276225181
	H310	-5.359461695	-0.790384675	-6.449724072
	H311	5.494697384	-9.457750528	5.095314763
	H312	4.534826922	-11.898314235	3.513703382
	H313	0.104249289	-0.056995287	-4.304459366
	H314	5.865053769	-11.751229120	6.109749166
	H315	4.305230650	-12.105650126	5.756318989
	H316	5.593728549	-3.275053680	2.184233501
	H317	-0.882518261	2.114107076	-4.958988987
	H318	-3.524779767	-2.070438861	-5.444317696
	H319	7.207967085	-10.290736910	-1.416080697
	H320	6.213001472	-8.027956237	-3.655649694
	H321	6.290840559	-8.429373878	0.082678600
	H322	6.307307195	-10.896608401	-2.531952878
	H323	4.798605505	2.230993956	-9.644317282
	H324	6.302999044	-5.324718824	-0.323764500
	H325	1.996874825	-7.797577493	1.027069387
	H326	3.098941829	-2.311609782	-7.596402949
	H327	2.666228719	-9.302403925	-0.603669618
	H328	2.660667296	-7.138277498	-6.848642858
	H329	1.970316493	-5.522510245	-5.028715402
	H330	6.140327408	-12.520005676	3.913098801
	H331	5.368312612	-1.056184909	1.792361912
	H332	8.178521896	-6.095089747	0.823653798
	H333	7.173738106	-4.038136437	-0.250511773
	H334	8.681170576	-7.115476311	2.896771012
	H335	3.929133509	-2.659769777	5.304839470
	H336	3.461188381	-8.513479484	-3.455847307
	H337	4.635307271	-8.859566126	-2.179572859
	H338	1.570547345	-8.761663423	-1.880907009
	H339	1.203760202	-2.312848460	-6.082981938
	H340	-3.165933107	2.193321886	-5.992743129
	H341	-5.254232300	0.979698746	-6.659122585
	H342	2.141345070	-6.041018699	0.795463843
	H343	8.204978104	-7.651822285	0.719126655
	H344	7.014185822	-9.524113806	0.899765766
	H345	3.746019173	-0.990091998	5.002034782
	H346	5.056114508	-8.003060254	-6.520997952
	H347	6.217679669	-6.393482698	-3.012823025
	H348	0.248354522	-3.128402169	-7.355016509
	H349	4.265194789	3.862249669	-7.114888259
	H350	7.618320559	-0.136182387	3.964596648
	H351	7.686835794	0.246194542	2.229032790
	H352	8.609641087	-7.286246471	-6.331671001
	H353	7.821873669	-8.513898650	-5.589786759
	H354	9.883501076	-9.099264719	-1.576638394
	H355	8.120308535	-8.681933368	-2.955394238
	H356	11.299602297	-8.161315082	-3.732137019
	H357	9.824734902	-7.376987041	-1.146872749
	H358	10.411106450	-0.189881104	2.245663750
	H359	-2.417402791	13.360007776	3.370997448
	H360	9.897584923	-7.136098953	5.647717890
	H361	9.418692994	-4.710092646	4.463307983
	H362	-4.208887069	12.487079228	1.086001010
	H363	-3.689757767	10.549105770	3.424261385
	H364	-5.240314182	11.318714819	3.038817457
	H365	-6.224673159	6.993936633	-1.672090924
	H366	2.871430464	-1.061967657	0.571424150
	H367	4.556296703	-4.497070447	0.492845965

	H368	1.217258909	-1.275332078	0.534737162
	Mo369	3.654884816	-4.540952742	-2.296339998
	N370	2.043152651	-1.082558566	-0.031036063
	N371	0.676989081	1.464028401	-0.475059858
	N372	-0.060209550	2.119479108	0.068198697
	N373	-7.327758389	-6.916079736	-0.042500184
	N374	-8.905539947	-4.184967674	1.963239647
	N375	-8.299690821	-4.404768804	4.699947947
	N376	-5.656567557	-5.361008379	4.726891403
	N377	-4.301557224	-3.060068050	3.743221817
	N378	-4.774920326	-1.283274059	5.950616458
	N379	-3.734876161	-2.637140427	8.320588494
	N380	-1.372073848	-4.205789027	7.667018402
	N381	-0.011760157	-2.469024881	5.941819609
	N382	-0.028817970	0.549609967	6.498572513
	N383	-0.179919509	0.514783770	9.361646116
	N384	5.081595289	-11.490976609	5.506006945
	N385	5.936405964	-9.321069994	4.180510334
	N386	5.128772623	-3.246532185	3.092762073
	N387	4.237560341	-1.856284747	4.761357942
	N388	4.958815564	-0.962426069	2.742185150
	N389	8.740833797	-7.207085969	3.935712224
	N390	-5.993393704	-2.824815950	-2.768789403
	N391	-2.173243736	-5.608387428	-3.011138614
	N392	-6.105622978	-0.307346074	-2.019771371
	N393	-8.356374960	0.933088783	-0.909873243
	N394	-7.887666431	0.727075228	1.855030648
	N395	-5.662548644	2.383734649	2.348175473
	N396	-3.046047616	2.461262821	-0.957705237
	N397	-2.642496992	0.365382561	-0.324259446
	N398	-6.107941735	5.077619707	2.760068984
	N399	-9.487515020	8.181248900	1.498654186
	N400	-9.269147350	9.994575413	2.759196249
	N401	9.504712088	2.235933069	2.404362304
	N402	6.550736456	6.888734337	-0.502524836
	N403	4.454876206	8.200761051	-3.031244195
	N404	1.775668882	7.664860783	-3.453905065
	N405	-2.965348944	6.391420149	-4.547654031
	N406	-3.208011830	8.164362728	-6.062697591
	N407	-4.192635823	8.274423696	-3.969323085
	N408	0.765391765	6.200599557	-1.170618392
	N409	1.200646558	8.120308679	0.810121943
	N410	0.052168060	10.578800217	0.379547025
	N411	-1.574171711	10.301898595	-3.220691178
	N412	-2.286111956	11.693440930	1.415173943
	N413	3.710267613	-5.164933048	-10.200247419
	N414	2.252028077	-2.840570433	-7.820206311
	N415	1.290425804	0.016104524	-7.326087769
	N416	3.419183430	1.944279280	-7.451922229
	N417	-1.923873004	-3.228189119	-9.993814198
	N418	-3.410267288	-1.303280996	-8.449234270
	N419	7.730009819	-7.531717993	-5.868044111
	N420	3.955792812	-5.854216673	-4.245881545
	N421	3.282717042	-6.835939813	-6.103930858
	N422	8.834699970	-7.948980625	-2.945821059
	O423	-6.738061691	-4.887258639	1.919983437
	O424	-7.303625255	-2.355867716	4.645696513
	O425	-5.469209832	-4.704062607	6.908124855
	O426	-2.562634104	-3.691450257	5.069856826
	O427	-2.854371166	-0.229494635	5.342059994
	O428	-2.952216067	-0.566330870	8.895506466
	O429	-0.610518457	-3.705900572	9.770497491
	O430	1.947081364	-3.617844827	6.306559820
	O431	2.191755347	0.128301725	6.147190860
	O432	1.550037862	1.982160625	9.293903917
	O433	2.178290002	-0.292926205	11.939185625
	O434	6.630521071	-10.538223157	2.342750196
	O435	7.677176532	-7.814632606	5.881118717
	O436	11.441200849	-4.935615521	4.599438909
	O437	-5.922765680	-0.805399856	0.220482333
	O438	-3.941095487	-4.443408882	-3.836909595
	O439	-7.086922404	2.759191854	-0.398475560
	O440	-6.200601271	3.142533190	-3.314183174
	O441	-9.796676942	1.932313036	2.279383632
	O442	-5.708613508	1.863471036	4.573336512
	O443	-4.104627714	5.262305645	3.831987000
	O444	-6.163896408	7.720443965	5.108690402
	O445	10.590858573	-0.564795625	4.244280197
	O446	3.673932960	4.621350242	3.851433167
	O447	3.906484421	8.208366858	-0.816732620

O448	1.282789755	9.894445916	-3.725443117
O449	-1.214446616	7.233911864	-1.661530513
O450	-0.526747011	7.224721369	2.027684620
O451	2.502966667	5.510915611	1.320240641
O452	1.267497506	11.541723356	2.064383854
O453	-1.859351049	13.692094101	0.409707446
O454	-2.892331928	11.218834799	-1.594008402
O455	-4.350907516	14.000996431	3.223806859
O456	-5.279465429	6.993342799	-1.426233316
O457	1.426307382	-4.797469556	-8.694195626
O458	-0.392561438	-0.963032831	-8.540182293
O459	2.059999168	3.354173181	-8.605880179
O460	5.755056999	3.995687300	-9.279520960
O461	-5.192485175	-2.672751474	-8.840662250
O462	-1.764365801	-6.732524246	-5.825750545
O463	0.138580257	-5.929321111	-6.669240993
O464	-3.975291469	2.231929849	-8.530156959
O465	9.639925109	-5.946094819	-3.637238741
O466	12.288868370	-7.593083411	-2.039057325
O467	0.075454245	-6.223612679	-1.144830967
O468	-0.513263830	-7.239855128	0.774005572
O469	4.665512138	-11.193819521	-3.046330977
O470	2.405031863	-11.096669472	-3.035977441
O471	4.864370893	-6.049739603	-1.244910106
O472	4.750106859	-7.674221232	0.307423182
O473	2.361451791	-6.209618666	-1.923832089
O474	8.693290648	-6.883367616	1.126156777
O475	6.929047937	-4.829974043	0.264663546
O476	-4.212093461	4.836819667	-2.487847854
O477	7.266166831	-10.822692072	-2.241249503
O478	7.145288565	-8.937094746	0.111708610
S479	3.592365356	4.755638305	-2.428509372
S480	1.906023636	1.767160661	-3.251160845
S481	1.847479016	-3.485676442	-3.410376217
S482	3.587568080	1.568097847	-0.167891872
S483	0.258443785	-1.047788643	-2.022889747
S484	3.890750349	-0.943983706	-5.526229364
S485	3.555823533	-3.682374289	0.024025281
S486	5.477389035	1.481877785	-3.224162321
S487	5.555414704	-3.376836586	-3.114412946
S488	6.221285079	-1.055688540	-0.311099401
end			

TS

Fe(139)	-2.458
Fe(140)	-0.153
Fe(141)	-2.598
Fe(142)	2.480
Fe(143)	2.697
Fe(144)	1.954
Fe(145)	-0.852

bm5expro2b2n2x3b4c.car_1

C1	-7.916992291	-5.032058910	1.552174689
C2	-8.364671816	-6.178212319	0.639437564
C3	-8.606845451	-2.984631726	2.723463697
C4	-8.015316497	-3.226636460	4.109359473
C5	-7.695649383	-4.770257790	5.976873391
C6	-6.173029192	-4.936666332	5.915190691
C7	-4.217351308	-5.384814506	4.520288372
C8	-3.609999315	-3.980167960	4.471992935
C9	-3.887099920	-1.671032722	3.692792679
C10	-3.790116697	-1.005620346	5.074132557
C11	-4.752782726	-0.673850386	7.269878981
C12	-3.723746612	-1.291992176	8.228680273
C13	-2.962425910	-3.338632517	9.343893371
C14	-1.537464922	-3.752655105	8.953737989
C15	-0.080396761	-4.602903949	7.165249156
C16	0.712436964	-3.512006515	6.440871413
C17	0.630031309	-1.423524586	5.173293600
C18	0.996342320	-0.184837709	6.000716039
C19	-0.111557111	-1.087373462	3.857286512
C20	0.530793881	0.113628508	3.153067387
C21	-0.136210569	-2.311919209	2.934973261
C22	0.217431627	1.761607373	7.281249922
C23	0.578548870	1.431130967	8.740963734
C24	-0.951449941	2.765727411	7.139066673
C25	-0.726783777	3.999144464	8.022730949
C26	-1.120350619	3.184206723	5.671669529
C27	0.126410674	0.139235807	10.742669948
C28	1.566777361	-0.314035744	10.883878007
C29	5.451291277	-11.685979628	4.089285670
C30	6.089949022	-10.453665530	3.453262216
C31	6.452199832	-8.013267701	3.796460069
C32	7.698703651	-7.680347012	4.636507930
C33	5.357274419	-6.951628652	3.991259602
C34	5.812972072	-5.552515413	3.578543460
C35	4.658691792	-4.556077796	3.587447130

	C36	4.762731827	-2.047105764	3.505380704
	C37	10.002421451	-6.858203410	4.574843972
	C38	10.317991910	-5.380743498	4.532227871
	C39	-5.452109335	-2.567303198	-1.435634899
	C40	-5.846912748	-1.147514168	-0.981799591
	C41	-3.915044419	-2.700263997	-1.421827836
	C42	-3.377845348	-4.144861446	-1.483415288
	C43	-3.183412623	-4.733630828	-2.880511324
	C44	-6.125237465	1.139083766	-1.928785894
	C45	-7.228464648	1.687042926	-1.017700644
	C46	-6.228858266	1.696340121	-3.355595977
	C47	-9.418383458	1.291401563	0.003079761
	C48	-9.034786082	1.343365303	1.486789964
	C49	-7.375585211	0.824574129	3.209785929
	C50	-6.172380505	1.743653516	3.434962611
	C51	-4.507304843	3.275918433	2.491833868
	C52	-4.881962045	4.622582925	3.129444913
	C53	-3.871612639	3.595719352	1.128391619
	C54	-3.373128467	2.400830996	0.390058205
	C55	-3.112956472	1.096928444	0.758913453
	C56	-2.626292622	1.207705992	-1.330396195
	C57	-6.543797621	6.431082395	3.096033747
	C58	-6.396184957	6.653909744	4.599240289
	C59	-8.027492681	6.537555122	2.671775531
	C60	-8.610348813	7.914354066	2.549516043
	C61	-8.461736455	9.036510960	3.342631915
	C62	-9.852084764	9.453770548	1.667996201
	C63	9.199965381	1.244737048	3.425645661
	C64	10.158254357	0.075411633	3.295602913
	C65	7.769912927	0.682020336	3.230886346
	C66	6.698003560	1.729453833	3.419451909
	C67	6.125172531	1.968855397	4.681793642
	C68	6.240198162	2.497153820	2.332711023
	C69	5.122911713	2.928420804	4.856364364
	C70	5.253764262	3.468870737	2.495072927
	C71	4.688671468	3.677036569	3.755784941
	C72	5.569608371	6.580122032	-1.553956779
	C73	4.573083191	7.734858570	-1.757564110
	C74	4.814031659	5.310269203	-1.165139588
	C75	3.585886839	9.311891846	-3.383057695
	C76	2.100939365	8.973748526	-3.538468633
	C77	0.415472909	7.139754339	-3.454379416
	C78	-0.079888234	6.857405652	-2.012545963
	C79	0.412825782	5.880123875	-4.347368551
	C80	-0.865336024	5.041467982	-4.456684827
	C81	-1.995345027	5.611837190	-5.327426912
	C82	-3.457747894	7.587097519	-4.880160883
	C83	0.367766820	5.905856433	0.197583006
	C84	0.283849478	7.153645216	1.085636034
	C85	1.314425080	4.905148486	0.891045373
	C86	1.495324096	9.167914820	1.769272886
	C87	0.946163266	10.542017654	1.423756666
	C88	-0.360852946	11.873582625	-0.139439649
	C89	-1.579078353	12.504537084	0.575987594
	C90	-0.558698115	11.882019635	-1.659721850
	C91	-1.778159759	11.097771365	-2.130022107
	C92	-3.579162978	12.131622771	1.924549160
	C93	-3.425649784	13.270604765	2.920395952
	C94	-4.305047437	10.934678920	2.598688572
	C95	-4.608251753	9.834662013	1.608579578
	C96	-5.786058186	9.868856404	0.846825923
	C97	-3.695524024	8.793389735	1.370896155
	C98	-6.036056626	8.913532690	-0.139519069
	C99	-3.916339981	7.843640407	0.370135496
	C100	-5.086083515	7.920927064	-0.381867644
	C101	3.779464265	-4.315983032	-8.997528964
	C102	2.379980688	-4.001746883	-8.496594832
	C103	1.027624262	-2.396303175	-7.163189853
	C104	0.562359280	-1.050924106	-7.734106348
	C105	1.024807354	1.364026768	-7.764466708
	C106	2.230225575	2.272741090	-7.990536782
	C107	4.540326620	2.861360693	-7.530484140
	C108	5.049067360	3.059653971	-8.943138503
	C109	-2.976005899	-3.654499255	-9.079238110
	C110	-3.951227669	-2.505875240	-8.796676737
	C111	-2.520171089	-4.302829746	-7.751519952
	C112	-1.877802834	-5.680983251	-7.998993941
	C113	-1.183107468	-6.180546645	-6.752806887
	C114	-4.242749195	-0.154388147	-8.201260930
	C115	-3.664746948	1.084811074	-8.855884702

	C116	-4.665047261	0.037789183	-6.722602352
	C117	-3.446742695	0.055385137	-5.846643703
	C118	-2.718573121	1.241873063	-5.658445991
	C119	-2.920396869	-1.153895924	-5.364142915
	C120	-1.444195915	1.196413787	-5.086594438
	C121	-1.648414085	-1.195395725	-4.791072320
	C122	-0.890799961	-0.024070860	-4.694492035
	C123	7.579609112	-6.714896935	-4.694859964
	C124	8.758545857	-6.830510404	-3.715593645
	C125	6.248763327	-7.005562465	-3.985634100
	C126	5.007403781	-6.736986318	-4.773309061
	C127	4.585199614	-7.341907937	-5.938992304
	C128	2.971905998	-6.026250645	-5.180592360
	C129	9.923558312	-8.069219910	-1.990715026
	C130	11.277804011	-7.904711930	-2.644278839
	C131	0.365295860	-6.851884616	-0.038397325
	C132	1.844119712	-7.039207176	0.284001394
	C133	2.766257032	-7.279659664	-0.953179376
	C134	2.627644468	-8.741233588	-1.439752469
	C135	3.646848447	-9.148115328	-2.504480332
	C136	3.581674736	-10.636007076	-2.908035255
	C137	4.219817421	-7.028189305	-0.517513537
	C138	3.520282308	-0.922168872	-2.269494301
	Fe139	3.684099464	-2.275999044	-3.710295668
	Fe140	1.880568239	0.582881895	-1.366900274
	Fe141	4.868962472	0.292324343	-1.449908084
	Fe142	3.642584200	0.316990463	-3.747310430
	Fe143	3.776572411	2.530489137	-2.250243148
	Fe144	4.687736777	-2.287406587	-1.369194556
	Fe145	2.098345538	-1.965096996	-1.515562593
	H146	-6.620461772	-7.234924454	0.648115712
	H147	-6.800718562	-6.293019574	-0.671913455
	H148	-9.831802335	-4.281307522	1.551455273
	H149	-9.072199400	-5.782911404	-0.106705128
	H150	-8.944309678	-6.875908158	1.268462264
	H151	-7.883861904	-2.355906764	2.182103012
	H152	-8.889878638	-5.076602161	4.212452408
	H153	-9.535297466	-2.409996569	2.839762702
	H154	-8.141925705	-5.713950278	6.317432725
	H155	-6.260388660	-5.415962965	3.909198524
	H156	-7.897493360	-4.001448365	6.734114896
	H157	-2.903664451	-1.573594612	3.217070324
	H158	-5.121966953	-3.359680266	3.227363314
	H159	-4.018488034	-5.902143734	3.572122444
	H160	-4.509008187	0.392138571	7.178756784
	H161	-5.609764700	-1.797702010	5.638017514
	H162	-3.719752607	-5.934414828	5.327548558
	H163	-4.615991617	-1.133772700	3.070521085
	H164	-4.416582233	-3.188474844	7.777791722
	H165	-5.751999305	-0.773370507	7.716259146
	H166	-0.603485122	-3.182698896	3.416080357
	H167	-0.699712864	-2.092519636	2.014611197
	H168	0.887631046	-2.594553898	2.637954534
	H169	-1.142124051	-0.805602507	4.119405680
	H170	-3.506236357	-4.247810391	9.638292652
	H171	-1.332260332	2.328919943	5.015254484
	H172	-1.025390323	0.109980843	8.943273733
	H173	-0.662076626	3.743908091	9.090121512
	H174	-0.013511242	0.339172400	2.225289619
	H175	1.582536996	-0.082675176	2.890014096
	H176	9.279012350	1.622094850	4.463042294
	H177	-1.950367153	3.898492221	5.566340667
	H178	-1.557794358	4.707489980	7.889193541
	H179	-0.201984818	3.674459494	5.307844997
	H180	0.206205385	4.512340434	7.739242046
	H181	-1.877759877	2.263121664	7.472277136
	H182	1.129078858	2.224251091	6.874399591
	H183	-0.992250103	0.274266697	6.291152555
	H184	0.507145974	1.021641385	3.772286615
	H185	1.550087441	4.092141075	0.189406449
	H186	0.761786263	4.482513938	1.742867821
	H187	2.760864323	5.173356923	2.203028597
	H188	3.348059739	4.652541738	4.764289680
	H189	4.888534140	4.038926418	1.640398116
	H190	6.635018462	2.306825255	1.331834483
	H191	6.455573632	1.383555043	5.544626991
	H192	-1.029061011	-2.536696677	5.943797315
	H193	-0.212575007	-5.438471953	6.461191605
	H194	0.540346006	-4.953137514	7.999010366
	H195	-2.149332922	-4.150736933	7.011741792

H196	-2.866182454	-2.691405306	10.224398232
H197	4.672103322	3.083468992	5.840581546
H198	1.607106822	-1.838856798	4.892409073
H199	-0.065030367	0.953457183	11.459970032
H200	-0.510491596	-0.716952654	11.015874383
H201	2.023863320	-0.727726229	9.954147706
H202	10.846797997	-7.426274948	4.154907325
H203	-1.999830600	-6.030300287	-3.931796230
H204	1.094326011	-6.152510994	-1.691680413
H205	-2.663093758	-6.402123047	-8.266752243
H206	-1.162311444	-5.621501208	-8.832366905
H207	-3.376405938	-4.427245307	-7.070078800
H208	-2.964978325	0.879717743	-9.699988583
H209	-3.594138808	-4.393607976	-9.611754885
H210	-3.502434112	-2.107024440	-2.252558729
H211	-4.056593456	-4.826902992	-0.941222384
H212	-2.382315290	-1.206268427	-8.390367595
H213	-5.174081685	-0.344617893	-8.774839394
H214	-1.220956358	-2.639713605	-9.525969657
H215	-1.489213608	-5.788903279	-2.265820773
H216	-1.813349504	-3.626273308	-7.246602989
H217	-1.408212474	-4.054684600	-10.342549201
H218	-6.918603508	-3.263476304	-2.716537362
H219	-3.539746549	-2.226534180	-0.504707932
H220	0.501741584	-5.481319559	-7.485765653
H221	-2.418868666	-4.191606372	-0.946209057
H222	-3.773590853	2.804480865	3.160602273
H223	-3.248827683	0.646458607	1.738532084
H224	-8.191653887	1.207568289	3.838372896
H225	-7.300329296	0.239543463	1.152156223
H226	-6.716877784	4.485987118	2.220230295
H227	-7.859273313	9.231882759	4.221905042
H228	-6.595005970	5.741272289	5.212116397
H229	-8.634235913	5.914457248	3.353246533
H230	-5.934412363	7.182406523	2.568621108
H231	-10.538686784	10.007679704	1.033252292
H232	-3.049139526	4.305907865	1.318927862
H233	-4.613218462	4.128186978	0.509209421
H234	-9.369382435	10.957603137	3.096708033
H235	-8.119081261	6.074687769	1.676027562
H236	-6.071364327	2.254727709	1.425064702
H237	-7.113498236	-0.169496088	3.602234109
H238	-9.818264540	2.285814393	-0.239543351
H239	-2.286655673	0.978639353	-2.337710277
H240	-10.234652504	0.566747743	-0.118863727
H241	10.351645460	2.761858787	2.671485739
H242	8.759996236	2.924176406	2.341709830
H243	1.647577917	5.776352406	-1.536547899
H244	-0.639467654	5.465119189	0.181435929
H245	1.946743023	7.896138450	0.145185702
H246	-3.180240580	3.283783857	-1.548969158
H247	-4.942059511	4.215602451	-2.762018414
H248	-6.447043157	3.443643008	-4.213368662
H249	-4.611881008	5.478671251	-1.866090460
H250	-5.378706466	1.306050448	-3.939981158
H251	-7.169366753	1.354950261	-3.818013751
H252	0.363254441	11.540207454	-2.150465062
H253	-0.722221281	12.931358949	-1.951842642
H254	-0.610324314	10.068014559	-3.493464360
H255	-2.325261829	9.655719113	-3.431815357
H256	-3.387199806	9.160258334	-6.199383559
H257	-2.537800253	4.790764970	-5.820747827
H258	-6.963347448	8.944808375	-0.717378074
H259	-2.790809056	8.698002754	1.976976559
H260	-4.434884604	7.824129059	-3.086471337
H261	1.206399208	5.211318345	-3.978351637
H262	2.536557044	6.992890658	-3.308919048
H263	-1.257093014	4.771342818	-3.463806895
H264	-8.366087015	0.031439438	-1.382538670
H265	3.929867963	9.756422381	-4.326530773
H266	-6.033791652	-0.806062174	-2.929756490
H267	0.724986555	6.194906377	-5.357417602
H268	-3.170752982	7.075264996	0.164129118
H269	3.643786392	10.086445455	-2.604950365
H270	-2.684644642	7.689420592	-6.789550245
H271	-4.861074502	8.960999487	-4.317452580
H272	-5.356280150	-3.471585437	-3.281368108
H273	-5.858733872	-3.242912461	-0.658990101
H274	-6.525596600	10.653139579	1.026051358
H275	-5.186709783	1.492271692	-1.477724204

H276	-0.250521175	7.910456347	-3.854551744
H277	-1.562665882	6.233309112	-6.122725945
H278	-0.557024670	4.096477969	-4.924391193
H279	-3.400874178	5.894703197	-3.748983225
H280	1.081932831	8.880494819	2.747570008
H281	-0.079829449	9.732129484	-0.152342066
H282	0.457098395	12.577324899	0.081670182
H283	2.584305540	9.269652218	1.872753226
H284	-2.088205497	10.708197838	1.370673330
H285	4.313374384	-3.381190590	-9.223539293
H286	4.303128793	-4.784885173	-8.137955959
H287	7.587578223	-5.665939120	-5.026311971
H288	4.639369747	-5.575788751	-10.350117815
H289	3.076306727	-5.932696089	-10.010432968
H290	5.550565003	4.509991027	-1.015794329
H291	6.051622834	7.186064850	0.336541500
H292	5.020200999	7.776523254	-3.764727915
H293	4.259171716	5.472816022	-0.230605908
H294	7.117413939	7.696785644	-0.794655772
H295	5.370987286	2.447002936	-6.937429531
H296	6.117298327	6.399665582	-2.492120573
H297	5.038067846	-6.956674132	5.047046137
H298	6.720443052	-8.060051346	2.733807831
H299	3.541793395	1.092109518	-6.872089729
H300	4.971046534	-0.045368202	3.148436728
H301	0.468888851	1.311278731	-8.708552233
H302	-1.237878161	-2.136073555	-4.417675945
H303	0.375927677	1.884042017	-7.038545375
H304	4.280523007	-4.426482788	4.608045364
H305	1.965681395	-0.169183997	-6.555502203
H306	3.818133482	-4.938995992	2.983172924
H307	4.496494200	-7.261635211	3.377770856
H308	6.241760140	-5.583523796	2.566029680
H309	6.602142764	-5.184142520	4.252452846
H310	-5.339194375	-0.789553183	-6.456337773
H311	5.496768117	-9.455807469	5.084080602
H312	4.543648654	-11.895519791	3.502789608
H313	0.108924970	-0.054627255	-4.263472377
H314	5.861670853	-11.755784300	6.105079164
H315	4.303980870	-12.108048568	5.743778114
H316	5.557523187	-3.304940578	2.132605020
H317	-0.867364373	2.110127522	-4.941130611
H318	-3.504450613	-2.072565204	-5.453987427
H319	7.206376627	-10.284981107	-1.424300753
H320	6.232000700	-8.064895839	-3.678377122
H321	6.283598415	-8.434290453	0.115751723
H322	6.320733099	-10.909034984	-2.544786301
H323	4.792610915	2.239073677	-9.656055065
H324	6.208878754	-5.287383714	-0.173754676
H325	1.958418515	-7.851919160	1.010951144
H326	3.095588671	-2.312759006	-7.585644429
H327	2.736329489	-9.393800581	-0.559055205
H328	2.684986656	-7.181306987	-6.918574567
H329	2.004465529	-5.547314825	-5.093960789
H330	6.144389596	-12.525267616	3.909545246
H331	5.364492773	-1.057656580	1.777301751
H332	8.170020997	-6.092765325	0.834318976
H333	7.132311762	-4.041641919	-0.200254083
H334	8.692812426	-7.111180319	2.889241993
H335	3.927232719	-2.680218427	5.283125803
H336	3.475700756	-8.550637671	-3.414079930
H337	4.666478962	-8.926132420	-2.159818146
H338	1.606321201	-8.880772802	-1.826387220
H339	1.202157261	-2.323761395	-6.078058597
H340	-3.139720758	2.189819987	-6.002661267
H341	-5.231162651	0.979721201	-6.665423268
H342	2.154688169	-6.110328505	0.789775970
H343	8.213147659	-7.646889068	0.713686360
H344	7.037289316	-9.529431777	0.898107065
H345	3.767525006	-1.005856937	4.991090420
H346	5.073266366	-8.065712779	-6.581778914
H347	6.207616642	-6.420279362	-3.058685621
H348	0.247345995	-3.135905643	-7.354354805
H349	4.280191436	3.840833034	-7.099571679
H350	7.630479042	-0.135144283	3.954520423
H351	7.701566286	0.250861405	2.219096121
H352	8.636570546	-7.312334436	-6.348465813
H353	7.851202979	-8.542288304	-5.605535893
H354	9.886918751	-9.084498745	-1.565591082
H355	8.131000984	-8.690457681	-2.959694338

H356	11.304333970	-8.162018754	-3.730895351
H357	9.823744101	-7.357833292	-1.155188068
H358	10.421986410	-0.188263953	2.242487692
H359	-2.414544973	13.366902926	3.381361969
H360	9.906964599	-7.134172268	5.640581704
H361	9.430271271	-4.706888386	4.448361920
H362	-4.202154552	12.499838250	1.091491047
H363	-3.682956417	10.555296265	3.424580415
H364	-5.235196211	11.321976829	3.039288774
H365	-6.217352499	6.995019736	-1.666710517
H366	2.861701031	-0.750639264	0.632428355
H367	2.642860530	-3.948995306	0.909649465
H368	1.195822397	-0.924057636	0.611059589
Mo369	3.687898969	-4.707671435	-2.406177393
N370	2.028041144	-0.788563718	0.037004717
N371	0.672307183	1.634169084	-0.551649162
N372	-0.093213556	2.253159124	-0.002741836
N373	-7.309330107	-6.912116871	-0.036003836
N374	-8.908246173	-4.191696264	1.968037715
N375	-8.302633921	-4.408280277	4.705573159
N376	-5.658271037	-5.362953946	4.734349888
N377	-4.295732224	-3.066866260	3.745738470
N378	-4.778334364	-1.292840076	5.953668637
N379	-3.744515454	-2.647798428	8.329018855
N380	-1.376210646	-4.199782732	7.679307077
N381	-0.012115663	-2.470563621	5.950659299
N382	-0.029645106	0.542677641	6.510983486
N383	-0.192284818	0.513184852	9.376935381
N384	5.081699970	-11.493440703	5.497867385
N385	5.945596247	-9.322927802	4.172095339
N386	5.083043079	-3.264223987	3.038448042
N387	4.242048305	-1.878524315	4.740481621
N388	4.948315866	-0.970572175	2.725320577
N389	8.752954954	-7.204778231	3.928061395
N390	-5.994531697	-2.829769019	-2.776486125
N391	-2.164174471	-5.604398982	-3.014656246
N392	-6.106207773	-0.312294225	-2.025944442
N393	-8.350405305	0.934296237	-0.913095018
N394	-7.877739391	0.726906947	1.851517337
N395	-5.660916663	2.391606561	2.351573737
N396	-3.057016952	2.455504986	-0.955582317
N397	-2.655127572	0.363178347	-0.310541259
N398	-6.107975887	5.082890792	2.775142792
N399	-9.483308022	8.188223090	1.508397557
N400	-9.255582839	10.004201733	2.763333658
N401	9.517391601	2.237520209	2.396476714
N402	6.555194132	6.890829843	-0.506302579
N403	4.459461363	8.207326190	-3.034123098
N404	1.777705854	7.668138044	-3.460040944
N405	-2.967078228	6.393619838	-4.555587396
N406	-3.205230974	8.171132674	-6.065852679
N407	-4.192904232	8.276282559	-3.973194797
N408	0.775933907	6.204623634	-1.176503798
N409	1.199086341	8.125761538	0.804753742
N410	0.056476665	10.587377575	0.386278814
N411	-1.570945428	10.306625344	-3.216136438
N412	-2.279365916	11.706461259	1.421595169
N413	3.719665997	-5.157145535	-10.194878381
N414	2.251468053	-2.844668178	-7.816422741
N415	1.290116851	0.008252021	-7.319389230
N416	3.428590165	1.927051708	-7.456161199
N417	-1.912025537	-3.233112207	-10.001144965
N418	-3.399978498	-1.305969833	-8.461692040
N419	7.756480237	-7.561734332	-5.888081303
N420	3.983969989	-5.903040394	-4.320630053
N421	3.307139604	-6.880857100	-6.172503986
N422	8.839927807	-7.952193158	-2.949721088
O423	-6.741773067	-4.893773515	1.938205229
O424	-7.301903200	-2.361480993	4.646195543
O425	-5.474325967	-4.712005969	6.917565194
O426	-2.560605410	-3.697136044	5.077549699
O427	-2.850187498	-0.245707747	5.358215141
O428	-2.961996757	-0.576155757	8.900733000
O429	-0.627783181	-3.727684204	9.794705766
O430	1.942484097	-3.627598450	6.308313597
O431	2.192142078	0.132634641	6.149632676
O432	1.545711476	1.970154993	9.306054326
O433	2.177122312	-0.295275111	11.942349503
O434	6.659550789	-10.548705260	2.346914873
O435	7.696783723	-7.831444277	5.871604086

O436	11.451451187	-4.932849002	4.606817400
O437	-5.927820849	-0.812384793	0.214695074
O438	-3.940895607	-4.451679898	-3.837691766
O439	-7.076504345	2.758340320	-0.405399137
O440	-6.200315988	3.136706527	-3.322976456
O441	-9.788353842	1.927990544	2.279667864
O442	-5.709291196	1.870445214	4.576242025
O443	-4.096685176	5.270850413	3.831425455
O444	-6.152064504	7.731950474	5.116433624
O445	10.603070838	-0.558464666	4.242043130
O446	3.686178499	4.623058595	3.850617048
O447	3.913525173	8.213441915	-0.818762989
O448	1.284795501	9.898268906	-3.726994504
O449	-1.208869870	7.232498230	-1.660123220
O450	-0.521273489	7.216489235	2.023503910
O451	2.517228996	5.543304202	1.331084836
O452	1.288535946	11.543752377	2.063770542
O453	-1.855446661	13.701376659	0.406908694
O454	-2.884126779	11.220378471	-1.583836733
O455	-4.348054355	14.007740258	3.234187450
O456	-5.271551427	6.995434092	-1.422998681
O457	1.436595281	-4.809047050	-8.683607137
O458	-0.389934856	-0.968720659	-8.538575271
O459	2.074130522	3.334892883	-8.617834578
O460	5.729690437	4.013334332	-9.284525160
O461	-5.181047667	-2.677221867	-8.852992651
O462	-1.769001888	-6.731642805	-5.827783465
O463	0.142794852	-5.945286964	-6.667470927
O464	-3.962613858	2.229644376	-8.547242211
O465	9.642872607	-5.954235900	-3.656587518
O466	12.292771506	-7.590165582	-2.038295873
O467	0.121283716	-6.228297922	-1.192668271
O468	-0.533926900	-7.191938197	0.730460359
O469	4.697279211	-11.245709625	-3.069953832
O470	2.436899423	-11.142777592	-3.080294102
O471	4.856655434	-6.074622323	-1.139706594
O472	4.728317280	-7.721874886	0.386866525
O473	2.410625113	-6.363911172	-1.987714440
O474	8.701995769	-6.877255984	1.117965412
O475	6.885705792	-4.799393614	0.363746376
O476	-4.217914236	4.838946821	-2.498318068
O477	7.277413789	-10.812568983	-2.250808843
O478	7.145757580	-8.932205123	0.115321912
S479	3.604349898	4.788636445	-2.467814623
S480	1.955921489	1.776240846	-3.367540354
S481	1.847739199	-3.508579426	-3.342788089
S482	3.628143166	1.696430268	-0.219498370
S483	0.258766424	-0.971273083	-1.932282783
S484	3.860650885	-0.988618209	-5.528870870
S485	3.112120036	-3.540267748	-0.320441298
S486	5.509741267	1.474897867	-3.241432752
S487	5.558208149	-3.408684630	-3.070193103
S488	6.194666718	-1.074440495	-0.312948480
end			

product

Fe(139)	-2.383
Fe(140)	0.055
Fe(141)	-2.612
Fe(142)	2.696
Fe(143)	3.138
Fe(144)	1.275
Fe(145)	-0.430

bm5expro2b2n2x3b3.car_2

C1	-7.912921835	-5.028200451	1.545689061
C2	-8.371154145	-6.177179027	0.640843677
C3	-8.598214729	-2.980560935	2.721125428
C4	-8.007813800	-3.222661774	4.107285168
C5	-7.686037731	-4.766960781	5.973355821
C6	-6.163244687	-4.932334633	5.908907368
C7	-4.210497445	-5.380307355	4.511231640
C8	-3.605034427	-3.974612016	4.466587126
C9	-3.885993541	-1.664677536	3.688268677
C10	-3.785225735	-0.996016885	5.067424975
C11	-4.736920958	-0.661318880	7.266316827
C12	-3.707462617	-1.281423782	8.223493327
C13	-2.944400255	-3.330163801	9.334010401
C14	-1.520889289	-3.744365176	8.939005464
C15	-0.070513758	-4.606503696	7.152088273
C16	0.722178010	-3.511439205	6.432976671
C17	0.634863741	-1.421042172	5.166615911
C18	1.002274272	-0.183956286	5.997432303
C19	-0.111363367	-1.084410944	3.855190339
C20	0.530780422	0.114677027	3.147988453
C21	-0.142486048	-2.309646900	2.934153868
C22	0.229003937	1.768321131	7.271872725
C23	0.592512140	1.437925063	8.731021382

	C24	-0.939440554	2.773499401	7.132017655
	C25	-0.716799929	4.006227084	8.017270344
	C26	-1.107970103	3.195710949	5.665699208
	C27	0.140825204	0.140629105	10.730940366
	C28	1.581082962	-0.309744642	10.878131306
	C29	5.456153329	-11.687270791	4.103684406
	C30	6.081123232	-10.451484317	3.461293593
	C31	6.445704745	-8.012753168	3.805739497
	C32	7.690124792	-7.675407554	4.647482241
	C33	5.346469367	-6.954546423	3.994445912
	C34	5.801468050	-5.554845224	3.584418668
	C35	4.645619104	-4.559688882	3.577818253
	C36	4.766040633	-2.050553872	3.497155747
	C37	9.999294864	-6.865129543	4.586318688
	C38	10.314566068	-5.387838355	4.540349991
	C39	-5.443820213	-2.563000147	-1.428661707
	C40	-5.839975564	-1.143373009	-0.974950139
	C41	-3.908179568	-2.700482855	-1.414917976
	C42	-3.378313934	-4.148165409	-1.475501158
	C43	-3.179744814	-4.734918852	-2.872546666
	C44	-6.123709776	1.143373050	-1.919400316
	C45	-7.229310194	1.688518394	-1.010208294
	C46	-6.225447151	1.702571161	-3.345864870
	C47	-9.420623532	1.288486758	0.005289177
	C48	-9.037330845	1.344656877	1.489117507
	C49	-7.379086609	0.827943370	3.213812041
	C50	-6.168591522	1.738170576	3.434936194
	C51	-4.500588539	3.264656147	2.489089354
	C52	-4.877460147	4.612741105	3.123818690
	C53	-3.862622528	3.583249529	1.126669331
	C54	-3.356532310	2.392186207	0.385805797
	C55	-3.090079006	1.087053791	0.746170770
	C56	-2.602321655	1.214693495	-1.342567442
	C57	-6.534834986	6.422789050	3.082926379
	C58	-6.395460499	6.642366405	4.587332183
	C59	-8.019781949	6.532266891	2.659077573
	C60	-8.605647971	7.908668333	2.540866478
	C61	-8.463624365	9.029185595	3.337606998
	C62	-9.856303881	9.444246347	1.664270448
	C63	9.193925083	1.240126467	3.433240950
	C64	10.152457643	0.071296012	3.300267684
	C65	7.763746045	0.677743627	3.239594867
	C66	6.692747633	1.726347164	3.427707201
	C67	6.128469139	1.975349568	4.692312333
	C68	6.229424039	2.487793267	2.338890184
	C69	5.127307173	2.935955849	4.866116586
	C70	5.243446269	3.460153565	2.500210079
	C71	4.685127607	3.676094447	3.762602841
	C72	5.580350918	6.566631365	-1.547747121
	C73	4.588771307	7.728226497	-1.751048273
	C74	4.811445584	5.310171733	-1.163743549
	C75	3.596156118	9.306126746	-3.373652328
	C76	2.111604925	8.963538997	-3.529335262
	C77	0.427357054	7.129860609	-3.448951986
	C78	-0.076106787	6.851253350	-2.009348480
	C79	0.423678646	5.870570472	-4.342980541
	C80	-0.855228947	5.032905312	-4.450321056
	C81	-1.986951199	5.603455709	-5.318674334
	C82	-3.447583198	7.579130250	-4.872088816
	C83	0.358637008	5.904010722	0.204256594
	C84	0.285521649	7.152742063	1.091728579
	C85	1.291011940	4.891701738	0.898630190
	C86	1.504593920	9.164280167	1.770612761
	C87	0.944381223	10.533264693	1.423006768
	C88	-0.357363759	11.859246552	-0.144208469
	C89	-1.574597701	12.488047507	0.573688844
	C90	-0.557224492	11.875112601	-1.664032012
	C91	-1.776044112	11.091883485	-2.137009971
	C92	-3.574976169	12.116010393	1.919570950
	C93	-3.419220531	13.259371839	2.910296045
	C94	-4.304065802	10.924936721	2.599302953
	C95	-4.611215090	9.824828591	1.611732404
	C96	-5.789563425	9.859996013	0.850798794
	C97	-3.697793961	8.784882576	1.371656934
	C98	-6.039398173	8.906207654	-0.137494512
	C99	-3.918145056	7.837243350	0.368870620
	C100	-5.088519354	7.914854913	-0.382122294
	C101	3.777341894	-4.314269360	-9.010577935
	C102	2.379501479	-3.995142376	-8.506484358
	C103	1.033780531	-2.385790540	-7.169890864

C104	0.564398117	-1.042139081	-7.743061701
C105	1.019036011	1.376619558	-7.767697727
C106	2.222476003	2.289692263	-7.986939910
C107	4.535142026	2.873463535	-7.521482004
C108	5.064871627	3.054148347	-8.928782130
C109	-2.983821004	-3.652082105	-9.073534827
C110	-3.957083087	-2.502862485	-8.787019898
C111	-2.528457826	-4.302716401	-7.746678123
C112	-1.881481558	-5.678609478	-7.994668703
C113	-1.179712054	-6.173224755	-6.750116200
C114	-4.246312198	-0.153372451	-8.187177817
C115	-3.673989831	1.087181434	-8.842916982
C116	-4.684536794	0.037393533	-6.714508642
C117	-3.471314156	0.055895531	-5.833399013
C118	-2.740824280	1.242130403	-5.651759455
C119	-2.945303528	-1.154524106	-5.354900429
C120	-1.463042286	1.194038072	-5.090385630
C121	-1.669220858	-1.197452858	-4.791629073
C122	-0.910825751	-0.027408877	-4.701525411
C123	7.578375882	-6.690089834	-4.668889703
C124	8.762278569	-6.821241641	-3.697709041
C125	6.251491931	-6.966991459	-3.947407996
C126	5.001139433	-6.681366997	-4.717169837
C127	4.580396643	-7.293303816	-5.880075052
C128	2.957405257	-5.999210883	-5.112794202
C129	9.931300505	-8.083277973	-1.989799344
C130	11.284128052	-7.911119889	-2.643867167
C131	0.342367401	-6.861141624	0.001805431
C132	1.834751043	-7.053169080	0.310895841
C133	2.735770097	-7.209164531	-0.949178936
C134	2.604850824	-8.657957364	-1.489483231
C135	3.633534614	-9.089934642	-2.536735110
C136	3.567355270	-10.592389415	-2.899630532
C137	4.201578702	-6.956847702	-0.583715580
C138	3.530402381	-0.934354103	-2.275090257
Fe139	3.741652100	-2.215497104	-3.792063659
Fe140	1.864213921	0.453826507	-1.304963429
Fe141	4.813130941	0.355025588	-1.434335861
Fe142	3.629583305	0.351755732	-3.747468457
Fe143	3.670972215	2.571758426	-2.242011387
Fe144	4.772584905	-2.179635894	-1.486647359
Fe145	2.215589679	-2.154535285	-1.533347246
H146	-6.628819219	-7.234972281	0.638794914
H147	-6.821946453	-6.300714140	-0.684581005
H148	-9.828481076	-4.278122694	1.556628853
H149	-9.085787417	-5.784311182	-0.099831406
H150	-8.946782907	-6.870503660	1.278020384
H151	-7.873996154	-2.354389358	2.178882317
H152	-8.881335791	-5.073234545	4.209308281
H153	-9.524864224	-2.403042634	2.836547625
H154	-8.131826074	-5.710797748	6.314230839
H155	-6.254971160	-5.416799674	3.904520443
H156	-7.887307268	-3.997952688	6.730705506
H157	-2.903831969	-1.568510363	3.209676651
H158	-5.121350656	-3.353892230	3.228332841
H159	-4.012095511	-5.893994802	3.561066103
H160	-4.487781286	0.403044290	7.170890620
H161	-5.599896663	-1.791726352	5.641675057
H162	-3.710894124	-5.932013159	5.315841785
H163	-4.616447816	-1.128933565	3.066534229
H164	-4.400527974	-3.176955284	7.769462189
H165	-5.735129921	-0.754250031	7.716184591
H166	-0.612667887	-3.178138400	3.416257531
H167	-0.708271039	-2.087014378	2.016000245
H168	0.879897112	-2.598202355	2.637722972
H169	-1.140361378	-0.800936581	4.121744159
H170	-3.488724436	-4.238730747	9.629063227
H171	-1.321135110	2.342876832	5.006504289
H172	-1.005162549	0.109892023	8.928184610
H173	-0.649502154	3.750305830	9.084271262
H174	-0.017560060	0.341711337	2.223734089
H175	1.579881529	-0.085904913	2.877475270
H176	9.273720612	1.615892589	4.471110404
H177	-1.936943228	3.911478011	5.562450370
H178	-1.550341522	4.712068853	7.885540502
H179	-0.188902499	3.686000712	5.303787675
H180	0.213867998	4.523017327	7.732857287
H181	-1.865895539	2.270357312	7.464031399
H182	1.140139471	2.230338437	6.863114467
H183	-0.985401676	0.288459385	6.275836312

H184	0.514049106	1.022030033	3.768897353
H185	1.508049047	4.069186738	0.201129862
H186	0.736950424	4.484392449	1.756985964
H187	2.747160452	5.152030195	2.203152432
H188	3.351537800	4.658416951	4.772789816
H189	4.873262659	4.023585044	1.643365114
H190	6.620059029	2.293374642	1.337186040
H191	6.465665935	1.397918034	5.557729596
H192	-1.020858976	-2.542221124	5.933784400
H193	-0.203836301	-5.438363175	6.444042675
H194	0.551082993	-4.961849590	7.983011679
H195	-2.140908969	-4.161411387	7.003220669
H196	-2.844898994	-2.683613697	10.214557339
H197	4.682951548	3.097958793	5.852175997
H198	1.611992150	-1.834639605	4.883651531
H199	-0.055655133	0.954135792	11.447777599
H200	-0.496269546	-0.716617639	11.000852926
H201	2.043482512	-0.720276992	9.949924359
H202	10.842999647	-7.434295204	4.166308747
H203	-1.998538944	-6.035716253	-3.918348218
H204	1.166457096	-6.105569672	-1.653738255
H205	-2.664316976	-6.403817991	-8.258432580
H206	-1.168976180	-5.617532477	-8.830709378
H207	-3.386173784	-4.430684946	-7.067997970
H208	-2.982009347	0.884201995	-9.694067674
H209	-3.602592581	-4.388880821	-9.607863817
H210	-3.492779903	-2.108808068	-2.245403357
H211	-4.065388978	-4.826298915	-0.938673755
H212	-2.385889386	-1.204564512	-8.383256939
H213	-5.173822134	-0.344495079	-8.767583594
H214	-1.228454676	-2.636374794	-9.519000059
H215	-1.499087430	-5.806792196	-2.242820479
H216	-1.825418579	-3.624440968	-7.238713599
H217	-1.415842722	-4.050222054	-10.337315457
H218	-6.913218430	-3.262014604	-2.705220136
H219	-3.532863701	-2.229230537	-0.496535321
H220	0.498698433	-5.464855311	-7.489986789
H221	-2.422302388	-4.202262405	-0.933759625
H222	-3.769203086	2.794563122	3.161149777
H223	-3.223427642	0.630869785	1.723401073
H224	-8.192125923	1.221633196	3.839638535
H225	-7.302922747	0.239980412	1.157563741
H226	-6.710204736	4.478702746	2.206763207
H227	-7.863209800	9.225355833	4.217998313
H228	-6.599591876	5.728173723	5.195949072
H229	-8.625997770	5.907671182	3.339826921
H230	-5.924682263	7.175045355	2.558081836
H231	-10.546025881	9.996546333	1.031197222
H232	-3.043503128	4.297003297	1.318618892
H233	-4.604792538	4.113148806	0.506001354
H234	-9.381696751	10.946533711	3.097326812
H235	-8.112899363	6.071968878	1.662210153
H236	-6.072960281	2.252892629	1.425297723
H237	-7.125620461	-0.166268432	3.611594736
H238	-9.826459253	2.279938205	-0.240005471
H239	-2.260151856	0.995188884	-2.351338407
H240	-10.233178581	0.559314261	-0.114790918
H241	10.342190072	2.759627780	2.678703862
H242	8.749862710	2.919799508	2.351481649
H243	1.652106877	5.776234564	-1.523330242
H244	-0.653428101	5.474871312	0.185525996
H245	1.956354529	7.883387381	0.152756947
H246	-3.170036328	3.288619595	-1.547671071
H247	-4.928704342	4.215445827	-2.753303643
H248	-6.445832080	3.451777419	-4.199162187
H249	-4.601389446	5.478657620	-1.859093408
H250	-5.376070342	1.310352440	-3.930158429
H251	-7.166482009	1.364008978	-3.809340566
H252	0.364367573	11.538171009	-2.158716473
H253	-0.723207290	12.926215565	-1.948784304
H254	-0.604366209	10.058221949	-3.493428689
H255	-2.319173145	9.646122055	-3.435342428
H256	-3.380362498	9.150091756	-6.193541609
H257	-2.531763097	4.782449416	-5.809672446
H258	-6.967246290	8.937920116	-0.714805690
H259	-2.792287110	8.690063583	1.976779827
H260	-4.425551665	7.819938754	-3.079590730
H261	1.216971398	5.200561632	-3.976572427
H262	2.547446498	6.980912822	-3.288351057
H263	-1.244980667	4.764505218	-3.456287753

	H264	-8.363329077	0.031815363	-1.379439755
	H265	3.937622512	9.752632951	-4.317344362
	H266	-6.032280703	-0.800748624	-2.923026636
	H267	0.733839202	6.186796261	-5.353113340
	H268	-3.171509106	7.070659928	0.159938077
	H269	3.651727502	10.081317710	-2.596042555
	H270	-2.674596889	7.679907033	-6.782131540
	H271	-4.850395024	8.954267108	-4.312615767
	H272	-5.352439948	-3.467133627	-3.274831879
	H273	-5.851657504	-3.237527596	-0.651577662
	H274	-6.529085717	10.644290474	1.031344275
	H275	-5.186160644	1.497257777	-1.466592163
	H276	-0.236585745	7.901086753	-3.852089173
	H277	-1.556197157	6.224190259	-6.115646999
	H278	-0.548672370	4.086713977	-4.916586458
	H279	-3.386790251	5.889978838	-3.736243430
	H280	1.100364775	8.879386129	2.753341893
	H281	-0.068397916	9.717922995	-0.160353677
	H282	0.459441320	12.563808106	0.078321640
	H283	2.593465767	9.274717463	1.865947079
	H284	-2.089203908	10.689015980	1.360686519
	H285	4.310900661	-3.381480854	-9.245439531
	H286	4.304824318	-4.774931319	-8.148657378
	H287	7.593671020	-5.640718988	-4.998451887
	H288	4.636681548	-5.578742842	-10.357819032
	H289	3.077590996	-5.942702858	-10.007064494
	H290	5.529871774	4.491126526	-1.026850939
	H291	6.061469365	7.172982579	0.341349409
	H292	5.022624411	7.764664383	-3.760404371
	H293	4.262378065	5.472435924	-0.226048134
	H294	7.130508627	7.679520507	-0.788296146
	H295	5.356201347	2.464590869	-6.911117311
	H296	6.126287773	6.382835431	-2.486105217
	H297	5.023474955	-6.959805825	5.049047686
	H298	6.717149316	-8.062702867	2.744106113
	H299	3.541335207	1.090618674	-6.895392359
	H300	4.982575300	-0.049176526	3.124822905
	H301	0.463869064	1.326245791	-8.712041739
	H302	-1.256333980	-2.139458896	-4.424114970
	H303	0.368589708	1.891083838	-7.039575729
	H304	4.249259332	-4.429752184	4.591801779
	H305	1.963836197	-0.153544203	-6.564954756
	H306	3.817088710	-4.934270484	2.953592875
	H307	4.487752272	-7.266794494	3.378963373
	H308	6.240012436	-5.585588130	2.575833587
	H309	6.584267085	-5.186370290	4.265700276
	H310	-5.359892906	-0.790762222	-6.454338234
	H311	5.503899783	-9.458080601	5.100237753
	H312	4.548669580	-11.907375071	3.520776322
	H313	0.091551004	-0.051083270	-4.277857070
	H314	5.873131722	-11.754946775	6.119635950
	H315	4.314408351	-12.108586539	5.761969934
	H316	5.528292309	-3.313598633	2.117841210
	H317	-0.877994494	2.103100111	-4.948866929
	H318	-3.530221639	-2.072707464	-5.444858504
	H319	7.214453254	-10.300372031	-1.413976029
	H320	6.224226043	-8.029506835	-3.650975151
	H321	6.299844976	-8.427665721	0.075109351
	H322	6.319351295	-10.905177725	-2.534476399
	H323	4.805104341	2.231619070	-9.638299017
	H324	6.212465010	-5.286908302	-0.213448937
	H325	1.957949398	-7.917668506	0.974481047
	H326	3.102207992	-2.305582512	-7.600916244
	H327	2.700536790	-9.319955767	-0.616128578
	H328	2.678104551	-7.148335999	-6.855434609
	H329	1.982258004	-5.535406845	-5.039709259
	H330	6.154727525	-12.521852079	3.923283823
	H331	5.363091987	-1.072855123	1.767595224
	H332	8.164367463	-6.113092597	0.834900086
	H333	7.176918760	-4.074182013	-0.208568686
	H334	8.691721954	-7.120588066	2.900080650
	H335	3.941224124	-2.674962959	5.278653342
	H336	3.477348376	-8.524016499	-3.467956535
	H337	4.652446422	-8.869560085	-2.189988527
	H338	1.584744875	-8.790206047	-1.879801692
	H339	1.207652762	-2.305117086	-6.085817787
	H340	-3.162725028	2.189501038	-5.996017522
	H341	-5.251488269	0.979090671	-6.662916929
	H342	2.139756118	-6.160889750	0.883035626
	H343	8.218817748	-7.665402501	0.722723812

H344	7.002929134	-9.527934746	0.905387954
H345	3.779924189	-1.001120678	4.984626054
H346	5.075561444	-8.012706671	-6.522707458
H347	6.227394796	-6.394094838	-3.013791857
H348	0.252742055	-3.126497446	-7.354347574
H349	4.272302664	3.857677949	-7.104029870
H350	7.624289616	-0.139305628	3.963325048
H351	7.695966160	0.247328125	2.227531320
H352	8.618458847	-7.285221857	-6.331804810
H353	7.836501531	-8.516313971	-5.588825366
H354	9.892222190	-9.103347842	-1.576726990
H355	8.131638140	-8.687160005	-2.957400200
H356	11.310766640	-8.160397929	-3.732194495
H357	9.835998626	-7.381391874	-1.145499309
H358	10.415645114	-0.189915974	2.246299387
H359	-2.408191211	13.358047658	3.370697114
H360	9.903624977	-7.140370488	5.652275447
H361	9.426103755	-4.714342659	4.465122433
H362	-4.198623898	12.483139958	1.086353660
H363	-3.682698966	10.545783283	3.425500848
H364	-5.232084932	11.318241463	3.038622103
H365	-6.216814390	6.991209384	-1.671810247
H366	2.847236899	-0.890164780	0.669584867
H367	1.962223424	-4.076996397	0.230757103
H368	1.207222493	-1.171879340	0.581138292
Mo369	3.652420677	-4.527208739	-2.346111720
N370	2.043619684	-0.959882785	0.036930635
N371	0.667784769	1.487692967	-0.486274247
N372	-0.080846930	2.138829481	0.049831923
N373	-7.324177279	-6.916692139	-0.040843298
N374	-8.901849516	-4.186695783	1.965800751
N375	-8.294179841	-4.404969607	4.702577976
N376	-5.650831800	-5.360667234	4.727759277
N377	-4.294243128	-3.060360979	3.744735034
N378	-4.768740796	-1.283463826	5.952067712
N379	-3.728841299	-2.637312851	8.322069369
N380	-1.365935399	-4.205619152	7.668791165
N381	-0.004212922	-2.470327339	5.944742967
N382	-0.022336084	0.549454822	6.502558316
N383	-0.173922225	0.514488292	9.364217677
N384	5.091552980	-11.494307286	5.513607441
N385	5.944506845	-9.323157215	4.184608642
N386	5.085160851	-3.268628558	3.037823101
N387	4.253988170	-1.873908336	4.733856991
N388	4.942601253	-0.976809309	2.706474003
N389	8.749380130	-7.210525060	3.939045120
N390	-5.990059713	-2.826401190	-2.768174917
N391	-2.159634343	-5.605096577	-3.003030388
N392	-6.102261981	-0.308096199	-2.018459652
N393	-8.350520976	0.934452039	-0.909582548
N394	-7.880967247	0.727984515	1.855747398
N395	-5.654586593	2.380889835	2.349541890
N396	-3.038499904	2.457658974	-0.959129946
N397	-2.627357130	0.362460420	-0.328431525
N398	-6.100370434	5.075217103	2.761034131
N399	-9.480432392	8.181224118	1.500785943
N400	-9.262737198	9.994525536	2.761339073
N401	9.508385575	2.234180704	2.404613772
N402	6.567164561	6.874347219	-0.499368333
N403	4.476757988	8.206555825	-3.025320039
N404	1.790107973	7.657832952	-3.449894789
N405	-2.956380518	6.386846493	-4.545682670
N406	-3.196984756	8.161233126	-6.059336137
N407	-4.181995576	8.270941313	-3.966292822
N408	0.773283050	6.197736927	-1.168503767
N409	1.209599915	8.116707809	0.811887551
N410	0.061686880	10.573291359	0.379537212
N411	-1.565977027	10.298729632	-3.221081012
N412	-2.276829921	11.687724563	1.415482342
N413	3.715116055	-5.164709238	-10.200707278
N414	2.256706430	-2.839263448	-7.821281262
N415	1.287916949	0.019622135	-7.327562818
N416	3.419748916	1.943932386	-7.450828357
N417	-1.919756175	-3.229204912	-9.994667405
N418	-3.403751330	-1.304855834	-8.447275461
N419	7.740907538	-7.534261114	-5.866345379
N420	3.969809789	-5.858697030	-4.254655972
N421	3.297350262	-6.848651561	-6.107217507
N422	8.843628406	-7.951749318	-2.943650329
O423	-6.735618920	-4.891102005	1.925120489

	O424 -7.295291508 -2.357243102 4.644833211<br>O425 -5.462285585 -4.703355308 6.908649759<br>O426 -2.554435364 -3.692439481 5.070434949<br>O427 -2.846135506 -0.232825812 5.345342499<br>O428 -2.944776237 -0.566882084 8.895897612<br>O429 -0.605122123 -3.707372080 9.772884282<br>O430 1.952794355 -3.623544655 6.302937374<br>O431 2.198674139 0.126870847 6.154964274<br>O432 1.556031907 1.982088892 9.297423899<br>O433 2.186046529 -0.291618444 11.939751505<br>O434 6.633370408 -10.540844701 2.345592466<br>O435 7.683384331 -7.817656081 5.883415152<br>O436 11.448521413 -4.939447926 4.602946483<br>O437 -5.920935652 -0.807824868 0.221442286<br>O438 -3.933171109 -4.449076072 -3.832083996<br>O439 -7.079865187 2.758972936 -0.395724982<br>O440 -6.192898444 3.142492069 -3.311404488<br>O441 -9.791231443 1.930851563 2.280439217<br>O442 -5.701828909 1.862907654 4.574874811<br>O443 -4.096307081 5.260611993 3.830713696<br>O444 -6.157361588 7.719157507 5.109680751<br>O445 10.596864981 -0.565281246 4.245079219<br>O446 3.682097024 4.621173026 3.856654611<br>O447 3.931652946 8.207007992 -0.810235989<br>O448 1.294430643 9.887138528 -3.720681212<br>O449 -1.206681461 7.229985623 -1.664084598<br>O450 -0.520868751 7.225555846 2.027934383<br>O451 2.507153790 5.513256950 1.326701803<br>O452 1.273882564 11.537807330 2.065575810<br>O453 -1.849596655 13.686095401 0.410149772<br>O454 -2.883665953 11.216522251 -1.594482532<br>O455 -4.341931060 13.997184570 3.221978640<br>O456 -5.272051955 6.990890577 -1.424511261<br>O457 1.432733325 -4.797798172 -8.693614658<br>O458 -0.388344340 -0.963859525 -8.546938997<br>O459 2.064046307 3.355475447 -8.606809185<br>O460 5.761681617 3.996903633 -9.268555531<br>O461 -5.187193206 -2.671952658 -8.842338625<br>O462 -1.756870377 -6.732223804 -5.824799220<br>O463 0.144836807 -5.923838383 -6.666998398<br>O464 -3.971924333 2.231237432 -8.531210219<br>O465 9.649197847 -5.948192913 -3.633617070<br>O466 12.298049190 -7.596577450 -2.036129649<br>O467 0.082209920 -6.214395625 -1.118911064<br>O468 -0.525735136 -7.243027575 0.795078811<br>O469 4.682219663 -11.204821616 -3.054043854<br>O470 2.422109027 -11.104633166 -3.050260241<br>O471 4.831122809 -6.027739061 -1.257907183<br>O472 4.743183663 -7.645173868 0.301934205<br>O473 2.329395198 -6.228105182 -1.922944335<br>O474 8.700993037 -6.891589304 1.125562836<br>O475 6.890350669 -4.826382609 0.343264927<br>O476 -4.202719734 4.834324505 -2.484064334<br>O477 7.276977230 -10.828716225 -2.240806719<br>O478 7.145935474 -8.947339883 0.114905672<br>S479 3.591007569 4.845739901 -2.472204492<br>S480 1.846598267 1.714542328 -3.355789910<br>S481 1.880189716 -3.445348043 -3.419527275<br>S482 3.573142414 1.700570095 -0.179804216<br>S483 0.381355902 -1.115121053 -2.022686065<br>S484 3.913997219 -0.923009973 -5.573992467<br>S485 3.220363483 -3.617500392 -0.150527177<br>S486 5.486008556 1.562015506 -3.180434788<br>S487 5.568725043 -3.356674949 -3.122223395<br>S488 6.246420605 -1.009772386 -0.415963673<br>end

Fe2-brNH2-Fe6-3b3 to Fe2-brNH2-Fe6-3b2

35, S=1,

reactant

Fe(139) -2.346<br>Fe(140) 0.050<br>Fe(141) -2.581<br>Fe(142) 2.636<br>Fe(143) 3.099	bm5expro2b2n2x3b32tc.car_3<br>C1 -7.902910492 -5.023782478 1.556926523<br>C2 -8.343170558 -6.170751283 0.640726146<br>C3 -8.610797908 -2.983848918 2.731432806<br>C4 -8.021798510 -3.226499158 4.118241176<br>C5 -7.705284141 -4.771400821 5.984990027<br>C6 -6.182415493 -4.937938877 5.928771683

Fe(144) 1.286<br>Fe(145) -0.395	C7 -4.221221618 -5.385583603 4.539966513<br>C8 -3.611994536 -3.981863576 4.486017562<br>C9 -3.887283415 -1.675027631 3.704073625<br>C10 -3.792887956 -1.012520672 5.087157332<br>C11 -4.769434603 -0.683780256 7.279137753<br>C12 -3.742170920 -1.300101863 8.240915779<br>C13 -2.979944305 -3.344743033 9.358458809<br>C14 -1.551539639 -3.750921291 8.972296929<br>C15 -0.085397039 -4.583006900 7.180168882<br>C16 0.711529595 -3.501591211 6.444777896<br>C17 0.639080953 -1.420513361 5.159742936<br>C18 1.000880151 -0.181643608 5.991260341<br>C19 -0.102372028 -1.086706603 3.845710211<br>C20 0.540781648 0.110922889 3.136788425<br>C21 -0.132338143 -2.315566083 2.929261496<br>C22 0.209066843 1.750122524 7.289787019<br>C23 0.565383047 1.418642469 8.750443632<br>C24 -0.962401663 2.751751227 7.148045864<br>C25 -0.738648556 3.985996697 8.031533568<br>C26 -1.134929555 3.169766935 5.680952148<br>C27 0.106922769 0.138977727 10.756876755<br>C28 1.546327131 -0.317246727 10.897434504<br>C29 5.452840909 -11.691852058 4.084452539<br>C30 6.086790641 -10.457586350 3.448137197<br>C31 6.455549073 -8.019381722 3.800011553<br>C32 7.701166165 -7.682037358 4.640411004<br>C33 5.355389919 -6.962652246 3.994188903<br>C34 5.806007216 -5.562009273 3.583179952<br>C35 4.648894970 -4.568018415 3.580820164<br>C36 4.759620133 -2.060490672 3.468120095<br>C37 10.005259664 -6.858502920 4.579995323<br>C38 10.321507288 -5.381043254 4.535358491<br>C39 -5.438489476 -2.567862823 -1.439740262<br>C40 -5.840131687 -1.148962351 -0.989467344<br>C41 -3.902366930 -2.698987460 -1.415429362<br>C42 -3.363144391 -4.143695120 -1.461530184<br>C43 -3.155048183 -4.741089718 -2.852370639<br>C44 -6.122896618 1.136214070 -1.935931968<br>C45 -7.223094522 1.684956517 -1.021791124<br>C46 -6.228868164 1.695222152 -3.361826634<br>C47 -9.412695009 1.297175902 0.002159845<br>C48 -9.030017290 1.343452910 1.486295040<br>C49 -7.371920403 0.818917378 3.208497315<br>C50 -6.170318128 1.739705686 3.435168751<br>C51 -4.498933286 3.263933897 2.489245931<br>C52 -4.868221338 4.613488602 3.124551493<br>C53 -3.863057524 3.579861358 1.124947222<br>C54 -3.362318019 2.386470312 0.384273619<br>C55 -3.094470130 1.081967742 0.746083999<br>C56 -2.613926415 1.206138043 -1.344474154<br>C57 -6.521538154 6.427657100 3.092546463<br>C58 -6.366214001 6.657039577 4.593938687<br>C59 -8.008924820 6.539420583 2.679378260<br>C60 -8.590456485 7.917317248 2.557925128<br>C61 -8.443240992 9.039228843 3.351825200<br>C62 -9.834239491 9.456226765 1.677622879<br>C63 9.202523169 1.249237141 3.420211810<br>C64 10.163714855 0.082492230 3.292962030<br>C65 7.773265283 0.683158071 3.220599816<br>C66 6.698136929 1.727520604 3.409706821<br>C67 6.115964247 1.954925713 4.669879201<br>C68 6.250370238 2.507036749 2.327036745<br>C69 5.115607902 2.915934227 4.846658562<br>C70 5.265678647 3.479987135 2.491742829<br>C71 4.692401747 3.677345524 3.750559457<br>C72 5.581218815 6.572106947 -1.558712476<br>C73 4.593211967 7.738812935 -1.753044807<br>C74 4.808180469 5.315510125 -1.178954112<br>C75 3.587043594 9.312846341 -3.372859446<br>C76 2.103226876 8.968143848 -3.534969210<br>C77 0.421633517 7.130773348 -3.452052886<br>C78 -0.080870935 6.848781603 -2.012656966<br>C79 0.420243799 5.872386884 -4.346414419<br>C80 -0.859112741 5.034988560 -4.456979306<br>C81 -1.987739409 5.605812231 -5.328946951<br>C82 -3.451480187 7.577246126 -4.877139378<br>C83 0.362752028 5.897160819 0.197989459<br>C84 0.283057065 7.142270239 1.090020924<br>C85 1.304216524 4.889488530 0.887761727<br>C86 1.496959806 9.155360360 1.776547834

	C87	0.958309015	10.531323385	1.423600623
	C88	-0.347842294	11.863228844	-0.139849473
	C89	-1.564905379	12.493929995	0.576872436
	C90	-0.548542009	11.872634183	-1.659637604
	C91	-1.771504639	11.092376370	-2.127451191
	C92	-3.564236597	12.120270037	1.923869373
	C93	-3.415187196	13.261471970	2.918133086
	C94	-4.295279140	10.926173482	2.596335705
	C95	-4.598908942	9.827431863	1.605641999
	C96	-5.775439131	9.862608995	0.842081475
	C97	-3.686561655	8.785728734	1.369498716
	C98	-6.025087189	8.906509959	-0.143933940
	C99	-3.906881838	7.835372312	0.369324154
	C100	-5.075947297	7.912249914	-0.383739317
	C101	3.774062064	-4.319906591	-9.012589141
	C102	2.377141572	-4.001267892	-8.505212832
	C103	1.029936308	-2.385207191	-7.179057444
	C104	0.559346575	-1.043838940	-7.756350024
	C105	1.003412080	1.377900226	-7.776075824
	C106	2.210220298	2.285268322	-8.003993926
	C107	4.529093679	2.854036104	-7.547140787
	C108	5.028436183	3.073825409	-8.960123489
	C109	-2.970524081	-3.663088351	-9.082933413
	C110	-3.942221728	-2.510547065	-8.803304371
	C111	-2.511404092	-4.304623054	-7.753567264
	C112	-1.870011947	-5.684179396	-7.993163891
	C113	-1.179967507	-6.180377835	-6.741865417
	C114	-4.226803860	-0.157484587	-8.215157411
	C115	-3.637920360	1.078876627	-8.864719468
	C116	-4.679398039	0.038192772	-6.746951545
	C117	-3.475582753	0.061094738	-5.852643694
	C118	-2.749334126	1.249330282	-5.664873424
	C119	-2.955392777	-1.146703218	-5.360890327
	C120	-1.480540517	1.205260791	-5.082856435
	C121	-1.689260160	-1.185035968	-4.774490382
	C122	-0.934580375	-0.013203562	-4.677644300
	C123	7.583369173	-6.693729910	-4.675452183
	C124	8.772304083	-6.822272339	-3.708280263
	C125	6.260633298	-6.934611412	-3.933022234
	C126	5.008324627	-6.660078338	-4.700488332
	C127	4.583955736	-7.275021558	-5.860045947
	C128	2.957377163	-5.991604580	-5.083400385
	C129	9.937704736	-8.076942884	-1.994196347
	C130	11.292555659	-7.910752248	-2.645937600
	C131	0.333427014	-6.821664621	0.019532747
	C132	1.829751887	-7.034865554	0.318989897
	C133	2.732550734	-7.203340643	-0.938550032
	C134	2.598737249	-8.650542073	-1.478535274
	C135	3.633458153	-9.076813493	-2.523182549
	C136	3.568699720	-10.573859479	-2.904075775
	C137	4.200940095	-6.945095499	-0.575365913
	C138	3.534501440	-0.930175140	-2.281769594
	Fe139	3.741688092	-2.212302158	-3.796622902
	Fe140	1.852199147	0.457212077	-1.307829808
	Fe141	4.802659777	0.372388659	-1.449854045
	Fe142	3.605924112	0.351867710	-3.753844686
	Fe143	3.663712995	2.583206956	-2.246989895
	Fe144	4.802997728	-2.146208115	-1.505785617
	Fe145	2.237894263	-2.147408822	-1.511864825
	H146	-6.601549171	-7.231846512	0.662434955
	H147	-6.767879786	-6.284822135	-0.655733454
	H148	-9.823104609	-4.284912410	1.551466858
	H149	-9.045445937	-5.775273679	-0.110606949
	H150	-8.928619294	-6.868189112	1.265131098
	H151	-7.890198058	-2.349297581	2.194013151
	H152	-8.894257251	-5.077773734	4.217008731
	H153	-9.543321759	-2.415517444	2.847092602
	H154	-8.152828935	-5.714797429	6.324756077
	H155	-6.262145219	-5.413308701	3.922206228
	H156	-7.909530306	-4.002283721	6.741347136
	H157	-2.902795969	-1.576890720	3.230681758
	H158	-5.122088730	-3.363645482	3.237059271
	H159	-4.019444035	-5.907284127	3.594800236
	H160	-4.531400125	0.383909456	7.193132952
	H161	-5.617666748	-1.800547851	5.639234003
	H162	-3.726424050	-5.931757557	5.351200671
	H163	-4.615348942	-1.137541560	3.081178244
	H164	-4.434051942	-3.197942370	7.792256922
	H165	-5.770332263	-0.790506377	7.720093969
	H166	-0.602898647	-3.182319511	3.414338366

H167	-0.696273035	-2.097832680	2.008870498
H168	0.890480954	-2.605504722	2.635317693
H169	-1.132128408	-0.802038433	4.109198023
H170	-3.519533606	-4.257839445	9.648808195
H171	-1.346776696	2.313316044	5.026263231
H172	-1.048927723	0.109324008	8.959278292
H173	-0.675587141	3.732082632	9.099483361
H174	-0.001875313	0.330194518	2.207169827
H175	1.592535751	-0.086661476	2.874168161
H176	9.276602702	1.627794634	4.457648989
H177	-1.966365118	3.882562398	5.577363004
H178	-1.569110533	4.694856905	7.896425229
H179	-0.218460923	3.661453338	5.314843338
H180	0.194963090	4.498533072	7.748874829
H181	-1.887842390	2.248031170	7.482539824
H182	1.120856870	2.217158457	6.889163857
H183	-0.990130584	0.256590255	6.297707037
H184	0.516688237	1.021715032	3.752472624
H185	1.526803886	4.070664707	0.187371674
H186	0.754315087	4.474917758	1.745129268
H187	2.757447199	5.158359654	2.193425011
H188	3.347293146	4.646175702	4.758890641
H189	4.908297597	4.060401868	1.640551743
H190	6.653393879	2.326575752	1.327561064
H191	6.438345548	1.360183442	5.529211683
H192	-1.026008483	-2.524852575	5.935627208
H193	-0.218948279	-5.424438975	6.483377765
H194	0.534221797	-4.927045176	8.017208150
H195	-2.152331036	-4.124268627	7.022372492
H196	-2.891117735	-2.700177712	10.241836604
H197	4.658775111	3.063559303	5.829192775
H198	1.617027729	-1.837468802	4.884169156
H199	-0.080999376	0.957039108	11.470921691
H200	-0.532338220	-0.713925498	11.034975873
H201	2.000554869	-0.735749617	9.968566044
H202	10.849481947	-7.427167124	4.160509529
H203	-1.959235788	-6.036654112	-3.883239748
H204	1.232639775	-6.073592205	-1.664752251
H205	-2.655357863	-6.405670444	-8.260487567
H206	-1.151649894	-5.629800098	-8.824529304
H207	-3.366272057	-4.424378677	-7.069760529
H208	-2.926382072	0.871034222	-9.698355570
H209	-3.591669190	-4.403149306	-9.610316685
H210	-3.487255690	-2.112022862	-2.249263200
H211	-4.047792982	-4.821732778	-0.921417114
H212	-2.368972388	-1.215136264	-8.397992070
H213	-5.148802552	-0.344331656	-8.805914752
H214	-1.218283110	-2.648712984	-9.543703513
H215	-1.462016675	-5.788342785	-2.206837056
H216	-1.803779812	-3.624995183	-7.254367538
H217	-1.406013313	-4.070407721	-10.348476381
H218	-6.897950121	-3.265134868	-2.729022808
H219	-3.533812454	-2.217724608	-0.499874605
H220	0.504980978	-5.483396430	-7.473675621
H221	-2.409223136	-4.187862003	-0.915398678
H222	-3.766546303	2.791615470	3.158879828
H223	-3.226671642	0.626480804	1.723854346
H224	-8.188532425	1.197551275	3.838825971
H225	-7.297536337	0.237612281	1.149487024
H226	-6.709062129	4.480716582	2.224899703
H227	-7.841326266	9.235223832	4.231224420
H228	-6.566900256	5.748596090	5.212180455
H229	-8.612178309	5.920161937	3.367598163
H230	-5.913061752	7.175095366	2.558890669
H231	-10.521815101	10.009872920	1.043530620
H232	-3.041009538	4.290754858	1.314367148
H233	-4.605190928	4.111616107	0.505725224
H234	-9.352787812	10.959718063	3.107056065
H235	-8.111129949	6.074140930	1.685684328
H236	-6.065355797	2.245448142	1.424093079
H237	-7.107058286	-0.175675141	3.597419927
H238	-9.805134512	2.295217491	-0.237823856
H239	-2.280317797	0.983752985	-2.355392862
H240	-10.234094258	0.578571228	-0.121812796
H241	10.355943629	2.764476065	2.665427011
H242	8.765047606	2.928860974	2.334325762
H243	1.651435630	5.778924696	-1.532730537
H244	-0.646122303	5.460375557	0.179996976
H245	1.947639346	7.883800366	0.151684963
H246	-3.181626904	3.279467837	-1.551711908

	H247	-4.935131134	4.208695581	-2.763601804
	H248	-6.440510790	3.443942186	-4.218227587
	H249	-4.600033988	5.468950823	-1.865500928
	H250	-5.380779112	1.304077008	-3.948704642
	H251	-7.171220706	1.356534764	-3.822453979
	H252	0.371243171	11.528773981	-2.152915881
	H253	-0.710112612	12.922584828	-1.950427909
	H254	-0.610362353	10.061004494	-3.494802766
	H255	-2.326134264	9.652186525	-3.428131502
	H256	-3.381544848	9.153856863	-6.192342131
	H257	-2.527412692	4.785680299	-5.826636515
	H258	-6.951704097	8.938652859	-0.723067046
	H259	-2.782753597	8.690413631	1.976977942
	H260	-4.428519431	7.812408417	-3.083204840
	H261	1.212336389	5.201990792	-3.977891309
	H262	2.541832381	6.986907699	-3.288649505
	H263	-1.252800676	4.767587279	-3.464076189
	H264	-8.366487891	0.032046523	-1.383162989
	H265	3.930008984	9.764985260	-4.313321233
	H266	-6.025853112	-0.808387344	-2.938224330
	H267	0.732865152	6.188361509	-5.355958650
	H268	-3.161519048	7.066419610	0.164639161
	H269	3.639453928	10.083732939	-2.590674858
	H270	-2.681412526	7.684256347	-6.788631303
	H271	-4.855863426	8.948726361	-4.313693922
	H272	-5.331463964	-3.481293508	-3.279487865
	H273	-5.850107870	-3.242886987	-0.664893446
	H274	-6.514321787	10.648099861	1.019533586
	H275	-5.183292075	1.488608984	-1.486456047
	H276	-0.243342094	7.901167151	-3.854720422
	H277	-1.554780828	6.231969636	-6.120392438
	H278	-0.551358393	4.088538554	-4.921746790
	H279	-3.394365915	5.882677923	-3.749436992
	H280	1.072481792	8.872343571	2.751490264
	H281	-0.073740933	9.720450179	-0.148763906
	H282	0.471104826	12.565847605	0.080458643
	H283	2.585544145	9.249905737	1.890167027
	H284	-2.069654986	10.698238908	1.376625825
	H285	4.310032625	-3.386974005	-9.240644762
	H286	4.298815088	-4.789792775	-8.155824146
	H287	7.612293335	-5.651460439	-5.025874560
	H288	4.626025829	-5.576775204	-10.371994381
	H289	3.066364471	-5.937264408	-10.020939818
	H290	5.526105395	4.495275010	-1.044510334
	H291	6.071000813	7.164133707	0.332345407
	H292	5.006516304	7.767588909	-3.766188136
	H293	4.258265796	5.475823398	-0.241305687
	H294	7.138745365	7.671257377	-0.797762154
	H295	5.359221176	2.420278986	-6.967073238
	H296	6.123579922	6.391203475	-2.499806725
	H297	5.034921712	-6.969507882	5.049395086
	H298	6.725265431	-8.064647702	2.737601180
	H299	3.530841386	1.071054848	-6.932134729
	H300	4.969502396	-0.063084084	3.072859981
	H301	0.443555273	1.324906087	-8.717488456
	H302	-1.281140846	-2.123664811	-4.393059380
	H303	0.359408239	1.898503490	-7.046705278
	H304	4.265229874	-4.428743578	4.598353544
	H305	1.939430169	-0.147466785	-6.561900822
	H306	3.812708470	-4.949950114	2.971082367
	H307	4.495701309	-7.275560301	3.380580241
	H308	6.239525777	-5.592818331	2.572770449
	H309	6.591200180	-5.190422322	4.259991732
	H310	-5.356588649	-0.789682432	-6.491156850
	H311	5.508346624	-9.467289568	5.088123981
	H312	4.546926379	-11.904942443	3.496674205
	H313	0.060585813	-0.035681748	-4.236798148
	H314	5.856721146	-11.769952357	6.102431169
	H315	4.294669365	-12.103030942	5.735413714
	H316	5.518030925	-3.338662155	2.099909527
	H317	-0.898944888	2.115825349	-4.937413609
	H318	-3.537573098	-2.066167655	-5.455084174
	H319	7.223931935	-10.296445668	-1.425730627
	H320	6.227062768	-7.986856678	-3.605332810
	H321	6.296702522	-8.428572284	0.073870018
	H322	6.327751491	-10.893407397	-2.548321466
	H323	4.773236059	2.258568733	-9.679581666
	H324	6.203843001	-5.278140057	-0.197766955
	H325	1.949297490	-7.897601590	0.986049612
	H326	3.095214429	-2.298077585	-7.623880960

H327	2.682927072	-9.317491212	-0.605652333
H328	2.671221223	-7.151167963	-6.818124996
H329	1.979845074	-5.534533990	-5.000577134
H330	6.148424590	-12.529518475	3.906240109
H331	5.359286282	-1.099348810	1.729801229
H332	8.158488886	-6.112609839	0.839764152
H333	7.171490316	-4.067753493	-0.182798450
H334	8.696968630	-7.116164691	2.893573413
H335	3.941479924	-2.664618969	5.262610835
H336	3.485987511	-8.500087880	-3.449404120
H337	4.649231777	-8.860429341	-2.165217125
H338	1.581301351	-8.776451991	-1.879051854
H339	1.204507208	-2.302500597	-6.095224395
H340	-3.167108782	2.195664544	-6.017198697
H341	-5.247541235	0.979531471	-6.703206959
H342	2.153907810	-6.150806118	0.893017022
H343	8.217316714	-7.664769283	0.713838498
H344	7.006114186	-9.529938476	0.897111706
H345	3.783502517	-0.992082495	4.948505821
H346	5.076211494	-7.996329405	-6.501569804
H347	6.252272380	-6.332166402	-3.017784504
H348	0.250051512	-3.127396902	-7.362628151
H349	4.279599861	3.827119286	-7.096359849
H350	7.633987930	-0.136563797	3.941599139
H351	7.710032309	0.255132612	2.207099550
H352	8.594290187	-7.340805794	-6.339472442
H353	7.799927266	-8.542784462	-5.562322934
H354	9.897112030	-9.093760311	-1.573323978
H355	8.144249615	-8.688508356	-2.967998592
H356	11.319102337	-8.165371862	-3.733157060
H357	9.838044502	-7.368253259	-1.156585502
H358	10.423057926	-0.186438259	2.240250974
H359	-2.404938088	13.362443603	3.379991492
H360	9.909299700	-7.133545792	5.645919988
H361	9.434386139	-4.707009017	4.447605210
H362	-4.184003419	12.488391597	1.088322022
H363	-3.677176126	10.545201436	3.424415353
H364	-5.225182784	11.317571655	3.033783874
H365	-6.207571569	6.982321342	-1.665261698
H366	2.843550092	-0.853123979	0.673418883
H367	2.024332920	-4.063005481	0.270357899
H368	1.205241439	-1.159257780	0.595612146
Mo369	3.661121631	-4.503329001	-2.338682143
N370	2.037713386	-0.941826694	0.046871587
N371	0.656266245	1.490783606	-0.494641864
N372	-0.083361528	2.148052493	0.046154564
N373	-7.283397998	-6.904960840	-0.026638228
N374	-8.901368176	-4.190699228	1.971378126
N375	-8.308348416	-4.409606152	4.711916446
N376	-5.662795546	-5.361410333	4.749322108
N377	-4.295875653	-3.070728514	3.755528159
N378	-4.786390493	-1.299159458	5.961025383
N379	-3.762992502	-2.655638955	8.343321464
N380	-1.380426061	-4.173934581	7.691259052
N381	-0.008634329	-2.464457856	5.939693050
N382	-0.028883359	0.533469038	6.512693492
N383	-0.211760127	0.508148608	9.389479110
N384	5.081791277	-11.499367237	5.492566051
N385	5.954793498	-9.331268600	4.175342118
N386	5.081694028	-3.282578936	3.022771346
N387	4.250174691	-1.870649019	4.704366874
N388	4.935201036	-0.995313286	2.665890763
N389	8.755668267	-7.205150571	3.932546410
N390	-5.972085375	-2.834016671	-2.783951127
N391	-2.124956252	-5.600435674	-2.971740127
N392	-6.104111300	-0.315526831	-2.034313359
N393	-8.346860723	0.935297311	-0.914775051
N394	-7.874075862	0.724277713	1.849972377
N395	-5.655369983	2.383253503	2.350631012
N396	-3.048664589	2.449749364	-0.961674034
N397	-2.634808203	0.355835917	-0.328772322
N398	-6.094174453	5.076810076	2.774086855
N399	-9.464181235	8.191130838	1.517191338
N400	-9.238058544	10.006532287	2.773227731
N401	9.521227096	2.241039385	2.390501565
N402	6.571934524	6.867703757	-0.511844279
N403	4.467981773	8.212739881	-3.027822414
N404	1.783168863	7.662158059	-3.451437209
N405	-2.962642924	6.382504429	-4.556105694
N406	-3.199060340	8.164371494	-6.061626767

N407	-4.186216227	8.265737998	-3.969130984
N408	0.773274512	6.198431555	-1.174040475
N409	1.200932377	8.113144963	0.812043040
N410	0.068732767	10.577030197	0.386090291
N411	-1.569795196	10.301690788	-3.214817367
N412	-2.262782645	11.696203963	1.425139532
N413	3.706473122	-5.160199652	-10.210177585
N414	2.251573850	-2.838544507	-7.832084251
N415	1.269324321	0.022493211	-7.330226660
N416	3.410506606	1.927706170	-7.482563189
N417	-1.910055454	-3.246899881	-10.011838913
N418	-3.387059953	-1.313708319	-8.462650483
N419	7.719355035	-7.564538337	-5.856738749
N420	3.974920793	-5.843557065	-4.232660015
N421	3.296155120	-6.839501306	-6.079151997
N422	8.855106019	-7.952136743	-2.953580273
O423	-6.730575909	-4.881260373	1.949685794
O424	-7.311727320	-2.360830710	4.658669992
O425	-5.488202419	-4.715386675	6.934803932
O426	-2.562650239	-3.696620707	5.090941877
O427	-2.851226661	-0.257044289	5.376938288
O428	-2.981700306	-0.583343438	8.913553888
O429	-0.650060874	-3.741395485	9.822390922
O430	1.941716874	-3.621430872	6.316749470
O431	2.194869301	0.144085439	6.135243500
O432	1.535840100	1.951866653	9.315746797
O433	2.158386092	-0.296753759	11.954880574
O434	6.641402342	-10.546180130	2.333503303
O435	7.698435106	-7.831900211	5.875462117
O436	11.454897336	-4.933226338	4.611502438
O437	-5.921558688	-0.811661031	0.206300547
O438	-3.910751805	-4.472004463	-3.815824245
O439	-7.067642166	2.755912387	-0.409742250
O440	-6.196675111	3.135632485	-3.327495724
O441	-9.782837681	1.927426492	2.280420373
O442	-5.710171874	1.870302107	4.577163595
O443	-4.078131871	5.261599310	3.821320137
O444	-6.117238614	7.737045243	5.104847865
O445	10.616142234	-0.543870246	4.240737418
O446	3.693445653	4.627027813	3.847902804
O447	3.951159989	8.223261593	-0.805308888
O448	1.285950379	9.890360196	-3.732681007
O449	-1.212723950	7.221651489	-1.666040859
O450	-0.522103654	7.205403992	2.027934729
O451	2.515879380	5.518658606	1.316971646
O452	1.306261438	11.533806371	2.059441110
O453	-1.843348740	13.690062498	0.406633737
O454	-2.875376583	11.218168625	-1.577562728
O455	-4.340037560	13.996564937	3.229682065
O456	-5.261480018	6.983733524	-1.422677692
O457	1.433161827	-4.809279926	-8.681463718
O458	-0.383415990	-0.972348921	-8.572673591
O459	2.052303425	3.355690393	-8.615849816
O460	5.697022696	4.037139793	-9.296291850
O461	-5.172459368	-2.675887248	-8.865195379
O462	-1.767537198	-6.729567094	-5.817733360
O463	0.146790666	-5.942186692	-6.652778620
O464	-3.943679724	2.224666664	-8.567567121
O465	9.657695735	-5.947666205	-3.644395462
O466	12.306469990	-7.595851436	-2.039018204
O467	0.071244151	-6.173762997	-1.090857823
O468	-0.524001938	-7.200228725	0.831239244
O469	4.685203517	-11.180621395	-3.067991273
O470	2.424215527	-11.086740623	-3.058283180
O471	4.823325442	-6.004797032	-1.238669523
O472	4.751888338	-7.635406458	0.303153311
O473	2.327620687	-6.225540081	-1.922693439
O474	8.700574671	-6.891883832	1.117909376
O475	6.875881969	-4.817788688	0.366728272
O476	-4.207387053	4.826535367	-2.496123067
O477	7.285369083	-10.822930815	-2.253889487
O478	7.144012906	-8.946053157	0.107629262
S479	3.585622094	4.854911254	-2.486067530
S480	1.840906731	1.721785185	-3.344766803
S481	1.880617698	-3.430546051	-3.401919353
S482	3.560062882	1.707625490	-0.192845009
S483	0.400136027	-1.128074715	-2.032610198
S484	3.888080084	-0.917901379	-5.577927129
S485	3.271997561	-3.582032311	-0.126773458
S486	5.446085386	1.569906301	-3.210092533

	S487 5.577498902 -3.338095389 -3.128457538<br>S488 6.257241450 -0.970544878 -0.429797219<br>end

TS

Fe(139) -2.301<br>Fe(140) -0.015<br>Fe(141) -2.622<br>Fe(142) 2.622<br>Fe(143) 3.047<br>Fe(144) 1.659<br>Fe(145) -0.659	bm5expro2b2n2x3b32td.car_1<br>C1 -7.911196097 -5.026721429 1.549180599<br>C2 -8.363452449 -6.175373231 0.640980468<br>C3 -8.603837762 -2.981493340 2.724290335<br>C4 -8.013226748 -3.224064436 4.110454352<br>C5 -7.693240033 -4.768606712 5.976829560<br>C6 -6.170481100 -4.934238522 5.915064680<br>C7 -4.214651948 -5.382049752 4.519869698<br>C8 -3.608239001 -3.976990131 4.472217856<br>C9 -3.887479879 -1.668091584 3.693105028<br>C10 -3.788927837 -1.001689350 5.073738053<br>C11 -4.748826643 -0.669019402 7.270517857<br>C12 -3.719910543 -1.287951224 8.228903629<br>C13 -2.957102950 -3.335225416 9.341718706<br>C14 -1.532090298 -3.747807661 8.949823709<br>C15 -0.076263313 -4.599035885 7.160445661<br>C16 0.717836172 -3.508983728 6.435344282<br>C17 0.636005769 -1.421244402 5.163391676<br>C18 1.001054107 -0.183383761 5.994513450<br>C19 -0.108768855 -1.085587591 3.850718330<br>C20 0.533554381 0.112800208 3.142647450<br>C21 -0.140098412 -2.312369048 2.931724503<br>C22 0.221100021 1.762044635 7.277773803<br>C23 0.582138242 1.431622223 8.737666958<br>C24 -0.947879557 2.766768620 7.137079802<br>C25 -0.723791445 3.999668999 8.021906759<br>C26 -1.117401871 3.187579414 5.670453138<br>C27 0.129625302 0.140637018 10.740125150<br>C28 1.570091917 -0.311555111 10.883402186<br>C29 5.453068166 -11.689303274 4.097327383<br>C30 6.081351331 -10.454212264 3.456890090<br>C31 6.447341838 -8.015051040 3.803665980<br>C32 7.692406829 -7.677317016 4.644604200<br>C33 5.347612364 -6.957527684 3.994672432<br>C34 5.801935736 -5.557926664 3.584339805<br>C35 4.647063783 -4.561853110 3.579651086<br>C36 4.765092735 -2.052859422 3.489562564<br>C37 9.999846487 -6.862861085 4.583829716<br>C38 10.316022537 -5.385277640 4.538853615<br>C39 -5.443607674 -2.564780157 -1.431809337<br>C40 -5.840391420 -1.145089112 -0.979032809<br>C41 -3.907455090 -2.700445866 -1.415795815<br>C42 -3.374416690 -4.147074779 -1.471624417<br>C43 -3.172292027 -4.738072212 -2.866317158<br>C44 -6.124322592 1.140735732 -1.924503789<br>C45 -7.228563018 1.687132216 -1.013462105<br>C46 -6.227453024 1.699788930 -3.350644556<br>C47 -9.419612143 1.290780416 0.004932175<br>C48 -9.036863556 1.343774060 1.488813931<br>C49 -7.378234504 0.824985199 3.212669619<br>C50 -6.170844315 1.738900549 3.435434997<br>C51 -4.501138060 3.263833768 2.489299869<br>C52 -4.875613001 4.612288008 3.123671464<br>C53 -3.864062663 3.582025596 1.126177043<br>C54 -3.359289696 2.390347498 0.385617383<br>C55 -3.093183307 1.085260362 0.746429705<br>C56 -2.608636488 1.211336836 -1.343057304<br>C57 -6.531545926 6.423299311 3.085079687<br>C58 -6.387611209 6.646002391 4.588900974<br>C59 -8.017036007 6.533665908 2.664725220<br>C60 -8.601610516 7.910580889 2.545558502<br>C61 -8.458654551 9.031396810 3.341814843<br>C62 -9.850595272 9.447079481 1.668057781<br>C63 9.194697112 1.242353010 3.427753630<br>C64 10.154263553 0.074170304 3.297312730<br>C65 7.764752754 0.678703032 3.233096098<br>C66 6.692607368 1.726027497 3.422311641<br>C67 6.122704034 1.968025854 4.685615515<br>C68 6.234222510 2.493528263 2.335566714<br>C69 5.122427321 2.929405447 4.860801557<br>C70 5.249186364 3.466543069 2.498465488<br>C71 4.686717506 3.676956332 3.759940038<br>C72 5.577833906 6.567328795 -1.551518813<br>C73 4.588556525 7.731667517 -1.751367236<br>C74 4.807778049 5.309881953 -1.166584474

	C75	3.591798780	9.308228679	-3.373398616
	C76	2.107422082	8.965224043	-3.531423806
	C77	0.424364313	7.129246328	-3.449794195
	C78	-0.078797727	6.850290462	-2.010191041
	C79	0.421264235	5.870822333	-4.343921487
	C80	-0.858165767	5.033640943	-4.452123097
	C81	-1.988594145	5.604036650	-5.322074556
	C82	-3.449876918	7.578090717	-4.873691288
	C83	0.358849538	5.901419585	0.202392620
	C84	0.283500451	7.149297679	1.090911069
	C85	1.294307166	4.890241895	0.895171926
	C86	1.501478316	9.160824429	1.772552857
	C87	0.948278019	10.532233747	1.423338038
	C88	-0.355343588	11.859146131	-0.143013239
	C89	-1.572672108	12.488964016	0.574762793
	C90	-0.555324572	11.873080270	-1.662752057
	C91	-1.775700899	11.091260171	-2.134041682
	C92	-3.572139627	12.116130695	1.920736382
	C93	-3.419084296	13.259200423	2.912519458
	C94	-4.302554348	10.924819262	2.598349743
	C95	-4.608842059	9.824966366	1.609734571
	C96	-5.786533993	9.860249364	0.847962063
	C97	-3.695718311	8.784582117	1.370971156
	C98	-6.036282569	8.905770078	-0.139418629
	C99	-3.916067939	7.836100097	0.369179904
	C100	-5.085918180	7.913635958	-0.382512702
	C101	3.774571027	-4.316281885	-9.013355546
	C102	2.377755244	-3.997778992	-8.507238648
	C103	1.032210095	-2.385829076	-7.173383420
	C104	0.561841906	-1.042877112	-7.746872832
	C105	1.013288500	1.376652637	-7.771249518
	C106	2.217675466	2.288412416	-7.994060643
	C107	4.531834642	2.867800610	-7.529943392
	C108	5.053712805	3.061309925	-8.938886547
	C109	-2.982070073	-3.655760836	-9.077606134
	C110	-3.954755837	-2.505652627	-8.792773341
	C111	-2.524841499	-4.303353089	-7.749955085
	C112	-1.879588103	-5.680729387	-7.994888155
	C113	-1.181832548	-6.176352971	-6.748351761
	C114	-4.242711207	-0.155168183	-8.196103537
	C115	-3.664491361	1.084297994	-8.849105886
	C116	-4.681146154	0.036954594	-6.722893271
	C117	-3.468986351	0.056322688	-5.839866725
	C118	-2.739829107	1.242981833	-5.655284433
	C119	-2.945362833	-1.152907871	-5.356012709
	C120	-1.466720144	1.196732766	-5.082338993
	C121	-1.674461012	-1.194211689	-4.780327372
	C122	-0.917675247	-0.023203680	-4.683331268
	C123	7.579823041	-6.692964762	-4.673047736
	C124	8.763883165	-6.822460716	-3.701027100
	C125	6.253760191	-6.959958617	-3.946154149
	C126	5.002618233	-6.678792252	-4.714940477
	C127	4.579900470	-7.289272004	-5.877566467
	C128	2.955700453	-6.000737535	-5.103857925
	C129	9.931814952	-8.082503548	-1.990893546
	C130	11.285142739	-7.911856462	-2.644532301
	C131	0.343427602	-6.843042124	0.000306557
	C132	1.839354317	-7.033279164	0.309456572
	C133	2.741306004	-7.201743352	-0.947432194
	C134	2.602667414	-8.649908210	-1.488763762
	C135	3.632796726	-9.082933738	-2.534304053
	C136	3.565430731	-10.583741210	-2.899816759
	C137	4.212055398	-6.956832353	-0.583666004
	C138	3.530228182	-0.934301606	-2.278809829
	Fe139	3.739256348	-2.216187488	-3.789880728
	Fe140	1.858030180	0.439265501	-1.323324000
	Fe141	4.819665391	0.351934007	-1.447984212
	Fe142	3.611541184	0.343984309	-3.753449705
	Fe143	3.672525251	2.569162129	-2.234175330
	Fe144	4.811885332	-2.180900546	-1.478019028
	Fe145	2.251564298	-2.162922939	-1.499764862
	H146	-6.621912208	-7.236285252	0.645505298
	H147	-6.805504084	-6.296819843	-0.676239233
	H148	-9.828400419	-4.280328564	1.555122488
	H149	-9.074058010	-5.781915818	-0.103309655
	H150	-8.941973385	-6.870610374	1.273726954
	H151	-7.880942777	-2.352325409	2.183645895
	H152	-8.887292872	-5.074436996	4.211939639
	H153	-9.532531891	-2.407135988	2.840508116
	H154	-8.139400694	-5.712400365	6.317200638

H155	-6.258126598	-5.416230119	3.909913462
H156	-7.895353951	-3.999857439	6.734059668
H157	-2.904426105	-1.571120275	3.216510396
H158	-5.121420956	-3.357682664	3.228597407
H159	-4.015891566	-5.898439604	3.571177418
H160	-4.503562448	0.396542872	7.178279948
H161	-5.607143028	-1.794889183	5.640664921
H162	-3.716676418	-5.932410415	5.326370415
H163	-4.617034105	-1.131368790	3.071134880
H164	-4.412393499	-3.184275902	7.776592659
H165	-5.747848485	-0.766814677	7.717726820
H166	-0.610381441	-3.180078709	3.415261451
H167	-0.705311036	-2.091847105	2.012719185
H168	0.882251670	-2.602055617	2.636008222
H169	-1.137971657	-0.801492372	4.116133333
H170	-3.500183211	-4.244882828	9.636000830
H171	-1.329476933	2.333369834	5.012703811
H172	-1.020843279	0.109681480	8.940021030
H173	-0.658671182	3.744270323	9.089229653
H174	-0.013411503	0.337454571	2.216942054
H175	1.583572076	-0.086517363	2.874792107
H176	9.273038086	1.619250117	4.465249060
H177	-1.947519222	3.901978615	5.566645327
H178	-1.555464323	4.707417216	7.888876016
H179	-0.199245812	3.678527262	5.307145695
H180	0.208510489	4.514180210	7.738578987
H181	-1.874272382	2.264025422	7.469922305
H182	1.132787275	2.225356705	6.871890794
H183	-0.987937107	0.276790629	6.283233810
H184	0.514500948	1.021173068	3.761858780
H185	1.512092919	4.068426666	0.196783994
H186	0.741594053	4.481459905	1.753523206
H187	2.748514455	5.155538385	2.200714360
H188	3.348703499	4.654765024	4.769350847
H189	4.882699164	4.035605258	1.643697095
H190	6.627798512	2.302525664	1.334360072
H191	6.454356326	1.383934940	5.548759637
H192	-1.023164007	-2.534969106	5.935690421
H193	-0.210015957	-5.434414384	6.456526104
H194	0.544511896	-4.950287391	7.993704353
H195	-2.145716244	-4.148879902	7.008952703
H196	-2.860318296	-2.688688157	10.222645097
H197	4.673985996	3.086568478	5.845798370
H198	1.613143301	-1.835749612	4.882557451
H199	-0.063385919	0.955136942	11.456720714
H200	-0.506910039	-0.715883705	11.013139017
H201	2.028705707	-0.724683457	9.954225170
H202	10.843366137	-7.431912570	4.163606521
H203	-1.987142007	-6.037215466	-3.906978645
H204	1.213371599	-6.079418858	-1.664360236
H205	-2.663369400	-6.404406917	-8.260101031
H206	-1.165228977	-5.621546773	-8.829303129
H207	-3.381009395	-4.428237299	-7.068521039
H208	-2.963806699	0.879813630	-9.692605498
H209	-3.601772708	-4.393894809	-9.609690478
H210	-3.493912815	-2.110464515	-2.248405970
H211	-4.060806809	-4.825276621	-0.934203536
H212	-2.383274519	-1.208054878	-8.386553136
H213	-5.169662306	-0.344985459	-8.777911962
H214	-1.228277683	-2.640080349	-9.527137900
H215	-1.483753644	-5.798972342	-2.232870982
H216	-1.819597717	-3.624236630	-7.246143924
H217	-1.414432581	-4.056371350	-10.341242195
H218	-6.909824770	-3.261836902	-2.713151670
H219	-3.533111630	-2.225179623	-0.499206085
H220	0.499480700	-5.471690419	-7.484575585
H221	-2.419397805	-4.198247530	-0.928135575
H222	-3.768821476	2.793391957	3.160153975
H223	-3.226498307	0.629177981	1.723738996
H224	-8.192681097	1.213497293	3.839973350
H225	-7.302818738	0.239359509	1.155390314
H226	-6.711495670	4.477895049	2.213052414
H227	-7.857813913	9.227620344	4.221973041
H228	-6.590889547	5.733697743	5.201035807
H229	-8.622613258	5.910963213	3.347731491
H230	-5.921868537	7.174512783	2.558162084
H231	-10.540198600	9.999481766	1.035068528
H232	-3.044230193	4.295167001	1.317316102
H233	-4.606393198	4.112422863	0.505983946
H234	-9.374411053	10.949425292	3.100510039

H235	-8.113464099	6.071875911	1.668984393
H236	-6.070601866	2.248588253	1.424883186
H237	-7.121201778	-0.169435857	3.607807445
H238	-9.820740575	2.284488283	-0.238594083
H239	-2.270581098	0.990409124	-2.352832637
H240	-10.234905588	0.565037590	-0.117041654
H241	10.345290728	2.760008193	2.673900884
H242	8.753569964	2.922327123	2.344721626
H243	1.650265245	5.776520502	-1.526010637
H244	-0.652192345	5.469575551	0.183546826
H245	1.952186260	7.883621468	0.151684951
H246	-3.174293283	3.285820339	-1.548458509
H247	-4.932182071	4.213482475	-2.756689166
H248	-6.445639803	3.448722540	-4.205311207
H249	-4.601572021	5.475319337	-1.860864257
H250	-5.378494763	1.308210829	-3.935892531
H251	-7.168895821	1.361113331	-3.813057357
H252	0.365706043	11.533573726	-2.156677596
H253	-0.719554834	12.923810514	-1.949594730
H254	-0.607604818	10.058591889	-3.494073943
H255	-2.322589036	9.647423814	-3.433341630
H256	-3.381705194	9.151040565	-6.193204898
H257	-2.531598735	4.783313853	-5.815380059
H258	-6.963625651	8.937733715	-0.717106532
H259	-2.790997072	8.689494208	1.977002430
H260	-4.428499541	7.817863605	-3.080738987
H261	1.214072888	5.200762988	-3.976566581
H262	2.544348151	6.983441487	-3.290507099
H263	-1.249748850	4.766137989	-3.458463924
H264	-8.365949281	0.031868954	-1.380760545
H265	3.933987675	9.756536379	-4.315863015
H266	-6.031775086	-0.804044043	-2.927055922
H267	0.732499523	6.186763496	-5.353942536
H268	-3.169863735	7.068827487	0.161812228
H269	3.646335448	10.081710750	-2.594024938
H270	-2.678289089	7.681226171	-6.784175540
H271	-4.853223388	8.952805833	-4.313394650
H272	-5.347028005	-3.472426329	-3.275852327
H273	-5.852202428	-3.239461295	-0.655189973
H274	-6.525780303	10.644749709	1.027781027
H275	-5.186490823	1.494515912	-1.472287808
H276	-0.239797296	7.900256086	-3.852916681
H277	-1.557152301	6.226234919	-6.117474216
H278	-0.551596400	4.087220603	-4.917712019
H279	-3.391037033	5.887310331	-3.740417094
H280	1.090790965	8.876153906	2.752726808
H281	-0.070949350	9.717775225	-0.156167621
H282	0.462162801	12.563202327	0.078716823
H283	2.590368273	9.266050424	1.872888068
H284	-2.083418341	10.691088333	1.366349869
H285	4.308864648	-3.383647522	-9.247126424
H286	4.301463361	-4.780045267	-8.153127699
H287	7.598926267	-5.645369541	-5.007868608
H288	4.631542893	-5.579797708	-10.363159294
H289	3.072538940	-5.942438055	-10.012241303
H290	5.527797775	4.491958271	-1.031173433
H291	6.062013079	7.167402407	0.338309028
H292	5.016858150	7.766411034	-3.762150414
H293	4.260511523	5.472187876	-0.227861174
H294	7.131132393	7.673730613	-0.790638473
H295	5.356010025	2.451058507	-6.929473692
H296	6.122931075	6.384312171	-2.490725935
H297	5.025352763	-6.963095314	5.049446771
H298	6.717862944	-8.063009558	2.741539903
H299	3.537435692	1.084866881	-6.908046985
H300	4.978062547	-0.054169516	3.107421014
H301	0.456237263	1.325396367	-8.714576080
H302	-1.263906532	-2.135242204	-4.407778335
H303	0.365544760	1.893431396	-7.042210553
H304	4.253978315	-4.430344805	4.594668890
H305	1.957967371	-0.152502580	-6.566560793
H306	3.816400495	-4.936609874	2.958212252
H307	4.488754822	-7.269906525	3.379498951
H308	6.238419855	-5.589083751	2.575019742
H309	6.585528489	-5.188221717	4.263961993
H310	-5.357037007	-0.790873825	-6.462827558
H311	5.504002997	-9.461641067	5.096217876
H312	4.545555840	-11.906262695	3.513501548
H313	0.080757638	-0.049312715	-4.248666099
H314	5.866871341	-11.758304631	6.113105158

	H315	4.307131213	-12.106696364	5.754427496
	H316	5.522885048	-3.320469657	2.112838201
	H317	-0.884940989	2.107789196	-4.940149552
	H318	-3.529238310	-2.071405464	-5.447834195
	H319	7.216393182	-10.299401365	-1.417763728
	H320	6.225702184	-8.019509686	-3.639336968
	H321	6.295882615	-8.429078366	0.078189206
	H322	6.320835469	-10.901589650	-2.538795050
	H323	4.796103535	2.241989358	-9.652848440
	H324	6.213025843	-5.289474254	-0.215495666
	H325	1.964366108	-7.890137141	0.982229050
	H326	3.099537722	-2.304924023	-7.607797951
	H327	2.687127584	-9.315121938	-0.615604952
	H328	2.674088117	-7.152393975	-6.845449204
	H329	1.979572406	-5.539535124	-5.025765976
	H330	6.150866185	-12.524779635	3.917378659
	H331	5.368238556	-1.080502936	1.756062366
	H332	8.160315618	-6.113444562	0.837270330
	H333	7.170912505	-4.071394225	-0.202453176
	H334	8.691406864	-7.119104614	2.897192308
	H335	3.941165463	-2.669510783	5.275360703
	H336	3.478370046	-8.514843863	-3.464716942
	H337	4.650671645	-8.862510756	-2.184975447
	H338	1.584505206	-8.775700782	-1.886594682
	H339	1.208084542	-2.305493451	-6.089495308
	H340	-3.159939907	2.189959898	-6.003081671
	H341	-5.248607206	0.978407116	-6.672117022
	H342	2.150719115	-6.137589655	0.872215629
	H343	8.215192201	-7.665849931	0.719237151
	H344	7.003739316	-9.529713811	0.903001403
	H345	3.778505674	-0.997604959	4.973277966
	H346	5.073361701	-8.008992091	-6.520330474
	H347	6.234196595	-6.378107996	-3.017966639
	H348	0.251417639	-3.126978312	-7.357190005
	H349	4.272662708	3.848908579	-7.102560522
	H350	7.626049284	-0.139027362	3.956293239
	H351	7.696820514	0.248572926	2.220816724
	H352	8.611489737	-7.302496746	-6.336882513
	H353	7.826189186	-8.525566911	-5.583973521
	H354	9.893081650	-9.101568372	-1.575261026
	H355	8.134670950	-8.688505038	-2.960794863
	H356	11.310301260	-8.162189251	-3.732734156
	H357	9.834042706	-7.378043099	-1.149082780
	H358	10.418275088	-0.189262258	2.243974277
	H359	-2.408260747	13.358774353	3.373195580
	H360	9.903646336	-7.137213922	5.649856948
	H361	9.428205799	-4.710956159	4.460487415
	H362	-4.194446383	12.483312580	1.086591285
	H363	-3.682821798	10.544993294	3.425782266
	H364	-5.231259253	11.317876145	3.037023775
	H365	-6.215658838	6.988070524	-1.669163619
	H366	2.859246032	-0.871609181	0.676049547
	H367	2.075404105	-3.853553026	0.291009058
	H368	1.216334218	-1.163983314	0.588969827
	Mo369	3.684511750	-4.523729201	-2.351653886
	N370	2.054968175	-0.956150752	0.046034883
	N371	0.661878303	1.471358819	-0.501135097
	N372	-0.075781886	2.125710845	0.043421233
	N373	-7.312601059	-6.913245252	-0.036642511
	N374	-8.903286639	-4.187926049	1.967564357
	N375	-8.299998054	-4.406440637	4.705467756
	N376	-5.655589271	-5.360797430	4.734429637
	N377	-4.295843111	-3.063860437	3.747439663
	N378	-4.775994344	-1.288613762	5.954693526
	N379	-3.740744567	-2.643747303	8.328453070
	N380	-1.371533964	-4.195105172	7.675463130
	N381	-0.006148927	-2.468788605	5.941841456
	N382	-0.025478729	0.543757579	6.505455785
	N383	-0.187983651	0.513288036	9.373775401
	N384	5.086737485	-11.495441663	5.506470149
	N385	5.946614890	-9.326362549	4.181465112
	N386	5.087798897	-3.272342689	3.036688941
	N387	4.252364027	-1.871223535	4.725541663
	N388	4.940975082	-0.983370033	2.693021299
	N389	8.749777109	-7.208000589	3.936310360
	N390	-5.985272472	-2.828847271	-2.773056325
	N391	-2.150667990	-5.607059427	-2.991909940
	N392	-6.103282962	-0.310576902	-2.023027957
	N393	-8.350451866	0.934187274	-0.910257643
	N394	-7.880531067	0.726447497	1.854487357

N395	-5.655610942	2.380715744	2.350046064
N396	-3.042678057	2.455087774	-0.959637498
N397	-2.632492951	0.359867837	-0.328417186
N398	-6.099327354	5.074863317	2.764269617
N399	-9.476157583	8.183561009	1.505317173
N400	-9.256509468	9.997270960	2.764807924
N401	9.511055228	2.235666863	2.398846968
N402	6.565757079	6.869539257	-0.503635164
N403	4.471612231	8.207750631	-3.026285325
N404	1.786306537	7.659279589	-3.450800480
N405	-2.959602581	6.385083653	-4.548952658
N406	-3.199018558	8.161970164	-6.060029639
N407	-4.185018046	8.268851559	-3.967598577
N408	0.771983003	6.197797294	-1.170057046
N409	1.205766710	8.115285125	0.811688917
N410	0.062923928	10.573593044	0.382017704
N411	-1.568545842	10.299294189	-3.219555034
N412	-2.272957549	11.689501355	1.419196266
N413	3.710599781	-5.164662436	-10.205168843
N414	2.254134900	-2.838914381	-7.827191851
N415	1.281650767	0.020326504	-7.329065472
N416	3.416033632	1.938839851	-7.462655316
N417	-1.919061884	-3.234887365	-10.000801865
N418	-3.401006647	-1.307936511	-8.452856564
N419	7.735031962	-7.544458771	-5.865937406
N420	3.971992910	-5.858678020	-4.249558037
N421	3.295046170	-6.847863616	-6.100401103
N422	8.846364072	-7.953111959	-2.947384312
O423	-6.735352156	-4.887048951	1.932056337
O424	-7.300863068	-2.358829662	4.648565105
O425	-5.471777436	-4.708303365	6.917145779
O426	-2.558399138	-3.693207986	5.076730710
O427	-2.848818351	-0.241300493	5.355888247
O428	-2.958011040	-0.572523091	8.901300960
O429	-0.621481256	-3.721307868	9.789844572
O430	1.948080319	-3.624590648	6.304831030
O431	2.196615359	0.132588088	6.147138126
O432	1.548322735	1.972312825	9.302955708
O433	2.178938317	-0.292538721	11.942752751
O434	6.634612853	-10.543561034	2.341710752
O435	7.687499249	-7.821990566	5.880293166
O436	11.449915099	-4.937379684	4.606853540
O437	-5.920701291	-0.808766245	0.217187884
O438	-3.926088525	-4.456877783	-3.827517686
O439	-7.077194607	2.757982827	-0.400101865
O440	-6.195177286	3.140138463	-3.316510892
O441	-9.790389459	1.929422347	2.280921225
O442	-5.707540724	1.867016127	4.576418955
O443	-4.091632333	5.260671168	3.826997505
O444	-6.146099464	7.723980302	5.107404456
O445	10.599958980	-0.559172109	4.243543227
O446	3.685232354	4.623925474	3.855119653
O447	3.937898095	8.213374095	-0.807957011
O448	1.290266375	9.888328451	-3.724660250
O449	-1.209492672	7.227683694	-1.664287996
O450	-0.522980417	7.219433061	2.027213149
O451	2.509558682	5.513415194	1.322628765
O452	1.284084776	11.536441504	2.063086703
O453	-1.849173389	13.686212089	0.408467329
O454	-2.881903903	11.216003658	-1.588706495
O455	-4.342396886	13.996219033	3.224163582
O456	-5.270155621	6.988144228	-1.424210863
O457	1.432124544	-4.802889705	-8.688607058
O458	-0.389098627	-0.966938098	-8.553103451
O459	2.058963457	3.356039027	-8.610681050
O460	5.744065940	4.009513556	-9.276147085
O461	-5.184869800	-2.673615571	-8.850452601
O462	-1.762614324	-6.732289478	-5.823701250
O463	0.143656235	-5.931624811	-6.663189323
O464	-3.966151392	2.228752934	-8.542676575
O465	9.649629692	-5.948342355	-3.635794053
O466	12.299976179	-7.597851751	-2.038125820
O467	0.081548289	-6.201306160	-1.117753070
O468	-0.518609946	-7.230068083	0.801386085
O469	4.680101623	-11.195515428	-3.057075596
O470	2.420120439	-11.095904934	-3.050216113
O471	4.848812563	-6.035506011	-1.262258907
O472	4.752367715	-7.643101948	0.304820343
O473	2.338784487	-6.219970284	-1.924094620
O474	8.698578995	-6.892898907	1.122861549

	O475	6.885237098	-4.826079971	0.346238103
	O476	-4.205117570	4.831397446	-2.487713324
	O477	7.278352952	-10.828076540	-2.244483717
	O478	7.143457282	-8.947742854	0.113142271
	S479	3.582589718	4.839290870	-2.468493105
	S480	1.854223235	1.719478787	-3.337057791
	S481	1.891294214	-3.444009929	-3.392648012
	S482	3.567376645	1.686363304	-0.190886313
	S483	0.391280756	-1.151488225	-2.012272726
	S484	3.899747454	-0.926083091	-5.575949862
	S485	3.383298398	-3.605427150	-0.120762669
	S486	5.448953081	1.567255652	-3.208345740
	S487	5.588828024	-3.325328529	-3.150121261
	S488	6.263530249	-0.982875016	-0.402464887
	end			

product

Fe(139) -2.332<br>Fe(140) 0.055<br>Fe(141) -2.621<br>Fe(142) 2.806<br>Fe(143) 3.151<br>Fe(144) 2.201<br>Fe(145) -1.496	bm5expro2b2n2x3b32te.car_3			
	C1	-7.914169203	-5.030340036	1.559142430
	C2	-8.355383285	-6.175850213	0.641618156
	C3	-8.612634933	-2.985594976	2.730235027
	C4	-8.021847324	-3.227536460	4.116388187
	C5	-7.703762343	-4.771500303	5.983821045
	C6	-6.180973675	-4.938033665	5.925668503
	C7	-4.221712889	-5.385561709	4.534917194
	C8	-3.612348365	-3.981884824	4.482223244
	C9	-3.888432428	-1.673962475	3.701231043
	C10	-3.793213262	-1.010769391	5.084015423
	C11	-4.765779473	-0.681272155	7.277141446
	C12	-3.738012898	-1.298130124	8.238108189
	C13	-2.976331167	-3.343216083	9.355415284
	C14	-1.549043562	-3.752341081	8.968473676
	C15	-0.086210863	-4.591039254	7.177886903
	C16	0.708836956	-3.505466959	6.447444930
	C17	0.633332427	-1.421997683	5.169145870
	C18	0.996915794	-0.182005124	5.996904714
	C19	-0.105313701	-1.088248759	3.852287643
	C20	0.539764549	0.107200588	3.141311353
	C21	-0.134026027	-2.316398038	2.935300678
	C22	0.210013023	1.754759115	7.289249072
	C23	0.567951354	1.422963411	8.749421546
	C24	-0.960884545	2.756416464	7.146705119
	C25	-0.736784557	3.990324148	8.029895146
	C26	-1.131878449	3.173670972	5.679264209
	C27	0.111323228	0.138650972	10.753524106
	C28	1.551104007	-0.316688119	10.894239758
	C29	5.452509613	-11.694805992	4.089987691
	C30	6.086269372	-10.460457953	3.451768849
	C31	6.452290283	-8.019884524	3.800890764
	C32	7.698286956	-7.683362464	4.641380771
	C33	5.353815147	-6.961251712	3.996715751
	C34	5.807505614	-5.560551196	3.587961474
	C35	4.656671148	-4.559499794	3.595268817
	C36	4.767047484	-2.049425577	3.505194159
	C37	10.002897943	-6.860952743	4.580719926
	C38	10.318897792	-5.383487010	4.535936268
	C39	-5.449865631	-2.569570717	-1.437305653
	C40	-5.846522742	-1.149269260	-0.986106151
	C41	-3.913673203	-2.700997369	-1.416627768
	C42	-3.372687782	-4.144551508	-1.470611280
	C43	-3.173750784	-4.736827397	-2.865822881
	C44	-6.124254225	1.136499207	-1.933157688
	C45	-7.225268697	1.685083946	-1.019725077
	C46	-6.229729978	1.695030043	-3.359375720
	C47	-9.415228677	1.295116017	0.002890128
	C48	-9.032534679	1.342899575	1.486961370
	C49	-7.373897836	0.820402958	3.209401753
	C50	-6.171585059	1.740582995	3.435125027
	C51	-4.502599073	3.267733960	2.490335261
	C52	-4.874007655	4.616658115	3.126296899
	C53	-3.865699635	3.584278394	1.126604481
	C54	-3.363574884	2.390654483	0.387255114
	C55	-3.098113193	1.086141573	0.750751493
	C56	-2.614852626	1.207150974	-1.339357323
	C57	-6.531113570	6.428534463	3.095517998
	C58	-6.377694653	6.655669314	4.597538367
	C59	-8.017270939	6.538280483	2.678512290
	C60	-8.599209562	7.915798147	2.556622828
	C61	-8.450678541	9.037937022	3.349875204
	C62	-9.841403327	9.455502156	1.675659077

C63	9.198755903	1.246515106	3.420482678
C64	10.159915779	0.079659122	3.293253240
C65	7.769338578	0.681725309	3.223095950
C66	6.696352451	1.727834353	3.412922582
C67	6.117307885	1.959143354	4.673832161
C68	6.245429780	2.503641563	2.329073619
C69	5.116250394	2.919723878	4.849831064
C70	5.260319432	3.476367163	2.492960489
C71	4.689668795	3.677148889	3.752313380
C72	5.579238649	6.566820178	-1.559038407
C73	4.590499766	7.732995220	-1.753420638
C74	4.811934540	5.307103854	-1.179615794
C75	3.587412436	9.309996945	-3.372820249
C76	2.103584598	8.966487348	-3.533407187
C77	0.422001171	7.130231481	-3.451993531
C78	-0.078651422	6.848521897	-2.012347036
C79	0.419483505	5.871538624	-4.346395252
C80	-0.860442954	5.035090044	-4.455839903
C81	-1.989406394	5.606257387	-5.327677562
C82	-3.453464426	7.578372946	-4.877015714
C83	0.361927890	5.897229457	0.198024435
C84	0.282683829	7.143060036	1.089400490
C85	1.301171830	4.888581197	0.888779294
C86	1.496196361	9.156289854	1.775417206
C87	0.953647399	10.531319730	1.423848834
C88	-0.351765304	11.862744230	-0.139570314
C89	-1.568925983	12.493846358	0.576779800
C90	-0.551512846	11.873702311	-1.659475195
C91	-1.773635155	11.092940003	-2.128385759
C92	-3.568406806	12.122224891	1.924524133
C93	-3.418610032	13.262858110	2.918917045
C94	-4.298740869	10.928098955	2.598001042
C95	-4.602959400	9.829015358	1.607633170
C96	-5.779897214	9.864221675	0.844727372
C97	-3.690501568	8.787559183	1.370744727
C98	-6.029674837	8.908484443	-0.141363986
C99	-3.911005037	7.837594608	0.370305698
C100	-5.080441964	7.914726961	-0.382079780
C101	3.772547436	-4.318443452	-9.007459544
C102	2.374748345	-4.001941891	-8.503359529
C103	1.024527774	-2.388433373	-7.176713085
C104	0.555918245	-1.045902284	-7.751816482
C105	1.005891683	1.373264335	-7.770788946
C106	2.214028754	2.278750103	-7.996546787
C107	4.530162129	2.850971545	-7.537096514
C108	5.032433556	3.066771111	-8.949022516
C109	-2.975136339	-3.662047431	-9.083113292
C110	-3.947447829	-2.510198922	-8.801727117
C111	-2.516372341	-4.304812687	-7.753853919
C112	-1.873614935	-5.684039783	-7.994137687
C113	-1.182421311	-6.178980927	-6.743606121
C114	-4.233405455	-0.157396636	-8.212438549
C115	-3.645996632	1.079858174	-8.861469860
C116	-4.683212444	0.037554544	-6.742924390
C117	-3.477291217	0.057024715	-5.851828859
C118	-2.748750401	1.243465662	-5.662560858
C119	-2.958161653	-1.153055961	-5.364802634
C120	-1.477483946	1.195033316	-5.086376934
C121	-1.688731971	-1.196354750	-4.786705192
C122	-0.930213368	-0.026690723	-4.692485824
C123	7.584323803	-6.697846671	-4.687767459
C124	8.768551513	-6.824523756	-3.714016252
C125	6.256882429	-6.948416761	-3.955737728
C126	5.004039661	-6.673241486	-4.723870559
C127	4.580403455	-7.290243776	-5.882599145
C128	2.955336625	-5.999970814	-5.113859916
C129	9.932096107	-8.076530270	-1.995292319
C130	11.287493291	-7.910003672	-2.645591046
C131	0.379362417	-6.827755472	0.006459061
C132	1.867918315	-6.995548943	0.297115643
C133	2.767342296	-7.186620395	-0.963986412
C134	2.616480547	-8.643339134	-1.486597221
C135	3.644357793	-9.086439510	-2.530966202
C136	3.570232307	-10.585120672	-2.904058151
C137	4.237225938	-6.967566332	-0.569763980
C138	3.523949260	-0.933858112	-2.274378436
Fe139	3.699686529	-2.231639679	-3.759148179
Fe140	1.883766330	0.464440407	-1.333792940
Fe141	4.826271947	0.319561280	-1.438028156
Fe142	3.618411268	0.357619414	-3.755284642

Fe143	3.692290394	2.560319802	-2.259919564
Fe144	4.724130754	-2.252864470	-1.399144499
Fe145	2.159488458	-2.113789250	-1.623638682
H146	-6.610695176	-7.231089119	0.660934609
H147	-6.781802371	-6.285153588	-0.656810641
H148	-9.831514773	-4.282765132	1.553273883
H149	-9.058487804	-5.780172112	-0.108413120
H150	-8.939119372	-6.875499722	1.264601784
H151	-7.890024021	-2.355750189	2.190015952
H152	-8.894591125	-5.078308802	4.217209347
H153	-9.542478945	-2.412732577	2.846090926
H154	-8.150674744	-5.715078841	6.323870662
H155	-6.263250229	-5.413252013	3.918705053
H156	-7.907153351	-4.002510953	6.740505645
H157	-2.904193945	-1.575495618	3.227406841
H158	-5.125613260	-3.361354674	3.238547005
H159	-4.021023622	-5.906889168	3.589330315
H160	-4.526234101	0.386011703	7.189901387
H161	-5.616653227	-1.799796411	5.639498332
H162	-3.726390967	-5.932541130	5.345263618
H163	-4.617039607	-1.136769306	3.078560887
H164	-4.430305446	-3.195576301	7.789095716
H165	-5.766159216	-0.786171556	7.719693635
H166	-0.604838713	-3.184138702	3.418453383
H167	-0.696286826	-2.096442067	2.014364852
H168	0.889581444	-2.605353294	2.642714375
H169	-1.135234317	-0.802599037	4.113485612
H170	-3.517316378	-4.255059685	9.646789855
H171	-1.343352704	2.317369672	5.024066339
H172	-1.043155218	0.109655183	8.955505430
H173	-0.673153291	3.735586069	9.097508515
H174	-0.005398187	0.327109391	2.213144311
H175	1.590364100	-0.093153702	2.875956878
H176	9.274062954	1.624547415	4.457904991
H177	-1.962961489	3.886779901	5.574325602
H178	-1.567197204	4.699185415	7.895414312
H179	-0.214818728	3.665053298	5.313728477
H180	0.196858650	4.502645843	7.746977105
H181	-1.886233971	2.252546091	7.480755910
H182	1.121848490	2.220125954	6.886275279
H183	-0.993087534	0.262515354	6.299660407
H184	0.519546397	1.018832692	3.755672331
H185	1.522422779	4.069555649	0.188452808
H186	0.750122800	4.475255789	1.746031360
H187	2.754356574	5.156570217	2.194306591
H188	3.346516158	4.647703975	4.761128098
H189	4.899808477	4.052500716	1.640229884
H190	6.645180054	2.319225993	1.329093212
H191	6.442038741	1.367248993	5.534347990
H192	-1.030052829	-2.529489691	5.942801844
H193	-0.218570843	-5.430541963	6.478525122
H194	0.533483014	-4.937749455	8.013811351
H195	-2.153263488	-4.132778425	7.020621775
H196	-2.885427560	-2.697793190	10.237849591
H197	4.660864118	3.069336131	5.832804009
H198	1.611014670	-1.839377881	4.894209998
H199	-0.077488449	0.955474689	11.468587687
H200	-0.527207325	-0.715203036	11.030104432
H201	2.006360503	-0.733650813	9.965047713
H202	10.847719519	-7.429240932	4.161983524
H203	-1.981347775	-6.031684365	-3.910538811
H204	1.043350172	-6.059995164	-1.638008824
H205	-2.658474284	-6.406204874	-8.260604427
H206	-1.155899669	-5.628660145	-8.825840680
H207	-3.371470135	-4.425712081	-7.070343306
H208	-2.935723002	0.873204537	-9.696369231
H209	-3.595539072	-4.401699856	-9.612128640
H210	-3.500424004	-2.110421206	-2.248814403
H211	-4.051159273	-4.826323398	-0.927504639
H212	-2.375294139	-1.213403119	-8.395785883
H213	-5.155983861	-0.344698364	-8.801784391
H214	-1.223058080	-2.646955485	-9.539035945
H215	-1.481999188	-5.784833952	-2.239588251
H216	-1.809380705	-3.624638072	-7.254137416
H217	-1.407689634	-4.067442583	-10.346148125
H218	-6.911985996	-3.266833836	-2.725640973
H219	-3.543194861	-2.222344998	-0.500222429
H220	0.503557476	-5.483473268	-7.475635017
H221	-2.414854217	-4.185510947	-0.930684192
H222	-3.770190449	2.796175743	3.160503529

	H223	-3.231394380	0.631774508	1.728938558
	H224	-8.190184626	1.200753365	3.839266154
	H225	-7.299375042	0.237777076	1.150952341
	H226	-6.713241298	4.482602184	2.224161366
	H227	-7.848174559	9.233525586	4.229010286
	H228	-6.577114707	5.745794263	5.214308211
	H229	-8.621848008	5.917652507	3.364145892
	H230	-5.922663677	7.177656592	2.563971447
	H231	-10.528079610	10.009681628	1.041158319
	H232	-3.044399817	4.295931311	1.316646928
	H233	-4.607521255	4.115227898	0.506262889
	H234	-9.358101944	10.959272435	3.104252124
	H235	-8.115492397	6.073968965	1.684108238
	H236	-6.068430445	2.248640251	1.424518164
	H237	-7.109724743	-0.173971936	3.599626518
	H238	-9.809553825	2.292175319	-0.237855082
	H239	-2.278557138	0.983723013	-2.349170101
	H240	-10.235167638	0.574917086	-0.120875673
	H241	10.351775379	2.762637658	2.668691507
	H242	8.761215891	2.926422699	2.335548084
	H243	1.649874566	5.771500083	-1.532936619
	H244	-0.647963708	5.462633739	0.178731125
	H245	1.947556260	7.883998035	0.150903808
	H246	-3.178944737	3.281158447	-1.549322498
	H247	-4.939065945	4.210534375	-2.761681309
	H248	-6.443588064	3.443122150	-4.216428528
	H249	-4.605890151	5.471875489	-1.865049821
	H250	-5.381084062	1.304217551	-3.945613964
	H251	-7.171531908	1.355331000	-3.820370050
	H252	0.368689984	11.531280574	-2.153096227
	H253	-0.714047735	12.924083932	-1.948345176
	H254	-0.610358867	10.061205244	-3.493445797
	H255	-2.325551460	9.651364968	-3.428657977
	H256	-3.382981099	9.154578256	-6.192203770
	H257	-2.530126826	4.785966933	-5.824103582
	H258	-6.956261163	8.940415843	-0.720204864
	H259	-2.786314766	8.692382354	1.977577439
	H260	-4.430549805	7.814696993	-3.083307711
	H261	1.211505865	5.200258183	-3.979092356
	H262	2.542755849	6.985062460	-3.292742604
	H263	-1.253751824	4.769478520	-3.462367322
	H264	-8.367462910	0.031836221	-1.383166511
	H265	3.929913977	9.760916489	-4.313938171
	H266	-6.030473515	-0.807714417	-2.934953056
	H267	0.731373375	6.187922089	-5.356037916
	H268	-3.165444473	7.069164659	0.164752077
	H269	3.641218683	10.081792461	-2.591753935
	H270	-2.681758777	7.684709258	-6.787059083
	H271	-4.857394383	8.951120526	-4.314496509
	H272	-5.346741073	-3.477971568	-3.279724282
	H273	-5.861376716	-3.244178293	-0.662049671
	H274	-6.518762744	10.649480140	1.022672371
	H275	-5.184671538	1.488839976	-1.483583690
	H276	-0.243354174	7.901002298	-3.853522120
	H277	-1.556806382	6.231530562	-6.120106354
	H278	-0.553875216	4.087348765	-4.918927376
	H279	-3.395491719	5.884404204	-3.748034339
	H280	1.075044431	8.872610939	2.751610521
	H281	-0.076599465	9.719238837	-0.148630413
	H282	0.467365398	12.565079787	0.081581414
	H283	2.584843619	9.253704254	1.885789993
	H284	-2.075694854	10.698462893	1.375493715
	H285	4.308644221	-3.385197665	-9.234238431
	H286	4.296827716	-4.788234358	-8.148652688
	H287	7.611671014	-5.654123937	-5.034078982
	H288	4.629868483	-5.575795345	-10.363101915
	H289	3.069331507	-5.936992280	-10.016499608
	H290	5.535358886	4.493177532	-1.039641572
	H291	6.063081990	7.160732297	0.331208188
	H292	5.007317018	7.765547688	-3.766437386
	H293	4.260913553	5.465834037	-0.242340214
	H294	7.131902595	7.672313570	-0.796130124
	H295	5.359516364	2.422389089	-6.952312876
	H296	6.122266672	6.387198960	-2.499821270
	H297	5.033703477	-6.969162445	5.052143133
	H298	6.721032661	-8.064979629	2.738143099
	H299	3.531369499	1.066600912	-6.919710506
	H300	4.957864716	-0.050681195	3.117064455
	H301	0.446919924	1.322351802	-8.713059227
	H302	-1.281451843	-2.136596461	-4.408273155

H303	0.362337251	1.894103338	-7.041085746
H304	4.269906410	-4.431393778	4.613223065
H305	1.939324970	-0.156047046	-6.557417088
H306	3.822602219	-4.925696472	2.972961769
H307	4.493801037	-7.271476926	3.381812129
H308	6.236260571	-5.590095943	2.575447079
H309	6.597763748	-5.188791406	4.258767975
H310	-5.361402741	-0.789457712	-6.486562265
H311	5.506082307	-9.467687216	5.089894702
H312	4.546987448	-11.909399428	3.502074445
H313	0.068441864	-0.049381411	-4.260302660
H314	5.856938222	-11.769631008	6.108476182
H315	4.297639371	-12.109613947	5.741424724
H316	5.530731917	-3.313632299	2.132083403
H317	-0.892945630	2.103000608	-4.934941229
H318	-3.543802406	-2.070356078	-5.457453994
H319	7.219074992	-10.293649972	-1.425780638
H320	6.226049680	-8.003682636	-3.636287614
H321	6.301843502	-8.429073973	0.084206271
H322	6.323216044	-10.900304506	-2.545221652
H323	4.775930575	2.251012368	-9.667440080
H324	6.234620019	-5.296410090	-0.225921904
H325	2.001861106	-7.827727585	0.999481113
H326	3.090052454	-2.300640644	-7.615281769
H327	2.695346234	-9.305530245	-0.609525062
H328	2.671163299	-7.161932242	-6.847538574
H329	1.980867010	-5.535061118	-5.035395624
H330	6.147521378	-12.532724424	3.911638994
H331	5.360957386	-1.075223933	1.764503605
H332	8.164642209	-6.106546369	0.836808747
H333	7.175894408	-4.062630056	-0.218554837
H334	8.694578601	-7.117214491	2.894496903
H335	3.932362698	-2.665694999	5.286864990
H336	3.491489782	-8.513591629	-3.458610437
H337	4.664028234	-8.872133393	-2.181575025
H338	1.597774465	-8.766071851	-1.884868118
H339	1.200172547	-2.308121582	-6.092823613
H340	-3.166825378	2.191536401	-6.009897441
H341	-5.249639321	0.979907948	-6.697258564
H342	2.176768114	-6.071773046	0.814996569
H343	8.217289874	-7.659921662	0.717632958
H344	7.022323888	-9.531550832	0.893425261
H345	3.765497547	-0.995131961	4.978705747
H346	5.073524413	-8.012969315	-6.521653174
H347	6.243323411	-6.356010880	-3.034210789
H348	0.244832687	-3.130334916	-7.361426105
H349	4.277108932	3.824440503	-7.089303144
H350	7.630179970	-0.136344872	3.945764459
H351	7.701859460	0.251915549	2.210613844
H352	8.612949762	-7.335964923	-6.343306088
H353	7.813905661	-8.543061632	-5.579652230
H354	9.891620254	-9.093048975	-1.573685353
H355	8.139196447	-8.688703111	-2.969167491
H356	11.315256594	-8.164136110	-3.732775788
H357	9.832002327	-7.367842747	-1.157560060
H358	10.422793944	-0.187939817	2.240815942
H359	-2.408398658	13.363033570	3.381009809
H360	9.907812248	-7.135599479	5.646738817
H361	9.431649364	-4.709042072	4.452353997
H362	-4.188879342	12.489786608	1.089280989
H363	-3.679965315	10.547028609	3.425659669
H364	-5.228547555	11.319105620	3.036113535
H365	-6.212240072	6.986314033	-1.664757901
H366	2.884451294	-0.906837334	0.614205168
H367	2.317380371	-3.254465568	0.268860701
H368	1.226982241	-1.121057335	0.580747230
Mo369	3.688940717	-4.561531565	-2.346735065
N370	2.058217678	-0.963641030	0.011083441
N371	0.679446537	1.489860626	-0.511630290
N372	-0.063420932	2.138479506	0.034580238
N373	-7.294726675	-6.907642562	-0.027802797
N374	-8.909229533	-4.192932820	1.972880173
N375	-8.308374349	-4.409905211	4.711406618
N376	-5.663093653	-5.362120497	4.745468340
N377	-4.296312849	-3.069922357	3.752769743
N378	-4.785178746	-1.297672592	5.959501171
N379	-3.758578762	-2.653811116	8.339790519
N380	-1.381767994	-4.184828181	7.689866864
N381	-0.012958695	-2.465957630	5.948906409
N382	-0.031231640	0.537115655	6.515007442

N383	-0.207265744	0.509679107	9.386884925
N384	5.081349779	-11.501803928	5.498113418
N385	5.950907634	-9.332200022	4.176265825
N386	5.103746251	-3.268388911	3.059813469
N387	4.245874153	-1.867359616	4.738428354
N388	4.937026330	-0.981743304	2.706461686
N389	8.753538923	-7.208417714	3.933573192
N390	-5.986950391	-2.834063037	-2.780166415
N391	-2.147329641	-5.599565953	-2.996606184
N392	-6.106305754	-0.315032360	-2.030954629
N393	-8.348617226	0.934583744	-0.913658230
N394	-7.876274626	0.724588464	1.851057142
N395	-5.657957910	2.385680064	2.350967238
N396	-3.048061206	2.451898607	-0.958421684
N397	-2.638702974	0.358032720	-0.322716693
N398	-6.100620968	5.078495308	2.776047729
N399	-9.472707717	8.189941122	1.515887201
N400	-9.244557101	10.005799520	2.770878312
N401	9.517868516	2.238992798	2.391403460
N402	6.568208043	6.866180846	-0.511128224
N403	4.467150406	8.208737640	-3.027986342
N404	1.783815966	7.660416458	-3.452351189
N405	-2.963933363	6.384038575	-4.555068077
N406	-3.201477202	8.164907688	-6.061777025
N407	-4.188317586	8.267538451	-3.969556091
N408	0.774841206	6.196648691	-1.173996267
N409	1.200841401	8.113466390	0.811322236
N410	0.064370005	10.576181894	0.386101850
N411	-1.570251768	10.301921273	-3.215205758
N412	-2.267647666	11.696690403	1.424881956
N413	3.709546732	-5.159709632	-10.204284315
N414	2.247038669	-2.840122323	-7.830203125
N415	1.270133131	0.017392384	-7.326541413
N416	3.413408231	1.921961352	-7.472330141
N417	-1.913298836	-3.244890297	-10.009506347
N418	-3.393137213	-1.312776514	-8.462302705
N419	7.733250926	-7.563856500	-5.871251480
N420	3.971811345	-5.852652650	-4.262815179
N421	3.294328686	-6.852108283	-6.106526281
N422	8.849993650	-7.952404962	-2.956084238
O423	-6.741326681	-4.891414058	1.952246474
O424	-7.310440278	-2.361793412	4.654932440
O425	-5.485025661	-4.715378161	6.930418534
O426	-2.562285778	-3.698459152	5.086658317
O427	-2.852113860	-0.254024308	5.372243638
O428	-2.977363021	-0.581623420	8.910685151
O429	-0.644645201	-3.737142027	9.815342136
O430	1.938794347	-3.624005289	6.317525565
O431	2.191657762	0.141702051	6.140529259
O432	1.537498503	1.957530960	9.314831044
O433	2.162561767	-0.296681197	11.952046735
O434	6.643186582	-10.551169980	2.338611080
O435	7.694981248	-7.831936091	5.876719476
O436	11.452564738	-4.935867234	4.609656172
O437	-5.927314111	-0.812486318	0.209823193
O438	-3.932033377	-4.463164330	-3.824504183
O439	-7.070673714	2.755989332	-0.407398210
O440	-6.198986328	3.135435847	-3.325659926
O441	-9.785801373	1.926893035	2.280618702
O442	-5.710208208	1.869782946	4.576791661
O443	-4.085150270	5.265035290	3.824165844
O444	-6.129927412	7.735099785	5.110248412
O445	10.609444724	-0.548225968	4.241515743
O446	3.689282307	4.625461418	3.848982068
O447	3.946187657	8.216317010	-0.806456912
O448	1.285602814	9.888621363	-3.728561342
O449	-1.209557858	7.222846992	-1.664143807
O450	-0.522623218	7.206919327	2.027140112
O451	2.513579951	5.516310381	1.317487499
O452	1.299183182	11.534239605	2.060336003
O453	-1.846081186	13.690231851	0.406242695
O454	-2.878168241	11.218426804	-1.579727102
O455	-4.343559423	13.997838366	3.230519462
O456	-5.266205968	6.987222508	-1.421866223
O457	1.431964582	-4.811720308	-8.680198903
O458	-0.388115976	-0.971338691	-8.566621720
O459	2.058473717	3.348576748	-8.610181271
O460	5.705040694	4.027956589	-9.284786596
O461	-5.177679249	-2.676859885	-8.861767398
O462	-1.770462868	-6.726388046	-5.817971727

	O463 0.143839045 -5.943071381 -6.655452518<br>O464 -3.951129319 2.225134737 -8.561784674<br>O465 9.654369386 -5.950267135 -3.650186556<br>O466 12.301086165 -7.595171596 -2.037754996<br>O467 0.090164182 -6.180629623 -1.128822074<br>O468 -0.497817347 -7.203722446 0.782156040<br>O469 4.682818662 -11.199592731 -3.066372906<br>O470 2.423386200 -11.093736170 -3.056268230<br>O471 4.883620771 -6.041502785 -1.232047025<br>O472 4.760859857 -7.669878381 0.316653511<br>O473 2.387912569 -6.215559609 -1.938778174<br>O474 8.699727554 -6.887691173 1.124340671<br>O475 6.900680102 -4.818806541 0.332874670<br>O476 -4.212323573 4.830132196 -2.495734961<br>O477 7.280686679 -10.824398516 -2.251244924<br>O478 7.153950942 -8.942668439 0.107668257<br>S479 3.597468037 4.832117073 -2.489329928<br>S480 1.836613198 1.739079502 -3.373900417<br>S481 1.851081645 -3.495013219 -3.378695032<br>S482 3.588923249 1.689692370 -0.198577503<br>S483 0.320830337 -1.095192600 -2.022928101<br>S484 3.871570368 -0.950275044 -5.562912525<br>S485 3.580171356 -3.749049758 -0.012034178<br>S486 5.516840269 1.520282704 -3.174918592<br>S487 5.565519002 -3.360694920 -3.143345525<br>S488 6.221326773 -1.030053825 -0.350147397<br>end

Fe2-brNH2-Fe6-3b2 to Fe2-brNH2-Fe6H

35, S=1

reactant

Fe(139) -2.426<br>Fe(140) -0.018<br>Fe(141) -2.534<br>Fe(142) 2.637<br>Fe(143) 2.576<br>Fe(144) 1.872<br>Fe(145) -0.876	9651826nh22n2x3b26hx135tc.car_3<br>C1 -9.403305187 0.828014853 1.065317340<br>C2 -10.358270638 -0.063363042 0.262862238<br>C3 -8.875840320 3.122900889 1.763137090<br>C4 -8.595055499 2.922936408 3.249114127<br>C5 -9.304129367 1.941529227 5.369714048<br>C6 -8.173430732 0.931038330 5.596838632<br>C7 -6.789753773 -0.840873637 4.639562280<br>C8 -5.473026642 -0.080795248 4.459539068<br>C9 -4.297346721 1.720510417 3.302709907<br>C10 -3.910340706 2.529190908 4.549378907<br>C11 -4.626824234 3.871679006 6.435783429<br>C12 -4.216429270 3.059947009 7.672930830<br>C13 -4.821169905 1.257159715 9.222542077<br>C14 -3.929466566 0.011145296 9.143748975<br>C15 -3.172907114 -1.865765551 7.748057373<br>C16 -1.822231075 -1.614832860 7.069699340<br>C17 -0.527959300 -0.209550697 5.531759650<br>C18 0.388887140 0.799436969 6.248369862<br>C19 -0.801325420 0.139013462 4.049947758<br>C20 0.490385613 0.542390797 3.327365243<br>C21 -1.476414301 -1.038976144 3.336000355<br>C22 0.776985467 3.091357387 7.014001690<br>C23 0.802467646 3.024765649 8.553116230<br>C24 0.441041482 4.499550026 6.467237734<br>C25 1.334081183 5.559916948 7.123717223<br>C26 0.601207807 4.536433223 4.941843356<br>C27 -0.406331372 2.892318613 10.649835093<br>C28 0.467139852 1.772958518 11.183838598<br>C29 -2.612758558 -11.370401013 6.799064358<br>C30 -1.332777018 -10.937256073 6.086418709<br>C31 0.454035138 -9.213513303 6.091254606<br>C32 1.629488082 -9.524075559 7.034667653<br>C33 0.281782947 -7.690811907 5.939727840<br>C34 1.542511606 -7.031103310 5.382479399<br>C35 1.312702751 -5.585047051 4.967212636<br>C36 2.995948058 -3.831590591 4.393644479<br>C37 4.011307776 -10.084932876 7.137617406<br>C38 5.056223317 -9.013997974 6.919181761<br>C39 -5.871808843 0.691905005 -1.860730201<br>C40 -5.377841539 2.145810005 -1.737125456<br>C41 -4.685597157 -0.264000384 -1.608504462<br>C42 -5.073429748 -1.727187523 -1.314079216<br>C43 -5.218285138 -2.636166525 -2.534361838<br>C44 -4.244744524 3.892115506 -3.091172577<br>C45 -4.836330912 5.160812291 -2.468831862

	C46	-3.954342197	4.064179201	-4.589113993
	C47	-6.863049211	6.326907324	-1.748207669
	C48	-6.559978168	6.491588321	-0.254181969
	C49	-5.600919225	5.520212776	1.777574004
	C50	-4.097377746	5.633713372	2.042774563
	C51	-1.814958783	5.706051872	1.159237273
	C52	-1.359992543	7.135338088	1.491839111
	C53	-1.043739313	5.283916393	-0.102207923
	C54	-1.310352080	3.883434698	-0.538543736
	C55	-1.853730912	2.784417767	0.094802309
	C56	-1.376304324	2.123902434	-1.892870774
	C57	-1.639997096	9.493789414	0.868649096
	C58	-1.433274789	9.938771935	2.313950789
	C59	-2.768190870	10.318654588	0.203351528
	C60	-2.433187297	11.717773369	-0.221419530
	C61	-1.745390354	12.723184711	0.429472059
	C62	-2.478329766	13.438704970	-1.535425114
	C63	8.224242383	-3.404684479	4.440781436
	C64	8.346760667	-4.904722075	4.642132651
	C65	6.727703995	-3.106502901	4.190804887
	C66	6.428390368	-1.632256800	4.044754971
	C67	6.073516511	-0.846218392	5.156463108
	C68	6.498019238	-1.001344016	2.788601214
	C69	5.789310051	0.517099236	5.023729661
	C70	6.231548077	0.360274591	2.643247778
	C71	5.871284688	1.117533679	3.760747255
	C72	8.403519236	1.734903444	-1.750188310
	C73	8.269305984	3.165251420	-2.296901202
	C74	7.038461117	1.215615637	-1.298131326
	C75	8.459149205	4.615577668	-4.286103655
	C76	7.059310922	5.139605151	-4.613186374
	C77	4.623701603	4.634893439	-4.499942268
	C78	4.024216240	5.012355894	-3.120304013
	C79	3.906717049	3.433872090	-5.156327152
	C80	2.383248868	3.471122350	-5.317602114
	C81	1.827858141	4.362683120	-6.439398651
	C82	1.778767858	6.855392084	-6.557593052
	C83	3.765598013	4.490772522	-0.742544436
	C84	4.370294732	5.752256796	-0.114106149
	C85	3.965202093	3.333787341	0.259067436
	C86	6.486970309	6.849031441	0.427052991
	C87	6.875694976	8.155994427	-0.240878388
	C88	6.652293227	9.589509512	-2.190327314
	C89	6.019879345	10.943693312	-1.782644716
	C90	6.539233736	9.379260069	-3.704986197
	C91	5.106697116	9.342143955	-4.222832716
	C92	4.128414504	12.077684420	-0.721539392
	C93	4.884643117	13.123817990	0.081641405
	C94	2.827727267	11.666606140	0.024433109
	C95	1.969427755	10.745643290	-0.813210145
	C96	1.054003337	11.272166998	-1.736544505
	C97	2.103971264	9.348952157	-0.739516358
	C98	0.310023353	10.440759644	-2.575827767
	C99	1.389271166	8.501678102	-1.591469013
	C100	0.501702363	9.058893686	-2.507702224
	C101	0.821673316	-7.626235150	-7.436055719
	C102	-0.163857599	-6.489080060	-7.221015962
	C103	-0.378251096	-4.137044193	-6.439447985
	C104	0.060443880	-2.940981625	-7.292598160
	C105	1.844565195	-1.293540073	-7.658541080
	C106	3.361927675	-1.293286513	-7.813457788
	C107	5.557008456	-2.070139061	-7.128814187
	C108	6.122726097	-2.467817904	-8.477834499
	C109	-4.251179003	-3.330151257	-8.708848884
	C110	-4.365270050	-1.803922594	-8.806870748
	C111	-4.304225726	-3.781575280	-7.231625933
	C112	-4.589313826	-5.292460387	-7.130079805
	C113	-4.370828345	-5.792251024	-5.720343391
	C114	-3.227821079	0.355176648	-8.681001549
	C115	-2.006218760	0.839662672	-9.436348892
	C116	-3.505817646	1.105222239	-7.354994074
	C117	-2.556178543	0.636000617	-6.290854102
	C118	-1.273495869	1.195221985	-6.179766281
	C119	-2.879114289	-0.491075815	-5.516604965
	C120	-0.306694269	0.582515669	-5.377930711
	C121	-1.913088409	-1.098265708	-4.713053827
	C122	-0.612944713	-0.584121567	-4.675529586
	C123	2.336886086	-10.727162319	-2.234847085
	C124	3.200829800	-11.239649760	-1.071687876
	C125	1.064438159	-10.045281972	-1.708043122

	C126	0.249443074	-9.297055130	-2.712899486
	C127	-0.398491149	-9.788988029	-3.826965255
	C128	-0.952478022	-7.662596268	-3.541627324
	C129	3.385943711	-12.455314965	1.011525146
	C130	4.589889241	-13.257892023	0.570369783
	C131	-3.671705156	-5.664641936	1.167427741
	C132	-2.575526735	-6.542692245	1.768452019
	C133	-1.931041172	-7.554309316	0.771956123
	C134	-2.879854151	-8.761584231	0.555643940
	C135	-2.277776182	-9.910006895	-0.255674271
	C136	-3.193368960	-11.146926275	-0.392159779
	C137	-0.611607884	-8.064359131	1.371966251
	C138	2.391635324	-3.244342146	-1.504290959
	Fe139	1.677275126	-4.694602838	-2.671901712
	Fe140	1.963232800	-0.975284462	-1.142113340
	Fe141	4.222933290	-2.932981701	-0.803338620
	Fe142	3.238423432	-2.705604250	-3.193779516
	Fe143	4.676913931	-0.730831168	-2.108762293
	Fe144	2.519344303	-4.800993325	-0.280464019
	Fe145	0.644405558	-3.084240118	-0.734554735
	H146	-9.572840184	-1.866719213	0.805349330
	H147	-9.048258932	-1.341882590	-0.646930470
	H148	-10.553801362	2.471828984	0.616864476
	H149	-10.632008564	0.456637311	-0.669116410
	H150	-11.289737542	-0.141898695	0.849060735
	H151	-7.898450303	3.116207103	1.257804909
	H152	-10.375949273	1.940867701	3.503596145
	H153	-9.321037492	4.117642598	1.624986602
	H154	-10.232218365	1.533379971	5.791952775
	H155	-8.429781441	0.149788461	3.705014384
	H156	-9.037848094	2.842037935	5.938037968
	H157	-3.424456345	1.116254296	3.026912688
	H158	-6.275844289	0.989140091	2.895513530
	H159	-6.894837760	-1.589292944	3.842602569
	H160	-3.799708336	4.559724255	6.217718852
	H161	-5.877843058	3.048860582	4.884801028
	H162	-6.740275014	-1.362565213	5.602968916
	H163	-4.511910006	2.403537918	2.469320762
	H164	-5.834395694	1.796304526	7.413656575
	H165	-5.517643466	4.463320840	6.687636581
	H166	-2.411590139	-1.339954773	3.829557809
	H167	-1.716637047	-0.771640750	2.294577255
	H168	-0.807709792	-1.915622523	3.311890962
	H169	-1.477562777	1.007975476	4.032511570
	H170	-5.805399529	0.937052981	9.594955271
	H171	-0.066628021	3.826419716	4.435395114
	H172	-1.281256167	2.969798167	8.696548746
	H173	1.180363240	5.620908908	8.210783622
	H174	0.270588485	0.806486402	2.281813518
	H175	1.222366764	-0.280775451	3.325021503
	H176	8.519337616	-2.918578497	5.390531553
	H177	0.378314289	5.541652736	4.555966726
	H178	1.125343558	6.549311650	6.690349200
	H179	1.638905858	4.290129212	4.660693142
	H180	2.397495079	5.329309920	6.948181548
	H181	-0.611509255	4.723041075	6.720350537
	H182	1.808217109	2.842424769	6.722358160
	H183	-1.001458980	2.321274333	6.057653209
	H184	0.969875101	1.417618835	3.790139536
	H185	3.744572164	2.382499527	-0.244843469
	H186	3.231688933	3.479217388	1.065901690
	H187	5.251318042	3.070758557	1.728695780
	H188	5.280114942	2.857356496	4.387473659
	H189	6.261179226	0.831162024	1.660623319
	H190	6.733264944	-1.591823071	1.900361403
	H191	6.005644090	-1.307716893	6.145528208
	H192	-2.597298765	-0.001345848	6.053432230
	H193	-3.732472724	-2.576885109	7.122087619
	H194	-2.963396548	-2.349366152	8.709470356
	H195	-4.530007371	-0.335965123	7.189413060
	H196	-4.366003490	1.917651390	9.970634395
	H197	5.492054070	1.107276321	5.894734123
	H198	0.062483346	-1.137145324	5.558296419
	H199	-0.089557246	3.841120538	11.112044124
	H200	-1.439582714	2.684647007	10.967560776
	H201	0.610074020	0.915069735	10.486340417
	H202	4.446004732	-11.069397827	6.904001106
	H203	-5.003117636	-4.580732059	-3.130140049
	H204	-2.624551516	-5.955952733	-0.443462056
	H205	-5.638636897	-5.478192084	-7.401537351

H206	-3.948119582	-5.846617356	-7.831106879
H207	-5.093125354	-3.232821940	-6.694161852
H208	-1.536095610	0.074885255	-10.098097853
H209	-5.173554529	-3.687648034	-9.191914324
H210	-4.010996936	-0.222138573	-2.478063027
H211	-6.034961151	-1.765227452	-0.772007795
H212	-2.349897220	-1.575794339	-8.358053380
H213	-4.068507600	0.592696862	-9.365498735
H214	-2.225860908	-3.632677570	-9.052087308
H215	-4.496202535	-4.286902100	-1.471155863
H216	-3.353755250	-3.522231425	-6.740627194
H217	-3.193683514	-4.839722902	-9.615697887
H218	-7.459481877	0.641013708	-3.186643582
H219	-4.101173436	0.123390247	-0.762939561
H220	-2.571856953	-6.358484261	-6.268936486
H221	-4.329907144	-2.168022386	-0.633855884
H222	-1.527429486	5.080259397	2.015780854
H223	-2.226760773	2.723029031	1.113032794
H224	-6.064799258	6.420630732	2.203467610
H225	-5.811093986	4.553057576	-0.119933708
H226	-2.883892835	7.841788202	0.315728969
H227	-1.227893206	12.756150822	1.381856814
H228	-2.161846489	9.485733117	3.028943050
H229	-3.647508584	10.311618473	0.871556136
H230	-0.693186691	9.624766857	0.321012497
H231	-2.656090881	14.115687592	-2.367552182
H232	0.026932218	5.428462575	0.119545999
H233	-1.293076341	5.979963741	-0.921019359
H234	-1.355506639	14.713465448	-0.249996644
H235	-3.071684236	9.783079292	-0.709688108
H236	-3.632473743	5.514170106	0.021945561
H237	-5.977837515	4.665236799	2.359027583
H238	-6.594943546	7.282862292	-2.219568237
H239	-1.220246463	1.519854528	-2.783221452
H240	-7.946000355	6.189441034	-1.862537022
H241	10.026284414	-3.029478698	3.543660695
H242	8.825503715	-2.047306087	3.031134577
H243	4.768561945	3.277390706	-2.222237831
H244	2.686338395	4.676641403	-0.837380718
H245	6.198693814	5.200112308	-0.859355100
H246	-0.627435753	4.038163628	-2.558783002
H247	-1.455483690	5.484565207	-4.186843543
H248	-3.054456640	5.362852832	-5.748360976
H249	-0.497437026	6.516594073	-3.462337739
H250	-3.490559588	3.132430893	-4.954799226
H251	-4.898668729	4.232202446	-5.131675457
H252	7.101428074	8.481018442	-3.993915627
H253	7.025176607	10.246201478	-4.179731010
H254	5.481827500	7.540550598	-5.170061263
H255	3.850533556	8.200632573	-5.316306348
H256	2.817455602	7.763932997	-8.075602763
H257	0.929728539	3.898974513	-6.876202906
H258	-0.411674856	10.867749093	-3.276328466
H259	2.764414866	8.889752623	0.001525995
H260	1.064008407	8.001295490	-5.006820854
H261	4.146901520	2.541113413	-4.557100818
H262	6.261045879	3.355469607	-4.025843955
H263	1.885428895	3.714000168	-4.365697208
H264	-6.721823986	4.414867467	-2.717283887
H265	9.029423848	4.566943796	-5.222822200
H266	-5.299008611	2.078988092	-3.724634000
H267	4.373737351	3.274386872	-6.142424171
H268	1.549455019	7.423390245	-1.556385631
H269	8.930178679	5.375427052	-3.645428496
H270	2.535829351	6.068569812	-8.305333045
H271	1.435202634	8.864971038	-6.456277789
H272	-6.280686059	-0.511922409	-3.477663297
H273	-6.596152344	0.556874003	-1.034908799
H274	0.908429177	12.353387219	-1.797873538
H275	-3.292973059	3.735459634	-2.563002178
H276	4.541155662	5.533653603	-5.118855309
H277	2.569818888	4.440716149	-7.245122408
H278	2.085256282	2.441836118	-5.558269738
H279	0.798240309	5.733469454	-5.167600052
H280	5.922443345	7.100216447	1.337434764
H281	5.616037396	7.732651243	-1.801052269
H282	7.720272668	9.731624405	-1.963165091
H283	7.417530644	6.348923386	0.729765911
H284	4.530958812	9.982863357	-0.794988456
H285	1.793270979	-7.218808724	-7.753081756

	H286	0.975849569	-8.083465230	-6.436828768
	H287	2.961142422	-9.979401391	-2.746547278
	H288	0.814588749	-9.444850088	-8.358425141
	H289	-0.666341836	-8.752497803	-8.241973637
	H290	7.179780518	0.208532966	-0.886043591
	H291	9.047498555	2.381006157	0.076181952
	H292	8.765832630	2.504243548	-4.181072734
	H293	6.626823158	1.871919068	-0.518858234
	H294	10.269607091	1.958414082	-0.923732078
	H295	5.965779872	-2.765598207	-6.379575160
	H296	8.793288944	1.073524387	-2.539468941
	H297	0.008721491	-7.254512899	6.915023914
	H298	0.647181683	-9.645469529	5.101131272
	H299	3.685548399	-2.784284578	-6.385509081
	H300	4.220330368	-2.429016223	3.571899920
	H301	1.408843442	-1.269413886	-8.665297697
	H302	-2.166044362	-1.974050427	-4.111779181
	H303	1.577093204	-0.338525409	-7.174528490
	H304	1.100994674	-4.952287899	5.837632192
	H305	1.666431712	-2.749882576	-6.059266384
	H306	0.438599002	-5.510467014	4.298432846
	H307	-0.561595807	-7.526813401	5.250612601
	H308	1.880216358	-7.589122431	4.498480252
	H309	2.362022920	-7.064483027	6.116209917
	H310	-4.550971443	0.910332296	-7.072749821
	H311	-1.231551407	-9.446649299	7.420981401
	H312	-3.437286937	-11.149608620	6.103808541
	H313	0.144598892	-1.069933864	-4.060817813
	H314	-2.434656884	-11.187929639	8.843148263
	H315	-3.846089752	-10.600748353	8.255120855
	H316	2.701420008	-5.564348420	3.375623065
	H317	0.696253325	1.003030395	-5.294123794
	H318	-3.889240484	-0.905120371	-5.553831055
	H319	-0.095616507	-12.563751479	1.505235991
	H320	0.416834156	-10.808653136	-1.243609273
	H321	0.177220656	-10.195305237	2.549618848
	H322	-1.142206118	-12.824282239	0.383164025
	H323	5.438725023	-3.077677542	-9.116312169
	H324	1.973250908	-7.731068732	1.723022732
	H325	-2.969760629	-7.063460956	2.649179912
	H326	1.367007455	-5.321338449	-6.530280376
	H327	-3.163857153	-9.132049738	1.552663935
	H328	-1.790461848	-8.792881805	-5.114039333
	H329	-1.448246731	-6.712107365	-3.694567823
	H330	-2.562635780	-12.468928348	6.889167700
	H331	3.735421778	-3.678806766	2.456521301
	H332	3.050222424	-9.256183369	3.127451155
	H333	3.471531414	-7.339680616	1.619607196
	H334	2.815961575	-9.940263330	5.365009895
	H335	2.491480410	-3.638586432	6.383968411
	H336	-2.054346188	-9.555227537	-1.273845519
	H337	-1.326379066	-10.233419739	0.190204688
	H338	-3.800659073	-8.400497415	0.071197764
	H339	-0.242210078	-3.921574109	-5.367893955
	H340	-1.024746772	2.084801806	-6.763102447
	H341	-3.399071411	2.180987342	-7.559978045
	H342	-1.798555516	-5.843680658	2.120640428
	H343	2.176740842	-10.541627507	3.272052451
	H344	0.096563346	-11.324369656	3.599366659
	H345	2.986188834	-2.175927453	5.618842599
	H346	-0.401593333	-10.777341754	-4.271659300
	H347	1.338068889	-9.352018285	-0.902352417
	H348	-1.433954720	-4.329447517	-6.639422406
	H349	5.914239440	-1.059894163	-6.876905479
	H350	6.157244725	-3.519256308	5.036942743
	H351	6.422400805	-3.645912140	3.279952153
	H352	2.892565968	-12.199188728	-3.551601190
	H353	1.507665569	-12.542035716	-2.749033061
	H354	2.750107373	-13.122622064	1.614238557
	H355	1.591719681	-12.199651982	-0.108016148
	H356	4.487620510	-13.740114089	-0.432124238
	H357	3.711429118	-11.630604914	1.664831049
	H358	8.356170000	-5.495613992	3.694983645
	H359	5.736894356	12.737453498	0.688011536
	H360	3.762989883	-10.078209214	8.214497193
	H361	4.658555345	-8.023750432	6.587498589
	H362	3.852271626	12.537816700	-1.685592267
	H363	3.099026202	11.184528625	0.976687994
	H364	2.279711611	12.588912970	0.263465552
	H365	-0.925961213	8.672234725	-3.776429161

	H366	1.990964935	-2.024583849	1.235739487
	H367	-0.358278102	-3.740645109	0.625189181
	H368	0.546424035	-1.222677388	1.010767466
	Mo369	0.363786433	-6.361093317	-0.951793779
	N370	1.290711402	-1.740418191	0.543626038
	N371	1.646353727	0.773376212	-0.838570492
	N372	1.391475994	1.844833552	-0.607787979
	N373	-9.878284386	-1.398166595	-0.051584185
	N374	-9.770077581	2.137522020	1.172603473
	N375	-9.535747020	2.270089423	3.973208157
	N376	-7.950197650	0.037611761	4.601081227
	N377	-5.463293893	0.871316954	3.498369631
	N378	-4.928328638	3.065460184	5.264156268
	N379	-5.011394142	2.002400188	7.986451370
	N380	-3.990348400	-0.690153351	7.981253533
	N381	-1.742135807	-0.506253350	6.280606968
	N382	-0.086931758	2.062005178	6.435179095
	N383	-0.389807246	2.947760409	9.197615644
	N384	-2.844898099	-10.665346707	8.066303426
	N385	-0.768533859	-9.819292130	6.586201557
	N386	2.480258716	-5.059111079	4.240702968
	N387	2.915083178	-3.188927892	5.580532870
	N388	3.584478380	-3.199726621	3.369193680
	N389	2.785316978	-9.853884188	6.405213147
	N390	-6.451578580	0.469445765	-3.192773560
	N391	-4.900583907	-3.931432615	-2.344078321
	N392	-5.087695957	2.714948331	-2.940202519
	N393	-6.189457494	5.227162868	-2.413069422
	N394	-6.015028693	5.423390555	0.390053539
	N395	-3.259412592	5.615462310	0.968225835
	N396	-1.013099785	3.444946800	-1.814870248
	N397	-1.893307111	1.699361491	-0.750174993
	N398	-2.067657048	8.106352690	0.862005524
	N399	-2.887176438	12.178983445	-1.447655322
	N400	-1.781028686	13.806599398	-0.422905544
	N401	9.036362085	-3.016464137	3.285273596
	N402	9.335478749	1.677670065	-0.611933814
	N403	8.516309928	3.321880700	-3.630867707
	N404	6.038142538	4.287431517	-4.392769624
	N405	1.463899625	5.703625899	-5.970073299
	N406	2.360073797	6.910829531	-7.769925175
	N407	1.561176470	8.021837735	-5.902159437
	N408	4.312611081	4.192449270	-2.068993301
	N409	5.691327422	5.954303693	-0.389619638
	N410	6.218127940	8.453182930	-1.402415889
	N411	4.837060642	8.338042291	-5.099815380
	N412	4.952368227	10.896746073	-0.946773520
	N413	0.317557203	-8.556299217	-8.449453168
	N414	0.377451296	-5.330779455	-6.794391674
	N415	1.249530240	-2.407373585	-6.942940281
	N416	4.106636097	-2.157555001	-7.077933193
	N417	-3.124131479	-3.825217359	-9.511172350
	N418	-3.225177369	-1.092135371	-8.584027186
	N419	2.019913676	-11.784638545	-3.211733575
	N420	-0.101564888	-7.956209336	-2.558222008
	N421	-1.144152762	-8.745018774	-4.330534025
	N422	2.595369257	-11.992045857	-0.113888799
	O423	-8.381678882	0.390879851	1.625579922
	O424	-7.541861495	3.350879233	3.752094159
	O425	-7.526182653	0.941324695	6.657801935
	O426	-4.479666819	-0.310772877	5.171863741
	O427	-2.720695493	2.673271839	4.872803303
	O428	-3.244097668	3.404744013	8.366139809
	O429	-3.255171928	-0.335793110	10.123330902
	O430	-0.890689057	-2.416680647	7.233296034
	O431	1.526987321	0.456737598	6.608681941
	O432	1.875226425	3.041718690	9.182513568
	O433	0.931700423	1.764621296	12.314377172
	O434	-0.906322509	-11.593942830	5.114273597
	O435	1.507413604	-9.423622818	8.267573478
	O436	6.250176843	-9.190267648	7.103958076
	O437	-5.250863512	2.734649878	-0.647644287
	O438	-5.635995243	-2.215497299	-3.638696768
	O439	-4.100140809	6.059612854	-2.027214825
	O440	-3.070214727	5.183767594	-4.791320708
	O441	-6.838743963	7.565760728	0.299558812
	O442	-3.691080185	5.739028831	3.207683518
	O443	-0.371875854	7.372188712	2.196125764
	O444	-0.609005969	10.762798145	2.674750933
	O445	8.411059062	-5.448391970	5.735159464

O446	5.588726634	2.452882119	3.555607763
O447	7.961339325	4.123560533	-1.567830430
O448	6.930778050	6.296104560	-5.062625715
O449	3.324050946	6.029129890	-3.003989270
O450	3.708526726	6.474397633	0.641431097
O451	5.297467817	3.313895847	0.782113344
O452	7.724418884	8.901398432	0.264416014
O453	6.512835231	12.009069590	-2.179607147
O454	4.271338818	10.200389381	-3.901958789
O455	4.562605062	14.301613642	0.103454541
O456	-0.164312382	8.194635338	-3.394578076
O457	-1.393526385	-6.662954033	-7.405273549
O458	-0.626249040	-2.529077862	-8.251762004
O459	3.886359145	-0.474868257	-8.587761918
O460	7.255723721	-2.188535064	-8.837056076
O461	-5.449734653	-1.252581586	-9.062404618
O462	-5.208710301	-5.696972837	-4.830772041
O463	-3.159133822	-6.323405837	-5.452359652
O464	-1.578276149	1.984505394	-9.391429016
O465	4.426950154	-11.016775372	-1.023168424
O466	5.578878123	-13.440453478	1.267110216
O467	-3.497695387	-5.347764340	-0.114974025
O468	-4.613998570	-5.233621702	1.834235238
O469	-2.636937057	-12.298531884	-0.288924124
O470	-4.417177350	-10.947102666	-0.630441399
O471	0.474985483	-7.830098663	0.682796062
O472	-0.614804025	-8.680593549	2.456805723
O473	-1.663560255	-6.879848654	-0.455751259
O474	3.020266504	-10.122762752	3.602591949
O475	2.789997789	-7.587985514	2.272873286
O476	-0.518869484	5.638455028	-3.901659552
O477	-0.309785970	-13.211313747	0.797491161
O478	0.574983100	-11.083169437	2.767086029
S479	5.815974252	1.141861465	-2.689206495
S480	2.765786501	-0.510953265	-3.306379007
S481	-0.513434412	-4.558985077	-2.216918493
S482	4.051206656	-0.857356342	-0.005674865
S483	-0.260235872	-1.350797370	-1.606767489
S484	2.632591395	-4.271766095	-4.642549083
S485	0.597015758	-4.804496000	0.957663338
S486	5.435265400	-2.779983812	-2.678270680
S487	2.602796002	-6.507326975	-1.685419846
S488	4.516623370	-4.583955357	0.634064312
end			

TS

Fe(139)	-2.455
Fe(140)	-0.049
Fe(141)	-2.540
Fe(142)	2.573
Fe(143)	2.621
Fe(144)	1.846
Fe(145)	-0.685

9651826nh22n2x3b26hx135tb.car_2

C1	-9.399323473	0.820934345	1.057644394
C2	-10.371706682	-0.058793007	0.263692685
C3	-8.839340246	3.110091270	1.751351921
C4	-8.564738796	2.913028498	3.238961205
C5	-9.278132382	1.931271815	5.357280337
C6	-8.148906464	0.918471191	5.582651988
C7	-6.769737155	-0.853471684	4.620317655
C8	-5.456453688	-0.086572419	4.444639657
C9	-4.287899370	1.718469117	3.279400937
C10	-3.893285048	2.525953987	4.524474959
C11	-4.588932002	3.854893746	6.424974231
C12	-4.166652470	3.036866394	7.654112464
C13	-4.763223398	1.226328295	9.199857487
C14	-3.874363168	-0.021382364	9.116532554
C15	-3.145440444	-1.912539147	7.731228280
C16	-1.801134634	-1.641091261	7.048556634
C17	-0.532798233	-0.210805182	5.517366618
C18	0.401355486	0.788521067	6.223740847
C19	-0.825349594	0.139104693	4.038333665
C20	0.455724919	0.542828615	3.298266744
C21	-1.507309229	-1.039657937	3.332436508
C22	0.809958099	3.068786132	7.013617029
C23	0.848031475	3.006831054	8.552336483
C24	0.490485703	4.476636521	6.456565616
C25	1.389485394	5.534487392	7.107769128
C26	0.658955607	4.497466998	4.931295117
C27	-0.333861550	2.871259798	10.664208946
C28	0.527198681	1.731187431	11.176479531
C29	-2.610107273	-11.361450675	6.805669546
C30	-1.337396610	-10.912187096	6.088989682
C31	0.430163928	-9.170746045	6.085626552
C32	1.599743505	-9.484699053	7.035820022
C33	0.265329497	-7.646997841	5.936171213

	C34	1.531174614	-7.000782221	5.374315750
	C35	1.345245694	-5.531513361	5.028783165
	C36	3.042165638	-3.799996863	4.435815999
	C37	3.975507725	-10.064262449	7.158519946
	C38	5.035764688	-9.010702060	6.930135060
	C39	-5.865412899	0.691597783	-1.852939292
	C40	-5.370226854	2.145225087	-1.725235645
	C41	-4.685549194	-0.270184773	-1.594949348
	C42	-5.082161103	-1.731759445	-1.306290773
	C43	-5.222300048	-2.638375034	-2.529260115
	C44	-4.242263521	3.897979838	-3.077819788
	C45	-4.841554903	5.162494035	-2.454947791
	C46	-3.954920782	4.073779946	-4.575924663
	C47	-6.877398309	6.317513020	-1.739854250
	C48	-6.573150294	6.490021238	-0.247318154
	C49	-5.607247182	5.528844581	1.787157339
	C50	-4.102481405	5.639117458	2.045069843
	C51	-1.824089223	5.693671555	1.151486682
	C52	-1.368898955	7.123962926	1.479761468
	C53	-1.057591278	5.272293623	-0.113198788
	C54	-1.320752900	3.872251624	-0.553793020
	C55	-1.871238315	2.771026849	0.069136795
	C56	-1.371943209	2.117354846	-1.915440161
	C57	-1.655132609	9.481662577	0.855337256
	C58	-1.457643087	9.928243640	2.301610231
	C59	-2.780368026	10.301800215	0.180411716
	C60	-2.447030729	11.702327844	-0.242746441
	C61	-1.752154683	12.704960524	0.405335135
	C62	-2.501654064	13.427795222	-1.550690621
	C63	8.193283515	-3.403106449	4.466696006
	C64	8.298408298	-4.904080343	4.672409396
	C65	6.701118936	-3.091005129	4.214743507
	C66	6.412717469	-1.614025212	4.067164783
	C67	6.064247929	-0.823442439	5.177918716
	C68	6.478032969	-0.987554289	2.808378720
	C69	5.779174912	0.539100211	5.040697568
	C70	6.209991167	0.373148883	2.658693654
	C71	5.853566767	1.134337403	3.774573413
	C72	8.403828082	1.733671669	-1.725645426
	C73	8.272786971	3.164503498	-2.274515279
	C74	7.039682733	1.222071650	-1.263696122
	C75	8.463889239	4.610129325	-4.269158396
	C76	7.065611578	5.134806399	-4.601153075
	C77	4.628837882	4.634411874	-4.489974623
	C78	4.022063451	5.019598537	-3.116174265
	C79	3.913586751	3.432897731	-5.145627758
	C80	2.390084433	3.471174264	-5.308734077
	C81	1.836916216	4.357710017	-6.436002286
	C82	1.779164566	6.850270737	-6.561622679
	C83	3.745459793	4.514390337	-0.739210410
	C84	4.367468540	5.765078197	-0.105187057
	C85	3.909520011	3.354385622	0.263083765
	C86	6.495886122	6.846628018	0.427376679
	C87	6.876513008	8.155026191	-0.243504831
	C88	6.644840893	9.586516837	-2.193158530
	C89	6.006819292	10.939792981	-1.789753779
	C90	6.536746236	9.372981127	-3.707605837
	C91	5.105872747	9.328747604	-4.229298436
	C92	4.115183413	12.072532492	-0.728922131
	C93	4.871394727	13.120821881	0.072207852
	C94	2.815166373	11.663166223	0.018290610
	C95	1.956555539	10.742885546	-0.819139227
	C96	1.042287531	11.269902847	-1.743331190
	C97	2.090880776	9.346291325	-0.745516377
	C98	0.299403123	10.438817851	-2.584114069
	C99	1.377129300	8.499512998	-1.598726509
	C100	0.491046214	9.057027425	-2.516043552
	C101	0.823609822	-7.628722217	-7.447179253
	C102	-0.154458314	-6.486523589	-7.223085563
	C103	-0.357067840	-4.140518141	-6.422430590
	C104	0.075539047	-2.941037309	-7.274269551
	C105	1.864961512	-1.301142657	-7.652750447
	C106	3.382363064	-1.290903152	-7.811495987
	C107	5.584355583	-2.035624462	-7.114957172
	C108	6.148981628	-2.475280272	-8.452350796
	C109	-4.260500966	-3.315395667	-8.705171635
	C110	-4.379848380	-1.789477126	-8.795757292
	C111	-4.306404493	-3.775263408	-7.230144525
	C112	-4.584106311	-5.287770292	-7.133956793
	C113	-4.366082812	-5.791010507	-5.725250201

	C114	-3.249416139	0.372593668	-8.656919678
	C115	-2.026359794	0.862602019	-9.406387607
	C116	-3.534941727	1.119753209	-7.330527864
	C117	-2.582017614	0.648863687	-6.271120474
	C118	-1.300288349	1.210893319	-6.164192228
	C119	-2.895712557	-0.489175490	-5.509057460
	C120	-0.322293568	0.585329331	-5.386247767
	C121	-1.918088035	-1.109242918	-4.729746610
	C122	-0.617801646	-0.595005040	-4.701793723
	C123	2.355398738	-10.701639968	-2.222391987
	C124	3.209084477	-11.218739583	-1.053574407
	C125	1.072834535	-10.035081810	-1.705246748
	C126	0.252963893	-9.299107143	-2.714294368
	C127	-0.392436240	-9.796451813	-3.826279304
	C128	-0.971874018	-7.677509919	-3.536893818
	C129	3.375099395	-12.459846593	1.018137116
	C130	4.582689634	-13.252206792	0.568555751
	C131	-3.681096893	-5.651557541	1.184706075
	C132	-2.587760197	-6.541166609	1.771434987
	C133	-1.948383003	-7.540363513	0.758116623
	C134	-2.905538365	-8.735230551	0.514865910
	C135	-2.299871369	-9.881199802	-0.297408541
	C136	-3.213015754	-11.119786577	-0.429445437
	C137	-0.634658946	-8.068477724	1.356315710
	C138	2.395501145	-3.251376698	-1.512568540
	Fe139	1.717019687	-4.720526673	-2.684044475
	Fe140	1.942384555	-1.014994687	-1.145977887
	Fe141	4.208295124	-2.934157243	-0.776350397
	Fe142	3.254383617	-2.723295437	-3.179732860
	Fe143	4.656993853	-0.714065933	-2.088424962
	Fe144	2.513122027	-4.800814251	-0.287411739
	Fe145	0.615857880	-3.118786696	-0.711009342
	H146	-9.582151542	-1.868545063	0.777286310
	H147	-9.087218971	-1.335715775	-0.682489276
	H148	-10.527804500	2.480277187	0.609741731
	H149	-10.659618200	0.469927610	-0.658911041
	H150	-11.292308618	-0.137269451	0.866696129
	H151	-7.861005439	3.087336575	1.248114704
	H152	-10.347087820	1.933576971	3.489126116
	H153	-9.268259593	4.111229077	1.608193728
	H154	-10.207390231	1.524079524	5.777721391
	H155	-8.414650865	0.134631178	3.692957655
	H156	-9.010343941	2.830381631	5.927010249
	H157	-3.414424223	1.119086187	2.995157378
	H158	-6.260177847	0.970732745	2.874400138
	H159	-6.873563522	-1.596928108	3.818345253
	H160	-3.762689537	4.542099234	6.200786627
	H161	-5.858545109	3.039829716	4.883120621
	H162	-6.717566755	-1.381321474	5.580144659
	H163	-4.511761683	2.402309232	2.448921482
	H164	-5.777350303	1.766963419	7.391301669
	H165	-5.475252731	4.447364223	6.690349761
	H166	-2.436343673	-1.341615992	3.836340968
	H167	-1.760602114	-0.771351228	2.294751956
	H168	-0.838338521	-1.915299543	3.298069904
	H169	-1.501583512	1.007646831	4.031006604
	H170	-5.747618085	0.910389444	9.575288929
	H171	-0.006387022	3.781835518	4.429027195
	H172	-1.233707077	2.952904278	8.719742945
	H173	1.233691530	5.602809750	8.193912067
	H174	0.219420176	0.803094555	2.255935114
	H175	1.187766604	-0.280274799	3.288071637
	H176	8.490193720	-2.918492804	5.417284372
	H177	0.439011839	5.498682114	4.532519992
	H178	1.187825941	6.522421286	6.667945256
	H179	1.698013369	4.246611923	4.658459868
	H180	2.451605595	5.295600537	6.935728829
	H181	-0.561338165	4.712689870	6.701067696
	H182	1.835584817	2.803797296	6.716843176
	H183	-0.986792002	2.315171854	6.079727738
	H184	0.940383502	1.419741499	3.751758555
	H185	3.655742882	2.410836070	-0.240036523
	H186	3.182142827	3.522830116	1.071170135
	H187	5.192319981	3.066579664	1.732344642
	H188	5.260371218	2.874799016	4.396885661
	H189	6.234407486	0.839817188	1.674443581
	H190	6.709263422	-1.582040442	1.921593270
	H191	6.002281132	-1.280407259	6.169241437
	H192	-2.595504164	-0.019218737	6.061889700
	H193	-3.698735188	-2.628577688	7.105366928

	H194	-2.923894188	-2.397656690	8.689298526
	H195	-4.520809193	-0.396856987	7.181725264
	H196	-4.302521233	1.885481025	9.945532383
	H197	5.486088692	1.132847364	5.910798841
	H198	0.058961043	-1.138387394	5.530194038
	H199	0.006504230	3.811024373	11.126834205
	H200	-1.365305089	2.679501461	10.996546650
	H201	0.650639492	0.879689990	10.466490424
	H202	4.397907315	-11.058317231	6.944132206
	H203	-5.015063574	-4.584362409	-3.124214598
	H204	-2.627712006	-5.914410145	-0.427942045
	H205	-5.632267524	-5.477882563	-7.407034012
	H206	-3.939458490	-5.837093805	-7.835628767
	H207	-5.095128136	-3.233353948	-6.685614201
	H208	-1.547264684	0.098338770	-10.062417203
	H209	-5.181123790	-3.673730256	-9.189393566
	H210	-4.006617117	-0.228705949	-2.460980703
	H211	-6.047382690	-1.767541060	-0.770664184
	H212	-2.368186274	-1.556554259	-8.331467996
	H213	-4.088154739	0.608905980	-9.343585179
	H214	-2.234902123	-3.612752651	-9.044875891
	H215	-4.514914521	-4.291630069	-1.462286404
	H216	-3.353947748	-3.514393378	-6.743420452
	H217	-3.199415960	-4.814189166	-9.624590056
	H218	-7.446006919	0.644329867	-3.188409409
	H219	-4.104535580	0.113810868	-0.745787535
	H220	-2.565186541	-6.353422611	-6.272951479
	H221	-4.344645514	-2.176696648	-0.622689417
	H222	-1.532306039	5.069071547	2.007233160
	H223	-2.256698268	2.705947355	1.082439197
	H224	-6.068012687	6.432021931	2.210777890
	H225	-5.820762754	4.553529920	-0.104993754
	H226	-2.898007750	7.827201920	0.307221166
	H227	-1.226230978	12.734341104	1.353316119
	H228	-2.189428278	9.473641282	3.012194536
	H229	-3.665454664	10.293765197	0.841478395
	H230	-0.705401454	9.613721647	0.313014247
	H231	-2.685053356	14.107382232	-2.379371266
	H232	0.013413766	5.418103204	0.105791064
	H233	-1.310623061	5.969582365	-0.929930030
	H234	-1.366537647	14.697113631	-0.270912369
	H235	-3.075838827	9.764572802	-0.734374730
	H236	-3.646377495	5.500523210	0.022934751
	H237	-5.983778070	4.676345704	2.372762315
	H238	-6.620531985	7.274606552	-2.214893329
	H239	-1.209843621	1.517439064	-2.807382120
	H240	-7.959043685	6.167593563	-1.851662955
	H241	10.000181467	-3.045058442	3.573711310
	H242	8.810208923	-2.051136073	3.059719837
	H243	4.757880691	3.286695820	-2.202202860
	H244	2.671426945	4.722449375	-0.847338716
	H245	6.191114873	5.195210630	-0.852603239
	H246	-0.617766900	4.034455607	-2.567026720
	H247	-1.450431990	5.480641243	-4.188537736
	H248	-3.063042503	5.379166773	-5.733371174
	H249	-0.493196064	6.513721141	-3.470047219
	H250	-3.489819064	3.143611520	-4.944018131
	H251	-4.900793860	4.240351611	-5.115893229
	H252	7.104719771	8.477505972	-3.993573994
	H253	7.019784999	10.241560503	-4.182552172
	H254	5.492517838	7.529351671	-5.176270809
	H255	3.858988074	8.182556629	-5.326663457
	H256	2.820788299	7.758461016	-8.083288673
	H257	0.941437421	3.891132089	-6.874269683
	H258	-0.421093136	10.866094737	-3.285811238
	H259	2.750492038	8.886761703	-0.003924093
	H260	1.061811514	8.000001019	-5.017008918
	H261	4.152114560	2.541184320	-4.544243132
	H262	6.262868855	3.355992674	-4.000908157
	H263	1.892341384	3.721021943	-4.358618624
	H264	-6.721279768	4.406070168	-2.708366015
	H265	9.035041204	4.557522895	-5.205159658
	H266	-5.285444639	2.078849133	-3.712211127
	H267	4.381541845	3.271246730	-6.130971782
	H268	1.537120870	7.421253590	-1.563720264
	H269	8.934781901	5.371467886	-3.630263341
	H270	2.544881958	6.060479415	-8.306694201
	H271	1.439639000	8.861113066	-6.466718167
	H272	-6.265456100	-0.507941105	-3.475312336
	H273	-6.595771189	0.559386017	-1.032199936

H274	0.896684636	12.351232838	-1.804218750
H275	-3.289129675	3.746271945	-2.550780256
H276	4.551484855	5.531350368	-5.113569599
H277	2.581341238	4.434589027	-7.239042123
H278	2.091080041	2.440398490	-5.542583828
H279	0.800785340	5.730559449	-5.170553483
H280	5.944869432	7.097876542	1.346053649
H281	5.612025156	7.729257902	-1.800351237
H282	7.711586579	9.734276648	-1.963601905
H283	7.430354698	6.346556738	0.717864477
H284	4.520644641	9.978129821	-0.797448090
H285	1.796540993	-7.224869111	-7.764552107
H286	0.978133500	-8.092462596	-6.450869038
H287	2.979945360	-9.945172590	-2.721047590
H288	0.811351561	-9.438152740	-8.387266908
H289	-0.669569063	-8.749149173	-8.253548574
H290	7.181636431	0.212830408	-0.853000090
H291	9.066424907	2.382667398	0.091632011
H292	8.761307456	2.498235324	-4.158339325
H293	6.636224944	1.880404360	-0.479461217
H294	10.279745445	1.936207603	-0.911491802
H295	6.001648554	-2.704534931	-6.345645338
H296	8.781344601	1.070447813	-2.518452060
H297	0.002686666	-7.210041916	6.914267428
H298	0.626917127	-9.601867814	5.095915075
H299	3.721568314	-2.747629022	-6.350669662
H300	4.231666407	-2.386495101	3.586055994
H301	1.427129990	-1.272362644	-8.658324957
H302	-2.162737686	-1.993474438	-4.137774892
H303	1.593597680	-0.350211914	-7.162411117
H304	1.189112377	-4.929900680	5.932473382
H305	1.703480630	-2.772706338	-6.065838944
H306	0.453321134	-5.388367755	4.395706127
H307	-0.579970588	-7.473242706	5.251757973
H308	1.831381350	-7.530735289	4.459766142
H309	2.365598141	-7.092386238	6.086304223
H310	-4.579314655	0.919201812	-7.049884667
H311	-1.258538686	-9.408134044	7.410861328
H312	-3.439925701	-11.144565352	6.115299812
H313	0.151052796	-1.090685909	-4.109872771
H314	-2.404555979	-11.176855047	8.845499291
H315	-3.841988892	-10.631678189	8.281580536
H316	2.698986087	-5.510414616	3.403531113
H317	0.680455341	1.006924668	-5.306233596
H318	-3.905344916	-0.905025403	-5.540923006
H319	-0.120386067	-12.554876983	1.508976359
H320	0.429870321	-10.803621609	-1.243431584
H321	0.166049654	-10.198094318	2.540844327
H322	-1.166900914	-12.807777023	0.384923514
H323	5.459683127	-3.094114495	-9.076677048
H324	1.954496897	-7.736017972	1.705551028
H325	-2.983391876	-7.074546665	2.643790886
H326	1.384915192	-5.331835024	-6.526411370
H327	-3.216076181	-9.114074351	1.500948635
H328	-1.800359142	-8.814410102	-5.106877171
H329	-1.478845541	-6.731697612	-3.686375778
H330	-2.548590220	-12.459794482	6.888969977
H331	3.725068414	-3.638196747	2.481586770
H332	3.038563251	-9.254915898	3.129363821
H333	3.449684270	-7.336178879	1.596567978
H334	2.792691696	-9.919098248	5.375275975
H335	2.595726796	-3.630398154	6.438569339
H336	-2.079069754	-9.524368171	-1.315256999
H337	-1.347266924	-10.201459486	0.147909113
H338	-3.810670190	-8.358146512	0.014626924
H339	-0.214404958	-3.927297675	-5.351095983
H340	-1.059859403	2.109650756	-6.737113214
H341	-3.431489662	2.196123210	-7.533496728
H342	-1.808040051	-5.850595357	2.134732495
H343	2.165241254	-10.537649016	3.281843116
H344	0.092885577	-11.316613120	3.602209117
H345	3.061786129	-2.159790461	5.677022622
H346	-0.386738715	-10.783139794	-4.271190479
H347	1.333711483	-9.338701865	-0.897714651
H348	-1.414189949	-4.331326636	-6.616392834
H349	5.937479539	-1.016616499	-6.897723185
H350	6.126119936	-3.498320817	5.060523342
H351	6.390921592	-3.627647817	3.303939447
H352	2.937102966	-12.166273703	-3.536301640
H353	1.536748684	-12.513942303	-2.762124598

H354	2.735280572	-13.142671006	1.598869030
H355	1.589794486	-12.176067301	-0.108750472
H356	4.481832154	-13.720885051	-0.440473650
H357	3.692386612	-11.648573123	1.691960914
H358	8.326757382	-5.497596450	3.727613232
H359	5.724041308	12.735671600	0.678919959
H360	3.719597547	-10.036003346	8.233040152
H361	4.654593970	-8.019043847	6.582888896
H362	3.838738911	12.531558167	-1.693383458
H363	3.086760531	11.181311590	0.970456648
H364	2.267925375	12.585975063	0.257090423
H365	-0.933777203	8.670476005	-3.788371169
H366	1.942301377	-2.052719817	1.257052256
H367	-0.521007460	-3.653048867	0.363165249
H368	0.506121506	-1.239310483	1.000384861
Mo369	0.366405677	-6.368860168	-0.970390716
N370	1.259624376	-1.760599175	0.550519434
N371	1.603203579	0.726985939	-0.826388934
N372	1.333220375	1.787422603	-0.569407470
N373	-9.904659507	-1.393777049	-0.069999866
N374	-9.745656719	2.136553406	1.161336043
N375	-9.507867686	2.262072869	3.960886378
N376	-7.932377858	0.022360239	4.587622862
N377	-5.448644159	0.862802484	3.480483599
N378	-4.905228258	3.055065229	5.252862334
N379	-4.954725626	1.972925909	7.964412028
N380	-3.975305971	-0.747153865	7.971608277
N381	-1.733210589	-0.515545284	6.281428869
N382	-0.069353888	2.047692851	6.443896382
N383	-0.336854660	2.935534202	9.211309597
N384	-2.842208995	-10.664419348	8.076810335
N385	-0.794462375	-9.777219185	6.575241883
N386	2.513262306	-5.023424532	4.286620550
N387	3.010215015	-3.174067486	5.634008369
N388	3.589796095	-3.156202322	3.397204446
N389	2.756490815	-9.827580687	6.415213526
N390	-6.438252398	0.472367360	-3.188456459
N391	-4.920435006	-3.937154144	-2.335355765
N392	-5.078330081	2.715876927	-2.927341644
N393	-6.194813525	5.221453007	-2.401993363
N394	-6.025965976	5.425632854	0.401556530
N395	-3.269161334	5.602256557	0.967432437
N396	-1.008356726	3.437671905	-1.828611579
N397	-1.901193691	1.688789965	-0.780013264
N398	-2.078882664	8.092762913	0.848957396
N399	-2.911178112	12.168273519	-1.463649219
N400	-1.794012668	13.791175572	-0.443280855
N401	9.011013240	-3.022461525	3.313261050
N402	9.342792229	1.671886202	-0.593626975
N403	8.518632231	3.318506671	-3.609156557
N404	6.042582854	4.286075114	-4.374703510
N405	1.468707893	5.699997365	-5.972455575
N406	2.365284592	6.904531385	-7.772394105
N407	1.562659824	8.017799327	-5.911223996
N408	4.299883105	4.203194692	-2.059397136
N409	5.690715164	5.951557685	-0.379196183
N410	6.214695816	8.449994776	-1.403377020
N411	4.844171282	8.324325157	-5.109035359
N412	4.940049320	10.892246630	-0.952868806
N413	0.312570373	-8.549400196	-8.465357631
N414	0.393459514	-5.335980976	-6.783095222
N415	1.277088267	-2.422484842	-6.941996765
N416	4.135592008	-2.130372356	-7.055849457
N417	-3.130997861	-3.800632256	-9.510264148
N418	-3.242728582	-1.075058990	-8.564094864
N419	2.058035932	-11.754077063	-3.210562009
N420	-0.113055874	-7.964085097	-2.558148787
N421	-1.152642212	-8.761593700	-4.325410165
N422	2.594264237	-11.976015718	-0.105822557
O423	-8.381503807	0.371449937	1.613839939
O424	-7.512573302	3.340835810	3.744196227
O425	-7.497307425	0.929752430	6.640824006
O426	-4.465993760	-0.308891740	5.163041233
O427	-2.701247397	2.676165837	4.836143221
O428	-3.192695773	3.383689835	8.344006435
O429	-3.163032744	-0.348103220	10.076888675
O430	-0.863852487	-2.439449664	7.194073211
O431	1.548278116	0.438761506	6.547963885
O432	1.927511017	3.019388523	9.170659855
O433	1.003732021	1.701175087	12.301431848

	O434 -0.897850663 -11.572430140 5.125308211<br>O435 1.472081172 -9.375163163 8.267418658<br>O436 6.226269302 -9.200691575 7.123938815<br>O437 -5.247623766 2.733668312 -0.635103985<br>O438 -5.623862512 -2.213182605 -3.637706738<br>O439 -4.110466323 6.065081330 -2.012294074<br>O440 -3.073951246 5.195608472 -4.777090419<br>O441 -6.853225573 7.566333029 0.301778254<br>O442 -3.690286978 5.756129642 3.206983137<br>O443 -0.379425952 7.362870956 2.181266901<br>O444 -0.635177822 10.751675778 2.667828718<br>O445 8.326561058 -5.445484537 5.768370097<br>O446 5.566572107 2.467970627 3.565195324<br>O447 7.968827494 4.124780950 -1.546266772<br>O448 6.940004984 6.288470163 -5.058351413<br>O449 3.323558587 6.038930317 -3.008836493<br>O450 3.713265935 6.494537369 0.650036004<br>O451 5.241760814 3.294168754 0.782268829<br>O452 7.724638255 8.902886367 0.258967995<br>O453 6.496418339 12.005611816 -2.189635151<br>O454 4.265326265 10.181748162 -3.909177679<br>O455 4.548925221 14.298460340 0.092526996<br>O456 -0.173224100 8.192890964 -3.404293145<br>O457 -1.384869254 -6.650337217 -7.410948789<br>O458 -0.626806662 -2.513548293 -8.214931681<br>O459 3.898863056 -0.485360326 -8.604434650<br>O460 7.285258532 -2.217148783 -8.816787815<br>O461 -5.464331003 -1.239862441 -9.054455796<br>O462 -5.205339481 -5.700147114 -4.836523109<br>O463 -3.154185420 -6.321809082 -5.457553174<br>O464 -1.606560099 2.010489316 -9.362672175<br>O465 4.435164800 -10.999317469 -0.995543363<br>O466 5.570322580 -13.443473090 1.264663325<br>O467 -3.500554831 -5.311352010 -0.091648317<br>O468 -4.626395922 -5.232981351 1.855296989<br>O469 -2.657344019 -12.269963591 -0.308152172<br>O470 -4.435263149 -10.922061229 -0.678562756<br>O471 0.455021406 -7.834388410 0.672297627<br>O472 -0.645426524 -8.694043611 2.435975948<br>O473 -1.671898660 -6.848350562 -0.458601796<br>O474 3.005331443 -10.115210705 3.616173244<br>O475 2.777487269 -7.609192721 2.249934918<br>O476 -0.513944568 5.633374059 -3.903976232<br>O477 -0.343482580 -13.204246653 0.805782449<br>O478 0.567286374 -11.083611721 2.765962401<br>S479 5.813070473 1.160661669 -2.655818803<br>S480 2.736132242 -0.545569549 -3.302452870<br>S481 -0.483975348 -4.593709970 -2.277384755<br>S482 4.025306868 -0.849904672 0.006534526<br>S483 -0.277100437 -1.400858619 -1.622931263<br>S484 2.683052885 -4.276166715 -4.646899941<br>S485 0.561987530 -4.775955603 0.873953146<br>S486 5.435504892 -2.755470164 -2.649440663<br>S487 2.621809677 -6.530812756 -1.668179199<br>S488 4.472538908 -4.571303521 0.691346089<br>end

product

Fe(139) -2.441<br>Fe(140) -0.400<br>Fe(141) -2.523<br>Fe(142) 2.786<br>Fe(143) 2.967<br>Fe(144) 1.948<br>Fe(145) -0.673	9651826nh22n2x3b26hx135tb.car_7<br>C1 -9.387280769 0.825717591 1.060558422<br>C2 -10.345846282 -0.067067850 0.262295949<br>C3 -8.873212719 3.122890149 1.764840300<br>C4 -8.595281131 2.923660758 3.251134449<br>C5 -9.306622682 1.941553169 5.370269314<br>C6 -8.175801265 0.931127011 5.598646185<br>C7 -6.790664350 -0.840072035 4.641744241<br>C8 -5.473981950 -0.079705551 4.461454027<br>C9 -4.298213395 1.720673009 3.304725645<br>C10 -3.912003119 2.529844489 4.551277106<br>C11 -4.630018883 3.873149214 6.437054188<br>C12 -4.220928595 3.061994397 7.674960827<br>C13 -4.827720352 1.259809918 9.224347070<br>C14 -3.939443894 0.010839462 9.149074217<br>C15 -3.170922735 -1.859491945 7.749527132<br>C16 -1.820488095 -1.611954770 7.069081232<br>C17 -0.524322583 -0.211701825 5.524597145<br>C18 0.389554949 0.798948259 6.245713871<br>C19 -0.797607406 0.138500140 4.045414007<br>C20 0.494584902 0.539595324 3.322238195<br>C21 -1.474139637 -1.038323679 3.331439399

C22	0.773051019	3.092289111	7.011272846
C23	0.797426446	3.024323496	8.550201205
C24	0.433953705	4.500682408	6.467599316
C25	1.324580992	5.562519195	7.125204284
C26	0.593876632	4.539694362	4.942290004
C27	-0.412864215	2.889808535	10.643527042
C28	0.459907515	1.772819903	11.181967716
C29	-2.610236824	-11.366604288	6.787523741
C30	-1.329089056	-10.934472458	6.079303610
C31	0.460465072	-9.215710012	6.091504957
C32	1.636562496	-9.525327035	7.034488143
C33	0.285688999	-7.693225283	5.940190259
C34	1.545580988	-7.029859791	5.385819898
C35	1.308219232	-5.588980645	4.959070431
C36	2.994099024	-3.838723599	4.385375163
C37	4.018857600	-10.084606608	7.134478553
C38	5.063612547	-9.013817287	6.914681862
C39	-5.856996195	0.688200628	-1.858795479
C40	-5.366738471	2.143374785	-1.737004974
C41	-4.673382545	-0.267213651	-1.609205364
C42	-5.066961016	-1.729866209	-1.316063512
C43	-5.213802934	-2.637107809	-2.537882187
C44	-4.240294289	3.891423023	-3.092607474
C45	-4.832540751	5.159641034	-2.469242202
C46	-3.951249932	4.063690072	-4.590476327
C47	-6.859327428	6.326894547	-1.749231525
C48	-6.557793673	6.490864451	-0.255257172
C49	-5.599206890	5.519091594	1.776361481
C50	-4.095666717	5.632393588	2.041940927
C51	-1.812439208	5.704290739	1.160835636
C52	-1.358334660	7.134554735	1.492726868
C53	-1.037868028	5.283507282	-0.098920821
C54	-1.294026241	3.880560257	-0.533739470
C55	-1.835441339	2.778869876	0.097075684
C56	-1.362057341	2.125638397	-1.893666465
C57	-1.638222809	9.490552865	0.869424792
C58	-1.430698350	9.936791320	2.314454810
C59	-2.766476594	10.319911597	0.207086135
C60	-2.430172608	11.719242085	-0.217645488
C61	-1.743558465	12.725534588	0.433370306
C62	-2.475249609	13.439756256	-1.532475818
C63	8.234173761	-3.405127915	4.445434227
C64	8.356744377	-4.904892944	4.644145222
C65	6.735614853	-3.107273183	4.193537033
C66	6.434228824	-1.634899033	4.044709674
C67	6.076975205	-0.848248363	5.154568322
C68	6.501905392	-1.006494284	2.787251098
C69	5.790476693	0.514182409	5.019892835
C70	6.232965672	0.354658676	2.639236390
C71	5.872451824	1.113768329	3.756018255
C72	8.418889233	1.740926541	-1.751840016
C73	8.280672397	3.170309820	-2.298512683
C74	7.051106094	1.217769207	-1.306819290
C75	8.462901914	4.620616766	-4.288754196
C76	7.062049600	5.143838991	-4.614637913
C77	4.627368474	4.641616144	-4.503252335
C78	4.025826385	5.014905583	-3.124043527
C79	3.909095981	3.440621748	-5.160509427
C80	2.384340001	3.475794078	-5.319900547
C81	1.825391467	4.366759622	-6.439800310
C82	1.776554950	6.858926567	-6.556627188
C83	3.760706474	4.488041647	-0.749826910
C84	4.369368027	5.745869869	-0.114915819
C85	3.942986932	3.325364563	0.247781316
C86	6.483907263	6.849720431	0.423979895
C87	6.873784088	8.158329542	-0.241971706
C88	6.654888525	9.597514908	-2.189914970
C89	6.021852208	10.950890682	-1.781879371
C90	6.542259005	9.386740717	-3.704436076
C91	5.109424732	9.347639642	-4.221068409
C92	4.129377595	12.082058461	-0.720125942
C93	4.884853520	13.128408785	0.083999105
C94	2.828895874	11.669806649	0.025333009
C95	1.971592170	10.748333665	-0.812496371
C96	1.055821489	11.273796936	-1.735959637
C97	2.107193890	9.351805832	-0.738353398
C98	0.311902339	10.441484001	-2.574381598
C99	1.392623732	8.503429527	-1.589416856
C100	0.504167923	9.059667448	-2.505419482
C101	0.820756336	-7.627132248	-7.431588267

	C102	-0.164389609	-6.489129740	-7.218279263
	C103	-0.376728925	-4.135402555	-6.442254919
	C104	0.065637268	-2.939804370	-7.293786501
	C105	1.844923185	-1.286446896	-7.652328472
	C106	3.361470840	-1.289037997	-7.815158874
	C107	5.558815250	-2.071652111	-7.142050435
	C108	6.122773107	-2.465724571	-8.491586445
	C109	-4.243549839	-3.334923930	-8.702351566
	C110	-4.356963264	-1.807519246	-8.802388461
	C111	-4.299874783	-3.783943177	-7.225292065
	C112	-4.588443748	-5.294346374	-7.124130060
	C113	-4.370894766	-5.795009771	-5.714607883
	C114	-3.217158490	0.350576865	-8.682082663
	C115	-1.997895449	0.836206009	-9.439742653
	C116	-3.489163440	1.095640040	-7.352843223
	C117	-2.544757622	0.621056308	-6.285804676
	C118	-1.261325429	1.177026702	-6.163585484
	C119	-2.881379945	-0.496821574	-5.504762310
	C120	-0.313448292	0.579944325	-5.328301276
	C121	-1.933754306	-1.089132331	-4.667822772
	C122	-0.636825850	-0.569993284	-4.605037248
	C123	2.335412615	-10.718332686	-2.226788970
	C124	3.204187086	-11.240166319	-1.072566906
	C125	1.077002019	-10.025301863	-1.683026183
	C126	0.261659608	-9.258989419	-2.674229226
	C127	-0.377883787	-9.748211359	-3.794470531
	C128	-0.966869504	-7.637769676	-3.483422789
	C129	3.390879685	-12.452992487	1.012122704
	C130	4.593837020	-13.257406562	0.572520506
	C131	-3.703640059	-5.657081193	1.222796793
	C132	-2.566449322	-6.508778174	1.785445345
	C133	-1.919620210	-7.483866843	0.759662447
	C134	-2.864927579	-8.695407658	0.517127636
	C135	-2.261299388	-9.862207433	-0.268804599
	C136	-3.183445934	-11.097136138	-0.380907240
	C137	-0.602631756	-8.033178786	1.325291167
	C138	2.409708836	-3.241659612	-1.507530295
	Fe139	1.756092313	-4.724885038	-2.701097636
	Fe140	1.834891309	-1.220069816	-1.147730823
	Fe141	4.223991769	-2.933947089	-0.807010821
	Fe142	3.284965457	-2.759509415	-3.214055804
	Fe143	4.632659473	-0.702681265	-2.105925185
	Fe144	2.586530223	-4.797142796	-0.314351769
	Fe145	0.480878890	-3.374253539	-0.594024924
	H146	-9.568172318	-1.872062347	0.807871187
	H147	-9.040877509	-1.351184130	-0.644573251
	H148	-10.549271507	2.464219482	0.619823728
	H149	-10.620542687	0.452318073	-0.669773460
	H150	-11.276840261	-0.140785881	0.851156205
	H151	-7.894886279	3.121718571	1.261103843
	H152	-10.375702525	1.939668080	3.502686819
	H153	-9.323822757	4.115217837	1.625584596
	H154	-10.235079948	1.533421080	5.791935024
	H155	-8.430754868	0.150006975	3.706887653
	H156	-9.040697700	2.842269472	5.938647243
	H157	-3.425371327	1.115689306	3.030610783
	H158	-6.276442409	0.988622554	2.895712907
	H159	-6.895290967	-1.588771475	3.845057192
	H160	-3.802797541	4.561309009	6.219311467
	H161	-5.879856693	3.049586770	4.885595773
	H162	-6.741247651	-1.361376016	5.605336098
	H163	-4.511467914	2.403115163	2.470578139
	H164	-5.838011509	1.797855614	7.414079402
	H165	-5.521199800	4.464941421	6.687792669
	H166	-2.409550157	-1.339214245	3.825252845
	H167	-1.714676859	-0.771505997	2.289703236
	H168	-0.806267018	-1.915854979	3.305885299
	H169	-1.472361601	1.008463454	4.028846919
	H170	-5.812630440	0.942338974	9.597093030
	H171	-0.072496990	3.828387504	4.435296077
	H172	-1.286790226	2.969968047	8.691052408
	H173	1.170456962	5.622684110	8.212178612
	H174	0.275989266	0.800055479	2.275255223
	H175	1.227020183	-0.283677081	3.323203982
	H176	8.528405510	-2.918771364	5.395029309
	H177	0.369147922	5.544906105	4.557049403
	H178	1.113754492	6.551843201	6.692559076
	H179	1.632312073	4.295454758	4.661143342
	H180	2.388542466	5.334677675	6.949789172
	H181	-0.619013287	4.721631630	6.721058019

H182	1.805271401	2.846430929	6.720238184
H183	-1.002993617	2.319607721	6.053014620
H184	0.973593768	1.416316323	3.783048227
H185	3.697902821	2.378233430	-0.256048473
H186	3.218482779	3.483349727	1.060371494
H187	5.235970575	3.059910863	1.713560451
H188	5.282690477	2.854103321	4.381553082
H189	6.261890329	0.824588348	1.655449748
H190	6.738354557	-1.597024304	1.899350443
H191	6.009398363	-1.307287971	6.144020396
H192	-2.593651809	-0.000336545	6.047987966
H193	-3.731714920	-2.571230669	7.125376768
H194	-2.961764195	-2.341129362	8.711905695
H195	-4.522811717	-0.326018403	7.188130389
H196	-4.371582768	1.920024276	9.972186159
H197	5.491787867	1.105728500	5.889589195
H198	0.066393974	-1.139153301	5.553123161
H199	-0.097578880	3.840321528	11.106341251
H200	-1.447052513	2.682902809	10.961459275
H201	0.605845020	0.914007418	10.487278543
H202	4.453032551	-11.069053202	6.901030762
H203	-5.005107457	-4.584190430	-3.129842764
H204	-2.690872576	-5.858345188	-0.408605040
H205	-5.638213763	-5.477799998	-7.396033769
H206	-3.948059700	-5.849609262	-7.825220503
H207	-5.089280019	-3.233922851	-6.689741261
H208	-1.531522279	0.073511849	-10.105994350
H209	-5.167117030	-3.691723467	-9.185811570
H210	-3.998919763	-0.228285274	-2.478375536
H211	-6.029560755	-1.763915092	-0.774399015
H212	-2.338899514	-1.580607314	-8.365256114
H213	-4.061881276	0.590736140	-9.363062877
H214	-2.218351646	-3.638113653	-9.048690270
H215	-4.514682888	-4.286483321	-1.465661640
H216	-3.350597817	-3.526875929	-6.731310318
H217	-3.187973669	-4.845412500	-9.609849942
H218	-7.450081196	0.639811541	-3.180817271
H219	-4.087725124	0.116536676	-0.763208639
H220	-2.570513140	-6.358511220	-6.262075406
H221	-4.325636076	-2.174890814	-0.635290520
H222	-1.526508343	5.079961990	2.019003209
H223	-2.207861781	2.714424771	1.115280931
H224	-6.063150171	6.419306893	2.202527078
H225	-5.807800434	4.552915821	-0.121441349
H226	-2.882301872	7.839460100	0.315961296
H227	-1.227377697	12.760215010	1.386354797
H228	-2.158814772	9.484471336	3.030310086
H229	-3.644840364	10.314353970	0.877002221
H230	-0.692244006	9.621383913	0.320915197
H231	-2.652383147	14.116346698	-2.365096052
H232	0.031566805	5.437255528	0.123000123
H233	-1.292221725	5.976066035	-0.919030031
H234	-1.354089666	14.715741011	-0.247011754
H235	-3.073529592	9.786249559	-0.706025582
H236	-3.628899284	5.514907868	0.021026600
H237	-5.975577879	4.664174736	2.358358690
H238	-6.589515035	7.282396177	-2.220680151
H239	-1.209499405	1.526339734	-2.788023704
H240	-7.942258665	6.190610331	-1.865293838
H241	10.035088617	-3.022865141	3.550345194
H242	8.829870284	-2.048567468	3.031515112
H243	4.775342491	3.279289556	-2.227035006
H244	2.683145890	4.682433231	-0.850484999
H245	6.199205305	5.202242178	-0.865692308
H246	-0.618308872	4.043035146	-2.556411358
H247	-1.454240965	5.484766238	-4.185433634
H248	-3.051147656	5.361753327	-5.750246032
H249	-0.495236342	6.518042817	-3.459371130
H250	-3.488744402	3.131535072	-4.956841069
H251	-4.896107362	4.232561606	-5.132012694
H252	7.105141714	8.488784883	-3.992822189
H253	7.027210047	10.253665716	-4.179643641
H254	5.485367726	7.545043473	-5.167083149
H255	3.853240151	8.203724614	-5.312390638
H256	2.815451067	7.767828913	-8.075770262
H257	0.926252642	3.902791736	-6.873386457
H258	-0.410315916	10.867586002	-3.274812058
H259	2.768531467	8.893591716	0.002540662
H260	1.065789928	8.004802239	-5.004226279
H261	4.151071499	2.547370722	-4.561912978

H262	6.261841865	3.359959909	-4.024947615
H263	1.888085252	3.717890779	-4.366982435
H264	-6.718631244	4.414441002	-2.718487939
H265	9.031201831	4.572329152	-5.227141964
H266	-5.294529602	2.077909329	-3.725052663
H267	4.374498955	3.281902068	-6.147830545
H268	1.553727861	7.425146579	-1.553977750
H269	8.934922574	5.381760750	-3.649955827
H270	2.531573116	6.073527254	-8.305758120
H271	1.434899076	8.869509919	-6.454187732
H272	-6.272118065	-0.513598448	-3.477059948
H273	-6.581062704	0.552942029	-1.032359482
H274	0.909642851	12.354720657	-1.798161093
H275	-3.288428896	3.735837063	-2.564324103
H276	4.545209896	5.541114995	-5.121392450
H277	2.564804835	4.445102871	-7.247871734
H278	2.086777843	2.446000817	-5.559861668
H279	0.798251715	5.736579554	-5.166018286
H280	5.918630306	7.101256639	1.333573909
H281	5.616338319	7.740668216	-1.805542791
H282	7.722677248	9.738943304	-1.961525314
H283	7.415153811	6.351848843	0.727638067
H284	4.533910811	9.987994675	-0.795344826
H285	1.793357683	-7.219818586	-7.745842889
H286	0.971962283	-8.082077637	-6.430497059
H287	2.961046147	-9.975109249	-2.742871437
H288	0.819414754	-9.446585172	-8.348912509
H289	-0.663294170	-8.758024332	-8.237078367
H290	7.189700589	0.206746879	-0.901201862
H291	9.055938705	2.387349766	0.078541168
H292	8.764059513	2.509064536	-4.185392666
H293	6.637776585	1.870473600	-0.524239010
H294	10.281055696	1.970828911	-0.920162260
H295	5.967767300	-2.768752251	-6.394001287
H296	8.815457671	1.080146868	-2.538828463
H297	0.008925652	-7.258154615	6.915135971
H298	0.655187238	-9.646916628	5.101351524
H299	3.688086650	-2.787223273	-6.396223846
H300	4.223419520	-2.438477953	3.567899418
H301	1.405370892	-1.262173432	-8.657166564
H302	-2.198807782	-1.957971998	-4.061414030
H303	1.581820651	-0.331683380	-7.166583369
H304	1.088743564	-4.949693050	5.822976276
H305	1.661640786	-2.734377867	-6.048720900
H306	0.436463646	-5.525747051	4.286235038
H307	-0.557016696	-7.530656479	5.249584263
H308	1.891321704	-7.591255002	4.506765622
H309	2.361143583	-7.053244586	6.124330671
H310	-4.535229376	0.905023189	-7.070054185
H311	-1.225694348	-9.449241781	7.419391071
H312	-3.433841513	-11.142313442	6.092311010
H313	0.113163451	-1.043723363	-3.968612283
H314	-2.434693486	-11.189576453	8.833222315
H315	-3.844890443	-10.598448018	8.244787239
H316	2.687018275	-5.563559486	3.361475567
H317	0.690257474	0.996771390	-5.233381271
H318	-3.890056637	-0.912645420	-5.558946322
H319	-0.086033773	-12.561305400	1.507227757
H320	0.421888060	-10.786487040	-1.225612337
H321	0.191542526	-10.194186380	2.524807427
H322	-1.133735835	-12.806381621	0.384294098
H323	5.440075869	-3.076141458	-9.129799771
H324	1.987374461	-7.728123252	1.686869669
H325	-2.929053752	-7.050611322	2.667245137
H326	1.367543350	-5.317649111	-6.531272491
H327	-3.164939613	-9.058323298	1.512708124
H328	-1.781557484	-8.756544896	-5.073420711
H329	-1.479808523	-6.696670731	-3.633003378
H330	-2.563461802	-12.465430761	6.875974909
H331	3.759354636	-3.697998822	2.457282322
H332	3.055814321	-9.256386892	3.119687718
H333	3.482371588	-7.322203386	1.614399193
H334	2.821873140	-9.938685560	5.363527813
H335	2.474451237	-3.640438163	6.371635248
H336	-2.028780953	-9.534468189	-1.293455349
H337	-1.315098559	-10.183250411	0.188843062
H338	-3.779407866	-8.339784711	0.018139841
H339	-0.242426137	-3.919387548	-5.370822738
H340	-0.999031882	2.055721041	-6.757955827
H341	-3.378627515	2.172231089	-7.552690416

H342	-1.801506929	-5.790113654	2.125521248
H343	2.184325978	-10.541015426	3.272131301
H344	0.090081823	-11.305055156	3.594802471
H345	2.987880459	-2.181152432	5.609671445
H346	-0.367470804	-10.732977902	-4.245266698
H347	1.364772932	-9.349530140	-0.868954320
H348	-1.432605560	-4.325730166	-6.644643433
H349	5.914080471	-1.061441102	-6.886436034
H350	6.166169324	-3.520865599	5.039630375
H351	6.434224696	-3.649988298	3.283452488
H352	2.872200223	-12.189543653	-3.551072496
H353	1.495830288	-12.530822796	-2.734872717
H354	2.755333765	-13.118068302	1.617978895
H355	1.594721516	-12.195918921	-0.104140260
H356	4.490739926	-13.742884625	-0.428219159
H357	3.717476854	-11.625748928	1.662192148
H358	8.362167669	-5.493697283	3.695939735
H359	5.737334088	12.742175062	0.690105799
H360	3.772244774	-10.077289682	8.211414414
H361	4.665332998	-8.023925715	6.582394088
H362	3.853115632	12.543437376	-1.683613734
H363	3.100828521	11.188002229	0.977487744
H364	2.280169803	12.591560680	0.264562129
H365	-0.923555912	8.671654447	-3.773595655
H366	1.934541417	-2.213170844	1.232477234
H367	-0.895939403	-3.302029023	0.027745577
H368	0.482083587	-1.401677454	1.059797823
Mo369	0.370972240	-6.225855933	-0.904826217
N370	1.198437272	-1.942159828	0.573753706
N371	1.531749350	0.569398211	-0.838495249
N372	1.334552341	1.652493890	-0.623497661
N373	-9.870816834	-1.403085292	-0.049413332
N374	-9.761108964	2.132825316	1.171661442
N375	-9.536313138	2.269212427	3.973307529
N376	-7.951335557	0.038008267	4.602872411
N377	-5.464654735	0.872192400	3.500084155
N378	-4.930448602	3.066459624	5.265307896
N379	-5.015678870	2.004117747	7.987676259
N380	-3.986128388	-0.682051058	7.981224363
N381	-1.739672189	-0.507583916	6.274189247
N382	-0.088368364	2.061375408	6.431080437
N383	-0.395680030	2.945254288	9.192638591
N384	-2.843285623	-10.664338805	8.056939733
N385	-0.761538121	-9.821741386	6.585361739
N386	2.475296132	-5.064431992	4.231992698
N387	2.907540444	-3.193832250	5.570943328
N388	3.591010728	-3.211324030	3.362712527
N389	2.791972849	-9.854063860	6.403821842
N390	-6.441857474	0.467592310	-3.190416575
N391	-4.899749420	-3.932744288	-2.346742560
N392	-5.081554024	2.713529715	-2.940891720
N393	-6.185758076	5.226320729	-2.413696768
N394	-6.013274903	5.422445707	0.388900279
N395	-3.256711046	5.612830014	0.967949279
N396	-0.996529893	3.445842967	-1.811371480
N397	-1.876296423	1.696393369	-0.751468340
N398	-2.067536588	8.104884620	0.863787925
N399	-2.883371426	12.179793763	-1.444590940
N400	-1.779253531	13.808578616	-0.419454413
N401	9.045758281	-3.015506293	3.290175346
N402	9.347538183	1.685947952	-0.610144328
N403	8.524734365	3.327839834	-3.632676393
N404	6.040854299	4.291806684	-4.395117712
N405	1.461304441	5.707220591	-5.969142103
N406	2.356547964	6.915420342	-7.769782003
N407	1.560963424	8.025632587	-5.900737139
N408	4.311682586	4.191447934	-2.074837396
N409	5.689841827	5.952245215	-0.391475128
N410	6.218639086	8.459495544	-1.403894882
N411	4.839774537	8.342038926	-5.096185454
N412	4.954221826	10.902436830	-0.946341266
N413	0.320233247	-8.559554919	-8.444009219
N414	0.378045995	-5.328907669	-6.798007455
N415	1.249501982	-2.399376167	-6.935232127
N416	4.107760868	-2.159428147	-7.088516109
N417	-3.117689705	-3.831182617	-9.505025895
N418	-3.214753549	-1.096494691	-8.587550473
N419	2.003325021	-11.773209007	-3.202907739
N420	-0.112967783	-7.927051837	-2.500803428
N421	-1.140187934	-8.712527919	-4.286136240

N422	2.599498599	-11.992835094	-0.114163878
O423	-8.363383561	0.390450601	1.619578702
O424	-7.544020127	3.353575715	3.756296300
O425	-7.529308625	0.941633365	6.660035333
O426	-4.480327259	-0.308816943	5.173778778
O427	-2.722545592	2.674152708	4.875285963
O428	-3.249741615	3.407820920	8.369288682
O429	-3.281286467	-0.344311683	10.136720276
O430	-0.889533432	-2.413987265	7.235536826
O431	1.526726465	0.458005336	6.610499053
O432	1.869301825	3.042182073	9.181039418
O433	0.920445986	1.767918555	12.314195404
O434	-0.903909798	-11.587509796	5.103963990
O435	1.514766004	-9.426398638	8.267542865
O436	6.257597314	-9.189721091	7.098971827
O437	-5.237719983	2.732839369	-0.648254043
O438	-5.626169702	-2.213718735	-3.642913284
O439	-4.096412987	6.058237512	-2.026964545
O440	-3.066325604	5.182568212	-4.793211146
O441	-6.836751301	7.564818591	0.298660216
O442	-3.689895232	5.737805331	3.206963369
O443	-0.368859093	7.371571532	2.194991528
O444	-0.606435342	10.761600801	2.674647562
O445	8.426256278	-5.450407017	5.735690755
O446	5.589465716	2.449647737	3.548832433
O447	7.973166650	4.128313962	-1.568588749
O448	6.933845699	6.300623285	-5.064038705
O449	3.324593301	6.030953762	-3.005547223
O450	3.707542823	6.464559748	0.643990926
O451	5.278072419	3.280351283	0.761342913
O452	7.721780728	8.902284278	0.266853868
O453	6.514268509	12.016684478	-2.178289437
O454	4.273498038	10.205637741	-3.900584445
O455	4.562268998	14.305900895	0.106726860
O456	-0.162031705	8.194193167	-3.391400130
O457	-1.394498641	-6.664303745	-7.398680148
O458	-0.613826619	-2.533918491	-8.260409067
O459	3.883701038	-0.467723439	-8.587777220
O460	7.254466481	-2.182418163	-8.851258313
O461	-5.442802056	-1.255914260	-9.051419974
O462	-5.210006877	-5.701913430	-4.825875603
O463	-3.158036070	-6.323451579	-5.445417982
O464	-1.567115142	1.979687639	-9.390124798
O465	4.431461169	-11.022835379	-1.028753228
O466	5.583305617	-13.437552438	1.269067966
O467	-3.579919597	-5.323274570	-0.064900787
O468	-4.636694523	-5.255616673	1.917708491
O469	-2.629174409	-12.249516225	-0.278581283
O470	-4.410263826	-10.895402047	-0.600629306
O471	0.469967163	-7.845418654	0.599807259
O472	-0.591457931	-8.652085843	2.407365012
O473	-1.654728613	-6.748869492	-0.435470780
O474	3.025898985	-10.119242214	3.601996882
O475	2.796303604	-7.604010378	2.249242538
O476	-0.517387221	5.637823236	-3.896964625
O477	-0.305325052	-13.204530101	0.795989427
O478	0.576472185	-11.078529863	2.760351089
S479	5.827902564	1.153344515	-2.696659007
S480	2.689427008	-0.591500958	-3.338678747
S481	-0.450997807	-4.585964577	-2.365569410
S482	3.970817615	-0.874346113	0.002670220
S483	-0.344108899	-1.550573136	-1.542019853
S484	2.685224884	-4.336359924	-4.675401487
S485	0.783082475	-4.879249479	0.997361228
S486	5.478749276	-2.759500331	-2.627997803
S487	2.591880465	-6.541741750	-1.679230759
S488	4.557608696	-4.571582363	0.635810769
end			

2N2x-Fe2-brNH2-Fe6H to Fe2-brNH2-Fe6H

35, S=1

reactant

Fe(139) -2.442<br>Fe(140) -0.417<br>Fe(141) -2.544<br>Fe(142) 2.794	9651826nh26hx2n2xd35tc.car_3<br>C1 -9.381916192 0.814082164 1.049263895<br>C2 -10.365908248 -0.060979698 0.264303602<br>C3 -8.816344665 3.102042998 1.745092764<br>C4 -8.546718768 2.905797172 3.233720439

Fe(143) 2.958<br>Fe(144) 1.967<br>Fe(145) -0.662	C5 -9.265022352 1.922811890 5.350102873<br>C6 -8.135807918 0.910271283 5.576771931<br>C7 -6.755499059 -0.860809044 4.616449418<br>C8 -5.444187412 -0.090631142 4.440493894<br>C9 -4.279843866 1.714528368 3.269192780<br>C10 -3.881541359 2.522379198 4.512980765<br>C11 -4.568558179 3.845925949 6.419263582<br>C12 -4.141691903 3.025013651 7.644983436<br>C13 -4.735355538 1.211079690 9.188855630<br>C14 -3.849355883 -0.038590524 9.105979076<br>C15 -3.127451242 -1.934407307 7.725833261<br>C16 -1.785164107 -1.656448662 7.041794161<br>C17 -0.527459130 -0.219587155 5.508349625<br>C18 0.412556357 0.777903101 6.210156464<br>C19 -0.829641627 0.131994412 4.032450007<br>C20 0.446231856 0.533796305 3.281083116<br>C21 -1.517440379 -1.045153163 3.329631070<br>C22 0.825407426 3.053850594 7.011971492<br>C23 0.868286195 2.994949911 8.550596371<br>C24 0.510175978 4.460871579 6.449827920<br>C25 1.409553883 5.519401314 7.099675678<br>C26 0.682995963 4.475755978 4.925059285<br>C27 -0.301775725 2.856986186 10.667445116<br>C28 0.553250959 1.707801913 11.171447824<br>C29 -2.608230241 -11.352007194 6.796928600<br>C30 -1.337467940 -10.895155807 6.084998670<br>C31 0.421344603 -9.148676593 6.090246172<br>C32 1.591470649 -9.460797021 7.041799497<br>C33 0.253026893 -7.623677462 5.944040569<br>C34 1.517352559 -6.977328167 5.380678513<br>C35 1.336221224 -5.506309347 5.042428978<br>C36 3.048606272 -3.802025373 4.408213930<br>C37 3.964372786 -10.051224010 7.167544519<br>C38 5.031068535 -9.007143920 6.931786910<br>C39 -5.847161237 0.688824166 -1.846327954<br>C40 -5.354447254 2.143507295 -1.717614226<br>C41 -4.673364310 -0.278085131 -1.588565628<br>C42 -5.081622590 -1.738209477 -1.307218777<br>C43 -5.222763285 -2.638901318 -2.535076768<br>C44 -4.238317442 3.902186418 -3.072695178<br>C45 -4.842549855 5.164372848 -2.449762197<br>C46 -3.953818207 4.077615455 -4.571368811<br>C47 -6.882367142 6.312612912 -1.735331838<br>C48 -6.577604874 6.487637552 -0.242941489<br>C49 -5.608367913 5.531350513 1.791632522<br>C50 -4.102454065 5.639912424 2.046554928<br>C51 -1.825785609 5.684620062 1.148750051<br>C52 -1.372598732 7.116901720 1.476786594<br>C53 -1.059947256 5.262629443 -0.116210750<br>C54 -1.319319248 3.861291969 -0.558852197<br>C55 -1.883560334 2.761655561 0.053057378<br>C56 -1.377395440 2.116574535 -1.933159097<br>C57 -1.657875680 9.474759708 0.847492828<br>C58 -1.464263143 9.918310541 2.295147549<br>C59 -2.784902653 10.293180107 0.173801505<br>C60 -2.452699538 11.693833967 -0.249061574<br>C61 -1.754895845 12.695664527 0.396997587<br>C62 -2.510778051 13.420341413 -1.555991782<br>C63 8.186058184 -3.401491713 4.486524658<br>C64 8.284277541 -4.902259935 4.693312883<br>C65 6.694267002 -3.086274418 4.229186203<br>C66 6.406683355 -1.610427578 4.075926244<br>C67 6.058561754 -0.815839455 5.183622737<br>C68 6.467042407 -0.989368042 2.814498523<br>C69 5.770508262 0.545230667 5.042022879<br>C70 6.195025686 0.370280829 2.659102229<br>C71 5.842312643 1.135987350 3.773459551<br>C72 8.414712490 1.745677997 -1.718268036<br>C73 8.279014332 3.172305427 -2.272677657<br>C74 7.053519412 1.235309890 -1.242992190<br>C75 8.468311547 4.611556705 -4.271202602<br>C76 7.069841507 5.137286963 -4.603381816<br>C77 4.632434978 4.641424712 -4.491039206<br>C78 4.025126839 5.026655143 -3.117267344<br>C79 3.915388581 3.437611728 -5.144390636<br>C80 2.391663308 3.474820413 -5.306884253<br>C81 1.837385089 4.358738094 -6.435204143<br>C82 1.782050735 6.851204481 -6.564710923<br>C83 3.749611942 4.519890024 -0.742494033<br>C84 4.374649934 5.768844997 -0.106522590

	C85	3.905688635	3.355189791	0.258527925
	C86	6.504853847	6.850973333	0.423457154
	C87	6.880388357	8.161260634	-0.244584957
	C88	6.644129125	9.593935677	-2.194056140
	C89	6.003279300	10.944569029	-1.792187522
	C90	6.536787990	9.377248492	-3.708138264
	C91	5.106207927	9.329831700	-4.229640131
	C92	4.109727791	12.075328054	-0.730349123
	C93	4.864681334	13.123410310	0.070224963
	C94	2.809112315	11.663784042	0.016057852
	C95	1.952474098	10.743511304	-0.821868812
	C96	1.038093592	11.268797463	-1.746950752
	C97	2.088001787	9.347145877	-0.747556066
	C98	0.295987837	10.436698805	-2.587623532
	C99	1.374742778	8.499164332	-1.600647976
	C100	0.488196806	9.055069040	-2.518956797
	C101	0.825088608	-7.630683337	-7.447273137
	C102	-0.146908410	-6.483943919	-7.222504199
	C103	-0.342523379	-4.137066449	-6.425713557
	C104	0.089039075	-2.936749928	-7.276347521
	C105	1.873769854	-1.290666340	-7.646142839
	C106	3.389927453	-1.285780637	-7.820999996
	C107	5.598661662	-2.014282761	-7.120376520
	C108	6.156058329	-2.478064519	-8.451870006
	C109	-4.256162778	-3.312606849	-8.697161317
	C110	-4.377135704	-1.785766044	-8.783704048
	C111	-4.301758064	-3.775104528	-7.223085159
	C112	-4.578414821	-5.288499355	-7.130140831
	C113	-4.361407173	-5.795126300	-5.722677795
	C114	-3.249148137	0.376969464	-8.645684276
	C115	-2.028987195	0.871777586	-9.396227099
	C116	-3.530959753	1.118906589	-7.315366411
	C117	-2.574685876	0.647052523	-6.258395029
	C118	-1.293848676	1.210920077	-6.150061884
	C119	-2.887038328	-0.490572129	-5.496201432
	C120	-0.318633813	0.592765582	-5.363113918
	C121	-1.911792960	-1.104167765	-4.708961751
	C122	-0.613885859	-0.583553988	-4.670148531
	C123	2.361917373	-10.681805920	-2.202251601
	C124	3.216073445	-11.208906748	-1.038142108
	C125	1.086044867	-10.010364363	-1.674818337
	C126	0.261802267	-9.262097679	-2.673082368
	C127	-0.375371181	-9.766614942	-3.788079668
	C128	-1.000108378	-7.665332536	-3.480962072
	C129	3.378908483	-12.456175831	1.030275802
	C130	4.587697260	-13.248329538	0.579804371
	C131	-3.721366651	-5.645209444	1.240196560
	C132	-2.577753862	-6.498927870	1.780523047
	C133	-1.943508746	-7.460367529	0.732606027
	C134	-2.907223601	-8.649728709	0.457873183
	C135	-2.303854229	-9.819663683	-0.325222426
	C136	-3.219975959	-11.059219552	-0.429028985
	C137	-0.632415259	-8.031987020	1.293045505
	C138	2.415711903	-3.251169764	-1.519256734
	Fe139	1.809389453	-4.747218772	-2.717239768
	Fe140	1.788979931	-1.244864626	-1.146291682
	Fe141	4.208336420	-2.926392180	-0.761870005
	Fe142	3.302307766	-2.716896485	-3.185502025
	Fe143	4.595968380	-0.657604232	-2.009927613
	Fe144	2.577414611	-4.808559286	-0.316003409
	Fe145	0.457000844	-3.428066729	-0.655382205
	H146	-9.579881313	-1.875479527	0.765845050
	H147	-9.099454119	-1.342926420	-0.699151988
	H148	-10.508565431	2.476041492	0.606545806
	H149	-10.660974300	0.471139464	-0.653982408
	H150	-11.279949899	-0.135245678	0.878762105
	H151	-7.836635289	3.076587421	1.244602218
	H152	-10.329811706	1.926199784	3.479559588
	H153	-9.242654127	4.103983552	1.599826821
	H154	-10.195249103	1.515003482	5.767941552
	H155	-8.402716597	0.122966180	3.688674043
	H156	-8.998694347	2.821724278	5.920995747
	H157	-3.406260509	1.116996145	2.981249760
	H158	-6.249271632	0.958745182	2.866271783
	H159	-6.855969920	-1.606027688	3.815512346
	H160	-3.742606728	4.532723545	6.192797411
	H161	-5.845912010	3.033692619	4.881845513
	H162	-6.702252115	-1.387735425	5.576844354
	H163	-4.508206618	2.398335710	2.439892278
	H164	-5.748790627	1.752043302	7.379882045

	H165	-5.452818089	4.438949143	6.690215352
	H166	-2.443803039	-1.347289553	3.838109097
	H167	-1.778060086	-0.774618226	2.293967691
	H168	-0.849378727	-1.921428226	3.289456733
	H169	-1.505172410	1.001270901	4.030206322
	H170	-5.720402462	0.897601750	9.564849617
	H171	0.019936710	3.757190876	4.424425561
	H172	-1.212772236	2.941870190	8.727669439
	H173	1.252162169	5.590964376	8.185587256
	H174	0.201151330	0.791048885	2.239938652
	H175	1.177117929	-0.290195857	3.265659650
	H176	8.481272147	-2.917166393	5.436978939
	H177	0.463335312	5.475001982	4.521502940
	H178	1.209703689	6.506456349	6.656748217
	H179	1.722863896	4.224493508	4.656550839
	H180	2.471784180	5.278720607	6.929766618
	H181	-0.541809703	4.700365071	6.690553377
	H182	1.849373853	2.784022135	6.713175260
	H183	-0.977837087	2.304121105	6.088025161
	H184	0.934007843	1.411598251	3.729654878
	H185	3.647198766	2.412526156	-0.248500798
	H186	3.178150768	3.524963287	1.066219656
	H187	5.188025453	3.073445062	1.731078940
	H188	5.246319629	2.875642614	4.391803742
	H189	6.215848949	0.833455470	1.672388610
	H190	6.695928136	-1.585871319	1.928791974
	H191	6.000326143	-1.269243989	6.176182996
	H192	-2.587091441	-0.034769756	6.062202977
	H193	-3.678538577	-2.652443846	7.100389586
	H194	-2.902816176	-2.419224570	8.683347087
	H195	-4.506790775	-0.422326232	7.176857000
	H196	-4.272655542	1.870049814	9.933584287
	H197	5.479939041	1.142340161	5.910516287
	H198	0.065572427	-1.146389815	5.515097940
	H199	0.050791042	3.792370030	11.130672240
	H200	-1.332533161	2.673728706	11.007362105
	H201	0.668404843	0.858261872	10.456803134
	H202	4.379216643	-11.050104789	6.960443266
	H203	-5.032199801	-4.588621800	-3.129867662
	H204	-2.702769475	-5.798498285	-0.395144906
	H205	-5.626362567	-5.478040365	-7.404372954
	H206	-3.933102145	-5.835413774	-7.833010293
	H207	-5.090946284	-3.235858729	-6.676315318
	H208	-1.549878009	0.110574593	-10.055154615
	H209	-5.177426701	-3.671214627	-9.182283894
	H210	-3.989856267	-0.239264298	-2.450790831
	H211	-6.048781247	-1.769209747	-0.774286271
	H212	-2.363392043	-1.551170736	-8.328660911
	H213	-4.090905064	0.614257835	-9.328860116
	H214	-2.230105826	-3.606215935	-9.038047662
	H215	-4.561064864	-4.299462051	-1.457201309
	H216	-3.350018045	-3.515348600	-6.734532314
	H217	-3.192581875	-4.808265005	-9.618790133
	H218	-7.428890793	0.645269183	-3.181839790
	H219	-4.092258160	0.098642525	-0.736388207
	H220	-2.557836817	-6.351685064	-6.267751347
	H221	-4.348977461	-2.191589582	-0.623392597
	H222	-1.531174425	5.060701779	2.004089471
	H223	-2.275974952	2.693409118	1.062828036
	H224	-6.066938045	6.437337516	2.211780679
	H225	-5.821925900	4.553077036	-0.099810103
	H226	-2.889465972	7.816761553	0.284100991
	H227	-1.225221001	12.724273567	1.342743611
	H228	-2.198858111	9.464903044	3.003153654
	H229	-3.668719819	10.283618996	0.836087827
	H230	-0.707036899	9.610798457	0.307994342
	H231	-2.696022281	14.100552632	-2.384061891
	H232	0.011911951	5.410953723	0.100033080
	H233	-1.315088183	5.959692109	-0.933676145
	H234	-1.369252422	14.687718989	-0.280097593
	H235	-3.080803774	9.756036309	-0.740828988
	H236	-3.650646381	5.492368597	0.024118458
	H237	-5.984667162	4.682306351	2.382628145
	H238	-6.629815392	7.270310326	-2.211377487
	H239	-1.224981365	1.526470592	-2.832615697
	H240	-7.963483966	6.158323354	-1.846106037
	H241	9.995904586	-3.048547015	3.598068297
	H242	8.809533757	-2.052583221	3.079071763
	H243	4.771906959	3.296645844	-2.201174984
	H244	2.676228461	4.731817695	-0.851115926

H245	6.199309244	5.198526387	-0.855853651
H246	-0.617047770	4.034979311	-2.572408814
H247	-1.448623761	5.479122713	-4.189857364
H248	-3.066235011	5.385761977	-5.730436091
H249	-0.489404841	6.511395847	-3.470214319
H250	-3.487696943	3.148353478	-4.939482565
H251	-4.899853739	4.243347251	-5.110666643
H252	7.105866308	8.481890619	-3.992474409
H253	7.018970603	10.245448412	-4.183853036
H254	5.497309166	7.532790110	-5.178603723
H255	3.861907433	8.181941661	-5.327718758
H256	2.823384356	7.756186802	-8.083138278
H257	0.941371615	3.891010642	-6.872038019
H258	-0.424160763	10.863362005	-3.289638992
H259	2.748251082	8.887884259	-0.005947601
H260	1.062467964	8.002543840	-5.019163391
H261	4.154773514	2.546697510	-4.541698689
H262	6.264431922	3.361788193	-3.995214452
H263	1.893801600	3.725540108	-4.357040640
H264	-6.719952895	4.401450772	-2.703216405
H265	9.037633444	4.556044644	-5.208314990
H266	-5.276724995	2.078985926	-3.704056337
H267	4.382769433	3.274059290	-6.129902086
H268	1.535964106	7.420296603	-1.565092156
H269	8.941235881	5.375032284	-3.636316320
H270	2.547225550	6.060054061	-8.306896612
H271	1.438109589	8.861413295	-6.471558113
H272	-6.247232339	-0.504840514	-3.474705913
H273	-6.579593596	0.556290141	-1.027384289
H274	0.891849284	12.350142013	-1.808464502
H275	-3.283496445	3.755355409	-2.547478930
H276	4.554516485	5.535948563	-5.117103749
H277	2.580580126	4.435016025	-7.239910883
H278	2.092716308	2.444045828	-5.538724630
H279	0.801479659	5.731690178	-5.171441647
H280	5.956094035	7.098079921	1.344621029
H281	5.616067099	7.732778776	-1.800558722
H282	7.710280855	9.743127887	-1.963838875
H283	7.440992970	6.351526828	0.709611949
H284	4.518680888	9.980238675	-0.799403010
H285	1.800606702	-7.232369796	-7.763388464
H286	0.976449070	-8.095710215	-6.451149144
H287	2.988903446	-9.926250503	-2.698967977
H288	0.809172741	-9.437198474	-8.393142085
H289	-0.671694134	-8.747933435	-8.252139304
H290	7.190470007	0.222656158	-0.845271071
H291	9.085888062	2.399155558	0.096834890
H292	8.762017572	2.500117312	-4.154860633
H293	6.664827223	1.890513789	-0.453222956
H294	10.293994405	1.965952015	-0.917479880
H295	6.018936969	-2.668545144	-6.341090088
H296	8.789857526	1.076229724	-2.508609434
H297	-0.008684581	-7.189999869	6.923222681
H298	0.623066632	-9.575777300	5.099043991
H299	3.739666875	-2.720264272	-6.339934114
H300	4.243569650	-2.408570859	3.537458064
H301	1.427031965	-1.251690248	-8.647034557
H302	-2.156718370	-1.989441740	-4.119193992
H303	1.612538234	-0.343223719	-7.144070384
H304	1.207555649	-4.904894878	5.950166669
H305	1.716430229	-2.769520784	-6.068037188
H306	0.431506712	-5.352936360	4.430212398
H307	-0.593908912	-7.450304541	5.261534300
H308	1.812253030	-7.503465143	4.462031161
H309	2.354327818	-7.075037712	6.088776061
H310	-4.574398178	0.915146920	-7.031963142
H311	-1.268007998	-9.394548194	7.409900690
H312	-3.439969611	-11.132146301	6.108869567
H313	0.155769161	-1.072877921	-4.069706829
H314	-2.391112684	-11.173100280	8.836514638
H315	-3.840669063	-10.646404844	8.285672898
H316	2.644992504	-5.502823219	3.384882689
H317	0.683526584	1.015455199	-5.282214611
H318	-3.894783665	-0.909817110	-5.530661816
H319	-0.126100356	-12.546906026	1.514539696
H320	0.438977912	-10.779620498	-1.219298395
H321	0.161615248	-10.194164966	2.512426396
H322	-1.170958212	-12.781710289	0.387166477
H323	5.463132277	-3.103804132	-9.063808369
H324	1.943610960	-7.746622332	1.671998114

H325	-2.927909924	-7.053979817	2.658685002
H326	1.396802642	-5.331927147	-6.525167026
H327	-3.245076266	-9.017675325	1.439521277
H328	-1.806551858	-8.806626402	-5.060186008
H329	-1.531797517	-6.735037438	-3.631662051
H330	-2.545946179	-12.451417959	6.873619759
H331	3.732656360	-3.668152619	2.450771677
H332	3.021298213	-9.249940969	3.133601342
H333	3.430672513	-7.310772778	1.609957236
H334	2.781975639	-9.907697014	5.383488331
H335	2.609880557	-3.603895558	6.408893422
H336	-2.074877407	-9.496179349	-1.351811052
H337	-1.355611054	-10.136313582	0.130678852
H338	-3.796661489	-8.266871257	-0.065286725
H339	-0.197395380	-3.921928282	-5.355478633
H340	-1.053156160	2.107518758	-6.725581865
H341	-3.431688448	2.196859612	-7.514797219
H342	-1.806672177	-5.788170322	2.122142942
H343	2.149532317	-10.532027004	3.287250149
H344	0.072736543	-11.291244781	3.599221495
H345	3.111533306	-2.153650118	5.634808666
H346	-0.354803940	-10.753895728	-4.233574515
H347	1.353118962	-9.325696558	-0.860990909
H348	-1.400750970	-4.326961599	-6.616030438
H349	5.952940532	-0.990839748	-6.922313498
H350	6.117528402	-3.490780483	5.074877337
H351	6.388406275	-3.627777434	3.319686171
H352	2.940068801	-12.133742299	-3.531083121
H353	1.549754107	-12.496336404	-2.747250253
H354	2.738497437	-13.140261532	1.609671552
H355	1.595185953	-12.163144913	-0.095613651
H356	4.485735417	-13.716661548	-0.430123105
H357	3.697006097	-11.646925964	1.706228802
H358	8.319672687	-5.495753673	3.748270231
H359	5.717105749	12.740002429	0.677527337
H360	3.708644915	-10.014931329	8.242393345
H361	4.658150398	-8.017013280	6.573240989
H362	3.833022453	12.533492820	-1.695061438
H363	3.081130813	11.182634883	0.968103795
H364	2.262454110	12.586425905	0.254021158
H365	-0.936190415	8.664761340	-3.789813373
H366	1.765641579	-2.208683074	1.248753956
H367	-0.950670070	-3.379552780	-0.120608906
H368	0.303314374	-1.449723375	0.967619081
Mo369	0.359879126	-6.223788174	-0.930737902
N370	1.067119370	-1.975142738	0.539589477
N371	1.442291490	0.526889630	-0.747296243
N372	1.233612005	1.574693780	-0.406908571
N373	-9.909446226	-1.397260690	-0.076605772
N374	-9.723901016	2.130363332	1.153773531
N375	-9.491393401	2.254162671	3.953145600
N376	-7.919898766	0.012336288	4.583047902
N377	-5.438252637	0.856356255	3.474059396
N378	-4.890910006	3.048862333	5.247102231
N379	-4.926160649	1.957574983	7.953021601
N380	-3.961067528	-0.771960009	7.966980560
N381	-1.722334212	-0.527634837	6.279147745
N382	-0.058516298	2.034383643	6.446071219
N383	-0.313663527	2.924905838	9.215146788
N384	-2.841770185	-10.663443552	8.073233716
N385	-0.800693591	-9.760063189	6.575340345
N386	2.491985410	-5.013851038	4.272687847
N387	3.040986140	-3.167020810	5.602402447
N388	3.595914587	-3.175454150	3.359070620
N389	2.747060755	-9.811737715	6.423028086
N390	-6.420884033	0.473863147	-3.183051233
N391	-4.936290354	-3.941401175	-2.341300987
N392	-5.067721141	2.716285534	-2.919728387
N393	-6.196037838	5.218333372	-2.396468943
N394	-6.030065951	5.423997881	0.406897163
N395	-3.270680410	5.592198164	0.967688388
N396	-0.997501653	3.430763266	-1.833883171
N397	-1.917321409	1.686167584	-0.803768922
N398	-2.077592008	8.083954225	0.836494159
N399	-2.921132638	12.161114608	-1.467683737
N400	-1.798859837	13.782455894	-0.450834106
N401	9.007543839	-3.024166224	3.334584872
N402	9.362720312	1.691120198	-0.591612803
N403	8.523876541	3.322468930	-3.607416716
N404	6.045721734	4.290337571	-4.375470042

N405	1.468302639	5.701414643	-5.973643910
N406	2.367547083	6.903447206	-7.775223081
N407	1.560595256	8.019938197	-5.914130260
N408	4.304734993	4.210498906	-2.061147143
N409	5.698247218	5.954615492	-0.381974759
N410	6.216364183	8.455842565	-1.403182620
N411	4.847261902	8.326443128	-5.110799673
N412	4.936648390	10.895228591	-0.955058550
N413	0.310013467	-8.547904308	-8.467011818
N414	0.405935807	-5.334246837	-6.785502999
N415	1.288146924	-2.415636925	-6.939993098
N416	4.149704641	-2.106694006	-7.051113996
N417	-3.126060676	-3.794862667	-9.502950216
N418	-3.239609889	-1.070714599	-8.556270000
N419	2.061261545	-11.729431526	-3.194751506
N420	-0.134160513	-7.934994597	-2.503317968
N421	-1.158780474	-8.745998842	-4.278992459
N422	2.600241457	-11.969157831	-0.093325776
O423	-8.363283158	0.360055872	1.602083694
O424	-7.496356479	3.334473067	3.742161779
O425	-7.483467203	0.923262627	6.634348939
O426	-4.454154106	-0.308947904	5.160694883
O427	-2.688553005	2.675446497	4.819274784
O428	-3.167879620	3.373070107	8.334462788
O429	-3.130792775	-0.360553874	10.062523078
O430	-0.845170330	-2.452385168	7.183058357
O431	1.563452950	0.427498494	6.520237337
O432	1.950567095	3.006764412	9.164084996
O433	1.033465456	1.669507097	12.294521239
O434	-0.894796464	-11.551382595	5.119955885
O435	1.462833833	-9.344384286	8.272617555
O436	6.219872324	-9.202978214	7.130010390
O437	-5.235074820	2.732183334	-0.627050135
O438	-5.607076148	-2.206145138	-3.646179413
O439	-4.114825473	6.069810618	-2.007003077
O440	-3.074482213	5.200784862	-4.774315528
O441	-6.857977757	7.564297137	0.304784682
O442	-3.688314930	5.762643304	3.207131631
O443	-0.390853558	7.359042695	2.188075912
O444	-0.643932899	10.743006916	2.663507345
O445	8.299167297	-5.444253428	5.789335635
O446	5.556576809	2.469617574	3.561165312
O447	7.975882506	4.135000919	-1.547120885
O448	6.946131851	6.290463069	-5.063165189
O449	3.325251179	6.045352366	-3.011716526
O450	3.722868740	6.500934093	0.648483356
O451	5.235359064	3.290088251	0.778577949
O452	7.726674933	8.911040323	0.258233992
O453	6.490191398	12.011681272	-2.192067440
O454	4.263325614	10.180311247	-3.908038731
O455	4.540957246	14.300622695	0.090552223
O456	-0.174236486	8.189836747	-3.406062914
O457	-1.378390949	-6.642639633	-7.407507365
O458	-0.613344523	-2.508579950	-8.216513371
O459	3.900253972	-0.498825327	-8.636349252
O460	7.292179267	-2.229253214	-8.823169156
O461	-5.463768091	-1.236966408	-9.035055945
O462	-5.203401977	-5.710037460	-4.835732620
O463	-3.148659082	-6.322802066	-5.453609537
O464	-1.611459808	2.020285807	-9.350144912
O465	4.442991477	-10.994727655	-0.980243350
O466	5.575855537	-13.438876299	1.275446776
O467	-3.589923015	-5.274900607	-0.038048953
O468	-4.666995032	-5.278040298	1.935898536
O469	-2.661311266	-12.206972661	-0.302938654
O470	-4.445632323	-10.865106928	-0.664488300
O471	0.443904469	-7.837609318	0.577054345
O472	-0.634940085	-8.666293702	2.366537300
O473	-1.668934851	-6.703840976	-0.448962500
O474	2.987432831	-10.107900151	3.623357851
O475	2.749120837	-7.617386681	2.238121316
O476	-0.512224669	5.629482821	-3.903307348
O477	-0.356299427	-13.192577457	0.808997281
O478	0.552290463	-11.075093272	2.759292204
S479	5.802532907	1.186597741	-2.609121747
S480	2.675093870	-0.550069150	-3.291540433
S481	-0.407876411	-4.613055446	-2.443156187
S482	3.900383175	-0.881341202	0.087535185
S483	-0.372931695	-1.592683171	-1.625479128
S484	2.763461001	-4.298302378	-4.667745882

	S485 0.759728036 -4.871310952 0.980426257<br>S486 5.489748580 -2.685238018 -2.553178491<br>S487 2.598648221 -6.563608481 -1.665802530<br>S488 4.530830885 -4.585104555 0.661627882<br>end

TS

Fe(139) -2.424<br>Fe(140) -0.730<br>Fe(141) -2.577<br>Fe(142) 2.733<br>Fe(143) 3.087<br>Fe(144) 2.007<br>Fe(145) -0.515	9651826nh26hx2n2xd35td_1_53215.82

```
C1 -9.371100354 0.809573870 1.038458942
C2 -10.375743422 -0.053952925 0.266304894
C3 -8.794044954 3.095698143 1.739736582
C4 -8.527382581 2.900262268 3.228841258
C5 -9.245381207 1.917158220 5.344223657
C6 -8.117884896 0.901732772 5.566492573
C7 -6.743673166 -0.868846702 4.597714672
C8 -5.435266758 -0.092744698 4.428034858
C9 -4.274913739 1.713766878 3.249188937
C10 -3.868139277 2.523332050 4.488753440
C11 -4.533376065 3.834946988 6.408107958
C12 -4.099923376 3.008942708 7.628181912
C13 -4.692322294 1.188616825 9.166916675
C14 -3.810939771 -0.064548356 9.087937098
C15 -3.098587307 -1.969955075 7.718846084
C16 -1.765550543 -1.676608415 7.024212111
C17 -0.533750525 -0.219589429 5.491911296
C18 0.416917503 0.767890247 6.193056843
C19 -0.849422151 0.139260538 4.021411495
C20 0.418533054 0.546120607 3.260245783
C21 -1.541084079 -1.034673393 3.316864058
C22 0.846424069 3.034676782 7.011279341
C23 0.898980009 2.980221146 8.550348296
C24 0.539361489 4.439323246 6.439699384
C25 1.435379949 5.503016367 7.084195841
C26 0.720964160 4.439357911 4.915712751
C27 -0.252789284 2.837153586 10.675953440
C28 0.588446249 1.669281882 11.160351028
C29 -2.594391485 -11.351989387 6.801311842
C30 -1.329623890 -10.883532230 6.088595964
C31 0.407028357 -9.112986244 6.088190224
C32 1.572838211 -9.421745979 7.044948423
C33 0.230490042 -7.590783560 5.945193375
C34 1.497550273 -6.942905531 5.391105153
C35 1.336259917 -5.462025876 5.088889799
C36 3.074089395 -3.786148181 4.445224811
C37 3.937337256 -10.038141698 7.185170035
C38 5.023543777 -9.015441138 6.941220795
C39 -5.833281657 0.688335317 -1.839953966
C40 -5.340769679 2.143442120 -1.707701661
C41 -4.667783164 -0.284763373 -1.573882218
C42 -5.088748534 -1.742749285 -1.297236825
C43 -5.229505959 -2.641680333 -2.526357851
C44 -4.233749502 3.908188722 -3.062363615
C45 -4.843917026 5.168151830 -2.441255987
C46 -3.947757280 4.083814363 -4.560653045
C47 -6.889078386 6.310215488 -1.732185202
C48 -6.585154431 6.489532970 -0.240180055
C49 -5.617211870 5.537864829 1.798071325
C50 -4.111731874 5.645016258 2.053153458
C51 -1.834726716 5.676074584 1.150202669
C52 -1.384065242 7.108439371 1.481407367
C53 -1.079180770 5.262207968 -0.123995009
C54 -1.337446362 3.862234598 -0.568550631
C55 -1.890020146 2.758543241 0.048661777
C56 -1.383814917 2.111515394 -1.937407994
C57 -1.659676311 9.463289496 0.837116131
C58 -1.471931387 9.911046424 2.284548000
C59 -2.786010623 10.280674330 0.159642876
C60 -2.456438853 11.682590931 -0.261403089
C61 -1.754809966 12.683046656 0.382836039
C62 -2.524120309 13.411941087 -1.563475999
C63 8.166478725 -3.402337751 4.509821942
C64 8.247523728 -4.904260168 4.718491072
C65 6.679535350 -3.074906411 4.250993954
C66 6.399078074 -1.597590591 4.095431803
C67 6.051193651 -0.800307456 5.201721315
C68 6.459364122 -0.979711619 2.832139332
C69 5.761721847 0.560261916 5.055838087
C70 6.185209133 0.378971641 2.673169783
C71 5.830038642 1.146929124 3.785276510
C72 8.420356330 1.744177435 -1.699180729
```

C73	8.282565454	3.170169314	-2.257542080
C74	7.059096795	1.239033404	-1.226355644
C75	8.468349904	4.600782196	-4.263587314
C76	7.070635634	5.127703328	-4.595617540
C77	4.632227035	4.639848499	-4.486761423
C78	4.019893400	5.032031413	-3.118017240
C79	3.915662089	3.434445944	-5.136710382
C80	2.392146198	3.470401192	-5.301936396
C81	1.839547408	4.352938054	-6.432511235
C82	1.783723071	6.845473033	-6.564042580
C83	3.738069460	4.538276825	-0.739600979
C84	4.370102477	5.783542588	-0.105024556
C85	3.888158519	3.378508351	0.265086209
C86	6.507641855	6.847973282	0.424315023
C87	6.879128886	8.160356705	-0.243623969
C88	6.639620314	9.590843392	-2.193910162
C89	5.994452685	10.940630494	-1.793286032
C90	6.532970285	9.371355474	-3.707510967
C91	5.102632981	9.318667353	-4.229486330
C92	4.101693260	12.068911528	-0.734073275
C93	4.857879946	13.119085921	0.064987192
C94	2.800680238	11.662190612	0.012304771
C95	1.942811999	10.741438735	-0.825099490
C96	1.028943709	11.267304351	-1.750285787
C97	2.078890803	9.345153856	-0.750711166
C98	0.288006998	10.435309764	-2.591905738
C99	1.367252259	8.497538301	-1.604517586
C100	0.481513032	9.053808813	-2.522979806
C101	0.823977346	-7.634482203	-7.453218276
C102	-0.145679996	-6.485313139	-7.227582955
C103	-0.334745634	-4.139947482	-6.424095561
C104	0.097359130	-2.937754746	-7.271989927
C105	1.885117827	-1.292474404	-7.637147114
C106	3.400252399	-1.282431885	-7.815729078
C107	5.615711723	-1.992548930	-7.115695123
C108	6.174177357	-2.483380379	-8.436207578
C109	-4.259595796	-3.302932971	-8.689960081
C110	-4.380930617	-1.776373834	-8.766772842
C111	-4.307017582	-3.771906807	-7.217766273
C112	-4.578621526	-5.286302954	-7.130349131
C113	-4.358750019	-5.796069041	-5.724198982
C114	-3.258301525	0.386923384	-8.626297779
C115	-2.043197995	0.888706083	-9.379919246
C116	-3.549057218	1.128542823	-7.298140208
C117	-2.591631171	0.656286160	-6.244194679
C118	-1.309462861	1.218632037	-6.143749398
C119	-2.897755028	-0.489730030	-5.491705080
C120	-0.322814558	0.582996456	-5.386220672
C121	-1.910094733	-1.121033849	-4.734957824
C122	-0.609516882	-0.607420122	-4.715728222
C123	2.361527000	-10.670218273	-2.192952841
C124	3.211584786	-11.201075461	-1.027610935
C125	1.074269448	-10.014146637	-1.673897812
C126	0.253345553	-9.269113238	-2.676944147
C127	-0.377129822	-9.775283703	-3.795053149
C128	-0.999557341	-7.672091635	-3.496044896
C129	3.370295489	-12.464333278	1.032451274
C130	4.581697807	-13.247744431	0.577866880
C131	-3.729135219	-5.646325668	1.213718679
C132	-2.603296422	-6.517551611	1.766478021
C133	-1.962680734	-7.481155774	0.724298457
C134	-2.920482672	-8.674738251	0.449463211
C135	-2.312745659	-9.839414265	-0.337451631
C136	-3.226157031	-11.080967014	-0.445668589
C137	-0.650871613	-8.045895131	1.289460818
C138	2.426303804	-3.259530291	-1.524985335
Fe139	1.787711842	-4.737258917	-2.714366561
Fe140	1.828673948	-1.426491641	-1.242523114
Fe141	4.213010544	-2.912704816	-0.744745706
Fe142	3.327714593	-2.731094595	-3.182463107
Fe143	4.587618836	-0.652139008	-2.058775772
Fe144	2.565422232	-4.820099506	-0.303465126
Fe145	0.395017166	-3.454648507	-0.661849723
H146	-9.603579904	-1.881532342	0.743586397
H147	-9.142930452	-1.345307736	-0.727658149
H148	-10.495304154	2.477614591	0.611058246
H149	-10.682608124	0.486161044	-0.643362815
H150	-11.278838363	-0.121255729	0.897983852
H151	-7.814101746	3.065712597	1.240194268
H152	-10.312287361	1.923085031	3.474595864

	H153	-9.215437054	4.099445608	1.593218920
	H154	-10.175683872	1.512325600	5.764589239
	H155	-8.396717963	0.114288902	3.680326951
	H156	-8.974628572	2.814969793	5.914636146
	H157	-3.402208758	1.117863157	2.955322261
	H158	-6.239634708	0.947654092	2.848405158
	H159	-6.841030950	-1.607888544	3.790635148
	H160	-3.705792851	4.517620670	6.175108826
	H161	-5.830116764	3.025288613	4.883739561
	H162	-6.688559448	-1.403659880	5.553375808
	H163	-4.509801056	2.396536586	2.420770452
	H164	-5.697937330	1.728969257	7.353798599
	H165	-5.411272006	4.432509305	6.689920970
	H166	-2.466926503	-1.336434685	3.826446918
	H167	-1.802925389	-0.760508096	2.282258564
	H168	-0.874694977	-1.911879139	3.270009924
	H169	-1.524404454	1.008086425	4.028284654
	H170	-5.679497095	0.879654724	9.540777177
	H171	0.067357626	3.708833313	4.419433249
	H172	-1.180243794	2.931582168	8.742837238
	H173	1.284735963	5.572621293	8.171032854
	H174	0.158517402	0.806093397	2.223575361
	H175	1.151837252	-0.275277239	3.232396246
	H176	8.464344222	-2.921073646	5.461498851
	H177	0.494908068	5.431791505	4.498704544
	H178	1.224906430	6.488762622	6.643411529
	H179	1.764781856	4.193844083	4.656385460
	H180	2.498021497	5.270788130	6.906658400
	H181	-0.513813042	4.682254636	6.671319325
	H182	1.866533176	2.753904095	6.709656765
	H183	-0.968611232	2.298826373	6.105005395
	H184	0.908836952	1.423670306	3.706137149
	H185	3.634737807	2.434196628	-0.238526046
	H186	3.157056952	3.555337270	1.068126074
	H187	5.157835553	3.070209417	1.739143663
	H188	5.247715961	2.893597099	4.400652339
	H189	6.206040966	0.838882945	1.685197673
	H190	6.691664035	-1.578905919	1.948865280
	H191	5.992116097	-1.250639936	6.196258968
	H192	-2.588897315	-0.051854177	6.066227583
	H193	-3.645587448	-2.696434599	7.099698100
	H194	-2.859619252	-2.448967688	8.675803131
	H195	-4.493448153	-0.470947849	7.172191846
	H196	-4.228070124	1.846596342	9.911321248
	H197	5.467874727	1.158955447	5.922290984
	H198	0.060023360	-1.146010868	5.484264012
	H199	0.120027251	3.763212242	11.143016997
	H200	-1.283073783	2.667476384	11.025206085
	H201	0.679406225	0.824604105	10.437115494
	H202	4.335471806	-11.046305853	6.992386814
	H203	-5.033283180	-4.587618400	-3.126822450
	H204	-2.694576464	-5.829191693	-0.412813400
	H205	-5.626109775	-5.478884507	-7.404296814
	H206	-3.931841424	-5.828728233	-7.835391558
	H207	-5.099069758	-3.236931332	-6.671052071
	H208	-1.566650661	0.131182007	-10.045452652
	H209	-5.179478142	-3.659188727	-9.178272436
	H210	-3.975836055	-0.248648006	-2.429400645
	H211	-6.060047630	-1.765872032	-0.771492656
	H212	-2.364778016	-1.541047200	-8.320697694
	H213	-4.100598976	0.620397071	-9.311211505
	H214	-2.232469523	-3.600815253	-9.025237153
	H215	-4.554492736	-4.302626078	-1.455855213
	H216	-3.356811227	-3.510500564	-6.726664985
	H217	-3.197981763	-4.792433234	-9.623543955
	H218	-7.404528575	0.658764876	-3.187055445
	H219	-4.094516255	0.086899585	-0.714302118
	H220	-2.556375869	-6.352234053	-6.274354196
	H221	-4.365110323	-2.201940029	-0.607946744
	H222	-1.533848173	5.049918918	2.001995375
	H223	-2.275347997	2.689557293	1.061662859
	H224	-6.075403274	6.445266091	2.215220819
	H225	-5.828444695	4.556097350	-0.092247193
	H226	-2.875462062	7.801712698	0.255859321
	H227	-1.218152186	12.709342763	1.324798393
	H228	-2.211655434	9.461330349	2.989913650
	H229	-3.672517780	10.268545967	0.818723005
	H230	-0.708071486	9.599762928	0.299460308
	H231	-2.715467489	14.094179914	-2.388328270
	H232	-0.006210764	5.414522669	0.081252789

	H233	-1.348106975	5.959584820	-0.935339718
	H234	-1.375868262	14.677277979	-0.291380603
	H235	-3.077326174	9.743312309	-0.756469360
	H236	-3.662160128	5.484191110	0.031914806
	H237	-5.994832572	4.690040608	2.389964429
	H238	-6.641365217	7.268026248	-2.210538704
	H239	-1.215015005	1.513213959	-2.829385500
	H240	-7.969345663	6.149883812	-1.843195236
	H241	9.981362778	-3.055575330	3.629264488
	H242	8.798514055	-2.061747012	3.099276458
	H243	4.767326505	3.308694188	-2.188777616
	H244	2.667038287	4.759907572	-0.853210546
	H245	6.188675793	5.196337975	-0.851490946
	H246	-0.628753138	4.034224854	-2.579119399
	H247	-1.441438007	5.477648564	-4.187506581
	H248	-3.065202529	5.396156647	-5.718373672
	H249	-0.480941090	6.508093771	-3.469180963
	H250	-3.479147659	3.155179405	-4.927761520
	H251	-4.893978659	4.247016668	-5.101104721
	H252	7.104527249	8.476910878	-3.989972344
	H253	7.012994531	10.239673954	-4.185756144
	H254	5.501045439	7.525628830	-5.183083048
	H255	3.863989360	8.169643589	-5.331853694
	H256	2.827942329	7.749870706	-8.081833122
	H257	0.945016899	3.884336658	-6.871087076
	H258	-0.431809766	10.861282930	-3.295017024
	H259	2.738935888	8.886631493	-0.008961780
	H260	1.061459345	7.997544346	-5.020940734
	H261	4.154556163	2.545807992	-4.530828867
	H262	6.257926776	3.358430664	-3.974365197
	H263	1.892575660	3.722901189	-4.353395920
	H264	-6.717828780	4.398887936	-2.698886065
	H265	9.034604285	4.537902141	-5.201941315
	H266	-5.264149814	2.078579793	-3.693146670
	H267	4.384267976	3.267411002	-6.120736390
	H268	1.529140215	7.419534654	-1.570029592
	H269	8.945037004	5.366497485	-3.634450508
	H270	2.552071429	6.052861952	-8.304375762
	H271	1.438840669	8.856037752	-6.472910470
	H272	-6.232155189	-0.503651391	-3.469837611
	H273	-6.572304055	0.558218127	-1.026234115
	H274	0.882102319	12.348295161	-1.811711213
	H275	-3.279205339	3.767370039	-2.535963960
	H276	4.560405495	5.532279407	-5.116820275
	H277	2.585011511	4.428143743	-7.234981863
	H278	2.094280077	2.438663935	-5.532698645
	H279	0.804229291	5.726593942	-5.170226786
	H280	5.968898973	7.092508560	1.352053174
	H281	5.614932033	7.730029931	-1.798962989
	H282	7.705613452	9.743541157	-1.963817274
	H283	7.445338646	6.345820909	0.699582972
	H284	4.511849879	9.974288889	-0.799065214
	H285	1.801112122	-7.237420364	-7.766395638
	H286	0.972048715	-8.101854606	-6.457293651
	H287	2.985920950	-9.905770232	-2.678906987
	H288	0.809695146	-9.435584486	-8.408162898
	H289	-0.671809561	-8.750370582	-8.261269260
	H290	7.187754060	0.223950725	-0.830313334
	H291	9.104807368	2.406382984	0.108731080
	H292	8.755571676	2.488650395	-4.137943514
	H293	6.669503505	1.893318500	-0.434441627
	H294	10.303928208	1.962881573	-0.909350070
	H295	6.041435528	-2.624463879	-6.320321415
	H296	8.792055282	1.073064676	-2.488877366
	H297	-0.038760191	-7.159454410	6.923932927
	H298	0.613513915	-9.538141826	5.097742801
	H299	3.761443241	-2.701737434	-6.323975240
	H300	4.271045016	-2.397868889	3.569191796
	H301	1.435750117	-1.245549168	-8.636610249
	H302	-2.147050357	-2.013881028	-4.153326119
	H303	1.622438770	-0.349964732	-7.126218788
	H304	1.242305384	-4.878539516	6.012388764
	H305	1.727484859	-2.779913447	-6.065698268
	H306	0.420329880	-5.277538383	4.501930412
	H307	-0.612750664	-7.417298350	5.257954929
	H308	1.782337703	-7.450842581	4.458428594
	H309	2.337170631	-7.066958018	6.091791283
	H310	-4.591885074	0.920275341	-7.017430563
	H311	-1.284853055	-9.372231539	7.404226121
	H312	-3.429359006	-11.136445752	6.116949790

	H313	0.173091724	-1.108359780	-4.145009998
	H314	-2.360211571	-11.170474046	8.838040073
	H315	-3.826148130	-10.675311945	8.303145335
	H316	2.613304046	-5.458861761	3.405694729
	H317	0.682211132	0.999860331	-5.312470774
	H318	-3.907184227	-0.905901525	-5.518017378
	H319	-0.134221806	-12.545010676	1.517244577
	H320	0.430785700	-10.791305144	-1.226574903
	H321	0.158825845	-10.197188242	2.510935569
	H322	-1.179118642	-12.787450192	0.390259878
	H323	5.483262082	-3.124447636	-9.034460403
	H324	1.937457296	-7.751770306	1.667583367
	H325	-2.973426288	-7.072730500	2.636438000
	H326	1.401240404	-5.338685380	-6.530925876
	H327	-3.253117088	-9.046621490	1.431276366
	H328	-1.796516419	-8.814771823	-5.079809522
	H329	-1.527972343	-6.740619651	-3.651729610
	H330	-2.522285422	-12.450820849	6.874544598
	H331	3.743003584	-3.651363687	2.482028336
	H332	3.013958715	-9.247292740	3.142222403
	H333	3.424122140	-7.310235404	1.615863673
	H334	2.765599106	-9.891137398	5.393916407
	H335	2.640791444	-3.589526946	6.445566819
	H336	-2.084663858	-9.511183189	-1.362727778
	H337	-1.364036768	-10.155304938	0.118172871
	H338	-3.813533757	-8.295983184	-0.070303370
	H339	-0.185660248	-3.928585204	-5.353744820
	H340	-1.075874031	2.123388302	-6.709676043
	H341	-3.452225619	2.206316058	-7.497352540
	H342	-1.830157991	-5.816056162	2.122862346
	H343	2.146287660	-10.531523391	3.296047570
	H344	0.078806621	-11.289145730	3.602864315
	H345	3.179004530	-2.149407929	5.682275436
	H346	-0.356026975	-10.763080411	-4.239201697
	H347	1.326968542	-9.328901503	-0.856021553
	H348	-1.393789881	-4.326600621	-6.611952134
	H349	5.962088515	-0.961778757	-6.942647252
	H350	6.098527963	-3.474952858	5.095902248
	H351	6.370613149	-3.613649154	3.341368589
	H352	2.964824395	-12.105533726	-3.528582243
	H353	1.573161237	-12.488990328	-2.756781292
	H354	2.729829658	-13.157331809	1.600095192
	H355	1.587411024	-12.158837651	-0.093015036
	H356	4.483434599	-13.711535791	-0.433598926
	H357	3.682892540	-11.661561650	1.719018549
	H358	8.288918733	-5.498715172	3.774546931
	H359	5.711085615	12.734890937	0.671666533
	H360	3.677401813	-9.981053690	8.257567289
	H361	4.668062685	-8.019055746	6.580731271
	H362	3.825957035	12.526255472	-1.699646201
	H363	3.069600440	11.182156164	0.966138172
	H364	2.254539046	12.586575091	0.247569025
	H365	-0.943006451	8.661207777	-3.795102513
	H366	1.731388672	-2.227622675	1.231574199
	H367	-1.000047056	-3.387213343	-0.119862354
	H368	0.325876291	-1.398774972	0.873537979
	Mo369	0.347922814	-6.233146097	-0.926977086
	N370	1.063897586	-1.991144097	0.491394764
	N371	1.328868440	1.038580562	-0.692534910
	N372	1.217470221	2.061371555	-0.272484103
	N373	-9.941110397	-1.392913089	-0.089624052
	N374	-9.705031517	2.128151872	1.147728469
	N375	-9.473150158	2.249685587	3.947783835
	N376	-7.909555149	0.002558653	4.572010732
	N377	-5.430492304	0.853072541	3.460159560
	N378	-4.870727186	3.042707933	5.236993267
	N379	-4.877755847	1.934866706	7.929981929
	N380	-3.942514774	-0.814779954	7.961688279
	N381	-1.718037834	-0.538124820	6.273926086
	N382	-0.048584638	2.021329307	6.453803579
	N383	-0.277933861	2.918279253	9.224536996
	N384	-2.829503914	-10.669342328	8.080266675
	N385	-0.813336191	-9.734093769	6.569569208
	N386	2.485337376	-4.981494386	4.303109886
	N387	3.096137609	-3.162481963	5.646507643
	N388	3.618171060	-3.161230596	3.394094490
	N389	2.727101020	-9.786851361	6.432683517
	N390	-6.398241865	0.477492492	-3.181023996
	N391	-4.939704288	-3.943304575	-2.335778578
	N392	-5.056539847	2.718070158	-2.909384727

N393	-6.197404985	5.217432155	-2.390495941
N394	-6.037619053	5.427576886	0.412792049
N395	-3.280421741	5.582092789	0.974740391
N396	-1.019284181	3.431594976	-1.844058967
N397	-1.914936388	1.678759154	-0.803954030
N398	-2.079212115	8.073344490	0.827860541
N399	-2.932872671	12.152224165	-1.476078660
N400	-1.805117473	13.771844546	-0.462089639
N401	8.994394137	-3.032026139	3.360680096
N402	9.373296593	1.693515406	-0.577560812
N403	8.525470342	3.315314780	-3.593024239
N404	6.043998633	4.285793568	-4.361521477
N405	1.468943856	5.695408756	-5.973042092
N406	2.371497538	6.897013057	-7.773633128
N407	1.560898639	8.014601973	-5.914987541
N408	4.292588183	4.219681791	-2.056973188
N409	5.694673059	5.956931118	-0.378240936
N410	6.214476053	8.453528908	-1.402218374
N411	4.848798694	8.317342941	-5.114726159
N412	4.928135188	10.889580990	-0.955980609
N413	0.308903384	-8.547118141	-8.475871649
N414	0.409916816	-5.337723351	-6.788827055
N415	1.301001708	-2.423850142	-6.938511744
N416	4.167027728	-2.092016492	-7.040513900
N417	-3.127572132	-3.780821389	-9.495422833
N418	-3.243208812	-1.060938452	-8.540664327
N419	2.079072901	-11.713464977	-3.195948186
N420	-0.141364767	-7.941896736	-2.511833725
N421	-1.155362922	-8.754321156	-4.293161926
N422	2.593301619	-11.967322531	-0.089045817
O423	-8.351119658	0.348176735	1.581755664
O424	-7.477372517	3.328337513	3.738402729
O425	-7.459841456	0.914399039	6.620413641
O426	-4.447883444	-0.306076901	5.153347311
O427	-2.672623022	2.685422757	4.780040275
O428	-3.127815545	3.359937210	8.318735038
O429	-3.078312666	-0.375816251	10.037400941
O430	-0.818336276	-2.466672263	7.149014708
O431	1.572140265	0.412314511	6.480123396
O432	1.986247179	2.986886418	9.155330879
O433	1.082914440	1.613195225	12.276249733
O434	-0.872824610	-11.541012181	5.131277985
O435	1.443006364	-9.292567254	8.274451727
O436	6.209205979	-9.232401331	7.136890146
O437	-5.221934195	2.731045545	-0.616277458
O438	-5.618543779	-2.208167552	-3.635859068
O439	-4.119926361	6.076326543	-1.997377352
O440	-3.071125371	5.209272210	-4.762732078
O441	-6.866216945	7.567387756	0.304573100
O442	-3.696088685	5.777507203	3.211941565
O443	-0.413571032	7.353099230	2.207065760
O444	-0.650746994	10.734253384	2.654202284
O445	8.239539557	-5.445610127	5.814864517
O446	5.537729021	2.477908064	3.567665248
O447	7.978737497	4.134490000	-1.533952468
O448	6.949682782	6.278278265	-5.062618930
O449	3.318379998	6.050018089	-3.017880004
O450	3.720843564	6.520120726	0.647606738
O451	5.215261834	3.309314377	0.792375050
O452	7.724267748	8.911310594	0.259149418
O453	6.478847159	12.008428800	-2.194427681
O454	4.255608494	10.163886373	-3.905210613
O455	4.534707532	14.296533035	0.083448388
O456	-0.179646091	8.187828281	-3.411579082
O457	-1.377409222	-6.640985193	-7.414018165
O458	-0.608112967	-2.503092265	-8.206916943
O459	3.903809722	-0.499461477	-8.638876529
O460	7.308667733	-2.237223646	-8.814258413
O461	-5.469087071	-1.226897900	-9.009582658
O462	-5.198485688	-5.710889553	-4.835095951
O463	-3.145869320	-6.325236303	-5.458980591
O464	-1.628941635	2.038362600	-9.332023336
O465	4.438552826	-10.987418763	-0.965734014
O466	5.569626883	-13.439651683	1.273534370
O467	-3.585305001	-5.285467182	-0.063400422
O468	-4.670997359	-5.258563356	1.905324699
O469	-2.667970732	-12.227893524	-0.308419726
O470	-4.449706259	-10.889747220	-0.694092532
O471	0.427051446	-7.846616714	0.577947800
O472	-0.653559038	-8.681408374	2.362306129

	O473 -1.684006395 -6.728780936 -0.458102119<br>O474 2.980996229 -10.103585008 3.636345079<br>O475 2.739703922 -7.622841923 2.237486204<br>O476 -0.504913387 5.623437178 -3.896346709<br>O477 -0.363167193 -13.193437263 0.814404352<br>O478 0.553031297 -11.077657186 2.759853294<br>S479 5.820432975 1.204941099 -2.602025027<br>S480 2.633722220 -0.576622316 -3.302365106<br>S481 -0.436333320 -4.624419825 -2.441293883<br>S482 3.802131204 -0.841618074 0.022614523<br>S483 -0.302893352 -1.622558816 -1.727302333<br>S484 2.757214417 -4.303741016 -4.659628122<br>S485 0.739359563 -4.879478011 0.982548092<br>S486 5.514364762 -2.694185502 -2.538284164<br>S487 2.575907175 -6.562960596 -1.679008057<br>S488 4.512814155 -4.579384273 0.687071156<br>end

product

Fe(139) -2.441<br>Fe(140) -0.694<br>Fe(141) -2.522<br>Fe(142) 2.697<br>Fe(143) 3.011<br>Fe(144) 1.953<br>Fe(145) -0.429	9651826nh26hx2n2xd35te.car_2<br>C1 -9.368705693 0.811025094 1.046502131<br>C2 -10.351962513 -0.066224916 0.262511677<br>C3 -8.814489936 3.101673608 1.745656692<br>C4 -8.547279771 2.905398892 3.234496077<br>C5 -9.268438023 1.922200255 5.349717441<br>C6 -8.139236512 0.910122552 5.578241734<br>C7 -6.757593757 -0.861331147 4.619585979<br>C8 -5.446382278 -0.091774935 4.441859817<br>C9 -4.281303825 1.712921346 3.272582296<br>C10 -3.883687451 2.520405861 4.516846671<br>C11 -4.574311648 3.846649014 6.421049075<br>C12 -4.148969991 3.026675814 7.647831697<br>C13 -4.742097974 1.213496683 9.191904842<br>C14 -3.853374281 -0.034533186 9.108273976<br>C15 -3.126622298 -1.927192430 7.723975844<br>C16 -1.784091323 -1.654165583 7.039179101<br>C17 -0.524397181 -0.225278187 5.499182480<br>C18 0.414015835 0.774064728 6.201771965<br>C19 -0.825172719 0.123574789 4.025552783<br>C20 0.452970253 0.519474469 3.278256975<br>C21 -1.513677236 -1.051622368 3.320867110<br>C22 0.823488770 3.050919249 7.002535283<br>C23 0.865146756 2.987968188 8.541981300<br>C24 0.505567067 4.459865108 6.446918757<br>C25 1.404141554 5.517161371 7.100191087<br>C26 0.676066443 4.482013373 4.922116558<br>C27 -0.309408254 2.850980786 10.657376082<br>C28 0.548151770 1.707295905 11.165580490<br>C29 -2.610991908 -11.350445842 6.793881368<br>C30 -1.338443662 -10.898675897 6.083353566<br>C31 0.426453045 -9.155949417 6.090739418<br>C32 1.594690364 -9.469263687 7.042656371<br>C33 0.257909997 -7.632734981 5.944193888<br>C34 1.521899619 -6.986913842 5.379481238<br>C35 1.339183256 -5.517710560 5.032926839<br>C36 3.050304290 -3.811230826 4.406088161<br>C37 3.968176249 -10.057125257 7.166252710<br>C38 5.033100133 -9.009630140 6.931723725<br>C39 -5.835585701 0.681946707 -1.847623411<br>C40 -5.348650270 2.139264057 -1.720434085<br>C41 -4.663086163 -0.285017786 -1.591419051<br>C42 -5.074867338 -1.744382621 -1.309396734<br>C43 -5.217885201 -2.645953049 -2.536008512<br>C44 -4.243179187 3.901875222 -3.078374112<br>C45 -4.847158247 5.163683175 -2.454387075<br>C46 -3.956061304 4.077355222 -4.576462823<br>C47 -6.884338100 6.312752190 -1.734769146<br>C48 -6.578724119 6.486689271 -0.242517451<br>C49 -5.612465528 5.528932023 1.792898476<br>C50 -4.107239881 5.635934646 2.047301503<br>C51 -1.831741766 5.675891214 1.142659628<br>C52 -1.370597346 7.106105073 1.471462053<br>C53 -1.074262194 5.253647959 -0.128076963<br>C54 -1.348997158 3.858760697 -0.575669030<br>C55 -1.928862579 2.763094288 0.033610233<br>C56 -1.407593483 2.109596653 -1.944677213<br>C57 -1.649156790 9.465333840 0.843730000<br>C58 -1.454762275 9.910006264 2.291001910<br>C59 -2.776036620 10.286187590 0.172102814<br>C60 -2.445261779 11.687478557 -0.249221901

	C61	-1.751201516	12.690306152	0.399202370
	C62	-2.506186171	13.414894628	-1.554273403
	C63	8.193662091	-3.402153863	4.485247276
	C64	8.294216282	-4.903067969	4.691187524
	C65	6.701468027	-3.090946339	4.226604352
	C66	6.409352617	-1.615894726	4.071218468
	C67	6.062157135	-0.821233911	5.179305945
	C68	6.465325610	-0.995618134	2.808722914
	C69	5.772838094	0.539748471	5.037369669
	C70	6.191050546	0.363629630	2.653476411
	C71	5.840758659	1.129881876	3.768322726
	C72	8.419246207	1.744821842	-1.721113077
	C73	8.284565611	3.172385095	-2.276102840
	C74	7.055463024	1.240548927	-1.251293474
	C75	8.468659941	4.611666886	-4.275040111
	C76	7.069280586	5.134586041	-4.607420614
	C77	4.632562884	4.636808228	-4.493207611
	C78	4.031028500	5.018704233	-3.115386156
	C79	3.915259574	3.432690766	-5.144275859
	C80	2.391583049	3.468251790	-5.305590506
	C81	1.836779704	4.352781340	-6.433512188
	C82	1.782200140	6.845667705	-6.561919353
	C83	3.779295493	4.504635351	-0.735073332
	C84	4.390282533	5.765992179	-0.109126288
	C85	3.978095515	3.345546611	0.264098997
	C86	6.513543207	6.854116017	0.427238418
	C87	6.888726559	8.163792597	-0.242965820
	C88	6.646358416	9.588368420	-2.195935652
	C89	6.004253132	10.939237463	-1.791532425
	C90	6.536406432	9.372188200	-3.709837806
	C91	5.105598079	9.326687243	-4.230963794
	C92	4.110766556	12.067689048	-0.731840583
	C93	4.865939834	13.117081802	0.069260481
	C94	2.810341021	11.657327503	0.014360027
	C95	1.953012679	10.735013357	-0.822861892
	C96	1.038549761	11.260141349	-1.747944446
	C97	2.088615879	9.338648812	-0.747627102
	C98	0.296931027	10.427716476	-2.588374321
	C99	1.376244654	8.490393660	-1.600280722
	C100	0.490131007	9.046240634	-2.518551466
	C101	0.824516670	-7.634225290	-7.452528666
	C102	-0.146339536	-6.486680065	-7.224803358
	C103	-0.340179978	-4.140849516	-6.427464555
	C104	0.089819636	-2.940722166	-7.279239252
	C105	1.873958323	-1.293361669	-7.651138233
	C106	3.389207011	-1.286806735	-7.826692742
	C107	5.598506384	-2.017118497	-7.130017302
	C108	6.154737491	-2.478589460	-8.462892629
	C109	-4.252036881	-3.316330005	-8.695533697
	C110	-4.371329024	-1.789991467	-8.782590547
	C111	-4.298758162	-3.778542693	-7.221317348
	C112	-4.578092806	-5.291268623	-7.129390664
	C113	-4.361610734	-5.799087136	-5.722226856
	C114	-3.242760852	0.371753920	-8.645023621
	C115	-2.022733049	0.865241742	-9.396423240
	C116	-3.529004163	1.117200848	-7.318386258
	C117	-2.574911935	0.648307237	-6.259195511
	C118	-1.294139949	1.212903468	-6.151594151
	C119	-2.887978097	-0.488648657	-5.495947880
	C120	-0.318486858	0.593897136	-5.366336753
	C121	-1.911995695	-1.102704225	-4.710257802
	C122	-0.614329380	-0.582905327	-4.676607283
	C123	2.361186575	-10.686586216	-2.200912178
	C124	3.215358228	-11.215516770	-1.038041164
	C125	1.086384825	-10.012727430	-1.672745865
	C126	0.261716078	-9.264706818	-2.670589674
	C127	-0.374429805	-9.768551073	-3.786546847
	C128	-1.000768401	-7.668081832	-3.476064936
	C129	3.380189041	-12.464837313	1.028900397
	C130	4.588645406	-13.254833775	0.577555780
	C131	-3.722079442	-5.648659683	1.246170496
	C132	-2.577424547	-6.500298905	1.787728785
	C133	-1.944970091	-7.463430876	0.740093191
	C134	-2.909884052	-8.652045206	0.465742383
	C135	-2.305141146	-9.819617022	-0.319286010
	C136	-3.219875349	-11.059957104	-0.426723328
	C137	-0.634714760	-8.035582023	1.300809340
	C138	2.426614798	-3.259733570	-1.523894426
	Fe139	1.801372582	-4.735645797	-2.714978577
	Fe140	1.833035559	-1.452855621	-1.238216958

	Fe141	4.208145697	-2.890331297	-0.742929115
	Fe142	3.299079618	-2.686815438	-3.181375042
	Fe143	4.570551812	-0.626194845	-2.017863261
	Fe144	2.571673738	-4.809767150	-0.300922971
	Fe145	0.400860819	-3.441540728	-0.645887774
	H146	-9.571282735	-1.882235262	0.768203539
	H147	-9.087215445	-1.352536932	-0.697028071
	H148	-10.502959184	2.468150963	0.605403832
	H149	-10.647164757	0.463932383	-0.656881073
	H150	-11.266388327	-0.138089492	0.876182071
	H151	-7.834262557	3.080577482	1.246690802
	H152	-10.329841881	1.924235432	3.476902897
	H153	-9.244860909	4.101870652	1.600334469
	H154	-10.199184759	1.514165314	5.766030726
	H155	-8.404148186	0.121954308	3.690299278
	H156	-9.003263357	2.821511260	5.920491389
	H157	-3.407754617	1.114772892	2.985880221
	H158	-6.251126261	0.958114578	2.867189756
	H159	-6.858793676	-1.608034945	3.820309591
	H160	-3.748582168	4.534155884	6.195806986
	H161	-5.848251516	3.032835336	4.881994907
	H162	-6.704245504	-1.386166653	5.580998142
	H163	-4.508584929	2.397170655	2.443434421
	H164	-5.757964341	1.755203718	7.384550126
	H165	-5.459823590	4.438950873	6.689605580
	H166	-2.440800906	-1.353589659	3.827959151
	H167	-1.771608614	-0.779189693	2.284784771
	H168	-0.846475310	-1.928558987	3.279316106
	H169	-1.497766781	0.995023008	4.020349141
	H170	-5.726142210	0.897346770	9.568098747
	H171	0.011734021	3.766608228	4.418342745
	H172	-1.216336317	2.931697390	8.715736147
	H173	1.245230239	5.586687848	8.185852048
	H174	0.209133565	0.765389445	2.235885695
	H175	1.186758999	-0.301961681	3.271878455
	H176	8.487932635	-2.917082135	5.435940300
	H177	0.456723023	5.483431934	4.523856801
	H178	1.204944408	6.505079694	6.658888115
	H179	1.715362533	4.231328450	4.650427034
	H180	2.466490653	5.276903875	6.931418480
	H181	-0.546304681	4.696451160	6.690436553
	H182	1.848566242	2.785058432	6.704838144
	H183	-0.975518578	2.300673088	6.072848813
	H184	0.937826408	1.402756405	3.718706693
	H185	3.749148007	2.391738987	-0.236790880
	H186	3.255419326	3.495485950	1.078944832
	H187	5.268393920	3.098341108	1.731934654
	H188	5.253393503	2.873559241	4.387757298
	H189	6.211240726	0.826554369	1.666929001
	H190	6.696437187	-1.592949966	1.923856337
	H191	6.005879767	-1.274304749	6.172675205
	H192	-2.584410079	-0.035623287	6.052387683
	H193	-3.680156101	-2.643694918	7.098950834
	H194	-2.903472198	-2.411915620	8.681683483
	H195	-4.502636257	-0.411835520	7.174740114
	H196	-4.280741633	1.872581423	9.937266050
	H197	5.482476999	1.136267233	5.906361544
	H198	0.067180126	-1.152782057	5.509650926
	H199	0.035608199	3.789633518	11.119969895
	H200	-1.340529881	2.663597326	10.993876357
	H201	0.668509111	0.857318523	10.454099721
	H202	4.386188684	-11.054384389	6.957318110
	H203	-5.030137795	-4.595695397	-3.129513685
	H204	-2.698157031	-5.806455991	-0.386979327
	H205	-5.626349689	-5.479190934	-7.403585743
	H206	-3.933516158	-5.838935463	-7.832458456
	H207	-5.087569001	-3.237447944	-6.675777902
	H208	-1.544238099	0.103188880	-10.055259026
	H209	-5.173355288	-3.673748128	-9.180266709
	H210	-3.981378276	-0.246864856	-2.455042493
	H211	-6.042358352	-1.772484860	-0.776765975
	H212	-2.357859807	-1.556883700	-8.325520072
	H213	-4.083403052	0.606721022	-9.330596495
	H214	-2.226239085	-3.611566729	-9.037899605
	H215	-4.557511978	-4.306061312	-1.457627887
	H216	-3.346673887	-3.520029065	-6.732524432
	H217	-3.190326950	-4.813549721	-9.617003497
	H218	-7.420720226	0.638581552	-3.178865098
	H219	-4.080038070	0.091612121	-0.740395726
	H220	-2.557963043	-6.354088182	-6.267420326

H221	-4.343634942	-2.198567730	-0.624778714
H222	-1.536301142	5.050380655	1.997048022
H223	-2.325852369	2.698592989	1.042677288
H224	-6.070985186	6.434940610	2.212631833
H225	-5.824479730	4.551428698	-0.099764334
H226	-2.889077702	7.812635061	0.287510599
H227	-1.223432103	12.719695055	1.346120750
H228	-2.191073913	9.457434834	2.998418992
H229	-3.659795714	10.275969413	0.834774146
H230	-0.700033479	9.600254004	0.301711412
H231	-2.692815065	14.095577488	-2.381630137
H232	-0.001743697	5.393950682	0.085451664
H233	-1.325708055	5.954331029	-0.942107949
H234	-1.371958876	14.684996139	-0.274307158
H235	-3.073052064	9.750383782	-0.742893953
H236	-3.659992782	5.493321092	0.023810838
H237	-5.989767088	4.680076109	2.383183426
H238	-6.629890515	7.270037305	-2.210848904
H239	-1.250712342	1.516046947	-2.841022366
H240	-7.965858300	6.160581753	-1.844901411
H241	10.002824334	-3.047239183	3.594592367
H242	8.814522378	-2.052654697	3.077327112
H243	4.792679766	3.291472225	-2.205599957
H244	2.700459998	4.698399512	-0.826709961
H245	6.217275694	5.201452338	-0.851083271
H246	-0.633863676	4.026199949	-2.583009148
H247	-1.448586525	5.477786303	-4.193903474
H248	-3.064372485	5.384155879	-5.733352818
H249	-0.484749201	6.500739080	-3.466525639
H250	-3.490973503	3.146981112	-4.943904320
H251	-4.901251060	4.244547016	-5.117704526
H252	7.104424960	8.476399621	-3.995091327
H253	7.018969519	10.239972184	-4.186668179
H254	5.493339992	7.528935976	-5.180175475
H255	3.859101987	8.181847494	-5.329918901
H256	2.824865079	7.750232773	-8.080116218
H257	0.941518135	3.884635475	-6.871060174
H258	-0.423376381	10.853458574	-3.290974913
H259	2.748475625	8.880537677	-0.005512189
H260	1.061657545	7.994562422	-5.016332171
H261	4.156810574	2.543106794	-4.541031980
H262	6.264360565	3.358375367	-4.000090161
H263	1.894194781	3.718278573	-4.355376084
H264	-6.725134556	4.401045715	-2.703402350
H265	9.039338702	4.557972152	-5.211339519
H266	-5.277738736	2.075170947	-3.707280261
H267	4.381080163	3.267995244	-6.130061069
H268	1.537973970	7.412422684	-1.564244786
H269	8.939267228	5.375597258	-3.639026852
H270	2.546331276	6.054071345	-8.304265502
H271	1.438094467	8.855903796	-6.466656830
H272	-6.240345573	-0.512257471	-3.474151215
H273	-6.566597415	0.549898778	-1.026988311
H274	0.891690609	12.341042912	-1.809716047
H275	-3.288861605	3.757724585	-2.552753990
H276	4.550973186	5.532744073	-5.116373573
H277	2.580687962	4.428092026	-7.237374770
H278	2.093295149	2.437226161	-5.538654642
H279	0.802333453	5.725132411	-5.169996128
H280	5.960859726	7.101931360	1.345878625
H281	5.626931868	7.725497130	-1.797155504
H282	7.713106975	9.741300561	-1.969969840
H283	7.449647204	6.356646323	0.716673276
H284	4.520228831	9.973497220	-0.799079355
H285	1.799397625	-7.235274830	-7.771012576
H286	0.978169086	-8.099061734	-6.456262187
H287	2.988659489	-9.931841880	-2.698269341
H288	0.803546678	-9.439809649	-8.399498766
H289	-0.675945467	-8.748936904	-8.252762175
H290	7.181886778	0.222299953	-0.862263396
H291	9.088675076	2.397036035	0.095399243
H292	8.763170780	2.499393334	-4.159942877
H293	6.672472214	1.892232293	-0.454098181
H294	10.296800200	1.965398363	-0.917328049
H295	6.019441732	-2.673888740	-6.352666457
H296	8.795278693	1.074918423	-2.509938525
H297	-0.003134093	-7.197823605	6.923592851
H298	0.626388993	-9.584875414	5.100650499
H299	3.740034521	-2.725187745	-6.350619552
H300	4.240557872	-2.411715504	3.538533056

H301	1.425260810	-1.254180867	-8.651363065
H302	-2.154595333	-1.986602493	-4.117039444
H303	1.611082848	-0.347054662	-7.147525883
H304	1.201619309	-4.912156939	5.936849215
H305	1.716477439	-2.770499608	-6.069544886
H306	0.438611901	-5.370250883	4.413167713
H307	-0.589292578	-7.459066991	5.262091191
H308	1.818808347	-7.517050402	4.463760901
H309	2.358312476	-7.079484960	6.089154399
H310	-4.572488428	0.913244852	-7.036611815
H311	-1.264569159	-9.399568784	7.411129433
H312	-3.440858281	-11.128997033	6.105197421
H313	0.157907472	-1.072927251	-4.082057695
H314	-2.396757291	-11.174644334	8.833916611
H315	-3.842674187	-10.641418088	8.280633585
H316	2.652217957	-5.511057264	3.379418837
H317	0.684010485	1.015490350	-5.283260716
H318	-3.895620859	-0.908530788	-5.530082659
H319	-0.127022539	-12.551619634	1.516609131
H320	0.439563849	-10.780864083	-1.214468746
H321	0.160285601	-10.195366985	2.514789819
H322	-1.170844071	-12.784054258	0.388570823
H323	5.460201811	-3.102710994	-9.075285366
H324	1.943155462	-7.748949863	1.673037169
H325	-2.925961229	-7.051928956	2.668997963
H326	1.399151494	-5.336346624	-6.531545252
H327	-3.248879185	-9.020965523	1.446839044
H328	-1.805632898	-8.807872368	-5.057758353
H329	-1.532603397	-6.737437984	-3.625094274
H330	-2.550475013	-12.449607719	6.870682060
H331	3.733374745	-3.670157129	2.447099832
H332	3.021255451	-9.256072090	3.132026095
H333	3.433611962	-7.322775647	1.609526813
H334	2.783904092	-9.915410251	5.383417592
H335	2.606030018	-3.614796215	6.406476298
H336	-2.076119308	-9.492837235	-1.344895556
H337	-1.356580365	-10.136660533	0.135721546
H338	-3.798302049	-8.268728793	-0.058587473
H339	-0.194158477	-3.925456676	-5.357671479
H340	-1.053036929	2.109115242	-6.727904398
H341	-3.429044159	2.194149022	-7.520413891
H342	-1.804926928	-5.788257279	2.124655949
H343	2.149502049	-10.538454661	3.286759440
H344	0.073237620	-11.296144014	3.597879457
H345	3.101844711	-2.161693623	5.632112360
H346	-0.353061241	-10.754952549	-4.233841074
H347	1.356204456	-9.326512108	-0.861157871
H348	-1.398090204	-4.331545205	-6.617112329
H349	5.953596800	-0.994503586	-6.929055615
H350	6.124118569	-3.495693130	5.071870150
H351	6.396402837	-3.632811315	3.317272400
H352	2.936433570	-12.140613927	-3.528974512
H353	1.544958659	-12.499680356	-2.745312771
H354	2.740997723	-13.151889959	1.605449754
H355	1.594339332	-12.171549112	-0.094971717
H356	4.487998898	-13.724260073	-0.431092692
H357	3.696197309	-11.656327540	1.707015233
H358	8.326661319	-5.495809336	3.745909585
H359	5.718920070	12.732155488	0.675818322
H360	3.713449308	-10.022497108	8.241224632
H361	4.657008090	-8.019192456	6.575523639
H362	3.834685179	12.526074455	-1.696790199
H363	3.081500224	11.176694830	0.967458069
H364	2.261411767	12.579738706	0.251721601
H365	-0.935599826	8.654529723	-3.788774593
H366	1.754325229	-2.203899209	1.236832269
H367	-1.002419101	-3.385632686	-0.094438791
H368	0.332069864	-1.393796896	0.884892171
Mo369	0.359405964	-6.223331307	-0.920896251
N370	1.066955980	-1.992953452	0.507528315
N371	1.193240430	1.531936014	-0.613502495
N372	1.080681271	2.188957702	0.276355015
N373	-9.897922944	-1.403851451	-0.075281142
N374	-9.716002106	2.126097892	1.151532291
N375	-9.492499218	2.252666464	3.952102202
N376	-7.922025691	0.011829988	4.585042970
N377	-5.440658988	0.855714812	3.475885872
N378	-4.893781961	3.048452083	5.248734188
N379	-4.934780117	1.960619361	7.956923787
N380	-3.957262002	-0.762302796	7.964867441

N381	-1.720354877	-0.529199173	6.271066611
N382	-0.057428277	2.031263330	6.433346840
N383	-0.317906053	2.916871213	9.204692318
N384	-2.843557575	-10.662176938	8.070264777
N385	-0.797458936	-9.766247571	6.576174435
N386	2.498559427	-5.025400391	4.269020126
N387	3.038628620	-3.176198206	5.601197743
N388	3.595352361	-3.180546727	3.358597470
N389	2.749682760	-9.819619689	6.423011076
N390	-6.412853266	0.466658147	-3.182939982
N391	-4.934641855	-3.948633811	-2.340985193
N392	-5.069680298	2.714122105	-2.923888439
N393	-6.200314996	5.217493841	-2.397044895
N394	-6.032683530	5.422084718	0.407430151
N395	-3.277657703	5.588063960	0.966696626
N396	-1.021313391	3.424056003	-1.845495336
N397	-1.965673707	1.685678666	-0.820336395
N398	-2.072221695	8.076562397	0.833382576
N399	-2.912637071	12.154278389	-1.468445246
N400	-1.797026126	13.777941937	-0.447543698
N401	9.013993595	-3.023733647	3.332989332
N402	9.364979547	1.690319939	-0.594038032
N403	8.528800125	3.322491009	-3.611270186
N404	6.046120583	4.286419181	-4.382202420
N405	1.467790076	5.694903305	-5.971821895
N406	2.367387582	6.897894532	-7.772424127
N407	1.560694762	8.013511643	-5.910571253
N408	4.321239775	4.203538557	-2.061785596
N409	5.712736070	5.958616597	-0.383134844
N410	6.224111673	8.452244217	-1.402776081
N411	4.844605167	8.323613432	-5.111883170
N412	4.937221207	10.888533580	-0.955164473
N413	0.305160199	-8.550172273	-8.470536131
N414	0.407699955	-5.337771373	-6.787300293
N415	1.289693386	-2.419728927	-6.944672002
N416	4.149700757	-2.109177386	-7.059500570
N417	-3.122893279	-3.800102987	-9.501723929
N418	-3.233783916	-1.075677699	-8.553086246
N419	2.058155686	-11.734141335	-3.193501058
N420	-0.134918737	-7.938603272	-2.498844897
N421	-1.158507424	-8.747848965	-4.276115608
N422	2.599877196	-11.977488288	-0.094038129
O423	-8.348412619	0.360694594	1.598986446
O424	-7.498281600	3.335359410	3.744765855
O425	-7.487832410	0.923718183	6.636356584
O426	-4.455438568	-0.310478584	5.160625831
O427	-2.690814850	2.671913001	4.824412111
O428	-3.174492091	3.373695949	8.336868484
O429	-3.140023085	-0.359665671	10.067704453
O430	-0.845185262	-2.450826220	7.183039740
O431	1.563784281	0.424481335	6.516038465
O432	1.946457121	3.000680993	9.157247122
O433	1.025851095	1.673035753	12.289798840
O434	-0.896358600	-11.555044106	5.118165539
O435	1.467026213	-9.354098914	8.273714549
O436	6.222369036	-9.203207522	7.130091980
O437	-5.228904805	2.729222054	-0.630589576
O438	-5.601585347	-2.213470298	-3.647473592
O439	-4.118565573	6.069069814	-2.012626413
O440	-3.073623496	5.198999484	-4.777355228
O441	-6.857712042	7.563529281	0.305327670
O442	-3.689809204	5.757410000	3.206586273
O443	-0.383776011	7.340649378	2.177818915
O444	-0.631012819	10.730650369	2.660139277
O445	8.312454530	-5.445909064	5.786830789
O446	5.555841882	2.463642700	3.556280399
O447	7.982644226	4.135106036	-1.549988739
O448	6.944398014	6.288081756	-5.066172587
O449	3.326324068	6.033534616	-3.003818293
O450	3.730986465	6.496111168	0.640894828
O451	5.313435492	3.319503177	0.779637368
O452	7.733602594	8.916508576	0.257720119
O453	6.490908781	12.007147742	-2.189510403
O454	4.264211419	10.178932412	-3.909979606
O455	4.543084866	14.294649277	0.088472761
O456	-0.172825166	8.179959571	-3.405592736
O457	-1.378001777	-6.643455533	-7.409350467
O458	-0.613615944	-2.513519758	-8.219293374
O459	3.898387931	-0.497126451	-8.639896112
O460	7.290577296	-2.230332512	-8.835117782

	O461	-5.456843138	-1.240140277	-9.036551010
	O462	-5.203969240	-5.714873832	-4.835534965
	O463	-3.148486443	-6.326235504	-5.453121206
	O464	-1.604566349	2.013637410	-9.351042592
	O465	4.442437466	-11.001375251	-0.980406593
	O466	5.576274735	-13.446853652	1.273620477
	O467	-3.589810243	-5.281473920	-0.032297477
	O468	-4.667877760	-5.281078342	1.942218097
	O469	-2.660702946	-12.207904970	-0.303063517
	O470	-4.445787987	-10.866484767	-0.661732073
	O471	0.441434325	-7.841091713	0.585725908
	O472	-0.637446458	-8.671713419	2.373621553
	O473	-1.668607275	-6.708476530	-0.440556523
	O474	2.988525895	-10.114705849	3.622144106
	O475	2.750020583	-7.622537360	2.238319442
	O476	-0.511877475	5.619349798	-3.900792055
	O477	-0.357355028	-13.196281738	0.810826756
	O478	0.552404468	-11.078473450	2.759415376
	S479	5.807372733	1.214796800	-2.618584680
	S480	2.596566857	-0.536263647	-3.232952211
	S481	-0.423998241	-4.607690273	-2.430137485
	S482	3.756080670	-0.824747907	0.023488197
	S483	-0.311249517	-1.596034456	-1.689863377
	S484	2.763376946	-4.269299411	-4.660156853
	S485	0.751664452	-4.868003243	0.988193904
	S486	5.488900612	-2.650912195	-2.540610124
	S487	2.588111366	-6.559488578	-1.674331985
	S488	4.530548114	-4.563350046	0.666479601
	end			

Fe2-brNH2-Fe6H to Fe6NH3

47, S=1

reactant

	9651826nh26hxtontm.car_4			
Fe(139) 2.431				
Fe(140) -1.113	1	-9.355258120	0.819819685	1.038887683
	2	-10.334338690	-0.062075677	0.254372648
Fe(141) 2.489	3	-8.817933397	3.113211783	1.743034299
Fe(142) -2.540	4	-8.551858128	2.916826846	3.232167657
	5	-9.274296715	1.932392696	5.346295583
Fe(143) 2.915	6	-8.144006009	0.921821560	5.576253934
Fe(144) -2.159	7	-6.760141612	-0.849759464	4.619608949
	8	-5.447778220	-0.082182430	4.441260421
Fe(145) -0.273	9	-4.279516308	1.719968927	3.273034719
	10	-3.884814067	2.528377745	4.517540160
	11	-4.581941342	3.858062189	6.417888236
	12	-4.160349174	3.040584951	7.647593315
	13	-4.756396127	1.230558365	9.193854382
	14	-3.865554933	-0.015961055	9.110308156
	15	-3.132222197	-1.904503573	7.722630009
	16	-1.787711086	-1.635391678	7.039055894
	17	-0.516959476	-0.212323570	5.500371845
	18	0.415499452	0.789347101	6.207123857
	19	-0.810238592	0.137249972	4.022114143
	20	0.470697631	0.535527309	3.277699218
	21	-1.497447162	-1.038938703	3.317652960
	22	0.817496194	3.069727293	7.002544011
	23	0.854282231	3.006907730	8.541490624
	24	0.495482694	4.478038757	6.447932378
	25	1.392916859	5.536723497	7.100537118
	26	0.663681835	4.501622325	4.922873385
	27	-0.328454504	2.869782763	10.650958835
	28	0.533023938	1.731347262	11.164870894
	29	-2.611688648	-11.344536999	6.789439974
	30	-1.336732453	-10.898264420	6.079076252
	31	0.435794796	-9.162954607	6.088398280
	32	1.604581778	-9.475805481	7.039781202
	33	0.266641886	-7.639957777	5.938982126
	34	1.529110906	-6.992191054	5.373342509
	35	1.338193474	-5.525907898	5.017951345
	36	3.039175593	-3.810423394	4.386825517
	37	3.978128955	-10.063852426	7.163565008
	38	5.039154832	-9.011067989	6.935813969
	39	-5.817702840	0.692685639	-1.842582662

	40	-5.311112797	2.142011366	-1.718120838
	41	-4.644985639	-0.276923758	-1.589774596
	42	-5.055413563	-1.736394316	-1.307768198
	43	-5.204887355	-2.636297128	-2.534843069
	44	-4.196981455	3.895678113	-3.075299248
	45	-4.802349112	5.157203837	-2.449211167
	46	-3.921530490	4.075317074	-4.574841918
	47	-6.847045983	6.307934800	-1.747364195
	48	-6.552649806	6.484234682	-0.253133923
	49	-5.591599414	5.527156601	1.786370203
	50	-4.086865142	5.633614363	2.047486922
	51	-1.806522971	5.688710821	1.156567183
	52	-1.367115831	7.122989558	1.486425927
	53	-1.038523559	5.284011773	-0.111807444
	54	-1.228891208	3.862791558	-0.518031720
	55	-1.727945004	2.752798526	0.131129175
	56	-1.170319216	2.067388903	-1.828486662
	57	-1.643938863	9.470904359	0.841932523
	58	-1.444949075	9.920152683	2.287406046
	59	-2.769389894	10.294045376	0.169584896
	60	-2.437486848	11.695851289	-0.250864095
	61	-1.746621023	12.699542148	0.399751232
	62	-2.495608397	13.423568678	-1.556058441
	63	8.214337504	-3.390050231	4.488343236
	64	8.321429606	-4.891263877	4.689582312
	65	6.720202848	-3.085877579	4.228769663
	66	6.419882743	-1.613790223	4.063059474
	67	6.070576082	-0.812320948	5.165611259
	68	6.468461817	-1.003110596	2.795261125
	69	5.770752034	0.545412769	5.012794060
	70	6.182539626	0.352412088	2.629077942
	71	5.828049872	1.125453580	3.738170857
	72	8.430476022	1.757977615	-1.718968293
	73	8.285906501	3.186609365	-2.267758684
	74	7.067705209	1.232781169	-1.262140404
	75	8.463965157	4.627081038	-4.268563962
	76	7.063400944	5.144807607	-4.603051288
	77	4.626876650	4.642384198	-4.506910358
	78	4.000130217	5.034882413	-3.145924122
	79	3.918542333	3.434787663	-5.160274778
	80	2.394644838	3.465336002	-5.322875726
	81	1.835811041	4.355068722	-6.444330397
	82	1.785579731	6.847456634	-6.564243019
	83	3.663525103	4.537620847	-0.779777106
	84	4.326707564	5.750610021	-0.107581503
	85	3.711005261	3.351633203	0.200395872
	86	6.466745514	6.822740822	0.415159463
	87	6.860811681	8.133689735	-0.249492936
	88	6.654511770	9.598578329	-2.185602535
	89	6.015491784	10.948581477	-1.778551963
	90	6.549850845	9.391369455	-3.702121597
	91	5.118570040	9.346414986	-4.224447131
	92	4.116551167	12.076441433	-0.728481429
	93	4.870996365	13.126881204	0.071751756
	94	2.815207515	11.667149630	0.015865055
	95	1.958422945	10.746704401	-0.823211586
	96	1.045429928	11.273322353	-1.748842972
	97	2.092972599	9.350210035	-0.748919804
	98	0.303282429	10.442129536	-2.590019774
	99	1.379860431	8.503436231	-1.602396139
	100	0.494420702	9.060336189	-2.520789933
	101	0.827897409	-7.621989661	-7.445328861
	102	-0.147333403	-6.477411287	-7.222909545
	103	-0.343838533	-4.126584560	-6.434626149
	104	0.089663076	-2.928241722	-7.287050982
	105	1.872452117	-1.281095307	-7.661388320
	106	3.388631083	-1.276922446	-7.831762282
	107	5.596221586	-2.011983407	-7.133661265
	108	6.150679054	-2.467720245	-8.468951209
	109	-4.235829785	-3.316396710	-8.688192616
	110	-4.352833979	-1.790195639	-8.778529940
	111	-4.286811400	-3.777152573	-7.214311660
	112	-4.574620527	-5.288225161	-7.122664751
	113	-4.359783571	-5.797196648	-5.715773203
	114	-3.221150873	0.368957442	-8.647235192
	115	-1.997987877	0.858648986	-9.395182824
	116	-3.521575161	1.125300821	-7.330706437
	117	-2.584700942	0.654313103	-6.258712250
	118	-1.297082689	1.201481018	-6.146728228
	119	-2.920034480	-0.474601999	-5.493351259

	120	-0.335444365	0.570649276	-5.353620665
	121	-1.960143315	-1.097341117	-4.695113288
	122	-0.655274036	-0.597986720	-4.660166908
	123	2.356115223	-10.676173982	-2.205627162
	124	3.213207805	-11.210016701	-1.046154775
	125	1.089821813	-9.990659312	-1.670371652
	126	0.264457613	-9.234908113	-2.662810556
	127	-0.372571469	-9.729515988	-3.782546642
	128	-0.993814651	-7.629863957	-3.458005815
	129	3.380431582	-12.463887059	1.018736116
	130	4.589682202	-13.253092442	0.568045151
	131	-3.708723630	-5.640381894	1.267762624
	132	-2.559214674	-6.487879295	1.808768687
	133	-1.928631305	-7.449396341	0.760982914
	134	-2.895124819	-8.632962550	0.476179423
	135	-2.293437927	-9.800451213	-0.311431490
	136	-3.212590262	-11.038023501	-0.420985894
	137	-0.617721253	-8.025498224	1.318788196
	138	2.442820051	-3.252324680	-1.516614582
	139	1.730036936	-4.685326115	-2.694989903
	140	1.939151726	-1.441993915	-1.151565039
	141	4.185815278	-2.792519713	-0.760052210
	142	3.352156474	-2.734484559	-3.192468247
	143	4.511236445	-0.577817829	-2.280715659
	144	2.661055949	-4.820989784	-0.325986103
	145	0.413426359	-3.436558328	-0.603089550
	146	-9.557554402	-1.876202771	0.772335404
	147	-9.062279510	-1.351932066	-0.691185988
	148	-10.502014156	2.469330915	0.601795162
	149	-10.625513121	0.463919634	-0.668826576
	150	-11.251782774	-0.131972213	0.863918391
	151	-7.836736760	3.100310671	1.245930263
	152	-10.333983891	1.933457479	3.472660521
	153	-9.255523704	4.110304870	1.598215452
	154	-10.204878691	1.522961973	5.761548774
	155	-8.405560205	0.133826238	3.688258398
	156	-9.011074641	2.832071539	5.917363664
	157	-3.406434951	1.119379764	2.990247267
	158	-6.251428343	0.969946460	2.867074891
	159	-6.860001345	-1.598211659	3.821818284
	160	-3.755807433	4.545573424	6.194275171
	161	-5.850442501	3.041577788	4.876407490
	162	-6.708066776	-1.373233012	5.581855051
	163	-4.502027667	2.402482254	2.441203674
	164	-5.771763615	1.771310551	7.385985060
	165	-5.468760056	4.450249640	6.682370650
	166	-2.426479342	-1.337519203	3.823552748
	167	-1.749795731	-0.770603289	2.279599038
	168	-0.831537421	-1.916913551	3.281075569
	169	-1.483662422	1.008234125	4.015347264
	170	-5.740307845	0.912637954	9.568927407
	171	-0.000957379	3.785790553	4.420011696
	172	-1.227932987	2.953469345	8.707184250
	173	1.236886919	5.604195362	8.186758508
	174	0.233811671	0.784534406	2.232153397
	175	1.204051759	-0.286159971	3.274410687
	176	8.506479368	-2.905353620	5.439603609
	177	0.442703531	5.503221932	4.525928023
	178	1.189982948	6.524821598	6.661440828
	179	1.702985733	4.252185799	4.649737176
	180	2.455435250	5.299600366	6.928467226
	181	-0.556612364	4.712056517	6.693258997
	182	1.844177345	2.807568459	6.706870190
	183	-0.977825518	2.312412170	6.069752332
	184	0.953372472	1.416854612	3.724713740
	185	3.348206728	2.442305288	-0.309527335
	186	3.021046015	3.585526391	1.025545454
	187	5.004145433	2.976489284	1.650111186
	188	5.237070158	2.869627789	4.346179857
	189	6.194101094	0.807067940	1.638601241
	190	6.702666687	-1.605523009	1.914612619
	191	6.019973066	-1.257433783	6.162879053
	192	-2.580967342	-0.018313184	6.043890968
	193	-3.686653198	-2.620167919	7.097390477
	194	-2.911191725	-2.389453390	8.680796628
	195	-4.506596797	-0.387939036	7.173070302
	196	-4.296949256	1.890103012	9.940021936
	197	5.478056837	1.147017439	5.877734522
	198	0.073996204	-1.140211065	5.515237569
	199	0.010456876	3.810393399	11.114553637

200	-1.360191694	2.677739753	10.983446915
201	0.657110253	0.879467607	10.455970311
202	4.399178567	-11.058501427	6.948923498
203	-5.026608846	-4.588324742	-3.126836510
204	-2.689020224	-5.781008082	-0.362962450
205	-5.623818989	-5.470879073	-7.396704141
206	-3.933044214	-5.839165328	-7.825993724
207	-5.073878484	-3.231166059	-6.671275979
208	-1.520596504	0.094968975	-10.052763860
209	-5.158241271	-3.671900521	-9.172858446
210	-3.965192499	-0.237952657	-2.455197691
211	-6.020638082	-1.764100771	-0.771410292
212	-2.338976725	-1.559921173	-8.323009916
213	-4.056120978	0.600544356	-9.341791960
214	-2.210556696	-3.614531974	-9.036192434
215	-4.551263328	-4.298039842	-1.455870101
216	-3.335128285	-3.523147450	-6.722937491
217	-3.178655615	-4.817654382	-9.608322943
218	-7.408729381	0.650451567	-3.165679972
219	-4.064608492	0.096187315	-0.734859987
220	-2.555974793	-6.350245611	-6.260552993
221	-4.321347920	-2.191747878	-0.627057768
222	-1.505794836	5.065944234	2.010591344
223	-2.126447057	2.697872391	1.140062566
224	-6.050716061	6.432973014	2.205779032
225	-5.795312663	4.550767898	-0.105098437
226	-2.880005512	7.815188901	0.283315669
227	-1.222499641	12.729474846	1.348620142
228	-2.180187758	9.472608436	2.998725550
229	-3.653544810	10.284464928	0.831971396
230	-0.694807520	9.601277499	0.298372036
231	-2.679856189	14.104171316	-2.383931791
232	0.026438054	5.495364958	0.083387774
233	-1.346550585	5.948267837	-0.936852547
234	-1.365946196	14.694192644	-0.272744141
235	-3.066489661	9.759212705	-0.746119180
236	-3.626386407	5.488256535	0.026427144
237	-5.971929600	4.677625139	2.373400970
238	-6.591304332	7.265127686	-2.222893029
239	-0.982395142	1.454053889	-2.706235712
240	-7.927335842	6.153246302	-1.865911996
241	10.021709050	-3.017656171	3.600165679
242	8.822601561	-2.039355662	3.075180668
243	4.730645486	3.309435323	-2.204868547
244	2.608996136	4.814861011	-0.928120487
245	6.146010164	5.179974362	-0.876320630
246	-0.525227081	4.006083290	-2.534031594
247	-1.425906494	5.472290759	-4.178087795
248	-3.034861698	5.382724869	-5.734803935
249	-0.475617154	6.515342331	-3.464812798
250	-3.455861592	3.147995563	-4.948644587
251	-4.872223742	4.240174132	-5.107181141
252	7.118078972	8.496435737	-3.989609169
253	7.032826742	10.260939802	-4.174800990
254	5.502109198	7.544765117	-5.171196264
255	3.869285590	8.198692165	-5.318276310
256	2.824687699	7.756207280	-8.083045305
257	0.937395783	3.889825245	-6.878367819
258	-0.416448641	10.869046818	-3.292538601
259	2.751420363	8.891211046	-0.005965222
260	1.070098884	7.995873490	-5.016662344
261	4.161920347	2.546643955	-4.555814113
262	6.257411388	3.370474803	-3.985052715
263	1.896635546	3.706645721	-4.370726511
264	-6.679860757	4.398042902	-2.716206181
265	9.033136769	4.572555411	-5.205917245
266	-5.252279636	2.080180887	-3.707029312
267	4.387466498	3.271986010	-6.144784348
268	1.538876270	7.425173234	-1.567438446
269	8.934072212	5.394326679	-3.636145667
270	2.546521276	6.060278698	-8.310992736
271	1.445093044	8.857428053	-6.466358732
272	-6.229542057	-0.498998969	-3.469622649
273	-6.545643316	0.565399877	-1.018869497
274	0.900116497	12.354510282	-1.810407139
275	-3.240162833	3.748101816	-2.551897603
276	4.556464664	5.533575705	-5.138668455
277	2.576069405	4.432194550	-7.251438338
278	2.099398877	2.434864693	-5.562933057
279	0.811012805	5.727047712	-5.169103569

280	5.921640599	7.077712476	1.335979035
281	5.600369542	7.746300385	-1.821366690
282	7.720368006	9.738268394	-1.948646157
283	7.399219258	6.317718997	0.702992718
284	4.523620415	9.982541408	-0.798790007
285	1.802753920	-7.220896941	-7.760281842
286	0.978905142	-8.085264893	-6.448102377
287	2.986808431	-9.926685794	-2.706755124
288	0.816304460	-9.429408785	-8.388517283
289	-0.665365637	-8.742083221	-8.251002545
290	7.196180716	0.209470349	-0.888352189
291	9.088943007	2.403572448	0.104039101
292	8.770923027	2.518132440	-4.150243981
293	6.663724642	1.870945917	-0.464534100
294	10.303471150	1.974995072	-0.904876200
295	6.014298505	-2.675501917	-6.360549491
296	8.815601782	1.097080954	-2.511154275
297	0.004539971	-7.203226063	6.917220514
298	0.634591493	-9.593760503	5.098901039
299	3.736367448	-2.709225907	-6.348478785
300	4.246320022	-2.423252918	3.519226385
301	1.428837891	-1.253634995	-8.664221782
302	-2.219747061	-1.975564743	-4.100118614
303	1.606838160	-0.328822828	-7.170660351
304	1.189750017	-4.916447501	5.917460354
305	1.708351591	-2.747147842	-6.069147084
306	0.441790425	-5.389064450	4.389935246
307	-0.580558063	-7.469148801	5.256191390
308	1.829625179	-7.526707042	4.461424894
309	2.364848675	-7.076610229	6.084723649
310	-4.569761797	0.930465395	-7.060349843
311	-1.253531965	-9.404423898	7.411747194
312	-3.440023223	-11.120770387	6.099610318
313	0.097989228	-1.097051159	-4.051515586
314	-2.406134079	-11.172072437	8.830786536
315	-3.842189592	-10.619445331	8.269853570
316	2.671691700	-5.531200378	3.381983569
317	0.673893854	0.976676501	-5.270402904
318	-3.933117669	-0.879717284	-5.538890299
319	-0.122889659	-12.544538414	1.507350779
320	0.441032923	-10.754479249	-1.207860361
321	0.168954302	-10.190292080	2.515173624
322	-1.169489188	-12.772599380	0.381663476
323	5.457409360	-3.091965704	-9.082387121
324	1.967877120	-7.738879781	1.678299007
325	-2.904227476	-7.040824913	2.690611892
326	1.396955137	-5.319190083	-6.544892549
327	-3.238097834	-9.003394358	1.454588707
328	-1.801810705	-8.757728228	-5.047267697
329	-1.521160745	-6.695473337	-3.599734029
330	-2.555219094	-12.443792023	6.867708783
331	3.748837010	-3.685046444	2.433901946
332	3.030522280	-9.257295189	3.129931690
333	3.470551919	-7.361778873	1.597708680
334	2.794330409	-9.919348381	5.380337508
335	2.577773945	-3.590601034	6.381585987
336	-2.064674173	-9.472633711	-1.336802758
337	-1.345334496	-10.120683464	0.142372301
338	-3.780827567	-8.245451039	-0.049541944
339	-0.199905096	-3.911173152	-5.364261881
340	-1.040890735	2.091313372	-6.726600421
341	-3.411182055	2.200206139	-7.538227442
342	-1.787465904	-5.772396793	2.141028882
343	2.156256052	-10.537936861	3.281596683
344	0.078614069	-11.296448266	3.591354862
345	3.079454771	-2.144494176	5.593418361
346	-0.353499087	-10.712881623	-4.236444362
347	1.370519907	-9.307327616	-0.860018802
348	-1.401661432	-4.316521233	-6.626331492
349	5.955631239	-0.992196578	-6.926810412
350	6.144860528	-3.488626044	5.076336654
351	6.417650736	-3.635470457	3.323200139
352	2.911050645	-12.135991498	-3.536928351
353	1.522471596	-12.483342348	-2.744069844
354	2.741071752	-13.148942919	1.597376024
355	1.593971864	-12.168934747	-0.103873484
356	4.489936576	-13.721600887	-0.441217249
357	3.697112257	-11.654491197	1.695308920
358	8.347494123	-5.480421068	3.741780371
359	5.723256703	12.743472413	0.679803289

360	3.721995599	-10.036091677	8.238224915
361	4.657879279	-8.019218447	6.589455866
362	3.842290208	12.533685527	-1.694534947
363	3.085021664	11.185777808	0.968749340
364	2.267366705	12.589978100	0.253124702
365	-0.930344291	8.670837045	-3.792950265
366	1.852687252	-2.297492779	1.291844681
367	-0.961489427	-3.308999139	-0.011354247
368	0.479595360	-1.385661673	0.958257425
369	0.365162872	-6.201632223	-0.891295924
370	1.172923015	-2.028774708	0.571356392
371	-9.877575507	-1.400699516	-0.075298693
372	-9.711905782	2.132119887	1.146459783
373	-9.497091307	2.262280862	3.948378253
374	-7.924130435	0.023991367	4.583195215
375	-5.440938431	0.865338973	3.475347461
376	-4.897038174	3.057915014	5.245757206
377	-4.949046889	1.977448297	7.958822845
378	-3.961459375	-0.738643812	7.963101127
379	-1.719149224	-0.514611746	6.265290763
380	-0.058750350	2.046956308	6.431182033
381	-0.331218807	2.935183058	9.199005915
382	-2.842419068	-10.653984109	8.064899669
383	-0.788206668	-9.771312803	6.575812198
384	2.499701051	-5.030374143	4.259872040
385	3.006463011	-3.158286441	5.571051782
386	3.602087221	-3.193006439	3.339250384
387	2.759909890	-9.824961463	6.419954830
388	-6.400816135	0.479290306	-3.175230370
389	-4.925745751	-3.940016145	-2.339954983
390	-5.027193155	2.711163981	-2.921851986
391	-6.156041459	5.212442978	-2.402575027
392	-6.007267818	5.421205178	0.399782385
393	-3.250338178	5.581896986	0.972206013
394	-0.873989076	3.407491915	-1.776438230
395	-1.690833808	1.645451598	-0.686707177
396	-2.070996334	8.084220453	0.838354824
397	-2.901392881	12.162653902	-1.471573669
398	-1.790490228	13.787140424	-0.447077393
399	9.033257342	-3.006498845	3.336587854
400	9.369430120	1.700488214	-0.587360301
401	8.528618758	3.339554503	-3.602647654
402	6.040233301	4.297665333	-4.370434472
403	1.471244806	5.696274891	-5.976506926
404	2.366989748	6.902949222	-7.777014602
405	1.569823581	8.015056520	-5.910453454
406	4.242759336	4.214341777	-2.084644288
407	5.651548842	5.927183960	-0.382691161
408	6.211510253	8.451836847	-1.410248035
409	4.855041573	8.340651169	-5.101231649
410	4.943897126	10.897167125	-0.948319744
411	0.316226646	-8.541094192	-8.464336649
412	0.404374570	-5.323462792	-6.794991868
413	1.284262648	-2.399851100	-6.946246280
414	4.146761877	-2.099623331	-7.062391367
415	-3.109248481	-3.804123343	-9.495408126
416	-3.215071428	-1.077288610	-8.547280676
417	2.038481356	-11.721825095	-3.195850000
418	-0.131263067	-7.909911336	-2.481429864
419	-1.154017399	-8.703786272	-4.265849268
420	2.599723681	-11.976103273	-0.104090416
421	-8.332186202	0.374954873	1.590433959
422	-7.504394261	3.348369029	3.744161407
423	-7.494346652	0.935890954	6.635427062
424	-4.456533884	-0.302576404	5.159231749
425	-2.692868556	2.678843028	4.828866460
426	-3.186127729	3.387147958	8.337268168
427	-3.157479209	-0.343947393	10.072667202
428	-0.850496045	-2.433222982	7.188841419
429	1.563985303	0.442488452	6.529405918
430	1.933234313	3.019538719	9.160901148
431	1.008315798	1.703111965	12.290404252
432	-0.899621141	-11.553442591	5.110783882
433	1.477313100	-9.361481194	8.270920087
434	6.229614701	-9.201786964	7.128945371
435	-5.170809213	2.729008213	-0.628796337
436	-5.589240075	-2.201716184	-3.645175458
437	-4.075394188	6.060843005	-2.001619884
438	-3.047119618	5.201572242	-4.778132001
439	-6.837451511	7.560632117	0.292244155

	440 -3.676582240 5.759303214 3.208809586<br>441 -0.390669873 7.373794218 2.202076917<br>442 -0.621418008 10.744202018 2.650916755<br>443 8.351373406 -5.437867523 5.783433016<br>444 5.526015216 2.453079512 3.513058082<br>445 7.974400020 4.144837046 -1.539469381<br>446 6.937901310 6.294876989 -5.069810414<br>447 3.306769876 6.059413997 -3.052044653<br>448 3.685123577 6.475120399 0.663789514<br>449 5.040450633 3.149231756 0.688683528<br>450 7.712955364 8.867361990 0.267145937<br>451 6.506250042 12.016324838 -2.171895676<br>452 4.278386059 10.200345032 -3.904829350<br>453 4.547849453 14.304383811 0.089472303<br>454 -0.169270630 8.195104339 -3.407764897<br>455 -1.378617689 -6.640506692 -7.402692931<br>456 -0.606404385 -2.508240214 -8.235656302<br>457 3.900498258 -0.490262441 -8.646092374<br>458 7.285838488 -2.216113915 -8.841444306<br>459 -5.437038563 -1.239405638 -9.036508420<br>460 -5.202927673 -5.714240320 -4.829717995<br>461 -3.146232555 -6.323219097 -5.446089565<br>462 -1.578144512 2.006679768 -9.352137489<br>463 4.440117820 -10.994645955 -0.988769175<br>464 5.577955740 -13.442155306 1.264072748<br>465 -3.583468196 -5.270220404 -0.011358376<br>466 -4.655274411 -5.278598465 1.965224686<br>467 -2.656283232 -12.188183302 -0.305526951<br>468 -4.438772500 -10.839838093 -0.650186185<br>469 0.456846883 -7.829884263 0.602233237<br>470 -0.619824444 -8.664257490 2.390275184<br>471 -1.652208029 -6.685497762 -0.412623999<br>472 2.995706284 -10.116414412 3.618235894<br>473 2.780749027 -7.621236734 2.237225077<br>474 -0.491070513 5.629559561 -3.888727291<br>475 -0.356541806 -13.188364014 0.801810654<br>476 0.558214291 -11.075788765 2.753487402<br>477 5.830949801 1.218573183 -2.635845323<br>478 2.575002938 -0.639875609 -3.342857028<br>479 -0.489621921 -4.563201719 -2.358473972<br>480 3.767839074 -0.655680191 -0.100678580<br>481 -0.193511793 -1.471232578 -1.608923088<br>482 2.722821851 -4.260220971 -4.653803457<br>483 0.837942598 -4.875697538 1.000757147<br>484 5.502647551 -2.596875286 -2.602688427<br>485 2.572844254 -6.540518039 -1.707995746<br>486 4.617188264 -4.485402295 0.605100217<br>end

TS

Fe(139) 2.497<br>Fe(140) -1.097<br>Fe(141) 2.693<br>Fe(142) -2.473<br>Fe(143) 3.080<br>Fe(144) -2.021<br>Fe(145) -1.245	9651826nh26hxtontn_1_52966.47<br>!DATE Jan 02 11:14:07 2025<br>1 -9.366312084 0.818363505 1.037617800<br>2 -10.357193178 -0.054487484 0.258297890<br>3 -8.803822364 3.107140123 1.737479850<br>4 -8.537265472 2.911304830 3.226560559<br>5 -9.257971248 1.927772265 5.341463098<br>6 -8.129654851 0.914258337 5.567939644<br>7 -6.750488735 -0.856655362 4.605687561<br>8 -5.440482359 -0.084552422 4.430459230<br>9 -4.276760419 1.720403379 3.257703001<br>10 -3.875763764 2.528076185 4.500657675<br>11 -4.556662518 3.849262399 6.410521088<br>12 -4.128344814 3.027035766 7.634830862<br>13 -4.720982731 1.211302432 9.177233787<br>14 -3.835125261 -0.038654015 9.094827603<br>15 -3.113752719 -1.935151421 7.715103798<br>16 -1.775320052 -1.653129972 7.025964676<br>17 -0.525562476 -0.209661392 5.493652439<br>18 0.417365759 0.784377230 6.195433441<br>19 -0.829832005 0.142974594 4.018140420<br>20 0.444977491 0.544740056 3.264938698<br>21 -1.520973475 -1.032936578 3.316235322<br>22 0.834204769 3.056380817 7.003593743<br>23 0.879662687 2.996572721 8.542788503<br>24 0.521355260 4.463029182 6.440276728<br>25 1.419771823 5.522295862 7.089399232<br>26 0.696583758 4.474556712 4.915620460<br>27 -0.285739469 2.855812899 10.662373102<br>28 0.565562903 1.701684783 11.160105311

	29	-2.603558080	-11.350411919	6.800561933
	30	-1.335391433	-10.890097663	6.085605826
	31	0.420083932	-9.135785075	6.087137439
	32	1.586936311	-9.447030168	7.042507175
	33	0.249166695	-7.612522753	5.940726212
	34	1.514683326	-6.965691659	5.380168968
	35	1.340701354	-5.491503524	5.051129483
	36	3.057385168	-3.792076276	4.416067025
	37	3.956592734	-10.049580245	7.177964773
	38	5.029584427	-9.011367408	6.936796873
	39	-5.826915370	0.694919192	-1.843815723
	40	-5.323560345	2.145681925	-1.714732305
	41	-4.657184609	-0.276724617	-1.585440424
	42	-5.072058913	-1.736022552	-1.307352380
	43	-5.216177970	-2.634385958	-2.536601201
	44	-4.211845447	3.904155756	-3.069713960
	45	-4.820811596	5.164521008	-2.445340911
	46	-3.933237655	4.083486584	-4.568981359
	47	-6.867566290	6.310679114	-1.742742184
	48	-6.569140936	6.489470712	-0.249656507
	49	-5.602884405	5.536744010	1.789217197
	50	-4.097361123	5.640886860	2.045940887
	51	-1.818495288	5.681948916	1.151120107
	52	-1.376597090	7.116012107	1.482389268
	53	-1.053112888	5.275796887	-0.118800307
	54	-1.259067012	3.860167476	-0.538276232
	55	-1.775139448	2.749942585	0.097200619
	56	-1.209907717	2.075564439	-1.864249526
	57	-1.649960236	9.466167423	0.833757056
	58	-1.456626645	9.914068786	2.280376415
	59	-2.776397978	10.287538570	0.161020619
	60	-2.445789682	11.689652374	-0.259338207
	61	-1.749797064	12.691989291	0.388075118
	62	-2.509886196	13.418469527	-1.562672123
	63	8.189462808	-3.393008726	4.494544895
	64	8.281878314	-4.894473511	4.699894864
	65	6.698453603	-3.075735746	4.234726775
	66	6.409278529	-1.600596227	4.074580625
	67	6.062481702	-0.801202039	5.179517690
	68	6.462571495	-0.986061452	2.808977430
	69	5.769271266	0.558433319	5.030727548
	70	6.183125772	0.371156503	2.646703218
	71	5.831005019	1.141987194	3.758038501
	72	8.420567222	1.745670811	-1.708815487
	73	8.281318627	3.173987586	-2.263557922
	74	7.060470966	1.237057584	-1.241207367
	75	8.467518427	4.612098638	-4.265397244
	76	7.067328078	5.132866888	-4.598083607
	77	4.631143604	4.635616041	-4.494589933
	78	4.009848323	5.029600134	-3.131244299
	79	3.918626523	3.430711468	-5.148085140
	80	2.394874378	3.466509391	-5.311243351
	81	1.840065301	4.351948395	-6.438069244
	82	1.786606137	6.843149740	-6.563144653
	83	3.685302808	4.536896489	-0.763293677
	84	4.345128421	5.756796568	-0.101031049
	85	3.749746933	3.357922802	0.224459195
	86	6.485209584	6.828584687	0.419126570
	87	6.866898426	8.139309996	-0.251233790
	88	6.645683736	9.588709939	-2.193394187
	89	6.004620323	10.938623200	-1.788466171
	90	6.541185999	9.378721747	-3.709056421
	91	5.110980899	9.331265749	-4.232711541
	92	4.109491164	12.067799497	-0.735062010
	93	4.864719642	13.119569452	0.062762709
	94	2.807824142	11.662612535	0.010767410
	95	1.949810575	10.742763600	-0.827242784
	96	1.036980825	11.269543748	-1.752905269
	97	2.085065572	9.346375214	-0.753289471
	98	0.295525648	10.438240566	-2.594678988
	99	1.373018472	8.499579774	-1.607539671
	100	0.487590086	9.056562852	-2.525859181
	101	0.827360861	-7.626288612	-7.456058554
	102	-0.143230797	-6.478757196	-7.228588381
	103	-0.335894905	-4.132058957	-6.431141103
	104	0.094739455	-2.931314621	-7.282421015
	105	1.880382011	-1.286462390	-7.654820042
	106	3.396443099	-1.277858777	-7.826033725
	107	5.605508248	-1.999502652	-7.120321555
	108	6.162062166	-2.472228329	-8.448969958

109	-4.248416311	-3.304980654	-8.694000863
110	-4.367899202	-1.778583789	-8.777076702
111	-4.296769407	-3.770439710	-7.221449112
112	-4.576208392	-5.283310003	-7.132432837
113	-4.358831540	-5.792273738	-5.725910465
114	-3.240882695	0.382124017	-8.636230629
115	-2.022289613	0.878634289	-9.387273860
116	-3.539506456	1.132779276	-7.315615824
117	-2.590873342	0.662614822	-6.253813618
118	-1.306193444	1.218265918	-6.150835358
119	-2.908548519	-0.478112572	-5.498094660
120	-0.327622705	0.580864035	-5.384174040
121	-1.930360720	-1.108533431	-4.729389756
122	-0.627840378	-0.602159837	-4.707010218
123	2.362037728	-10.674049569	-2.202138444
124	3.213334268	-11.204925412	-1.036761011
125	1.082537376	-10.005381916	-1.679603295
126	0.260348665	-9.256100166	-2.678916000
127	-0.373666501	-9.758518440	-3.796606915
128	-0.997621399	-7.656855875	-3.487233176
129	3.375103643	-12.468372716	1.023902340
130	4.586835842	-13.251494396	0.569166986
131	-3.716248527	-5.639882024	1.231594764
132	-2.578843452	-6.500731384	1.778350148
133	-1.945324065	-7.461934923	0.730543157
134	-2.909186575	-8.651118637	0.452839819
135	-2.305220837	-9.818839995	-0.332578553
136	-3.220374708	-11.059379684	-0.439211796
137	-0.635681093	-8.037901481	1.286966848
138	2.428836117	-3.260323025	-1.520974602
139	1.776580238	-4.712148255	-2.735452467
140	1.900107847	-1.429635450	-1.217672513
141	4.169094278	-2.790953847	-0.718517108
142	3.342825170	-2.709492526	-3.175429324
143	4.487494977	-0.554211296	-2.198273462
144	2.667556550	-4.857058208	-0.364190783
145	0.418141563	-3.422182941	-0.599058911
146	-9.578148610	-1.873761962	0.754852091
147	-9.103376215	-1.342546977	-0.713310710
148	-10.497101549	2.479633440	0.601384964
149	-10.656322823	0.478509179	-0.658151602
150	-11.267716531	-0.124719893	0.878402357
151	-7.823381808	3.082687130	1.238840231
152	-10.320781141	1.931536357	3.469650693
153	-9.230455500	4.108754848	1.591316596
154	-10.188773714	1.521165391	5.759010136
155	-8.399165786	0.126527560	3.680401113
156	-8.990700905	2.826368643	5.912264824
157	-3.403957625	1.122396609	2.968664826
158	-6.245177588	0.962064710	2.854196710
159	-6.850402830	-1.600774483	3.803764399
160	-3.730241915	4.535083554	6.182690190
161	-5.839194217	3.037844102	4.876472921
162	-6.696264116	-1.384777504	5.565231400
163	-4.505251339	2.403802627	2.428095125
164	-5.733476768	1.752443630	7.367953462
165	-5.439316252	4.443560126	6.684080349
166	-2.448189172	-1.331862817	3.825086274
167	-1.780299140	-0.763894958	2.279625538
168	-0.855853395	-1.912083874	3.279326474
169	-1.504042288	1.012809545	4.016190056
170	-5.706104834	0.898166207	9.553043341
171	0.036049828	3.752819641	4.415616665
172	-1.200961405	2.944334081	8.723952721
173	1.263433670	5.592610099	8.175349392
174	0.197814273	0.801140772	2.223042776
175	1.181247176	-0.274446211	3.254553130
176	8.484578197	-2.909414287	5.445634846
177	0.475086833	5.472114615	4.508690660
178	1.217653105	6.509232696	6.647463384
179	1.737624882	4.224952178	4.649245649
180	2.481967626	5.283425945	6.917927572
181	-0.531027419	4.703060749	6.678423889
182	1.857196937	2.783762236	6.704460264
183	-0.971993773	2.311329243	6.084116898
184	0.931220717	1.425625279	3.708415120
185	3.402973567	2.438904257	-0.279397962
186	3.055769675	3.587389550	1.047510257
187	5.043266775	3.002773332	1.677780966
188	5.245245932	2.886542228	4.370879395

189	6.195993851	0.827735963	1.657068615
190	6.693197340	-1.587066990	1.926512694
191	6.008694484	-1.249183029	6.175416037
192	-2.584191780	-0.029075775	6.054658639
193	-3.664504912	-2.655818723	7.092420565
194	-2.883398524	-2.417880194	8.672238721
195	-4.498316026	-0.426817846	7.168324674
196	-4.258116167	1.869952298	9.921989292
197	5.478193148	1.158683520	5.897117931
198	0.067317146	-1.136792191	5.497037069
199	0.071440485	3.789124794	11.126512823
200	-1.316651899	2.675973202	11.003976685
201	0.673588046	0.854190019	10.442800314
202	4.367050808	-11.051141787	6.976029840
203	-5.031369051	-4.583451439	-3.134030170
204	-2.682343935	-5.811004646	-0.397198940
205	-5.624520577	-5.471051459	-7.406594241
206	-3.931851109	-5.829755070	-7.836679822
207	-5.086202124	-3.230530437	-6.675797195
208	-1.544047540	0.117867984	-10.047761501
209	-5.169695229	-3.660850479	-9.180541190
210	-3.971594404	-0.238413527	-2.446244876
211	-6.040416289	-1.762760003	-0.776545400
212	-2.353971834	-1.546396000	-8.320684694
213	-4.078733690	0.614146654	-9.327407154
214	-2.222473620	-3.601137029	-9.036275223
215	-4.557841585	-4.298510422	-1.461535650
216	-3.345443208	-3.512677954	-6.731020543
217	-3.188353373	-4.799478149	-9.621520183
218	-7.408066409	0.658277858	-3.179622283
219	-4.080480129	0.094505091	-0.727447065
220	-2.555489297	-6.347511116	-6.273341689
221	-4.343112263	-2.193258323	-0.622383314
222	-1.518265056	5.058123745	2.004680770
223	-2.182683727	2.691634215	1.102334314
224	-6.059609072	6.445185762	2.205826836
225	-5.811326275	4.556982230	-0.101575266
226	-2.872964834	7.807595098	0.258817980
227	-1.219247382	12.720469897	1.333429102
228	-2.195719401	9.466545908	2.987794069
229	-3.660523249	10.276821502	0.823413872
230	-0.699947643	9.600260921	0.292636042
231	-2.698187837	14.099905175	-2.388922245
232	0.014291456	5.470338131	0.080182180
233	-1.349399102	5.950964280	-0.939344602
234	-1.370784517	14.686645719	-0.285231170
235	-3.073105490	9.752578460	-0.754726452
236	-3.641695769	5.487540226	0.024689671
237	-5.983060433	4.690193966	2.381257324
238	-6.617410899	7.268300512	-2.220243236
239	-1.024597148	1.468205871	-2.746766374
240	-7.947650633	6.151529309	-1.857616596
241	10.000571148	-3.038531091	3.607725516
242	8.811760605	-2.048272010	3.082356050
243	4.745178729	3.304056807	-2.191346779
244	2.628053308	4.806721058	-0.905391602
245	6.164798409	5.178842893	-0.863480134
246	-0.544353553	4.015051992	-2.549621531
247	-1.431669720	5.472796563	-4.182210079
248	-3.050081836	5.395185980	-5.726910649
249	-0.477405995	6.512041201	-3.469162889
250	-3.467278601	3.155765748	-4.941445286
251	-4.882601003	4.248682404	-5.103430138
252	7.111423376	8.484906205	-3.996063252
253	7.022700667	10.249113277	-4.181937412
254	5.501338435	7.534015097	-5.182279480
255	3.867278467	8.184333628	-5.331837606
256	2.828611636	7.750604278	-8.080497776
257	0.944634830	3.884711117	-6.876149968
258	-0.423716871	10.865039543	-3.297807937
259	2.744139328	8.887612228	-0.010745027
260	1.065386512	7.995862945	-5.019711668
261	4.159617922	2.541592883	-4.544161042
262	6.258987362	3.361048839	-3.971988948
263	1.897419246	3.716916726	-4.361143517
264	-6.696763488	4.400956332	-2.711224095
265	9.034505943	4.554186611	-5.203853476
266	-5.259343428	2.083893901	-3.702364437
267	4.387053816	3.267397218	-6.132829265
268	1.534024380	7.421514802	-1.573771589

269	8.940688619	5.379065035	-3.635035973
270	2.551760384	6.054699772	-8.307477513
271	1.441627491	8.853352055	-6.471817416
272	-6.229810413	-0.494676327	-3.474542173
273	-6.560252172	0.566342763	-1.024970455
274	0.891496023	12.350762507	-1.814370877
275	-3.255612876	3.759427851	-2.544854694
276	4.560659597	5.527707750	-5.125108143
277	2.583852733	4.429770689	-7.241856160
278	2.095785406	2.435872056	-5.544431987
279	0.807786541	5.724145628	-5.169408453
280	5.944585066	7.082916191	1.342942383
281	5.603432316	7.732345481	-1.815849221
282	7.711497013	9.733725296	-1.958931795
283	7.421820867	6.328385542	0.702005309
284	4.516366496	9.973307545	-0.802246018
285	1.803062726	-7.228084984	-7.771941817
286	0.978806596	-8.092922656	-6.460520381
287	2.989978724	-9.915891025	-2.693515351
288	0.810462497	-9.429875837	-8.407034182
289	-0.670292330	-8.741955178	-8.261468026
290	7.179656505	0.213849915	-0.863991250
291	9.098834333	2.405039373	0.100616313
292	8.760654107	2.500954947	-4.145618229
293	6.668313971	1.880456401	-0.441900044
294	10.302612631	1.968219590	-0.915541494
295	6.025950604	-2.647851517	-6.335604553
296	8.795101523	1.079147619	-2.501113307
297	-0.015142917	-7.178243957	6.919511906
298	0.623030597	-9.563917024	5.097254577
299	3.748401837	-2.702636649	-6.334464639
300	4.248495168	-2.397746726	3.544246895
301	1.435159531	-1.247808941	-8.656528415
302	-2.173627539	-1.995586567	-4.141296548
303	1.614454359	-0.339874926	-7.153024740
304	1.218360363	-4.894869715	5.963037745
305	1.729057636	-2.775053687	-6.081014049
306	0.435117190	-5.329525195	4.441993443
307	-0.596432083	-7.438853392	5.256512809
308	1.807180772	-7.487132009	4.457840060
309	2.352084055	-7.070638873	6.086813615
310	-4.584820924	0.929647238	-7.040275825
311	-1.270622242	-9.384633400	7.406916974
312	-3.435949969	-11.133767748	6.113247021
313	0.138412080	-1.105275865	-4.119093008
314	-2.382207131	-11.167116371	8.838634799
315	-3.835656356	-10.648902579	8.290325065
316	2.642849895	-5.485174046	3.389012633
317	0.679071505	0.994207148	-5.307479096
318	-3.919674062	-0.889359249	-5.530787260
319	-0.129825656	-12.549966247	1.513622655
320	0.435434481	-10.775927786	-1.225966282
321	0.161877621	-10.198441070	2.508697380
322	-1.173289218	-12.783439581	0.385209108
323	5.468471603	-3.102175319	-9.056423292
324	1.951549367	-7.759685057	1.665984292
325	-2.936294231	-7.054257710	2.654721603
326	1.404417696	-5.326285392	-6.541761456
327	-3.246028045	-9.021181123	1.433919312
328	-1.799911277	-8.796801582	-5.071828268
329	-1.528855280	-6.726063065	-3.638886147
330	-2.536311357	-12.449198036	6.877549116
331	3.751406635	-3.658200076	2.456543484
332	3.020422166	-9.254981114	3.138265886
333	3.439602352	-7.323080438	1.615134075
334	2.779486468	-9.906696921	5.389399759
335	2.622295423	-3.596404250	6.417837851
336	-2.076340802	-9.492979129	-1.358415915
337	-1.357105298	-10.136611597	0.122769316
338	-3.799258052	-8.267961208	-0.068644836
339	-0.188893545	-3.915013151	-5.361638834
340	-1.064555574	2.118375322	-6.720879225
341	-3.437445804	2.208971620	-7.520560929
342	-1.805194170	-5.793127570	2.122335669
343	2.148578286	-10.536863195	3.290490639
344	0.072138260	-11.290925256	3.599688779
345	3.132789239	-2.146429893	5.646856780
346	-0.353139673	-10.745141894	-4.243631230
347	1.346385796	-9.320792098	-0.865167880
348	-1.394147219	-4.321680912	-6.620289311

349	5.960198342	-0.974760012	-6.929932289
350	6.119920257	-3.476743437	5.081029406
351	6.391449570	-3.618764125	3.326732699
352	2.948124013	-12.120489715	-3.533609529
353	1.555423955	-12.488070103	-2.755847054
354	2.735180343	-13.159894399	1.594075821
355	1.591152357	-12.164814257	-0.100693902
356	4.488778004	-13.714996230	-0.442500488
357	3.688899781	-11.664202473	1.708108053
358	8.316650775	-5.486455275	3.754099866
359	5.717882661	12.737395675	0.670413010
360	3.698959581	-10.004135011	8.251375564
361	4.661047707	-8.020376268	6.574288059
362	3.834711232	12.523860318	-1.701544670
363	3.076108926	11.182483160	0.964703746
364	2.262442342	12.587542488	0.245653177
365	-0.937080820	8.665883514	-3.797977154
366	1.775190318	-2.203864368	1.337223597
367	-0.089286729	-2.894689583	0.744657838
368	0.510271327	-1.180311184	0.893966585
369	0.383672448	-6.227546539	-0.918653502
370	1.122040389	-1.941195415	0.588479778
371	-9.909327357	-1.392634957	-0.085275069
372	-9.708902959	2.134687101	1.144017042
373	-9.482847213	2.259180131	3.944265900
374	-7.915528487	0.015700160	4.574190950
375	-5.435091383	0.862327141	3.463840409
376	-4.882878123	3.053641080	5.238319507
377	-4.911173625	1.957938189	7.941424345
378	-3.950098987	-0.774738012	7.957651777
379	-1.717703088	-0.521079648	6.266952137
380	-0.052388722	2.039475490	6.439036002
381	-0.300997089	2.928034418	9.209984879
382	-2.837289843	-10.661021869	8.075710741
383	-0.803110461	-9.750104889	6.571655415
384	2.497759289	-5.002821585	4.281183775
385	3.055335365	-3.159681948	5.611771343
386	3.598352981	-3.163073772	3.364014433
387	2.742161288	-9.806564853	6.428478146
388	-6.400651418	0.483907501	-3.180700561
389	-4.933748288	-3.937823829	-2.344450884
390	-5.038816279	2.717130962	-2.917190753
391	-6.174562454	5.216670532	-2.398352799
392	-6.022748758	5.427257735	0.403937194
393	-3.263008130	5.578538706	0.969611006
394	-0.899864785	3.411820098	-1.798128740
395	-1.742656251	1.648932349	-0.729457709
396	-2.073454541	8.077837164	0.826924529
397	-2.916591613	12.157972626	-1.476785982
398	-1.797567949	13.780305127	-0.457651585
399	9.012470782	-3.017663049	3.343324880
400	9.372308899	1.694577660	-0.586210201
401	8.530858103	3.325524461	-3.597391706
402	6.043856501	4.287201647	-4.363280961
403	1.470444834	5.692940232	-5.974341603
404	2.372588624	6.897066828	-7.773644129
405	1.565096498	8.012382651	-5.913497960
406	4.258814758	4.211635804	-2.070011323
407	5.670478752	5.929513965	-0.374633188
408	6.210326107	8.445305287	-1.411269642
409	4.852779873	8.329030232	-5.115408984
410	4.935765164	10.887911029	-0.954499850
411	0.310796022	-8.540810188	-8.476839277
412	0.411024325	-5.330021510	-6.790625140
413	1.295311088	-2.412850283	-6.949408196
414	4.156836624	-2.091349905	-7.049553779
415	-3.119215436	-3.786787815	-9.501079637
416	-3.230872739	-1.064720201	-8.543828426
417	2.067128690	-11.718961962	-3.199538847
418	-0.136017220	-7.930015889	-2.506800429
419	-1.154475164	-8.736583386	-4.288566185
420	2.596759087	-11.971977872	-0.097329658
421	-8.346046844	0.364281771	1.586881681
422	-7.488124448	3.340698867	3.736894298
423	-7.476004420	0.927275957	6.624629844
424	-4.450632741	-0.301410501	5.151406981
425	-2.681824491	2.682064383	4.803128546
426	-3.154783345	3.375670689	8.324323603
427	-3.113556411	-0.358529943	10.049788789
428	-0.833145200	-2.447562055	7.160458356

	429	1.569688803	0.431995156	6.497442641
	430	1.963292451	3.006428335	9.153986817
	431	1.051028739	1.658091373	12.280613843
	432	-0.889533484	-11.546669890	5.122365130
	433	1.457128557	-9.323331114	8.272467834
	434	6.217638415	-9.212191561	7.134514111
	435	-5.189338273	2.731582916	-0.624052745
	436	-5.599845907	-2.198581725	-3.646616219
	437	-4.096011883	6.070334141	-1.998437095
	438	-3.057694712	5.209126521	-4.771103839
	439	-6.852535431	7.566634918	0.295082000
	440	-3.684125867	5.771700078	3.205803491
	441	-0.406691508	7.365645816	2.207219988
	442	-0.634328766	10.737400039	2.647394513
	443	8.290233281	-5.438434314	5.795079883
	444	5.535552414	2.471953448	3.537340701
	445	7.973064498	4.135436454	-1.537675460
	446	6.942854347	6.282933624	-5.066106642
	447	3.315829768	6.054154207	-3.037040357
	448	3.703083860	6.487882401	0.663980162
	449	5.081371874	3.180765215	0.717453523
	450	7.715695314	8.882366911	0.257418109
	451	6.492557301	12.006365638	-2.185386905
	452	4.267442079	10.181019158	-3.910532543
	453	4.540693933	14.296820450	0.079250444
	454	-0.174826461	8.191263393	-3.413825629
	455	-1.375052467	-6.636175316	-7.412175110
	456	-0.611130002	-2.501138450	-8.219537543
	457	3.905216569	-0.493791928	-8.645226883
	458	7.297425452	-2.225386574	-8.824034456
	459	-5.453483774	-1.228505354	-9.030626880
	460	-5.200072469	-5.707868274	-4.838160539
	461	-3.145352797	-6.319669804	-5.458308563
	462	-1.607575902	2.028441843	-9.343927674
	463	4.439928715	-10.989445101	-0.975626656
	464	5.574985996	-13.442210271	1.264838231
	465	-3.576211959	-5.270876269	-0.044561211
	466	-4.662003639	-5.267434366	1.925397543
	467	-2.661576861	-12.206837776	-0.310891545
	468	-4.445431952	-10.866244340	-0.678626977
	469	0.439296078	-7.855062723	0.563793724
	470	-0.633863511	-8.670443291	2.360836445
	471	-1.664647613	-6.701632040	-0.445431506
	472	2.985622110	-10.112619216	3.629183288
	473	2.751544649	-7.626597207	2.237270900
	474	-0.496455142	5.624975224	-3.890921035
	475	-0.361299480	-13.196130311	0.809535411
	476	0.551120470	-11.080070242	2.758959663
	477	5.831656693	1.235800107	-2.620632551
	478	2.541235256	-0.613611235	-3.287890892
	479	-0.470765164	-4.551415041	-2.344032618
	480	3.696062341	-0.657954789	-0.018281240
	481	-0.271528167	-1.534990187	-1.661301409
	482	2.765348181	-4.242217508	-4.679517894
	483	0.883471793	-4.933595891	0.980658215
	484	5.476775031	-2.578263284	-2.574117360
	485	2.586868151	-6.571982855	-1.764089866
	486	4.577024630	-4.500403115	0.654742842
	end			

product

	9651826nh26hxtontl.car_7			
Fe(139) 2.357	1	-9.378015751	0.826198025	1.049189230
Fe(140) -2.330	2	-10.345953724	-0.060189016	0.256154346
Fe(141) 2.776	3	-8.837925197	3.118429264	1.748183747
Fe(142) -2.386	4	-8.564416421	2.919237088	3.235512317
Fe(143) 2.932	5	-9.281157510	1.935997276	5.352501264
Fe(144) -2.279	6	-8.149583030	0.926568105	5.580626703
Fe(145) 0.849	7	-6.766081478	-0.845676010	4.624295890
	8	-5.452250304	-0.080715248	4.443939437
	9	-4.286896882	1.724630133	3.277420207
	10	-3.893383170	2.531313486	4.523457650
	11	-4.593367820	3.862876327	6.421442650
	12	-4.173432912	3.045310881	7.651636774
	13	-4.771208553	1.238374121	9.199948891
	14	-3.877369457	-0.005959344	9.117154442
	15	-3.135824645	-1.890757417	7.726709968
	16	-1.791260055	-1.626706390	7.041288316
	17	-0.519974037	-0.202519639	5.499234359
	18	0.411931166	0.795169726	6.212475652

19	-0.821703007	0.156240260	4.030105616
20	0.458370244	0.553714130	3.283632805
21	-1.533935438	-1.018032729	3.349246247
22	0.812868022	3.077659012	7.000822377
23	0.846011546	3.011070068	8.539736300
24	0.484701363	4.485608940	6.449572187
25	1.379994135	5.544629958	7.104008178
26	0.648975027	4.515209909	4.924082517
27	-0.346954912	2.870762115	10.643968721
28	0.518523975	1.738369762	11.163736274
29	-2.614421377	-11.352892618	6.802802532
30	-1.342370058	-10.907871612	6.086109215
31	0.438197729	-9.180637345	6.091305886
32	1.610177065	-9.491743314	7.039358844
33	0.271324028	-7.657714965	5.938502237
34	1.533262879	-7.010547487	5.370177678
35	1.331292576	-5.549326932	5.000675658
36	3.020378702	-3.817170171	4.389006556
37	3.986857925	-10.068820920	7.154847272
38	5.041894206	-9.009079745	6.931657260
39	-5.841415708	0.699848615	-1.844584915
40	-5.326188408	2.145778253	-1.721366647
41	-4.665233972	-0.266813539	-1.590364786
42	-5.066301349	-1.728492699	-1.307128009
43	-5.212843150	-2.629049255	-2.534379351
44	-4.195189073	3.889625318	-3.077352205
45	-4.795322750	5.153385474	-2.450602636
46	-3.918846643	4.068537177	-4.576999603
47	-6.837117258	6.310304276	-1.749499991
48	-6.544333851	6.483358261	-0.254536933
49	-5.586725004	5.521975439	1.784493497
50	-4.081972269	5.627474865	2.047062742
51	-1.800724041	5.690246388	1.158684667
52	-1.363046174	7.124421301	1.489718879
53	-1.031572696	5.287039858	-0.109605568
54	-1.215544895	3.863287545	-0.509033341
55	-1.712321005	2.755724196	0.146063139
56	-1.159580082	2.062750884	-1.812211012
57	-1.639945742	9.471684078	0.844522532
58	-1.437070375	9.920621086	2.289665629
59	-2.765785539	10.294837398	0.172627486
60	-2.433765418	11.696655557	-0.247331399
61	-1.744833632	12.700417140	0.405078625
62	-2.488599646	13.424134189	-1.552690093
63	8.205675643	-3.392440257	4.461721590
64	8.323198975	-4.892790000	4.664926084
65	6.709420451	-3.095238420	4.203321333
66	6.405073491	-1.623640205	4.040677340
67	6.062781471	-0.823756906	5.146412117
68	6.446154756	-1.011119287	2.773064581
69	5.764535033	0.534851421	4.997729988
70	6.160840776	0.344997499	2.610461021
71	5.815173862	1.116871751	3.723599893
72	8.406096071	1.740386531	-1.723722434
73	8.276626599	3.173617591	-2.265533646
74	7.035808353	1.228641380	-1.280934621
75	8.456778292	4.620136512	-4.261564062
76	7.054876344	5.133062199	-4.599739898
77	4.620554220	4.627673162	-4.507918716
78	3.990740417	5.024561397	-3.149621308
79	3.912593968	3.421337108	-5.163010344
80	2.389235091	3.455925339	-5.328807748
81	1.833729228	4.350704261	-6.447665724
82	1.785098575	6.843163851	-6.563234192
83	3.648770966	4.533209506	-0.784332041
84	4.314318913	5.744077301	-0.111700139
85	3.691791777	3.349130903	0.197800743
86	6.457232859	6.805754990	0.417274750
87	6.854935510	8.115707236	-0.246587682
88	6.654241918	9.579561586	-2.182666252
89	6.019472774	10.930714795	-1.772046163
90	6.548508995	9.376797582	-3.699583163
91	5.117407208	9.337988274	-4.222517659
92	4.121853185	12.062831429	-0.725778157
93	4.878760828	13.113520465	0.071908998
94	2.819194208	11.658931906	0.019197426
95	1.960367613	10.741032735	-0.819999533
96	1.049279064	11.270493079	-1.745734661
97	2.092397263	9.344276463	-0.747026822
98	0.306321007	10.441665732	-2.588331024

	99	1.378241142	8.499633947	-1.601638227
	100	0.494051857	9.059404384	-2.519660667
	101	0.824844569	-7.627495146	-7.460493069
	102	-0.144124585	-6.479610078	-7.227961713
	103	-0.333398935	-4.136128788	-6.421646915
	104	0.094884940	-2.937551088	-7.274269750
	105	1.869832939	-1.288061614	-7.658193539
	106	3.384689959	-1.281841625	-7.828349874
	107	5.586399785	-2.023389892	-7.126792181
	108	6.147988775	-2.465187155	-8.463498439
	109	-4.250734355	-3.319532434	-8.708405807
	110	-4.364618670	-1.793270550	-8.800919754
	111	-4.297069246	-3.775772510	-7.232731376
	112	-4.578393609	-5.287616662	-7.133257242
	113	-4.360309953	-5.787783230	-5.723495052
	114	-3.227817338	0.363653168	-8.669686772
	115	-2.000940444	0.849023853	-9.414699840
	116	-3.522641514	1.116655292	-7.350253562
	117	-2.588687797	0.644595273	-6.274905171
	118	-1.301437248	1.191988314	-6.155997560
	119	-2.929661991	-0.477998375	-5.502543086
	120	-0.350601589	0.573691489	-5.340307589
	121	-1.980060643	-1.087567318	-4.681188716
	122	-0.678469884	-0.582005853	-4.628896263
	123	2.363125079	-10.695640432	-2.220683824
	124	3.216548695	-11.223019591	-1.055287021
	125	1.099670364	-10.001978268	-1.690169490
	126	0.276847302	-9.243306680	-2.682491448
	127	-0.379165779	-9.743327850	-3.788025334
	128	-0.974417506	-7.633230453	-3.483256541
	129	3.379552164	-12.469656488	1.014783612
	130	4.588985447	-13.259296986	0.565436575
	131	-3.662706449	-5.609914441	1.231070867
	132	-2.521287873	-6.471615426	1.764281802
	133	-1.898831944	-7.434221659	0.709928751
	134	-2.871957707	-8.618192413	0.443195386
	135	-2.281658489	-9.799965336	-0.331395427
	136	-3.210177540	-11.031875841	-0.418688167
	137	-0.589578739	-8.019813635	1.258896407
	138	2.411399155	-3.261491849	-1.521430867
	139	1.814114794	-4.732579553	-2.721760197
	140	2.006395271	-1.387228927	-1.705434924
	141	4.117054520	-2.686912365	-0.699527112
	142	3.483848041	-2.830464481	-3.214686651
	143	4.463532430	-0.539571148	-2.334409685
	144	2.720269647	-4.849278089	-0.328561699
	145	0.608614013	-3.385536953	-0.651943297
	146	-9.559814374	-1.867629560	0.783583357
	147	-9.055361218	-1.341508498	-0.675648699
	148	-10.520678848	2.477495959	0.603545290
	149	-10.630585255	0.463032123	-0.670684742
	150	-11.269635248	-0.136209493	0.855153061
	151	-7.858849415	3.104690626	1.246551480
	152	-10.346752410	1.938871777	3.482547086
	153	-9.274653089	4.116430073	1.606833541
	154	-10.210289365	1.524939023	5.769464593
	155	-8.410841772	0.138940566	3.691876178
	156	-9.018260342	2.835639485	5.923715201
	157	-3.413633482	1.125473636	2.992732774
	158	-6.261568797	0.978553971	2.878020497
	159	-6.869330912	-1.593587102	3.826605965
	160	-3.767031750	4.550521917	6.198986391
	161	-5.858886769	3.047138964	4.877143407
	162	-6.714610248	-1.368422678	5.587044689
	163	-4.509963585	2.407953528	2.446450551
	164	-5.790127685	1.780854052	7.394750100
	165	-5.480880339	4.454841711	6.683887332
	166	-2.493081747	-1.250056372	3.832096855
	167	-1.729508563	-0.793998305	2.289491356
	168	-0.914475124	-1.928400900	3.406642423
	169	-1.490586049	1.030686235	4.028091601
	170	-5.754624222	0.918019618	9.574163034
	171	-0.017618183	3.802598467	4.419138077
	172	-1.236820396	2.955910034	8.697040244
	173	1.224267148	5.608548577	8.190470988
	174	0.224080682	0.795963092	2.236392183
	175	1.201444497	-0.259102671	3.289534032
	176	8.496129510	-2.904838702	5.412451271
	177	0.427027958	5.518655700	4.532584769
	178	1.174841337	6.533355255	6.667612692

	179	1.687359935	4.267047876	4.646483925
	180	2.442768701	5.309787208	6.930728509
	181	-0.567791758	4.714382618	6.698233960
	182	1.840793855	2.819831040	6.705487936
	183	-0.977936667	2.319686370	6.061077251
	184	0.932009650	1.441221602	3.728943865
	185	3.329330886	2.438897435	-0.310276148
	186	3.000744551	3.586099010	1.021405364
	187	4.980199073	2.966877123	1.649248165
	188	5.235245418	2.861715210	4.340078687
	189	6.169294396	0.800709294	1.620251057
	190	6.676526934	-1.611954967	1.890274557
	191	6.019551119	-1.270183793	6.143482459
	192	-2.580499951	-0.001587751	6.056769483
	193	-3.692139130	-2.606187259	7.102751960
	194	-2.916186896	-2.374331236	8.685898537
	195	-4.508612121	-0.372629241	7.175677611
	196	-4.314219923	1.898538027	9.947083216
	197	5.479379225	1.135864096	5.865605053
	198	0.070400814	-1.130965633	5.508495001
	199	-0.017415199	3.813806102	11.109457230
	200	-1.379559810	2.672560263	10.970249591
	201	0.650200811	0.885998851	10.456990276
	202	4.413149240	-11.059512108	6.933541927
	203	-5.023789402	-4.578586529	-3.129788633
	204	-2.640241531	-5.770759802	-0.405818982
	205	-5.627181475	-5.475851067	-7.405346158
	206	-3.935324351	-5.839501197	-7.834401167
	207	-5.084630070	-3.230753437	-6.689436501
	208	-1.522163823	0.083301180	-10.068909608
	209	-5.173718366	-3.676175439	-9.191226599
	210	-3.985512979	-0.224997735	-2.455841509
	211	-6.029598052	-1.762621186	-0.767839566
	212	-2.351893459	-1.566277581	-8.336140848
	213	-4.062743205	0.598152348	-9.363188619
	214	-2.225707712	-3.620006034	-9.051954309
	215	-4.542274394	-4.288508464	-1.460184952
	216	-3.344618056	-3.516424373	-6.745324575
	217	-3.192383644	-4.822726174	-9.624514385
	218	-7.431066854	0.653121905	-3.170193096
	219	-4.086733881	0.110060109	-0.735686892
	220	-2.556236755	-6.344670711	-6.267838376
	221	-4.327642936	-2.178824947	-0.627813529
	222	-1.500278682	5.067561173	2.012924018
	223	-2.106166377	2.704235099	1.157006865
	224	-6.045407560	6.428029627	2.204252743
	225	-5.791143432	4.548711983	-0.109794119
	226	-2.875888102	7.816182337	0.286393704
	227	-1.223166197	12.730203435	1.355285085
	228	-2.170077430	9.472278382	3.002901490
	229	-3.649984700	10.284563766	0.834856340
	230	-0.691745682	9.601125394	0.299184667
	231	-2.670645176	14.104623109	-2.381070207
	232	0.032702245	5.506234568	0.080953382
	233	-1.345797299	5.945869305	-0.936633706
	234	-1.362647545	14.695022634	-0.266260190
	235	-3.062462645	9.760085502	-0.743265828
	236	-3.619485595	5.488620356	0.026406881
	237	-5.968552392	4.673079851	2.371981062
	238	-6.577965929	7.267250307	-2.223590725
	239	-0.972329972	1.445859456	-2.687467049
	240	-7.917657013	6.159222038	-1.869938794
	241	10.010088558	-3.013722453	3.569845681
	242	8.806171582	-2.040431804	3.046103704
	243	4.720004305	3.299853919	-2.206235735
	244	2.595148707	4.812945042	-0.934901928
	245	6.132806773	5.164460599	-0.875386994
	246	-0.515888110	3.998732309	-2.526885488
	247	-1.427030577	5.471688919	-4.177409161
	248	-3.028930800	5.372540946	-5.737904594
	249	-0.478007218	6.517675923	-3.466430784
	250	-3.451759554	3.141772525	-4.950554788
	251	-4.869684959	4.232015155	-5.109512011
	252	7.113395663	8.480565640	-3.989717590
	253	7.034038102	10.246086080	-4.170023241
	254	5.494211805	7.533770204	-5.166543040
	255	3.864046340	8.193530699	-5.315065721
	256	2.825624485	7.751883595	-8.080110815
	257	0.935013010	3.888546081	-6.884532382
	258	-0.411580700	10.869899238	-3.291900549

	259	2.749760515	8.883177645	-0.004525399
	260	1.070862950	7.990290701	-5.013280736
	261	4.152437461	2.532011363	-4.559055887
	262	6.248103326	3.357625175	-3.973577045
	263	1.889651692	3.693689944	-4.376612152
	264	-6.675232012	4.401775926	-2.720881291
	265	9.029594087	4.570984229	-5.197001164
	266	-5.260497510	2.080649307	-3.710382738
	267	4.383389177	3.258964275	-6.146722025
	268	1.536036907	7.421044038	-1.568341965
	269	8.921588943	5.386965826	-3.624872290
	270	2.546912479	6.057590347	-8.309462961
	271	1.442289376	8.852765912	-6.463155792
	272	-6.248565398	-0.493697945	-3.470625472
	273	-6.569249745	0.574569824	-1.020584036
	274	0.906333692	12.352002727	-1.806649822
	275	-3.239359552	3.736818576	-2.553428550
	276	4.552129278	5.518626350	-5.140742037
	277	2.575507701	4.428944322	-7.253365151
	278	2.092468500	2.427219155	-5.574255978
	279	0.813046683	5.721866972	-5.167383431
	280	5.911065681	7.062135795	1.337178038
	281	5.599680582	7.726771883	-1.821617594
	282	7.720696925	9.715930965	-1.946459482
	283	7.387772925	6.297753626	0.706110425
	284	4.520597839	9.967843207	-0.800345464
	285	1.799306172	-7.229685524	-7.780689253
	286	0.981203992	-8.092097500	-6.464677558
	287	2.997439565	-9.952544982	-2.726327643
	288	0.802349707	-9.432988273	-8.406044102
	289	-0.676931852	-8.742821141	-8.255311362
	290	7.147566627	0.197547670	-0.923690915
	291	9.065695484	2.375116858	0.100926671
	292	8.749582771	2.508677062	-4.151684668
	293	6.641088409	1.858240882	-0.471009047
	294	10.279516545	1.944225755	-0.905048191
	295	5.999522807	-2.692685924	-6.356010030
	296	8.786249579	1.081196039	-2.519415422
	297	0.010227724	-7.218216872	6.915767320
	298	0.633913366	-9.614103290	5.102260739
	299	3.725078338	-2.715563121	-6.347014615
	300	4.209033646	-2.409528048	3.540111259
	301	1.423681151	-1.269987821	-8.660290834
	302	-2.244479079	-1.961319319	-4.081696243
	303	1.600528626	-0.334377114	-7.173023287
	304	1.165270984	-4.933320624	5.892667342
	305	1.717358263	-2.745009696	-6.058526902
	306	0.439213888	-5.430846685	4.362392636
	307	-0.576521988	-7.487251099	5.256297336
	308	1.838806288	-7.551091648	4.463605254
	309	2.367517789	-7.082604824	6.084426450
	310	-4.571429280	0.925231664	-7.079538764
	311	-1.245477063	-9.418341367	7.422975985
	312	-3.444549244	-11.133429137	6.113996239
	313	0.065775178	-1.065730476	-3.998409860
	314	-2.415830246	-11.177846444	8.845234836
	315	-3.843178100	-10.610693665	8.276456789
	316	2.667967912	-5.530205567	3.365511982
	317	0.658810118	0.978630948	-5.253543867
	318	-3.941065518	-0.886664453	-5.555665598
	319	-0.128543324	-12.559264210	1.513179792
	320	0.445616920	-10.762010773	-1.229135941
	321	0.168305436	-10.199145858	2.500594207
	322	-1.172508555	-12.781458554	0.384661367
	323	5.457213702	-3.085910481	-9.084064551
	324	1.983170838	-7.758351521	1.646027652
	325	-2.869772846	-7.026587608	2.642932702
	326	1.406091397	-5.329187813	-6.541954100
	327	-3.208951166	-8.976999450	1.428475927
	328	-1.815335107	-8.774319632	-5.045478235
	329	-1.494320584	-6.695743827	-3.633544877
	330	-2.556865251	-12.451683442	6.885534912
	331	3.769731456	-3.682149730	2.447506307
	332	3.022919425	-9.265347506	3.123418954
	333	3.485539220	-7.371752928	1.610973805
	334	2.796961030	-9.927611450	5.375872890
	335	2.556926786	-3.615294705	6.388831568
	336	-2.052438027	-9.490384977	-1.362430677
	337	-1.335029150	-10.122278239	0.123942383
	338	-3.759700860	-8.231265922	-0.079071683

	339	-0.182105182	-3.923850185	-5.351791040
	340	-1.037352190	2.074761662	-6.743436850
	341	-3.409511232	2.191735293	-7.555463078
	342	-1.743947039	-5.765964556	2.103081393
	343	2.148823040	-10.546342856	3.280987573
	344	0.056283511	-11.290046002	3.595374555
	345	3.070022417	-2.163884572	5.613696633
	346	-0.373784546	-10.733071544	-4.227864410
	347	1.384311594	-9.322357273	-0.878895440
	348	-1.392520854	-4.324426679	-6.607761458
	349	5.940714856	-1.004286684	-6.908134009
	350	6.137254897	-3.500867777	5.051810497
	351	6.405928852	-3.643159794	3.297020734
	352	2.915775827	-12.167331593	-3.538354027
	353	1.524514325	-12.503778588	-2.746085211
	354	2.740000131	-13.153750377	1.594153610
	355	1.594733050	-12.176766834	-0.111269828
	356	4.489885846	-13.728500426	-0.443290404
	357	3.696220694	-11.659000657	1.689927852
	358	8.347171356	-5.483379049	3.717799823
	359	5.730600497	12.730081800	0.680659463
	360	3.733622350	-10.049503606	8.230149701
	361	4.654614430	-8.017508140	6.591414979
	362	3.848212882	12.519022367	-1.692662766
	363	3.086583495	11.177808781	0.972839556
	364	2.274756147	12.584236038	0.254645489
	365	-0.932045768	8.675259471	-3.791134142
	366	1.753228751	-2.348403220	1.538211086
	367	0.098431848	-2.368979135	1.714873394
	368	0.799104704	-1.227724622	0.754185594
	369	0.415611395	-6.207933457	-0.952139643
	370	0.861402164	-2.205064737	1.054581855
	371	-9.876229112	-1.396133670	-0.067697305
	372	-9.735764500	2.138413493	1.154802246
	373	-9.507807471	2.266411180	3.955475961
	374	-7.928867933	0.029817663	4.586939723
	375	-5.446926333	0.868283298	3.479593487
	376	-4.906265833	3.062400057	5.248787205
	377	-4.964775911	1.984701309	7.964768365
	378	-3.962050177	-0.722426157	7.964927483
	379	-1.721390314	-0.506945672	6.266754402
	380	-0.060469911	2.054449508	6.426606948
	381	-0.342118029	2.935535874	9.192238510
	382	-2.843121144	-10.657661162	8.075871201
	383	-0.785525030	-9.785404085	6.583968092
	384	2.492041849	-5.040858197	4.249069059
	385	2.976635092	-3.175597518	5.577401435
	386	3.567603464	-3.178633229	3.342944679
	387	2.765450045	-9.833624461	6.415562898
	388	-6.422801567	0.484143903	-3.176886136
	389	-4.925839730	-3.931550105	-2.341271709
	390	-5.032578312	2.709798419	-2.924728241
	391	-6.148856944	5.213805874	-2.405556493
	392	-6.001948157	5.418315660	0.397506628
	393	-3.244337944	5.581245094	0.972749905
	394	-0.863927241	3.403228260	-1.766455418
	395	-1.677856374	1.645332891	-0.667648179
	396	-2.067907578	8.085355256	0.842745127
	397	-2.894480050	12.163189163	-1.469304558
	398	-1.786610064	13.787937074	-0.441837683
	399	9.021051754	-3.006287012	3.308551787
	400	9.343801579	1.670943785	-0.590394325
	401	8.526637689	3.331901687	-3.598472802
	402	6.033924629	4.283802346	-4.368043960
	403	1.470964643	5.691304300	-5.976410867
	404	2.362499672	6.900417658	-7.777945340
	405	1.569460527	8.010385523	-5.907661215
	406	4.229962214	4.205921899	-2.086872407
	407	5.640705120	5.914124265	-0.382999847
	408	6.208806415	8.433221731	-1.409105337
	409	4.850360380	8.332737168	-5.098434643
	410	4.946014342	10.881104637	-0.943846649
	411	0.303256411	-8.543645071	-8.476457108
	412	0.412369684	-5.331794920	-6.790605355
	413	1.288180846	-2.404968576	-6.934424443
	414	4.137682328	-2.115776170	-7.068341261
	415	-3.123194476	-3.808783809	-9.514094634
	416	-3.224921608	-1.083013859	-8.570509082
	417	2.043589591	-11.747559228	-3.203151039
	418	-0.101894132	-7.911332356	-2.514387948

	419 -1.154700240 -8.715241557 -4.275357694<br>420 2.600142724 -11.983093782 -0.109794365<br>421 -8.357202251 0.383321502 1.606955279<br>422 -7.513213885 3.347279798 3.742692612<br>423 -7.499647850 0.939913851 6.639712337<br>424 -4.458392423 -0.304713075 5.157916412<br>425 -2.701742357 2.678357634 4.838533688<br>426 -3.197182900 3.388825764 8.339849154<br>427 -3.176672028 -0.337792404 10.083337361<br>428 -0.856107965 -2.427397822 7.188105864<br>429 1.555486081 0.444413679 6.546664831<br>430 1.922641326 3.023989835 9.162591109<br>431 0.988785640 1.714751425 12.291422846<br>432 -0.914467644 -11.560295288 5.111789690<br>433 1.485232052 -9.380651743 8.270975011<br>434 6.233418004 -9.194556376 7.122629043<br>435 -5.183499651 2.732340603 -0.632159537<br>436 -5.601659529 -2.195444295 -3.643288452<br>437 -4.065114407 6.053233086 -2.001114636<br>438 -3.046650621 5.196140984 -4.780397525<br>439 -6.827852817 7.559395526 0.292591191<br>440 -3.673441545 5.748675960 3.209679360<br>441 -0.386196170 7.374871974 2.204947491<br>442 -0.612396390 10.744142808 2.651407602<br>443 8.364211964 -5.436776747 5.759220985<br>444 5.516669366 2.446008701 3.503990720<br>445 7.969441487 4.130982885 -1.533703075<br>446 6.927891737 6.282863393 -5.068568667<br>447 3.297424617 6.050011906 -3.060223302<br>448 3.674253531 6.472223997 0.657870981<br>449 5.020172895 3.146097053 0.689425760<br>450 7.706333803 8.849281354 0.271554306<br>451 6.514339545 11.998056835 -2.161503445<br>452 4.280744673 10.196442567 -3.904533429<br>453 4.557011780 14.291613430 0.086986261<br>454 -0.172480557 8.196798035 -3.406462555<br>455 -1.376754515 -6.636203974 -7.406862223<br>456 -0.602406918 -2.521094223 -8.223662256<br>457 3.898600806 -0.488830776 -8.635746703<br>458 7.283246581 -2.206674965 -8.831040126<br>459 -5.447137245 -1.240422618 -9.061517175<br>460 -5.200955668 -5.697582099 -4.835783169<br>461 -3.146675485 -6.313918798 -5.453128296<br>462 -1.578677671 1.995982999 -9.371439809<br>463 4.443481656 -11.009197466 -0.996329283<br>464 5.577347274 -13.447420814 1.261433801<br>465 -3.524549670 -5.228042903 -0.042469999<br>466 -4.607842859 -5.248529755 1.931106907<br>467 -2.659690443 -12.184472517 -0.301778104<br>468 -4.437830650 -10.826453905 -0.632705865<br>469 0.482648841 -7.849158837 0.531762165<br>470 -0.589225664 -8.649545121 2.335296331<br>471 -1.625385829 -6.673209178 -0.469136705<br>472 2.989407491 -10.123896073 3.612905985<br>473 2.776529250 -7.631133537 2.229010865<br>474 -0.492383754 5.631386411 -3.889396484<br>475 -0.363719234 -13.201962331 0.807123298<br>476 0.545027935 -11.083685729 2.758994523<br>477 5.824958687 1.250163193 -2.678631896<br>478 2.759857074 -0.750214588 -3.739965187<br>479 -0.385628956 -4.491720100 -2.370185029<br>480 3.479858702 -0.513946420 -0.244111262<br>481 -0.170119629 -1.409420910 -1.478565033<br>482 2.815793901 -4.359209936 -4.670392780<br>483 0.864491835 -4.934290964 0.951640114<br>484 5.554777622 -2.557898067 -2.469049917<br>485 2.643321959 -6.574511685 -1.737501246<br>486 4.633241170 -4.400238476 0.637562720<br>end

Fe6NH3 to core

35, S=1

reactant

Fe(139) -2.370<br>Fe(140) -2.202<br>Fe(141) -2.276	9651826nh3d35tb.car_4<br>C1 -8.030443159 -4.997428818 -0.547480714<br>C2 -8.393293582 -5.779550208 -1.814805208<br>C3 -8.863113413 -3.473724241 1.194904603

Fe(142) 2.758<br>Fe(143) 2.960<br>Fe(144) 2.268<br>Fe(145) 0.890	C4	-8.346529619	-4.165146964	2.452863645
	C5	-8.106799801	-6.252969690	3.706127174
	C6	-6.583574203	-6.420393055	3.705951176
	C7	-4.514253334	-6.392639562	2.405881971
	C8	-3.925818601	-5.058741579	2.877193170
	C9	-4.201057368	-2.626896187	2.914057574
	C10	-4.266110620	-2.497012369	4.441580777
	C11	-5.469954789	-2.923013532	6.495735580
	C12	-4.509803829	-3.817861223	7.294179505
	C13	-3.684887397	-6.098399373	7.662056792
	C14	-2.212633136	-6.354709761	7.307883731
	C15	-0.541811784	-6.514695679	5.503760728
	C16	0.250915065	-5.255360062	5.139262497
	C17	0.163751922	-2.830557197	4.719845554
	C18	0.317709164	-1.975872170	5.994802436
	C19	-0.473360777	-2.101629594	3.518788295
	C20	0.189169896	-0.740186548	3.283249645
	C21	-0.401462632	-2.988597775	2.271335624
	C22	-0.758296151	-0.628284812	7.735848098
	C23	-0.485448958	-1.415311493	9.030337030
	C24	-2.019944275	0.264183907	7.814804810
	C25	-2.036823181	1.094587475	9.102649015
	C26	-2.096180897	1.180047302	6.586304837
	C27	-1.057002970	-3.261659543	10.489049792
	C28	0.389716766	-3.626687283	10.756878072
	C29	4.910302976	-12.599210841	0.339785835
	C30	5.695224280	-11.297323760	0.240675911
	C31	6.185153417	-9.193555629	1.458365293
	C32	7.406076581	-9.156079597	2.394017064
	C33	5.088804713	-8.239635469	1.963397864
	C34	5.548265990	-6.786754800	1.928421309
	C35	4.432749465	-5.798553299	2.246724835
	C36	4.973535140	-3.439201054	2.895581665
	C37	9.665855237	-8.267282763	2.707321046
	C38	9.897577302	-6.828408551	3.112561855
	C39	-5.360087372	-1.726709901	-2.459190495
	C40	-5.726573030	-0.492537333	-1.615453230
	C41	-3.832348251	-1.931484884	-2.407249562
	C42	-3.334702757	-3.313159964	-2.878686925
	C43	-3.078137342	-3.469431477	-4.377171615
	C44	-5.910032855	1.976132629	-1.818885586
	C45	-7.043165738	2.241636298	-0.821057065
	C46	-5.952852607	2.952221811	-3.003379278
	C47	-9.296461518	1.633412296	-0.079149928
	C48	-8.995057144	1.184348789	1.355946297
	C49	-7.448759550	0.049712840	2.877067153
	C50	-6.249406118	0.815050876	3.444266066
	C51	-4.541002012	2.547190447	3.139725625
	C52	-4.961127248	3.614084260	4.161464892
	C53	-3.840659815	3.294865293	1.994149221
	C54	-3.226220913	2.399739047	0.972375202
	C55	-2.966340314	1.045249747	0.938091501
	C56	-2.279035586	1.808599129	-0.949846028
	C57	-6.586478925	5.395298129	4.591828330
	C58	-6.493656206	5.152033092	6.096194535
	C59	-8.057863071	5.661244512	4.183860365
	C60	-8.599420553	7.034933820	4.449490103
	C61	-8.541179582	7.827845941	5.579186409
	C62	-9.696202404	8.843037987	3.984186556
	C63	9.253629972	0.161340468	3.943898805
	C64	10.252499607	-0.887203211	3.489299302
	C65	7.857795980	-0.381401028	3.557578364
	C66	6.718156033	0.514842589	3.984734377
	C67	6.101440187	0.367917401	5.240261717
	C68	6.226336647	1.513311851	3.123971328
	C69	5.028934778	1.181286063	5.625035458
	C70	5.167436877	2.340642029	3.498107153
	C71	4.566817624	2.172022965	4.749230677
	C72	5.786016990	6.613174445	0.788189461
	C73	4.818990434	7.803025199	0.909118934
	C74	4.996085570	5.311979368	0.659371539
	C75	3.941937146	9.831408935	-0.199656714
	C76	2.474613185	9.595325288	-0.568454197
	C77	0.772911249	7.875916251	-1.160659100
	C78	0.163138926	7.172434582	0.077257764
	C79	0.816441823	6.962075164	-2.405145064
	C80	-0.460186448	6.230167589	-2.836947466
	C81	-1.545441414	7.074812604	-3.521895439
	C82	-2.968207087	8.869131368	-2.536934277
	C83	0.408744702	5.563714307	1.895643758

	C84	0.342877320	6.450828273	3.149462349
	C85	1.202751442	4.295072764	2.248378312
	C86	1.550797407	8.097457841	4.504149145
	C87	1.059001349	9.534559754	4.608257618
	C88	-0.149230350	11.364134483	3.529354920
	C89	-1.403114807	11.783868685	4.331410783
	C90	-0.240284805	11.857662280	2.080497533
	C91	-1.423888144	11.286510329	1.306063722
	C92	-3.470428077	11.065986331	5.425080524
	C93	-3.370661284	11.825274280	6.738672545
	C94	-4.242145568	9.732248088	5.634724471
	C95	-4.496677678	9.023154459	4.325185513
	C96	-5.622735994	9.344729632	3.553270554
	C97	-3.585640247	8.087039876	3.810052720
	C98	-5.822048528	8.774170389	2.295349149
	C99	-3.755693520	7.525370010	2.542204677
	C100	-4.873535013	7.882219743	1.793109479
	C101	4.272356011	-1.504477221	-9.647953382
	C102	2.849383224	-1.306922122	-9.153716155
	C103	1.434793118	-0.123132078	-7.484726517
	C104	1.040094442	1.352234840	-7.626664603
	C105	1.540750991	3.640214540	-6.884456030
	C106	2.787075984	4.518251150	-6.795890743
	C107	5.100304908	4.819529135	-6.114396941
	C108	5.651352038	5.444814810	-7.379320214
	C109	-2.434501235	-0.539275814	-9.885403649
	C110	-3.404362069	0.502901454	-9.317145429
	C111	-2.093151214	-1.595054097	-8.808307531
	C112	-1.441449577	-2.837005531	-9.443651844
	C113	-0.871949850	-3.753528490	-8.383876253
	C114	-3.690813992	2.562833774	-8.032594903
	C115	-3.064396375	3.923954718	-8.256195734
	C116	-4.179809274	2.301953371	-6.588858924
	C117	-3.031271248	1.923829700	-5.696953105
	C118	-2.222094167	2.908649381	-5.108073911
	C119	-2.666320509	0.572695210	-5.566924912
	C120	-1.041521240	2.549934657	-4.452090646
	C121	-1.486909219	0.217090710	-4.909286249
	C122	-0.656444781	1.208189451	-4.378579994
	C123	7.762771556	-5.214637529	-6.192020717
	C124	8.901428898	-5.643212651	-5.250391260
	C125	6.400479170	-5.605759483	-5.599478025
	C126	5.186649793	-5.081100147	-6.298854721
	C127	4.807860312	-5.287288276	-7.609783569
	C128	3.163347191	-4.272336326	-6.528603922
	C129	9.945217498	-7.358783822	-3.886918866
	C130	11.336304106	-7.035881891	-4.382834632
	C131	0.264420081	-6.437378496	-2.069837677
	C132	1.734395200	-6.716170492	-1.756543212
	C133	2.681925239	-6.569807297	-2.985420877
	C134	2.503101881	-7.787549481	-3.935976084
	C135	3.565471247	-7.931977373	-5.029094560
	C136	3.492277836	-9.256289196	-5.820766399
	C137	4.144641588	-6.556819083	-2.514871329
	C138	3.681241664	-0.274621847	-2.232453428
	Fe139	3.870298200	-1.071156366	-4.064488901
	Fe140	2.309343243	0.924310997	-1.596653615
	Fe141	4.873634993	0.670744276	-0.992347455
	Fe142	4.088702267	1.385917585	-3.370029584
	Fe143	3.854779821	3.065733062	-1.281805201
	Fe144	4.975241436	-1.813180896	-1.847298909
	Fe145	2.396522379	-1.720937659	-1.797551758
	H146	-6.653696073	-6.826218803	-2.010411829
	H147	-6.721857214	-5.496534509	-2.954284534
	H148	-9.967742837	-4.313799863	-0.425258978
	H149	-9.037328213	-5.147367581	-2.447573835
	H150	-9.021348549	-6.629222415	-1.495205577
	H151	-8.138272254	-2.682918615	0.951426573
	H152	-9.158430425	-5.945975994	1.854663578
	H153	-9.818866471	-2.990069742	1.438001259
	H154	-8.571612677	-7.247340714	3.690519331
	H155	-6.505129697	-6.179018934	1.660675911
	H156	-8.370242935	-5.770747773	4.656992535
	H157	-3.178068864	-2.377121912	2.610205555
	H158	-5.343639866	-4.046199178	1.783032893
	H159	-4.228594354	-6.571485679	1.359962932
	H160	-5.274692726	-1.888380582	6.807323233
	H161	-6.136678782	-3.371593298	4.495743513
	H162	-4.077608156	-7.184105609	3.026332940
	H163	-4.877985933	-1.898527863	2.448198413

H164	-4.965539027	-5.427315430	6.083458591
H165	-6.500090052	-3.185771422	6.774149796
H166	-0.939913146	-3.935737470	2.416895752
H167	-0.832693286	-2.477297031	1.396862185
H168	0.644795004	-3.241681165	2.032185587
H169	-1.529580395	-1.919930336	3.766745169
H170	-4.215823738	-7.058521390	7.571364962
H171	-2.131873065	0.612610881	5.646646118
H172	-2.012685148	-2.780932285	8.643893073
H173	-2.051252130	0.466397663	10.005214195
H174	1.272364490	-0.841625804	3.107160048
H175	0.051132110	-0.062240448	4.139197275
H176	9.310244422	0.215483226	5.047318273
H177	-2.996571856	1.809196563	6.625388069
H178	-2.929512071	1.737187304	9.119329198
H179	-1.219235833	1.847721800	6.553481220
H180	-1.149641514	1.745966469	9.157136712
H181	-2.902909097	-0.400282753	7.816863248
H182	0.124526978	0.019175117	7.620884680
H183	-1.725945471	-1.670175094	6.116745922
H184	-0.255979884	-0.253945073	2.401474988
H185	1.360508988	3.696974904	1.333938216
H186	0.584218860	3.710197077	2.945753065
H187	2.638448894	4.012601205	3.575939913
H188	3.143180236	2.754969937	5.928895157
H189	4.785528871	3.096627705	2.812370980
H190	6.658992532	1.628320279	2.127480782
H191	6.450969564	-0.406770461	5.928172284
H192	-1.485282365	-4.163188131	5.045196007
H193	-0.562180976	-7.164311401	4.615473404
H194	0.027287092	-7.032269953	6.286276309
H195	-2.601998195	-6.043484514	5.293266957
H196	-3.698804231	-5.792335430	8.715056440
H197	4.548217012	1.035566810	6.596437141
H198	1.202372611	-3.078196182	4.454543457
H199	-1.446371058	-2.701560878	11.355432412
H200	-1.619638132	-4.205194251	10.408082115
H201	1.016030837	-3.773501059	9.846094728
H202	10.559615130	-8.646646298	2.188679867
H203	-1.876932904	-4.454312570	-5.708403259
H204	1.077352935	-5.231867588	-3.355428609
H205	-2.202520120	-3.395577477	-10.007748031
H206	-0.649799636	-2.529441539	-10.142046153
H207	-3.006827238	-1.901551644	-8.276011372
H208	-2.388966123	3.975755546	-9.142803939
H209	-3.031825569	-1.045426525	-10.659351969
H210	-3.348527911	-1.134059460	-2.991695812
H211	-4.063562373	-4.094298099	-2.596764095
H212	-1.840679913	1.556139300	-8.446727670
H213	-4.592642115	2.583796811	-8.679104969
H214	-0.627022578	0.484662360	-9.897854200
H215	-1.456319455	-4.735685350	-4.020640783
H216	-1.429853638	-1.141044407	-8.055607335
H217	-0.808794771	-0.585211482	-11.137885101
H218	-6.767226942	-1.936463686	-3.959557944
H219	-3.500300312	-1.785813911	-1.369803052
H220	0.889114352	-2.963012751	-8.741652095
H221	-2.412364783	-3.564664353	-2.335182717
H222	-3.838070606	1.878682931	3.655771945
H223	-3.187164202	0.308953845	1.706384693
H224	-8.286937480	0.222775683	3.565997323
H225	-7.279914389	0.170034529	0.750015687
H226	-6.716420919	3.844756696	3.123210991
H227	-8.058033871	7.693222158	6.540268560
H228	-6.740431676	4.104158421	6.394333486
H229	-8.697935580	4.889806010	4.647729276
H230	-5.956235532	6.260651121	4.330459721
H231	-10.288509810	9.610568787	3.491650712
H232	-3.077063666	3.942625810	2.457460923
H233	-4.573049086	3.963664842	1.510716766
H234	-9.382503328	9.771804756	5.875023315
H235	-8.128832585	5.507822564	3.095662752
H236	-6.043298581	1.955219112	1.720210198
H237	-7.215512691	-1.024467153	2.924724566
H238	-9.654539916	2.669181728	-0.002945352
H239	-1.851784495	1.904976897	-1.945077049
H240	-10.125615576	1.023187388	-0.461919282
H241	10.357564261	1.864638041	3.681251222
H242	8.760496986	2.069130644	3.394678392
H243	1.837944878	5.938834387	0.312683929

H244	-0.629357265	5.267981491	1.684185231
H245	2.063599438	7.411789136	2.570307139
H246	-2.902960085	3.842401096	-0.578175780
H247	-4.622889347	5.095294182	-1.596552554
H248	-6.108680774	4.885868506	-3.272204681
H249	-4.342667116	6.023435584	-0.347012358
H250	-5.098798890	2.730559972	-3.664796159
H251	-6.889218611	2.805822611	-3.565841551
H252	0.713771877	11.672080162	1.567728404
H253	-0.386759350	12.948424266	2.121405401
H254	-0.177534669	10.756414531	-0.267645733
H255	-1.893721792	10.390208957	-0.441055218
H256	-2.894390985	10.750824348	-3.355006538
H257	-2.082769340	6.459068788	-4.260066037
H258	-6.709744926	9.025733096	1.709504718
H259	-2.722712864	7.768436013	4.401586556
H260	-3.993786544	8.605790597	-0.772240495
H261	1.583432258	6.193405997	-2.222273230
H262	2.872906836	7.615027009	-0.901672146
H263	-0.899624158	5.663992522	-2.000567716
H264	-8.203584968	0.838420491	-1.747352820
H265	4.358505474	10.545967578	-0.921469736
H266	-5.822193193	0.432241803	-3.373696837
H267	1.187416231	7.572065708	-3.245511743
H268	-3.008741634	6.844421249	2.134105902
H269	3.936938679	10.316102184	0.787126795
H270	-2.246001335	9.497704072	-4.364878956
H271	-4.357424510	10.060165257	-1.630921437
H272	-5.183118093	-2.033439106	-4.486814850
H273	-5.836809809	-2.584224276	-1.946049253
H274	-6.361454126	10.050727046	3.938920843
H275	-4.982136082	2.141473562	-1.250255311
H276	0.149611129	8.758229851	-1.338456246
H277	-1.070755215	7.897081146	-4.073064916
H278	-0.140696169	5.476753950	-3.571384496
H279	-2.998357297	6.915689384	-1.963371714
H280	1.070749448	7.527378427	5.313545465
H281	0.058595521	9.331030901	2.834131223
H282	0.666077458	11.922315284	4.014687928
H283	2.633106862	8.114044586	4.694455003
H284	-1.940114732	9.842217505	4.572692180
H285	4.843894682	-0.572805508	-9.525534691
H286	4.724080402	-2.249626564	-8.960521358
H287	7.831249449	-4.117979409	-6.244350596
H288	5.194942384	-2.294067173	-11.284511005
H289	3.606203604	-2.676067637	-11.170589347
H290	5.714997743	4.487906951	0.558710377
H291	6.112953997	6.524685162	2.801823268
H292	5.371680183	8.444328164	-0.963424228
H293	4.377989760	5.152394854	1.553304760
H294	7.274052895	7.349316122	1.997145838
H295	5.893053031	4.193538400	-5.675372859
H296	6.410363303	6.741172769	-0.109987043
H297	4.791997416	-8.536286653	2.983547948
H298	6.481614732	-8.891790457	0.445532382
H299	4.003271850	2.990756400	-6.060549605
H300	5.379124312	-1.462120129	3.155396480
H301	1.031779487	3.884889481	-7.824669208
H302	-1.207521057	-0.834190972	-4.808000056
H303	0.875839311	3.960795234	-6.064700353
H304	4.107172080	-5.907206873	3.288394062
H305	2.355929348	1.780115584	-6.131938508
H306	3.551796361	-5.980320880	1.611117381
H307	4.213993144	-8.370778830	1.306513755
H308	5.932425612	-6.557387370	0.924177082
H309	6.375754522	-6.617822841	2.634816959
H310	-4.925045761	1.494398966	-6.625748527
H311	5.136308597	-10.956250533	2.134633593
H312	4.012456802	-12.452491198	-0.279378199
H313	0.261103886	0.931101557	-3.861486951
H314	5.222234988	-13.498778141	2.166078144
H315	3.652935409	-13.499387251	1.697649143
H316	5.327353321	-4.274737210	1.084900852
H317	-0.402811914	3.307559494	-3.992747923
H318	-3.307609534	-0.204380785	-5.989302271
H319	7.105228903	-9.567153574	-4.208687834
H320	6.325827321	-6.706871780	-5.579159153
H321	6.139131502	-8.238896144	-2.251287577
H322	6.245653842	-9.759922054	-5.488314628
H323	5.347496326	4.943254814	-8.328681032

H324	6.112626919	-5.189840019	-1.508969120
H325	1.828424925	-7.711220080	-1.305909786
H326	3.526902353	0.022157878	-7.748916881
H327	2.523857075	-8.685348071	-3.298693245
H328	2.948370416	-4.805509570	-8.558712766
H329	2.191720235	-3.831710575	-6.342138432
H330	5.515960852	-13.374875439	-0.159161715
H331	5.779638201	-2.038503162	1.573815762
H332	7.962446173	-6.373287173	-0.645972519
H333	7.058595882	-4.075951328	-0.990576260
H334	8.429612998	-8.025246246	0.966432432
H335	3.809715585	-4.309636475	4.366855169
H336	3.456008010	-7.110960026	-5.754212462
H337	4.573016085	-7.846654462	-4.599498912
H338	1.500906883	-7.738020801	-4.388917665
H339	1.529155430	-0.401581886	-6.423474565
H340	-2.509112729	3.959823723	-5.196528242
H341	-4.680545047	3.220577397	-6.245995381
H342	2.028837506	-5.973427451	-0.995500216
H343	8.021343448	-7.791112305	-1.289082901
H344	6.782375318	-9.573236554	-1.803159114
H345	4.458800797	-2.773051644	4.752429150
H346	5.323217460	-5.776174739	-8.427240855
H347	6.366435530	-5.287348496	-4.551846878
H348	0.655183767	-0.729509424	-7.951270368
H349	4.872429270	5.619294329	-5.392007958
H350	7.749119796	-1.374468988	4.019477956
H351	7.828733489	-0.518203013	2.464335692
H352	8.847731303	-5.429082017	-7.919356232
H353	7.968556646	-6.758046074	-7.544158687
H354	9.877171696	-8.454054042	-3.791162921
H355	8.192905879	-7.611362441	-5.077448968
H356	11.438065947	-6.976030470	-5.492670312
H357	9.799834669	-6.928236753	-2.882980554
H358	10.495073395	-0.850841228	2.400252111
H359	-2.382137391	11.764758502	7.252034170
H360	9.543195522	-8.848991264	3.638290290
H361	8.986893069	-6.181219881	3.115013144
H362	-4.040510668	11.697133748	4.722607600
H363	-3.670103714	9.090184176	6.322257381
H364	-5.192639316	9.981630580	6.127536627
H365	3.108565477	-1.032234572	0.618815639
H366	-5.922852912	7.484378834	0.202194768
H367	2.262679355	-2.454295520	0.803251474
H368	1.450505779	-1.019204805	0.500913933
Mo369	3.756101829	-3.684935790	-3.434256969
N370	2.294491033	-1.567763448	0.292537192
N371	-7.281075160	-6.278525052	-2.604675911
N372	-9.073595542	-4.357079630	0.057849314
N373	-8.624671138	-5.484174975	2.587006681
N374	-5.968744087	-6.419971631	2.497285069
N375	-4.569009115	-3.951449925	2.437364234
N376	-5.342654627	-3.045141504	5.053222479
N377	-4.395927954	-5.104198863	6.871007315
N378	-1.893688144	-6.298457591	5.986553937
N379	-0.470032338	-4.112552330	5.006121050
N380	-0.821458436	-1.504216398	6.566490913
N381	-1.217861760	-2.538779943	9.240258595
N382	4.502308447	-12.933018385	1.710834624
N383	5.659310510	-10.542158679	1.356559677
N384	4.904581411	-4.432318197	2.005370385
N385	4.505845306	-3.596288303	4.158094482
N386	5.536752147	-2.269567596	2.556887674
N387	8.465844638	-8.455037141	1.917530931
N388	-5.829858307	-1.545204393	-3.840995924
N389	-2.075575727	-4.303996291	-4.714729746
N390	-5.919043346	0.626838432	-2.364504993
N391	-8.189846451	1.544752976	-1.014978182
N392	-7.885650559	0.419511795	1.543967293
N393	-5.676501664	1.767494293	2.656201371
N394	-2.775688013	2.878049164	-0.246649958
N395	-2.384695748	0.685057732	-0.256068247
N396	-6.155865115	4.197937789	3.895142241
N397	-9.325371716	7.681235284	3.460609890
N398	-9.240820620	8.973163611	5.262574957
N399	9.540597625	1.421106080	3.254264641
N400	6.682788590	6.514737921	1.949834171
N401	4.791138171	8.656754739	-0.156088102
N402	2.136122539	8.333428576	-0.900271571
N403	-2.528654600	7.614715135	-2.577477047

N404	-2.626023939	9.778763758	-3.468439757
N405	-3.713752468	9.286485544	-1.483956187
N406	0.939428149	6.255817711	0.720409607
N407	1.290724789	7.429680370	3.240672888
N408	0.206666087	9.957073654	3.625589336
N409	-1.150675774	10.904748225	0.030448097
N410	-2.147809142	10.790311467	4.877675119
N411	4.275727086	-1.909445343	-11.055555159
N412	2.692104182	-0.396106619	-8.171489043
N413	1.738073722	2.202107034	-6.844102969
N414	3.948230263	3.968864739	-6.362000706
N415	-1.305179561	0.103193232	-10.568278458
N416	-2.853412157	1.521855748	-8.600039302
N417	7.938405877	-5.733804259	-7.560520952
N418	4.141925745	-4.433228438	-5.639156514
N419	3.536850087	-4.768326395	-7.730635890
N420	8.917567606	-6.934989144	-4.823484380
O421	-6.886016296	-4.983632447	-0.060081262
O422	-7.704888328	-3.521528625	3.300996561
O423	-5.972555176	-6.570517166	4.777991818
O424	-2.936282457	-4.998596116	3.629844427
O425	-3.370078534	-1.920478844	5.076257234
O426	-3.923233036	-3.378724122	8.299118558
O427	-1.406784163	-6.651147312	8.202067912
O428	1.480539081	-5.332838580	4.968372089
O429	1.445104802	-1.740988094	6.458674973
O430	0.369448967	-1.039622621	9.850628225
O431	0.833630096	-3.825972475	11.878399655
O432	6.276828389	-10.999138286	-0.822558031
O433	7.374914928	-9.702968486	3.510308335
O434	10.988634035	-6.386444240	3.437727035
O435	-5.831338110	-0.520420787	-0.374927346
O436	-3.771893491	-2.887570914	-5.242796918
O437	-6.896355006	3.047910159	0.113395350
O438	-5.881733212	4.307868549	-2.522046636
O439	-9.768839294	1.517504275	2.266126685
O440	-5.844668467	0.551318984	4.584221462
O441	-4.228257153	3.977031826	5.088909982
O442	-6.257862084	6.016135321	6.925001531
O443	10.742996051	-1.737366782	4.218626984
O444	3.511417924	3.004394662	5.061216842
O445	4.110682816	7.981590949	1.915620659
O446	1.684515941	10.561323732	-0.549453833
O447	-0.986105774	7.457301363	0.451147901
O448	-0.501900505	6.232391635	4.026653434
O449	2.456361464	4.638236342	2.847171646
O450	1.408870609	10.253973177	5.552076532
O451	-1.665262349	12.984591564	4.497277060
O452	-2.559619311	11.232258053	1.800924672
O453	-4.311489229	12.432334905	7.227118407
O454	-5.002068618	7.350094180	0.498958112
O455	1.911203701	-1.996690522	-9.623929654
O456	0.160593685	1.719164130	-8.434648003
O457	2.696180439	5.724301604	-7.083039030
O458	6.405906206	6.405027782	-7.376471883
O459	-4.630946117	0.406885902	-9.492895889
O460	-1.551224763	-4.540087931	-7.732970209
O461	0.450147386	-3.637768373	-8.136890638
O462	-3.299507136	4.916691209	-7.582024135
O463	9.807562919	-4.853107707	-4.918764017
O464	12.308041248	-6.923197867	-3.647335123
O465	0.060898358	-5.469535832	-2.963719723
O466	-0.659517638	-7.022458392	-1.503603132
O467	4.603361410	-9.826977815	-6.106413726
O468	2.348096254	-9.671095457	-6.160118122
O469	4.863853620	-5.522071297	-2.859342593
O470	4.598750669	-7.517909466	-1.861867832
O471	2.374215718	-5.329001834	-3.626412632
O472	8.487202365	-7.210547167	-0.625038832
O473	6.711147430	-4.956530512	-0.751810741
O474	-3.908552976	5.577878972	-1.107397046
O475	7.190478433	-9.818080336	-5.155475438
O476	6.979837222	-8.765769486	-2.340986210
S477	3.876121906	5.314431518	-0.815674200
S478	2.272214548	2.709315875	-2.917909555
S479	1.957673197	-2.233324431	-4.001538089
S480	3.308550632	1.624060623	0.340747940
S481	0.568722798	-0.362111229	-1.630217212
S482	4.310657984	0.630212223	-5.432394036
S483	3.631994594	-3.466927672	-1.131645910

	S484 5.810444372 2.294650482 -2.188403110<br>S485 5.698484921 -2.365073800 -3.877318329<br>S486 6.271318184 -0.941001512 -0.318915207<br>end

TS

Fe(139) -2.353<br>Fe(140) -2.337<br>Fe(141) -2.434<br>Fe(142) 2.758<br>Fe(143) 2.984<br>Fe(144) 2.239<br>Fe(145) 1.179	9651826nh3d35td_1_53010.00

C1	-8.038981530	-4.994292567	-0.551095123
C2	-8.412780030	-5.775211853	-1.815974212
C3	-8.856194088	-3.465385806	1.194316303
C4	-8.337858946	-4.156742563	2.451218460
C5	-8.098747413	-6.242449599	3.707509657
C6	-6.575575360	-6.410700496	3.704113437
C7	-4.508688028	-6.387115580	2.400706663
C8	-3.919576888	-5.053902481	2.873703898
C9	-4.196872710	-2.619803448	2.902864121
C10	-4.261588891	-2.484557047	4.430122458
C11	-5.457083345	-2.910220266	6.487516391
C12	-4.494660585	-3.802247175	7.286289155
C13	-3.664670651	-6.081142668	7.654201395
C14	-2.193705358	-6.344124742	7.299214891
C15	-0.526559694	-6.534943151	5.495899334
C16	0.268335323	-5.274925314	5.137193075
C17	0.170768198	-2.849500153	4.707945184
C18	0.327130935	-1.995313842	5.983484740
C19	-0.491885168	-2.128439794	3.517943558
C20	0.132568816	-0.746892760	3.297848620
C21	-0.419570241	-3.011412836	2.266907939
C22	-0.742235188	-0.637083121	7.720860126
C23	-0.466538137	-1.418810631	9.017316065
C24	-2.003333670	0.256844440	7.801781039
C25	-2.020077765	1.086476568	9.090238561
C26	-2.079368957	1.174226014	6.574365170
C27	-1.040204460	-3.255524983	10.486938885
C28	0.404854798	-3.629348469	10.751476401
C29	4.919798493	-12.594538133	0.350954265
C30	5.694956189	-11.287202734	0.248332324
C31	6.171614767	-9.178137551	1.462296780
C32	7.391575390	-9.141033255	2.398747676
C33	5.074593208	-8.226271071	1.968981288
C34	5.528755805	-6.771572609	1.931049774
C35	4.414284610	-5.791677487	2.279860579
C36	4.990720879	-3.434172018	2.902780597
C37	9.653793388	-8.261639721	2.711926516
C38	9.895591450	-6.824210758	3.115438239
C39	-5.368508406	-1.726472983	-2.461993690
C40	-5.734667330	-0.493372381	-1.616621302
C41	-3.840892722	-1.934593978	-2.408364193
C42	-3.346786003	-3.315827865	-2.884717288
C43	-3.086007967	-3.465047340	-4.383192018
C44	-5.923406282	1.975185115	-1.818846744
C45	-7.057641520	2.237984377	-0.821229541
C46	-5.967473465	2.951518019	-3.003042429
C47	-9.308313890	1.621294451	-0.077648208
C48	-9.003636913	1.175465640	1.357870419
C49	-7.450756817	0.051192111	2.880044953
C50	-6.249724764	0.818149015	3.441610201
C51	-4.539295069	2.546113859	3.125475461
C52	-4.958723033	3.612700016	4.148405787
C53	-3.845505311	3.295000294	1.976719521
C54	-3.232429935	2.402986311	0.951092728
C55	-2.971781487	1.048632046	0.910231418
C56	-2.279880338	1.822354554	-0.971767376
C57	-6.592712199	5.385698912	4.587133991
C58	-6.506971445	5.135672769	6.090812691
C59	-8.060841825	5.653426275	4.171444946
C60	-8.603232307	7.026668510	4.437273326
C61	-8.537559584	7.823438665	5.563949253
C62	-9.708815333	8.830035894	3.975292221
C63	9.232551480	0.131230821	3.944591205
C64	10.224968042	-0.924373021	3.488890639
C65	7.830915958	-0.396286305	3.560062795
C66	6.698521376	0.505819828	3.993251522
C67	6.081274460	0.356300341	5.248482527
C68	6.218842076	1.517876234	3.141514633
C69	5.020653435	1.181348753	5.641889462
C70	5.173223644	2.357116104	3.525261447
C71	4.571650631	2.186232495	4.775684497
C72	5.793925144	6.603758261	0.780540442
C73	4.830646040	7.795846063	0.904070067

	C74	4.999940993	5.303993959	0.650262350
	C75	3.956388592	9.825763794	-0.203907324
	C76	2.486812439	9.592440271	-0.566623741
	C77	0.779932740	7.875912892	-1.150517892
	C78	0.173467115	7.173848706	0.090113401
	C79	0.818764298	6.959356255	-2.393665155
	C80	-0.460913012	6.232310229	-2.824752265
	C81	-1.541175290	7.079260825	-3.515123218
	C82	-2.969267186	8.872386998	-2.533339428
	C83	0.425271225	5.567660072	1.910526287
	C84	0.358975311	6.457431119	3.162336526
	C85	1.224194508	4.302240508	2.265063961
	C86	1.565054968	8.109432258	4.511747047
	C87	1.063437909	9.543592678	4.609858384
	C88	-0.152669675	11.361286959	3.520420382
	C89	-1.410125420	11.780266647	4.316749709
	C90	-0.239754675	11.848386447	2.069388393
	C91	-1.420467463	11.274754272	1.292559542
	C92	-3.473534976	11.062354457	5.418420410
	C93	-3.374665160	11.823086375	6.730905867
	C94	-4.243431282	9.727372896	5.628446590
	C95	-4.498565642	9.017648582	4.319220556
	C96	-5.626995659	9.336743984	3.549770171
	C97	-3.586505923	8.083553937	3.802183125
	C98	-5.827544362	8.765880972	2.292253243
	C99	-3.757782029	7.521407523	2.534758320
	C100	-4.878090600	7.876043791	1.788351756
	C101	4.271161155	-1.505862885	-9.660740131
	C102	2.847965951	-1.305310660	-9.169678403
	C103	1.432660952	-0.116978803	-7.506632253
	C104	1.038005632	1.358545422	-7.643897510
	C105	1.547440794	3.645460234	-6.905517955
	C106	2.791220586	4.525999782	-6.807413620
	C107	5.097527333	4.834137843	-6.103620329
	C108	5.659680817	5.447666498	-7.369947529
	C109	-2.454136813	-0.538041231	-9.889216248
	C110	-3.425630157	0.499698320	-9.315599175
	C111	-2.104815591	-1.593133297	-8.814286265
	C112	-1.448316106	-2.831274369	-9.451853994
	C113	-0.877010860	-3.748483670	-8.393224891
	C114	-3.714389839	2.556958181	-8.027463949
	C115	-3.094788787	3.920755939	-8.255382212
	C116	-4.191954276	2.294637886	-6.579847308
	C117	-3.032879945	1.929156079	-5.695243819
	C118	-2.224223113	2.922131430	-5.119077265
	C119	-2.656648272	0.581274320	-5.563219725
	C120	-1.034891642	2.573423384	-4.472992923
	C121	-1.467753894	0.235592165	-4.916770867
	C122	-0.640953066	1.234177094	-4.394651252
	C123	7.769775593	-5.208285106	-6.183467518
	C124	8.903672361	-5.638472542	-5.237943989
	C125	6.404342800	-5.614202417	-5.605872721
	C126	5.194740555	-5.091924599	-6.313440339
	C127	4.820337812	-5.310122072	-7.623441889
	C128	3.175824571	-4.279766805	-6.558115906
	C129	9.946072883	-7.358056243	-3.879666531
	C130	11.337675382	-7.031044277	-4.372014593
	C131	0.261148140	-6.437800559	-2.096999818
	C132	1.729477190	-6.715421116	-1.777490121
	C133	2.680856240	-6.569939468	-3.002330889
	C134	2.507590779	-7.786576468	-3.953181878
	C135	3.572253512	-7.933609721	-5.043899596
	C136	3.499383804	-9.262998107	-5.828639834
	C137	4.137152189	-6.549742609	-2.509931967
	C138	3.673461372	-0.276792521	-2.245637887
	Fe139	3.896945708	-1.079354692	-4.071547979
	Fe140	2.282035416	0.891708659	-1.564357340
	Fe141	4.867889181	0.661258941	-0.985761775
	Fe142	4.074199359	1.370025977	-3.375061762
	Fe143	3.816316713	3.067384650	-1.295600920
	Fe144	4.900199785	-1.820106422	-1.815409694
	Fe145	2.329688227	-1.701700073	-2.002915634
	H146	-6.680983282	-6.832869976	-2.020463131
	H147	-6.745001579	-5.502275379	-2.963279808
	H148	-9.972924238	-4.303994445	-0.418178192
	H149	-9.056614858	-5.139999296	-2.445864909
	H150	-9.043588226	-6.621189887	-1.492632939
	H151	-8.128418411	-2.678867280	0.945998794
	H152	-9.156281401	-5.936787362	1.859049925
	H153	-9.808415612	-2.976587420	1.440963770

	H154	-8.564127523	-7.236617631	3.694202533
	H155	-6.501269678	-6.176289347	1.657818168
	H156	-8.359514519	-5.758844299	4.658431440
	H157	-3.175414209	-2.366793382	2.596771824
	H158	-5.334256862	-4.045106479	1.774942239
	H159	-4.224797039	-6.564444840	1.354081977
	H160	-5.260631226	-1.874367071	6.794438750
	H161	-6.128037454	-3.368016180	4.490494963
	H162	-4.071579181	-7.179897708	3.019167436
	H163	-4.876678060	-1.894969046	2.435743383
	H164	-4.941214368	-5.410495371	6.071815299
	H165	-6.486375312	-3.171711992	6.770125685
	H166	-0.949058595	-3.961619522	2.419749682
	H167	-0.865373890	-2.505929216	1.396939998
	H168	0.622831869	-3.255298078	2.012302277
	H169	-1.549003066	-1.972777625	3.778665268
	H170	-4.198556266	-7.040534541	7.568531235
	H171	-2.115080572	0.607830653	5.634094727
	H172	-1.997139994	-2.782584755	8.639852421
	H173	-2.036935131	0.458039492	9.992526712
	H174	1.217697866	-0.811860585	3.127376550
	H175	-0.034500932	-0.085675578	4.160696655
	H176	9.287457590	0.185581171	5.048857148
	H177	-2.979961258	1.803256532	6.613868801
	H178	-2.911762139	1.730511567	9.106113351
	H179	-1.202356297	1.841944543	6.542712614
	H180	-1.132175961	1.736587842	9.146355416
	H181	-2.886911750	-0.406768302	7.803562639
	H182	0.140438270	0.009535138	7.600328873
	H183	-1.715411356	-1.675773361	6.103519714
	H184	-0.319471667	-0.264227314	2.418462504
	H185	1.383888537	3.703690881	1.351370808
	H186	0.608031021	3.715990073	2.963124118
	H187	2.654232673	4.034519559	3.602046320
	H188	3.166673356	2.792744552	5.968051612
	H189	4.802131325	3.125136363	2.847209861
	H190	6.652201749	1.637334258	2.145772023
	H191	6.423115791	-0.426213647	5.931527993
	H192	-1.470182919	-4.188913313	5.033218269
	H193	-0.546265484	-7.181921015	4.605879433
	H194	0.039794447	-7.055526312	6.278305606
	H195	-2.587148424	-6.064747359	5.280077014
	H196	-3.676049497	-5.770564870	8.705811867
	H197	4.539202413	1.034047661	6.612436237
	H198	1.207577488	-3.088575714	4.431550586
	H199	-1.421741230	-2.686065614	11.350570021
	H200	-1.610350252	-4.194867485	10.414365745
	H201	1.028735816	-3.782506216	9.839860507
	H202	10.545507716	-8.649044754	2.195929005
	H203	-1.883819714	-4.445866307	-5.716452931
	H204	1.066935263	-5.230678198	-3.378600888
	H205	-2.207509300	-3.391136023	-10.017496777
	H206	-0.657452120	-2.519843492	-10.149341721
	H207	-3.015278838	-1.904545505	-8.279393086
	H208	-2.420520718	3.973630931	-9.142789211
	H209	-3.052047921	-1.045393829	-10.662216851
	H210	-3.354147300	-1.136235213	-2.989130116
	H211	-4.080480576	-4.095133408	-2.610773193
	H212	-1.862380749	1.556924733	-8.448687747
	H213	-4.620309616	2.572962734	-8.668659149
	H214	-0.649831015	0.491770815	-9.905006886
	H215	-1.464035043	-4.732008109	-4.029848669
	H216	-1.441545246	-1.136403424	-8.063224850
	H217	-0.832894088	-0.574471729	-11.147686416
	H218	-6.773384667	-1.932453005	-3.963882890
	H219	-3.510935690	-1.792454124	-1.369812570
	H220	0.885253407	-2.963647899	-8.756383097
	H221	-2.427827443	-3.574742636	-2.339020258
	H222	-3.832831632	1.880022085	3.639723939
	H223	-3.196104966	0.307516857	1.672517884
	H224	-8.287565804	0.227519055	3.569834507
	H225	-7.284686386	0.167586819	0.752155934
	H226	-6.720740602	3.833145607	3.119542662
	H227	-8.046070318	7.692778329	6.521271974
	H228	-6.747096153	4.084690702	6.383461174
	H229	-8.704163209	4.881765479	4.630315839
	H230	-5.959942521	6.251501878	4.333263626
	H231	-10.307236619	9.594221994	3.484921974
	H232	-3.080611970	3.943436885	2.436966259
	H233	-4.581333972	3.963059858	1.497215844

H234	-9.381143871	9.766597944	5.859563423
H235	-8.126124669	5.502047728	3.082588295
H236	-6.045143598	1.949971477	1.711774145
H237	-7.218069098	-1.022975894	2.931632796
H238	-9.671637840	2.655267784	-0.001914220
H239	-1.850550752	1.924140723	-1.965729710
H240	-10.134853458	1.006783895	-0.459186221
H241	10.352179437	1.825709053	3.683163154
H242	8.757365777	2.048064299	3.405445912
H243	1.849858160	5.942158585	0.323093114
H244	-0.612370593	5.268443409	1.701152080
H245	2.081069863	7.415324649	2.581192275
H246	-2.908567133	3.853618903	-0.591811576
H247	-4.630971128	5.093868514	-1.604927224
H248	-6.126768923	4.885051711	-3.270718175
H249	-4.347333453	6.020422491	-0.354532282
H250	-5.113866199	2.730115207	-3.665110298
H251	-6.904108172	2.805101462	-3.564988674
H252	0.715858543	11.661656173	1.559928077
H253	-0.387412582	12.939172802	2.105843772
H254	-0.170784821	10.754947700	-0.280905439
H255	-1.885507009	10.385362017	-0.458274073
H256	-2.890003868	10.756547996	-3.344038310
H257	-2.074327556	6.464697992	-4.257215794
H258	-6.716842389	9.015385664	1.708063907
H259	-2.721294297	7.767693110	4.391602290
H260	-4.005285882	8.603278148	-0.775310658
H261	1.581797801	6.187143343	-2.209019980
H262	2.880653607	7.610861788	-0.900883243
H263	-0.904675863	5.671401591	-1.987227579
H264	-8.213497543	0.830592524	-1.746468207
H265	4.372452236	10.537094517	-0.929219277
H266	-5.833572860	0.431065365	-3.374255615
H267	1.193557945	7.565531038	-3.235234462
H268	-3.009783757	6.842875865	2.124301590
H269	3.956815561	10.312355614	0.781958152
H270	-2.234667027	9.507015991	-4.353515037
H271	-4.362318086	10.061993779	-1.630749530
H272	-5.188729160	-2.029054346	-4.490232072
H273	-5.846532375	-2.584424195	-1.950837310
H274	-6.366695935	10.040689833	3.937205200
H275	-4.996252117	2.142037837	-1.249402275
H276	0.157660393	8.758876686	-1.327933576
H277	-1.063129958	7.902344918	-4.061889446
H278	-0.143241119	5.474458952	-3.555093079
H279	-3.004580791	6.916294229	-1.967882412
H280	1.089325101	7.539874093	5.324026656
H281	0.066068115	9.325120372	2.835950953
H282	0.659494848	11.923942869	4.005912317
H283	2.647369605	8.133708977	4.700643564
H284	-1.935121062	9.837351986	4.578399157
H285	4.844087214	-0.574788074	-9.540612738
H286	4.721095111	-2.250356610	-8.971734721
H287	7.831031021	-4.110766843	-6.226306758
H288	5.195292989	-2.299137071	-11.295405060
H289	3.606457445	-2.681327883	-11.181806077
H290	5.716577919	4.478818477	0.542176311
H291	6.123906916	6.516807559	2.792862825
H292	5.382661934	8.435866760	-0.968613359
H293	4.383761701	5.141283131	1.545375882
H294	7.292217139	7.327484593	1.984042286
H295	5.885976049	4.211187858	-5.653247123
H296	6.418041988	6.732112906	-0.117869446
H297	4.781239803	-8.523269450	2.990100393
H298	6.468153777	-8.875624101	0.449687748
H299	4.003131672	3.004286449	-6.052666773
H300	5.406903382	-1.456224345	3.137486498
H301	1.043132267	3.893071256	-7.847561641
H302	-1.178566039	-0.813312518	-4.819219185
H303	0.876609536	3.961997670	-6.088759665
H304	4.124035683	-5.896979984	3.332362751
H305	2.370042521	1.783710665	-6.163215524
H306	3.514262148	-5.986380831	1.675774050
H307	4.198024882	-8.360309611	1.315143154
H308	5.890442920	-6.536297081	0.919982503
H309	6.371083664	-6.604063905	2.620001316
H310	-4.929930818	1.480261494	-6.610498013
H311	5.127921869	-10.943789237	2.139019577
H312	4.022228315	-12.458657542	-0.271231773
H313	0.287403579	0.969303086	-3.890333840

H314	5.235344515	-13.482666249	2.182071232
H315	3.667702944	-13.500955287	1.709479066
H316	5.242561474	-4.262300647	1.076217910
H317	-0.396824743	3.338078949	-4.025253938
H318	-3.295856072	-0.202370335	-5.976077516
H319	7.106561130	-9.568921097	-4.209808430
H320	6.336963649	-6.715933963	-5.599692343
H321	6.139076560	-8.238450643	-2.258995505
H322	6.248643060	-9.763888508	-5.490997469
H323	5.362110589	4.938048566	-8.317151770
H324	6.094252989	-5.183657769	-1.509480559
H325	1.823796478	-7.709832217	-1.324084393
H326	3.526060946	0.023791524	-7.765580003
H327	2.524599077	-8.685197286	-3.317726478
H328	2.965202271	-4.830702864	-8.582638755
H329	2.203784947	-3.835165242	-6.380804495
H330	5.533414480	-13.367193812	-0.142921912
H331	5.752891587	-2.042131090	1.544236125
H332	7.948772652	-6.375306665	-0.646503728
H333	7.059539514	-4.084441867	-0.991875369
H334	8.420295899	-8.017862580	0.968632268
H335	3.901276834	-4.298397207	4.430165031
H336	3.464256621	-7.116498486	-5.773700662
H337	4.578921166	-7.846490902	-4.612706981
H338	1.507431994	-7.737484053	-4.410732687
H339	1.523392354	-0.398804990	-6.445891352
H340	-2.518516128	3.971155047	-5.209974577
H341	-4.699637165	3.209041416	-6.235959342
H342	2.021057163	-5.969761160	-1.018467246
H343	8.017527178	-7.792599065	-1.288933976
H344	6.780234562	-9.570204436	-1.803020115
H345	4.584307687	-2.769895148	4.786508139
H346	5.335222339	-5.806802437	-8.435318363
H347	6.357417067	-5.303624850	-4.555419326
H348	0.653571331	-0.721243484	-7.976636648
H349	4.864883578	5.640302901	-5.389749840
H350	7.713875384	-1.388166850	4.021161344
H351	7.800041196	-0.532370554	2.466996069
H352	8.871704781	-5.403107281	-7.902336295
H353	7.995326655	-6.738823426	-7.547599992
H354	9.879976285	-8.453874991	-3.789819901
H355	8.196938016	-7.608461759	-5.075057370
H356	11.441012679	-6.967460129	-5.481625813
H357	9.798555101	-6.932871579	-2.873840307
H358	10.470755428	-0.883747555	2.400093470
H359	-2.385483493	11.765372775	7.243398216
H360	9.525835229	-8.840340715	3.644108446
H361	8.989835455	-6.170221026	3.117748681
H362	-4.043643971	11.691770765	4.714430044
H363	-3.670417749	9.085762001	6.315581795
H364	-5.193615809	9.975748648	6.122246133
H365	3.222600297	-0.942619651	0.988833408
H366	-5.930088802	7.476490670	0.199332972
H367	2.432445299	-2.374589041	1.326657750
H368	1.568767955	-1.039716668	0.799672208
Mo369	3.745231406	-3.690970829	-3.453731610
N370	2.447824055	-1.553643208	0.721990013
N371	-7.307569967	-6.280965073	-2.611616765
N372	-9.076248198	-4.349489234	0.059895953
N373	-8.618762830	-5.474760578	2.588513726
N374	-5.963102885	-6.413479953	2.494259153
N375	-4.560909362	-3.946947829	2.430070097
N376	-5.333775605	-3.037623635	5.045237676
N377	-4.374187733	-5.087335082	6.861117034
N378	-1.880026327	-6.318060012	5.975295517
N379	-0.454912849	-4.134460566	5.000922219
N380	-0.810510303	-1.518318542	6.555243488
N381	-1.200669081	-2.539898068	9.233899516
N382	4.511187530	-12.925785677	1.722599584
N383	5.647989585	-10.527407776	1.360212469
N384	4.856654278	-4.420433237	2.012562725
N385	4.592330999	-3.591466305	4.188578988
N386	5.537689799	-2.266297672	2.535980312
N387	8.453337085	-8.443596429	1.921905551
N388	-5.836035475	-1.541738361	-3.844066383
N389	-2.083984531	-4.299236510	-4.722513488
N390	-5.928975162	0.626110970	-2.364836278
N391	-8.202160831	1.537369752	-1.014518666
N392	-7.890311782	0.416452718	1.546355986
N393	-5.674717872	1.763575922	2.646494146

	N394	-2.778676551	2.887949806	-0.264327847
	N395	-2.386064360	0.695072408	-0.284017395
	N396	-6.157066874	4.191852366	3.886654179
	N397	-9.339362277	7.667396828	3.452531680
	N398	-9.242919605	8.965830885	5.249157750
	N399	9.528921910	1.391041261	3.259544053
	N400	6.692230534	6.498937593	1.940100293
	N401	4.803149814	8.649467948	-0.161004128
	N402	2.145121129	8.330935575	-0.896649770
	N403	-2.530513655	7.617560679	-2.576187690
	N404	-2.617097594	9.785791256	-3.457366550
	N405	-3.722053927	9.285513527	-1.484257888
	N406	0.952386769	6.259433265	0.733249793
	N407	1.308020604	7.435215084	3.251296091
	N408	0.209031062	9.955955715	3.624674274
	N409	-1.144965946	10.902587335	0.014034729
	N410	-2.150381723	10.787722624	4.870813852
	N411	4.275714884	-1.914149759	-11.067904051
	N412	2.690939697	-0.391032217	-8.190300168
	N413	1.748542303	2.207452958	-6.870441606
	N414	3.947214324	3.981842446	-6.353143141
	N415	-1.329366265	0.110539117	-10.574140871
	N416	-2.875413214	1.519216502	-8.598238780
	N417	7.960019471	-5.714598740	-7.555251690
	N418	4.150594509	-4.435975949	-5.663289378
	N419	3.551718347	-4.788961109	-7.753722745
	N420	8.919864169	-6.931996606	-4.816282277
	O421	-6.891226995	-4.983235922	-0.071718596
	O422	-7.691739297	-3.513563167	3.296280233
	O423	-5.962410917	-6.558376871	4.775246956
	O424	-2.933013047	-4.995584244	3.630123691
	O425	-3.369968294	-1.898814696	5.062218084
	O426	-3.912554339	-3.361836796	8.293345883
	O427	-1.383520215	-6.622537783	8.195203635
	O428	1.498562914	-5.350248691	4.972729940
	O429	1.454504831	-1.765434283	6.449058585
	O430	0.390425003	-1.041023150	9.834625249
	O431	0.849969999	-3.827776291	11.872799551
	O432	6.279053739	-10.989798800	-0.813836338
	O433	7.357932454	-9.685673809	3.516161391
	O434	10.990017235	-6.390416725	3.441080200
	O435	-5.837770966	-0.522356254	-0.375981466
	O436	-3.777742425	-2.879133917	-5.247872041
	O437	-6.912715269	3.044964396	0.112895169
	O438	-5.896343487	4.307048004	-2.521620338
	O439	-9.778326872	1.506472753	2.268009623
	O440	-5.845013514	0.561663447	4.582915093
	O441	-4.223232520	3.979405312	5.072051479
	O442	-6.278864427	5.997407401	6.923907492
	O443	10.706167676	-1.778080841	4.219643972
	O444	3.530424822	3.033355960	5.095916345
	O445	4.125151986	7.976185578	1.912063902
	O446	1.698242001	10.559598753	-0.545025818
	O447	-0.975861869	7.457514501	0.465634500
	O448	-0.486101985	6.242371821	4.039989911
	O449	2.477501186	4.648718462	2.862387978
	O450	1.407066113	10.269711012	5.550873836
	O451	-1.679511668	12.980853711	4.471967545
	O452	-2.556164477	11.213099857	1.785944766
	O453	-4.316363013	12.428901570	7.218763544
	O454	-5.008419514	7.344237560	0.494238474
	O455	1.909229862	-1.994353457	-9.639049356
	O456	0.146642819	1.726580657	-8.437664058
	O457	2.702682960	5.729696439	-7.103490278
	O458	6.415778724	6.406245681	-7.369814932
	O459	-4.652328510	0.399765179	-9.486744510
	O460	-1.556691732	-4.532329244	-7.739626980
	O461	0.446251325	-3.637196377	-8.150884165
	O462	-3.333303731	4.913595504	-7.582790556
	O463	9.806861886	-4.847801854	-4.899601744
	O464	12.308399771	-6.919851279	-3.635006492
	O465	0.057209528	-5.470289551	-2.993198574
	O466	-0.664836841	-7.022560630	-1.535968820
	O467	4.609927344	-9.836498034	-6.110540365
	O468	2.355109272	-9.678946954	-6.166329679
	O469	4.841779959	-5.490362175	-2.809401358
	O470	4.600855192	-7.519764321	-1.879468186
	O471	2.375108843	-5.332787374	-3.649585017
	O472	8.479365586	-7.209524397	-0.624337928
	O473	6.697547010	-4.958468797	-0.752414047

	O474 -3.915414519 5.576227514 -1.117090369<br>O475 7.193491025 -9.819951060 -5.156637187<br>O476 6.979330448 -8.765227685 -2.344623741<br>S477 3.875389969 5.320565216 -0.822287446<br>S478 2.239468884 2.675627454 -2.910654097<br>S479 1.982858522 -2.236894311 -4.152870373<br>S480 3.302716296 1.651640790 0.344624689<br>S481 0.556314636 -0.448083207 -1.575937789<br>S482 4.318464357 0.616111482 -5.441654467<br>S483 3.479797436 -3.419517393 -1.161750940<br>S484 5.784358176 2.297269833 -2.187357009<br>S485 5.703247629 -2.390645337 -3.825647005<br>S486 6.262994103 -0.946641702 -0.326538360<br>end

product

Fe(139) -2.427<br>Fe(140) -2.397<br>Fe(141) -2.442<br>Fe(142) 2.825<br>Fe(143) 2.942<br>Fe(144) 1.808<br>Fe(145) 1.636	9651826nh3d35ta.car_5<br>C1 -8.023712003 -4.998468265 -0.548953040<br>C2 -8.377521294 -5.780760063 -1.818330844<br>C3 -8.867818547 -3.480010380 1.191691116<br>C4 -8.354096530 -4.171526771 2.450727535<br>C5 -8.112335892 -6.261063322 3.701313618<br>C6 -6.588917838 -6.427638439 3.704966251<br>C7 -4.515573021 -6.394101304 2.410534894<br>C8 -3.930388931 -5.059054712 2.882371348<br>C9 -4.209229559 -2.628566856 2.923102915<br>C10 -4.279629176 -2.499567665 4.450481695<br>C11 -5.484216712 -2.934964855 6.501889552<br>C12 -4.521238001 -3.829670472 7.297344885<br>C13 -3.690297727 -6.108467652 7.659326796<br>C14 -2.215376991 -6.355113146 7.307301217<br>C15 -0.536788821 -6.486261137 5.505382144<br>C16 0.255778858 -5.229744375 5.127423581<br>C17 0.165076550 -2.804031954 4.688667272<br>C18 0.322711424 -1.958147663 5.968972330<br>C19 -0.509355436 -2.069292332 3.513344053<br>C20 0.097999071 -0.675651961 3.307650899<br>C21 -0.454911512 -2.935778944 2.247091034<br>C22 -0.755564033 -0.619787486 7.718823092<br>C23 -0.479955434 -1.414328020 9.007141908<br>C24 -2.022719477 0.265283194 7.805625508<br>C25 -2.040808328 1.089242306 9.097801164<br>C26 -2.108791233 1.187756152 6.582803802<br>C27 -1.050916078 -3.273703164 10.447571024<br>C28 0.395567413 -3.623910091 10.730569488<br>C29 4.891658365 -12.589245145 0.320414428<br>C30 5.688184570 -11.296166818 0.225919298<br>C31 6.190988985 -9.201557017 1.450698157<br>C32 7.413374178 -9.157545135 2.383852456<br>C33 5.091209615 -8.252319345 1.955462526<br>C34 5.548519741 -6.800178023 1.920405285<br>C35 4.427237433 -5.808613999 2.204817818<br>C36 4.973365470 -3.464440193 2.878519770<br>C37 9.665213514 -8.243830139 2.691475059<br>C38 9.889507008 -6.806280225 3.103192621<br>C39 -5.362039449 -1.727041378 -2.457917647<br>C40 -5.723496708 -0.490497620 -1.615705941<br>C41 -3.835254220 -1.932186641 -2.406187155<br>C42 -3.333416405 -3.311054962 -2.878944190<br>C43 -3.080262956 -3.465146059 -4.378527940<br>C44 -5.901226512 1.977481207 -1.822488938<br>C45 -7.033147682 2.244935634 -0.823657442<br>C46 -5.944054820 2.952593424 -3.007430037<br>C47 -9.288077475 1.642193306 -0.082260296<br>C48 -8.989343081 1.190861156 1.352636091<br>C49 -7.448853651 0.047347792 2.873277831<br>C50 -6.249864871 0.810452901 3.444680132<br>C51 -4.536537675 2.538738523 3.144511046<br>C52 -4.956059077 3.606215065 4.164480332<br>C53 -3.834129305 3.287286685 2.001161583<br>C54 -3.211979883 2.393607396 0.983530609<br>C55 -2.933206400 1.042735553 0.955725664<br>C56 -2.257005966 1.806565660 -0.934978937<br>C57 -6.569901838 5.398040051 4.583757207<br>C58 -6.470957316 5.163458410 6.089245861<br>C59 -8.044024317 5.664713071 4.183440456<br>C60 -8.586099162 7.038066711 4.451260148<br>C61 -8.535810214 7.826264839 5.584652299<br>C62 -9.681296563 8.847809569 3.986777095<br>C63 9.250037501 0.175983770 3.904572237

C64	10.263770075	-0.863479934	3.460081998
C65	7.859213616	-0.371599512	3.528032781
C66	6.712959696	0.515229685	3.959491952
C67	6.106706382	0.373922604	5.221300623
C68	6.219470844	1.516961116	3.103577588
C69	5.031376564	1.182352614	5.607466849
C70	5.156168914	2.336961891	3.479916700
C71	4.556780497	2.164670070	4.730414581
C72	5.774207601	6.606330514	0.786707794
C73	4.804027038	7.792720275	0.902297223
C74	4.986128908	5.296970294	0.668821708
C75	3.931990509	9.830145150	-0.199558218
C76	2.464560151	9.596001107	-0.571914172
C77	0.762553659	7.876110848	-1.169488608
C78	0.153548627	7.169999435	0.067791020
C79	0.809704200	6.964607957	-2.416025018
C80	-0.466372234	6.232298050	-2.848373021
C81	-1.553896522	7.078119100	-3.528735360
C82	-2.975135657	8.874099627	-2.541186344
C83	0.393160123	5.559440730	1.886378347
C84	0.330291012	6.444077878	3.141815460
C85	1.184232245	4.287065121	2.235712860
C86	1.545658012	8.081719510	4.499529435
C87	1.061346819	9.520693838	4.603312057
C88	-0.140652692	11.355833383	3.527794314
C89	-1.390476825	11.772871410	4.337081336
C90	-0.238013259	11.856727083	2.081611861
C91	-1.425884665	11.291326420	1.309610978
C92	-3.459293653	11.056300174	5.422517096
C93	-3.358731614	11.819046173	6.734023584
C94	-4.236806502	9.727045770	5.635774179
C95	-4.494266016	9.018284852	4.326986168
C96	-5.618933603	9.344526371	3.555050776
C97	-3.584769139	8.081388759	3.810853729
C98	-5.818328506	8.777423599	2.295837122
C99	-3.755006643	7.523430839	2.541429429
C100	-4.871605676	7.884408765	1.792681515
C101	4.273199246	-1.503891712	-9.686624141
C102	2.857939941	-1.301411934	-9.174391511
C103	1.458443506	-0.115038329	-7.500647017
C104	1.053890792	1.357118023	-7.634645766
C105	1.541778980	3.646559570	-6.878688419
C106	2.782044754	4.532580104	-6.790548286
C107	5.104100423	4.835110023	-6.141268809
C108	5.649309803	5.462641053	-7.406738736
C109	-2.447561565	-0.536325943	-9.916615636
C110	-3.419071554	0.507083595	-9.351084842
C111	-2.106638546	-1.587654001	-8.833963480
C112	-1.449371483	-2.831354837	-9.459961989
C113	-0.882439957	-3.742093543	-8.392666212
C114	-3.702391808	2.568612087	-8.061453069
C115	-3.071210266	3.929442714	-8.281538921
C116	-4.167185539	2.300539104	-6.610431107
C117	-3.016267547	1.910715335	-5.723375327
C118	-2.209258219	2.886351263	-5.115537050
C119	-2.665754815	0.557038488	-5.583477165
C120	-1.054902377	2.516491763	-4.420349510
C121	-1.510732322	0.188226124	-4.889292485
C122	-0.689473867	1.168963377	-4.326124858
C123	7.770631687	-5.228044368	-6.196063363
C124	8.904886298	-5.650669955	-5.249037121
C125	6.404820290	-5.621330475	-5.612765930
C126	5.203473121	-5.119570708	-6.346542351
C127	4.856915846	-5.354530844	-7.662171848
C128	3.180097044	-4.342451028	-6.630682713
C129	9.947191275	-7.358428777	-3.880775797
C130	11.336148432	-7.039478901	-4.386340646
C131	0.247646833	-6.417568317	-2.070279205
C132	1.718904056	-6.677422145	-1.758916832
C133	2.669518411	-6.542364926	-2.988096436
C134	2.510972452	-7.780634338	-3.914971870
C135	3.573555110	-7.923385294	-5.008040820
C136	3.495988812	-9.245522610	-5.803353035
C137	4.121124154	-6.495081260	-2.484450372
C138	3.679008210	-0.267359099	-2.272480629
Fe139	3.997205577	-1.042103353	-4.171255024
Fe140	2.281118966	0.829342008	-1.477956166
Fe141	4.881297134	0.619325689	-0.977373133
Fe142	4.063622370	1.398570270	-3.351871985
Fe143	3.810823993	3.007069135	-1.180075968

	Fe144	4.819505796	-1.814649080	-1.855461501
	Fe145	2.298101858	-1.633184498	-2.333708731
	H146	-6.633307785	-6.820343995	-2.005219692
	H147	-6.702160087	-5.492723212	-2.951588955
	H148	-9.964099886	-4.322313973	-0.433860191
	H149	-9.022063841	-5.151672799	-2.453649294
	H150	-9.003133886	-6.632695058	-1.500216322
	H151	-8.144057408	-2.687048158	0.951876461
	H152	-9.156502710	-5.953981445	1.845841943
	H153	-9.825620544	-2.999180390	1.431665410
	H154	-8.576796934	-7.255637585	3.683869742
	H155	-6.504380000	-6.179903593	1.660891252
	H156	-8.378586356	-5.779557171	4.651907694
	H157	-3.185137539	-2.379677770	2.622662749
	H158	-5.349089110	-4.047775089	1.787885518
	H159	-4.226818609	-6.573006371	1.365518481
	H160	-5.291148986	-1.900734674	6.816242609
	H161	-6.147321229	-3.381099068	4.499925253
	H162	-4.078866332	-7.184194178	3.032655101
	H163	-4.883908307	-1.899216915	2.455312645
	H164	-4.978030911	-5.438758031	6.086081977
	H165	-6.514014534	-3.200635975	6.778844882
	H166	-1.004106641	-3.877724983	2.391297538
	H167	-0.896015856	-2.401270102	1.391284027
	H168	0.578965772	-3.195967594	1.972473765
	H169	-1.565116614	-1.920253450	3.785819356
	H170	-4.216311418	-7.070540583	7.564527091
	H171	-2.148053593	0.626496910	5.639604024
	H172	-1.999529867	-2.783964491	8.603293664
	H173	-2.051403659	0.456332838	9.997079227
	H174	1.184687525	-0.715753074	3.149608954
	H175	-0.091044431	-0.020594337	4.170689814
	H176	9.304928484	0.239910923	5.011343174
	H177	-3.011523006	1.812774525	6.631009148
	H178	-2.935674768	1.728682461	9.119172379
	H179	-1.234739814	1.859103591	6.548996136
	H180	-1.155741938	1.743339393	9.153365469
	H181	-2.902077470	-0.403936507	7.806367576
	H182	0.123791504	0.033022066	7.608962755
	H183	-1.721387613	-1.651934244	6.096135735
	H184	-0.341860789	-0.202034333	2.417291266
	H185	1.337374806	3.691116406	1.318722227
	H186	0.565856855	3.701665039	2.932924641
	H187	2.621795888	3.999631131	3.560445383
	H188	3.123011852	2.735899020	5.907586831
	H189	4.764644813	3.087752341	2.794643112
	H190	6.647856186	1.638423878	2.105575333
	H191	6.462013470	-0.396701654	5.910477100
	H192	-1.480400870	-4.141355080	5.021120368
	H193	-0.559633536	-7.142473804	4.621791821
	H194	0.033063562	-6.998880594	6.290546660
	H195	-2.596715515	-6.012100115	5.297440850
	H196	-3.707311233	-5.807208973	8.713790858
	H197	4.555674698	1.035644789	6.580242677
	H198	1.197905124	-3.047638343	4.409167455
	H199	-1.456274909	-2.726934395	11.314415359
	H200	-1.601367067	-4.223264531	10.354650148
	H201	1.033368259	-3.763054579	9.827028173
	H202	10.558591338	-8.613597967	2.165387808
	H203	-1.884315443	-4.450267044	-5.714043504
	H204	1.038868463	-5.225247213	-3.377912523
	H205	-2.206046171	-3.396056114	-10.024209692
	H206	-0.654323533	-2.526979905	-10.155884949
	H207	-3.019913484	-1.895262990	-8.301685331
	H208	-2.395646085	3.982167769	-9.168148824
	H209	-3.041394360	-1.044705299	-10.693393308
	H210	-3.353978981	-1.133836970	-2.991341845
	H211	-4.055796958	-4.097521474	-2.594770026
	H212	-1.855437184	1.560213611	-8.480411157
	H213	-4.613071568	2.591854622	-8.696145164
	H214	-0.637136854	0.481918328	-9.916259584
	H215	-1.460118453	-4.733863661	-4.027090702
	H216	-1.444636741	-1.129894901	-8.081896382
	H217	-0.814713203	-0.584606505	-11.157815982
	H218	-6.768326764	-1.940567601	-3.959480954
	H219	-3.502041248	-1.786226218	-1.369358016
	H220	0.882491044	-2.957368618	-8.749064841
	H221	-2.407465755	-3.555265167	-2.338476296
	H222	-3.835658863	1.870204170	3.662468474
	H223	-3.143105527	0.307561144	1.727979427

	H224	-8.287721986	0.219146011	3.561556004
	H225	-7.277504962	0.171058418	0.746826234
	H226	-6.707159368	3.844788275	3.119483424
	H227	-8.058548813	7.688874128	6.548424561
	H228	-6.725623709	4.119615537	6.394524823
	H229	-8.682037593	4.893200212	4.649992269
	H230	-5.940792853	6.261576912	4.313806181
	H231	-10.270945686	9.617353805	3.494059350
	H232	-3.073138671	3.936885452	2.466192942
	H233	-4.566369678	3.953829578	1.514008059
	H234	-9.382932524	9.767148292	5.885040878
	H235	-8.120070476	5.511462626	3.095643965
	H236	-6.034801591	1.948331535	1.720373061
	H237	-7.216919063	-1.027314226	2.917876632
	H238	-9.642745121	2.679133674	-0.006090170
	H239	-1.836683367	1.904593162	-1.932923866
	H240	-10.118663493	1.034563263	-0.465941759
	H241	10.345762174	1.885140996	3.635417750
	H242	8.749101562	2.085573012	3.358860967
	H243	1.828467667	5.936683293	0.312176124
	H244	-0.645663264	5.267213618	1.673849595
	H245	2.054418492	7.399860649	2.563154079
	H246	-2.901240199	3.834547205	-0.573528221
	H247	-4.621468155	5.097685231	-1.594788252
	H248	-6.100557375	4.885976070	-3.276767682
	H249	-4.341340064	6.026533223	-0.346114751
	H250	-5.089324059	2.731265366	-3.668065913
	H251	-6.879628849	2.804333012	-3.570657640
	H252	0.713789800	11.672556490	1.564163154
	H253	-0.382545670	12.947579981	2.128565884
	H254	-0.186064729	10.755873739	-0.267532487
	H255	-1.904027906	10.394835475	-0.435220836
	H256	-2.900484320	10.754140599	-3.363544955
	H257	-2.094311547	6.462679163	-4.265030767
	H258	-6.704336770	9.032894303	1.709340157
	H259	-2.723696459	7.758629042	4.402887953
	H260	-3.992123445	8.614133019	-0.771089287
	H261	1.578599230	6.197519362	-2.233754359
	H262	2.860748664	7.618252034	-0.905498527
	H263	-0.902415907	5.664860755	-2.011120027
	H264	-8.197322349	0.844743422	-1.750635998
	H265	4.348955310	10.545978932	-0.920626179
	H266	-5.821504834	0.430052860	-3.375935389
	H267	1.180871608	7.575868903	-3.255665641
	H268	-3.009162458	6.842315436	2.131948571
	H269	3.924167912	10.316996693	0.786471094
	H270	-2.257249951	9.497295528	-4.372627469
	H271	-4.361146776	10.065864221	-1.631456162
	H272	-5.183047274	-2.033469340	-4.484690722
	H273	-5.841925955	-2.581856639	-1.942872589
	H274	-6.356117041	10.051466731	3.941831009
	H275	-4.972377458	2.141598008	-1.254921425
	H276	0.136621421	8.755938428	-1.346745456
	H277	-1.080348328	7.899786593	-4.081967647
	H278	-0.148153424	5.480566524	-3.585288937
	H279	-2.998411419	6.921098260	-1.961750483
	H280	1.062194545	7.513206975	5.307789913
	H281	0.061214847	9.324919094	2.826386080
	H282	0.675802143	11.913202895	4.012065641
	H283	2.627513814	8.092331151	4.691365880
	H284	-1.937603633	9.831241766	4.559578896
	H285	4.846211841	-0.572715861	-9.573083425
	H286	4.731055434	-2.244727543	-8.999190199
	H287	7.835749815	-4.131178956	-6.253495951
	H288	5.180501455	-2.298027451	-11.327414570
	H289	3.594596857	-2.686937326	-11.192839695
	H290	5.708584715	4.477689508	0.565469649
	H291	6.110809026	6.531103118	2.803369349
	H292	5.368376566	8.451479840	-0.962732797
	H293	4.374891682	5.136548993	1.566060949
	H294	7.257004734	7.364845086	1.986950276
	H295	5.901417568	4.209623280	-5.709744282
	H296	6.400015459	6.721697695	-0.113122851
	H297	4.793320197	-8.549325475	2.975185863
	H298	6.485111671	-8.897814836	0.437799788
	H299	4.026227548	2.994253713	-6.119214342
	H300	5.400533174	-1.498721478	3.142481250
	H301	1.022329851	3.895553647	-7.811812869
	H302	-1.243951328	-0.866459017	-4.789626104
	H303	0.881202594	3.955040598	-6.050865187

	H304	4.067023023	-5.919533079	3.234661401
	H305	2.385048388	1.790622921	-6.155774910
	H306	3.566524377	-5.979745192	1.539704649
	H307	4.218061416	-8.384837090	1.296796017
	H308	5.955608666	-6.576973814	0.923894076
	H309	6.360306002	-6.624438394	2.643570433
	H310	-4.916612397	1.496909425	-6.640658880
	H311	5.141601004	-10.964151415	2.123217938
	H312	3.992502265	-12.434379043	-0.294389780
	H313	0.216397400	0.888036411	-3.789289381
	H314	5.206033291	-13.503003759	2.139192165
	H315	3.635944638	-13.492896662	1.677074606
	H316	5.346681242	-4.284599394	1.065229668
	H317	-0.421646580	3.271003264	-3.947569851
	H318	-3.302928915	-0.213815778	-6.022602343
	H319	7.106184527	-9.572420805	-4.208840552
	H320	6.337782743	-6.722536613	-5.575783553
	H321	6.130030761	-8.230331174	-2.257417149
	H322	6.244838755	-9.762720991	-5.486078306
	H323	5.338102859	4.962860670	-8.354397366
	H324	6.067388300	-5.147184691	-1.450768762
	H325	1.823300999	-7.663892112	-1.292809585
	H326	3.550768686	0.038105498	-7.785384126
	H327	2.548241705	-8.668564762	-3.264033974
	H328	3.012924011	-4.908868037	-8.655598520
	H329	2.198013133	-3.915344312	-6.473688774
	H330	5.489734111	-13.368344691	-0.182474160
	H331	5.779266278	-2.086458420	1.581393966
	H332	7.939704896	-6.371348337	-0.644921316
	H333	7.063644074	-4.071225797	-0.943805191
	H334	8.421576023	-8.012385305	0.956893808
	H335	3.736444465	-4.310170019	4.304913979
	H336	3.461670173	-7.100885894	-5.730961226
	H337	4.581289511	-7.837074401	-4.578091703
	H338	1.508489200	-7.759813483	-4.370883767
	H339	1.550655962	-0.399977906	-6.441174931
	H340	-2.483239811	3.940244685	-5.212966861
	H341	-4.659588267	3.218175161	-6.253526634
	H342	2.005446750	-5.922654586	-1.007958200
	H343	8.014183338	-7.786552999	-1.295742721
	H344	6.764727259	-9.570365814	-1.807348935
	H345	4.428453190	-2.801970565	4.730992166
	H346	5.397645501	-5.844740253	-8.462121734
	H347	6.348812132	-5.282148558	-4.570916130
	H348	0.680657999	-0.723039927	-7.967963080
	H349	4.875431362	5.629609670	-5.413840580
	H350	7.761735991	-1.363805458	3.993911336
	H351	7.828528928	-0.519576755	2.436231570
	H352	8.867879541	-5.449929388	-7.916234259
	H353	7.984571368	-6.777157003	-7.541845609
	H354	9.880311447	-8.453456544	-3.780545305
	H355	8.196827579	-7.618387816	-5.073211000
	H356	11.429753639	-6.981365958	-5.497090348
	H357	9.805451947	-6.923330702	-2.878936162
	H358	10.509788096	-0.822151408	2.362399005
	H359	-2.371058282	11.759095471	7.248089151
	H360	9.555807492	-8.831652162	3.619848478
	H361	8.976213241	-6.163559542	3.110834607
	H362	-4.027017543	11.688060064	4.718732964
	H363	-3.667202098	9.084547859	6.324976997
	H364	-5.186259063	9.982277850	6.127903469
	H365	3.301192804	-0.866904510	1.537705465
	H366	-5.921343493	7.492359082	0.200645076
	H367	2.454789215	-2.027888660	2.328913224
	H368	1.970967169	-1.482337560	0.839091968
	Mo369	3.701666919	-3.656919477	-3.522420046
	N370	2.791041112	-1.745556551	1.404451985
	N371	-7.260222068	-6.276135604	-2.603209530
	N372	-9.071318882	-4.361881344	0.052111795
	N373	-8.628037836	-5.491759371	2.581610822
	N374	-5.970385287	-6.423990537	2.498114315
	N375	-4.576104988	-3.952776403	2.444164580
	N376	-5.355353776	-3.053189985	5.059157019
	N377	-4.406870402	-5.115346671	6.872298630
	N378	-1.888022648	-6.267608602	5.990343344
	N379	-0.465029336	-4.089811970	4.980149820
	N380	-0.816455385	-1.490556763	6.545820209
	N381	-1.208276147	-2.542484303	9.204358115
	N382	4.487253124	-12.929391241	1.691618504
	N383	5.669430652	-10.551215901	1.347968109

	N384	4.919989117	-4.449103449	1.983586837
	N385	4.456083743	-3.613827530	4.121483079
	N386	5.612586836	-2.317665883	2.575481996
	N387	8.462800261	-8.440320270	1.908326719
	N388	-5.832161124	-1.546430046	-3.840052381
	N389	-2.080294624	-4.300723360	-4.719591970
	N390	-5.913135786	0.627873689	-2.366732833
	N391	-8.181168287	1.550136982	-1.017392560
	N392	-7.883332632	0.421104292	1.540408989
	N393	-5.671932481	1.760947763	2.657810506
	N394	-2.767202646	2.872961348	-0.236862776
	N395	-2.346521435	0.684833898	-0.236606462
	N396	-6.146982827	4.195244026	3.892930205
	N397	-9.306412943	7.688472220	3.460562545
	N398	-9.233851148	8.972775696	5.268614919
	N399	9.528496916	1.437153812	3.214536694
	N400	6.675446523	6.522416973	1.947532464
	N401	4.781925162	8.654504509	-0.157203620
	N402	2.124586944	8.335320627	-0.907855905
	N403	-2.533048466	7.619774354	-2.580091646
	N404	-2.639809728	9.780287302	-3.477990237
	N405	-3.716428806	9.292931383	-1.486154595
	N406	0.926527309	6.252035597	0.712307743
	N407	1.283399754	7.417040956	3.235543599
	N408	0.214319186	9.948853755	3.618359376
	N409	-1.157637231	10.906722965	0.033831603
	N410	-2.137244367	10.777202597	4.876605763
	N411	4.261639315	-1.915988587	-11.091716987
	N412	2.713123788	-0.388716052	-8.190551142
	N413	1.751357005	2.209708655	-6.852713443
	N414	3.953204591	3.982181175	-6.386094156
	N415	-1.312982099	0.106025800	-10.592394211
	N416	-2.867427996	1.529485340	-8.638242155
	N417	7.956014213	-5.752842215	-7.561996948
	N418	4.142255469	-4.471368195	-5.717231869
	N419	3.582425628	-4.855636188	-7.815701717
	N420	8.917859778	-6.939673127	-4.815458898
	O421	-6.881923846	-4.982648424	-0.055322764
	O422	-7.718963042	-3.526287611	3.302355333
	O423	-5.981505382	-6.580034012	4.778624911
	O424	-2.940364018	-4.997004434	3.634295523
	O425	-3.389398073	-1.918869260	5.089341623
	O426	-3.931160321	-3.391259917	8.300498400
	O427	-1.415613604	-6.668812346	8.201421824
	O428	1.485872660	-5.308318605	4.958192240
	O429	1.450156121	-1.725747656	6.432798365
	O430	0.371390751	-1.043268632	9.833329530
	O431	0.828385937	-3.823357450	11.856729335
	O432	6.265930004	-10.996712795	-0.839373871
	O433	7.391905233	-9.712201430	3.496556384
	O434	10.979500203	-6.361786866	3.428939542
	O435	-5.822724315	-0.515134550	-0.374816908
	O436	-3.775053374	-2.881363841	-5.242192925
	O437	-6.884400204	3.050871180	0.110694204
	O438	-5.874841260	4.307954190	-2.526397922
	O439	-9.762615427	1.525741888	2.262515037
	O440	-5.849484472	0.546429390	4.585892988
	O441	-4.225728644	3.967211221	5.094607812
	O442	-6.228397209	6.030812521	6.912766310
	O443	10.745838694	-1.713734100	4.195259633
	O444	3.495092679	2.989351356	5.042581254
	O445	4.088080141	7.963775545	1.904535970
	O446	1.676238673	10.562990695	-0.553966962
	O447	-0.995347310	7.455961377	0.441471518
	O448	-0.515931109	6.227503145	4.018010991
	O449	2.440293330	4.625094373	2.831223616
	O450	1.411519062	10.237775445	5.548708244
	O451	-1.648735567	12.972843202	4.513891847
	O452	-2.560900068	11.242975579	1.806805579
	O453	-4.298675445	12.427974968	7.221661788
	O454	-5.000826119	7.355784042	0.497325054
	O455	1.911116386	-1.986425054	-9.631888121
	O456	0.162854983	1.720836930	-8.430541009
	O457	2.677953622	5.744151815	-7.048578253
	O458	6.406687473	6.420250973	-7.406752334
	O459	-4.645735699	0.408252145	-9.521824681
	O460	-1.564848337	-4.522481187	-7.737484508
	O461	0.439504392	-3.629805643	-8.145952345
	O462	-3.297637004	4.918751185	-7.599128458
	O463	9.810935194	-4.859592059	-4.919222805

O464	12.313751248	-6.928328006	-3.658626753
O465	0.038072410	-5.462045892	-2.978699266
O466	-0.673214997	-6.999002346	-1.497638234
O467	4.604552761	-9.818488882	-6.094192835
O468	2.349057866	-9.656117993	-6.139183666
O469	4.800233084	-5.417090154	-2.766421342
O470	4.592765266	-7.462363022	-1.852843372
O471	2.355299853	-5.320228081	-3.665224126
O472	8.477663743	-7.201254445	-0.634768352
O473	6.682667667	-4.935848814	-0.698905971
O474	-3.907464625	5.581509192	-1.106859958
O475	7.190588721	-9.822023952	-5.155809237
O476	6.966238429	-8.763294007	-2.345359138
S477	3.850103565	5.269582098	-0.790694225
S478	2.210151909	2.629175961	-2.796058507
S479	2.091208436	-2.173420737	-4.477432646
S480	3.335486296	1.532757364	0.423130493
S481	0.666495693	-0.631809150	-1.337258977
S482	4.430343862	0.718365359	-5.447702360
S483	3.267448728	-3.260978691	-1.216960580
S484	5.776249914	2.294141467	-2.123345222
S485	5.723216546	-2.425461146	-3.794548747
S486	6.249260461	-1.011437669	-0.395629128
end			

core-S2BH to resting

35, S=3/2

reactant

bm502bhtohiso.car_1

C1	-3.225899167	3.305625220	-8.414186250
C2	-2.621339857	4.493432903	-9.171877346
C3	-5.296000955	2.264274398	-7.601390574
C4	-5.081429226	0.813004772	-8.024906182
C5	-4.450592096	-0.757687306	-9.783750855
C6	-3.227699635	-1.387916978	-9.107308593
C7	-1.089135652	-1.025368344	-7.987007728
C8	-1.439023542	-1.448039897	-6.558566824
C9	-2.800785479	-1.030289441	-4.573029634
C10	-3.534686388	-2.380074997	-4.540516566
C11	-5.152588841	-3.860990327	-5.596988359
C12	-4.343900075	-5.101077451	-5.997227668
C13	-2.801339096	-6.088972461	-7.628100764
C14	-1.399516337	-6.359412000	-7.064349887
C15	0.683942849	-5.352962736	-6.218439474
C16	0.918093187	-5.208181453	-4.710429898
C17	0.189406728	-4.183701546	-2.579618254
C18	-0.549507335	-5.315562751	-1.829175703
C19	-0.260508528	-2.778664852	-2.139335683
C20	-0.208096260	-2.657462116	-0.612396964
C21	0.611667735	-1.706986040	-2.803849770
C22	-2.701660010	-6.454713904	-1.417067965
C23	-2.661420485	-7.822951819	-2.131285686
C24	-4.146505560	-5.943314459	-1.203631832
C25	-5.040211688	-7.039137935	-0.607964534
C26	-4.153345275	-4.699702522	-0.308193590
C27	-3.069442283	-9.143659006	-4.128337028
C28	-1.834757420	-9.994196591	-3.906431552
C29	9.935329806	-4.457917532	-8.140571810
C30	9.804148063	-4.346945792	-6.622800439
C31	8.458505174	-5.167044920	-4.685861888
C32	8.903419615	-6.549960573	-4.173614904
C33	6.950766904	-4.937748806	-4.481778662
C34	6.523282855	-5.010713628	-3.014228881
C35	5.084985401	-4.529694779	-2.828808108
C36	3.629662379	-5.057845785	-0.846695091
C37	10.172656065	-7.740018410	-2.446645245
C38	9.503166167	-8.093046134	-1.138264707
C39	-2.056187266	4.256802784	-4.064321333
C40	-3.288017866	3.816498728	-3.252825075
C41	-0.830550420	3.498717390	-3.500442245
C42	0.448367127	3.554125939	-4.362621940
C43	1.380114872	4.740112720	-4.105151621
C44	-4.579383215	4.421806779	-1.222194645
C45	-5.991887967	4.076742336	-1.715725065
C46	-4.581691798	5.622458498	-0.261999806
C47	-7.641343022	4.328626721	-3.510268147
C48	-7.842137630	2.853177145	-3.881010550

C49	-6.814073059	0.650722005	-4.199989834
C50	-6.521363445	-0.251952432	-3.001393588
C51	-6.082623167	-0.476146224	-0.608922366
C52	-7.403469231	-1.041735187	-0.069964791
C53	-5.468734093	0.325986076	0.548688351
C54	-4.035234396	0.678217871	0.375734810
C55	-3.016593855	0.124570016	-0.368093700
C56	-2.113023085	1.724667903	0.758342555
C57	-9.726858095	-0.490113117	0.447989099
C58	-10.219680962	-1.862378249	-0.007790770
C59	-10.736946403	0.600362071	0.009782968
C60	-11.960742127	0.771409845	0.864307798
C61	-12.789147085	-0.160521340	1.460929579
C62	-13.512825461	1.892138633	1.877314875
C63	4.944901399	-7.657961182	3.952714561
C64	6.413385146	-7.792052570	3.595126444
C65	4.308596981	-6.706653056	2.910686577
C66	2.830397165	-6.485541904	3.129999496
C67	1.870402669	-7.351530676	2.578084377
C68	2.371029666	-5.391347007	3.884776420
C69	0.500091503	-7.127863804	2.753645603
C70	1.008843597	-5.159728242	4.075421763
C71	0.073272855	-6.023410831	3.499623226
C72	0.498181858	-2.432395354	8.366381002
C73	-0.859892144	-1.938057402	8.889523521
C74	0.573009169	-2.220588481	6.855522220
C75	-2.022830620	-0.291377526	10.311891607
C76	-2.866240998	0.606863198	9.404115646
C77	-3.011739797	1.662258140	7.149203730
C78	-3.668072404	0.736860052	6.094451188
C79	-1.967952628	2.614411869	6.522268926
C80	-2.378572353	3.448074415	5.304141304
C81	-3.271356585	4.668469724	5.585411335
C82	-5.682776289	4.704276969	6.236951465
C83	-3.497075995	-1.204769462	4.625270239
C84	-4.536633972	-2.171267796	5.214324175
C85	-2.431828071	-2.039026398	3.893373732
C86	-5.034790731	-3.659449779	7.098045995
C87	-6.195906197	-3.323349953	8.023889487
C88	-7.533731050	-1.558918041	9.061442073
C89	-9.026977392	-1.639099002	8.667751504
C90	-7.204683603	-0.164580545	9.608832569
C91	-7.419856873	0.956777532	8.597724991
C92	-10.675909177	-1.843361853	6.879176145
C93	-11.558974015	-2.963042134	7.411022085
C94	-10.668758680	-1.872417422	5.325836745
C95	-9.914579122	-0.696216361	4.751005694
C96	-10.568891015	0.520322590	4.504547655
C97	-8.530981523	-0.765899854	4.511651436
C98	-9.860910060	1.644546681	4.072959348
C99	-7.801765734	0.356437223	4.111079778
C100	-8.478177731	1.556593457	3.901548716
C101	8.103362700	6.451792308	2.145463019
C102	6.734836094	6.666529156	1.522330363
C103	4.367469402	5.907246371	1.557219367
C104	3.390895698	6.424388674	2.618843031
C105	2.261095893	5.857973628	4.732433690
C106	2.661550338	5.314131812	6.100217626
C107	3.834355077	3.668459057	7.445128384
C108	4.566026887	4.576894036	8.411378660
C109	2.782548635	9.771633673	-0.523072962
C110	1.279526734	9.818516534	-0.227918269
C111	3.113643336	8.514223028	-1.359699929
C112	4.505675657	8.633327996	-2.007556166
C113	4.949922159	7.307308183	-2.587137745
C114	-0.571170153	9.076789459	1.185144489
C115	-0.741874803	9.195906287	2.687394956
C116	-1.410759905	7.963234297	0.513766233
C117	-0.752370101	6.624828614	0.686822029
C118	-0.904088461	5.913648714	1.888663058
C119	0.149893263	6.150650050	-0.279444767
C120	-0.103664454	4.800633847	2.150136910
C121	0.947092790	5.033508161	-0.018005709
C122	0.848838358	4.385503302	1.216321730
C123	10.983938915	1.321771022	0.357293623
C124	11.635847698	-0.066257937	0.449874524
C125	9.967997981	1.367796262	-0.793521934
C126	9.136774349	2.606463258	-0.886517313
C127	9.580886896	3.900069724	-1.065571561
C128	7.377866318	3.845738695	-1.268225494

	C129	12.754494553	-1.905811477	-0.677901985
	C130	13.847664491	-2.025424242	0.361667460
	C131	4.336964331	0.947989960	-5.198901439
	C132	5.431448749	-0.030400079	-4.756938916
	C133	6.600413909	0.629572353	-3.971381155
	C134	7.539983444	1.388717104	-4.945613680
	C135	8.857402069	1.878167238	-4.339389422
	C136	9.866684552	2.434148915	-5.365333940
	C137	7.417708222	-0.456783872	-3.252764480
	C138	3.750814817	0.303843606	1.814321641
	Fe139	5.239847064	1.683208037	1.595456674
	Fe140	1.824901609	0.481335132	1.972012781
	Fe141	3.559454374	-1.297646018	2.947449304
	Fe142	3.699682037	1.237500651	3.603869065
	Fe143	1.660602498	-0.334714064	4.502559618
	Fe144	5.158040888	-0.832926453	0.939258036
	Fe145	3.487736223	0.787733739	-0.069015173
	H146	-0.680185561	3.867928713	-9.164475237
	H147	-1.021606993	4.943562994	-7.985605594
	H148	-5.115890588	4.043275809	-8.766916641
	H149	-3.183857234	5.404957872	-8.912733438
	H150	-2.808743991	4.313633904	-10.244521309
	H151	-4.985223397	2.324168375	-6.548129822
	H152	-4.637020331	1.382540800	-9.942436149
	H153	-6.371655047	2.485302024	-7.644439405
	H154	-4.274714812	-0.720347562	-10.867173775
	H155	-2.367750736	0.470712484	-8.823570699
	H156	-5.295012750	-1.433995920	-9.596995009
	H157	-1.960561254	-1.108505156	-3.872774548
	H158	-2.717816711	0.145212658	-6.367004841
	H159	-0.332942318	-0.229781576	-7.950378928
	H160	-5.586949822	-4.059562084	-4.608199300
	H161	-4.572829670	-1.896934710	-6.256831049
	H162	-0.651279512	-1.898969251	-8.484239327
	H163	-3.459156110	-0.224887376	-4.218032266
	H164	-3.489104660	-4.061371074	-7.564276150
	H165	-5.974909068	-3.735727638	-6.314649117
	H166	0.633906327	-1.798443004	-3.899963531
	H167	0.235199833	-0.703450553	-2.559720691
	H168	1.646987380	-1.766059968	-2.433441234
	H169	-1.308877478	-2.649640094	-2.458006122
	H170	-2.702626369	-5.933736865	-8.712943284
	H171	-3.557573687	-3.881779790	-0.735613450
	H172	-3.354853887	-7.043210109	-3.940488028
	H173	-5.124045620	-7.916272330	-1.267048954
	H174	-0.520364051	-1.651916933	-0.298843067
	H175	0.814788938	-2.816441294	-0.241864769
	H176	4.483088138	-8.657406998	3.833632836
	H177	-5.181177113	-4.331842325	-0.177541505
	H178	-6.052775662	-6.643100375	-0.443414635
	H179	-3.748134754	-4.937084813	0.689648004
	H180	-4.645031670	-7.379062712	0.362991614
	H181	-4.552930539	-5.664125684	-2.192785921
	H182	-2.238776352	-6.645649147	-0.438632901
	H183	-2.337610794	-4.760478986	-2.694773364
	H184	-0.862143032	-3.386660617	-0.109978025
	H185	-1.611610955	-1.377691623	3.564294634
	H186	-2.914763342	-2.467648750	3.002777866
	H187	-1.761421704	-3.872591557	4.209536890
	H188	-1.814178625	-6.374008751	3.218658777
	H189	0.665154971	-4.295902605	4.644634208
	H190	3.090129844	-4.688314147	4.308772336
	H191	2.192599871	-8.202472805	1.972259871
	H192	-0.592914237	-3.819496498	-4.551131871
	H193	1.285743266	-4.583414333	-6.727923283
	H194	1.079935332	-6.335101289	-6.499952544
	H195	-1.100533787	-4.331535593	-6.743751345
	H196	-3.384700568	-7.003571682	-7.465861133
	H197	-0.232906508	-7.789888634	2.284955325
	H198	1.247157827	-4.308786816	-2.317167883
	H199	-3.955426621	-9.722959128	-3.821567381
	H200	-3.143807111	-8.944036026	-5.208862541
	H201	-0.885333722	-9.433009149	-3.747374809
	H202	11.263877774	-7.690857054	-2.303565599
	H203	3.352143978	5.271412313	-4.086952454
	H204	5.007008604	1.847137459	-3.547720785
	H205	4.459668215	9.365417552	-2.826534230
	H206	5.239267139	8.985178282	-1.266516248
	H207	2.361567744	8.369210393	-2.150830317
	H208	0.098648929	9.719437804	3.202561353

	H209	2.952452870	10.650840807	-1.162739824
	H210	-0.622631069	3.874389730	-2.485580937
	H211	0.180771598	3.581019786	-5.433786090
	H212	1.491224629	8.513354416	1.375643592
	H213	-0.964551349	10.045290847	0.810194570
	H214	3.581927650	9.114210738	1.276484528
	H215	3.088413088	3.554452859	-4.384244701
	H216	3.055909961	7.630203761	-0.704918734
[truncated: 382,495 more chars]
