# Supplementary material for: A Proposed Complete‐Cycle Mechanism for Conversion of N2 to NH3 by Mo‐Nitrogenase
Source: Chembiochem. 2026 Jul 24;27(14):e70429. doi: 10.1002/cbic.70429 (PMC13398036; doi:10.1002/cbic.70429)
Supplement: Supplementary file 2 — Supplementary Material [file CBIC-27-e70429-s001.pdf]

A Proposed Complete-cycle Mechanism for Conversion of N_2 to NH_3 by Mo-Nitrogenase

Ian Dance

School of Chemistry, UNSW Sydney, NSW 2052, Australia

E-mail: i.dance@unsw.edu.au

Supporting Information

Contents

1. Protein model

2. Computational constraints

3. Density functional procedures

4. Validation

5. Determination of transition states

6. Energetically favorable electronic states

7. Movement of Val70 and the contiguous chain

8. Libratory movement of the Arg96 sidechain

9. Non-obligatory H2 evolution

10. Truncated models for quantum tunneling calculations

References

1. Protein model

The quantum computed protein model is an extract from crystal 3U7Q, shown in Chart S1. The six principal chains surrounding FeMo-co are highlighted with different colours, and the connecting hydrogen bonds are marked. Homocitrate, the His442 ligation of Mo, and relevant nearby water molecules are included (485 atoms total). Details, and the rationale for inclusion of amino acids and for truncation of uninvolved sidechains, are provided in the caption for Chart S1. Investigations of the protonation state of homocitrate using crystal structures at various pH (summarised in ref [1]), vibrational circular dichroism spectroscopy,[2] QM/MM calculations,[1, 3] and quantum refinement,[3] indicate that the coordinated alcoholate O7 atom is protonated, and hydrogen bonded to O1. This proton is now included in the model, although it is not shown in Chart S1.

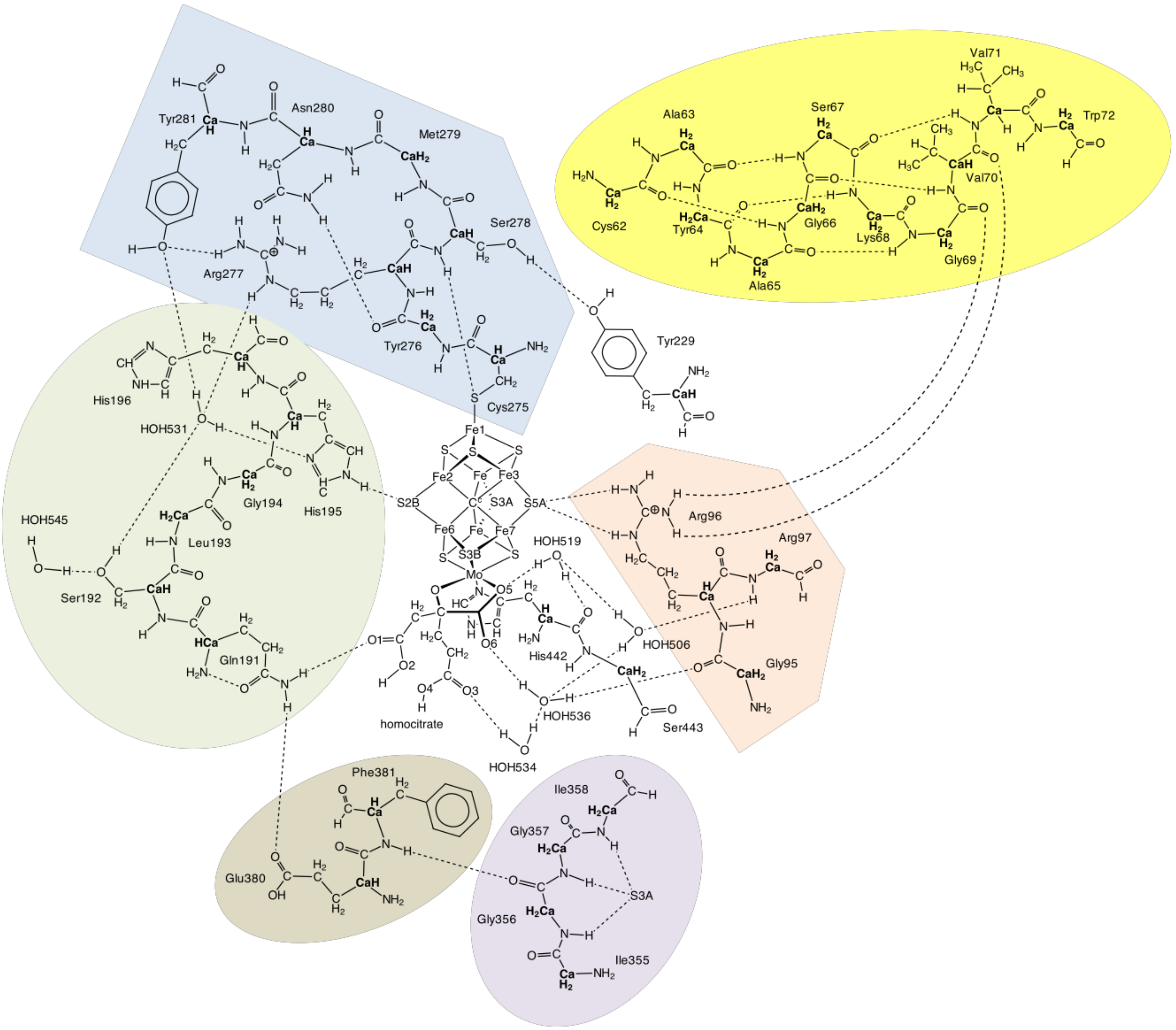

Chart S1. The quantum computed model, with residues and water molecules labelled according to *Azotobacter vinelandii* protein, crystal structure 3U7Q. Cα are labelled as Ca, and hydrogen bonds are broken lines. Non-influential sidechains are truncated to gly. In the ‘front’ chain (yellow highlight) that extends over the Fe2Fe3Fe6Fe7 face of FeMo-co the crucial residues Val70 and Val71 are complete, while 62, 63, 64, 65 67, 68 and 72 are modelled as Gly. In the 191 to 196 chain (highlight green) the significant sidechains of Gln191, Ser193, His195 and His196 are complete. In the 275-281 chain (blue highlight) Cys275, Arg277, Ser278, Asn280, Tyr281 near the N_2 pathway are complete: Tyr229 is included in full because it borders the reaction domain and has an important hydrogen bond with Ser278. In chain 95-97 (salmon highlight) the sidechain of Arg96 is included because it forms crucial hydrogen bonds with S5A, Gly69 and Val70: the sidechain of Arg97 is not nearby and is truncated to gly. In the rear chain (mauve highlight) with hydrogen bonds to S5A, Ile355 and Ile358 are truncated to gly. Glu380 and Phe381 (fawn highlight) are included in full. Ligand His442 is complete and Ser443 is truncated to gly. Homocitrate is complete. HOH531 has hydrogen bonds with two chains, Ser192, His195 and Arg277, Tyr281, and is part of the putative proton transfer from surface water (HOH545) to His195.[4] The four penultimate waters of the proton wire[5] (HOH519, HOH506, HOH536 and HOH534) are included. A proton on the coordinated alcoholate O atom of homocitrate is not shown in this Figure.

Experimental and computational studies are in general agreement that the redox level of the [CFe_7MoS_9] core of resting FeMo-co cluster is described by net charge -1,[1, 6-8] and so the net charge for the complete model in Chart S1 is -2 (-1 when the His195 side chain is protonated).

2. Computational constraints

Some constraints on the protein structure are required during optimization calculations because the modelled protein is incomplete and the bonding and dispersion influences of the complete protein outside the computational model are absent. The modelled protein must also maintain flexibility sufficient to accommodate the coordination of N_2 and of H_2, and the diffusion of these molecules to and from FeMo-co. The constraints used here involved the Cα atoms at the ends of the modelled protein chains, allowing movement of the intermediate residues and all sidechains. The constraints maintained the crystallographic distances between C^c at the centre of FeMo-co and the Cα atoms of residues 62, 95, 97, 191, 196, 229, 281, 355, 358, 380, 381, 443, and C^c -O distances for the included water molecules. The lengths of the hydrogen bonds between the backside water HOH531 and Arg277, Tyr281 and Ser192 (Chart S1) were also constrained.

As previously explained[9] trial calculations on the modes of diffusion and coordination of N_2 / H_2 led to the conclusion that the front chain near Val70 should move away from FeMo-co. Details are provided in section 7 below. This is consistent with experimental data on mutation of amino acid 70 with smaller and larger side chains.[10-12] All calculations in the present paper constrained Cα(Val70) -- C^c as 7.4Å and the Cα(Val70) -- CZ(Arg96) distance to 4.4Å.

There were no constraints on bond distances or bond angles, including during transition state searches.

This overall strategy for constraints provides a computationally stable model, able to adjust to non-bonding dispersion interactions in the vicinity of the active site.

3. Density functional procedures

Density functional (DF) calculations use the DMol33 methodology of Delley,[13-18] with accurate DNP (double numerical plus polarisation) basis sets.[16] The real-space cutoff in the calculation of atomic basis sets was 9 au (4.8 Å), which gives results within 0.1% of the limit for larger extensions of the basis sets. The gradient-corrected functional PBE[19] was used because validation tests demonstrate that when used with the numerical basis sets of DMol3 it is more accurate than other commonly used functionals.[20] See section 4 Validation, below.

The DMol3 method uses numerical solutions of DFT-free atoms as part of its basis set, and thus gives highly accurate DFT solutions for the separated atoms limit.[16] These numerical basis sets avoid the need for basis set superposition corrections, and provide reliable energies at long intermolecular distances. This advantage of the DMol3 methodology obviates the need for empirical treatment[21] of the long-range dispersion energies that are involved when the computational model is comprised of separate molecular units. This ability of the DMol3 methodology to incorporate long-range dispersion has been well demonstrated. Zhang *et al* reported the excellent agreement between the experimental interatomic potential energy curves and the DMol3/PBE calculated potential energy curves for the inert-gas pairs He_2, Ne_2, Ar_2, HeNe, HeAr and NeAr.[22] Todorova and Delley used this DMol3 / DNP / PBE methodology to optimize the crystal structures of a comprehensive set over 40 molecular crystals in which dispersion and intermolecular interactions determine the crystal packing and crystal lattice parameters.[18] This set of molecular crystals ranged over amino acids, aliphatic and aromatic hydrocarbons, ice, H_2S, S_8, C_{60}, and the inert gases Ne, Ar, Kr and Xe. The PBE functional overestimates lattice parameters by about 4%, while the PBE_{sol} functional yields lattice parameters with an average systematic error of less than 1%. Todorova and Delley emphasise that the DMol3 numerical basis sets have low BSSE because of the accurate tails of the molecular orbitals.

The calculations were all-electron, spin-unrestricted, with no imposed symmetry. The conductor-like screening model (COSMO)[23-25] was used with a dielectric constant of 5. Constraints on interatomic distances used the Lagrange Multiplier Algorithm. Control of electronic states was via the input spin populations for Fe1, Fe3, Fe4, Fe5 and Fe7. Output spin populations are calculated by the Mulliken method.[26]

4. Validation

Table S1 contains the results of calculations of the resting state of FeMo-co using the full protein model (482 atoms) and the computational methodology of this paper. The basis set is DNP, functional PBE, spin S=3/2, electronic state 235 (ie Fe2, Fe3 and Fe5 have negative spin densities). Results are for unprotonated and protonated His195. Calculated bonded and non-bonded distances are compared with the best crystallographic values, from PDB 3U7Q. The agreement between calculated and experimental lengths for C^c-Fe bonds and for Fe-S bonds is 0.01Å or better. The overall average deviation is 0.02Å. The longer distance between Nε of His195 and S2B, an interaction which may or may not be hydrogen bonded in the crystal structure, is calculated to be 0.12 Å longer with unprotonated His195 and 0.06Å shorter with His195 protonated at Nε.

Table S1. Calculated distances, bonded and non-bonded, for the resting state of FeMo-co, for unprotonated and protonated forms of His195, in comparison with the crystallographic distances (PDB 3U7Q). Basis set DNP, functional PBE, S=3/2, electronic state 235. Cc is the C atom at the centre of FeMo-co.

distance	xtl 3U7Q	calculated with His195 unprotonated	calc - xtl	calculated with Hs195 protonated	calc - xtl
Cc-Fe2	1.997	1.945	**-0.052**	1.962	**-0.036**
Cc-Fe3	1.984	1.982	**-0.001**	1.982	**-0.002**
Cc-Fe4	1.990	2.005	**0.015**	1.995	**0.004**
Cc-Fe5	2.008	2.045	**0.036**	2.023	**0.015**
Cc-Fe6	2.018	1.969	**-0.049**	2.009	**-0.009**
Cc-Fe7	1.997	2.011	**0.014**	1.998	**0.000**
Fe1-Fe2	2.668	2.673	**0.005**	2.671	**0.003**
Fe1-Fe3	2.668	2.653	**-0.015**	2.652	**-0.015**
Fe1-Fe4	2.655	2.681	**0.027**	2.687	**0.032**
Fe2-Fe6	2.576	2.630	**0.054**	2.643	**0.067**
Fe3-Fe7	2.583	2.603	**0.021**	2.606	**0.024**
Fe4-Fe5	2.612	2.573	**-0.039**	2.555	**-0.057**
Mo-Fe5	2.727	2.733	**0.006**	2.731	**0.004**
Mo-Fe6	2.674	2.717	**0.044**	2.730	**0.056**
Mo-Fe7	2.681	2.679	**-0.002**	2.687	**0.006**
Fe2-S1A	2.261	2.257	**-0.004**	2.252	**-0.009**
Fe2-S2A	2.254	2.291	**0.037**	2.296	**0.042**
Fe3-S2A	2.275	2.285	**0.010**	2.288	**0.013**
Fe3-S4A	2.254	2.233	**-0.021**	2.228	**-0.026**
Fe2-S2B	2.206	2.178	**-0.028**	2.219	**0.013**
Fe3-S5A	2.221	2.236	**0.015**	2.226	**0.005**
Fe6-S2B	2.173	2.160	**-0.013**	2.219	**0.047**

Fe7-S5A	2.210	2.209	**0.000**	2.199	**-0.011**
Fe6-S1B	2.235	2.226	**-0.008**	2.234	**0.000**
Fe6-S3B	2.218	2.238	**0.020**	2.250	**0.032**
Fe7-S3B	2.245	2.254	**0.009**	2.252	**0.007**
Fe7-S4B	2.220	2.228	**0.009**	2.222	**0.002**
**average deviation**			**0.020**		**0.020**
HisN-S2B	3.216	3.341	**0.125**	3.153	**-0.063**
mean Fe--Fe	2.627	2.636	**0.009**	2.636	**0.009**
mean Fe-S	2.231	2.233	**0.002**	2.240	**0.010**
mean Fe--Mo	2.694	2.710	**0.016**	2.716	**0.022**
mean Cc-Fe	1.999	1.993	**-0.006**	1.995	**-0.004**

Thorhallsson et al consider that agreement with the dimensions of the crystal structure is essential for gauging the quality of the computational protocols.[27] Raugei et al[28] also present comparative results on the accuracy of the PBE functional to reproduce nearest neighbour Fe-Fe distances in resting FeMo-co.

A comprehensive examination of the ability of the DMol3 methodology to calculate experimental data is reported in ref [20]. Nineteen experimetal test systems were used to evaluate eleven density functionals. The data included geometries and energies for coordination of N2, CO, H2 and C2H4 at metal sites, structures of Fe_xH_y clusters, and hydrogen bonding by water. The conclusion was that functionals PBE, PW91 and BP provide acceptably accurate results, with the best functional being PBE, which yields reaction energies within or very close the experimental error range. Other assessments of DMol3 accuracy vs experiment data have examined the electron affinities of atoms and molecules,[29] the enthalpies of formation for small (<100 electrons) molecular and atomic species,[30] the structures of metal-sulfide clusters,[31] photo-induced conversion of $[Os(NH_3)_5(\eta^1{-}N_2]^{2+}$ to $[Os(NH_3)_5(\eta^2{-}N_2]^{2+}$ (i.e. end-on to side-on bonding of N_2),[32] the geometry and vibrational frequencies of $[(NH_3)_5Ru(pyrazine)Ru(NH_3)_5]^{4+,5+,6+}$,[33] and protonation reactions involving hydrogen bonding.[4]

5. Determination of transition states

The following pragmatic procedure is used to map reaction energy surfaces and locate transition states. [5, 8, 34-35] A chemically reasonable geometry between energy minima is evaluated by observing the directions and extent of geometry change during an energy minimisation calculation that is restricted to small (ca 0.03 Å) atom displacements at each cycle. This reveals significant geometrical variables (i.e. which atoms are moving, which bond distances and angles are changing) and also the energy gradients involved. This calculation also indicates the direction of the energy saddle relative the 'reactant' and 'product' minima. Then, as illustrated in Figure S1, a point **Δ1** that is a short distance along this first optimization path is selected, and from this structure an atomic arrangement (**2** on Figure S2) on the other side of the saddle is found (by trial and error and chemical experience) and similarly evaluated with small-displacement energy minimisation steps. A point **Δ2** along the second optimisation path is selected, and from this the key moving atoms are adjusted to be just back across the suspected saddle-point towards the 'reactant' state. Repetition of this cycle, with subsequent short small-step energy minimisations, always starting from a point just on the other side of the barrier, allows the barrier to be straddled from opposing trial positions that progressively approach each other in geometry and energy, and converge at the transition state (TS). Confirmation of the TS is obtained by following the complete trajectory of geometries from positions nudged just away from the TS, linking the TS with the connected reactant and product energy minima on the same potential energy surface. The protocol uses human participation, which is itself insightful, and with chemical experience the procedure works efficiently.

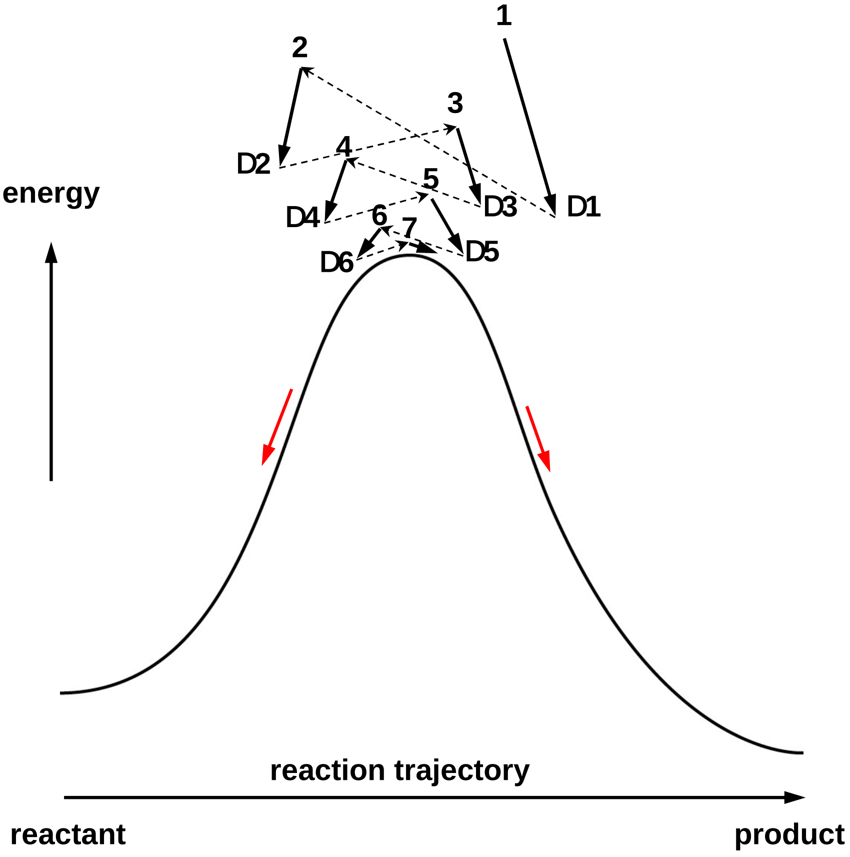

Figure. S1. Generalised diagram of the procedure for locating a reaction transition state. The solid arrows represent the course of limited small-step energy reductions from the numbered positions, which are generated (broken arrows) from Δ positions partway along the previous energy reduction, chosen according to geometry and energy gradient. The postulated trials necessarily oscillate across the barrier, and therefore optimise all other variables and decrease the energy and gradient while approaching the top of the barrier. Confirmation that the transition state, reactant and product are on the same potential energy surface is obtained by complete energy minimisations (red arrows).

6. Energetically favorable electronic states

The magnitudes of calculated Fe spin populations in FeMo-co, listed in refs [28, 36-37], generally range 2.0 to 3.3 for Fe and < 0.4 for Mo in the more stable electronic states of unligated FeMo-co. The spin population of the unique Fe1 atom is usually larger than those of the other six Fe atoms. However the binding of ligands at Fe atoms, and the distortions of FeMo-co associated with ligation, often reduce the magnitudes of Fe spin populations, sometimes almost to zero.[9, 38-39] This can blur differences between electronic states. Therefore, full account of the electronic states of ligated FeMo-co in reaction intermediates needs to include the magnitudes as well as signs of the spin populations on the set of seven Fe atoms. The ligated Fe atoms, Fe2 and/or Fe6, often have smaller spin populations approaching zero, while the remaining Fe atoms have spin population magnitudes ca 2.5. A survey of the geometric and electronic structures of the hydrogenated forms of FeMo-co, involving more than 100 structures with one, two, three of four H atoms bonded to Fe and/or S and including structures with bound H_2, showed, where four or more electronic states were tested, that the most stable state contains either the Fe4-Fe7 pair or the Fe3-Fe5 pair with negative spin populations.[38] These states are denoted '47' and '35'.

This energy advantage of the 47 and 35 electronic states is readily understood from the general principle that oppositely-signed pairs of adjacent Fe atoms in FeMo-co are more stable than same-signed, and the stabilisation is proportional to the magnitude of the spin populations. Antiferromagnetism (ie weak Fe-Fe

bonding) operates in the FeMo-co cluster. Interactions between the spins at Fe2 and Fe6 are of minor importance because they are diminished by ligation, and the Fe2--Fe6 separation is increased due to extensions of one or both Fe-C^c distances. For the same reason interactions between the Fe2 and Fe6 spins and those at Fe3, Fe4, Fe5 and Fe7 are less influential than those between these four Fe atoms. Therefore interactions within the set Fe3, Fe4, Fe5, Fe7 on the opposite face of the Fe6 trigonal prism are dominant, and, as shown in Figure S2, the maximum number of oppositely-signed shorter edges for this group is obtained with same-signed spins along one diagonal, 47 or 35. Therefore the most stable electronic states include either 47 or 35 with negative spin populations.

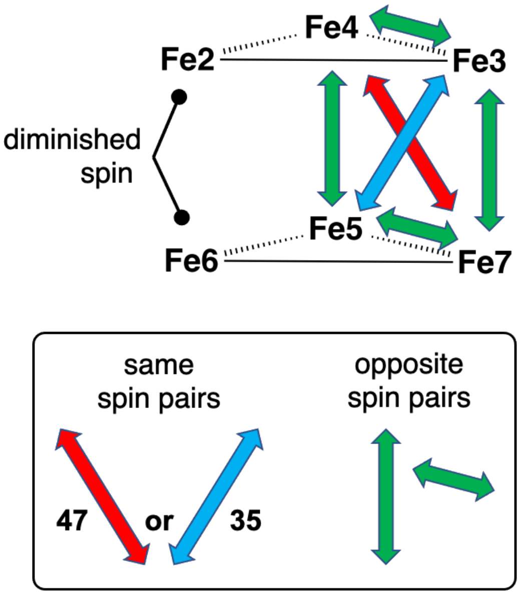

Figure S2. When the spin populations of Fe2 and Fe6 are diminished due to ligation, there are two pairs of negative spin, red or blue, that yield four opposite (green) spin pairs along the shorter edges of the Fe3, Fe4, Fe5, Fe7 face where the spin populations have largest magnitude.

7. Movement of Val70 and the contiguous chain

Previous calculations have revealed some steric interference between diffusing N_2 in the Fe2--Fe6 reaction zone and the immediately contiguous protein (mainly the side chain of Val70) in its resting position.[9, 40] This is consistent with experimental data on mutation of residue 70 to larger side chains.[11-12] Therefore I postulated that the protein dynamics can accommodate and incorporate movement of Val70 and adjacent residues away from FeMo-co.[9] Figure S3 shows the relevant protein structure. The side chain of Val70 that protrudes towards Fe2 and Fe6 at Fe--C distances of 4.4Å extends from an α- and π- helical chain. This chain loops back along the opposite side of the helix, where it forms β-sheet with a later section of the chain. The helical section engages three hydrogen bonds (Fig. S3 (a)), of which Ser67 → Gln151 is most relevant because it is almost opposite Val70. Fig. S3 (b) shows how the helical section of the chain can rotate and slide slightly to increase the Val70 - Fe separations without disrupting this hydrogen bond. Qualitative manipulation of this protein assemblage confirmed that the Cα(Val70)-C^c and Cα(Trp72)-C^c distances in the constrained expanded model could be attained. Obviously analysis of the dynamics of the complete protein is required to explore and quantitate the protein dynamics that would increase the space between Fe2, Fe6 and Val70. The sidechain of Arg96 with possible hydrogen bonds to the mainchain at Gly69 and Val70 is also significant in this domain.

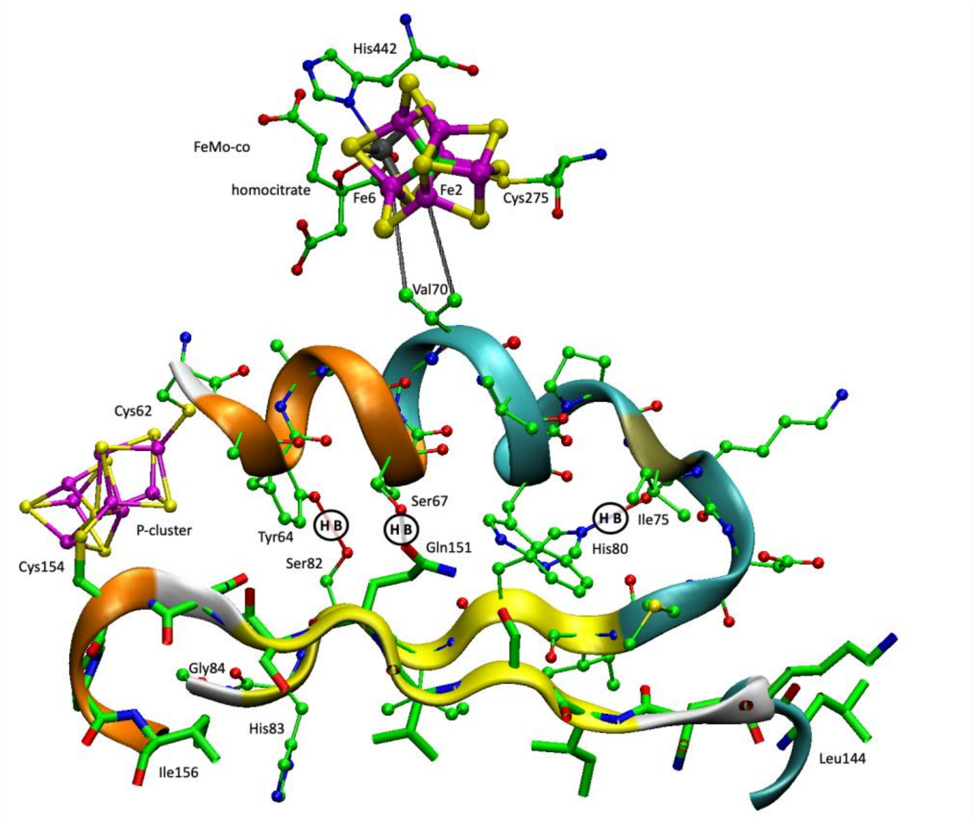
His442
FeMo-co
homocitrate
Fe6
Fe2
Cys275
Val70
Cys62
P-cluster
Tyr64
Ser67
Ile75
Cys154
Ser82
Gln151
His80
Gly84
His83
Ile156
Leu144

(a)

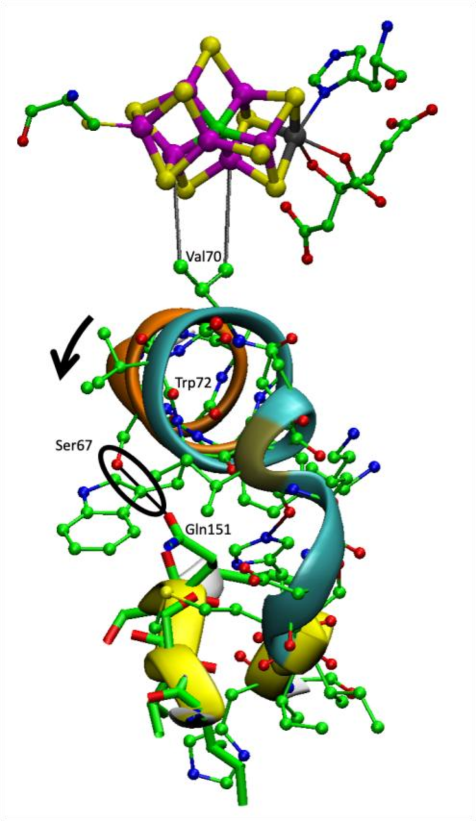
Val70
Trp72
Ser67
Gln151

(b)

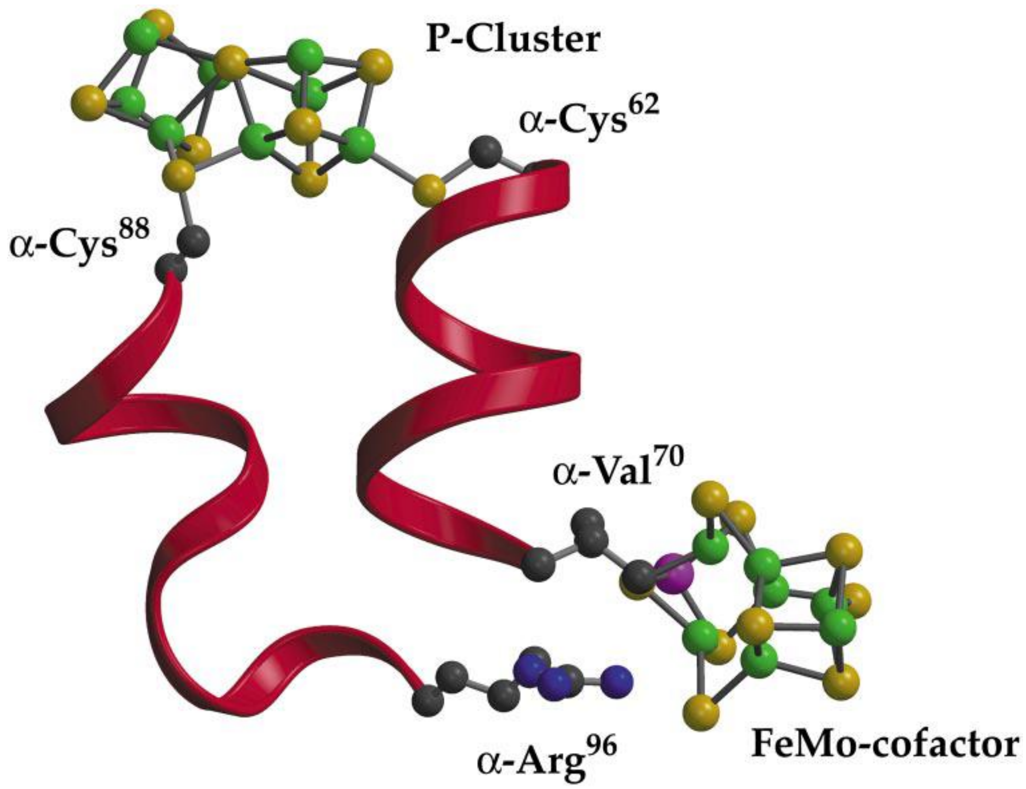

(c)

Figure S3. (a) Structure of the protein containing Val70, adjacent to the Fe2,3,6,7 face of FeMo-co (from crystal PDB 3U7Q, all residues are in the a chain). The grey lines identify the approaches (C--Fe 4.37, 4.44Å) of the side chain methyl groups of Val70 to Fe2 and Fe6 of FeMo-co. Two relevant sections of the protein chain are drawn, with secondary structure superimposed as ribbons. The closest section of the chain, containing Val70, is drawn with thin bonds and spherical atoms and extends from Cys62 (a ligand of the P-cluster) to Gly84. A more distant part of the chain, drawn with thick bonds, is from Leu144 to Cys154 (P-cluster ligand). Both sections are part of a β-sheet (yellow ribbons). The α-helical section of the closest chain (residues 63-68, orange ribbon) changes to π-turn (cyan ribbon) through Val70, before looping back to the β-sheet. There are three hydrogen bonds (labelled HB) involving the closest section of chain, and only one of these (Ser67 → Gln151) connects the two sections of chain. (b) End view of (a), showing how the closest section of chain containing Val70 is located between FeMo-co and the distant section of chain containing Gln151. The large black arrow depicts the slight rotation and displacement of the section containing Val70 and Trp72 that can move them away from FeMo-co, without disrupting the Ser67 → Gln151 hydrogen bond. (c) Peptide chain connections between Val70 and Arg96 around FeMo-co and two Cys ligands of the P-cluster. From Ref [41].

8. Libratory movement of the Arg96 sidechain

In the present investigation of NH_3 formed in the reaction zone, and diffusing away between the side chains of Val70 and Arg96, it became apparent that a small separation of these sidechains should occur, by a libratory movement of the sidechain of Arg96, without changing the resting state hydrogen bond from NH2 of Arg96 to S5A. This is shown in Figure S4. Therefore the Cα(Val70) -- C^c and Cα(Val70) -- CZ(Arg96) distances were fixed, at 8.2 and 5.18 Å respectively.

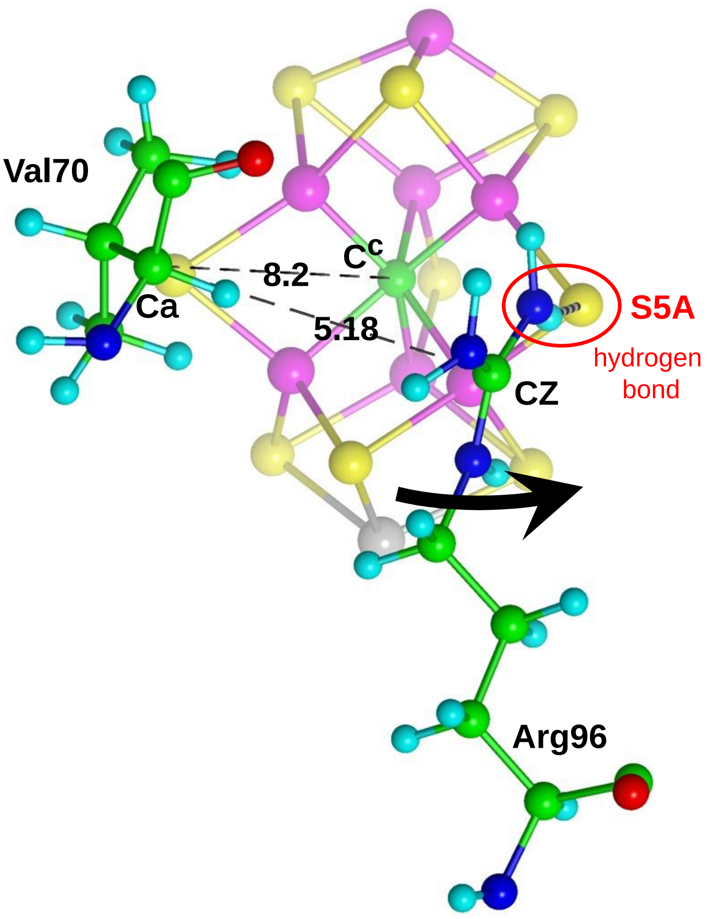

Figure S4. Expansion of the reaction zone by swinging the guanidine sidechain of Arg96 by about 1 Å around the unchanged hydrogen bond from NH2 to S5A

9. Non-obligatory H_2 evolution

Scheme S1 shows computed pathways for some reactions forming and dissociating H_2 at Fe6, at the E2, E3 and E4 stages.

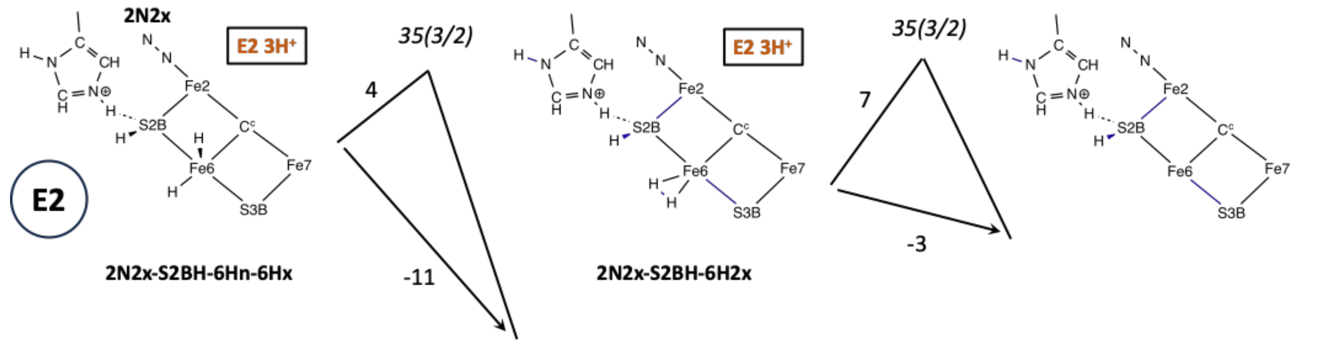

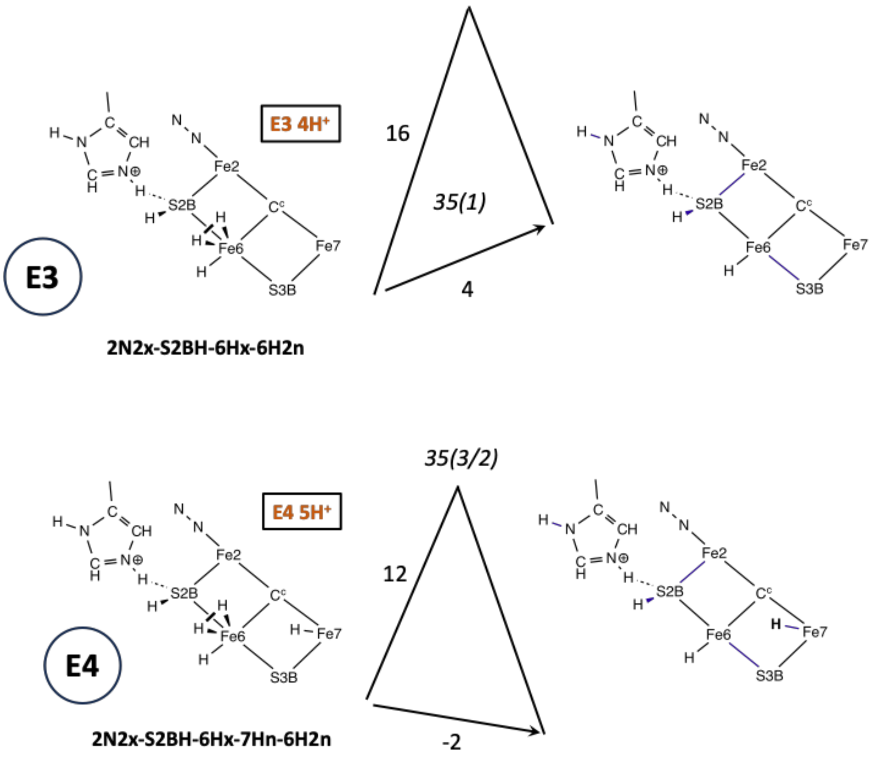

Scheme S1.   Some pathways for H_2 release at the E2, E3 and E4 stages of the complete mechanism. Energies in kcal mol^{-1}.

10. Truncated models for quantum tunneling calculations

Table S2 contains coordinates for truncated forms of the transition states for the N_2-catpure reaction and the N-N breaking reaction. These are provided for quantum tunneling calculations. Both structures can be simplified further, by converting SCH_3 to SH, imidazole to NH_3, and the glycollate ligand to two OH ligands.

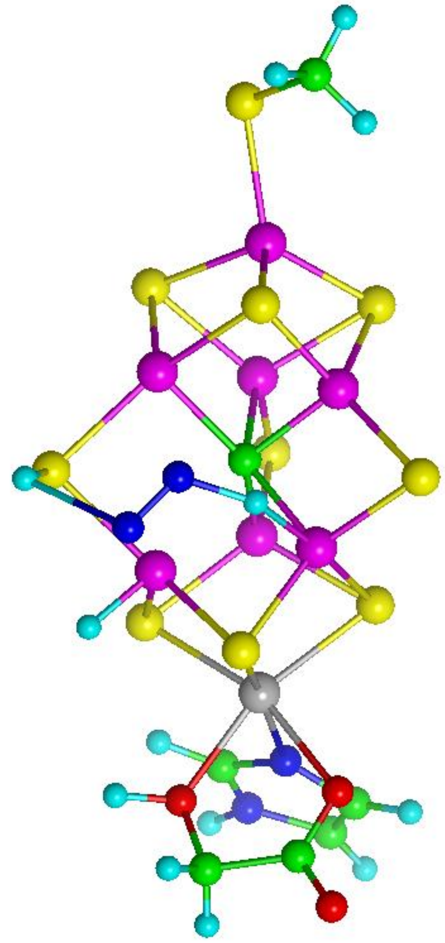

TS for **pre-capture → Fe2-brNH-NH-Fe6**

1	5.168029000	6.421560000	-1.263240000	H
2	4.696126000	5.527085000	-1.100486000	C
3	6.221042000	-6.449692000	-4.225651000	H
4	5.333324000	-6.357632000	-4.811101000	C
5	4.957011000	-6.923965000	-6.011897000	C
6	3.246288000	-5.802070000	-5.168286000	C
7	2.408965000	-7.017548000	-0.255172000	H
8	3.001321000	-7.100354000	-1.120025000	C
9	3.018457000	-8.029445000	-1.589301000	H
10	4.460346000	-6.857000000	-0.699203000	C
11	3.600406000	-0.689531000	-2.236110000	C
12	3.791378000	-2.020962000	-3.648059000	Fe
13	2.218526000	0.764833000	-1.518808000	Fe
14	5.079271000	0.497840000	-1.556022000	Fe
15	3.793783000	0.497385000	-3.793761000	Fe
16	3.923717000	2.744615000	-2.383934000	Fe
17	4.741903000	-1.996976000	-1.241410000	Fe
18	2.196838000	-2.397386000	-1.548492000	Fe
19	1.563660000	-5.994458000	-1.816942000	H
20	5.484441000	4.778727000	-0.950280000	H
21	4.091203000	5.600813000	-0.186111000	H
22	3.044822000	-6.863909000	-6.977059000	H
23	2.241090000	-5.416052000	-5.065952000	H
24	5.502396000	-7.560841000	-6.696667000	H
25	1.142805000	-3.239584000	-0.879623000	H
26	3.779092000	-1.170841000	0.051993000	H
27	0.127380000	-0.853502000	-0.534912000	H
28	3.840840000	-4.400029000	-2.336337000	Mo
29	4.248199000	-5.644850000	-4.303708000	N
30	3.644595000	-6.558497000	-6.215321000	N
31	2.554622000	-0.747594000	0.363376000	N
32	1.768145000	-1.521066000	0.737839000	N
33	5.085346000	-5.880791000	-1.303028000	O
34	4.997608000	-7.586768000	0.154233000	O
35	2.599223000	-6.091649000	-2.047973000	O
36	3.586959000	4.983562000	-2.485345000	S
37	2.105369000	1.943526000	-3.458182000	S
38	2.021060000	-3.378227000	-3.483621000	S
39	3.850692000	1.933373000	-0.348718000	S
40	0.544576000	-0.829576000	-1.846927000	S
41	4.013285000	-0.847324000	-5.521808000	S
42	3.554975000	-3.547616000	-0.166459000	S
43	5.675846000	1.681561000	-3.358104000	S
44	5.667618000	-3.061949000	-3.045814000	S
45	6.390017000	-0.803308000	-0.372549000	S
end				

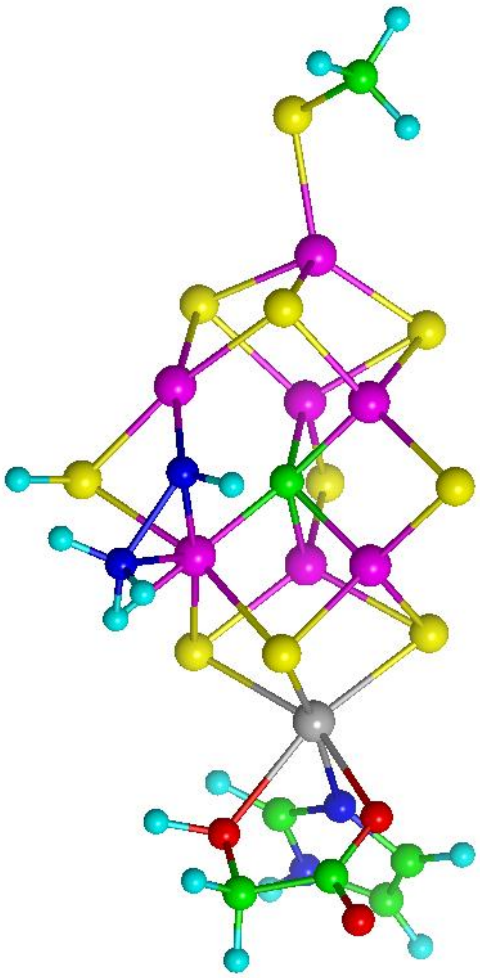

TS for **S2BH-Fe2-brNH-NH2-Fe6H → S2BH-Fe2-brNH-Fe6NH3**

1	6.968620000	-4.462203000	0.687767000	H
2	6.065448000	-3.851299000	0.799439000	C
3	-5.047846000	-6.075622000	6.078426000	H
4	-5.620416000	-6.122777000	5.152618000	C
5	-6.651213000	-6.848998000	4.593589000	C
6	-5.813602000	-5.479305000	3.067820000	C
7	-5.613896000	-0.639954000	4.970241000	H
8	-5.785187000	-1.652648000	5.215659000	C
9	-6.839490000	-1.815766000	5.480060000	H
10	-4.921586000	-2.072323000	6.414755000	C
11	-0.218491000	-3.496364000	2.299427000	C
12	-1.746878000	-4.781051000	2.365905000	Fe
13	0.776088000	-1.966653000	0.725074000	Fe
14	1.577204000	-3.613785000	3.133360000	Fe
15	0.509369000	-4.970409000	1.211153000	Fe
16	3.023170000	-4.106167000	0.998525000	Fe
17	-0.694670000	-3.479631000	4.266641000	Fe
18	-1.506744000	-2.044390000	2.085489000	Fe
19	-5.541330000	-1.785091000	3.255248000	H
20	5.688607000	-3.994047000	1.822244000	H
21	6.312601000	-2.789857000	0.652743000	H
22	-7.459721000	-6.718940000	2.614006000	H
23	-5.691712000	-4.956858000	2.127285000	H
24	-7.315587000	-7.589952000	5.022802000	H
25	-2.504764000	-0.542183000	2.043458000	H
26	-1.416871000	0.520034000	1.247041000	H
27	0.356307000	-0.678295000	3.096499000	H
28	-1.510867000	0.467964000	2.953820000	H
29	-1.337946000	-0.486024000	-0.472343000	H
30	-3.465021000	-3.626153000	4.195616000	Mo
31	0.065115000	-0.794092000	2.110980000	N
32	-1.484564000	-0.056788000	2.082426000	N
33	-5.099807000	-5.276520000	4.174485000	N
34	-6.753775000	-6.428923000	3.285088000	N
35	-4.059826000	-3.035408000	6.230125000	O
36	-5.086421000	-1.495944000	7.508920000	O
37	-5.392667000	-2.456249000	4.078588000	O
38	4.744446000	-4.313355000	-0.404272000	S
39	1.420658000	-3.844933000	-0.523102000	S
40	-3.422416000	-3.421964000	1.833287000	S
41	2.768630000	-2.120594000	1.988322000	S
42	-1.310099000	-1.828187000	-0.187098000	S
43	-1.088405000	-6.510501000	1.125531000	S
44	-2.019520000	-1.693179000	4.398697000	S
45	2.316072000	-5.630215000	2.432702000	S
46	-1.886260000	-5.344206000	4.560154000	S
47	1.228690000	-3.326844000	5.316867000	S
end				

References

[1] B. Benediktsson, R. Bjornsson, “QM/MM Study of the Nitrogenase MoFe Protein Resting State: Broken-Symmetry States, Protonation States, and QM Region Convergence in the FeMoco Active Site” *Inorganic Chemistry* **2017**, *56*, 13417-13429.10.1021/acs.inorgchem.7b02158

[2] L. Deng, H. Wang, C. H. Dapper, W. E. Newton, S. Shilov, S. Wang, S. P. Cramer, Z.-H. Zhou, “Assignment of protonated R-homocitrate in extracted FeMo-cofactor of nitrogenase via vibrational circular dichroism spectroscopy” *Communications Chemistry* **2020**, *3*, 145.10.1038/s42004-020-00392-z

[3] L. Cao, O. Caldararu, U. Ryde, “Protonation States of Homocitrate and Nearby Residues in Nitrogenase Studied by Computational Methods and Quantum Refinement” *The Journal of Physical Chemistry B* **2017**, *121*, 8242-8262.10.1021/acs.jpcb.7b02714

[4] I. Dance, “New insights into the reaction capabilities of His195 adjacent to the active site of nitrogenase” *Journal of Inorganic Biochemistry* **2017**, *169*, 32-43.10.1016/j.jinorgbio.2017.01.005

[5] I. Dance, “The pathway for serial proton supply to the active site of nitrogenase: enhanced density functional modeling of the Grotthuss mechanism” *Dalton Transactions* **2015**, *44*, 18167-18186.10.1039/C5DT03223G

[6] T. Spatzal, J. Schlesier, E.-M. Burger, D. Sippel, L. Zhang, S. L. A. Andrade, D. C. Rees, O. Einsle, “Nitrogenase FeMoco investigated by spatially resolved anomalous dispersion refinement” *Nat Commun* **2016**, *7*, 10902.10.1038/ncomms10902

[7] R. Bjornsson, F. Neese, S. DeBeer, “Revisiting the Mössbauer Isomer Shifts of the FeMoco Cluster of Nitrogenase and the Cofactor Charge” *Inorganic Chemistry* **2017**, *56*, 1470-1477.10.1021/acs.inorgchem.6b02540

[8] I. Dance, “Computational Investigations of the Chemical Mechanism of the Enzyme Nitrogenase” *Chembiochem* **2020**, *21*, 1671-1709.10.1002/cbic.201900636

[9] I. Dance, “Structures and reaction dynamics of N_2 and H_2 binding at FeMo-co, the active site of nitrogenase” *Dalton Transactions* **2021**, *50*, 18212-18237.10.1039/d1dt03548g.

[10] P. C. Dos Santos, R. Igarashi, H.-I. Lee, B. M. Hoffman, L. C. Seefeldt, D. R. Dean, “Substrate Interactions with the Nitrogenase Active Site” *Acc. Chem. Res.* **2005**, *38*, 208-214

[11] L. C. Seefeldt, B. M. Hoffman, D. R. Dean, “Mechanism of Mo-Dependent Nitrogenase” *Annu. Rev. Biochem.* **2009**, *78*, 701-722

[12] R. Sarma, B. M. Barney, S. Keable, D. R. Dean, L. C. Seefeldt, J. W. Peters, “Insights into substrate binding at FeMo-cofactor in nitrogenase from the structure of an a-70Ile MoFe protein variant” *J. Inorg. Biochem.* **2010**, *104*, 385-389

[13] B. Delley, “An all-electron numerical method for solving the local density functional for polyatomic molecules” *J. Chem. Phys.* **1990**, *92*, 508-517

[14] B. Delley, in *Modern density functional theory: a tool for chemistry, Vol. 2* (Eds.: J. M. Seminario, P. Politzer), Elsevier, Amsterdam, **1995**, pp. 221-254.

[15] J. Baker, A. Kessi, B. Delley, “The generation and use of delocalized internal coordinates in geometry optimization” *The Journal of Chemical Physics* **1996**, *105*, 192-212.doi:http://dx.doi.org/10.1063/1.471864

[16] B. Delley, “From molecules to solids with the DMol3 approach” *J. Chem. Phys.* **2000**, *113*, 7756-7764

[17] J. Andzelm, R. D. King-Smith, G. Fitzgerald, “Geometry optimization of solids using delocalized internal coordinates” *Chemical Physics Letters* **2001**, *335*, 321-326.http://dx.doi.org/10.1016/S0009-2614(01)00030-6

[18] T. Todorova, B. Delley, “Molecular Crystals: A Test System for Weak Bonding†” *The Journal of Physical Chemistry C* **2010**, *114*, 20523-20530.10.1021/jp1049759

[19] J. P. Perdew, K. Burke, M. Ernzerhof, “Generalized Gradient Approximation Made Simple” *Phys. Rev. Lett.* **1996**, *77*, 3865-3868

[20] I. Dance, “Evaluations of the Accuracies of DMol3 Density Functionals for Calculations of Experimental Binding Enthalpies of N_2, CO, H_2, C_2H_2 at Catalytic Metal Sites ” *Molecular Simulation* **2018**, *44*, 568-581.doi.org/10.1080/08927022.2017.1413711

[21] S. Grimme, “Semiempirical GGA-Type Density Functional Constructed with a Long-Range Dispersion Correction” *J. Comput. Chem.* **2006**, *27*, 1787-1799

[22] Y. Zhang, W. Pan, W. Yang, “Describing van der Waals Interaction in diatomic molecules with generalized gradient approximations: The role of the exchange functional” *J. Chem. Phys.* **1997**, *107*, 7921-7925

[23] J. Andzelm, C. Kolmel, A. Klamt, “Incorporation of solvent effects into density functional calculations of molecular energies and geometries” *J. Chem. Phys.* **1995**, *103*, 9312-9320

[24] A. Klamt, V. Jonas, T. Burger, J. C. W. Lohrenz, “Refinement and Parametrization of COSMO-RS” *J. Phys. Chem. A* **1998**, *102*, 5074-5085

[25] B. Delley, “The conductor-like screening model for polymers and surfaces” *Molecular Simulation* **2006**, *32*, 117-123

[26] R. S. Mulliken, “Electronic population analysis on LCAO-MO molecular wavefunctions. II. Overlap populations, bond orders, and covalent bond energies” *J. Chem. Phys.* **1955**, *23*, 1833-1846

[27] A. T. Thorhallsson, B. Benediktsson, R. Bjornsson, “A model for dinitrogen binding in the E4 state of nitrogenase” *Chemical Science* **2019**, *10*, 11110-11124.10.1039/C9SC03610E

[28] S. Raugei, L. C. Seefeldt, B. M. Hoffman, “Critical computational analysis illuminates the reductive-elimination mechanism that activates nitrogenase for N_2 reduction” *Proc Natl Acad Sci U S A* **2018**, *115*, E10521-E10530.10.1073/pnas.1810211115

[29] M. Meunier, N. Quirke, D. Binesti, “The calculation of the electron affinity of atoms and molecules” *Molec. Simulation* **1999**, *23*, 109-125

[30] B. Delley, “Ground-State Enthalpies: Evaluation of Electronic Structure Approaches with Emphasis on the Density Functional Method” *The Journal of Physical Chemistry A* **2006**, *110*, 13632-13639.10.1021/jp0653611

[31] I. Dance, “Understanding structure and reactivity of new fundamental inorganic molecules: metal sulfides, metallocarbohedrenes, and nitrogenase.” *J. Chem. Soc., Chem. Commun.* **1998**, 523-530

[32] D. Schaniel, T. Wolke, B. Delley, C. Boskovic, H. U. Gudel, “Photogeneration of metastable side-on N2 linkage isomers in [Ru(NH3)5N2]Cl2, [Ru(NH3)5N2]Br2 and [Os(NH3)5N2]Cl2” *Phys. Chem. Chem. Phys.* **2008**, *10*, 5531-5538.10.1039/b806933f

[33] T. Todorova, B. Delley, “The Creutz−Taube Complex Revisited: DFT Study of the Infrared Frequencies” *Inorganic Chemistry* **2008**, *47*, 11269-11277.10.1021/ic8018748

[34] I. Dance, “A pragmatic method for location of transition states and calculation of reaction paths.” *Molecular Simulation* **2008**, *34*, 923-929

[35] I. Dance, “A pragmatic method for location of transition states and calculation of reaction paths: erratum.” *Molecular Simulation* **2011**, *37*, 257

[36] I. Dance, “Ramifications of C-centering rather than N-centering of the active site FeMo-co of the enzyme nitrogenase.” *Dalton Trans.* **2012**, *41*, 4859-4865.DOI: 10.1039/c2dt00049k.

[37] L. Cao, U. Ryde, “Influence of the protein and DFT method on the broken-symmetry and spin states in nitrogenase” *International Journal of Quantum Chemistry* **2018**, *118*, e25627.doi:10.1002/qua.25627

[38] I. Dance, “Survey of the geometric and electronic structures of the key hydrogenated forms of FeMo-co, the active site of the enzyme nitrogenase: principles of the mechanistically significant coordination chemistry” *Inorganics* **2019**, *7*, 8.10.3390/inorganics7010008

[39] L. Cao, U. Ryde, “What Is the Structure of the E4 Intermediate in Nitrogenase?” *Journal of Chemical Theory and Computation* **2020**, *16*, 1936-1952.10.1021/acs.jctc.9b01254

[40] I. Dance, “The binding of reducible N_2 in the reaction domain of nitrogenase” *Dalton Transactions* **2023**, *52*, 2013-2026.10.1039/D2DT03599E

[41] J. Christiansen, D. R. Dean, L. C. Seefeldt, “Mechanistic features of the Mo-containing nitrogenase” *Annu. Rev. Plant Physiol. Plant Mol. Biol.* **2001**, *52*, 269-295
